# Supplementary figures and images for: Long-term patterns of gender imbalance in an industry without ability or level of interest differences
Source: PLoS One. 2020 Apr 1;15(4):e0229662. doi: 10.1371/journal.pone.0229662 (PMC7112163; doi:10.1371/journal.pone.0229662)

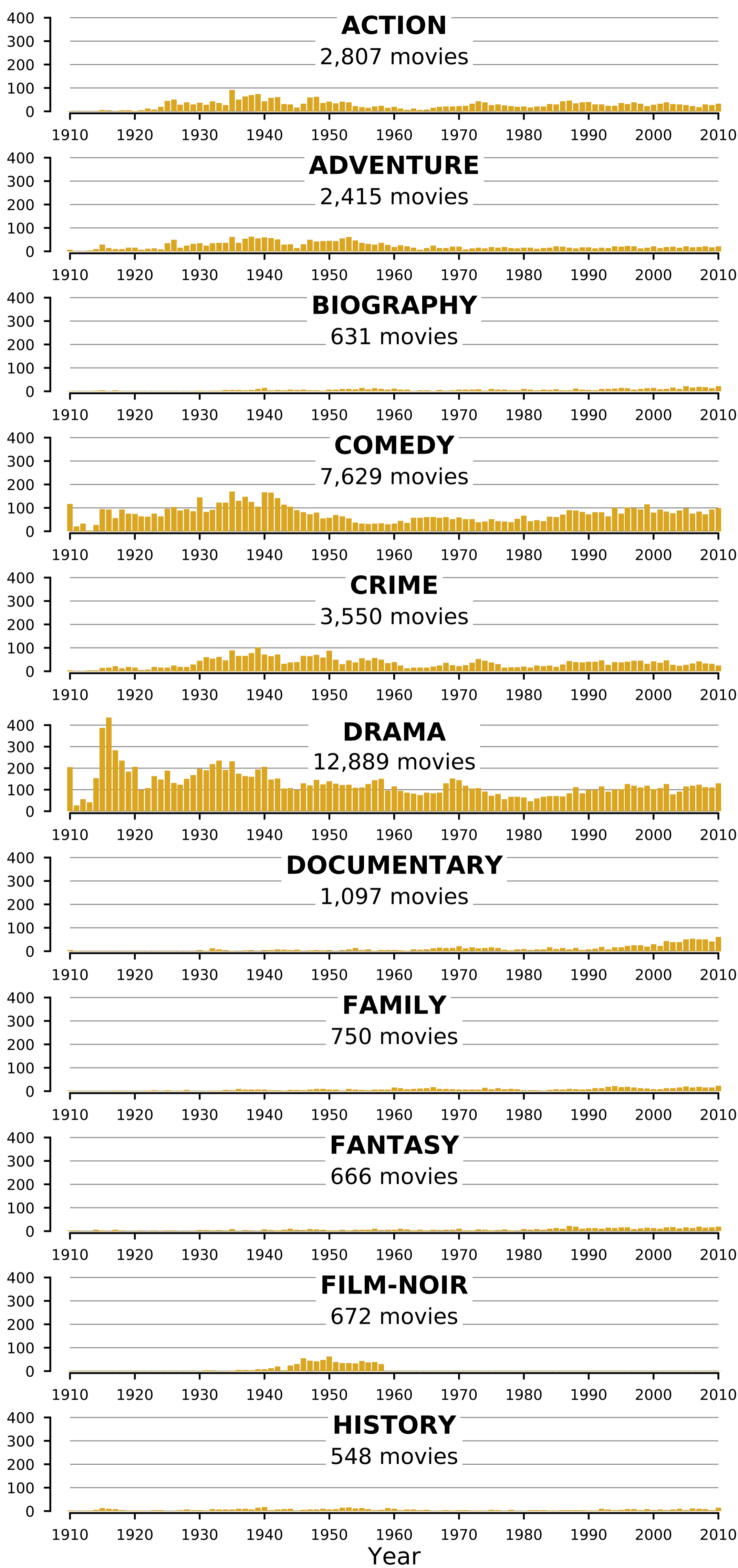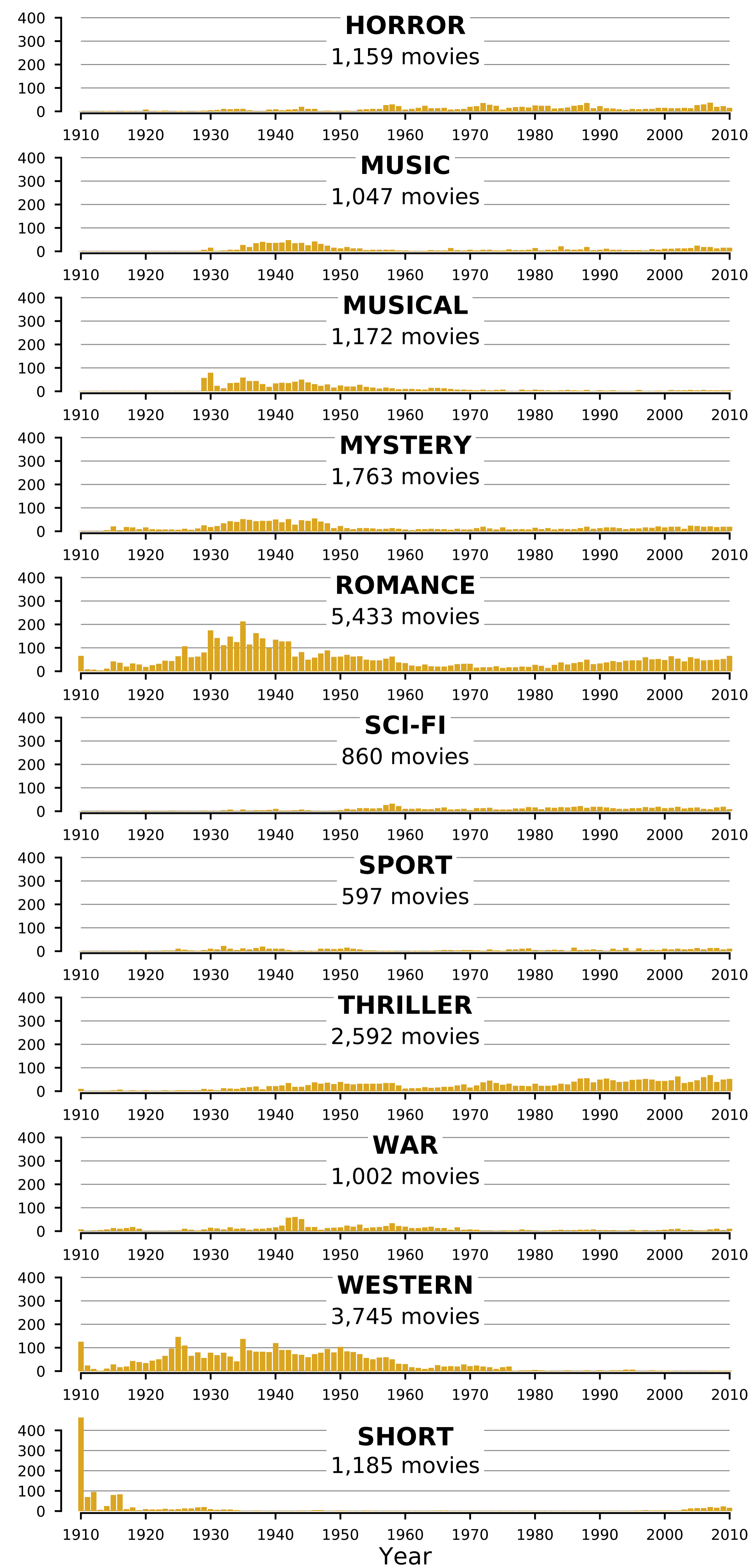

Supplement: S1 Fig — (PDF) [file pone.0229662.s001.pdf]

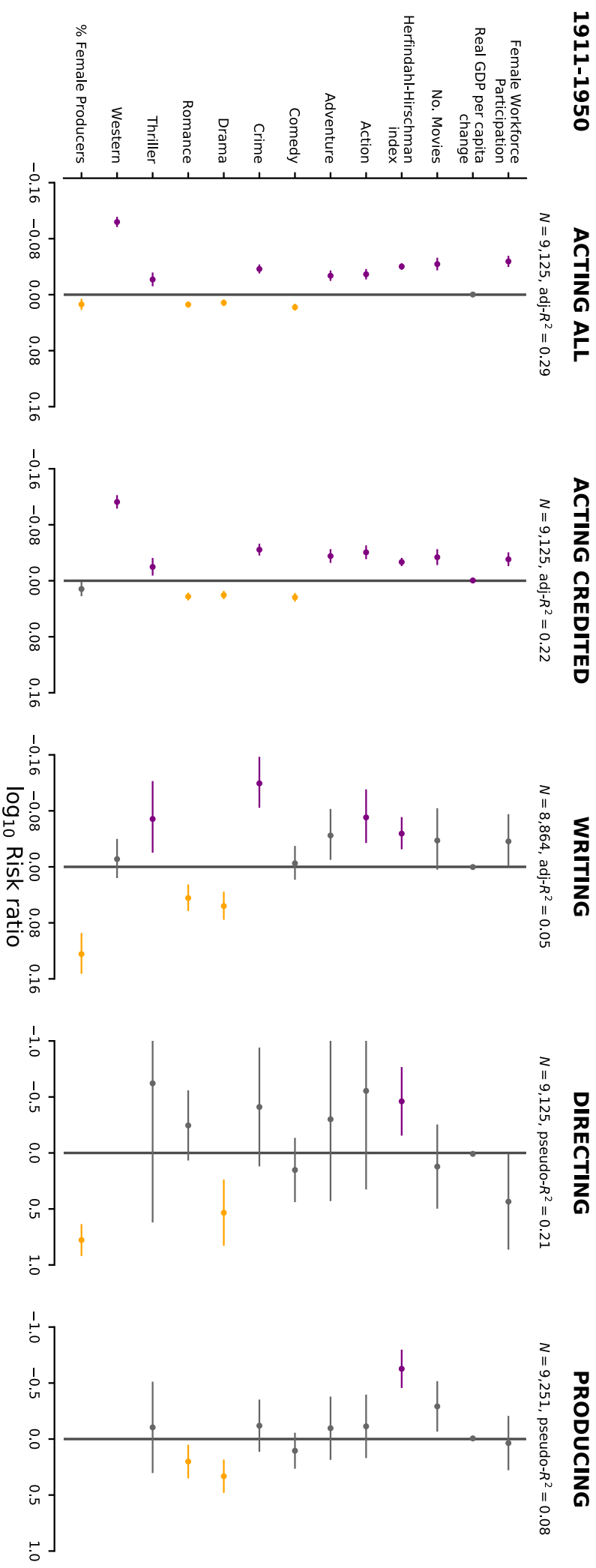

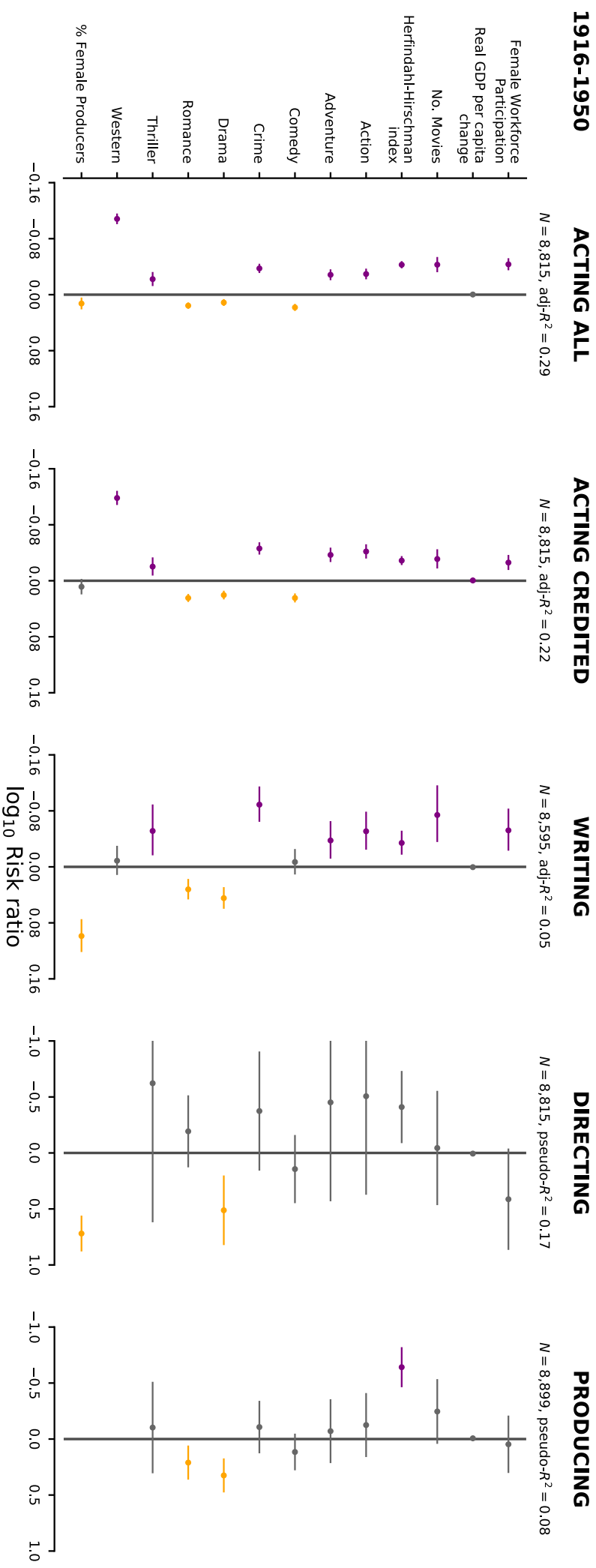

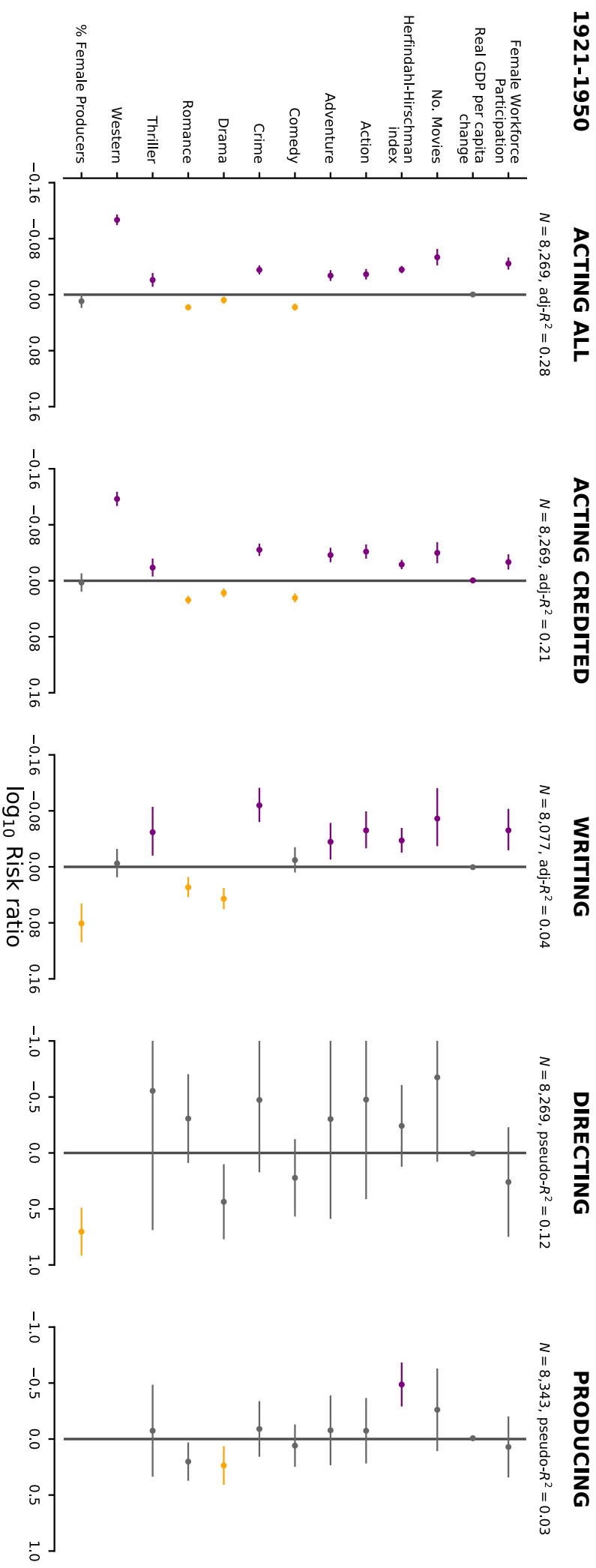

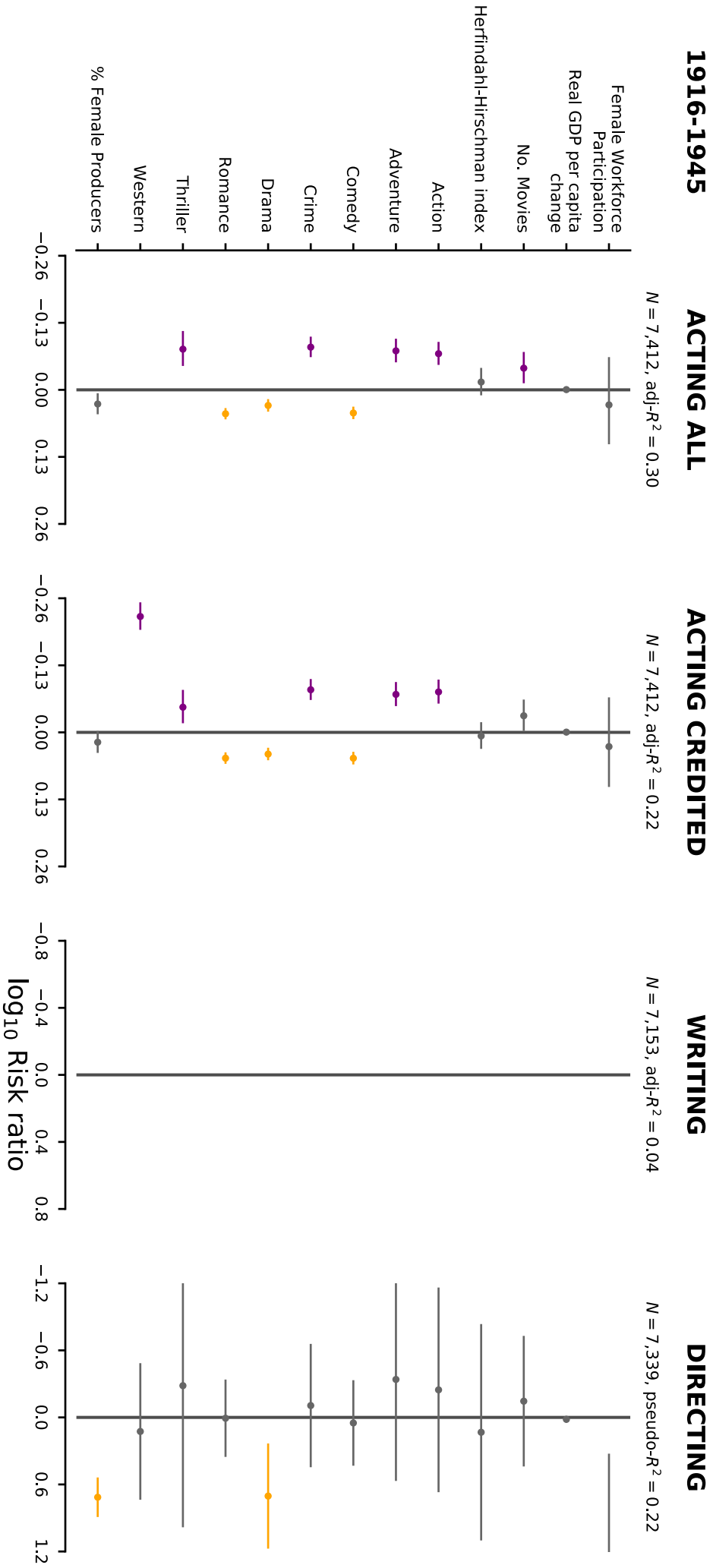

Supplement: S2 Fig — We do not include time dummies in our model. We plot the base 10 logarithm of the risk ratio for each factor. The risk factor for female workforce participation was calculated for an increase of 10%. The risk factor for change in real GPD per capita was calculated for an increase of 1%. The risk factor for number of movies was calculated for an increase of 300. The risk factor for industry concentration was calculate for a change of 0.03. The risk factor for budget was calculated for an increase by 100 fold. The risk ratio for percentage female producers was calculated for an increase of 50%. This value is one of the four typical values observed, the other three being 0%, 25% and 100%. The risk factor for percentage female directors was calculated for an increase of 100% since typically there is a single director. Risk ratios for which we are not able to reject the null hypothesis at the 0.01 level are shown in grey. Risk ratios significantly larger than 1 are shown in yellow and those significantly smaller than 1 are shown in purple. If a risk ratio estimate and its confidence bands fall outside the range displayed, we mark this with an arrow. The color of the arrow falls the same convention as the color of the risk ratio estimates. (PDF) [file pone.0229662.s002.pdf]

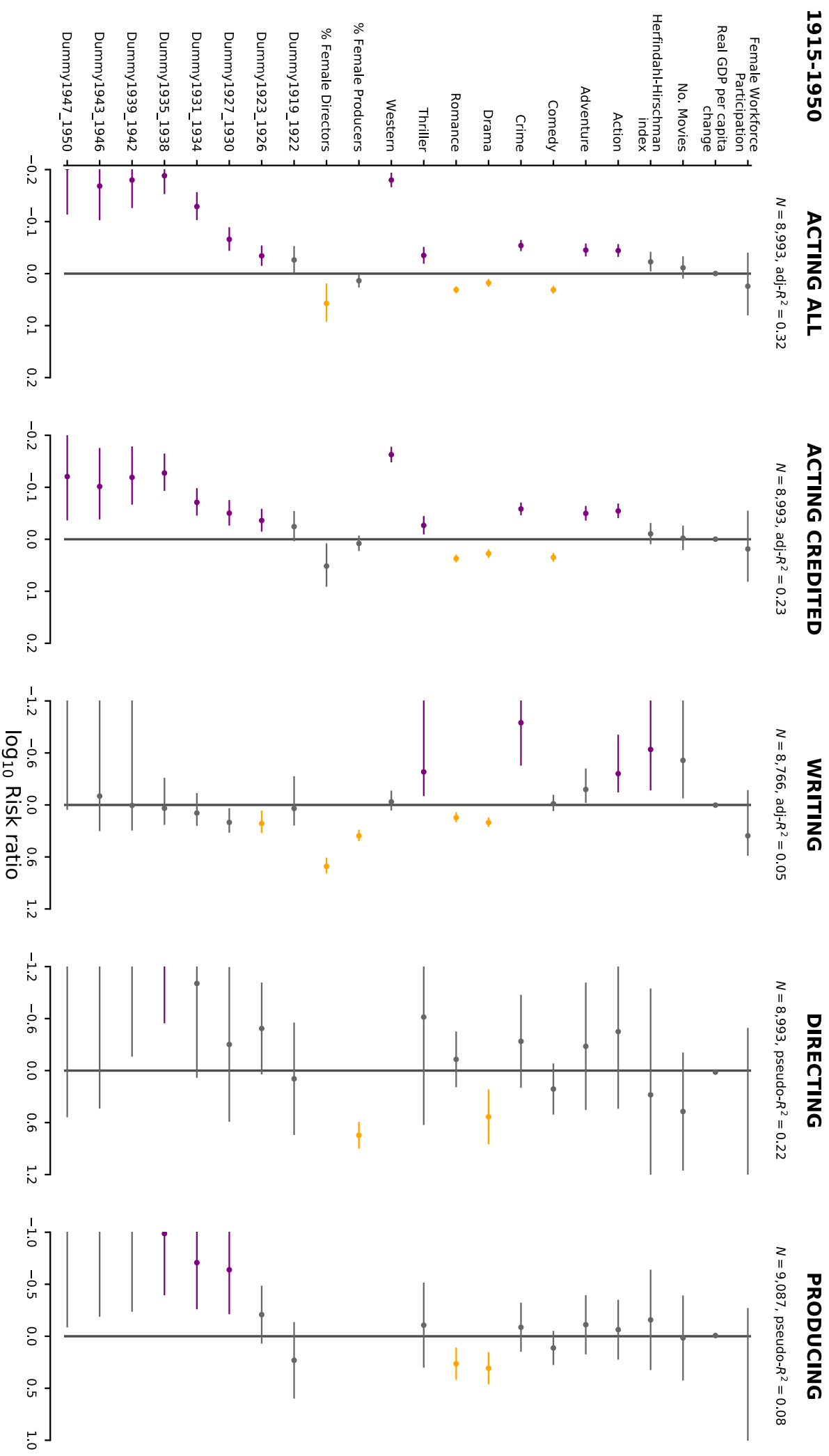

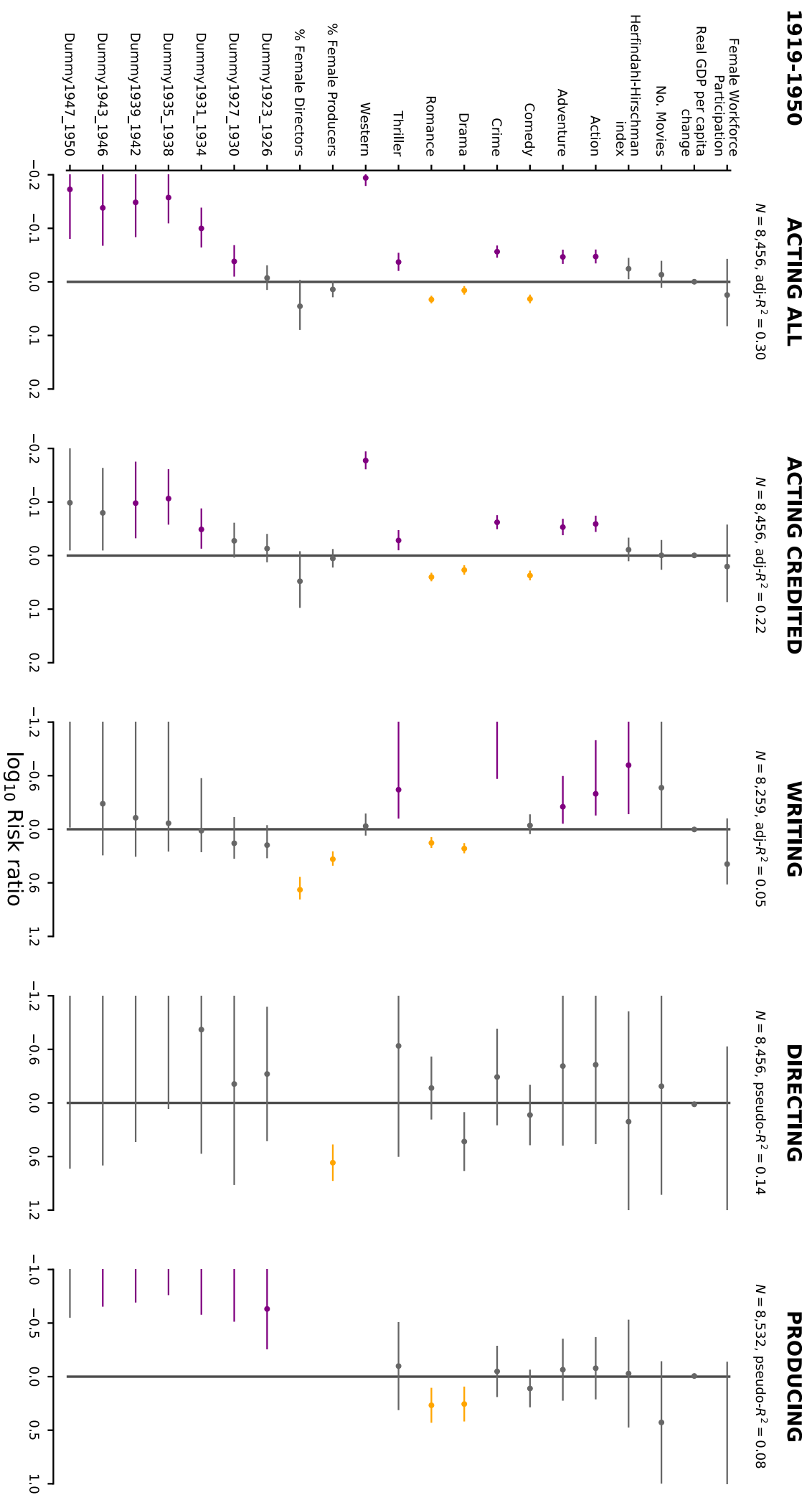

1927-1950

ACTING ALL

ACTING CREDITED

WRITING

DIRECTING

PRODUCING

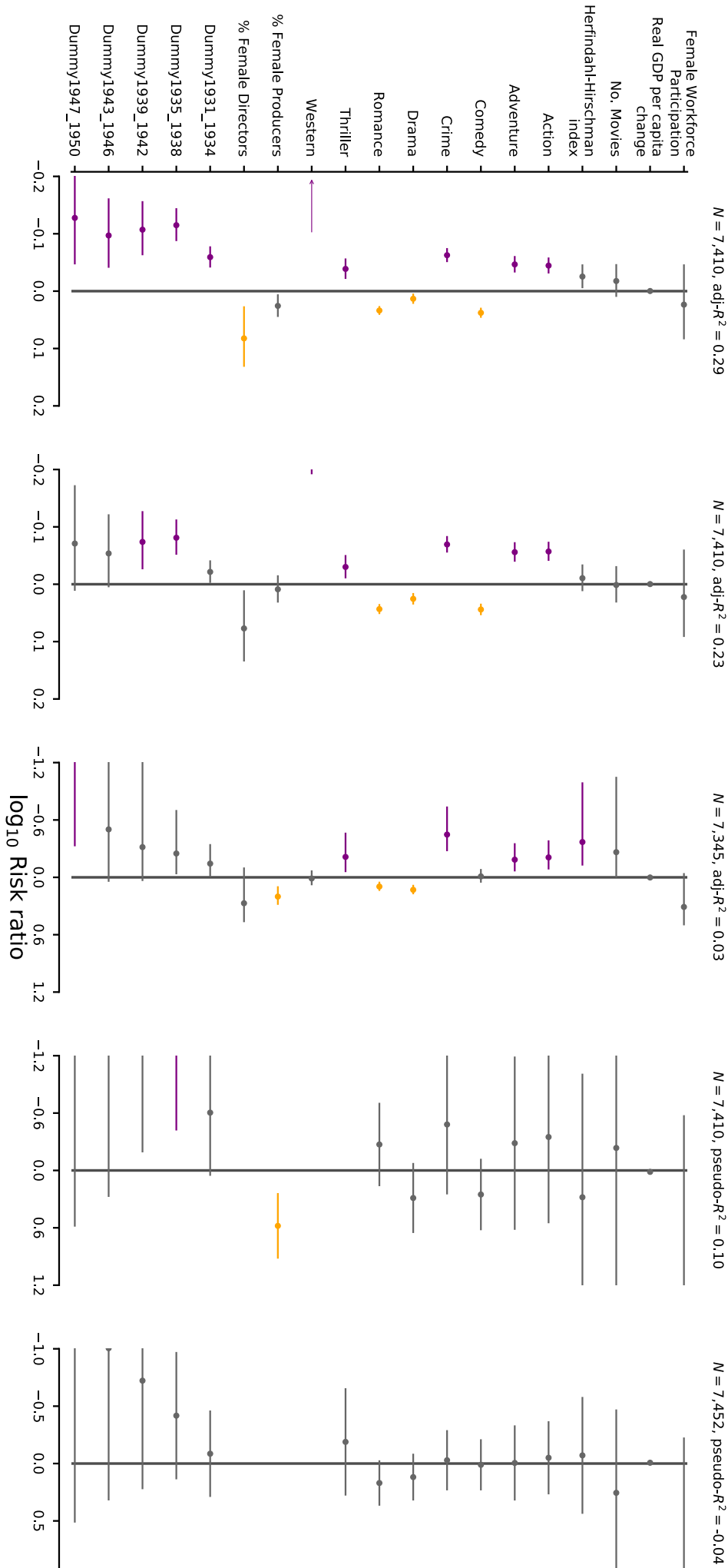

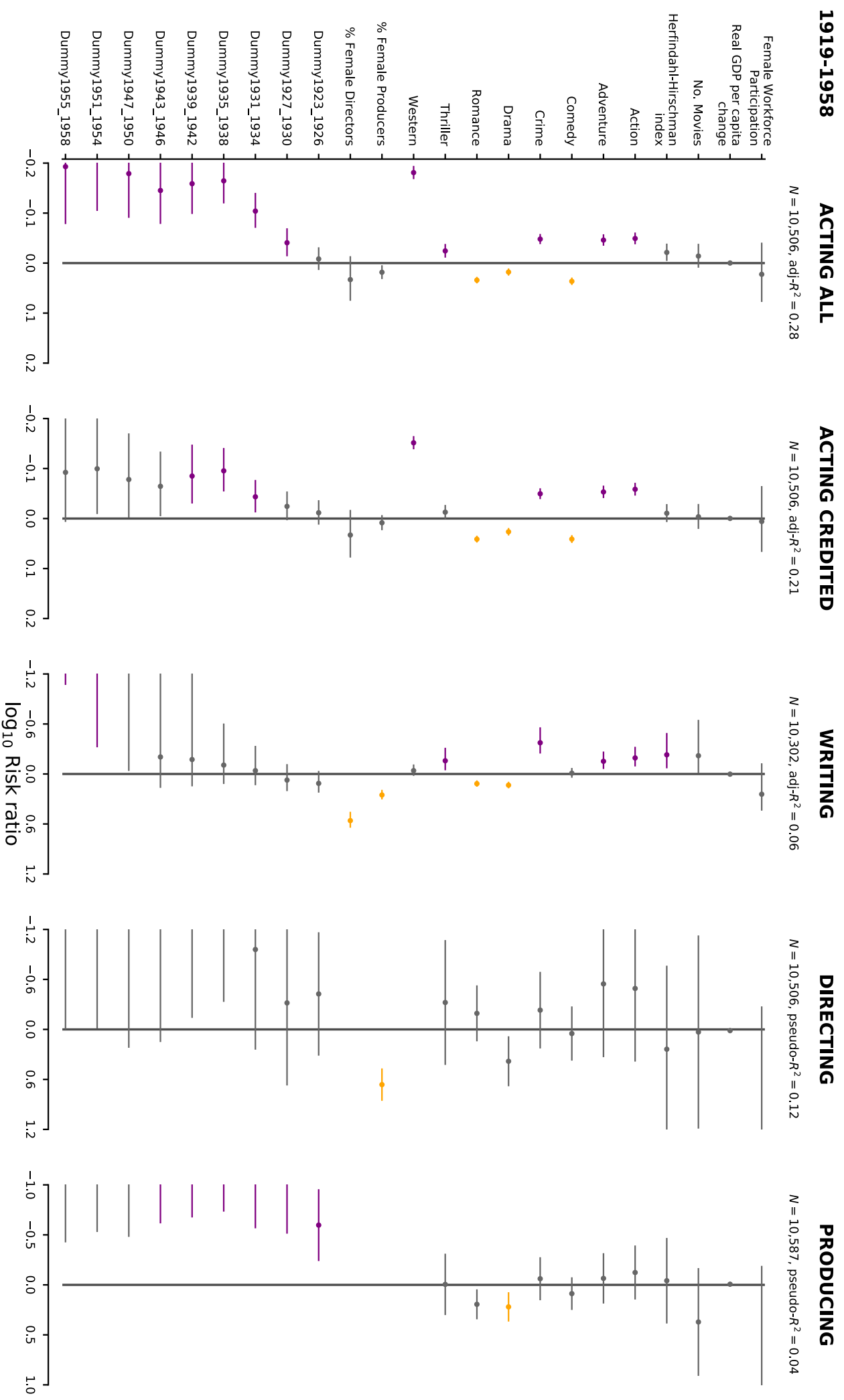

Supplement: S6 Fig — We consider 4-year time dummies in this case. (PDF) [file pone.0229662.s006.pdf]
