# Supplementary material for: Long-term patterns of gender imbalance in an industry without ability or level of interest differences
Source: PLoS One. 2020 Apr 1;15(4):e0229662. doi: 10.1371/journal.pone.0229662 (PMC7112163; doi:10.1371/journal.pone.0229662)
Supplement: S3 Fig — We do not include time dummies in our model. (PDF) [file pone.0229662.s003.pdf]

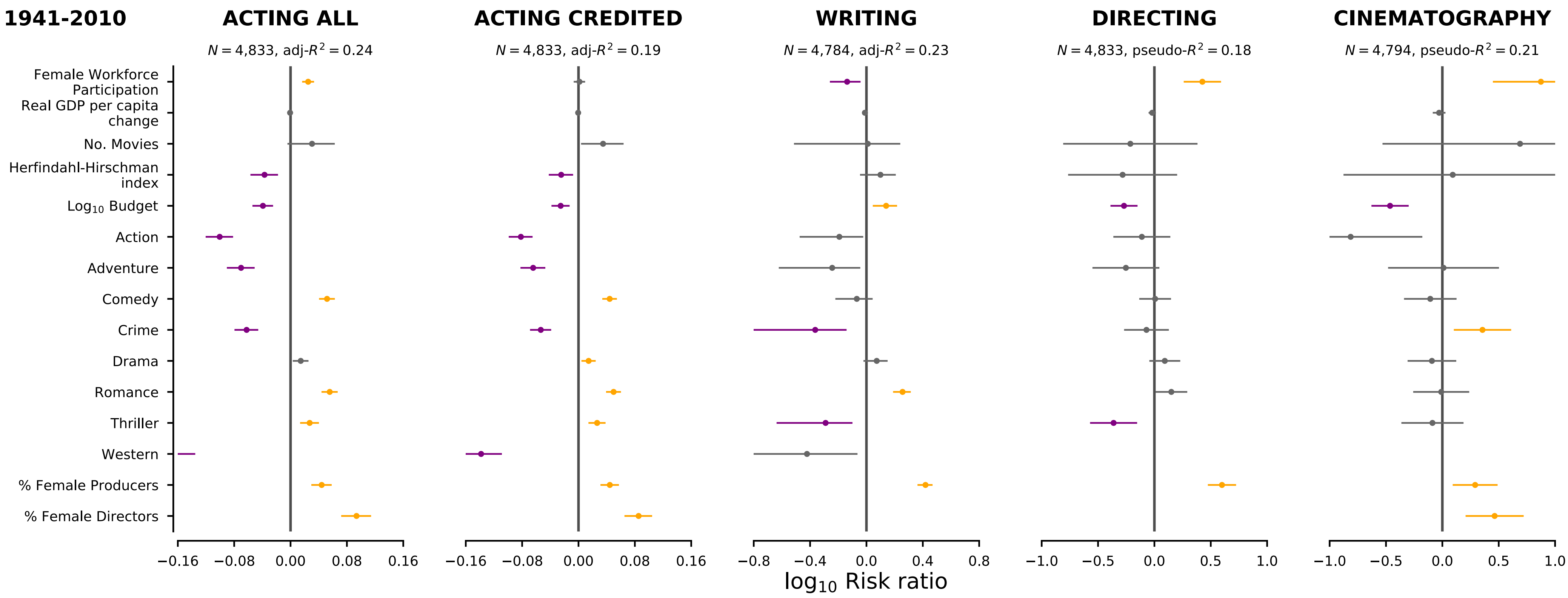
1941-2010
ACTING ALL
$N = 4{,}833$, adj-$R^2 = 0.24$
ACTING CREDITED
$N = 4{,}833$, adj-$R^2 = 0.19$
WRITING
$N = 4{,}784$, adj-$R^2 = 0.23$
DIRECTING
$N = 4{,}833$, pseudo-$R^2 = 0.18$
CINEMATOGRAPHY
$N = 4{,}794$, pseudo-$R^2 = 0.21$
Female Workforce Participation
Real GDP per capita change
No. Movies
Herfindahl-Hirschman index
$\log_{10}$ Budget
Action
Adventure
Comedy
Crime
Drama
Romance
Thriller
Western
% Female Producers
% Female Directors
−0.16 −0.08 0.00 0.08 0.16
−0.8 −0.4 0.0 0.4 0.8
−1.0 −0.5 0.0 0.5 1.0
$\log_{10}$ Risk ratio

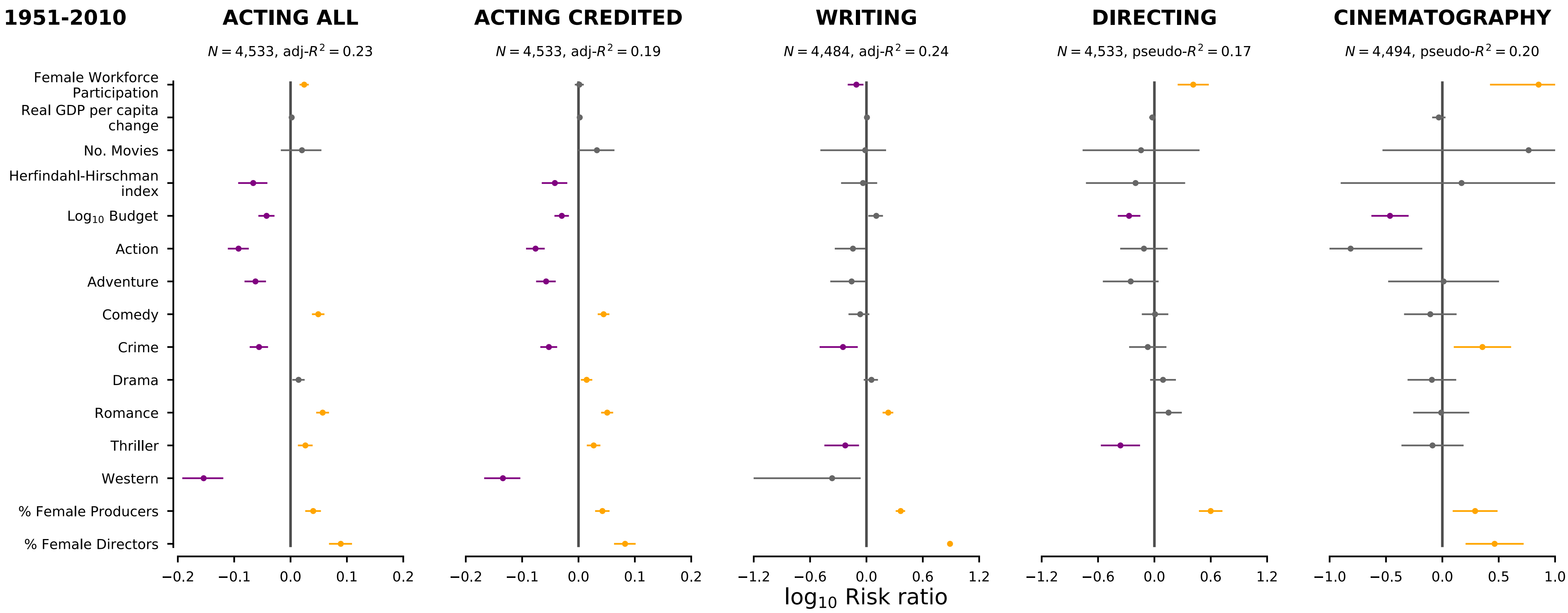
1951-2010
ACTING ALL
$N = 4{,}533$, adj-$R^2 = 0.23$
ACTING CREDITED
$N = 4{,}533$, adj-$R^2 = 0.19$
WRITING
$N = 4{,}484$, adj-$R^2 = 0.24$
DIRECTING
$N = 4{,}533$, pseudo-$R^2 = 0.17$
CINEMATOGRAPHY
$N = 4{,}494$, pseudo-$R^2 = 0.20$
Female Workforce Participation
Real GDP per capita change
No. Movies
Herfindahl-Hirschman index
$Log_{10}$ Budget
Action
Adventure
Comedy
Crime
Drama
Romance
Thriller
Western
% Female Producers
% Female Directors
−0.2 −0.1 0.0 0.1 0.2
−1.2 −0.6 0.0 0.6 1.2
−1.0 −0.5 0.0 0.5 1.0
$log_{10}$ Risk ratio

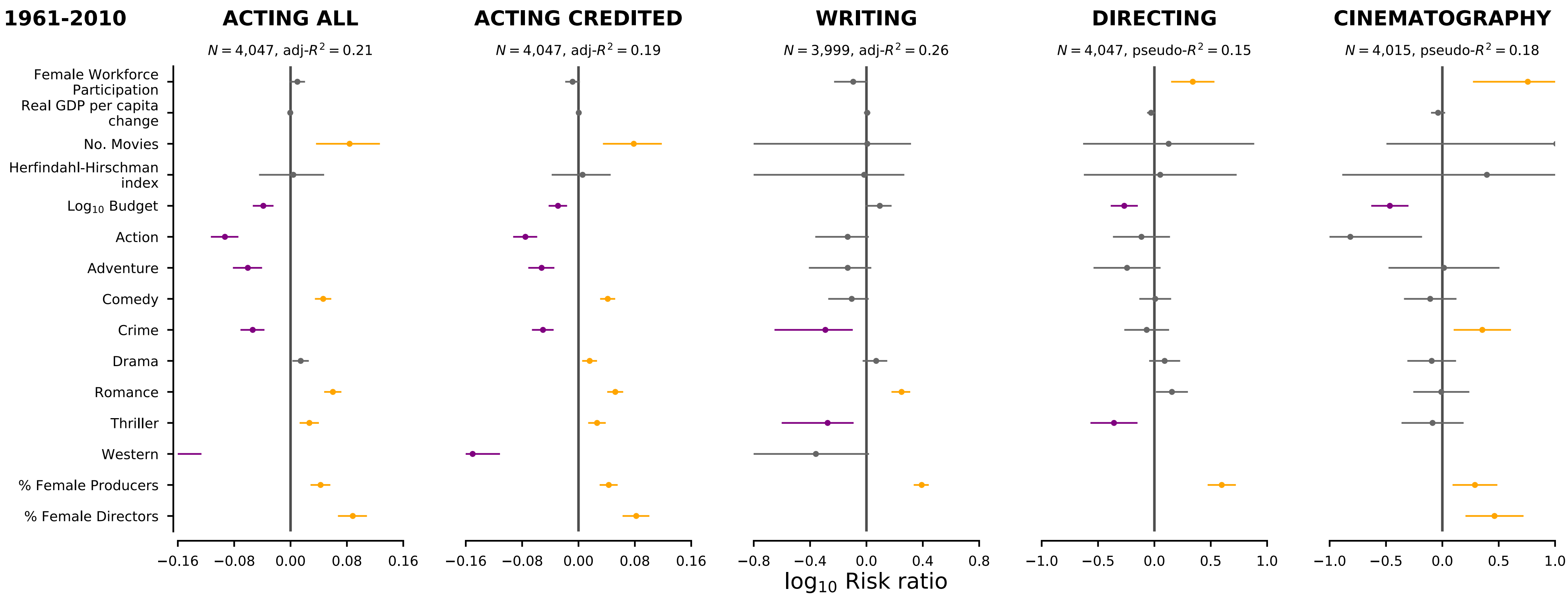

1961-2010
ACTING ALL
N = 4,047, adj-R² = 0.21
ACTING CREDITED
N = 4,047, adj-R² = 0.19
WRITING
N = 3,999, adj-R² = 0.26
DIRECTING
N = 4,047, pseudo-R² = 0.15
CINEMATOGRAPHY
N = 4,015, pseudo-R² = 0.18
Female Workforce Participation
Real GDP per capita change
No. Movies
Herfindahl-Hirschman index
Log10 Budget
Action
Adventure
Comedy
Crime
Drama
Romance
Thriller
Western
% Female Producers
% Female Directors
−0.16 −0.08 0.00 0.08 0.16
−0.8 −0.4 0.0 0.4 0.8
−1.0 −0.5 0.0 0.5 1.0
log10 Risk ratio

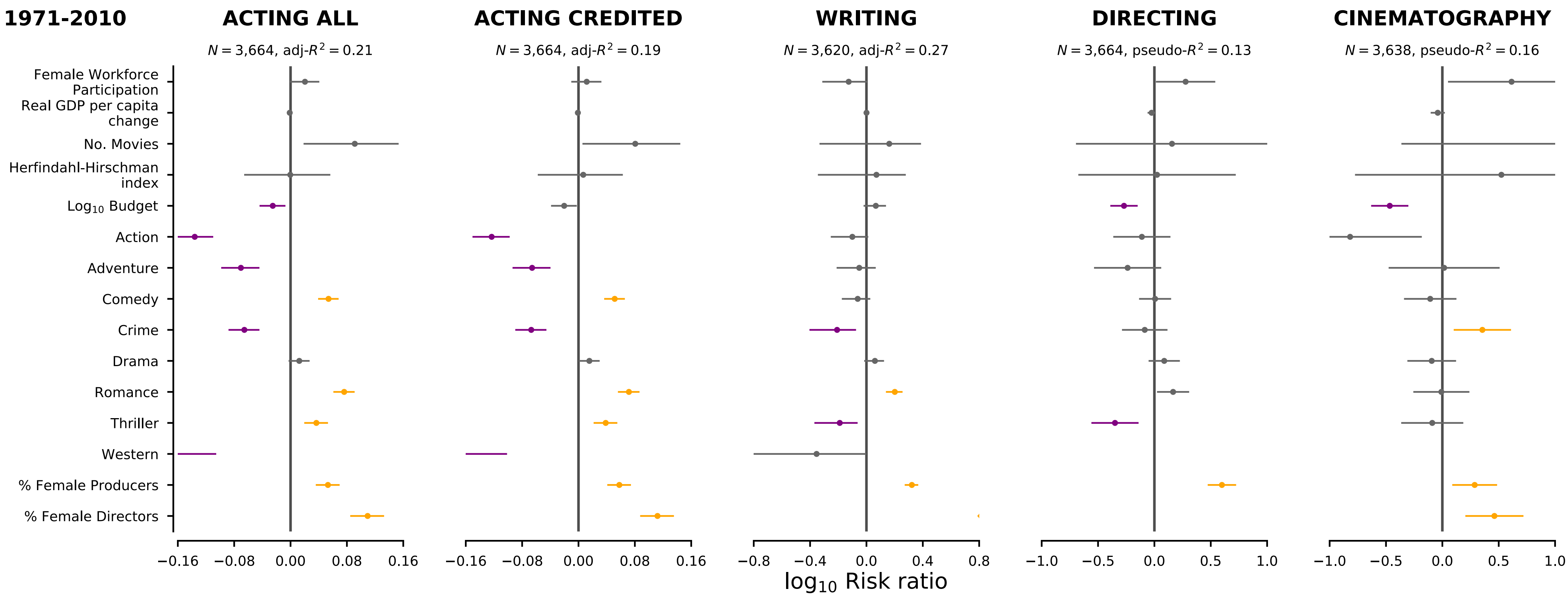
1971-2010
ACTING ALL
N = 3,664, adj-R² = 0.21
ACTING CREDITED
N = 3,664, adj-R² = 0.19
WRITING
N = 3,620, adj-R² = 0.27
DIRECTING
N = 3,664, pseudo-R² = 0.13
CINEMATOGRAPHY
N = 3,638, pseudo-R² = 0.16
Female Workforce Participation
Real GDP per capita change
No. Movies
Herfindahl-Hirschman index
Log10 Budget
Action
Adventure
Comedy
Crime
Drama
Romance
Thriller
Western
% Female Producers
% Female Directors
−0.16
−0.08
0.00
0.08
0.16
−0.8
−0.4
0.0
0.4
0.8
−1.0
−0.5
0.5
1.0
log10 Risk ratio

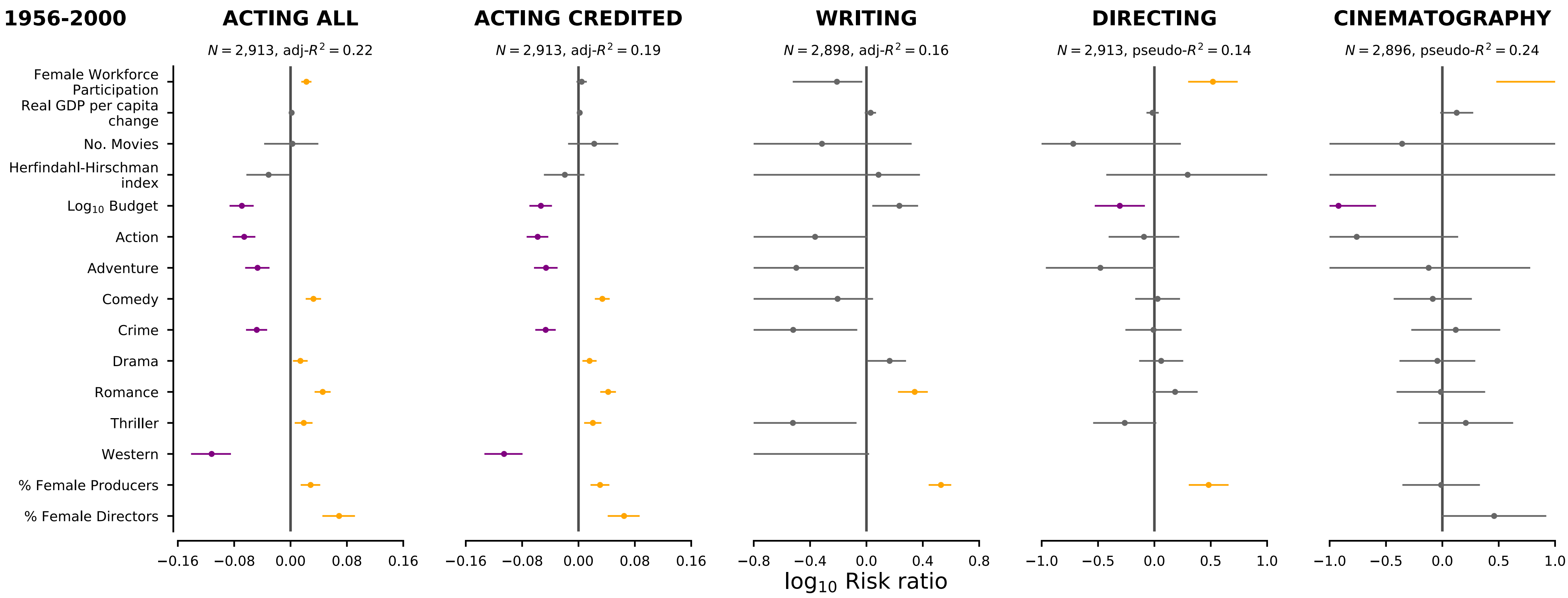

1956-2000
ACTING ALL
N = 2,913, adj-R² = 0.22
ACTING CREDITED
N = 2,913, adj-R² = 0.19
WRITING
N = 2,898, adj-R² = 0.16
DIRECTING
N = 2,913, pseudo-R² = 0.14
CINEMATOGRAPHY
N = 2,896, pseudo-R² = 0.24
Female Workforce Participation
Real GDP per capita change
No. Movies
Herfindahl-Hirschman index
Log10 Budget
Action
Adventure
Comedy
Crime
Drama
Romance
Thriller
Western
% Female Producers
% Female Directors
−0.16
−0.08
0.00
0.08
0.16
−0.8
−0.4
0.0
0.4
0.8
−1.0
−0.5
0.5
1.0
log10 Risk ratio
