# Supplementary material for: Long-term patterns of gender imbalance in an industry without ability or level of interest differences
Source: PLoS One. 2020 Apr 1;15(4):e0229662. doi: 10.1371/journal.pone.0229662 (PMC7112163; doi:10.1371/journal.pone.0229662)
Supplement: S4 Fig — We consider 1-year time dummies in this case. Note that for directors the maximum number of iterations (35) was exceeded before the convergence criterion was reached. This is due to the time dummies whose estimation uncertainty is enormous and destabilizes the fit. We do not show results for cinematographers because the time dummies make the matrix singular. (PDF) [file pone.0229662.s004.pdf]

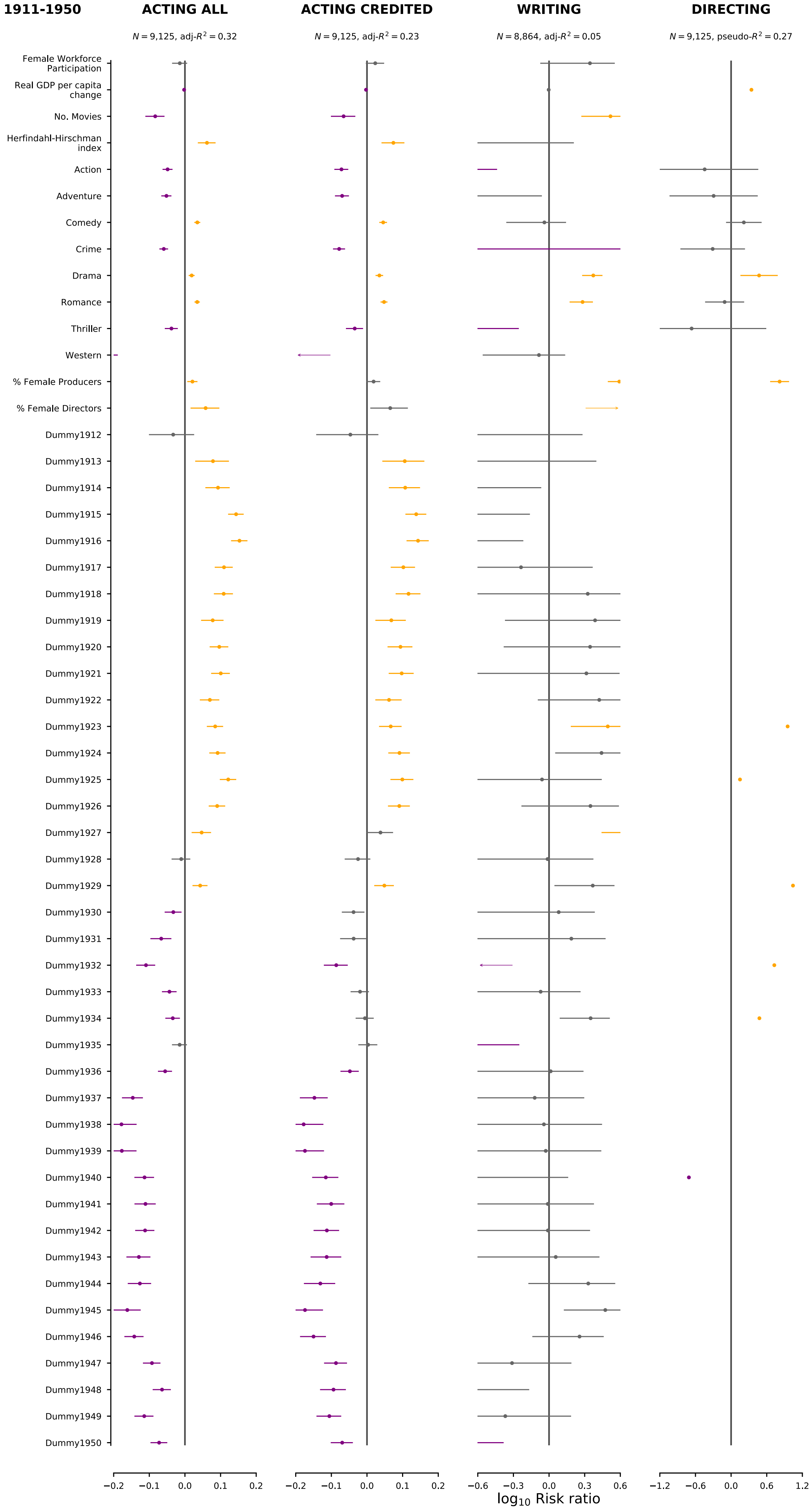

1911-1950
ACTING ALL
$N = 9{,}125$, adj-$R^2 = 0.32$
ACTING CREDITED
$N = 9{,}125$, adj-$R^2 = 0.23$
WRITING
$N = 8{,}864$, adj-$R^2 = 0.05$
DIRECTING
$N = 9{,}125$, pseudo-$R^2 = 0.27$
Female Workforce Participation
Real GDP per capita change
No. Movies
Herfindahl-Hirschman index
Action
Adventure
Comedy
Crime
Drama
Romance
Thriller
Western
% Female Producers
% Female Directors
Dummy1912
Dummy1913
Dummy1914
Dummy1915
Dummy1916
Dummy1917
Dummy1918
Dummy1919
Dummy1920
Dummy1921
Dummy1922
Dummy1923
Dummy1924
Dummy1925
Dummy1926
Dummy1927
Dummy1928
Dummy1929
Dummy1930
Dummy1931
Dummy1932
Dummy1933
Dummy1934
Dummy1935
Dummy1936
Dummy1937
Dummy1938
Dummy1939
Dummy1940
Dummy1941
Dummy1942
Dummy1943
Dummy1944
Dummy1945
Dummy1946
Dummy1947
Dummy1948
Dummy1949
Dummy1950
−0.2 −0.1 0.0 0.1 0.2
−0.2 −0.1 0.0 0.1 0.2
−0.6 −0.3 0.0 0.3 0.6
−1.2 −0.6 0.0 0.6 1.2
$\log_{10}$ Risk ratio

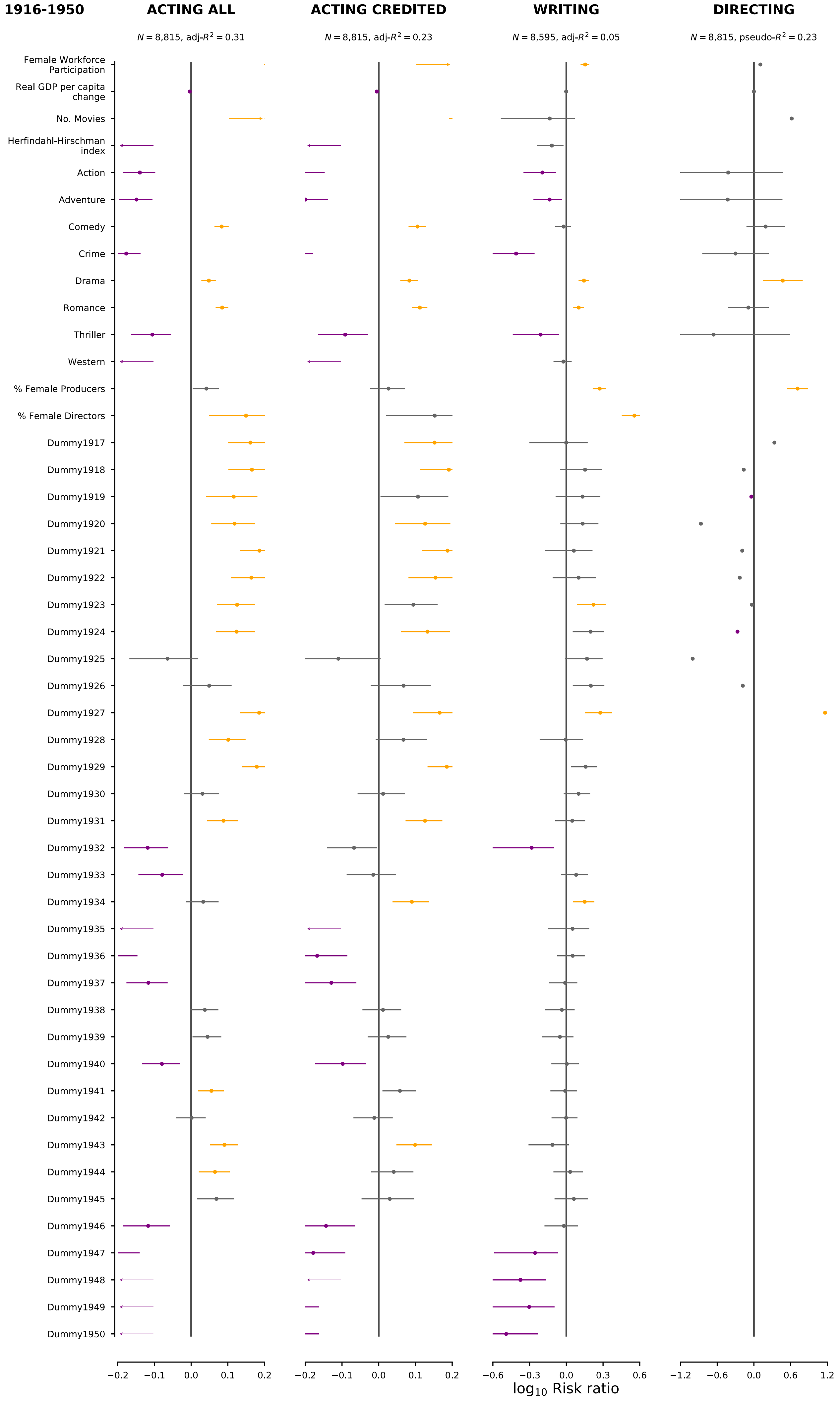

1916-1950
ACTING ALL
ACTING CREDITED
WRITING
DIRECTING
$N = 8{,}815$, adj-$R^2 = 0.31$
$N = 8{,}815$, adj-$R^2 = 0.23$
$N = 8{,}595$, adj-$R^2 = 0.05$
$N = 8{,}815$, pseudo-$R^2 = 0.23$
Female Workforce Participation
Real GDP per capita change
No. Movies
Herfindahl-Hirschman index
Action
Adventure
Comedy
Crime
Drama
Romance
Thriller
Western
% Female Producers
% Female Directors
Dummy1917
Dummy1918
Dummy1919
Dummy1920
Dummy1921
Dummy1922
Dummy1923
Dummy1924
Dummy1925
Dummy1926
Dummy1927
Dummy1928
Dummy1929
Dummy1930
Dummy1931
Dummy1932
Dummy1933
Dummy1934
Dummy1935
Dummy1936
Dummy1937
Dummy1938
Dummy1939
Dummy1940
Dummy1941
Dummy1942
Dummy1943
Dummy1944
Dummy1945
Dummy1946
Dummy1947
Dummy1948
Dummy1949
Dummy1950
−0.2 −0.1 0.0 0.1 0.2
−0.2 −0.1 0.0 0.1 0.2
−0.6 −0.3 0.0 0.3 0.6
−1.2 −0.6 0.0 0.6 1.2
$\log_{10}$ Risk ratio

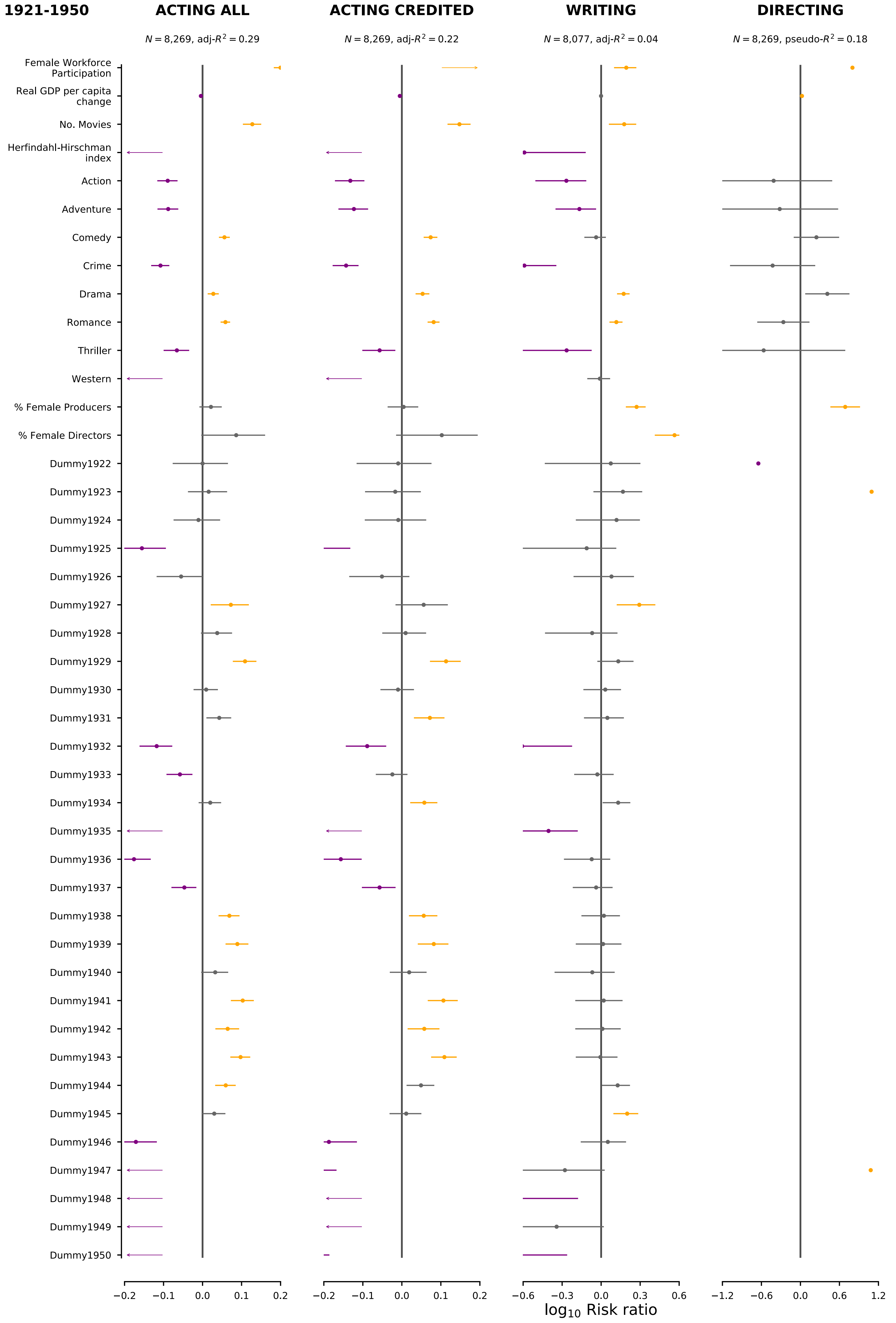
1921-1950
ACTING ALL
ACTING CREDITED
WRITING
DIRECTING
$N = 8{,}269$, adj-$R^2 = 0.29$
$N = 8{,}269$, adj-$R^2 = 0.22$
$N = 8{,}077$, adj-$R^2 = 0.04$
$N = 8{,}269$, pseudo-$R^2 = 0.18$
Female Workforce Participation
Real GDP per capita change
No. Movies
Herfindahl-Hirschman index
Action
Adventure
Comedy
Crime
Drama
Romance
Thriller
Western
% Female Producers
% Female Directors
Dummy1922
Dummy1923
Dummy1924
Dummy1925
Dummy1926
Dummy1927
Dummy1928
Dummy1929
Dummy1930
Dummy1931
Dummy1932
Dummy1933
Dummy1934
Dummy1935
Dummy1936
Dummy1937
Dummy1938
Dummy1939
Dummy1940
Dummy1941
Dummy1942
Dummy1943
Dummy1944
Dummy1945
Dummy1946
Dummy1947
Dummy1948
Dummy1949
Dummy1950
−0.2 −0.1 0.0 0.1 0.2
−0.2 −0.1 0.0 0.1 0.2
−0.6 −0.3 0.0 0.3 0.6
−1.2 −0.6 0.0 0.6 1.2
$\log_{10}$ Risk ratio

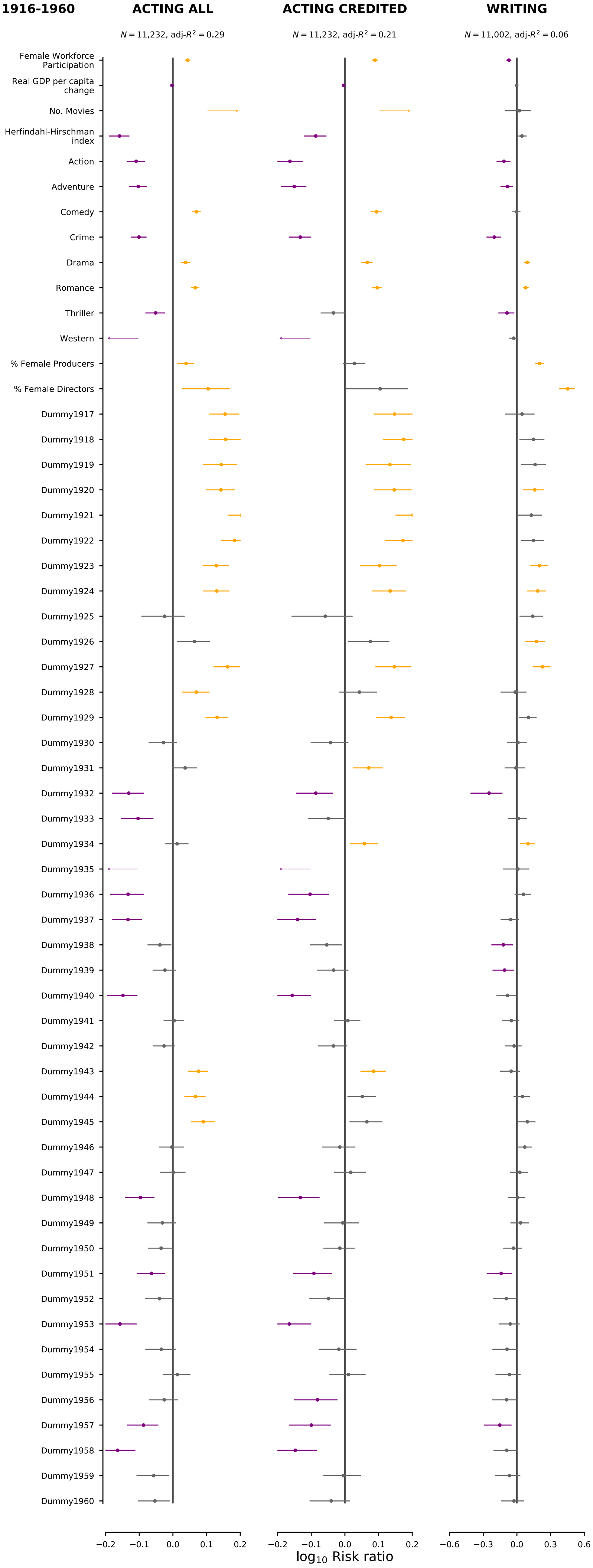
1916-1960
ACTING ALL
ACTING CREDITED
WRITING
N = 11,232, adj-R² = 0.29
N = 11,232, adj-R² = 0.21
N = 11,002, adj-R² = 0.06
Female Workforce Participation
Real GDP per capita change
No. Movies
Herfindahl-Hirschman index
Action
Adventure
Comedy
Crime
Drama
Romance
Thriller
Western
% Female Producers
% Female Directors
Dummy1917
Dummy1918
Dummy1919
Dummy1920
Dummy1921
Dummy1922
Dummy1923
Dummy1924
Dummy1925
Dummy1926
Dummy1927
Dummy1928
Dummy1929
Dummy1930
Dummy1931
Dummy1932
Dummy1933
Dummy1934
Dummy1935
Dummy1936
Dummy1937
Dummy1938
Dummy1939
Dummy1940
Dummy1941
Dummy1942
Dummy1943
Dummy1944
Dummy1945
Dummy1946
Dummy1947
Dummy1948
Dummy1949
Dummy1950
Dummy1951
Dummy1952
Dummy1953
Dummy1954
Dummy1955
Dummy1956
Dummy1957
Dummy1958
Dummy1959
Dummy1960
−0.2 −0.1 0.0 0.1 0.2
−0.2 −0.1 0.0 0.1 0.2
−0.6 −0.3 0.0 0.3 0.6
log10 Risk ratio
