# Supplementary material for: Long-term patterns of gender imbalance in an industry without ability or level of interest differences
Source: PLoS One. 2020 Apr 1;15(4):e0229662. doi: 10.1371/journal.pone.0229662 (PMC7112163; doi:10.1371/journal.pone.0229662)
Supplement: S5 Fig — We consider 1-year time dummies in this case. Note that for directors the maximum number of iterations (35) was exceeded before the convergence criterion was reached. This is due to the time dummies coefficients whose estimation uncertainty is enormous and destabilizes the fit. We do not show results for cinematographers because the time dummies make the matrix singular. (PDF) [file pone.0229662.s005.pdf]

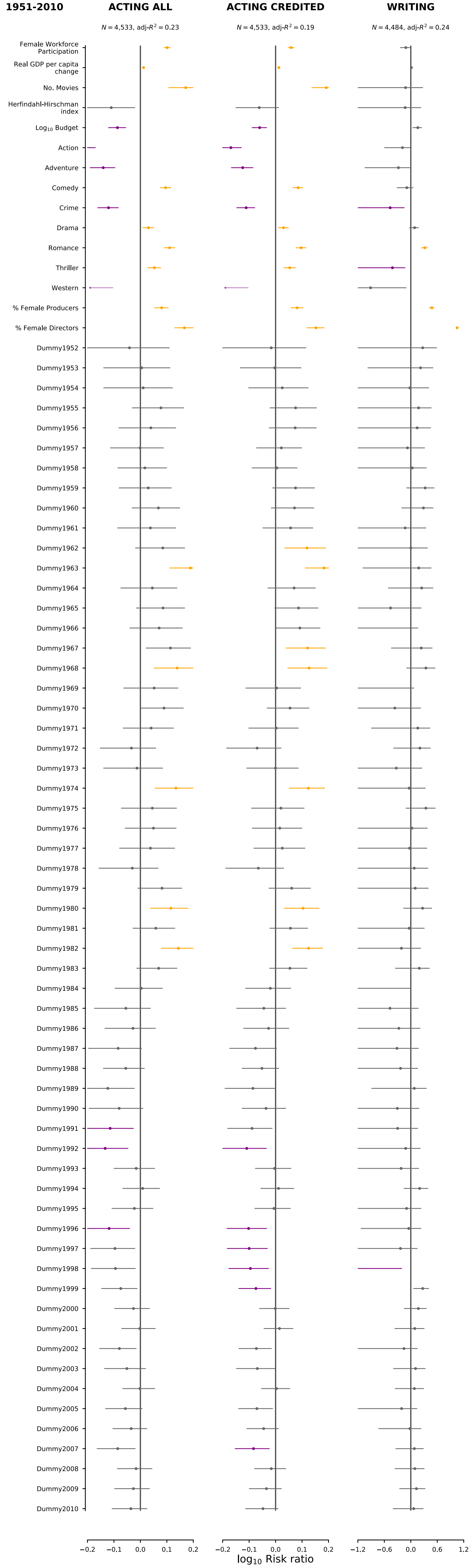

1951-2010
ACTING ALL
ACTING CREDITED
WRITING
$N = 4{,}533$, adj-$R^2 = 0.23$
$N = 4{,}533$, adj-$R^2 = 0.19$
$N = 4{,}484$, adj-$R^2 = 0.24$
Female Workforce Participation
Real GDP per capita change
No. Movies
Herfindahl-Hirschman index
$\log_{10}$ Budget
Action
Adventure
Comedy
Crime
Drama
Romance
Thriller
Western
% Female Producers
% Female Directors
Dummy1952
Dummy1953
Dummy1954
Dummy1955
Dummy1956
Dummy1957
Dummy1958
Dummy1959
Dummy1960
Dummy1961
Dummy1962
Dummy1963
Dummy1964
Dummy1965
Dummy1966
Dummy1967
Dummy1968
Dummy1969
Dummy1970
Dummy1971
Dummy1972
Dummy1973
Dummy1974
Dummy1975
Dummy1976
Dummy1977
Dummy1978
Dummy1979
Dummy1980
Dummy1981
Dummy1982
Dummy1983
Dummy1984
Dummy1985
Dummy1986
Dummy1987
Dummy1988
Dummy1989
Dummy1990
Dummy1991
Dummy1992
Dummy1993
Dummy1994
Dummy1995
Dummy1996
Dummy1997
Dummy1998
Dummy1999
Dummy2000
Dummy2001
Dummy2002
Dummy2003
Dummy2004
Dummy2005
Dummy2006
Dummy2007
Dummy2008
Dummy2009
Dummy2010
−0.2 −0.1 0.0 0.1 0.2
−0.2 −0.1 0.0 0.1 0.2
−1.2 −0.6 0.0 0.6 1.2
$\log_{10}$ Risk ratio

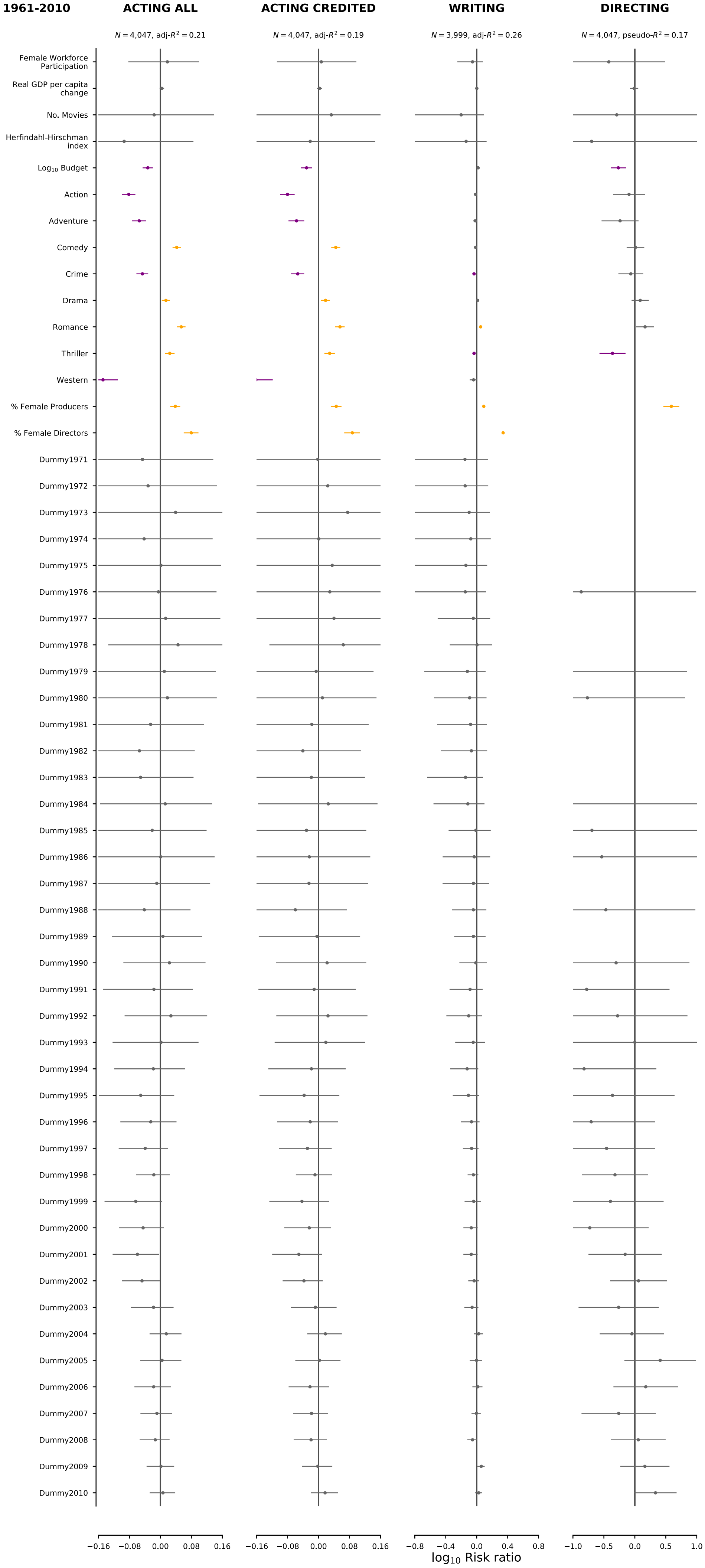

1961-2010
ACTING ALL
ACTING CREDITED
WRITING
DIRECTING
$N = 4{,}047$, adj-$R^2 = 0.21$
$N = 4{,}047$, adj-$R^2 = 0.19$
$N = 3{,}999$, adj-$R^2 = 0.26$
$N = 4{,}047$, pseudo-$R^2 = 0.17$
Female Workforce Participation
Real GDP per capita change
No. Movies
Herfindahl-Hirschman index
Log_{10} Budget
Action
Adventure
Comedy
Crime
Drama
Romance
Thriller
Western
% Female Producers
% Female Directors
Dummy1971
Dummy1972
Dummy1973
Dummy1974
Dummy1975
Dummy1976
Dummy1977
Dummy1978
Dummy1979
Dummy1980
Dummy1981
Dummy1982
Dummy1983
Dummy1984
Dummy1985
Dummy1986
Dummy1987
Dummy1988
Dummy1989
Dummy1990
Dummy1991
Dummy1992
Dummy1993
Dummy1994
Dummy1995
Dummy1996
Dummy1997
Dummy1998
Dummy1999
Dummy2000
Dummy2001
Dummy2002
Dummy2003
Dummy2004
Dummy2005
Dummy2006
Dummy2007
Dummy2008
Dummy2009
Dummy2010
−0.16 −0.08 0.00 0.08 0.16
−0.16 −0.08 0.00 0.08 0.16
−0.8 −0.4 0.0 0.4 0.8
−1.0 −0.5 0.0 0.5 1.0
$\log_{10}$ Risk ratio

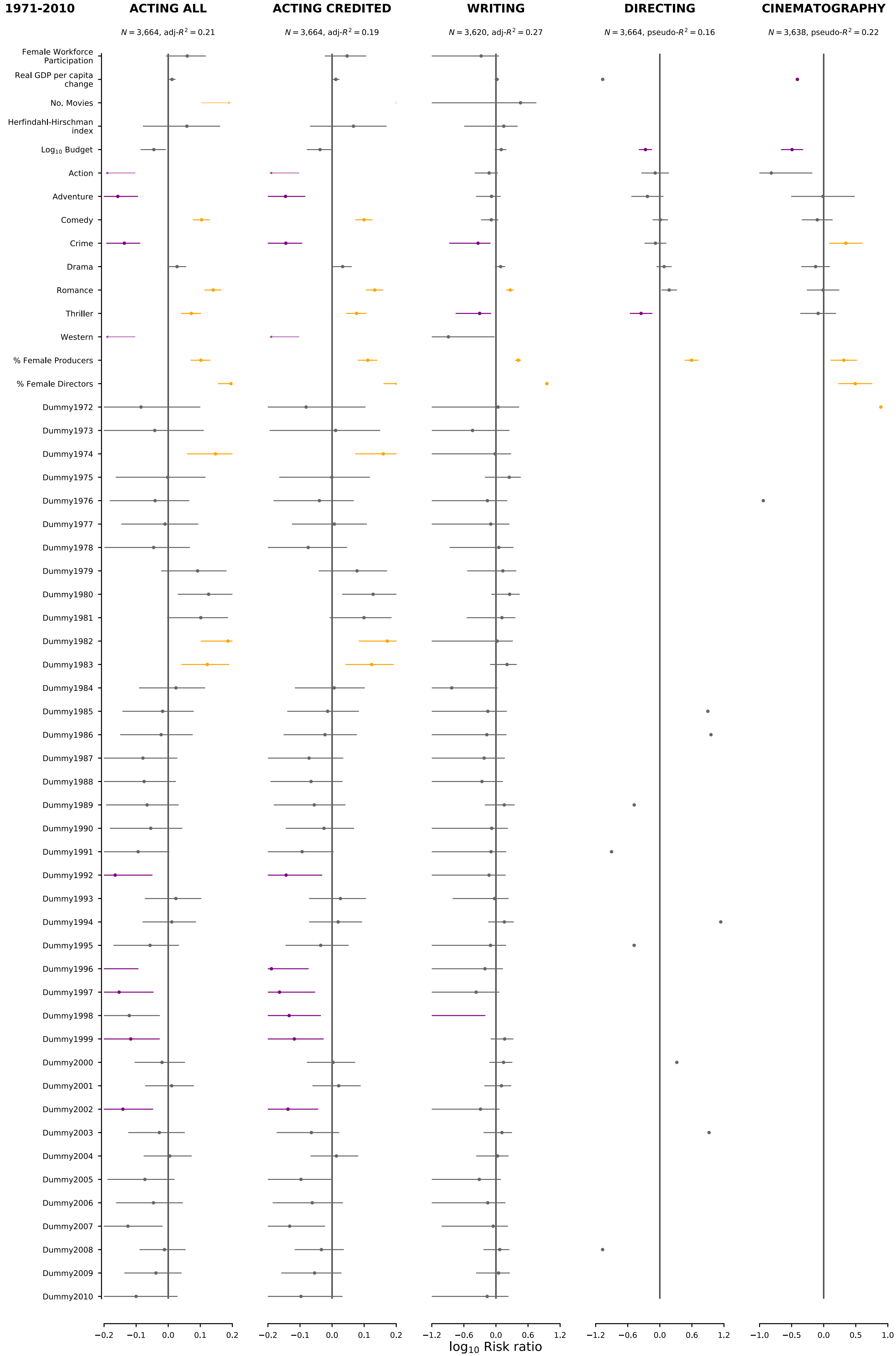

1971-2010
ACTING ALL
ACTING CREDITED
WRITING
DIRECTING
CINEMATOGRAPHY
N = 3,664, adj-R² = 0.21
N = 3,664, adj-R² = 0.19
N = 3,620, adj-R² = 0.27
N = 3,664, pseudo-R² = 0.16
N = 3,638, pseudo-R² = 0.22
Female Workforce Participation
Real GDP per capita change
No. Movies
Herfindahl-Hirschman index
Log10 Budget
Action
Adventure
Comedy
Crime
Drama
Romance
Thriller
Western
% Female Producers
% Female Directors
Dummy1972
Dummy1973
Dummy1974
Dummy1975
Dummy1976
Dummy1977
Dummy1978
Dummy1979
Dummy1980
Dummy1981
Dummy1982
Dummy1983
Dummy1984
Dummy1985
Dummy1986
Dummy1987
Dummy1988
Dummy1989
Dummy1990
Dummy1991
Dummy1992
Dummy1993
Dummy1994
Dummy1995
Dummy1996
Dummy1997
Dummy1998
Dummy1999
Dummy2000
Dummy2001
Dummy2002
Dummy2003
Dummy2004
Dummy2005
Dummy2006
Dummy2007
Dummy2008
Dummy2009
Dummy2010
−0.2 −0.1 0.0 0.1 0.2
−0.2 −0.1 0.0 0.1 0.2
−1.2 −0.6 0.0 0.6 1.2
−1.2 −0.6 0.0 0.6 1.2
−1.0 −0.5 0.0 0.5 1.0
log10 Risk ratio

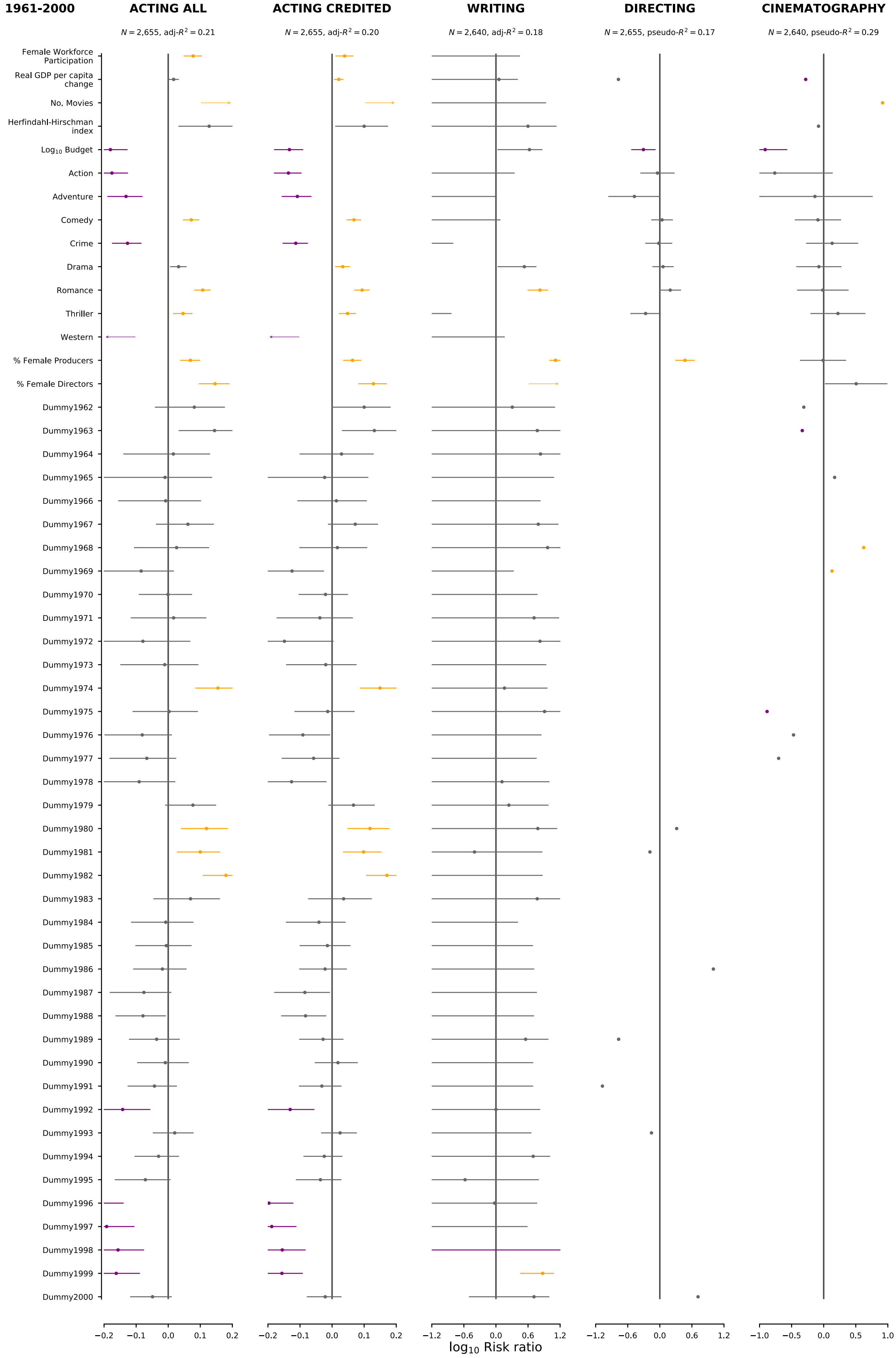

1961-2000
ACTING ALL
$N = 2{,}655$, adj-$R^2 = 0.21$
ACTING CREDITED
$N = 2{,}655$, adj-$R^2 = 0.20$
WRITING
$N = 2{,}640$, adj-$R^2 = 0.18$
DIRECTING
$N = 2{,}655$, pseudo-$R^2 = 0.17$
CINEMATOGRAPHY
$N = 2{,}640$, pseudo-$R^2 = 0.29$
Female Workforce Participation
Real GDP per capita change
No. Movies
Herfindahl-Hirschman index
Log_{10} Budget
Action
Adventure
Comedy
Crime
Drama
Romance
Thriller
Western
% Female Producers
% Female Directors
Dummy1962
Dummy1963
Dummy1964
Dummy1965
Dummy1966
Dummy1967
Dummy1968
Dummy1969
Dummy1970
Dummy1971
Dummy1972
Dummy1973
Dummy1974
Dummy1975
Dummy1976
Dummy1977
Dummy1978
Dummy1979
Dummy1980
Dummy1981
Dummy1982
Dummy1983
Dummy1984
Dummy1985
Dummy1986
Dummy1987
Dummy1988
Dummy1989
Dummy1990
Dummy1991
Dummy1992
Dummy1993
Dummy1994
Dummy1995
Dummy1996
Dummy1997
Dummy1998
Dummy1999
Dummy2000
−0.2 −0.1 0.0 0.1 0.2
−0.2 −0.1 0.0 0.1 0.2
−1.2 −0.6 0.0 0.6 1.2
−1.2 −0.6 0.0 0.6 1.2
−1.0 −0.5 0.0 0.5 1.0
log_{10} Risk ratio
