# Supplementary material for: Long-term patterns of gender imbalance in an industry without ability or level of interest differences
Source: PLoS One. 2020 Apr 1;15(4):e0229662. doi: 10.1371/journal.pone.0229662 (PMC7112163; doi:10.1371/journal.pone.0229662)
Supplement: S7 Fig — We consider 4-year time dummies in this case. (PDF) [file pone.0229662.s007.pdf]

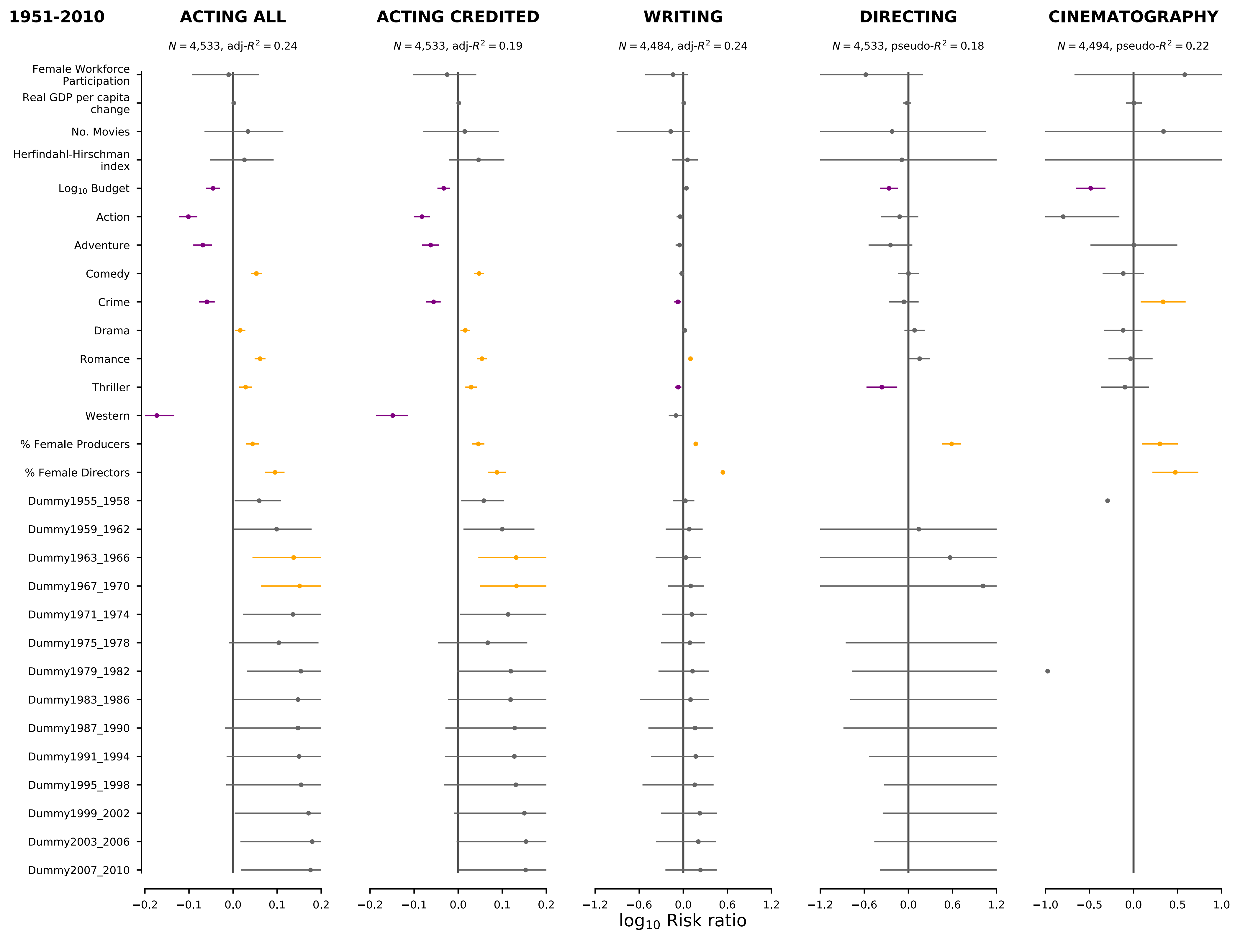
1951-2010
ACTING ALL
N = 4,533, adj-R2 = 0.24
ACTING CREDITED
N = 4,533, adj-R2 = 0.19
WRITING
N = 4,484, adj-R2 = 0.24
DIRECTING
N = 4,533, pseudo-R2 = 0.18
CINEMATOGRAPHY
N = 4,494, pseudo-R2 = 0.22
Female Workforce Participation
Real GDP per capita change
No. Movies
Herfindahl-Hirschman index
Log10 Budget
Action
Adventure
Comedy
Crime
Drama
Romance
Thriller
Western
% Female Producers
% Female Directors
Dummy1955_1958
Dummy1959_1962
Dummy1963_1966
Dummy1967_1970
Dummy1971_1974
Dummy1975_1978
Dummy1979_1982
Dummy1983_1986
Dummy1987_1990
Dummy1991_1994
Dummy1995_1998
Dummy1999_2002
Dummy2003_2006
Dummy2007_2010
−0.2 −0.1 0.0 0.1 0.2
−0.2 −0.1 0.0 0.1 0.2
−1.2 −0.6 0.0 0.6 1.2
−1.2 −0.6 0.0 0.6 1.2
−1.0 −0.5 0.0 0.5 1.0
log10 Risk ratio

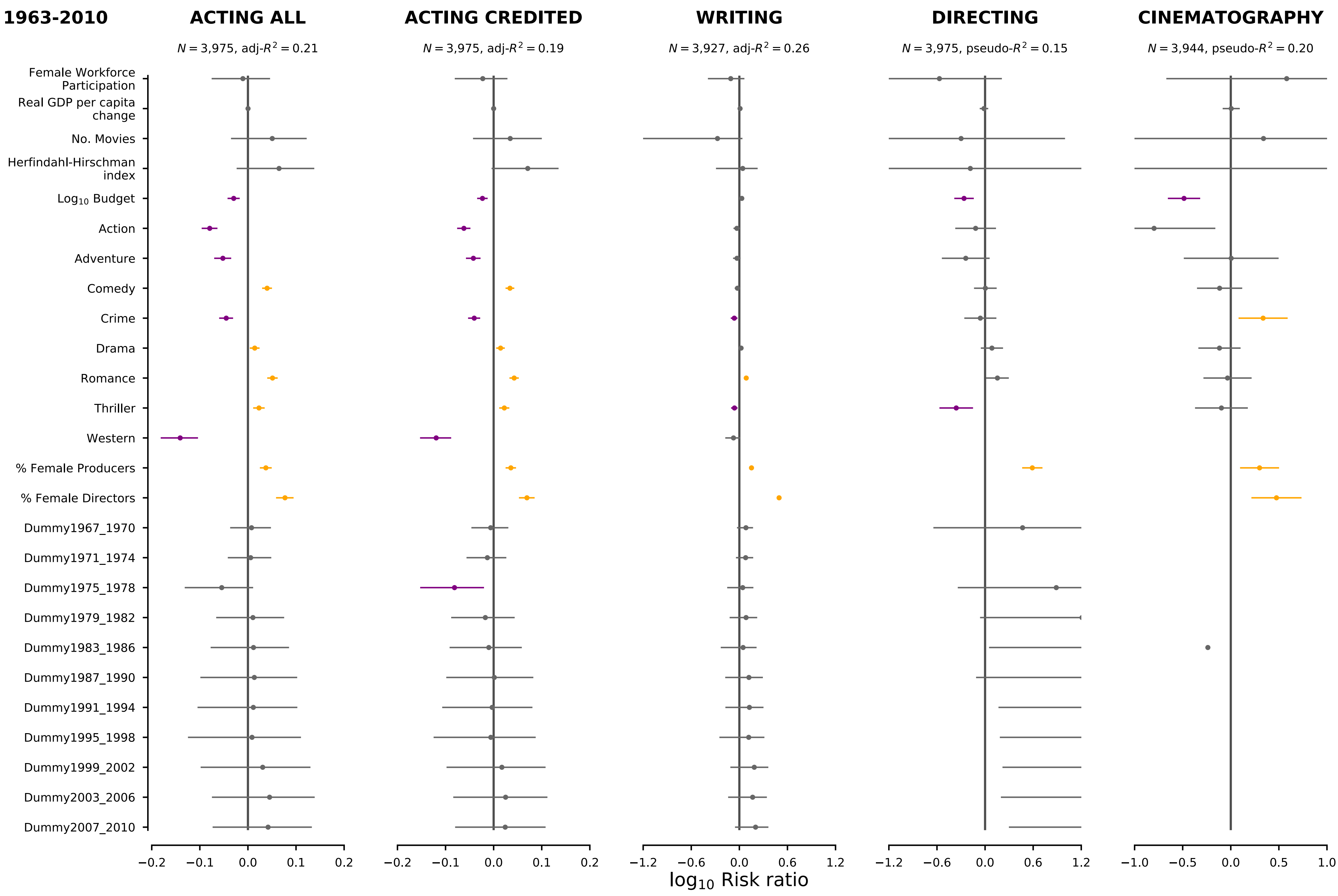
1963-2010
ACTING ALL
N = 3,975, adj-R² = 0.21
ACTING CREDITED
N = 3,975, adj-R² = 0.19
WRITING
N = 3,927, adj-R² = 0.26
DIRECTING
N = 3,975, pseudo-R² = 0.15
CINEMATOGRAPHY
N = 3,944, pseudo-R² = 0.20
Female Workforce Participation
Real GDP per capita change
No. Movies
Herfindahl-Hirschman index
Log10 Budget
Action
Adventure
Comedy
Crime
Drama
Romance
Thriller
Western
% Female Producers
% Female Directors
Dummy1967_1970
Dummy1971_1974
Dummy1975_1978
Dummy1979_1982
Dummy1983_1986
Dummy1987_1990
Dummy1991_1994
Dummy1995_1998
Dummy1999_2002
Dummy2003_2006
Dummy2007_2010
−0.2 −0.1 0.0 0.1 0.2
−1.2 −0.6 0.0 0.6 1.2
−1.0 −0.5 0.0 0.5 1.0
log10 Risk ratio

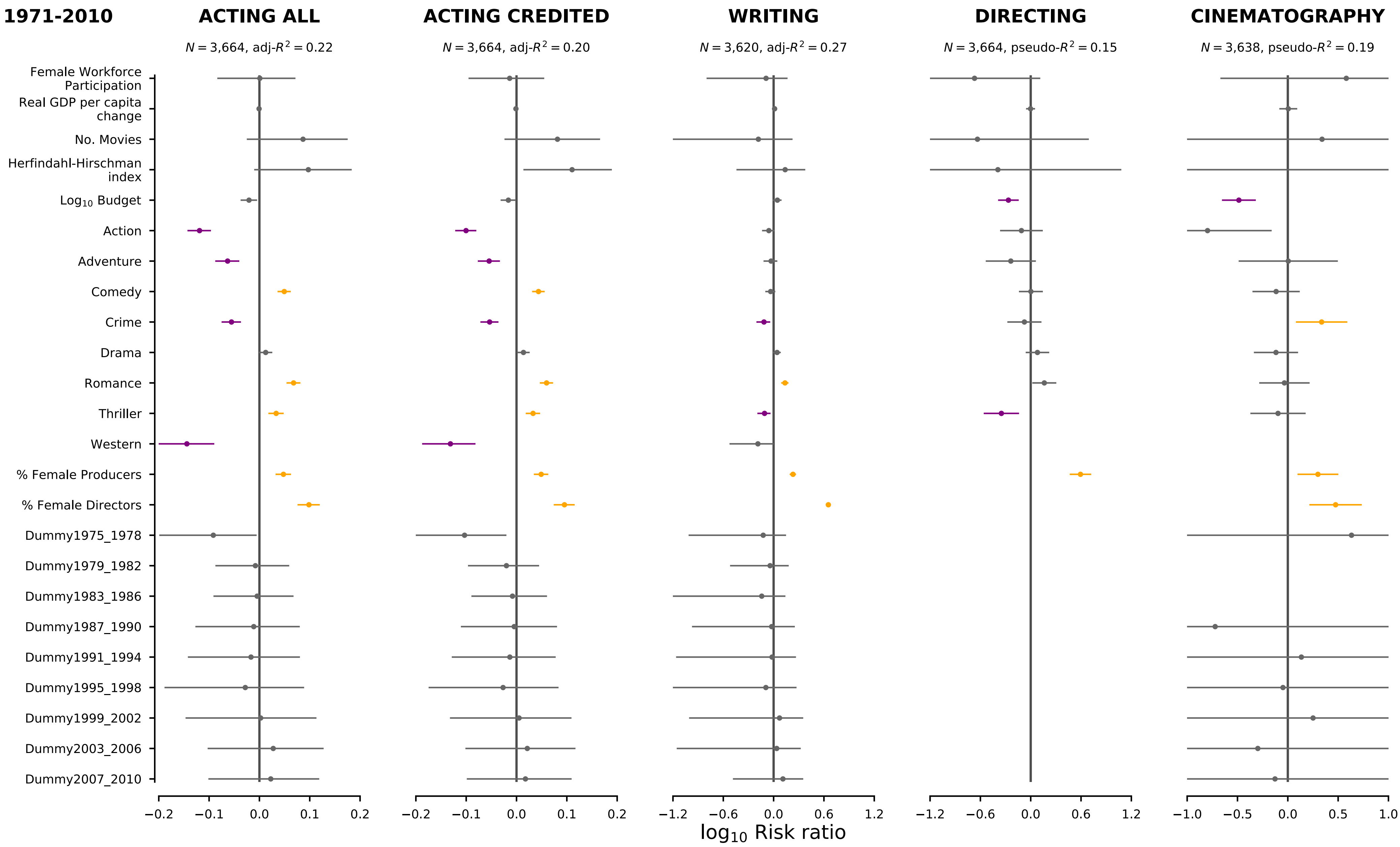
1971-2010
ACTING ALL
N = 3,664, adj-R² = 0.22
ACTING CREDITED
N = 3,664, adj-R² = 0.20
WRITING
N = 3,620, adj-R² = 0.27
DIRECTING
N = 3,664, pseudo-R² = 0.15
CINEMATOGRAPHY
N = 3,638, pseudo-R² = 0.19
Female Workforce Participation
Real GDP per capita change
No. Movies
Herfindahl-Hirschman index
Log10 Budget
Action
Adventure
Comedy
Crime
Drama
Romance
Thriller
Western
% Female Producers
% Female Directors
Dummy1975_1978
Dummy1979_1982
Dummy1983_1986
Dummy1987_1990
Dummy1991_1994
Dummy1995_1998
Dummy1999_2002
Dummy2003_2006
Dummy2007_2010
−0.2 −0.1 0.0 0.1 0.2
−1.2 −0.6 0.0 0.6 1.2
−1.0 −0.5 0.0 0.5 1.0
log10 Risk ratio

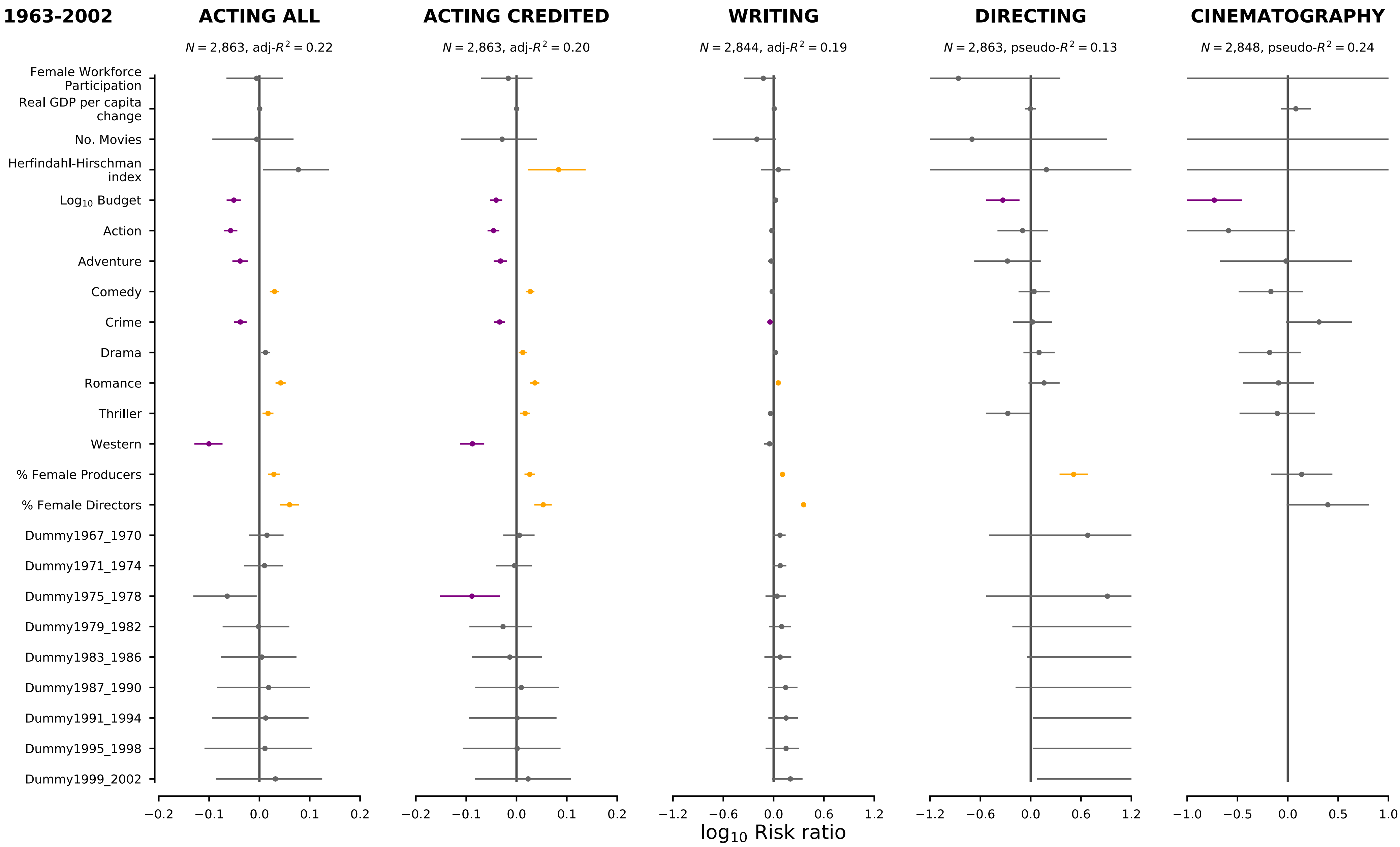
1963-2002
ACTING ALL
N = 2,863, adj-R² = 0.22
ACTING CREDITED
N = 2,863, adj-R² = 0.20
WRITING
N = 2,844, adj-R² = 0.19
DIRECTING
N = 2,863, pseudo-R² = 0.13
CINEMATOGRAPHY
N = 2,848, pseudo-R² = 0.24
Female Workforce Participation
Real GDP per capita change
No. Movies
Herfindahl-Hirschman index
Log10 Budget
Action
Adventure
Comedy
Crime
Drama
Romance
Thriller
Western
% Female Producers
% Female Directors
Dummy1967_1970
Dummy1971_1974
Dummy1975_1978
Dummy1979_1982
Dummy1983_1986
Dummy1987_1990
Dummy1991_1994
Dummy1995_1998
Dummy1999_2002
−0.2 −0.1 0.0 0.1 0.2
−1.2 −0.6 0.0 0.6 1.2
−1.0 −0.5 0.0 0.5 1.0
log10 Risk ratio
