# Supplementary material for: Long-term patterns of gender imbalance in an industry without ability or level of interest differences
Source: PLoS One. 2020 Apr 1;15(4):e0229662. doi: 10.1371/journal.pone.0229662 (PMC7112163; doi:10.1371/journal.pone.0229662)
Supplement: S2 File — (PDF) [file pone.0229662.s009.pdf]

AFI id	AFI year	IMDb year	AFI title	IMDb title	AFI director	IMDb director	IMDb id
1746584115	1938	1938	He Couldn't Say No	He Couldn't Say No	Seiler, Lew	Lewis Seiler	tt0030220
1746577375	1950	1950	Operation Haylift	Operation Haylift	Berke, William	William Berke	tt0042811
1746585256	1929	1929	Brothers	Brothers	Pembroke, Scott	Scott Pembroke	tt0019732
1746491880	1944	1944	Dead Man's Eyes	Dead Man's Eyes	LeBorg, Reginald	Reginald Le Borg	tt0036749
1746576720	1932	1932	The Hatchet Man	The Hatchet Man	Wellman, William A.	William A. Wellman	tt0022979
1746580531	1939	1939	At the Circus	At the Circus	Buzzell, Edward	Edward Buzzell	tt0031060
1746475271	1947	1947	Heaven Only Knows	Heaven Only Knows	Rogell, Albert S.	Albert S. Rogell	tt0039453
1746581852	1928	1928	Quick Triggers	Quick Triggers	Taylor, Ray	Ray Taylor	tt0019301
1746513091	1914	1914	The Ragged Earl	The Ragged Earl	Carleton, Lloyd B.	Lloyd B. Carleton	tt0004509
1746410689	1992	1992	Reservoir Dogs	Reservoir Dogs	Tarantino, Quentin	Quentin Tarantino	tt0105236
1746579027	1959	1959	The Truth About Fidel Castro Revolution	The Truth About Fidel Castro Revolution	Flynn, Errol	Errol Flynn	tt0321867
1746495762	1921	1921	A Trip to Paradise	A Trip to Paradise	Karger, Maxwell	Maxwell Karger	tt0012779
1746562312	1954	1954	Private Hell 36	Private Hell 36	Siegel, Don	Don Siegel	tt0047370
1746208587	1967	1967	Hombre	Hombre	Ritt, Martin	Martin Ritt	tt0061770
1745372631	2010	2009	I Love You Phillip Morris	I Love You Phillip Morris	Requa, John; Ficarra, Glenn	Glenn Ficarra	tt1045772
1746571106	1935	1935	Waterfront Lady	Waterfront Lady	Santley, Joseph	Joseph Santley	tt0027184
1746420551	1994	1994	Death Wish V: The Face of Death	Death Wish V: The Face of Death	Goldstein, Allan A.	Allan A. Goldstein	tt0109578
1746516004	1920	1920	Nothing But the Truth	Nothing But the Truth	Kirkland, David	David Kirkland	tt0196797
1746464910	1946	1946	The Time, the Place and the Girl	The Time, the Place and the Girl	Butler, David	David Butler	tt0039038
1746585227	1930	1930	A Lady's Morals	A Lady's Morals	Franklin, Sidney	Sidney Franklin	tt0021045
1746565494	1932	1932	Radio Patrol	Radio Patrol	Cahn, Edward L.	Edward L. Cahn	tt0023368
1746422653	1994	1994	Drop Squad	Drop Squad	Johnson, David Clark	David C. Johnson	tt0109675
1746475535	1947	1946	Winter Wonderland	Winter Wonderland	Vorhaus, Bernard	Bernard Vorhaus	tt0039997
1746578576	1960	1960	The Bellboy	The Bellboy	Lewis, Jerry	Jerry Lewis	tt0053644
1746562340	1955	1955	The King's Thief	The King's Thief	Leonard, Robert Z.; Fregonese, Hugo	Robert Z. Leonard	tt0048259
1746438960	1941	1941	Wrangler's Roost	Wrangler's Roost	Luby, S. Roy	S. Roy Luby	tt0034403
1746580996	1961	1961	The Fiercest Heart	The Fiercest Heart	Sherman, George	George Sherman	tt0054874
1746583533	1931	1931	Frankenstein	Frankenstein	Whale, James	James Whale	tt0021884
1746583381	1929	1929	Behind That Curtain	Behind That Curtain	Cummings, Irving	Irving Cummings	tt0019684
1746475522	1948	1948	Road House	Road House	Negulesco, Jean	Jean Negulesco	tt0040740
1746523385	1915	1915	Hearts and the Highway	Hearts and the Highway	North, Wilfred	Wilfrid North	tt0005459
1746422968	1996	1996	Dunston Checks In	Dunston Checks In	Kwapis, Ken	Ken Kwapis	tt0116151
1746580906	1939	1939	Tower of London	Tower of London	Lee, Rowland V.	Rowland V. Lee	tt0032049
1746500654	1925	1925	American Pluck	American Pluck	Stanton, Richard	Richard Stanton	tt0015570
1746491837	1948	1948	Miss Tatlock's Millions	Miss Tatlock's Millions	Haydn, Richard	Richard Haydn	tt0040599
1746422944	1994	1994	Wyatt Earp	Wyatt Earp	Kasdan, Lawrence	Lawrence Kasdan	tt0111756
1746208547	1968	1968	The One and Only, Genuine, Original Family Ban	The One and Only, Genuine, Original Family	O'Herlihy, Michael	Michael O'Herlihy	tt0063389
1746514994	1913	1913	Moths	Moths		Lawrence Marston	tt0003171
1746582552	1936	1936	Silverspurs	Silver Spurs	Taylor, Ray	Ray Taylor	tt0028251
1746569619	1958	1958	Cry Terror!	Cry Terror!	Stone, Andrew L.	Andrew L. Stone	tt0051501
1746422947	1994	1994	Crooklyn	Crooklyn	Lee, Spike	Spike Lee	tt0109504
1746562402	1953	1953	99 River Street	99 River Street	Karlson, Phil	Phil Karlson	tt0045465
1745341993	2004	2004	Paparazzi	Paparazzi	Abascal, Paul	Paul Abascal	tt0338325
1746420857	1990	1990	Madhouse	Madhouse	Ropelewski, Tom	Tom Ropelewski	tt0100087
1746474513	1946	1946	The Thrill of Brazil	The Thrill of Brazil	Simon, S. Sylvan	S. Sylvan Simon	tt0039032
1746561838	1956	1955	The Court Jester	The Court Jester	Panama, Norman; Frank, Melvin	Melvin Frank	tt0049096
1746198737	1968	1968	With Six You Get Eggroll	With Six You Get Eggroll	Morris, Howard	Howard Morris	tt0063821
1746566171	1953	1953	Sweethearts on Parade	Sweethearts on Parade	Dwan, Allan	Allan Dwan	tt0046386
1746513623	1916	1916	The Trail of the Lonesome Pine	The Trail of the Lonesome Pine	DeMille, Cecil B.	Cecil B. DeMille	tt0007473
1746555297	1957	1957	Cyclops	The Cyclops	Gordon, Bert I.	Bert I. Gordon	tt0050281
1746511813	1916	1916	A Law unto Himself	A Law Unto Himself	Broadwell, Robert	Robert Broadwell	tt0163049
1746509321	1949	1949	Gun Law Justice	Gun Law Justice	Hillyer, Lambert	Lambert Hillyer	tt0041439
1746435346	1999	1999	Cradle Will Rock	Cradle Will Rock	Robbins, Tim	Tim Robbins	tt0150216
1746500620	1926	1926	The Border Sheriff	The Border Sheriff	Bradbury, Robert North	Robert N. Bradbury	tt0016673
1746473777	1942	1942	True to the Army	True to the Army	Rogell, Albert S.	Albert S. Rogell	tt0035474
1746464949	1946	1946	Wife Wanted	Wife Wanted	Karlson, Phil	Phil Karlson	tt0039105

1746585388	1939	1939	Confessions of a Nazi Spy	Confessions of a Nazi Spy	Litvak, Anatole	Anatole Litvak	tt0031173
1746512148	1927	1927	The Girl from Chicago	The Girl from Chicago	Enright, Ray	Ray Enright	tt0017934
1746578621	1950	1950	The Secret Fury	The Secret Fury	Ferrer, Mel	Mel Ferrer	tt0042935
1769971933	1969	1969	The Pleasure Machines	The Pleasure Machines	Garcia, Ron	Ronald Víctor García	tt0134032
1746217618	1966	1966	The Ugly Dachshund	The Ugly Dachshund	Tokar, Norman	Norman Tokar	tt0061135
1746574679	1936	1936	Postal Inspector	Postal Inspector	Brower, Otto	Otto Brower	tt0028132
1746580703	1937	1937	Courage of the West	Courage of the West	Lewis, Joseph H.	Joseph H. Lewis	tt0028746
1746454259	1941	1941	Three Girls About Town	Three Girls About Town	Jason, Leigh	Leigh Jason	tt0034286
1746560758	1932	1932	Hello Trouble	Hello Trouble	Hillyer, Lambert	Lambert Hillyer	tt0022992
1746421716	1996	1996	The Great White Hype	The Great White Hype	Hudlin, Reginald	Reginald Hudlin	tt0116448
1746566082	1956	1956	Tribute to a Bad Man	Tribute to a Bad Man	Wise, Robert	Robert Wise	tt0049881
1745358540	2003	2002	Phone Booth	Phone Booth	Schumacher, Joel	Joel Schumacher	tt0183649
1746420403	1987	1987	Surf Nazis Must Die	Surf Nazis Must Die	George, Peter	Peter George	tt0094077
1746585405	1940	1940	Blondie Plays Cupid	Blondie Plays Cupid	Strayer, Frank R.	Frank R. Strayer	tt0032262
1746491748	1944	1944	Boss of Boomtown	Boss of Boomtown	Taylor, Ray	Ray Taylor	tt0036663
1746454675	1941	1941	It Started with Eve	It Started with Eve	Koster, Henry	Henry Koster	tt0033766
1746520712	1925	1925	The Danger Signal	The Danger Signal	Kenton, Erle C.	Erle C. Kenton	tt0015728
1745282897	2006	2006	Little Miss Sunshine	Little Miss Sunshine	Dayton, Jonathan; Faris, Valerie	Jonathan Dayton	tt0449059
1746584721	1930	1930	Worldly Goods	Worldly Goods	Rosen, Phil	Phil Rosen	tt0021563
1746566091	1955	1955	The Purple Mask	The Purple Mask	Humberstone, Bruce	H. Bruce Humberstone	tt0048522
1746208588	1967	1967	Teenage Mother	Teenage Mother	Gross, Jerry	Jerry Gross	tt0061071
1746436581	1942	1942	Lady for a Night	Lady for a Night	Jason, Leigh	Leigh Jason	tt0033806
1746421232	1982	1982	Summer Lovers	Summer Lovers	Kleiser, Randal	Randal Kleiser	tt0084737
1746421239	1993	1993	Josh and S.A.M.	Josh and S.A.M.	Weber, Billy	Billy Weber	tt0107277
1746565072	1952	1952	Park Row	Park Row	Fuller, Samuel	Samuel Fuller	tt0045009
1746492073	1948	1948	The Prince of Thieves	The Prince of Thieves	Bretherton, Howard; Nosseck, Max	Howard Bretherton	tt0040710
1769972677	1963	1963	All of Me	All of Me	Martin, Jay	Joel Holt	tt0445173
1746475259	1944	1944	Goodnight, Sweetheart	Goodnight, Sweetheart	Santley, Joseph	Joseph Santley	tt0036875
1746491798	1948	1948	Sorry, Wrong Number	Sorry, Wrong Number	Litvak, Anatole	Anatole Litvak	tt0040823
1746454063	1941	1941	Three Sons O'Guns	Three Sons o' Guns	Stoloff, Ben	Benjamin Stoloff	tt0034287
1746502064	1916	1916	The Honorable Friend	The Honorable Friend	Le Saint, Edward J.	Edward LeSaint	tt0006825
1746524845	1923	1923	The Bad Man	The Bad Man	Carewe, Edwin	Edwin Carewe	tt0013856
1746576017	1931	1931	Law of the Sea	The Law of the Sea	Brower, Otto	Otto Brower	tt0023123
1746501041	1910	1910	Patricia of the Plains	Patricia of the Plains		Gilbert M. 'Broncho Billy' Anderson	tt0001357
1746509198	1925	1925	Wild Justice	Wild Justice	Franklin, Chester	Chester M. Franklin	tt0016535
1746235044	1972	1972	Slaughter	Slaughter	Starrett, Jack	Jack Starrett	tt0069279
1746499870	1950	1950	The Blazing Sun	The Blazing Sun	English, John	John English	tt0042260
1746580285	1938	1938	Mr. Moto Takes a Chance	Mr. Moto Takes a Chance	Foster, Norman	Norman Foster	tt0030469
1746577961	1935	1935	What Price Crime?	What Price Crime	Herman, Albert	Albert Herman	tt0027201
1746495801	1920	1921	The Rich Slave	The Rich Slave	Fielding, Romaine	Romaine Fielding	tt0152187
1746501628	1915	1915	The Scarlet Sin	The Scarlet Sin	Turner, Otis	Otis Turner	tt0006010
1746394009	1981	1981	Shock Treatment	Shock Treatment	Sharman, Jim	Jim Sharman	tt0083067
1746508877	1926	1926	The Truthful Sex	The Truthful Sex	Thomas, Richard	Richard Thomas	tt0017489
1746582331	1930	1930	A Soldier's Plaything	A Soldier's Plaything	Curtiz, Michael	Michael Curtiz	tt0022409
1746569677	1953	1953	Texas Bad Man	Texas Bad Man	Collins, Lewis D.	Lewis D. Collins	tt0162004
1745374774	2009	2008	The Informers	The Informers	Jordan, Gregor	Gregor Jordan	tt0865554
1746520912	1948	1948	Parole, Inc.	Parole, Inc.	Zeisler, Alfred	Alfred Zeisler	tt0041733
1746503554	1921	1921	The Sea Lion	The Sea Lion	Lee, Rowland V.	Rowland V. Lee	tt0012657
1745302645	2006	2006	Wicked Little Things	Wicked Little Things	Cardone, J. S.	J.S. Cardone	tt0470000
1746533002	1923	1923	Sixty Cents an Hour	Sixty Cents an Hour	Henabery, Joseph	Joseph Henabery	tt0014480
1746575783	1931	1931	The Painted Desert	The Painted Desert	Higgin, Howard	Howard Higgin	tt0022243
1746567265	1933	1933	Rafter Romance	Rafter Romance	Seiter, William	William A. Seiter	tt0024484
1746565938	1957	1957	Lizzie	Lizzie	Haas, Hugo	Hugo Haas	tt0050650
1746577906	1937	1937	Maid of Salem	Maid of Salem	Lloyd, Frank	Frank Lloyd	tt0029190
1746502713	1910	1910	The Long Trail	The Long Trail	Boggs, Frank	Francis Boggs	tt0001290
1746500818	1914	1914	The Redemption of David Corson	The Redemption of David Corson		Frederick A. Thomson	tt0004522
1746578669	1960	1960	Hideout in the Sun	Hideout in the Sun	Wolk, Lazarus L.	Larry Wolk	tt0053908

1746532616	1923	1923	Sawdust	Sawdust	Conway, Jack	Jack Conway	tt0014439
1746574778	1936	1936	Lady from Nowhere	Lady from Nowhere	Wiles, Gordon	Gordon Wiles	tt0027864
1746574528	1933	1933	Blind Adventure	Blind Adventure	Schoedsack, Ernest B.	Ernest B. Schoedsack	tt0023816
1746453621	1944	1944	Oath of Vengeance	Oath of Vengeance	Newfield, Sam	Sam Newfield	tt0037142
1745373934	2009	2009	He's Just Not That Into You	He's Just Not That Into You	Kwapis, Ken	Ken Kwapis	tt1001508
1746570070	1952	1952	Washington Story	Washington Story	Pirosh, Robert	Robert Pirosh	tt0045312
2007986729	1922	1922	Bow Wow	Bow Wow	Jackman, Fred W.	Fred Jackman	tt0123000
1746577754	1936	1936	A Message to Garcia	A Message to Garcia	Marshall, George	George Marshall	tt0027959
1746564466	1952	1952	The Marrying Kind	The Marrying Kind	Cukor, George	George Cukor	tt0044888
1746584418	1940	1940	The Crooked Road	The Crooked Road	Rosen, Philip	Phil Rosen	tt0032367
1746474524	1947	1947	Crossfire	Crossfire	Dmytryk, Edward	Edward Dmytryk	tt0039286
1746219421	1971	1970	The Animals	The Animals	Joy, Ron	Ron Joy	tt0065407
1746524632	1910	1910	Love's "C.Q.D."	Love's C. Q. D.		William F. Haddock	tt0001294
1746570569	1933	1933	The Narrow Corner	The Narrow Corner	Green, Alfred E.	Alfred E. Green	tt0024373
1746184863	1965	1965	The Naked Brigade	The Naked Brigade	Dexter, Maury	Maury Dexter	tt0059494
1746583685	1951	1951	The Man with My Face	The Man with My Face	Montagne, Edward J.	Edward Montagne	tt0121515
1746561268	1932	1932	Panama Flo	Panama Flo	Murphy, Ralph	Ralph Murphy	tt0023316
1746583033	1936	1936	Hopalong Cassidy Returns	Hopalong Cassidy Returns	Watt, Nate	Nate Watt	tt0027760
1746487732	1918	1918	Beans	Beans	Dillon, Jack	John Francis Dillon	tt0008865
1746577351	1963	1963	Passion Holiday	Passion Holiday	Miles, Wynn	Wynn Mavis	tt0056334
1746500933	1915	1915	Betty in Search of a Thrill	Betty in Search of a Thrill	Smalley, Phillips; Weber, Lois	Phillips Smalley	tt0004961
1746580352	1938	1938	Durango Valley Raiders	Durango Valley Raiders	Newfield, Sam	Sam Newfield	tt0030091
1745358676	2009	2008	The Hurt Locker	The Hurt Locker	Bigelow, Kathryn	Kathryn Bigelow	tt0887912
1746555037	1957	1957	Death in Small Doses	Death in Small Doses	Newman, Joseph	Joseph M. Newman	tt0050295
1746580458	1939	1939	The Rookie Cop	The Rookie Cop	Howard, David	David Howard	tt0031875
1746465177	1941	1941	Billy the Kid's Range War	Billy the Kid's Range War	Stewart, Peter	Sam Newfield	tt0033394
1746501664	1916	1916	Nearly a King	Nearly a King	Thomson, Frederick	Frederick A. Thomson	tt0007118
1746574563	1933	1933	By Appointment Only	By Appointment Only	Strayer, Frank R.	Frank R. Strayer	tt0023859
1746577610	1934	1934	Murder in the Private Car	Murder in the Private Car	Beaumont, Harry	Harry Beaumont	tt0024359
1746185394	1965	1965	Nudes on Tiger Reef	Nudes on Tiger Reef	Mahon, Barry	Barry Mahon	tt0134858
1746509295	1949	1949	Riders in the Sky	Riders in the Sky	English, John	John English	tt0041807
1746410442	1987	1987	Wall Street	Wall Street	Stone, Oliver	Oliver Stone	tt0094291
1746513654	1923	1923	The Old Fool	The Old Fool	Venturini, Edward	Edward D. Venturini	tt0014326
1746512758	1923	1923	Boy of Mine	Boy of Mine	Beaudine, William	William Beaudine	tt0013884
1746581995	1928	1928	Texas Tommy	Texas Tommy	McGowan, J. P.	J.P. McGowan	tt0019458
1746585186	1930	1930	Rough Romance	Rough Romance	Erickson, A. F.	A.F. Erickson	tt0021319
1746530803	1949	1949	A Connecticut Yankee in King Arthur's Court	A Connecticut Yankee in King Arthur's Court	Garnett, Tay	Tay Garnett	tt0041259
1746514529	1923	1923	The Exciters	The Exciters	Campbell, Maurice	Maurice Campbell	tt0014027
1746394203	1983	1982	Liquid Sky	Liquid Sky	Tsukerman, Slava	Slava Tsukerman	tt0085852
1746584163	1939	1939	The Stranger from Texas	The Stranger from Texas	Nelson, Sam	Sam Nelson	tt0033106
1746580396	1939	1939	In Name Only	In Name Only	Cromwell, John	John Cromwell	tt0031477
1746583636	1931	1931	Too Many Cooks	Too Many Cooks	Seiter, William	William A. Seiter	tt0022491
1746509009	1925	1925	Free To Love	Free to Love	O'Connor, Frank	Frank O'Connor	tt0015840
1746475282	1947	1947	California	California	Farrow, John	John Farrow	tt0038392
1746555639	1957	1957	The Buster Keaton Story	The Buster Keaton Story	Sheldon, Sidney	Sidney Sheldon	tt0050218
1746582148	1928	1928	Their Hour	Their Hour	Raboch, Alfred	Alfred Raboch	tt0019464
1746499867	1949	1949	A Letter to Three Wives	A Letter to Three Wives	Mankiewicz, Joseph L.	Joseph L. Mankiewicz	tt0041587
1746520880	1925	1925	Some Pun'kins	Some Pun'kins	Storm, Jerome	Jerome Storm	tt0016371
1746497038	1920	1920	The Sacred Ruby	The Sacred Ruby	White, Glenn	Glen White	tt0188188
1746584670	1929	1929	Illusion	Illusion	Mendes, Lothar	Lothar Mendes	tt0020016
1746578640	1958	1958	Villa!!	Villa!!	Clark, James B.	James B. Clark	tt0052366
1746475722	1947	1947	The Web	The Web	Gordon, Michael	Michael Gordon	tt0039973
1746566285	1954	1954	Bitter Creek	Bitter Creek	Carr, Thomas	Thomas Carr	tt0046782
1746394698	1988	1988	Alien Nation	Alien Nation	Baker, Graham	Graham Baker	tt0094631
1746562018	1954	1953	Barefoot Battalion	The Barefoot Battalion	Tallas, Gregg G.	Gregg G. Tallas	tt0168487
1769970846	1915	1915	The Thirteenth Girl	The Thirteenth Girl	Marston, Theodore	Theodore Marston	tt0344448
1746560748	1965	1965	Dark Intruder	Dark Intruder	Hart, Harvey	Harvey Hart	tt0059083

1746523813	1915	1915	May Blossom	May Blossom	Dwan, Allan	Allan Dwan	tt0005732
1746580513	1938	1938	Just Around the Corner	Just Around the Corner	Cummings, Irving	Irving Cummings	tt0030302
1746516136	1920	1920	Outside the Law	Outside the Law	Browning, Tod	Tod Browning	tt0012538
1746562178	1953	1953	Mission over Korea	Mission Over Korea	Sears, Fred F.	Fred F. Sears	tt0046079
1746584577	1929	1929	Father and Son	Father and Son	Kenton, Erle C.	Erle C. Kenton	tt0019874
1746584895	1940	1940	Son of the Navy	Son of the Navy	Nigh, William	William Nigh	tt0033078
1746501388	1910	1910	A Tale of the Sea	A Tale of the Sea		Francis Boggs	tt0234847
1746514787	1916	1916	Sally in Our Alley	Sally in Our Alley	Vale, Travers	Travers Vale	tt0007283
1746585290	1930	1930	Ladies of Leisure	Ladies of Leisure	Capra, Frank	Frank Capra	tt0021040
1746582265	1931	1931	Merely Mary Ann	Merely Mary Ann	King, Henry	Henry King	tt0022142
1746580659	1935	1935	The Fighting Lady	Fighting Lady	Borcosque, Carlos	Carlos F. Borcosque	tt0026351
1746467030	1917	1917	The Reed Case	The Reed Case	Holubar, Allen	Allen Holubar	tt0008505
1746566259	1954	1954	Charge of the Lancers	Charge of the Lancers	Castle, William	William Castle	tt0046847
1746492442	1948	1948	Four Faces West	Four Faces West	Green, Alfred E.	Alfred E. Green	tt0040370
1746513444	1916	1916	The Devil's Double	The Devil's Double	Hart, William S.	William S. Hart	tt0006588
1746491869	1948	1948	Shanghai Chest	The Shanghai Chest	Beaudine, William	William Beaudine	tt0040778
1746474975	1947	1947	Jungle Flight	Jungle Flight	Stewart, Peter	Sam Newfield	tt0039523
1746484442	1918	1918	A Woman's Fool	A Woman's Fool	Ford, Jack	John Ford	tt0009837
1746574721	1935	1935	Dinky	Dinky	Lederman, D. Ross; Bretherton, How	Howard Bretherton	tt0026279
1746530794	1949	1949	Prince of the Plains	Prince of the Plains	Ford, Philip	Philip Ford	tt0040711
1746565921	1953	1953	The Marksman	The Marksman	Collins, Lewis	Lewis D. Collins	tt0046047
1746497872	1919	1919	What Every Woman Wants	What Every Woman Wants	Hampton, Jesse D.	Jesse D. Hampton	tt0194528
1745372307	2002	2002	Full Frontal	Full Frontal	Soderbergh, Steven	Steven Soderbergh	tt0290212
1746520885	1948	1948	Whiplash	Whiplash	Seiler, Lew	Lewis Seiler	tt0040963
1746500277	1924	1924	Chalk Marks	Chalk Marks	Adolfi, John G.	John G. Adolfi	tt0014766
1746574049	1963	1963	The Day Mars Invaded Earth	The Day Mars Invaded Earth	Dexter, Maury	Maury Dexter	tt0055893
1746583344	1951	1951	Let's Go Navy	Let's Go Navy!	Beaudine, William	William Beaudine	tt0043735
1746418919	1940	1940	You'll Find Out	You'll Find Out	Butler, David	David Butler	tt0033283
1746580853	1938	1938	Army Girl	Army Girl	Nicholls, George	George Nichols Jr.	tt0029880
1746580510	1938	1938	Professor Beware	Professor Beware	Nugent, Elliott	Elliott Nugent	tt0030631
1746507850	1949	1949	The Clay Pigeon	The Clay Pigeon	Fleischer, Richard	Richard Fleischer	tt0041252
1746454453	1999	1999	Muppets from Space	Muppets from Space	Hill, Tim	Tim Hill	tt0158811
1746570402	1934	1934	By Your Leave	By Your Leave	Corrigan, Lloyd	Lloyd Corrigan	tt0024939
1746524262	1915	1915	The Blindness of Virtue	The Blindness of Virtue	Totten, Joseph Byron	Joseph Byron Totten	tt0004981
1746508097	1926	1926	The Romance of a Million Dollars	The Romance of a Million Dollars	Terriss, Tom	Tom Terriss	tt0158159
1746502402	1916	1916	By Love Redeemed	By Love Redeemed	Williams, C. Jay	C.J. Williams	tt0382587
1746584367	1936	1936	Three Cheers for Love	Three Cheers for Love	McCarey, Ray	Ray McCarey	tt0028366
1746568348	1934	1934	Treasure Island	Treasure Island	Fleming, Victor	Victor Fleming	tt0025907
1746582661	1937	1937	Too Many Wives	Too Many Wives	Holmes, Ben	Ben Holmes	tt0029679
1746574609	1937	1937	Breakfast for Two	Breakfast for Two	Santell, Alfred	Alfred Santell	tt0028659
1746584133	1937	1937	Parnell	Parnell	Stahl, John M.	John M. Stahl	tt0029377
1746578982	1950	1950	Never Fear	Never Fear	Lupino, Ida	Ida Lupino	tt0042783
1746409984	1982	1982	Creepshow	Creepshow	Romero, George A.	George A. Romero	tt0083767
1746394534	1988	1988	Maniac Cop	Maniac Cop	Lustig, William	William Lustig	tt0095583
1746512110	1910	1910	The Wild Man of Borneo	The Wild Man of Borneo			tt1799144
1746583017	1935	1935	Atlantic Adventure	Atlantic Adventure	Rogell, Albert	Albert S. Rogell	tt0026085
1746582374	1930	1930	College Lovers	College Lovers	Adolfi, John G.	John G. Adolfi	tt0020778
1746501155	1915	1915	Madame Butterfly	Madame Butterfly	Olcott, Sidney	Sidney Olcott	tt0005682
1746576860	1932	1932	Scarlet Dawn	Scarlet Dawn	Dieterle, William	William Dieterle	tt0022308
1746410585	1990	1990	Flatliners	Flatliners	Schumacher, Joel	Joel Schumacher	tt0099582
1746514129	1917	1917	The Flame of the Yukon	The Flame of the Yukon	Miller, Charles	Charles Miller	tt0196562
1746575023	1936	1936	The Case of the Velvet Claws	The Case of the Velvet Claws	Clemens, William	William Clemens	tt0027429
1746465398	1944	1944	You Can't Ration Love	You Can't Ration Love	Fuller, Lester	Lester Fuller	tt0037475
1746578537	1959	1959	City of Fear	City of Fear	Lerner, Irving	Irving Lerner	tt0052696
1746249483	1978	1978	Foul Play	Foul Play	Higgins, Colin	Colin Higgins	tt0077578
1746561973	1954	1954	Southwest Passage	Southwest Passage	Nazzaro, Ray	Ray Nazarro	tt0047517
1746574850	1937	1936	The Plainsman	The Plainsman	DeMille, Cecil B.	Cecil B. DeMille	tt0028108

1746476204	1944	1944	Song of Russia	Song of Russia	Ratoff, Gregory	Gregory Ratoff	tt0036378	
1745341279	2004	2004	Garden State	Garden State	Braff, Zach	Zach Braff	tt0333766	
1746514751	1914	1914	Fighting Death	Fighting Death	Blaché, Herbert	Herbert Blaché	tt0003959	
1746496128	1918	1918	Hell's Crater	Hell's Crater	Pearson, W. B.	W.B. Pearson	tt0009155	
1746580685	1939	1939	The Private Lives of Elizabeth and Essex	The Private Lives of Elizabeth and Essex	Curtiz, Michael	Michael Curtiz	tt0031826	
1746577622	1934	1934	Wonder Bar	Wonder Bar	Bacon, Lloyd	Lloyd Bacon	tt0026007	
1746211214	1972	1972	Winter Soldier	Winter Soldier			tt0204058	
1746474808	1947	1947	Smoky River Serenade	Smoky River Serenade	Abrahams, Derwin	Derwin Abrahams	tt0039841	
1746574396	1950	1950	Harvey	Harvey	Koster, Henry	Henry Koster	tt0042546	
1746583800	1931	1931	The Big Gamble	The Big Gamble	Niblo, Fred	Fred Niblo	tt0021659	
1746577899	1938	1938	Tough Kid	Tough Kid	Bretherton, Howard	Howard Bretherton	tt0032048	
1746574387	1963	1963	Who's Minding the Store?	Who's Minding the Store?	Tashlin, Frank	Frank Tashlin	tt0057683	
1746553835	1963	1962	How the West Was Won	How the West Was Won		John Ford	tt0056085	
1746584928	1940	1940	Arise, My Love	Arise, My Love	Leisen, Mitchell	Mitchell Leisen	tt0032220	
1745359580	2003	2003	Cheaper by the Dozen	Cheaper by the Dozen	Levy, Shawn	Shawn Levy	tt0349205	
1769970405	1965	1965	Naughty Nudes	Naughty Nudes	Michael, James	Barry Mahon	tt0126504	
1745293531	2006	2005	The Lady in Question is Charles Busch	The Lady in Question Is Charles Busch	Catania, John ; Ignacio, Charles	John Catania	tt0426508	
1746580321	1939	1939	Frontier Pony Express	Frontier Pony Express	Kane, Joseph	Joseph Kane	tt0031347	
1746473929	1944	1944	Shine on Harvest Moon	Shine on Harvest Moon	Butler, David	David Butler	tt0037273	
1746580283	1937	1937	I'll Take Romance	I'll Take Romance	Griffith, Edward H.	Edward H. Griffith	tt0029043	
1746464915	1941	1941	In Old Colorado	In Old Colorado	Bretherton, Howard	Howard Bretherton	tt0033753	
1746574623	1935	1935	The Dark Angel	The Dark Angel	Franklin, Sidney	Sidney Franklin	tt0026264	
1746523833	1923	1923	A Chapter in Her Life	A Chapter in Her Life	Weber, Lois	Lois Weber	tt0013917	
1746498486	1920	1920	A Cumberland Romance	A Cumberland Romance	Maigne, Charles	Charles Maigne	tt0011082	
1746424237	1943	1943	Bataan	Batman	Garnett, Tay	Lambert Hillyer	tt0035665	
1746530739	1949	1949	Homicide	Homicide	Jacoves, Felix	Felix Jacoves	tt0041482	
1746235021	1971	1971	The Return of Count Yorga	The Return of Count Yorga	Kelljan, Bob	Bob Kelljan	tt0067671	
1746435837	1996	1996	Moll Flanders	Moll Flanders	Densham, Pen	Pen Densham	tt0117071	
1746569540	1958	1958	Manhunt in the Jungle	Manhunt in the Jungle	McGowan, Tom	Tom McGowan	tt0051903	
1746565885	1954	1954	The Yellow Mountain	The Yellow Mountain	Hibbs, Jesse	Jesse Hibbs	tt0047685	
1746514321	1916	1916	A Yoke of Gold	A Yoke of Gold	Carleton, Lloyd B.	Lloyd B. Carleton	tt0007601	
1746520359	1924	1924	After a Million	After a Million	Nelson, Jack	Jack Nelson	tt0014648	
1746217383	1967	1967	Spree	Spree	Leisen, Mitchell; Green, Walon	Walon Green	tt0062299	
1746475917	1948	1948	B. F.'s Daughter	B.F.'s Daughter	Leonard, Robert Z.	Robert Z. Leonard	tt0040141	
1746583887	1930	1930	Tom Sawyer	Tom Sawyer	Cromwell, John	John Cromwell	tt0021483	
**1746454624**	**1940**	**1940**	**Young As You Feel**	**Young as You Feel**	**St. Clair, Malcolm**	**Malcolm St. Clair**	**tt0033290**	1
1746437414	1940	1940	I Married Adventure	I Married Adventure			tt0301435	
1746495975	1920	1920	The Devil to Pay	The Devil to Pay	Warde, Ernest C.	Ernest C. Warde	tt0011111	
1745359994	2003	2003	Bulletproof Monk	Bulletproof Monk	Hunter, Paul	Paul Hunter	tt0245803	
1746583463	1929	1929	After the Fog	After the Fog	De Cordova, Leander	Leander De Cordova	tt0019625	
1746523556	1923	1923	Lawful Larceny	Lawful Larceny	Dwan, Allan	Allan Dwan	tt0014190	
1746567934	1952	1952	This Is Cinerama	This Is Cinerama		Merian C. Cooper	tt0045231	
1746420976	1992	1992	Frozen Assets	Frozen Assets	Miller, George	George Miller	tt0104309	
**1746514953**	**1917**	**1917**	**An Amateur Orphan**	**An Amateur Orphan**	**Brooke, Van Dyke**	**Van Dyke Brooke**	**tt0007630**	1
1746454493	1941	1941	Meet Boston Blackie	Meet Boston Blackie	Florey, Robert	Robert Florey	tt0033890	
1746507836	1949	1949	Laramie	Laramie	Nazarro, Ray	Ray Nazarro	tt0041577	
1746562329	1953	1953	Kansas Pacific	Kansas Pacific	Nazarro, Ray	Ray Nazarro	tt0045954	
1746454365	1944	1943	Thousands Cheer	Thousands Cheer	Sidney, George	George Sidney	tt0036432	
1746514951	1927	1927	Finger Prints	Finger Prints	Bacon, Lloyd	Lloyd Bacon	tt0017883	
1746475967	1944	1943	Swingtime Johnny	Swingtime Johnny	Cline, Edward F.	Edward F. Cline	tt0036409	
1746508038	1924	1924	Trail Dust	Trail Dust	Hines, Gordon	Gordon Hines	tt0173346	
1746503695	1919	1919	The Betrayal	The Betrayal	Barry, J. A.	J.A. Barry	tt0186869	
1746491980	1945	1945	Song of Old Wyoming	Song of Old Wyoming	Emmett, Robert	Robert Emmett Tansey	tt0038101	
1746393365	1988	1988	The Decline of Western Civilization Part II: The M	The Decline of Western Civilization Part II: T	Spheeris, Penelope	Penelope Spheeris	tt0094980	
1746524836	1916	1916	Ramona	Ramona	Crisp, Donald	Donald Crisp	tt0007248	
1746209905	1970	1970	Start the Revolution Without Me	Start the Revolution Without Me	Yorkin, Bud	Bud Yorkin	tt0066402	
1746500210	1925	1925	The Outlaw's Daughter	The Outlaw's Daughter	O'Brien, John B.	John B. O'Brien	tt0016189	

1746580899	1934	1934	The Last Gentleman	The Last Gentleman	Lanfield, Sidney	Sidney Lanfield	tt0025368
1746585024	1939	1939	Two Thoroughbreds	Two Thoroughbreds	Hively, Jack	Jack Hively	tt0032067
1769970536	1967	1967	A Good Time With a Bad Girl	A Good Time with a Bad Girl		Barry Mahon	tt0139258
1746533012	1916	1916	Hoodoo Ann	Hoodoo Ann	Ingraham, Lloyd	Lloyd Ingraham	tt0006826
1746583526	1930	1930	Whoopee!	Whoopee!	Freeland, Thornton	Thornton Freeland	tt0021549
1746509218	1925	1925	Quick Change	Quick Change	Henderson, Dell	Dell Henderson	tt0016256
1746393490	1988	1988	Shakedown	Shakedown	Glickenhaus, James	James Glickenhaus	tt0096087
1746454055	1945	1945	The Corn Is Green	The Corn Is Green	Rapper, Irving	Irving Rapper	tt0037614
1769970054	1912	1912	Carmen of the Isles	Carmen of the Isles		Colin Campbell	tt0421952
1746393121	1986	1986	Youngblood	Youngblood	Markle, Peter	Peter Markle	tt0092272
1746582201	1929	1929	The Long, Long Trail	The Long, Long Trail	Rosson, Arthur	Arthur Rosson	tt0020107
1746492069	1946	1946	Gentleman Joe Palooka	Gentleman Joe Palooka	Endfield, Cyril	Cy Endfield	tt0038553
1746504401	1919	1919	Men, Women and Money	Men, Women, and Money	Melford, George	George Melford	tt0010452
1745342695	2002	2002	The Country Bears	The Country Bears	Hastings, Peter	Peter Hastings	tt0276033
1746454495	1940	1940	Stagecoach War	Stagecoach War	Selander, Lesley	Lesley Selander	tt0033099
1746533032	1927	1927	The Desert Pirate	The Desert Pirate	Dugan, James	James Dugan	tt0017803
1746571224	1910	1910	Francesca Da Rimini	Francesca da Rimini	Blackton, J. Stuart	J. Stuart Blackton	tt0200648
1746216685	1967	1967	The Tiger Makes Out	The Tiger Makes Out	Hiller, Arthur	Arthur Hiller	tt0062369
1746576719	1932	1933	Diamond Trail	Diamond Trail	Fraser, Harry	Harry L. Fraser	tt0022819
1746464956	1947	1947	Boy! What a Girl!	Boy! What a Girl!	Leonard, Arthur	Arthur H. Leonard	tt0135169
1746498691	1918	1918	Kildare of Storm	Kildare of Storm	Franklin, Harry L.	Harry L. Franklin	tt0009261
1746500428	1925	1925	The Monster	The Monster	West, Roland	Roland West	tt0016123
1746513435	1910	1910	Ononko's Vow	Ononko's Vow	McGlynn, Frank	Frank McGlynn Sr.	tt0001347
1746492489	1948	1948	The Fuller Brush Man	The Fuller Brush Man	Simon, S. Sylvan	S. Sylvan Simon	tt0040379
1745342529	2004	2004	Jersey Girl	Jersey Girl	Smith, Kevin	Kevin Smith	tt0300051
1746436571	1942	1942	Hay Foot	Hay Foot	Guiol, Fred	Fred Guiol	tt0033696
1746585496	1940	1940	City for Conquest	City for Conquest	Litvak, Anatole	Anatole Litvak	tt0032342
1745373914	2002	2002	Empire	Empire	Reyes, Franc.	Franc. Reyes	tt0262396
1746434354	1991	1990	Resident Alien	Resident Alien	Nossiter, Jonathan	Jonathan Nossiter	tt0102777
1746582165	1928	1928	A Trick of Hearts	A Trick of Hearts	Eason, Reeves	B. Reeves Eason	tt0019492
1746218919	1975	1975	Let's Do It Again	Let's Do It Again	Poitier, Sidney	Sidney Poitier	tt0073282
1746524394	1923	1923	The Eagle's Feather	The Eagle's Feather	Sloman, Edward	Edward Sloman	tt0014010
1746475962	1948	1948	Indian Agent	Indian Agent	Selander, Lesley	Lesley Selander	tt0040470
1746563861	1952	1952	The Golden Hawk	The Golden Hawk	Salkow, Sidney	Sidney Salkow	tt0044670
1746423999	1942	1942	Blondie's Blessed Event	Blondie's Blessed Event	Strayer, Frank R.	Frank R. Strayer	tt0034527
1745340181	2002	2001	Impostor	Impostor	Fleder, Gary	Gary Fleder	tt0160399
1746250181	1979	1979	Hot Stuff	Hot Stuff	DeLuise, Dom	Dom DeLuise	tt0079308
1746575379	1931	1930	Men on Call	Men on Call	Blystone, John	John G. Blystone	tt0021138
1746583349	1930	1930	O'Malley Rides Alone	O'Malley Rides Alone	McGowan, J. P.	J.P. McGowan	tt0021195
1746577105	1963	1963	Police Nurse	Police Nurse	Dexter, Maury	Maury Dexter	tt0057417
1746491854	1947	1947	Song of the Thin Man	Song of the Thin Man	Buzzell, Edward	Edward Buzzell	tt0039853
1746585117	1928	1928	Sealed Orders	Sealed Orders	Levigard, Josef	Joseph Levigard	tt0347968
1746555207	1957	1957	Bernardine	Bernardine	Levin, Henry	Henry Levin	tt0050184
1746577928	1937	1937	Renfrew of the Royal Mounted	Renfrew of the Royal Mounted	Herman, Al	Albert Herman	tt0029475
1746393274	1988	1988	Monkey Shines	Monkey Shines	Romero, George A.	George A. Romero	tt0095652
1746581258	1961	1961	The Hoodlum Priest	The Hoodlum Priest	Kershner, Irvin	Irvin Kershner	tt0054991
1746580391	1935	1935	Bar 20 Rides Again	Bar 20 Rides Again	Bretherton, Howard	Howard Bretherton	tt0027332
1746465328	1947	1947	The Roosevelt Story	The Roosevelt Story		Lawrence M. Klee	tt0218560
1746465095	1942	1942	Juke Girl	Juke Girl	Bernhardt, Curtis	Curtis Bernhardt	tt0034926
1746569506	1955	1955	Murder in Villa Capri	Murder in Villa Capri	Simetti, Otto	Otto Simetti	tt1183718
1746473753	1946	1946	One Exciting Week	One Exciting Week	Beaudine, William	William Beaudine	tt0038801
1746571315	1934	1934	Eight Girls in a Boat	Eight Girls in a Boat	Wallace, Richard	Richard Wallace	tt0025077
1746473889	1947	1946	The Beast with Five Fingers	The Beast with Five Fingers	Florey, Robert	Robert Florey	tt0038338
1746503537	1918	1918	The One Woman	The One Woman	Barker, Reginald	Reginald Barker	tt0009449
1746583392	1931	1931	50 Million Frenchmen	50 Million Frenchmen	Bacon, Lloyd	Lloyd Bacon	tt0021859
1746475973	1945	1945	The Great Flamarion	The Great Flamarion	Mann, Anthony	Anthony Mann	tt0037749
1746581195	1951	1950	Born Yesterday	Born Yesterday	Cukor, George	George Cukor	tt0042276

1746503999	1917	1917	The Stolen Play	The Stolen Play	Harvey, Harry	Harry Harvey	tt0008630
1746581454	1928	1928	The Spieler	The Spieler	Garnett, Tay	Tay Garnett	tt0019414
1746465389	1945	1945	There Goes Kelly	There Goes Kelly	Karlstein, Phil	Phil Karlson	tt0038158
1746581415	1928	1928	The Tragedy of Youth	The Tragedy of Youth	Archainbaud, George	George Archainbaud	tt0019487
1746566865	1933	1933	Ever in My Heart	Ever in My Heart	Mayo, Archie	Archie Mayo	tt0023992
1746566118	1957	1957	Valerie	Valerie	Oswald, Gerd	Gerd Oswald	tt0051149
1746584623	1930	1929	Blaze o' Glory	Blaze o' Glory	Hoffman, Renaud; Crone, George J.	George Crone	tt0019703
1746464881	1944	1944	The Hairy Ape	The Hairy Ape	Santell, Alfred	Alfred Santell	tt0036892
1746574764	1937	1937	Tex Rides with the Boy Scouts	Tex Rides with the Boy Scouts	Taylor, Ray	Ray Taylor	tt0030849
1746422656	1996	1996	Eddie	Eddie	Rash, Steve	Steve Rash	tt0116168
1745302851	2006	2006	LOL	LOL	Swanberg, Joe	Joe Swanberg	tt0462392
1746580734	1935	1935	Alias Mary Dow	Alias Mary Dow	Neumann, Kurt	Kurt Neumann	tt0026053
1746491960	1948	1948	Dream Girl	Dream Girl	Leisen, Mitchell	Mitchell Leisen	tt0040302
1746203031	1970	1970	Count Yorga, Vampire	Count Yorga, Vampire	Kelljan, Robert	Bob Kelljan	tt0066952
1746437397	1941	1941	Married Bachelor	Married Bachelor	Buzzell, Edward	Edward Buzzell	tt0033883
1746409071	1991	1990	Paris Is Burning	Paris Is Burning	Livingston, Jennie	Jennie Livingston	tt0100332
1746198285	1969	1969	The Witchmaker	The Witchmaker	Brown, William O.	William O. Brown	tt0065217
1746211057	1972	1972	Scarecrow in a Garden of Cucumbers	Scarecrow in a Garden of Cucumbers	Kaplan, Robert J.	Robert J. Kaplan	tt0135644
1746514921	1915	1915	A Man and His Mate	A Man and His Mate	Adolfi, John G.	John G. Adolfi	tt0005700
1746183279	1910	1910	A Central American Romance	A Central American Romance	Dawley, J. Searle	J. Searle Dawley	tt0233420
1745359586	2008	2008	Punisher: War Zone	Punisher: War Zone	Alexander, Lexi	Lexi Alexander	tt0450314
1746562429	1953	1953	Take the High Ground!	Take the High Ground!	Brooks, Richard	Richard Brooks	tt0046398
1746475934	1947	1947	Step-Child	Stepchild	Flood, James	James Flood	tt0039867
1746584638	1930	1930	For the Defense	For the Defense	Cromwell, John	John Cromwell	tt0020896
1746574635	1934	1934	Honor of the Range	Honor of the Range	James, Alan	Alan James	tt0025269
1746574211	1958	1958	Tank Force	Tank Force	Young, Terence	Terence Young	tt0052271
1746514530	1915	1915	Via Wireless	Via Wireless	Fitzmaurice, George	George Fitzmaurice	tt0006217
1746208457	1969	1969	Eye of the Cat	Eye of the Cat	Rich, David Lowell	David Lowell Rich	tt0064310
1746569943	1958	1957	The Mark of the Hawk	The Mark of the Hawk	Audley, Michael	Michael Audley	tt0050692
1746210636	1972	1972	Hit Man	Hit Man	Armitage, George	George Armitage	tt0068704
1746455009	1997	1997	Event Horizon	Event Horizon	Anderson, Paul	Paul W.S. Anderson	tt0119081
1746555131	1956	1956	Thunderstorm	Thunderstorm	Guillermin, John	John Guillermin	tt0048737
1746584834	1930	1930	Lilies of the Field	Lilies of the Field	Korda, Alexander	Alexander Korda	tt0021073
1746581052	1958	1957	Man Without a Body	The Man Without a Body	Wilder, W. Lee; Saunders, Charles	Charles Saunders	tt0050676
1746566267	1957	1957	Pharaoh's Curse	Pharaoh's Curse	Sholem, Lee	Lee Sholem	tt0049616
1746525071	1921	1921	Oh Mary Be Careful	Oh Mary Be Careful	Ashley, Arthur	Arthur Ashley	tt0144531
1746577718	1936	1936	Pepper	Pepper	Tinling, James	James Tinling	tt0028094
1746218750	1972	1972	Junior Bonner	Junior Bonner	Peckinpah, Sam	Sam Peckinpah	tt0068786
1746502906	1914	1914	Dope	Dope	Lieb, Herman	Herman Lieb	tt0003859
1746585362	1939	1939	The Spellbinder	The Spellbinder	Hively, Jack	Jack Hively	tt0031961
1746454400	1945	1945	Gun Smoke	Gun Smoke	Bretherton, Howard	Howard Bretherton	tt0037757
1746508727	1925	1925	Drusilla With a Million	Drusilla with a Million	Weight, F. Harmon	F. Harmon Weight	tt0015767
1746251016	1977	1977	Oh, God!	Oh, God!	Reiner, Carl	Carl Reiner	tt0076489
1746585041	1940	1940	Knute Rockne--All American	Knute Rockne All American	Bacon, Lloyd	Lloyd Bacon	tt0032676
1746408636	1986	1986	The Money Pit	The Money Pit	Benjamin, Richard	Richard Benjamin	tt0091541
1746563638	1953	1953	Below the Sahara	Below the Sahara	Denis, Armand	Armand Denis	tt0209936
1746436536	1943	1942	Seven Miles from Alcatraz	Seven Miles from Alcatraz	Dmytryk, Edward	Edward Dmytryk	tt0035308
1745359403	2003	2003	A Decade Under the Influence	A Decade Under the Influence	LaGravenese, Richard; Demme, Ted	Ted Demme	tt0342275
1746474139	1947	1947	The Unfinished Dance	The Unfinished Dance	Koster, Henry	Henry Koster	tt0039938
1746439702	1997	1997	A Smile Like Yours	A Smile Like Yours	Samples, Keith	Keith Samples	tt0120151
1746553740	1965	1965	Taffy and the Jungle Hunter	Taffy and the Jungle Hunter	Morse, Terry O.	Terry O. Morse	tt0059779
1746394219	1990	1990	The Godfather Part III	The Godfather: Part III	Coppola, Francis Ford	Francis Ford Coppola	tt0099674
1746512442	1916	1916	Poor Little Peppina	Poor Little Peppina	Olcott, Sidney	Sidney Olcott	tt0007202
1746582632	1937	1937	Roaring Timber	Roaring Timber	Rosen, Phil	Phil Rosen	tt0029494
1746235418	1975	1975	The Rocky Horror Picture Show	The Rocky Horror Picture Show	Sharman, Jim	Jim Sharman	tt0073629
1746585173	1930	1930	Numbered Men	Numbered Men	LeRoy, Mervyn	Mervyn LeRoy	tt0021192
1746499987	1948	1948	Black Bart	Black Bart	Sherman, George	George Sherman	tt0040167

1746507911	1924	1924	Helen's Babies	Helen's Babies	Seiter, William A.	William A. Seiter	tt0014978
1746486428	1917	1917	The Silence Sellers	The Silence Sellers	King, Burton L.	Burton L. King	tt0182422
1746439483	1944	1944	Sailor's Holiday	Sailor's Holiday	Berke, William	William Berke	tt0037242
1746454705	1941	1941	Niagara Falls	Niagara Falls	Douglas, Gordon	Gordon Douglas	tt0033949
1746508579	1927	1927	Sorrell and Son	Sorrell and Son		Herbert Brenon	tt0018429
1746577336	1958	1958	Terror in a Texas Town	Terror in a Texas Town	Lewis, Joseph H.	Joseph H. Lewis	tt0052287
1746574465	1952	1952	Indian Uprising	Indian Uprising	Nazarro, Ray	Ray Nazarro	tt0179868
1746520873	1950	1950	Kill the Umpire	Kill the Umpire	Bacon, Lloyd	Lloyd Bacon	tt0042641
1746503689	1921	1921	The Love Light	The Love Light	Marion, Frances	Frances Marion	tt0012408
1746555891	1954	1954	Human Desire	Human Desire	Lang, Fritz	Fritz Lang	tt0047101
1746583491	1929	1929	Far Western Trails	Far Western Trails	Horner, Robert J.	Robert J. Horner	tt0325358
1746574906	1938	1938	There Goes My Heart	There Goes My Heart	McLeod, Norman Z.	Norman Z. McLeod	tt0030856
1746183504	1910	1910	Away Out West	Away Out West	Anderson, G. M.	Gilbert M. 'Broncho Billy' Anderson	tt0001123
1746394021	1986	1986	Howard the Duck	Howard the Duck	Huyck, Willard	Willard Huyck	tt0091225
1746566220	1954	1953	Sea of Lost Ships	Sea of Lost Ships	Kane, Joseph	Joseph Kane	tt0046287
1769970685	1968	1968	Motel Wives	Motel Wives	Clay, Adam	Adam Clay	tt0251864
1746569773	1953	1953	Paris Model	Paris Model	Green, Alfred E.	Alfred E. Green	tt0046165
1746394728	1992	1992	Beethoven	Beethoven	Levant, Brian	Brian Levant	tt0103786
1746553228	1963	1962	Diamond Head	Diamond Head	Green, Guy	Guy Green	tt0055905
1746491988	1945	1945	Patrick the Great	Patrick the Great	Ryan, Frank	Frank Ryan	tt0037975
1746216650	1967	1967	The Busy Body	The Busy Body	Castle, William	William Castle	tt0061431
1746486466	1921	1921	The Sage Hen	The Sage Hen	Lewis, Edgar	Edgar Lewis	tt0012641
1746208689	1966	1966	The Russians Are Coming The Russians Are Comir	The Russians Are Coming the Russians Are Co	Jewison, Norman	Norman Jewison	tt0060921
1746585472	1939	1939	City in Darkness	City in Darkness	Leeds, Herbert I.	Herbert I. Leeds	tt0031148
1746520032	1949	1949	Mr. Belvedere Goes to College	Mr. Belvedere Goes to College	Nugent, Elliott	Elliott Nugent	tt0041662
1746514392	1916	1916	The Embodied Thought	The Embodied Thought	Sloman, Edward	Edward Sloman	tt0322163
1746410701	1994	1994	Angie	Angie	Coolidge, Martha	Martha Coolidge	tt0109129
1746564485	1952	1952	Scaramouche	Scaramouche	Sidney, George	George Sidney	tt0045125
1746580923	1963	1963	All the Way Home	All the Way Home	Segal, Alex	Alex Segal	tt0056818
1746570286	1933	1933	Our Betters	Our Betters	Cukor, George	George Cukor	tt0024421
1746585125	1929	1929	Two Sisters	Two Sisters	Pembroke, Scott	Scott Pembroke	tt0020526
1746581520	1928	1928	Scarlet Youth	Scarlet Youth	Curran, William Hughes	William Hughes Curran	tt0019359
1746502913	1927	1927	The Heart Thief	The Heart Thief	Chrisander, Nils Olaf	Nils Olaf Chrisander	tt0017972
1746217646	1967	1967	The Born Losers	The Born Losers	Frank, T. C.	Tom Laughlin	tt0061420
1746566168	1956	1956	The Man Who Knew Too Much	The Man Who Knew Too Much	Hitchcock, Alfred	Alfred Hitchcock	tt0049470
1746567748	1952	1952	Because of You	Because of You	Pevney, Joseph	Joseph Pevney	tt0044403
1746507576	1926	1926	King of the Pack	King of the Pack	Richardson, Frank	Frank Richardson	tt0126369
1746408921	1992	1992	Innocent Blood	Innocent Blood	Landis, John	John Landis	tt0104511
1746568276	1934	1934	Hitler's Reign of Terror	Hitler's Reign of Terror	Mindlin, Michael	Michael Mindlin	tt0213716
1746578034	1935	1935	Stone of Silver Creek	Stone of Silver Creek	Grinde, Nick	Nick Grinde	tt0027044
1746500087	1948	1948	In This Corner	In This Corner	Riesner, Charles F.	Charles Reisner	tt0189604
1746454067	1943	1943	This Land Is Mine	This Land Is Mine	Renoir, Jean	Jean Renoir	tt0036431
1746580249	1938	1938	Nancy Drew, Detective	Nancy Drew: Detective	Clemens, William	William Clemens	tt0030491
1746567584	1952	1952	Fearless Fagan	Fearless Fagan	Donen, Stanley	Stanley Donen	tt0044608
1746574530	1933	1933	Gigolettes of Paris	Gigolettes of Paris	Martell, Alphonse	Alphonse Martell	tt0024059
1746409946	1984	1984	Unfaithfully Yours	Unfaithfully Yours	Zieff, Howard	Howard Zieff	tt0088326
1746208614	1969	1968	I Sailed to Tahiti With an All Girl Crew	I Sailed to Tahiti with an All Girl Crew	Bare, Richard	Richard L. Bare	tt0063117
1746570357	1934	1934	George White's Scandals	George White's Scandals		Thornton Freeland	tt0025166
1746520476	1925	1925	The White Monkey	The White Monkey	Rosen, Phil	Phil Rosen	tt0016525
1746235803	1977	1977	Pumping Iron	Pumping Iron	Butler, George; Fiore, Robert	George Butler	tt0076578
1746579056	1951	1951	Street Bandits	Street Bandits	Springsteen, R. G.	R.G. Springsteen	tt0044080
1746483203	1917	1917	The Devil's Playground	The Devil's Playground	Webster, Harry McRae	Harry McRae Webster	tt0182976
1746497056	1922	1922	The Marshal of Moneymint	The Marshal of Moneymint	Clements, Roy	Roy Clements	tt0332700
1746186088	1968	1968	The Stalking Moon	The Stalking Moon	Mulligan, Robert	Robert Mulligan	tt0065032
1746512315	1922	1922	Domestic Relations	Domestic Relations	Withey, Chet	Chester Withey	tt0013077
1746410543	1987	1987	Down Twisted	Down Twisted	Pyun, Albert	Albert Pyun	tt0092922
1746560086	1965	1965	Fort Courageous	Fort Courageous	Selander, Lesley	Lesley Selander	tt0122071

1746475267	1948	1948	16 Fathoms Deep	16 Fathoms Deep	Allen, Irving	Irving Allen	tt0040797
1746508474	1926	1926	The Mystery Club	The Mystery Club	Blaché, Herbert	Herbert Blaché	tt0017191
1746497934	1919	1919	A Taste of Life	A Taste of Life	Dillon, Jack	John Francis Dillon	tt0010759
1746503013	1923	1923	Little Old New York	Little Old New York	Olcott, Sidney	Sidney Olcott	tt0014205
1746219345	1975	1975	Cleopatra Jones and the Casino of Gold	Cleopatra Jones and the Casino of Gold	Bail, Chuck	Charles Bail	tt0072791
1746581164	1960	1960	Walk Like a Dragon	Walk Like a Dragon	Clavell, James	James Clavell	tt0054457
1746570153	1933	1933	India Speaks	India Speaks		Walter Futter	tt0024178
1746475926	1944	1944	Jungle Woman	Jungle Woman	LeBorg, Reginald	Reginald Le Borg	tt0036975
1746504394	1919	1919	Jinx	Jinx	Schertzinger, Victor L.	Victor Schertzinger	tt0010313
1746583274	1928	1928	Craig's Wife	Craig's Wife	de Mille, William C.	William C. de Mille	tt0018800
1746582046	1928	1928	Adoration	Adoration	Lloyd, Frank	Frank Lloyd	tt0018622
1746475725	1948	1948	The Gallant Legion	The Gallant Legion	Kane, Joe	Joseph Kane	tt0040383
1746210799	1970	1970	Airport	Airport	Seaton, George	George Seaton	tt0065377
1746578911	1950	1950	The Texan Meets Calamity Jane	The Texan Meets Calamity Jane	Lamb, Ande	Ande Lamb	tt0043035
1746454181	1996	1996	The Phantom	The Phantom	Wincer, Simon	Simon Wincer	tt0117331
1746502586	1910	1910	The Salt on the Bird's Tail	Salt on the Bird's Tail		Gaston Méliès	tt0274077
1746507731	1926	1926	The Speeding Venus	The Speeding Venus	Thornby, Robert	Robert Thornby	tt0017424
1746270447	1979	1979	Swap Meet	Swap Meet	Mack, Brice	Brice Mack	tt0079974
1746520440	1926	1926	The Ramblin' Galoot	The Ramblin' Galoot	Bain, Fred	Fred Bain	tt0017308
1746566342	1953	1953	Stalag 17	Stalag 17	Wilder, Billy	Billy Wilder	tt0046359
1746198445	1966	1966	Las Vegas Hillbillys	Las Vegas Hillbillys	Pierce, Arthur C.	Arthur C. Pierce	tt0060618
1746570416	1934	1934	Thirty Day Princess	Thirty Day Princess	Gering, Marion	Marion Gering	tt0025880
1746257196	1979	1978	Starcrash	Starcrash	Coates, Lewis	Luigi Cozzi	tt0079946
1746584891	1939	1939	Sued for Libel	Sued for Libel	Goodwins, Leslie	Leslie Goodwins	tt0033113
1746566373	1956	1956	The Ten Commandments	The Ten Commandments	DeMille, Cecil B.	Cecil B. DeMille	tt0049833
1746555962	1957	1957	Quantez	Quantez	Keller, Harry	Harry Keller	tt0050872
1746542629	1926	1926	The Roaring Road	Roaring Road	Hurst, Paul	Paul Hurst	tt0461833
1746578865	1958	1958	The Thing That Couldn't Die	The Thing That Couldn't Die	Cowan, Will	Will Cowan	tt0052289
1746570994	1933	1933	The Fiddlin' Buckaroo	The Fiddlin' Buckaroo	Maynard, Ken	Ken Maynard	tt0025108
1746408459	1983	1982	The Dorm that Dripped Blood	The Dorm That Dripped Blood	Obrow, Jeffrey; Carpenter, Stephen	Stephen Carpenter	tt0082279
1746570394	1933	1933	A Study in Scarlet	A Study in Scarlet	Marin, Edwin L.	Edwin L. Marin	tt0024625
1746409244	1988	1988	18 Again!	18 Again!	Flaherty, Paul	Paul Flaherty	tt0094593
1746574670	1936	1936	Beloved Enemy	Beloved Enemy	Potter, H. C.	H.C. Potter	tt0027345
1746436546	1940	1940	Charlie Chan at the Wax Museum	Charlie Chan at the Wax Museum	Shores, Lynn	Lynn Shores	tt0032324
1745359934	2004	2004	Anacondas: The Hunt for the Blood Orchid	Anacondas: The Hunt for the Blood Orchid	Little, Dwight	Dwight H. Little	tt0366174
1746512401	1916	1916	The Stronger Love	The Stronger Love	Lloyd, Frank	Frank Lloyd	tt0007405
1746578500	1951	1951	Cyclone Fury	Cyclone Fury	Nazarro, Ray	Ray Nazarro	tt0043441
1746270332	1978	1978	Here Come the Tigers	Here Come the Tigers	Cunningham, Sean S.	Sean S. Cunningham	tt0077668
1745302643	2005	2005	The Amityville Horror	The Amityville Horror	Douglas, Andrew	Andrew Douglas	tt0384806
1746393154	1985	1985	1918	1918	Harrison, Ken	Ken Harrison	tt0088645
1746497861	1917	1917	Sold at Auction	Sold at Auction	MacDonald, Sherwood	Sherwood MacDonald	tt0008600
1746465195	1947	1947	Where There's Life	Where There's Life	Lanfield, Sidney	Sidney Lanfield	tt0039982
1746420889	1996	1996	Man with a Plan	Man with a Plan	O'Brien, John	John O'Brien	tt0113758
1746582390	1930	1930	Good Intentions	Good Intentions	Howard, William K.	William K. Howard	tt0020928
1746560634	1932	1932	Her Mad Night	Her Mad Night	Hopper, E. Mason	E. Mason Hopper	tt0022995
1769970174	1912	1912	The End of the Romance	The End of the Romance		Frank Montgomery	tt0401400
1746581737	1928	1927	The Shield of Honor	The Shield of Honor	Johnson, Emory	Emory Johnson	tt0018388
1746525043	1921	1921	Mother Eternal	Mother Eternal	Abramson, Ivan	Ivan Abramson	tt0012478
1746393362	1986	1986	Ferris Bueller's Day Off	Ferris Bueller's Day Off	Hughes, John	John Hughes	tt0091042
1746578720	1959	1959	Al Capone	Al Capone	Wilson, Richard	Richard Wilson	tt0052543
1746495624	1920	1920	Homer Comes Home	Homer Comes Home	Storm, Jerome	Jerome Storm	tt0195795
1746568174	1933	1933	On Your Guard	On Your Guard	Crone, George	George Crone	tt0187371
1746474120	1946	1946	Lady Luck	Lady Luck	Marin, Edwin L.	Edwin L. Marin	tt0038680
1746584988	1940	1940	Road to Singapore	Road to Singapore	Schertzinger, Victor	Victor Schertzinger	tt0032993
1746208370	1966	1966	Beau Geste	Beau Geste	Heyes, Douglas	Douglas Heyes	tt0060155
1746465033	1945	1945	Outlaws of the Rockies	Outlaws of the Rockies	Nazarro, Ray	Ray Nazarro	tt0037965
1746570138	1952	1952	With a Song in My Heart	With a Song in My Heart	Lang, Walter	Walter Lang	tt0045333

1746563815	1952	1952	Boots Malone	Boots Malone	Dieterle, William	William Dieterle	tt0044437
1746582816	1935	1935	Peter Ibbetson	Peter Ibbetson	Hathaway, Henry	Henry Hathaway	tt0026866
1745302452	2006	2006	The Beales of Grey Gardens	The Beales of Grey Gardens	Maysles, Albert; Maysles, David	Albert Maysles	tt0839739
1746581809	1950	1950	Snow Dog	Snow Dog	McDonald, Frank	Frank McDonald	tt0042979
1746393642	1981	1981	The Four Seasons	The Four Seasons	Alda, Alan	Alan Alda	tt0082405
1746585437	1940	1940	Marked Men	Marked Men	Scott, Sherman	Sam Newfield	tt0032763
1769970697	1968	1968	The Young Man's Bride	The Young Man's Bride	Gunter, George	George Gunter	tt0261443
1746507880	1925	1925	My Neighbor's Wife	My Neighbor's Wife	Geldert, Clarence	Clarence Geldart	tt0016135
1746583507	1930	1930	Man to Man	Man to Man	Dwan, Allan	Allan Dwan	tt0021118
1746509171	1924	1924	Feet of Clay	Feet of Clay	De Mille, Cecil B.	Cecil B. DeMille	tt0014881
1746563364	1952	1953	A Virgin in Hollywood	A Virgin in Hollywood	Kirby, Klaytan W.	Klaytan W. Kirby	tt0129544
1746580643	1935	1935	Timber Terrors	Timber Terrors	Emmett, Robert	Robert Emmett Tansey	tt0027108
1746465330	1947	1947	Bowery Buckaroos	Bowery Buckaroos	Beaudine, William	William Beaudine	tt0039216
1746583814	1929	1929	Queen of the Night Clubs	Queen of the Night Clubs	Foy, Bryan	Bryan Foy	tt0020299
1746423468	1996	1996	Eye for an Eye	Eye for an Eye	Schlesinger, John	John Schlesinger	tt0116260
1746555965	1956	1956	Hot Shots	Hot Shots	Yarbrough, Jean	Jean Yarbrough	tt0049336
1746561784	1957	1956	Beyond Mombasa	Beyond Mombasa	Marshall, George	George Marshall	tt0049005
1746453775	1944	1944	Carolina Blues	Carolina Blues	Jason, Leigh	Leigh Jason	tt0036698
1746454875	1994	1994	PCU	PCU	Bochner, Hart	Hart Bochner	tt0110759
1746582051	1950	1950	Riding High	Riding High	Capra, Frank	Frank Capra	tt0042893
1746570461	1934	1934	Death on the Diamond	Death on the Diamond	Sedgwick, Edward	Edward Sedgwick	tt0025039
1746465200	1944	1944	Pin Up Girl	Pin Up Girl	Humberstone, Bruce	H. Bruce Humberstone	tt0037175
1746573766	1958	1958	Girl with an Itch	Girl with an Itch	Ashcroft, Ronnie	Ronald V. Ashcroft	tt0160310
1746454997	1996	1995	Heavy	Heavy	Mangold, James	James Mangold	tt0113280
1746584255	1939	1939	The Day the Bookies Wept	The Day the Bookies Wept	Goodwins, Leslie	Leslie Goodwins	tt0031214
1746440252	1996	1996	The Arrival	The Arrival	Twohy, David	David Twohy	tt0115571
1745375293	2008	2008	Zack and Miri Make a Porno	Zack and Miri Make a Porno	Smith, Kevin	Kevin Smith	tt1007028
1746499862	1949	1949	Impact	Impact	Lubin, Arthur	Arthur Lubin	tt0041503
1746501897	1910	1910	A Postal Substitute	A Postal Substitute		William F. Haddock	tt2236946
1746577200	1951	1951	The Racket	The Racket	Cromwell, John	John Cromwell	tt0043955
1746423372	1999	1999	Terror Firmer	Terror Firmer	Kaufman, Lloyd	Lloyd Kaufman	tt0169299
1745342931	2008	2008	Expelled: No Intelligence Allowed	Expelled: No Intelligence Allowed	Frankowski, Nathan	Nathan Frankowski	tt1091617
1746497640	1919	1919	What Every Woman Learns	What Every Woman Learns	Niblo, Fred	Fred Niblo	tt0010869
1746583144	1939	1939	All Women Have Secrets	All Women Have Secrets	Neumann, Kurt	Kurt Neumann	tt0031031
1746523056	1927	1927	Hands Off	Hands Off	Laemmle, Ernst	Ernst Laemmle	tt0017959
1746503682	1922	1921	The Kingfisher's Roost	KingFisher's Roost	Chaudet, Louis; Hurst, Paul	Louis Chaudet	tt0013294
1746509010	1924	1924	The Heritage of the Desert	The Heritage of the Desert	Willat, Irvin	Irvin Willat	tt0014984
1746569728	1958	1957	Lost Lagoon	Lost Lagoon	Rawlins, John	John Rawlins	tt0051880
1746410416	1990	1990	Gremlins 2: The New Batch	Gremlins 2: The New Batch	Dante, Joe	Joe Dante	tt0099700
1746509265	1924	1924	Why Men Leave Home	Why Men Leave Home	Stahl, John M.	John M. Stahl	tt0015497
1746474966	1946	1946	Monsieur Beaucaire	Monsieur Beaucaire	Marshall, George	George Marshall	tt0038747
1746520244	1949	1949	Hellfire	Hellfire	Springsteen, R. G.	R.G. Springsteen	tt0041453
1746499841	1947	1947	Hit Parade of 1947	Hit Parade of 1947	McDonald, Frank	Frank McDonald	tt0039465
1746208259	1969	1969	Me and My Brother	Me and My Brother	Frank, Robert	Robert Frank	tt0063286
1746437428	1942	1942	Wildcat	Wildcat	McDonald, Frank	Frank McDonald	tt0035562
1746577145	1962	1962	Lonely Are the Brave	Lonely Are the Brave	Miller, David	David Miller	tt0056195
1745309532	2005	2005	Hitch	Hitch	Tennant, Andy	Andy Tennant	tt0386588
1746555741	1955	1955	I'll Cry Tomorrow	I'll Cry Tomorrow	Mann, Daniel	Daniel Mann	tt0048191
1746524857	1916	1916	Purity	Purity	Berger, Rea	Rae Berger	tt0007229
1746500456	1925	1925	New Champion	The New Champion	Eason, Reeves	B. Reeves Eason	tt0016152
1746473937	1945	1945	Dangerous Partners	Dangerous Partners	Cahn, Edward L.	Edward L. Cahn	tt0037634
1746520806	1925	1925	Range Justice	Range Justice	Hayes, Ward	Ward Hayes	tt0016262
1746496117	1917	1917	The Penny Philanthropist	The Penny Philanthropist	McConnell, Guy	Guy McConnell	tt0181762
1746574046	1959	1959	Odds Against Tomorrow	Odds Against Tomorrow	Wise, Robert	Robert Wise	tt0053133
1746554726	1965	1965	Dr. Goldfoot and the Bikini Machine	Dr. Goldfoot and the Bikini Machine	Taurog, Norman	Norman Taurog	tt0059124
1746582853	1935	1935	Five Bad Men	Five Bad Men	Smith, Cliff	Clifford Smith	tt0206751
1746520268	1926	1926	The Social Highwayman	The Social Highwayman	Beaudine, William	William Beaudine	tt0017412

1746453819	1940	1940	The Showdown	The Showdown	Bretherton, Howard	Howard Bretherton	tt0033047
1745372902	2004	2004	Walking Tall	Walking Tall	Bray, Kevin	Kevin Bray	tt0351977
1746574697	1933	1933	Hallelujah I'm a Bum	Hallelujah I'm a Bum	Milestone, Lewis	Lewis Milestone	tt0024083
1746583945	1929	1929	Married in Hollywood	Married in Hollywood	Silver, Marcel	Marcel Silver	tt0020147
1746410874	1993	1993	Wrestling Ernest Hemingway	Wrestling Ernest Hemingway	Haines, Randa	Randa Haines	tt0108596
1746485154	1919	1919	Heads Win	Heads Win	Kendall, Preston	Preston Kendall	tt0010224
1746424620	1941	1941	Federal Fugitives	Federal Fugitives	Beaudine, William	William Beaudine	tt0033595
1746503762	1921	1921	The Sheik	The Sheik	Melford, George	George Melford	tt0012675
1746576996	1958	1959	The Mating Urge	The Mating Urge			tt0272722
1746496805	1919	1919	Taxi	Taxi	Windom, Lawrence	Lawrence C. Windom	tt0194434
1746421591	1992	1992	Encino Man	Encino Man	Mayfield, Les	Les Mayfield	tt0104187
1746475986	1944	1944	Bowery Champs	Bowery Champs	Beaudine, William	William Beaudine	tt0036667
1746410715	1994	1994	Silent Fall	Silent Fall	Beresford, Bruce	Bruce Beresford	tt0111187
1746577300	1960	1960	Ten Who Dared	Ten Who Dared	Beaudine, William	William Beaudine	tt0054372
1745341767	2003	2003	A Man Apart	A Man Apart	Gray, F. Gary	F. Gary Gray	tt0266465
1769969343	1931	1931	A Burglar to the Rescue	A Burglar to the Rescue	Cochrane, George	George Cochrane	tt0174527
1745372344	2009	2009	Post Grad	Post Grad	Jenson, Vicky	Vicky Jenson	tt1142433
1746509327	1925	1925	Pretty Ladies	Pretty Ladies	Bell, Monta	Monta Bell	tt0016241
1746516139	1920	1920	The Love Flower	The Love Flower	Griffith, D. W.	D.W. Griffith	tt0011415
1746523317	1913	1913	Checkers	Checkers	Thomas, Augustus	Augustus E. Thomas	tt0002746
1746497050	1917	1917	The Greater Woman	The Greater Woman	Powell, Frank	Frank Powell	tt0006741
1769970844	1915	1915	The Hunt	The Taunt	Sterling, Ford; Parrott, Charles	Edward Sloman	tt0463221
1746422733	1940	1940	Seventeen	Seventeen	King, Louis	Louis King	tt0033039
1746576994	1961	1961	Marines, Let's Go!	Marines, Let's Go	Walsh, Raoul	Raoul Walsh	tt0055144
1746420960	1994	1994	Love and a .45	Love and a .45	Talkington, C. M.	C.M. Talkington	tt0110395
1746584012	1935	1935	Justice of the Range	Justice of the Range	Selman, David	David Selman	tt0026555
1746571573	1932	1932	End of the Trail	End of the Trail	Lederman, D. Ross	D. Ross Lederman	tt0022856
1746465111	1944	1944	Sing a Jingle	Sing a Jingle	Lilley, Edward	Edward C. Lilley	tt0036358
1746410388	1992	1992	Brain Donors	Brain Donors	Dugan, Dennis	Dennis Dugan	tt0103872
1746409693	1991	1991	The Commitments	The Commitments	Parker, Alan	Alan Parker	tt0101605
1746562130	1954	1954	The Shanghai Story	The Shanghai Story	Lloyd, Frank	Frank Lloyd	tt0047475
1746454370	1941	1941	Tarzan's Secret Treasure	Tarzan's Secret Treasure	Thorpe, Richard	Richard Thorpe	tt0034266
1746585094	1930	1930	The Squealer	The Squealer	Brown, Harry J.	Harry Joe Brown	tt0021416
1745340484	2003	2002	XX/XY	XX/XY	Chick, Austin	Austin Chick	tt0245573
1746584161	1937	1937	The Awful Truth	The Awful Truth	McCarey, Leo	Leo McCarey	tt0028597
1746533200	1915	1915	The Fairy and the Waif	The Fairy and the Waif	Irving, George; Frohman, Marie Hub	Marie Hubert Frohman	tt0005298
1746570433	1933	1933	By Candlelight	By Candlelight	Whale, James; Wyler, Robert	James Whale	tt0023860
1746574298	1950	1950	Mule Train	Mule Train	English, John	John English	tt0042761
1746435105	1941	1941	Ellery Queen's Penthouse Mystery	Ellery Queen's Penthouse Mystery	Hogan, James	James P. Hogan	tt0033574
1746582482	1935	1935	The Affair of Susan	The Affair of Susan	Neumann, Kurt	Kurt Neumann	tt0026041
1746573990	1956	1956	The Werewolf	The Werewolf	Sears, Fred F.	Fred F. Sears	tt0049944
1769970421	1965	1965	The Smut Peddler	The Smut Peddler	Rose, Warner	William Rose	tt0242893
1745282798	2006	2006	The Wicker Man	The Wicker Man	LaBute, Neil	Neil LaBute	tt0450345
1746436126	1942	1942	Rubber Racketeers	Rubber Racketeers	Young, Harold	Harold Young	tt0035273
1746520988	1949	1949	Haunted Trails	Haunted Trails	Hillyer, Lambert	Lambert Hillyer	tt0041448
1746409310	1984	1984	They're Playing With Fire	They're Playing with Fire	Avedis, Howard	Howard Avedis	tt0088255
1746561178	1932	1932	The World and the Flesh	World and the Flesh	Cromwell, John	John Cromwell	tt0023709
1746570854	1934	1934	Long Lost Father	Long Lost Father	Schoedsack, Ernest B.	Ernest B. Schoedsack	tt0025417
1745271727	2001	2001	The Anniversary Party	The Anniversary Party	Leigh, Jennifer Jason; Cumming, Alar	Alan Cumming	tt0254099
1746577898	1939	1939	Dark Victory	Dark Victory	Goulding, Edmund	Edmund Goulding	tt0031210
1746257245	1980	1980	Urban Cowboy	Urban Cowboy	Bridges, James	James Bridges	tt0081696
1746454710	1995	1995	Nick of Time	Nick of Time	Badham, John	John Badham	tt0113972
1746580374	1935	1935	The Public Menace	The Public Menace	Kenton, Erle C.	Erle C. Kenton	tt0026897
1746545214	1940	1940	Sailor's Lady	Sailor's Lady	Dwan, Allan	Allan Dwan	tt0033011
1746455135	1999	1999	The Bone Collector	The Bone Collector	Noyce, Phillip	Phillip Noyce	tt0145681
1746420866	1990	1990	Listen Up: The Lives of Quincy Jones	Listen Up: The Lives of Quincy Jones	Weissbrod, Ellen	Ellen Weissbrod	tt0102315
1746410712	1991	1991	Don't Tell Mom the Babysitter's Dead	Don't Tell Mom the Babysitter's Dead	Herek, Stephen	Stephen Herek	tt0101757

1746500083	1946	1946	The Bamboo Blonde	The Bamboo Blonde	Mann, Anthony	Anthony Mann	tt0038324
1746421320	1994	1994	Camp Nowhere	Camp Nowhere	Prince, Jonathan	Jonathan Prince	tt0109369
1746524747	1910	1910	Speed Versus Death	Speed Versus Death		William F. Haddock	tt2239144
1746554974	1965	1965	Deadwood '76	Deadwood '76	Landis, James	James Landis	tt0059093
1746577846	1938	1938	Rhythm of the Saddle	Rhythm of the Saddle	Sherman, George	George Sherman	tt0030678
1746580908	1938	1938	Gangs of New York	Gangs of New York	Cruze, James	James Cruze	tt0030169
1746454965	1942	1942	Outlaws of Pine Ridge	Outlaws of Pine Ridge	Witney, William	William Witney	tt0035163
1746409311	1987	1987	Broadcast News	Broadcast News	Brooks, James L.	James L. Brooks	tt0092699
1746576976	1951	1951	Disc Jockey	Disc Jockey	Jason, Will	Will Jason	tt0133713
1746498323	1921	1921	Tuning Up	Tuning Up	Smith, Hamilton	Hamilton Smith	tt1669255
1746569995	1958	1958	Gunsmoke in Tucson	Gunsmoke in Tucson	Carr, Thomas	Thomas Carr	tt0051691
1746211072	1973	1973	Five on the Black Hand Side	Five on the Black Hand Side	Williams, Oscar	Oscar Williams	tt0070063
1746500418	1925	1925	A Daughter of the Sioux	A Daughter of the Sioux	Wilson, Ben	Ben F. Wilson	tt0015738
1746514620	1923	1923	The Spider and the Rose	The Spider and the Rose	McDermott, John	John McDermott	tt0014502
1746504354	1922	1922	Smiling Jim	Smiling Jim	Franz, Joseph	Joseph Franz	tt0013612
1746580588	1935	1935	Legong (Dance of the Virgins)	Legong: Dance of the Virgins	Falaise, Henri de la	Henri de la Falaise	tt0129196
1746574636	1936	1936	Avenging Waters	Avenging Waters	Bennet, Spencer Gordon	Spencer Gordon Bennet	tt0027322
1746249073	1975	1974	The Life and Times of Grizzly Adams	The Life and Times of Grizzly Adams	Friedenberg, Richard	Richard Friedenberg	tt0074797
1746453575	1941	1941	Sundown	Sundown	Hathaway, Henry	Henry Hathaway	tt0034242
1746491867	1948	1948	The Sainted Sisters	The Sainted Sisters	Russell, William D.	William D. Russell	tt0040754
1745339962	2002	2001	No Such Thing	No Such Thing	Hartley, Hal	Hal Hartley	tt0248190
1746435697	1941	1941	South of Tahiti	South of Tahiti	Waggner, George	George Waggner	tt0034222
1745293626	2006	2006	Keeping Up with the Steins	Keeping Up with the Steins	Marshall, Scott	Scott Marshall	tt0415949
1746439453	1998	1998	The Parent Trap	The Parent Trap	Meyers, Nancy	Nancy Meyers	tt0120783
1746419926	1986	1986	Flight of the Navigator	Flight of the Navigator	Kleiser, Randal	Randal Kleiser	tt0091059
1746570360	1934	1934	Whom the Gods Destroy	Whom the Gods Destroy	Lang, Walter	Walter Lang	tt0025987
1746474539	1944	1944	Reckless Age	Reckless Age	Feist, Felix E.	Felix E. Feist	tt0037214
1746583109	1939	1939	Chicken Wagon Family	Chicken Wagon Family	Leeds, Herbert I.	Herbert I. Leeds	tt0031154
1746500883	1910	1910	Pals of the Range	Pals of the Range		Gilbert M. 'Broncho Billy' Anderson	tt0001354
1746530757	1948	1948	Gun Smugglers	Gun Smugglers	McDonald, Frank	Frank McDonald	tt0040409
1746550131	1954	1954	The Desperado	The Desperado	Carr, Thomas	Thomas Carr	tt0046905
1746235282	1976	1976	I Will...I Will...For Now	I Will... I Will... For Now	Panama, Norman	Norman Panama	tt0074659
1746491703	1944	1944	Vigilantes of Dodge City	Vigilantes of Dodge City	Grissell, Wallace	Wallace Grissell	tt0037433
1746575029	1933	1933	Matto-Grosso	Matto Grosso	Clarke, John S.; Crosby, Floyd; Newel	John S. Clark Jr.	tt0024312
1746474823	1947	1947	Devil Ship	Devil Ship	Landers, Lew	Lew Landers	tt0039316
1746436085	1943	1943	Follow the Band	Follow the Band	Yarbrough, Jean	Jean Yarbrough	tt0035894
1745271956	2001	2001	Harry Potter and the Sorcerer's Stone	Harry Potter and the Sorcerer's Stone	Columbus, Chris	Chris Columbus	tt0241527
1746581470	1951	1951	Tomorrow Is Another Day	Tomorrow Is Another Day	Feist, Felix	Felix E. Feist	tt0044136
1746273854	1980	1980	Divine Madness	Divine Madness	Ritchie, Michael	Michael Ritchie	tt0080634
1746575014	1938	1938	The Crowd Roars	The Crowd Roars	Thorpe, Richard	Richard Thorpe	tt0030031
1746424202	1940	1940	Sky Murder	Sky Murder	Seitz, George B.	George B. Seitz	tt0033059
1746581860	1950	1950	South Sea Sinner	South Sea Sinner	Humberstone, Bruce	H. Bruce Humberstone	tt0041904
1746566257	1954	1954	Them!	Them!	Douglas, Gordon	Gordon Douglas	tt0047573
1746508984	1926	1926	Going Crooked	Going Crooked	Melford, George	George Melford	tt0016927
1746581654	1928	1928	South of Panama	South of Panama	Hunt, Charles J.	Charles J. Hunt	tt0165954
1746503801	1919	1919	The Unwritten Code	The Unwritten Code	Durning, Bernard J.	Bernard J. Durning	tt0010827
1746514887	1923	1923	Forbidden Lover	Forbidden Lover	Deverich, Nat	Nat G. Deverich	tt0014055
1746465181	1942	1943	Keeper of the Flame	Keeper of the Flame	Cukor, George	George Cukor	tt0034936
1746573572	1910	1910	Dora Thorne	Dora Thorne			tt0221130
1746570756	1952	1952	Apache Country	Apache Country	Archainbaud, George	George Archainbaud	tt0044367
1746499980	1924	1924	Dorothy Vernon of Haddon Hall	Dorothy Vernon of Haddon Hall	Neilan, Marshall	Marshall Neilan	tt0014854
1746580827	1935	1935	China Seas	China Seas	Garnett, Tay	Tay Garnett	tt0026205
1746581603	1928	1928	State Street Sadie	State Street Sadie	Mayo, Archie	Archie Mayo	tt0166822
1746437025	1940	1940	The Mummy's Hand	The Mummy's Hand	Cabanne, Christy	Christy Cabanne	tt0032818
1746465309	1947	1947	Stork Bites Man	Stork Bites Man	Endfield, Cyril	Cy Endfield	tt0039869
1746566857	1933	1933	Girl Missing	Girl Missing	Florey, Robert	Robert Florey	tt0024062
1746580754	1937	1937	She Asked for It	She Asked for It	Kenton, Erle C.	Erle C. Kenton	tt0029547

1746492441	1947	1947	West of Dodge City	West of Dodge City	Nazarro, Ray	Ray Nazarro	tt0039978
1746465152	1947	1947	Banjo	Banjo	Fleischer, Richard O.	Richard Fleischer	tt0039174
1746486293	1917	1917	Mr. Opp	Mr. Opp	Reynolds, Lynn F.	Lynn Reynolds	tt0008333
1746491803	1948	1948	Every Girl Should Be Married	Every Girl Should Be Married	Hartman, Don	Don Hartman	tt0040331
1746569511	1954	1954	Cannibal Attack	Cannibal Attack	Sholem, Lee	Lee Sholem	tt0046822
1746584828	1929	1929	The Madonna of Avenue A	Madonna of Avenue A	Curtiz, Michael	Michael Curtiz	tt0020128
1746580437	1938	1938	Romance on the Run	Romance on the Run	Meins, Gus	Gus Meins	tt0030694
1746233035	1967	1967	The Cool Ones	The Cool Ones	Nelson, Gene	Gene Nelson	tt0061514
1746566882	1932	1932	Skyscraper Souls	Skyscraper Souls	Selwyn, Edgar	Edgar Selwyn	tt0023486
1746524838	1923	1923	Jamestown	Jamestown	Hollywood, Edwin L.	Edwin L. Hollywood	tt0135479
1746577199	1959	1959	The Shaggy Dog	The Shaggy Dog	Barton, Charles	Charles Barton	tt0053271
1746420874	1993	1993	Mrs. Doubtfire	Mrs. Doubtfire	Columbus, Chris	Chris Columbus	tt0107614
1746513718	1928	1928	Lights of New York	Lights of New York	Foy, Bryan	Bryan Foy	tt0019096
1746182746	1910	1910	The Broken Doll	The Broken Doll	Griffith, D. W.	D.W. Griffith	tt0001135
1746502001	1915	1915	The Flaming Sword	The Flaming Sword	Middleton, E.	Edwin Middleton	tt0005330
1746421192	1999	1999	Drive Me Crazy	Drive Me Crazy	Schultz, John	John Schultz	tt0164114
1746584160	1936	1936	Fatal Lady	Fatal Lady	Ludwig, Edward	Edward Ludwig	tt0027611
1746455274	1998	1998	Playing by Heart	Playing by Heart	Carroll, Willard	Willard Carroll	tt0145734
1746583802	1930	1930	The Silver Horde	The Silver Horde	Archainbaud, George	George Archainbaud	tt0021374
1746512750	1916	1916	Sudden Riches	Sudden Riches	Chautard, Emile	Emile Chautard	tt0007406
1746508136	1926	1926	Her Honor the Governor	Her Honor, the Governor	Withey, Chet	Chester Withey	tt0016958
1746514072	1915	1915	The Vampire	The Vampire	Blaché, Alice	Alice Guy	tt0146338
1746584947	1940	1940	Ride, Tenderfoot, Ride	Ride, Tenderfoot, Ride	McDonald, Frank	Frank McDonald	tt0032987
1746585048	1939	1939	Society Smugglers	Society Smugglers	May, Joe	Joe May	tt0031947
1746436534	1943	1943	O, My Darling Clementine	O, My Darling Clementine	McDonald, Frank	Frank McDonald	tt0036226
1746569660	1953	1953	Winning of the West	Winning of the West	Archainbaud, George	George Archainbaud	tt0046553
1746219422	1976	1976	Freewheelin'	Freewheelin'	Dittrich, Scott	Scott Dittrich	tt0198494
1746475928	1947	1946	Till the Clouds Roll By	Till the Clouds Roll By	Whorf, Richard	Richard Whorf	tt0039035
1745395411	2010	2010	Shutter Island	Shutter Island	Scorsese, Martin	Martin Scorsese	tt1130884
1746200480	1968	1968	Firecreek	Firecreek	McEveety, Vincent	Vincent McEveety	tt0062975
1746453794	1944	1944	Hail the Conquering Hero	Hail the Conquering Hero	Sturges, Preston	Preston Sturges	tt0036891
1746393094	1982	1982	The Last American Virgin	The Last American Virgin	Davidson, Boaz	Boaz Davidson	tt0084234
1746584268	1939	1939	Spirit of Culver	The Spirit of Culver	Santley, Joseph	Joseph Santley	tt0031962
1769970437	1965	1965	The Sexperts--Touched by Temptation	The Sexperts: Touched by Temptation	Nehemiah, J.	Jerald Intrator	tt0183762
1746497452	1920	1920	Someone in the House	Someone in the House	Ince, John E.	John Ince	tt0197902
1746408442	1982	1982	It Came from Hollywood	It Came from Hollywood	Solt, Andrew; Leo, Malcolm	Malcolm Leo	tt0084156
1746512916	1915	1915	The Eagle's Nest	The Eagle's Nest	Fielding, Romaine	Edwin Arden	tt0005233
1746520039	1925	1925	All Around Frying Pan	All Around Frying Pan		David Kirkland	tt0015565
1746410435	1986	1985	Sherman's March	Sherman's March		Ross McElwee	tt0091943
1746583257	1931	1931	The Criminal Code	The Criminal Code	Hawks, Howard	Howard Hawks	tt0021770
1746582523	1938	1938	His Exciting Night	His Exciting Night	Meins, Gus	Gus Meins	tt0030236
1746513693	1923	1923	Salome	Salome	Strauss, Malcolm	Malcolm Strauss	tt0161953
1769970171	1912	1912	Their First Kidnapping Case	Their First Kidnapping Case		Mack Sennett	tt0229772
1746533052	1914	1914	The Ghost Breaker	The Ghost Breaker	DeMille, Cecil B.; Apfel, Oscar C.	Oscar Apfel	tt0004013
1746438290	1940	1940	Sandy Gets Her Man	Sandy Gets Her Man	Garrett, Otis; Smith, Paul Gerard	Otis Garrett	tt0033018
1746199207	1969	1969	The Good Guys and the Bad Guys	The Good Guys and the Bad Guys	Kennedy, Burt	Burt Kennedy	tt0064379
1746513668	1915	1915	The Girl I Left Behind Me	The Girl I Left Behind Me	Carleton, Lloyd B.	Lloyd B. Carleton	tt0005387
1746492451	1944	1944	Two Girls and a Sailor	Two Girls and a Sailor	Thorpe, Richard	Richard Thorpe	tt0037408
1745272582	2000	2000	The Perfect Storm	The Perfect Storm	Petersen, Wolfgang	Wolfgang Petersen	tt0177971
1746580752	1936	1936	The Traitor	The Traitor	Newfield, Sam	Sam Newfield	tt0028404
1746577293	1951	1951	Darling, How Could You!	Darling, How Could You!	Leisen, Mitchell	Mitchell Leisen	tt0043454
1746583812	1928	1928	Wild West Romance	Wild West Romance	Hough, R. Lee	R.L. Hough	tt0019581
1746569502	1958	1958	Maracaibo	Maracaibo	Wilde, Cornel	Cornel Wilde	tt0051907
1746467148	1917	1917	Sins of Ambition	Sins of Ambition	Abramson, Ivan	Ivan Abramson	tt0008577
1746582607	1935	1935	The Case of the Curious Bride	The Case of the Curious Bride	Curtiz, Michael	Michael Curtiz	tt0026184
1746508937	1925	1925	The Freshman	The Freshman	Taylor, Sam; Newmeyer, Fred	Fred C. Newmeyer	tt0015841
1746513038	1915	1915	A Black Sheep	A Black Sheep	Heffron, Thomas N.	Thomas N. Heffron	tt0004977

1746520307	1925	1925	The White Outlaw	The White Outlaw	Smith, Clifford	Clifford Smith	tt0016526
1746473724	1942	1942	A Night to Remember	A Night to Remember	Wallace, Richard	Richard Wallace	tt0036203
1745359169	2010	2010	The Bounty Hunter	The Bounty Hunter	Tennant, Andy	Andy Tennant	tt1038919
1746567009	1933	1933	The Past of Mary Holmes	The Past of Mary Holmes	Thompson, Harlan	Harlan Thompson	tt0024431
1746583848	1928	1928	Burning Daylight	Burning Daylight	Brabin, Charles J.	Charles Brabin	tt0018733
1746560103	1963	1963	In the French Style	In the French Style	Parrish, Robert	Robert Parrish	tt0056096
1769976705	1929	1929	Frontier Romance	Frontier Romance	Clifton, Elmer	Elmer Clifton	tt1049075
1746582003	1928	1928	See You Later	See You Later	Yaconelli, Frank	Frank Yaconelli	tt0019365
1745309118	2005	2005	Rock School	Rock School	Argott, Don	Don Argott	tt0436727
1746453799	1944	1944	Goin' to Town	Goin' to Town	Goodwins, Leslie	Leslie Goodwins	tt0036871
1746560060	1964	1964	The Inheritance	The Inheritance	Mayer, Harold	Harold Mayer	tt0357795
1746492213	1948	1948	Secret Service Investigator	Secret Service Investigator	Springsteen, R. G.	R.G. Springsteen	tt0040768
1745271894	2000	2000	Red Planet	Red Planet	Hoffman, Antony	Antony Hoffman	tt0199753
1746561538	1955	1955	Strips Around the World	Strips Around the World	Tucker, Phil	Phil Tucker	tt1141334
1746584917	1940	1940	Broadway Melody of 1940	Broadway Melody of 1940	Taurog, Norman	Norman Taurog	tt0032284
1746475734	1948	1948	Red River	Red River	Hawks, Howard	Howard Hawks	tt0040724
1746454652	1946	1946	Strange Impersonation	Strange Impersonation	Mann, Anthony	Anthony Mann	tt0038986
1746515278	1921	1921	Patsy	Patsy	McDermott, John	John McDermott	tt0012559
1746566021	1958	1958	The Littlest Hobo	The Littlest Hobo	Rondeau, Charles R.	Charles R. Rondeau	tt0051870
1746550274	1956	1956	Picnic	Picnic	Logan, Joshua	Joshua Logan	tt0048491
1746569715	1953	1953	Sword of Venus	Sword of Venus	Daniels, Harold	Harold Daniels	tt0046388
1746585006	1939	1939	Arizona Legion	Arizona Legion	Howard, David	David Howard	tt0031052
1746485894	1918	1918	Too Fat to Fight	Too Fat to Fight	Henley, Hobart	Hobart Henley	tt0009712
1746555143	1955	1954	3 Ring Circus	3 Ring Circus	Pevney, Joseph	Joseph Pevney	tt0047582
1746509035	1926	1926	Her Sacrifice	Her Sacrifice	Lucas, Wilfred	Wilfred Lucas	tt0016961
1746523745	1916	1916	The Black Butterfly	The Black Butterfly	King, Burton L.	Burton L. King	tt0157406
1746566270	1957	1957	Copper Sky	Copper Sky	Warren, Charles Marquis	Charles Marquis Warren	tt0050265
1746582852	1935	1935	Western Frontier	Western Frontier	Herman, Albert	Albert Herman	tt0027198
1746555820	1956	1956	The Leather Saint	The Leather Saint	Ganzer, Alvin	Alvin Ganzer	tt0049436
1746509030	1927	1927	The Romantic Age	The Romantic Age	Florey, Robert	Robert Florey	tt0018336
1746453485	1941	1941	Hands Across the Rockies	Hands Across the Rockies	Hillyer, Lambert	Lambert Hillyer	tt0129982
1746500021	1924	1924	Walloping Wallace	Walloping Wallace	Thorpe, Richard; Myles, Norbert	Norbert A. Myles	tt0177351
1746453651	1941	1941	Puddin' Head	Puddin' Head	Santley, Joseph	Joseph Santley	tt0034074
1746435162	1942	1942	Mug Town	Mug Town	Taylor, Ray	Ray Taylor	tt0036176
1746550017	1954	1954	Highway Dragnet	Highway Dragnet	Juran, Nathan	Nathan Juran	tt0047087
1746532467	1923	1923	Thundering Dawn	Thundering Dawn	Garson, Harry	Harry Garson	tt0014545
1746570755	1933	1933	The Crime of the Century	The Crime of the Century	Beaudine, William	William Beaudine	tt0023918
1746475745	1946	1946	The Last Crooked Mile	The Last Crooked Mile	Ford, Philip	Philip Ford	tt0038683
1746560657	1932	1932	The Texan	The Texan	Smith, Cliff	Clifford Smith	tt0023564
1746573385	1931	1931	Ten Cents a Dance	Ten Cents a Dance	Barrymore, Lionel	Lionel Barrymore	tt0022469
1746582602	1939	1939	Flight at Midnight	Flight at Midnight	Salkow, Sidney	Sidney Salkow	tt0031320
1746508904	1925	1925	No Man's Law	No Man's Law	Andrews, Del	Del Andrews	tt0334008
1769970424	1966	1966	All My Men	All My Men		C. Davis Smith	tt0131270
1746496285	1918	1918	The Goddess of Lost Lake	The Goddess of Lost Lake	Worsley, Wallace	Wallace Worsley	tt0009120
1746408463	1984	1984	Romancing the Stone	Romancing the Stone	Zemeckis, Robert	Robert Zemeckis	tt0088011
1746495902	1918	1918	The Daredevil	The Daredevil	Grandon, Francis	Francis J. Grandon	tt0184379
1746474457	1944	1944	Enter Arsene Lupin	Enter Arsene Lupin	Beebe, Ford	Ford Beebe	tt0036794
1746219186	1973	1974	The Single Girls	The Single Girls	Sebastian, Ferd; Sebastian, Beverly	Beverly Sebastian	tt0070697
1746513846	1913	1913	The Port of Doom	The Port of Doom	Dawley, J. Searle	J. Searle Dawley	tt0003289
1746574332	1958	1958	The 7th Voyage of Sinbad	The 7th Voyage of Sinbad	Juran, Nathan	Nathan Juran	tt0051337
1746583865	1930	1930	Only the Brave	Only the Brave	Tuttle, Frank	Frank Tuttle	tt0021219
1746580837	1937	1937	The Firefly	The Firefly	Leonard, Robert Z.	Robert Z. Leonard	tt0028873
1746569527	1958	1958	As Young as We Are	As Young as We Are	Girard, Bernard	Bernard Girard	tt0051377
1746571143	1953	1952	My Cousin Rachel	My Cousin Rachel	Koster, Henry	Henry Koster	tt0044937
1746508044	1924	1924	Lightning Romance	Lightning Romance	Rogell, Albert	Albert S. Rogell	tt0015070
1746561534	1955	1955	Francis in the Navy	Francis in the Navy	Lubin, Arthur	Arthur Lubin	tt0048092
1746580711	1937	1937	Small Town Boy	Small Town Boy	Tryon, Glenn	Glenn Tryon	tt0029580

1746562425	1953	1953	Pickup on South Street	Pickup on South Street	Fuller, Samuel	Samuel Fuller	tt0046187	
1746439225	1997	1997	Cop Land	Cop Land	Mangold, James	James Mangold	tt0118887	
1746520965	1948	1948	Words and Music	Words and Music	Taurog, Norman	Norman Taurog	tt0040976	
1746235205	1979	1979	The Jerk	The Jerk	Reiner, Carl	Carl Reiner	tt0079367	
1746504898	1918	1918	The Married Virgin	The Married Virgin	Maxwell, Joe	Joseph Maxwell	tt0009356	
1746464980	1946	1946	Terror Trail	Terror Trail	Nazarro, Ray	Ray Nazarro	tt0039891	
1746561175	1932	1932	The Crusader	The Crusader	Strayer, Frank	Frank R. Strayer	tt0022794	
1746582383	1930	1930	The Devil to Pay	The Devil to Pay!	Fitzmaurice, George	George Fitzmaurice	tt0020821	
1746436551	1942	1942	Tarzan's New York Adventure	Tarzan's New York Adventure	Thorpe, Richard	Richard Thorpe	tt0035419	
1746498278	1921	1920	Guile of Women	Guile of Women	Badger, Clarence	Clarence G. Badger	tt0012244	
1746394723	1992	1992	The Distinguished Gentleman	The Distinguished Gentleman	Lynn, Jonathan	Jonathan Lynn	tt0104114	
1746572859	1910	1910	Dooley Referees the Big Fight	Dooley Referees the Big Fight		Fred J. Balshofer	tt0359180	
1746454230	1943	1943	Rhythm of the Islands	Rhythm of the Islands	Neill, Roy William	Roy William Neill	tt0036311	
1745293423	2007	2007	Blades of Glory	Blades of Glory	Speck, Will ; Gordon, Josh	Josh Gordon	tt0445934	
1746574587	1935	1935	Car 99	Car 99	Barton, Charles	Charles Barton	tt0026178	
1746523449	1916	1916	Her Father's Son	Her Father's Son	Taylor, William D.	William Desmond Taylor	tt0006789	
**1746574210**	**1962**	**1962**	**Stakeout!**	**Stakeout!**	**Landis, James**	**James Landis**	**tt0056524**	1
1745359362	2008	2008	The Strangers	The Strangers	Bertino, Bryan	Bryan Bertino	tt0482606	
1746581917	1951	1951	Buckaroo Sheriff of Texas	Buckaroo Sheriff of Texas	Ford, Philip	Philip Ford	tt0042288	
1746473908	1947	1947	Monsieur Verdoux	Monsieur Verdoux	Chaplin, Charles	Charles Chaplin	tt0039631	
1745342815	2003	2003	Bruce Almighty	Bruce Almighty	Shadyac, Tom	Tom Shadyac	tt0315327	
1746509302	1924	1924	Her Love Story	Her Love Story	Dwan, Allan	Allan Dwan	tt0014981	
1746509585	1925	1925	Borrowed Finery	Borrowed Finery	Apfel, Oscar	Oscar Apfel	tt0015640	
1746580839	1937	1937	We Have Our Moments	We Have Our Moments	Werker, Alfred L.	Alfred L. Werker	tt0029749	
1746249032	1975	1975	Blazing Stewardesses	Blazing Stewardesses	Adamson, Al	Al Adamson	tt0071231	
1746575380	1910	1910	The Count of Montebello	The Count of Montebello		Harry Solter	tt0200548	
1746491735	1947	1947	Twilight on the Rio Grande	Twilight on the Rio Grande	McDonald, Frank	Frank McDonald	tt0039924	
1746571038	1910	1910	The Confederate Spy	The Confederate Spy		Sidney Olcott	tt0139125	
1745282672	2004	2004	Malevolence	Malevolence	Mena, Stevan	Nick Groff	tt0389139	
1746516032	1920	1920	Dangerous Days	Dangerous Days	Barker, Reginald	Reginald Barker	tt0011091	
1746513786	1915	1915	Under Southern Skies	Under Southern Skies	Henderson, Lucius	Lucius Henderson	tt0006196	
1746575917	1910	1910	The Converts	The Converts	Griffith, D. W.	D.W. Griffith	tt0001161	
1746577091	1951	1951	Bride of the Gorilla	Bride of the Gorilla	Siodmak, Curt	Curt Siodmak	tt0043360	
1746492455	1948	1948	Give My Regards to Broadway	Give My Regards to Broadway	Bacon, Lloyd	Lloyd Bacon	tt0040390	
1746524953	1914	1914	Washington at Valley Forge	Washington at Valley Forge	Ford, Francis	Francis Ford	tt0004781	
1746583093	1935	1935	Racing Luck	Racing Luck	Newfield, Sam	Sam Newfield	tt0265608	
1746520984	1949	1949	Desert Vigilante	Desert Vigilante	Sears, Fred F.	Fred F. Sears	tt0041291	
1746577379	1950	1950	Wyoming Mail	Wyoming Mail	LeBorg, Reginald	Reginald Le Borg	tt0043149	
1746577539	1936	1936	The Charge of the Light Brigade	The Charge of the Light Brigade	Curtiz, Michael	Michael Curtiz	tt0027438	
1769969944	1912	1912	Shall Never Hunger	Shall Never Hunger			tt0358134	
1746491951	1944	1944	The Hitler Gang	The Hitler Gang	Farrow, John	John Farrow	tt0036921	
1746578696	1960	1960	Psycho	Psycho	Hitchcock, Alfred	Alfred Hitchcock	tt0054215	
1746524756	1914	1914	Rip Van Winkle	Rip Van Winkle			tt0004537	
1746219152	1979	1979	The Main Event	The Main Event	Zieff, Howard	Howard Zieff	tt0079510	
1746577567	1933	1933	Lady for a Day	Lady for a Day	Capra, Frank	Frank Capra	tt0024240	
1746574716	1934	1934	Harlem After Midnight	Harlem After Midnight	Micheaux, Oscar	Oscar Micheaux	tt0139339	
1746475520	1948	1948	Sword of the Avenger	Sword of the Avenger	Salkow, Sidney	Sidney Salkow	tt0040854	
1746575575	1931	1931	Bad Girl	Bad Girl	Borzage, Frank	Frank Borzage	tt0021635	
1746580489	1937	1937	New Faces of 1937	New Faces of 1937	Jason, Leigh	Leigh Jason	tt0029303	
1746583436	1951	1951	Vengeance Valley	Vengeance Valley	Thorpe, Richard	Richard Thorpe	tt0044186	
1746571111	1934	1934	Circus Clown	The Circus Clown	Enright, Ray	Ray Enright	tt0024981	
1769970472	1965	1966	Corruption of the Damned	Corruption of the Damned	Kuchar, George	George Kuchar	tt0215672	
1746568139	1933	1933	The Woman Accused	The Woman Accused	Sloane, Paul	Paul Sloane	tt0024781	
1746567798	1932	1932	Congorilla	Congorilla		Martin E. Johnson	tt0129840	
1746542705	1926	1926	Unknown Dangers	Unknown Dangers	Jones, Grover	Grover Jones	tt0017501	
1746454045	1941	1941	Sleepers West	Sleepers West	Forde, Eugene	Eugene Forde	tt0034199	
1746568216	1952	1952	One Minute to Zero	One Minute to Zero	Garnett, Tay	Tay Garnett	tt0044997	

1746583302	1930	1930	Our Blushing Brides	Our Blushing Brides	Beaumont, Harry	Harry Beaumont	tt0021223
1746562166	1953	1953	I Confess	I Confess	Hitchcock, Alfred	Alfred Hitchcock	tt0045897
1746569600	1955	1955	The Big Combo	The Big Combo	Lewis, Joseph	Joseph H. Lewis	tt0047878
1746513836	1916	1916	The Haunted Manor	The Haunted Manor	Middleton, Edwin	Edwin Middleton	tt0006758
1746570419	1933	1933	The World Changes	The World Changes	Le Roy, Mervyn	Mervyn LeRoy	tt0024786
1746438139	1998	1998	Phoenix	Phoenix	Cannon, Danny	Danny Cannon	tt0119892
1746574466	1933	1932	Devil's Playground	Devil's Playground			tt1270625
1746553496	1963	1963	Cleopatra	Cleopatra	Mankiewicz, Joseph L.	Joseph L. Mankiewicz	tt0056937
1746503544	1919	1919	Getting Mary Married	Getting Mary Married	Dwan, Allan	Allan Dwan	tt0010162
1746419104	1940	1940	The Girl from Avenue A	Girl from Avenue A	Brower, Otto	Otto Brower	tt0032525
1746571656	1910	1910	The Dumb Half Breed's Defence	The Dumb Half Breed's Defense		Gilbert M. 'Broncho Billy' Anderson	tt0001188
1746421332	1997	1997	Liar Liar	Liar Liar	Shadyac, Tom	Tom Shadyac	tt0119528
1746423021	1998	1998	Zero Effect	Zero Effect	Kasdan, Jake	Jake Kasdan	tt0120906
1746566153	1958	1958	The Goddess	The Goddess	Cromwell, John	John Cromwell	tt0051667
1746455013	1996	1996	Jingle All the Way	Jingle All the Way	Levant, Brian	Brian Levant	tt0116705
1745303218	2005	2005	Batman Begins	Batman Begins	Nolan, Christopher	Christopher Nolan	tt0372784
1746437316	1942	1942	Hi, Neighbor	Hi, Neighbor	Lamont, Charles	Charles Lamont	tt0034849
1746496330	1918	1918	Ruggles of Red Gap	Ruggles of Red Gap	Windom, Lawrence C.	Lawrence C. Windom	tt0152242
1746542635	1926	1926	Children of Fate	Children of Fate			tt0321663
1745341318	2001	2001	Donnie Darko	Donnie Darko	Kelly, Richard	Richard Kelly	tt0246578
1746569522	1954	1954	Target Earth	Target Earth	Rose, Sherman A.	Sherman A. Rose	tt0047559
1746584455	1928	1928	The Home Towners	The Home Towners	Foy, Bryan	Bryan Foy	tt0019012
1746393301	1983	1983	Nightmares	Nightmares	Sargent, Joseph	Joseph Sargent	tt0086014
1745359377	2009	2009	Ghosts of Girlfriends Past	Ghosts of Girlfriends Past	Waters, Mark	Mark Waters	tt0821640
1746499855	1946	1946	No Leave, No Love	No Leave, No Love	Martin, Charles	Charles Martin	tt0038779
1746530775	1950	1950	Breakthrough	Breakthrough	Seiler, Lewis	Lewis Seiler	tt0042282
1746507768	1926	1926	Flashing Fangs	Flashing Fangs	McCarthy, Henry	Henry McCarty	tt0132963
1746464786	1940	1940	I Take This Oath	I Take This Oath	Scott, Sherman	Sam Newfield	tt0032618
1746474081	1945	1945	China's Little Devils	China's Little Devils	Bell, Monta	Monta Bell	tt0037594
1746580749	1938	1938	Mr. Doodle Kicks Off	Mr. Doodle Kicks Off	Goodwins, Leslie	Leslie Goodwins	tt0030468
1746439426	1996	1996	The Cable Guy	The Cable Guy	Stiller, Ben	Ben Stiller	tt0115798
1746439154	1942	1942	City of Silent Men	City of Silent Men	Nigh, William	William Nigh	tt0034598
1746500923	1916	1916	The Dividend	The Dividend	Edwards, Walter	Walter Edwards	tt0006596
1745282746	2005	2005	The Ice Harvest	The Ice Harvest	Ramis, Harold	Harold Ramis	tt0400525
1746578040	1935	1935	The Laramie Kid	The Laramie Kid	Webb, Harry S.	Harry S. Webb	tt0026602
1769970453	1967	1967	The Degenerates	The Degenerates	Milligan, Andy	Andy Milligan	tt0216679
1746577579	1938	1938	Spring Madness	Spring Madness	Simon, S. Sylvan	S. Sylvan Simon	tt0030783
1746507761	1925	1925	Peggy of the Secret Service	Peggy of the Secret Service	McGowan, J. P.	J.P. McGowan	tt0016208
1746514267	1922	1922	Boomerang Justice	Boomerang Justice	Sedgwick, Edward	Edward Sedgwick	tt0012962
1746465417	1946	1946	Idea Girl	Idea Girl	Jason, Will	Will Jason	tt0038630
1746545083	1940	1940	Pioneers of the West	Pioneers of the West	Orlebeck, Lester	Lester Orlebeck	tt0032914
1746561994	1955	1955	Night Freight	Night Freight	Yarbrough, Jean	Jean Yarbrough	tt0048422
1746454837	1941	1941	Scotland Yard	Scotland Yard	Foster, Norman	Norman Foster	tt0034158
1746567256	1932	1932	Sherlock Holmes	Sherlock Holmes	Howard, William K.	William K. Howard	tt0023460
1746424575	1942	1942	Ride 'Em Cowboy	Ride 'Em Cowboy	Lubin, Arthur	Arthur Lubin	tt0035252
1746508967	1924	1924	Heart of Alaska	Heart of Alaska	McCracken, Harold	Harold McCracken	tt0014975
1746514114	1916	1916	The Tale of a Coat	The Tale of a Coat	Beaudine, William	William Beaudine	tt0463194
1746500702	1924	1924	The New School Teacher	The New School Teacher	La Cava, Gregory	Gregory La Cava	tt0015171
1746577767	1936	1936	Next Time We Love	Next Time We Love	Griffith, Edward H.	Edward H. Griffith	tt0028029
1746584919	1940	1940	Laughing at Danger	Laughing at Danger	Bretherton, Howard	Howard Bretherton	tt0032691
1746497893	1917	1917	The Natural Law	The Natural Law	France, Charles H.	Charles H. France	tt0008363
1746577224	1963	1963	It's a Mad, Mad, Mad, Mad World	It's a Mad Mad Mad Mad World	Kramer, Stanley	Stanley Kramer	tt0057193
1745302530	2006	2005	Zerophilia	Zerophilia	Curland, Martin	Martin Curland	tt0421090
1746508998	1925	1925	Classified	Classified	Santell, Alfred	Alfred Santell	tt0015688
1746530778	1950	1950	Captive Girl	Captive Girl	Berke, William	William Berke	tt0042311
1746516503	1921	1921	The Rage of Paris	The Rage of Paris	Conway, Jack	Jack Conway	tt0012604
1746474417	1944	1944	Detective Kitty O'Day	Detective Kitty O'Day	Beaudine, William	William Beaudine	tt0207407

1746554694	1964	1964	The Fall of the Roman Empire	The Fall of the Roman Empire	Mann, Anthony	Anthony Mann	tt0058085
1746520985	1949	1949	Manhattan Angel	Manhattan Angel	Dreifuss, Arthur	Arthur Dreifuss	tt0041632
1746497514	1917	1917	The Little Boy Scout	The Little Boy Scout	Grandon, Francis J.	Francis J. Grandon	tt0176936
1745340778	2003	2003	Chasing Papi	Chasing Papi	Mendoza, Linda	Linda Mendoza	tt0323572
1746501867	1916	1916	The Torch Bearer	The Torch Bearer	Prescott, Jack; Russell, William	Jack Prescott	tt0159040
1746523997	1915	1915	All for a Girl	All for a Girl	Applegate, Roy	Roy Applegate	tt0004878
1746508050	1924	1924	Flashing Spurs	Flashing Spurs	Eason, Reeves	B. Reeves Eason	tt0014909
1746508718	1923	1923	Hearts Aflame	Hearts Aflame	Barker, Reginald	Reginald Barker	tt0014121
1746567268	1933	1933	Good Housewrecking	Good Housewrecking	Sweet, Harry	Harry Sweet	tt0024074
1746569917	1959	1959	For the First Time	For the First Time	Maté, Rudolph	Rudolph Maté	tt0052817
1746504575	1920	1920	When Quackel Did Hide	When Quackel Did Hide	Gramlich, Charles	Charlie Joy	tt0203195
1746199844	1968	1968	Finders Keepers, Lovers Weepers	Finders Keepers, Lovers Weepers!	Meyer, Russ	Russ Meyer	tt0062973
1746503265	1917	1917	The Deemster	The Deemster	Hansel, Howell	Howell Hansel	tt0007845
1746210679	1973	1973	Trader Horn	Trader Horn	Badiyi, Reza S.	Reza Badiyi	tt0070823
1746454503	1946	1946	Night Editor	Night Editor	Levin, Henry	Henry Levin	tt0038774
1746235456	1974	1974	That's Entertainment	That's Entertainment!	Haley, Jack	Jack Haley Jr.	tt0072272
1746210949	1970	1970	Which Way to the Front?	Which Way to the Front?	Lewis, Jerry	Jerry Lewis	tt0066564
1746491760	1948	1948	One Touch of Venus	One Touch of Venus	Seiter, William A.	William A. Seiter	tt0040669
1746514676	1916	1916	Not My Sister	Not My Sister	Giblyn, Charles	Charles Giblyn	tt0007132
1746560749	1965	1965	How To Murder Your Wife	How to Murder Your Wife	Quine, Richard	Richard Quine	tt0058212
1745372597	2002	2001	By Hook or By Crook	By Hook or by Crook	Dodge, Harry; Howard, Silas	Harry Dodge	tt0290498
1746508224	1925	1925	The Sporting Chance	The Sporting Chance	Apfel, Oscar	Oscar Apfel	tt0016388
1746569735	1958	1958	I Married a Monster from Outer Space	I Married a Monster from Outer Space	Fowler, Gene	Gene Fowler Jr.	tt0051756
1746433942	1995	1995	Braveheart	Braveheart	Gibson, Mel	Mel Gibson	tt0112573
1746562294	1953	1953	Forbidden	Forbidden	Maté, Rudolph	Rudolph Maté	tt0045780
1746419591	1991	1990	Old Explorers	Old Explorers	Pohlad, William	Bill Pohlad	tt0100287
1746574545	1935	1935	Riding Wild	Riding Wild	Selman, David	David Selman	tt0026931
1746234025	1966	1966	For Pete's Sake!	For Pete's Sake	Collier, James F.	James F. Collier	tt0212947
1769975281	1916	1916	The Vengeance of the Oppressed	Vengeance of the Oppressed	Sloman, Edward	Edward Sloman	tt0453291
1746503359	1919	1919	For Better, for Worse	For Better, for Worse	DeMille, Cecil B.	Cecil B. DeMille	tt0010137
1746503468	1917	1917	Mentioned in Confidence	Mentioned in Confidence	Jones, Edgar	Edgar Jones	tt0178753
1746421968	1997	1997	Flubber	Flubber	Mayfield, Les	Les Mayfield	tt0119137
1746503103	1910	1910	The Man Who Died	The Man Who Died			tt1846776
1745293869	2005	2005	Prime	Prime	Younger, Ben	Ben Younger	tt0387514
1746508869	1925	1925	High and Handsome	High and Handsome	Garson, Harry	Harry Garson	tt0135447
1746513050	1923	1923	The Supreme Test	The Supreme Test	MacNamara, W. P.	W.P. MacNamara	tt0014520
1746570176	1933	1933	The Fighting Cowboy	The Fighting Cowboy	Dixon, Denver	Victor Adamson	tt0132157
1745341611	2002	2002	Bad Company	Bad Company	Schumacher, Joel	Joel Schumacher	tt0280486
1746454222	1942	1942	Rhythm Parade	Rhythm Parade	Bretherton, Howard; Gould, Dave	Howard Bretherton	tt0036309
1746455929	1995	1995	Hackers	Hackers	Softley, Iain	Iain Softley	tt0113243
1746409295	1988	1988	Powaqqatsi	Powaqqatsi	Reggio, Godfrey	Godfrey Reggio	tt0095895
1746520337	1926	1926	The Dixie Merchant	The Dixie Merchant	Borzage, Frank	Frank Borzage	tt0016799
1746571855	1931	1931	Murder by the Clock	Murder by the Clock	Sloman, Edward	Edward Sloman	tt0022173
1746524620	1916	1916	The Lost Bridegroom	The Lost Bridegroom	Kirkwood, James	James Kirkwood	tt0006942
1746202378	1970	1970	The Amazing Transplant	The Amazing Transplant	Silverman, Louis	Doris Wishman	tt0065390
1746492578	1945	1945	Lone Texas Ranger	Lone Texas Ranger	Bennet, Spencer	Spencer Gordon Bennet	tt0210814
1745341753	2010	2010	Tooth Fairy	Tooth Fairy	Lembeck, Michael	Michael Lembeck	tt0808510
1746582902	1938	1938	Keep Smiling	Keep Smiling	Leeds, Herbert I.	Herbert I. Leeds	tt0030316
1746569900	1954	1954	Make Haste to Live	Make Haste to Live	Seiter, William A.	William A. Seiter	tt0047205
1746514112	1915	1914	The Chocolate Soldier	The Chocolate Soldier	Morton, Walter; Stange, Stanislaus	Walter Morton	tt0005091
1746512054	1917	1917	The Climber	The Climber	King, Henry	Henry King	tt0007803
1746500086	1924	1924	The Desert Secret	The Desert Secret	Reel, Frederick	Frederick Reel Jr.	tt0243850
1746520289	1924	1924	Flowing Gold	Flowing Gold	De Grasse, Joseph	Joseph De Grasse	tt0014915
1746502012	1914	1914	Shorty Escapes Marriage	Shorty Escapes Marriage	Stanton, Richard	Richard Stanton	tt0004588
1746569515	1958	1958	The Gun Runners	The Gun Runners	Siegel, Donald	Don Siegel	tt0051687
1746581805	1950	1950	Woman on the Run	Woman on the Run	Foster, Norman	Norman Foster	tt0043142
1746520118	1923	1923	Hollywood	Hollywood	Cruze, James	James Cruze	tt0014137

1746532945	1915	1915	The Martyrs of the Alamo	Martyrs of the Alamo	Cabanne, W. Christy	Christy Cabanne	tt0005719	
1746485175	1918	1918	The Locked Heart	The Locked Heart	King, Henry	Henry King	tt0009313	
1746473708	1944	1944	Law Men	Law Men	Hillyer, Lambert	Lambert Hillyer	tt0037009	
1769969954	1912	1912	The New Member of the Life Saving Crew	The New Member of the Life Saving Crew		Harold M. Shaw	tt0002389	
1746422578	1994	1994	That's Entertainment! III	That's Entertainment! III	Friedgen, Bud; Sheridan, Michael J.	Bud Friedgen	tt0111408	
1746501342	1916	1916	Joan the Woman	Joan the Woman	DeMille, Cecil B.	Cecil B. DeMille	tt0008150	
1746523819	1915	1915	The Concealed Truth	The Concealed Truth	Abramson, Ivan	Ivan Abramson	tt0005129	
1746585031	1940	1940	Waterloo Bridge	Waterloo Bridge	LeRoy, Mervyn	Mervyn LeRoy	tt0033238	
**1746584674**	**1928**	**1928**	**Fangs of Fate**	**Fangs of Fate**	**Smith, Noel Mason**	**Noel M. Smith**	**tt0131384**	1
1746466875	1917	1917	The Price of Pride	The Price of Pride	Knoles, Harley	Harley Knoles	tt0008452	
1746574321	1961	1961	The Gambler Wore a Gun	The Gambler Wore a Gun	Cahn, Edward L.	Edward L. Cahn	tt0054909	
1746503889	1920	1920	The U.P. Trail	The U.P. Trail	Conway, Jack	Jack Conway	tt0011802	
1746523750	1913	1913	Leah Kleschna	Leah Kleschna	Dawley, J. Searle	J. Searle Dawley	tt0003056	
1746577848	1937	1937	Lightnin' Crandall	Lightnin' Crandall	Newfield, Sam	Sam Newfield	tt0029147	
1746422960	1994	1994	There Goes My Baby	There Goes My Baby	Mutrux, Floyd	Floyd Mutrux	tt0108320	
1746578592	1951	1951	Tales of Robin Hood	Tales of Robin Hood	Tinling, James	James Tinling	tt0044104	
1745302958	2005	2005	Grizzly Man	Grizzly Man	Herzog, Werner	Werner Herzog	tt0427312	
1746409443	1987	1987	Over the Top	Over the Top	Golan, Menahem	Menahem Golan	tt0093692	
1746581268	1910	1910	The Last of the Saxons	The Last of the Saxons	Blackton (?), J. Stuart	J. Stuart Blackton	tt0143360	
1746415813	1917	1917	The Little Terror	The Little Terror	Ingram, Rex	Rex Ingram	tt0008199	
1745393834	2009	2009	12 Rounds	12 Rounds	Harlin, Renny	Renny Harlin	tt1160368	
1746578401	1951	1951	My Forbidden Past	My Forbidden Past	Stevenson, Robert	Robert Stevenson	tt0043828	
1746532485	1916	1916	The Open Track	The Open Track	Davis, James	J. Gunnis Davis	tt1534533	
1746578969	1951	1951	Angels in the Outfield	Angels in the Outfield	Brown, Clarence	Clarence Brown	tt0043286	
1746523571	1927	1927	Duty's Reward	Duty's Reward	Bracken, Bertram	Bertram Bracken	tt0017838	
1746578457	1963	1963	The Terror	The Terror	Corman, Roger	Roger Corman	tt0057569	
1746569695	1956	1956	The Bad Seed	The Bad Seed	LeRoy, Mervyn	Mervyn LeRoy	tt0048977	
1746492486	1946	1946	The Hoodlum Saint	The Hoodlum Saint	Taurog, Norman	Norman Taurog	tt0038615	
1746465174	1946	1946	Slightly Scandalous	Slightly Scandalous	Jason, Will	Will Jason	tt0038952	
1746499874	1950	1950	Motor Patrol	Motor Patrol	Newfield, Sam	Sam Newfield	tt0042751	
1746569732	1958	1958	Edge of Fury	Edge of Fury	Gurney, Jr., Robert; Lerner, Irving	Robert J. Gurney Jr.	tt0051572	
1746199181	1968	1968	The Boston Strangler	The Boston Strangler	Fleischer, Richard	Richard Fleischer	tt0062755	
1746569645	1955	1955	Conquest of Space	Conquest of Space	Haskin, Byron	Byron Haskin	tt0047947	
1746491840	1947	1947	Kilroy Was Here	Kilroy Was Here	Karlson, Phil	Phil Karlson	tt0039532	
1746197899	1967	1967	A Taste of Flesh	A Taste of Flesh	Silverman, Louis	Doris Wishman	tt0062346	
1746578387	1951	1951	Close to My Heart	Close to My Heart	Keighley, William	William Keighley	tt0043417	
1746476203	1945	1944	The Suspect	The Suspect	Siodmak, Robert	Robert Siodmak	tt0037330	
1746497282	1918	1918	A Mother's Secret	A Mother's Secret	Gerrard, Douglas	Douglas Gerrard	tt0009395	
1746566223	1957	1957	Under Fire	Under Fire	Clark, James B.	James B. Clark	tt0051129	
1746454734	1998	1998	Hard Rain	Hard Rain	Salomon, Mikael	Mikael Salomon	tt0120696	
1746249784	1975	1974	Beyond the Door	Beyond the Door	Hellman, O. ; Barrett, R.	Ovidio G. Assonitis	tt0071212	
1746570462	1952	1952	The Big Sky	The Big Sky	Hawks, Howard	Howard Hawks	tt0044419	
1745359236	2008	2007	In Seach of a Midnight Kiss	In Search of a Midnight Kiss	Holdridge, Alex	Alex Holdridge	tt0989000	
1746453475	1941	1941	Mountain Moonlight	Mountain Moonlight	Grinde, Nick	Nick Grinde	tt0033921	
1746501993	1916	1916	The Way of the World	Ways of the World	Carleton, Lloyd B.	Carl M. Leviness	tt0433212	
1746422609	1997	1997	Men in Black	Men in Black	Sonnenfeld, Barry	Barry Sonnenfeld	tt0119654	
1746574266	1951	1951	Two Tickets to Broadway	Two Tickets to Broadway	Kern, James V.	James V. Kern	tt0044158	
1746524257	1916	1916	Idols	Idols	Cullison, Webster	Webster Cullison	tt0157787	
1746584308	1936	1936	The Reckless Way	The Reckless Way	Johnson, Raymond K.	Bernard B. Ray	tt0329531	
1746420250	1981	1981	My Dinner with Andre	My Dinner with Andre	Malle, Louis	Louis Malle	tt0082783	
1746434889	1999	1999	The Best Man	The Best Man	Lee, Malcolm D.	Malcolm D. Lee	tt0168501	
1746574287	1951	1951	Criminal Lawyer	Criminal Lawyer	Friedman, Seymour	Seymour Friedman	tt0043433	
1745302409	2007	2006	Boy Culture	Boy Culture	Brocka, Q. Allan	Q. Allan Brocka	tt0433350	
1746408478	1982	1982	Friday the 13th -- Part III	Friday the 13th Part III	Miner, Steve	Steve Miner	tt0083972	
1746393495	1986	1986	Brighton Beach Memoirs	Brighton Beach Memoirs	Saks, Gene	Gene Saks	tt0090774	
1746497304	1920	1920	Cynthia-of-the-Minute	Cynthia of the Minute	Vekroff, Perry	Perry N. Vekroff	tt0011087	
1746561909	1957	1957	The Big Boodle	The Big Boodle	Wilson, Richard	Richard Wilson	tt0050189	

1746584983	1940	1940	Dance, Girl, Dance	Dance, Girl, Dance	Arzner, Dorothy	Dorothy Arzner	tt0032376
1746491864	1945	1945	Lawless Empire	Lawless Empire	Keays, Vernon	Vernon Keays	tt0038686
1769970623	1968	1968	Sex Family Robinson	Sex Family Robinson	Vair, Linda	Linda Vair	tt0257141
1746464966	1942	1941	Suicide Squadron	Suicide Squadron	Hurst, Brian Desmond	Brian Desmond Hurst	tt0033511
1746509013	1925	1925	The Storm Breaker	The Storm Breaker	Sloman, Edward	Edward Sloman	tt0016399
1746500287	1924	1924	The Beauty Prize	The Beauty Prize	Ingraham, Lloyd	Lloyd Ingraham	tt0014704
1746484690	1918	1918	A Society Sensation	A Society Sensation	Powell, Paul	Edmund Mortimer	tt0009629
1746582638	1938	1938	Songs and Saddles	Songs and Saddles	Fraser, Harry	Harry L. Fraser	tt0169253
1746508127	1924	1924	$50,000 Reward	$50,000 Reward	Elfelt, Clifford S.	Clifford S. Elfelt	tt0014639
1746533206	1915	1915	The Love Route	The Love Route	Dwan, Allan	Allan Dwan	tt0005653
1746418631	1940	1940	Phantom Rancher	Phantom Rancher	Fraser, Harry	Harry L. Fraser	tt0032902
1746218764	1973	1973	Electra Glide in Blue	Electra Glide in Blue	Guercio, James William	James William Guercio	tt0070022
1746567252	1933	1933	Second Hand Wife	Second Hand Wife	MacFadden, Hamilton	Hamilton MacFadden	tt0024535
1746437248	1940	1940	Prairie Schooners	Prairie Schooners	Nelson, Sam	Sam Nelson	tt0032940
1746553988	1964	1964	Black Like Me	Black Like Me	Lerner, Carl	Carl Lerner	tt0057889
1746524458	1915	1915	Seven Sisters	The Seven Sisters	Olcott, Sidney	Sidney Olcott	tt0006027
1746581794	1928	1928	Power	Power	Higgin, Howard	Howard Higgin	tt0019288
1746533008	1923	1923	Dangerous Trails	Dangerous Trails	Neitz, Alvin J.	Alan James	tt0013972
1746580933	1959	1959	Surf Crazy	Surf Crazy	Brown, Bruce	Bruce Brown	tt0298170
1746235077	1974	1974	Airport 1975	Airport 1975	Smight, Jack	Jack Smight	tt0071110
1745302747	2005	2005	XXX: State of the Union	xXx: State of the Union	Tamahori, Lee	Lee Tamahori	tt0329774
1746583233	1930	1930	Part Time Wife	Part Time Wife	McCarey, Leo	Leo McCarey	tt0021237
1746513230	1928	1928	Jazzland	Jazzland	Fitzgerald, Dallas M.	Dallas M. Fitzgerald	tt0019042
1746555367	1954	1954	Love Me Madly	Love Me Madly	Kirby, Klaytan W.	Klaytan W. Kirby	tt1003011
1746530760	1949	1949	Night unto Night	Night Unto Night	Siegel, Don	Don Siegel	tt0039660
1745272052	2000	2000	Where the Heart Is	Where the Heart Is	Williams, Matt	Matt Williams	tt0198021
1746581474	1928	1928	Put 'Em Up	Put 'Em Up	Lewis, Edgar	Edgar Lewis	tt0019297
1746583614	1951	1951	Halls of Montezuma	Halls of Montezuma	Milestone, Lewis	Lewis Milestone	tt0042539
1769969496	1911	1911	The Old Curiosity Shop	The Old Curiosity Shop	O`Neil, Barry	Barry O'Neil	tt0330716
1746583522	1951	1951	The Groom Wore Spurs	The Groom Wore Spurs	Whorf, Richard	Richard Whorf	tt0043603
1746464885	1942	1942	Now, Voyager	Now, Voyager	Rapper, Irving	Irving Rapper	tt0035140
1746208977	1967	1967	Shanty Tramp	Shanty Tramp	Prieto, Joseph	Joseph P. Mawra	tt0060968
1746574844	1935	1935	I Found Stella Parish	I Found Stella Parish	LeRoy, Mervyn	Mervyn LeRoy	tt0026508
1746423797	1940	1940	Charlie Chan's Murder Cruise	Charlie Chan's Murder Cruise	Forde, Eugene	Eugene Forde	tt0032326
1746453840	1941	1941	Bahama Passage	Bahama Passage	Griffith, Edward H.	Edward H. Griffith	tt0033372
1746491853	1945	1945	The Bullfighters	The Bullfighters	St. Clair, Mal	Malcolm St. Clair	tt0037563
1746561472	1932	1932	The Girl from Calgary	The Girl from Calgary	Whitman, Phil; d'Usseau, Leon	Phil Whitman	tt0022940
1746568343	1933	1933	The Mad Game	The Mad Game	Cummings, Irving	Irving Cummings	tt0024286
1746503508	1917	1916	Pardners	Partners		Hobart Henley	tt0335342
1746565473	1952	1952	Navajo	Navajo	Foster, Norman	Norman Foster	tt0044955
1746514749	1915	1915	The Unwelcome Wife	The Unwelcome Wife	Abramson, Ivan	Ivan Abramson	tt0006203
1746492717	1947	1947	Escape Me Never	Escape Me Never	Godfrey, Peter	Peter Godfrey	tt0039357
1746520568	1925	1925	The Fate of a Flirt	The Fate of a Flirt	Strayer, Frank R.	Frank R. Strayer	tt0015796
1746392879	1985	1985	Silver Bullet	Silver Bullet	Attias, Daniel	Daniel Attias	tt0090021
1746503636	1918	1918	Wanted for Murder	Wanted for Murder	Crane, Frank	Frank Hall Crane	tt0171911
1746567262	1933	1933	The Big Bluff	The Big Bluff	Denny, Reginald	Reginald Denny	tt0023805
1746573802	1958	1958	Dragstrip Riot	Dragstrip Riot	Bradley, David	David Bradley	tt0051555
1746581853	1951	1951	Journey into Light	Journey Into Light	Heisler, Stuart	Stuart Heisler	tt0043692
1746524780	1916	1916	Life's Whirlpool	Life's Whirlpool	O'Neil, Barry	Barry O'Neil	tt0005733
1745293385	2006	2006	Phat Girlz	Phat Girlz	Likke, Nnegest	Nnegest Likké	tt0490196
1746570355	1934	1934	Wagon Wheels	Wagon Wheels	Barton, Charles	Charles Barton	tt0025954
1745373315	2002	2002	Red Dragon	Red Dragon	Ratner, Brett	Brett Ratner	tt0289765
1746571765	1932	1932	The Purchase Price	The Purchase Price	Wellman, William A.	William A. Wellman	tt0023362
1746474529	1948	1948	Crossed Trails	Crossed Trails	Hillyer, Lambert	Lambert Hillyer	tt0040256
1746486841	1920	1920	Madonnas and Men	Madonnas and Men	Rolfe, B. A.	B.A. Rolfe	tt0196730
1746465405	1942	1942	The War Against Mrs. Hadley	The War Against Mrs. Hadley	Bucquet, Harold S.	Harold S. Bucquet	tt0035531
1746516297	1921	1921	I Am Guilty	I Am Guilty	Nelson, Jack	Jack Nelson	tt0012302

1746569890	1960	1960	Dinosaurus!	Dinosaurus!	Yeaworth, Irvin S.	Irvin S. Yeaworth Jr.	tt0053768	
1746496576	1918	1918	Little Red Riding Hood	Little Red Riding Hood	Thayer, Otis B.	Otis Thayer	tt0186314	
1746419927	1988	1988	Elvira, Mistress of the Dark	Elvira: Mistress of the Dark	Signorelli, James	James Signorelli	tt0095088	
1746562183	1954	1954	Killers from Space	Killers from Space	Wilder, W. Lee	W. Lee Wilder	tt0047149	
1746503317	1918	1918	The Gun Woman	The Gun Woman	Borzage, Frank	Frank Borzage	tt0009137	
1746583816	1930	1930	Clancy in Wall Street	Clancy in Wall Street	Wilde, Ted	Ted Wilde	tt0020770	
1745294039	2006	2006	The Grudge 2	The Grudge 2	Shimizu, Takashi	Takashi Shimizu	tt0433386	
1746185575	1965	1965	All Men Are Apes!	All Men Are Apes!	Mawra, Joseph P.	Joseph P. Mawra	tt0058894	
1746208381	1969	1969	Support Your Local Sheriff!	Support Your Local Sheriff!	Kennedy, Burt	Burt Kennedy	tt0065051	
**1746408718**	**1982**	**1982**	**Forced Vengeance**	**Forced Vengeance**	**Fargo, James**	**James Fargo**	**tt0083960**	1
1746583587	1929	1929	The Sky Skidder	The Sky Skidder	Mitchell, Bruce	Bruce Mitchell	tt0020417	
1746513105	1910	1910	The Merry Wives of Windsor	The Merry Wives of Windsor		Francis Boggs	tt0221390	
1746570139	1934	1934	Hips, Hips, Hooray!	Hips, Hips, Hooray!	Sandrich, Mark	Mark Sandrich	tt0025254	
1746584898	1939	1939	The Kansas Terrors	The Kansas Terrors	Sherman, George	George Sherman	tt0031528	
1746515379	1920	1920	The Cradle of Courage	The Cradle of Courage	Hillyer, Lambert	William S. Hart	tt0011078	
1746492860	1948	1948	The Enchanted Valley	The Enchanted Valley	Tansey, Robert Emmett	Robert Emmett Tansey	tt0040320	
1746533219	1915	1915	The Miracle of Life	The Miracle of Life	Pollard, Harry	Harry A. Pollard	tt0005750	
1746483878	1919	1919	Out of the Shadow	Out of the Shadow	Chautard, Emile	Emile Chautard	tt0010543	
1746565941	1953	1953	Gentlemen Prefer Blondes	Gentlemen Prefer Blondes	Hawks, Howard	Howard Hawks	tt0045810	
1746583384	1931	1931	Kick In	Kick In	Wallace, Richard	Richard Wallace	tt0022022	
1746585106	1930	1930	Street of Chance	Street of Chance	Cromwell, John	John Cromwell	tt0021420	
1746503983	1921	1921	Everyman's Price	Everyman's Price	King, Burton	Burton L. King	tt0130670	
1745342068	2008	2008	Beverly Hills Chihuahua	Beverly Hills Chihuahua	Gosnell, Raja	Raja Gosnell	tt1014775	
1746570232	1933	1933	Straightaway	Straightaway	Brower, Otto	Otto Brower	tt0025837	
1746467346	1917	1917	The Accomplice	The Accomplice	Dean, Ralph	Ralph Dean	tt0007611	
1746553883	1965	1965	Brainstorm	Brainstorm	Conrad, William	William Conrad	tt0058990	
1746576851	1932	1932	Movie Crazy	Movie Crazy	Bruckman, Clyde	Clyde Bruckman	tt0023241	
1746501273	1914	1914	The Typhoon	The Typhoon	Barker, Reginald	Reginald Barker	tt0004740	
1746520546	1926	1926	The Bonanza Buckaroo	The Bonanza Buckaroo	Thorpe, Richard	Richard Thorpe	tt0016671	
1746419793	1994	1994	Cobb	Cobb	Shelton, Ron	Ron Shelton	tt0109450	
1746560676	1932	1932	Merrily We Go to Hell	Merrily We Go to Hell	Arzner, Dorothy	Dorothy Arzner	tt0023213	
1746491849	1945	1945	Frontier Gal	Frontier Gal	Lamont, Charles	Charles Lamont	tt0037721	
1746474403	1945	1945	The Phantom of 42nd Street	The Phantom of 42nd Street	Herman, Albert	Albert Herman	tt0037985	
1746580912	1938	1938	Rolling Caravans	Rolling Caravans	Levering, Joseph	Joseph Levering	tt0030690	
1746516319	1921	1921	Hold Your Horses	Hold Your Horses	Hopper, E. Mason	E. Mason Hopper	tt0012287	
1746581997	1951	1951	Tarzan's Peril	Tarzan's Peril	Haskin, Byron	Byron Haskin	tt0043695	
1746475498	1947	1947	Angel and the Badman	Angel and the Badman	Grant, James Edward	James Edward Grant	tt0039152	
1746496811	1919	1919	Virtuous Sinners	Virtuous Sinners	Flynn, Emmett J.	Emmett J. Flynn	tt0010847	
1746515412	1922	1922	Ashes	Ashes	Anderson, G. M.	Gilbert M. 'Broncho Billy' Anderson	tt0012907	
1746492567	1947	1947	The Crime Doctor's Gamble	The Crime Doctor's Gamble	Castle, William	William Castle	tt0039283	
1745358071	2009	2009	The Boys: The Sherman Brothers' Story	The Boys: The Sherman Brothers' Story	Sherman, Jeffrey C. ; Sherman, Grego	Gregory V. Sherman	tt1015971	
1746574398	1958	1958	Houseboat	Houseboat	Shavelson, Melville	Melville Shavelson	tt0051745	
1746234103	1968	1968	The Split	The Split	Flemyng, Gordon	Gordon Flemyng	tt0063636	
1746577706	1937	1937	The Good Old Soak	Good Old Soak	Ruben, J. Walter	J. Walter Ruben	tt0028946	
1769977809	1985	1984	George Stevens: A Filmmaker's Journey	George Stevens: A Filmmaker's Journey	Stevens, Jr., George	George Stevens Jr.	tt0087322	
1746555252	1956	1956	Westward Ho the Wagons!	Westward Ho, the Wagons!	Beaudine, William	William Beaudine	tt0049945	
1746500638	1926	1926	The Terror	The Terror	Smith, Clifford S.	Clifford Smith	tt0017452	
1746572546	1910	1910	A Discontented Woman	A Discontented Woman		Harry Solter	tt0361513	
1746409975	1987	1987	Dragnet	Dragnet	Mankiewicz, Tom	Tom Mankiewicz	tt0092925	
1746507722	1949	1948	Portrait of Jennie	Portrait of Jennie	Dieterle, William	William Dieterle	tt0040705	
1746584171	1934	1934	One Exciting Adventure	One Exciting Adventure	Frank, Ernst L.	Ernst L. Frank	tt0025597	
1746566308	1958	1958	Cop Hater	Cop Hater	Berke, William	William Berke	tt0051489	
1769970492	1965	1965	The Dirty Girls	The Dirty Girls	Metzger, Radley H.	Radley Metzger	tt0062895	
1746524421	1923	1923	The Drums of Jeopardy	The Drums of Jeopardy	Dillon, Edward	Edward Dillon	tt0014007	
1746208150	1966	1966	Cinerama's Russian Adventure	Cinerama's Russian Adventure	Kristi, Leonid; Karmen, Roman; Dolir	Boris Dolin	tt0219592	
1746454207	1942	1942	The Man in the Trunk	The Man in the Trunk	St. Clair, Malcolm	Malcolm St. Clair	tt0035030	
1746498296	1922	1922	Sherlock Brown	Sherlock Brown	Veiller, Bayard	Bayard Veiller	tt0013596	

1745309210	2007	2007	I'm Not There	I'm Not There.	Haynes, Todd	Todd Haynes	tt0368794
1769970833	1915	1915	The Chinatown Mystery	The Chinatown Mystery	Barker, Reginald	Reginald Barker	tt0362504
1746532827	1915	1915	The Last Concert	The Last Concert	Glickman, Ellis F.	Ellis F. Glickman	tt0005605
1746582764	1937	1937	Paradise Isle	Paradise Isle	Collins, Arthur Greville	Arthur Greville Collins	tt0029374
1746435136	1942	1942	Give Out, Sisters	Give Out, Sisters	Cline, Edward F.	Edward F. Cline	tt0034797
1746514825	1927	1927	California	California	Van Dyke, W. S.	W.S. Van Dyke	tt0017727
1746580630	1938	1938	Female Fugitive	Female Fugitive	Nigh, William	William Nigh	tt0030126
1746454669	1943	1943	White Savage	White Savage	Lubin, Arthur	Arthur Lubin	tt0036534
1746555328	1957	1957	Badlands of Montana	Badlands of Montana	Ullman, Daniel B.	Daniel B. Ullman	tt0050159
1746583259	1929	1929	The Greene Murder Case	The Greene Murder Case	Tuttle, Frank	Frank Tuttle	tt0019949
1746394182	1984	1983	Bless Their Little Hearts	Bless Their Little Hearts	Woodberry, Billy	Billy Woodberry	tt0086977
1746508082	1925	1925	The Fighting Sheriff	The Fighting Sheriff	McGowan, J. P.	J.P. McGowan	tt0015813
1746501130	1916	1916	The Supreme Sacrifice	The Supreme Sacrifice	Knoles, Harley; Belmore, Lionel	Lionel Belmore	tt0007411
1746576903	1932	1932	Play Girl	Play Girl	Enright, Ray	Ray Enright	tt0023342
1746577352	1958	1958	Lust to Kill	A Lust to Kill	Drake, Oliver	Oliver Drake	tt0053026
1746454539	1999	1999	Body Shots	Body Shots	Cristofer, Michael	Michael Cristofer	tt0172627
1745309479	2005	2005	Emmanuel's Gift	Emmanuel's Gift	Lax, Lisa ; Stern, Nancy	Lisa Lax	tt0447016
1746217424	1966	1966	Mister Buddwing	Mister Buddwing	Mann, Delbert	Delbert Mann	tt0059453
1746582350	1951	1951	Cause for Alarm!	Cause for Alarm!	Garnett, Tay	Tay Garnett	tt0043390
1746520580	1927	1927	Twelve Miles Out	Twelve Miles Out	Conway, Jack	Jack Conway	tt0018513
1746502345	1916	1916	Sold for Marriage	Sold for Marriage	Cabanne, William Christy	Christy Cabanne	tt0007368
1746499877	1923	1923	Forgive and Forget	Forgive and Forget	Mitchell, Howard M.	Howard M. Mitchell	tt0014056
1746393635	1987	1987	No Way Out	No Way Out	Donaldson, Roger	Roger Donaldson	tt0093640
1746566848	1933	1933	Police Car 17	Police Car 17	Hillyer, Lambert	Lambert Hillyer	tt0024456
1746582676	1939	1939	They Made Her a Spy	They Made Her a Spy	Hively, Jack	Jack Hively	tt0032021
1746235889	1976	1976	Jackson County Jail	Jackson County Jail	Miller, Michael	Michael Miller	tt0074706
1746524089	1910	1910	More Than His Duty	More Than His Duty	Dawley, J. Searle	J. Searle Dawley	tt1542707
1746584390	1936	1936	My Marriage	My Marriage	Archainbaud, George	George Archainbaud	tt0028011
1746454809	1942	1942	Freckles Comes Home	Freckles Comes Home	Yarbrough, Jean	Jean Yarbrough	tt0034754
1746555669	1955	1955	The Crooked Web	The Crooked Web	Juran, Nathan Hertz	Nathan Juran	tt0047962
1746182876	1910	1910	Baby's First Tooth	Baby's First Tooth		Tom Ricketts	tt1644508
1746563179	1952	1952	Anything Can Happen	Anything Can Happen	Seaton, George	George Seaton	tt0044366
1746555030	1956	1956	The Naked Hills	The Naked Hills	Shaftel, Josef	Josef Shaftel	tt0049540
1746503997	1918	1918	Virtuous Wives	Virtuous Wives	Tucker, George Loane	George Loane Tucker	tt0009765
1746574775	1933	1933	The King's Vacation	The King's Vacation	Adolfi, John G.	John G. Adolfi	tt0024221
1746216959	1966	1966	Fantastic Voyage	Fantastic Voyage	Fleischer, Richard	Richard Fleischer	tt0060397
1746455110	1999	1999	Breakfast of Champions	Breakfast of Champions	Rudolph, Alan	Alan Rudolph	tt0120618
1746464896	1944	1944	Take It or Leave It	Take It or Leave It	Stoloff, Benjamin	Benjamin Stoloff	tt0037341
1746483850	1918	1918	The Mysterious Client	The Mysterious Client	Wright, Fred E.	Fred E. Wright	tt0009411
1746499865	1946	1946	The Bride Wore Boots	The Bride Wore Boots	Pichel, Irving	Irving Pichel	tt0038383
1746503489	1919	1919	True Heart Susie	True Heart Susie	Griffith, D. W.	D.W. Griffith	tt0010806
1746524171	1915	1915	The Chorus Lady	The Chorus Lady	Reicher, Frank	Frank Reicher	tt0005092
1746491882	1947	1947	Riffraff	Riffraff	Tetzlaff, Ted	Ted Tetzlaff	tt0039772
1746436678	1941	1941	Cadet Girl	Cadet Girl	McCarey, Ray	Ray McCarey	tt0033445
1769970035	1911	1911	The Foreman's Courage	The Foreman's Courage		Thomas H. Ince	tt0358484
1746569779	1958	1958	Hong Kong Confidential	Hong Kong Confidential	Cahn, Edward L.	Edward L. Cahn	tt0051736
1746424389	1941	1941	The Lone Rider in Frontier Fury	The Lone Rider in Frontier Fury	Newfield, Sam	Sam Newfield	tt0033845
1746421737	1996	1996	The English Patient	The English Patient	Minghella, Anthony	Anthony Minghella	tt0116209
1746570353	1952	1952	Smoky Canyon	Smoky Canyon	Sears, Fred F.	Fred F. Sears	tt0045159
1745359497	2010	2010	Freakonomics	Freakonomics		Heidi Ewing	tt1152822
1746465075	1945	1945	Both Barrels Blazing	Both Barrels Blazing	Abrahams, Derwin	Derwin Abrahams	tt0036666
1746509328	1950	1950	Champagne for Caesar	Champagne for Caesar	Whorf, Richard B.	Richard Whorf	tt0042325
1745359137	2002	2002	Sorority Boys	Sorority Boys	Wolodarsky, Wallace	Wallace Wolodarsky	tt0279781
1746520509	1926	1925	Mistaken Orders	Mistaken Orders	McGowan, J. P.	J.P. McGowan	tt0017161
1746574450	1933	1933	Saturday's Millions	Saturday's Millions	Sedgwick, Edward	Edward Sedgwick	tt0024523
1746454238	1995	1995	Funny Bones	Funny Bones	Chelsom, Peter	Peter Chelsom	tt0113133
1745302480	2005	2005	The Aristocrats	The Aristocrats	Provenza, Paul	Paul Provenza	tt0436078

1746410379	1986	1986	One Crazy Summer	One Crazy Summer	Holland, Savage Steve	Savage Steve Holland	tt0091680
1746520725	1950	1950	California Passage	California Passage	Kane, Joseph	Joseph Kane	tt0042299
1746580737	1939	1939	Son of Frankenstein	Son of Frankenstein	Lee, Rowland V.	Rowland V. Lee	tt0031951
1746573944	1963	1963	Irma La Douce	Irma la Douce	Wilder, Billy	Billy Wilder	tt0057187
1769976164	1928	1928	Cleopatra	Cleopatra	Neill, R. William	Roy William Neill	tt0428392
1746560863	1932	1932	Trapped in Tia Juana	Trapped in Tia Juana	Fox, Wallace W.	Wallace Fox	tt0283071
1746270344	1978	1978	Harper Valley P. T. A.	Harper Valley P.T.A.	Bennett, Richard	Richard C. Bennett	tt0077660
1746439412	1943	1942	Prelude to War	Prelude to War		Frank Capra	tt0035209
1746495959	1919	1919	His Wife's Friend	His Wife's Friend	De Grasse, Joseph	Joseph De Grasse	tt0190458
1746562112	1953	1953	The Moon Is Blue	The Moon Is Blue	Preminger, Otto	Otto Preminger	tt0046094
1746433727	1991	1991	Point Break	Point Break	Bigelow, Kathryn	Kathryn Bigelow	tt0102685
1746217001	1967	1967	The Caper of the Golden Bulls	The Caper of the Golden Bulls	Rouse, Russell	Russell Rouse	tt0060209
1746503328	1917	1917	Her Own People	Her Own People	Sidney, Scott	Scott Sidney	tt0175725
1746454516	1996	1996	Extreme Measures	Extreme Measures	Apted, Michael	Michael Apted	tt0116259
1746576683	1932	1932	Winner Take All	Winner Take All	Del Ruth, Roy	Roy Del Ruth	tt0023701
1746474974	1943	1943	The Underdog	The Underdog	Nigh, William	William Nigh	tt0036474
1746248146	1966	1966	The Big T.N.T. Show	The Big T.N.T. Show	Peerce, Larry	Larry Peerce	tt0060167
1746437295	1942	1942	Dudes Are Pretty People	Dudes Are Pretty People	Roach, Hal	Hal Roach Jr.	tt0034686
1746569922	1957	1957	Don't Go Near the Water	Don't Go Near the Water	Walters, Charles	Charles Walters	tt0050327
1746424184	1942	1942	Whistling in Dixie	Whistling in Dixie	Simon, S. Sylvan	S. Sylvan Simon	tt0035552
1746455809	1999	1999	Deuce Bigalow: Male Gigolo	Deuce Bigalow: Male Gigolo	Mitchell, Mike	Mike Mitchell	tt0205000
1746571278	1933	1932	Sister to Judas	Sister to Judas	Hopper, E. Mason	E. Mason Hopper	tt0024568
1746453456	1943	1943	Jive Junction	Jive Junction	Ulmer, Edgar G.	Edgar G. Ulmer	tt0034917
1745373515	2004	2004	Soul Plane	Soul Plane	Terrero, Jessy	Jessy Terrero	tt0367085
1746219588	1974	1974	Lenny	Lenny	Fosse, Bob	Bob Fosse	tt0071746
1746465333	1946	1946	Two-Fisted Stranger	Two Fisted Stranger	Nazarro, Ray	Ray Nazarro	tt0039057
1745342024	2004	2004	In Good Company	In Good Company	Weitz, Paul	Paul Weitz	tt0385267
1745293252	2007	2007	Shoot 'Em Up	Shoot 'Em Up	Davis, Michael	Michael Davis	tt0465602
1746475021	1948	1948	The Challenge	The Challenge	Yarbrough, Jean	Jean Yarbrough	tt0040224
1746503331	1919	1919	The Littlest Scout	The Littlest Scout	Blackton, Paula	Paula Blackton	tt0010374
1769974394	1916	1916	The Fate of America	The Fate of America	Ellis, Robert	Robert Ellis	tt3155452
1746424240	1940	1940	My Son, My Son!	My Son, My Son!	Vidor, Charles	Charles Vidor	tt0032831
1745282742	2007	2007	Freedom Writers	Freedom Writers	LaGravenese, Richard	Richard LaGravenese	tt0463998
1746524921	1923	1924	Trail of the Law	The Trail of the Law	Apfel, Oscar	Oscar Apfel	tt0015427
1745293567	2005	2004	A League of Ordinary Gentlemen	A League of Ordinary Gentlemen	Browne, Christopher	Christopher Browne	tt0430289
1746422908	1997	1996	johns	Johns	Silver, Scott	Scott Silver	tt0116714
1746509490	1948	1948	The Treasure of the Sierra Madre	The Treasure of the Sierra Madre	Huston, John	John Huston	tt0040897
1746562081	1957	1957	Designing Woman	Designing Woman	Minnelli, Vincente	Vincente Minnelli	tt0050306
1746575934	1931	1931	Texas Ranger	The Texas Ranger	Lederman, D. Ross	D. Ross Lederman	tt0022473
1745272190	2000	2000	Paragraph 175	Paragraph 175	Epstein, Rob; Friedman, Jeffrey	Rob Epstein	tt0236576
1746433971	1992	1992	Claire of the Moon	Claire of the Moon	Conn, Nicole	Nicole Conn	tt0103977
1746210644	1972	1972	Across 110th Street	Across 110th Street	Shear, Barry	Barry Shear	tt0068168
1746454557	1997	1997	Starship Troopers	Starship Troopers	Verhoeven, Paul	Paul Verhoeven	tt0120201
1746485557	1919	1919	The Cambric Mask	The Cambric Mask	Terriss, Tom	Tom Terriss	tt0009981
1746514689	1915	1915	The Mummy and the Humming Bird	The Mummy and the Humming Bird	Durkin, James	James Durkin	tt0005786
1746579058	1959	1959	Thunder in the Sun	Thunder in the Sun	Rouse, Russell	Russell Rouse	tt0053359
1746570426	1933	1933	Don't Bet on Love	Don't Bet on Love	Roth, Murray	Murray Roth	tt0023957
1746509180	1926	1926	Dancing Mothers	Dancing Mothers	Brenon, Herbert	Herbert Brenon	tt0016762
1746580725	1937	1937	Telephone Operator	Telephone Operator	Pembroke, Scott	Scott Pembroke	tt0030839
1746520112	1924	1924	The Story Without a Name	The Story Without a Name	Willat, Irvin	Irvin Willat	tt0015371
1746515846	1922	1922	The Man from Beyond	The Man from Beyond	King, Burton	Burton L. King	tt0013367
1769970574	1968	1968	For Single Swingers Only	For Single Swingers Only	Davis, Don	Donald A. Davis	tt0137552
1746438942	1945	1945	Christmas in Connecticut	Christmas in Connecticut	Godfrey, Peter	Peter Godfrey	tt0037595
1746580850	1938	1938	The Adventures of Robin Hood	The Adventures of Robin Hood	Curtiz, Michael; Keighley, William	Michael Curtiz	tt0029843
1746421659	1993	1993	For Love or Money	For Love or Money	Sonnenfeld, Barry	Barry Sonnenfeld	tt0106941
1746508370	1924	1924	Welcome Stranger	Welcome Stranger	Young, James	James Young	tt0015476
1746577805	1934	1934	Fugitive Road	Fugitive Road	Strayer, Frank	Frank R. Strayer	tt0025152

1746434302	1995	1995	Get Shorty	Get Shorty	Sonnenfeld, Barry	Barry Sonnenfeld	tt0113161
1746500359	1925	1925	Don Q, Son of Zorro	Don Q Son of Zorro	Crisp, Donald	Donald Crisp	tt0015758
1769970089	1912	1912	John Sterling, Alderman	John Sterling, Alderman		James Kirkwood	tt0327994
1746421273	1940	1940	Wagons Westward	Wagons Westward	Landers, Lew	Lew Landers	tt0033235
1746569566	1955	1954	Destry	Destry	Marshall, George	George Marshall	tt0046906
1746577501	1935	1935	Bordertown	Bordertown	Mayo, Archie	Archie Mayo	tt0026129
1746581643	1928	1928	Secrets of the Range	Secrets of the Range	Horner, Robert J.	Robert J. Horner	tt0019364
1746574622	1935	1935	Broadway Hostess	Broadway Hostess	McDonald, Frank	Frank McDonald	tt0026143
1746566155	1955	1955	Target Zero	Target Zero	Jones, Harmon	Harmon Jones	tt0048698
1746435288	1998	1998	Gods and Monsters	Gods and Monsters	Condon, Bill	Bill Condon	tt0120684
1746573060	1931	1931	Gentleman's Fate	Gentleman's Fate	LeRoy, Mervyn	Mervyn LeRoy	tt0021906
1746583218	1929	1929	The Isle of Lost Ships	The Isle of Lost Ships	Willat, Irvin	Irvin Willat	tt0020032
1745341239	2008	2008	Hamlet 2	Hamlet 2	Fleming, Andrew	Andrew Fleming	tt1104733
1746582845	1937	1937	Step Lively, Jeeves!	Step Lively, Jeeves!	Forde, Eugene	Eugene Forde	tt0029609
1746566140	1953	1953	Oiltown U.S.A.	Oiltown, U.S.A.	Ross, Dick	Dick Ross	tt0991279
1746520417	1926	1926	Hair Trigger Baxter	Hair Trigger Baxter	Nelson, Jack	Jack Nelson	tt0133830
1746467424	1917	1917	The Mysterious Mr. Tiller	The Mysterious Mr. Tiller	Julian, Rupert	Rupert Julian	tt0008350
1746424448	1943	1943	No Place for a Lady	No Place for a Lady	Hogan, James	James P. Hogan	tt0036207
1746410420	1983	1983	Tough Enough	Tough Enough	Fleischer, Richard O.	Richard Fleischer	tt0086461
1746574919	1933	1932	Women Won't Tell	Women Won't Tell	Thorpe, Richard	Richard Thorpe	tt0024784
1746584736	1928	1928	Across the Plains	Across the Plains	Horner, Robert J.	Robert J. Horner	tt0018617
1746580852	1936	1936	Educating Father	Educating Father	Tinling, James	James Tinling	tt0027567
1746574408	1960	1960	Esther and the King	Esther and the King	Walsh, Raoul	Raoul Walsh	tt0053800
1746419205	1981	1981	Mommie Dearest	Mommie Dearest	Perry, Frank	Frank Perry	tt0082766
1746437924	1941	1941	Tanks a Million	Tanks a Million	Guiol, Fred	Fred Guiol	tt0034264
1746440172	1999	1999	The Muse	The Muse	Brooks, Albert	Albert Brooks	tt0164108
1746524312	1922	1922	Fascination	Fascination	Leonard, Robert Z.	Robert Z. Leonard	tt0013119
1769971174	1969	1969	The Secret Sex Lives of Romeo and Juliet	The Secret Sex Lives of Romeo and Juliet	Stootsberry, A. P.	Peter Perry Jr.	tt0127819
1746420776	1990	1990	Home Alone	Home Alone	Columbus, Chris	Chris Columbus	tt0099785
1746514667	1914	1914	The Lion and the Mouse	The Lion and the Mouse	O'Neil, Barry	Barry O'Neil	tt0004232
1746424035	1940	1940	Dr. Kildare's Crisis	Dr. Kildare's Crisis	Bucquet, Harold S.	Harold S. Bucquet	tt0032415
1746185151	1965	1965	Satan's Bed	Satan's Bed	Smith, Marshall	Michael Findlay	tt0059679
1746580797	1935	1935	The Calling of Dan Matthews	The Calling of Dan Matthews	Rosen, Phil	Phil Rosen	tt0027494
1745357704	2009	2009	Home	The Hole	Haverstick, Mary	Joe Dante	tt1085779
1746475983	1945	1945	Week-End at the Waldorf	Week End at the Waldorf	Leonard, Robert Z.	Robert Z. Leonard	tt0038239
1746466926	1917	1917	The Hunting of the Hawk	The Hunting of the Hawk	Fitzmaurice, George	George Fitzmaurice	tt0008127
1746210613	1971	1971	Johnny Got His Gun	Johnny Got His Gun	Trumbo, Dalton	Dalton Trumbo	tt0067277
1746491964	1945	1946	One Way to Love	One Way to Love	Enright, Ray	Ray Enright	tt0038803
1746580382	1937	1936	The Lash of the Penitentes	Lash of the Penitentes	Carroll, Zelma	Roland Price	tt0029114
1746583426	1929	1929	Evidence	Evidence	Adolfi, John G.	John G. Adolfi	tt0019859
1746487281	1919	1919	Erstwhile Susan	Erstwhile Susan	Robertson, John S.	John S. Robertson	tt0010090
1746501060	1916	1916	The Twinkler	The Twinkler	Sloman, Edward	Edward Sloman	tt0007485
1746578851	1960	1960	Guns of the Timberland	Guns of the Timberland	Webb, Robert D.	Robert D. Webb	tt0053884
1746561958	1957	1957	Men in War	Men in War	Mann, Anthony	Anthony Mann	tt0050699
1745340000	2001	2001	The Majestic	The Majestic	Darabont, Frank	Frank Darabont	tt0268995
1746583139	1934	1934	Great Expectations	Great Expectations	Walker, Stuart	Stuart Walker	tt0025202
1746491838	1948	1948	Bill and Coo	Bill and Coo	Reisner, Dean	Dean Riesner	tt0039188
1746495851	1920	1920	Lady Rose's Daughter	Lady Rose's Daughter	Ford, Hugh	Hugh Ford	tt0011383
1746503672	1919	1919	A Romance of Happy Valley	A Romance of Happy Valley	Griffith, D. W.	D.W. Griffith	tt0009559
1746434026	1992	1992	Big Girls Don't Cry...They Get Even	Big Girls Don't Cry... They Get Even	Silver, Joan Micklin	Joan Micklin Silver	tt0101444
1745342228	2008	2008	Quid Pro Quo	Quid Pro Quo	Brooks, Carlos	Carlos Brooks	tt0414426
1745340239	2004	2004	Primer	Primer	Carruth, Shane	Shane Carruth	tt0390384
1746423345	1996	1996	Scream	Scream	Craven, Wes	Wes Craven	tt0117571
1746554591	1964	1964	The Killers	The Killers	Siegel, Donald	Don Siegel	tt0058262
1746514299	1915	1915	Cora	Coral	Carewe, Edwin	Henry MacRae	tt0005136
1746571253	1934	1934	You Can't Buy Everything	You Can't Buy Everything	Riesner, Charles F.	Charles Reisner	tt0026015
1746577107	1951	1951	The Family Secret	The Family Secret	Levin, Henry	Henry Levin	tt0043521

1746491738	1946	1946	G. I. War Brides	G.I. War Brides	Blair, George	George Blair	tt0038545
1746492868	1946	1946	Whistle Stop	Whistle Stop	Moguy, Leonide	Léonide Moguy	tt0039101
1746581946	1929	1929	Midnight on the Barbary Coast	Midnight on the Barbary Coast	Horner, Robert J.	Robert J. Horner	tt0020170
1746577397	1958	1958	Wink of an Eye	Wink of an Eye	Jones, Winston	Winston Jones	tt0052399
1746582667	1937	1937	Federal Bullets	Federal Bullets	Brown, Karl	Karl Brown	tt0028857
1746410165	1990	1990	The Rookie	The Rookie	Eastwood, Clint	Clint Eastwood	tt0100514
1746512418	1916	1916	The Unborn	The Unborn	Fair, Otis B.	Otis Thayer	tt0159071
1746564827	1952	1952	The Wild Heart	The Wild Heart	Powell, Michael; Pressburger, Emeri	Michael Powell	tt0045328
1746503417	1918	1918	The Clutch of Circumstance	The Clutch of Circumstance	Henry-Houry	Henry Houry	tt0008969
1746520585	1927	1927	The Thirteenth Hour	The Thirteenth Hour	Franklin, Chester	Chester M. Franklin	tt0018480
1745341839	2009	2009	A Perfect Getaway	A Perfect Getaway	Twohy, David	David Twohy	tt0971209
1746182852	1910	1910	Big Medicine	Big Medicine			tt1386592
1746577890	1938	1938	The Affairs of Annabel	The Affairs of Annabel	Stoloff, Ben	Benjamin Stoloff	tt0029845
1746524508	1923	1923	The Virginian	The Virginian	Forman, Tom	Tom Forman	tt0014582
1746566738	1952	1952	Pat and Mike	Pat and Mike	Cukor, George	George Cukor	tt0045012
1746520306	1950	1950	Cargo to Capetown	Cargo to Capetown	McEvoy, Earl	Earl McEvoy	tt0042314
1746582147	1928	1928	The Power of the Press	The Power of the Press	Capra, Frank R.	Frank Capra	tt0019290
1746514583	1915	1915	The Mill on the Floss	The Mill on the Floss	Moore, W. Eugene	Eugene Moore	tt0005745
1746514251	1915	1915	Divorced	Divorced	Warren, Edward	Edward Warren	tt0005202
1746185323	1965	1965	The Very Naked Canvas	The Very Naked Canvas	Jacobsen, Jerome	Jerome Jacobsen	tt0291543
1746582568	1937	1937	Public Wedding	Public Wedding	Grinde, Nick	Nick Grinde	tt0029446
1746567010	1952	1952	Young Man With Ideas	Young Man with Ideas	Leisen, Mitchell	Mitchell Leisen	tt0045347
1746567098	1933	1933	Arizona to Broadway	Arizona to Broadway	Tinling, James	James Tinling	tt0023764
1746476197	1948	1948	A Foreign Affair	A Foreign Affair	Wilder, Billy	Billy Wilder	tt0040367
1746585122	1928	1928	The Desert Bride	The Desert Bride	Lang, Walter	Walter Lang	tt0018822
1746504532	1920	1920	Deep Waters	Deep Waters	Tourneur, Maurice	Maurice Tourneur	tt0011103
1746453648	1940	1940	Stage to Chino	Stage to Chino	Killy, Edward	Edward Killy	tt0033098
1746584032	1938	1938	You Can't Take It with You	You Can't Take It with You	Capra, Frank	Frank Capra	tt0030993
1746394716	1987	1987	Return to Horror High	Return to Horror High	Froehlich, Bill	Bill Froehlich	tt0093854
1746454647	1941	1941	Back Street	Back Street	Stevenson, Robert	Robert Stevenson	tt0033365
1746495852	1917	1917	The Square Deal Man	The Square Deal Man	Hart, William S.	William S. Hart	tt0008623
1746582162	1928	1928	Queen of the Chorus	Queen of the Chorus	Hunt, Charles J.	Charles J. Hunt	tt0019299
1746437982	1942	1942	She's in the Army	She's in the Army	Yarbrough, Jean	Jean Yarbrough	tt0035313
1746515026	1910	1910	Under Western Skies	Under Western Skies	Barker, Reginald	Gilbert M. 'Broncho Billy' Anderson	tt0001434
1746512430	1927	1927	Brass Knuckles	Brass Knuckles	Bacon, Lloyd	Lloyd Bacon	tt0017696
1746474510	1947	1947	Blaze of Noon	Blaze of Noon	Farrow, John	John Farrow	tt0039196
1746555747	1955	1955	Unchained	Unchained	Bartlett, Hall	Hall Bartlett	tt0048762
1746583967	1940	1940	Marines Fly High	The Marines Fly High	Nicholls, George; Stoloff, Ben	George Nichols Jr.	tt0032761
1746437352	1941	1941	Blondie Goes Latin	Blondie Goes Latin	Strayer, Frank R.	Frank R. Strayer	tt0033403
1746583866	1931	1931	The Miracle Woman	The Miracle Woman	Capra, Frank	Frank Capra	tt0022153
1746408865	1988	1988	Hairspray	Hairspray	Waters, John	John Waters	tt0095270
1746578062	1937	1937	You're a Sweetheart	You're a Sweetheart	Butler, David	David Butler	tt0029809
1746580802	1939	1939	Meet Dr. Christian	Meet Dr. Christian	Vorhaus, Bernard	Bernard Vorhaus	tt0031635
1745342830	2004	2004	Riding Giants	Riding Giants	Peralta, Stacy	Stacy Peralta	tt0389326
1746218817	1973	1973	The Crazies	The Crazies	Romero, George A.	George A. Romero	tt0069895
1745342510	2001	2001	Heartbreakers	Heartbreakers	Mirkin, David	David Mirkin	tt0125022
1746570077	1933	1933	Havana Widows	Havana Widows	Enright, Ray	Ray Enright	tt0024092
1746562090	1953	1953	The Homesteaders	The Homesteaders	Collins, Lewis	Lewis D. Collins	tt0179221
1746555476	1957	1957	The Tall Stranger	The Tall Stranger	Carr, Thomas	Thomas Carr	tt0051046
1745282813	2007	2007	The Jane Austen Book Club	The Jane Austen Book Club	Swicord, Robin	Robin Swicord	tt0866437
1746568138	1933	1933	Victims of Persecution	Victims of Persecution	Pollard, Bud	Bud Pollard	tt0156169
1746578540	1962	1962	We'll Bury You!	We'll Bury You!			tt6152518
1769969828	1912	1912	The Call of the Desert	The Call of the Desert			tt0345006
1746585127	1930	1930	Oh Sailor Behave	Oh, Sailor Behave!	Mayo, Archie	Archie Mayo	tt0021200
1746578562	1959	1959	Hey Boy! Hey Girl!	Hey Boy! Hey Girl!	Rich, David Lowell	David Lowell Rich	tt0052887
1746570289	1952	1952	Woman in the Dark	Woman in the Dark	Blair, George	George Blair	tt0045336
1746585419	1939	1939	Hawaiian Nights	Hawaiian Nights	Rogell, Albert S.	Albert S. Rogell	tt0031408

1746217460	1966	1966	Torn Curtain	Torn Curtain	Hitchcock, Alfred	Alfred Hitchcock	tt0061107
1746198081	1970	1970	Equinox	Equinox		Jack Woods	tt0067055
1746435506	1941	1941	Outlaws of Cherokee Trail	Outlaws of Cherokee Trail	Orlebeck, Les	Lester Orlebeck	tt0033988
1746487476	1918	1918	Morgan's Raiders	Morgan's Raiders	Lucas, Wilfred; Meredyth, Bess	Wilfred Lucas	tt0009393
1769971201	1969	1969	Over 18, ... and Ready!	Over 18... and Ready!	Allen, Lloyd	Lloyd Allen	tt0131200
1746572221	1931	1931	The Night Angel	The Night Angel	Goulding, Edmund	Edmund Goulding	tt0022205
1745293993	2007	2006	Ten 'Til Noon	Ten 'til Noon	Storm, Scott	Scott Storm	tt0438486
1769970630	1968	1968	The Procurer	The Procurer	Place, Graham	Graham Place	tt0063461
1746584340	1936	1936	Bulldog Edition	Bulldog Edition	Lamont, Charles	Charles Lamont	tt0027406
1746569498	1954	1954	Dial M for Murder	Dial M for Murder	Hitchcock, Alfred	Alfred Hitchcock	tt0046912
1746475904	1947	1947	Hollywood Barn Dance	Hollywood Barn Dance	Ray, Bernard B.	Bernard B. Ray	tt0177841
1746554061	1964	1964	He Rides Tall	He Rides Tall	Springsteen, R. G.	R.G. Springsteen	tt0058186
1746582927	1937	1937	High Hat	High Hat	Sanforth, Clifford	Clifford Sanforth	tt0028999
1746569945	1960	1960	Sink the Bismarck!	Sink the Bismarck!	Gilbert, Lewis	Lewis Gilbert	tt0054310
1746514339	1916	1916	The Woman in 47	The Woman in 47	Irving, George	George Irving	tt0007579
1746248033	1968	1968	Angelique in Black Leather	Angelique in Black Leather	Bouchet, Angelique	Angélique Bouchet	tt0210508
1746503556	1921	1921	Desperate Trails	Desperate Trails	Ford, Jack	John Ford	tt0012102
1746574626	1937	1937	Sing, Cowboy, Sing	Sing, Cowboy, Sing	Bradbury, R. N.	Robert N. Bradbury	tt0029568
1746509311	1949	1949	Range Justice	Range Justice	Taylor, Ray	Ray Taylor	tt0165440
1746585425	1939	1939	Trigger Smith	Trigger Smith	James, Alan	Alan James	tt0032056
1746512439	1916	1916	The Lords of High Decision	The Lords of High Decision	Harvey, Jack	Jack Harvey	tt0006941
1746512757	1916	1916	The Return of Draw Egan	The Return of Draw Egan	Hart, William S.	William S. Hart	tt0007260
1746496101	1919	1919	Mary Regan	Mary Regan	Weber, Lois	Lois Weber	tt0010443
1746235229	1978	1978	The Wiz	The Wiz	Lumet, Sidney	Sidney Lumet	tt0078504
1746520077	1926	1926	Two Fisted Buckaroo	Two Fisted Buckaroo			tt0174293
1746585076	1930	1930	The Sap From Syracuse	The Sap from Syracuse	Sutherland, A. Edward	A. Edward Sutherland	tt0021334
1745282895	2006	2006	The Santa Clause 3: The Escape Clause	The Santa Clause 3: The Escape Clause	Lembeck, Michael	Michael Lembeck	tt0452681
1746584650	1928	1928	A Certain Young Man	A Certain Young Man	Henley, Hobart	Hobart Henley	tt0018754
1746504090	1919	1919	The Loves of Letty	The Loves of Letty	Lloyd, Frank	Frank Lloyd	tt0010403
1746524464	1923	1923	Brass	Brass	Franklin, Sidney A.	Sidney Franklin	tt0013886
1746435002	1991	1991	Defending Your Life	Defending Your Life	Brooks, Albert	Albert Brooks	tt0101698
1746504757	1921	1922	Beyond the Crossroads	Beyond the Crossroads	Carleton, Lloyd	Lloyd B. Carleton	tt0012936
1746503734	1918	1918	More Trouble	More Trouble	Warde, Ernest C.	Ernest C. Warde	tt0009392
1746454996	1996	1996	Tromeo and Juliet	Tromeo and Juliet	Kaufman, Lloyd	Lloyd Kaufman	tt0114733
1746410725	1990	1990	Postcards from the Edge	Postcards from the Edge	Nichols, Mike	Mike Nichols	tt0100395
1746583014	1935	1934	Sequoia	Sequoia	Franklin, Chester M.; Marin, Edwin; (	Chester M. Franklin	tt0025763
1746577182	1951	1951	Night Riders of Montana	Night Riders of Montana	Brannon, Fred C.	Fred C. Brannon	tt0043853
1746497234	1921	1921	Little Italy	Little Italy	Terwilliger, George	George Terwilliger	tt0012396
1746423456	1940	1940	Covered Wagon Trails	Covered Wagon Trails	Johnson, Raymond K.	Bernard B. Ray	tt0032363
1746455330	1995	1995	Sudden Death	Sudden Death	Hyams, Peter	Peter Hyams	tt0114576
1746234757	1968	1968	Sweet November	Sweet November	Miller, Robert Ellis	Robert Ellis Miller	tt0063661
1746581158	1959	1959	The Wonderful Country	The Wonderful Country	Parrish, Robert	Robert Parrish	tt0053453
1746562355	1957	1957	The Badge of Marshal Brennan	The Badge of Marshal Brennan	Gannaway, Albert C.	Albert C. Gannaway	tt0050158
1746416069	1917	1918	The Law of the North	The Law of the North	George, Burton	Irvin Willat	tt0186292
1746523316	1916	1916	The River of Romance	The River of Romance	Otto, Henry	Henry Otto	tt0007266
1746566046	1954	1954	Sign of the Pagan	Sign of the Pagan	Sirk, Douglas	Douglas Sirk	tt0047490
1746574076	1962	1962	Pressure Point	Pressure Point	Cornfield, Hubert	Hubert Cornfield	tt0056370
1746474140	1944	1944	Up in Mabel's Room	Up in Mabel's Room	Dwan, Allan	Allan Dwan	tt0037421
1746453577	1943	1943	This Is the Army	This Is the Army	Curtiz, Michael	Michael Curtiz	tt0036430
1746501554	1916	1916	Pudd'nhead Wilson	Pudd'nhead Wilson	Reicher, Frank	Frank Reicher	tt0007227
1746436780	1989	1989	The January Man	The January Man	O'Connor, Pat	Pat O'Connor	tt0097613
1746484116	1919	1918	The Heart of Humanity	The Heart of Humanity	Holubar, Allen	Allen Holubar	tt0009145
1746569944	1954	1954	Gog	Gog	Strock, Herbert L.	Herbert L. Strock	tt0047033
1746492200	1944	1944	The Singing Sheriff	The Singing Sheriff	Goodwins, Leslie	Leslie Goodwins	tt0037282
1746524476	1922	1922	The Country Flapper	The Country Flapper	Jones, F. Richard	F. Richard Jones	tt0013027
1746572309	1932	1932	American Madness	American Madness	Capra, Frank R.	Frank Capra	tt0022626
1746508054	1924	1924	Pied Piper Malone	Pied Piper Malone	Green, Alfred E.	Alfred E. Green	tt0015229

1746409444	1988	1988	The Serpent and the Rainbow	The Serpent and the Rainbow	Craven, Wes	Wes Craven	tt0096071
1746439130	1943	1943	The Mantrap	The Mantrap	Sherman, George	George Sherman	tt0036139
1746465218	1946	1946	The Falcon's Alibi	The Falcon's Alibi	McCarey, Ray	Ray McCarey	tt0038519
1746520090	1948	1948	Wallflower	Wallflower	Cordova, Frederick de	Frederick De Cordova	tt0040948
1746523566	1916	1916	The Havoc	The Havoc	Berthelet, Arthur	Arthur Berthelet	tt0006760
1746492483	1944	1944	Sheriff of Las Vegas	Sheriff of Las Vegas	Selander, Lesley	Lesley Selander	tt0037270
1745309402	2007	2007	The Ten	Teeth	Wain, David	Mitchell Lichtenstein	tt0780622
1746210046	1971	1972	Bury Me an Angel	Bury Me an Angel	Peeters, Barbara	Barbara Peeters	tt0066872
1746520752	1950	1950	The Silver Bandit	The Silver Bandit	Clifton, Elmer	Elmer Clifton	tt0180165
1745282607	2007	2007	Transformers	Transformers	Bay, Michael	Michael Bay	tt0418279
1746248747	1976	1976	Mikey and Nicky	Mikey and Nicky	May, Elaine	Elaine May	tt0074901
1746569538	1953	1953	Sabre Jet	Sabre Jet	King, Lewis	Louis King	tt0046262
1746465276	1944	1944	Song of the Range	Songs of the Range	Fox, Wallace	Jack Scholl	tt0360788
1746569643	1953	1953	Wings of the Hawk	Wings of the Hawk	Boetticher, Budd	Budd Boetticher	tt0046552
1746508389	1925	1925	Soft Shoes	Soft Shoes	Ingraham, Lloyd	Lloyd Ingraham	tt0016368
1746561610	1932	1932	The Fighting Champ	The Fighting Champ	McCarthy, John P.	John P. McCarthy	tt0022884
1746394767	1991	1991	Double Impact	Double Impact	Lettich, Sheldon	Sheldon Lettich	tt0101764
1746560716	1932	1932	The Man from Arizona	The Man from Arizona	Fraser, Harry	Harry L. Fraser	tt0023182
1746454625	1944	1944	Allergic to Love	Allergic to Love	Lilley, Edward; Wilson, Warren	Edward C. Lilley	tt0036592
1746584262	1940	1940	Tomboy	Tomboy	McGowan, Robert	Robert F. McGowan	tt0033172
1746208589	1969	1969	Take the Money and Run	Take the Money and Run	Allen, Woody	Woody Allen	tt0065063
1746485871	1921	1921	Orphans of the Storm	Orphans of the Storm	Griffith, D. W.	D.W. Griffith	tt0012532
1746530761	1949	1949	Tell It to the Judge	Tell It to the Judge	Foster, Norman	Norman Foster	tt0041949
1746500004	1924	1924	In Love With Love	In Love with Love	Lee, Rowland V.	Rowland V. Lee	tt0015942
1746257703	1976	1976	Carrie	Carrie	DePalma, Brian	Brian De Palma	tt0074285
1746583997	1936	1935	Thunderbolt	Thunderbolt	Paton, Stuart	Stuart Paton	tt0028379
1746580845	1938	1938	State Police	State Police	Rawlins, John	John Rawlins	tt0030794
1746574531	1936	1936	Girls' Dormitory	Girls' Dormitory	Cummings, Irving	Irving Cummings	tt0027677
1746409755	1986	1986	Sweet Liberty	Sweet Liberty	Alda, Alan	Alan Alda	tt0092035
1746574500	1934	1934	The Fighting Hero	Fighting Hero	Webb, Harry S.	Harry S. Webb	tt0026350
1746473918	1942	1942	Syncopation	Syncopation	Dieterle, William	William Dieterle	tt0035405
1769970666	1968	1968	All the Way Down	All the Way Down	Spencer, Zoltan G.	Zoltan G. Spencer	tt0133357
1746570351	1934	1934	This Man Is Mine	This Man Is Mine	Cromwell, John	John Cromwell	tt0025882
1745309493	2007	2006	I'll Believe You	I'll Believe You	Sullivan, Paul Francis	Paul Francis Sullivan	tt0377061
1746578703	1951	1951	Strictly Dishonorable	Strictly Dishonorable	Frank, Melvin; Panama, Norman	Melvin Frank	tt0044082
1746419778	1983	1983	Curse of the Pink Panther	Curse of the Pink Panther	Edwards, Blake	Blake Edwards	tt0085384
1746584488	1930	1930	Queen High	Queen High	Newmeyer, Fred	Fred C. Newmeyer	tt0021279
1746523714	1916	1916	The Destroyers	The Destroyers	Ince, Ralph W.	Ralph Ince	tt0006584
1746567625	1952	1952	Night Stage to Galveston	Night Stage to Galveston	Archainbaud, George	George Archainbaud	tt0044966
1746420747	1983	1983	Terms of Endearment	Terms of Endearment	Brooks, James L.	James L. Brooks	tt0086425
1746582113	1951	1951	The Big Night	The Big Night	Losey, Joseph	Joseph Losey	tt0043340
1746584841	1930	1930	Sweethearts on Parade	Sweethearts on Parade	Neilan, Marshall	Marshall Neilan	tt0021442
1746574120	1960	1960	Wake Me When It's Over	Wake Me When It's Over	LeRoy, Mervyn	Mervyn LeRoy	tt0054455
1746473879	1942	1942	Westward Ho	Westward Ho!	English, John	John English	tt0035545
1746420327	1940	1940	20 Mule Team	20 Mule Team	Thorpe, Richard	Richard Thorpe	tt0032178
1746235445	1978	1978	The Brink's Job	The Brink's Job	Friedkin, William	William Friedkin	tt0077275
1746474534	1946	1946	The Searching Wind	The Searching Wind	Dieterle, William	William Dieterle	tt0038915
1746584003	1940	1940	The Thief of Bagdad	The Thief of Bagdad	Berger, Ludwig; Powell, Michael; Wh	Ludwig Berger	tt0033152
1746198490	1969	1969	The Comic	The Comic	Reiner, Carl	Carl Reiner	tt0064179
1745373696	2003	2002	Dummy	Dummy	Pritikin, Greg	Greg Pritikin	tt0246592
1746234783	1975	1975	Three Days of the Condor	Three Days of the Condor	Pollack, Sydney	Sydney Pollack	tt0073802
1746580434	1937	1937	Bar-Z Bad Men	Bar Z Bad Men	Newfield, Sam	Sam Newfield	tt0028604
1746509325	1924	1924	No More Women	No More Women	Ingraham, Lloyd	Lloyd Ingraham	tt0015178
1746520082	1927	1927	The Princess on Broadway	The Princess on Broadway	Fitzgerald, Dallas M.	Dallas M. Fitzgerald	tt0018286
1746562288	1956	1956	Please Murder Me	Please Murder Me!	Godfrey, Peter	Peter Godfrey	tt0049621
1746573880	1958	1958	The Hideous Sun Demon	The Hideous Sun Demon	Clarke, Robert	Robert Clarke	tt0052888
1745302306	2005	2005	My Big Fat Independent Movie	My Big Fat Independent Movie	Zlotorynski, Phil	Philip Zlotorynski	tt0385890

1746474799	1946	1946	The March of Crime	The March of Crime			tt5370178	
1769975771	1918	1918	Love and Lunch	Love and Lunch	Seiter, William A.	William A. Seiter	tt1312991	
1746500143	1950	1950	The Jackie Robinson Story	The Jackie Robinson Story	Green, Alfred E.	Alfred E. Green	tt0042609	
1746576937	1963	1963	Sound of Laughter	The Sound of Laughter	O'Shaughnessy, John	John O'Shaughnessy	tt0221565	
1746520954	1950	1950	Farewell to Yesterday	Farewell to Yesterday			tt0338990	
1746574807	1933	1933	King for a Night	King for a Night	Neumann, Kurt	Kurt Neumann	tt0024217	
1746486868	1918	1918	Love's Pay Day	Love's Pay Day	Hopper, E. Mason	E. Mason Hopper	tt0186323	
1746571099	1952	1952	The Iron Mistress	The Iron Mistress	Douglas, Gordon	Gordon Douglas	tt0044753	
1746583882	1929	1928	The River	The River	Borzage, Frank	Frank Borzage	tt0020335	
1746563663	1952	1951	Golden Girl	Golden Girl	Bacon, Lloyd	Lloyd Bacon	tt0043592	
1746487589	1919	1919	Calibre 38	Calibre 38	Lewis, Edgar	Edgar Lewis	tt0188469	
1745359054	2003	2002	Ben & Arthur	Ben & Arthur	Mraovich, Sam	Sam Mraovich	tt0364986	
1746571067	1934	1934	Successful Failure	A Successful Failure	Lubin, Arthur	Arthur Lubin	tt0025845	
1746580243	1938	1938	Vacation from Love	Vacation from Love	Fitzmaurice, Geo.	George Fitzmaurice	tt0030922	
1745372565	2010	2009	Tales from the Script	Tales from the Script	Hanson, Peter	Peter Hanson	tt1045642	
1746272134	1966	1966	The Love Merchant	The Love Merchant	Sarno, Joe	Joseph W. Sarno	tt0059405	
1746453628	1943	1943	One Dangerous Night	One Dangerous Night	Gordon, Michael	Michael Gordon	tt0036237	
1746511807	1922	1922	A Dangerous Game	A Dangerous Game	Baggot, King	King Baggot	tt0013047	
**1745271929**	**2000**	**2000**	**The Yards**	**The Yards**	**Gray, James**	**James Gray**	**tt0138946**	1
1746562194	1953	1953	The Juggler	The Juggler	Dmytryk, Edward	Edward Dmytryk	tt0045941	
1746580649	1938	1938	Invisible Enemy	Invisible Enemy	Auer, John H.	John H. Auer	tt0030276	
1746420039	1994	1994	Star Trek Generations	Star Trek: Generations	Carson, David	David Carson	tt0111280	
1746564933	1952	1952	Lady Possessed	Lady Possessed	Spier, William; Kellino, Roy	Roy Kellino	tt0044817	
1745340088	2002	2002	Spellbound	Spellbound	Blitz, Jeff	Jeffrey Blitz	tt0334405	
1746581921	1930	1930	Ladies Love Brutes	Ladies Love Brutes	Lee, Rowland V.	Rowland V. Lee	tt0021037	
1746583470	1950	1950	When You're Smiling	When You're Smiling	Santley, Joseph	Joseph Santley	tt0043130	
1745373954	2010	2010	Grown Ups	Grown Ups	Dugan, Dennis	Dennis Dugan	tt1375670	
1746580410	1937	1937	You Can't Buy Luck	You Can't Buy Luck	Landers, Lew	Lew Landers	tt0029805	
1746561965	1956	1956	The White Squaw	The White Squaw	Nazarro, Ray	Ray Nazarro	tt0049950	
1746524842	1923	1923	Does It Pay?	Does It Pay?	Horan, Charles	Charles Horan	tt0013993	
1746217368	1968	1968	Journey to Shiloh	Journey to Shiloh	Hale, William	William Hale	tt0063161	
1745282536	2007	2007	Live Free or Die Hard	Live Free or Die Hard	Wiseman, Len	Len Wiseman	tt0337978	
1746454382	1941	1941	Saddlemates	Saddlemates	Orlebeck, Les	Lester Orlebeck	tt0034142	
1746509538	1926	1927	Through Thick and Thin	Through Thick and Thin	Eason, Breezy Reeves; Nelson, Jack	Jack Nelson	tt0018485	
1746493706	1914	1914	Hearts Adrift	Hearts Adrift	Porter, Edwin S.	Edwin S. Porter	tt0004057	
1746474951	1948	1948	The Man from Colorado	The Man from Colorado	Levin, Henry; Vidor, Charles	Henry Levin	tt0041625	
1746508157	1924	1924	Men, Women and Money	Men, Women and Money			tt0330623	
1745293823	2005	2005	Cursed	Cursed	Craven, Wes	Wes Craven	tt0257516	
1746578740	1951	1951	Here Comes the Groom	Here Comes the Groom	Capra, Frank	Frank Capra	tt0043633	
1746580206	1935	1935	O'Shaughnessy's Boy	O'Shaughnessy's Boy	Boleslawski, Richard	Richard Boleslawski	tt0026799	
1746436681	1941	1941	The Wolf Man	The Wolf Man	Waggner, George	George Waggner	tt0034398	
1746248264	1967	1967	A Time for Burning	A Time for Burning	Jersey, William C.; Connell, Barbara	Barbara Connell	tt0062372	
1746508737	1950	1950	The Damned Don't Cry	The Damned Don't Cry	Sherman, Vincent	Vincent Sherman	tt0042376	
1746453553	1942	1942	Code of the Outlaw	Code of the Outlaw	English, John	John English	tt0034604	
1746454248	1995	1994	S. F. W.	S.F.W.	Levy, Jefery	Jefery Levy	tt0111048	
1746508387	1950	1949	Dancing in the Dark	Dancing in the Dark	Reis, Irving	Irving Reis	tt0041277	
1746571469	1931	1931	Mother's Millions	Mother's Millions	Flood, James	James Flood	tt0022370	
1746567738	1952	1952	Flaming Feather	Flaming Feather	Enright, Ray	Ray Enright	tt0043544	
1746249405	1976	1975	The California Reich	The California Reich	Parkes, Walter F.; Critchlow, Keith F.	Keith Critchlow	tt0072757	
1746582878	1935	1935	The Live Wire	The Live Wire	Webb, Harry S.	Harry S. Webb	tt0130085	
1746532745	1915	1915	Blue Grass	Blue Grass	Seay, Charles	Charles M. Seay	tt0004983	
1746512154	1915	1915	Sunday	Sunday	Lederer, George W.	George W. Lederer	tt0006118	
1746560780	1932	1932	Hell's Headquarters	Hell's Headquarters	Stone, Andrew L.	Andrew L. Stone	tt0022989	
1746584175	1934	1934	That's Gratitude	That's Gratitude	Craven, Frank	Frank Craven	tt0025869	
1745309125	2006	2006	For Your Consideration	For Your Consideration	Guest, Christopher	Christopher Guest	tt0470765	
1746583267	1929	1929	In Old California	In Old California	King, Burton	Burton L. King	tt0020019	
1746465408	1946	1946	Strange Journey	Strange Journey	Tinling, James	James Tinling	tt0038987	

1746234004	1967	1967	Hostile Guns	Hostile Guns	Springsteen, R. G.	R.G. Springsteen	tt0061783
1745309438	2006	2006	My Super Ex-Girlfriend	My Super Ex Girlfriend	Reitman, Ivan	Ivan Reitman	tt0465624
1746566055	1953	1953	Niagara	Niagara	Hathaway, Henry	Henry Hathaway	tt0046126
1746199203	1969	1969	Blood of Dracula's Castle	Blood of Dracula's Castle	Adamson, Al	Al Adamson	tt0061412
1745293807	2006	2007	What Love Is	What Love Is	Callahan, Mars	Mars Callahan	tt0439876
1746573675	1932	1932	Drifting Souls	Drifting Souls	King, Louis	Louis King	tt0022841
1746577510	1934	1934	Strictly Dynamite	Strictly Dynamite	Nugent, Elliott	Elliott Nugent	tt0025838
1746581161	1951	1951	The People Against O'Hara	The People Against O'Hara	Sturges, John	John Sturges	tt0043914
1746574077	1959	1959	The Little Savage	The Little Savage	Haskin, Byron	Byron Haskin	tt0053012
1745341204	2002	2002	Brown Sugar	Brown Sugar	Famuyiwa, Rick	Rick Famuyiwa	tt0297037
1746583700	1929	1929	The Pagan	The Pagan	Van Dyke, W. S.	W.S. Van Dyke	tt0020253
1746585339	1929	1929	Making the Grade	Making the Grade	Green, Alfred E.	Alfred E. Green	tt0020132
1746514948	1923	1923	Single Handed	Single Handed	Sedgwick, Edward	Edward Sedgwick	tt0014474
1746561878	1954	1954	Drums Across the River	Drums Across the River	Juran, Nathan	Nathan Juran	tt0046937
1746465210	1946	1946	The Story of Kenneth W. Randall, M.D.	The Story of Kenneth W. Randall, M.D.	Kerbawy, Haford	Haford Kerbawy	tt1013720
1746570417	1934	1934	Sleepers East	Sleepers East	MacKenna, Kenneth	Kenneth MacKenna	tt0025801
1746582798	1938	1938	Daredevil Drivers	The Daredevil Drivers	Eason, B. Reeves	B. Reeves Eason	tt0030041
1746454035	1943	1943	The Saint Meets the Tiger	The Saint Meets the Tiger	Stein, Paul	Paul L. Stein	tt0036324
1746454492	1945	1945	Shady Lady	Shady Lady	Waggner, George	George Waggner	tt0038079
1746574313	1961	1961	Blast of Silence	Blast of Silence	Baron, Allen	Allen Baron	tt0054687
1746577212	1960	1960	Twelve Hours to Kill	Twelve Hours to Kill	Cahn, Edward L.	Edward L. Cahn	tt0053557
1746520157	1925	1925	Three Wise Goofs	Three Wise Goofs	Davis, Jimmy	James D. Davis	tt0348203
1746525120	1919	1919	Sadie Love	Sadie Love	Robertson, John S.	John S. Robertson	tt0010652
1746393014	1981	1981	Student Bodies	Student Bodies	Rose, Mickey	Mickey Rose	tt0083133
1746211078	1972	1972	Pete 'n' Tillie	Pete 'n' Tillie	Ritt, Martin	Martin Ritt	tt0069080
1745374340	2003	2002	Dark Blue	Dark Blue	Shelton, Ron	Ron Shelton	tt0279331
1746514147	1923	1923	Daughters of the Rich	Daughters of the Rich	Gasnier, Louis	Louis J. Gasnier	tt0013976
1746514723	1916	1916	The Love Hermit	The Love Hermit	Prescott, Jack	Jack Prescott	tt0163073
1746571014	1952	1952	Japanese War Bride	Japanese War Bride	Vidor, King	King Vidor	tt0044764
1746571127	1933	1933	The Telegraph Trail	The Telegraph Trail	Wright, Tenny	Tenny Wright	tt0024647
1746520904	1950	1950	High Lonesome	High Lonesome	LeMay, Alan	Alan Le May	tt0042552
1746574845	1934	1934	Kid Millions	Kid Millions	Del Ruth, Roy	Roy Del Ruth	tt0025347
1746509289	1949	1949	The Fountainhead	The Fountainhead	Vidor, King	King Vidor	tt0041386
1746465347	1942	1942	A Gentleman at Heart	A Gentleman at Heart	McCarey, Ray	Ray McCarey	tt0034779
1746509178	1924	1924	Trouping With Ellen	Trouping with Ellen	Hunter, T. Hayes	T. Hayes Hunter	tt0015436
1746523977	1923	1923	Fashion Row	Fashion Row	Leonard, Robert Z.	Robert Z. Leonard	tt0014037
1746421354	1993	1993	The Three Musketeers	The Three Musketeers	Herek, Stephen	Stephen Herek	tt0108333
1746503521	1918	1918	He Comes Up Smiling	He Comes Up Smiling	Dwan, Allan	Allan Dwan	tt0009142
1746582731	1939	1940	Cafe Hostess	Cafe Hostess	Salkow, Sidney	Sidney Salkow	tt0032296
1746581480	1910	1910	Fruit and Flowers	Fruit and Flowers			tt0449265
1746574593	1935	1935	Goin' to Town	Goin' to Town	Hall, Alexander	Alexander Hall	tt0026419
1745359661	2002	2002	Abandon	Abandon	Gaghan, Stephen	Stephen Gaghan	tt0267248
1746568165	1952	1952	High Noon	High Noon	Zinnemann, Fred	Fred Zinnemann	tt0044706
1746272696	1980	1980	Motel Hell	Motel Hell	Connor, Kevin	Kevin Connor	tt0081184
1746453796	1943	1943	Johnny Come Lately	Johnny Come Lately	Howard, William K.	William K. Howard	tt0036057
1746504080	1921	1921	Devil Dog Dawson	Devil Dog Dawson		Karl R. Coolidge	tt0012104
1746553288	1963	1963	A Child Is Waiting	A Child Is Waiting	Cassavetes, John	John Cassavetes	tt0056930
1746251504	1977	1977	Slap Shot	Slap Shot	Hill, George Roy	George Roy Hill	tt0076723
1745271843	2000	2000	102 Dalmatians	102 Dalmatians	Lima, Kevin	Kevin Lima	tt0211181
1746584079	1935	1935	Under the Pampas Moon	Under the Pampas Moon	Tinling, James	James Tinling	tt0027156
1746584500	1929	1929	The Idle Rich	The Idle Rich	de Mille, William C.	William C. de Mille	tt0020014
1746533209	1915	1915	Armstrong's Wife	Armstrong's Wife	Melford, George H.	George Melford	tt0004909
1746500503	1927	1927	The Snarl of Hate	The Snarl of Hate	Smith, Noel Mason	Noel M. Smith	tt0018420
1746512073	1923	1923	The Broken Wing	The Broken Wing	Forman, Tom	Tom Forman	tt0013898
1746524710	1913	1913	Ten Nights in a Barroom	Ten Nights in a Barroom	Beggs, Lee	Lee Beggs	tt0003438
1746578699	1958	1958	Tarzan's Fight for Life	Tarzan's Fight for Life	Humberstone, Bruce	H. Bruce Humberstone	tt0052275
1746492414	1944	1944	The Utah Kid	The Utah Kid	Keays, Vernon	Vernon Keays	tt0037422

1746210170	1970	1970	The Magic Garden of Stanley Sweetheart	The Magic Garden of Stanley Sweetheart	Horn, Leonard	Leonard Horn	tt0066036
1746571083	1935	1935	The Pace That Kills	The Pace That Kills	O'Connor, Wm. A.	William A. O'Connor	tt0028074
1746578057	1935	1935	Strangers All	Strangers All	Vidor, Charles	Charles Vidor	tt0027052
1746583340	1930	1930	Let Us Be Gay	Let Us Be Gay	Leonard, Robert Z.	Robert Z. Leonard	tt0021060
1746475942	1947	1947	My Brother Talks to Horses	My Brother Talks to Horses	Zinnemann, Fred	Fred Zinnemann	tt0038761
1746409371	1984	1984	A Soldier's Story	A Soldier's Story	Jewison, Norman	Norman Jewison	tt0088146
1746584139	1940	1940	Yukon Flight	Yukon Flight	Staub, Ralph	Ralph Staub	tt0033292
1746515603	1919	1919	The Pointing Finger	The Pointing Finger	Kull, Edward; Morrissey, Edward	Edward A. Kull	tt0010575
1746564206	1952	1952	Singin' in the Rain	Singin' in the Rain	Kelly, Gene; Donen, Stanley	Stanley Donen	tt0045152
1746555202	1955	1955	Shock-O-Rama	Shock O Rama	Ibenthinkin, Ruben	Ruben I. Binthinkin	tt0160866
1746580498	1939	1939	Back Door to Heaven	Back Door to Heaven	Howard, William K.	William K. Howard	tt0031068
1746455747	1999	1999	The Omega Code	The Omega Code	Marcarelli, Rob	Robert Marcarelli	tt0203408
1746582163	1951	1951	Love Nest	Love Nest	Newman, Joseph	Joseph M. Newman	tt0043759
1746409115	1988	1988	For Keeps?	For Keeps?	Avildsen, John G.	John G. Avildsen	tt0095169
1746410400	1992	1992	Death Becomes Her	Death Becomes Her	Zemeckis, Robert	Robert Zemeckis	tt0104070
1746578055	1937	1937	Lost Horizon	Lost Horizon	Capra, Frank	Frank Capra	tt0029162
1746583938	1930	1930	Back Pay	Back Pay		William A. Seiter	tt0020661
1746434926	1997	1996	Bandwagon	Bandwagon	Schultz, John	John Schultz	tt0112454
1746516024	1920	1920	Everything But the Truth	Everything But the Truth	Lyons, Eddie; Moran, Lee	Eddie Lyons	tt0011161
1746210911	1970	1970	There Was a Crooked Man ...	There Was a Crooked Man...	Mankiewicz, Joseph L.	Joseph L. Mankiewicz	tt0066448
1746219651	1974	1974	Thieves Like Us	Thieves Like Us	Altman, Robert	Robert Altman	tt0072274
1746574351	1951	1951	Gasoline Alley	Gasoline Alley	Bernds, Edward	Edward Bernds	tt0125761
1746581555	1910	1910	His Yankee Girl	His Yankee Girl			tt0413503
1746555528	1957	1957	3:10 to Yuma	3:10 to Yuma	Daves, Delmer	Delmer Daves	tt0050086
1746580990	1958	1958	Marjorie Morningstar	Marjorie Morningstar	Rapper, Irving	Irving Rapper	tt0051911
1746583191	1930	1930	The Call of the Circus	Call of the Circus	O'Connor, Frank	Frank O'Connor	tt0020722
1746250385	1976	1976	Tunnelvision	Tunnel Vision	Swirnoff, Brad; Israel, Neil	Neal Israel	tt0075357
1746584388	1937	1937	Back in Circulation	Back in Circulation	Enright, Ray	Ray Enright	tt0028599
1746584235	1939	1939	Two Bright Boys	Two Bright Boys	Santley, Joseph	Joseph Santley	tt0032066
1745309533	2005	2005	Bigger Than the Sky	Bigger Than the Sky	Corley, Al	Al Corley	tt0363504
1746271435	1979	1979	Hardcore	Hardcore	Schrader, Paul	Paul Schrader	tt0079271
1746566289	1957	1957	Teenage Doll	Teenage Doll	Corman, Roger	Roger Corman	tt0051062
1746218180	1968	1968	The Lion in Winter	The Lion in Winter	Harvey, Anthony	Anthony Harvey	tt0063227
1746585489	1940	1940	Tom Brown's School Days	Tom Brown's School Days	Stevenson, Robert	Robert Stevenson	tt0033169
1746566922	1933	1933	Voltaire	Voltaire	Adolfi, John G.	John G. Adolfi	tt0024742
1746514565	1916	1916	The Mark of Cain	The Mark of Cain	De Grasse, Joseph	Joseph De Grasse	tt0007039
1746582283	1930	1930	Lawful Larceny	Lawful Larceny	Sherman, Lowell	Lowell Sherman	tt0021056
1746567014	1933	1933	Broadway Bad	Broadway Bad	Lanfield, Sidney	Sidney Lanfield	tt0023846
1746496089	1919	1919	The Wilderness Trail	The Wilderness Trail	Le Saint, Edward J.	Edward LeSaint	tt0010895
1746577661	1933	1933	White Woman	White Woman	Walker, Stuart	Stuart Walker	tt0024771
1746560697	1932	1932	The Mummy	The Mummy	Freund, Karl	Karl Freund	tt0023245
1746555883	1954	1953	Paratrooper	Paratrooper	Young, Terence	Terence Young	tt0046161
1746437402	1940	1940	Covered Wagon Days	Covered Wagon Days	Sherman, George	George Sherman	tt0032362
1746513134	1927	1927	The Coward	The Coward	Raboch, Alfred	Alfred Raboch	tt0017777
1746582153	1929	1929	Painted Faces	Painted Faces	Rogell, Albert	Albert S. Rogell	tt0020255
1746394193	1983	1983	Smokey and the Bandit--Part 3	Smokey and the Bandit Part 3	Lowry, Dick	Dick Lowry	tt0086325
1746500132	1946	1946	Little Miss Big	Little Miss Big	Kenton, Erle C.	Erle C. Kenton	tt0038696
1746573839	1961	1961	The Hustler	The Hustler	Rossen, Robert	Robert Rossen	tt0054997
1745293386	2005	2005	Hostage	Hostage	Siri, Florent	Florent	tt0340163
1746502271	1914	1914	Beating Back	Beating Back	Fleming, Caryl S.	Caryl S. Fleming	tt0003668
1746473654	1946	1945	Saratoga Trunk	Saratoga Trunk	Wood, Sam	Sam Wood	tt0038053
1746581018	1962	1962	Panic in Year Zero!	Panic in Year Zero!	Milland, Ray	Ray Milland	tt0056331
1746577084	1951	1951	Across the Wide Missouri	Across the Wide Missouri	Wellman, William	William A. Wellman	tt0043262
1746454635	1944	1944	The Lady and the Monster	The Lady and the Monster	Sherman, George	George Sherman	tt0036999
1746579068	1963	1963	The Gun Hawk	The Gun Hawk	Ludwig, Edward	Edward Ludwig	tt0057121
1746561720	1932	1932	The Silver Lining	The Silver Lining	Crosland, Alan	Alan Crosland	tt0023474
1745373975	2002	2001	A Beautiful Mind	A Beautiful Mind	Howard, Ron	Ron Howard	tt0268978

1746516293	1921	1921	White Oak	White Oak	Hillyer, Lambert	Lambert Hillyer	tt0012843
1746569736	1954	1954	Désirée	Désirée	Koster, Henry	Henry Koster	tt0046903
1746580913	1935	1935	Werewolf of London	Werewolf of London	Walker, Stuart	Stuart Walker	tt0027194
1746410710	1989	1989	Penn & Teller Get Killed	Penn & Teller Get Killed	Penn, Arthur	Arthur Penn	tt0098073
1746185603	1965	1965	The Prince and the Nature Girl	The Prince and the Nature Girl	Wishman, Doris	Doris Wishman	tt0187420
1746487924	1919	1919	Bullin' the Bullsheviki	Bullin' the Bullsheviki	Donovan, Frank P.	Frank P. Donovan	tt0187766
1746500540	1925	1925	A Woman's Faith	A Woman's Faith	Laemmle, Edward	Edward Laemmle	tt0016549
1745294017	2005	2005	Underclassman	Underclassman	Siega, Marcos	Marcos Siega	tt0373416
1746566293	1958	1957	Paths of Glory	Paths of Glory	Kubrick, Stanley	Stanley Kubrick	tt0050825
1746569884	1960	1959	Vice Raid	Vice Raid	Cahn, Edward L.	Edward L. Cahn	tt0053418
1746520901	1950	1950	Forbidden Jungle	Forbidden Jungle	Tansey, Robert	Robert Emmett Tansey	tt0042473
1746577964	1939	1939	Bachelor Mother	Bachelor Mother	Kanin, Garson	Garson Kanin	tt0031067
1746209002	1966	1966	Nashville Rebel	Nashville Rebel	Sheridan, Jay	Jay Sheridan	tt0060739
1746274113	1980	1980	Gorp	Gorp	Ruben, Joseph	Joseph Ruben	tt0080809
1746583084	1938	1938	Prairie Justice	Prairie Justice	Waggner, George	George Waggner	tt0030619
1746513705	1915	1915	Still Waters	Still Waters	Dawley, J. Searle	J. Searle Dawley	tt0006096
1746454053	1942	1942	Ship Ahoy	Ship Ahoy	Buzzell, Edward	Edward Buzzell	tt0035320
1746577592	1934	1934	Tomorrow's Children	Tomorrow's Children	Wilbur, Crane	Crane Wilbur	tt0220107
1746508485	1924	1924	The Fifth Horseman	The Fifth Horseman		E.M. McMahon	tt0014887
1746581067	1910	1910	The Girl on Triple X	The Girl on Triple X		Gilbert M. 'Broncho Billy' Anderson	tt0001233
1746408440	1987	1987	The Outing	The Outing	Daley, Tom	Tom Daley	tt0091707
1746508115	1925	1925	Billy the Kid	Billy the Kid	McGowan, J. P.	J.P. McGowan	tt2295738
1746520696	1925	1925	Sand Blind	Sand Blind	Jaccard, Jacques	Jacques Jaccard	tt0161957
1746573734	1959	1959	The Immoral Mr. Teas	The Immoral Mr. Teas	Meyer, Russ	Russ Meyer	tt0052920
1746438185	1941	1941	Four Mothers	Four Mothers	Keighley, William	William Keighley	tt0033628
1746503820	1921	1921	Gilded Lies	Gilded Lies	Earle, William P. S.	William P.S. Earle	tt0133798
1746570429	1934	1934	Fifteen Wives	Fifteen Wives	Strayer, Frank R.	Frank R. Strayer	tt0025109
1746423757	2000	2000	Charlie's Angels	Charlie's Angels	McG	McG	tt0160127
1746577422	1959	1959	Invisible Invaders	Invisible Invaders	Cahn, Edward L.	Edward L. Cahn	tt0052929
1745342691	2002	2001	The Man from Elysian Fields	The Man from Elysian Fields	Hickenlooper, George	George Hickenlooper	tt0265307
1746574090	1959	1959	The Jayhawkers!	The Jayhawkers!	Frank, Melvin	Melvin Frank	tt0052941
1746555114	1955	1955	Six Bridges to Cross	Six Bridges to Cross	Pevney, Joseph	Joseph Pevney	tt0048628
1746577029	1961	1961	Town Without Pity	Town Without Pity	Reinhardt, Gottfried	Gottfried Reinhardt	tt0055539
1746498303	1920	1920	Bitter Fruit	Bitter Fruit	Bradley, Will	Will H. Bradley	tt0130506
1746520548	1927	1927	The Tigress	The Tigress	Seitz, George B.	George B. Seitz	tt0018486
1769976185	1920	1920	A Home Spun Hero	A Home Spun Hero	Beaudine [Sr.], William	William Beaudine	tt0248872
1745340785	2003	2003	Holes	Holes	Davis, Andrew	Andrew Davis	tt0311289
1746394681	1988	1988	Poltergeist III	Poltergeist III	Sherman, Gary	Gary Sherman	tt0095889
1746273483	1980	1980	The Elephant Man	The Elephant Man	Lynch, David	David Lynch	tt0080678
1745341773	2002	2002	The First $20 Million Is Always the Hardest	The First $20 Million Is Always the Hardest	Jackson, Mick	Mick Jackson	tt0280674
1746514221	1915	1915	The Call of the Dance	The Call of the Dance	Sargent, George L.	George L. Sargent	tt0005048
1746512582	1916	1916	The Price of Silence	The Price of Silence	De Grasse, Joseph	Joseph De Grasse	tt0007213
1746495690	1917	1917	Treason	Treason	Holubar, Allen	Allen Holubar	tt0008694
1746523555	1916	1916	The Phantom Buccaneer	The Phantom Buccaneer	Haydon, J. Charles	J. Charles Haydon	tt0007178
1746514828	1928	1928	Law of Fear	Law of Fear	Storm, Jerome	Jerome Storm	tt0126997
1746502459	1917	1917	The Hand That Rocks the Cradle	The Hand That Rocks the Cradle	Weber, Lois; Smalley, Phillips	Phillips Smalley	tt0008041
1746532828	1914	1914	The Gamblers	The Gambler	Terwilliger, George W.		tt0366506
1746577389	1962	1962	The Longest Day	The Longest Day		Ken Annakin	tt0056197
1746520902	1949	1949	I Was a Male War Bride	I Was a Male War Bride	Hawks, Howard	Howard Hawks	tt0041498
1746499873	1946	1945	Masquerade in Mexico	Masquerade in Mexico	Leisen, Mitchell	Mitchell Leisen	tt0037903
1746515577	1918	1918	The Girl from Bohemia	The Girl from Bohemia	McGill, Lawrence B.	Lawrence B. McGill	tt0184518
1746235060	1973	1973	The Last American Hero	The Last American Hero	Johnson, Lamont	Lamont Johnson	tt0070287
1746577450	1962	1962	Ride the High Country	Ride the High Country	Peckinpah, Sam	Sam Peckinpah	tt0056412
1746420783	1993	1993	Sister Act 2: Back in the Habit	Sister Act 2: Back in the Habit	Duke, Bill	Bill Duke	tt0108147
1746555295	1956	1956	Meet Me in Las Vegas	Meet Me in Las Vegas	Rowland, Roy	Roy Rowland	tt0049490
1746234827	1978	1978	The Cheap Detective	The Cheap Detective	Moore, Robert	Robert Moore	tt0077321
1746574284	1950	1950	The Old Frontier	The Old Frontier	Ford, Philip	Philip Ford	tt0042801

1746410755	1991	1991	Jungle Fever	Jungle Fever	Lee, Spike	Spike Lee	tt0102175	
1746503249	1918	1918	The Belgian	The Belgian	Olcott, Sidney	Sidney Olcott	tt0182778	
1746524208	1923	1923	The Bishop of the Ozarks	The Bishop of the Ozarks	Fox, Finis	Finis Fox	tt0013873	
1746198518	1969	1968	Where Eagles Dare	Where Eagles Dare	Hutton, Brian G.	Brian G. Hutton	tt0065207	
1769970815	1915	1915	The Birth of Emotion	The Birth of Emotion	Otto, Henry	Henry Otto	tt1284609	
1745342000	2002	2002	Dead Above Ground	Dead Above Ground	Bowman, Chuck	Chuck Bowman	tt0284045	
1746564274	1952	1951	Flame of Araby	Flame of Araby	Lamont, Charles	Charles Lamont	tt0043542	
1746503274	1918	1918	The Marriage Lie	The Marriage Lie	Paton, Stuart	Stuart Paton	tt0009353	
1746524656	1915	1915	The Kindling	Kindling	DeMille, Cecil B.	Cecil B. DeMille	tt0005589	
1746508885	1925	1925	What Fools Men	What Fools Men	Archainbaud, George	George Archainbaud	tt0016513	
1746422064	2000	1999	Judy Berlin	Judy Berlin	Mendelsohn, Eric	Eric Mendelsohn	tt0181618	
**1746484711**	**1918**	**1918**	**The Soul of Buddha**	**The Soul of Buddha**	**Edwards, J. Gordon**	**J. Gordon Edwards**	**tt0009637**	1
1769970759	1915	1915	Light Fingered Syd	Light Fingered Syd	De Grey, Sydney	Sidney De Gray	tt1341209	
1746410859	1993	1992	Fortress	Fortress	Gordon, Stuart	Stuart Gordon	tt0106950	
1746436073	1940	1940	Cowboy from Sundown	The Cowboy from Sundown	Bennett, Spencer Gordon	Spencer Gordon Bennet	tt0032364	
1746580354	1935	1935	Mutiny Ahead	Mutiny Ahead	Atkins, Tommy	Thomas Atkins	tt0026751	
1746198024	1970	1970	Trash	Trash	Morrissey, Paul	Paul Morrissey	tt0066482	
1746585142	1929	1929	Hot for Paris	Hot for Paris	Walsh, Raoul	Raoul Walsh	tt0020001	
1746511769	1927	1927	A Yellow Streak	A Yellow Streak	Wilson, Ben	Ben F. Wilson	tt0018602	
1746424422	1942	1942	Texas Manhunt	Texas Man Hunt	Stewart, Peter	Sam Newfield	tt0035424	
1746566999	1934	1935	Girl in the Case	Girl in the Case	Frenke, Eugene	Eugene Frenke	tt0347078	
1746465041	1945	1945	Let's Go Steady	Let's Go Steady	Lord, Del	Del Lord	tt0037869	
1746515099	1920	1920	The Fighting Kentuckians	The Fighting Kentuckians	Edwards, J. Harrison	J. Harrison Edwards	tt0194883	
1746499896	1948	1948	Fighter Squadron	Fighter Squadron	Walsh, Raoul	Raoul Walsh	tt0040353	
1746578903	1958	1958	The Badlanders	The Badlanders	Daves, Delmer	Delmer Daves	tt0051393	
1746584570	1930	1930	The Green Goddess	The Green Goddess	Green, Alfred	Alfred E. Green	tt0020938	
1746584165	1939	1939	Lure of the Wasteland	Lure of the Wasteland	Fraser, Harry	Harry L. Fraser	tt0031597	
1769969757	1912	1912	A Thrilling Rescue by "Uncle Mun"	A Thrilling Rescue by Uncle Mun	Williams, C. J.	C.J. Williams	tt0234885	
1746570148	1934	1934	The Cat's-Paw	The Cat's Paw	Taylor, Sam	Sam Taylor	tt0024961	
1745357712	2001	2001	Riding in Cars with Boys	Riding in Cars with Boys	Marshall, Penny	Penny Marshall	tt0200027	
1746454690	1942	1942	Little Tokyo, U.S.A.	Little Tokyo, U.S.A.	Brower, Otto	Otto Brower	tt0034983	
1746507729	1926	1926	Forever After	Forever After	Weight, F. Harmon	F. Harmon Weight	tt0016901	
1746520154	1925	1925	Wandering Fires	Wandering Fires	Campbell, Maurice	Maurice Campbell	tt0016501	
1746578468	1950	1950	The Avengers	The Avengers	Auer, John H.	John H. Auer	tt0167779	
1746569957	1960	1960	Can-Can	Can Can	Lang, Walter	Walter Lang	tt0053690	
1746524465	1916	1915	Don Quixote	Don Quixote	Dillon, Edward	Edward Dillon	tt0005209	
1746524613	1916	1916	The Victoria Cross	The Victoria Cross	Le Saint, E. J.	Edward LeSaint	tt0007516	
1746554304	1964	1964	The Curse of the Living Corpse	The Curse of the Living Corpse		Del Tenney	tt0057985	
1746474952	1947	1947	Trailing Danger	Trailing Danger	Hillyer, Lambert	Lambert Hillyer	tt0039910	
1746185694	1968	1967	The Illiac Passion	The Illiac Passion	Markopoulos, Gregory J.	Gregory J. Markopoulos	tt0136261	
1746454245	1942	1942	The Rangers Take Over	The Rangers Take Over	Herman, Albert	Albert Herman	tt0036292	
1746453557	1942	1942	Kings Row	Kings Row	Wood, Sam	Sam Wood	tt0034946	
1746435171	1942	1942	That Other Woman	That Other Woman	McCarey, Ray	Ray McCarey	tt0035426	
1746583576	1931	1931	Son of the Plains	A Son of the Plains	Bradbury, Robert North	Robert N. Bradbury	tt0022413	
1746501947	1916	1916	In the Web of the Grafters	In the Web of the Grafters	MacQuarrie, Murdock; Jefferson, Al V	Al V. Jefferson	tt0157816	
1746577889	1936	1936	Captain January	Captain January	Butler, David	David Butler	tt0027421	
1746491747	1943	1943	The Woman of the Town	The Woman of the Town	Archainbaud, George	George Archainbaud	tt0037470	
1746573791	1958	1958	The Rawhide Trail	The Rawhide Trail	Gordon, Robert	Robert Gordon	tt0052122	
1746582272	1931	1931	Broadminded	Broad Minded	LeRoy, Mervyn	Mervyn LeRoy	tt0021697	
1746567693	1933	1932	The Death Kiss	The Death Kiss	Marin, Edwin L.	Edwin L. Marin	tt0023935	
1746507802	1924	1924	Tainted Money	Tainted Money	MacRae, Henry	Henry MacRae	tt0015385	
1746421898	1940	1940	Queen of the Mob	Queen of the Mob	Hogan, James	James P. Hogan	tt0032958	
1746514557	1923	1923	Skylarking	Skylarking	Del Ruth, Roy	Roy Del Ruth	tt0195269	
1746554995	1964	1964	The T.A.M.I. Show	The T.A.M.I. Show	Binder, Steve	Steve Binder	tt0058631	
1746393133	1982	1982	The Best Little Whorehouse in Texas	The Best Little Whorehouse in Texas	Higgins, Colin	Colin Higgins	tt0083642	
1746508729	1926	1926	The Stolen Ranch	The Stolen Ranch	Wyler, William	William Wyler	tt0017432	
1746465156	1946	1946	Man from Rainbow Valley	Man from Rainbow Valley	Springsteen, Robert	R.G. Springsteen	tt0038724	

1746474530	1947	1947	Queen of the Amazons	Queen of the Amazons	Finney, Edward	Edward Finney	tt0039743
1746524717	1928	1928	The Lookout Girl	The Look Out Girl	Fitzgerald, Dallas M.	Dallas M. Fitzgerald	tt0129210
1746572433	1931	1931	Father's Son	Father's Son	Beaudine, William	William Beaudine	tt0021854
1746561968	1957	1957	The Long Haul	The Long Haul	Hughes, Ken	Ken Hughes	tt0050653
1746582928	1939	1939	Nurse Edith Cavell	Nurse Edith Cavell	Wilcox, Herbert	Herbert Wilcox	tt0031737
1746571552	1932	1932	Ten Minutes to Live	Ten Minutes to Live	Micheaux, Oscar	Oscar Micheaux	tt0139639
1746569713	1956	1956	Crime Against Joe	Crime Against Joe	Sholem, Lee	Lee Sholem	tt0049105
1746577433	1958	1958	Sorcerers' Village	Sorcerers' Village	Davis, Capt. Hassoldt	Hassoldt Davis	tt0052222
1746583169	1940	1940	Take Me Back to Oklahoma	Take Me Back to Oklahoma	Herman, Al	Albert Herman	tt0033132
1746583837	1931	1931	Way Back Home	Way Back Home	Seiter, William	William A. Seiter	tt0022551
1746568134	1933	1933	Before Midnight	Before Midnight	Hillyer, Lambert	Lambert Hillyer	tt0024868
1746583914	1930	1930	A Lady to Love	A Lady to Love	Seastrom, Victor	Victor Sjöström	tt0021044
1746464877	1941	1941	San Antonio Rose	San Antonio Rose	Lamont, Charles	Charles Lamont	tt0034148
1746496849	1919	1919	The Sealed Envelope	The Sealed Envelope	Gerrard, Douglas	Douglas Gerrard	tt0010672
1746410408	1985	1985	Sweet Dreams	Sweet Dreams	Reisz, Karel	Karel Reisz	tt0090110
1746260916	1980	1980	Can't Stop the Music	Can't Stop the Music	Walker, Nancy	Nancy Walker	tt0080492
1746583205	1929	1929	The Invaders	The Invaders	McGowan, J. P.	J.P. McGowan	tt0020029
1746570450	1953	1953	The Knockout Parade	The Knockout Parade	Roberts, Joe	Joe Roberts	tt1173952
1746198028	1965	1965	Those Calloways	Those Calloways	Tokar, Norman	Norman Tokar	tt0059796
1746580555	1938	1938	Speed to Burn	Speed to Burn	Brower, Otto	Otto Brower	tt0030778
1746566205	1954	1954	The Lonely Night	The Lonely Night	Jacoby, Irving	Irving Jacoby	tt0360750
1746434630	1998	1998	Half Baked	Half Baked	Davis, Tamra	Tamra Davis	tt0120693
1746507863	1949	1949	Zamba	Zamba	Berke, William	William Berke	tt0042064
1746581947	1930	1929	Overland Bound	Overland Bound	Maloney, Leo	Leo D. Maloney	tt0020250
1746564814	1953	1953	Clipped Wings	Clipped Wings	Bernds, Edward	Edward Bernds	tt0045633
1746423580	1994	1993	Body Snatchers	Body Snatchers	Ferrara, Abel	Abel Ferrara	tt0106452
1746439149	1940	1940	A Little Bit of Heaven	A Little Bit of Heaven	Marton, Andrew	Andrew Marton	tt0032714
1746465237	1942	1942	Stagecoach Express	Stagecoach Express	Sherman, George	George Sherman	tt0035377
1746507826	1926	1926	The Marriage Clause	The Marriage Clause	Weber, Lois	Lois Weber	tt0017122
1746570996	1934	1934	Bolero	Bolero	Ruggles, Wesley	Wesley Ruggles	tt0024903
1746248533	1967	1967	Tammy and the Millionaire	Tammy and the Millionaire	Miller, Sidney; Stone, Ezra; Goodwin	Leslie Goodwins	tt0062334
1746577368	1959	1959	It Started with a Kiss	It Started with a Kiss	Marshall, George	George Marshall	tt0052934
1746524748	1923	1923	Broken Hearts of Broadway	Broken Hearts of Broadway		Irving Cummings	tt0013896
1746410433	1988	1988	A Cry in the Dark	A Cry in the Dark	Schepisi, Fred	Fred Schepisi	tt0094924
1746578601	1961	1961	Run Across the River	Run Across the River	Chambers, Everett	Everett Chambers	tt0271151
1746454359	1941	1941	Nothing But the Truth	Nothing But the Truth	Nugent, Elliott	Elliott Nugent	tt0033963
1746532986	1915	1915	Her Shattered Idol	Her Shattered Idol	O'Brien, John D.	John B. O'Brien	tt0005477
1746454955	1941	1941	A Yank in the R.A.F.	A Yank in the R.A.F.	King, Henry	Henry King	tt0034405
1745373172	2008	2007	Shotgun Stories	Shotgun Stories	Nichols, Jeff	Jeff Nichols	tt0952682
1746209364	1966	1966	Hallucination Generation	Hallucination Generation	Mann, Edward Andrew	Edward Mann	tt0060488
1746577686	1933	1933	Cheating Blondes	Cheating Blondes	Levering, Joseph	Joseph Levering	tt0023883
1746208196	1970	1969	Scratch Harry	Scratch Harry	Matter, Alex	Alex Matter	tt0064947
1746438148	1943	1943	The Girl from Monterrey	The Girl from Monterrey	Fox, Wallace	Wallace Fox	tt0035943
1746545066	1940	1940	The Lone Wolf Keeps a Date	The Lone Wolf Keeps a Date	Salkow, Sidney	Sidney Salkow	tt0033848
1746578007	1937	1937	Hoosier Schoolboy	Hoosier Schoolboy	Nigh, William	William Nigh	tt0029019
1746419769	1991	1991	Run	Run	Burrowes, Geoff	Geoff Burrowes	tt0102818
1746500000	1948	1948	Madonna of the Desert	Madonna of the Desert	Blair, George	George Blair	tt0040561
1746409042	1989	1989	Trust Me	Trust Me	Houston, Bobby	Robert Houston	tt0098529
1746454386	1942	1942	Woman of the Year	Woman of the Year	Stevens, George	George Stevens	tt0035567
1746580887	1938	1938	Wives Under Suspicion	Wives Under Suspicion	Whale, James	James Whale	tt0030981
1746513827	1916	1916	Dorian's Divorce	Dorian's Divorce	Lund, O. A. C.	O.A.C. Lund	tt0157552
1746560983	1932	1932	Vanity Fair	Vanity Fair	Franklin, Chester M.	Chester M. Franklin	tt0023652
1746574255	1963	1963	Twilight of Honor	Twilight of Honor	Sagal, Boris	Boris Sagal	tt0057609
1746583769	1929	1929	The Clean-Up	The Clean Up	McEveety, Bernard F.	Bernard McEveety	tt0019772
1746235544	1977	1977	Julia	Julia	Zinnemann, Fred	Fred Zinnemann	tt0076245
1746508150	1924	1924	Revelation	Revelation	Baker, George D.	George D. Baker	tt0015272
1746499962	1948	1948	Phantom Valley	Phantom Valley	Nazarro, Ray	Ray Nazarro	tt0040691

1746584245	1935	1935	Danger Trails	Danger Trails	Hill, Bob	Robert F. Hill	tt0026260	
1746436268	2000	1999	Catfish in Black Bean Sauce	Catfish in Black Bean Sauce	Lo, Chi Muoi	Chi Muoi Lo	tt0162903	
1746507856	1926	1926	Then Came the Woman	Then Came the Woman	Hartford, David	David Hartford	tt0017459	
1746524730	1922	1922	Channing of the Northwest	Channing of the Northwest	Ince, Ralph	Ralph Ince	tt0013009	
1746576722	1931	1931	The Secret Witness	The Secret Witness	Freeland, Thornton	Thornton Freeland	tt0022358	
1746580278	1939	1939	The Sun Never Sets	The Sun Never Sets	Lee, Rowland V.	Rowland V. Lee	tt0022448	
1746583455	1931	1931	Gun Smoke	Gun Smoke	Sloman, Edward	Edward Sloman	tt0021935	
1746433728	1996	1996	Feeling Minnesota	Feeling Minnesota	Baigelman, Steven	Steven Baigelman	tt0116289	
1746566365	1953	1953	Abbott and Costello Go to Mars	Abbott and Costello Go to Mars	Lamont, Charles	Charles Lamont	tt0045468	
**1746394227**	**1986**	**1986**	**April Fool's Day**	**April Fool's Day**	**Walton, Fred**	**Fred Walton**	**tt0090655**	1
1746438204	1942	1942	What's Cookin'?	What's Cookin'	Cline, Edward F.	Edward F. Cline	tt0035548	
1746520935	1948	1948	Wake of the Red Witch	Wake of the Red Witch	Ludwig, Edward	Edward Ludwig	tt0040946	
1746578708	1961	1961	Wild in the Country	Wild in the Country	Dunne, Philip	Philip Dunne	tt0055623	
**1746520363**	**1924**	**1924**	**South of the Equator**	**South of the Equator**	**Craft, William James**	**William James Craft**	**tt0165955**	1
1746577219	1958	1957	This Angry Age	This Angry Age	Clément, René	René Clément	tt0051400	
1746583584	1931	1931	Skyline	Skyline	Taylor, Sam	Sam Taylor	tt0022400	
1745341000	2002	2002	We Were Soldiers	We Were Soldiers	Wallace, Randall	Randall Wallace	tt0277434	
1746509484	1927	1927	Paying the Price	Paying the Price	Selman, David	David Selman	tt0018254	
1746582460	1937	1937	Sing and Be Happy	Sing and Be Happy	Tinling, James	James Tinling	tt0029567	
**1746584102**	**1939**	**1939**	**That's Right--You're Wrong**	**That's Right You're Wrong**	**Butler, David**	**David Butler**	**tt0032015**	1
1746560939	1965	1965	Fluffy	Fluffy	Bellamy, Earl	Earl Bellamy	tt0059188	
1746577529	1935	1935	Outlawed Guns	Outlawed Guns	Taylor, Ray	Ray Taylor	tt0026838	
1745358904	2003	2003	The Recruit	The Recruit	Donaldson, Roger	Roger Donaldson	tt0292506	
1746475902	1947	1947	Last of the Redmen	Last of the Redmen	Sherman, George	George Sherman	tt0039554	
1746585194	1928	1928	Tyrant of Red Gulch	Tyrant of Red Gulch	De Lacy, Robert	Robert De Lacey	tt0019505	
1745342853	2004	2004	Meet the Fockers	Meet the Fockers	Roach, Jay	Jay Roach	tt0290002	
1769970651	1968	1968	Ready for Anything!	Ready for Anything!	Darcia	Irvin Berwick	tt0256314	
1746553486	1965	1965	The Great Race	The Great Race	Edwards, Blake	Blake Edwards	tt0059243	
1746433555	1999	1999	Teaching Mrs. Tingle	Teaching Mrs. Tingle	Williamson, Kevin	Kevin Williamson	tt0133046	
1746453470	1942	1942	A Man's World	A Man's World	Barton, Charles	Charles Barton	tt0035032	
1746492473	1947	1947	Juke Joint	Juke Joint	Williams, Spencer	Spencer Williams	tt0039521	
1746574354	1963	1963	The Skydivers	The Skydivers	Francis, Coleman	Coleman Francis	tt0057507	
1746503309	1922	1922	Shadows of the Sea	Shadows of the Sea	Crosland, Alan	Alan Crosland	tt0013593	
1746555832	1955	1955	New Orleans Uncensored	New Orleans Uncensored	Castle, William	William Castle	tt0048420	
1745293332	2006	2006	Larry the Cable Guy: Health Inspector	Larry the Cable Guy: Health Inspector	Cooper, Trent	Trent Cooper	tt0462395	
1746503719	1921	1921	The Burden of Race	The Burden of Race			tt0012015	
1769971899	1969	1969	The Girl From Pussy Cat	The Girl from Pussycat		Smythe David	tt0137708	
1745341330	2009	2009	Hotel for Dogs	Hotel for Dogs	Freudenthal, Thor	Thor Freudenthal	tt0785006	
1746508995	1925	1925	Stampedin' Trouble	Stampedin' Trouble	Sheldon, Forrest	Forrest Sheldon	tt0341005	
1746520400	1926	1926	Into Her Kingdom	Into Her Kingdom	Gade, Svend	Svend Gade	tt0016998	
1746584010	1939	1939	Mr. Moto's Last Warning	Mr. Moto's Last Warning	Foster, Norman	Norman Foster	tt0031678	
1746562199	1953	1953	House of Wax	House of Wax	DeToth, Andre	André De Toth	tt0045888	
1746574188	1956	1956	Tea and Sympathy	Tea and Sympathy	Minnelli, Vincente	Vincente Minnelli	tt0049829	
1746465202	1947	1947	Hi De Ho	Hi De Ho	Binney, Josh	Josh Binney	tt0039460	
1746186046	1968	1968	Where Angels Go ... Trouble Follows!	Where Angels Go Trouble Follows!	Neilson, James	James Neilson	tt0063800	
1769969755	1912	1912	A Queen for a Day	A Queen for a Day		C.J. Williams	tt0228758	
1746454506	1999	1999	The Sixth Sense	The Sixth Sense	Shyamalan, M. Night	M. Night Shyamalan	tt0167404	
1746233531	1966	1966	Intimacy	Intimacy	Stoloff, Victor	Victor Stoloff	tt0060542	
1746520325	1926	1926	Exit Smiling	Exit Smiling	Taylor, Sam	Sam Taylor	tt0016832	
1746573214	1931	1931	Mata Hari	Mata Hari	Fitzmaurice, George	George Fitzmaurice	tt0023196	
1746581009	1959	1959	Journey to the Center of the Earth	Journey to the Center of the Earth	Levin, Henry	Henry Levin	tt0052948	
1746520030	1949	1949	Search for Danger	Search for Danger	Bernhard, Jack	Jack Bernhard	tt0041854	
1746577440	1951	1951	An American in Paris	An American in Paris	Minnelli, Vincente	Vincente Minnelli	tt0043278	
1746583736	1929	1929	Girls Who Dare	Girls Who Dare	Mattison, Frank S.	Frank S. Mattison	tt0133804	
1746502198	1910	1910	Wilful Peggy	Wilful Peggy	Griffith, D. W.	D.W. Griffith	tt0001458	
1746422308	1994	1994	The Santa Clause	The Santa Clause	Pasquin, John	John Pasquin	tt0111070	
1746574108	1961	1961	Twist Around the Clock	Twist Around the Clock	Rudolph, Oscar	Oscar Rudolph	tt0055554	

1746439155	1943	1942	Over My Dead Body	Over My Dead Body	St. Clair, Malcolm	Malcolm St. Clair	tt0035164
1746524606	1927	1927	Your Wife and Mine	Your Wife and Mine	O'Connor, Frank	Frank O'Connor	tt0184062
1746513431	1915	1915	A Submarine Pirate	A Submarine Pirate	Avery, Charles; Chaplin, Sydney	Charles Avery	tt0006115
1746580622	1935	1935	She Gets Her Man	She Gets Her Man	Nigh, William	William Nigh	tt0026985
1746583650	1930	1930	Billy the Kid	Billy the Kid	Vidor, King	King Vidor	tt0020693
1746525010	1914	1914	The Ring and the Man	The Ring and the Man	Powers, Francis	Francis Powers	tt0004535
1746584086	1935	1935	Ginger	Ginger	Seiler, Lewis	Lewis Seiler	tt0026410
1746523701	1928	1928	Life's Crossroads	Life's Crossroads	Lewis, Edgar	Edgar Lewis	tt0019092
1746569789	1957	1957	Zombies of Mora-Tau	Zombies of Mora Tau	Cahn, Edward	Edward L. Cahn	tt0051226
1746520366	1926	1926	The Girl From Montmartre	The Girl from Montmartre	Green, Alfred E.	Alfred E. Green	tt0016924
1746234834	1966	1966	What Did You Do in the War, Daddy?	What Did You Do in the War, Daddy?	Edwards, Blake	Blake Edwards	tt0061176
1746583081	1938	1938	Smashing the Spy Ring	Smashing the Spy Ring	Cabanne, Christy	Christy Cabanne	tt0031938
1746562365	1953	1953	The Last Posse	The Last Posse	Werker, Alfred	Alfred L. Werker	tt0045986
1746410394	1991	1991	Return to the Blue Lagoon	Return to the Blue Lagoon	Graham, William A.	William A. Graham	tt0102782
1746198435	1969	1969	If It's Tuesday, This Must Be Belgium	If It's Tuesday, This Must Be Belgium	Stuart, Mel	Mel Stuart	tt0064471
1746566325	1954	1954	Arrow in the Dust	Arrow in the Dust	Selander, Lesley	Lesley Selander	tt0046722
1746584785	1930	1930	Beyond the Rio Grande	Beyond the Rio Grande	Webb, Harry	Harry S. Webb	tt0020682
1746584901	1939	1939	Sweepstakes Winner	Sweepstakes Winner	McGann, William	William C. McGann	tt0031997
1746508514	1949	1949	The Doolins of Oklahoma	The Doolins of Oklahoma	Douglas, Gordon	Gordon Douglas	tt0041308
1746570979	1933	1933	Ladies They Talk About	Ladies They Talk About	Bretherton, Howard; Keighley, Willi	Howard Bretherton	tt0024238
1746465138	1941	1941	This Woman Is Mine	This Woman Is Mine	Lloyd, Frank	Frank Lloyd	tt0034285
1746436201	2000	1999	The Girl Next Door	The Girl Next Door	Fugate, Christine	Christine Fugate	tt0189553
1746566069	1957	1957	Jungle Heat	Jungle Heat	Koch, Howard W.	Howard W. Koch	tt0050579
1746454074	1944	1944	Together Again	Together Again	Vidor, Charles	Charles Vidor	tt0037384
1745340128	2003	2003	Party Monster	Party Monster	Bailey, Fenton ; Barbato, Randy	Fenton Bailey	tt0320244
1746423304	1990	1990	Edward Scissorhands	Edward Scissorhands	Burton, Tim	Tim Burton	tt0099487
1746583924	1931	1931	The Guilty Generation	The Guilty Generation	Lee, Rowland V.	Rowland V. Lee	tt0021932
1746467822	1917	1917	Married in Name Only	Married in Name Only	Lawrence, Edmund	Edmund Lawrence	tt0008273
1746497854	1919	1919	The Battler	The Battler	Reicher, Frank	Frank Reicher	tt0009907
1746499926	1948	1948	Oklahoma Badlands	Oklahoma Badlands	Canutt, Yakima	Yakima Canutt	tt0040659
1746186073	1967	1967	The Ride to Hangman's Tree	Ride to Hangman's Tree	Rafkin, Alan	Alan Rafkin	tt0062198
1746508759	1949	1949	The Dalton Gang	The Dalton Gang	Beebe, Ford	Ford Beebe	tt0041276
1746555635	1956	1956	Frontier Woman	Frontier Woman	Ormond, Ron	Ron Ormond	tt0048097
1746572974	1932	1932	Life Begins	Life Begins	Flood, James	James Flood	tt0023139
1746513077	1916	1916	Ben Blair	Ben Blair	Taylor, William D.	William Desmond Taylor	tt0006417
1746475989	1947	1947	Dick Tracy's Dilemma	Dick Tracy's Dilemma	Rawlins, John	John Rawlins	tt0039321
1746433685	1992	1992	Sister Act	Sister Act	Ardolino, Emile	Emile Ardolino	tt0105417
1746500706	1924	1924	A Society Scandal	A Society Scandal	Dwan, Allan	Allan Dwan	tt0015349
1746523741	1915	1915	The Marble Heart	The Marble Heart	Lessey, George A.	George Lessey	tt0005713
1746577837	1934	1935	White Lies	White Lies	Bulgakov, Leo	Leo Bulgakov	tt0027209
1746508750	1925	1925	Where Was I?	Where Was I?	Seiter, William A.	William A. Seiter	tt0016519
1746453591	1942	1942	Rock River Renegades	Rock River Renegades	Luby, S. Roy	S. Roy Luby	tt0035263
1746218862	1972	1972	Frogs	Frogs	McCowan, George	George McCowan	tt0068615
1746504099	1919	1919	Open Your Eyes	Open Your Eyes	Hamilton, Gilbert P.	Gilbert P. Hamilton	tt0010532
1746584368	1939	1939	Wings of the Navy	Wings of the Navy	Bacon, Lloyd	Lloyd Bacon	tt0032130
1746199732	1968	1968	Devil in Velvet	The Devil in Velvet	Crane, Larry	Larry Crane	tt0216694
1746512585	1916	1916	Human Driftwood	Human Driftwood	Chautard, Emile	Emile Chautard	tt0006833
1746422163	1992	1992	Lethal Weapon 3	Lethal Weapon 3	Donner, Richard	Richard Donner	tt0104714
1746502816	1916	1916	Shoes	Shoes	Weber, Lois	Lois Weber	tt0007340
1746219618	1973	1973	The Doll Squad	The Doll Squad	Mikels, Ted V.	Ted V. Mikels	tt0069986
1746582682	1938	1938	Hold That Co-Ed	Hold That Co ed	Marshall, George	George Marshall	tt0030239
1746525178	1920	1920	The Inferior Sex	The Inferior Sex	Henabery, Joseph	Joseph Henabery	tt0011332
1769976691	1979	1979	Unidentified Flying Oddball	Unidentified Flying Oddball	Mayberry, Russ	Russ Mayberry	tt0080062
1746465381	1946	1946	Cloak and Dagger	Cloak and Dagger	Lang, Fritz	Fritz Lang	tt0038417
1746571298	1934	1934	The Merry Frinks	The Merry Frinks	Green, Alfred E.	Alfred E. Green	tt0025492
1746578503	1962	1962	To Kill a Mockingbird	To Kill a Mockingbird	Mulligan, Robert	Robert Mulligan	tt0056592
1746509290	1926	1926	Her Big Adventure	Her Big Adventure	Ince, John	John Ince	tt0016956

1746502195	1915	1915	The Spanish Jade	The Spanish Jade	Lucas, Wilfred	Wilfred Lucas	tt0006080
1746562151	1957	1957	Destination 60,000	Destination 60,000	Waggner, George	George Waggner	tt0050309
1769970821	1912	1912	The Reformation of Kid Hogan	The Reformation of Kid Hogan		Lloyd B. Carleton	tt0358072
1746577904	1938	1938	Breaking the Ice	Breaking the Ice	Cline, Edward F.	Edward F. Cline	tt0029945
1746513633	1927	1927	Jaws of Steel	Jaws of Steel	Enright, Ray	Ray Enright	tt0018036
1746248362	1966	1966	Good Times, Wonderful Times	Good Times, Wonderful Times	Rogosin, Lionel	Lionel Rogosin	tt0144017
1746482885	1917	1917	The Rescue	The Rescue	Park, Ida May	Ida May Park	tt0008508
1746511783	1923	1923	To the Ladies	To the Ladies	Cruze, James	James Cruze	tt0014552
1746582599	1938	1938	Jezebel	Jezebel	Wyler, William	William Wyler	tt0030287
1746568339	1934	1934	Wharf Angel	Wharf Angel	Menzies, William Cameron; Somnes,	William Cameron Menzies	tt0025972
1769977772	1988	1988	The Chocolate War	The Chocolate War	Gordon, Keith	Keith Gordon	tt0094869
1746520094	1923	1923	It Happened Out West	It Happened Out West			tt0014159
1746520252	1927	1927	The Night Bride	Night Bride	Hopper, E. Mason	E. Mason Hopper	tt0018201
1745340508	2004	2003	Happy Hour	Happy Hour	Bencivenga, Mike	Mike Bencivenga	tt0282543
1746464950	1944	1944	Secrets of Scotland Yard	Secrets of Scotland Yard	Blair, George	George Blair	tt0037255
1746584515	1930	1929	Sagebrush Politics	Sagebrush Politics	Adamson, Victor	Victor Adamson	tt0021328
1746409763	1989	1989	The Return of Swamp Thing	The Return of Swamp Thing	Wynorski, Jim	Jim Wynorski	tt0098193
1746566268	1956	1956	High Society	High Society	Walters, Charles	Charles Walters	tt0049314
1746197878	1966	1966	The Oscar	The Oscar	Rouse, Russell	Russell Rouse	tt0060801
1746514942	1910	1910	The Master Mechanic	The Master Mechanic			tt1798277
1746496836	1918	1918	Hidden Pearls	The Hidden Pearls	Melford, George H.	George Melford	tt0009173
1745293157	2007	2006	Eye of the Dolphin	Eye of the Dolphin	Sellers, Michael D.	Michael D. Sellers	tt0465407
1746410022	1981	1981	The Burning	The Burning	Maylam, Tony	Tony Maylam	tt0082118
1746576292	1931	1931	Men in Her Life	Men in Her Life	Beaudine, William	William Beaudine	tt0022137
1746569888	1955	1955	Summertime	Summertime	Lean, David	David Lean	tt0048673
1746422918	1994	1994	Greedy	Greedy	Lynn, Jonathan	Jonathan Lynn	tt0109936
1769970383	1932	1932	Artistic Temper	Artistic Temper	Mack, Roy	Roy Mack	tt0022429
1746567954	1932	1932	Fast Companions	Fast Companions	Neumann, Kurt	Kurt Neumann	tt0022881
1746435159	1940	1940	It's a Date	It's a Date	Seiter, William A.	William A. Seiter	tt0032644
1746422021	1993	1993	A Dangerous Woman	A Dangerous Woman	Gyllenhaal, Stephen	Stephen Gyllenhaal	tt0106661
1746434329	1992	1992	Lorenzo's Oil	Lorenzo's Oil	Miller, George	George Miller	tt0104756
1746582437	1936	1936	Rainbow on the River	Rainbow on the River	Neumann, Kurt	Kurt Neumann	tt0028157
1746570849	1934	1934	Good Dame	Good Dame	Gering, Marion	Marion Gering	tt0025192
1746581495	1961	1961	All Hands on Deck	All Hands on Deck	Taurog, Norman	Norman Taurog	tt0054613
1746578046	1938	1938	Little Orphan Annie	Little Orphan Annie	Holmes, Ben	Ben Holmes	tt0030375
1745340183	2003	2003	Anger Management	Anger Management	Segal, Peter	Peter Segal	tt0305224
1746582936	1937	1937	Missing Witnesses	Missing Witnesses	Clemens, William	William Clemens	tt0029253
1746393625	1985	1985	Commando	Commando	Lester, Mark L.	Mark L. Lester	tt0088944
1746574583	1938	1938	Everybody Sing	Everybody Sing	Marin, Edwin L.	Edwin L. Marin	tt0030108
1746422126	1982	1982	Burden of Dreams	Burden of Dreams	Blank, Les	Les Blank	tt0083702
1746565217	1953	1953	Champ for a Day	Champ for a Day	Seiter, William A.	William A. Seiter	tt0045619
1745359252	2004	2004	Around the World in 80 Days	Around the World in 80 Days	Coraci, Frank	Frank Coraci	tt0327437
1745309497	2005	2005	The Brothers Grimm	The Brothers Grimm	Gilliam, Terry	Terry Gilliam	tt0355295
1745372639	2002	2002	Confessions of a Dangerous Mind	Confessions of a Dangerous Mind	Clooney, George	George Clooney	tt0270288
1746485931	1918	1918	The Testing of Mildred Vane	The Testing of Mildred Vane	Lucas, Wilfred	Wilfred Lucas	tt0009687
1746582527	1935	1935	Music Is Magic	Music Is Magic	Marshall, George	George Marshall	tt0026747
1746555157	1954	1954	Siege at Red River	Siege at Red River	Maté, Rudolph	Rudolph Maté	tt0047488
1746454606	1942	1942	Submarine Raider	Submarine Raider	Landers, Lew	Lew Landers	tt0035391
1746582745	1938	1938	The Mad Miss Manton	The Mad Miss Manton	Jason, Leigh	Leigh Jason	tt0030396
1746503488	1920	1920	Smoldering Embers	Smoldering Embers	Keenan, Frank	Frank Keenan	tt0152485
1745272664	2000	2000	Remember the Titans	Remember the Titans	Yakin, Boaz	Boaz Yakin	tt0210945
1746208960	1966	1966	Any Wednesday	Any Wednesday	Miller, Robert Ellis	Robert Ellis Miller	tt0060112
1746475716	1947	1947	The Stranger from Ponca City	The Stranger from Ponca City	Abrahams, Derwin	Derwin Abrahams	tt0240045
1746421142	1993	1993	The Pelican Brief	The Pelican Brief	Pakula, Alan J.	Alan J. Pakula	tt0107798
1746420513	1994	1994	Dumb and Dumber	Dumb and Dumber	Farrelly, Peter	Peter Farrelly	tt0109686
1746585431	1939	1939	Lady of the Tropics	Lady of the Tropics	Conway, Jack	Jack Conway	tt0031549
1746572560	1931	1931	The Homicide Squad	Homicide Squad	Melford, George; Cahn, Edward L.	Edward L. Cahn	tt0021967

1746581019	1958	1958	The Last Hurrah	The Last Hurrah	Ford, John	John Ford	tt0051845
1746410854	1982	1982	The Junkman	The Junkman	Halicki, H. B.	H.B. Halicki	tt0084184
1746583734	1931	1931	Air Eagles	Air Eagles	Whitman, Phil	Phil Whitman	tt0021591
1746501399	1915	1915	The Cheat	The Cheat		Cecil B. DeMille	tt0005078
1746516267	1920	1920	The Fighting Shepherdess	The Fighting Shepherdess	José, Edward	Edward José	tt0011181
1746583553	1929	1929	Hallelujah	Hallelujah	Vidor, King	King Vidor	tt0019959
1746423045	1994	1994	Guarding Tess	Guarding Tess	Wilson, Hugh	Hugh Wilson	tt0109951
1746454801	1997	1997	'Til There Was You	'Til There Was You	Winant, Scott	Scott Winant	tt0118523
1746566018	1953	1952	Paris Express	The Paris Express	French, Harold	Harold French	tt0046034
1746513079	1910	1910	Percy, the Cowboy	Percy the Cowboy			tt0348711
1746509251	1925	1925	The Fighting Demon	The Fighting Demon	Rosson, Arthur	Arthur Rosson	tt0131392
1769970394	1966	1966	The Sadistic Lover	The Sadistic Lover	Gunter, George	George Gunter	tt0257079
1746465422	1942	1942	The Gay Sisters	The Gay Sisters	Rapper, Irving	Irving Rapper	tt0034770
1746422509	1995	1995	Things to Do in Denver When You're Dead	Things to Do in Denver When You're Dead	Fleder, Gary	Gary Fleder	tt0114660
1745394898	2009	2009	My Son, My Son, What Have Ye Done	My Son, My Son, What Have Ye Done	Herzog, Werner	Werner Herzog	tt1233219
1746439464	1996	1996	D3: The Mighty Ducks	D3: The Mighty Ducks	Lieberman, Robert	Robert Lieberman	tt0116000
1746585321	1930	1930	Shadow of the Law	Shadow of the Law	Gasnier, Louis	Louis J. Gasnier	tt0021359
1746562253	1953	1953	Louisiana Territory	Louisiana Territory	Smith, Harry W.	Harry W. Smith	tt0230469
1746585465	1939	1939	Mr. Moto in Danger Island	Mr. Moto in Danger Island	Leeds, Herbert I.	Herbert I. Leeds	tt0031677
1746482897	1917	1917	The Moral Code	The Moral Code	Miller, Ashley	Ashley Miller	tt0008315
1745272460	2000	2000	Mission to Mars	Mission to Mars	De Palma, Brian	Brian De Palma	tt0183523
1746533167	1915	1915	The Gentleman from Indiana	The Gentleman from Indiana	Lloyd, Frank	Frank Lloyd	tt0005377
1746423485	1996	1996	The Funeral	The Funeral	Ferrara, Abel	Abel Ferrara	tt0116378
1746258165	1975	1975	The Hindenburg	The Hindenburg	Wise, Robert	Robert Wise	tt0073113
1746569701	1953	1953	Vigilante Terror	Vigilante Terror	Collins, Lewis	Lewis D. Collins	tt0046516
1746578441	1950	1950	Women from Headquarters	Women from Headquarters	Blair, George	George Blair	tt0043144
1746508419	1923	1923	Gold Madness	Gold Madness	Thornby, Robert T.	Robert Thornby	tt0014091
1746440019	1994	1994	Nobody's Fool	Nobody's Fool	Benton, Robert	Robert Benton	tt0110684
1746487480	1917	1917	The Fair Barbarian	The Fair Barbarian	Thornby, Robert	Robert Thornby	tt0007926
1746211033	1973	1973	Westworld	Westworld	Crichton, Michael	Michael Crichton	tt0070909
1746453595	1942	1942	Atlantic Convoy	Atlantic Convoy	Landers, Lew	Lew Landers	tt0034480
1746582462	1935	1935	Two Fisted	Two Fisted	Cruze, James	James Cruze	tt0027148
1746423307	1995	1995	The Scarlet Letter	The Scarlet Letter	Joffé, Roland	Roland Joffé	tt0114345
1746583833	1931	1931	Bad Company	Bad Company	Garnett, Tay	Tay Garnett	tt0021634
1746583280	1929	1929	The Harvest of Hate	The Harvest of Hate	MacRae, Henry	Henry MacRae	tt0019966
1746554846	1964	1964	Fate Is the Hunter	Fate Is the Hunter	Nelson, Ralph	Ralph Nelson	tt0058091
1746580705	1935	1935	Society Fever	Society Fever	Strayer, Frank	Frank R. Strayer	tt0027021
1746570613	1952	1952	My Man and I	My Man and I	Wellman, William A.	William A. Wellman	tt0044938
1746504604	1918	1918	String Beans	String Beans	Schertzinger, Victor L.	Victor Schertzinger	tt0009660
1745309311	2007	2007	For the Bible Tells Me So	For the Bible Tells Me So	Karslake, Daniel	Daniel G. Karslake	tt0912583
1746503531	1919	1919	The Joyous Liar	The Joyous Liar	Warde, Ernest C.	Ernest C. Warde	tt0190497
1746500664	1925	1925	The Rainbow Trail	The Rainbow Trail	Reynolds, Lynn	Lynn Reynolds	tt0016259
1746562447	1957	1957	Short Cut to Hell	Short Cut to Hell	Cagney, James	James Cagney	tt0050964
1746491781	1948	1948	Leather Gloves	Leather Gloves	Quine, Richard; Asher, William	William Asher	tt0040531
1746584935	1940	1940	Mystery in Swing	Mystery in Swing	Dreifuss, Arthur	Arthur Dreifuss	tt0032833
1746410188	1988	1988	Thelonious Monk: Straight, No Chaser	Thelonious Monk: Straight, No Chaser	Zwerin, Charlotte	Charlotte Zwerin	tt0098465
1746582566	1934	1934	Pursued	Pursued	King, Louis	Louis King	tt0025689
1746475968	1947	1947	The Man I Love	The Man I Love	Walsh, Raoul	Raoul Walsh	tt0038721
1746577746	1937	1936	Charlie Chan at the Opera	Charlie Chan at the Opera	Humberstone, H. Bruce	H. Bruce Humberstone	tt0027440
1746217855	1967	1966	One-Eyed Soldiers	The One Eyed Soldiers	Christophe, Jean	John Ainsworth	tt0223839
1746575061	1937	1937	Angel	Angel	Lubitsch, Ernst	Ernst Lubitsch	tt0028575
1746420872	1991	1991	Father of the Bride	Father of the Bride	Shyer, Charles	Charles Shyer	tt0101862
1746439408	1943	1943	Teen Age	Teen Age	L'Estrange, Dick	Richard L'Estrange	tt0037356
1746392737	1981	1981	History of the World: Part I	History of the World: Part I	Brooks, Mel	Mel Brooks	tt0082517
1746520889	1949	1948	Incident	Incident	Beaudine, William	William Beaudine	tt0041508
1769976685	1979	1979	Up from the Depths	Up from the Depths	Griffith, Charles B.	Charles B. Griffith	tt0080066
1746496407	1919	1919	The Man Who Turned White	The Man Who Turned White	Frame, Park	Park Frame	tt0010422

1746566010	1953	1953	Captain John Smith and Pocahontas	Captain John Smith and Pocahontas	Landers, Lew	Lew Landers	tt0045603
1746584582	1931	1931	Other Men's Women	Other Men's Women	Wellman, William A.	William A. Wellman	tt0022236
1746569592	1954	1953	Money from Home	Money from Home	Marshall, George	George Marshall	tt0046087
1746453786	1942	1942	This Above All	This Above All	Litvak, Anatole	Anatole Litvak	tt0035431
1746483912	1918	1918	The Count and the Wedding Guest	The Count and the Wedding Guest	Justice, Martin	Martin Justice	tt0425919
1746563633	1952	1952	Talk About a Stranger	Talk About a Stranger	Bradley, David	David Bradley	tt0045216
1746573885	1958	1958	Lafayette Escadrille	Lafayette Escadrille	Wellman, William A.	William A. Wellman	tt0051840
1746520071	1926	1926	Under Fire	Under Fire	Elfelt, Clifford S.	Clifford S. Elfelt	tt0017496
1746560629	1932	1932	Crooner	Crooner	Bacon, Lloyd	Lloyd Bacon	tt0022789
1746574772	1935	1935	The Lone Wolf Returns	The Lone Wolf Returns	Neill, Roy William	Roy William Neill	tt0026646
1746475969	1946	1946	Sing While You Dance	Sing While You Dance	Lederman, D. Ross	D. Ross Lederman	tt0038943
1746453588	1942	1942	Highways by Night	Highways by Night	Godfrey, Peter	Peter Godfrey	tt0034853
1746571072	1935	1935	Chasing Yesterday	Chasing Yesterday	Nicholls, George	George Nichols Jr.	tt0026200
1746423559	1993	1993	Demolition Man	Demolition Man	Brambilla, Marco	Marco Brambilla	tt0106697
1745303169	2006	2006	Just My Luck	Just My Luck	Petrie, Donald	Donald Petrie	tt0397078
1746438208	1943	1943	Flesh and Fantasy	Flesh and Fantasy	Duvivier, Julien	Julien Duvivier	tt0035885
1746434901	1995	1995	Before Sunrise	Before Sunrise	Linklater, Richard	Richard Linklater	tt0112471
1746233021	1968	1968	I Love You, Alice B. Toklas!	I Love You, Alice B. Toklas!	Averback, Hy	Hy Averback	tt0063115
1746503819	1922	1922	Always the Woman	Always the Woman	Rosson, Arthur	Arthur Rosson	tt0012892
1746421000	1989	1989	Split	Split	Shaw, Chris	Chris Shaw	tt0098374
1746419918	1990	1990	Funny About Love	Funny About Love	Nimoy, Leonard	Leonard Nimoy	tt0099623
1746582648	1936	1936	The Accusing Finger	The Accusing Finger	Hogan, James	James P. Hogan	tt0027253
1746435905	1996	1996	Primal Fear	Primal Fear	Hoblit, Gregory	Gregory Hoblit	tt0117381
1746569574	1953	1953	The Veils of Bagdad	The Veils of Bagdad	Sherman, George	George Sherman	tt0046497
1746210173	1971	1971	Dracula vs. Frankenstein	Dracula vs. Frankenstein	Adamson, Al	Al Adamson	tt0067017
1746504127	1917	1917	A Night in New Arabia	A Night in New Arabia	Mills, Thomas R.	Thomas R. Mills	tt0181721
1746394758	1981	1981	Modern Problems	Modern Problems	Shapiro, Ken	Ken Shapiro	tt0082763
1746569951	1957	1957	The Tall T	The Tall T	Boetticher, Budd	Budd Boetticher	tt0051047
1746578510	1961	1961	The Sand Castle	The Sand Castle	Hill, Jerome	Jerome Hill	tt0055403
1746573955	1962	1961	Sail a Crooked Ship	Sail a Crooked Ship	Brecher, Irving	Irving Brecher	tt0055394
1746577645	1937	1937	Don't Tell the Wife	Don't Tell the Wife	Cabanne, Christy	Christy Cabanne	tt0028799
1746575071	1938	1938	Sergeant Murphy	Sergeant Murphy	Eason, B. Reeves	B. Reeves Eason	tt0030731
1746496415	1919	1919	Who's Your Brother?	Who's Your Brother?	Adolfi, John G.	John G. Adolfi	tt0010885
1746582218	1951	1951	Secrets of Monte Carlo	Secrets of Monte Carlo	Blair, George	George Blair	tt0044016
1746235078	1980	1980	The Hunter	The Hunter	Kulik, Buzz	Buzz Kulik	tt0080907
1746500148	1945	1945	Senorita from the West	Senorita from the West	Strayer, Frank	Frank R. Strayer	tt0038069
1746486323	1918	1918	T'other Dear Charmer	T'Other Dear Charmer	Earle, William P. S.	William P.S. Earle	tt0267008
1746465395	1941	1941	Meet John Doe	Meet John Doe	Capra, Frank	Frank Capra	tt0033891
1745374446	2008	2007	Sex and Death 101	Sex and Death 101	Waters, Daniel	Daniel Waters	tt0497972
1769969989	1912	1912	Riley and Schultze	Riley and Schultz	Sennett, Mack	Mack Sennett	tt0002462
1746577014	1959	1959	Solomon and Sheba	Solomon and Sheba	Vidor, King	King Vidor	tt0053290
1746565973	1954	1954	Jubilee Trail	Jubilee Trail	Kane, Joseph Inman	Joseph Kane	tt0047137
1746496513	1918	1918	Staking His Life	Staking His Life			tt0190033
1769974379	1916	1916	When Seconds Count	When Seconds Count		J. Gunnis Davis	tt1534566
1769974372	1916	1916	A Life Chase	A Life Chase		Travers Vale	tt0906039
1746532730	1915	1915	Pretenses	Pretenses	Dillon, Jack	James Douglass	tt0481957
1746502487	1915	1914	Environment	Environment		Christy Cabanne	tt0357637
1746409363	1988	1988	U2: Rattle and Hum	U2: Rattle and Hum	Joanou, Phil	Phil Joanou	tt0096328
1746393752	1984	1984	The Good Fight: The Abraham Lincoln Brigade in	The Good Fight: The Abraham Lincoln Briga	Buckner, Noel; Dore, Mary; Sills, Sam	Noel Buckner	tt0085610
1746219685	1974	1974	Zardoz	Zardoz	Boorman, John	John Boorman	tt0070948
1746581579	1951	1951	Two of a Kind	Two of a Kind	Levin, Henry	Henry Levin	tt0044159
1746475960	1948	1948	Assigned to Danger	Assigned to Danger	Boetticher, Oscar	Budd Boetticher	tt0040119
1746474440	1947	1947	King of the Wild Horses	King of the Wild Horses	Archainbaud, George	George Archainbaud	tt0039534
1746500019	1945	1945	Rough Ridin' Justice	Rough Ridin' Justice	Abrahams, Derwin	Derwin Abrahams	tt0236697
1746524573	1928	1928	Jesus of Nazareth	Jesus of Nazareth		Jean Conover	tt0330487
1746582294	1928	1928	Brand of Courage	The Brand of Courage	Mitchell, Bruce	Bruce Mitchell	tt0301064
1746500501	1925	1925	The Lost Chord	The Lost Cord	Noy, Wilfred	Richard Smith	tt0347438

1746473665	1942	1942	Jungle Siren	Jungle Siren	Newfield, Sam	Sam Newfield	tt0034929
1746439148	1941	1941	The Singing Hill	The Singing Hills	Landers, Lew	Josef Berne	tt0385234
1746580706	1937	1937	Night Must Fall	Night Must Fall	Thorpe, Richard	Richard Thorpe	tt0029310
1746561901	1954	1954	The Snow Creature	The Snow Creature	Wilder, W. Lee	W. Lee Wilder	tt0047507
1746562241	1953	1953	Mahatma Gandhi: 20th Century Prophet	Mahatma Gandhi 20th Century Prophet		Stanley Neal	tt0352551
1746198422	1968	1968	The Golden Breed	The Golden Breed	Davis, Dale	Dale Davis	tt1165283
1746574259	1963	1963	Rampage	Rampage	Karlson, Phil	Phil Karlson	tt0057447
1746574724	1935	1935	The Right to Live	The Right to Live	Keighley, William	William Keighley	tt0026934
1746574193	1962	1962	The Underwater City	The Underwater City	McDonald, Frank	Frank McDonald	tt0056637
1746509509	1925	1925	A Lover's Oath	A Lover's Oath	Earle, Ferdinand P.	Ferdinand P. Earle	tt0013339
1746565936	1957	1957	Boy on a Dolphin	Boy on a Dolphin	Negulesco, Jean	Jean Negulesco	tt0050208
1746576167	1931	1931	This Modern Age	This Modern Age	Grindé, Nicholas	Nick Grinde	tt0022478
1746567442	1933	1933	The Flaming Signal	The Flaming Signal	Jeske, George; Roberts, C. Edward	George Jeske	tt0024019
1746419370	1981	1981	The Howling	The Howling	Dante, Joe	Joe Dante	tt0082533
1746583946	1930	1930	Canyon Hawks	Canyon Hawks	Neitz, Alvin J.	Alan James	tt0020736
1746572112	1932	1932	Veiled Aristocrats	Veiled Aristocrats	Micheaux, Oscar	Oscar Micheaux	tt0023655
1746571285	1934	1934	A Girl of the Limberlost	A Girl of the Limberlost	Cabanne, Christy	Christy Cabanne	tt0025178
1746566819	1933	1933	Broken Dreams	Broken Dreams	Vignola, Robert	Robert G. Vignola	tt0023849
1746533175	1928	1928	A Midnight Adventure	The Midnight Adventure	Worne, Duke	Duke Worne	tt0019161
1746492205	1947	1947	Blind Spot	Blind Spot	Gordon, Robert	Robert Gordon	tt0039197
1746198747	1967	1967	Fitzwilly	Fitzwilly	Mann, Delbert	Delbert Mann	tt0061669
1746583701	1931	1931	Friends and Lovers	Friends and Lovers	Schertzinger, Victor	Victor Schertzinger	tt0021889
1746532414	1927	1927	Winners of the Wilderness	Winners of the Wilderness	Van Dyke, W. S.	W.S. Van Dyke	tt0018579
1746500103	1923	1923	The Covered Wagon	The Covered Wagon	Cruze, James	James Cruze	tt0013951
1746578606	1960	1960	The Beatniks	The Beatniks	Frees, Paul	Paul Frees	tt0053640
1746580570	1938	1938	Tropic Holiday	Tropic Holiday	Reed, Theodore	Theodore Reed	tt0030897
1746578496	1958	1958	Teenage Monster	Teenage Monster	Marquette, Jacques	Jacques R. Marquette	tt0051063
1746583630	1928	1928	The Mating Call	The Mating Call	Cruze, James	James Cruze	tt0019152
1746501133	1910	1910	The Sea Wolves	The Sea Wolves	Balshofer, Fred J.	Fred J. Balshofer	tt0257113
1746577721	1933	1933	The Secret of Madame Blanche	The Secret of Madame Blanche	Brabin, Charles	Charles Brabin	tt0024537
1746235288	1981	1981	Absence of Malice	Absence of Malice	Pollack, Sydney	Sydney Pollack	tt0081974
1746583299	1931	1931	The Sin of Madelon Claudet	The Sin of Madelon Claudet	Selwyn, Edgar	Edgar Selwyn	tt0022386
1746218667	1972	1971	ZAAT	Zaat	Barton, Don	Don Barton	tt0072666
1746453585	1943	1942	Yankee Doodle Dandy	Yankee Doodle Dandy	Curtiz, Michael	Michael Curtiz	tt0035575
1746514330	1910	1910	An Unselfish Love	An Unselfish Love	Dawley, J. Searle	J. Searle Dawley	tt0759945
1746583067	1935	1935	The Best Man Wins	The Best Man Wins	Kenton, Erle C.	Erle C. Kenton	tt0026108
1746423478	1996	1996	The Stupids	The Stupids	Landis, John	John Landis	tt0117768
1745302835	2006	2006	Date Movie	Date Movie	Seltzer, Aaron	Aaron Seltzer	tt0466342
1746566166	1958	1958	The Decks Ran Red	The Decks Ran Red	Stone, Andrew L.	Andrew L. Stone	tt0051524
1746491768	1945	1944	Music for Millions	Music for Millions	Koster, Henry	Henry Koster	tt0037104
1746585075	1930	1930	The Right to Love	The Right to Love	Wallace, Richard	Richard Wallace	tt0021301
1746484151	1918	1918	Secret Strings	Secret Strings	Ince, John	John Ince	tt0009594
1746465409	1947	1947	Rustlers of Devil's Canyon	Rustlers of Devil's Canyon	Springsteen, R. G.	R.G. Springsteen	tt0039791
1746583492	1950	1950	Highway 301	Highway 301	Stone, Andrew	Andrew L. Stone	tt0042250
1746438512	1997	1997	Kiss the Girls	Kiss the Girls	Fleder, Gary	Gary Fleder	tt0119468
1746504786	1922	1922	The Better Man Wins	The Better Man Wins	Perez, Marcel	Frank S. Mattison	tt0012933
1746580983	1950	1950	Rocky Mountain	Rocky Mountain	Keighley, William	William Keighley	tt0042899
1746569760	1958	1958	Hot Spell	Hot Spell	Mann, Daniel	Daniel Mann	tt0051742
1745374188	2002	2002	Ash Wednesday	Ash Wednesday	Burns, Edward	Edward Burns	tt0280438
1746578444	1963	1963	Shock Corridor	Shock Corridor	Fuller, Samuel	Samuel Fuller	tt0057495
1746581399	1961	1961	Living Venus	Living Venus	Lewis, Herschell Gordon	Herschell Gordon Lewis	tt0054035
1746578497	1962	1962	Escape from Zahrain	Escape from Zahrain	Neame, Ronald	Ronald Neame	tt0055963
1746438207	1942	1942	The Man with Two Lives	Man with Two Lives	Rosen, Phil	Phil Rosen	tt0035031
1746503788	1921	1921	Nobody's Fool	Nobody's Fool	Baggot, King	King Baggot	tt0012507
1746514944	1923	1923	Bright Lights of Broadway	Bright Lights of Broadway	Campbell, Webster	Webster Campbell	tt0013891
1746577514	1935	1935	To Beat the Band	To Beat the Band	Stoloff, Ben	Benjamin Stoloff	tt0027115
1746513679	1910	1910	The Paleface Princess	The Paleface Princess		William F. Haddock	tt2239160

1746507764	1926	1926	Deuce High	Deuce High	Thorpe, Richard	Richard Thorpe	tt0016780
1746574481	1934	1934	Chained	Chained	Brown, Clarence	Clarence Brown	tt0024963
1769969879	1912	1912	Two Women	Two Women			tt0385303
1746584027	1935	1935	The Nut Farm	The Nut Farm	Brown, Melville	Melville W. Brown	tt0026797
1746582458	1938	1938	Blond Cheat	Blond Cheat	Santley, Joseph	Joseph Santley	tt0029925
1746570728	1933	1933	Drum Taps	Drum Taps	McGowan, J. P.	J.P. McGowan	tt0023967
1746568023	1933	1933	One Year Later	One Year Later	Hopper, E. Mason	E. Mason Hopper	tt0024416
1746554872	1955	1954	Black Tuesday	Black Tuesday	Fregonese, Hugo	Hugo Fregonese	tt0046790
1746569703	1956	1956	On the Threshold of Space	On the Threshold of Space	Webb, Robert D.	Robert D. Webb	tt0049571
1746554555	1965	1965	Strange Bedfellows	Strange Bedfellows	Frank, Melvin	Melvin Frank	tt0058621
1746524712	1917	1917	The Girl and the Crisis	The Girl and the Crisis	Mong, William V.	William V. Mong	tt0008005
1746524593	1910	1910	When We Were in Our Teens	When We Were in Our Teens	Powell, Frank	Frank Powell	tt0001454
1746530694	1949	1949	Oh, You Beautiful Doll	Oh, You Beautiful Doll	Stahl, John M.	John M. Stahl	tt0041712
1745374804	2008	2007	Cthulhu	Cthulhu	Gildark, Dan	Dan Gildark	tt0478126
1746571873	1910	1910	The Fire Chief's Daughter	The Fire Chief's Daughter		Francis Boggs	tt0221176
1746504086	1919	1919	A Man of Honor	A Man of Honor	Balshofer, Fred J.	Fred J. Balshofer	tt0010427
1746582789	1937	1937	Public Cowboy No. 1	Public Cowboy No. 1	Kane, Joe	Joseph Kane	tt0029445
1746584703	1929	1929	Captain Cowboy	Captain Cowboy	McGowan, J. P.	J.P. McGowan	tt0019747
1746454456	1942	1942	House of Errors	House of Errors	Ray, Bernard B.	Bernard B. Ray	tt0034871
1746584872	1939	1939	Hidden Power	Hidden Power	Collins, Lewis D.	Lewis D. Collins	tt0031423
1746500346	1926	1926	Beyond the Trail	Beyond the Trail	Herman, Al	Albert Herman	tt0016648
1746492082	1946	1946	Inside Job	Inside Job	Yarbrough, Jean	Jean Yarbrough	tt0038643
1746234349	1968	1968	Killers Three	Killers Three	Kessler, Bruce	Bruce Kessler	tt0063184
1746465046	1947	1947	Buffalo Bill Rides Again	Buffalo Bill Rides Again	Ray, Bernard B.	Bernard B. Ray	tt0039227
1746507587	1949	1949	The Kid from Cleveland	The Kid from Cleveland	Kline, Herbert	Herbert Kline	tt0041545
1746524159	1915	1915	Alias Jimmy Valentine	Alias Jimmy Valentine	Tourneur, Maurice	Maurice Tourneur	tt0004872
1746584096	1938	1938	Smashing the Rackets	Smashing the Rackets	Landers, Lew	Lew Landers	tt0030765
1746233979	1967	1967	Portrait of Jason	Portrait of Jason	Clarke, Shirley	Shirley Clarke	tt0062144
1746574549	1936	1936	Valiant Is the Word for Carrie	Valiant Is the Word for Carrie	Ruggles, Wesley	Wesley Ruggles	tt0028455
1746577043	1959	1959	Rio Bravo	Rio Bravo	Hawks, Howard	Howard Hawks	tt0053221
1746569902	1957	1957	Ride a Violent Mile	Ride a Violent Mile	Warren, Charles Marquis	Charles Marquis Warren	tt0050901
1746503577	1922	1922	Oh! Mabel Behave!	Oh, Mabel Behave	Sennett, Mack; Sterling, Ford	Mack Sennett	tt0013448
1746500886	1916	1916	The Dawn of Love	The Dawn of Love	Carewe, Edwin	Edwin Carewe	tt0006572
1746562339	1956	1956	The Mole People	The Mole People	Vogel, Virgil	Virgil W. Vogel	tt0049516
1746585101	1928	1928	The Clean-Up Man	The Clean Up Man	Taylor, Ray	Ray Taylor	tt0018776
1746219391	1973	1973	The Last of Sheila	The Last of Sheila	Ross, Herbert	Herbert Ross	tt0070291
1746408427	1990	1989	Fun Down There	Fun Down There	Stigliano, Roger	Roger Stigliano	tt0095187
1746584737	1928	1928	The Blue Danube	The Blue Danube	Sloane, Paul	Paul Sloane	tt0018711
1746486480	1920	1920	Dangerous Love	Dangerous Love	Bartlett, Charles E.	Charles Bartlett	tt0150268
1746454381	1943	1942	The Pride of the Yankees	The Pride of the Yankees	Wood, Sam	Sam Wood	tt0035211
1745374139	2002	2001	Knockaround Guys	Knockaround Guys	Levien, David ; Koppelman, Brian	Brian Koppelman	tt0211465
1746573881	1958	1958	Handle with Care	Handle with Care	Friedkin, David	David Friedkin	tt0051697
1746408467	1987	1987	RoboCop	RoboCop	Verhoeven, Paul	Paul Verhoeven	tt0093870
1746423313	1990	1990	Misery	Misery	Reiner, Rob	Rob Reiner	tt0100157
1746583965	1935	1935	Mary Burns, Fugitive	Mary Burns, Fugitive	Howard, William K.	William K. Howard	tt0026688
1746487912	1919	1919	The Right to Lie	The Right to Lie	Carewe, Edwin	Edwin Carewe	tt0011628
1746235240	1973	1973	Papillon	Papillon	Schaffner, Franklin J.	Franklin J. Schaffner	tt0070511
1746210740	1972	1972	Avanti!	Avanti!	Wilder, Billy	Billy Wilder	tt0068240
1746525024	1920	1920	The Jungle Princess	The Jungle Princess	Martin, E. A.	E.A. Martin	tt0195853
1746564135	1953	1953	Bandits of Corsica	Bandits of Corsica	Nazarro, Ray	Ray Nazarro	tt0045539
1746436230	1990	1989	For All Mankind	For All Mankind	Reinert, Al	Al Reinert	tt0097372
1746436638	1943	1943	Dead Men Walk	Dead Men Walk	Newfield, Sam	Sam Newfield	tt0035784
1746508043	1926	1926	The Mile-a-Minute Man	The Mile a Minute Man	Nelson, Jack	Jack Nelson	tt0017150
1746410361	1990	1990	The Freshman	The Freshman	Bergman, Andrew	Andrew Bergman	tt0099615
1746571141	1935	1935	Anna Karénina	Anna Karenina	Brown, Clarence	Clarence Brown	tt0026071
1746409372	1990	1990	Total Recall	Total Recall	Verhoeven, Paul	Paul Verhoeven	tt0100802
1746582486	1938	1938	Straight Place and Show	Straight Place and Show	Butler, David	David Butler	tt0030802

1746562144	1954	1954	The White Orchid	The White Orchid	Le Borg, Reginald	Reginald Le Borg	tt0047675
1746533169	1916	1916	The Dead Alive	The Dead Alive	Vernot, Henry J.	Henry J. Vernot	tt0157514
1769969499	1911	1911	Niagara Falls Celebration	Niagara Falls Celebration			tt1282450
1746584498	1931	1931	Scandal Sheet	Scandal Sheet	Cromwell, John	John Cromwell	tt0022343
1746582580	1936	1936	Easy to Take	Easy to Take	Tryon, Glenn	Glenn Tryon	tt0027565
1746553786	1964	1964	Emil and the Detectives	Emil and the Detectives	Tewksbury, Peter	Peter Tewksbury	tt0058056
1745341750	2002	2002	Panic Room	Panic Room	Fincher, David	David Fincher	tt0258000
1746235927	1976	1975	The Wild Party	The Wild Party	Ivory, James	James Ivory	tt0073903
1746474532	1948	1948	The Hunted	The Hunted	Bernhard, Jack	Jack Bernhard	tt0040455
1746583291	1930	1930	A Daughter of the Congo	A Daughter of the Congo	Micheaux, Oscar	Oscar Micheaux	tt0020813
1746494197	1910	1910	The Love of Lady Irma	The Love of Lady Irma	Powell, Frank	Frank Powell	tt0361854
1746504077	1920	1920	The Penalty	The Penalty	Worsley, Wallace	Wallace Worsley	tt0011565
1746524940	1914	1914	The Lure	The Lure		Alice Guy	tt0004275
1746439717	1999	1999	The Cider House Rules	The Cider House Rules	Hallström, Lasse	Lasse Hallström	tt0124315
1746420801	1997	1997	The Locusts	The Locusts	Kelley, John Patrick	John Patrick Kelley	tt0119557
1745374798	2010	2010	Due Date	Due Date	Phillips, Todd	Todd Phillips	tt1231583
1746433567	1992	1992	The Playboys	The Playboys	MacKinnon, Gillies	Gillies MacKinnon	tt0105150
1746464917	1941	1941	Outlaws of the Panhandle	Outlaws of the Panhandle	Nelson, Sam	Sam Nelson	tt0033990
1746569752	1955	1955	Chicago Syndicate	Chicago Syndicate	Sears, Fred F.	Fred F. Sears	tt0047934
1746584412	1939	1939	These Glamour Girls	These Glamour Girls	Simon, S. Sylvan	S. Sylvan Simon	tt0032018
1746501341	1915	1915	The Fight	The Fight	Lederer, George W.	George W. Lederer	tt0005322
1746570154	1933	1933	The Bowery	The Bowery	Walsh, Raoul	Raoul Walsh	tt0023838
1746250967	1977	1977	3 Women	3 Women	Altman, Robert	Robert Altman	tt0075612
1746436332	1943	1943	China	China	Farrow, John	John Farrow	tt0035735
1746577098	1961	1961	The Fiend of Dope Island	The Fiend of Dope Island	Watt, Nate	Nate Watt	tt0194879
1746507847	1948	1948	The Westward Trail	The Westward Trail	Taylor, Ray	Ray Taylor	tt0040957
1746465159	1942	1942	Sabotage Squad	Sabotage Squad	Landers, Lew	Lew Landers	tt0035277
1746208180	1967	1967	****	****	Warhol, Andy	Andy Warhol	tt0179184
1746514085	1928	1928	The Little Wild Girl	The Little Wild Girl	Mattison, Frank S.	Frank S. Mattison	tt0019106
1746573673	1932	1932	State's Attorney	State's Attorney	Archainbaud, George	George Archainbaud	tt0023507
1746497263	1922	1922	The Man She Brought Back	The Man She Brought Back	Miller, Charles	Charles Miller	tt0013357
1745340773	2001	2001	K-Pax	K PAX	Softley, Iain	Iain Softley	tt0272152
1745358065	2010	2010	Inception	Inception	Nolan, Christopher	Christopher Nolan	tt1375666
1745372699	2003	2003	The Big Empty	The Big Empty	Anderson, Steve	Steve Anderson	tt0321442
1746487308	1919	1919	Muggsy	Muggsy	MacDonald, Sherwood	Sherwood MacDonald	tt0191296
1746543071	1914	1914	Home, Sweet Home	Home, Sweet Home	Griffith, D. W.	D.W. Griffith	tt0003167
1746555411	1955	1955	Apache Woman	Apache Woman	Corman, Roger	Roger Corman	tt0047837
1746474467	1947	1947	My Favorite Brunette	My Favorite Brunette	Nugent, Elliott	Elliott Nugent	tt0039645
1746550303	1954	1954	Racing Blood	Racing Blood	Barry, Wesley	Wesley Barry	tt0047386
1746497002	1922	1922	Trail's End	Trail's End	Ford, Francis	Francis Ford	tt0013703
1746584718	1929	1929	In the Headlines	In the Headlines	Adolfi, John G.	John G. Adolfi	tt0020021
1746584708	1929	1929	Side Street	Side Street	St. Clair, Malcolm	Malcolm St. Clair	tt0020404
1746561255	1932	1932	The Unwritten Law	The Unwritten Law	Cabanne, Christy	Christy Cabanne	tt0023644
1745373159	2003	2003	The Life of David Gale	The Life of David Gale	Parker, Alan	Alan Parker	tt0289992
1746467981	1917	1917	A Marked Man	A Marked Man	Ford, Jack	John Ford	tt0008271
1746564504	1952	1952	Hurricane Smith	Hurricane Smith	Hopper, Jerry	Jerry Hopper	tt0044729
1746584014	1939	1939	Paris Honeymoon	Paris Honeymoon	Tuttle, Frank	Frank Tuttle	tt0031787
1746502656	1916	1916	Susan Rocks the Boat	Susan Rocks the Boat	Powell, Paul	Paul Powell	tt0007414
1746507874	1923	1923	If Winter Comes	If Winter Comes	Millarde, Harry	Harry F. Millarde	tt0014147
1746408906	1990	1990	Men Don't Leave	Men Don't Leave	Brickman, Paul	Paul Brickman	tt0100134
1746234738	1972	1972	The Last House on the Left	The Last House on the Left	Craven, Wes	Wes Craven	tt0068833
1746497913	1921	1921	Princess Jones	Princess Jones	Seyffertitz, G. V.	Gustav von Seyffertitz	tt0012590
1746524393	1923	1923	The Ten Commandments	The Ten Commandments	De Mille, Cecil B.	Cecil B. DeMille	tt0014532
1746583059	1937	1937	One Mile from Heaven	One Mile from Heaven	Dwan, Allan	Allan Dwan	tt0029349
1746574033	1960	1961	Five Minutes to Live	Five Minutes to Live	Karn, Bill	Bill Karn	tt0054817
1746577382	1951	1951	Chain of Circumstance	Chain of Circumstance	Jason, Will	Will Jason	tt0043396
1746248751	1974	1973	Superdad	Superdad	McEveety, Vincent	Vincent McEveety	tt0072229

1746487329	1921	1921	Remorseless Love	Remorseless Love	Ince, Ralph	Ralph Ince	tt0012617
1745322643	2007	2007	Pirates of the Caribbean: At World's End	Pirates of the Caribbean: At World's End	Verbinski, Gore	Gore Verbinski	tt0449088
1745309509	2006	2006	Art School Confidential	Art School Confidential	Zwigoff, Terry	Terry Zwigoff	tt0364955
1746422041	1992	1992	Hoffa	Hoffa	DeVito, Danny	Danny DeVito	tt0104427
1746574867	1935	1935	Mark of the Vampire	Mark of the Vampire	Browning, Tod	Tod Browning	tt0026685
1745293355	2007	2007	Descent	Descent	Lugacy, Talia	Talia Lugacy	tt0463027
1746497587	1920	1920	The Key to Power	The Key to Power	Parke, William	William Parke	tt0009254
1746453601	1941	1941	The Feminine Touch	The Feminine Touch	Van Dyke, W. S.	W.S. Van Dyke	tt0033596
1746495731	1919	1919	The Feud	The Feud	Le Saint, Edward	Edward LeSaint	tt0010117
1746503231	1922	1923	The Right That Failed	The Light That Failed	Veiller, Bayard	George Melford	tt0014198
1746435019	1998	1998	Pleasantville	Pleasantville	Ross, Gary	Gary Ross	tt0120789
1746508743	1925	1925	Gold and Grit	Gold and Grit	Thorpe, Richard	Richard Thorpe	tt0015865
1746394650	1990	1990	Henry & June	Henry & June	Kaufman, Philip	Philip Kaufman	tt0099762
1746582509	1935	1935	Texas Jack	Texas Jack	Ray, B. B.	Bernard B. Ray	tt0027085
1746584433	1931	1931	The Exile	The Exile	Micheaux, Oscar	Oscar Micheaux	tt0021844
1746520900	1949	1949	Thieves' Highway	Thieves' Highway	Dassin, Jules	Jules Dassin	tt0041958
1746492462	1948	1948	Let's Live Again	Let's Live Again	Leeds, Herbert I.	Herbert I. Leeds	tt0174866
1746584206	1939	1939	You Can't Get Away with Murder	You Can't Get Away with Murder	Seiler, Lewis	Lewis Seiler	tt0032153
1746577572	1935	1935	Devil's Canyon	Devil's Canyon		Clifford Smith	tt0238160
1746577922	1936	1936	Early to Bed	Early to Bed	McLeod, Norman	Norman Z. McLeod	tt0027561
1746394153	1986	1986	Jumpin' Jack Flash	Jumpin' Jack Flash	Marshall, Penny	Penny Marshall	tt0091306
1746394208	1984	1983	Seeing Red	Seeing Red	Reichert, Julia; Klein, James	Jim Klein	tt0086273
1746235461	1971	1971	The Last Picture Show	The Last Picture Show	Bogdanovich, Peter	Peter Bogdanovich	tt0067328
1746497530	1918	1918	The Ranger	The Ranger	Gray, Bob	Robert Gray	tt0009534
1746234956	1965	1965	When the Boys Meet the Girls	When the Boys Meet the Girls	Ganzer, Alvin	Alvin Ganzer	tt0059904
1746580242	1935	1935	Star of Midnight	Star of Midnight	Roberts, Stephen	Stephen Roberts	tt0027040
1745340003	2004	2004	Surviving Christmas	Surviving Christmas	Mitchell, Mike	Mike Mitchell	tt0252028
1746504135	1918	1917	The Gulf Between	The Gulf Between	Physioc, Wray	Wray Bartlett Physioc	tt0009136
1746514499	1915	1915	On Her Wedding Night	On Her Wedding Night	Humphrey, William	George D. Baker	tt0005828
1746554240	1963	1963	The Stripper	The Stripper	Schaffner, Franklin J.	Franklin J. Schaffner	tt0057537
1745282624	2007	2007	It Is Fine! Everything Is Fine	It Is Fine! Everything Is Fine.	Glover, Crispin Hellion; Brothers, Da	David Brothers	tt0795405
1746524983	1923	1923	Thundergate	Thundergate	De Grasse, Joseph	Joseph De Grasse	tt0014544
1746208878	1968	1968	Madigan	Madigan	Siegel, Donald	Don Siegel	tt0063256
1746436351	1941	1940	The Bride Wore Crutches	The Bride Wore Crutches	Traube, Shepard	Shepard Traube	tt0032279
1746492591	1945	1945	A Guy, a Gal, and a Pal	A Guy, a Gal and a Pal	Boetticher, Oscar	Budd Boetticher	tt0037759
1746454052	1943	1943	Shantytown	Shantytown	Santley, Joseph	Joseph Santley	tt0036344
1746523347	1923	1923	The Scarlet Lily	The Scarlet Lily	Schertzinger, Victor	Victor Schertzinger	tt0014442
1746509186	1926	1926	Old Loves and New	Old Loves and New	Tourneur, Maurice	Maurice Tourneur	tt0017227
1746516344	1921	1921	Jim the Penman	Jim the Penman	Webb, Kenneth	Kenneth S. Webb	tt0012333
1746577770	1938	1938	Rascals	Rascals	Humberstone, H. Bruce	H. Bruce Humberstone	tt0030654
1746235766	1977	1977	The Hills Have Eyes	The Hills Have Eyes	Craven, Wes	Wes Craven	tt0077681
1746219026	1974	1974	The Godfather Part II	The Godfather: Part II	Coppola, Francis Ford	Francis Ford Coppola	tt0071562
1746514575	1916	1916	Unto Those Who Sin	Unto Those Who Sin	Daly, William Robert	William Robert Daly	tt0159081
1746436886	1994	1994	Princess Caraboo	Princess Caraboo	Austin, Michael	Michael Austin	tt0110892
1746420882	1990	1990	Mr. & Mrs. Bridge	Mr. & Mrs. Bridge	Ivory, James	James Ivory	tt0100200
1746555378	1955	1955	Trial	Trial	Robson, Mark	Mark Robson	tt0048748
1746524799	1914	1914	The Patchwork Girl of Oz	The Patchwork Girl of Oz	MacDonald, J. Farrell	J. Farrell MacDonald	tt0004457
1746555185	1957	1957	Operation Mad Ball	Operation Mad Ball	Quine, Richard	Richard Quine	tt0050805
1746577048	1963	1963	Bunny Yeager's Nude Camera	Bunny Yeager's Nude Camera	Mahon, Barry	Barry Mahon	tt0057906
1746503940	1918	1917	The Lesson	The Lesson	Giblyn, Charles	Charles Giblyn	tt0009288
1746530769	1949	1949	Riders of the Whistling Pines	Riders of the Whistling Pines	English, John	John English	tt0041809
1746567078	1932	1932	Fast Life	Fast Life	Pollard, Harry	Harry A. Pollard	tt0022882
1746454402	1945	1945	Rough, Tough and Ready	Rough, Tough and Ready	Lord, Del	Del Lord	tt0038037
1746583377	1950	1950	Saddle Tramp	Saddle Tramp	Fregonese, Hugo	Hugo Fregonese	tt0042915
1746574203	1960	1960	The Magnificent Seven	The Magnificent Seven	Sturges, John	John Sturges	tt0054047
1746455257	1999	1999	Blue Streak	Blue Streak	Mayfield, Les	Les Mayfield	tt0181316
1746465225	1947	1947	Love and Learn	Love and Learn	Cordova, Frederick de	Frederick De Cordova	tt0039585

1746198769	1969	1969	Blue Movie	Blue Movie	Warhol, Andy	Andy Warhol	tt0062745
1745394877	2010	2010	Furry Vengeance	Furry Vengeance	Kumble, Roger	Roger Kumble	tt0492389
1769970687	1968	1968	Indecent Desires	Indecent Desires	Silverman, Louis	Doris Wishman	tt0061561
1746560558	1932	1932	Business and Pleasure	Business and Pleasure	Butler, David; Taylor, Sam	David Butler	tt0021702
1746503568	1921	1921	Forbidden Love	Forbidden Love	Van Loan, Philip	Philip Van Loan	tt0212169
1746585396	1940	1940	Grandpa Goes to Town	Grandpa Goes to Town	Meins, Gus	Gus Meins	tt0032547
1746455959	1997	1997	That Darn Cat	That Darn Cat	Spiers, Bob	Bob Spiers	tt0120317
1746465073	1945	1945	Radio Stars on Parade	Radio Stars on Parade	Goodwins, Leslie	Leslie Goodwins	tt0038012
1746580450	1935	1935	Whispering Smith Speaks	Whispering Smith Speaks	Howard, David	David Howard	tt0027206
1746421291	1994	1994	Exit to Eden	Exit to Eden	Marshall, Garry	Garry Marshall	tt0109758
1746500422	1925	1925	New Brooms	New Brooms	de Mille, William	William C. de Mille	tt0016151
1746474496	1944	1944	Shadows in the Night	Shadows in the Night	Forde, Eugene J.	Eugene Forde	tt0037266
1746566303	1957	1957	Edge of the City	Edge of the City	Ritt, Martin	Martin Ritt	tt0050347
1746568344	1933	1933	After Tonight	After Tonight	Archainbaud, George	George Archainbaud	tt0023742
1746566347	1958	1958	The Left Handed Gun	The Left Handed Gun	Penn, Arthur	Arthur Penn	tt0051849
1769970571	1966	1966	Assignment--Female	Assignment: Female	Phelan, Raymond A.	Raymond Phelan	tt0210549
1746503904	1921	1921	Go Get Him	Go Get Him		Phil Goldstone	tt0359336
1746410576	1987	1987	Suspect	Suspect	Yates, Peter	Peter Yates	tt0094082
1746524143	1923	1923	Desire	Desire	Lee, Rowland V.	Rowland V. Lee	tt0013985
1746578607	1962	1962	The Reluctant Saint	The Reluctant Saint	Dmytryk, Edward	Edward Dmytryk	tt0056403
1746217413	1967	1967	Camelot	Camelot	Logan, Joshua	Joshua Logan	tt0061439
1746564318	1953	1953	Count the Hours	Count the Hours!	Siegel, Don	Don Siegel	tt0045652
1745342017	2002	2001	Frailty	Frailty	Paxton, Bill	Bill Paxton	tt0264616
1746504762	1921	1921	The Hole in the Wall	Hole in the Wall	Karger, Maxwell	Maxwell Karger	tt0012288
1746424062	1941	1941	Mr. District Attorney	Mr. District Attorney	Morgan, William	William Morgan	tt0033923
1746408456	1989	1989	Three Fugitives	Three Fugitives	Veber, Francis	Francis Veber	tt0098471
1746520843	1950	1949	Love Happy	Love Happy	Miller, David	David Miller	tt0041604
1746576981	1951	1951	Savage Drums	Savage Drums	Berke, William	William Berke	tt0043996
1746199209	1969	1969	De Sade	De Sade	Endfield, Cy	Cy Endfield	tt0064212
1746198224	1969	1969	Hell's Belles	Hell's Belles	Dexter, Maury	Maury Dexter	tt0063058
1746520033	1924	1924	The Western Wallop	The Western Wallop	Smith, Clifford	Clifford Smith	tt0015481
1746566985	1934	1934	The Old Fashioned Way	The Old Fashioned Way	Beaudine, William	William Beaudine	tt0025590
1746562320	1956	1956	The Vagabond King	The Vagabond King	Curtiz, Michael	Michael Curtiz	tt0049909
1746574512	1935	1935	The Headline Woman	The Headline Woman	Nigh, William	William Nigh	tt0026458
1746454820	1941	1941	Tight Shoes	Tight Shoes	Rogell, Albert S.	Albert S. Rogell	tt0034293
1746566160	1955	1955	The Second Greatest Sex	The Second Greatest Sex	Marshall, George	George Marshall	tt0048594
1746554412	1963	1963	The Courtship of Eddie's Father	The Courtship of Eddie's Father	Minnelli, Vincente	Vincente Minnelli	tt0056956
1746582721	1936	1936	Nobody's Fool	Nobody's Fool	Collins, Arthur Greville	Arthur Greville Collins	tt0028037
1746574468	1934	1934	Music in the Air	Music in the Air	May, Joe	Joe May	tt0025536
1769977881	1988	1988	Above the Law	Above the Law	Davis, Andrew	Andrew Davis	tt0094602
1746492223	1945	1945	Springtime in Texas	Springtime in Texas	Drake, Oliver	Oliver Drake	tt0163278
1746394759	1993	1993	Alive	Alive	Marshall, Frank	Frank Marshall	tt0106246
1745293303	2006	2006	Inside Man	Inside Man	Lee, Spike	Spike Lee	tt0454848
1746454830	1941	1941	Footlight Fever	Footlight Fever	Reis, Irving	Irving Reis	tt0033615
1746394540	1984	1984	Footloose	Footloose	Ross, Herbert	Herbert Ross	tt0087277
1746533203	1915	1915	Shadows from the Past	Shadows from the Past	Ridgely, Richard J.	Richard Ridgely	tt0006029
1746571496	1931	1931	Arizona	Arizona	Seitz, George B.	George B. Seitz	tt0022134
1745393726	2009	2009	Whip It	Whip It	Barrymore, Drew	Drew Barrymore	tt1172233
1746438961	1940	1940	Triple Justice	Triple Justice	Howard, David	David Howard	tt0033185
1746464867	1944	1944	South of Dixie	South of Dixie	Yarbrough, Jean	Jean Yarbrough	tt0037302
1746500453	1926	1926	The Auction Block	The Auction Block	Henley, Hobart	Hobart Henley	tt0016616
1746512918	1916	1916	Miss Petticoats	Miss Petticoats	Knoles, Harley	Harley Knoles	tt0007079
1746567590	1932	1932	Handle with Care	Handle with Care	Butler, David	David Butler	tt0022974
1746475539	1946	1946	The Devil's Mask	The Devil's Mask	Levin, Henry	Henry Levin	tt0038471
1746232951	1966	1966	Weekend of Fear	Weekend of Fear	Danford, Joe	Joe Danford	tt0229034
1746504182	1922	1923	The Sleepwalker	The Sleepwalker	Le Saint, Edward	Gilbert Pratt	tt0783815
1746503958	1919	1919	Rough Riding Romance	Rough Riding Romance	Rosson, Arthur	Arthur Rosson	tt0010645

1746496814	1919	1919	Miss Arizona	Miss Arizona	Thayer, Otis B.	Otis Thayer	tt0010471	
1746573924	1958	1958	The Space Children	The Space Children	Arnold, Jack	Jack Arnold	tt0052227	
1746554096	1964	1964	Where Love Has Gone	Where Love Has Gone	Dmytryk, Edward	Edward Dmytryk	tt0058745	
1746584637	1930	1930	Common Clay	Common Clay	Fleming, Victor	Victor Fleming	tt0020781	
1746508192	1924	1924	The Law Demands	The Law Demands	Hoyt, Harry O.	Harry O. Hoyt	tt0015055	
1746582382	1950	1950	To Please a Lady	To Please a Lady	Brown, Clarence	Clarence Brown	tt0043052	
1746577019	1951	1951	Whirlwind	Whirlwind	English, John	John English	tt0044210	
1746584484	1930	1930	Playing Around	Playing Around	LeRoy, Mervyn	Mervyn LeRoy	tt0021263	
1746533229	1916	1916	Dimples	Dimples	Jones, Edgar	Edgar Jones	tt0006594	
1745359851	2001	2001	Out Cold	Out Cold	Malloy, Emmett ; Malloy, Brendan	Brendan Malloy	tt0253798	
1746577562	1936	1936	Dancing Pirate	Dancing Pirate	Corrigan, Lloyd	Lloyd Corrigan	tt0027496	
1746198424	1969	1969	Gathering of Evil	Gathering of Evil	Bertini, Victor	Victor Bertini	tt0219006	
1746509503	1924	1924	Restless Wives	Restless Wives	La Cava, Gregory	Gregory La Cava	tt0015271	
1746582693	1937	1937	Woman in Distress	Woman in Distress	Shores, Lynn	Lynn Shores	tt0029792	
1746520266	1926	1926	A Woman's Heart	A Woman's Heart	Rosen, Phil	Phil Rosen	tt0017576	
1746574482	1933	1933	Her Secret	Her Secret	Millais, Warren	Warren Millais	tt0024113	
1746571137	1933	1933	Skyway	Skyway	Collins, Lew	Lewis D. Collins	tt0024573	
1746577344	1960	1960	Who Was That Lady?	Who Was That Lady?	Sidney, George	George Sidney	tt0054473	
1746566328	1955	1955	The Big Knife	The Big Knife	Aldrich, Robert	Robert Aldrich	tt0047880	
1746563887	1953	1953	Big Leaguer	Big Leaguer	Aldrich, Robert	Robert Aldrich	tt0045556	
1746516071	1921	1921	Ducks and Drakes	Ducks and Drakes	Campbell, Maurice	Maurice Campbell	tt0012125	
1746410196	1984	1984	The Flamingo Kid	The Flamingo Kid	Marshall, Garry	Garry Marshall	tt0087265	
1746218088	1967	1967	The Adventures of Bullwhip Griffin	The Adventures of Bullwhip Griffin	Neilson, James	James Neilson	tt0061333	
1746570316	1933	1933	Circle Canyon	Circle Canyon	Adamson, Victor	Victor Adamson	tt0132039	
1746583859	1931	1931	Man from Death Valley	The Man from Death Valley	Nosler, Lloyd	Lloyd Nosler	tt0022116	
1746578595	1961	1961	On the Double	On the Double	Shavelson, Melville	Melville Shavelson	tt0055253	
1746504621	1922	1922	Blue Blazes	Blue Blazes	Kelly, Robert; Mack, Charles W.	Robert Kelly	tt0012953	
1769976193	1921	1922	Holding His Own	Holding His Own	Fahrney, Milton	Milton J. Fahrney	tt5547700	
1746577558	1935	1935	Carnival	Carnival	Lang, Walter	Walter Lang	tt0026181	
1746218187	1966	1966	The Black Cat	The Black Cat	Hoffman, Harold	Harold Hoffman	tt0060171	
1746409280	1984	1984	Broadway Danny Rose	Broadway Danny Rose	Allen, Woody	Woody Allen	tt0087003	
1746208346	1969	1969	The Monitors	The Monitors	Shea, Jack	Jack Shea	tt0064684	
1746499912	1945	1945	The Man Who Walked Alone	The Man Who Walked Alone	Cabanne, Christy	Christy Cabanne	tt0037893	
1746393524	1988	1988	Mac and Me	Mac and Me	Raffill, Stewart	Stewart Raffill	tt0095560	
1746575033	1934	1934	Strange Wives	Strange Wives	Thorpe, Richard	Richard Thorpe	tt0027051	
1746566319	1958	1958	A Certain Smile	A Certain Smile	Negulesco, Jean	Jean Negulesco	tt0051466	
1746493426	1910	1910	One Night, and Then ---	One Night, and Then	Griffith, D. W.	D.W. Griffith	tt0001346	
1746419170	1994	1994	The Client	The Client	Schumacher, Joel	Joel Schumacher	tt0109446	
1746561998	1954	1953	Rob Roy the Highland Rogue	Rob Roy: The Highland Rogue	French, Harold	Harold French	tt0046246	
1746524221	1915	1915	The Goose Girl	The Goose Girl	Thomson, Frederick	Frederick A. Thomson	tt0005405	
1746578754	1962	1961	Swingin' Along	Swingin' Along	Barton, Charles	Charles Barton	tt0056543	
1746219543	1972	1972	Prime Cut	Prime Cut	Ritchie, Michael	Michael Ritchie	tt0069121	
1746421626	1991	1991	Curly Sue	Curly Sue	Hughes, John	John Hughes	tt0101635	
1746501064	1914	1914	The Greyhound	The Greyhound	McGill, Lawrence	Lawrence B. McGill	tt0004043	
1746584022	1935	1935	Tombstone Terror	Tombstone Terror	Bradbury, Robert N.	Robert N. Bradbury	tt0027120	
1746438949	1941	1941	Silver Stallion	Silver Stallion	Finney, Edward	Edward Finney	tt0034189	
1746569572	1957	1957	Man in the Shadow	Man in the Shadow	Arnold, Jack	Jack Arnold	tt0050680	
1746583629	1930	1930	The Dawn Trail	The Dawn Trail	Cabanne, Christy	Christy Cabanne	tt0020816	
1746410007	1992	1992	The Hand That Rocks the Cradle	The Hand That Rocks the Cradle	Hanson, Curtis	Curtis Hanson	tt0104389	
1746420743	1991	1991	Eve of Destruction	Eve of Destruction	Gibbins, Duncan	Duncan Gibbins	tt0101831	
1746583891	1931	1931	Smart Money	Smart Money	Green, Alfred E.	Alfred E. Green	tt0022403	
1746582409	1936	1936	Murder with Pictures	Murder with Pictures	Barton, Charles	Charles Barton	tt0028004	
1746581865	1929	1929	The Amazing Vagabond	The Amazing Vagabond	Fox, Wallace W.	Wallace Fox	tt0019636	
1746500331	1926	1925	Hidden Loot	Hidden Loot	Bradbury, Robert North	Robert N. Bradbury	tt0015910	
1746409932	1986	1986	Half Moon Street	Half Moon Street	Swaim, Bob	Bob Swaim	tt0091164	
**1746454023**	**1941**	**1941**	**Hellzapoppin'**	**Hellzapoppin'**	**Potter, H. C.**	**H.C. Potter**	**tt0033704**	1
1746514541	1914	1914	What's His Name	What's His Name	DeMille, Cecil B.	Cecil B. DeMille	tt0004792	

1746508677	1926	1926	Brown of Harvard	Brown of Harvard	Conway, Jack	Jack Conway	tt0016690
1746491822	1948	1948	Mary Lou	Mary Lou	Dreifuss, Arthur	Arthur Dreifuss	tt0039613
1746574427	1962	1962	The Music Man	The Music Man	DaCosta, Morton	Morton DaCosta	tt0056262
1746583170	1934	1934	Ladies Should Listen	Ladies Should Listen	Tuttle, Frank	Frank Tuttle	tt0025363
1746503038	1910	1910	A Quiet Boarding House	A Quiet Boarding House		Tom Ricketts	tt1644501
1746582064	1930	1930	Personality	Personality	Heerman, Victor	Victor Heerman	tt0021245
1746570006	1959	1959	The Sound and the Fury	The Sound and the Fury	Ritt, Martin	Martin Ritt	tt0053298
1746520591	1925	1925	Desert Madness	Desert Madness	Webb, Harry	Harry S. Webb	tt0298831
1746503487	1918	1918	The Brazen Beauty	The Brazen Beauty	Browning, Tod	Tod Browning	tt0009963
1746532654	1915	1915	Greater Love Hath No Man	Greater Love Hath No Man	Blaché, Herbert	Herbert Blaché	tt0005423
1746580944	1960	1960	High Time	High Time	Edwards, Blake	Blake Edwards	tt0053912
1746503391	1918	1918	The Life Mask	The Life Mask	Crane, Frank	Frank Hall Crane	tt0009297
1746409298	1984	1984	Iceman	Iceman	Schepisi, Fred	Fred Schepisi	tt0087452
1746498665	1917	1917	Raffles, the Amateur Cracksman	Raffles, the Amateur Cracksman	Irving, George	George Irving	tt0008489
1746199014	1969	1969	More Dead Than Alive	More Dead Than Alive	Sparr, Robert	Robert Sparr	tt0064695
1746555090	1957	1957	Last of the Bad Men	Last of the Badmen	Landres, Paul	Paul Landres	tt0050625
1746496270	1919	1919	Modern Husbands	Modern Husbands	Grandon, Francis J.	Francis J. Grandon	tt0010473
1746503522	1921	1921	Cheated Love	Cheated Love	Baggot, King	King Baggot	tt0012040
1746393998	1982	1981	One from the Heart	One from the Heart	Coppola, Francis	Francis Ford Coppola	tt0084445
1746577831	1935	1935	Hot off the Press	Hot Off the Press	Herman, Al	Albert Herman	tt0026495
1746487903	1917	1917	Sunny Jane	Sunny Jane		Sherwood MacDonald	tt0008641
1746568219	1932	1932	Strangers in Love	Strangers in Love	Mendes, Lothar	Lothar Mendes	tt0023527
1746582749	1937	1937	Come On, Cowboys!	Come on, Cowboys	Kane, Joseph	Joseph Kane	tt0028732
1746520989	1949	1949	Gun Runner	Gun Runner	Hillyer, Lambert	Lambert Hillyer	tt0041440
1746465129	1942	1942	Across the Pacific	Across the Pacific	Huston, John; Sherman, Vincent	John Huston	tt0034428
1746578814	1961	1961	The Happy Thieves	The Happy Thieves	Marshall, George	George Marshall	tt0056057
1746513725	1915	1915	The Man from Oregon	The Man from Oregon	Barker, Reginald	Reginald Barker	tt0005702
1746585124	1929	1928	Red Hot Speed	Red Hot Speed	Henabery, Joseph E.	Joseph Henabery	tt0020316
1746235340	1974	1974	Freebie and the Bean	Freebie and the Bean	Rush, Richard	Richard Rush	tt0071521
1746508912	1925	1925	Ship of Souls	Ship of Souls	Miller, Charles	Charles Miller	tt0016343
1746562272	1953	1953	Marry Me Again	Marry Me Again	Tashlin, Frank	Frank Tashlin	tt0046048
1745359690	2004	2004	Ray	Troy	Hackford, Taylor	Wolfgang Petersen	tt0332452
1746520255	1924	1924	Ridin' West	Ridin' West	Webb, Harry	Harry S. Webb	tt0299113
1746555513	1955	1955	The Court-Martial of Billy Mitchell	The Court Martial of Billy Mitchell	Preminger, Otto	Otto Preminger	tt0047956
1746573784	1958	1958	The Law and Jake Wade	The Law and Jake Wade	Sturges, John	John Sturges	tt0051848
1745302498	2005	2005	House of Wax	House of Wax	Collet-Serra, Jaume	Jaume Collet	tt0397065
1746499985	1948	1948	A Southern Yankee	A Southern Yankee	Sedgwick, Edward; Simon, S. Sylvan	Edward Sedgwick	tt0040825
1746474506	1944	1944	Beautiful But Broke	Beautiful But Broke	Barton, Charles	Charles Barton	tt0036633
1746394218	1988	1988	Scrooged	Scrooged	Donner, Richard	Richard Donner	tt0096061
1746577461	1961	1960	The Sinister Urge	The Sinister Urge	Wood, Edward Davis	Edward D. Wood Jr.	tt0055452
1746487139	1919	1919	The Unbroken Promise	The Unbroken Promise	Powell, Frank	Frank Powell	tt0194486
1746491826	1947	1947	Framed	Framed	Wallace, Richard	Richard Wallace	tt0039396
1769971952	1969	1968	Desire Under the Palms	Desire Under the Palms	Sarno, Joseph W.	Joseph W. Sarno	tt0062876
1745293991	2007	2007	National Treasure: Book of Secrets	National Treasure: Book of Secrets	Turteltaub, Jon	Jon Turteltaub	tt0465234
1746453825	1944	1944	Voice in the Wind	Voice in the Wind	Ripley, Arthur	Arthur Ripley	tt0037438
1746454186	1996	1996	Sleepers	Sleepers	Levinson, Barry	Barry Levinson	tt0117665
1746524876	1928	1928	Must We Marry?	Must We Marry?	Mattison, Frank S.	Frank S. Mattison	tt0019190
1746497651	1921	1921	The Butterfly Girl	The Butterfly Girl	Gorman, John	John Gorman	tt0012018
1746515827	1920	1920	Dollar for Dollar	Dollar for Dollar	Keenan, Frank	Frank Keenan	tt0150365
1746534804	1921	1921	Bare Knuckles	Bare Knuckles	Hogan, James P.	James P. Hogan	tt0011945
1746584549	1928	1928	The Avenging Shadow	The Avenging Shadow	Taylor, Ray	Ray Taylor	tt0018664
1745374739	2008	2008	One Missed Call	One Missed Call	Valette, Eric	Eric Valette	tt0479968
1746573797	1959	1959	Blood and Steel	Blood and Steel	Kowalski, Bernard L.	Bernard L. Kowalski	tt0052636
1745359710	2002	2002	The Master of Disguise	The Master of Disguise	Blake, Perry Andelin	Perry Andelin Blake	tt0295427
1745357928	2002	2002	John Q.	John Q	Cassavetes, Nick	Nick Cassavetes	tt0251160
1746208316	1968	1968	The Secret War of Harry Frigg	The Secret War of Harry Frigg	Smight, Jack	Jack Smight	tt0063573
1746473916	1946	1946	The Runaround	The Runaround	Lamont, Charles	Charles Lamont	tt0038894

1746584209	1935	1935	She Couldn't Take It	She Couldn't Take It	Garnett, Tay	Tay Garnett	tt0026984
1746584910	1939	1939	The Phantom Stage	The Phantom Stage	Waggner, George	George Waggner	tt0031797
1746508716	1924	1924	The Tomboy	The Tomboy	Kirkland, David	David Kirkland	tt0015419
1769971919	1969	1969	Sex Family Robinson on the Farm	Sex Family Robinson on the Farm	Vair, Linda	Linda Vair	tt0257142
1746454257	1941	1941	Blonde Inspiration	Blonde Inspiration	Berkeley, Busby	Busby Berkeley	tt0033400
1746439481	1945	1945	Beyond the Pecos	Beyond the Pecos	Hillyer, Lambert	Lambert Hillyer	tt0169607
1746584793	1930	1930	The Big Party	The Big Party	Blystone, John	John G. Blystone	tt0020689
1746491789	1945	1945	Arson Squad	Arson Squad	Landers, Lew	Lew Landers	tt0037521
1746487054	1921	1921	The Barricade	The Barricade	Cabanne, William Christy	Christy Cabanne	tt0011946
1746492093	1946	1946	Cluny Brown	Cluny Brown	Lubitsch, Ernst	Ernst Lubitsch	tt0038419
1746424583	1941	1941	Ellery Queen and the Perfect Crime	Ellery Queen and the Perfect Crime	Hogan, James	James P. Hogan	tt0033573
1746465087	1942	1942	West of the Law	West of the Law	Bretherton, Howard P.	Howard Bretherton	tt0035543
1746532497	1922	1922	Broad Daylight	Broad Daylight	Cummings, Irving	Irving Cummings	tt0012976
1746474542	1948	1948	Easter Parade	Easter Parade	Walters, Charles	Charles Walters	tt0040308
1746577034	1961	1961	The Guns of Navarone	The Guns of Navarone	Thompson, J. Lee	J. Lee Thompson	tt0054953
1769970432	1965	1964	The Thrill Killer	The Thrill Killers		Ray Dennis Steckler	tt0058653
1746583372	1950	1950	With These Hands	With These Hands	Arnold, Jack	Jack Arnold	tt0043138
1746582706	1938	1938	Gone Harlem	Gone Harlem		Irwin Franklyn	tt0174701
1746434335	1999	1999	At First Sight	At First Sight	Winkler, Irwin	Irwin Winkler	tt0132512
1746434615	1992	1992	Swoon	Swoon	Kalin, Tom	Tom Kalin	tt0105508
1746492438	1945	1945	Frontier Feud	Frontier Feud	Hillyer, Lambert	Lambert Hillyer	tt0037719
1746487334	1920	1920	The Discarded Woman	The Discarded Woman	King, Burton L.	Burton L. King	tt0193881
1746421714	1999	1998	God Said Ha!	God Said, 'Ha!'	Sweeney, Julia	Julia Sweeney	tt0119207
1746567599	1933	1933	Broadway to Hollywood	Broadway to Hollywood	Mack, Willard	Willard Mack	tt0023848
1746566067	1957	1957	Pal Joey	Pal Joey	Sidney, George	George Sidney	tt0050815
1746583591	1951	1951	Yellow Fin	Yellow Fin	McDonald, Frank	Frank McDonald	tt0044221
1745339880	2004	2004	De-Lovely	De Lovely	Winkler, Irwin	Irwin Winkler	tt0352277
1746520418	1925	1925	Galloping Jinx	The Galloping Jinx	Eddy, Robert	Robert Eddy	tt0015851
1746577745	1935	1935	It's in the Air	It's in the Air	Riesner, Charles F.	Charles Reisner	tt0026540
1746567241	1932	1932	Back Street	Back Street	Stahl, John M.	John M. Stahl	tt0022651
1746533000	1916	1916	Going Straight	Going Straight	Franklin, C. M.; Franklin, S. A.	Chester M. Franklin	tt0006731
1746394209	1992	1992	Honeymoon in Vegas	Honeymoon in Vegas	Bergman, Andrew	Andrew Bergman	tt0104438
1746574410	1962	1962	Secret File: Hollywood	Secret File: Hollywood	Cushman, Ralph	Rudolph Cusumano	tt0056460
1746566299	1957	1957	Beginning of the End	Beginning of the End	Gordon, Bert I.	Bert I. Gordon	tt0050177
1746577640	1937	1937	The Westland Case	The Westland Case	Cabanne, Christy	Christy Cabanne	tt0029757
1746524872	1915	1915	The Master Hand	The Master Hand	Knoles, Harley	Harley Knoles	tt0141587
1746577492	1951	1951	The Whip Hand	The Whip Hand	Menzies, William Cameron	William Cameron Menzies	tt0044209
1746524900	1915	1915	Sin	The Son	Brenon, Herbert	Percy Winter	tt0284502
1746585140	1928	1928	The Circus	The Circus	Chaplin, Charles	Charles Chaplin	tt0018773
1746553433	1965	1965	Curse of the Stone Hand	Curse of the Stone Hand	Warren, Jerry; Schleipper, Carl	Carlos Hugo Christensen	tt0057987
1746584377	1937	1937	Her Husband Lies	Her Husband Lies	Ludwig, Edward	Edward Ludwig	tt0028992
1746464868	1945	1945	Oregon Trail	Oregon Trail	Carr, Thomas	Thomas Carr	tt0037961
1745357705	2002	2002	I Am Trying to Break Your Heart	I Am Trying to Break Your Heart	Jones, Sam	Sam Jones	tt0327920
1746565911	1957	1957	Shoot-Out at Medicine Bend	Shoot Out at Medicine Bend	Bare, Richard L.	Richard L. Bare	tt0050963
1746520479	1925	1926	The Return of Grey Wolf	The Return of Grey Wolf	Rollens, Jacques	Jack Rollens	tt0137192
1746496055	1919	1919	The Rider of the Law	Rider of the Law	Ford, Jack	John Ford	tt0010628
1746514866	1915	1915	The Silent Command	The Silent Command	Leonard, Robert	Robert Z. Leonard	tt0006042
1746435429	1996	1996	Faithful	Faithful	Mazursky, Paul	Paul Mazursky	tt0116269
1746581397	1962	1962	The Savage Guns	The Savage Guns	Carreras, Michael	Michael Carreras	tt0056448
1746408916	1982	1982	Making Love	Making Love	Hiller, Arthur	Arthur Hiller	tt0084293
1746584911	1939	1939	Nancy Drew and the Hidden Staircase	Nancy Drew and the Hidden Staircase	Clemens, William	William Clemens	tt0031708
1746577789	1935	1935	Social Error	Social Error	Fraser, Harry	Harry L. Fraser	tt0183796
1746582395	1931	1931	Everything's Rosie	Everything's Rosie	Bruckman, Clyde	Clyde Bruckman	tt0021840
1745340327	2002	2001	Elvira's Haunted Hills	Elvira's Haunted Hills	Irvin, Sam	Sam Irvin	tt0265171
1745293820	2007	2007	Next	Next	Tamahori, Lee	Lee Tamahori	tt0435705
1745293372	2007	2007	Before the Devil Knows You're Dead	Before the Devil Knows You're Dead	Lumet, Sidney	Sidney Lumet	tt0292963
1746520350	1925	1925	Ten Days	Ten Days	Worne, Duke	Duke Worne	tt0168218

1746582985	1936	1936	Country Gentlemen	Country Gentlemen	Staub, Ralph	Ralph Staub	tt0027470
1746575079	1938	1938	City Girl	City Girl	Werker, Alfred	Alfred L. Werker	tt0028721
1746574954	1938	1938	Having Wonderful Time	Having Wonderful Time	Santell, Alfred	Alfred Santell	tt0030216
1746454081	1943	1943	Corregidor	Corregidor	Nigh, William	William Nigh	tt0035755
1746583395	1930	1930	Free and Easy	Free and Easy	Sedgwick, Edward	Edward Sedgwick	tt0020902
1746509557	1927	1927	The Potters	The Potters	Newmeyer, Fred	Fred C. Newmeyer	tt0018274
1746393776	1987	1987	Sign O' the Times	Sign 'o' the Times	Prince	Prince	tt0093970
1746424414	1942	1942	Calling Dr. Gillespie	Calling Dr. Gillespie	Bucquet, Harold S.	Harold S. Bucquet	tt0034570
1746491809	1945	1945	It's in the Bag!	It's in the Bag!	Wallace, Richard	Richard Wallace	tt0037823
1746421352	1992	1991	Shakes the Clown	Shakes the Clown	Goldthwait, Bobcat	Bobcat Goldthwait	tt0102898
1746576909	1958	1958	St. Louis Blues	St. Louis Blues	Reisner, Allen	Allen Reisner	tt0052234
1746436336	1943	1943	Five Graves to Cairo	Five Graves to Cairo	Wilder, Billy	Billy Wilder	tt0035884
1746565884	1957	1957	Gun for a Coward	Gun for a Coward	Biberman, Abner	Abner Biberman	tt0049285
1746520631	1949	1949	Holiday Affair	Holiday Affair	Hartman, Don	Don Hartman	tt0041473
1745372544	2009	2009	I Love You, Beth Cooper	I Love You, Beth Cooper	Columbus, Chris	Chris Columbus	tt1032815
1746572555	1932	1932	Hypnotized	Hypnotized	Sennett, Mack	Mack Sennett	tt0023040
1746465133	1946	1946	Swing Parade of 1946	Swing Parade of 1946	Karlson, Phil	Phil Karlson	tt0039003
1746578797	1963	1963	The Checkered Flag	The Checkered Flag	Grefé, William	William Grefé	tt0055843
1746582476	1935	1935	Charlie Chan in Shanghai	Charlie Chan in Shanghai	Tinling, James	James Tinling	tt0026199
1746582519	1935	1935	Another Face	Another Face	Cabanne, Christy	Christy Cabanne	tt0026075
1746503053	1916	1916	Hulda from Holland	Hulda from Holland	O'Brien, John B.	John B. O'Brien	tt0006831
1746409751	1988	1988	Arthur 2 On the Rocks	Arthur 2: On the Rocks	Yorkin, Bud	Bud Yorkin	tt0094678
1746572187	1932	1932	Young Bride	Young Bride	Seiter, William	William A. Seiter	tt0023720
1746555841	1955	1955	Jump into Hell	Jump Into Hell	Butler, David	David Butler	tt0048235
1746435839	1996	1996	Up Close and Personal	Up Close & Personal	Avnet, Jon	Jon Avnet	tt0118055
1746199217	1965	1965	The Rounders	The Rounders	Kennedy, Burt	Burt Kennedy	tt0059661
1746572352	1932	1932	Horse Feathers	Horse Feathers	McLeod, Norman	Norman Z. McLeod	tt0023027
1746574494	1933	1934	Public Stenographer	Public Stenographer	Collins, Lew	Lewis D. Collins	tt0025687
1746577509	1935	1935	The Gay Deception	The Gay Deception	Wyler, William	William Wyler	tt0026400
1746439223	1997	1997	Speed 2: Cruise Control	Speed 2: Cruise Control	de Bont, Jan	Jan de Bont	tt0120179
1745293780	2007	2007	Music Within	Music Within	Sawalich, Steven	Steven Sawalich	tt0422783
1746570125	1933	1933	The Phantom Broadcast	The Phantom Broadcast	Rosen, Phil	Phil Rosen	tt0024443
1746500915	1914	1914	The Path Forbidden	The Path Forbidden	Handworth, Harry	Harry Handworth	tt0004458
1746473738	1944	1944	Gaslight	Gaslight	Cukor, George	George Cukor	tt0036855
1746580259	1935	1935	People Will Talk	People Will Talk	Santell, Alfred	Alfred Santell	tt0026860
1746218252	1965	1965	The Collector	The Collector	Wyler, William	William Wyler	tt0059043
1746580244	1934	1934	I Give My Love	I Give My Love	Freund, Karl	Karl Freund	tt0025281
1746584359	1940	1940	Boom Town	Boom Town	Conway, Jack	Jack Conway	tt0032273
1746217463	1966	1966	The Night of the Grizzly	The Night of the Grizzly	Pevney, Joseph	Joseph Pevney	tt0060754
1746555357	1957	1957	The Crooked Circle	The Crooked Circle	Kane, Joe	Joseph Kane	tt0044520
1746574568	1952	1952	Yankee Buccaneer	Yankee Buccaneer	De Cordova, Frederick	Frederick De Cordova	tt0045343
1746581455	1951	1951	Texas Carnival	Texas Carnival	Walters, Charles	Charles Walters	tt0044117
1746585473	1940	1940	The Shop Around the Corner	The Shop Around the Corner	Lubitsch, Ernst	Ernst Lubitsch	tt0033045
1746580346	1935	1935	The Shadow of Silk Lennox	The Shadow of Silk Lennox	Kirkwood, Ray	Ray Kirkwood	tt0026981
1746582858	1938	1938	You and Me	You and Me	Lang, Fritz	Fritz Lang	tt0030996
1745360012	2002	2001	Who Is Cletis Tout?	Who Is Cletis Tout?	Ver Wiel, Chris	Chris Ver Wiel	tt0246500
1745342039	2008	2008	Tropic Thunder	Tropic Thunder	Stiller, Ben	Ben Stiller	tt0942385
1746584667	1928	1928	The Bullet Mark	The Bullet Mark	Paton, Stuart	Stuart Paton	tt0018731
1746571245	1934	1934	The Curtain Falls	The Curtain Falls	Lamont, Charles	Charles Lamont	tt0025024
1746567436	1952	1952	Monsoon	Monsoon	Amateau, Rodney	Rod Amateau	tt0046090
1746570410	1934	1934	The Dude Ranger	The Dude Ranger	Cline, Edward F.	Edward F. Cline	tt0025073
1746555015	1964	1964	Taggart	Taggart	Springsteen, R. G.	R.G. Springsteen	tt0058633
1746465053	1942	1942	This Gun for Hire	This Gun for Hire	Tuttle, Frank	Frank Tuttle	tt0035432
1746570089	1934	1935	Convention Girl	Convention Girl	Reed, Luther	Luther Reed	tt0026232
1746583189	1929	1929	Dance Hall	Dance Hall	Brown, Melville	Melville W. Brown	tt0019797
1746186060	1966	1966	Don't Worry, We'll Think of a Title	Don't Worry, We'll Think of a Title	Jones, Harmon	Harmon Jones	tt0060332
1746455146	1998	1998	Enemy of the State	Enemy of the State	Scott, Tony	Tony Scott	tt0120660

1746582467	1939	1939	Another Thin Man	Another Thin Man	Van Dyke, W. S.	W.S. Van Dyke	tt0031047
1746574082	1959	1958	Missile to the Moon	Missile to the Moon	Cunha, Richard	Richard E. Cunha	tt0053072
1746492419	1946	1946	The Return of Rusty	The Return of Rusty	Castle, William	William Castle	tt0299112
1746424434	1943	1943	Happy Go Lucky	Happy Go Lucky	Bernhardt, Curtis	Curtis Bernhardt	tt0035969
1746473720	1942	1942	American Empire	American Empire	McGann, William	William C. McGann	tt0034455
1746584493	1931	1931	Young Sinners	Young Sinners	Blystone, John	John G. Blystone	tt0022590
1746583590	1950	1950	Vendetta	Vendetta	Ferrer, Mel; Opuls, Max; Sturges, Pre	Mel Ferrer	tt0043100
1746580387	1937	1937	Law of the Ranger	Law of the Ranger	Bennett, Spencer Gordon	Spencer Gordon Bennet	tt0029124
1769970578	1968	1968	The Gay Life	The Gay Life	Foley, John	John Foley	tt0219016
1746438935	1941	1941	Cyclone on Horseback	Cyclone on Horseback	Killy, Edward	Edward Killy	tt0033505
1746474813	1947	1947	Dark Passage	Dark Passage	Daves, Delmer	Delmer Daves	tt0039302
1746566921	1933	1933	Mary Stevens, M.D.	Mary Stevens, M.D.	Bacon, Lloyd	Lloyd Bacon	tt0024307
1746553995	1965	1965	Red Line 7000	Red Line 7000	Hawks, Howard	Howard Hawks	tt0059641
1746410125	1991	1991	Cape Fear	Cape Fear	Scorsese, Martin	Martin Scorsese	tt0101540
1746249060	1976	1976	The Killer Inside Me	The Killer Inside Me	Kennedy, Burt	Burt Kennedy	tt0074746
1746524483	1927	1927	Spoilers of the West	Spoilers of the West	Van Dyke, W. S.	W.S. Van Dyke	tt0018438
1746438694	1996	1996	House Arrest	House Arrest	Winer, Harry	Harry Winer	tt0116571
1746435701	1941	1941	Her First Beau	Her First Beau	Reed, Theodore	Theodore Reed	tt0033709
1769970672	1968	1968	Come Play With Me	Come Play with Me	Cosentino, Nick	Nicolas Cosentino	tt0215643
1746585264	1929	1929	Little Johnny Jones	Little Johnny Jones	LeRoy, Mervyn	Mervyn LeRoy	tt0020101
1746582980	1935	1935	Curly Top	Curly Top	Cummings, Irving	Irving Cummings	tt0026252
1746437926	1944	1944	Marked Trails	Marked Trails	McCarthy, J. P.	John P. McCarthy	tt0037050
1746566221	1958	1958	I Bury the Living	I Bury the Living	Band, Albert	Albert Band	tt0051755
1746393963	1990	1990	Dances with Wolves	Dances with Wolves	Costner, Kevin	Kevin Costner	tt0099348
1746583617	1930	1930	The Fourth Alarm	The Fourth Alarm	Whitman, Phil	Phil Whitman	tt0132982
1746487915	1917	1916	The Burglar	The Burglar	Knoles, Harley	Pat Hartigan	tt0321552
1746569865	1959	1959	Anatomy of a Murder	Anatomy of a Murder	Preminger, Otto	Otto Preminger	tt0052561
1746515300	1922	1922	Too Much Wife	Too Much Wife	Heffron, Thomas N.	Thomas N. Heffron	tt0013693
1746566856	1933	1933	Justice Takes a Holiday	Justice Takes a Holiday	Bennet, Spencer Gordon	Spencer Gordon Bennet	tt0024201
1746554221	1964	1964	Fail Safe	Fail Safe	Lumet, Sidney	Sidney Lumet	tt0058083
1746186097	1966	1965	Son of a Gunfighter	Son of a Gunfighter	Landres, Paul	Paul Landres	tt0059737
1746566219	1958	1958	The Blob	The Blob	Yeaworth, Irvin S.	Irvin S. Yeaworth Jr.	tt0051418
1746520846	1949	1949	Ride, Ryder, Ride!	Ride, Ryder, Ride!	Collins, Lewis D.	Lewis D. Collins	tt0180098
1746501010	1916	1916	The Sable Blessing	The Sable Blessing	Sargent, George L.	George L. Sargent	tt0158907
1746464878	1942	1942	Arabian Nights	Arabian Nights	Rawlins, John	John Rawlins	tt0034465
1746435161	1942	1941	Invasion	Invasion		Bud Pollard	tt1417584
1745358083	2010	2010	Wall Street: Money Never Sleeps	Wall Street: Money Never Sleeps	Stone, Oliver	Oliver Stone	tt1027718
1746423273	1995	1995	Mighty Aphrodite	Mighty Aphrodite	Allen, Woody	Woody Allen	tt0113819
1745357707	2008	2008	The Secret Life of Bees	The Secret Life of Bees	Prince-Bythewood, Gina	Gina Prince	tt0416212
1746474113	1945	1945	Tarzan and the Amazons	Tarzan and the Amazons	Neumann, Kurt	Kurt Neumann	tt0038151
1746185228	1965	1965	One Way Wahine	One Way Wahine	Brown, William O.	William O. Brown	tt0059543
1746496999	1922	1922	Rose o' the Sea	Rose o' the Sea	Niblo, Fred	Fred Niblo	tt0013560
1746504149	1918	1918	Under Suspicion	Under Suspicion	Davis, Will S.	Will S. Davis	tt0009743
1746563917	1951	1951	Tomahawk	Tomahawk	Sherman, George	George Sherman	tt0044135
1746216793	1967	1967	The Last Challenge	The Last Challenge	Thorpe, Richard	Richard Thorpe	tt0061893
1746582193	1928	1928	The Flyin' Cowboy	The Flyin' Cowboy	Eason, Reaves	B. Reeves Eason	tt0018901
1746394549	1984	1984	Revenge of the Nerds	Revenge of the Nerds	Kanew, Jeff	Jeff Kanew	tt0088000
1746509482	1925	1925	Stella Maris	Stella Maris	Brabin, Charles J.	Charles Brabin	tt0016396
1746453688	1999	1999	The Rage: Carrie 2	The Rage: Carrie 2	Shea, Katt	Katt Shea	tt0144814
1746571841	1932	1932	Unashamed	Unashamed	Beaumont, Harry	Harry Beaumont	tt0023635
1746565479	1952	1952	And Now Tomorrow	And Now Tomorrow	Watson, William	William Watson	tt0044354
1746473926	1945	1945	The Power of the Whistler	The Power of the Whistler	Landers, Lew	Lew Landers	tt0037997
1746584788	1930	1930	Paid	Paid		Sam Wood	tt0021228
1746582773	1938	1938	Blondie!	Blondie	Strayer, Frank R.	Frank R. Strayer	tt0029927
1746487479	1918	1917	The Goat	The Goat	Crisp, Donald	Arvid E. Gillstrom	tt0269282
1746409970	1984	1984	Conan the Destroyer	Conan the Destroyer	Fleischer, Richard	Richard Fleischer	tt0087078
1746577264	1950	1950	Deported	Deported	Siodmak, Robert	Robert Siodmak	tt0042387

1746409136	1986	1986	Quicksilver	Quicksilver	Donnelly, Tom	Thomas Michael Donnelly	tt0091814
1746500572	1924	1924	Daughters of Pleasure	Daughters of Pleasure	Beaudine, William	William Beaudine	tt0014833
1746577801	1935	1935	Mad Love	Mad Love	Freund, Karl	Karl Freund	tt0026663
1746456055	1997	1997	Absolute Power	Absolute Power	Eastwood, Clint	Clint Eastwood	tt0118548
1746520128	1925	1925	Flying Fool	The Flying Fool	Mattison, Frank S.	Lewis Seiler	tt0450709
1746585360	1939	1939	Mr. Smith Goes to Washington	Mr. Smith Goes to Washington	Capra, Frank	Frank Capra	tt0031679
1746582972	1935	1935	Speed Devils	Speed Devils	Henabery, Joseph	Joseph Henabery	tt0027030
1746585255	1930	1930	Lovin' the Ladies	Lovin' the Ladies	Brown, Melville	Melville W. Brown	tt0021102
1746492735	1948	1948	Port Said	Port Said	LeBorg, Reginald	Reginald Le Borg	tt0040702
1746585248	1929	1929	The Return of Sherlock Holmes	The Return of Sherlock Holmes	Dean, Basil	Basil Dean	tt0020324
1746199829	1969	1969	Wild Gypsies	Wild Gypsies	Ray, Marc B.	Marc B. Ray	tt0192796
1746475250	1948	1948	The Checkered Coat	The Checkered Coat	Cahn, Edward L.	Edward L. Cahn	tt0040228
1746579066	1962	1962	Big Red	Big Red	Tokar, Norman	Norman Tokar	tt0055793
1745375290	2009	2008	Goodbye Solo	Goodbye Solo	Bahrani, Ramin	Ramin Bahrani	tt1095442
1746515056	1927	1927	When a Man Loves	When a Man Loves	Crosland, Alan	Alan Crosland	tt0018566
1746525088	1919	1919	Blind Husbands	Blind Husbands	Stroheim, Erich	Erich von Stroheim	tt0009937
1746502927	1914	1914	Without Hope	Without Hope	Mace, Fred	Fred Mace	tt0004828
1746218782	1977	1977	Kingdom of the Spiders	Kingdom of the Spiders	Cardos, John "Bud"	John 'Bud' Cardos	tt0076271
1746520503	1925	1925	The Calgary Stampede	The Calgary Stampede	Blaché, Herbert	Herbert Blaché	tt0015662
1746393503	1984	1984	Body Double	Body Double	De Palma, Brian	Brian De Palma	tt0086984
1746554005	1964	1964	How To Succeed With Girls	How to Succeed with Girls	Biery, Edward A.	Edward A. Biery	tt0150987
1746474520	1948	1948	The Boy with Green Hair	The Boy with Green Hair	Losey, Joseph	Joseph Losey	tt0040185
1746512596	1915	1915	The Lily and the Rose	The Lily and the Rose	Powell, Paul	Paul Powell	tt0005621
1746563511	1952	1951	Chicago Calling	Chicago Calling	Reinhardt, John	John Reinhardt	tt0043401
1746570717	1952	1951	A Streetcar Named Desire	A Streetcar Named Desire	Kazan, Elia	Elia Kazan	tt0044081
1746454811	1940	1940	Legion of the Lawless	Legion of the Lawless	Howard, David	David Howard	tt0032696
1746508002	1924	1924	The Desert Sheik	The Desert Sheik	Terriss, Tom	Tom Terriss	tt0014844
1746520626	1949	1949	Adventure in Baltimore	Adventure in Baltimore	Wallace, Richard	Richard Wallace	tt0041093
1746574664	1933	1933	Men Must Fight	Men Must Fight	Selwyn, Edgar	Edgar Selwyn	tt0024325
1745339912	2001	2001	The Others	The Others	Amenábar, Alejandro	Alejandro Amenábar	tt0230600
1746577455	1960	1960	Kidnapped	Kidnapped	Stevenson, Robert	Robert Stevenson	tt0053994
1746453706	2000	2000	The Ladies Man	The Ladies Man	Hudlin, Reginald	Reginald Hudlin	tt0213790
1746420886	1994	1994	It Could Happen to You	It Could Happen to You	Bergman, Andrew	Andrew Bergman	tt0110167
1746520962	1949	1949	Red Stallion in the Rockies	Red Stallion in the Rockies	Murphy, Ralph	Ralph Murphy	tt0041794
1746508036	1925	1925	Fifth Avenue Models	Fifth Avenue Models	Gade, Svend	Svend Gade	tt0015803
1746508921	1926	1926	Hearts and Spangles	Hearts and Spangles	O'Connor, Frank	Frank O'Connor	tt0134710
1746583215	1930	1930	The Climax	The Climax	Hoffman, Renaud	Renaud Hoffman	tt0020771
1746582424	1939	1939	Bulldog Drummond's Bride	Bulldog Drummond's Bride	Hogan, James	James P. Hogan	tt0031124
1746569962	1958	1958	War of the Colossal Beast	War of the Colossal Beast	Gordon, Bert I.	Bert I. Gordon	tt0052378
1745272030	2001	2001	All Over the Guy	All Over the Guy	Davis, Julie	Julie Davis	tt0250202
1746520924	1950	1950	The Great Rupert	The Great Rupert	Pichel, Irving	Irving Pichel	tt0042524
1746574717	1937	1937	Love Takes Flight	Love Takes Flight	Nagel, Conrad	Conrad Nagel	tt0029168
1746574061	1957	1957	Plunder Road	Plunder Road	Cornfield, Hubert	Hubert Cornfield	tt0050847
1746249802	1979	1979	The Rose	The Rose	Rydell, Mark	Mark Rydell	tt0079826
1746410737	1989	1989	Jacknife	Jacknife	Jones, David	David Hugh Jones	tt0097607
1746583608	1930	1930	Cock o' the Walk	Cock o' the Walk	Neill, R. William; Lang, Walter	Walter Lang	tt0020773
1746550031	1954	1954	Deep in My Heart	Deep in My Heart	Donen, Stanley	Stanley Donen	tt0046896
1746581322	1962	1962	No Man Is an Island	No Man Is an Island	Monks, Jr., John; Goldstone, Richarc	Richard Goldstone	tt0056283
1746424616	1942	1942	Hillbilly Blitzkrieg	Hillbilly Blitzkrieg	Mack, Roy	Roy Mack	tt0034854
1746520434	1927	1927	Ranger of the North	Ranger of the North	Storm, Jerome	Jerome Storm	tt0018306
1746574158	1959	1958	Rally 'Round the Flag, Boys!	Rally 'Round the Flag, Boys!	McCarey, Leo	Leo McCarey	tt0052117
1769971944	1969	1969	House of Pain and Pleasure	House of Pain and Pleasure	Rotsler, William	William Rotsler	tt0220540
1746453766	1941	1941	I Wake Up Screaming	I Wake Up Screaming	Humberstone, Bruce	H. Bruce Humberstone	tt0033740
1746503401	1920	1920	Treasure Island	Treasure Island	Tourneur, Maurice	Maurice Tourneur	tt0011785
1746577940	1934	1934	Speed Wings	Speed Wings	Brower, Otto	Otto Brower	tt0025819
1746420785	1993	1993	Ed and His Dead Mother	Ed and His Dead Mother	Wacks, Jonathan	Jonathan Wacks	tt0106792
1746574684	1935	1935	Captured in Chinatown	Captured in Chinatown	Clifton, Elmer	Elmer Clifton	tt0026177

1746503056	1916	1916	The Revolt	The Revolt	O'Neil, Barry	Barry O'Neil	tt0007261
1746492208	1947	1947	Black Gold	Black Gold	Karlson, Phil	Phil Karlson	tt0039191
1746580293	1937	1937	45 Fathers	45 Fathers	Tinling, James	James Tinling	tt0028543
1746584544	1931	1931	Defenders of the Law	Defenders of the Law	Levering, Joseph	Joseph Levering	tt0021792
1745309111	2005	2005	Zathura: A Space Adventure	Zathura: A Space Adventure	Favreau, Jon	Jon Favreau	tt0406375
1746502730	1916	1916	The Love Girl	The Love Girl	Leonard, Robert Z.	Robert Z. Leonard	tt0006946
1746577497	1936	1936	Wives Never Know	Wives Never Know	Nugent, Elliott	Elliott Nugent	tt0028514
1746580712	1936	1936	Second Wife	Second Wife	Killy, Edward	Edward Killy	tt0028229
1746410206	1988	1988	They Live	They Live	Carpenter, John	John Carpenter	tt0096256
1746582217	1929	1929	Welcome Danger	Welcome Danger	Bruckman, Clyde	Clyde Bruckman	tt0020572
1746570103	1952	1952	Kangaroo	Kangaroo	Milestone, Lewis	Lewis Milestone	tt0044788
1746497923	1918	1918	The Sign Invisible	The Sign Invisible	Lewis, Edgar	Edgar Lewis	tt0009615
1746573785	1960	1960	Many Ways to Sin	Many Ways to Sin		J.G. Tiger	tt2791252
1746524092	1910	1910	Stunts on Skates	Stunts on Skates			tt1907764
1745358687	2004	2003	The United States of Leland	The United States of Leland	Hoge, Matthew Ryan	Matthew Ryan Hoge	tt0301976
1746583058	1935	1935	Let's Live Tonight	Let's Live Tonight	Schertzinger, Victor	Victor Schertzinger	tt0025593
1746520797	1925	1925	Quicker'n Lightnin'	Quicker'n Lightnin'	Thorpe, Richard	Richard Thorpe	tt0152119
1746392819	1984	1984	The Wild Life	The Wild Life	Linson, Art	Art Linson	tt0088402
1746216789	1968	1968	Jigsaw	Jigsaw	Goldstone, James	James Goldstone	tt0063155
1746576614	1910	1910	The Desperado	The Desperado		Gilbert M. 'Broncho Billy' Anderson	tt0001180
1746574094	1960	1960	Key Witness	Key Witness	Karlson, Phil	Phil Karlson	tt0053993
1746584775	1930	1930	The Little Accident	Little Accident	Craft, William James	William James Craft	tt0021078
1746584885	1940	1940	Men Against the Sky	Men Against the Sky	Goodwins, Leslie	Leslie Goodwins	tt0032781
1746524038	1916	1916	Alien Souls	Alien Souls	Reicher, Frank	Frank Reicher	tt0006343
1745293890	2007	2007	The Messengers	The Messengers	Pang, Danny ; Pang, Oxide	Danny Pang	tt0425430
1746581084	1951	1951	The Medium	The Medium	Menotti, Gian-Carlo	Gian Carlo Menotti	tt0043794
1746560488	1932	1932	Cannonball Express	Cannonball Express	Fox, Wallace W.	Wallace Fox	tt0022742
1746467406	1917	1917	The Seventh Sin	The Seventh Sin		Theodore Marston	tt0182413
1746577678	1936	1936	The Cowboy and the Kid	The Cowboy and the Kid	Taylor, Ray	Ray Taylor	tt0027472
1769969380	1932	1932	The Boudoir Butler	The Boudoir Butler	Pearce, Leslie	Leslie Pearce	tt0022717
1746491861	1947	1947	Ramrod	Ramrod	DeToth, Andre	André De Toth	tt0039750
1746423055	1994	1994	The Mask	The Mask	Russell, Charles	Chuck Russell	tt0110475
1746580260	1936	1936	Cain and Mabel	Cain and Mabel	Bacon, Lloyd	Lloyd Bacon	tt0027413
1746422360	1940	1940	Millionaire Playboy	Millionaire Playboy	Goodwins, Leslie	Leslie Goodwins	tt0032795
1746454210	1944	1944	My Buddy	My Buddy	Sekely, Steve	Steve Sekely	tt0037109
1746454599	1998	1998	The Horse Whisperer	The Horse Whisperer	Redford, Robert	Robert Redford	tt0119314
1745374717	2009	2009	Alien Trespass	Alien Trespass	Goodwin, R. W.	R.W. Goodwin	tt1122836
1746577585	1933	1933	Laughter in Hell	Laughter in Hell	Cahn, Edward L.	Edward L. Cahn	tt0024247
1746495784	1919	1919	Hell Roarin' Reform	Hell Roarin' Reform	LeSaint, Edward J.	Edward LeSaint	tt0010235
1746513392	1928	1928	Masked Angel	The Masked Angel	O'Connor, Frank	Frank O'Connor	tt0019149
1746585410	1940	1940	Rebecca	Rebecca	Hitchcock, Alfred	Alfred Hitchcock	tt0032976
1746474967	1948	1948	Mystery in Mexico	Mystery in Mexico	Wise, Robert	Robert Wise	tt0040631
1746520974	1949	1949	The Younger Brothers	The Younger Brothers	Marin, Edwin L.	Edwin L. Marin	tt0042061
1746581290	1910	1910	The Little Doctor of the Foothills	The Little Doctor of the Foothills	Anderson, G. M.	Gilbert M. 'Broncho Billy' Anderson	tt0372382
1746410632	1993	1993	The Secret Garden	The Secret Garden	Holland, Agnieszka	Agnieszka Holland	tt0108071
1746393446	1984	1983	Sleepaway Camp	Sleepaway Camp	Hiltzik, Robert	Robert Hiltzik	tt0086320
1746572307	1931	1931	Unfaithful	Unfaithful	Cromwell, John	John Cromwell	tt0022520
1746575661	1910	1910	A Darling Confusion	A Darling Confusion		Tom Ricketts	tt1644500
1746514071	1916	1916	Lieutenant Danny, U.S.A.	Lieutenant Danny, U.S.A.	Edwards, Walter	Walter Edwards	tt0159534
1746514765	1916	1916	Mice and Men	Mice and Men	Dawley, J. Searle	J. Searle Dawley	tt0007060
1746512319	1913	1913	A Prisoner in the Harem	A Prisoner in the Harem	Blaché, Herbert	Herbert Blaché	tt0003299
1746554917	1964	1964	The Visit	The Visit	Wicki, Bernhard	Bernhard Wicki	tt0058724
1746200058	1966	1966	Hold On!	Hold On!	Lubin, Arthur	Arthur Lubin	tt0060512
1746507885	1924	1924	The Smoking Trail	Smoking Trails	Bertram, William	William Bertram	tt0015344
1746562011	1955	1955	Cult of the Cobra	Cult of the Cobra	Lyon, Francis D.	Francis D. Lyon	tt0047966
1746543069	1915	1915	Silver Threads Among the Gold	Silver Threads Among the Gold	Kingsley, Pierce	Pierce Kingsley	tt0006046
1746582685	1937	1937	Let's Get Married	Let's Get Married	Green, Alfred E.	Alfred E. Green	tt0029135

1746492215	1945	1945	Texas Panhandle	Texas Panhandle	Nazarro, Ray	Ray Nazarro	tt0039019
1746573988	1959	1959	Inside the Mafia	Inside the Mafia	Cahn, Edward L.	Edward L. Cahn	tt0052927
1746501040	1916	1916	The Garden of Allah	The Garden of Allah	Campbell, Colin	Colin Campbell	tt0007988
1746500294	1948	1948	False Paradise	False Paradise	Archainbaud, George	George Archainbaud	tt0040339
1746570764	1933	1934	Picture Brides	Picture Brides	Rosen, Phil	Phil Rosen	tt0024449
1746500677	1926	1926	The Taxi Mystery	The Taxi Mystery	Windermere, Fred	Fred Windemere	tt0168212
1746514965	1927	1927	Web of Fate	Web of Fate	Fitzgerald, Dallas M.	Dallas M. Fitzgerald	tt0018551
1746579024	1959	1959	The Young Land	The Young Land	Tetzlaff, Ted	Ted Tetzlaff	tt0053461
1746584051	1940	1940	Cross-Country Romance	Cross Country Romance	Woodruff, Frank	Frank Woodruff	tt0032369
1745357620	2004	2004	The Ladykillers	The Ladykillers	Coen, Joel; Coen, Ethan	Ethan Coen	tt0335245
1746571008	1935	1935	Dante's Inferno	Dante's Inferno	Lachman, Harry	Harry Lachman	tt0026262
1746572108	1931	1931	The Spirit of Notre Dame	The Spirit of Notre Dame	Mack, Russell	Russell Mack	tt0022422
1746554679	1964	1964	Zorba the Greek	Zorba the Greek	Cacoyannis, Michael	Mihalis Kakogiannis	tt0057831
1746465162	1942	1942	Four Jacks and a Jill	Four Jacks and a Jill	Hively, Jack	Jack Hively	tt0034751
1746514125	1914	1914	The Pursuit of the Phantom	The Pursuit of the Phantom	Bosworth, Hobart	Hobart Bosworth	tt0004504
1746497946	1918	1918	Irish Eyes	Irish Eyes	Dowlan, William	William C. Dowlan	tt0128276
1746570074	1934	1934	Palooka	Palooka	Stoloff, Benjamin	Benjamin Stoloff	tt0025619
1746509452	1927	1927	Pioneers of the West	Pioneers of the West	Perez, Marcel	Marcel Perez	tt0018264
1746530766	1949	1949	Prejudice	Prejudice	Cahn, Edward L.	Edward L. Cahn	tt0040708
1746393344	1981	1981	The Cannonball Run	The Cannonball Run	Needham, Hal	Hal Needham	tt0082136
1769971962	1969	1969	Eat Drink and Make Merrie	Eat, Drink and Make Merrie	Boyd, Whit	Whit Boyd	tt0136111
1746555823	1956	1956	The Brave One	The Brave One	Rapper, Irving	Irving Rapper	tt0049030
1746185335	1965	1965	The Glory Guys	The Glory Guys	Laven, Arnold	Arnold Laven	tt0059232
1746580399	1935	1935	Awakening of Jim Burke	The Awakening of Jim Burke	Hillyer, Lambert	Lambert Hillyer	tt0026089
1746465282	1947	1947	Killer at Large	Killer at Large	Beaudine, William	William Beaudine	tt0130060
1746503923	1918	1918	The Eyes of Mystery	The Eyes of Mystery	Browning, Tod	Tod Browning	tt0009048
1746554068	1964	1964	Rhino!	Rhino!	Tors, Ivan	Ivan Tors	tt0058522
1746435398	1998	1998	Fear and Loathing in Las Vegas	Fear and Loathing in Las Vegas	Gilliam, Terry	Terry Gilliam	tt0120669
1746581904	1929	1929	Marquis Preferred	Marquis Preferred	Tuttle, Frank	Frank Tuttle	tt0020145
1746183860	1910	1910	The Barge Man of Old Holland	The Barge Man of Old Holland			tt0354404
1746582073	1928	1928	Greased Lightning	Greased Lightning	Taylor, Ray	Ray Taylor	tt0018957
1746554680	1964	1964	The Flesh Eaters	The Flesh Eaters	Curtis, Jack	Jack Curtis	tt0058101
1746583037	1935	1935	Skybound	Skybound	Johnson, Raymond K.	Bernard B. Ray	tt0027008
1746561960	1954	1954	Saskatchewan	Saskatchewan	Walsh, Raoul	Raoul Walsh	tt0047449
1746492570	1948	1948	Street Corner	Street Corner	Kelley, Albert	Albert H. Kelley	tt0040842
1746423754	1990	1990	Days of Thunder	Days of Thunder	Scott, Tony	Tony Scott	tt0099371
1746582909	1934	1934	Whirlpool	Whirlpool	Neill, Roy William	Roy William Neill	tt0025983
1746503854	1919	1919	Through the Toils	Through the Toils	Hoyt, Harry O.	Harry O. Hoyt	tt0010777
1746520593	1925	1925	Bashful Jim	Bashful Jim	Cline, Eddie	Edward F. Cline	tt0015605
1745272602	2000	2000	The Original Kings of Comedy	The Original Kings of Comedy	Lee, Spike	Spike Lee	tt0236388
1746577364	1961	1961	Pocketful of Miracles	Pocketful of Miracles	Capra, Frank	Frank Capra	tt0055312
1745359821	2009	2009	The Lovely Bones	The Lovely Bones	Jackson, Peter	Peter Jackson	tt0380510
1746525000	1923	1923	To the Last Man	To the Last Man	Fleming, Victor	Victor Fleming	tt0014553
1746574191	1962	1962	Jessica	Jessica	Negulesco, Jean	Jean Negulesco	tt0056117
1746567351	1932	1932	McKenna of the Mounted	McKenna of the Mounted	Lederman, D. Ross	D. Ross Lederman	tt0023201
1746520533	1927	1927	Tillie the Toiler	Tillie the Toiler	Henley, Hobart	Hobart Henley	tt0018487
1746581200	1910	1910	Human Hearts	Human Hearts		Hal Reid	tt1156060
1746454201	1999	1999	Galaxy Quest	Galaxy Quest	Parisot, Dean	Dean Parisot	tt0177789
1746584632	1930	1930	The Lash	The Lash	Lloyd, Frank	Frank Lloyd	tt0021048
1746503399	1917	1917	Under False Colors	Under False Colors	Chautard, Emile	Emile Chautard	tt0008715
1746585253	1930	1930	The Spoilers	The Spoilers	Carewe, Edwin	Edwin Carewe	tt0021412
1746580448	1938	1938	Paroled--To Die	Paroled To Die	Newfield, Sam	Sam Newfield	tt0030556
1746475531	1945	1945	Honeymoon Ahead	Honeymoon Ahead	Le Borg, Reginald	Reginald Le Borg	tt0037789
1746424058	1941	1941	Dead Men Tell	Dead Men Tell	Lachman, Harry	Harry Lachman	tt0033519
1746497527	1917	1917	God's Man	God's Man	Irving, George	George Irving	tt0008018
1746492060	1947	1947	Tycoon	Tycoon	Wallace, Richard	Richard Wallace	tt0039927
1746504392	1922	1922	Sunshine Harbor	Sunshine Harbor	Hemmer, Edward L.	Edward L. Hemmer	tt0167425

1746574375	1958	1957	Snowfire	Snowfire	McGowan, Dorrell; McGowan, Stuar	Dorrell McGowan	tt0147519
1746274170	1979	1979	Real Life	Real Life	Brooks, Albert	Albert Brooks	tt0079781
1746475744	1947	1947	Daisy Kenyon	Daisy Kenyon	Preminger, Otto	Otto Preminger	tt0039294
1746475914	1948	1948	For the Love of Mary	For the Love of Mary	De Cordova, Frederick	Frederick De Cordova	tt0040364
1746453924	1996	1996	Kingpin	Kingpin	Farrelly, Peter; Farrelly, Bobby	Bobby Farrelly	tt0116778
1746569937	1960	1959	The Purple Gang	The Purple Gang	McDonald, Frank	Frank McDonald	tt0054218
1745302863	2005	2005	Assault on Precinct 13	Assault on Precinct 13	Richet, Jean-François	Jean	tt0398712
1746560861	1932	1932	The Penal Code	The Penal Code	Melford, George	George Melford	tt0024434
1746520834	1925	1925	Fangs of Fate	Fangs of Fate	Carpenter, Horace B.	Horace B. Carpenter	tt0131383
1769970150	1912	1912	The Worth of a Man	The Worth of a Man	MacDonald, J. Farrell	J. Farrell MacDonald	tt0257276
1746583794	1930	1930	One Night at Susie's	One Night at Susie's	Dillon, John Francis	John Francis Dillon	tt0021216
1746497456	1921	1921	The Broken Spur	The Broken Spur	Wilson, Ben	Ben F. Wilson	tt0012004
1746520465	1926	1926	The Night Patrol	The Night Patrol	Smith, Noel Mason	Noel M. Smith	tt0017207
1746509437	1925	1925	Cactus Trails	Cactus Trails	Webb, Harry	Harry S. Webb	tt0015661
1746514279	1927	1927	The Down Grade	The Down Grade	Hutchison, Charles	Charles Hutchison	tt0017823
1746435340	1997	1997	Metro	Metro	Carter, Thomas	Thomas Carter	tt0119664
1746570292	1952	1952	My Son John	My Son John	McCarey, Leo	Leo McCarey	tt0044941
1746440021	1996	1996	The Crucible	The Crucible	Hytner, Nicholas	Nicholas Hytner	tt0115988
1746465416	1947	1947	Linda, Be Good	Linda, Be Good	McDonald, Frank	Frank McDonald	tt0039569
1746583937	1930	1930	The Bad Man	The Bad Man	Badger, Clarence	Clarence G. Badger	tt0020662
1746423494	1999	1999	Jawbreaker	Jawbreaker	Stein, Darren	Darren Stein	tt0155776
1746577971	1936	1936	Soak the Rich	Soak the Rich	Hecht, Ben; MacArthur, Charles	Ben Hecht	tt0028273
1746453469	1942	1942	Rings on Her Fingers	Rings on Her Fingers	Mamoulian, Rouben	Rouben Mamoulian	tt0035258
1746583970	1940	1940	So You Won't Talk	So You Won't Talk	Sedgwick, Edward	Edward Sedgwick	tt0033069
1746185700	1968	1967	Battle Beneath the Earth	Battle Beneath the Earth	Tully, Montgomery	Montgomery Tully	tt0061387
1746583963	1930	1930	Brothers	Brothers	Lang, Walter	Walter Lang	tt0020716
1746416030	1917	1917	The Long Trail	The Long Trail	Hansell, Howell	Howell Hansel	tt0008214
1746574128	1961	1961	Buffalo Gun	Buffalo Gun	Gannaway, Albert C.	Albert C. Gannaway	tt0174522
1746574424	1960	1959	The Gazebo	The Gazebo	Marshall, George	George Marshall	tt0052837
1746582742	1936	1936	Under Two Flags	Under Two Flags	Lloyd, Frank	Frank Lloyd	tt0028442
1746573380	1932	1932	Silver Dollar	Silver Dollar	Green, Alfred E.	Alfred E. Green	tt0023472
1746513243	1916	1916	The Shrine of Happiness	The Shrine of Happiness	Bracken, Bertram	Bertram Bracken	tt0158954
1745272126	2001	2001	Bandits	Bandits	Levinson, Barry	Barry Levinson	tt0219965
1746566027	1955	1955	5 Against the House	5 Against the House	Karlson, Phil	Phil Karlson	tt0048077
1746584125	1939	1939	Bridal Suite	Bridal Suite	Thiele, William	Wilhelm Thiele	tt0031120
1746577405	1962	1962	Woman Hunt	Womanhunt	Dexter, Maury	Maury Dexter	tt0056699
1746454959	1945	1945	Life with Blondie	Life with Blondie	Berlin, Abby	Abby Berlin	tt0038691
1746500122	1925	1925	Just Plain Folks	Just Plain Folks	Bradbury, Robert N.	Robert N. Bradbury	tt4577762
1746560331	1965	1965	What's New Pussycat?	What's New Pussycat	Donner, Clive	Clive Donner	tt0059903
1746582674	1938	1938	Go Chase Yourself	Go Chase Yourself	Cline, Edward F.	Edward F. Cline	tt0030188
1746502383	1923	1923	Little Johnny Jones	Little Johnny Jones	Rosson, Arthur; Hines, Johnny	Johnny Hines	tt0014204
1746581560	1928	1928	A Race for Life	A Race for Life	Lederman, D. Ross	D. Ross Lederman	tt0019302
1746570340	1933	1933	Tillie and Gus	Tillie and Gus	Martin, Francis	Francis Martin	tt0024672
1746508063	1926	1926	Beyond the Rockies	Beyond the Rockies	Nelson, Jack	Jack Nelson	tt0016647
1746530798	1950	1950	The Arizona Cowboy	The Arizona Cowboy	Springsteen, R. G.	R.G. Springsteen	tt0042203
1746555316	1954	1954	The Golden Mistress	The Golden Mistress	Judge, Joel	Abner Biberman	tt0047037
1746566898	1932	1932	Born to Fight	Born to Fight	Mayo, Walter	Walter Mayo	tt2088709
1746533004	1927	1927	Eager Lips	Eager Lips	Noy, Wilfred	Wilfred Noy	tt0017840
1746566908	1933	1933	Morning Glory	Morning Glory	Sherman, Lowell	Lowell Sherman	tt0024353
1746235686	1973	1973	Superchick	Superchick	Forsyth, Ed	Ed Forsyth	tt0070752
1746496850	1920	1920	Away Goes Prudence	Away Goes Prudence	Robertson, John S.	John S. Robertson	tt0010975
1746435239	1941	1941	They Met in Bombay	They Met in Bombay	Brown, Clarence	Clarence Brown	tt0034281
1746580281	1935	1935	Mutiny on the Bounty	Mutiny on the Bounty	Lloyd, Frank	Frank Lloyd	tt0026752
1746520779	1926	1926	Kiki	Kiki	Brown, Clarence	Clarence Brown	tt0017029
1746492409	1945	1945	Fashion Model	Fashion Model	Beaudine, William	William Beaudine	tt0037692
1746567021	1933	1933	Forgotten Men	Forgotten Men	Pollard, Bud	Bud Pollard	tt0150614
1746410371	1983	1982	Purple Haze	Purple Haze	Morris, David Burton	David Burton Morris	tt0084553

1746523964	1915	1915	Beulah	Beulah	Bracken, Bertram	Bertram Bracken	tt0004963
1746583752	1928	1928	Desperate Courage	Desperate Courage	Thorpe, Richard	Richard Thorpe	tt0018823
1746574718	1936	1936	The Lawless Nineties	The Lawless Nineties	Kane, Joseph	Joseph Kane	tt0027876
1746524001	1917	1917	The Dream Doll	The Dream Doll	Moss, Howard S.	Howard S. Moss	tt0193113
1746423340	1994	1994	Fresh	Fresh	Yakin, Boaz	Boaz Yakin	tt0109842
1746501280	1917	1917	His Father's Son	His Father's Son	Baker, George D.	George D. Baker	tt0008106
1746570752	1934	1934	The Trumpet Blows	The Trumpet Blows	Roberts, Stephen	Stephen Roberts	tt0025915
1746508109	1926	1926	Keep Going	Keep Going	Harvey, John	Jack Harvey	tt4205688
1746570013	1960	1960	The Wizard of Baghdad	The Wizard of Baghdad	Sherman, George	George Sherman	tt0054481
1746514602	1923	1923	The Famous Mrs. Fair	The Famous Mrs. Fair	Niblo, Fred	Fred Niblo	tt0014036
1746273680	1978	1978	Midnight Express	Midnight Express	Parker, Alan	Alan Parker	tt0077928
1746437416	1942	1942	Junior Army	Junior Army	Landers, Lew	Lew Landers	tt0036061
1746582741	1937	1937	They Wanted to Marry	They Wanted to Marry	Landers, Lew	Lew Landers	tt0029657
1746491717	1947	1947	It Happened in Brooklyn	It Happened in Brooklyn	Whorf, Richard	Richard Whorf	tt0039501
1769974260	1916	1916	The Little Trespasser	The Little Trespasser	Williams, C. J.	C.J. Williams	tt0490644
1746503270	1918	1918	Hearts of the World	Hearts of the World	Griffith, D. W.	D.W. Griffith	tt0009150
1746582942	1937	1937	Easy Living	Easy Living	Leisen, Mitchell	Mitchell Leisen	tt0028816
1746394710	1990	1990	The Adventures of Ford Fairlane	The Adventures of Ford Fairlane	Harlin, Renny	Renny Harlin	tt0098987
1746420138	1994	1994	Cops & Robbersons	Cops and Robbersons	Ritchie, Michael	Michael Ritchie	tt0109480
1746508114	1925	1925	My Son	My Son	Carewe, Edwin	Edwin Carewe	tt0016137
1746454588	1997	1997	Mad City	Mad City	Gavras, Costa	Costa	tt0119592
1746508203	1926	1926	Brooding Eyes	Brooding Eyes	Le Saint, Edward J.	Edward LeSaint	tt0016688
1746393981	1985	1985	The Color Purple	The Color Purple	Spielberg, Steven	Steven Spielberg	tt0088939
1746453855	1942	1942	Cadets on Parade	Cadets on Parade	Landers, Lew	Lew Landers	tt0034564
1746583637	1929	1929	The Delightful Rogue	The Delightful Rogue	Shores, Lynn	Lynn Shores	tt0019810
1746500384	1927	1927	The Kid Brother	The Kid Brother	Wilde, Ted	Ted Wilde	tt0018051
1746420931	1940	1940	Five Little Peppers at Home	Five Little Peppers at Home	Barton, Charles	Charles Barton	tt0032472
1746574655	1938	1938	Gun Law	Gun Law	Howard, David	David Howard	tt0030207
1746424039	1941	1941	Fugitive Valley	Fugitive Valley	Luby, S. Roy	S. Roy Luby	tt0033641
1769971200	1969	1969	Slaves of Love	Slaves of Love	Nizet, Charles	Charles Nizet	tt0128531
1745340787	2002	2001	Storytelling	Storytelling	Solondz, Todd	Todd Solondz	tt0250081
1746438926	1940	1940	I'm Nobody's Sweetheart Now	I'm Nobody's Sweetheart Now	Lubin, Arthur	Arthur Lubin	tt0032621
1746514940	1927	1927	The Fighting Three	The Fighting Three	Rogell, Albert	Albert S. Rogell	tt0017879
1746501211	1916	1915	Who Killed Joe Merrion?	Who Killed Joe Merrion?	Johnson, Tefft	Tefft Johnson	tt0007562
1746583841	1931	1931	Alice in Wonderland	Alice in Wonderland	Pollard, "Bud"	Bud Pollard	tt0021599
1746582143	1928	1928	Sally of the Scandals	Sally of the Scandals	Shores, Lynn	Lynn Shores	tt0019349
1746512888	1915	1915	Vengeance of the Wilds	Vengeance of the Wilds	Big Otto	Otto Breitkreutz	tt0006212
1745340086	2003	2002	The Anarchist Cookbook	The Anarchist Cookbook	Susman, Jordan	Jordan Susman	tt0284850
1746515808	1920	1920	Mrs. Temple's Telegram	Mrs. Temple's Telegram	Cruze, James	James Cruze	tt0011486
1746578539	1950	1950	Wabash Avenue	Wabash Avenue	Koster, Henry	Henry Koster	tt0043116
1746422591	1996	1996	Muppet Treasure Island	Muppet Treasure Island	Henson, Brian	Brian Henson	tt0117110
1746408836	1990	1990	Arachnophobia	Arachnophobia	Marshall, Frank	Frank Marshall	tt0099052
1746499940	1946	1946	Her Adventurous Night	Her Adventurous Night	Rawlins, John	John Rawlins	tt0038596
1746420770	1989	1989	In Country	In Country	Jewison, Norman	Norman Jewison	tt0097570
1746500091	1924	1924	Fools Highway	Fools Highway	Cummings, Irving	Irving Cummings	tt0014921
1746574501	1933	1933	Dangerous Crossroads	Dangerous Crossroads	Hillyer, Lambert	Lambert Hillyer	tt0023927
1746409725	1981	1981	The Postman Always Rings Twice	The Postman Always Rings Twice	Rafelson, Bob	Bob Rafelson	tt0082934
1746576871	1931	1931	The Finger Points	Finger Prints	Dillon, John Francis	Ray Taylor	tt0021866
1746584607	1930	1930	Kathleen Mavourneen	Kathleen Mavourneen	Ray, Albert	Albert Ray	tt0021023
1746576853	1931	1930	New Moon	New Moon	Conway, Jack	Jack Conway	tt0021178
1746584978	1940	1940	Buck Benny Rides Again	Buck Benny Rides Again	Sandrich, Mark	Mark Sandrich	tt0032289
1746533033	1915	1915	God's Witness	God's Witness	Moore, Eugene W.	Eugene Moore	tt0005399
1746583824	1930	1930	Du Barry, Woman of Passion	Du Barry, Woman of Passion	Taylor, Sam	Sam Taylor	tt0020844
1746500403	1927	1927	Let It Rain	Let It Rain	Cline, Eddie	Edward F. Cline	tt0018084
1746492061	1944	1944	Bermuda Mystery	Bermuda Mystery	Stoloff, Benjamin	Benjamin Stoloff	tt0036638
1746393540	1983	1983	Silkwood	Silkwood	Nichols, Mike	Mike Nichols	tt0086312
1746455487	1997	1997	Shadow Conspiracy	Shadow Conspiracy	Cosmatos, George P.	George P. Cosmatos	tt0120107

1746554821	1955	1955	African Manhunt	African Manhunt	Friedman, Seymour	Seymour Friedman	tt0050106	
1746421001	1990	1990	A Shock to the System	A Shock to the System	Egleson, Jan	Jan Egleson	tt0100602	
1746582734	1937	1937	Call It a Day	Call It a Day	Mayo, Archie	Archie Mayo	tt0028679	
1745373751	2009	2009	Halloween II	Halloween II	Zombie, Rob	Rob Zombie	tt1311067	
1746409089	1988	1988	Tucker: The Man and His Dream	Tucker: The Man and His Dream	Coppola, Francis Ford	Francis Ford Coppola	tt0096316	
1746573961	1957	1957	Tammy and the Bachelor	Tammy and the Bachelor	Pevney, Joseph	Joseph Pevney	tt0051051	
1746464912	1942	1942	Springtime in the Rockies	Springtime in the Rockies	Cummings, Irving	Irving Cummings	tt0035370	
1745294007	2007	2007	Everything's Cool	Everything's Cool	Gold, Daniel B.; Helfand, Judith	Daniel B. Gold	tt0810970	
1746466658	1917	1917	The Snarl	The Snarl	West, Raymond B.	Raymond B. West	tt0182431	
1746579006	1951	1951	Utah Wagon Train	Utah Wagon Train	Ford, Philip	Philip Ford	tt0044175	
1746512139	1916	1916	The Devil's Bondwoman	The Devil's Bondwoman	Carleton, Lloyd B.	Lloyd B. Carleton	tt0006587	
1746577997	1935	1935	Square Shooter	Square Shooter	Selman, David	David Selman	tt0027036	
1746578999	1961	1961	Romanoff and Juliet	Romanoff and Juliet	Ustinov, Peter	Peter Ustinov	tt0055383	
1746576027	1931	1931	Pardon Us	Pardon Us	Parrott, James	James Parrott	tt0022251	
1746582615	1936	1936	The Crime Patrol	The Crime Patrol	Cummings, Eugene	Eugene Cummings	tt0027477	
1746422535	1991	1991	New Jack City	New Jack City	Van Peebles, Mario	Mario Van Peebles	tt0102526	
1746435617	1940	1940	Charter Pilot	Charter Pilot	Forde, Eugene	Eugene Forde	tt0032328	
1746583969	1939	1939	The Escape	The Escape	Cortez, Richard	Ricardo Cortez	tt0031279	
**1746565991**	**1957**	**1957**	**Domino Kid**	**Domino Kid**	**Nazarro, Ray**	**Ray Nazarro**	**tt0050325**	1
1746453602	1941	1941	Billy the Kid's Fighting Pals	Billy the Kid's Fighting Pals	Scott, Sherman	Sam Newfield	tt0033393	
1746500737	1949	1949	Stallion Canyon	Stallion Canyon	Fraser, Harry	Harry L. Fraser	tt0130281	
1746438144	1996	1995	Dead Man	Dead Man	Jarmusch, Jim	Jim Jarmusch	tt0112817	
1746550221	1955	1955	Lady Godiva of Coventry	Lady Godiva of Coventry	Lubin, Arthur	Arthur Lubin	tt0048279	
1746421950	1995	1995	Kiss of Death	Kiss of Death	Schroeder, Barbet	Barbet Schroeder	tt0113552	
1746577184	1963	1963	Terrified!	Terrified	Landers, Lew	Lew Landers	tt0142906	
1746502018	1915	1915	Father and the Boys	Father and the Boys	De Grasse, Joseph	Joseph De Grasse	tt0005307	
1746423745	1992	1992	Honey, I Blew Up the Kid	Honey I Blew Up the Kid	Kleiser, Randal	Randal Kleiser	tt0104437	
1746419972	1993	1993	Dragon: The Bruce Lee Story	Dragon: The Bruce Lee Story	Cohen, Rob	Rob Cohen	tt0106770	
1746577972	1939	1939	Heroes in Blue	Heroes in Blue	Watson, William	William Watson	tt0031421	
1746465380	1947	1947	Down to Earth	Down to Earth	Hall, Alexander	Alexander Hall	tt0039337	
1746508542	1926	1926	Looking for Trouble	Looking for Trouble	Bradbury, Robert North	Robert N. Bradbury	tt0017079	
1745358883	2004	2004	Scooby-Doo 2: Monsters Unleashed	Scooby Doo 2: Monsters Unleashed	Gosnell, Raja	Raja Gosnell	tt0331632	
1746453519	1942	1942	The Lady Has Plans	The Lady Has Plans	Lanfield, Sidney	Sidney Lanfield	tt0034960	
1746530688	1948	1948	Yellow Sky	Yellow Sky	Wellman, William A.	William A. Wellman	tt0040978	
1746435503	1941	1941	Fiesta	Fiesta	Prinz, LeRoy	LeRoy Prinz	tt0034730	
1746582439	1937	1937	Boss of Lonely Valley	Boss of Lonely Valley	Taylor, Ray	Ray Taylor	tt0028657	
1746423029	1991	1991	Harley Davidson and the Marlboro Man	Harley Davidson and the Marlboro Man	Wincer, Simon	Simon Wincer	tt0102005	
1746581766	1910	1910	Justinian and Theodora	Justinian and Theodora		Otis Turner	tt0354677	
1746565963	1953	1953	Jamaica Run	Jamaica Run	Foster, Lewis R.	Lewis R. Foster	tt0045927	
1769971146	1969	1969	Shannon's Women	Shannon's Women	Rotsler, William	William Rotsler	tt0174198	
1746584061	1935	1935	The Lady in Scarlet	The Lady in Scarlet	Lamont, Charles	Charles Lamont	tt0026599	
1746410398	1992	1992	A River Runs Through It	A River Runs Through It	Redford, Robert	Robert Redford	tt0105265	
1769969877	1912	1912	A Dreamland Tragedy	A Dreamland Tragedy			tt0361539	
1746465173	1946	1946	Home on the Range	Home on the Range	Springsteen, Robert	R.G. Springsteen	tt0038612	
1746578658	1951	1951	Danger Zone	Danger Zone	Berke, William	William Berke	tt0043448	
1746585397	1940	1940	Sing, Dance, Plenty Hot	Sing, Dance, Plenty Hot	Landers, Lew	Lew Landers	tt0033054	
1746580599	1935	1935	A Feather in Her Hat	A Feather in Her Hat	Santell, Alfred	Alfred Santell	tt0026343	
1769970540	1967	1967	To Turn a Trick	To Turn a Trick		C. Davis Smith	tt0128767	
1746574462	1936	1936	Kelly the Second	Kelly the Second	Meins, Gus	Gus Meins	tt0027840	
1746453500	1941	1941	The Great Lie	The Great Lie	Goulding, Edmund	Edmund Goulding	tt0033677	
1745309382	2005	2004	Black Cloud	Black Cloud	Schroder, Rick	Ricky Schroder	tt0363475	
1746584492	1930	1930	She Got What She Wanted	She Got What She Wanted	Cruze, James	James Cruze	tt0021361	
1746394162	1982	1982	The Verdict	The Verdict	Lumet, Sidney	Sidney Lumet	tt0084855	
1746583201	1931	1931	The Good Bad Girl	The Good Bad Girl	Neill, R. William	Roy William Neill	tt0021921	
1746524990	1928	1928	Melody of Love	Melody of Love	Heath, A. B.	Arch Heath	tt0019156	
1746492742	1946	1946	That Texas Jamboree	That Texas Jamboree	Nazarro, Ray	Ray Nazarro	tt0039021	
1746554886	1957	1957	Time Limit	Time Limit	Malden, Karl	Karl Malden	tt0051083	

1746208648	1968	1968	No More Excuses	No More Excuses	Downey, Robert	Robert Downey Sr.	tt0251300
1746583495	1930	1930	Holiday	Holiday	Griffith, Edward H.	Edward H. Griffith	tt0020985
1746500562	1926	1926	Mike	Mike	Neilan, Marshall	Marshall Neilan	tt0017148
1746475284	1944	1944	Bride by Mistake	Bride by Mistake	Wallace, Richard	Richard Wallace	tt0036671
1746500246	1926	1926	The Test of Donald Norton	The Test of Donald Norton	Eason, B. Reeves	B. Reeves Eason	tt0017454
1746524264	1915	1915	From the Valley of the Missing	From the Valley of the Missing	Powell, Frank	Frank Powell	tt0005363
1746198083	1968	1968	Day of the Evil Gun	Day of the Evil Gun	Thorpe, Jerry	Jerry Thorpe	tt0062865
1746394242	1987	1987	The Princess Bride	The Princess Bride	Reiner, Rob	Rob Reiner	tt0093779
1746577003	1961	1961	A Raisin in the Sun	A Raisin in the Sun	Petrie, Daniel	Daniel Petrie	tt0055353
1769970396	1966	1966	Indiscreet Stairway	Indiscreet Stairway	Ren-Mart	Ron Mart	tt0221274
1746577520	1935	1935	Men of Action	Men of Action	James, Alan	Alan James	tt0026700
1746576516	1931	1931	East of Borneo	East of Borneo	Melford, George	George Melford	tt0021828
1746409438	1982	1982	Young Doctors in Love	Young Doctors in Love	Marshall, Garry	Garry Marshall	tt0084938
1746581117	1950	1950	Where the Sidewalk Ends	Where the Sidewalk Ends	Preminger, Otto	Otto Preminger	tt0043132
1745360014	2002	2002	Friday After Next	Friday After Next	Raboy, Marcus	Marcus Raboy	tt0293815
1746514296	1915	1915	The Truth About Helen	The Truth About Helen	McGlynn, Frank	Frank McGlynn Sr.	tt0006185
1746508991	1924	1924	A Self-Made Failure	A Self Made Failure	Beaudine, William	William Beaudine	tt0015314
1746561055	1932	1932	The Famous Ferguson Case	The Famous Ferguson Case	Bacon, Lloyd	Lloyd Bacon	tt0022876
1745309375	2007	2007	Talk to Me	Talk to Me	Lemmons, Kasi	Kasi Lemmons	tt0796368
1745282858	2005	2005	Roll Bounce	Roll Bounce	Lee, Malcolm D.	Malcolm D. Lee	tt0403455
1746487340	1920	1920	Skyfire	Skyfire	Hart, Neal	Neal Hart	tt0197891
1746574414	1960	1960	The Crowning Experience	The Crowning Experience	Tegström, Rickard	Marion Clayton Anderson	tt0052715
1746492400	1947	1947	The Big Fix	The Big Fix	Flood, James	James Flood	tt0039185
1746563920	1952	1952	The Big Trees	The Big Trees	Feist, Felix	Felix E. Feist	tt0044420
1746580315	1937	1937	Stars over Arizona	Stars Over Arizona	Bradbury, Robert N.	Robert N. Bradbury	tt0030792
1745374345	2010	2010	The Karate Kid	The Karate Kid	Zwart, Harald	Harald Zwart	tt1155076
1746409263	1992	1992	Singles	Singles	Crowe, Cameron	Cameron Crowe	tt0105415
1746554913	1965	1965	The Family Jewels	The Family Jewels	Lewis, Jerry	Jerry Lewis	tt0059166
1746491710	1946	1946	Tomorrow Is Forever	Tomorrow Is Forever	Pichel, Irving	Irving Pichel	tt0039041
1746500033	1946	1946	The Secret of the Whistler	Secret of the Whistler	Sherman, George	George Sherman	tt0038917
1746454650	1940	1940	Diamond Frontier	Diamond Frontier	Schuster, Harold	Harold D. Schuster	tt0032394
1746492757	1946	1946	The Shadow Returns	The Shadow Returns	Rosen, Phil	Phil Rosen	tt0038926
1746574499	1935	1935	The Healer	The Healer	Barker, Reginald	Reginald Barker	tt0026637
1746584626	1930	1930	Mountain Justice	Mountain Justice	Brown, Harry J.	Harry Joe Brown	tt0021160
1746434883	1999	1999	The Mummy	The Mummy	Sommers, Stephen	Stephen Sommers	tt0120616
1746577361	1959	1959	Beloved Infidel	Beloved Infidel	King, Henry	Henry King	tt0052617
1746577989	1937	1937	Love Under Fire	Love Under Fire	Marshall, George	George Marshall	tt0029169
1746577983	1935	1935	Bride of Frankenstein	Bride of Frankenstein	Whale, James	James Whale	tt0026138
1746516275	1921	1921	Lavender and Old Lace	Lavender and Old Lace	Ingraham, Lloyd	Lloyd Ingraham	tt0012381
1746233144	1969	1969	Castle Keep	Castle Keep	Pollack, Sydney	Sydney Pollack	tt0064137
1746256785	1978	1978	Same Time, Next Year	Same Time, Next Year	Mulligan, Robert	Robert Mulligan	tt0078199
1746185350	1965	1965	A Thousand Clowns	A Thousand Clowns	Coe, Fred	Fred Coe	tt0059798
1746582996	1939	1939	The Forgotten Woman	The Forgotten Woman	Young, Harold	Harold Young	tt0031331
1746508479	1925	1925	The Prairie Wife	The Prairie Wife	Ballin, Hugo	Hugo Ballin	tt0016239
1746454028	1943	1943	First Comes Courage	First Comes Courage	Arzner, Dorothy; Vidor, Charles	Dorothy Arzner	tt0035883
1746393522	1981	1981	Prince of the City	Prince of the City	Lumet, Sidney	Sidney Lumet	tt0082945
1746561491	1932	1932	Just a Pain in the Parlor	Just a Pain in the Parlor	Marshall, George	George Marshall	tt0456488
1746422040	1992	1992	Malcolm X	Malcolm X	Lee, Spike	Spike Lee	tt0104797
1746508571	1950	1950	Key to the City	Key to the City	Sidney, George	George Sidney	tt0042634
1746524287	1916	1916	The Moment Before	The Moment Before	Vignola, Robert G.	Robert G. Vignola	tt0007089
1746503677	1920	1920	Sic-Em	Sic Em	Sullivan, Frederic	Frederick Sullivan	tt0177222
1746533010	1914	1914	The Mystery of Edwin Drood	The Mystery of Edwin Drood	Blaché, Herbert; Terriss, Tom	Herbert Blaché	tt0004376
1746251471	1975	1975	The Strongest Man in the World	The Strongest Man in the World	McEveety, Vincent	Vincent McEveety	tt0073760
1746583061	1940	1940	Secrets of a Model	Secrets of a Model	Newfield, Sam	Sam Newfield	tt0033033
1746515691	1922	1922	The Woman Who Believed	The Woman Who Believed	Harvey, John	Jack Harvey	tt0181169
1746561411	1932	1932	Gambling Sex	Gambling Sex	Newmeyer, Fred	Fred C. Newmeyer	tt0022926
1746578869	1962	1962	Don't Knock the Twist	Don't Knock the Twist	Rudolph, Oscar	Oscar Rudolph	tt0055922

1745342925	2003	2003	The Matrix Reloaded	The Matrix Reloaded	Wachowski, Andy; Wachowski, Larry	Lana Wachowski	tt0234215
1745272453	2000	2000	Time Code	Timecode	Figgis, Mike	Mike Figgis	tt0220100
1746423495	2000	2000	The Cell	The Cell	Singh, Tarsem	Tarsem Singh	tt0209958
1745372569	2008	2008	Over Her Dead Body	Over Her Dead Body	Lowell, Jeff	Jeff Lowell	tt0785007
1746581569	1910	1910	The Honor of His Family	The Honor of His Family	Griffith, D. W.	D.W. Griffith	tt0001254
1746465114	1946	1946	Sunset Pass	Sunset Pass	Berke, William	William Berke	tt0038996
1746583984	1938	1938	Who Killed Gail Preston?	Who Killed Gail Preston?	Barsha, Leon	Leon Barsha	tt0030974
1746570364	1933	1933	The Invisible Man	The Invisible Man	Whale, James	James Whale	tt0024184
1746583703	1930	1930	Redemption	Redemption	Niblo, Fred	Fred Niblo	tt0021292
1746218681	1973	1973	Terror in the Wax Museum	Terror in the Wax Museum	Fenady, Georg	Georg Fenady	tt0070783
1745357641	2010	2010	Dinner for Schmucks	Dinner for Schmucks	Roach, Jay	Jay Roach	tt0427152
1746560695	1965	1965	Finger on the Trigger	Finger on the Trigger	Pink, Sidney	Sidney W. Pink	tt0059178
1746574078	1956	1956	The Houston Story	The Houston Story	Castle, William	William Castle	tt0049340
1746567998	1952	1952	What Price Glory	What Price Glory	Ford, John	John Ford	tt0045323
1746525042	1920	1920	Live Sparks	Live Sparks	Warde, Ernest C.	Ernest C. Warde	tt0196706
1746584945	1939	1939	The Man in the Iron Mask	The Man in the Iron Mask	Whale, James	James Whale	tt0031619
1746581619	1928	1928	The Scarlet Lady	The Scarlet Lady	Crosland, Alan	Alan Crosland	tt0019358
1746584520	1930	1930	Golden Dawn	Golden Dawn	Enright, Ray	Ray Enright	tt0020926
1746501085	1916	1916	The Masked Rider	The Masked Rider	Balshofer, Fred J.	Fred J. Balshofer	tt0007046
1746424551	1943	1943	Happy Land	Happy Land	Pichel, Irving	Irving Pichel	tt0035970
1746569637	1958	1957	Paradise Lagoon	Paradise Lagoon	Gilbert, Lewis	Lewis Gilbert	tt0050100
1746532957	1915	1915	Fanchon the Cricket	Fanchon, the Cricket	Kirkwood, James	James Kirkwood	tt0005302
1746497639	1921	1921	The Call of His People	The Call of His People			tt0012993
1746571013	1934	1934	This Side of Heaven	This Side of Heaven	Howard, William K.	William K. Howard	tt0025883
1746409357	1990	1990	Hard to Kill	Hard to Kill	Malmuth, Bruce	Bruce Malmuth	tt0099739
1746584730	1928	1928	The Wild West Show	The Wild West Show	Andrews, Del	Del Andrews	tt0019582
1746582663	1937	1937	Maytime	Maytime	Leonard, Robert Z.	Robert Z. Leonard	tt0029222
1746500400	1925	1925	Eve's Secret	Eve's Secret	Badger, Clarence	Clarence G. Badger	tt0015784
1746585408	1939	1939	First Offenders	First Offenders	McDonald, Frank	Frank McDonald	tt0031312
1746565947	1953	1953	Problem Girls	Problem Girls	Dupont, E. A.	Ewald André Dupont	tt0046212
1746580739	1936	1936	Charlie Chan at the Race Track	Charlie Chan at the Race Track	Humberstone, H. Bruce	H. Bruce Humberstone	tt0027441
1746524388	1923	1923	Vanity Fair	Vanity Fair	Ballin, Hugo	Hugo Ballin	tt0014572
1746454372	1945	1945	Flaming Bullets	Flaming Bullets	Fraser, Harry	Harry L. Fraser	tt0037704
1746532452	1923	1923	Wolves of the Border	Wolves of the Border	Neitz, Alvin J.	Alan James	tt0014620
1769973191	1970	1969	I Want More	I Want More	Beap, Jack	Jack Beap	tt0125210
1746422516	1994	1994	The Shawshank Redemption	The Shawshank Redemption	Darabont, Frank	Frank Darabont	tt0111161
1746583899	1931	1931	Fair Warning	Fair Warning	Werker, Alfred L.	Alfred L. Werker	tt0020868
1746509041	1925	1925	Galloping On	Galloping On	Thorpe, Richard	Richard Thorpe	tt0015852
1745359222	2010	2010	Cyrus	Cyrus	Duplass, Jay ; Duplass, Mark	Jay Duplass	tt1336617
1745340973	2003	2003	Matchstick Men	Matchstick Men	Scott, Ridley	Ridley Scott	tt0325805
1746508755	1927	1927	The Lovelorn	The Lovelorn	McCarthy, John P.	John P. McCarthy	tt0018116
1746486803	1919	1919	The Invisible Bond	The Invisible Bond	Maigne, Charles	Charles Maigne	tt0010301
1746567104	1952	1952	Son of Paleface	Son of Paleface	Tashlin, Frank	Frank Tashlin	tt0045177
1745272336	2000	2000	Screwed	Screwed	Alexander, Scott; Karaszewski, Larry	Scott Alexander	tt0156323
1746564804	1952	1952	Waco	Waco	Collins, Lewis	Lewis D. Collins	tt0045304
1746566253	1953	1953	Fort Ti	Fort Ti	Castle, William	William Castle	tt0045784
1746514204	1917	1917	Somewhere in America	Somewhere in America	Dowlan, William C.	William C. Dowlan	tt0125506
1746585105	1928	1928	The Bronc Stomper	The Bronc Stomper	Maloney, Leo D.	Leo D. Maloney	tt0018726
1746580665	1935	1935	The Last Wilderness	The Last Wilderness			tt0126381
1745302816	2007	2007	Brooklyn Rules	Brooklyn Rules	Corrente, Michael	Michael Corrente	tt0283503
1746235281	1972	1972	Fat City	Fat City	Huston, John	John Huston	tt0068575
1746464808	1945	1945	The Enchanted Forest	The Enchanted Forest	Landers, Lew	Lew Landers	tt0037672
1746572784	1931	1931	Secret Service	Secret Service	Ruben, J. Walter	J. Walter Ruben	tt0022356
1746487278	1920	1920	Down on the Farm	Down on the Farm	Kenton, Erle; Grey, Ray; Jones, Richa	Ray Grey	tt0011128
1745342865	2010	2010	The Other Guys	The Other Guys	McKay, Adam	Adam McKay	tt1386588
1746577800	1936	1936	Theodora Goes Wild	Theodora Goes Wild	Boleslawski, Richard	Richard Boleslawski	tt0028355
1746574693	1937	1937	Wild and Woolly	Wild and Woolly	Werker, Alfred	Alfred L. Werker	tt0029779

1746573718	1932	1932	Love Me Tonight	Love Me Tonight	Mamoulian, Rouben	Rouben Mamoulian	tt0023158
1746574701	1938	1938	Tarnished Angel	Tarnished Angel	Goodwins, Leslie	Leslie Goodwins	tt0030831
1746438098	1999	1999	Runaway Bride	Runaway Bride	Marshall, Garry	Garry Marshall	tt0163187
1746483175	1917	1917	The Apple-Tree Girl	The Apple Tree Girl	Crosland, Alan	Alan Crosland	tt0007645
1746520288	1924	1924	Women Who Give	Women Who Give	Barker, Reginald	Reginald Barker	tt0015510
1746532749	1916	1916	Ignorance	Ignorance	Fitzgerald, James A.	James A. Fitzgerald	tt0157788
1746465314	1947	1947	Shoot to Kill	Shoot to Kill	Berke, William	William Berke	tt0039820
1746582337	1928	1928	Dog Justice	Dog Justice	Storm, Jerome	Jerome Storm	tt0126266
1746392966	1981	1981	Final Exam	Final Exam	Huston, Jimmy	Jimmy Huston	tt0082378
1746577784	1936	1937	Border Phantom	Border Phantom	Luby, S. Roy	S. Roy Luby	tt0028649
1746500625	1949	1949	Little Women	Little Women	LeRoy, Mervyn	Mervyn LeRoy	tt0041594
1746515084	1921	1921	The Fire Eater	The Fire Eater	Eason, Reaves	B. Reeves Eason	tt0012171
1746393539	1984	1984	Killpoint	Killpoint	Harris, Frank	Frank Harris	tt0087556
1746508452	1925	1925	Marry Me	Marry Me	Cruze, James	James Cruze	tt0016095
1746435725	1943	1943	Follies Girl	Follies Girl	Rowland, William	William Rowland	tt0035893
1746392928	1985	1985	Agnes of God	Agnes of God	Jewison, Norman	Norman Jewison	tt0088683
1746530672	1949	1949	The Walking Hills	The Walking Hills	Sturges, John	John Sturges	tt0042033
1746561805	1957	1957	Daughter of Dr. Jekyll	Daughter of Dr. Jekyll	Ulmer, Edgar G.	Edgar G. Ulmer	tt0050292
1746454046	1942	1942	The Yanks Are Coming	The Yanks Are Coming	Thurn-Taxis, Alexis	Alexis Thurn	tt0035576
1746570104	1934	1934	I Sell Anything	I Sell Anything	Florey, Robert	Robert Florey	tt0025284
1746507859	1925	1925	The Necessary Evil	The Necessary Evil	Archainbaud, George	George Archainbaud	tt0016148
1746500223	1926	1926	The Still Alarm	The Still Alarm	Laemmle, Edward	Edward Laemmle	tt0017431
1746583935	1928	1928	The Head of the Family	The Head of the Family	Boyle, Joseph C.	Joseph Boyle	tt0018982
1746486330	1917	1917	Weavers of Life	The Weavers of Life	Warren, Edward	Edward Warren	tt0008753
1746409358	1981	1981	Saturday the 14th	Saturday the 14th	Cohen, Howard R.	Howard R. Cohen	tt0083033
1746583693	1931	1931	Salvation Nell	Salvation Nell	Cruze, James	James Cruze	tt0022338
1746514900	1917	1916	Her Better Self	Her Better Self	Vignola, Robert G.	Grace Cunard	tt0329169
1745374402	2009	2009	Gamer	Gamer	Neveldine, Mark ; Taylor, Brian	Mark Neveldine	tt1034032
1746393992	1986	1986	The Mosquito Coast	The Mosquito Coast	Weir, Peter	Peter Weir	tt0091557
1746584057	1939	1939	Three Smart Girls Grow Up	Three Smart Girls Grow Up	Koster, Henry	Henry Koster	tt0032030
1746208621	1969	1969	The Babysitter	The Babysitter	Henderson, Don	Don Henderson	tt0064055
1746499935	1924	1924	Christine of the Hungry Heart	Christine of the Hungry Heart	Archainbaud, George	George Archainbaud	tt0014772
1746487285	1920	1920	A Beggar in Purple	A Beggar in Purple	Lewis, Edgar	Edgar Lewis	tt0186863
1746218100	1966	1966	The Year of the Horse	The Year of the Horse	Sunasky, Irving	Irving Sunasky	tt0303246
1746210455	1971	1971	Minnie and Moskowitz	Minnie and Moskowitz	Cassavetes, John	John Cassavetes	tt0067433
1746581529	1928	1928	The Trail of '98	The Trail of '98	Brown, Clarence	Clarence Brown	tt0019489
1769969407	1911	1911	The Price	The Price		Edwin S. Porter	tt1298595
1746583074	1935	1935	Gold Diggers of 1935	Gold Diggers of 1935	Berkeley, Busby	Busby Berkeley	tt0026421
1746421595	1989	1989	Casualties of War	Casualties of War	De Palma, Brian	Brian De Palma	tt0097027
1746582711	1938	1938	Wild Horse Canyon	Wild Horse Canyon	Hill, Robert	Robert F. Hill	tt0032129
1746217265	1966	1966	The Carolyn Lima Story	The Carolyn Lima Story	Rowe, John	John Rowe	tt0212044
1746574069	1961	1961	Portrait of a Mobster	Portrait of a Mobster	Pevney, Joseph	Joseph Pevney	tt0055317
1746570403	1933	1933	Made on Broadway	Made on Broadway	Beaumont, Harry	Harry Beaumont	tt0024288
1746210946	1971	1971	The Abominable Dr. Phibes	The Abominable Dr. Phibes	Fuest, Robert	Robert Fuest	tt0066740
1746424429	1942	1942	Stand by All Networks	Stand By All Networks	Landers, Lew	Lew Landers	tt0035378
1746563506	1952	1952	Face to Face	Face to Face		John Brahm	tt0044601
1746576835	1910	1910	Examination Day at School	Examination Day at School	Griffith, D. W.	D.W. Griffith	tt0001202
1746415773	1917	1917	Miss Nobody	Miss Nobody	Parke, William	William Parke	tt0008302
1746511798	1915	1915	The Campbells Are Coming	The Campbells Are Coming	Ford, Francis	Francis Ford	tt0005050
1746500069	1924	1924	Daring Chances	Daring Chances	Smith, Clifford S.	Clifford Smith	tt0014827
1746422563	1940	1940	Calling Philo Vance	Calling Philo Vance	Clemens, William	William Clemens	tt0032299
1746578671	1951	1951	The Bushwhackers	The Bushwhackers	Amateau, Rod	Rod Amateau	tt0044460
1746500558	1926	1926	The Devil's Gulch	The Devil's Gulch	Nelson, Jack	Jack Nelson	tt0016786
1745309606	2005	2005	Rize	Rize	LaChapelle, David	David LaChapelle	tt0436724
1746209559	1968	1968	The Acid Eaters	The Acid Eaters	Elliott, B. Ron	Byron Mabe	tt0061329
1746577254	1962	1962	Moon Pilot	Moon Pilot	Neilson, James	James Neilson	tt0056249
1746555414	1956	1956	Hollywood or Bust	Hollywood or Bust	Tashlin, Frank	Frank Tashlin	tt0049322

1746574617	1936	1936	The Three Mesquiteers	The Three Mesquiteers	Taylor, Ray	Ray Taylor	tt0028372
1746542636	1924	1924	Is Love Everything?	Is Love Everything?	Cabanne, William Christy	Christy Cabanne	tt0015017
1746585482	1939	1939	Romance of the Redwoods	Romance of the Redwoods	Vidor, Charles	Charles Vidor	tt0031873
1746200031	1967	1967	For Love & Money	For Love and Money	Davis, Don	Donald A. Davis	tt0129097
1746464979	1942	1942	Eyes in the Night	Eyes in the Night	Zinnemann, Fred	Fred Zinnemann	tt0034711
1746514435	1910	1910	Ramona	Ramona	Griffith, D. W.	D.W. Griffith	tt0001371
1745372663	2010	2010	Greenberg	Greenberg	Baumbach, Noah	Noah Baumbach	tt1234654
1746520795	1926	1926	The Man From the West	The Man from the West	Rogell, Albert	Albert S. Rogell	tt0017108
1745358669	2009	2009	The Twilight Saga: New Moon	The Twilight Saga: New Moon	Weitz, Chris	Chris Weitz	tt1259571
1746584148	1936	1936	Jailbreak	Jailbreak	Grinde, Nick	Nick Grinde	tt0027814
1746582452	1937	1937	Love on Toast	Love on Toast	Dupont, E. A.	Ewald André Dupont	tt0030389
1769970454	1967	1967	Uncle Tomcat's House of Kittens	Uncle Tomcat's House of Kittens	Purdue, Gunther	Gunther Perdue	tt0260473
1746467439	1917	1917	Betty to the Rescue	Betty to the Rescue	Reicher, Frank	Frank Reicher	tt0007700
1746532851	1927	1927	Clancy's Kosher Wedding	Clancy's Kosher Wedding	Gillstrom, Arvid E.	Arvid E. Gillstrom	tt0017756
1746509312	1924	1923	Don't Call It Love	Don't Call It Love	de Mille, William C.	William C. de Mille	tt0014852
1746492599	1947	1947	Ladies' Man	Ladies' Man	Russell, William D.	William D. Russell	tt0039544
1746574550	1933	1933	Day of Reckoning	Day of Reckoning	Brabin, Charles	Charles Brabin	tt0023932
1746578697	1950	1950	The Tougher They Come	The Tougher They Come	Nazarro, Ray	Ray Nazarro	tt0043060
1746574658	1933	1933	The Power and the Glory	The Power and the Glory	Howard, William K.	William K. Howard	tt0024465
1746514691	1927	1927	Don Desperado	Don Desperado	Maloney, Leo	Leo D. Maloney	tt0130627
1746514244	1927	1927	If I Were Single	If I Were Single	Del Ruth, Roy	Roy Del Ruth	tt0018022
1746454014	1942	1942	Gallant Lady	Gallant Lady	Beaudine, William	William Beaudine	tt0034766
1745282900	2005	2005	Syriana	Syriana	Gaghan, Stephen	Stephen Gaghan	tt0365737
1745359437	2004	2004	Eurotrip	EuroTrip	Schaffer, Jeff	Jeff Schaffer	tt0356150
1746554888	1956	1956	Moby Dick	Moby Dick	Huston, John	John Huston	tt0049513
1746574973	1933	1933	Bed of Roses	Bed of Roses	LaCava, Gregory	Gregory La Cava	tt0023786
1746184716	1965	1965	Dear Brigitte	Dear Brigitte	Koster, Henry	Henry Koster	tt0059094
1746502232	1916	1916	Thrown to the Lions	Thrown to the Lions	Henderson, Lucius	Lucius Henderson	tt0007458
1746580213	1938	1938	Time Out for Murder	Time Out for Murder	Humberstone, H. Bruce	H. Bruce Humberstone	tt0030873
1746580217	1938	1938	The Invisible Menace	The Invisible Menace	Farrow, John	John Farrow	tt0030277
1746185662	1965	1965	The Greatest Story Ever Told	The Greatest Story Ever Told	Stevens, George	George Stevens	tt0059245
1745372417	2003	2003	Hollywood Homicide	Hollywood Homicide	Shelton, Ron	Ron Shelton	tt0329717
1746577531	1937	1937	Life Begins with Love	Life Begins with Love	McCarey, Raymond B.	Ray McCarey	tt0029143
1746435616	1942	1942	Tumbleweed Trail	Tumbleweed Trail	Stewart, Peter	Sam Newfield	tt0235121
1746454936	1945	1945	Delightfully Dangerous	Delightfully Dangerous	Lubin, Arthur	Arthur Lubin	tt0037636
1746514635	1915	1915	Buckshot John	Buckshot John	Bosworth, Hobart	Hobart Bosworth	tt0005032
1746581034	1960	1960	Tess of the Storm Country	Tess of the Storm Country	Guilfoyle, Paul	Paul Guilfoyle	tt0054376
1746584743	1928	1927	The Jazz Singer	The Jazz Singer	Crosland, Alan	Alan Crosland	tt0018037
1746453569	1944	1944	See Here, Private Hargrove	See Here, Private Hargrove	Ruggles, Wesley	Wesley Ruggles	tt0037256
1745359677	2001	2001	Joy Ride	Joy Ride	Dahl, John	John Dahl	tt0206314
1769971184	1969	1969	Ragina's Secrets	Ragina's Secrets	Hand, Fletcher	Fletcher Hand	tt0256298
1746574735	1937	1937	The Roaming Cowboy	The Roaming Cowboy	Hill, Robert	Robert F. Hill	tt0029493
1746408713	1985	1984	Night Patrol	Night Patrol	Kong, Jackie	Jackie Kong	tt0087795
1746475503	1944	1944	Hi, Good Lookin'!	Hi, Good Lookin'!	Lilley, Edward	Edward C. Lilley	tt0036917
1745358021	2009	2009	Brüno	Brüno	Charles, Larry	Larry Charles	tt0889583
1746234416	1966	1966	Stagecoach	Stagecoach	Douglas, Gordon	Gordon Douglas	tt0061024
1746508247	1927	1927	Polly of the Movies	Polly of the Movies	Pembroke, Scott	Scott Pembroke	tt0018270
1746542688	1926	1926	Racing Blood	Racing Blood	Richardson, Frank	Frank Richardson	tt0017303
1746475774	1947	1947	Green Dolphin Street	Green Dolphin Street	Saville, Victor	Victor Saville	tt0039437
1746514496	1916	1916	Jealousy	Jealousy	Davis, Will S.	Will S. Davis	tt0006879
1746574856	1934	1934	The Secret of the Chateau	Secret of the Chateau	Thorpe, Richard	Richard Thorpe	tt0025758
1745340931	2001	2001	Save the Last Dance	Save the Last Dance	Carter, Thomas	Thomas Carter	tt0206275
1746454694	1945	1945	Brewster's Millions	Brewster's Millions	Dwan, Allan	Allan Dwan	tt0037557
1746273013	1979	1979	Phantasm	Phantasm	Coscarelli, Don	Don Coscarelli	tt0079714
1745358518	2009	2009	State of Play	State of Play	Macdonald, Kevin	Kevin Macdonald	tt0473705
1746577292	1961	1961	Gun Fight	Gun Fight	Cahn, Edward L.	Edward L. Cahn	tt0054951
1746584415	1936	1936	Song of the Gringo	Song of the Gringo	McCarthy, John	John P. McCarthy	tt0028283

1746465271	1942	1942	X Marks the Spot	X Marks the Spot	Sherman, George	George Sherman	tt0035570	
1746570737	1933	1933	Whistling in the Dark	Whistling in the Dark	Nugent, Elliott	Elliott Nugent	tt0024769	
1746524759	1923	1923	The Woman With Four Faces	The Woman with Four Faces	Brenon, Herbert	Herbert Brenon	tt0014621	
1769970469	1967	1967	Scarf of Mist, Thigh of Satin	Scarf of Mist Thigh of Satin	Sarno, Joe	Joseph W. Sarno	tt0060945	
1746464861	1941	1941	The Bride Came C.O.D.	The Bride Came C.O.D.	Keighley, William	William Keighley	tt0033432	
1746553932	1965	1965	Harum Scarum	Harum Scarum	Nelson, Gene	Gene Nelson	tt0059255	
1746410665	1994	1994	I Love Trouble	I Love Trouble	Shyer, Charles	Charles Shyer	tt0110093	
1746454411	1997	1997	Gattaca	Gattaca	Niccol, Andrew	Andrew Niccol	tt0119177	
1746508711	1926	1926	The Fighting Cheat	The Fighting Cheat	Thorpe, Richard	Richard Thorpe	tt0016858	
1746525141	1920	1920	Uncle Sam of Freedom Ridge	Uncle Sam of Freedom Ridge	Beranger, George A.	George Beranger	tt0145550	
1746573364	1932	1932	Rain	Rain	Milestone, Lewis	Lewis Milestone	tt0023369	
1769970663	1968	1968	Wall of Flesh	The Wall of Flesh	Sarno, Joe	Joseph W. Sarno	tt0062468	
1746436697	1942	1942	Journey for Margaret	Journey for Margaret	Van Dyke, W. S.; Kline, Herbert	W.S. Van Dyke	tt0034923	
1746581594	1951	1951	The Wild Blue Yonder	The Wild Blue Yonder	Dwan, Allan	Allan Dwan	tt0044216	
1746563333	1951	1951	Mr. Texas	Mr. Texas	Ross, Dick	Dick Ross	tt7814732	
1746210718	1970	1970	Love Story	Love Story	Hiller, Arthur	Arthur Hiller	tt0066011	
1746508124	1925	1925	Was It Bigamy?	Was It Bigamy?	Hutchinson, Charles	Charles Hutchison	tt0016503	
1746514578	1916	1916	Her Maternal Right	Her Maternal Right	Thornby, Robert; Ince, John	John Ince	tt0157747	
1746574162	1957	1957	Jailhouse Rock	Jailhouse Rock	Thorpe, Richard	Richard Thorpe	tt0050556	
1746565202	1933	1933	The Son of Kong	The Son of Kong	Schoedsack, Ernest B.	Ernest B. Schoedsack	tt0024593	
1746501308	1916	1916	Tempest and Sunshine	Tempest and Sunshine	King, Carlton; Hughes, Warren	Warren Hughes	tt0159014	
1746585026	1940	1940	Dr. Christian Meets the Women	Dr. Christian Meets the Women	McGann, William	William C. McGann	tt0032411	
1746585259	1930	1930	Trigger Tricks	Trigger Tricks	Eason, Reaves	B. Reeves Eason	tt0021490	
1746530726	1950	1950	Kill or Be Killed	Kill or Be Killed	Nosseck, Max	Max Nosseck	tt0042640	
1746574260	1963	1963	Mary Mary	Mary, Mary	LeRoy, Mervyn	Mervyn LeRoy	tt0057293	
1746520927	1949	1949	House of Strangers	House of Strangers	Mankiewicz, Joseph L.	Joseph L. Mankiewicz	tt0041487	
1746571139	1935	1935	The Flame Within	The Flame Within	Goulding, Edmund	Edmund Goulding	tt0026363	
1746554198	1964	1964	The Best Man	The Best Man	Schaffner, Franklin J.	Franklin J. Schaffner	tt0057883	
1746514705	1915	1915	Aloha Oe	Aloha Oe	Stanton, Richard; Swickard, Charles	Richard Stanton	tt0004883	
1746542638	1924	1924	The Lure of Love	The Lure of Love	Dadmun, Leon E.	Leon Danmun	tt0015098	
1746422226	1995	1995	Now and Then	Now and Then	Glatter, Lesli Linka	Lesli Linka Glatter	tt0114011	
1746566859	1952	1952	The Captive City	The Captive City	Wise, Robert	Robert Wise	tt0044476	
1769970777	1912	1912	Yvonne the Foreign Spy	Yvonne, the Foreign Spy			tt1282508	
1746420388	1993	1992	Star Time	Star Time	Cassini, Alexander	Alexander Cassini	tt0105464	
1746500183	1945	1945	Youth on Trial	Youth on Trial	Boetticher, Oscar	Budd Boetticher	tt0038266	
1745272142	2000	2000	U-571	U 571	Mostow, Jonathan	Jonathan Mostow	tt0141926	
1746491808	1948	1948	Open Secret	Open Secret	Reinhardt, John	John Reinhardt	tt0040671	
1746485227	1918	1918	The Cruise of the Make-Believes	The Cruise of the Make Believes	Melford, George	George Melford	tt0008981	
1746584229	1935	1935	The White Cockatoo	The White Cockatoo	Crosland, Alan	Alan Crosland	tt0027207	
1746520051	1926	1926	The Dude Cowboy	The Dude Cowboy	Nelson, Jack	Jack Nelson	tt0016813	
1746532506	1922	1922	Gas, Oil and Water	Gas, Oil and Water	Ray, Charles	Charles Ray	tt0013165	
1746514210	1916	1916	The Unwritten Law	The Unwritten Law	Middleton, George E.	George E. Middleton	tt0159082	
1746566890	1952	1952	Operation Secret	Operation Secret	Seiler, Lewis	Lewis Seiler	tt0044999	
1746569868	1955	1955	Mister Roberts	Mister Roberts	Ford, John; LeRoy, Mervyn	John Ford	tt0048380	
1746582808	1935	1935	Swifty	Swifty	James, Alan	Alan James	tt0028332	
1746580856	1938	1938	Judge Hardy's Children	Judge Hardy's Children	Seitz, George B.	George B. Seitz	tt0030295	
1745309626	2005	2005	A History of Violence	A History of Violence	Cronenberg, David	David Cronenberg	tt0399146	
1769971129	1969	1969	The Stewardesses	The Stewardesses	Silliman, Alf	Al Silliman Jr.	tt0168192	
1746420134	1982	1982	Night Shift	Night Shift	Howard, Ron	Ron Howard	tt0084412	
1746580830	1938	1938	My Bill	My Bill	Farrow, John	John Farrow	tt0030479	
1746569785	1955	1955	Tall Man Riding	Tall Man Riding	Selander, Lesley	Lesley Selander	tt0048690	
1746420970	1992	1992	Toys	Toys	Levinson, Barry	Barry Levinson	tt0105629	
**1769969569**	**1912**	**1912**	**Into the Desert**	**Into the Desert**		**George Nichols**	**tt0415883**	1
1746475528	1946	1946	The Fabulous Suzanne	The Fabulous Suzanne	Sekely, Steve	Steve Sekely	tt0038513	
1746567105	1933	1933	Midnight Mary	Midnight Mary	Wellman, William	William A. Wellman	tt0024334	
1746565924	1957	1957	The Hellcats of the Navy	Hellcats of the Navy	Juran, Nathan	Nathan Juran	tt0050500	
1746574978	1934	1934	Black Moon	Black Moon	Neill, Roy William	Roy William Neill	tt0024895	

1746555575	1955	1955	This Island Earth	This Island Earth	Newman, Joseph	Joseph M. Newman	tt0047577
1746198750	1967	1967	The Big Mouth	The Big Mouth	Lewis, Jerry	Jerry Lewis	tt0061401
1746570060	1952	1952	Hellgate	Hellgate	Warren, Charles Marquis	Charles Marquis Warren	tt0044698
1746542755	1926	1926	Cupid's Knockout	Cupid's Knockout	Mitchell, Bruce	Bruce Mitchell	tt0016754
1746530744	1949	1949	Alimony	Alimony	Zeisler, Alfred	Alfred Zeisler	tt0041109
1746583739	1931	1931	The Gang Buster	The Gang Buster	Sutherland, A. Edward	A. Edward Sutherland	tt0021898
1746218017	1965	1965	Die, Monster, Die!	Die, Monster, Die!	Haller, Daniel	Daniel Haller	tt0059465
1746409133	1990	1990	Tremors	Tremors	Underwood, Ron	Ron Underwood	tt0100814
1746507737	1924	1924	Miami	Miami	Crosland, Alan	Alan Crosland	tt0015132
1746464936	1944	1944	Wild Horse Phantom	Wild Horse Phantom	Newfield, Sam	Sam Newfield	tt0037464
1746465412	1944	1944	Girl Rush	Girl Rush	Douglas, Gordon	Gordon Douglas	tt0036865
1746465154	1942	1942	A Gentleman After Dark	A Gentleman After Dark	Marin, Edwin L.	Edwin L. Marin	tt0034777
1746199517	1966	1966	Blindfold	Blindfold	Dunne, Philip	Philip Dunne	tt0058977
1746583638	1928	1928	The Fearless Rider	The Fearless Rider	Lewis, Edgar	Edgar Lewis	tt0018880
1746198509	1968	1968	The Hooked Generation	The Hooked Generation	Grefe, William	William Grefé	tt0063082
1746585226	1929	1929	The Studio Murder Mystery	The Studio Murder Mystery	Tuttle, Frank	Frank Tuttle	tt0020461
1746524906	1916	1916	Less Than the Dust	Less Than the Dust	Emerson, John	John Emerson	tt0006914
1745272360	2001	2001	Cats & Dogs	Cats & Dogs	Guterman, Lawrence	Lawrence Guterman	tt0239395
1745294026	2006	2005	American Gun	American Gun	Avelino, Aric	Aric Avelino	tt0416471
1745373788	2003	2003	Wonderland	Wonderland	Cox, James	James Cox	tt0335563
1746211269	1971	1971	Summer of '42	Summer of '42	Mulligan, Robert	Robert Mulligan	tt0067803
1746576914	1951	1951	Nevada Badmen	Nevada Badmen	Collins, Lewis	Lewis D. Collins	tt0043847
1746542682	1949	1949	Song of India	Song of India	Rogell, Albert S.	Albert S. Rogell	tt0041898
1746582636	1938	1938	Bulldog Drummond's Peril	Bulldog Drummond's Peril	Hogan, James	James P. Hogan	tt0029952
1746508175	1924	1924	The Phantom Horseman	The Phantom Horseman	Bradbury, Robert North	Robert N. Bradbury	tt0015226
1746561688	1965	1965	Wild Seed	Wild Seed	Hutton, Brian G.	Brian G. Hutton	tt0059912
1746583360	1951	1951	Bonanza Town	Bonanza Town	Sears, Fred F.	Fred F. Sears	tt0043353
1746423502	1999	1999	Being John Malkovich	Being John Malkovich	Jonze, Spike	Spike Jonze	tt0120601
1746570277	1934	1934	The Woman Condemned	The Woman Condemned	Reid, Wallace	Dorothy Davenport	tt0024782
1746574062	1960	1960	From the Terrace	From the Terrace	Robson, Mark	Mark Robson	tt0053841
1746523293	1916	1916	Phantom Fortunes	Phantom Fortunes	Scardon, Paul	Paul Scardon	tt0007179
1746500698	1925	1925	Phantom Shadows	Phantom Shadows	Grandon, Frank	Al Ferguson	tt0016219
1746454105	1942	1942	The Dawn Express	The Dawn Express	Herman, Albert	Albert Herman	tt0034637
1746520637	1949	1950	Woman in Hiding	Woman in Hiding	Gordon, Michael	Michael Gordon	tt0042052
1746513818	1923	1923	The Leopardess	The Leopardess	Kolker, Henry	Henry Kolker	tt0014195
1746583052	1935	1935	The Sagebrush Troubadour	Sagebrush Troubadour	Kane, Joseph	Joseph Kane	tt0026962
1746235877	1973	1973	The Friends of Eddie Coyle	The Friends of Eddie Coyle	Yates, Peter	Peter Yates	tt0070077
1746453521	1940	1940	The Man Who Wouldn't Talk	The Man Who Wouldn't Talk	Burton, David	David Burton	tt0032748
1746464981	1945	1945	A Song for Miss Julie	A Song for Miss Julie	Rowland, William	William Rowland	tt0038099
1746504582	1922	1922	The Power of Love	The Power of Love	Deverich, Nat	Nat G. Deverich	tt0013506
1746474498	1944	1944	Storm Over Lisbon	Storm Over Lisbon	Sherman, George	George Sherman	tt0037315
1746424603	1941	1941	The Great Swindle	The Great Swindle	Collins, Lewis D.	Lewis D. Collins	tt0033679
1746577978	1938	1938	Four Daughters	Four Daughters	Curtiz, Michael	Michael Curtiz	tt0030149
1746569837	1954	1954	Lucky Me	Lucky Me	Donohue, Jack	Jack Donohue	tt0047194
1746410691	1993	1992	Nitrate Kisses	Nitrate Kisses	Hammer, Barbara	Barbara Hammer	tt0107693
1746492063	1945	1945	Black Market Babies	Black Market Babies	Beaudine, William	William Beaudine	tt0037543
1746435706	1940	1940	The Invisible Woman	The Invisible Woman	Sutherland, A. Edward	A. Edward Sutherland	tt0032637
1745302610	2006	2005	Fuck	Fuck	Anderson, Steve	Steve Anderson	tt0486585
1746433736	1998	1997	Moon over Broadway	Moon Over Broadway	Hegedus, Chris; Pennebaker, D. A.	Chris Hegedus	tt0125412
1746574575	1935	1935	She Married Her Boss	She Married Her Boss	La Cava, Gregory	Gregory La Cava	tt0026986
1746198870	1965	1965	Indian Paint	Indian Paint	Foster, Norman	Norman Foster	tt0057182
1746422602	1991	1991	Teenage Mutant Ninja Turtles II: The Secret of th	Teenage Mutant Ninja Turtles II: The Secret c	Pressman, Michael	Michael Pressman	tt0103060
1746504425	1921	1921	Home-Keeping Hearts	Home Keeping Hearts	Ellis, Carlyle	Carlyle Ellis	tt0135451
1745374468	2002	2002	Divine Secrets of the Ya-Ya Sisterhood	Divine Secrets of the Ya Ya Sisterhood	Khouri, Callie	Callie Khouri	tt0279778
1746217975	1968	1968	Panic in the City	Panic in the City	Davis, Eddie	Eddie Davis	tt0062094
1746515989	1922	1922	The Trouper	The Trouper	Harris, Harry B.	Harry B. Harris	tt0013714
1769971102	1969	1969	Flesh of My Flesh	Flesh of My Flesh	Macready, Michael; Kelljan, Robert	Bob Kelljan	tt0218256

1746508978	1925	1925	The Substitute Wife	The Substitute Wife	May, Wilfred	Wilfred Noy	tt0016405	
1746525009	1915	1915	The Caprices of Kitty	The Caprices of Kitty	Smalley, Phillips	Phillips Smalley	tt0005055	
1746423991	1943	1943	Background to Danger	Background to Danger	Walsh, Raoul	Raoul Walsh	tt0035659	
1746497252	1918	1918	A Romance of the Underworld	A Romance of the Underworld	Kirkwood, James	James Kirkwood	tt0009562	
1746574151	1960	1960	The Enemy General	The Enemy General	Sherman, George	George Sherman	tt0053795	
1746520616	1925	1925	Where Romance Rides	Where Romance Rides	Hayes, Ward	Ward Hayes	tt0179512	
1746433563	1997	1997	Eve's Bayou	Eve's Bayou	Lemmons, Kasi	Kasi Lemmons	tt0119080	
1746578535	1959	1959	The Mating Game	The Mating Game	Marshall, George	George Marshall	tt0053054	
1746210072	1970	1970	Lovers and Other Strangers	Lovers and Other Strangers	Howard, Cy	Cy Howard	tt0066016	
1746491739	1944	1944	Ever Since Venus	Ever Since Venus	Dreifuss, Arthur	Arthur Dreifuss	tt0036804	
1746409684	1988	1988	Assault of the Killer Bimbos	Assault of the Killer Bimbos	Rosenberg, Anita	Anita Rosenberg	tt0092587	
1746515377	1920	1920	The Wall Street Mystery	The Wall Street Mystery	Collins, Tom	Tom Collins	tt0011836	
1745342649	2008	2008	The Incredible Hulk	The Incredible Hulk	Leterrier, Louis	Louis Leterrier	tt0800080	
1746580500	1939	1939	Double Deal	Double Deal	Dreifuss, Arthur	Arthur Dreifuss	tt0031245	
1746566490	1932	1932	Make Me a Star	Make Me a Star	Beaudine, William	William Beaudine	tt0023175	
1746501997	1915	1915	The Outcast	The Outcast	O'Brien, John B.	John B. O'Brien	tt0005850	
1746566979	1932	1932	Thirteen Women	Thirteen Women	Archainbaud, George	George Archainbaud	tt0023582	
1746580316	1939	1939	The House of Fear	The House of Fear	May, Joe	Joe May	tt0031449	
1745293417	2005	2005	War of the Worlds	War of the Worlds	Spielberg, Steven	Steven Spielberg	tt0407304	
1746514709	1915	1915	David Harum	David Harum		Allan Dwan	tt0005179	
1746473928	1946	1946	The Inner Circle	The Inner Circle	Ford, Phil	Philip Ford	tt0038640	
1746258311	1977	1977	The Last Remake of Beau Geste	The Last Remake of Beau Geste	Feldman, Marty	Marty Feldman	tt0076297	
1746496766	1917	1917	Susan's Gentleman	Susan's Gentleman	Stevens, Edwin	Edwin Stevens	tt0008645	
1746524213	1915	1915	The Penitentes	The Penitentes	Conway, John	Jack Conway	tt0005883	
1746516329	1920	1920	Double Speed	Double Speed	Wood, Sam	Sam Wood	tt0011125	
1746501109	1915	1915	The Plunderer	The Plunderer	Lewis, Edgar	Edgar Lewis	tt0005905	
1746440183	1997	1996	Albino Alligator	Albino Alligator	Spacey, Kevin	Kevin Spacey	tt0115495	
1746393146	1987	1987	The Glass Menagerie	The Glass Menagerie	Newman, Paul	Paul Newman	tt0093093	
1746582933	1936	1936	The White Angel	The White Angel	Dieterle, William	William Dieterle	tt0028499	
1769970684	1968	1968	Help Wanted Female	Help Wanted Female	Perkins, Harold	John Hayes	tt0063061	
**1746514872**	**1916**	**1916**	**The Footlights of Fate**	**The Footlights of Fate**	**Humphrey, William**	**William Humphrey**	**tt0156542**	1
1745272367	2000	2000	American Psycho	American Psycho	Harron, Mary	Mary Harron	tt0144084	
1746582347	1931	1931	Oklahoma Jim	Oklahoma Jim	Fraser, Harry	Harry L. Fraser	tt0022218	
1746577146	1950	1950	Vigilante Hideout	Vigilante Hideout	Brannon, Fred C.	Fred C. Brannon	tt0043106	
1746503248	1919	1919	False Gods	False Gods	Van (pseud. of Wally Van Nostrand),	Wally Van	tt0190392	
1746577542	1936	1936	After the Thin Man	After the Thin Man	Van Dyke, W. S.	W.S. Van Dyke	tt0027260	
1746436576	1940	1940	River's End	River's End	Enright, Ray	Ray Enright	tt0034113	
1746520319	1949	1949	The Wolf Hunters	The Wolf Hunters	Boetticher, Oscar	Budd Boetticher	tt0042050	
1746520060	1925	1925	Off the Highway	Off the Highway	Forman, Tom	Tom Forman	tt0016171	
1745396264	2008	2008	Frost/Nixon	Frost/Nixon	Howard, Ron	Ron Howard	tt0870111	
1746568164	1934	1934	Badge of Honor	Badge of Honor	Bennet, Spencer Gordon	Spencer Gordon Bennet	tt0024858	
1746555875	1954	1953	The Wild One	The Wild One	Benedek, Laslo	Laslo Benedek	tt0047677	
1746186403	1965	1965	Day of the Nightmare	Day of the Nightmare	Bushelman, John	John A. Bushelman	tt0059087	
1746503666	1920	1920	West Is West	West Is Best	Paul, Val	Phil Rosen	tt0222002	
1746562222	1954	1954	Riot in Cell Block 11	Riot in Cell Block 11	Siegel, Don	Don Siegel	tt0047417	
1746394427	1988	1988	1969	1969	Thompson, Ernest	Ernest Thompson	tt0094594	
1746545415	1940	1940	Bullets for Rustlers	Bullets for Rustlers	Nelson, Sam	Sam Nelson	tt0032293	
1746464829	1942	1942	Night Monster	Night Monster	Beebe, Ford	Ford Beebe	tt0035124	
1746270168	1978	1978	Laserblast	Laserblast	Rae, Michael	Michael Rae	tt0077834	
1746580323	1935	1935	Silk Hat Kid	Silk Hat Kid	Humberstone, H. Bruce	H. Bruce Humberstone	tt0026998	
1745293432	2005	2005	Junebug	Junebug	Morrison, Phil	Phil Morrison	tt0418773	
1746424459	1940	1940	Gambling on the High Seas	Gambling on the High Seas	Amy, George	George Amy	tt0032505	
1745358885	2010	2010	Bitter Feast	Bitter Feast	Maggio, Joe	Joe Maggio	tt1477835	
1746576939	1959	1959	Hound-Dog Man	Hound Dog Man	Siegel, Don	Don Siegel	tt0052906	
1769976183	1920	1919	Fireman, Save My Gal	Fireman, Save My Gal!	Hutchinson, Craig	Craig Hutchinson	tt0450674	
1746584200	1940	1940	The Notorious Elinor Lee	The Notorious Elinor Lee	Micheaux, Oscar	Oscar Micheaux	tt0139504	
1746562134	1953	1953	Flight to Tangier	Flight to Tangier	Warren, Charles Marquis	Charles Marquis Warren	tt0045774	

1746453649	1942	1942	So's Your Aunt Emma	So's Your Aunt Emma!	Yarbrough, Jean	Jean Yarbrough	tt0035051
1746582542	1935	1935	Rainbow's End	Rainbow's End	Spencer, Norval	Norval Spencer	tt0026909
1746199055	1969	1969	Futz	Futz	O'Horgan, Tom	Tom O'Horgan	tt0064354
1746475981	1947	1947	Web of Danger	Web of Danger	Ford, Philip	Philip Ford	tt0039972
1746436684	1940	1940	Hit Parade of 1941	Hit Parade of 1941	Auer, John H.	John H. Auer	tt0032600
1746475758	1944	1944	Cover Girl	Cover Girl	Vidor, Charles	Charles Vidor	tt0036723
1746562326	1956	1955	The Last Frontier	The Last Frontier	Mann, Anthony	Anthony Mann	tt0049431
1746573666	1932	1932	20,000 Years in Sing Sing	20,000 Years in Sing Sing	Curtiz, Michael	Michael Curtiz	tt0023731
1746520736	1925	1925	Wasted Lives	Wasted Lives	Gorman, John	John Gorman	tt0016504
1746208112	1968	1968	Don't Just Stand There!	Don't Just Stand There	Winston, Ron	Ron Winston	tt0062901
1746393738	1987	1987	Eddie Murphy Raw	Eddie Murphy: Raw	Townsend, Robert	Robert Townsend	tt0092948
1745372540	2002	2002	Mr. Deeds	Mr. Deeds	Brill, Steven	Steven Brill	tt0280590
1746410657	1992	1992	My Cousin Vinny	My Cousin Vinny	Lynn, Jonathan	Jonathan Lynn	tt0104952
1746580336	1938	1938	Bluebeard's Eighth Wife	Bluebeard's Eighth Wife	Lubitsch, Ernst	Ernst Lubitsch	tt0029929
1746584691	1930	1930	Spring Is Here	Spring Is Here	Dillon, John Francis	John Francis Dillon	tt0021413
1746420110	1981	1981	Under the Rainbow	Under the Rainbow	Rash, Steve	Steve Rash	tt0083254
1746572033	1932	1932	Mr. Robinson Crusoe	Mr. Robinson Crusoe	Sutherland, Edward	A. Edward Sutherland	tt0023243
1746210258	1970	1970	I Never Sang for My Father	I Never Sang for My Father	Cates, Gilbert	Gilbert Cates	tt0065872
1746578028	1935	1935	Top Hat	Top Hat	Sandrich, Mark	Mark Sandrich	tt0027125
1746566631	1932	1932	Attorney for the Defense	Attorney for the Defense	Cummings, Irving	Irving Cummings	tt0022644
1746210772	1973	1973	Jeremy	Jeremy	Barron, Arthur	Arthur Barron	tt0070238
1746393095	1988	1988	Ghost Town	Ghost Town	Governor, Richard	Richard McCarthy	tt0095215
1746570106	1934	1934	Ridin' Thru	Ridin' Thru	Samuels, Henri	Harry S. Webb	tt0025719
1746436379	1943	1943	False Faces	False Faces	Sherman, George	George Sherman	tt0035863
1746503908	1922	1922	Man's Law and God's	Man's Law and God's	Fox, Finis	Finis Fox	tt0333865
1746409710	1990	1990	Darkman	Darkman	Raimi, Sam	Sam Raimi	tt0099365
1746486163	1919	1919	The Man Who Stayed at Home	The Man Who Stayed at Home	Blaché, Herbert	Herbert Blaché	tt0010421
1746525073	1920	1920	Women Men Forget	Women Men Forget	Stahl, John M.	John M. Stahl	tt0011879
1769971169	1969	1969	Pieces	Pieces			tt0254690
1746409995	1985	1985	Runaway Train	Runaway Train	Konchalovsky, Andrei	Andrey Konchalovskiy	tt0089941
1746439118	1997	1997	Fierce Creatures	Fierce Creatures	Young, Robert; Schepisi, Fred	Fred Schepisi	tt0119115
1745302649	2007	2007	Redline	Redline	Cheng, Andy	Andy Cheng	tt0780595
1746570217	1933	1933	Ladies Must Love	Ladies Must Love	Dupont, E. A.	Ewald André Dupont	tt0024237
1746508417	1925	1925	Clothes Make the Pirate	Clothes Make the Pirate	Tourneur, Maurice	Maurice Tourneur	tt0015689
1746465386	1942	1942	I Married an Angel	I Married an Angel	Van Dyke, W. S.; Del Ruth, Roy	W.S. Van Dyke	tt0034882
1746567345	1932	1932	Doomed Battalion	The Doomed Battalion	Gardner, Cyril	Cyril Gardner	tt0022831
1746569834	1957	1957	Ride Out for Revenge	Ride Out for Revenge	Girard, Bernard	Bernard Girard	tt0050900
1746569768	1955	1955	Pete Kelly's Blues	Pete Kelly's Blues	Webb, Jack	Jack Webb	tt0048484
1746423572	1990	1990	Repossessed	Repossessed	Logan, Bob	Bob Logan	tt0100475
1746577757	1933	1933	Bureau of Missing Persons	Bureau of Missing Persons	Del Ruth, Roy	Roy Del Ruth	tt0023856
1745302477	2006	2006	United 93	United 93	Greengrass, Paul	Paul Greengrass	tt0475276
1746562363	1953	1953	The Glass Web	The Glass Web	Arnold, Jack	Jack Arnold	tt0045825
1746523965	1916	1916	The Fugitive	The Fugitive	Sullivan, Frederick	Frederick Sullivan	tt0162337
1745293461	2005	2004	Million Dollar Baby	Million Dollar Baby	Eastwood, Clint	Clint Eastwood	tt0405159
1746574105	1957	1957	The Delicate Delinquent	The Delicate Delinquent	McGuire, Don; Lewis, Jerry	Don McGuire	tt0050301
1746475543	1947	1947	Where the North Begins	Where the North Begins	Bretherton, Howard	Howard Bretherton	tt0039983
1745374030	2002	2002	Moonlight Mile	Moonlight Mile	Silberling, Brad	Brad Silberling	tt0179098
1746454085	1944	1944	Alaska	Alaska	Archainbaud, George	George Archainbaud	tt0036590
1746577575	1937	1937	Outlaws of the Prairie	Outlaws of the Prairie	Nelson, Sam	Sam Nelson	tt0030539
1746532732	1923	1923	Temptation	Temptation	Le Saint, Edward J.	Edward LeSaint	tt0014530
1746394528	1988	1988	The Great Outdoors	The Great Outdoors	Deutch, Howard	Howard Deutch	tt0095253
1769974393	1916	1916	Stronger Than Woman's Will	Stronger Than Woman's Will		J. Farrell MacDonald	tt0797935
1746503578	1922	1922	Head over Heels	Head Over Heels	Schertzinger, Victor; Bern, Paul	Paul Bern	tt0013221
1746502277	1916	1916	The Chaperon	The Chaperon	Berthelet, Arthur	Arthur Berthelet	tt0157455
1746436896	2000	2000	Drowning Mona	Drowning Mona	Gomez, Nick	Nick Gomez	tt0186045
1746567787	1933	1933	Walls of Gold	Walls of Gold	MacKenna, Kenneth	Kenneth MacKenna	tt0024745
1746565996	1954	1954	Phantom Stallion	Phantom Stallion	Keller, Harry	Harry Keller	tt0047345

1746569603	1956	1956	Pillars of the Sky	Pillars of the Sky	Marshall, George	George Marshall	tt0049619
1746499868	1945	1945	San Pietro	San Pietro	Huston, John	John Huston	tt0036630
1746578009	1938	1938	Gold Mine in the Sky	Gold Mine in the Sky	Kane, Joe	Joseph Kane	tt0030193
1746576843	1931	1931	Once a Lady	Once a Lady	McClintic, Guthrie	Guthrie McClintic	tt0022226
1746524223	1916	1916	The Man Who Stood Still	The Man Who Stood Still	Crane, Frank	Frank Hall Crane	tt0007017
1746208684	1969	1969	Pendulum	Pendulum	Schaefer, George	George Schaefer	tt0064797
1746421264	1995	1995	Cutthroat Island	Cutthroat Island	Harlin, Renny	Renny Harlin	tt0112760
1745393720	2009	2009	Dance Flick	Dance Flick	Wayans, Damien Dante	Damien Dante Wayans	tt1153706
1746454640	1942	1942	You Were Never Lovelier	You Were Never Lovelier	Seiter, William A.	William A. Seiter	tt0035583
1746575984	1910	1910	The Cowboy's Sweetheart and the Bandit	The Cowboy's Sweetheart and the Bandit		Theodore Wharton	tt1227834
1746569859	1960	1960	Pollyanna	Pollyanna	Swift, David	David Swift	tt0054195
1746497491	1920	1920	The Brute Master	The Brute Master	Marshall, Roy H.	Roy Marshall	tt0012010
1746492456	1947	1947	The Homestretch	The Homestretch	Humberstone, Bruce	H. Bruce Humberstone	tt0039471
1746562369	1953	1953	I, the Jury	I, the Jury	Essex, Harry	Harry Essex	tt0045902
1746520187	1924	1924	His Darker Self	His Darker Self	Noble, John W.	John W. Noble	tt0014989
1745357924	2002	2002	Enough	Enough	Apted, Michael	Michael Apted	tt0278435
1746515031	1915	1915	Niobe	Niobe	Ford, Hugh; Porter, Edwin S.	Hugh Ford	tt0005814
1746524670	1917	1917	The Great Bradley Mystery	The Great Bradley Mystery	Ridgley, Richard	Richard Ridgely	tt0175683
1745393832	2010	2010	Diary of a Wimpy Kid	Diary of a Wimpy Kid	Freudenthal, Thor	Thor Freudenthal	tt1196141
1745373494	2009	2008	Explicit Ills	Explicit Ills	Webber, Mark	Mark Webber	tt1095001
1746584247	1937	1937	Danger Patrol	Danger Patrol	Landers, Lew	Lew Landers	tt0028759
1746503469	1921	1921	After Midnight	After Midnight	Ince, Ralph	Ralph Ince	tt0011910
1746513717	1916	1916	The Sphinx	The Sphinx	Adolfi, Jack	John G. Adolfi	tt0007382
1746542624	1924	1924	Jack o' Clubs	Jack O'Clubs	Hill, Robert F.	Robert F. Hill	tt0015020
1746509547	1949	1948	The Judge Steps Out	The Judge Steps Out	Ingster, Boris	Boris Ingster	tt0041531
1746235332	1972	1972	Come Back Charleston Blue	Come Back Charleston Blue	Warren, Mark	Mark Warren	tt0068403
1746257758	1978	1978	Gray Lady Down	Gray Lady Down	Greene, David	David Greene	tt0077629
1746573843	1956	1956	Man Beast	Man Beast	Warren, Jerry	Jerry Warren	tt0049468
1745340013	2001	2001	Glitter	Glitter	Hall, Vondie Curtis	Vondie Curtis	tt0118589
1746453845	1941	1941	I'll Wait for You	I'll Wait for You	Sinclair, Robert B.	Robert B. Sinclair	tt0033748
1746584487	1929	1929	Desert Nights	Desert Nights	Nigh, William	William Nigh	tt0019811
1746584054	1939	1939	Range War	Range War	Selander, Lesley	Lesley Selander	tt0031838
1746580273	1939	1939	Mr. Moto Takes a Vacation	Mr. Moto Takes a Vacation	Foster, Norman	Norman Foster	tt0031676
1746574348	1958	1958	Summer Love	Summer Love	Haas, Charles	Charles F. Haas	tt0052258
1746573075	1931	1931	Man of the World	Man of the World	Wallace, Richard	Richard Wallace	tt0022119
1746554758	1964	1964	I'd Rather Be Rich	I'd Rather Be Rich	Smight, Jack	Jack Smight	tt0058222
1746408460	1992	1992	The Player	The Player	Altman, Robert	Robert Altman	tt0105151
1746394706	1985	1985	The New Kids	The New Kids	Cunningham, Sean S.	Sean S. Cunningham	tt0089679
1746584397	1937	1937	San Quentin	San Quentin	Bacon, Lloyd	Lloyd Bacon	tt0029511
1745359125	2004	2004	Torque	Torque	Kahn, Joseph	Joseph Kahn	tt0329691
1746567344	1933	1933	Sagebrush Trail	Sagebrush Trail	Schaefer, Armand	Armand Schaefer	tt0024516
1746436262	1999	1999	The Blair Witch Project	The Blair Witch Project	Myrick, Daniel ; Sanchez, Eduardo	Daniel Myrick	tt0185937
1746520215	1948	1948	Gunning for Justice	Gunning for Justice	Taylor, Ray	Ray Taylor	tt0040410
1746509026	1923	1923	In the West	In the West	Holt, George	George Holt	tt0124753
1746576865	1932	1932	The Man from Hell's Edges	The Man from Hell's Edges	Bradbury, Robert N.	Robert N. Bradbury	tt0023183
1746501038	1910	1910	The Princess and the Peasant	The Princess and the Peasant	Dawley, J. Searle	J. Searle Dawley	tt0001368
1746454299	1944	1944	Gypsy Wildcat	Gypsy Wildcat	Neill, Roy William	Roy William Neill	tt0036890
1745358586	2002	2001	Thirteen Conversations About One Thing	Thirteen Conversations About One Thing	Sprecher, Jill	Jill Sprecher	tt0268690
1746497682	1919	1919	Her Code of Honor	Her Code of Honor	Stahl, John M.	John M. Stahl	tt0010238
1746393112	1987	1987	The Hanoi Hilton	The Hanoi Hilton	Chetwynd, Lionel	Lionel Chetwynd	tt0093143
1746439147	1943	1943	Gangway for Tomorrow	Gangway for Tomorrow	Auer, John H.	John H. Auer	tt0035917
1746509064	1950	1950	Gunslingers	Gunslingers	Fox, Wallace W.	Wallace Fox	tt0042535
1746555786	1955	1955	Chief Crazy Horse	Chief Crazy Horse	Sherman, George	George Sherman	tt0047935
1746556005	1954	1954	Return from the Sea	Return from the Sea	Selander, Lesley	Lesley Selander	tt0047405
1746235064	1975	1975	Shampoo	Shampoo	Ashby, Hal	Hal Ashby	tt0073692
1746578006	1939	1939	Calling All Marines	Calling All Marines	Auer, John H.	John H. Auer	tt0031132
1746487574	1920	1920	Captain Swift	Captain Swift	Terriss, Tom	Tom Terriss	tt0188481

1746208866	1967	1967	Jack of Diamonds	Jack of Diamonds	Taylor, Don	Don Taylor	tt0061831
1746564272	1953	1953	The Clown	The Clown	Leonard, Robert Z.	Robert Z. Leonard	tt0044503
1746508961	1949	1949	Home in San Antone	Home in San Antone	Nazarro, Ray	Ray Nazarro	tt0041480
1746491892	1947	1947	The Exile	The Exile	Opuls, Max	Max Ophüls	tt0039360
1746576256	1932	1932	Broken Lullaby	Broken Lullaby	Lubitsch, Ernst	Ernst Lubitsch	tt0022725
1769971180	1969	1969	Animal Love	Animal Love	Kenny	Kenny	tt0210512
1746555523	1954	1954	Loophole	Loophole	Schuster, Harold	Harold D. Schuster	tt0047192
1746542712	1925	1925	Adventure	Adventure	Fleming, Victor	Victor Fleming	tt0015542
1746520940	1950	1950	The Asphalt Jungle	The Asphalt Jungle	Huston, John	John Huston	tt0042208
1746492428	1948	1948	The Miracle of the Bells	The Miracle of the Bells	Pichel, Irving	Irving Pichel	tt0040594
1746235905	1975	1975	Mandingo	Mandingo	Fleischer, Richard	Richard Fleischer	tt0073349
1746492417	1944	1944	A Night of Adventure	A Night of Adventure	Douglas, Gordon	Gordon Douglas	tt0037130
1746569916	1959	1959	The Beat Generation	The Beat Generation	Haas, Charles	Charles F. Haas	tt0052610
1746393991	1989	1989	Eat a Bowl of Tea	Eat a Bowl of Tea	Wang, Wayne	Wayne Wang	tt0097261
1746577578	1938	1938	Crime School	Crime School	Seiler, Lewis	Lewis Seiler	tt0030026
1745340323	2004	2004	Taking Lives	Taking Lives	Caruso, D. J.	D.J. Caruso	tt0364045
1746500635	1924	1924	Riding Double	Riding Double	Maloney, Leo	Leo D. Maloney	tt0015280
1746454874	1999	1998	The Adventures of Sebastian Cole	The Adventures of Sebastian Cole	Williams, Tod	Tod Williams	tt0168449
1746583678	1929	1929	The Arizona Kid	The Arizona Kid	Carpenter, Horace B.	Horace B. Carpenter	tt0019648
1746561915	1955	1955	Cell 2455, Death Row	Cell 2455, Death Row	Sears, Fred F.	Fred F. Sears	tt0047926
1746569550	1954	1954	Cattle Queen of Montana	Cattle Queen of Montana	Dwan, Allan	Allan Dwan	tt0046839
1745342247	2004	2003	Open Water	Open Water	Kentis, Chris	Chris Kentis	tt0374102
1746566920	1953	1953	Perils of the Jungle	Perils of the Jungle	Blair, George	George Blair	tt0046179
1746419173	1994	1994	A Low Down Dirty Shame	A Low Down Dirty Shame	Wayans, Keenen Ivory	Keenen Ivory Wayans	tt0110399
1746502865	1916	1916	Sherlock Holmes	Sherlock Holmes	Berthelet, Arthur	Arthur Berthelet	tt0007338
1746580479	1936	1936	China Clipper	China Clipper	Enright, Ray	Ray Enright	tt0027445
1746524922	1915	1915	The Golden Claw	The Golden Claw	Barker, Reginald	Reginald Barker	tt0005403
1746585151	1930	1930	One Romantic Night	One Romantic Night	Stein, Paul L.	Paul L. Stein	tt0021217
1746393285	1984	1984	Repo Man	Repo Man	Cox, Alex	Alex Cox	tt0087995
1746520468	1926	1926	The Gentle Cyclone	The Gentle Cyclone	Van Dyke, William S.	W.S. Van Dyke	tt0016916
1746454376	1940	1940	Forty Little Mothers	Forty Little Mothers	Berkeley, Busby	Busby Berkeley	tt0032487
1746523585	1915	1915	Should a Wife Forgive?	Should a Wife Forgive?		Henry King	tt0006039
1746523354	1916	1916	The Serpent	The Serpent	Walsh, Raoul A.	Raoul Walsh	tt0007325
1746520272	1925	1925	Infatuation	Infatuation	Cummings, Irving	Irving Cummings	tt0015946
1746467623	1917	1918	Hands Up!	Hands Up!	Browning, Tod; Lucas, Wilfred	Louis J. Gasnier	tt0221228
1746454632	1945	1945	The Topeka Terror	The Topeka Terror	Bretherton, Howard	Howard Bretherton	tt0038181
1746570037	1933	1933	Heroes for Sale	Heroes for Sale	Wellman, William A.	William A. Wellman	tt0024115
1746580632	1937	1937	Paid to Dance	Paid to Dance	Coleman, C. C.	Charles C. Coleman	tt0029366
1746515050	1916	1916	The Conquest of Canaan	The Conquest of Canaan	Irving, George	George Irving	tt0157479
1746579018	1962	1961	Madison Avenue	Madison Avenue	Humberstone, Bruce	H. Bruce Humberstone	tt0056209
1746500475	1924	1924	Let Not Man Put Asunder	Let Not Man Put Asunder	Blackton, J. Stuart	J. Stuart Blackton	tt0015062
1746495589	1917	1917	A Sleeping Memory	A Sleeping Memory	Baker, George D.	George D. Baker	tt0182428
1746514493	1923	1923	Robin Hood, Jr.	Robin Hood, Jr.	Bricker, Clarence	Clarence Bricker	tt0014411
1746578821	1951	1951	Air Cadet	Air Cadet	Pevney, Joseph	Joseph Pevney	tt0043270
1746583310	1951	1951	Heart of the Rockies	Heart of the Rockies	Witney, William	William Witney	tt0043626
1746492710	1944	1944	Ali Baba and the Forty Thieves	Ali Baba and the Forty Thieves	Lubin, Arthur	Arthur Lubin	tt0036591
1746582427	1935	1935	A Tale of Two Cities	A Tale of Two Cities	Conway, Jack	Jack Conway	tt0027075
1746503035	1923	1923	The Empty Cradle	The Empty Cradle	King, Burton	Burton L. King	tt0014017
1746424607	1941	1941	Dangerous Lady	Dangerous Lady	Ray, Bernard B.	Bernard B. Ray	tt0033510
1746524091	1923	1923	Playing Double	Playing Double		Dick Rush	tt0338347
1769970773	1912	1912	White Aprons	The White Aprons		Étienne Arnaud	tt0002580
1746504144	1919	1919	The Brute Breaker	The Brute Breaker	Reynolds, Lynn F.	Lynn Reynolds	tt0009972
1746582274	1951	1951	The Magic Face	The Magic Face	Tuttle, Frank	Frank Tuttle	tt0043771
1746569526	1955	1955	The Tall Men	The Tall Men	Walsh, Raoul	Raoul Walsh	tt0048691
1746570191	1952	1952	Tembo	Tembo	Hill, Howard	Howard Hill	tt0238655
1746582627	1937	1936	Great Guy	Great Guy	Blystone, John G.	John G. Blystone	tt0027697
1746578520	1951	1951	Kind Lady	Kind Lady	Sturges, John	John Sturges	tt0043709

1746554553	1964	1964	Becket	Becket	Glenville, Peter	Peter Glenville	tt0057877	
1746504429	1920	1920	The Midnight Bride	The Midnight Bride	Humphrey, William J.	William Humphrey	tt0011461	
1746500573	1926	1926	The Cave Man	The Caveman	Milestone, Lewis	Lewis Milestone	tt0016716	
1746454621	1941	1941	A Missouri Outlaw	A Missouri Outlaw	Sherman, George	George Sherman	tt0035071	
1746409701	1986	1986	Desert Bloom	Desert Bloom	Corr, Eugene	Eugene Corr	tt0090934	
1746532435	1928	1928	Making the Varsity	Making the Varsity	Wheeler, Cliff	Cliff Wheeler	tt0132297	
1746553831	1964	1964	A Global Affair	A Global Affair	Arnold, Jack	Jack Arnold	tt0058145	
1746491969	1943	1943	The Stranger from Pecos	The Stranger from Pecos	Hillyer, Lambert	Lambert Hillyer	tt0036394	
1746562181	1954	1954	Thunder Pass	Thunder Pass	McDonald, Frank	Frank McDonald	tt0047584	
1746435216	1941	1941	They Dare Not Love	They Dare Not Love	Whale, James	James Whale	tt0034276	
1746574808	1934	1934	We're Rich Again	We're Rich Again	Seiter, William A.	William A. Seiter	tt0025966	
1746523557	1916	1916	Tangled Fates	Tangled Fates	Vale, Travers	Travers Vale	tt0007426	
1746491708	1948	1948	The Noose Hangs High	The Noose Hangs High	Barton, Charles	Charles Barton	tt0040652	
1746436373	1942	1942	Timber	Timber!	Cabanne, Christy	Christy Cabanne	tt0035442	
1769969480	1965	1965	Freudus Sexualis	Freudus Sexualis	Starkey, William H.	William H. Starkey	tt0218981	
1746584889	1940	1940	Flowing Gold	Flowing Gold	Green, Alfred E.	Alfred E. Green	tt0032481	
1746542660	1949	1949	The Wyoming Bandit	The Wyoming Bandit	Ford, Philip	Philip Ford	tt0042055	
1746523295	1923	1923	Smilin' On	Smilin' On	Craft, William J.	William James Craft	tt0340360	
1746500289	1925	1925	The Re-creation of Brian Kent	The Re Creation of Brian Kent	Wood, Sam; Weil, Harry	Sam Wood	tt0016270	
1745339953	2003	2003	Mayor of the Sunset Strip	Mayor of the Sunset Strip	Hickenlooper, George	George Hickenlooper	tt0230512	
1746455292	1997	1997	The Edge	The Edge	Tamahori, Lee	Lee Tamahori	tt0119051	
1746465356	1941	1940	Second Chorus	Second Chorus	Potter, H. C.	H.C. Potter	tt0033029	
1745340463	2002	2001	Wendigo	Wendigo		Larry Fessenden	tt0275067	
1746409704	1983	1983	Sudden Impact	Sudden Impact	Eastwood, Clint	Clint Eastwood	tt0086383	
1746574469	1934	1934	She Loves Me Not	She Loves Me Not	Nugent, Elliott	Elliott Nugent	tt0025774	
1745357965	2004	2004	Spartan	Spartan	Mamet, David	David Mamet	tt0360009	
1746508437	1923	1923	High Speed Lee	High Speed Lee	Murphy, Dudley	Dudley Murphy	tt0329181	
1746409674	1984	1984	Places in the Heart	Places in the Heart	Benton, Robert	Robert Benton	tt0087921	
1746572545	1931	1931	Son of India	Son of India	Feyder, Jacques	Jacques Feyder	tt0022412	
1746516270	1921	1921	Flowing Gold	Flowing Gold	Franchon, Leonard	Leonard Franchon	tt0325396	
1746567585	1952	1952	My Wife's Best Friend	My Wife's Best Friend	Sale, Richard	Richard Sale	tt0044942	
1746436566	1941	1941	The Bandit Trail	The Bandit Trail	Killy, Edward	Edward Killy	tt0033375	
1769971980	1969	1969	Marcy	Marcy	Sarno, Joseph W.	Joseph W. Sarno	tt0064637	
1746578067	1939	1939	Gunga Din	Gunga Din	Stevens, George	George Stevens	tt0031398	
1746516353	1920	1920	Black Is White	Black Is White	Giblyn, Charles	Charles Giblyn	tt0010995	
1746566035	1955	1955	Ma and Pa Kettle at Waikiki	Ma and Pa Kettle at Waikiki	Sholem, Lee	Lee Sholem	tt0048328	
1746570839	1934	1934	The Painted Veil	The Painted Veil	Boleslawski, Richard	Richard Boleslawski	tt0025617	
1746565902	1953	1953	Loose in London	Loose in London	Bernds, Edward	Edward Bernds	tt0046011	
1746487725	1921	1921	Marry the Poor Girl	Marry the Poor Girl	Ingraham, Lloyd	Lloyd Ingraham	tt0012449	
1746524239	1915	1914	How Cissy Made Good	How Cissy Made Good	Baker, George D.	George D. Baker	tt0005520	
1746573749	1958	1958	The Lone Ranger and the Lost City of Gold	The Lone Ranger and the Lost City of Gold	Selander, Lesley	Lesley Selander	tt0051876	
1746584663	1928	1928	Danger Street	Danger Street	Ince, Ralph	Ralph Ince	tt0018814	
1746269707	1973	1973	From the Mixed-Up Files of Mrs. Basil E. Frankwe	From the Mixed Up Files of Mrs. Basil E. Fran	Cook, Fielder	Fielder Cook	tt0070079	
1745293895	2006	2005	Sir! No Sir!	Sir! No Sir!	Zeiger, David	David Zeiger	tt0469589	
1746507909	1950	1949	Battleground	Battleground	Wellman, William	William A. Wellman	tt0041163	
1746514458	1916	1916	The Gods of Fate	The Gods of Fate	Pratt, Jack	Jack Pratt	tt0157707	
1746574013	1962	1962	Mr. Hobbs Takes a Vacation	Mr. Hobbs Takes a Vacation	Koster, Henry	Henry Koster	tt0056255	
1746512760	1910	1910	Wifie's Mamma	Wifie's Mamma			tt1799147	
1746218753	1973	1973	Enter the Dragon	Enter the Dragon	Clouse, Robert	Robert Clouse	tt0070034	
1746500248	1923	1923	Innocence	Innocence	Le Saint, Edward J.	Edward LeSaint	tt0014154	
**1746576005**	**1910**	**1910**	**The Flower of the Ranch**	**The Flower of the Ranch**		**Gilbert M. 'Broncho Billy' Anderso**	**tt0001220**	1
1746501777	1910	1910	The Silent Message	The Silent Message		Gilbert M. 'Broncho Billy' Anderson	tt0001395	
1745340165	2003	2003	Duplex	Duplex	DeVito, Danny	Danny DeVito	tt0266489	
1746561554	1954	1954	Rails into Laramie	Rails Into Laramie	Hibbs, Jesse	Jesse Hibbs	tt0047389	
1746583514	1928	1928	The Drifting Kid	The Drifting Kid			tt0322087	
1746569591	1956	1956	Nightmare	Nightmare	Shane, Maxwell	Maxwell Shane	tt0049553	
1746566843	1952	1952	Francis Goes to West Point	Francis Goes to West Point	Lubin, Arthur	Arthur Lubin	tt0044633	

1746464880	1945	1945	Salty O'Rourke	Salty O'Rourke	Walsh, Raoul	Raoul Walsh	tt0038047	
1746467779	1917	1917	A Mormon Maid	A Mormon Maid	Leonard, Robert	Robert Z. Leonard	tt0008319	
1746569883	1958	1958	Torpedo Run	Torpedo Run	Pevney, Joseph	Joseph Pevney	tt0052303	
1746582688	1937	1937	Murder Goes to College	Murder Goes to College	Riesner, Charles	Charles Reisner	tt0029273	
1746574605	1933	1933	The Fighting President	The Fighting President			tt0183083	
1746465310	1942	1942	Overland to Deadwood	Overland to Deadwood	Berke, William	William Berke	tt0203046	
1745373938	2009	2009	Beyond a Reasonable Doubt	Beyond a Reasonable Doubt	Hyams, Peter	Peter Hyams	tt1183251	
1746571085	1952	1952	Rose of Cimarron	Rose of Cimarron	Keller, Harry	Harry Keller	tt0045106	
1746553924	1964	1964	Light Fantastic	Light Fantastic	McCarty, Robert	Robert McCarty	tt0145045	
1746584553	1931	1931	Her Majesty Love	Her Majesty, Love	Dieterle, William	William Dieterle	tt0021952	
1746583315	1929	1929	The Divine Lady	The Divine Lady	Lloyd, Frank	Frank Lloyd	tt0019824	
1746420862	1995	1995	Mad Love	Mad Love	Bird, Antonia	Antonia Bird	tt0113729	
1746569802	1956	1956	Mohawk	Mohawk	Neumann, Kurt	Kurt Neumann	tt0049515	
1746573215	1931	1931	Seas Beneath	Seas Beneath	Ford, John	John Ford	tt0022353	
1746524877	1922	1922	The Clean Up	The Clean Up			tt0355332	
1746511742	1916	1916	The Pawn of Fate	Pawn of Fate	Tourneur, Maurice	Maurice Tourneur	tt0007161	
1745375128	2009	2009	Cold Souls	Cold Souls	Barthes, Sophie	Sophie Barthes	tt1127877	
1746514795	1914	1914	The Criminal Path	The Criminal Path	Davis, Will S.	Will S. Davis	tt0002772	
1745359045	2010	2010	Frozen	Frozen	Green, Adam	Adam Green	tt1323045	
1746454449	1942	1942	Blondie Goes to College	Blondie Goes to College	Strayer, Frank R.	Frank R. Strayer	tt0034525	
1746572862	1931	1930	Free Love	Free Love	Henley, Hobart	Hobart Henley	tt0020901	
1746570231	1952	1952	Lady in the Iron Mask	Lady in the Iron Mask	Murphy, Ralph	Ralph Murphy	tt0044818	
1746584628	1929	1929	The Drake Case	The Drake Case	Laemmle, Edward	Edward Laemmle	tt0019834	
1745271694	2000	2000	Rules of Engagement	Rules of Engagement	Friedkin, William	William Friedkin	tt0160797	
1746474969	1948	1948	Angel in Exile	Angel in Exile	Dwan, Allan; Ford, Philip	Allan Dwan	tt0040094	
1746582397	1929	1929	The Redeeming Sin	The Redeeming Sin		Howard Bretherton	tt0020319	
1746577697	1933	1932	Bachelor Mother	Bachelor Mother	Hutchison, Charles	Charles Hutchison	tt0129768	
1746577588	1933	1933	Broadway Thru a Keyhole	Broadway Thru a Keyhole	Sherman, Lowell	Lowell Sherman	tt0023847	
1746410021	1983	1983	Star 80	Star 80	Fosse, Bob	Bob Fosse	tt0086355	
1746256124	1976	1976	Stay Hungry	Stay Hungry	Rafelson, Bob	Bob Rafelson	tt0075268	
1746217161	1968	1968	High, Wild and Free	High, Wild and Free			tt0252506	
**1746580268**	**1939**	**1939**	**Hollywood Cavalcade**	**Hollywood Cavalcade**	**Cummings, Irving**	**Irving Cummings**	**tt0031433**	1
1746524481	1922	1922	Fighting Hearts	Fighting Hearts			tt0357661	
1746235825	1978	1978	The Alpha Incident	The Alpha Incident	Rebane, Bill	Bill Rebane	tt0075662	
**1769970167**	**1912**	**1912**	**The Other Woman**	**The Other Woman**		**George L. Cox**	**tt0441409**	1
1746567799	1932	1932	The Miracle Man	The Miracle Man	McLeod, Norman	Norman Z. McLeod	tt0023228	
1746502560	1915	1915	Colonel Carter of Cartersville	Colonel Carter of Cartersville	Hansell, Howell	Howell Hansel	tt0005107	
1746523476	1926	1926	Beau Geste	Beau Geste	Brenon, Herbert	Herbert Brenon	tt0016634	
1746583665	1930	1930	Call of the Desert	Call of the Desert	McGowan, J. P.	J.P. McGowan	tt0020723	
1746410191	1983	1983	Amityville 3-D	Amityville 3 D	Fleischer, Richard	Richard Fleischer	tt0085159	
1746577209	1962	1962	The Bashful Elephant	The Bashful Elephant	McGowan, Dorrell; McGowan, Stuar	Dorrell McGowan	tt0055777	
1769969706	1911	1911	Little Dove's Romance	Little Dove's Romance		Fred J. Balshofer	tt0250503	
1746514542	1915	1915	Heartaches	Heartaches	Kaufman, Joseph	Joseph Kaufman	tt0005455	
1746492390	1948	1948	Man-Eater of Kumaon	Man Eater of Kumaon	Haskin, Byron	Byron Haskin	tt0040567	
1746581281	1961	1961	Mysterious Island	Mysterious Island	Endfield, Cy	Cy Endfield	tt0055207	
1746421588	1993	1993	Mr. Nanny	Mr. Nanny	Gottlieb, Michael	Michael Gottlieb	tt0107612	
1746502436	1916	1916	The Chalice of Sorrow	The Chalice of Sorrow	Ingram, Rex	Rex Ingram	tt0006495	
1746392909	1984	1984	Top Secret!	Top Secret!	Abrahams, Jim; Zucker, David; Zucke	Jim Abrahams	tt0088286	
1746233348	1968	1968	Sergeant Ryker	Sergeant Ryker	Kulik, Buzz	Buzz Kulik	tt0063584	
1746512584	1923	1923	A Million To Burn	A Million to Burn	Parke, William	William Parke	tt0014280	
1769970504	1967	1967	The Touch of Her Flesh	The Touch of Her Flesh	Marsh, Julian	Michael Findlay	tt0062385	
1746580805	1938	1938	Torchy Gets Her Man	Torchy Gets Her Man	Beaudine, William	William Beaudine	tt0030884	
1746520973	1949	1949	South of Rio	South of Rio	Ford, Philip	Philip Ford	tt0041906	
1746574800	1933	1933	Dangerously Yours	Dangerously Yours	Tuttle, Frank	Frank Tuttle	tt0023928	
1746520226	1924	1924	The Humming Bird	The Humming Bird	Olcott, Sidney	Sidney Olcott	tt0015004	
1746492426	1947	1947	Lighthouse	Lighthouse	Wisbar, Frank	Frank Wisbar	tt0039567	
1746420961	1997	1997	Kull the Conqueror	Kull the Conqueror	Nicolella, John	John Nicolella	tt0119484	

1746508909	1925	1925	The Knockout	The Knockout	Hillyer, Lambert	Lambert Hillyer	tt0015983
1746512443	1916	1916	The Gates of Eden	The Gates of Eden	Collins, John H.	John H. Collins	tt0006711
1746543070	1915	1915	Saved from the Harem	Saved from the Harem	Melville, Wilbert	Wilbert Melville	tt0006007
1746586158	1940	1940	Free, Blonde and 21	Free, Blonde and 21	Cortez, Ricardo	Ricardo Cortez	tt0032492
1746567791	1952	1952	Just This Once	Just This Once	Weis, Don	Don Weis	tt0044781
1746208634	1966	1966	Madame X	Madame X	Rich, David Lowell	David Lowell Rich	tt0060645
1746583131	1937	1937	Blazing Sixes	Blazing Sixes	Smith, Noel	Noel M. Smith	tt0028639
1745340970	2003	2003	The Haunted Mansion	The Haunted Mansion	Minkoff, Rob	Rob Minkoff	tt0338094
1746502828	1916	1916	He Fell in Love with His Wife	He Fell in Love with His Wife	Taylor, William D.	William Desmond Taylor	tt0006765
1745309369	2005	2005	Just Like Heaven	Just Like Heaven	Waters, Mark	Mark Waters	tt0425123
1746423291	1991	1991	The Addams Family	The Addams Family	Sonnenfeld, Barry	Barry Sonnenfeld	tt0101272
1746525143	1919	1919	The Winchester Woman	The Winchester Woman	Ruggles, Wesley	Wesley Ruggles	tt0010898
1745357904	2004	2004	White Chicks	White Chicks	Wayans, Keenen Ivory	Keenen Ivory Wayans	tt0381707
1746561761	1956	1955	Artists and Models	Artists and Models	Tashlin, Frank	Frank Tashlin	tt0047840
1746497608	1920	1920	The Prince Chap	The Prince Chap	de Mille, William	William C. de Mille	tt0011601
1746583445	1931	1931	Branded Men	Branded Men	Rosen, Phil	Phil Rosen	tt0021694
1746185846	1969	1969	Stiletto	Stiletto	Kowalski, Bernard L.	Bernard L. Kowalski	tt0065038
1746584299	1935	1935	Jalna	Jalna	Cromwell, John	John Cromwell	tt0026542
1746507790	1927	1927	Tarzan and the Golden Lion	Tarzan and the Golden Lion	McGowan, J. P.	J.P. McGowan	tt0018464
1746421984	1994	1994	House Party 3	House Party 3	Meza, Eric	Eric Meza	tt0110064
1746465169	1942	1942	Rodeo Rhythm	Rodeo Rhythm	Newmeyer, Fred	Fred C. Newmeyer	tt0034123
1746424195	1941	1941	Country Fair	Country Fair	McDonald, Frank	Frank McDonald	tt0033492
1746410005	1992	1992	The Cutting Edge	The Cutting Edge	Glaser, Paul M.	Paul Michael Glaser	tt0104040
1746513690	1916	1916	The Rise of Susan	The Rise of Susan	Taylor, S. E. V.	Stanner E.V. Taylor	tt0007265
1746439707	1996	1996	The Truth About Cats and Dogs	The Truth About Cats & Dogs	Lehmann, Michael	Michael Lehmann	tt0117979
1746566157	1958	1958	Fort Dobbs	Fort Dobbs	Douglas, Gordon	Gordon Douglas	tt0051628
1746435568	1940	1940	Married and in Love	Married and in Love	Farrow, John	John Farrow	tt0032764
1746492581	1944	1944	Brand of the Devil	Brand of the Devil	Fraser, Harry	Harry L. Fraser	tt0036669
1746502468	1916	1916	Molly Make-Believe	Molly Make Believe	Dawley, J. Searle	J. Searle Dawley	tt0007088
1746584679	1930	1930	The Lady of Scandal	The Lady of Scandal	Franklin, Sidney	Sidney Franklin	tt0021042
1746577783	1938	1938	Prairie Moon	Prairie Moon	Staub, Ralph	Ralph Staub	tt0030620
1746423484	1996	1996	A Time to Kill	A Time to Kill	Schumacher, Joel	Joel Schumacher	tt0117913
1746578742	1960	1959	The Angry Red Planet	The Angry Red Planet	Melchior, Ib	Ib Melchior	tt0052564
1746583731	1931	1931	Five and Ten	Five and Ten		Robert Z. Leonard	tt0021874
1746578499	1959	1959	Island of Lost Women	Island of Lost Women	Tuttle, Frank W.	Frank Tuttle	tt0052932
1746525180	1921	1921	Woman's Place	Woman's Place	Fleming, Victor	Victor Fleming	tt0012862
1746575053	1934	1934	Hell Bent for Love	Hell Bent for Love	Lederman, D. Ross	D. Ross Lederman	tt0025231
1746394450	1985	1985	Prizzi's Honor	Prizzi's Honor	Huston, John	John Huston	tt0089841
1746454845	1946	1946	Boston Blackie and the Law	Boston Blackie and the Law	Lederman, D. Ross	D. Ross Lederman	tt0038377
1746583133	1937	1937	The Road Back	The Road Back	Whale, James	James Whale	tt0029491
1746586155	1939	1939	Chasing Danger	Chasing Danger	Cortez, Richard	Ricardo Cortez	tt0031151
1746474972	1944	1944	The Town Went Wild	The Town Went Wild	Murphy, Ralph	Ralph Murphy	tt0037388
1745342869	2003	2003	Radio	Radio	Tollin, Mike	Michael Tollin	tt0316465
1746577028	1951	1950	A Wonderful Life	A Wonderful Life	Beaudine, William	William Beaudine	tt0297468
1746453513	1943	1943	Truck Busters	Truck Busters	Eason, B. Reeves	B. Reeves Eason	tt0036460
1746569580	1956	1956	The Wild Party	The Wild Party	Horner, Harry	Harry Horner	tt0049955
1746582569	1938	1938	Guilty Trails	Guilty Trails	Waggner, George	George Waggner	tt0030206
1746514587	1923	1923	Rupert of Hentzau	Rupert of Hentzau	Heerman, Victor	Victor Heerman	tt0014424
1746211103	1973	1972	Ciao Manhattan	Ciao Manhattan	Palmer, John; Weisman, David	John Palmer	tt0068379
1746259493	1978	1978	A Wedding	A Wedding	Altman, Robert	Robert Altman	tt0078481
1746569518	1958	1958	I Married a Woman	I Married a Woman	Kanter, Hal	Hal Kanter	tt0051757
1746520535	1926	1926	Red Hot Hoofs	Red Hot Hoofs	De Lacey, Robert	Robert De Lacey	tt0017318
1746515384	1917	1917	The Wildcat	The Wildcat	McDonald, Sherwood	Sherwood MacDonald	tt0156181
1746423746	1998	1998	Dirty Work	Dirty Work	Saget, Bob	Bob Saget	tt0120654
1746410437	1992	1992	A Few Good Men	A Few Good Men	Reiner, Rob	Rob Reiner	tt0104257
1746566116	1958	1958	Missile Monsters	Missile Monsters	Brannon, Fred C.	Fred C. Brannon	tt0051935
1746498662	1921	1921	Ever Since Eve	Ever Since Eve	Mitchell, Howard M.	Howard M. Mitchell	tt0012139

1746422599	1994	1994	Mr. Write	Mr. Write	Loventhal, Charlie	Charlie Loventhal	tt0110587
1746500514	1925	1925	The Girl of Gold	The Girl of Gold	Ince, John	John Ince	tt0133802
1746570460	1934	1934	Harold Teen	Harold Teen	Roth, Murray	Murray Roth	tt0025221
1746437389	1943	1943	Cowboy Commandos	Cowboy Commandos	Luby, S. Roy	S. Roy Luby	tt0035760
1746573412	1932	1932	Beauty Parlor	Beauty Parlor	Thorpe, Richard	Richard Thorpe	tt0022661
1746500361	1924	1924	White Man	White Man	Gasnier, Louis	Louis J. Gasnier	tt0015491
1746573819	1956	1956	The Great Locomotive Chase	The Great Locomotive Chase	Lyon, Francis	Francis D. Lyon	tt0049279
1746219016	1980	1980	Nine to Five	Nine to Five	Higgins, Colin	Colin Higgins	tt0080319
1746501255	1914	1914	John Barleycorn	John Barleycorn	Bosworth, Hobart; Haydon, J. Charle	Hobart Bosworth	tt0004175
1746423738	1994	1994	Clerks	Clerks	Smith, Kevin	Kevin Smith	tt0109445
1746566006	1957	1956	The Rainmaker	The Rainmaker	Anthony, Joseph	Joseph Anthony	tt0049653
1746570254	1933	1933	I Love That Man	I Love That Man	Brown, Harry Joe	Harry Joe Brown	tt0024158
1746577201	1950	1950	My Blue Heaven	My Blue Heaven	Koster, Henry	Henry Koster	tt0042767
1746585188	1930	1930	Murder Will Out	Murder Will Out	Badger, Clarence	Clarence G. Badger	tt0021163
1746235360	1973	1973	Savage!	Savage!	Santiago, Cirio H.	Cirio H. Santiago	tt0070639
1746574096	1962	1962	Walk on the Wild Side	Walk on the Wild Side	Dmytryk, Edward	Edward Dmytryk	tt0056671
1746504382	1922	1922	Once Upon a Time	Once Upon a Time	Owen, Ruth Bryan	Ruth Bryan Owen	tt4578178
1746580205	1938	1938	Campus Confessions	Campus Confessions	Archainbaud, George	George Archainbaud	tt0029965
1746496325	1918	1918	The Transgressor	The Transgressor	Levering, Joseph	Joseph Levering	tt0190083
1746584076	1937	1937	Think Fast, Mr. Moto	Think Fast, Mr. Moto	Foster, Norman	Norman Foster	tt0029660
1746514832	1910	1910	Two Boys in Blue	Two Boys in Blue	Turner, Otis	Otis Turner	tt0346071
1746504061	1917	1917	Barbary Sheep	Barbary Sheep	Tourneur, Maurice	Maurice Tourneur	tt0007678
1746453760	1943	1943	Rookies in Burma	Rookies in Burma	Goodwins, Leslie	Leslie Goodwins	tt0036317
1746420730	1993	1993	RoboCop 3	RoboCop 3	Dekker, Fred	Fred Dekker	tt0107978
1746577625	1936	1936	Murder on a Bridle Path	Murder on a Bridle Path	Killy, Edward; Hamilton, William	William Hamilton	tt0028003
1746566188	1958	1958	Damn Citizen	Damn Citizen	Gordon, Robert	Robert Gordon	tt0051515
1746585283	1930	1930	The Break Up	The Break Up	Robertson, Jack	Jack Robertson	tt0370368
1746454010	1943	1943	Claudia	Claudia	Goulding, Edmund	Edmund Goulding	tt0035742
1746199188	1968	1968	Have You Heard of the San Francisco Mime Troup	Have You Heard of the San Francisco Mime T	Lenzer, Don; Wardenburg, Fred	Don Lenzer	tt1608627
1746508228	1950	1950	Cherokee Uprising	Cherokee Uprising	Collins, Lewis	Lewis D. Collins	tt0042328
1746502190	1910	1910	A Salutary Lesson	A Salutary Lesson	Griffith, D. W.	D.W. Griffith	tt0001390
1746573477	1932	1932	You Said a Mouthful	You Said a Mouthful	Bacon, Lloyd	Lloyd Bacon	tt0023716
1746583301	1950	1950	The Nevadan	The Nevadan	Douglas, Gordon	Gordon Douglas	tt0042782
1746422252	1998	1998	Without Limits	Without Limits	Towne, Robert	Robert Towne	tt0119934
1746394687	1984	1984	The World of Tomorrow	The World of Tomorrow	Johnson, Tom; Bird, Lance	Lance Bird	tt0184038
1746515032	1910	1910	The Miser's Child	The Miser's Child		Sidney Olcott	tt0001323
1746437318	1943	1943	Isle of Forgotten Sins	Isle of Forgotten Sins	Ulmer, Edgar G.	Edgar G. Ulmer	tt0036046
1746561903	1957	1957	Peyton Place	Peyton Place	Robson, Mark	Mark Robson	tt0050839
1746577891	1936	1936	Aces Wild	Aces Wild	Fraser, Harry	Harry L. Fraser	tt0028550
1746465252	1947	1947	The Case of the Baby Sitter	The Case of the Baby Sitter	Hillyer, Lambert	Lambert Hillyer	tt0039249
1746271199	1978	1978	Fingers	Fingers	Toback, James	James Toback	tt0077549
1746574901	1933	1933	The Sphinx	The Sphinx	Rosen, Phil	Phil Rosen	tt0024605
1746574924	1937	1937	Music for Madame	Music for Madame	Blystone, John	John G. Blystone	tt0029277
1746219079	1972	1972	Black Rodeo	Black Rodeo	Kanew, Jeff	Jeff Kanew	tt0068283
1746582031	1928	1928	The Woman From Moscow	The Woman from Moscow	Berger, Ludwig	Ludwig Berger	tt0018592
1746507894	1926	1926	Sparrows	Sparrows	Beaudine, William	William Beaudine	tt0017423
1746585359	1940	1940	Young Buffalo Bill	Young Buffalo Bill	Kane, Joseph	Joseph Kane	tt0033287
1746465085	1941	1941	Peer Gynt	Peer Gynt	Bradley, David	David Bradley	tt0034010
1769974249	1916	1916	The Chain of Evidence	The Chain of Evidence		Walter V. Coyle	tt0245044
1746554463	1964	1964	What's Up Front	What's Up Front!	Wehling, Bob	Bob Wehling	tt0057679
1746500169	1949	1949	The Undercover Man	The Undercover Man	Lewis, Joseph H.	Joseph H. Lewis	tt0042006
1746436357	1941	1941	Wyoming Wildcat	Wyoming Wildcat	Sherman, George	George Sherman	tt0034404
1745359950	2004	2004	Seed of Chucky	Seed of Chucky	Mancini, Don	Don Mancini	tt0387575
1746509486	1950	1950	Guilty of Treason	Guilty of Treason	Feist, Felix	Felix E. Feist	tt0041437
1746511750	1928	1928	The Lion and the Mouse	The Lion and the Mouse	Bacon, Lloyd	Lloyd Bacon	tt0019100
1746235443	1973	1973	Lost Horizon	Lost Horizon	Jarrott, Charles	Charles Jarrott	tt0070337
1746453568	1943	1943	Dead Man's Gulch	Dead Man's Gulch	English, John	John English	tt0035783

1746583358	1929	1929	Mexicali Rose	Mexicali Rose	Kenton, Erle C.	Erle C. Kenton	tt0020166
1746585209	1930	1930	The Sea God	The Sea God	Abbott, George	George Abbott	tt0021346
1746501322	1910	1910	The Red Girl and the Child	The Red Girl and the Child	Young Deer, James	James Young Deer	tt0234581
1746585113	1930	1930	Seven Days Leave	Seven Days Leave	Wallace, Richard	Richard Wallace	tt0021357
1746500275	1927	1927	Silk Stockings	Silk Stockings	Ruggles, Wesley	Wesley Ruggles	tt0018403
1746493897	1910	1910	Unreasonable Jealousy	Unreasonable Jealousy			tt0360136
1746569808	1958	1958	Fort Bowie	Fort Bowie	Koch, Howard W.	Howard W. Koch	tt0051627
1746198495	1969	1969	The Vixens	The Vixens	Cort, Harvey	Harvey Cort	tt0248603
1746498334	1921	1921	A Knight of the West	A Knight of the West	McKenzie, Robert	Robert McKenzie	tt0126371
1746555487	1955	1955	High Society	High Society	Beaudine, William	William Beaudine	tt0048166
1746453969	1997	1997	The Postman	The Postman	Costner, Kevin	Kevin Costner	tt0119925
1746409697	1987	1987	Dirty Dancing	Dirty Dancing	Ardolino, Emile	Emile Ardolino	tt0092890
1746585488	1940	1940	On the Spot	On the Spot	Bretherton, Howard	Howard Bretherton	tt0032868
1746394513	1979	1979	Squeeze Play	Squeeze Play	Weil, Samuel	Lloyd Kaufman	tt0081552
1745395782	2008	2007	Frownland	Frownland	Bronstein, Ronald	Ronald Bronstein	tt0970935
1746434614	1992	1992	The Mambo Kings	The Mambo Kings	Glimcher, Arne	Arne Glimcher	tt0104802
1745342309	2008	2008	Hancock	Hancock	Berg, Peter	Peter Berg	tt0448157
1746582306	1951	1951	Mask of the Avenger	Mask of the Avenger	Karlson, Phil; Pichel, Irving	Phil Karlson	tt0043790
1746554936	1964	1964	Honeymoon of Horror	Honeymoon of Horror	Meyer, Irwin	Irwin Meyer	tt0058205
1746577860	1937	1936	Dodge City Trail	Dodge City Trail	Coleman, C. C.	Charles C. Coleman	tt0003854
1746583847	1931	1931	Wild West Whoopee	Wild West Whoopee	Horner, Robert J.	Robert J. Horner	tt0022563
1746453831	1940	1940	Hot Steel	Hot Steel	Cabanne, Christy	Christy Cabanne	tt0032608
1769969704	1911	1911	Mutt and Jeff Break Into Society	Mutt and Jeff Break Into Society			tt1235156
1746584504	1931	1931	The Tip-Off	The Tip Off	Rogell, Albert	Albert S. Rogell	tt0022484
1746218917	1975	1975	Night Moves	Night Moves	Penn, Arthur	Arthur Penn	tt0073453
1746569601	1953	1954	Dragon's Gold	Dragon's Gold	Wisberg, Aubrey; Pollexfen, Jack	Jack Pollexfen	tt0207433
1746577134	1962	1962	What Ever Happened to Baby Jane?	What Ever Happened to Baby Jane?	Aldrich, Robert	Robert Aldrich	tt0056687
1746513447	1916	1916	Dust	Dust	Sloman, Edward	Edward Sloman	tt0157561
1746573745	1957	1957	The Buckskin Lady	The Buckskin Lady	Hittleman, Carl K.	Carl K. Hittleman	tt0050215
1746582249	1928	1928	Tenderloin	Tenderloin	Curtiz, Michael	Michael Curtiz	tt0019453
1746422231	1999	1999	Love Stinks	Love Stinks	Franklin, Jeff	Jeff Franklin	tt0188863
1746580468	1937	1937	The 13th Man	The 13th Man	Nigh, William	William Nigh	tt0028360
1746583270	1930	1930	Dangerous Paradise	Dangerous Paradise	Wellman, William	William A. Wellman	tt0020810
1746565298	1932	1932	Scandal for Sale	Scandal for Sale	Mack, Russell	Russell Mack	tt0023426
1746581932	1930	1930	Let's Go Native	Let's Go Native	McCarey, Leo	Leo McCarey	tt0021061
1746524938	1923	1923	White Tiger	White Tiger	Browning, Tod	Tod Browning	tt0014606
1746570023	1952	1952	Room for One More	Room for One More	Taurog, Norman	Norman Taurog	tt0045102
1746566130	1954	1954	A Star Is Born	A Star Is Born	Cukor, George	George Cukor	tt0047522
1746497287	1919	1919	Hawthorne of the U.S.A.	Hawthorne of the U.S.A.	Cruze, James	James Cruze	tt0010219
1746514209	1916	1916	His Lordship	His Lordship	McKim, Edwin	Edwin McKim	tt0204377
1746421233	1994	1994	No Escape	No Escape	Campbell, Martin	Martin Campbell	tt0110678
1746520348	1924	1924	Ace of Cactus Range	Ace of Cactus Range	Dixon, Denver; Andrus, Malon	Victor Adamson	tt0014645
1746565962	1956	1956	Flight to Hong Kong	Flight to Hong Kong	Newman, Joseph M.	Joseph M. Newman	tt0049219
1746210722	1973	1973	Walking Tall	Walking Tall	Karlson, Phil	Phil Karlson	tt0070895
1746453996	1996	1995	Killer: A Journal of Murder	Killer: A Journal of Murder	Metcalfe, Tim	Tim Metcalfe	tt0113542
1746453572	1942	1942	Mississippi Gambler	Mississippi Gambler	Rawlins, John	John Rawlins	tt0035070
1746216726	1969	1969	Run, Angel, Run!	Run, Angel, Run!	Starrett, Jack	Jack Starrett	tt0064911
1746507765	1927	1927	The Magic Flame	The Magic Flame	King, Henry	Henry King	tt0018129
1746572242	1931	1931	Resurrection	Resurrection	Carewe, Edwin	Edwin Carewe	tt0022307
1746585129	1929	1929	Bellamy Trial	Bellamy Trial	Bell, Monta	Monta Bell	tt0018685
1746584017	1939	1939	Woman Doctor	Woman Doctor	Salkow, Sidney	Sidney Salkow	tt0032140
1746573960	1956	1956	The Fastest Gun Alive	The Fastest Gun Alive	Rouse, Russell	Russell Rouse	tt0049201
1746525098	1920	1920	Miss Nobody	Miss Nobody	Grandon, Francis J.	Francis J. Grandon	tt0011470
1746434633	1989	1989	Ghostbusters II	Ghostbusters II	Reitman, Ivan	Ivan Reitman	tt0097428
1746465352	1947	1947	Sinbad the Sailor	Sinbad, the Sailor	Wallace, Richard	Richard Wallace	tt0039826
1746514780	1923	1923	Battling Bates	Battling Bates	Cullison, Webster	Webster Cullison	tt0127452
1746514323	1915	1915	Enoch Arden	Enoch Arden	Cabanne, William Christy	Christy Cabanne	tt0005246

1769970670	1966	1966	Honey	Honey	Denby, Jerry	Jerry Denby	tt0219803
1746500647	1924	1924	The Cowboy and the Flapper	The Cowboy and the Flapper	Neitz, Alvin J.	Alan James	tt0014804
1746565987	1954	1954	Hell Below Zero	Hell Below Zero	Robson, Mark	Mark Robson	tt0047072
1746496540	1920	1920	The Stolen Kiss	The Stolen Kiss	Webb, Kenneth	Kenneth S. Webb	tt0130285
1746514702	1916	1916	The Question	The Question	Handworth, Harry	Harry Handworth	tt0158878
1746577828	1937	1937	All over Town	All Over Town	Horne, James	James W. Horne	tt0028568
1746580904	1937	1937	Mama Steps Out	Mama Steps Out	Seitz, George B.	George B. Seitz	tt0029196
1746500159	1925	1925	The Little French Girl	The Little French Girl	Brenon, Herbert	Herbert Brenon	tt0016030
1746582846	1938	1938	Love on a Budget	Love on a Budget	Leeds, Herbert I.	Herbert I. Leeds	tt0030390
1746465326	1942	1942	Fingers at the Window	Fingers at the Window	Lederer, Charles	Charles Lederer	tt0034732
1746419763	1989	1988	Pumpkinhead	Pumpkinhead	Winston, Stan	Stan Winston	tt0095925
1746501864	1916	1916	Pay Dirt	Pay Dirt	King, Henry	Henry King	tt0163145
1746464990	1947	1947	The Private Affairs of Bel Ami	The Private Affairs of Bel Ami	Lewin, Albert	Albert Lewin	tt0039735
1746582361	1930	1930	True to the Navy	True to the Navy	Tuttle, Frank	Frank Tuttle	tt0021492
1746500218	1950	1950	The Kid from Texas	The Kid from Texas	Neumann, Kurt	Kurt Neumann	tt0042638
1746514080	1915	1915	Just Out of College	Just Out of College	Irving, George	George Irving	tt0005583
1746474138	1948	1948	The Luck of the Irish	The Luck of the Irish	Koster, Henry	Henry Koster	tt0040553
1746198065	1968	1968	Cargo of Love	Cargo of Love	Holden, Anton	Anton Holden	tt0062780
1746492242	1948	1948	June Bride	June Bride	Windust, Bretaigne	Bretaigne Windust	tt0040499
1746435013	1992	1992	South Central	South Central	Anderson, Steve	Stephen Milburn Anderson	tt0105450
1746573727	1958	1958	The Key	The Key	Reed, Carol	Carol Reed	tt0051816
1746578661	1961	1961	The Ladies' Man	The Ladies Man	Lewis, Jerry	Jerry Lewis	tt0055069
1746523287	1915	1915	Midnight at Maxim's	Midnight at Maxim's	Sargent, George L.	George L. Sargent	tt0005740
1746392729	1982	1982	Death Wish II	Death Wish II	Winner, Michael	Michael Winner	tt0082250
1746570565	1934	1934	Mandalay	Mandalay	Curtiz, Michael	Michael Curtiz	tt0025461
1746550194	1955	1955	The Big Tip Off	The Big Tip Off	McDonald, Frank	Frank McDonald	tt0047881
1746580731	1935	1935	Devil Dogs of the Air	Devil Dogs of the Air	Bacon, Lloyd	Lloyd Bacon	tt0026275
1746577439	1958	1958	The Spider	The Spider	Gordon, Bert I.	Bert I. Gordon	tt0051570
1746475894	1944	1944	Tahiti Nights	Tahiti Nights	Jason, Will	Will Jason	tt0038147
1746454064	1940	1940	Riders of Pasco Basin	Riders of Pasco Basin	Taylor, Ray	Ray Taylor	tt0032990
1746572087	1932	1932	Strange Interlude	Strange Interlude	Leonard, Robert Z.	Robert Z. Leonard	tt0023523
1746500121	1945	1945	Song of the Prairie	Song of the Prairie	Nazarro, Ray	Ray Nazarro	tt0038102
1746582405	1938	1938	The Lady Objects	The Lady Objects	Kenton, Erle C.	Erle C. Kenton	tt0030340
1769970057	1912	1912	The Mother of the Ranch	The Mother of the Ranch		Arthur Mackley	tt0002376
1746580545	1935	1935	Coyote Trails	Coyote Trails	Ray, B. B.	Bernard B. Ray	tt0026244
1746584641	1930	1929	Dark Skies	Dark Skies	Webb, Harry S.	Harry S. Webb	tt0019804
1746559821	1965	1965	The Nest of the Cuckoo Birds	The Nest of the Cuckoo Birds	Williams, Bert	Bert Williams	tt0361953
1746569926	1954	1954	Prisoner of War	Prisoner of War	Marton, Andrew	Andrew Marton	tt0047369
1746491842	1947	1947	Big Town After Dark	Big Town After Dark	Thomas, William C.	William C. Thomas	tt0039187
1746439135	1943	1943	High Explosive	High Explosive	McDonald, Frank	Frank McDonald	tt0035999
1746508220	1926	1925	Riding for Life	Riding for Life	Wright, Mack V.	Mack V. Wright	tt0156960
1746499942	1923	1923	The Ghost Patrol	The Ghost Patrol	Ross, Nat	Nat Ross	tt0014080
1746419623	1982	1982	The World According to Garp	The World According to Garp	Hill, George Roy	George Roy Hill	tt0084917
1746577619	1937	1937	God's Country and the Man	God's Country and the Man	Bradbury, Robert N.	Robert N. Bradbury	tt0028940
1746433761	1996	1996	Homeward Bound II: Lost in San Francisco	Homeward Bound II: Lost in San Francisco	Ellis, David R.	David R. Ellis	tt0116552
1746491894	1948	1948	Blood on the Moon	Blood on the Moon	Wise, Robert	Robert Wise	tt0040175
1746423193	1940	1940	Love, Honor, and Oh-Baby!	Love, Honor and Oh Baby!	Lamont, Charles	Charles Lamont	tt0032731
1746419614	1993	1993	Mr. Jones	Mr. Jones	Figgis, Mike	Mike Figgis	tt0107611
1746454846	1945	1945	Three's a Crowd	Three's a Crowd	Selander, Lesley	Lesley Selander	tt0038169
1746576312	1931	1931	The Spider	The Spider	Menzies, William C.; MacKenna, Ken	Kenneth MacKenna	tt0022421
1746248814	1975	1975	The Happy Hooker	The Happy Hooker	Sgarro, Nicholas	Nicholas Sgarro	tt0073091
1746502880	1916	1916	The Thousand Dollar Husband	The Thousand Dollar Husband	Young, James	James Young	tt0007452
1746419979	1940	1940	Wolf of New York	Wolf of New York	McGann, William	William C. McGann	tt0033270
1746577191	1962	1962	Gigot	Gigot	Kelly, Gene	Gene Kelly	tt0056017
1746585391	1939	1939	Winter Carnival	Winter Carnival	Riesner, Charles F.	Charles Reisner	tt0032132
1746408907	1985	1985	Mask	Mask	Bogdanovich, Peter	Peter Bogdanovich	tt0089560
1746500370	1924	1924	The Guilty One	The Guilty One	Henabery, Joseph	Joseph Henabery	tt0014963

1746503656	1917	1917	Should She Obey?	Should She Obey?	Siegmann, George A.	George Siegmann	tt0182421	
1746410395	1991	1991	King Ralph	King Ralph	Ward, David S.	David S. Ward	tt0102216	
1746569607	1955	1955	Pearl of the South Pacific	Pearl of the South Pacific	Dwan, Allan	Allan Dwan	tt0048474	
1746420214	1983	1983	The Star Chamber	The Star Chamber	Hyams, Peter	Peter Hyams	tt0086356	
1746523278	1910	1910	The Perversity of Fate	The Perversity of Fate		Sidney Olcott	tt0393590	
1746500458	1925	1925	The Red Kimona	The Red Kimona	Lang, Walter	Walter Lang	tt0016276	
1746516130	1922	1922	The Dungeon	The Dungeon	Micheaux, Oscar	Oscar Micheaux	tt0130644	
1746523335	1927	1927	Blazing Days	Blazing Days	Wyler, William	William Wyler	tt0017682	
1746198745	1967	1967	Good Times	Good Times	Friedkin, William	William Friedkin	tt0061720	
1746408671	1986	1986	Band of the Hand	Band of the Hand	Glaser, Paul Michael	Paul Michael Glaser	tt0090693	
1746561876	1956	1956	Dakota Incident	Dakota Incident	Foster, Lewis R.	Lewis R. Foster	tt0049118	
1746464797	1940	1940	The Man from Tumbleweeds	The Man from Tumbleweeds	Lewis, Joseph H.	Joseph H. Lewis	tt0032750	
1746421312	1999	1999	The Limey	The Limey	Soderbergh, Steven	Steven Soderbergh	tt0165854	
1746508880	1926	1926	The Waning Sex	The Waning Sex	Leonard, Robert Z.	Robert Z. Leonard	tt0017526	
1746566206	1953	1953	The Sea Around Us	The Sea Around Us	Allen, Irwin	Irwin Allen	tt0044009	
1746524117	1927	1927	The Broken Gate	The Broken Gate	McKay, James C.	James C. McKay	tt0017707	
1746580690	1936	1936	Go-Get-'Em-Haines	Go Get 'Em, Haines	Newfield, Sam	Sam Newfield	tt0164611	
1746200200	1969	1969	King, Murray	King, Murray	Hoffman, David; Gordon, Jonathan	Jonathan Gordon	tt0064544	
1746583606	1930	1930	Conspiracy	Conspiracy	Cabanne, Christy	Christy Cabanne	tt0020785	
1746249048	1973	1973	Steel Arena	Steel Arena	Lester, Mark L.	Mark L. Lester	tt0070730	
1746562247	1953	1953	The Magnetic Monster	The Magnetic Monster	Siodmak, Curt	Curt Siodmak	tt0046026	
1746454388	1943	1943	Thundering Trails	Thundering Trails	English, John	John English	tt0036437	
1746524591	1914	1914	The Only Son	The Only Son	DeMille, Cecil B.; Apfel, Oscar; Mille,	Oscar Apfel	tt0004434	
1746455826	1998	1998	Senseless	Senseless	Spheeris, Penelope	Penelope Spheeris	tt0120820	
1746410216	1981	1981	Caveman	Caveman	Gottlieb, Carl	Carl Gottlieb	tt0082146	
1746515025	1916	1916	The Blue Envelope Mystery	The Blue Envelope Mystery	North, Wilfrid	Wilfrid North	tt0006442	
1746555761	1957	1957	Mister Rock and Roll	Mister Rock and Roll	Dubin, Charles	Charles S. Dubin	tt0050711	
1746499996	1949	1949	Lost Boundaries	Lost Boundaries	Werker, Alfred L.	Alfred L. Werker	tt0041600	
1746492864	1947	1947	Buck Privates Come Home	Buck Privates Come Home	Barton, Charles T.	Charles Barton	tt0039226	
1746437265	1941	1941	The Officer and the Lady	The Officer and the Lady	White, Sam	Sam White	tt0033971	
1746571444	1932	1932	Those We Love	Those We Love	Florey, Robert	Robert Florey	tt0023587	
1769970044	1912	1912	The Passing	The Passing	Nichols, George O.	George Nichols	tt0414354	
1746474494	1944	1944	Lady in the Death House	Lady in the Death House	Sekely, Steve	Steve Sekely	tt0037001	
1746566341	1954	1953	Crime Wave	Crime Wave	DeToth, Andre	André De Toth	tt0046878	
1746515085	1918	1918	Blindfolded	Blindfolded	West, Raymond B.	Raymond B. West	tt0182836	
1746420989	1992	1992	Stay Tuned	Stay Tuned	Hyams, Peter	Peter Hyams	tt0105466	
1746523365	1915	1915	Double Trouble	Double Trouble	Cabanne, William Christy	Christy Cabanne	tt0005214	
1746409998	1981	1981	Bustin' Loose	Bustin' Loose	Scott, Oz	Oz Scott	tt0082121	
**1746502168**	**1922**	**1922**	**French Heels**	**French Heels**	**Hollywood, Edwin L.**	**Edwin L. Hollywood**	**tt0013154**	1
1746515777	1922	1922	The Cub Reporter	The Cub Reporter	Dillon, Jack	John Francis Dillon	tt0013042	
1746577058	1951	1950	Everybody's Girl	Everybody's Girl		Lillian Hunt	tt0136823	
1745342193	2010	2010	Paranormal Activity 2	Paranormal Activity 2	Williams, Tod	Tod Williams	tt1536044	
1746516630	1921	1921	Miss 139	Miss 139	Rolfe, B. A.	B.A. Rolfe	tt0133971	
1746421616	1991	1991	Grand Canyon	Grand Canyon	Kasdan, Lawrence	Lawrence Kasdan	tt0101969	
1745272488	2000	2000	28 Days	28 Days	Thomas, Betty	Betty Thomas	tt0191754	
1746454676	1944	1944	Casanova Brown	Casanova Brown	Wood, Sam	Sam Wood	tt0036699	
1746566326	1953	1953	The Story of Three Loves	The Story of Three Loves		Vincente Minnelli	tt0046374	
1746574561	1936	1936	Jaws of the Jungle	Jaws of the Jungle		Eddie Granemann	tt0126184	
1746485047	1918	1918	Tempered Steel	Tempered Steel	Ince, Ralph	Ralph Ince	tt0009685	
1746582348	1930	1930	Girl of the Port	Girl of the Port	Glennon, Bert	Bert Glennon	tt0020922	
1746504414	1917	1917	The Show Down	The Show Down	Reynolds, Lynn F.	Lynn Reynolds	tt0008568	
1746394474	1992	1992	Single White Female	Single White Female	Schroeder, Barbet	Barbet Schroeder	tt0105414	
1746408445	1985	1985	Murphy's Romance	Murphy's Romance	Ritt, Martin	Martin Ritt	tt0089643	
1746578713	1960	1960	Squad Car	Squad Car	Leftwich, Ed	Ed Leftwich	tt0055472	
1746235684	1978	1978	Every Which Way But Loose	Every Which Way But Loose	Fargo, James	James Fargo	tt0077523	
1746569611	1953	1953	Second Chance	Second Chance	Maté, Rudy	Rudolph Maté	tt0046288	
1746578914	1951	1951	Mister Universe	Mister Universe	Lerner, Joseph	Joseph Lerner	tt0043823	

1746419780	1982	1982	Parasite	Parasite	Band, Charles	Charles Band	tt0084472
1746393972	1987	1987	Fatal Beauty	Fatal Beauty	Holland, Tom	Tom Holland	tt0093011
1746561801	1955	1955	Killer's Kiss	Killer's Kiss	Kubrick, Stanley	Stanley Kubrick	tt0048254
1746184112	1910	1910	The Bad Man's Last Deed	The Bad Man's Last Deed	Anderson, G. M.	Gilbert M. 'Broncho Billy' Anderson	tt0001125
1746473892	1944	1944	Take It Big	Take It Big	McDonald, Frank	Frank McDonald	tt0037340
1746409245	1989	1989	Second Sight	Second Sight	Zwick, Joel	Joel Zwick	tt0098276
1746500305	1925	1925	Kivalina of the Ice Lands	Kivalina of the Ice Lands	Rossman, Earl	Earl Rossman	tt0333730
1746524955	1927	1927	For Ladies Only	For Ladies Only	Pembroke, Scott; Lehrman, Henry	Henry Lehrman	tt0017897
1746436239	1990	1989	Blue Steel	Blue Steel	Bigelow, Kathryn	Kathryn Bigelow	tt0099160
1746578672	1958	1958	Home Before Dark	Home Before Dark	LeRoy, Mervyn	Mervyn LeRoy	tt0051732
1769977747	1986	1986	Club Life	Club Life	Vane, Norman Thaddeus	Norman Thaddeus Vane	tt0088929
1745393818	2009	2009	Taking Woodstock	Taking Woodstock	Lee, Ang	Ang Lee	tt1127896
1746577490	1959	1959	Alias Jesse James	Alias Jesse James	McLeod, Norman Z.	Norman Z. McLeod	tt0052545
1746453636	1943	1944	Know Your Ally: Britain	Know Your Ally: Britain		Anthony Veiller	tt1128215
1746509052	1925	1925	Seven Sinners	Seven Sinners	Milestone, Lewis	Lewis Milestone	tt0016334
1746574713	1933	1933	Meet the Baron	Meet the Baron	Lang, Walter	Walter Lang	tt0024320
1746562167	1956	1956	Bandido	Bandido!	Fleischer, Richard	Richard Fleischer	tt0048983
1746584811	1929	1928	The Shady Lady	The Shady Lady	Griffith, Edward H.	Edward H. Griffith	tt0020391
1746584349	1936	1936	Little Lord Fauntleroy	Little Lord Fauntleroy	Cromwell, John	John Cromwell	tt0027893
1746465192	1942	1942	Undercover Man	Undercover Man	Selander, Lesley	Lesley Selander	tt0035487
1746585131	1928	1928	Honeymoon	Honeymoon	Golden, Robert A.	Robert A. Golden	tt0019016
1769974305	1916	1916	The Manicure Girl	The Manicure Girl	King, Burton L.	Burton L. King	tt0812308
1746502921	1923	1923	Flaming Youth	Flaming Youth	Dillon, John Francis	John Francis Dillon	tt0014045
1746524450	1916	1916	The Pretenders	The Pretenders	Baker, George D.; Hundt, Charles J.	Bobby Burns	tt0922567
1746464869	1942	1942	Danger in the Pacific	Danger in the Pacific	Collins, Lewis D.	Lewis D. Collins	tt0034633
1746487133	1919	1919	The Girl Dodger	The Girl Dodger	Storm, Jerome	Jerome Storm	tt0010167
1746584326	1937	1937	The Man Who Found Himself	The Man Who Found Himself	Landers, Lew	Lew Landers	tt0028603
1746584796	1929	1929	Young Nowheres	Young Nowheres	Lloyd, Frank	Frank Lloyd	tt0020612
1746475281	1946	1946	Accomplice	Accomplice	Colmes, Walter	Walter Colmes	tt0038285
1746235609	1973	1973	Jimi Hendrix	Jimi Hendrix		Joe Boyd	tt0070242
1746408495	1981	1980	Maniac	Maniac	Lustig, William	William Lustig	tt0081114
1746581060	1951	1951	The Prowler	The Prowler	Losey, Joseph	Joseph Losey	tt0043938
1746434941	1990	1990	Hamlet	Hamlet	Zeffirelli, Franco	Franco Zeffirelli	tt0099726
1746500369	1926	1926	Prowlers of the Night	Prowlers of the Night	Laemmle, Ernst	Ernst Laemmle	tt0017294
1746585009	1940	1940	The Long Voyage Home	The Long Voyage Home	Ford, John	John Ford	tt0032728
1746504111	1920	1920	The Hidden Code	The Hidden Code	L'Estrange, Richard	Richard L'Estrange	tt0195780
1746582222	1931	1931	Smart Woman	Smart Woman	La Cava, Gregory	Gregory La Cava	tt0022404
1746474413	1947	1947	Beat the Band	Beat the Band	Auer, John H.	John H. Auer	tt0039177
1746582679	1937	1937	Circus Girl	Circus Girl	Auer, John H.	John H. Auer	tt0028719
1746578568	1961	1961	Back Street	Back Street	Miller, David	David Miller	tt0054653
1746248059	1967	1967	Come Spy with Me	Come Spy with Me	Stone, Marshall	Marshall Stone	tt0061498
1746495861	1920	1920	While New York Sleeps	While New York Sleeps	Brabin, Charles J.	Charles Brabin	tt0011855
1746455000	1997	1997	Good Will Hunting	Good Will Hunting	Van Sant, Gus	Gus Van Sant	tt0119217
1746571939	1932	1932	The Local Bad Man	The Local Bad Man	Brower, Otto	Otto Brower	tt0023144
1746524147	1915	1915	The Absentee	The Absentee	Cabanne, William Christy	Christy Cabanne	tt0004854
1746574786	1936	1936	In His Steps	In His Steps	Brown, Karl	Karl Brown	tt0027792
1746571256	1934	1934	Blue Steel	Blue Steel	Bradbury, Robert	Robert N. Bradbury	tt0024902
1746483876	1919	1919	It's a Bear	It's a Bear	Windom, Lawrence	Lawrence C. Windom	tt0190488
1746520235	1949	1948	One Sunday Afternoon	One Sunday Afternoon	Walsh, Raoul	Raoul Walsh	tt0040668
1746579031	1961	1961	Flight That Disappeared	Flight That Disappeared	Le Borg, Reginald	Reginald Le Borg	tt0054881
1746524021	1910	1910	The Sergeant	The Sergeant	Boggs, Francis	Francis Boggs	tt1380196
1746455859	1996	1996	Mulholland Falls	Mulholland Falls	Tamahori, Lee	Lee Tamahori	tt0117107
1746199528	1969	1969	The Wildest!	The Wildest!	Sully, Jim	Jim Sully	tt0261415
1746455767	1996	1996	A Very Brady Sequel	A Very Brady Sequel	Sanford, Arlene	Arlene Sanford	tt0118073
1746581774	1928	1928	The Patsy	The Patsy	Vidor, King	King Vidor	tt0019258
1746561819	1955	1956	Jungle Hell	Jungle Hell	Cerf, Norman A.	Norman A. Cerf	tt0049389
1746574126	1963	1963	Girl in Trouble	Girl in Trouble	Beale, Lee	Brandon Chase	tt0127575

1746508186	1949	1949	Red, Hot and Blue	Red, Hot and Blue	Farrow, John	John Farrow	tt0041795
1746499938	1948	1948	Adventures of Casanova	Adventures of Casanova	Galvaldón, Roberto	Roberto Gavaldón	tt0040075
1746408851	1988	1988	The In Crowd	The In Crowd	Rosenthal, Mark	Mark Rosenthal	tt0095362
1746475963	1943	1943	Song of Texas	Song of Texas	Kane, Joseph	Joseph Kane	tt0036379
1746487621	1918	1918	Johanna Enlists	Johanna Enlists	Taylor, William D.	William Desmond Taylor	tt0009241
1746575054	1935	1935	Bright Lights	Bright Lights	Berkeley, Busby	Busby Berkeley	tt0026139
1746250196	1979	1979	The Prisoner of Zenda	The Prisoner of Zenda	Quine, Richard	Richard Quine	tt0079753
1746421005	1998	1998	High Art	High Art	Cholodenko, Lisa	Lisa Cholodenko	tt0139362
1746570966	1934	1934	Now and Forever	Now and Forever	Hathaway, Henry	Henry Hathaway	tt0025580
1746564780	1952	1952	The Black Castle	The Black Castle	Juran, Nathan	Nathan Juran	tt0044423
1746583881	1928	1928	Celebrity	Celebrity	Garnett, Tay	Tay Garnett	tt0018753
1746580604	1935	1935	Mary Jane's Pa	Mary Jane's Pa	Keighley, William	William Keighley	tt0026689
1746435888	1998	1998	Lethal Weapon 4	Lethal Weapon 4	Donner, Richard	Richard Donner	tt0122151
1746453552	1941	1941	The Lone Rider in Ghost Town	The Lone Rider in Ghost Town	Newfield, Sam	Sam Newfield	tt0033846
1745340010	2002	2002	Pipe Dream	Pipe Dream	Walsh, John C.	John Walsh	tt0164810
1746516296	1921	1921	The Duke of Chimney Butte	The Duke of Chimney Butte	Borzage, Frank	Frank Borzage	tt0012127
1746582481	1938	1938	Tarzan's Revenge	Tarzan's Revenge	Lederman, D. Ross	D. Ross Lederman	tt0030833
1746516101	1922	1922	East Is West	East Is West	Franklin, Sidney	Sidney Franklin	tt0013093
1746580310	1936	1936	High Tension	High Tension	Dwan, Allan	Allan Dwan	tt0027742
1745395684	2009	2009	17 Again	17 Again	Steers, Burr	Burr Steers	tt0974661
1746508105	1949	1949	Abbott and Costello Meet the Killer: Boris Karlof	Abbott and Costello Meet the Killer, Boris K	Barton, Charles T.	Charles Barton	tt0041085
1746465168	1942	1942	Bombay Clipper	Bombay Clipper	Rawlins, John	John Rawlins	tt0034537
1746465214	1946	1946	Decoy	Decoy	Bernhard, Jack	Jack Bernhard	tt0038462
1746583601	1929	1929	The Cowboy and the Outlaw	The Cowboy and the Outlaw	McGowan, J. P.	J.P. McGowan	tt0019790
1746567940	1933	1933	Tonight Is Ours	Tonight Is Ours	Walker, Stuart	Stuart Walker	tt0024680
1746524256	1927	1927	Bitter Apples	Bitter Apples	Hoyt, Harry O.	Harry O. Hoyt	tt0017676
1746577945	1935	1935	The Code of the Mounted	Code of the Mounted	Newfield, Sam	Sam Newfield	tt0026223
1746197938	1969	1969	The Big Bounce	The Big Bounce	March, Alex	Alex March	tt0064087
1746453782	1942	1942	Man from Cheyenne	Man from Cheyenne	Kane, Joseph	Joseph Kane	tt0035025
1746566063	1955	1955	The Gun That Won the West	The Gun That Won the West	Castle, William	William Castle	tt0048139
1745374401	2002	2002	The Sum of All Fears	The Sum of All Fears	Robinson, Phil Alden	Phil Alden Robinson	tt0164184
1746577786	1937	1937	Rustler's Valley	Rustlers' Valley	Watt, Nate	Nate Watt	tt0029505
1746394003	1986	1986	Crawlspace	Crawlspace	Schmoeller, David	David Schmoeller	tt0090881
1746561615	1965	1965	Black Spurs	Black Spurs	Springsteen, R. G.	R.G. Springsteen	tt0058976
1746474536	1947	1947	Christmas Eve	Christmas Eve	Marin, Edwin L.	Edwin L. Marin	tt0039266
1746561983	1955	1955	Lay That Rifle Down	Lay That Rifle Down	Lamont, Charles	Charles Lamont	tt0048290
1746577744	1937	1937	When You're in Love	When You're in Love	Riskin, Robert; Lachman, Harry	Robert Riskin	tt0029761
1746570085	1933	1933	I'm No Angel	I'm No Angel	Ruggles, Wesley	Wesley Ruggles	tt0024166
1746501198	1915	1915	The Greater Will	The Greater Will	Knoles, Harley	Harley Knoles	tt0004901
1746520572	1925	1925	Red Blood and Blue	Red Blood and Blue	Hutchinson, James C.	James C. Hutchinson	tt0016274
1746454498	1942	1941	Remember the Day	Remember the Day	King, Henry	Henry King	tt0034092
1746574509	1933	1933	What Price Decency?	What Price Decency	Gregor, Arthur	Arthur Gregor	tt0024760
1746583471	1951	1951	I'd Climb the Highest Mountain	I'd Climb the Highest Mountain	King, Henry	Henry King	tt0043667
1746500127	1925	1925	Ridin' Pretty	Ridin' Pretty	Rosson, Arthur	Arthur Rosson	tt0016290
1746512604	1917	1917	Fear Not	Fear Not	Holubar, Allen J.	Allen Holubar	tt0007935
1746566054	1956	1955	Hell on Frisco Bay	Hell on Frisco Bay	Tuttle, Frank	Frank Tuttle	tt0048158
1746581690	1929	1929	Half-way to Heaven	Half Way to Heaven	Abbott, George	George Abbott	tt0019958
1746523548	1927	1926	The Dice Woman	The Dice Woman	Dillon, Edward	Edward Dillon	tt0017811
1746500682	1924	1924	Turned Up	Turned Up	Chapin, James	James Chapin	tt0174290
1746582420	1935	1935	Millions in the Air	Millions in the Air	McCarey, Ray	Ray McCarey	tt0026716
1746497562	1918	1918	A Pair of Sixes	A Pair of Sixes	Windom, Lawrence C.	Lawrence C. Windom	tt0187383
1746438974	1941	1941	Hold That Ghost	Hold That Ghost	Lubin, Arthur	Arthur Lubin	tt0033723
1746583839	1929	1929	Divorce Made Easy	Divorce Made Easy		Neal Burns	tt0019826
1746574288	1961	1961	The Pleasure of His Company	The Pleasure of His Company	Seaton, George	George Seaton	tt0055307
1746394012	1984	1984	Irreconcilable Differences	Irreconcilable Differences	Shyer, Charles	Charles Shyer	tt0087482
1746577392	1961	1960	Wild Youth	Wild Youth	Schreyer, John	John F. Schreyer	tt0053098
1746582997	1938	1938	The Spy Ring	The Spy Ring	Lewis, Joseph H.	Joseph H. Lewis	tt0030784

1746581878	1910	1910	The Golden Supper	The Golden Supper	Griffith, D. W.	D.W. Griffith	tt0001237
1746583227	1929	1929	Evangeline	Evangeline		Edwin Carewe	tt0019856
1746520163	1948	1948	Up in Central Park	Up in Central Park	Seiter, William A.	William A. Seiter	tt0040925
1746514978	1910	1910	Sentimental Sam	Sentimental Sam			tt1804633
1746514975	1917	1917	The Fires of Youth	The Fires of Youth	Chautard, Emile	Emile Chautard	tt0194889
1746514475	1916	1916	A Circus Romance	A Circus Romance	Seay, Charles M.	Charles M. Seay	tt0006393
1746216808	1968	1968	Petulia	Petulia	Lester, Richard	Richard Lester	tt0063426
1746436341	1940	1940	Wagon Train	Wagon Train	Killy, Edward	Edward Killy	tt0033234
1746583756	1930	1930	Cheer Up and Smile	Cheer Up and Smile	Lanfield, Sidney	Sidney Lanfield	tt0020759
1746424178	1943	1943	Cattle Stampede	Cattle Stampede	Newfield, Sam	Sam Newfield	tt0035723
1746525127	1921	1921	The Recoil	The Recoil	Moranti, Milburn	Milburn Morante	tt0012614
1746578462	1962	1962	Money in My Pocket	Money in My Pocket	Jourdan, Erven	Erven Jourdan	tt0312975
1746217988	1966	1966	Not With My Wife, You Don't!	Not with My Wife, You Don't!	Panama, Norman	Norman Panama	tt0060760
1745293806	2006	2006	The Night Listener	The Night Listener	Stettner, Patrick	Patrick Stettner	tt0448075
1746438611	1943	1943	The Mysterious Doctor	The Mysterious Doctor	Stoloff, Ben	Benjamin Stoloff	tt0036187
1746513639	1923	1923	The Brass Bottle	The Brass Bottle		Maurice Tourneur	tt0013887
1746524395	1910	1910	Two Gentlemen of the Road	Two Gentlemen of the Road			tt1799145
1745359811	2003	2003	American Splendor	American Splendor	Pulcini, Robert; Berman, Shari Sprin	Shari Springer Berman	tt0305206
1746566770	1933	1933	Phantom Thunderbolt	Phantom Thunderbolt	James, Alan	Alan James	tt0024444
1746438625	1943	1943	Frontier Fury	Frontier Fury	Berke, William	William Berke	tt0035905
1746500233	1926	1926	Wet Paint	Wet Paint	Rosson, Arthur	Arthur Rosson	tt0017538
1746566367	1955	1955	A Life at Stake	A Life at Stake	Guilfoyle, Paul	Paul Guilfoyle	tt0047178
1746524376	1922	1922	The Galloping Kid	The Galloping Kid	Ross, Nat	Nat Ross	tt0013161
1746532967	1915	1915	When It Strikes Home	When It Strikes Home	Vekroff, Perry N.	Perry N. Vekroff	tt0006270
1746580208	1938	1938	Service De Luxe	Service de Luxe	Lee, Rowland V.	Rowland V. Lee	tt0030732
1746566231	1956	1956	Trapeze	Trapeze	Reed, Carol	Carol Reed	tt0049875
1746439195	1998	1998	The Truman Show	The Truman Show	Weir, Peter	Peter Weir	tt0120382
1746454442	1942	1942	Sunset Serenade	Sunset Serenade	Kane, Joseph	Joseph Kane	tt0035396
1746584273	1939	1939	North of the Yukon	North of the Yukon	Nelson, Sam	Sam Nelson	tt0031733
1746566317	1954	1954	Return to Treasure Island	Return to Treasure Island	Dupont, E. A.	Ewald André Dupont	tt0047406
1746584120	1938	1938	Hard to Get	Hard to Get	Enright, Ray	Ray Enright	tt0030214
1746434251	1995	1995	Fair Game	Fair Game	Sipes, Andrew	Andrew Sipes	tt0113010
1746198513	1967	1967	Luv	Luv	Donner, Clive	Clive Donner	tt0061927
1746503445	1920	1920	The Sea Wolf	The Sea Wolf	Melford, George	George Melford	tt0011672
1746576897	1931	1931	Inspiration	Inspiration	Brown, Clarence	Clarence Brown	tt0022001
1746581179	1910	1910	The Fur Coat	The Fur Coat			tt0449268
1746393708	1982	1981	Enter the Ninja	Enter the Ninja	Golan, Menahem	Menahem Golan	tt0082332
1746464967	1946	1946	Passkey to Danger	Passkey to Danger	Selander, Lesley	Lesley Selander	tt0038828
1745340790	2003	2003	From Justin to Kelly	From Justin to Kelly	Iscove, Robert	Robert Iscove	tt0339034
1746524226	1923	1923	The Rum Runners	The Rum Runners			tt0159710
1746524733	1927	1927	Husband Hunters	Husband Hunters	Adolfi, John G.	John G. Adolfi	tt0018018
1746570830	1934	1934	Flirtation Walk	Flirtation Walk	Borzage, Frank	Frank Borzage	tt0025124
1746584495	1931	1931	Maid to Order	Maid to Order	Clifton, Elmer	Elmer Clifton	tt0022107
1746503304	1919	1919	The Fall of Babylon	The Fall of Babylon	Griffith, D. W.	D.W. Griffith	tt0010108
1746436044	1943	1943	Hit the Ice	Hit the Ice	Lamont, Charles	Charles Lamont	tt0036004
1745341740	2001	2001	Hannibal	Hannibal	Scott, Ridley	Ridley Scott	tt0212985
1746582473	1937	1937	Sh! The Octopus	Sh! The Octopus	McGann, William	William C. McGann	tt0029541
1746542760	1926	1926	My Own Pal	My Own Pal	Blystone, John G.	John G. Blystone	tt0017188
1746569814	1954	1954	Rhapsody	Rhapsody	Vidor, Charles	Charles Vidor	tt0047408
1746567083	1934	1934	She Was a Lady	She Was a Lady	MacFadden, Hamilton	Hamilton MacFadden	tt0025777
1746583631	1928	1928	Drums of Love	Drums of Love	Griffith, D. W.	D.W. Griffith	tt0018854
1746393486	1988	1988	She's Having a Baby	She's Having a Baby	Hughes, John	John Hughes	tt0096094
1746420049	1983	1983	Under Fire	Under Fire	Spottiswoode, Roger	Roger Spottiswoode	tt0086510
1746582196	1931	1931	Partners of the Trail	Partners of the Trail	Fox, Wallace	Wallace Fox	tt0022256
1746584241	1936	1936	The Return of Sophie Lang	The Return of Sophie Lang	Archainbaud, George	George Archainbaud	tt0028172
1745293781	2006	2005	The Notorious Bettie Page	The Notorious Bettie Page	Harron, Mary	Mary Harron	tt0404802
1746419775	1990	1990	Lord of the Flies	Lord of the Flies	Hook, Harry	Harry Hook	tt0100054

1746487156	1918	1918	Cecilia of the Pink Roses	Cecilia of the Pink Roses	Steger, Julius	Julius Steger	tt0008955
1746424445	1942	1942	Thru Different Eyes	Thru Different Eyes	Loring, Thomas Z.	Thomas Z. Loring	tt0035437
1746571118	1935	1935	Riddle Ranch	Riddle Ranch	Hutchison, Charles	Charles Hutchison	tt0137193
1746563773	1952	1952	Eight Iron Men	Eight Iron Men	Dmytryk, Edward	Edward Dmytryk	tt0044579
1769970480	1967	1967	Carmen, Baby	Carmen, Baby	Metzger, Radley	Radley Metzger	tt0061448
1746565993	1953	1953	The 5000 Fingers of Dr. T	The 5,000 Fingers of Dr. T.	Rowland, Roy	Roy Rowland	tt0045464
1746582755	1936	1936	The King Steps Out	The King Steps Out	Sternberg, Josef von	Josef von Sternberg	tt0027847
1746502926	1915	1915	Four Feathers	Four Feathers	Dawley, J. Searle	J. Searle Dawley	tt0005353
1746208796	1968	1968	The Wicked Dreams of Paula Schultz	The Wicked Dreams of Paula Schultz	Marshall, George	George Marshall	tt0063805
1746530770	1950	1950	Big Timber	Big Timber	Yarbrough, Jean	Jean Yarbrough	tt0042251
1746576595	1931	1931	Dude Ranch	Dude Ranch	Tuttle, Frank	Frank Tuttle	tt0021821
1746465404	1942	1942	Scattergood Survives a Murder	Scattergood Survives a Murder	Cabanne, Christy	Christy Cabanne	tt0035293
1746492505	1944	1944	Valley of Vengeance	Valley of Vengeance	Newfield, Sam	Sam Newfield	tt0037425
1746393429	1981	1981	Heartbeeps	Heartbeeps	Arkush, Allan	Allan Arkush	tt0082507
1746583807	1930	1930	Old English	Old English	Green, Alfred E.	Alfred E. Green	tt0021203
1746574326	1961	1961	The Young Doctors	The Young Doctors	Karlson, Phil	Phil Karlson	tt0055632
1746524047	1916	1916	Saving the Family Name	Saving the Family Name	Weber, Lois; Smalley, Phillips	Phillips Smalley	tt0007299
1746585298	1929	1929	The Spirit of Youth	The Spirit of Youth	Lang, Walter	Walter Lang	tt0020441
1746580922	1961	1960	The Private Lives of Adam & Eve	The Private Lives of Adam and Eve	Zugsmith, Albert; Rooney, Mickey	Mickey Rooney	tt0055328
1746500379	1926	1926	Code of the Northwest	Code of the Northwest	Mattison, Frank S.	Frank S. Mattison	tt0016731
1769970755	1915	1915	The Sacred Tiger of Agra	The Sacred Tiger of Agra	Carleton, Lloyd B.	Lloyd B. Carleton	tt0759661
1746574544	1952	1952	You For Me	You for Me	Weis, Don	Don Weis	tt0045345
1746584561	1930	1930	Hold Everything	Hold Everything	Del Ruth, Roy	Roy Del Ruth	tt0020984
1746501600	1915	1915	Fine Feathers	Fine Feathers	Golden, Joseph A.	Joseph A. Golden	tt0005327
1746584224	1938	1938	The Girl Downstairs	The Girl Downstairs	Taurog, Norman	Norman Taurog	tt0030181
1746569634	1955	1955	Land of the Pharaohs	Land of the Pharaohs	Hawks, Howard	Howard Hawks	tt0048283
1746577346	1958	1958	The Saga of Hemp Brown	The Saga of Hemp Brown	Carlson, Richard	Richard Carlson	tt0053236
1745340178	2002	2002	All About the Benjamins	All About the Benjamins	Bray, Kevin	Kevin Bray	tt0278295
1746567226	1934	1934	Wild Cargo	Wild Cargo	Denis, Armand	Armand Denis	tt0025991
1746583181	1939	1939	Career	Career	Jason, Leigh	Leigh Jason	tt0031139
1746507888	1926	1926	The Savage	The Savage	Newmeyer, Fred	Fred C. Newmeyer	tt0017348
1746555831	1957	1957	The Tattered Dress	The Tattered Dress	Arnold, Jack	Jack Arnold	tt0051058
1746508412	1926	1926	The Silent Power	The Silent Power	O'Connor, Frank	Frank O'Connor	tt0017389
1746509199	1927	1927	Life of an Actress	Life of an Actress	Nelson, Jack	Jack Nelson	tt0127653
1746454008	1942	1942	Scorched Earth	The Scorched Earth		Ben Mindenburg	tt0141812
1746570875	1952	1952	Battles of Chief Pontiac	Battles of Chief Pontiac	Feist, Felix	Felix E. Feist	tt0044400
1746567559	1952	1952	Big Jim McLain	Big Jim McLain	Ludwig, Edward	Edward Ludwig	tt0044418
1746576018	1932	1932	The Match King	The Match King	Bretherton, Howard; Keighley, Willi	Howard Bretherton	tt0023198
1746419785	1992	1992	Chaplin	Chaplin	Attenborough, Richard	Richard Attenborough	tt0103939
1746453603	1941	1941	Lady from Louisiana	Lady from Louisiana	Vorhaus, Bernard	Bernard Vorhaus	tt0033808
1746578569	1950	1950	The Vicious Years	The Vicious Years	Florey, Robert	Robert Florey	tt0043105
1746582510	1938	1938	Flirting with Fate	Flirting with Fate	McDonald, Frank	Frank McDonald	tt0030142
1746437564	1997	1997	Leave It to Beaver	Leave It to Beaver	Cadiff, Andy	Andy Cadiff	tt0119509
1746574028	1956	1956	Rock Around the Clock	Rock Around the Clock	Sears, Fred F.	Fred F. Sears	tt0049682
1746439468	1944	1944	Dangerous Passage	Dangerous Passage	Berke, William	William Berke	tt0036741
1746555020	1964	1964	Flipper's New Adventure	Flipper's New Adventure	Benson, Leon	Leon Benson	tt0058105
1769976706	1929	1929	Perfect Day	Perfect Day		James Parrott	tt0020264
1746500494	1925	1925	Keep Smiling	Keep Smiling	Pratt, Gilbert W.; Austin, Albert	Albert Austin	tt0015970
1745340520	2002	2002	Sonny	Sonny	Cage, Nicolas	Nicolas Cage	tt0305973
1746512327	1914	1914	The Man O' Warsman	The Man o' Warsman	Shea, Thomas E.	Thomas E. Shea	tt0004299
1746508069	1925	1925	The Devil's Cargo	The Devil's Cargo	Fleming, Victor	Victor Fleming	tt0015749
1746185853	1969	1969	The Minx	The Minx	Jacobs, Raymond	Raymond Jacobs	tt0064670
1746554863	1956	1956	Huk!	Huk!	Barnwell, John	John Barnwell	tt0049343
1746409690	1987	1987	Innerspace	Innerspace	Dante, Joe	Joe Dante	tt0093260
1746235462	1976	1976	Project: Kill	Project: Kill	Girdler, William	William Girdler	tt0081382
1746516082	1922	1922	The Pride of Palomar	The Pride of Palomar	Borzage, Frank	Frank Borzage	tt0013511
1746563803	1951	1951	Mask of the Dragon	Mask of the Dragon	Newfield, Samuel	Sam Newfield	tt0043791

1746503586	1921	1921	A Divorce of Convenience	A Divorce of Convenience	Ellis, Robert	Robert Ellis	tt0012112
1746570400	1934	1934	Madame Du Barry	Madame Du Barry	Dieterle, William	William Dieterle	tt0025443
1746454371	1942	1942	Little Joe, the Wrangler	Little Joe, the Wrangler	Collins, Lewis D.	Lewis D. Collins	tt0034982
1746571006	1934	1934	I Like It That Way	I Like It That Way	Lachman, Harry	Harry Lachman	tt0025283
1746502384	1916	1916	Tongues of Men	The Tongues of Men	Lloyd, Frank	Frank Lloyd	tt0007467
1746454006	1944	1944	The Yellow Rose of Texas	The Yellow Rose of Texas	Kane, Joseph	Joseph Kane	tt0037472
1745302571	2006	2006	Bobby	Bobby	Estevez, Emilio	Emilio Estevez	tt0308055
1746250943	1978	1978	The Toolbox Murders	The Toolbox Murders	Donnelly, Dennis	Dennis Donnelly	tt0078405
1746501013	1914	1914	The Woman Pays	The Woman Pays		Eugene Moore	tt0437630
1746508095	1925	1925	Fighting the Flames	Fighting the Flames	Eason, Reeves	B. Reeves Eason	tt0015816
1746530771	1948	1948	Boarding House Blues	Boarding House Blues	Binney, Josh	Josh Binney	tt0040176
1746584465	1930	1930	Sweeping Against the Winds	Sweeping Against the Winds		Victor Adamson	tt0167430
1746524445	1916	1916	The People Vs. John Doe	The People vs. John Doe	Weber, Lois	Lois Weber	tt0007170
1746514742	1923	1923	The Seventh Sheriff	The Seventh Sheriff	Hatton, Richard	Dick Hatton	tt0163212
1746524167	1923	1923	The Range Patrol	The Range Patrol	Moody, H. G.	Harry Moody	tt0014396
1746485617	1918	1918	The Curse of Iku	The Curse of Iku	Borzage, Frank	Frank Borzage	tt0008986
1746524033	1916	1915	Vanity	Vanity	O'Brien, John B.	Joseph De Grasse	tt0327222
1746420967	1982	1982	Chan Is Missing	Chan Is Missing	Wang, Wayne	Wayne Wang	tt0083728
1745393828	2009	2009	Old Dogs	Old Dogs	Becker, Walt	Jonathan Fahn	tt1130090
1746577255	1951	1951	The Rage of Burlesque	The Rage of Burlesque	Vox, Albert	Albert Vox	tt7846958
1746500723	1950	1950	The Eagle and the Hawk	The Eagle and the Hawk	Foster, Lewis R.	Lewis R. Foster	tt0042426
1746438326	1944	1944	Raiders of the Border	Raiders of the Border	McCarthy, John P.	John P. McCarthy	tt0037207
1746520409	1926	1926	Pony Express Rider	Pony Express Rider	Horner, Robert J.	Robert J. Horner	tt0313489
1746520394	1925	1925	Marcus Garland	Marcus Garland		Oscar Micheaux	tt0016090
1746577512	1937	1937	Off to the Races	Off to the Races	Strayer, Frank R.	Frank R. Strayer	tt0029332
1746574600	1936	1936	Revolt of the Zombies	Revolt of the Zombies	Halperin, Victor	Victor Halperin	tt0028174
1746580815	1936	1936	The Law Rides	The Law Rides	Bradbury, Robert N.	Robert N. Bradbury	tt0027873
1746574606	1935	1935	The Cyclone Ranger	The Cyclone Ranger	Hill, Bob	Robert F. Hill	tt0026253
1746581112	1962	1962	Girls on the Rocks	Girls on the Rocks	Santos, Anthony	Manuel Conde	tt0124668
1746574352	1959	1959	Five Gates to Hell	Five Gates to Hell	Clavell, James	James Clavell	tt0052808
1746209883	1970	1970	I Walk the Line	I Walk the Line	Frankenheimer, John	John Frankenheimer	tt0065874
1769971167	1969	1969	The Bride and the Beasts	The Bride and the Beasts	Donne, John	Donn Greer	tt0211993
1746208831	1968	1969	A Stranger in Town	A Stranger in Town	Lewis, Vance	Earl J. Miller	tt0126679
1746272154	1968	1968	A Journey to Jerusalem	Journey to Jerusalem	Mindlin, Michael	David Maysles	tt0128286
1769969342	1930	1930	Harmonizing Songs	Harmonizing Songs		Arthur Hurley	tt0122526
1746436245	1995	1995	Out of Sync	Out of Sync	Allen, Debbie	Debbie Allen	tt0114068
1746209509	1965	1965	Situation Hopeless--But Not Serious	Situation Hopeless... But Not Serious	Reinhardt, Gottfried	Gottfried Reinhardt	tt0059724
1746233730	1966	1966	... And Now Miguel	And Now Miguel	Clark, James B.	James B. Clark	tt0060106
1746572598	1931	1931	Waterloo Bridge	Waterloo Bridge	Whale, James	James Whale	tt0022550
1746570743	1934	1934	Rawhide Romance	Rawhide Romance	Dixon, Denver	Victor Adamson	tt0181784
1746503402	1921	1921	A Daughter of the Law	A Daughter of the Law	Conway, Jack	Grace Cunard	tt0310980
1746582026	1910	1910	Hugo, the Hunchback	Hugo the Hunchback		William Nicholas Selig	tt0241544
1746525058	1921	1921	A Sailor-Made Man	A Sailor Made Man	Newmeyer, Fred	Fred C. Newmeyer	tt0012642
1746581092	1961	1961	Gold of the Seven Saints	Gold of the Seven Saints	Douglas, Gordon	Gordon Douglas	tt0054934
1746503302	1922	1922	One Clear Call	One Clear Call	Stahl, John M.	John M. Stahl	tt0013456
1746497882	1922	1922	Superstition	Superstition	Dwan, Allan	Allan Dwan	tt0013649
1746577111	1961	1961	Two Rode Together	Two Rode Together	Ford, John	John Ford	tt0055558
1746436632	1940	1940	Texas Renegades	Texas Renegades	Stewart, Peter	Sam Newfield	tt0033141
1746567789	1933	1933	Rainbow over Broadway	Rainbow Over Broadway	Thorpe, Richard	Richard Thorpe	tt0024486
1746581090	1960	1960	The Secret of the Purple Reef	The Secret of the Purple Reef	Witney, William N.	William Witney	tt0054283
1746578560	1962	1962	War Hunt	War Hunt	Sanders, Denis	Denis Sanders	tt0056675
1746582448	1936	1936	Three Smart Girls	Three Smart Girls	Koster, Henry	Henry Koster	tt0028373
1746583049	1938	1937	Sailing Along	Trailing Along	Hale, Sonnie	Jean Yarbrough	tt1158907
1746581079	1960	1960	Valley of the Redwoods	Valley of the Redwoods	Witney, William N.	William Witney	tt0054435
1746573997	1962	1962	Two Tickets to Paris	Two Tickets to Paris	Garrison, Greg	Greg Garrison	tt0056624
1745342749	2009	2009	I Hope They Serve Beer in Hell	I Hope They Serve Beer in Hell	Gosse, Bob	Bob Gosse	tt1220628
1745309451	2006	2006	Akeelah and the Bee	Akeelah and the Bee	Atchison, Doug	Doug Atchison	tt0437800

**1746393122**	**1986**	**1986**	**Psycho III**	**Psycho III**	**Perkins, Anthony**	**Anthony Perkins**	**tt0091799**	1
1746566225	1953	1953	Tanga Tika	Tanga Tika	Long, Dwight	Dwight Long	tt0131071	
1746423758	1999	1999	House on Haunted Hill	House on Haunted Hill	Malone, William	William Malone	tt0185371	
1746509569	1924	1924	His Own Law	His Own Law			tt0014993	
1746497279	1918	1918	The Red, Red Heart	The Red, Red Heart	Lucas, Wilfred	Wilfred Lucas	tt0009540	
1746585490	1939	1939	Four Wives	Four Wives	Curtiz, Michael	Michael Curtiz	tt0031336	
1745374314	2008	2008	Harold	Harold	Shannon, T. Sean	T. Sean Shannon	tt1041753	
1746523336	1927	1927	The Cruel Truth	The Cruel Truth	Rosen, Phil	Phil Rosen	tt0017783	
1746500036	1924	1924	The Average Woman	The Average Woman	Cabanne, William Christy	Christy Cabanne	tt0014689	
1746514437	1916	1916	The Road to Love	The Road to Love	Sidney, Scott	Scott Sidney	tt0007267	
1746574601	1935	1935	Black Fury	Black Fury	Curtiz, Michael	Michael Curtiz	tt0026121	
1746554310	1964	1964	The Beautiful, the Bloody, and the Bare	The Beautiful, the Bloody, and the Bare	Johnsen, Sande N.	Sande N. Johnsen	tt0057876	
1746577168	1951	1951	Thunder on the Hill	Thunder on the Hill	Sirk, Douglas	Douglas Sirk	tt0044128	
1746260584	1978	1978	Pretty Baby	Pretty Baby	Malle, Louis	Louis Malle	tt0078111	
1746583960	1928	1928	Pass the Gravy	Pass the Gravy	Guiol, Fred L.	Fred Guiol	tt0019253	
1746420962	1994	1994	The Flintstones	The Flintstones	Levant, Brian	Brian Levant	tt0109813	
1746514618	1923	1923	The Temple of Venus	The Temple of Venus	Otto, Henry	Henry Otto	tt0014528	
1746520613	1926	1926	Corporal Kate	Corporal Kate	Sloane, Paul	Paul Sloane	tt0016743	
1746454193	1998	1998	Stepmom	Stepmom	Columbus, Chris	Chris Columbus	tt0120686	
1746475916	1945	1945	Along Came Jones	Along Came Jones	Heisler, Stuart	Stuart Heisler	tt0037508	
1746584413	1939	1939	Indianapolis Speedway	Indianapolis Speedway	Bacon, Lloyd	Lloyd Bacon	tt0031482	
1746473917	1947	1947	The Son of Rusty	The Son of Rusty	Landers, Lew	Lew Landers	tt0039848	
1746233790	1966	1965	John F. Kennedy: Years of Lightning, Day of Drun	John F. Kennedy: Years of Lightning, Day of I	Herschensohn, Bruce	Bruce Herschensohn	tt0060562	
1746200486	1969	1969	Five Bloody Graves	Five Bloody Graves	Adamson, Al	Al Adamson	tt0065723	
1746438331	1942	1942	The Sundown Kid	The Sundown Kid	Clifton, Elmer	Elmer Clifton	tt0035395	
1746573951	1963	1963	The Crawling Hand	The Crawling Hand	Strock, Herbert L.	Herbert L. Strock	tt0056961	
1746554523	1964	1964	The Devil's Bedroom	The Devil's Bedroom	Jones, L. Q.	L.Q. Jones	tt0058009	
1746513792	1923	1923	The Song of Love	The Song of Love	Franklin, Chester; Marion, Frances	Chester M. Franklin	tt0013624	
1746454171	1996	1996	Ed	Teeth	Couturié, Bill	Nicholas Thurkettle	tt0117871	
1746465317	1945	1945	Swing Out Sister	Swing Out, Sister	Lilley, Edward	Edward C. Lilley	tt0038142	
1746422053	1940	1940	He Stayed for Breakfast	He Stayed for Breakfast	Hall, Alexander	Alexander Hall	tt0032576	
1746454890	1996	1996	The Long Kiss Goodnight	The Long Kiss Goodnight	Harlin, Renny	Renny Harlin	tt0116908	
1746530737	1948	1948	The Walls of Jericho	The Walls of Jericho	Stahl, John M.	John M. Stahl	tt0040949	
1746249460	1977	1977	September 30, 1955	September 30, 1955	Bridges, James	James Bridges	tt0078231	
1745293614	2006	2006	The Devil Wears Prada	The Devil Wears Prada	Frankel, David	David Frankel	tt0458352	
1746560774	1932	1932	Tex Takes a Holiday	Tex Takes a Holiday	Neitz, Alvin J.	Alan James	tt0193559	
1746465283	1942	1942	Broadway Big Shot	Broadway Big Shot	Beaudine, William	William Beaudine	tt0034550	
1746503671	1919	1919	God's Outlaw	God's Outlaw	Cabanne, William Christy	Christy Cabanne	tt0010179	
1746582435	1936	1936	Crash Donovan	Crash Donovan	Nigh, William; Laemmle, Edward	William Nigh	tt0027475	
1746497914	1920	1920	Polly of the Storm Country	Polly of the Storm Country	Rosson, Arthur H.	Arthur Rosson	tt0011586	
1746581135	1910	1910	The Hand of Uncle Sam	The Hand of Uncle Sam	Ricketts, Thomas	Tom Ricketts	tt0233843	
1746574895	1933	1933	High Gear	High Gear	Jason, Leigh	Leigh Jason	tt0024119	
1746532445	1913	1913	In the Bishop's Carriage	In the Bishop's Carriage	Porter, Edwin S.; Dawley, J. Searle	J. Searle Dawley	tt0003006	
1746424000	1943	1943	Blazing Frontier	Blazing Frontier	Newfield, Sam	Sam Newfield	tt0035680	
1746454756	1996	1996	The Substitute	The Substitute	Mandel, Robert	Robert Mandel	tt0117774	
1746584422	1937	1937	We Who Are About to Die	We Who Are About to Die	Cabanne, Christy	Christy Cabanne	tt0028484	
1746503876	1919	1919	Stripped for a Million	Stripped for a Million	De La Parelle, L.	M. de la Parelle	tt0192639	
1746572106	1931	1931	Wicked	Wicked	Dwan, Allan	Allan Dwan	tt0022559	
1746424012	1942	1942	Daring Young Man	The Daring Young Man	Strayer, Frank R.	Frank R. Strayer	tt0035781	
1746438446	1943	1943	So This Is Washington	So This Is Washington	McCarey, Raymond	Ray McCarey	tt0036368	
1746199200	1967	1967	Cottonpickin' Chickenpickers	Cottonpickin' Chickenpickers	Jackson, Larry E.	Larry E. Jackson	tt0061522	
1746504412	1921	1921	The Freeze Out	The Freeze Out	Ford, Jack	John Ford	tt0012194	
1745357735	2002	2002	Ted Bundy	Ted Bundy	Bright, Matthew	Matthew Bright	tt0284929	
1746583263	1930	1930	Follow Thru	Follow Thru	Schwab, Laurence; Corrigan, Lloyd	Lloyd Corrigan	tt0020894	
1746408522	1982	1982	Tex	Q	Hunter, Tim	Larry Cohen	tt0084556	
1746491836	1945	1945	Junior Miss	Junior Miss	Seaton, George	George Seaton	tt0037840	
1746507598	1925	1925	The Thoroughbred	The Thoroughbred	Apfel, Oscar	Oscar Apfel	tt0016423	

1746420141	1993	1993	Heart and Souls	Heart and Souls	Underwood, Ron	Ron Underwood	tt0107091
1746199748	1969	1969	Going Down for the 3rd Time	Going Down for the 3rd Time	Goetz, Tommy	Tommy Goetz	tt0219700
1746393268	1984	1984	The Pope of Greenwich Village	The Pope of Greenwich Village	Rosenberg, Stuart	Stuart Rosenberg	tt0087932
1746250953	1977	1977	Demon Seed	Demon Seed	Cammell, Donald	Donald Cammell	tt0075931
1746250153	1979	1978	Dawn of the Dead	Dawn of the Dead	Romero, George A.	George A. Romero	tt0077402
1769970791	1912	1912	The Burglar's Reformation	The Burglar's Reformation	Terwilliger, George	George Terwilliger	tt0358401
1745342473	2002	2002	Halloween Resurrection	Halloween: Resurrection	Rosenthal, Rick	Rick Rosenthal	tt0220506
1746569953	1958	1958	Attack of the 50 Foot Woman	Attack of the 50 Foot Woman	Hertz, Nathan	Nathan Juran	tt0051380
1746574529	1935	1935	Wings in the Dark	Wings in the Dark	Flood, James	James Flood	tt0027221
1746584317	1940	1940	They Knew What They Wanted	They Knew What They Wanted	Kanin, Garson	Garson Kanin	tt0033150
1769969538	1912	1912	The Seal of Time	The Seal of Time	Taylor, Stanner E. V.	Stanner E.V. Taylor	tt0359948
1746513675	1914	1914	The Good-for-Nothing	The Good for Nothing	Anderson, George M.	Gilbert M. 'Broncho Billy' Anderson	tt0004028
1746485940	1919	1919	Lasca	Lasca	Dawn, Norman	Norman Dawn	tt0010341
1746421353	1994	1994	When a Man Loves a Woman	When a Man Loves a Woman	Mandoki, Luis	Luis Mandoki	tt0111693
1746435151	1941	1941	North from the Lone Star	North from the Lone Star	Hillyer, Lambert	Lambert Hillyer	tt0033960
1745302819	2007	2007	Protagonist	Protagonist	Yu, Jessica	Jessica Yu	tt0905361
1746584187	1939	1939	Swanee River	Swanee River	Lanfield, Sidney	Sidney Lanfield	tt0031996
1746420804	1996	1995	Wild Bill: Hollywood Maverick	Wild Bill: Hollywood Maverick	Robinson, Todd	Todd Robinson	tt0114939
1745357601	2008	2008	Disaster Movie	Disaster Movie	Friedberg, Jason; Seltzer, Aaron	Jason Friedberg	tt1213644
1746524174	1910	1910	The Oath and the Man	The Oath and the Man	Griffith, D. W.	D.W. Griffith	tt0001340
1746500204	1924	1924	The Alaskan	The Alaskan	Brenon, Herbert	Herbert Brenon	tt0014655
1746583316	1928	1928	The Candy Kid	The Candy Kid	Kirkland, David	David Kirkland	tt0018745
1746423465	1989	1989	See No Evil, Hear No Evil	See No Evil, Hear No Evil	Hiller, Arthur	Arthur Hiller	tt0098282
1746491817	1948	1948	Fury at Furnace Creek	Fury at Furnace Creek	Humberstone, Bruce	H. Bruce Humberstone	tt0040380
1746435282	1997	1997	Fools Rush In	Fools Rush In	Tennant, Andy	Andy Tennant	tt0119141
1746577709	1935	1935	Rio Rattler	Rio Rattler	Shamray, Franklin	Bernard B. Ray	tt0026935
1746508500	1925	1925	Exchange of Wives	Exchange of Wives	Henley, Hobart	Hobart Henley	tt0015787
1745374408	2009	2009	Jennifer's Body	Jennifer's Body	Kusama, Karyn	Karyn Kusama	tt1131734
1746409794	1988	1988	The Accused	The Accused	Kaplan, Jonathan	Jonathan Kaplan	tt0094608
1746199723	1969	1969	Weekend Lover	Weekend Lovers	Avery, Dwayne	Dwayne Avery	tt0065199
1746555928	1955	1955	The Virgin Queen	The Virgin Queen	Koster, Henry	Henry Koster	tt0048791
1746580870	1937	1937	Trailin' Trouble	Trailin' Trouble	Rosson, Arthur	Arthur Rosson	tt0030889
1746422056	1999	1999	Virus	Virus	Bruno, John	John Bruno	tt0120458
1746559930	1964	1964	A Distant Trumpet	A Distant Trumpet	Walsh, Raoul	Raoul Walsh	tt0058019
1746584550	1930	1930	Covered Wagon Trails	Covered Wagon Trails	McGowan, J. P.	J.P. McGowan	tt0020795
1746453599	1942	1942	Jackass Mail	Jackass Mail	McLeod, Norman Z.	Norman Z. McLeod	tt0034909
1746501593	1916	1916	April	April	MacDonald, Donald	Donald MacDonald	tt0006368
1746573888	1956	1956	The Bottom of the Bottle	The Bottom of the Bottle	Hathaway, Henry	Henry Hathaway	tt0049027
1746516151	1920	1920	An Adventuress	An Adventuress	Balshofer, Fred J.	Fred J. Balshofer	tt0135475
1746562413	1957	1957	Hot Rod Rumble	Hot Rod Rumble	Martinson, Leslie H.	Leslie H. Martinson	tt0050523
1746583524	1930	1930	Sweethearts and Wives	Sweethearts and Wives	Badger, Clarence	Clarence G. Badger	tt0021441
1746564797	1953	1953	Cow Country	Cow Country	Selander, Lesley	Lesley Selander	tt0045653
1746582504	1938	1938	Sky Giant	Sky Giant	Landers, Lew	Lew Landers	tt0030760
1746423040	1993	1993	Hard Target	Hard Target	Woo, John	John Woo	tt0107076
1746235216	1977	1977	The Other Side of Midnight	The Other Side of Midnight	Jarrott, Charles	Charles Jarrott	tt0076507
1746581163	1962	1962	The Weird Ones	The Weird Ones		Pat Boyette	tt0056684
1746578663	1963	1963	Greenwich Village Story	Greenwich Village Story	O'Connell, Jack	Jack O'Connell	tt0217523
1746219032	1972	1972	The Culpepper Cattle Co.	The Culpepper Cattle Co.	Richards, Dick	Dick Richards	tt0068435
1746585453	1940	1940	Boys of the City	Boys of the City	Lewis, Joseph H.	Joseph H. Lewis	tt0032277
1746511776	1916	1916	A Message to Garcia	A Message to Garcia	Ridgely, Richard	Richard Ridgely	tt0163099
1746577101	1961	1961	The Great Impostor	The Great Impostor	Mulligan, Robert	Robert Mulligan	tt0053879
1746574640	1937	1937	Artists and Models	Artists & Models	Walsh, Raoul	Raoul Walsh	tt0028587
1746584428	1939	1939	Two Gun Troubadour	Two Gun Troubador	Johnson, Raymond K.	Bernard B. Ray	tt0032068
1746554714	1964	1964	7 Faces of Dr. Lao	7 Faces of Dr. Lao	Pal, George	George Pal	tt0057812
1746574749	1938	1938	Love Finds Andy Hardy	Love Finds Andy Hardy	Seitz, George B.	George B. Seitz	tt0030386
1746520735	1950	1950	Mr. Music	Mr. Music	Haydn, Richard	Richard Haydn	tt0042753
1746235099	1980	1980	Galaxina	Galaxina	Sachs, William	William Sachs	tt0080771

1746569840	1958	1957	The Saga of the Viking Women and Their Voyage	The Saga of the Viking Women and Their Voy	Corman, Roger	Roger Corman	tt0052156	
1746581607	1930	1930	Swing High	Swing High	Santley, Joseph	Joseph Santley	tt0021445	
1746512754	1927	1927	Cyclone of the Range	Cyclone of the Range	De Lacy, Robert	Robert De Lacey	tt0017788	
1746520557	1925	1925	Folly of Youth	Folly of Youth	Hurst, Paul		tt0132967	
1746434022	1998	1998	I'll Be Home for Christmas	I'll Be Home for Christmas	Sanford, Arlene	Arlene Sanford	tt0155753	
1746198503	1969	1969	Salesman	Salesman	Maysles, David; Maysles, Albert; Zwe	Albert Maysles	tt0064921	
1746423113	1940	1940	The Man from Dakota	The Man from Dakota	Fenton, Leslie	Leslie Fenton	tt0031617	
1745359953	2008	2008	Role Models	Role Models	Wain, David	David Wain	tt0430922	
1746516561	1920	1920	Girl of the Sea	Girl of the Sea	Kelley, J. Winthrop	Winthrop Kelley	tt0195750	
1746584980	1939	1939	Missing Evidence	Missing Evidence	Rosen, Philip	Phil Rosen	tt0031662	
1746464821	1940	1940	Santa Fe Marshal	Santa Fe Marshal	Selander, Lesley	Lesley Selander	tt0033020	
1769970634	1968	1968	Seeds	Seeds	Milligan, Andy	Andy Milligan	tt0235751	
1745340799	2002	2002	Sunshine State	Sunshine State	Sayles, John	John Sayles	tt0286179	
1746495603	1919	1919	The Woman Under Oath	The Woman Under Oath	Stahl, John M.	John M. Stahl	tt0010915	
1746509422	1924	1924	Two Shall Be Born	Two Shall Be Born	Bennett, Whitman	Whitman Bennett	tt0175266	
1746217313	1968	1968	The Sweet Ride	The Sweet Ride	Hart, Harvey	Harvey Hart	tt0063662	
1746408917	1986	1986	Recruits	Recruits	Zielinski, Rafal	Rafal Zielinski	tt0091832	
1746500222	1926	1926	Shipwrecked	Shipwrecked	Henabery, Joseph	Joseph Henabery	tt0017379	
1746474812	1946	1946	Deadline at Dawn	Deadline at Dawn	Clurman, Harold	Harold Clurman	tt0038458	
1746515589	1919	1919	Mrs. Wiggs of the Cabbage Patch	Mrs. Wiggs of the Cabbage Patch	Ford, Hugh	Hugh Ford	tt0010490	
1746577663	1936	1936	Feud of the West	Feud of the West	Fraser, Harry	Harry L. Fraser	tt0027614	
1746507807	1926	1926	A Six Shootin' Romance	A Six Shootin' Romance	Smith, Clifford S.	Alan James	tt0017400	
1746435899	1997	1997	Kicked in the Head	Kicked in the Head	Harrison, Matthew	Matthew Harrison	tt0119457	
1746580403	1935	1935	If You Could Only Cook	If You Could Only Cook	Seiter, William A.	William A. Seiter	tt0026519	
1746571319	1934	1934	Take the Stand	Take the Stand	Rosen, Phil	Phil Rosen	tt0025857	
1746577996	1938	1938	Road Demon	Road Demon	Brower, Otto	Otto Brower	tt0030684	
1745359695	2002	2002	The Singles Ward	The Singles Ward	Hale, Kurt	Kurt Hale	tt0306069	
1746500957	1910	1910	When the World Sleeps	When the World Sleeps		Joseph A. Golden	tt0001455	
1746271943	1968	1968	The Party	The Party	Edwards, Blake	Blake Edwards	tt0063415	
1746560514	1932	1932	Lucky Larrigan	Lucky Larrigan	McCarthy, John P.	John P. McCarthy	tt0023164	
1746185596	1965	1965	Murder in Mississippi	Murder in Mississippi	Mawra, J. P.	Joseph P. Mawra	tt0059485	
1746496131	1919	1919	Sahara	Sahara	Rosson, Arthur	Arthur Rosson	tt0010654	
1746486455	1920	1920	Rogues and Romance	Rogues and Romance	Seitz, George B.	George B. Seitz	tt0011637	
1746219544	1977	1977	Day of the Animals	Day of the Animals	Girdler, William	William Girdler	tt0075913	
1746584212	1938	1938	Wide Open Faces	Wide Open Faces	Neumann, Kurt	Kurt Neumann	tt0030977	
1746583446	1929	1929	Silks and Saddles	Silks and Saddles	Hill, Robert F.	Robert F. Hill	tt0020406	
1746566149	1957	1957	Legend of the Lost	Legend of the Lost	Hathaway, Henry	Henry Hathaway	tt0050629	
1746473885	1945	1945	Docks of New York	Docks of New York	Fox, Wallace	Wallace Fox	tt0037648	
1746530729	1948	1948	You Were Meant for Me	You Were Meant for Me	Bacon, Lloyd	Lloyd Bacon	tt0040983	
1746580871	1938	1938	Tip-Off Girls	Tip Off Girls	King, Louis	Louis King	tt0030875	
1746208160	1968	1968	Project X	Project X		William Castle	tt0063465	
1746491723	1947	1947	Easy Come, Easy Go	Easy Come, Easy Go	Farrow, John	John Farrow	tt0039346	
1746514052	1922	1922	Free Air	Free Air	Griffith, E. H.	Edward H. Griffith	tt0013153	
1746524274	1916	1916	The Dawn of Freedom	The Dawn of Freedom	Scardon, Paul; Marston, Theodore	Theodore Marston	tt0157512	
1746502071	1915	1915	Old Dutch	Old Dutch	Crane, Frank	Frank Hall Crane	tt0005821	
1746500326	1926	1926	West of Broadway	West of Broadway	Thornby, Robert	Robert Thornby	tt0177372	
1746574792	1935	1935	Pursuit	Pursuit	Marin, Edwin L.	Edwin L. Marin	tt0026901	
1746574793	1937	1937	The Footloose Heiress	The Footloose Heiress	Clemens, William	William Clemens	tt0028887	
1746422104	1994	1994	Speed	Speed	de Bont, Jan	Jan de Bont	tt0111257	
1746500263	1949	1949	Port of New York	Port of New York	Benedek, Laslo	Laslo Benedek	tt0041761	
1746575805	1931	1931	The Big Shot	The Big Shot	Murphy, Ralph F.	Ralph Murphy	tt0021661	
1745375161	2009	2009	A Single Man	A Single Man	Ford, Tom	Tom Ford	tt1315981	
**1746465003**	**1942**	**1942**	**My Gal Sal**	**My Gal Sal**	**Cummings, Irving**	**Irving Cummings**	**tt0035103**	1
1746582813	1938	1938	Juvenile Court	Juvenile Court	Lederman, D. Ross	D. Ross Lederman	tt0030303	
1746574579	1935	1935	The Desert Trail	The Desert Trail	Lewis, Cullin	Lewis D. Collins	tt0026273	
1746570003	1957	1957	House of Numbers	House of Numbers	Rouse, Russell	Russell Rouse	tt0050526	
1746474819	1947	1947	Desperate	Desperate	Mann, Anthony	Anthony Mann	tt0039313	

1746562454	1957	1957	Pickup Alley	Pickup Alley	Gilling, John	John Gilling	tt0050841
1746496304	1917	1917	The Inner Shrine	The Inner Shrine	Reicher, Frank	Frank Reicher	tt0008137
1746198072	1968	1968	Daring Game	Daring Game	Benedek, Laslo	Laslo Benedek	tt0062862
1746504622	1921	1921	Such a Little Queen	Such a Little Queen	Fawcett, George	George Fawcett	tt0012733
1746502561	1916	1916	The Hand of Peril	The Hand of Peril	Tourneur, Maurice	Maurice Tourneur	tt0006755
1746219182	1978	1978	Martin	Martin	Romero, George A.	George A. Romero	tt0077914
1746256810	1975	1975	The Devil's Rain	The Devil's Rain	Fuest, Robert	Robert Fuest	tt0072869
1746583137	1939	1939	The Honeymoon's Over	The Honeymoon's Over	Forde, Eugene; Beaudine, William	Eugene Forde	tt0031441
1746555078	1957	1957	The Quiet Gun	The Quiet Gun	Claxton, William F.	William F. Claxton	tt0050874
1746570057	1934	1935	One in a Million	One in a Million	Strayer, Frank R.	Frank R. Strayer	tt0025594
1745293630	2007	2007	Breach	Breach	Ray, Billy	Billy Ray	tt0401997
1746516039	1921	1921	Is Life Worth Living?	Is Life Worth Living?	Crosland, Alan	Alan Crosland	tt0012324
1746584564	1931	1931	Devotion	Devotion	Milton, Robert	Robert Milton	tt0021798
1746421355	1994	1994	I'll Do Anything	I'll Do Anything	Brooks, James L.	James L. Brooks	tt0110097
1746493776	1914	1914	The Next in Command	The Next in Command	Dawley, J. Searle	J. Searle Dawley	tt0004398
1746409520	1984	1984	Alley Cat	Alley Cat	Victor, Edward; Palmos, Eduardo; Or	Victor M. Ordonez	tt0083541
1769970832	1915	1915	Blade O' Grass	Blade o' Grass	George, Burton	Burton George	tt0317168
1746465374	1947	1947	Saddle Pals	Saddle Pals	Selander, Lesley	Lesley Selander	tt0039795
1746564509	1952	1952	Desert Passage	Desert Passage	Selander, Lesley	Lesley Selander	tt0044540
1746570297	1934	1934	Happy Landing	Happy Landing	Bradbury, R. N.	Robert N. Bradbury	tt0025220
1745359155	2007	2007	3:10 to Yuma	3:10 to Yuma	Mangold, James	James Mangold	tt0381849
1746553962	1964	1964	Shell Shock	Shell Shock	Hayes, John	John Hayes	tt0155166
1746504134	1922	1922	The Primitive Lover	The Primitive Lover	Franklin, Sidney	Sidney Franklin	tt0013512
1746217417	1966	1966	Kid Rodelo	Kid Rodelo	Carlson, Richard	Richard Carlson	tt0059356
1746583611	1930	1930	A Royal Romance	A Royal Romance	Kenton, Erle C.	Erle C. Kenton	tt0021323
1746454012	1940	1940	Ragtime Cowboy Joe	Ragtime Cowboy Joe	Taylor, Ray	Ray Taylor	tt0032965
1746580910	1937	1937	The Outer Gate	The Outer Gate	Cannon, Ray	Raymond Cannon	tt0029362
1746578000	1937	1937	She Married an Artist	She Married an Artist	Gering, Marion	Marion Gering	tt0030740
1746438474	1941	1941	Six Lessons from Madame La Zonga	Six Lessons from Madame La Zonga	Rawlins, John	John Rawlins	tt0034196
1746393703	1985	1985	Fandango	Fandango	Reynolds, Kevin	Kevin Reynolds	tt0089126
1746503246	1917	1917	The Alien Blood	The Alien Blood	George, Burton	Burton George	tt0007622
1746486829	1917	1917	The Eternal Sin	The Eternal Sin	Brenon, Herbert	Herbert Brenon	tt0007900
1746496575	1920	1920	A Woman's Business	A Woman's Business	Rolfe, B. A.	B.A. Rolfe	tt0011878
1746569667	1956	1956	Inside Detroit	Inside Detroit	Sears, Fred F.	Fred F. Sears	tt0048209
1746453657	1998	1998	The Avengers	The Avengers	Chechik, Jeremiah	Jeremiah S. Chechik	tt0118661
1746420991	1992	1991	The Runestone	The Runestone	Carroll, Willard	Willard Carroll	tt0100528
1746234774	1968	1967	Funnyman	Funnyman	Korty, John	John Korty	tt0179189
1745373973	2008	2008	The Longshots	The Longshots	Durst, Fred	Fred Durst	tt1091751
1746492070	1948	1948	Green Grass of Wyoming	Green Grass of Wyoming	King, Louis	Louis King	tt0040402
1746565942	1953	1953	Let's Do It Again	Let's Do It Again	Hall, Alexander	Alexander Hall	tt0045995
1746583671	1929	1929	The Valiant	The Valiant	Howard, William K.	William K. Howard	tt0020543
1746492241	1944	1944	The Adventures of Mark Twain	The Adventures of Mark Twain	Rapper, Irving	Irving Rapper	tt0036582
1745342750	2010	2010	Love & Other Drugs	Love & Other Drugs	Zwick, Edward	Edward Zwick	tt0758752
1746581665	1929	1929	Broadway Fever	Broadway Fever	Cline, Edward	Edward F. Cline	tt0019727
1746542750	1926	1926	The Danger Girl	The Danger Girl	Dillon, Edward	Edward Dillon	tt0016763
1746583917	1930	1930	In Gay Madrid	In Gay Madrid	Leonard, Robert Z.	Robert Z. Leonard	tt0020997
1746520604	1925	1925	Tumbleweeds	Tumbleweeds	Baggot, King	King Baggot	tt0016461
1746501455	1915	1915	The Danger Signal	The Danger Signal	Edwin, Walter	Walter Edwin	tt0005171
1746485330	1917	1917	The Girl by the Roadside	The Girl by the Roadside	Marston, Theodore	Theodore Marston	tt0008008
1746515046	1916	1916	A Son of Erin	A Son of Erin	Ivers, Julia Crawford	Julia Crawford Ivers	tt0158973
1746392949	1982	1982	Trail of the Pink Panther	Trail of the Pink Panther	Edwards, Blake	Blake Edwards	tt0084814
1746409519	1985	1985	National Lampoon's European Vacation	National Lampoon's European Vacation	Heckerling, Amy	Amy Heckerling	tt0089670
1746578564	1951	1951	Fort Worth	Fort Worth	Marin, Edwin L.; McGann, William	Edwin L. Marin	tt0043557
1746422304	1993	1993	Jason Goes to Hell: The Final Friday	Jason Goes to Hell: The Final Friday	Marcus, Adam	Adam Marcus	tt0107254
1746475933	1946	1946	The Wife of Monte Cristo	The Wife of Monte Cristo	Ulmer, Edgar G.	Edgar G. Ulmer	tt0039106
1745272214	2001	2001	Bubble Boy	Bubble Boy	Hayes, Blair	Blair Hayes	tt0258470
1746577221	1950	1950	Silver Raiders	Silver Raiders	Fox, Wallace W.	Wallace Fox	tt0042967

1746584441	1931	1931	Alexander Hamilton	Alexander Hamilton	Adolfi, John G.	John G. Adolfi	tt0021595	
1746424408	1941	1941	The Maltese Falcon	The Maltese Falcon	Huston, John	John Huston	tt0033870	
1746516142	1919	1919	Fighting for Gold	Fighting for Gold	LeSaint, Edward J.	Edward LeSaint	tt0010123	
1746570175	1933	1933	The White Sister	The White Sister	Fleming, Victor	Victor Fleming	tt0024770	
1746394166	1981	1981	Lunch Wagon	Lunch Wagon	Pintoff, Ernest	Ernest Pintoff	tt0081088	
1746515799	1922	1922	The Ruling Passion	The Ruling Passion	Weight, Harmon	F. Harmon Weight	tt0013567	
1746570042	1934	1934	Beast of Borneo	The Beast of Borneo	Gordon, Harry	Harry Garson	tt0025075	
1746501765	1915	1915	The Morals of Marcus	The Morals of Marcus	Porter, Edwin S.; Ford, Hugh	Hugh Ford	tt0005770	
1745342693	2001	2001	The Glass House	The Glass House	Sackheim, Daniel	Daniel Sackheim	tt0221218	
1746584211	1936	1936	Killer at Large	Killer at Large	Selman, David	David Selman	tt0027843	
1746578824	1961	1961	Dark Odyssey	Dark Odyssey	Kyriakys, William; Metzger, Radley	William Kyriakis	tt0054787	
1746577668	1933	1933	Jennie Gerhardt	Jennie Gerhardt	Gering, Marion	Marion Gering	tt0024193	
1746492225	1944	1944	Gildersleeve's Ghost	Gildersleeve's Ghost	Douglas, Gordon	Gordon Douglas	tt0036864	
1746585412	1940	1940	The Invisible Man Returns	The Invisible Man Returns	May, Joe	Joe May	tt0032635	
1746567449	1932	1932	Heritage of the Desert	Heritage of the Desert	Hathaway, Henry	Henry Hathaway	tt0022999	
1746500168	1925	1925	Seven Chances	Seven Chances	Keaton, Buster	Buster Keaton	tt0016332	
1746436822	1991	1991	My Girl	My Girl	Zieff, Howard	Howard Zieff	tt0102492	
1746437151	2000	1999	Just Looking	Just Looking	Alexander, Jason	Jason Alexander	tt0162236	
1746235802	1974	1974	The Black Godfather	The Black Godfather	Evans, John	John Evans	tt0071225	
1746577821	1939	1939	The Thundering West	The Thundering West	Nelson, Sam	Sam Nelson	tt0032035	
1746438587	2000	2000	Best in Show	Best in Show	Guest, Christopher	Christopher Guest	tt0218839	
1746584409	1939	1939	Outside These Walls	Outside These Walls	McCarey, Raymond B.	Ray McCarey	tt0031771	
1746439480	1940	1940	Law and Order	Law and Order	Taylor, Ray	Ray Taylor	tt0032694	
1746542667	1924	1924	Bringin' Home the Bacon	Bringin' Home the Bacon	Thorpe, Richard	Richard Thorpe	tt0014742	
1746582459	1939	1939	Saga of Death Valley	Saga of Death Valley	Kane, Joseph	Joseph Kane	tt0031889	
1746567346	1953	1953	The Redhead from Wyoming	The Redhead from Wyoming	Sholem, Lee	Lee Sholem	tt0045077	
1746583497	1930	1930	The Lonesome Trail	The Lonesome Trail	Mitchell, Bruce	Bruce Mitchell	tt0021086	
1745341624	2003	2003	Freddy vs. Jason	Freddy vs. Jason	Yu, Ronny	Ronny Yu	tt0329101	
**1746422299**	**1995**	**1995**	**Kicking and Screaming**	**Kicking and Screaming**	**Baumbach, Noah**	**Noah Baumbach**	**tt0113537**	1
1746218909	1978	1978	Damien--Omen II	Damien: Omen II	Taylor, Don	Don Taylor	tt0077394	
1746435316	1998	1998	American History X	American History X	Kaye, Tony	Tony Kaye	tt0120586	
1746235481	1975	1975	Hustle	Hustle	Aldrich, Robert	Robert Aldrich	tt0073133	
1746516467	1921	1921	One Wild Week	One Wild Week	Campbell, Maurice	Maurice Campbell	tt0012528	
1746465058	1942	1942	Remember Pearl Harbor	Remember Pearl Harbor	Santley, Joseph	Joseph Santley	tt0035249	
1746208945	1966	1966	To the Shores of Hell	To the Shores of Hell	Zens, Will	Will Zens	tt0061099	
1746571918	1931	1931	Meet the Wife	Meet the Wife	Pearce, A. Leslie	Leslie Pearce	tt0022130	
1745342246	2004	2004	Raising Helen	Raising Helen	Marshall, Garry	Garry Marshall	tt0350028	
1746453587	1940	1939	Death Rides the Range	Death Rides the Range	Newfield, Sam	Sam Newfield	tt0032387	
1746582287	1930	1930	On the Border	On the Border	McGann, William	William C. McGann	tt0021208	
1746465142	1945	1945	Sensation Hunters	Sensation Hunters	Cabanne, Christy	Christy Cabanne	tt0038071	
1746433750	2000	2000	Book of Shadows: Blair Witch 2	Book of Shadows: Blair Witch 2	Berlinger, Joe	Joe Berlinger	tt0229260	
1746515606	1917	1917	The Skylight Room	The Skylight Room	Justice, Martin	Martin Justice	tt0182426	
1746410746	1988	1988	Rocket Gibraltar	Rocket Gibraltar	Petrie, Daniel	Daniel Petrie	tt0096003	
1746575389	1931	1931	The Girl Habit	The Girl Habit	Cline, Edward	Edward F. Cline	tt0021908	
1746564750	1952	1952	The Treasure of Lost Canyon	The Treasure of Lost Canyon	Tetzlaff, Ted	Ted Tetzlaff	tt0044144	
1746574892	1935	1935	Rustler's Paradise	Rustler's Paradise	Fraser, Harry	Harry L. Fraser	tt0026959	
1746580876	1936	1936	Racing Blood	Racing Blood	Hale, Rex	Victor Halperin	tt0030648	
1745341451	2002	2002	The Bourne Identity	The Bourne Identity	Liman, Doug	Doug Liman	tt0258463	
1745341565	2002	2002	Antwone Fisher	Antwone Fisher	Washington, Denzel	Denzel Washington	tt0168786	
1746582779	1939	1939	Pacific Liner	Pacific Liner	Landers, Lew	Lew Landers	tt0031774	
1746507605	1926	1925	Riding Romance	Riding Romance	McGowan, J. P.	J.P. McGowan	tt0017325	
**1746555503**	**1955**	**1955**	**The Night Holds Terror**	**The Night Holds Terror**	**Stone, Andrew**	**Andrew L. Stone**	**tt0048423**	1
1746532458	1928	1928	The Little Wildcat	The Little Wildcat	Enright, Ray	Ray Enright	tt0020102	
1746564327	1953	1953	China Venture	China Venture	Siegel, Don	Don Siegel	tt0045624	
1746569616	1955	1955	Abbott and Costello Meet the Keystone Kops	Abbott and Costello Meet the Keystone Kop	Lamont, Charles	Charles Lamont	tt0047794	
1746533208	1923	1923	The Untameable	The Untameable	Blaché, Herbert	Herbert Blaché	tt0014570	
1746564752	1953	1953	The Caddy	The Caddy	Taurog, Norman	Norman Taurog	tt0045589	

1746509174	1924	1924	The Dawn of a Tomorrow	The Dawn of a Tomorrow	Melford, George	George Melford	tt0014836
1746562382	1956	1956	Accused of Murder	Accused of Murder	Kane, Joe	Joseph Kane	tt0048924
1746198089	1968	1967	The Money Jungle	The Money Jungle	Lyon, Francis D.	Francis D. Lyon	tt0063322
1746421666	1994	1994	Federal Hill	Federal Hill	Corrente, Michael	Michael Corrente	tt0109785
1746491742	1946	1946	The Spiral Staircase	The Spiral Staircase	Siodmak, Robert	Robert Siodmak	tt0038975
1746524822	1916	1916	The Flirt	The Flirt	Weber, Lois; Smalley, Phillips	Phillips Smalley	tt0006687
1769970434	1967	1967	Fanny Hill Meets Lady Chatterly	Fanny Hill Meets Lady Chatterly	Mahon, Barry; Matsui, George	Barry Mahon	tt0218203
1746235398	1975	1975	The Great Waldo Pepper	The Great Waldo Pepper	Hill, George Roy	George Roy Hill	tt0073075
1746584439	1931	1931	Pleasure	Pleasure	Brower, Otto	Otto Brower	tt0023343
1746581270	1959	1959	The Best of Everything	The Best of Everything	Negulesco, Jean	Jean Negulesco	tt0052619
1746410721	1993	1992	Guncrazy	Guncrazy	Davis, Tamra	Tamra Davis	tt0104377
1746465015	1942	1942	Devil's Harvest	Devil's Harvest	Test, Ray	Ray Test	tt0157533
1746503369	1918	1918	Convict 993	Convict 993	Parke, William	William Parke	tt0184356
1746508952	1926	1925	Fighting Luck	Fighting Luck	McGowan, J. P.	J.P. McGowan	tt0016863
1745359738	2002	2002	Hollywood Ending	Hollywood Ending	Allen, Woody	Woody Allen	tt0278823
1746565979	1957	1957	Street of Sinners	Street of Sinners	Berke, William	William Berke	tt0051022
1746184193	1910	1910	The Blazed Trail	The Blazed Trail			tt0359014
1746571489	1931	1931	Neck and Neck	Neck and Neck	Thorpe, Richard	Richard Thorpe	tt0022193
1745359347	2001	2001	Ghost World	Ghost World	Zwigoff, Terry	Terry Zwigoff	tt0162346
1746582526	1938	1938	Mr. Wong, Detective	Mr. Wong, Detective	Nigh, William	William Nigh	tt0030473
1746542672	1926	1926	Stop, Look, and Listen	Stop, Look and Listen	Semon, Larry	Larry Semon	tt0017433
1746524072	1915	1915	Gretna Green	Gretna Green	Heffron, Thomas	Thomas N. Heffron	tt0005427
1746542716	1924	1924	The Man From Wyoming	The Man from Wyoming	Bradbury, Robert North	Robert N. Bradbury	tt0015109
1746574416	1961	1961	The 7th Commandment	The 7th Commandment	Berwick, Irvin	Irvin Berwick	tt0054297
1746559831	1965	1965	Beauty #2	Beauty #2	Warhol, Andy	Andy Warhol	tt0208725
1746584728	1929	1929	Tanned Legs	Tanned Legs	Neilan, Marshall	Marshall Neilan	tt0020480
1746573948	1957	1957	Gunsight Ridge	Gunsight Ridge	Lyon, Francis D.	Francis D. Lyon	tt0050471
1746198066	1969	1969	True Grit	True Grit	Hathaway, Henry	Henry Hathaway	tt0065126
1746523956	1911	1911	As a Boy Dreams	As a Boy Dreams		Thomas H. Ince	tt1174943
1746574300	1961	1961	Fear No More	Fear No More	Wiesen, Bernard	Bernard Wiesen	tt0150524
1746500916	1916	1916	The Power of Evil	The Power of Evil	Horkheimer, H. M.; Horkheimer, E. D	E.D. Horkheimer	tt0007206
1746421586	1995	1995	Two Bits	Two Bits	Foley, James	James Foley	tt0114753
1746465044	1942	1942	Tramp, Tramp, Tramp	Tramp, Tramp, Tramp!	Barton, Charles	Charles Barton	tt0035467
1746500309	1926	1926	Ranson's Folly	Ranson's Folly	Olcott, Sidney	Sidney Olcott	tt0017309
1745293328	2005	2006	Who Gets to Call It Art?	Who Gets to Call It Art?	Rosen, Peter	Peter Rosen	tt0472210
1746436091	1943	1943	Margin for Error	Margin for Error	Preminger, Otto	Otto Preminger	tt0036142
1746566128	1954	1954	The Black Knight	The Black Knight	Garnett, Tay	Tay Garnett	tt0046786
1746424554	1943	1943	You Can't Beat the Law	You Can't Beat the Law	Rosen, Phil	Phil Rosen	tt0036550
1746508766	1925	1925	Webs of Steel	Webs of Steel	McGowan, J. P.	J.P. McGowan	tt0016508
1746202400	1970	1970	Chisum	Chisum	McLaglen, Andrew V.	Andrew V. McLaglen	tt0065547
1746500488	1926	1926	Dangerous Traffic	Dangerous Traffic	Cohn, Bennett	Bennett Cohen	tt0016768
1746502460	1914	1914	In the Name of the Prince of Peace	In the Name of the Prince of Peace	Dawley, J. Searle	J. Searle Dawley	tt0004152
1746465367	1945	1945	Guest Wife	Guest Wife	Wood, Sam	Sam Wood	tt0037756
1746584580	1928	1928	Smith's Holiday	Smith's Holiday	Goulding, Alf	Alfred J. Goulding	tt0783827
1745340248	2003	2003	Cradle 2 the Grave	Cradle 2 the Grave	Bartkowiak, Andrzej	Andrzej Bartkowiak	tt0306685
1746219540	1976	1976	A Star Is Born	A Star Is Born	Pierson, Frank	Frank Pierson	tt0075265
1746509324	1925	1925	Lover's Island	Lover's Island	Diamant-Berger, Henri	Henri Diamant	tt0016047
1746584213	1936	1936	Palm Springs	Palm Springs	Scotto, Aubrey	Aubrey Scotto	tt0028079
1746524935	1914	1914	A Celebrated Case	A Celebrated Case		George Melford	tt0003761
1746208777	1966	1966	Judith	Judith	Mann, Daniel	Daniel Mann	tt0060568
1746567762	1933	1933	The Little Giant	The Little Giant	Del Ruth, Roy	Roy Del Ruth	tt0024262
1746491831	1948	1948	The Counterfeiters	The Counterfeiters	Stewart, Peter	Sam Newfield	tt0040250
1746454483	1940	1940	The House Across the Bay	The House Across the Bay	Mayo, Archie	Archie Mayo	tt0032609
1746585148	1929	1929	Glorifying the American Girl	Glorifying the American Girl	Webb, Millard	Millard Webb	tt0019933
1746500207	1950	1950	Bunco Squad	Bunco Squad	Leeds, Herbert I.	Herbert I. Leeds	tt0042289
1746567963	1952	1952	Night Without Sleep	Night Without Sleep	Baker, Roy	Roy Ward Baker	tt0044967
1746585461	1939	1939	Maisie	Maisie	Marin, Edwin L.	Edwin L. Marin	tt0031608

1746248506	1968	1968	Unstrap Me	Unstrap Me	Kuchar, George	George Kuchar	tt0207179
1746532833	1915	1915	The House of the Lost Court	The House of the Lost Court	Brabin, Charles J.	Charles Brabin	tt0005519
1746436348	1941	1941	Ridin' on a Rainbow	Ridin' on a Rainbow	Landers, Lew	Lew Landers	tt0034106
1745293919	2005	2005	Herbie: Fully Loaded	Herbie Fully Loaded	Robinson, Angela	Angela Robinson	tt0400497
1746454019	1941	1941	Mr. Celebrity	Mr. Celebrity	Beaudine, William	William Beaudine	tt0035089
1746503653	1921	1921	The Foolish Age	The Foolish Age	Seiter, William A.	William A. Seiter	tt0012182
1746573777	1961	1961	The Last Sunset	The Last Sunset	Aldrich, Robert	Robert Aldrich	tt0055073
1746475511	1945	1945	It's a Pleasure!	It's a Pleasure	Seiter, William A.	William A. Seiter	tt0037822
1746497425	1917	1917	Polly Redhead	Polly Redhead	Conway, Jack	Jack Conway	tt0008441
1746500031	1924	1924	The Masked Dancer	The Masked Dancer	King, Burton	Burton L. King	tt0015124
1745357635	2004	2004	Shall We Dance	Shall We Dance	Chelsom, Peter	Peter Chelsom	tt0358135
1746454269	1942	1942	They All Kissed the Bride	They All Kissed the Bride	Hall, Alexander	Alexander Hall	tt0035428
1746572844	1910	1910	The Dead Letter	The Dead Letter			tt1080590
1746474829	1945	1945	Riders of the Dawn	Riders of the Dawn	Drake, Oliver	Oliver Drake	tt0163182
1746475939	1945	1945	Renegades of the Rio Grande	Renegades of the Rio Grande	Bretherton, Howard	Howard Bretherton	tt0038021
1746501076	1915	1915	The Whirl of Life	The Whirl of Life	Bailey, Oliver D.	Oliver D. Bailey	tt0006276
1746530753	1948	1948	Hidden Danger	Hidden Danger	Taylor, Ray	Ray Taylor	tt0040436
1746553577	1963	1963	The Yellow Canary	The Yellow Canary	Kulik, Buzz	Buzz Kulik	tt0057700
1746496416	1917	1917	The Spreading Dawn	The Spreading Dawn	Trimble, Lawrence	Laurence Trimble	tt0008622
1746516310	1922	1922	The Stranger's Banquet	The Strangers' Banquet	Neilan, Marshall	Marshall Neilan	tt0013640
1746561432	1932	1932	Girl Crazy	Girl Crazy	Seiter, William A.	William A. Seiter	tt0022938
1746410820	1990	1990	Men at Work	Men at Work	Estevez, Emilio	Emilio Estevez	tt0100135
1746532736	1915	1915	Judge Not; or, the Woman of Mona Diggings	Judge Not; or The Woman of Mona Diggings	Leonard, Robert	Robert Z. Leonard	tt0005575
1746583298	1930	1930	Inside the Lines	Inside the Lines	Pomeroy, Roy J.	Roy Pomeroy	tt0021001
1746409958	1983	1983	Space Raiders	Space Raiders	Cohen, Howard R.	Howard R. Cohen	tt0086345
1746564287	1952	1951	It's a Big Country: An American Anthology	It's a Big Country: An American Anthology		Clarence Brown	tt0043680
1746562370	1957	1957	Teen Age Thunder	Teenage Thunder	Helmick, Paul	Paul Helmick	tt0048902
1746532978	1923	1922	Robin Hood	Robin Hood	Dwan, Allan	Allan Dwan	tt0013556
1746435582	1943	1943	Redhead from Manhattan	Redhead from Manhattan	Landers, Lew	Lew Landers	tt0036297
1746569669	1958	1958	No Place to Land	No Place to Land	Gannaway, Albert C.	Albert C. Gannaway	tt0052003
1746566232	1955	1955	What Price Freedom	What Price Freedom		Paul F. Heard	tt7255510
1745359512	2001	2000	Million Dollar Hotel	The Million Dollar Hotel	Wenders, Wim	Wim Wenders	tt0120753
1746566022	1954	1953	Forever Female	Forever Female	Rapper, Irving	Irving Rapper	tt0045782
1746565036	1953	1953	The Blue Gardenia	The Blue Gardenia	Lang, Fritz	Fritz Lang	tt0045564
1746554843	1964	1964	Marnie	Marnie	Hitchcock, Alfred	Alfred Hitchcock	tt0058329
1746570448	1934	1934	Of Human Bondage	Of Human Bondage	Cromwell, John	John Cromwell	tt0025586
1746577272	1958	1958	The Vikings	The Vikings	Fleischer, Richard	Richard Fleischer	tt0052365
1745340475	2001	2001	Double Take	Double Take	Gallo, George	George Gallo	tt0238948
1746574827	1933	1933	Destination Unknown	Destination Unknown	Garnett, Tay	Tay Garnett	tt0023941
1746583547	1951	1951	Fort Savage Raiders	Fort Savage Raiders	Nazarro, Ray	Ray Nazarro	tt0043556
1746583143	1936	1936	His Brother's Wife	His Brother's Wife	Van Dyke, W. S.	W.S. Van Dyke	tt0027746
1746464845	1941	1941	A Man Betrayed	A Man Betrayed	Auer, John H.	John H. Auer	tt0033871
1745372428	2001	2001	See Spot Run	See Spot Run	Whitesell, John	John Whitesell	tt0250720
1746464857	1942	1942	Arizona Terrors	Arizona Terrors	Sherman, George	George Sherman	tt0034470
1745359309	2002	2002	Orange County	Orange County	Kasdan, Jake	Jake Kasdan	tt0273923
1746570363	1934	1934	The Westerner	The Westerner	Selman, David	David Selman	tt0128838
1746574316	1959	1959	The Scavengers	The Scavengers	Cromwell, John	John Cromwell	tt0057474
1746584533	1931	1931	Dirigible	Dirigible	Capra, Frank R.	Frank Capra	tt0021799
1746464943	1947	1947	Blondie in the Dough	Blondie in the Dough	Berlin, Abby	Abby Berlin	tt0039200
1746210585	1973	1973	Paper Moon	Paper Moon	Bogdanovich, Peter	Peter Bogdanovich	tt0070510
1746582221	1928	1928	Pay As You Enter	Pay as You Enter	Bacon, Lloyd	Lloyd Bacon	tt0019259
1746584778	1930	1930	Only Saps Work	Only Saps Work	Gardner, Cyril; Knopf, Edwin H.	Cyril Gardner	tt0021218
1746524540	1910	1910	What Great Bear Learned	What Great Bear Learned		William F. Haddock	tt1084753
1746218882	1980	1980	Seems Like Old Times	Seems Like Old Times	Sandrich, Jay	Jay Sandrich	tt0081480
1746513791	1916	1916	The Dumb Girl of Portici	The Dumb Girl of Portici	Weber, Lois; Smalley, Phillips	Phillips Smalley	tt0006614
1746520106	1925	1925	California Straight Ahead	California Straight Ahead	Pollard, Harry	Harry A. Pollard	tt0015663
1746202418	1970	1970	Little Fauss and Big Halsy	Little Fauss and Big Halsy	Furie, Sidney J.	Sidney J. Furie	tt0065989

1746410374	1988	1988	Die Hard	Die Hard	McTiernan, John	John McTiernan	tt0095016	
1746218833	1972	1972	The Candidate	The Candidate	Ritchie, Michael	Michael Ritchie	tt0068334	
1746583504	1931	1931	The Maltese Falcon	The Maltese Falcon	Del Ruth, Roy	Roy Del Ruth	tt0022111	
1746514938	1916	1916	Drugged Waters	Drugged Waters	Dowlan, William	William C. Dowlan	tt0006612	
1746524349	1914	1914	When Fate Leads Trump	When Fate Leads Trump	Handworth, Harry	Harry Handworth	tt0004797	
1746437418	1940	1940	Black Diamonds	Black Diamonds	Cabanne, Christy	Christy Cabanne	tt0032257	
1746570344	1952	1952	The Lusty Men	The Lusty Men	Ray, Nicholas	Nicholas Ray	tt0044860	
1746523272	1910	1910	The Schoolmaster of Mariposa	The Schoolmaster of Mariposa		Francis Boggs	tt0001873	
1746570735	1934	1934	Where Sinners Meet	Where Sinners Meet	Ruben, J. Walter	J. Walter Ruben	tt0025982	
1746454866	1995	1995	Wild Bill	Wild Bill	Hill, Walter	Walter Hill	tt0114938	
1746487618	1918	1918	Rosemary Climbs the Heights	Rosemary Climbs the Heights	Ingraham, Lloyd	Lloyd Ingraham	tt0010643	
1746424008	1941	1941	Dude Cowboy	Dude Cowboy	Howard, David	David Howard	tt0033560	
1746582235	1929	1929	Cheyenne	Cheyenne	Rogell, Albert	Albert S. Rogell	tt0019761	
1746569766	1953	1953	The Royal African Rifles	The Royal African Rifles	Selander, Lesley	Lesley Selander	tt0046254	
1746508704	1925	1925	Red Hot Tires	Red Hot Tires	Kenton, Erle C.	Erle C. Kenton	tt0016275	
1746574854	1936	1936	Speed	Speed	Marin, Edwin L.	Edwin L. Marin	tt0028291	
1746408428	1984	1984	The Karate Kid	The Karate Kid	Avildsen, John G.	John G. Avildsen	tt0087538	
**1746198114**	**1969**	**1969**	**Coming Apart**	**Coming Apart**	**Ginsberg, Milton Moses**	**Milton Moses Ginsberg**	**tt0064180**	1
1746578774	1961	1960	Not Tonight Henry	Not Tonight Henry	Connell, W. Merle	W. Merle Connell	tt0055239	
1746492727	1946	1946	The Missing Lady	The Missing Lady	Karlson, Phil	Phil Karlson	tt0038743	
1746566755	1933	1933	Jimmy and Sally	Jimmy and Sally	Tinling, James	James Tinling	tt0024195	
1746563994	1952	1952	Bend of the River	Bend of the River	Mann, Anthony	Anthony Mann	tt0044413	
1769970490	1967	1967	Justine	Justine	Johnsen, S. N.	Sande N. Johnsen	tt0137859	
1746583903	1930	1930	Framed	Framed	Archainbaud, George	George Archainbaud	tt0020900	
1746549872	1954	1954	Riders to the Stars	Riders to the Stars	Carlson, Richard	Richard Carlson	tt0046240	
1746569705	1954	1954	On the Waterfront	On the Waterfront	Kazan, Elia	Elia Kazan	tt0047296	
1746409361	1986	1986	The Karate Kid Part II	The Karate Kid Part II	Avildsen, John G.	John G. Avildsen	tt0091326	
1746218844	1973	1973	Detroit 9000	Detroit 9000	Marks, Arthur	Arthur Marks	tt0069966	
1746578889	1951	1951	The Painted Hills	The Painted Hills	Kress, Harold	Harold F. Kress	tt0043895	
1746524052	1917	1917	The Good for Nothing	The Good for Nothing	Blackwell, Carlyle	Carlyle Blackwell	tt0008025	
1746491967	1947	1947	Gun Talk	Gun Talk	Hillyer, Lambert	Lambert Hillyer	tt0039440	
1745340322	2004	2004	The Perfect Score	The Perfect Score	Robbins, Brian	Brian Robbins	tt0314498	
1746508723	1949	1948	Trouble Makers	Trouble Makers	Le Borg, Reginald	Reginald Le Borg	tt0040905	
1746583223	1930	1930	Charley's Aunt	Charley's Aunt	Christie, Al	Al Christie	tt0020754	
1746508428	1925	1925	Desperate Odds	Desperate Odds	Carpenter, Horace B.	Horace B. Carpenter	tt0015747	
1746584013	1936	1936	The Big Game	The Big Game	Nicholls, George	George Nichols Jr.	tt0027358	
1769970824	1915	1915	Otto's Cabaret	Otto's Cabaret	McKim, Edward	Edwin McKim	tt0204536	
1746424242	1941	1941	Rags to Riches	Rags to Riches	Kane, Joseph	Joseph Kane	tt0034080	
1745272091	2001	2001	America's Sweethearts	America's Sweethearts	Roth, Joe	Joe Roth	tt0265029	
1746565303	1932	1932	Emma	Emma	Brown, Clarence	Clarence Brown	tt0022854	
1746508883	1925	1925	Heartbound	Heartbound	Lambert, Glen	Glen Lambert	tt0219755	
1746475951	1947	1948	Slippy McGee	Slippy McGee	Kelley, Albert	Albert H. Kelley	tt0040800	
1746467352	1917	1917	The Girl Who Couldn't Grow Up	The Girl Who Couldn't Grow Up	Pollard, Harry	Harry A. Pollard	tt0175667	
1746497245	1918	1918	Humility	Humility	Pratt, Jack	Jack Pratt	tt0184595	
1746543076	1915	1915	Jack Chanty	Jack Chanty	Figman, Max	Max Figman	tt0005559	
1746421653	1940	1940	The Biscuit Eater	The Biscuit Eater	Heisler, Stuart	Stuart Heisler	tt0032254	
1746218899	1972	1972	The Rats Are Coming! The Werewolves Are Here!	The Rats Are Coming! The Werewolves Are H	Milligan, Andy	Andy Milligan	tt0069162	
1746520355	1926	1926	For Wives Only	For Wives Only	Heerman, Victor	Victor Heerman	tt0016898	
1745340018	2004	2004	Without a Paddle	Without a Paddle	Brill, Steven	Steven Brill	tt0364751	
1746524099	1910	1910	Romeo and Juliet in Our Town	Romeo and Juliet in Our Town			tt1390979	
1746249751	1977	1977	The Choirboys	The Choirboys	Aldrich, Robert	Robert Aldrich	tt0075845	
1746422951	1994	1994	The Shadow	The Shadow	Mulcahy, Russell	Russell Mulcahy	tt0111143	
1746574293	1960	1960	It Started in Naples	It Started in Naples	Shavelson, Melville	Melville Shavelson	tt0053957	
1746513181	1923	1923	What Love Will Do	What Love Will Do	Bradbury, Robert North	Robert N. Bradbury	tt0178096	
1746408634	1986	1985	Thunder Alley	Thunder Alley	Cardone, J. S.	J.S. Cardone	tt0090168	
1746437371	1943	1943	Fugitive from Sonora	Fugitive from Sonora	Bretherton, Howard	Howard Bretherton	tt0035907	
1746520788	1925	1925	After Business Hours	After Business Hours	St. Clair, Mal	Malcolm St. Clair	tt0015545	

1746423039	1996	1996	The Chamber	The Chamber	Foley, James	James Foley	tt0115862
1746392882	1983	1983	The Lonely Lady	The Lonely Lady	Sasdy, Peter	Peter Sasdy	tt0085863
1746585046	1940	1940	The Golden Trail	The Golden Trail	Herman, Al	Albert Herman	tt0032540
1746507728	1925	1925	Capital Punishment	Capital Punishment	Hogan, James P.	James P. Hogan	tt0015669
1746500987	1922	1922	Heroes and Husbands	Heroes and Husbands	Withey, Chet	Chester Withey	tt0134720
1746502349	1915	1915	The Apaches of Paris	The Apaches of Paris	Ellis, Robert	Robert Ellis	tt0004902
1746566904	1933	1934	What's Your Racket	What's Your Racket?	Guiol, Fred	Fred Guiol	tt0165538
1746554018	1964	1964	Seven Days in May	Seven Days in May	Frankenheimer, John	John Frankenheimer	tt0058576
1746577532	1936	1936	Daniel Boone	Daniel Boone	Howard, David	David Howard	tt0027499
1746502422	1915	1915	Sweet Alyssum	Sweet Alyssum	Campbell, Colin	Colin Campbell	tt0006128
1746520048	1926	1926	Tin Hats	Tin Hats	Sedgwick, Edward	Edward Sedgwick	tt0017473
1746219172	1971	1971	Plaza Suite	Plaza Suite	Hiller, Arthur	Arthur Hiller	tt0067589
1745340979	2010	2010	Robin Hood	Robin Hood	Scott, Ridley	Ridley Scott	tt0955308
1746577760	1937	1937	Woman-Wise	Woman Wise	Dwan, Allan	Allan Dwan	tt0029791
1746271974	1966	1966	A Fine Madness	A Fine Madness	Kershner, Irvin	Irvin Kershner	tt0060414
1746578721	1950	1950	Mystery Submarine	Mystery Submarine	Sirk, Douglas	Douglas Sirk	tt0042772
1746498276	1920	1920	The Coast of Opportunity	The Coast of Opportunity	Warde, Ernest C.	Ernest C. Warde	tt0193826
1746580894	1936	1936	The Invisible Ray	The Invisible Ray	Hillyer, Lambert	Lambert Hillyer	tt0027800
1746581076	1951	1951	Fingerprints Don't Lie	Fingerprints Don't Lie	Newfield, Samuel	Sam Newfield	tt0043535
1746565896	1957	1957	The Sad Sack	The Sad Sack	Marshall, George	George Marshall	tt0050922
1746583758	1928	1928	Blockade	Blockade	Seitz, George B.	George B. Seitz	tt0018709
1746524524	1916	1916	Two News Items	Two News Items	Sloman, Edward	Edward Sloman	tt0468235
1746508416	1924	1924	The Slanderers	The Slanderers	Ross, Nat	Nat Ross	tt0015340
1746581949	1930	1930	Passion Flower	Passion Flower	de Mille, William	William C. de Mille	tt0021240
1746218999	1971	1971	Point of Terror	Point of Terror	Nicol, Alex	Alex Nicol	tt0067594
1746581225	1950	1950	Triple Trouble	Triple Trouble	Yarbrough, Jean	Jean Yarbrough	tt0043071
1746583527	1931	1931	Law of the Tong	The Law of the Tong	Collins, Lew	Lewis D. Collins	tt0022061
1746582100	1930	1930	Sin Takes a Holiday	Sin Takes a Holiday	Stein, Paul L.	Paul L. Stein	tt0021377
1746530713	1948	1948	The Wreck of the Hesperus	The Wreck of the Hesperus	Hoffman, John	John Hoffman	tt0040977
1746500106	1924	1924	Trigger Finger	Trigger Fingers	Eason, Breezy Reeves	B. Reeves Eason	tt0015430
1746423751	1998	1998	Black Dog	Black Dog	Hooks, Kevin	Kevin Hooks	tt0120610
1746581008	1961	1961	Creature From the Haunted Sea	Creature from the Haunted Sea	Corman, Roger	Roger Corman	tt0054768
1746435686	1943	1943	The Crime Doctor's Strangest Case	The Crime Doctor's Strangest Case	Forde, Eugene J.	Eugene Forde	tt0035767
1746566294	1954	1954	The Steel Cage	The Steel Cage	Doniger, Walter	Walter Doniger	tt0047524
1746509020	1924	1924	Young Ideas	Young Ideas	Hill, Robert F.	Robert F. Hill	tt0015519
1746570291	1933	1933	Terror Aboard	Terror Aboard	Sloane, Paul	Paul Sloane	tt0024649
1746578966	1951	1951	Hot Lead	Hot Lead	Gilmore, Stuart	Stuart Gilmore	tt0043657
1746500671	1926	1926	The Sea Beast	The Sea Beast	Webb, Millard	Millard Webb	tt0017354
1746210709	1971	1971	Willard	Willard	Mann, Daniel	Daniel Mann	tt0067991
1746578553	1961	1961	The Phantom Planet	The Phantom Planet	Marshall, William	William Marshall	tt0055294
1746465298	1942	1942	Secret Agent of Japan	Secret Agent of Japan	Pichel, Irving	Irving Pichel	tt0035298
1746514741	1927	1927	Better Days	Better Days	Mattison, Frank S.	Frank S. Mattison	tt0017670
1746500575	1925	1925	Smilin' at Trouble	Smilin' at Trouble	Garson, Harry	Harry Garson	tt0165481
1746523338	1916	1916	The Old Watchman	The Old Watchman	Kent, Leon D.	Leon De La Mothe	tt0864859
1746219374	1971	1971	The Panic in Needle Park	The Panic in Needle Park	Schatzberg, Jerry	Jerry Schatzberg	tt0067549
1746566340	1958	1958	Girls on the Loose	Girls on the Loose	Henreid, Paul	Paul Henreid	tt0051663
1769975292	1916	1916	Eleanor's Catch	Eleanor's Catch	Madison, Cleo	Cleo Madison	tt0251069
1746560011	1965	1965	The Sandpiper	The Sandpiper	Minnelli, Vincente	Vincente Minnelli	tt0059674
1746514418	1910	1910	The Stepmother	The Stepmother		Sidney Olcott	tt0255619
1746571660	1931	1931	Night Beat	Night Beat	Seitz, George	George B. Seitz	tt0022206
1746508244	1926	1926	Across the Pacific	Across the Pacific	Del Ruth, Roy	Roy Del Ruth	tt0016575
1746454699	1944	1944	Tampico	Tampico	Mendes, Lothar	Lothar Mendes	tt0037346
1746501173	1915	1915	A Man's Making	A Man's Making	Pratt, Jack	Jack Pratt	tt0005710
1746574965	1933	1934	The Whirlwind Rider	The Whirlwind Rider		Robert J. Horner	tt0176323
1746218686	1971	1971	Who Is Harry Kellerman and Why Is He Saying Th	Who Is Harry Kellerman and Why Is He Sayir	Grosbard, Ulu	Ulu Grosbard	tt0067980
1746577896	1935	1935	Life Begins at 40	Life Begins at 40	Marshall, George	George Marshall	tt0026629
1746421295	1994	1994	Love Affair	Love Affair	Caron, Glenn Gordon	Glenn Gordon Caron	tt0110391

1746251270	1979	1979	"10"	10	Edwards, Blake	Blake Edwards	tt0078721	
1746567451	1933	1933	The Vampire Bat	The Vampire Bat	Strayer, Frank	Frank R. Strayer	tt0024727	
1746574009	1960	1960	The Horse with the Flying Tail	The Horse with the Flying Tail	Lansburgh, Larry	Larry Lansburgh	tt0054993	
1746507611	1927	1927	The Thirteenth Juror	The Thirteenth Juror	Laemmle, Edward	Edward Laemmle	tt0018481	
1746561576	1932	1932	Midnight Morals	Midnight Morals	Hopper, E. Mason	B. Reeves Eason	tt0023219	
1746218777	1972	1972	Trouble Man	Trouble Man	Dixon, Ivan	Ivan Dixon	tt0069414	
1746566217	1953	1953	Sky Commando	Sky Commando	Sears, Fred F.	Fred F. Sears	tt0046326	
1746560788	1932	1932	Two Kinds of Women	Two Kinds of Women	deMille, William C.	William C. de Mille	tt0023627	
1746583683	1931	1931	Ambassador Bill	Ambassador Bill	Taylor, Sam	Sam Taylor	tt0021606	
1746574810	1935	1935	The Crusades	The Crusades	DeMille, Cecil B.	Cecil B. DeMille	tt0026249	
1746235338	1974	1974	Homebodies	Homebodies	Yust, Larry	Larry Yust	tt0071617	
1746410156	1992	1992	Boomerang	Boomerang	Hudlin, Reginald	Reginald Hudlin	tt0103859	
1746503236	1921	1921	My Lady Friends	My Lady Friends	Ingraham, Lloyd	Lloyd Ingraham	tt0012487	
1746568225	1934	1934	Racketeer Round-Up	Racketeer Round up	Hoyt, Robert	Jerry Callahan	tt0144227	
1746569843	1960	1960	Four Fast Guns	Four Fast Guns	Hole, William J.	William J. Hole Jr.	tt0052819	
**1746515040**	**1922**	**1922**	**A Dangerous Adventure**	**A Dangerous Adventure**	**Warner, Sam; Warner, Jack L.**	**Jack L. Warner**	**tt0013046**	1
1746500111	1949	1949	City Across the River	City Across the River	Shane, Maxwell	Maxwell Shane	tt0041251	
1746497076	1919	1919	Loot	Loot	Dowlan, William C.	William C. Dowlan	tt0010384	
1746248343	1966	1966	Waco	Waco	Springsteen, R. G.	R.G. Springsteen	tt0061169	
1746500364	1949	1949	Too Late for Tears	Too Late for Tears	Haskin, Byron	Byron Haskin	tt0041968	
1746500092	1924	1924	The Age of Innocence	The Age of Innocence	Ruggles, Wesley	Wesley Ruggles	tt0014651	
1746554824	1955	1955	Santa Fe Passage	Santa Fe Passage	Witney, William	William Witney	tt0048584	
1745342021	2004	2004	The Last Shot	The Last Shot	Nathanson, Jeff	Jeff Nathanson	tt0357054	
1746514958	1916	1916	The Writing on the Wall	The Writing on the Wall	Johnson, Tefft	Tefft Johnson	tt0007594	
1746500225	1926	1926	Christine of the Big Tops	Christine of the Big Tops	Mayo, Archie	Archie Mayo	tt0016727	
1746583005	1936	1936	The Unknown Ranger	The Unknown Ranger	Bennet, Spencer Gordon	Spencer Gordon Bennet	tt0028448	
1746532834	1928	1928	The Magnificent Flirt	The Magnificent Flirt	D'Arrast, Harry d'Abbadie	Harry d'Abbadie d'Arrast	tt0019125	
1746577608	1934	1934	Riptide	Riptide	Goulding, Edmund	Edmund Goulding	tt0025720	
1746574386	1962	1962	Shoot Out at Big Sag	Shoot Out at Big Sag	Kay, Roger	Roger Kay	tt0056484	
1746491963	1948	1948	The Sign of the Ram	The Sign of the Ram	Sturges, John	John Sturges	tt0040785	
1746394508	1982	1981	The Chosen	The Chosen	Kagan, Jeremy Paul	Jeremy Kagan	tt0082175	
1746584734	1929	1929	The Man and the Moment	The Man and the Moment	Fitzmaurice, George	George Fitzmaurice	tt0020136	
1745302758	2006	2006	Who Killed the Electric Car?	Who Killed the Electric Car?	Paine, Chris	Chris Paine	tt0489037	
1746199235	1969	1969	Easy Rider	Easy Rider	Hopper, Dennis	Dennis Hopper	tt0064276	
1746465137	1945	1945	Danger Signal	Danger Signal	Florey, Robert	Robert Florey	tt0037632	
1745342500	2009	2009	Miss March	Miss March	Cregger, Zach ; Moore, Trevor	Zach Cregger	tt1151922	
1746580239	1937	1937	Armored Car	Armored Car	Foster, Lewis R.	Lewis R. Foster	tt0028585	
1746454616	1945	1945	Bells of Rosarita	Bells of Rosarita	McDonald, Frank	Frank McDonald	tt0037535	
1746498260	1920	1920	The Lone Hand	The Lone Hand	Smith, Cliff	Clifford Smith	tt0196711	
1746575025	1938	1938	The Rangers' Round-Up	The Rangers' Round Up	Newfield, Sam	Sam Newfield	tt0030653	
1746566525	1952	1952	Love Is Better Than Ever	Love Is Better Than Ever	Donen, Stanley	Stanley Donen	tt0044852	
1746454764	1998	1998	Godzilla	Godzilla	Emmerich, Roland	Roland Emmerich	tt0120685	
1746199535	1969	1969	The Bridge at Remagen	The Bridge at Remagen	Guillermin, John	John Guillermin	tt0064110	
1746569648	1958	1958	Hong Kong Affair	Hong Kong Affair	Heard, Paul F.	Paul F. Heard	tt0051735	
1746577758	1936	1936	M'liss	M'Liss	Nicholls, George	George Nichols Jr.	tt0027921	
1746235698	1974	1974	Herbie Rides Again	Herbie Rides Again	Stevenson, Robert	Robert Stevenson	tt0071607	
1746219203	1975	1975	Hearts of the West	Hearts of the West	Zieff, Howard	Howard Zieff	tt0073096	
1746496248	1921	1921	Wise Husbands	Wise Husbands	Reicher, Frank	Frank Reicher	tt0012854	
1746454198	2000	2000	Get Carter	Get Carter	Kay, Stephen	Stephen Kay	tt0208988	
2007987176	1928	1928	All Washed Up	All Washed Up	Herman, Albert	Albert Herman	tt0412326	
1746487740	1917	1917	A Jewel in Pawn	A Jewel in Pawn	Conway, Jack	Jack Conway	tt0008147	
1746576626	1910	1910	The Dancing Girl of Butte	The Dancing Girl of Butte	Griffith, D. W.	D.W. Griffith	tt0001176	
1746580402	1936	1936	The House of a Thousand Candles	The House of a Thousand Candles	Lubin, Arthur	Arthur Lubin	tt0027764	
1746550120	1954	1954	Carnival Story	Carnival Story	Neumann, Kurt	Kurt Neumann	tt0046829	
1746566122	1954	1954	Roogie's Bump	Roogie's Bump	Young, Harold	Harold Young	tt0047428	
1745271906	2001	2001	Blow	Blow	Demme, Ted	Ted Demme	tt0221027	
1746554108	1964	1964	Honeymoon Hotel	Honeymoon Hotel	Levin, Henry	Henry Levin	tt0058204	

1746582311	1929	1929	The Forward Pass	The Forward Pass	Cline, Eddie	Edward F. Cline	tt0019895	
1746420391	1989	1988	Buy & Cell	Buy & Cell	Boris, Robert	Robert Boris	tt0096997	
1746523286	1916	1916	La Vie De Boheme	La vie de Bohème	Capellani, Albert	Albert Capellani	tt0006456	
1746580648	1935	1935	Dr. Socrates	Dr. Socrates	Dieterle, William	William Dieterle	tt0026293	
1746475504	1944	1944	Cowboy from Lonesome River	Cowboy from Lonesome River	Kline, Benjamin	Benjamin H. Kline	tt0036726	
1746500655	1926	1926	The Broadway Gallant	The Broadway Gallant	Mason, Noel	Noel M. Smith	tt0016683	
1746218823	1973	1973	Jesus Christ Superstar	Jesus Christ Superstar	Jewison, Norman	Norman Jewison	tt0070239	
1746454366	1943	1943	Guadalcanal Diary	Guadalcanal Diary	Seiler, Lewis	Lewis Seiler	tt0035957	
1746501622	1910	1910	A Westerner's Way	A Westerner's Way		Gilbert M. 'Broncho Billy' Anderson	tt0001451	
1769977352	1975	1975	Linda Lovelace for President	Linda Lovelace for President	Guzman, Claudio	Claudio Guzmán	tt0074800	
1746581337	1910	1910	The Kentucky Pioneer	The Kentucky Pioneer			tt1371128	
1746574868	1935	1935	The Great Impersonation	The Great Impersonation	Crosland, Alan	Alan Crosland	tt0026438	
1746500507	1949	1949	Forgotten Women	Forgotten Women	Beaudine, William	William Beaudine	tt0041384	
1746577438	1961	1961	The Silent Call	The Silent Call	Bushelman, John	John A. Bushelman	tt0055449	
1746474390	1948	1948	Race Street	Race Street	Marin, Edwin L.	Edwin L. Marin	tt0040719	
1746514896	1923	1923	The Marriage Market	The Marriage Market	Le Saint, Edward J.	Edward LeSaint	tt0014259	
1746553679	1964	1964	Bedtime Story	Bedtime Story	Levy, Ralph	Ralph Levy	tt0057878	
1746573985	1957	1957	Joe Butterfly	Joe Butterfly	Hibbs, Jesse	Jesse Hibbs	tt0050564	
1746501162	1916	1916	The Idol of the Stage	The Idol of the Stage	Garrick, Richard	Richard Garrick	tt0145833	
1746555416	1956	1956	Secret of Treasure Mountain	Secret of Treasure Mountain	Friedman, Seymour	Seymour Friedman	tt0049732	
1746549870	1955	1955	The Marauders	The Marauders	Mayer, Gerald	Gerald Mayer	tt0048351	
1746520976	1950	1950	Let's Dance	Let's Dance	McLeod, Norman Z.	Norman Z. McLeod	tt0042674	
1746250614	1975	1975	The Eiger Sanction	The Eiger Sanction	Eastwood, Clint	Clint Eastwood	tt0072926	
1746584905	1940	1940	Give Us Wings	Give Us Wings	Lamont, Charles	Charles Lamont	tt0032533	
**1746503025**	**1915**	**1915**	**The Breath of Araby**	**The Breath of Araby**	**Gaskill, Charles L.**	**Charles L. Gaskill**	**tt0004998**	1
1746572364	1910	1910	The Clay Baker	The Clay Baker			tt1146339	
1746453759	1941	1941	Secret of the Wastelands	Secret of the Wastelands	Abrahams, Derwin	Derwin Abrahams	tt0034166	
1746475931	1944	1945	The Man in Half Moon Street	The Man in Half Moon Street	Murphy, Ralph	Ralph Murphy	tt0037046	
1746433537	1990	1990	Mr. Destiny	Mr. Destiny	Orr, James	James Orr	tt0100201	
1746393964	1989	1988	Let's Get Lost	Let's Get Lost	Weber, Bruce	Bruce Weber	tt0095515	
1746577565	1935	1935	The Girl Friend	The Girl Friend	Buzzell, Edward N.	Edward Buzzell	tt0026411	
1746573356	1932	1932	My Pal the King	My Pal, the King	Neumann, Kurt	Kurt Neumann	tt0023255	
1746410662	1993	1992	The Opposite Sex and How to Live with Them	The Opposite Sex and How to Live with Ther	Meshekoff, Matthew	Matthew Meshekoff	tt0105063	
1746235872	1974	1974	Benji	Benji	Camp, Joe	Joe Camp	tt0071206	
1746514316	1915	1915	Vanity Fair	Vanity Fair	Nowland, Eugene	Charles Brabin	tt0006210	
1746583950	1931	1931	The Montana Kid	The Montana Kid	Fraser, Harry	Harry L. Fraser	tt0022159	
1746208376	1967	1967	The Christmas Kid	The Christmas Kid	Pink, Sidney	Sidney W. Pink	tt0061844	
1746574434	1958	1957	Spanish Affair	Spanish Affair	Siegel, Donald	Luis Marquina	tt0052230	
1746492378	1946	1946	The Glass Alibi	The Glass Alibi	Wilder, W. Lee	W. Lee Wilder	tt0038565	
1746574928	1933	1933	The Wrecker	The Wrecker	Rogell, Albert	Albert S. Rogell	tt0024790	
1746503652	1920	1920	Nurse Marjorie	Nurse Marjorie	Taylor, William Desmond	William Desmond Taylor	tt0011522	
1746565201	1952	1952	Okinawa	Okinawa	Jason, Leigh	Leigh Jason	tt0044987	
1746570369	1934	1934	Six of a Kind	Six of a Kind	McCarey, Leo	Leo McCarey	tt0025799	
1746508446	1926	1926	A Desperate Moment	A Desperate Moment	Dawn, Jack	Jack Dawn	tt0016779	
1746582240	1930	1930	Sarah and Son	Sarah and Son	Arzner, Dorothy	Dorothy Arzner	tt0021335	
1746583099	1938	1938	When G-Men Step In	When G Men Step In	Coleman, C. C.	Charles C. Coleman	tt0030967	
1746570381	1934	1934	Massacre	Massacre	Crosland, Alan	Alan Crosland	tt0025479	
1746581590	1928	1928	Turn Back the Hours	Turn Back the Hours	Bretherton, Howard	Howard Bretherton	tt0019497	
1746585422	1940	1940	Kit Carson	Kit Carson	Seitz, George B.	George B. Seitz	tt0032670	
1746555529	1954	1954	Monster from the Ocean Floor	Monster from the Ocean Floor	Ordung, Wyott	Wyott Ordung	tt0047244	
1746585200	1930	1930	Not So Dumb	Not So Dumb	Vidor, King	King Vidor	tt0021189	
1746498668	1921	1921	Dynamite Allen	Dynamite Allen	Henderson, Del	Dell Henderson	tt0012128	
1746271890	1967	1967	You've Got To Be Smart	You've Got to Be Smart	Kadison, Ellis	Ellis Kadison	tt0062514	
1746508881	1925	1925	Waking Up the Town	Waking Up the Town	Cruze, James	James Cruze	tt0016497	
1746509344	1925	1925	The Lure of the Wild	The Lure of the Wild	Strayer, Frank R.	Frank R. Strayer	tt0016057	
1746560397	1965	1965	Battle of the Bulge	Battle of the Bulge	Annakin, Ken	Ken Annakin	tt0058947	
1746533057	1923	1923	The Satin Girl	The Satin Girl	Rosson, Arthur	Arthur Rosson	tt0014437	

1746566051	1955	1955	A Life in the Balance	A Life in the Balance	Horner, Harry	Harry Horner	tt0048301	
1746581883	1930	1930	The Pay Off	The Pay Off	Sherman, Lowell	Lowell Sherman	tt0021092	
1746583867	1929	1929	The Doctor's Secret	The Doctor's Secret	de Mille, William C.	William C. de Mille	tt0019827	
1746523968	1915	1915	Scandal	Scandal	Weber, Lois; Smalley, Phillips	Phillips Smalley	tt0006008	
1746235348	1973	1973	The Werewolf of Washington	The Werewolf of Washington	Ginsberg, Milton Moses	Milton Moses Ginsberg	tt0070908	
**1746578014**	**1934**	**1934**	**Jealousy**	**Jealousy**	**Neill, Roy William**	**Roy William Neill**	**tt0025327**	1
1746500057	1946	1946	Three Strangers	Three Strangers	Negulesco, Jean	Jean Negulesco	tt0039029	
1746500094	1949	1949	Square Dance Jubilee	Square Dance Jubilee	Landres, Paul	Paul Landres	tt0041914	
1746581389	1928	1928	The Trail of Courage	Trail of Courage	Fox, Wallace W.	Wallace Fox	tt0019490	
1745303149	2005	2005	Red Eye	Red Eye	Craven, Wes	Wes Craven	tt0421239	
1746453607	1944	1944	Westward Bound	Westward Bound	Tansey, Robert	Robert Emmett Tansey	tt0037456	
1746553545	1965	1965	Man Outside	Man Outside	Marzano, Joseph	Joseph Marzano	tt0289308	
1746532855	1914	1914	The Christian	The Christian	Thomson, Frederick A.	Frederick A. Thomson	tt0003771	
1746580626	1938	1938	The Rage of Paris	The Rage of Paris	Koster, Henry	Henry Koster	tt0030652	
1746487612	1921	1921	The Whistle	The Whistle	Hillyer, Lambert	Lambert Hillyer	tt0012839	
1746585336	1930	1930	Sins of the Children	The Sins of the Children	Wood, Sam	Sam Wood	tt0021382	
1746585329	1929	1929	Don't Get Nervous	Don't Get Nervous		Bryan Foy	tt0230150	
1769969881	1912	1912	Winnie's Dance	Winnie's Dance		Ashley Miller	tt0438605	
1746487029	1918	1918	The Doctor and the Woman	The Doctor and the Woman	Weber, Lois	Phillips Smalley	tt0009016	
1745342458	2001	2001	Soul Survivors	Soul Survivors	Carpenter, Steve	Stephen Carpenter	tt0218619	
1746454286	1941	1941	Six-Gun Gold	Six Gun Gold	Howard, David	David Howard	tt0034195	
1746524714	1914	1914	The Little Angel of Canyon Creek	The Little Angel of Canyon Creek	Sturgeon, Rollin S.	Rollin S. Sturgeon	tt0004234	
1746423732	1941	1941	Reg'lar Fellers	Reg'lar Fellers	Dreifuss, Arthur	Arthur Dreifuss	tt0034089	
1746408461	1990	1989	Longtime Companion	Longtime Companion	Rene, Norman	Norman René	tt0100049	
1746487294	1920	1920	On with the Dance	On with the Dance	Fitzmaurice, George	George Fitzmaurice	tt0123205	
1746520068	1925	1925	Enemies of Youth	Enemies of Youth	Berthelet, Arthur	Arthur Berthelet	tt0015779	
1746574750	1933	1933	A Bedtime Story	A Bedtime Story	Taurog, Norman	Norman Taurog	tt0023787	
1746572000	1931	1931	It's a Wise Child	It's a Wise Child	Leonard, Robert Z.	Robert Z. Leonard	tt0022007	
1746515096	1921	1921	Society Snobs	Society Snobs	Henley, Hobart	Hobart Henley	tt0012709	
1746503043	1911	1911	Lassoing Wild Animals in Africa	Lassoing Wild Animals in Africa	Kearton (?), Cherry	Cherry Kearton	tt4850720	
1746512736	1915	1915	The Immigrant	The Immigrant	Melford, George	George Melford	tt0005535	
1746465421	1941	1941	Tom, Dick and Harry	Tom, Dick and Harry	Kanin, Garson	Garson Kanin	tt0034299	
1746561331	1957	1957	Silk Stockings	Silk Stockings	Mamoulian, Rouben	Rouben Mamoulian	tt0050972	
1746582662	1938	1938	Bar 20 Justice	Bar 20 Justice	Selander, Lesley	Lesley Selander	tt0029895	
1746520858	1948	1948	The Velvet Touch	The Velvet Touch	Gage, John	Jack Gage	tt0040934	
1769971984	1969	1969	Temporary Wives	Temporary Wives	Shamblin, Gene	Gene Shamblin	tt0259628	
1746567007	1932	1932	This Sporting Age	This Sporting Age	Bennison, Andrew W.; Erickson, A. F.	Andrew Bennison	tt0023586	
1746577982	1936	1936	Below the Deadline	Below the Deadline	Lamont, Charles	Charles Lamont	tt0027348	
1745357625	2008	2008	W.	W.	Stone, Oliver	Oliver Stone	tt1175491	
1746410813	1987	1987	Sweet Lorraine	Sweet Lorraine	Gomer, Steve	Steve Gomer	tt0094086	
1746501353	1915	1915	The Price of Her Silence	The Price of Her Silence		Eugene Moore	tt0005924	
1746438629	1941	1941	Men of the Timberland	Men of the Timberland	Rawlins, John	John Rawlins	tt0033903	
1746582059	1928	1928	The Big Hop	The Big Hop	Horne, James W.	James W. Horne	tt0018698	
**1746465060**	**1947**	**1947**	**Calcutta**	**Calcutta**	**Farrow, John**	**John Farrow**	**tt0039235**	1
1746435563	1943	1943	Wild Horse Rustlers	Wild Horse Rustlers	Newfield, Sam	Sam Newfield	tt0036536	
1746580702	1936	1936	Our Relations	Our Relations	Lachman, Harry	Harry Lachman	tt0028070	
1746570022	1934	1934	Call It Luck	Call It Luck	Tinling, James	James Tinling	tt0024943	
1746250941	1980	1980	Windows	Windows	Willis, Gordon	Gordon Willis	tt0081759	
1746532838	1923	1923	Trilby	Trilby	Young, James	James Young	tt0014562	
1746582423	1935	1935	The Little Colonel	The Little Colonel	Butler, David	David Butler	tt0016029	
1746583519	1951	1951	Saddle Legion	Saddle Legion	Selander, Lesley	Lesley Selander	tt0043987	
1746438717	1999	1999	The Hand Behind the Mouse: The Ub Iwerks Story	The Hand Behind the Mouse: The Ub Iwerks	Iwerks, Leslie	Leslie Iwerks	tt0179496	
1746465077	1946	1946	The Chase	The Chase	Ripley, Arthur D.	Arthur Ripley	tt0038409	
1746393683	1983	1983	National Lampoon's Vacation	National Lampoon's Vacation	Ramis, Harold	Harold Ramis	tt0085995	
1746566635	1932	1932	Border Devils	Border Devils	Nigh, William	William Nigh	tt0022705	
1745309431	2005	2005	Casanova	Casanova	Hallström, Lasse	Lasse Hallström	tt0402894	
1746438628	1943	1943	Nobody's Darling	Nobody's Darling	Mann, Anthony	Anthony Mann	tt0036211	

1746453644	1941	1941	Romance of the Rio Grande	Romance of the Rio Grande	Leeds, Herbert I.	Herbert I. Leeds	tt0034124
1746507812	1949	1949	Take One False Step	Take One False Step	Erskine, Chester	Chester Erskine	tt0041945
1746570978	1934	1934	Merry Wives of Reno	Merry Wives of Reno	Humberstone, H. Bruce	H. Bruce Humberstone	tt0025494
1746492472	1946	1946	A Stolen Life	A Stolen Life	Bernhardt, Curtis	Curtis Bernhardt	tt0038984
1746422515	1996	1996	One Fine Day	One Fine Day	Hoffman, Michael	Michael Hoffman	tt0117247
1746524443	1922	1922	Broken Chains	Broken Chains	Holubar, Allen	Allen Holubar	tt0012980
1746574407	1959	1958	Anna Lucasta	Anna Lucasta	Laven, Arnold	Arnold Laven	tt0051362
1746408628	1987	1986	Allan Quatermain and the Lost City of Gold	Allan Quatermain and the Lost City of Gold	Nelson, Gary	Gary Nelson	tt0092534
1746516328	1919	1919	Six Feet Four	Six Feet Four	King, Henry	Henry King	tt0010703
1746454236	1994	1994	Paul Bowles: The Complete Outsider	Paul Bowles: The Complete Outsider	Warnow, Catherine; Weinreich, Regi	Catherine Warnow	tt0110798
1746555021	1957	1957	The Girl in Black Stockings	The Girl in Black Stockings	Koch, Howard W.	Howard W. Koch	tt0050439
1746475925	1945	1945	Here Is Germany	Here Is Germany		Frank Capra	tt0037773
1746577708	1935	1935	Smart Girl	Smart Girl	Scotto, Aubrey	Aubrey Scotto	tt0027013
1746566076	1956	1956	Death of a Scoundrel	Death of a Scoundrel	Martin, Charles	Charles Martin	tt0049130
1746491884	1947	1947	Wild Horse Mesa	Wild Horse Mesa	Grissell, Wallace A.	Wallace Grissell	tt0179533
1746491871	1946	1946	Romance of the West	Romance of the West	Emmett, Robert	Robert Emmett Tansey	tt0038892
1746582103	1910	1910	In the Border States	In the Border States	Griffith, D. W.	D.W. Griffith	tt0001265
1746422068	1999	1999	American Movie	American Movie	Smith, Chris	Chris Smith	tt0181288
1746502734	1915	1915	The Shadows of a Great City	The Shadows of a Great City		Herbert Blaché	tt0006030
1746438602	1941	1941	Scattergood Pulls the Strings	Scattergood Pulls the Strings	Cabanne, Christy	Christy Cabanne	tt0034155
1746581104	1951	1951	As Young As You Feel	As Young as You Feel	Jones, Harmon	Harmon Jones	tt0043299
1746520689	1926	1926	Love's Blindness	Love's Blindness	Dillon, John Francis	John Francis Dillon	tt0017086
1745293507	2006	2006	Grandma's Boy	Grandma's Boy	Goossen, Nicholaus	Nicholaus Goossen	tt0456554
1746509050	1924	1924	Broadway After Dark	Broadway After Dark	Bell, Monta	Monta Bell	tt0014744
1746233779	1966	1966	Harper	Harper	Smight, Jack	Jack Smight	tt0060490
1746520159	1924	1924	Nellie, The Beautiful Cloak Model	Nellie the Beautiful Cloak Model	Flynn, Emmett	Emmett J. Flynn	tt0015166
1745359219	2002	2002	Cherish	Cherish	Taylor, Finn	Finn Taylor	tt0298798
1746409309	1992	1992	Consenting Adults	Consenting Adults	Pakula, Alan J.	Alan J. Pakula	tt0104006
1746582404	1937	1937	Navy Blue and Gold	Navy Blue and Gold	Wood, Sam	Sam Wood	tt0029297
1746433714	1996	1996	Dragonheart	DragonHeart	Cohen, Rob	Rob Cohen	tt0116136
1746581326	1959	1960	Career Girl	Career Girl	David, Harold	Harold David	tt0052674
1746487520	1918	1918	A Lion of the Hills	The Lion of the Hills		Lambert Hillyer	tt0332209
1746465153	1942	1942	Native Land	Native Land	Hurwitz, Leo; Strand, Paul	Leo Hurwitz	tt0035112
1746583805	1929	1929	Thru Different Eyes	Thru Different Eyes	Blystone, John	John G. Blystone	tt0020497
1746465091	1947	1947	Violence	Violence	Bernhard, Jack	Jack Bernhard	tt0039960
1746423973	1942	1942	Army Surgeon	Army Surgeon	Sutherland, A. Edward	A. Edward Sutherland	tt0034473
1746520603	1925	1925	The Pace That Thrills	The Pace That Thrills	Campbell, Webster	Webster Campbell	tt0016193
1745393833	2008	2007	Chris & Don: A Love Story	Chris & Don. A Love Story	Santi, Guido ; Mascara, Tina	Tina Mascara	tt1138002
1746394455	1986	1985	Seven Minutes in Heaven	Seven Minutes in Heaven	Feferman, Linda	Linda Feferman	tt0089997
1746570229	1934	1934	British Agent	British Agent	Curtiz, Michael	Michael Curtiz	tt0024915
1746569851	1960	1960	A Breath of Scandal	A Breath of Scandal	Curtiz, Michael	Michael Curtiz	tt0053675
1746523374	1916	1916	The Dollar and the Law	The Dollar and the Law	North, Wilfred	Wilfrid North	tt0157548
1746507788	1949	1949	The Big Sombrero	The Big Sombrero	McDonald, Frank	Frank McDonald	tt0041177
1745309401	2007	2007	The Nanny Diaries	The Nanny Diaries	Pulcini, Robert; Berman, Shari Sprin	Shari Springer Berman	tt0489237
1746524074	1916	1916	The Bugle Call	The Bugle Call	Barker, Reginald	Reginald Barker	tt0006475
1746491862	1944	1944	Frenchman's Creek	Frenchman's Creek	Leisen, Mitchell	Mitchell Leisen	tt0036840
1746509045	1926	1926	Eyes of the Desert	Eyes of the Desert	Reel, Frederick	Frederick Reel Jr.	tt0322229
1746574811	1937	1936	Sing Me a Love Song	Sing Me a Love Song	Enright, Ray	Ray Enright	tt0028254
1746435531	1942	1942	Tough As They Come	Tough As They Come	Nigh, William	William Nigh	tt0035461
1746474493	1947	1947	Three on a Ticket	Three on a Ticket	Newfield, Sam	Sam Newfield	tt0039899
1746454972	1997	1997	Jungle 2 Jungle	Jungle 2 Jungle	Pasquin, John	John Pasquin	tt0119432
1746486131	1919	1919	The Long Arm of Mannister	The Long Arm of Mannister	Bracken, Bertram	Bertram Bracken	tt0010380
1746271198	1979	1979	Meteor	Meteor	Neame, Ronald	Ronald Neame	tt0079550
1746530749	1949	1949	It's a Great Feeling	It's a Great Feeling	Butler, David	David Butler	tt0041515
1746419403	1986	1986	Running Scared	Running Scared	Hyams, Peter	Peter Hyams	tt0091875
1769969356	1964	1964	White Slaves of Chinatown	White Slaves of Chinatown	Mawra, Joseph P.	Joseph P. Mawra	tt0129598
1746475533	1945	1945	Love Letters	Love Letters	Dieterle, William	William Dieterle	tt0037885

1745302884	2007	2007	Underdog	Underdog	Du Chau, Frederik	Frederik Du Chau	tt0467110
1746419804	1990	1990	The Hunt for Red October	The Hunt for Red October	McTiernan, John	John McTiernan	tt0099810
1746520489	1925	1925	The Fugitive	The Fugitive	Wilson, Ben	Ben F. Wilson	tt0133783
1746553587	1965	1965	The Outlaws IS Coming	The Outlaws Is Coming	Maurer, Norman	Norman Maurer	tt0059558
1746509572	1949	1949	Project X	Project X	Montagne, Edward J.	Edward Montagne	tt0041770
1746486310	1919	1919	A Fight for Love	A Fight for Love	Ford, Jack	John Ford	tt0010119
1746269662	1979	1979	Star Trek: The Motion Picture	Star Trek: The Motion Picture	Wise, Robert	Robert Wise	tt0079945
1746408714	1985	1985	The Sure Thing	The Sure Thing	Reiner, Rob	Rob Reiner	tt0090103
1746498294	1919	1919	The Outcasts of Poker Flats	The Outcasts of Poker Flat	Ford, Jack	John Ford	tt0010544
1746581254	1960	1960	Pretty Boy Floyd	Pretty Boy Floyd	Leder, Herbert J.	Herbert J. Leder	tt0054204
1746524100	1910	1910	Whist	Whist!		Tom Ricketts	tt1644737
1746576952	1961	1961	The Devil's Partner	Devil's Partner	Rondeau, Charles R.	Charles R. Rondeau	tt0051536
1746234377	1968	1968	Lonesome Cowboys	Lonesome Cowboys	Warhol, Andy	Andy Warhol	tt0063236
1746581370	1960	1961	The Marriage-Go-Round	The Marriage Go Round	Lang, Walter	Walter Lang	tt0054064
1746199017	1969	1968	Krakatoa, East of Java	Krakatoa: East of Java	Kowalski, Bernard L.	Bernard L. Kowalski	tt0064555
1746577069	1951	1951	Crazy Over Horses	Crazy Over Horses	Beaudine, William	William Beaudine	tt0043432
1746503920	1921	1921	The Beautiful Liar	The Beautiful Liar	Worsley, Wallace	Wallace Worsley	tt0011955
1746580803	1936	1936	The Girl from Mandalay	The Girl from Mandalay	Bretherton, Howard	Howard Bretherton	tt0027673
1746474444	1946	1946	Tarzan and the Leopard Woman	Tarzan and the Leopard Woman	Neumann, Kurt	Kurt Neumann	tt0039011
1746497023	1920	1920	Empty Arms	Empty Arms	Reicher, Frank	Frank Reicher	tt0011152
1746408468	1988	1988	The Unbearable Lightness of Being	The Unbearable Lightness of Being	Kaufman, Philip	Philip Kaufman	tt0096332
1746439141	1944	1944	The Curse of the Cat People	The Curse of the Cat People	Fritsch, Gunther V.; Wise, Robert	Gunther von Fritsch	tt0036733
1769970694	1968	1968	Office Love-In, White Collar Style	Office Love in, White Collar Style	Stephen, A. C.	Stephen C. Apostolof	tt0063378
1746584466	1929	1929	The Great Power	The Great Power	Rock, Joe	Joe Rock	tt0019948
1746453509	1941	1941	Tonto Basin Outlaws	Tonto Basin Outlaws	Luby, S. Roy	S. Roy Luby	tt0034300
1746507797	1926	1926	The Brown Derby	The Brown Derby	Hines, Charles	Charles Hines	tt0016689
1746198171	1969	1969	Bob & Carol & Ted & Alice	Bob & Carol & Ted & Alice	Mazursky, Paul	Paul Mazursky	tt0064100
1746424451	1943	1943	Holy Matrimony	Holy Matrimony	Stahl, John M.	John M. Stahl	tt0036009
1746581948	1928	1928	Red Riders of Canada	Red Riders of Canada	De Lacy, Robert	Robert De Lacey	tt0019317
1746560921	1932	1932	Fighting for Justice	Fighting for Justice	Brower, Otto	Otto Brower	tt0024013
1746437375	1941	1941	South of Panama	South of Panama	Yarbrough, Jean	Jean Yarbrough	tt0034221
1746573893	1957	1957	The Persuader	The Persuader	Ross, Dick	Dick Ross	tt0050838
1746408493	1984	1984	Reckless	Reckless	Foley, James	James Foley	tt0087983
1746532479	1923	1923	Potash and Perlmutter	Potash and Perlmutter	Badger, Clarence	Clarence G. Badger	tt0014370
1746394738	1985	1985	Krush Groove	Krush Groove	Schultz, Michael	Michael Schultz	tt0089444
1746504145	1920	1920	The Confession	The Confession	Bracken, Bertram	Bertram Bracken	tt0011069
1746585417	1939	1939	Homicide Bureau	Homicide Bureau	Coleman, C. C.	Charles C. Coleman	tt0031438
1746504543	1919	1919	Carolyn of the Corners	Carolyn of the Corners	Thornby, Robert	Robert Thornby	tt0009990
1746484455	1918	1918	The Girl of Today	The Girl of Today	Robertson, John	John S. Robertson	tt0009114
1746199520	1965	1965	Inside Daisy Clover	Inside Daisy Clover	Mulligan, Robert	Robert Mulligan	tt0059314
1746492427	1946	1946	Lawless Breed	Lawless Breed	Fox, Wallace W.	Wallace Fox	tt0175840
1746492080	1944	1944	Million Dollar Kid	Million Dollar Kid	Fox, Wallace	Wallace Fox	tt0037074
1746577472	1960	1960	Cash McCall	Cash McCall	Pevney, Joseph	Joseph Pevney	tt0052680
1746273341	1979	1979	Gas Pump Girls	Gas Pump Girls	Bender, Joel	Joel Bender	tt0077597
1746574294	1959	1959	The Scapegoat	The Scapegoat	Hamer, Robert	Robert Hamer	tt0053247
1746408829	1987	1987	Black Widow	Black Widow	Rafelson, Bob	Bob Rafelson	tt0090738
1746580452	1935	1935	Society Doctor	Society Doctor	Seitz, George B.	George B. Seitz	tt0027020
1746409433	1988	1988	The Telephone	The Telephone	Torn, Rip	Rip Torn	tt0096241
1746582428	1935	1935	The Glass Key	The Glass Key	Tuttle, Frank	Frank Tuttle	tt0026416
1746512373	1910	1910	The Luck of Roaring Camp	Luck of Roaring Camp	Porter, Edwin S.	Edwin S. Porter	tt0001297
1746573979	1962	1962	The Four Horsemen of the Apocalypse	The Four Horsemen of the Apocalypse	Minnelli, Vincente	Vincente Minnelli	tt0054890
1746454093	1940	1940	The Tulsa Kid	The Tulsa Kid	Sherman, George	George Sherman	tt0033189
1746503473	1918	1918	Amarilly of Clothes-Line Alley	Amarilly of Clothes Line Alley	Neilan, Marshall A.	Marshall Neilan	tt0008827
1746438937	1941	1941	Private Nurse	Private Nurse	Burton, David	David Burton	tt0034068
1746234856	1966	1966	Another Day, Another Man	Another Day, Another Man	Wishman, Doris	Doris Wishman	tt0060111
1746584960	1940	1940	Lucky Partners	Lucky Partners	Milestone, Lewis	Lewis Milestone	tt0032733
1746503676	1918	1918	Revelation	Revelation	Baker, George D.	George D. Baker	tt0009543

1746491830	1948	1948	Jinx Money	Jinx Money	Beaudine, William	William Beaudine	tt0040489
1746210442	1971	1971	Man in the Wilderness	Man in the Wilderness	Sarafian, Richard C.	Richard C. Sarafian	tt0067388
1769969915	1911	1911	The Cowboy and the Outlaw	The Cowboy and the Outlaw		Allan Dwan	tt1290058
1745358895	2008	2008	Saw V	Saw V	Hackl, David	David Hackl	tt1132626
1769970658	1968	1968	A Piece of Her Action	A Piece of Her Action	Denby, Jerry	Jerry Denby	tt0254689
1746497237	1919	1919	Faith	Faith	Swickard, Charles	Charles Swickard	tt0190390
1746454660	1945	1945	The Lost Trail	The Lost Trail	Hillyer, Lambert	Lambert Hillyer	tt0037883
1769970606	1968	1968	College Girls	College Girls	Stephen, A. C.	Stephen C. Apostolof	tt0123705
1746569996	1956	1956	Walk the Proud Land	Walk the Proud Land	Hibbs, Jesse	Jesse Hibbs	tt0049933
1746496424	1920	1920	The Dangerous Paradise	The Dangerous Paradise	Earle, William P. S.	William P.S. Earle	tt0190348
1746500594	1950	1950	Federal Agent at Large	Federal Agent at Large	Blair, George	George Blair	tt0042453
1746424453	1941	1941	Appointment for Love	Appointment for Love	Seiter, William A.	William A. Seiter	tt0033348
1746577832	1935	1935	No More Ladies	No More Ladies	Griffith, Edward H.	Edward H. Griffith	tt0026787
1746585423	1939	1939	Texas Wildcats	Texas Wildcats	Newfield, Sam	Sam Newfield	tt0032014
1746492475	1948	1948	Oklahoma Blues	Oklahoma Blues	Hillyer, Lambert	Lambert Hillyer	tt0040660
1746585311	1928	1928	The Woman Disputed	The Woman Disputed	King, Henry; Taylor, Sam	Henry King	tt0019589
1746577121	1950	1950	The Toast of New Orleans	The Toast of New Orleans	Taurog, Norman	Norman Taurog	tt0043053
1746464788	1941	1941	How Green Was My Valley	How Green Was My Valley	Ford, John	John Ford	tt0033729
1746503512	1918	1918	The Great Adventure	The Great Adventure	Blaché, Alice	Alice Guy	tt0009128
1746561868	1954	1954	The Last Time I Saw Paris	The Last Time I Saw Paris	Brooks, Richard	Richard Brooks	tt0047162
1746520525	1926	1926	Watch Your Wife	Watch Your Wife	Gade, Svend	Svend Gade	tt0017530
1746569949	1954	1954	Tobor the Great	Tobor the Great	Sholem, Lee	Lee Sholem	tt0047590
1746520647	1948	1948	Train to Alcatraz	Train to Alcatraz	Ford, Philip	Philip Ford	tt0040895
1746573848	1958	1958	Chase a Crooked Shadow	Chase a Crooked Shadow	Anderson, Michael	Michael Anderson	tt0050246
1746500652	1926	1926	Crossed Signals	Crossed Signals	McGowan, J. P.	J.P. McGowan	tt0016749
1746437367	1941	1941	The Flame of New Orleans	The Flame of New Orleans	Clair, René	René Clair	tt0033606
1746577967	1937	1937	Fifty Roads to Town	Fifty Roads to Town	Taurog, Norman	Norman Taurog	tt0028866
1745358521	2002	2001	Black Hawk Down	Black Hawk Down	Scott, Ridley	Ridley Scott	tt0265086
1746512646	1913	1913	Ben Bolt	Ben Bolt	Hansel, Howell	Howell Hansel	tt0002678
1746422592	1994	1993	Fear of a Black Hat	Fear of a Black Hat	Cundieff, Rusty	Rusty Cundieff	tt0106880
1746269472	1980	1980	Wholly Moses!	Wholly Moses!	Weis, Gary	Gary Weis	tt0081751
1745309629	2006	2006	Half Nelson	Half Nelson	Fleck, Ryan	Ryan Fleck	tt0468489
1746584972	1939	1939	On Dress Parade	On Dress Parade	Clemens, William	William Clemens	tt0031012
1746409775	1991	1991	Soapdish	Soapdish	Hoffman, Michael	Michael Hoffman	tt0102951
1746562031	1955	1955	A Bullet for Joey	A Bullet for Joey	Allen, Lewis	Lewis Allen	tt0047904
1746513151	1917	1917	Glory	Glory	Berri, Maud Lillian	Francis J. Grandon	tt0008015
1746476209	1947	1947	Fun on a Weekend	'Fun on a Week End'	Stone, Andrew	Andrew L. Stone	tt0039405
1746465235	1947	1947	Rose of Santa Rosa	Rose of Santa Rosa	Nazarro, Ray	Ray Nazarro	tt0039786
1746532491	1916	1916	The Code of Marcia Gray	The Code of Marcia Gray	Lloyd, Frank	Frank Lloyd	tt0006523
1746475909	1944	1944	Harmony Trail	Harmony Trail	Emmett, Robert	Robert Emmett Tansey	tt0039988
1746562023	1953	1953	The Desert Rats	The Desert Rats	Wise, Robert	Robert Wise	tt0045679
1746408895	1987	1987	House of Games	House of Games	Mamet, David	David Mamet	tt0093223
1746581781	1910	1910	The Gold-Seekers	The Gold Seekers	Griffith, D. W.	D.W. Griffith	tt0001236
1746435258	1943	1943	It's a Great Life	It's a Great Life	Strayer, Frank	Frank R. Strayer	tt0036049
1746580874	1936	1936	The Unguarded Hour	The Unguarded Hour	Wood, Sam	Sam Wood	tt0028447
1746455685	1997	1997	Anaconda	Anaconda	Llosa, Luis	Luis Llosa	tt0118615
1746491974	1948	1948	Adventures in Silverado	Adventures in Silverado	Karlson, Phil	Phil Karlson	tt0040074
1746577198	1959	1959	Ghost of Dragstrip Hollow	Ghost of Dragstrip Hollow	Hole, William	William J. Hole Jr.	tt0052844
1746583148	1938	1938	Algiers	Algiers	Cromwell, John	John Cromwell	tt0029855
1746473684	1947	1947	Life with Father	Life with Father	Curtiz, Michael	Michael Curtiz	tt0039566
1746514616	1923	1923	$1,000 Reward	$1,000 Reward	Seeling, Charles R.	Charles R. Seeling	tt0013811
1745342007	2010	2010	The Twilight Saga: Eclipse	The Twilight Saga: Eclipse	Slade, David	David Slade	tt1325004
1746582399	1929	1929	Square Shoulders	Square Shoulders	Hopper, E. Mason	E. Mason Hopper	tt0126075
1746562154	1953	1953	Flame of Calcutta	Flame of Calcutta	Friedman, Seymour	Seymour Friedman	tt0045771
1746585118	1928	1928	The Grip of the Yukon	The Grip of the Yukon	Laemmle, Ernst	Ernst Laemmle	tt0018960
1746409441	1988	1988	A Fish Called Wanda	A Fish Called Wanda	Crichton, Charles	Charles Crichton	tt0095159
1746561935	1954	1954	His Majesty O'Keefe	His Majesty O'Keefe	Haskin, Byron	Byron Haskin	tt0045876

1746580642	1936	1936	Flying Hostess	Flying Hostess	Roth, Murray	Murray Roth	tt0027628	
1746570073	1934	1934	Moulin Rouge	Moulin Rouge	Lanfield, Sidney	Sidney Lanfield	tt0025520	
1746577523	1936	1936	Gold Diggers of 1937	Gold Diggers of 1937	Bacon, Lloyd	Lloyd Bacon	tt0027686	
1746514697	1923	1923	Money Money Money	Money! Money! Money!	Forman, Tom	Tom Forman	tt0138625	
1746580313	1937	1937	Law for Tombstone	Law for Tombstone	Jones, Charles "Buck"	W.B. Eason	tt0029123	
1746581899	1928	1928	Say It With Sables	Say It with Sables	Capra, Frank	Frank Capra	tt0019355	
1746454848	1941	1941	The Case of the Black Parrot	The Case of the Black Parrot	Smith, Noel M.	Noel M. Smith	tt0033452	
1746580567	1935	1935	Dizzy Dames	Dizzy Dames	Nigh, William	William Nigh	tt0026282	
1746394146	1982	1982	Honkytonk Man	Honkytonk Man	Eastwood, Clint	Clint Eastwood	tt0084088	
1769970644	1968	1968	A Sweet Sickness	A Sweet Sickness	Martin, Jon	Jon Martin	tt0128666	
1746235663	1981	1980	Gloria	Gloria	Cassavetes, John	John Cassavetes	tt0080798	
1746500613	1949	1949	A Kiss for Corliss	A Kiss for Corliss	Wallace, Richard	Richard Wallace	tt0041549	
1746585014	1940	1940	One Night in the Tropics	One Night in the Tropics	Sutherland, A. Edward	A. Edward Sutherland	tt0032873	
1746508101	1926	1926	The Passaic Textile Strike	The Passaic Textile Strike		Samuel Russak	tt0455674	
1769969439	1964	1963	The Fat Black Pussycat	The Fat Black Pussycat	Lea, Harold	Harold Lea	tt0058090	
1746585150	1929	1929	Smoke Bellew	Smoke Bellew	Dunlap, Scott	Scott R. Dunlap	tt0020423	
1746584278	1935	1935	The Rider of the Law	The Rider of the Law	Bradbury, Robert N.	Robert N. Bradbury	tt0026929	
1746569856	1957	1957	Slander	Slander	Rowland, Roy	Roy Rowland	tt0049767	
1746520958	1949	1949	She Wore a Yellow Ribbon	She Wore a Yellow Ribbon	Ford, John	John Ford	tt0041866	
1746423996	1941	1941	Too Many Blondes	Too Many Blondes	Freeland, Thornton	Thornton Freeland	tt0034301	
1745357906	2004	2004	Fat Albert	Fat Albert	Zwick, Joel	Joel Zwick	tt0396592	
1746409077	1985	1984	Kaddish	Kaddish	Brand, Steve	Steve Brand	tt0162419	
1746420741	1983	1983	Without a Trace	Without a Trace	Jaffe, Stanley R.	Stanley R. Jaffe	tt0086593	
1746484699	1917	1917	Nearly Married	Nearly Married	Withey, Chester	Chester Withey	tt0008365	
1746570011	1961	1961	Sniper's Ridge	Sniper's Ridge	Bushelman, John	John A. Bushelman	tt0055457	
1746422914	1991	1991	L. A. Story	L.A. Story	Jackson, Mick	Mick Jackson	tt0102250	
1745294020	2007	2007	P.S. I Love You	P.S. I Love You	LaGravenese, Richard	Richard LaGravenese	tt0431308	
1746568222	1953	1953	Peek-A-Boo	Peek a Boo	Hunt, Lillian	Lillian Hunt	tt0174109	
1746438182	1940	1940	Sandy Is a Lady	Sandy Is a Lady	Lamont, Charles	Charles Lamont	tt0033019	
1746199485	1968	1968	Did You Hear the One About the Traveling Salesla	Did You Hear the One About the Traveling Sa	Weis, Don	Don Weis	tt0062890	
1746577616	1935	1935	Let 'Em Have It	Let 'em Have It	Wood, Sam	Sam Wood	tt0026621	
1746556045	1955	1955	Top Gun	Top Gun	Nazarro, Ray	Ray Nazarro	tt0048734	
1746501120	1922	1922	The Devil's Ghost	The Devil's Ghost		Charles W. Mack	tt0356531	
1746218393	1966	1966	Jesse James Meets Frankenstein's Daughter	Jesse James Meets Frankenstein's Daughter	Beaudine, William	William Beaudine	tt0060558	
1746570071	1933	1933	The Fatal Glass of Beer	The Fatal Glass of Beer	Bruckman, Clyde	Clyde Bruckman	tt0022883	
1746520853	1948	1948	The Three Musketeers	The Three Musketeers	Sidney, George	George Sidney	tt0040876	
1746409478	1991	1991	Fried Green Tomatoes	Fried Green Tomatoes	Avnet, Jon	Jon Avnet	tt0101921	
1746578767	1962	1962	It Happened in Athens	It Happened in Athens	Marton, Andrew	Andrew Marton	tt0056109	
1746420521	1991	1991	Class Action	Class Action	Apted, Michael	Michael Apted	tt0101590	
1746520681	1950	1950	Convicted	Convicted	Levin, Henry	Henry Levin	tt0042343	
1746218878	1972	1972	Jeremiah Johnson	Jeremiah Johnson	Pollack, Sydney	Sydney Pollack	tt0068762	
1746465125	1947	1947	Undercover Maisie	Undercover Maisie	Beaumont, Harry	Harry Beaumont	tt0039935	
1746455185	2000	2000	Bittersweet Motel	Bittersweet Motel	Phillips, Todd	Todd Phillips	tt0168515	
1746566832	1933	1933	Racetrack	Racetrack	Cruze, James	James Cruze	tt0024482	
1745372469	2010	2010	Prince of Persia: The Sands of Time	Prince of Persia: The Sands of Time	Newell, Mike	Mike Newell	tt0473075	
1746583124	1935	1935	Sweet Surrender	Sweet Surrender	Brice, Monte	Monte Brice	tt0027065	
1746423026	1998	1997	Courting Courtney	Courting Courtney	Tarantino, Paul	Paul Tarantino	tt0118893	
1746585044	1939	1939	Wife, Husband and Friend	Wife, Husband and Friend	Ratoff, Gregory	Gregory Ratoff	tt0032128	
1746574988	1937	1937	Santa Fe Rides	Santa Fe Rides	Shamray, Franklin	Bernard B. Ray	tt0232581	
1746524543	1922	1922	Grand Larceny	Grand Larceny	Worsley, Wallace	Wallace Worsley	tt0013200	
1746454708	1999	1999	Get Bruce!	Get Bruce	Kuehn, Andrew J.	Andrew J. Kuehn	tt0184510	
1746520426	1924	1924	The Border Legion	The Border Legion	Howard, William K.	William K. Howard	tt0014728	
1746582168	1931	1931	Dancing Dynamite	Dancing Dynamite	Mason, Noel	Noel M. Smith	tt0021779	
1746453630	1943	1943	Here Comes Elmer	Here Comes Elmer	Santley, Joseph	Joseph Santley	tt0035987	
1746261188	1979	1979	A Perfect Couple	A Perfect Couple	Altman, Robert	Robert Altman	tt0079710	
**1746454360**	**1941**	**1941**	**King of Dodge City**	**King of Dodge City**	**Hillyer, Lambert**	**Lambert Hillyer**	**tt0033785**	1
1746576829	1910	1910	For the Love of Red Wing	For the Love of Red Wing		Fred J. Balshofer	tt0359288	

1746562377	1953	1953	The Naked Spur	The Naked Spur	Mann, Anthony	Anthony Mann	tt0044953
1769971108	1969	1969	All the Loving Couples	All the Loving Couples	Bing, Mack	Mack Bing	tt0064003
1746514932	1928	1928	Marked Money	Marked Money	Bennett, Spencer Gordon	Spencer Gordon Bennet	tt0019144
1746501093	1915	1915	The Bludgeon	The Bludgeon	Cullison, Webster	Webster Cullison	tt0004982
1746421274	1994	1994	The Air Up There	The Air Up There	Glaser, Paul M.	Paul Michael Glaser	tt0109067
1746508092	1924	1924	Wine of Youth	Wine of Youth	Vidor, King	King Vidor	tt0015500
1746584572	1931	1931	Women Men Marry	Women Men Marry	Hutchison, Charles	Charles Hutchison	tt0022575
1746475525	1946	1946	Centennial Summer	Centennial Summer	Preminger, Otto	Otto Preminger	tt0038406
1746184321	1965	1964	Quick, Before It Melts	Quick Before It Melts	Mann, Delbert	Delbert Mann	tt0058506
1746487576	1918	1918	Set Free	Set Free	Browning, Tod	Tod Browning	tt0010682
1746500829	1910	1910	May and December	May and December	Powell, Frank	Frank Powell	tt0001310
1746198019	1966	1966	Cast a Giant Shadow	Cast a Giant Shadow	Shavelson, Melville	Melville Shavelson	tt0060218
1746580501	1938	1938	Five of a Kind	Five of a Kind	Leeds, Herbert I.	Herbert I. Leeds	tt0030136
1746219609	1974	1974	The Super Cops	The Super Cops	Parks, Gordon	Gordon Parks	tt0072228
1746491896	1946	1946	Gilda	Gilda	Vidor, Charles	Charles Vidor	tt0038559
1746211094	1973	1973	Emperor of the North	Emperor of the North	Aldrich, Robert	Robert Aldrich	tt0070030
1746454058	1944	1944	The Doughgirls	The Doughgirls	Kern, James V.	James V. Kern	tt0036776
1746533046	1923	1923	Under the Red Robe	Under the Red Robe	Crosland, Alan	Alan Crosland	tt0014568
1746408918	1985	1985	Cocoon	Cocoon	Howard, Ron	Ron Howard	tt0088933
1746580784	1939	1939	Balalaika	Balalaika	Schunzel, Reinhold	Reinhold Schünzel	tt0031074
1746508506	1926	1926	Faithful Wives	Faithful Wives	Myles, Norbert	Norbert A. Myles	tt0016837
1746583075	1935	1935	Thunder Mountain	Thunder Mountain	Howard, David	David Howard	tt0027104
1746513082	1923	1923	The Dangerous Age	The Dangerous Age	Stahl, John M.	John M. Stahl	tt0013969
1746486507	1918	1918	In Bad	In Bad	Sloman, Edward	Edward Sloman	tt0012602
1746409507	1990	1990	Marked for Death	Marked for Death	Little, Dwight H.	Dwight H. Little	tt0100114
1746520870	1925	1925	Ridin' Thunder	Ridin' Thunder	Smith, Clifford S.	Clifford Smith	tt0016291
1746435849	1999	1999	Joe the King	Joe the King	Whaley, Frank	Frank Whaley	tt0160672
1746573393	1932	1932	Evenings for Sale	Evenings for Sale	Walker, Stuart	Stuart Walker	tt0022863
1769976712	1929	1929	Mexicana	Mexicana	Edwards, Gus	Gus Edwards	tt0370959
1746491792	1948	1948	The Denver Kid	The Denver Kid	Ford, Philip	Philip Ford	tt0040282
1746409440	1981	1981	So Fine	So Fine	Bergman, Andrew	Andrew Bergman	tt0083099
1746577017	1951	1951	Sealed Cargo	Sealed Cargo	Werker, Alfred	Alfred L. Werker	tt0044012
1745342928	2002	2002	Pumpkin	Pumpkin	Broder, Adam Larson; Abrams, Tony	Anthony Abrams	tt0265591
1769971896	1970	1970	The Dark Side of Tomorrow	The Dark Side of Tomorrow	Peeters, Barbara; Deerson, Jacque	Jack Deerson	tt0070257
1746216698	1967	1967	The War Wagon	The War Wagon	Kennedy, Burt	Burt Kennedy	tt0062472
1746584847	1928	1928	Headin' for Danger	Headin' for Danger	Bradbury, Robert North	Robert N. Bradbury	tt0018983
1746583764	1931	1931	Good Sport	Good Sport	MacKenna, Kenneth	Kenneth MacKenna	tt0021922
1746580264	1936	1936	Florida Special	Florida Special	Murphy, Ralph	Ralph Murphy	tt0027627
1746464850	1942	1942	Lure of the Islands	Lure of the Islands	Yarbrough, Jean	Jean Yarbrough	tt0035002
1745303055	2007	2007	Who's Your Caddy?	Who's Your Caddy?	Paul, Don Michael	Don Michael Paul	tt0785077
1746508375	1926	1926	Fighting Ranger	The Fighting Ranger	Hurst, Paul	Paul Hurst	tt0016866
1746498293	1919	1919	The Little Diplomat	The Little Diplomat	Paton, Stuart	Stuart Paton	tt0010369
1746514836	1915	1915	Evidence	Evidence	August, Edwin	Edwin August	tt0005256
1746570288	1934	1934	Handy Andy	Handy Andy	Butler, David	David Butler	tt0025217
1746484702	1918	1918	The Sins of the Children	The Sins of the Children	Lopez, John S.	John S. Lopez	tt0187510
1746582255	1950	1950	Winchester '73	Winchester '73	Mann, Anthony	Anthony Mann	tt0043137
1746483901	1917	1917	Little Miss Fortune	Little Miss Fortune	Levering, Joseph	Joseph Levering	tt0176939
1746409251	1985	1985	Creature	Creature	Malone, William	William Malone	tt0088961
1746198497	1969	1969	Fly Now, Pay Later	Fly Now, Pay Later	Dial, B. H.	B.H. Dial	tt0137536
1746250738	1979	1979	...And Justice for All	...and justice for all.	Jewison, Norman	Norman Jewison	tt0078718
1746581682	1928	1928	The Rustler's End	The Rustler's End	Horner, Robert J.	Robert J. Horner	tt0159712
1746437415	1940	1940	Slightly Tempted	Slightly Tempted	Landers, Lew	Lew Landers	tt0033063
1746575013	1937	1937	The Trusted Outlaw	The Trusted Outlaw	Bradbury, Robert N.	Robert N. Bradbury	tt0029696
1746570004	1958	1958	The Defiant Ones	The Defiant Ones	Kramer, Stanley	Stanley Kramer	tt0051525
1746410384	1980	1980	The Private Eyes	The Private Eyes	Elliott, Lang	Lang Elliott	tt0081376
1746422240	1998	1998	A Simple Plan	A Simple Plan	Raimi, Sam	Sam Raimi	tt0120324
1746584805	1930	1930	Undertow	Undertow	Pollard, Harry	Harry A. Pollard	tt0021504

1746455201	1998	1998	The Cruise	The Cruise	Miller, Bennett	Bennett Miller	tt0150230
1746524371	1910	1910	The Roman	The Roman	Boggs, Frank	Francis Boggs	tt0000751
1746566186	1956	1956	Storm Center	Storm Center	Taradash, Daniel	Daniel Taradash	tt0049800
1746423690	1940	1940	Pony Post	Pony Post	Taylor, Ray	Ray Taylor	tt0032923
1746582775	1938	1938	Hunted Men	Hunted Men	King, Louis	Louis King	tt0030251
1746523856	1916	1916	The Stolen Triumph	The Stolen Triumph	Thompson, David	David Thompson	tt0158993
1746553868	1963	1963	Cry of Battle	Cry of Battle	Lerner, Irving	Irving Lerner	tt0056967
1746508226	1924	1924	Payable on Demand	Payable on Demand	Maloney, Leo	Leo D. Maloney	tt0147156
1746453742	1996	1996	Fear	Fear	Foley, James	James Foley	tt0116287
1745372527	2002	2002	Changing Lanes	Changing Lanes	Michell, Roger	Roger Michell	tt0264472
1745293931	2007	2007	1408	1408	Hafstrom, Mikael	Mikael Håfström	tt0450385
1746233012	1969	1969	The Hanging of Jake Ellis	The Hanging of Jake Ellis	Van Hearn, J.	Jean Van Hearn	tt0218322
1746509255	1949	1949	The Gal Who Took the West	The Gal Who Took the West	De Cordova, Frederick	Frederick De Cordova	tt0041400
1746565290	1932	1932	Racing Youth	Racing Youth	Moore, Vin	Vin Moore	tt0024483
1746436354	1941	1941	The Texas Marshal	The Texas Marshal	Stewart, Peter	Sam Newfield	tt0034270
1746497646	1917	1917	The Lady in the Library	The Lady in the Library	Jones, Edgar	Edgar Jones	tt0174848
1746584242	1939	1939	Riders of the Frontier	Riders of the Frontier	Bennett, Spencer Gordon	Spencer Gordon Bennet	tt0031860
1746218987	1972	1972	Play It As It Lays	Play It As It Lays	Perry, Frank	Frank Perry	tt0069098
1746566109	1956	1956	Invasion of the Body Snatchers	Invasion of the Body Snatchers	Siegel, Don	Don Siegel	tt0049366
1746408913	1989	1989	The Terror Within	The Terror Within	Notz, Thierry	Thierry Notz	tt0096246
1746483510	1917	1917	The Checkmate	Checkmate	McDonald, Sherwood	Edward LeSaint	tt4830120
1746577467	1951	1951	My Favorite Spy	My Favorite Spy	McLeod, Norman Z.	Norman Z. McLeod	tt0043827
1746582190	1950	1949	A French Peep Show	The French Peep Show	Alexander, William	Russ Meyer	tt0347025
1746575366	1910	1910	The Flight of Red Wing	The Flight of Red Wing		Fred J. Balshofer	tt0359282
1746578866	1963	1963	Dime With a Halo	Dime with a Halo	Sagal, Boris	Boris Sagal	tt0056999
1746424006	1942	1942	Sing Your Worries Away	Sing Your Worries Away	Sutherland, A. Edward	A. Edward Sutherland	tt0035338
1746584751	1928	1927	The Gaucho	The Gaucho	Jones, F. Richard	F. Richard Jones	tt0017918
1746199026	1966	1966	Texas Across the River	Texas Across the River	Gordon, Michael	Michael Gordon	tt0061080
1746584361	1937	1937	One Man Justice	One Man Justice	Barsha, Leon	Leon Barsha	tt0029348
1746574356	1960	1960	The Adventures of Huckleberry Finn	The Adventures of Huckleberry Finn	Curtiz, Michael	Michael Curtiz	tt0053571
1769970494	1967	1967	Strange Rampage	Strange Rampage	Volpe, Ignatius	Harry Kerwin	tt0062308
1746408512	1986	1986	Vamp	Vamp	Wenk, Richard	Richard Wenk	tt0092147
1746474817	1946	1946	The Verdict	The Verdict	Siegel, Don	Don Siegel	tt0039080
1746523950	1916	1916	The Devil-in-Chief	The Devil in Chief	Campbell, Colin	Colin Campbell	tt0441199
1746583751	1929	1929	The Godless Girl	The Godless Girl	DeMille, Cecil B.	Cecil B. DeMille	tt0019935
1746585181	1928	1928	A Woman Against the World	A Woman Against the World	Archainbaud, George	George Archainbaud	tt0019588
1746502808	1917	1917	The Phantom Shotgun	The Phantom Shotgun	Harvey, Harry	Harry Harvey	tt0008428
1746566900	1932	1932	The Big Timer	The Big Timer	Buzzell, Eddie	Edward Buzzell	tt0022682
1746532998	1927	1927	The Girl From Gay Paree	The Girl from Gay Paree	Stone, Phil	Phil Goldstone	tt0017933
1746465226	1946	1946	High School Hero	High School Hero	Dreifuss, Arthur	Arthur Dreifuss	tt0038601
1746409039	1982	1982	Annie	Annie	Huston, John	John Huston	tt0083564
1746580722	1936	1936	Man Hunt	Man Hunt	Clemens, William	William Clemens	tt0027936
1746503015	1915	1915	Bondwomen	The Bondwoman	August, Edwin	Kenean Buel	tt1148237
1746584854	1930	1930	Officer O'Brien	Officer O'Brien	Garnett, Tay	Tay Garnett	tt0021198
1746567951	1934	1934	The World Moves On	The World Moves On	Ford, John	John Ford	tt0026008
1746487357	1919	1919	The Petal on the Current	A Petal on the Current	Browning, Tod	Tod Browning	tt0010561
1746580477	1935	1934	The Tonto Kid	The Tonto Kid	Fraser, Harry	Harry L. Fraser	tt0131072
1746569935	1957	1957	Raiders of Old California	Raiders of Old California	Gannaway, Albert C.	Albert C. Gannaway	tt0050879
1746410405	1989	1989	Heavy Petting	Heavy Petting	Benz, Obie	Obie Benz	tt0095289
1746555749	1957	1957	A Face in the Crowd	A Face in the Crowd	Kazan, Elia	Elia Kazan	tt0050371
1746474512	1944	1944	Tunisian Victory	Tunisian Victory	Stewart, Hugh; Capra, Frank	Frank Capra	tt0037404
1746475899	1945	1945	The Crimson Canary	The Crimson Canary	Hoffman, John	John Hoffman	tt0037623
1746563645	1951	1951	Yes Sir, Mr. Bones	Yes Sir, Mr. Bones	Ormond, Ron	Ron Ormond	tt0044222
1746520698	1925	1925	Ridin' Wild	Ridin' Wild	De La Mothe, Leon	Leon De La Mothe	tt0156959
1746554678	1964	1964	Ride the Wild Surf	Ride the Wild Surf	Taylor, Don	Don Taylor	tt0058523
1746491761	1944	1944	Stars on Parade	Stars on Parade	Landers, Lew	Lew Landers	tt0037311
1746580425	1939	1939	Fighting Thoroughbreds	Fighting Thoroughbreds	Salkow, Sidney	Sidney Salkow	tt0031307

1746473745	1946	1946	Easy to Wed	Easy to Wed	Buzzell, Edward	Edward Buzzell	tt0038505	
1746509288	1925	1925	Who's Your Friend	Who's Your Friend	Sheldon, Forrest K.	Forrest Sheldon	tt0179525	
1746515288	1917	1917	Blind Man's Holiday	Blind Man's Holiday	Justice, Martin	Martin Justice	tt0168520	
1746487040	1918	1918	Playthings	Playthings	Gerrard, Douglas	Douglas Gerrard	tt0009503	
1746577979	1936	1936	Three Married Men	Three Married Men	Buzzell, Edward	Edward Buzzell	tt0028369	
1746578848	1959	1959	Tarzan's Greatest Adventure	Tarzan's Greatest Adventure	Guillermin, John	John Guillermin	tt0053334	
1745293205	2006	2006	Running Scared	Running Scared	Kramer, Wayne	Wayne Kramer	tt0404390	
1746577400	1950	1950	Federal Man	Federal Man	Tansey, Robert	Robert Emmett Tansey	tt0176712	
1746486523	1918	1918	The Girl in the Dark	The Girl in the Dark	Paton, Stuart	Stuart Paton	tt0009113	
1746574360	1959	1959	The Killer Shrews	The Killer Shrews	Kellogg, Ray	Ray Kellogg	tt0052969	
1746500410	1950	1950	Joe Palooka in the Squared Circle	Joe Palooka in The Squared Circle	Le Borg, Reginald	Reginald Le Borg	tt0042617	
1746583958	1931	1931	The Fighting Marshal	The Fighting Marshal	Lederman, D. Ross	D. Ross Lederman	tt0022886	
1746566002	1955	1955	A Man Called Peter	A Man Called Peter	Koster, Henry	Henry Koster	tt0048337	
1746409087	1986	1986	Extremities	Extremities	Young, Robert M.	Robert M. Young	tt0091024	
1746183898	1910	1910	The Champion of the Race	The Champion of the Race		Theodore Wharton	tt1493240	
1746438968	1942	1942	Foreign Agent	Foreign Agent	Beaudine, William	William Beaudine	tt0034748	
1746574577	1937	1937	Double Wedding	Double Wedding	Thorpe, Richard	Richard Thorpe	tt0028804	
1746465366	1943	1943	You're a Lucky Fellow, Mr. Smith	You're a Lucky Fellow, Mr. Smith	Feist, Felix E.	Felix E. Feist	tt0036551	
1746582228	1951	1951	Roaring City	Roaring City	Berke, William	William Berke	tt0043975	
1746572320	1932	1932	Lovers Courageous	Lovers Courageous	Leonard, Robert Z.	Robert Z. Leonard	tt0023162	
1746500500	1950	1950	Edge of Doom	Edge of Doom	Robson, Mark	Mark Robson	tt0042428	
1746437005	1943	1943	Calling Wild Bill Elliott	Calling Wild Bill Elliott	Bennet, Spencer	Spencer Gordon Bennet	tt0035707	
1746485544	1918	1918	Madame Spy	Madame Spy	Gerrard, Douglas	Douglas Gerrard	tt0009331	
1746516536	1921	1921	The White Masks	The White Masks	Holt, George	George Holt	tt0012841	
1745272261	2000	2000	Nurse Betty	Nurse Betty	LaBute, Neil	Neil LaBute	tt0171580	
1746514848	1922	1922	The Bonded Woman	The Bonded Woman	Rosen, Philip E.	Phil Rosen	tt0012960	
1746585287	1928	1928	The Circus Kid	The Circus Kid	Seitz, George B.	George B. Seitz	tt0018771	
1746496317	1921	1921	The Love Charm	The Love Charm	Heffron, Thomas N.	Thomas N. Heffron	tt0012407	
1746512870	1917	1917	The Greatest Power	The Greatest Power	Carewe, Edwin	Edwin Carewe	tt0008033	
1746584701	1928	1928	Dugan of the Dugouts	Dugan of the Dugouts	Ray, Robert	Bobby Ray	tt0130642	
1746569823	1958	1958	The Last of the Fast Guns	The Last of the Fast Guns	Sherman, George	George Sherman	tt0051846	
1746435362	1999	1999	American Pie	American Pie	Weitz, Paul	Paul Weitz	tt0163651	
1746420387	1993	1993	Sniper	Sniper	Llosa, Luis	Luis Llosa	tt0108171	
1746570418	1952	1952	The Miracle of Our Lady of Fatima	The Miracle of Our Lady of Fatima	Brahm, John	John Brahm	tt0044905	
1746509233	1924	1924	Western Yesterdays	Western Yesterdays	Ford, Francis	Francis Ford	tt0177391	
1746475024	1943	1943	Black Market Rustlers	Black Market Rustlers	Luby, S. Roy	S. Roy Luby	tt0035678	
1746504093	1918	1918	Loaded Dice	Loaded Dice	Blaché, Herbert	Herbert Blaché	tt0009312	
1746524377	1915	1915	Matrimony	Matrimony	Sidney, Scott	Scott Sidney	tt0005730	
1745282892	2006	2006	Clerks II	Clerks II	Smith, Kevin	Kevin Smith	tt0424345	
1746503774	1919	1919	The Girl from Nowhere	Girl from Nowhere	Lucas, Wilfred	Wilfred Lucas	tt0010168	
1746499888	1946	1946	Alias Billy the Kid	Alias Billy the Kid	Carr, Thomas	Thomas Carr	tt0038294	
1745359145	2002	2001	Revolution OS	Revolution OS	Moore, J. T. S.	J.T.S. Moore	tt0308808	
1746520768	1950	1950	Destination Big House	Destination Big House	Blair, George	George Blair	tt0042392	
1746566647	1952	1952	Tropical Heat Wave	Tropical Heat Wave	Springsteen, R. G.	R.G. Springsteen	tt0045263	
1745342628	2003	2002	The Weather Underground	The Weather Underground	Green, Sam ; Siegel, Bill	Sam Green	tt0343168	
1746503551	1919	1919	The Bondage of Barbara	The Bondage of Barbara	Flynn, Emmett J.	Emmett J. Flynn	tt0009951	
**1746520813**	**1925**	**1925**	**The Pony Express**	**The Pony Express**	**Cruze, James**	**James Cruze**	**tt0016235**	1
1746474117	1946	1946	Black Angel	Black Angel	Neill, Roy William	Roy William Neill	tt0038360	
1746555077	1954	1954	Elephant Walk	Elephant Walk	Dieterle, William	William Dieterle	tt0046951	
1746580384	1938	1938	Partners of the Plains	Partners of the Plains	Selander, Lesley	Lesley Selander	tt0030558	
1769970551	1967	1967	Mr. Mari's Girls	Mr. Mari's Girls	Hennigar, William K.	William K. Hennigar	tt0126466	
1746424398	1940	1940	East of the River	East of the River	Green, Alfred E.	Alfred E. Green	tt0032429	
1746453462	1942	1942	Ghost Town Law	Ghost Town Law	Bretherton, Howard	Howard Bretherton	tt0034785	
1746561487	1932	1932	Lady and Gent	Lady and Gent	Roberts, Stephen	Stephen Roberts	tt0023109	
1746520649	1949	1949	Red Light	Red Light	Del Ruth, Roy	Roy Del Ruth	tt0041790	
1746524943	1928	1928	Love Over Night	Love Over Night	Griffith, Edward H.	Edward H. Griffith	tt0019114	
1746453518	1944	1944	Fuzzy Settles Down	Fuzzy Settles Down	Newfield, Sam	Sam Newfield	tt0036844	

1746474410	1947	1947	The Chinese Ring	The Chinese Ring	Beaudine, William	William Beaudine	tt0039262	
1746580257	1934	1934	Men of the Night	Men of the Night	Hillyer, Lambert	Lambert Hillyer	tt0025490	
1745293601	2006	2006	Cowboy Junction	Cowboy Junction	Christian, Gregory	Gregory Christian	tt0816145	
1746502928	1914	1914	The Mountain Rat	The Mountain Rat	Kirkwood, James	James Kirkwood	tt0004359	
1746525165	1920	1920	Married Life	Married Life	Sennett, Mack	Erle C. Kenton	tt0011444	
1746500186	1924	1924	Captain January	Captain January	Cline, Edward F.	Edward F. Cline	tt0014760	
1745272114	2000	2000	Supernova	Supernova	Lee, Thomas	Walter Hill	tt0134983	
1746532501	1923	1923	Shadows of the North	Shadows of the North	Hill, Robert F.	Robert F. Hill	tt0014461	
1746578429	1963	1963	House of the Damned	House of the Damned	Dexter, Maury	Maury Dexter	tt0057160	
1746454818	1942	1942	Apache Trail	Apache Trail	Thorpe, Richard; Rosson, Richard	Richard Thorpe	tt0035639	
1746580614	1935	1935	Village Tale	Village Tale	Cromwell, John	John Cromwell	tt0027176	
1746514853	1916	1916	Betty of Graystone	Betty of Greystone	Dwan, Allan	Allan Dwan	tt0006426	
1746530736	1949	1949	Black Midnight	Black Midnight	Boetticher, Oscar	Budd Boetticher	tt0041183	
1746562347	1957	1957	The Tijuana Story	The Tijuana Story	Kardos, Leslie	László Kardos	tt0051081	
1746416230	1917	1917	The Jury of Fate	The Jury of Fate	Browning, Tod	Tod Browning	tt0008155	
1746554608	1964	1964	The Secret Door	The Secret Door	Kay, Gilbert L.	Gilbert Kay	tt0056459	
1746585070	1930	1930	Oh, For a Man	Oh, for a Man!	MacFadden, Hamilton	Hamilton MacFadden	tt0021199	
1746492461	1946	1946	Sister Kenny	Sister Kenny	Nichols, Dudley	Dudley Nichols	tt0038948	
1746583954	1930	1930	Today	Today	Nigh, William	William Nigh	tt0021480	
1746573795	1956	1956	You Can't Run Away from It	You Can't Run Away from It	Powell, Dick	Dick Powell	tt0049973	
1769969925	1911	1911	Only an Iceman	Only an Iceman			tt1303886	
1746438163	1997	1997	For Richer or Poorer	For Richer or Poorer	Spicer, Bryan	Bryan Spicer	tt0119142	
1746520817	1948	1948	Trapped by Boston Blackie	Trapped by Boston Blackie	Friedman, Seymour	Seymour Friedman	tt0040896	
1746498487	1918	1918	The Eagle	The Eagle	Clifton, Elmer	Elmer Clifton	tt0009030	
1746507875	1926	1926	Millionaires	Millionaires	Raymaker, Herman C.	Herman C. Raymaker	tt0017154	
1746578633	1950	1950	One Way Street	One Way Street	Fregonese, Hugo	Hugo Fregonese	tt0042809	
1746584201	1936	1936	Banjo on My Knee	Banjo on My Knee	Cromwell, John	John Cromwell	tt0027331	
1746584435	1931	1931	The Star Witness	The Star Witness	Wellman, William A.	William A. Wellman	tt0022430	
1746200219	1969	1969	Justine	Justine	Cukor, George	George Cukor	tt0064526	
1746500341	1925	1925	Romance and Rustlers	Romance and Rustlers	Wilson, Ben	Ben F. Wilson	tt0127765	
1746248350	1966	1966	The Last of the Secret Agents?	The Last of the Secret Agents?	Abbott, Norman	Norman Abbott	tt0060619	
1746577802	1935	1935	Danger Ahead	Danger Ahead	Herman, Al	Albert Herman	tt0026259	
1746515704	1918	1918	Danger -Xxx Go Slow	Danger, Go Slow	Leonard, Robert	Robert Z. Leonard	tt0008993	
1745272315	2000	2000	Space Cowboys	Space Cowboys	Eastwood, Clint	Clint Eastwood	tt0186566	
1769970399	1966	1966	The Devil's Sisters	The Devil's Sisters	Grefé, William	William Grefé	tt0058008	
1746577245	1963	1963	The Man From Galveston	The Man from Galveston	Conrad, William	William Conrad	tt0057283	
1746508386	1925	1925	Down Upon the Suwannee River	Down Upon the Suwanee River	Kennedy, Lem F.	Lem F. Kennedy	tt0130635	
1746233855	1968	1968	Invitation to Ruin	Invitation to Ruin	Richter, Kurt	Kurt Richter	tt0063138	
1746235411	1971	1971	There's Always Vanilla	There's Always Vanilla		George A. Romero	tt0166843	
**1746566173**	**1956**	**1955**	**The Man with the Golden Arm**	**The Man with the Golden Arm**	**Preminger, Otto**	**Otto Preminger**	**tt0048347**	1
1746394452	1989	1989	Mystery Train	Mystery Train	Jarmusch, Jim	Jim Jarmusch	tt0097940	
1746515055	1923	1923	The Apache Dancer	The Apache Dancer	Seeling, Charles R.	Charles R. Seeling	tt0013836	
1746491779	1946	1946	Singing on the Trail	Singing on the Trail	Nazarro, Ray	Ray Nazarro	tt0038945	
1746475895	1946	1946	Her Kind of Man	Her Kind of Man	Cordova, Frederick de	Frederick De Cordova	tt0038597	
1746549972	1955	1955	No Man's Woman	No Man's Woman	Adreon, Franklin	Franklin Adreon	tt0048426	
1746584072	1939	1939	Here I Am a Stranger	Here I Am a Stranger	Del Ruth, Roy; Seiter, William	Roy Del Ruth	tt0031418	
1746473941	1947	1947	Blondie's Holiday	Blondie's Holiday	Berlin, Abby	Abby Berlin	tt0039203	
1746198880	1966	1966	The Alley Cats	The Alley Cats	Metzger, Radley H.	Radley Metzger	tt0064006	
1746584031	1938	1938	Man's Paradise	Man's Paradise	Shamroy, Leon	Leon Shamroy	tt0151481	
1769970533	1934	1934	The Camera Speaks	The Camera Speaks			tt1280500	
1746569517	1957	1957	Pawnee	Pawnee	Waggner, George	George Waggner	tt0050827	
1746467429	1917	1917	It Happened to Adele	It Happened to Adele	Brooke, Van Dyke	Van Dyke Brooke	tt0176858	
1746419612	1982	1982	Forty Deuce	Forty Deuce	Morrissey, Paul	Paul Morrissey	tt0083962	
1746578016	1938	1938	Prison Train	Prison Train	Wiles, Gordon	Gordon Wiles	tt0030628	
1745373972	2009	2009	Did You Hear About the Morgans?	Did You Hear About the Morgans?	Lawrence, Marc	Marc Lawrence	tt1314228	
1746420389	1992	1992	The Public Eye	The Public Eye	Franklin, Howard	Howard Franklin	tt0105187	
1746436633	1940	1940	The Sagebrush Family Trails West	The Sagebrush Family Trails West	Stewart, Peter	Sam Newfield	tt0033010	

1746568362	1933	1933	The World Gone Mad	The World Gone Mad	Cabanne, Christy	Christy Cabanne	tt0024787	
1746199547	1969	1969	Angel in My Pocket	Angel in My Pocket	Rafkin, Alan	Alan Rafkin	tt0064026	
1745373704	2008	2008	Cadillac Records	Cadillac Records	Martin, Darnell	Darnell Martin	tt1042877	
1745357960	2003	2003	It Runs in the Family	It Runs in the Family	Schepisi, Fred	Fred Schepisi	tt0311110	
1746578573	1959	1959	Lone Texan	Lone Texan	Landres, Paul	Paul Landres	tt0053016	
1746513864	1916	1916	The Buried Treasure of Cobre	The Buried Treasure of Cobre	Beal, Frank	Frank Beal	tt0421897	
1746453489	1942	1942	Border Roundup	Border Roundup	Newfield, Sam	Sam Newfield	tt0034542	
1746235752	1978	1978	An Unmarried Woman	An Unmarried Woman	Mazursky, Paul	Paul Mazursky	tt0078444	
1746271929	1965	1965	Boeing Boeing	Boeing, Boeing	Rich, John	John Rich	tt0058981	
1746504180	1921	1921	You Find It Everywhere	You Find It Everywhere	Horan, Charles	Charles Horan	tt0012871	
1746581432	1928	1928	Trails of Treachery	Trails of Treachery	Horner, Robert J.	Robert J. Horner	tt0173351	
1746532494	1914	1914	Detective Craig's Coup	Detective Craig's Coup		Donald MacKenzie	tt0003830	
1746565495	1952	1952	The Maverick	The Maverick	Carr, Thomas	Thomas Carr	tt0044890	
1746438925	1943	1943	Girl Crazy	Girl Crazy	Taurog, Norman	Norman Taurog	tt0035942	
1746500319	1926	1926	The Little Irish Girl	The Little Irish Girl	Del Ruth, Roy	Roy Del Ruth	tt0017071	
1746577973	1938	1938	Joy of Living	Joy of Living	Garnett, Tay	Tay Garnett	tt0030293	
1746491736	1948	1948	Fighting Back	Fighting Back	St. Clair, Mal	Malcolm St. Clair	tt0040354	
1746545084	1940	1940	Friendly Neighbors	Friendly Neighbors	Grinde, Nick	Nick Grinde	tt0032495	
1746436813	1990	1990	Berkeley in the Sixties	Berkeley in the Sixties	Kitchell, Mark	Mark Kitchell	tt0099121	
1746421326	1981	1981	Dragonslayer	Dragonslayer	Robbins, Matthew	Matthew Robbins	tt0082288	
1746474400	1948	1948	Jungle Patrol	Jungle Patrol	Newman, Joe	Joseph M. Newman	tt0040502	
1746464971	1944	1944	A WAVE, a WAC and a Marine	A Wave, a WAC and a Marine	Karlstein, Phil	Phil Karlson	tt0037448	
1746520943	1950	1950	Hollywood Varieties	Hollywood Varieties	Landres, Paul	Paul Landres	tt0042570	
1746410675	1988	1988	The Kiss	The Kiss	Densham, Pen	Pen Densham	tt0095454	
1746500072	1946	1946	Blue Skies	Blue Skies	Heisler, Stuart	Stuart Heisler	tt0038370	
1746563834	1952	1952	The Frontier Phantom	The Frontier Phantom	Ormond, Ron	Ron Ormond	tt0044639	
1746585297	1929	1929	Footlights and Fools	Footlights and Fools	Seiter, William A.	William A. Seiter	tt0019892	
1745342244	2001	2001	Pinero	Piñero	Ichaso, Leon	Leon Ichaso	tt0261066	
1746553736	1965	1965	Pussy Galore	Pussy Galore			tt0445652	
1746584407	1939	1939	Union Pacific	Union Pacific	De Mille, Cecil B.	Cecil B. DeMille	tt0032080	
1746420368	1992	1992	Juice	Juice	Dickerson, Ernest R.	Ernest R. Dickerson	tt0104573	
1746571381	1932	1932	Murders in the Rue Morgue	Murders in the Rue Morgue	Florey, Robert	Robert Florey	tt0023249	
1746492557	1944	1944	Brazil	Brazil	Santley, Joseph	Joseph Santley	tt0036670	
1746572401	1931	1931	Heartbreak	Heartbreak	Werker, Alfred	Alfred L. Werker	tt0021946	
1745293375	2006	2005	Shadowboxer	Shadowboxer	Daniels, Lee	Lee Daniels	tt0396857	
1746500274	1924	1924	That French Lady	That French Lady	Mortimer, Edmund	Edmund Mortimer	tt0015397	
1746568281	1934	1934	The Oil Raider	The Oil Raider	Bennet, Spencer Gordon	Spencer Gordon Bennet	tt0282813	
1746574644	1933	1933	Secret Sinners	Secret Sinners	Ford, Wesley	Wesley Ford	tt0024536	
1746394225	1990	1990	Look Who's Talking Too	Look Who's Talking Too	Heckerling, Amy	Amy Heckerling	tt0100050	
1746566198	1955	1955	Seven Cities of Gold	Seven Cities of Gold	Webb, Robert D.	Robert D. Webb	tt0048603	
1746560973	1932	1932	The Dentist	The Dentist	Pearce, Leslie	Leslie Pearce	tt0022807	
1746475526	1948	1948	Glamour Girl	Glamour Girl	Dreifuss, Arthur	Arthur Dreifuss	tt0040392	
1746580670	1939	1939	Trigger Pals	Trigger Pals	Newfield, Sam	Sam Newfield	tt0030893	
**1746577772**	**1933**	**1933**	**Nagana**	**Nagana**	**Frank, Ernst L.**	**Ernst L. Frank**	**tt0024372**	1
1746492091	1945	1945	Son of Lassie	Son of Lassie	Simon, S. Sylvan	S. Sylvan Simon	tt0038097	
1746454099	1941	1941	Under Age	Under Age	Dmytryk, Edward	Edward Dmytryk	tt0034335	
1746580492	1936	1936	Back to Nature	Back to Nature	Tinling, James	James Tinling	tt0027326	
1746500438	1927	1927	Lightning	Lightning	McKay, James C.	James C. McKay	tt0018092	
1746565897	1953	1954	Miss Robin Crusoe	Miss Robin Crusoe	Frenke, Eugene	Eugene Frenke	tt0047240	
1746533044	1915	1915	One Million Dollars	One Million Dollars	Noble, John W.	John W. Noble	tt0005837	
1746508530	1926	1926	Wild Oats Lane	Wild Oats Lane	Neilan, Marshall	Marshall Neilan	tt0017559	
1746523445	1910	1910	The Ruling Passion	The Ruling Passion		William F. Haddock	tt2239162	
1746503745	1920	1920	The Devil's Garden	The Devil's Garden	Webb, Kenneth	Kenneth S. Webb	tt0011113	
1746582970	1936	1936	Winterset	Winterset	Santell, Alfred	Alfred Santell	tt0028511	
1746507592	1926	1926	The Non-Stop Flight	The Non Stop Flight	Johnson, Emory	Emory Johnson	tt0338288	
1746549942	1954	1954	Dragnet	Dragnet	Webb, Jack	Jack Webb	tt0046931	
1746508066	1925	1925	Code of the West	Code of the West	Howard, William K.	William K. Howard	tt0015694	

1746560692	1932	1932	The Crooked Circle	The Crooked Circle	Humberstone, H. Bruce	H. Bruce Humberstone	tt0022788
1746497618	1920	1920	A Dark Lantern	A Dark Lantern	Robertson, John S.	John S. Robertson	tt0010048
1746584081	1935	1935	Navy Wife	Navy Wife	Dwan, Allan	Allan Dwan	tt0026769
1746273539	1979	1979	Van Nuys Blvd.	Van Nuys Blvd.	Sachs, William	William Sachs	tt0080081
1769970411	1966	1966	Aroused	Aroused	Holden, Anton	Anton Holden	tt0060127
1745340994	2008	2008	Prom Night	Prom Night	McCormick, Nelson	Nelson McCormick	tt0926129
1746553461	1964	1964	Santa Claus Conquers the Martians	Santa Claus Conquers the Martians	Webster, Nicholas	Nicholas Webster	tt0058548
1746393986	1992	1992	Housesitter	HouseSitter	Oz, Frank	Frank Oz	tt0104452
1746520706	1926	1926	Striving for Fortune	Striving for Fortune	Ross, Nat	Nat Ross	tt0017436
1746574938	1933	1932	The Horror	The Horror	Pollard, Bud	Bud Pollard	tt0194949
1746584715	1929	1929	Wolf Song	The Wolf Song	Fleming, Victor	Victor Fleming	tt0020595
1746577743	1934	1934	Dames	Dames	Enright, Ray	Ray Enright	tt0025028
1746570818	1933	1933	Turn Back the Clock	Turn Back the Clock	Selwyn, Edgar	Edgar Selwyn	tt0024704
1746500833	1910	1910	The Samaritan's Courtship	The Samaritan's Courtship			tt1787820
1746565956	1953	1953	Mexican Manhunt	Mexican Manhunt	Bailey, Rex	Rex Bailey	tt0046067
1746576859	1932	1932	Night Court	Night Court	Van Dyke, W. S.	W.S. Van Dyke	tt0023268
1746498304	1918	1918	The Splendid Sinner	The Splendid Sinner	Carewe, Edwin	Edwin Carewe	tt0009643
1746501332	1922	1922	I Can Explain	I Can Explain	Baker, George D.	George D. Baker	tt0013260
1746437337	1943	1943	Hoppy Serves a Writ	Hoppy Serves a Writ	Archainbaud, George	George Archainbaud	tt0036016
1745374557	2008	2008	Superhero Movie	Superhero Movie	Mazin, Craig	Craig Mazin	tt0426592
1746500971	1916	1916	The Lost Bracelet	The Lost Bracelet	Mayo, Melvin	Frank Mayo	tt0492180
1746574486	1935	1935	Manhattan Moon	Manhattan Moon	Walker, Stuart	Stuart Walker	tt0026679
1746500290	1949	1949	A Kiss in the Dark	A Kiss in the Dark	Daves, Delmer	Delmer Daves	tt0041550
1746454377	1944	1944	Secret Command	Secret Command	Sutherland, Eddie	A. Edward Sutherland	tt0037253
1746208420	1969	1969	The Love God?	The Love God?	Hiken, Nat	Nat Hiken	tt0064606
1746581499	1928	1928	Steamboat Bill, Jr.	Steamboat Bill, Jr.	Reisner, Charles F.	Charles Reisner	tt0019421
1746514341	1910	1910	The Message of the Violin	The Message of the Violin	Griffith, D. W.	D.W. Griffith	tt0001315
1746454649	1942	1942	Saboteur	Saboteur	Hitchcock, Alfred	Alfred Hitchcock	tt0035279
1746514876	1916	1916	Faith	Faith	Kirkwood, James	James Kirkwood	tt0006662
1746580502	1936	1936	Straight from the Shoulder	Straight from the Shoulder	Heisler, Stuart	Stuart Heisler	tt0028316
1746523352	1916	1916	The Purification of Mulfera	The Purification of Mulfers	Horne, James W.	James W. Horne	tt3140204
1746583320	1928	1928	The Floating College	The Floating College	Crone, George J.	George Crone	tt0018898
1746438301	1943	1943	Harrigan's Kid	Harrigan's Kid	Reisner, Charles F.	Charles Reisner	tt0035971
1746503829	1918	1918	The Inn of the Blue Moon	The Inn of the Blue Moon	O'Brien, John B.	John B. O'Brien	tt0184613
1746580890	1935	1935	Dressed to Thrill	Dressed to Thrill	Lachman, Harry	Harry Lachman	tt0026296
1746523484	1915	1915	Conscience	Conscience	Paton, Stuart	Stuart Paton	tt0189440
1746409711	1988	1988	A Night in the Life of Jimmy Reardon	A Night in the Life of Jimmy Reardon	Richert, William	William Richert	tt0095736
1746515993	1921	1921	Thunder Island	Thunder Island	Dawn, Norman	Norman Dawn	tt0012756
1746501478	1915	1915	A Phyllis of the Sierras	A Phyllis of the Sierras	Middleton, George E.	George E. Middleton	tt0005892
1746582208	1930	1930	The Concentratin' Kid	The Concentratin' Kid	Rosson, Arthur	Arthur Rosson	tt0020783
1746435541	1943	1943	My Son, the Hero	My Son, the Hero	Ulmer, Edgar G.	Edgar G. Ulmer	tt0036185
1746565223	1953	1953	City of Bad Men	City of Bad Men	Jones, Harmon	Harmon Jones	tt0045632
1746582948	1937	1937	Slim	Slim	Enright, Ray	Ray Enright	tt0029579
1746485616	1920	1920	The House of Whispers	The House of Whispers	Warde, Ernest C.	Ernest C. Warde	tt0011311
1746491870	1946	1946	Don Ricardo Returns	Don Ricardo Returns	Morse, T. O.	Terry O. Morse	tt0162292
1746438928	1942	1942	Between Us Girls	Between Us Girls	Koster, Henry	Henry Koster	tt0034509
1746454030	1943	1943	Tarzan's Desert Mystery	Tarzan's Desert Mystery	Thiele, William	Wilhelm Thiele	tt0035795
1746573902	1962	1962	Two Before Zero	Two Before Zero	Faralla, William	William D. Faralla	tt0056622
1746453539	1945	1944	To Have and Have Not	To Have and Have Not	Hawks, Howard	Howard Hawks	tt0037382
1746410519	1987	1987	Disorderlies	Disorderlies	Schultz, Michael	Michael Schultz	tt0092897
1746513032	1923	1923	Mothers-in-Law	Mothers in Law	Gasnier, Louis	Louis J. Gasnier	tt0014294
1746485956	1918	1918	Nine-Tenths of the Law	Nine Tenths of the Law	Eason, Reaves	B. Reeves Eason	tt0009428
1746524932	1923	1923	The Truth About Wives	The Truth About Wives	Windom, Lawrence	Lawrence C. Windom	tt0014564
1746584405	1939	1939	Fighting Mad	Fighting Mad	Newfield, Sam	Sam Newfield	tt0031305
1746516114	1920	1920	Conrad in Quest of His Youth	Conrad in Quest of His Youth	de Mille, William C.	William C. de Mille	tt0011070
1746501052	1916	1916	The Eternal Question	The Eternal Question	King, Burton L.	Burton L. King	tt0157604
1746508102	1925	1925	Midnight Molly	Midnight Molly	Ingraham, Lloyd	Lloyd Ingraham	tt0016108

1745358563	2009	2009	Fired Up	Fired Up!	Gluck, Will	Will Gluck	tt1083456
1746453870	1996	1996	The Mirror Has Two Faces	The Mirror Has Two Faces	Streisand, Barbra	Barbra Streisand	tt0117057
1745342711	2009	2009	Underworld: Rise of the Lycans	Underworld: Rise of the Lycans	Tatopoulos, Patrick	Patrick Tatopoulos	tt0834001
1746393474	1981	1981	Paternity	Paternity	Steinberg, David	David Steinberg	tt0082886
1746553443	1964	1964	Vice Girls, Ltd.	Vice Girls Ltd.	Chaudhri, Amin	Amin Q. Chaudhri	tt0260504
1746420042	1993	1993	Tombstone	Tombstone	Cosmatos, George P.	George P. Cosmatos	tt0108358
1746574224	1960	1960	Battle of Blood Island	Battle of Blood Island	Rapp, Joel M.	Joel Rapp	tt0168492
1746421146	1996	1996	Lone Star	Lone Star	Sayles, John	John Sayles	tt0116905
1745359631	2003	2003	Blue Collar Comedy Tour: The Movie	Blue Collar Comedy Tour: The Movie	Harding, C. B.	C.B. Harding	tt0330069
1746560946	1932	1932	Gold	Gold	Brower, Otto	Otto Brower	tt0022951
1746512432	1923	1923	The Little Girl Next Door	The Little Girl Next Door	Van Dyke, W. S.	W.S. Van Dyke	tt0014203
1746584816	1930	1930	Men Without Law	Men Without Law	King, Louis	Louis King	tt0021139
1746439127	1941	1941	Caught in the Act	Caught in the Act	Yarbrough, Jean	Jean Yarbrough	tt0033454
1746419745	1940	1940	Gun Code	Gun Code		Sam Newfield	tt0032563
1746580508	1935	1935	Together We Live	Together We Live	Mack, Willard	Willard Mack	tt0027116
1746410356	1987	1986	Sorority House Massacre	Sorority House Massacre	Frank, Carol	Carol Frank	tt0091990
1746418621	1940	1940	World in Flames	World in Flames			tt6424172
1746577998	1934	1935	I've Been Around	I've Been Around	Cahn, Phil	Philip Cahn	tt0026515
1746503429	1918	1918	The Wife He Bought	The Wife He Bought	Solter, Harry	Harry Solter	tt0009807
1746574884	1935	1934	Western Justice	Western Justice	Bradbury, Robert N.	Robert N. Bradbury	tt0027199
1746582396	1950	1950	Sunset Blvd.	Sunset Blvd.	Wilder, Billy	Billy Wilder	tt0043014
1746566124	1953	1953	Pony Express	Pony Express	Hopper, Jerry	Jerry Hopper	tt0046198
1746578998	1960	1960	North to Alaska	North to Alaska	Hathaway, Henry	Henry Hathaway	tt0054127
1746561522	1957	1957	Naked Paradise	Naked Paradise	Corman, Roger	Roger Corman	tt0050748
1746270442	1980	1979	The Great Santini	The Great Santini	Carlino, Lewis John	Lewis John Carlino	tt0079239
1746584970	1940	1940	The Border Legion	The Border Legion	Kane, Joseph	Joseph Kane	tt0032274
1746185870	1969	1969	Hail, Hero!	Hail, Hero!	Miller, David	David Miller	tt0064399
1745357931	2001	2000	Pie in the Sky: The Brigid Berlin Story	Pie in the Sky: The Brigid Berlin Story	Fremont, Vincent ; Fremont, Shelly D	Shelly Dunn Fremont	tt0261174
1746475720	1944	1944	The Merry Monahans	The Merry Monahans	Lamont, Charles	Charles Lamont	tt0037070
1746408424	1981	1981	Chu Chu and the Philly Flash	Chu Chu and the Philly Flash	Rich, David Lowell	David Lowell Rich	tt0082177
1745302364	2007	2006	Hatchet	Hatchet	Green, Adam	Adam Green	tt0422401
1746200493	1968	1968	5 Card Stud	5 Card Stud	Hathaway, Henry	Henry Hathaway	tt0062626
1746523737	1923	1923	Alice Adams	Alice Adams	Lee, Rowland V.	Rowland V. Lee	tt0013822
1746492405	1948	1948	Old Los Angeles	Old Los Angeles	Kane, Joe	Joseph Kane	tt0040469
1746437431	1940	1940	The Blue Bird	The Blue Bird	Lang, Walter	Walter Lang	tt0032264
1746572562	1932	1932	Careless Lady	Careless Lady	MacKenna, Kenneth	Kenneth MacKenna	tt0022746
1746208184	1969	1969	The House Near the Prado	The House Near the Prado	Van Hearn, J.	Jean Van Hearn	tt0064447
1746570584	1934	1934	Our Daily Bread	Our Daily Bread	Vidor, King	King Vidor	tt0025610
1746501929	1913	1913	One Hundred Years of Mormonism	One Hundred Years of Mormonism		Norval MacGregor	tt0003241
1746583413	1930	1930	Ladies in Love	Ladies in Love	Lewis, Edgar	Edgar Lewis	tt0021039
1746200188	1966	1965	The Money Trap	The Money Trap	Kennedy, Burt	Burt Kennedy	tt0059460
1746454641	1941	1941	Spooks Run Wild	Spooks Run Wild	Rosen, Phil	Phil Rosen	tt0034224
1746560094	1964	1964	Day of a Stripper	Day of a Stripper			tt0445318
1746434000	1997	1997	Gridlock'd	Gridlock'd	Hall, Vondie Curtis	Vondie Curtis	tt0119225
1746574091	1960	1960	13 Fighting Men	13 Fighting Men	Gerstad, Harry	Harry W. Gerstad	tt0053558
1746583878	1929	1929	Saturday's Children	Saturday's Children	La Cava, Gregory	Gregory La Cava	tt0020365
1746583207	1931	1931	Is There Justice?	Is There Justice?	Paton, Stuart	Stuart Paton	tt0022003
1746454836	1946	1946	Beware	Beware	Pollard, Bud	Bud Pollard	tt0038353
1746514850	1916	1916	Wild Oats	Wild Oats	Gollan, Campbell	Campbell Gullan	tt0274217
1746520451	1949	1949	Sky Liner	Sky Liner	Berke, Wm.	William Berke	tt0178896
1746501414	1923	1923	Jazzmania	Jazzmania	Leonard, Robert Z.	Robert Z. Leonard	tt0014164
1746493843	1913	1913	Robin Hood	Robin Hood		Theodore Marston	tt0003337
1746502215	1916	1916	The Criminal	The Criminal	Barker, Reginald	Reginald Barker	tt0161465
1746580628	1935	1935	Desert Mesa	Desert Mesa	James, Art	Victor Adamson	tt0026272
1746419138	1988	1988	The Thin Blue Line	The Thin Blue Line	Morris, Errol	Errol Morris	tt0096257
1746198153	1969	1969	My Girlfriend's Wedding	My Girlfriend's Wedding	McBride, Jim	Jim McBride	tt0064706
1746435238	1940	1940	South of Pago Pago	South of Pago Pago	Green, Alfred E.	Alfred E. Green	tt0033082

1746495874	1920	1920	Sophy of Kravonia; or, the Virgin of Paris	Sophy of Kravonia; or, The Virgin of Paris	Fontaine, Gerard	Gerard Fontaine	tt0197907	
1746393695	1984	1984	Friday the 13th: The Final Chapter	Friday the 13th: The Final Chapter	Zito, Joseph	Joseph Zito	tt0087298	
1746569792	1957	1957	Black Patch	Black Patch	Miner, Allen H.	Allen H. Miner	tt0050196	
1769969379	1930	1930	Western Knights	Western Knights	Roberts, Stephen	Stephen Roberts	tt0021541	
1746500506	1926	1926	The Strong Man	The Strong Man	Capra, Frank	Frank Capra	tt0017437	
1746574098	1961	1961	One-Eyed Jacks	One Eyed Jacks	Brando, Marlon	Marlon Brando	tt0055257	
1746563646	1952	1951	The Model and the Marriage Broker	The Model and the Marriage Broker	Cukor, George	George Cukor	tt0043813	
1746234825	1976	1976	The Shootist	The Shootist	Siegel, Don	Don Siegel	tt0075213	
**1746532713**	**1923**	**1923**	**Tea--With a Kick**	**Tea: With a Kick!**	**Kenton, Erle C.**	**Erle C. Kenton**	**tt0014527**	1
1746581653	1951	1951	The Lion Hunters	The Lion Hunters	Beebe, Ford	Ford Beebe	tt0043746	
1746202900	1970	1970	Little Big Man	Little Big Man	Penn, Arthur	Arthur Penn	tt0065988	
1746514947	1916	1916	For the Defense	For the Defense	Reicher, Frank	Frank Reicher	tt0006696	
1746582212	1931	1931	Quick Millions	Quick Millions	Brown, Rowland	Rowland Brown	tt0022290	
1745339879	2002	2002	Road to Perdition	Road to Perdition	Mendes, Sam	Sam Mendes	tt0257044	
1746421520	1940	1940	Comin' Round the Mountain	Comin' Round the Mountain	Archainbaud, George	George Archainbaud	tt0032347	
1745339992	2001	2001	The Fast and the Furious	The Fast and the Furious	Cohen, Rob	Rob Cohen	tt0232500	
1746584313	1940	1940	My Favorite Wife	My Favorite Wife	Kanin, Garson	Garson Kanin	tt0029284	
1746577307	1962	1962	Paradise Alley	Paradise Alley	Haas, Hugo	Hugo Haas	tt0055274	
1746503563	1922	1922	The Three Must-Get-Theres	The Three Must Get Theres	Linder, Max	Max Linder	tt0013674	
1746582639	1934	1934	The Show-Off	The Show Off	Riesner, Charles F.	Charles Reisner	tt0025785	
1746580370	1936	1936	Charlie Chan's Secret	Charlie Chan's Secret	Wiles, Gordon	Gordon Wiles	tt0027442	
1746499897	1948	1948	The Story of Life	The Story of Life	Bretherton, Howard	Howard Bretherton	tt0040836	
1746508276	1924	1924	The Passing of Wolf MacLean	The Passing of Wolf MacLean	Hurst, Paul	Paul Hurst	tt0015215	
1746438158	1944	1944	Mrs. Parkington	Mrs. Parkington	Garnett, Tay	Tay Garnett	tt0037096	
1746520794	1925	1925	Simon the Jester	Simon the Jester	Melford, George	George Melford	tt0016354	
1746465094	1947	1947	A Night at the Follies	A Night at the Follies	Connell, W. Merle	W. Merle Connell	tt0147089	
1745373462	2004	2004	Noel	Noel	Palminteri, Chazz	Chazz Palminteri	tt0383534	
1746217358	1968	1967	Wavelength	Wavelength	Snow, Michael	Michael Snow	tt0127354	
1746577536	1934	1934	Success at Any Price	Success at Any Price	Ruben, J. Walter	J. Walter Ruben	tt0025844	
1746583064	1939	1939	The Return of the Cisco Kid	Return of the Cisco Kid	Leeds, Herbert I.	Herbert I. Leeds	tt0031852	
1746569955	1959	1959	Good Day for a Hanging	Good Day for a Hanging	Juran, Nathan	Nathan Juran	tt0051673	
1745309489	2007	2007	Martian Child	Martian Child	Meyjes, Menno	Menno Meyjes	tt0415965	
1746584080	1939	1939	Silver on the Sage	Silver on the Sage	Selander, Lesley	Lesley Selander	tt0031925	
1746532940	1916	1916	The Man Behind the Curtain	The Man Behind the Curtain	Van Deusen, Courtlandt J.	Cortland Van Deusen	tt0163085	
1746520657	1926	1926	Sunshine of Paradise Alley	Sunshine of Paradise Alley	Nelson, Jack	Jack Nelson	tt0017442	
1746439234	1996	1996	Don't Be a Menace to South Central While Drink	Don't Be a Menace to South Central While D	Barclay, Paris	Paris Barclay	tt0116126	
1746574135	1958	1958	Ten North Frederick	Ten North Frederick	Dunne, Philip	Philip Dunne	tt0052283	
1746572418	1931	1931	The Avenger	The Avenger	Neill, Roy William	Roy William Neill	tt0021630	
1746394726	1989	1989	Drugstore Cowboy	Drugstore Cowboy	Van Sant, Gus	Gus Van Sant	tt0097240	
1746512921	1916	1916	Through the Wall	Through the Wall	Sturgeon, Rollin S.	Rollin S. Sturgeon	tt0007457	
1746583959	1930	1930	The Runaway Bride	Runaway Bride	Crisp, Donald	Donald Crisp	tt0021324	
1746474840	1947	1947	Desire Me	Desire Me	Cukor, George; LeRoy, Mervyn	Jack Conway	tt0039312	
1746464916	1941	1941	Gambling Daughters	Gambling Daughters	Nosseck, Max	Max Nosseck	tt0033645	
1746582930	1935	1935	Last of the Clintons	The Last of the Clintons	Fraser, Harry	Harry L. Fraser	tt0026608	
1746520463	1950	1950	Cry Murder	Cry Murder	Glenn, Jack	Jack Glenn	tt0042357	
1746508975	1926	1926	Walloping Kid	Walloping Kid	Horner, Robert J.	Robert J. Horner	tt0017523	
1746570715	1952	1952	The Steel Trap	The Steel Trap	Stone, Andrew	Andrew L. Stone	tt0045190	
1746520273	1925	1925	The Fighting Romeo	The Fighting Romeo	Ferguson, Al	Al Ferguson	tt0132949	
1746566154	1957	1957	Jet Pilot	Jet Pilot	Sternberg, Josef von	Josef von Sternberg	tt0050562	
1746500047	1924	1924	Range Blood	Range Blood	Ford, Francis	Francis Ford	tt0015260	
1746435241	1941	1941	When Ladies Meet	When Ladies Meet	Leonard, Robert Z.	Robert Z. Leonard	tt0034386	
1746553717	1964	1964	Strange Compulsion	Strange Compulsion	Berwick, Irvin	Irvin Berwick	tt0199052	
1746565944	1955	1955	Bobby Ware Is Missing	Bobby Ware Is Missing	Carr, Thomas	Thomas Carr	tt0047893	
1746562165	1954	1954	Sabrina	Sabrina	Wilder, Billy	Billy Wilder	tt0047437	
1746409957	1987	1987	Burglar	Burglar	Wilson, Hugh	Hugh Wilson	tt0092710	
1746436075	1943	1943	My Friend Flicka	My Friend Flicka	Schuster, Harold	Harold D. Schuster	tt0036182	
1746454828	1945	1945	Sunbonnet Sue	Sunbonnet Sue	Murphy, Ralph	Ralph Murphy	tt0038135	

1746555975	1955	1955	Seminole Uprising	Seminole Uprising	Bellamy, Earl	Earl Bellamy	tt0048600	
1746508715	1925	1925	Slow Dynamite	Slow Dynamite	Mattison, Frank S.	Frank S. Mattison	tt0165478	
1746410018	1988	1988	Torch Song Trilogy	Torch Song Trilogy	Bogart, Paul	Paul Bogart	tt0096289	
1746464920	1941	1941	Charlie Chan in Rio	Charlie Chan in Rio	Lachman, Harry	Harry Lachman	tt0033458	
1746454254	1944	1944	Bordertown Trail	Bordertown Trail	Selander, Lesley	Lesley Selander	tt0036662	
1746503384	1921	1921	The Iron Trail	The Iron Trail	Neill, R. William	Roy William Neill	tt0012322	
1746502432	1917	1917	The Girl Who Didn't Think	The Girl Who Didn't Think	Haddock, William	William F. Haddock	tt0175668	
1746434679	1992	1992	Newsies	Newsies	Ortega, Kenny	Kenny Ortega	tt0104990	
1746475779	1945	1945	Mom and Dad	Mom and Dad	Beaudine, William	William Beaudine	tt0040603	
1746577216	1961	1961	King of the Roaring 20's--The Story of Arnold Rot	King of the Roaring 20's: The Story of Arnold	Newman, Joseph M.	Joseph M. Newman	tt0055048	
1746583895	1931	1931	Blonde Crazy	Blonde Crazy	Del Ruth, Roy	Roy Del Ruth	tt0021673	
1746393507	1988	1988	Halloween 4: The Return of Michael Myers	Halloween 4: The Return of Michael Myers	Little, Dwight H.	Dwight H. Little	tt0095271	
1746580233	1938	1938	King of the Sierras	King of the Sierras	Diege, Samuel	Samuel Diege	tt0030324	
1746508121	1925	1925	The Night Ship	The Night Ship	McCarthy, Henry	Henry McCarty	tt0016158	
1746502582	1916	1916	The Surprises of an Empty Hotel	The Surprises of an Empty Hotel	Marston, Theodore	Theodore Marston	tt0159008	
1746566352	1958	1958	Desire Under the Elms	Desire Under the Elms	Mann, Delbert	Delbert Mann	tt0051534	
1746495587	1921	1921	Montana Bill	Montana Bill	Goldstone, Phil	Phil Goldstone	tt0012471	
1746574099	1956	1956	The Desperados Are in Town	The Desperados Are in Town	Neumann, Kurt	Kurt Neumann	tt0049135	
1746566248	1953	1953	Rebel City	Rebel City	Carr, Thomas	Thomas Carr	tt0046230	
1746515786	1922	1922	Rent Free	Rent Free	Higgin, Howard	Howard Higgin	tt0013546	
1746435119	1943	1943	The Outlaw	The Outlaw	Hughes, Howard; Hawks, Howard	Howard Hughes	tt0036241	
1746576968	1951	1951	The Strip	The Strip	Kardos, Leslie	László Kardos	tt0044083	
1746532651	1915	1915	Strathmore	Strathmore	Grandon, Francis	Francis J. Grandon	tt0006107	
1746198551	1969	1969	Flareup	Flareup	Neilson, James	James Neilson	tt0064333	
1746585326	1930	1930	The Lottery Bride	The Lottery Bride	Stein, Paul L.	Paul L. Stein	tt0021094	
1746508768	1949	1949	Bomba, the Jungle Boy	Bomba, the Jungle Boy	Beebe, Ford	Ford Beebe	tt0041196	
1746524551	1927	1927	The Cyclone Cowboy	The Cyclone Cowboy	Thorpe, Richard	Richard Thorpe	tt0017787	
1746512346	1927	1927	The Bandit's Son	The Bandit's Son	Fox, Wallace W.	Wallace Fox	tt0017659	
1746509271	1925	1926	Going the Limit	Going the Limit	Worne, Duke	Chester Withey	tt0133811	
1746574571	1936	1936	Satan Met a Lady	Satan Met a Lady	Dieterle, William	William Dieterle	tt0028219	
1746585247	1930	1930	Sunny Skies	Sunny Skies	Taurog, Norman	Norman Taurog	tt0021436	
1746584894	1939	1939	The Taming of the West	Taming of the West	Deming, Norman	Norman Deming	tt0032006	
1746208567	1970	1970	Zabriskie Point	Zabriskie Point	Antonioni, Michelangelo	Michelangelo Antonioni	tt0066601	
1746524895	1914	1914	Life's Shop Window	Life's Shop Window	Edwards, J. Gordon	J. Gordon Edwards	tt0004224	
1746581185	1910	1910	A Knot in the Plot	A Knot in the Plot	Powell, Frank	Frank Powell	tt0001278	
1746584748	1930	1930	Top Speed	Top Speed	LeRoy, Mervyn	Mervyn LeRoy	tt0021486	
1746502231	1915	1915	The Lamb	The Lamb	Cabanne, W. Christy	Christy Cabanne	tt0005600	
1746492751	1946	1946	The Spider Woman Strikes Back	The Spider Woman Strikes Back	Lubin, Arthur	Arthur Lubin	tt0038974	
1746454639	1942	1942	The Phantom Plainsmen	The Phantom Plainsmen	English, John	John English	tt0035186	
1746464905	1941	1941	Arkansas Judge	Arkansas Judge	McDonald, Frank	Frank McDonald	tt0033353	
1746454073	1940	1940	The Fighting 69th	The Fighting 69th	Keighley, William	William Keighley	tt0032467	
1746514582	1923	1923	McGuire of the Mounted	McGuire of the Mounted	Stanton, Richard	Richard Stanton	tt0014265	
1746524844	1928	1928	Object--Alimony	Object: Alimony	Dunlap, Scott R.	Scott R. Dunlap	tt0019224	
1746485591	1919	1919	Thunderbolts of Fate	Thunderbolts of Fate	Warren, Edward	Edward Warren	tt0010780	
1746464771	1942	1942	My Favorite Blonde	My Favorite Blonde	Lanfield, Sidney	Sidney Lanfield	tt0035100	
1746520177	1926	1926	The Set-Up	The Set Up	Smith, Clifford S.	Clifford Smith	tt0017367	
1746566196	1953	1953	The Vanquished	The Vanquished	Ludwig, Edward	Edward Ludwig	tt0046492	
1746436356	1943	1943	His Butler's Sister	His Butler's Sister	Borzage, Frank	Frank Borzage	tt0036001	
1746516321	1921	1921	The Stranger in Canyon Valley	The Stranger in Canyon Valley	Smith, Cliff	Clifford Smith	tt0341022	
1769970622	1968	1968	She-Devils on Wheels	She Devils on Wheels	Lewis, Herschell Gordon	Herschell Gordon Lewis	tt0063595	
1746218708	1974	1973	The Golden Voyage of Sinbad	The Golden Voyage of Sinbad	Hessler, Gordon	Gordon Hessler	tt0071569	
1746573967	1961	1961	Nikki, Wild Dog of the North	Nikki, Wild Dog of the North		Jack Couffer	tt0055232	
1769970109	1912	1912	Bessie's Dream	Bessie's Dream		Colin Campbell	tt0416520	
1746420957	1995	1996	Fun	The Fan	Zielinski, Rafal	Tony Scott	tt0116277	
1746530724	1949	1949	Tough Assignment	Tough Assignment	Beaudine, William	William Beaudine	tt0041976	
**1746585424**	**1940**	**1940**	**Brother Orchid**	**Brother Orchid**	**Bacon, Lloyd**	**Lloyd Bacon**	**tt0032285**	1
1746508184	1925	1925	The Overland Limited	The Overland Limited	O'Neill, Frank	Frank O'Neill	tt0016192	

1746487049	1919	1919	Fortune's Child	Fortune's Child	Gleason, Joseph	Joseph Gleason	tt0325415
1746453820	1942	1942	Drums of the Congo	Drums of the Congo	Cabanne, Christy	Christy Cabanne	tt0034684
1746566011	1953	1953	So Big	So Big	Wise, Robert	Robert Wise	tt0046333
1746437019	1943	1943	Jitterbugs	Jitterbugs	St. Clair, Mal	Malcolm St. Clair	tt0036055
1745342874	2008	2008	The Curious Case of Benjamin Button	The Curious Case of Benjamin Button	Fincher, David	David Fincher	tt0421715
1746520364	1925	1925	The Wrongdoers	The Wrongdoers	Dierker, Hugh	Hugh Dierker	tt0016553
1746465262	1944	1944	In Society	In Society	Yarbrough, Jean	Jean Yarbrough	tt0036953
1746520942	1949	1949	The Great Sinner	The Great Sinner	Siodmak, Robert	Robert Siodmak	tt0041430
1746576502	1932	1932	Strange Justice	Strange Justice	Schertzinger, Victor	Victor Schertzinger	tt0023524
1746409477	1987	1987	Barfly	Barfly	Schroeder, Barbet	Barbet Schroeder	tt0092618
1746454066	1943	1943	Night Plane from Chungking	Night Plane from Chungking	Murphy, Ralph	Ralph Murphy	tt0035125
1746513434	1917	1917	American--That's All	American That's All	Rosson, Arthur	Arthur Rosson	tt0007632
1746574303	1962	1962	Fallguy	Fallguy	Harling, Donn	Donn Harling	tt0055974
1746235860	1973	1973	Magnum Force	Magnum Force	Post, Ted	Ted Post	tt0070355
1745393664	2009	2008	It Might Get Loud	It Might Get Loud	Guggenheim, Davis	Davis Guggenheim	tt1229360
1746568224	1934	1934	Young and Beautiful	Young and Beautiful	Santley, Joseph	Joseph Santley	tt0026019
1746584444	1930	1930	The Other Tomorrow	The Other Tomorrow	Bacon, Lloyd	Lloyd Bacon	tt0021222
1745293591	2006	2005	Dave Chappelle's Block Party	Dave Chappelle's Block Party	Gondry, Michel	Michel Gondry	tt0425598
1746584288	1935	1935	The Irish Gringo	The Irish Gringo	Thompson, Wm. C.	William C. Thompson	tt0027801
1746454303	1941	1941	Rookies on Parade	Rookies on Parade	Santley, Joseph	Joseph Santley	tt0034129
1746520634	1926	1926	Tramp, Tramp, Tramp	Tramp, Tramp, Tramp	Edwards, Harry	Harry Edwards	tt0017483
1746500171	1926	1926	Lure of the West	Lure of the West	Neitz, Alvin J.	Alan James	tt0016056
1745340264	2002	2002	Austin Powers in Goldmember	Austin Powers in Goldmember	Roach, Jay	Jay Roach	tt0295178
1745358507	2004	2004	Benji: Off the Leash!	Benji: Off the Leash!	Camp, Joe	Joe Camp	tt0315273
1746520210	1949	1949	A Woman's Secret	A Woman's Secret	Ray, Nicholas	Nicholas Ray	tt0042053
1746491998	1947	1947	Killer McCoy	Killer McCoy	Rowland, Roy	Roy Rowland	tt0039531
1746437143	1997	1997	The Ice Storm	The Ice Storm	Lee, Ang	Ang Lee	tt0119349
1746584339	1936	1936	Stormy Trails	Stormy Trails	Newfield, Sam	Sam Newfield	tt0028311
1746502894	1912	1912	The Ambitious Butler	Ambitious Butler	Sennett, Mack	Mack Sennett	tt0002017
1746533223	1916	1915	By Whose Hand?	By Whose Hand?	Durkin, James	Hamilton Smith	tt1148240
1746520876	1950	1950	The Golden Twenties	The Golden Twenties			tt0249549
1746393469	1985	1985	The Man with One Red Shoe	The Man with One Red Shoe	Dragoti, Stan	Stan Dragoti	tt0089543
1746524272	1923	1923	The Ranchers	The Ranchers		George Elliot	tt0014395
1746507896	1925	1925	Tessie	Tessie	Fitzgerald, Dallas M.	Dallas M. Fitzgerald	tt0016415
1746410671	1992	1992	Hellraiser III: Hell on Earth	Hellraiser III: Hell on Earth	Hickox, Anthony	Anthony Hickox	tt0104409
1746524663	1917	1917	Hate	Hate	Stahl, Walter Richard	Walter Richard Stahl	tt0008050
1746572833	1931	1931	Too Young to Marry	Too Young to Marry	LeRoy, Mervyn	Mervyn LeRoy	tt0020715
1746464946	1942	1942	Boot Hill Bandits	Boot Hill Bandits	Luby, S. Roy	S. Roy Luby	tt0034541
1746421153	1997	1997	The Man Who Knew Too Little	The Man Who Knew Too Little	Amiel, Jon	Jon Amiel	tt0120483
1746577955	1934	1934	Looking for Trouble	Looking for Trouble	Wellman, William	William A. Wellman	tt0025418
1746475919	1946	1946	Night and Day	Night and Day	Curtiz, Michael	Michael Curtiz	tt0038776
1745396001	2009	2009	Paul Blart Mall Cop	Paul Blart: Mall Cop	Carr, Steve	Steve Carr	tt1114740
1746563836	1951	1951	When the Redskins Rode	When the Redskins Rode	Landers, Lew	Lew Landers	tt0044208
1746453814	1943	1943	The Battle of Russia	The Battle of Russia	Litvak, Anatole	Frank Capra	tt0036629
1746578402	1960	1960	Morals Squad	Morals Squad		Barry Mahon	tt0312977
1745341580	2003	2002	People I Know	People I Know	Algrant, Dan	Daniel Algrant	tt0274711
1746233616	1967	1967	Young Americans	Young Americans	Grasshoff, Alex	Alexander Grasshoff	tt0062515
1746271965	1966	1966	The Girl From Tobacco Row	Girl from Tobacco Row	Ormond, Ron	Ron Ormond	tt0060459
1746583760	1929	1928	Sal of Singapore	Sal of Singapore	Higgin, Howard	Howard Higgin	tt0019348
1745293730	2006	2006	Hollywoodland	Hollywoodland	Coulter, Allen	Allen Coulter	tt0427969
1746453713	1999	1999	Boys Don't Cry	Boys Don't Cry	Peirce, Kimberly	Kimberly Peirce	tt0171804
1746585414	1940	1940	The Doctor Takes A Wife	The Doctor Takes a Wife	Hall, Alexander	Alexander Hall	tt0032397
1746516122	1921	1921	False Women	False Women	Armstrong, R. Dale	R. Dale Armstrong	tt0131382
1746497550	1922	1922	Sherlock Holmes	Sherlock Holmes	Parker, Albert	Albert Parker	tt0013597
1746562318	1953	1953	The Diamond Queen	The Diamond Queen	Brahm, John	John Brahm	tt0045687
1746569536	1956	1956	These Wilder Years	These Wilder Years	Rowland, Roy	Roy Rowland	tt0049844
1746475901	1948	1948	Arch of Triumph	Arch of Triumph	Milestone, Lewis	Lewis Milestone	tt0040109

1745358982	2002	2002	Adaptation.	Adaptation.	Jonze, Spike	Spike Jonze	tt0268126
1746577218	1960	1960	The Night Fighters	The Night Fighters	Garnett, Tay	Tay Garnett	tt0054120
1746464770	1941	1941	Whistling in the Dark	Whistling in the Dark	Simon, S. Sylvan	S. Sylvan Simon	tt0034389
1746486287	1921	1921	A Message From Mars	A Message from Mars	Karger, Maxwell	Maxwell Karger	tt0012456
1746433768	2000	1999	Uninvited Guest	Uninvited Guest	Folsome, Timothy Wayne	Timothy Wayne Folsome	tt0188694
1746197921	1970	1970	Joe	Joe	Avildsen, John G.	John G. Avildsen	tt0065916
1746582277	1929	1928	The Viking	The Viking	Neill, R. William	Roy William Neill	tt0019532
1746454881	1997	1997	Dream with the Fishes	Dream with the Fishes	Taylor, Finn	Finn Taylor	tt0119019
1746210397	1971	1971	The Mephisto Waltz	The Mephisto Waltz	Wendkos, Paul	Paul Wendkos	tt0067419
1746464897	1941	1941	The Trail of the Silver Spurs	Trail of the Silver Spurs	Luby, S. Roy	S. Roy Luby	tt0034309
1746493652	1911	1911	Bertie's Bandit	Bertie's Bandit	Beal, Frank	Frank Beal	tt0398755
1746575889	1931	1931	Young As You Feel	Young as You Feel	Borzage, Frank	Frank Borzage	tt0022591
1769971096	1969	1969	Passion in Hot Hollows	Passion in Hot Hollows	Sarno, Joe	Joseph W. Sarno	tt0062100
1746578884	1962	1962	The Chapman Report	The Chapman Report	Cukor, George	George Cukor	tt0055841
1769969552	1911	1911	Desperate Desmond Pursued by Claude Eclaire	Desperate Desmond Pursued by Claude Eclaire		Tom Ricketts	tt0213606
1746198541	1966	1966	Dr. Goldfoot and the Girl Bombs	Dr. Goldfoot and the Girl Bombs	Bava, Mario	Mario Bava	tt0061014
1746570447	1953	1952	Road to Bali	Road to Bali	Walker, Hal	Hal Walker	tt0045094
1746520037	1925	1925	Without Mercy	Without Mercy	Melford, George	George Melford	tt0016543
1746466641	1917	1917	Mutiny	Mutiny	Reynolds, Lynn F.	Lynn Reynolds	tt0008340
1746473936	1944	1944	Sundown Valley	Sundown Valley	Kline, Benjamin	Benjamin H. Kline	tt0037328
1746543080	1915	1916	The Sins That Ye Sin	The Sins That Ye Sin	Thayer, Otis B.	Otis Thayer	tt0158961
1746585466	1939	1939	Million Dollar Legs	Million Dollar Legs	Grinde, Nick	Nick Grinde	tt0031652
1746453778	1942	1942	A Date with the Falcon	A Date with the Falcon	Reis, Irving	Irving Reis	tt0033517
1746571410	1932	1932	Tombstone Canyon	Tombstone Canyon	James, Alan	Alan James	tt0023604
1746562205	1955	1955	The Kentuckian	The Kentuckian	Lancaster, Burt	Burt Lancaster	tt0048248
1746248200	1967	1967	The Fastest Guitar Alive	The Fastest Guitar Alive	Moore, Michael	Michael D. Moore	tt0061652
1746581500	1928	1928	Triple Pass	Triple Pass			tt0174282
1746580325	1935	1936	Night Cargo	Night Cargo	Hutchison, Charles	Charles Hutchison	tt0165402
1746569581	1958	1958	The Inn of the Sixth Happiness	The Inn of the Sixth Happiness	Robson, Mark	Mark Robson	tt0051776
1746492232	1947	1947	The Mighty McGurk	The Mighty McGurk	Waters, John	John Waters	tt0038739
1746583521	1929	1929	Law of the Plains	The Law of the Plains	McGowan, J. P.	J.P. McGowan	tt0020084
1746513873	1915	1915	The Way Back	The Way Back	King, Carlton	Carlton S. King	tt0006247
1746514078	1916	1916	The Clown	The Clown	Mille, William C. de	William C. de Mille	tt0006520
1746514725	1915	1915	Pretty Mrs. Smith	Pretty Mrs. Smith	Bosworth, Hobart	Hobart Bosworth	tt0005921
1746507830	1926	1926	The Yellow Back	The Yellow Back	Andrews, Del	Del Andrews	tt0017582
1746419186	1992	1992	Gas, Food Lodging	Gas, Food Lodging	Anders, Allison	Allison Anders	tt0104321
1746465403	1946	1946	Devil Bat's Daughter	Devil Bat's Daughter	Wisbar, Frank	Frank Wisbar	tt0038470
1746508423	1925	1925	The Sign of the Cactus	The Sign of the Cactus	Smith, Clifford S.	Clifford Smith	tt0016349
1746569963	1958	1958	Frankenstein's Daughter	Frankenstein's Daughter	Cunha, Richard	Richard E. Cunha	tt0051631
1746555552	1957	1957	Dragstrip Girl	Dragstrip Girl	Cahn, Edward L.	Edward L. Cahn	tt0050337
1746554249	1964	1964	The Young Lovers	The Young Lovers	Goldwyn, Samuel	Samuel Goldwyn Jr.	tt0058763
1746508270	1925	1925	Win, Lose or Draw	Win, Lose or Draw	Maloney, Leo	Leo D. Maloney	tt0016539
1746474115	1948	1948	The Bold Frontiersman	The Bold Frontiersman	Ford, Philip	Philip Ford	tt0040180
1745302447	2005	2004	Jiminy Glick in Lalawood	Jiminy Glick in Lalawood	Jean, Vadim	Vadim Jean	tt0347369
1745340833	2003	2003	Malibu's Most Wanted	Malibu's Most Wanted	Whitesell, John	John Whitesell	tt0328099
1746580447	1937	1937	The Bride Wore Red	The Bride Wore Red	Arzner, Dorothy	Dorothy Arzner	tt0028661
1746566201	1956	1956	Runaway Daughters	Runaway Daughters	Cahn, Edward L.	Edward L. Cahn	tt0049697
1746394754	1981	1981	Pennies from Heaven	Pennies from Heaven	Ross, Herbert	Herbert Ross	tt0082894
1746507860	1927	1927	Splitting the Breeze	Splitting the Breeze	De Lacey, Robert	Robert De Lacey	tt0018437
1746455027	1996	1996	Michael Collins	Michael Collins	Jordan, Neil	Neil Jordan	tt0117039
1746581502	1960	1960	I Aim at the Stars	I Aim at the Stars	Thompson, J. Lee	J. Lee Thompson	tt0053440
1746437014	1943	1943	The Iron Major	The Iron Major	Enright, Ray	Ray Enright	tt0036044
1746491957	1946	1946	Courage of Lassie	Courage of Lassie	Wilcox, Fred M.	Fred M. Wilcox	tt0038427
1746394222	1990	1990	Nobody's Perfect	Nobody's Perfect	Kaylor, Robert	Robert Kaylor	tt0097994
1746583478	1931	1931	Dishonored	Dishonored	Sternberg, Josef von	Josef von Sternberg	tt0021800
1746573660	1931	1931	Forgotten Women	Forgotten Women	Thorpe, Richard	Richard Thorpe	tt0021882
1746555419	1955	1955	Hold Back Tomorrow	Hold Back Tomorrow	Haas, Hugo	Hugo Haas	tt0048174

1745357603	2008	2007	American Zombie	American Zombie	Lee, Grace	Grace Lee	tt0765430
1746438606	1945	1945	Adventures of Rusty	Adventures of Rusty	Burnford, Paul	Paul Burnford	tt0037496
1746503609	1918	1918	After the War	After the War	De Grasse, Joseph	Joseph De Grasse	tt0009863
1746520625	1925	1925	Hearts of the West	Hearts of the West			tt0245937
1746503952	1919	1919	The Gray Wolf's Ghost	The Gray Wolf's Ghost	Frame, Park	Park Frame	tt0011252
1746523726	1915	1915	The Model	The Model	Thomson, Frederick	Frederick A. Thomson	tt0005760
1746560110	1964	1964	The Lively Set	The Lively Set	Arnold, Jack	Jack Arnold	tt0058296
1746554320	1964	1964	Back Door to Hell	Back Door to Hell	Hellman, Monte	Monte Hellman	tt0057864
1746570742	1933	1933	Hold Me Tight	Hold Me Tight	Butler, David	David Butler	tt0024129
1746464763	1946	1946	Sentimental Journey	Sentimental Journey	Lang, Walter	Walter Lang	tt0038922
1746437240	1940	1939	Two-Fisted Rangers	Two Fisted Rangers	Lewis, Joseph H.	Joseph H. Lewis	tt0033195
1746584300	1935	1935	The Wedding Night	The Wedding Night	Vidor, King	King Vidor	tt0027189
1746492096	1944	1944	The Whistler	The Whistler	Castle, William	William Castle	tt0037461
1746514348	1914	1914	The Criminal Code	The Criminal Code		William Desmond Taylor	tt0003802
1746475513	1947	1947	Thunderbolt	Thunderbolt	Sturges, John; Wyler, William	John Sturges	tt0038171
1746584880	1939	1939	Trouble in Sundown	Trouble in Sundown	Howard, David	David Howard	tt0032060
1746453836	1943	1943	Let's Face It	Let's Face It	Lanfield, Sidney	Sidney Lanfield	tt0036105
1746499908	1923	1923	The Gentleman From America	The Gentleman from America	Sedgwick, Edward	Edward Sedgwick	tt0014078
1746525093	1919	1919	Marked Men	Marked Men	Ford, Jack	John Ford	tt0010438
1745302781	2006	2006	Material Girls	Material Girls	Coolidge, Martha	Martha Coolidge	tt0433412
1746524740	1910	1910	The Proposal	The Proposal	Powell, Frank	Frank Powell	tt0362994
1746198709	1970	1970	The Wizard of Gore	The Wizard of Gore	Lewis, Herschell Gordon	Herschell Gordon Lewis	tt0066578
1746585399	1939	1939	I Stole a Million	I Stole a Million	Tuttle, Frank	Frank Tuttle	tt0031465
1746508987	1949	1949	Brimstone	Brimstone	Kane, Joseph	Joseph Kane	tt0041210
1746512425	1916	1916	The Light	The Light	Dowlan, William C.	William C. Dowlan	tt0160469
1746524731	1917	1917	Fires of Rebellion	Fires of Rebellion	Park, Ida May	Ida May Park	tt0007950
1746465377	1942	1942	Yokel Boy	Yokel Boy	Santley, Joseph	Joseph Santley	tt0035581
1746501356	1915	1915	The Adventures of a Boy Scout	The Adventures of a Boy Scout	Warren, Edward	Edward Warren	tt0004859
1746423981	1940	1940	British Intelligence	British Intelligence	Morse, Terry	Terry O. Morse	tt0032283
1746570826	1952	1952	Oklahoma Annie	Oklahoma Annie	Springsteen, R. G.	R.G. Springsteen	tt0044988
1746508562	1926	1926	The Beautiful Cheat	The Beautiful Cheat	Sloman, Edward	Edward Sloman	tt0016635
1746581020	1910	1910	The Girl and the Fugitive	The Girl and the Fugitive		Gilbert M. 'Broncho Billy' Anderson	tt0001231
1745357732	2003	2003	Flesh for the Beast	Flesh for the Beast	West, Terry M.	Terry M. West	tt0372921
1746580334	1935	1935	Barbary Coast	Barbary Coast	Hawks, Howard	Howard Hawks	tt0026097
1746436544	1941	1941	Caught in the Draft	Caught in the Draft	Butler, David	David Butler	tt0033455
1746574317	1951	1951	The Blue Veil	The Blue Veil	Bernhardt, Curtis	Curtis Bernhardt	tt0043350
1746497681	1922	1922	The Sagebrush Trail	The Sagebrush Trail	Thornby, Robert T.	Robert Thornby	tt0013569
1746584707	1930	1930	Let's Go Places	Let's Go Places	Strayer, Frank	Frank R. Strayer	tt0021062
1746502938	1915	1915	The Valley of Lost Hope	The Valley of Lost Hope	Fielding, Romaine	Romaine Fielding	tt0006205
1746583008	1939	1939	The Lady and the Mob	The Lady and the Mob	Stoloff, Ben	Benjamin Stoloff	tt0031547
1746436195	1997	1997	Grosse Pointe Blank	Grosse Pointe Blank	Armitage, George	George Armitage	tt0119229
1746569721	1955	1955	Many Rivers to Cross	Many Rivers to Cross	Rowland, Roy	Roy Rowland	tt0048350
1746583741	1931	1931	Sunrise Trail	Sunrise Trail	McCarthy, J. P.	John P. McCarthy	tt0022450
1746580629	1937	1937	A Girl with Ideas	A Girl with Ideas	Simon, S. Sylvan	S. Sylvan Simon	tt0028932
1746496094	1919	1919	The Sundown Trail	The Sundown Trail	Sturgeon, Rollin	Rollin S. Sturgeon	tt0010745
1746582177	1930	1930	Slightly Scarlet	Slightly Scarlet	Gasnier, Louis; Knopf, Edwin H.	Louis J. Gasnier	tt0021393
1746573994	1959	1959	Go, Johnny, Go!	Go, Johnny, Go!	Landres, Paul	Paul Landres	tt0051665
1746583419	1929	1929	New Year's Eve	New Year's Eve	Lehrman, Henry	Henry Lehrman	tt0020216
1746436799	1997	1997	Contact	Contact	Zemeckis, Robert	Robert Zemeckis	tt0118884
1746497667	1920	1920	Firebrand Trevison	Firebrand Trevison	Heffron, Thomas N.	Thomas N. Heffron	tt0011182
1746408715	1987	1987	Blind Date	Blind Date	Edwards, Blake	Blake Edwards	tt0092666
1746560901	1932	1932	Isle of Paradise	Isle of Paradise	Trego, Charles T.	Charles T. Trego	tt2125530
1746555241	1956	1956	Running Target	Running Target	Weinstein, Marvin R.	Marvin R. Weinstein	tt0049698
1746516351	1921	1921	Rip Van Winkle	Rip Van Winkle	Lascelle, Ward	Edward Ludwig	tt0012627
1746483202	1917	1917	The Iced Bullet	The Iced Bullet	Barker, Reginald	Reginald Barker	tt0008131
1746423000	1999	1999	Anna and the King	Anna and the King	Tennant, Andy	Andy Tennant	tt0166485
1746410562	1984	1984	Rhinestone	Rhinestone	Clark, Bob	Bob Clark	tt0088001

1746523712	1916	1916	The Soul's Cycle	The Soul's Cycle	Davis, Ulysses	Ulysses Davis	tt0158981
1746515419	1920	1920	Human Collateral	Human Collateral	Windom, Lawrence C.	Lawrence C. Windom	tt0011315
1746503471	1921	1921	The Big Adventure	The Big Adventure	Eason, Reeves	B. Reeves Eason	tt0011966
1746436219	1992	1992	Candyman	Candyman	Rose, Bernard	Bernard Rose	tt0103919
1745293170	2007	2006	The Tripper	The Tripper	Arquette, David	David Arquette	tt0760187
1746513467	1914	1914	The Traitor	The Traitor	Weber, Lois; Smalley, Phillips	Phillips Smalley	tt0004724
1769971961	1969	1969	The Spy Who Came	The Spy Who Came	Wertheim, Ron	Ron Wertheim	tt0149101
1745272633	2001	2001	The Man Who Wasn't There	The Man Who Wasn't There	Coen, Joel	Joel Coen	tt0243133
1746524531	1915	1915	Mr. Grex of Monte Carlo	Mr. Grex of Monte Carlo	Reicher, Frank	Frank Reicher	tt0005781
1746436362	1941	1941	The Lady from Cheyenne	The Lady from Cheyenne	Lloyd, Frank	Frank Lloyd	tt0033807
1746581912	1951	1951	New Mexico	New Mexico	Reis, Irving	Irving Reis	tt0043849
1746583393	1931	1931	Little Caesar	Little Caesar	LeRoy, Mervyn	Mervyn LeRoy	tt0021079
1746583563	1930	1930	Caught Short	Caught Short	Reisner, Charles F.	Charles Reisner	tt0020749
1746500445	1926	1926	Footloose Widows	Footloose Widows	Del Ruth, Roy	Roy Del Ruth	tt0016893
1746453527	1941	1941	Father Takes a Wife	Father Takes a Wife	Hively, Jack	Jack Hively	tt0033592
1746577056	1959	1959	Porgy and Bess	Porgy and Bess	Preminger, Otto	Otto Preminger	tt0053182
1746198279	1967	1966	The Vulture	The Vulture	Huntington, Lawrence	Lawrence Huntington	tt0062463
1746423496	1995	1995	Blue in the Face	Blue in the Face	Wang, Wayne; Auster, Paul	Paul Auster	tt0112541
1746566000	1953	1953	The Tall Texan	The Tall Texan	Williams, Elmo	Elmo Williams	tt0046399
1746516128	1922	1922	They're Off	They're Off	Ford, Francis	Francis Ford	tt0169317
1746408681	1986	1986	True Stories	True Stories	Byrne, David	David Byrne	tt0092117
1746454687	1946	1946	The Killers	The Killers	Siodmak, Robert	Robert Siodmak	tt0038669
1746454439	1944	1943	Swing Out the Blues	Swing Out the Blues	St. Clair, Mal	Malcolm St. Clair	tt0037337
1746516507	1920	1920	The Man Who Had Everything	The Man Who Had Everything	Green, Alfred E.	Alfred E. Green	tt0012439
1746509554	1926	1925	The Lost Trail	The Lost Trail	McGowan, J. P.	J.P. McGowan	tt0264808
1746486127	1918	1918	With Neatness and Dispatch	With Neatness and Dispatch	Davis, William S.	Will S. Davis	tt0009821
1746574412	1963	1963	Yellowstone Cubs	Yellowstone Cubs		Charles L. Draper	tt0206446
1746492217	1945	1945	The Spider	The Spider	Webb, Robert	Robert D. Webb	tt0038110
1746410724	1992	1991	Where the Day Takes You	Where the Day Takes You	Rocco, Marc	Marc Rocco	tt0105810
1746584100	1936	1936	Cavalry	Cavalry	Bradbury, Robert N.	Robert N. Bradbury	tt0027436
1746577638	1937	1937	Old Louisiana	Old Louisiana	Willat, I. V.	Irvin Willat	tt0029338
1746520750	1925	1925	One of the Bravest	One of the Bravest	O'Connor, Frank	Frank O'Connor	tt0016183
1746520461	1926	1926	Almost a Lady	Almost a Lady	Hopper, E. Mason	E. Mason Hopper	tt0016597
1745393704	2009	2009	The Boondock Saints II: All Saints Day	The Boondock Saints II: All Saints Day	Duffy, Troy	Troy Duffy	tt1300851
1746584876	1939	1939	South of the Border	South of the Border	Sherman, George	George Sherman	tt0031957
1769969350	1964	1965	The Shocking Sex	Shocking Set!!?		Barry Mahon	tt2104085
1746583534	1931	1931	Possessed	Possessed		Clarence Brown	tt0022276
1746512411	1927	1927	The Fair Co-ed	The Fair Co Ed	Wood, Sam	Sam Wood	tt0017860
1745293833	2007	2007	Smiley Face	Smiley Face	Araki, Gregg	Gregg Araki	tt0780608
1746421056	1986	1986	TerrorVision	TerrorVision	Nicolaou, Ted	Ted Nicolaou	tt0092074
1746259530	1978	1978	Killer of Sheep	Killer of Sheep	Burnett, Charles	Charles Burnett	tt0076263
1746577083	1950	1950	Texas Dynamo	Texas Dynamo	Nazarro, Ray	Ray Nazarro	tt0043036
1746563794	1951	1951	Slaughter Trail	Slaughter Trail	Allen, Irving	Irving Allen	tt0044045
1745340821	2004	2004	Birth	Birth	Glazer, Jonathan	Jonathan Glazer	tt0337876
1746585067	1928	1928	Beware of Married Men	Beware of Married Men	Mayo, Archie L.	Archie Mayo	tt0018693
1746580974	1959	1959	Terror Is a Man	Terror Is a Man	De Leon, Gerry	Gerardo de Leon	tt0053344
1746583532	1930	1929	Seven Keys to Baldpate	Seven Keys to Baldpate	Barker, Reginald	Reginald Barker	tt0020388
1746492484	1945	1945	Divorce	Divorce	Nigh, William	William Nigh	tt0037645
1746584038	1935	1935	Fighting Caballero	Fighting Caballero	Clifton, Elmer	Elmer Clifton	tt0026349
1746496861	1917	1917	Her Soul's Inspiration	Her Soul's Inspiration	Conway, Jack	Jack Conway	tt0008083
1746497649	1920	1920	She Couldn't Help It	She Couldn't Help It	Campbell, Maurice	Maurice Campbell	tt0011684
1746566028	1953	1953	Robot Monster	Robot Monster	Tucker, Phil	Phil Tucker	tt0046248
1745341627	2001	2001	Get Over It	Get Over It	O'Haver, Tommy	Tommy O'Haver	tt0192071
1746507877	1926	1926	One Minute to Play	One Minute to Play	Wood, Sam	Sam Wood	tt0017233
1746467892	1917	1917	The Innocent Sinner	Innocent Sinners	Walsh, R. A.	Herman C. Raymaker	tt0330448
1746512749	1927	1927	Jake the Plumber	Jake the Plumber	Luddy, Edward I.	Edward Ludwig	tt0018035
1745339896	2004	2004	Raise Your Voice	Raise Your Voice	McNamara, Sean	Sean McNamara	tt0361696

1746500386	1925	1925	Stampede Thunder	Stampede Thunder	Gibson, Tom	Tom Gibson	tt0166815
1746250209	1978	1978	I Wanna Hold Your Hand	I Wanna Hold Your Hand	Zemeckis, Robert	Robert Zemeckis	tt0077714
1746501915	1915	1915	Tiger Bait	Tiger Bait	Chaudet, L. W.		tt1276431
1746582180	1928	1928	Broken Barriers	Broken Barriers	King, Burton	Burton L. King	tt0018724
1746235440	1971	1971	Honky	Honky	Graham, William A.	William A. Graham	tt0067214
1746514103	1910	1910	White-Doe's Lover	White Doe's Lovers		William F. Haddock	tt2236934
1746582474	1937	1937	52nd Street	52nd Street	Young, Harold	Harold Young	tt0028544
1746453946	1999	1999	October Sky	October Sky	Johnston, Joe	Joe Johnston	tt0132477
1746562448	1956	1956	The Brass Legend	The Brass Legend	Oswald, Gerd	Gerd Oswald	tt0049029
1746500730	1949	1949	The Doctor and the Girl	The Doctor and the Girl	Bernhardt, Curtis	Curtis Bernhardt	tt0041300
1746410628	1982	1982	The Toy	The Toy	Donner, Richard	Richard Donner	tt0084809
1746197869	1969	1969	Cactus Flower	Cactus Flower	Saks, Gene	Gene Saks	tt0064117
1746575058	1936	1936	Three Godfathers	Three Godfathers	Boleslawski, Richard	Richard Boleslawski	tt0028367
1746508765	1950	1950	Backfire	Backfire	Sherman, Vincent	Vincent Sherman	tt0042219
1745340781	2002	2002	The Mothman Prophecies	The Mothman Prophecies	Pellington, Mark	Mark Pellington	tt0265349
1746577163	1960	1960	Code of Silence	Code of Silence	Welles, Mel	Mel Welles	tt0050595
1746233963	1966	1966	Mondo Bizarro	Mondo Bizarro	Hakasomi, Seasu	Lee Frost	tt0188909
1746466882	1917	1917	Love or Justice	Love or Justice	Edwards, Walter	Walter Edwards	tt0178720
1746578978	1963	1963	In the Cool of the Day	In the Cool of the Day	Stevens, Robert	Robert Stevens	tt0057178
1769971126	1969	1969	Childish Things	Childish Things	Derek, John	John Derek	tt0068407
1746584692	1930	1930	Night Ride	The Night Ride	Robertson, John S.	John S. Robertson	tt0021183
1746184039	1910	1910	The Aspirations of Gerald and Percy	The Aspirations of Gerald and Percy			tt0398709
1746533055	1916	1916	The Gilded Cage	The Gilded Cage	Knoles, Harley	Harley Knoles	tt0006716
1746419598	1989	1989	Look Who's Talking	Look Who's Talking	Heckerling, Amy	Amy Heckerling	tt0097778
1746249514	1976	1976	Hollywood on Trial	Hollywood on Trial	Helpern, David	David Helpern	tt0074635
1746473713	1942	1942	Smart Alecks	Smart Alecks	Fox, Wallace	Wallace Fox	tt0035345
1746504565	1921	1921	The Ruse of the Rattler	The Ruse of the Rattler	McGowan, J. P.	J.P. McGowan	tt0012639
1746508489	1924	1924	Surging Seas	Surging Seas	Chapin, James	James Chapin	tt0015382
1746576967	1958	1958	Wolf Dog	Wolf Dog	Newfield, Sam	Sam Newfield	tt0052401
1746580577	1937	1937	Born Reckless	Born Reckless	St. Clair, Malcolm	Malcolm St. Clair	tt0028651
1746438487	1997	1997	Plan B	Plan B	Leva, Gary	Gary Leva	tt0119902
1746508125	1926	1926	The Cohens and Kellys	The Cohens and Kellys	Pollard, Harry	Harry A. Pollard	tt0016732
1746514285	1916	1916	The Turmoil	The Turmoil	Jones, Edgar	Edgar Jones	tt0159058
1745357912	2004	2004	When Will I Be Loved	When Will I Be Loved	Toback, James	James Toback	tt0396271
1746454466	1941	1941	World Premiere	World Premiere	Tetzlaff, Ted	Ted Tetzlaff	tt0034402
1746408498	1990	1990	The Two Jakes	The Two Jakes	Nicholson, Jack	Jack Nicholson	tt0100828
1746235810	1977	1977	New York, New York	New York, New York	Scorsese, Martin	Martin Scorsese	tt0076451
1745339970	2004	2004	Kill Bill -- Vol. 2	Kill Bill: Vol. 2	Tarantino, Quentin	Quentin Tarantino	tt0378194
1746501101	1915	1915	The Vanderhoff Affair	The Vanderhoff Affair	Vignola, Robert G.	Robert G. Vignola	tt0006207
1746554497	1965	1965	Goodbye in the Mirror	Goodbye in the Mirror	De Hirsch, Storm	Storm De Hirsch	tt0219708
1745309485	2005	2005	Fever Pitch	Fever Pitch	Farrelly, Peter; Farrelly, Bobby	Bobby Farrelly	tt0332047
1746465263	1945	1945	Out of the Depths	Out of the Depths	Lederman, D. Ross	D. Ross Lederman	tt0038817
1746524697	1916	1916	The Matrimaniac	The Matrimaniac	Powell, Paul	Paul Powell	tt0007047
1746394197	1988	1988	Another Woman	Another Woman	Allen, Woody	Woody Allen	tt0094663
1746574911	1935	1935	Two for Tonight	Two for Tonight	Tuttle, Frank	Frank Tuttle	tt0027147
1746566336	1958	1958	High School Hellcats	High School Hellcats	Bernds, Edward L.	Edward Bernds	tt0051725
1746581799	1928	1928	Ships of the Night	Ships of the Night	Worne, Duke	Duke Worne	tt0020400
1746466830	1917	1917	The Man from Montana	The Man from Montana	Marshall, George	George Marshall	tt0008262
1746563373	1952	1951	Birthright	Birthright	Clifford, Bill	Bill Clifford	tt0211259
1746584852	1929	1929	The College Coquette	The College Coquette	Archainbaud, George	George Archainbaud	tt0019781
1746585135	1930	1930	Way for a Sailor	Way for a Sailor		Sam Wood	tt0021534
1746504420	1919	1919	Hard Boiled	Hard Boiled	Schertzinger, Victor L.	Victor Schertzinger	tt0010209
1746580517	1938	1938	Going Places	Going Places	Enright, Ray	Ray Enright	tt0030190
1746567786	1933	1933	One Sunday Afternoon	One Sunday Afternoon	Roberts, Stephen	Stephen Roberts	tt0024414
1746550276	1954	1954	The Rocket Man	The Rocket Man	Rudolph, Oscar	Oscar Rudolph	tt0047423
1746499953	1923	1923	The Frame Up	The Frame Up	Moody, Harry	Harry Moody	tt0357670
1746579004	1959	1958	Intent to Kill	Intent to Kill	Cardiff, Jack	Jack Cardiff	tt0051781

1746465040	1944	1944	When the Lights Go on Again	When the Lights Go on Again	Howard, William K.	William K. Howard	tt0037459
1769970804	1912	1912	Cardinal Farley's Home-coming	Cardinal Farley's Home Coming			tt1300202
1745341991	2001	2001	ExtremeDays	Extremedays	Hannah, Eric	Eric Hannah	tt0245891
1746574921	1935	1935	Lawless Range	Lawless Range	Bradbury, R. N.	Robert N. Bradbury	tt0026614
1746454698	1944	1944	The Girl Who Dared	The Girl Who Dared	Bretherton, Howard	Howard Bretherton	tt0036866
1746465188	1944	1944	Code of the Prairie	Code of the Prairie	Bennet, Spencer	Spencer Gordon Bennet	tt0036717
1746514382	1917	1917	The Frozen Warning	The Frozen Warning	Eagle, Oscar	Oscar Eagle	tt0175625
1746513078	1922	1922	The Danger Point	The Danger Point	Ingraham, Lloyd	Lloyd Ingraham	tt0013045
1746514255	1923	1923	The Tie That Binds	The Tie That Binds	Levering, Joseph	Joseph Levering	tt0014546
1746235450	1972	1972	Children Shouldn't Play with Dead Things	Children Shouldn't Play with Dead Things	Clark, Benjamin	Bob Clark	tt0068370
1746502084	1917	1917	The Field of Honor	The Field of Honor	Holubar, Allen J.	Allen Holubar	tt0007939
1746493690	1910	1910	The Step-Daughter	The Step Daughter			tt1799143
1746581378	1951	1951	The Red Badge of Courage	The Red Badge of Courage	Huston, John	John Huston	tt0043961
1746409253	1990	1990	Pacific Heights	Pacific Heights	Schlesinger, John	John Schlesinger	tt0100318
1746567454	1952	1952	The Story of Will Rogers	The Story of Will Rogers	Curtiz, Michael	Michael Curtiz	tt0045198
1746532417	1910	1910	Transfusion	Transfusion		Harry Solter	tt0201268
1745373708	2009	2009	Shorts	Shorts	Rodriguez, Robert	Robert Rodriguez	tt1100119
1746419162	1940	1940	Who Killed Aunt Maggie?	Who Killed Aunt Maggie?	Lubin, Arthur	Arthur Lubin	tt0033257
1746564496	1952	1952	Sailor Beware	Sailor Beware	Walker, Hal	Hal Walker	tt0043989
1746504790	1918	1918	Fedora	Fedora	José, Edward	Edward José	tt0009060
1746492234	1947	1947	The Perils of Pauline	The Perils of Pauline	Marshall, George	George Marshall	tt0039698
1746567430	1932	1932	The Greeks Had a Word for Them	The Greeks Had a Word for Them	Sherman, Lowell	Lowell Sherman	tt0022961
1746520700	1925	1925	Daddy's Gone A-Hunting	Daddy's Gone A Hunting	Borzage, Frank	Frank Borzage	tt0015722
1746200203	1969	1969	Gaily, Gaily	Gaily, Gaily	Jewison, Norman	Norman Jewison	tt0064357
1746524064	1923	1923	Refuge	Refuge	Schertzinger, Victor	Victor Schertzinger	tt0014402
1746570327	1933	1933	Double Harness	Double Harness	Cromwell, John	John Cromwell	tt0023960
1746492373	1944	1944	Lumberjack	Lumberjack	Selander, Lesley	Lesley Selander	tt0037030
1746210044	1971	1971	Big Jake	Big Jake	Sherman, George	George Sherman	tt0066831
1746574362	1958	1958	Separate Tables	Separate Tables	Mann, Delbert	Delbert Mann	tt0052182
1746436237	1997	1997	Boogie Nights	Boogie Nights	Anderson, Paul Thomas	Paul Thomas Anderson	tt0118749
1746520388	1927	1927	Roaring Fires	Roaring Fires	Barringer, A. B.	Barry Barringer	tt0018333
1746585432	1939	1939	Torchy Runs for Mayor	Torchy Runs for Mayor	McCarey, Ray	Ray McCarey	tt0032045
1746436580	1940	1940	Hullabaloo	Hullabaloo	Marin, Edwin L.	Edwin L. Marin	tt0032614
1746503018	1927	1927	What Price Love	What Price Love?	Revier, Harry	Harry Revier	tt0018560
1745339959	2004	2004	The Princess Diaries 2: Royal Engagement	The Princess Diaries 2: Royal Engagement	Marshall, Garry	Garry Marshall	tt0368933
1746422912	1994	1993	Romeo Is Bleeding	Romeo Is Bleeding	Medak, Peter	Peter Medak	tt0107983
1746438607	1943	1943	The Kid Rides Again	The Kid Rides Again	Scott, Sherman	Sam Newfield	tt0036074
1746555468	1957	1957	Slim Carter	Slim Carter	Bartlett, Richard H.	Richard Bartlett	tt0050983
1746532819	1916	1916	The Black Sheep of the Family	The Black Sheep of the Family	Hunt, Jay	Jay Hunt	tt0006435
1745374144	2004	2003	Overnight	Overnight	Smith, Mark Brian ; Montana, Tony	Tony Montana	tt0390336
1746580921	1962	1962	Phaedra	Phaedra	Dassin, Jules	Jules Dassin	tt0056346
1746532999	1923	1923	Flames of Passion	Flames of Passion	Moody, H. G.	Harry Moody	tt0013132
1746218933	1977	1977	The Bad News Bears in Breaking Training	The Bad News Bears in Breaking Training	Pressman, Michael	Michael Pressman	tt0075718
1746491984	1946	1946	Bowery Bombshell	Bowery Bombshell	Karlson, Phil	Phil Karlson	tt0038378
1745342257	2004	2004	The Girl Next Door	The Girl Next Door	Greenfield, Luke	Luke Greenfield	tt0265208
1745293607	2004	2004	Word Wars	Word Wars	Chaikin, Eric ; Petrillo, Julian	Eric Chaikin	tt0390632
1746570609	1934	1934	Twentieth Century	Twentieth Century	Hawks, Howard	Howard Hawks	tt0025919
1746437422	1941	1941	The Devil and Miss Jones	The Devil and Miss Jones	Wood, Sam	Sam Wood	tt0033533
1746422920	1992	1992	In the Soup	In the Soup	Rockwell, Alexandre	Alexandre Rockwell	tt0104503
1746524629	1916	1916	The Wrong Door	The Wrong Door	DeHaven, Carter	Carter DeHaven	tt0007595
1746435696	1940	1940	Haunted House	Haunted House	McGowan, Robert	Robert F. McGowan	tt0032572
1746513075	1923	1923	The Mailman	The Mailman	Johnson, Emory	Emory Johnson	tt0014238
1746580617	1935	1935	Champagne for Breakfast	Champagne for Breakfast	Brown, Melville	Melville W. Brown	tt0026194
1746422130	1996	1996	A Family Thing	A Family Thing	Pearce, Richard	Richard Pearce	tt0116275
1746455915	1996	1996	Bulletproof	Bulletproof	Dickerson, Ernest	Ernest R. Dickerson	tt0115783
1746454505	1941	1941	Dangerously They Live	Dangerously They Live	Florey, Robert	Robert Florey	tt0033512
1746258238	1977	1977	The Happy Hooker Goes to Washington	The Happy Hooker Goes to Washington	Levey, William A.	William A. Levey	tt0076125

1746578750	1959	1959	The Wreck of the Mary Deare	The Wreck of the Mary Deare	Anderson, Michael	Michael Anderson	tt0053455
1746256144	1979	1979	When a Stranger Calls	When a Stranger Calls	Walton, Fred	Fred Walton	tt0080130
1746487582	1920	1920	The Golden Trail	The Golden Trail	Moomaw, L. H.; Hersholt, Jean	Jean Hersholt	tt0012229
1746582750	1939	1939	Jesse James	Jesse James	King, Henry	Henry King	tt0031507
1746422576	1993	1992	How U Like Me Now	How U Like Me Now	Roberts, Darryl	Darryl Roberts	tt0107154
1746582772	1935	1935	Frisco Waterfront	Frisco Waterfront	Lubin, Arthur	Arthur Lubin	tt0026386
1746582653	1938	1938	The Painted Trail	The Painted Trail	Hill, Robert	Robert F. Hill	tt0030549
1746509493	1924	1924	The Moral Sinner	The Moral Sinner	Ince, Ralph	Ralph Ince	tt0015149
1746581279	1950	1950	Storm Over Wyoming	Storm Over Wyoming	Selander, Lesley	Lesley Selander	tt0043003
1746421057	1991	1991	Blood in the Face	Blood in the Face	Bohlen, Anne; Rafferty, Kevin; Ridgev	Anne Bohlen	tt0101479
1746486832	1919	1919	Her Kingdom of Dreams	Her Kingdom of Dreams	Neilan, Marshall	Marshall Neilan	tt0010242
1746235503	1974	1974	Cockfighter	Cockfighter	Hellman, Monte	Monte Hellman	tt0071338
1746410382	1985	1985	Pale Rider	Pale Rider	Eastwood, Clint	Clint Eastwood	tt0089767
1746235773	1977	1977	You Light Up My Life	You Light Up My Life	Brooks, Joseph	Joseph Brooks	tt0076941
1746516338	1920	1920	Husbands and Wives	Husbands and Wives	Levering, Joseph	Joseph Levering	tt0195812
1746495608	1919	1919	Eve in Exile	Eve in Exile	George, Burton	Burton George	tt0190385
1746574814	1936	1936	Song of the Trail	Song of the Trail	Hopton, Russell	Russell Hopton	tt0028285
1746566345	1953	1953	Bright Road	Bright Road	Mayer, Gerald	Gerald Mayer	tt0045578
1746437357	1941	1941	Life Begins for Andy Hardy	Life Begins for Andy Hardy	Seitz, George B.	George B. Seitz	tt0033832
1746524898	1915	1915	The Glory of Youth	The Glory of Youth	Ellis, Robert	Robert Ellis	tt0005398
1746554958	1964	1964	A House Is Not a Home	A House Is Not a Home	Rouse, Russell	Russell Rouse	tt0058209
1746504910	1920	1920	For Love or Money	For Love or Money	King, Burton	Burton L. King	tt0156967
1746514547	1916	1916	The Martyrdom of Philip Strong	The Martyrdom of Philip Strong	Ridgely, Richard	Richard Ridgely	tt0163094
1746513830	1922	1922	The Face Between	The Face Between	Veiller, Bayard	Bayard Veiller	tt0013108
1746513457	1910	1910	Trailed to the Hills	Trailed to the Hills	Anderson, G. M.	Gilbert M. 'Broncho Billy' Anderson	tt0324304
1746497272	1922	1922	Tess of the Storm Country	Tess of the Storm Country	Robertson, John S.	John S. Robertson	tt0013662
1746409439	1983	1983	D.C. Cab	D.C. Cab	Schumacher, Joel	Joel Schumacher	tt0085387
1746501367	1910	1910	The Song That Reached His Heart	The Song That Reached His Heart	Dawley (?), J. Searle	J. Searle Dawley	tt1499694
1746509549	1924	1924	Trucker's Top Hand	Trucker's Top Hand	Hart, Neal	Neal Hart	tt0174286
1746438431	1942	1942	Boss of Hangtown Mesa	Boss of Hangtown Mesa	Lewis, Joseph H.	Joseph H. Lewis	tt0034544
1746524684	1917	1917	Hinton's Double	Hinton's Double	Warde, Ernest	Lloyd Lonergan	tt0175736
1746563374	1952	1952	Meet Danny Wilson	Meet Danny Wilson	Pevney, Joseph	Joseph Pevney	tt0044893
1745395368	2009	2009	Julie & Julia	Julie & Julia	Ephron, Nora	Nora Ephron	tt1135503
1746566204	1953	1953	Laughing Anne	Laughing Anne	Wilcox, Herbert	Herbert Wilcox	tt0045989
1746577561	1933	1933	Parole Girl	Parole Girl	Cline, Edward	Edward F. Cline	tt0024429
1746582049	1930	1930	Playboy of Paris	Playboy of Paris	Berger, Ludwig	Ludwig Berger	tt0021261
1746464811	1945	1945	Incendiary Blonde	Incendiary Blonde	Marshall, George	George Marshall	tt0037816
1746574818	1935	1935	Behind the Green Lights	Behind the Green Lights	Cabanne, Christy	Christy Cabanne	tt0026106
1746509462	1925	1925	Queen of Spades	Queen of Spades	Fraser, Harry L.	Harry L. Fraser	tt0016255
1746465021	1947	1947	Law of the Lash	Law of the Lash	Taylor, Ray	Ray Taylor	tt0039559
1746499934	1948	1948	Waterfront at Midnight	Waterfront at Midnight	Berke, William	William Berke	tt0040951
1746574104	1963	1963	Hallelujah the Hills	Hallelujah the Hills	Mekas, Adolfas	Adolfas Mekas	tt0056053
1746500606	1950	1950	It's a Small World	It's a Small World	Castle, William	William Castle	tt0042608
1746583735	1931	1931	Personal Maid	Personal Maid	Bell, Monta	Monta Bell	tt0022262
1746582983	1940	1940	Sky Bandits	Sky Bandits	Staub, Ralph	Ralph Staub	tt0033058
1746410179	1981	1981	Buddy Buddy	Buddy Buddy	Wilder, Billy	Billy Wilder	tt0082111
1746198091	1968	1968	Love Is Where It's At	Love Is Where It's At	Knight, Sidney	Sidney Knight	tt0125861
1746577864	1936	1936	Kelly of the Secret Service	Kelly of the Secret Service	Hill, Bob	Robert F. Hill	tt0027839
1746436374	1943	1943	Devil Riders	Devil Riders	Newfield, Sam	Sam Newfield	tt0035804
1746434604	1999	1999	Outside Providence	Outside Providence	Corrente, Michael	Michael Corrente	tt0125971
1746520963	1950	1950	The Missourians	The Missourians	Blair, George	George Blair	tt0042741
1746581800	1910	1910	His Sick Friend	His Sick Friend		Harry Solter	tt0200705
1746420207	1981	1981	Neighbors	Neighbors	Avildsen, John G.	John G. Avildsen	tt0082801
1746437424	1941	1941	Stick to Your Guns	Stick to Your Guns	Selander, Lesley	Lesley Selander	tt0034233
1746509021	1924	1924	Troubles of a Bride	Troubles of a Bride	Buckingham, Thomas	Tom Buckingham	tt0016458
1746580692	1937	1937	Black Legion	Black Legion	Mayo, Archie L.	Archie Mayo	tt0027367
1746530762	1950	1950	Last of the Buccaneers	Last of the Buccaneers	Landers, Lew	Lew Landers	tt0042666

1746508060	1926	1926	Poker Faces	Poker Faces	Pollard, Harry A.	Harry A. Pollard	tt0017272
1746554765	1965	1965	The Love Goddesses	The Love Goddesses		Saul J. Turell	tt0059402
1746523455	1916	1916	The Parson of Panamint	The Parson of Panamint	Taylor, William D.	William Desmond Taylor	tt0007154
1746423347	1993	1993	Point of No Return	Point of No Return	Badham, John	John Badham	tt0107843
1746580480	1939	1939	Midnight	Midnight	Leisen, Mitchell	Mitchell Leisen	tt0031647
1746465100	1943	1943	They Got Me Covered	They Got Me Covered	Butler, David	David Butler	tt0036427
1746500342	1925	1925	Ridin' Easy	Ridin' Easy	Hayes, Ward	Ward Hayes	tt0156954
1746500504	1923	1923	Human Wreckage	Human Wreckage	Wray, John Griffith	John Griffith Wray	tt0014141
1746501369	1917	1917	Babbling Tongues	Babbling Tongues	Humphrey, William J.	William Humphrey	tt0167013
1746578438	1960	1960	The Savage Eye	The Savage Eye	Maddow, Ben; Meyers, Sidney; Strick	Ben Maddow	tt0054270
1746583086	1939	1939	I'm from Missouri	I'm from Missouri	Reed, Theodore	Theodore Reed	tt0031469
1746582752	1939	1939	Code of the Fearless	Code of the Fearless	Johnson, Raymond K.	Bernard B. Ray	tt0031165
1746508907	1925	1925	The Denial	The Denial	Henley, Hobart	Hobart Henley	tt0015743
1746484717	1917	1917	The Greater Law	The Greater Law	Reynolds, Lynn F.	Lynn Reynolds	tt0008032
1746500308	1925	1925	Goat Getter	Goat Getter	Rogell, Albert	Albert S. Rogell	tt0133807
1746394027	1986	1986	Crossroads	Crossroads	Hill, Walter	Walter Hill	tt0090888
1746580880	1934	1934	Girl in Danger	Girl in Danger	Lederman, D. Ross	D. Ross Lederman	tt0025174
1746584451	1930	1930	Good News	Good News	Grinde, Nick	Nick Grinde	tt0020929
1746554532	1964	1964	Viva Las Vegas	Viva Las Vegas	Sidney, George	George Sidney	tt0058725
1746542646	1926	1926	Racewild	Race Wild	Apfel, Oscar	Oscar Apfel	tt0017302
1746574966	1937	1937	Wells Fargo	Wells Fargo	Lloyd, Frank	Frank Lloyd	tt0029752
1746502866	1916	1916	Charity	Charity	Powell, Frank	Frank Powell	tt0157456
1746520393	1924	1924	The Ridin' Kid From Powder River	The Ridin' Kid from Powder River	Sedgwick, Edward	Edward Sedgwick	tt0015279
1746584814	1930	1930	Animal Crackers	Animal Crackers	Heerman, Victor	Victor Heerman	tt0020640
1746199491	1969	1969	House of the Red Dragon	The House of the Red Dragon	Donne, John	Donn Greer	tt0220542
1746524673	1922	1922	Flaming Hearts	Flaming Hearts	Elfelt, Clifford S.	Clifford S. Elfelt	tt0132959
1746500510	1927	1927	The Show Girl	The Show Girl	Hunt, Charles J.	Charles J. Hunt	tt0134097
1746549953	1954	1954	A Bullet Is Waiting	A Bullet Is Waiting	Farrow, John	John Farrow	tt0046812
1745357624	2010	2010	Countdown to Zero	Countdown to Zero	Walker, Lucy	Lucy Walker	tt1572769
1746524560	1914	1914	Aristocracy	Aristocracy	Heffron, T. N.	Thomas N. Heffron	tt0003631
1746424374	1940	1940	The Wildcat of Tucson	The Wildcat of Tucson	Hillyer, Lambert	Lambert Hillyer	tt0034395
1746580196	1936	1936	The Country Beyond	The Country Beyond	Forde, Eugene	Eugene Forde	tt0027467
1746524643	1915	1915	The Magic Skin	The Magic Skin	Ridgely, Richard	Richard Ridgely	tt0005685
1746582479	1939	1939	Main Street Lawyer	Main Street Lawyer	Murphy, Dudley	Dudley Murphy	tt0031607
1746454958	1941	1941	The Body Disappears	The Body Disappears	Lederman, D. Ross	D. Ross Lederman	tt0033414
1746393988	1988	1988	Mystic Pizza	Mystic Pizza	Petrie, Donald	Donald Petrie	tt0095690
1746464891	1941	1941	The Great Train Robbery	The Great Train Robbery	Kane, Joseph	Joseph Kane	tt0033680
1746584343	1940	1940	Arizona	Arizona	Ruggles, Wesley	Wesley Ruggles	tt0032221
1746581723	1928	1928	The Singapore Mutiny	The Singapore Mutiny	Ince, Ralph	Ralph Ince	tt0164889
1746492244	1948	1947	Dangerous Years	Dangerous Years	Pierson, Arthur	Arthur Pierson	tt0040267
1746580235	1938	1938	Garden of the Moon	Garden of the Moon	Berkeley, Busby	Busby Berkeley	tt0030171
1746524255	1915	1915	Crooky	Crooky	Williams, C. Jay	C.J. Williams	tt0005159
1746583544	1951	1951	Al Jennings of Oklahoma	Al Jennings of Oklahoma	Nazarro, Ray	Ray Nazarro	tt0043272
1746574340	1959	1959	T-Bird Gang	T Bird Gang	Harbinger, Richard	Richard Harbinger	tt0163315
1746501843	1915	1915	Jimmy	Jimmy	Oliver, Guy		tt1268230
1746455885	1999	1999	8MM	8MM	Schumacher, Joel	Joel Schumacher	tt0134273
1746503806	1918	1918	Good-Bye, Bill	Good Bye, Bill	Emerson, John	John Emerson	tt0010185
1746566146	1954	1954	Challenge the Wild	Challenge the Wild	Graham, Frank A.	Frank A. Graham	tt0186915
1746585395	1940	1940	Remember the Night	Remember the Night	Leisen, Mitchell	Mitchell Leisen	tt0032981
1746533035	1928	1928	Manhattan Cowboy	Manhattan Cowboy	McGowan, J. P.	J.P. McGowan	tt0019137
1746585292	1928	1928	Lonesome	Lonesome	Fejos, Paul	Pál Fejös	tt0019109
1746473680	1945	1945	Duffy's Tavern	Duffy's Tavern	Walker, Hal	Hal Walker	tt0037662
1746524491	1922	1922	False Fronts	False Fronts	Bradley, Samuel R.	Samuel R. Bradley	tt0013115
1746580623	1938	1938	Come On, Leathernecks	Come On, Leathernecks!	Cruze, James	James Cruze	tt0030005
1746567802	1932	1932	Afraid to Talk	Afraid to Talk	Cahn, Edward L.	Edward L. Cahn	tt0022612
1746574969	1934	1934	All of Me	All of Me	Flood, James	James Flood	tt0024822
1746583557	1929	1929	The Dance of Life	The Dance of Life	Cromwell, John; Sutherland, A. Edwa	John Cromwell	tt0019798

1746577606	1936	1936	The Big Broadcast of 1937	The Big Broadcast of 1937	Leisen, Mitchell	Mitchell Leisen	tt0027356
1746500196	1950	1950	Kansas Raiders	Kansas Raiders	Enright, Ray	Ray Enright	tt0042629
1746560515	1932	1932	Partners	Partners	Allen, Fred	Fred Allen	tt0023321
1746423303	1999	1998	The Taxman	Taxman	Nesher, Avi	Avi Nesher	tt0138862
1745359123	2003	2003	Terminator 3: Rise of the Machines	Terminator 3: Rise of the Machines	Mostow, Jonathan	Jonathan Mostow	tt0181852
1746516080	1921	1921	Pilgrims of the Night	Pilgrims of the Night	Sloman, Edward	Edward Sloman	tt0012567
1769973326	1970	1969	The Master Piece	The Master Piece!	Van Horn, Lee	Lee Van Horn	tt0379374
1746467884	1917	1917	The Spirit of Romance	The Spirit of Romance	Hopper, E. Mason	E. Mason Hopper	tt0182445
1746492481	1944	1944	One Body Too Many	One Body Too Many	McDonald, Frank	Frank McDonald	tt0037151
1746576620	1931	1931	High Stakes	High Stakes	Sherman, Lowell	Lowell Sherman	tt0021959
1746581626	1928	1928	Riley of the Rainbow Division	Riley of the Rainbow Division	Ray, Robert	Bobby Ray	tt0156963
1746420399	1991	1991	Dead Again	Dead Again	Branagh, Kenneth	Kenneth Branagh	tt0101669
1746584877	1940	1940	The Ghost Breakers	The Ghost Breakers	Marshall, George	George Marshall	tt0032520
1746234788	1968	1968	Psych-Out	Psych Out	Rush, Richard	Richard Rush	tt0063469
1746465157	1947	1947	Dick Tracy Meets Gruesome	Dick Tracy Meets Gruesome	Rawlins, John	John Rawlins	tt0039320
1746218992	1975	1974	The Yakuza	The Yakuza	Pollack, Sydney	Sydney Pollack	tt0073918
1746434924	1995	1995	Jupiter's Wife	Jupiter's Wife	Negroponte, Michel	Michel Negroponte	tt0110217
1746585241	1929	1929	Marianne	Marianne	Leonard, Robert Z.	Robert Z. Leonard	tt0020143
1746582910	1936	1936	Ceiling Zero	Ceiling Zero	Hawks, Howard	Howard Hawks	tt0026191
1746570989	1934	1934	No Contest!	No Contest!	Henabery, Joseph	Joseph Henabery	tt0026783
1746419922	1988	1988	The Naked Gun: From the Files of Police Squad!	The Naked Gun: From the Files of Police Squ	Zucker, David	David Zucker	tt0095705
1746393560	1981	1981	Ragtime	Ragtime	Forman, Milos	Milos Forman	tt0082970
1746569967	1958	1958	Badman's Country	Badman's Country	Sears, Fred F.	Fred F. Sears	tt0051394
1746577655	1935	1935	Folies Bergère de Paris	Folies Bergère de Paris	Del Ruth, Roy	Roy Del Ruth	tt0026373
1746565214	1952	1952	The Winning Team	The Winning Team	Seiler, Lewis	Lewis Seiler	tt0045332
1746420047	1993	1993	Fatal Instinct	Fatal Instinct	Reiner, Carl	Carl Reiner	tt0106873
1746583918	1930	1930	Peacock Alley	Peacock Alley	De Sano, Marcel	Marcel De Sano	tt0021243
1746582744	1935	1935	Going Highbrow	Going Highbrow	Florey, Robert	Robert Florey	tt0026420
1746410760	1982	1982	Richard Pryor Live on the Sunset Strip	Richard Pryor: Live on the Sunset Strip	Layton, Joe	Joe Layton	tt0084597
1746453567	1943	1943	Confessions of a Vice Baron	Confessions of a Vice Baron	Melville, John	S. Roy Luby	tt0173742
1746484164	1919	1919	The Spirit of Lafayette	The Spirit of Lafayette	Vincent, James	James Vincent	tt0008618
1746584037	1938	1938	Man's Country	Man's Country	Hill, Robert	Robert F. Hill	tt0030411
1745359571	2001	2001	Down to Earth	Down to Earth	Weitz, Chris; Weitz, Paul	Chris Weitz	tt0231775
1746569743	1957	1957	The Land Unknown	The Land Unknown	Vogel, Virgil	Virgil W. Vogel	tt0050622
1746583863	1930	1930	Lord Byron of Broadway	Lord Byron of Broadway	Nigh, William; Beaumont, Harry	Harry Beaumont	tt0021090
1746520570	1926	1926	Devil's Dice	Devil's Dice	Forman, Tom	Tom Forman	tt0016784
1746513800	1912	1912	A Physician's Honor	The Physician's Honor			tt0358021
1746393962	1992	1992	Prelude to a Kiss	Prelude to a Kiss	René, Norman	Norman René	tt0105165
1746500254	1926	1926	The Road to Mandalay	The Road to Mandalay	Browning, Tod	Tod Browning	tt0017329
1746502285	1916	1916	Insomnia	Insomnia	Metcalfe, Earl	Earl Metcalfe	tt0483668
1746574816	1937	1937	Arizona Days	Arizona Days	English, John	John English	tt0028582
1746582033	1928	1928	Prowlers of the Sea	Prowlers of the Sea	Adolfi, John G.	John G. Adolfi	tt0019296
1746580771	1935	1935	Diamond Jim	Diamond Jim	Sutherland, A. Edward	A. Edward Sutherland	tt0026277
1746487370	1920	1920	The Last Straw	The Last Straw	Clift, Denison	Denison Clift	tt0011386
1746580314	1938	1938	Six Shootin' Sheriff	Six Shootin' Sheriff	Fraser, Harry	Harry L. Fraser	tt0030757
1746455290	1995	1995	Man of the House	Man of the House	Orr, James	James Orr	tt0113755
1746497579	1920	1920	Down Home	Down Home	Willat, Irvin V.	Irvin Willat	tt0011127
1746577605	1934	1934	I Believed in You	I Believed in You	Cummings, Irving	Irving Cummings	tt0025280
1746424196	1943	1943	Assignment in Brittany	Assignment in Brittany	Conway, Jack	Jack Conway	tt0035651
1746584505	1928	1928	Domestic Meddlers	Domestic Meddlers	Flood, James	James Flood	tt0018842
1746578381	1961	1961	Lover Come Back	Lover Come Back	Mann, Delbert	Delbert Mann	tt0055100
1746474538	1944	1944	Forty Thieves	Forty Thieves	Selander, Lesley	Lesley Selander	tt0036835
1746464939	1944	1944	The Climax	The Climax	Waggner, George	George Waggner	tt0036715
1746504154	1917	1917	The Woman and the Beast	The Woman and the Beast	Warde, Ernest C.	Ernest C. Warde	tt0176328
1746393546	1983	1983	All the Right Moves	All the Right Moves	Chapman, Michael	Michael Chapman	tt0085154
1746579055	1960	1960	The Bramble Bush	The Bramble Bush	Petrie, Daniel	Daniel Petrie	tt0053673
1746554478	1964	1964	Flight From Ashiya	Flight from Ashiya	Anderson, Michael	Michael Anderson	tt0058104

1746577004	1950	1950	Young Man with a Horn	Young Man with a Horn	Curtiz, Michael	Michael Curtiz	tt0043153	
1746392760	1984	1984	Ninja III: The Domination	Ninja III: The Domination	Firstenberg, Sam	Sam Firstenberg	tt0087805	
1746581433	1910	1910	A Gambler of the West	A Gambler of the West		Gilbert M. 'Broncho Billy' Anderson	tt0001228	
1746424220	1942	1941	"Kukan": The Battle Cry of China	'Kukan': The Battle Cry of China			tt0121453	
1746524327	1923	1923	At Devil's Gorge	At Devil's Gorge	Dearholt, Ashton	Ashton Dearholt	tt0013844	
1746584949	1939	1939	Death Goes North	Death Goes North	McDonald, Frank	Frank McDonald	tt0031220	
1746582941	1939	1939	They Made Me a Criminal	They Made Me a Criminal	Berkeley, Busby	Busby Berkeley	tt0032022	
1746580524	1935	1935	Pals of the Range	Pals of the Range	Clifton, Elmer	Elmer Clifton	tt0026843	
1769971121	1969	1969	The Secret Life of Hernando Cortez	The Secret Life of Hernando Cortez	Chamberlain, John	John Chamberlain	tt0064951	
1746584082	1935	1935	Trail of Terror	Trail of Terror	Bradbury, Robert N.	Robert N. Bradbury	tt0027128	
1746569899	1958	1958	From Hell to Texas	From Hell to Texas	Hathaway, Henry	Henry Hathaway	tt0051636	
1746513068	1927	1927	Wild Beauty	Wild Beauty	MacRae, Henry	Henry MacRae	tt0018575	
1746574483	1934	1934	Two Alone	Two Alone	Nugent, Elliott	Elliott Nugent	tt0025923	
1746500705	1925	1925	The Way of a Girl	The Way of a Girl	Vignola, Robert G.	Robert G. Vignola	tt0016505	
1746584158	1939	1939	Honolulu	Honolulu	Buzzell, Edward	Edward Buzzell	tt0031442	
1746571336	1952	1952	The Las Vegas Story	The Las Vegas Story	Stevenson, Robert	Robert Stevenson	tt0044825	
1746573929	1962	1962	The Couch	The Couch	Crump, Owen	Owen Crump	tt0055870	
1746576933	1962	1961	Forbid Them Not	Forbid Them Not	Kimble, Robert L.	Robert L. Kimble	tt0054888	
1745341997	2001	2001	Enemy at the Gates	Enemy at the Gates	Annaud, Jean-Jacques	Jean	tt0215750	
1746530689	1950	1950	Cow Town	Cow Town	English, John	John English	tt0042349	
1745340824	2003	2003	The Lizzie McGuire Movie	The Lizzie McGuire Movie	Fall, Jim	Jim Fall	tt0306841	
1746576695	1931	1931	The Naughty Flirt	The Naughty Flirt	Cline, Edward	Edward F. Cline	tt0022191	
1746569815	1957	1956	The Wrong Man	The Wrong Man	Hitchcock, Alfred	Alfred Hitchcock	tt0051207	
1746520382	1925	1925	Wild Horse Canyon	Wild Horse Canyon		Ben F. Wilson	tt0016533	
1746501943	1915	1915	Heléne of the North	Helene of the North	Dawley, J. Searle	J. Searle Dawley	tt0005464	
1746420100	1988	1988	The Presidio	The Presidio	Hyams, Peter	Peter Hyams	tt0095897	
1746562126	1957	1958	Half Human	Half Human	G. Crane, Kenneth	Kenneth G. Crane	tt0050480	
1746494004	1914	1914	Absinthe	Absinthe	Brenon, Herbert	Herbert Brenon	tt0003591	
1746424618	1943	1943	Cabin in the Sky	Cabin in the Sky	Minnelli, Vincente	Vincente Minnelli	tt0035703	
1746210807	1970	1969	Latitude Zero	Latitude Zero	Honda, Ishiro	Ishirô Honda	tt0064470	
1746582795	1939	1939	Exile Express	Exile Express	Garrett, Otis	Otis Garrett	tt0031288	
1746530777	1949	1949	Rio Grande	Rio Grande	Sheldon, Norman	Norman Sheldon	tt0244170	
1746585159	1929	1929	Girls Gone Wild	Girls Gone Wild	Seiler, Lewis	Lewis Seiler	tt0019931	
1746582818	1936	1936	The Magnificent Brute	Magnificent Brute	Blystone, John	John G. Blystone	tt0027930	
1746577203	1960	1960	Walk Tall	Walk Tall	Dexter, Maury	Maury Dexter	tt0054458	
1769977804	1985	1985	The Heavenly Kid	The Heavenly Kid	Medoway, Cary	Cary Medoway	tt0089265	
1746577526	1937	1937	Nation Aflame	Nation Aflame	Halperin, Victor	Victor Halperin	tt0029296	
1746474501	1945	1945	Tonight and Every Night	Tonight and Every Night	Saville, Victor	Victor Saville	tt0038178	
1746433960	1998	1998	There's Something About Mary	There's Something About Mary	Farrelly, Bobby; Farrelly, Peter	Bobby Farrelly	tt0129387	
1746184114	1910	1910	The Burlesque Queen	The Burlesque Queen		Joseph A. Golden	tt0291057	
1746485589	1920	1920	The Trail of the Cigarette	The Trail of the Cigarette	Collins, Tom	Tom Collins	tt0166850	
1746467923	1917	1917	The Butterfly Girl	The Butterfly Girl	Otto, Henry	Henry Otto	tt0168555	
1746509262	1925	1925	The Coast Patrol	The Coast Patrol	Barsky, Bud	Bud Barsky	tt0015691	
1746574987	1937	1937	Yodelin' Kid from Pine Ridge	Yodelin' Kid from Pine Ridge	Kane, Joe	Joseph Kane	tt0029801	
1746571736	1931	1931	Hush Money	Hush Money	Lanfield, Sidney	Sidney Lanfield	tt0021984	
1745372817	2009	2009	Year One	Year One	Ramis, Harold	Harold Ramis	tt1045778	
1746487905	1920	1920	Alarm Clock Andy	Alarm Clock Andy	Storm, Jerome	Jerome Storm	tt0126187	
1746211260	1973	1973	Bang the Drum Slowly	Bang the Drum Slowly	Hancock, John	John D. Hancock	tt0069765	
**1746582954**	**1938**	**1938**	**Blockade**	**Blockade**	**Dieterle, William**	**William Dieterle**	**tt0029924**	1
1746565970	1953	1953	Scared Stiff	Scared Stiff	Marshall, George	George Marshall	tt0046280	
1745282544	2006	2006	Shadow: Dead Riot	Shadow: Dead Riot	Wan, Derek	Derek Wan	tt0427848	
1746208212	1969	1969	The Hang-Up	Hang Up	Hayes, John	Joseph Marzano	tt0298355	
1746555898	1956	1956	The Amazon Trader	The Amazon Trader	McGowan, Tom	Tom McGowan	tt0143102	
1746523218	1916	1916	The Lure of Heart's Desire	The Lure of Heart's Desire	Grandon, Francis J.	Francis J. Grandon	tt0006992	
1746582825	1939	1939	You Can't Cheat an Honest Man	You Can't Cheat an Honest Man	Marshall, George	George Marshall	tt0032152	
1746465407	1947	1947	Stallion Road	Stallion Road	Kern, James V.	James V. Kern	tt0039865	
1746483911	1917	1917	The Eternal Mother	The Eternal Mother	Reicher, Frank	Frank Reicher	tt0007899	

1746486856	1920	1920	The Butterfly Man	The Butterfly Man	Park, Ida May	Louis J. Gasnier	tt0011041
1746485100	1918	1918	The Hired Man	The Hired Man	Schertzinger, Victor L.	Victor Schertzinger	tt0009177
1746235689	1976	1976	No Way Back	No Way Back	Williamson, Fred	Fred Williamson	tt0074969
1746514241	1916	1916	Just a Song at Twilight	Just a Song at Twilight	King, Carlton S.	Burton L. King	tt0013285
1746584794	1929	1929	Bye-bye Buddy	Bye, Bye, Buddy	Mattison, Frank S.	Frank S. Mattison	tt0019740
1746475772	1946	1946	Queen of Burlesque	Queen of Burlesque	Newfield, Sam	Sam Newfield	tt0038864
1746454040	1944	1944	Seven Doors to Death	Seven Doors to Death	Clifton, Elmer	Elmer Clifton	tt0037262
1746520389	1926	1926	So's Your Old Man	So's Your Old Man	La Cava, Gregory	Gregory La Cava	tt0017410
1746584541	1931	1931	Mother and Son	Mother and Son	McCarthy, John P.	John P. McCarthy	tt0022165
1746500298	1925	1925	Lord Jim	Lord Jim	Fleming, Victor	Victor Fleming	tt0016037
1746501495	1922	1922	The Bootleggers	The Bootleggers	Sheldon, Roy	Roy Sheldon	tt0012964
1745374589	2010	2010	Machete	Machete	Rodriguez, Robert; Maniquis, Ethan	Ethan Maniquis	tt0985694
1746581976	1930	1930	Lummox	Lummox	Brenon, Herbert	Herbert Brenon	tt0021105
1746410869	1991	1991	Ricochet	Ricochet	Mulcahy, Russell	Russell Mulcahy	tt0102789
1746492715	1946	1946	The Best Years of Our Lives	The Best Years of Our Lives	Wyler, William	William Wyler	tt0036868
1746578505	1951	1950	The Daltons' Women	The Daltons' Women	Carr, Thomas	Thomas Carr	tt0042374
1746409050	1986	1986	What Happened to Kerouac?	What Happened to Kerouac?	Lerner, Richard; MacAdams, Lewis	Richard Lerner	tt0090312
1746198504	1968	1968	Anzio	Anzio	Dmytryk, Edward	Edward Dmytryk	tt0062673
1746561755	1955	1954	The Country Girl	The Country Girl	Seaton, George	George Seaton	tt0046874
1746582677	1936	1936	Her Master's Voice	Her Master's Voice	Santley, Joseph	Joseph Santley	tt0027734
1746492738	1946	1946	One More Tomorrow	One More Tomorrow	Godfrey, Peter; Rapper, Irving	Peter Godfrey	tt0038802
1746569762	1954	1954	Francis Joins the WACS	Francis Joins the WACS	Lubin, Arthur	Arthur Lubin	tt0046995
1746554538	1965	1965	Second Fiddle to a Steel Guitar	Second Fiddle to a Steel Guitar	Duncan, Victor	Victor Duncan	tt0059692
1746453804	1942	1942	A Tornado in the Saddle	A Tornado in the Saddle	Berke, William	William Berke	tt0035457
1746453623	1945	1945	I Love a Mystery	I Love a Mystery	Levin, Henry	Henry Levin	tt0037803
1746421366	1940	1940	Frontier Crusader	Frontier Crusader	Stewart, Peter	Sam Newfield	tt0032497
1746513853	1923	1923	Regeneration	Regeneration		Richard E. Norman	tt0130218
1746198258	1969	1969	Heaven With a Gun	Heaven with a Gun	Katzin, Lee H.	Lee H. Katzin	tt0064409
1746520908	1949	1949	Arctic Manhunt	Arctic Manhunt	Scott, Ewing	Ewing Scott	tt0041132
1746569754	1953	1953	The System	The System	Seiler, Lewis	Lewis Seiler	tt0046389
1746583520	1951	1951	Man from Sonora	Man from Sonora	Collins, Lewis	Lewis D. Collins	tt0043779
1746570338	1953	1952	Androcles and the Lion	Androcles and the Lion	Erskine, Chester	Chester Erskine	tt0044355
1746421200	1997	1997	Switchback	Switchback	Stuart, Jeb	Jeb Stuart	tt0119210
1746584647	1929	1929	The Broadway Melody	The Broadway Melody	Beaumont, Harry	Harry Beaumont	tt0019729
1746492387	1946	1946	Mantan Messes Up	Mantan Messes Up		Sam Newfield	tt0135537
1746554353	1964	1964	The NEW Interns	The New Interns	Rich, John	John Rich	tt0058398
1746515586	1918	1918	The Claw	The Claw	Vignola, Robert G.	Robert G. Vignola	tt0008966
1746566060	1957	1956	Three Violent People	Three Violent People	Maté, Rudolph	Rudolph Maté	tt0049849
1746578407	1960	1959	Virgin Sacrifice	Virgin Sacrifice	Wagner, Fernando	Fernando Wagner	tt0188273
1746520279	1926	1926	Frenzied Flames	Frenzied Flames	Paton, Stuart	Stuart Paton	tt0133780
1746508741	1927	1927	The Lone Rider	The Lone Rider		Fred J. Balshofer	tt0492142
1746423698	1940	1940	Ski Patrol	Ski Patrol	Landers, Lew	Lew Landers	tt0033056
1746433561	1995	1995	Panther	Panther	Van Peebles, Mario	Mario Van Peebles	tt0114084
1746474957	1946	1946	The Catman of Paris	The Catman of Paris	Selander, Lesley	Lesley Selander	tt0038402
1745340265	2002	2001	Mule Skinner Blues	Mule Skinner Blues	Earnhart, Stephen	Stephen Earnhart	tt0290010
1746499886	1923	1923	The Girl I Loved	The Girl I Loved	De Grasse, Joseph	Joseph De Grasse	tt0014083
1746584151	1940	1940	Primrose Path	Primrose Path	La Cava, Gregory	Gregory La Cava	tt0032946
1746492057	1947	1947	The Macomber Affair	The Macomber Affair	Korda, Zoltan	Zoltan Korda	tt0039591
1746520948	1949	1949	Call of the Forest	Call of the Forest	Link, John F.	John F. Link Sr.	tt0041221
1746424394	1942	1942	Bad Men of the Hills	Bad Men of the Hills	Berke, William	William Berke	tt0034489
1746455316	1999	1999	Beyond the Mat	Beyond the Mat	Blaustein, Barry W.	Barry W. Blaustein	tt0218043
1746520838	1925	1925	The Fear Fighter	The Fear Fighter	Rogell, Albert	Albert S. Rogell	tt0131390
1746503757	1921	1921	Out of the Depths	Out of the Depths	Thayer, Otis B.; Reicher, Frank	Frank Reicher	tt0012536
1746566372	1954	1954	Can Can Follies	Can Can Follies			tt0134425
1746554212	1963	1963	Charade	Charade	Donen, Stanley	Stanley Donen	tt0056923
1746583415	1930	1930	What a Man	What a Man	Crone, George J.	George Crone	tt0021544
1746574380	1963	1963	Lonnie	Lonnie	Hale, William	William Hale	tt0240688

1746524858	1923	1923	Making Good	Making Good	Perez, Marcel	Marcel Perez	tt0266742
1746515010	1923	1923	East Side--West Side	East Side West Side	Cummings, Irving	Irving Cummings	tt0014012
1746393669	1982	1982	Rocky III	Rocky III	Stallone, Sylvester	Sylvester Stallone	tt0084602
1746185839	1966	1966	A Big Hand for the Little Lady	A Big Hand for the Little Lady	Cook, Fielder	Fielder Cook	tt0060165
1746583792	1930	1930	The Second Floor Mystery	The Second Floor Mystery	Del Ruth, Roy	Roy Del Ruth	tt0021351
1769971930	1969	1969	Detention Girls	The Detention Girls		John Rappoport	tt0197403
1746501875	1914	1914	The Wolf	The Wolf	O'Neil, Barry	Barry O'Neil	tt0004829
1746201703	1970	1970	Max-Out	Max Out	Kaylor, Robert	Robert Kaylor	tt0251241
1746500221	1924	1924	Crashin' Through	Crashin' Through	Neitz, Alvin J.	Alan James	tt0014806
1746569801	1957	1957	The Ride Back	The Ride Back	Miner, Allen H.	Allen H. Miner	tt0050899
1746571598	1931	1931	Daughter of the Dragon	Daughter of the Dragon	Corrigan, Lloyd	Lloyd Corrigan	tt0021785
1746520377	1926	1926	Silence	Silence	Julian, Rupert	Rupert Julian	tt0017385
1746217802	1966	1966	Lupe	Lupe	Warhol, Andy	Andy Warhol	tt0414248
1746574983	1937	1937	The Barrier	The Barrier	Selander, Lesley	Lesley Selander	tt0028611
1746234239	1966	1966	Boy, Did I Get a Wrong Number!	Boy, Did I Get a Wrong Number!	Marshall, George	George Marshall	tt0060186
1746571332	1934	1934	The Little Minister	The Little Minister	Wallace, Richard	Richard Wallace	tt0025409
1746438736	1995	1995	Tommy Boy	Tommy Boy	Segal, Peter	Peter Segal	tt0114694
1746500288	1926	1926	The High Hand	The High Hand	Maloney, Leo	Leo D. Maloney	tt0016966
1746582372	1930	1930	Embarrassing Moments	Embarrassing Moments	Craft, William James	William James Craft	tt0020853
1746424207	1941	1941	Tobacco Road	Tobacco Road	Ford, John	John Ford	tt0034297
1746501673	1916	1916	A Gutter Magdalene	A Gutter Magdalene	Melford, George H.	George Melford	tt0006749
1746574897	1933	1933	Neighbors' Wives	Neighbors' Wives	Eason, Breezy	B. Reeves Eason	tt0155909
1746566878	1933	1933	Professional Sweetheart	Professional Sweetheart	Seiter, William A.	William A. Seiter	tt0024476
1746467126	1917	1917	Mother O' Mine	Mother o' Mine	Julian, Rupert	Rupert Julian	tt0008325
1746520903	1949	1949	Undertow	Undertow	Castle, William	William Castle	tt0042007
1746509183	1948	1948	Joe Palooka in Winner Take All	Joe Palooka in Winner Take All	LeBorg, Reginald	Reginald Le Borg	tt0040493
1746455879	2000	2000	Requiem for a Dream	Requiem for a Dream	Aronofsky, Darren	Darren Aronofsky	tt0180093
1746577580	1934	1934	Red Morning	Red Morning	Fox, Wallace	Wallace Fox	tt0026918
1746577811	1938	1938	The Lady in the Morgue	The Lady in the Morgue	Garrett, Otis	Otis Garrett	tt0030342
1746520473	1950	1950	David Harding, Counterspy	David Harding, Counterspy	Nazarro, Ray	Ray Nazarro	tt0178372
1746574599	1938	1938	Walking Down Broadway	Walking Down Broadway	Foster, Norman	Norman Foster	tt0030953
1746542677	1926	1926	Hell-Bent fer Heaven	Hell Bent for Heaven	Blackton, J. Stuart	J. Stuart Blackton	tt0016955
1746454765	1999	1999	Three Kings	Three Kings	Russell, David O.	David O. Russell	tt0120188
1746563909	1952	1952	The Cimarron Kid	The Cimarron Kid	Boetticher, Budd	Budd Boetticher	tt0043408
1746569554	1953	1953	The Sun Shines Bright	The Sun Shines Bright	Ford, John	John Ford	tt0046384
1746574633	1933	1933	Baby Face	Baby Face	Green, Alfred E.	Alfred E. Green	tt0023775
1746465052	1946	1946	Breakfast in Hollywood	Breakfast in Hollywood	Schuster, Harold	Harold D. Schuster	tt0038382
1746574343	1960	1960	The High Powered Rifle	The High Powered Rifle	Dexter, Maury	Maury Dexter	tt0053910
1746453762	1943	1943	Minesweeper	Minesweeper	Berke, William	William Berke	tt0036161
1769976191	1921	1921	Free and Easy	Free and Easy	Kerr, Bob	Jack White	tt0248020
1746503864	1921	1921	The Millionaire	The Millionaire	Conway, Jack	Jack Conway	tt0012460
1746580788	1937	1937	Moonlight on the Range	Moonlight on the Range	Newfield, Sam	Sam Newfield	tt0029259
1746511849	1915	1915	Through Turbulent Waters	Through Turbulent Waters	McRae, Duncan	Duncan McRae	tt0006159
1746571581	1932	1932	Night World	Night World	Henley, Hobart	Hobart Henley	tt0023271
1746581091	1951	1951	Sirocco	Sirocco	Bernhardt, Curtis	Curtis Bernhardt	tt0044040
1769971959	1969	1969	Girls in the Saddle	Girls in the Saddle	Mauro, Ralph	Raf Mauro	tt0219051
1746503838	1918	1918	The Winding Trail	The Winding Trail	Collins, John H.	John H. Collins	tt0009814
1746530735	1949	1948	Force of Evil	Force of Evil	Polonsky, Abraham	Abraham Polonsky	tt0040366
1746454829	1941	1941	Henry Aldrich for President	Henry Aldrich for President	Bennett, Hugh	Hugh Bennett	tt0033708
1746492437	1947	1947	Pursued	Pursued	Walsh, Raoul	Raoul Walsh	tt0039737
1746203407	1970	1971	Erika's Hot Summer	Erika's Hot Summer	Graver, Gary	Gary Graver	tt0065701
1746504348	1918	1918	The Scarlet Trail	The Scarlet Trail	Lawrence, John S.	John S. Lawrence	tt0009582
1746235516	1978	1978	American Hot Wax	American Hot Wax	Mutrux, Floyd	Floyd Mutrux	tt0077158
1746580761	1936	1936	I'd Give My Life	I'd Give My Life	Marin, Edwin L.	Edwin L. Marin	tt0027784
1746577869	1935	1935	Valley of Wanted Men	Valley of Wanted Men	James, Alan	Alan James	tt0027165
1745342844	2003	2003	Spy Kids 3D: Game Over	Spy Kids 3: Game Over	Rodriguez, Robert	Robert Rodriguez	tt0338459
1746513879	1922	1922	The Deuce of Spades	The Deuce of Spades	Ray, Charles	Charles Ray	tt0013067

1746562098	1953	1953	The Marshal's Daughter	The Marshal's Daughter	Berke, William	William Berke	tt0046050	
1746492206	1944	1944	Sunday Dinner for a Soldier	Sunday Dinner for a Soldier	Bacon, Lloyd	Lloyd Bacon	tt0037326	
1746474464	1945	1945	Her Lucky Night	Her Lucky Night	Lilley, Edward	Edward C. Lilley	tt0037770	
1745359439	2002	2002	Martin Lawrence Live: Runteldat	Martin Lawrence Live: Runteldat	Raynr, David	David Raynr	tt0327036	
1746583153	1935	1935	Air Hawks	Air Hawks	Rogell, Albert	Albert S. Rogell	tt0026047	
1746542674	1924	1924	Merton of the Movies	Merton of the Movies	Cruze, James	James Cruze	tt0015131	
1746569970	1957	1957	Looking for Danger	Looking for Danger	Jewell, Austen	Austen Jewell	tt0050654	
1746575011	1936	1936	Dodsworth	Dodsworth	Wyler, William	William Wyler	tt0027532	
1746555715	1956	1956	The Swan	The Swan	Vidor, Charles	Charles Vidor	tt0049815	
1746553554	1964	1964	The Luck of Ginger Coffey	The Luck of Ginger Coffey	Kershner, Irvin	Irvin Kershner	tt0058305	
1745340130	2001	2001	Rat Race	Rat Race	Zucker, Jerry	Jerry Zucker	tt0250687	
1746524008	1928	1928	The Little Yellow House	The Little Yellow House	Meehan, James Leo	James Leo Meehan	tt0019107	
1746583868	1930	1930	The Dude Wrangler	The Dude Wrangler	Thorpe, Richard	Richard Thorpe	tt0020846	
1746235415	1974	1974	The Front Page	The Front Page	Wilder, Billy	Billy Wilder	tt0071524	
1746583892	1929	1929	Broadway Babies	Broadway Babies	LeRoy, Mervyn	Mervyn LeRoy	tt0019726	
1746394223	1992	1992	Mo' Money	Mo' Money	Macdonald, Peter	Peter MacDonald	tt0104897	
1746532718	1927	1927	The Devil's Masterpiece	The Devil's Masterpiece	McCarthy, John P.	John P. McCarthy	tt0123037	
1746583578	1930	1930	Doughboys	Doughboys	Sedgwick, Edward	Edward Sedgwick	tt0020838	
1746474407	1948	1948	Frontier Agent	Frontier Agent	Hillyer, Lambert	Lambert Hillyer	tt0040374	
1745341006	2001	2000	Sordid Lives	Sordid Lives	Shores, Del	Del Shores	tt0204640	
1746583843	1930	1930	Beau Bandit	Beau Bandit	Hillyer, Lambert	Lambert Hillyer	tt0020672	
1746235330	1975	1975	The Day of the Locust	The Day of the Locust	Schlesinger, John	John Schlesinger	tt0072848	
1746524942	1915	1915	Rule G	Rule G	Lawrence, George W.; Noble, G. M.	George W. Lawrence	tt0005985	
1746487124	1918	1918	The Street of Seven Stars	The Street of Seven Stars	O'Brien, John B.	John B. O'Brien	tt0190044	
1746585498	1940	1939	The Invisible Killer	The Invisible Killer	Scott, Sherman	Sam Newfield	tt0032634	
1746410200	1987	1987	Shy People	Shy People	Konchalovsky, Andrei	Andrey Konchalovskiy	tt0093964	
1746465005	1942	1942	Along the Sundown Trail	Along the Sundown Trail	Stewart, Peter	Sam Newfield	tt0034453	
1746569738	1953	1953	The War of the Worlds	The War of the Worlds	Haskin, Byron	Byron Haskin	tt0046534	
1746563859	1951	1951	Snake River Desperadoes	Snake River Desperadoes	Sears, Fred F.	Fred F. Sears	tt0044052	
1746578390	1950	1951	Two Lost Worlds	Two Lost Worlds	Dawn, Norman	Norman Dawn	tt0043080	
1746581061	1958	1958	Kings Go Forth	Kings Go Forth	Daves, Delmer	Delmer Daves	tt0051819	
1746574008	1962	1962	Brushfire!	Brushfire	Warner, Jack	Jack Warner Jr.	tt0055813	
1745282625	2007	2007	Hot Rod	Hot Rod	Schaffer, Akiva	Akiva Schaffer	tt0787475	
1746574986	1937	1937	Danger--Love at Work	Danger Love at Work	Preminger, Otto	Otto Preminger	tt0028761	
1746502141	1915	1915	It's No Laughing Matter	It's No Laughing Matter	Weber, Lois	Lois Weber	tt0005556	
**1746509222**	**1924**	**1924**	**The Lover of Camille**	**The Lover of Camille**	**Beaumont, Harry**	**Harry Beaumont**	**tt0015091**	1
1746509345	1950	1950	The Killer That Stalked New York	The Killer That Stalked New York	McEvoy, Earl	Earl McEvoy	tt0042643	
1745293351	2005	2005	The Honeymooners	The Honeymooners	Schultz, John	John Schultz	tt0373908	
1746500025	1945	1945	Voice of the Whistler	Voice of the Whistler	Castle, William	William Castle	tt0038230	
1746497260	1917	1917	A Song of Sixpence	A Song of Sixpence	Dean, Ralph	Ralph Dean	tt0182436	
1746492214	1945	1945	The Navajo Trail	The Navajo Trail	Bretherton, Howard	Howard Bretherton	tt0037940	
1746464793	1942	1942	Pittsburgh	Pittsburgh	Seiler, Lewis	Lewis Seiler	tt0035195	
1746465187	1947	1947	Dragnet	Dragnet	Goodwins, Leslie	Leslie Goodwins	tt0039339	
1746578791	1963	1963	Playgirls International	Playgirls International	Wishman, Doris	Doris Wishman	tt0187408	
1746578417	1959	1959	The Miracle	The Miracle	Rapper, Irving	Irving Rapper	tt0053069	
1746567797	1933	1933	Roman Scandals	Roman Scandals	Tuttle, Frank	Frank Tuttle	tt0024507	
1746500512	1924	1924	Husbands and Lovers	Husbands and Lovers	Stahl, John M.	John M. Stahl	tt0015005	
1769971168	1969	1969	Fulfillment, Something Worth Remembering	Fulfillment, Something Worth Rememberin	Conner, Charles M.	Charles M. Conner	tt0218990	
1746520324	1925	1925	With This Ring	With This Ring	Windermere, Fred	Fred Windemere	tt0180308	
1746282176	1979	1979	Alien	Alien	Scott, Ridley	Ridley Scott	tt0078748	
1746569741	1955	1955	Queen Bee	Queen Bee	MacDougall, Ranald	Ranald MacDougall	tt0048527	
1746520496	1926	1926	Ace of Action	Ace of Action	Bertram, William	William Bertram	tt0016571	
1746423976	1941	1941	Two in a Taxi	Two in a Taxi	Florey, Robert	Robert Florey	tt0034327	
1746513822	1923	1923	The Spanish Dancer	The Spanish Dancer	Brenon, Herbert	Herbert Brenon	tt0014499	
1746496287	1918	1918	The Pen Vulture	The Pen Vulture		William H. Clifford	tt0187397	
1746410622	1988	1988	Shoot to Kill	Shoot to Kill	Spottiswoode, Roger	Roger Spottiswoode	tt0096098	
1746584026	1936	1936	Ride 'Em Cowboy	Ride 'Em Cowboy	Selander, Leslie	Lesley Selander	tt0028180	

1746197925	1969	1969	The Desperados	The Desperados	Levin, Henry	Henry Levin	tt0064227
1746216962	1967	1967	The Glory Stompers	The Glory Stompers	Lanza, Anthony M.	Anthony M. Lanza	tt0061714
1746473939	1947	1947	Code of the West	Code of the West	Berke, William	William Berke	tt0039271
1745373769	2009	2009	Public Enemies	Public Enemies	Mann, Michael	Michael Mann	tt1152836
1746497602	1921	1921	A Broken Doll	A Broken Doll	Dwan, Allan	Allan Dwan	tt0012002
1746509357	1926	1926	Eyes Right	Eyes Right!	Chaudet, Louis	Louis Chaudet	tt0130676
1746578424	1951	1951	David and Bathsheba	David and Bathsheba	King, Henry	Henry King	tt0043455
1746569704	1958	1958	Dunkirk	Dunkirk	Norman, Leslie	Leslie Norman	tt0051565
1746454664	1945	1945	This Love of Ours	This Love of Ours	Dieterle, William	William Dieterle	tt0038162
1746495798	1917	1917	Polly of the Circus	Polly of the Circus	Horan, Charles T.; Hollywood, Edwin	Edwin L. Hollywood	tt0008442
1746492421	1945	1945	Jealousy	Jealousy	Machaty, Gustav	Gustav Machatý	tt0037829
1746424391	1940	1940	Strike Up the Band	Strike Up the Band	Berkeley, Busby	Busby Berkeley	tt0033110
1745342927	2008	2008	The Midnight Meat Train	The Midnight Meat Train	Kitamura, Ryuhei	Ryûhei Kitamura	tt0805570
1746235928	1978	1978	The Last Waltz	The Last Waltz	Scorsese, Martin	Martin Scorsese	tt0077838
1746523085	1910	1910	Out for Mischief	Out for Mischief		Gaston Méliès	tt0361994
1746580538	1936	1936	The Plot Thickens	The Plot Thickens	Holmes, Ben	Ben Holmes	tt0028111
1746578456	1960	1960	Freckles	Freckles	McLaglen, Andrew V.	Andrew V. McLaglen	tt0053837
1746502570	1913	1913	The Prisoner of Zenda	The Prisoner of Zenda	Porter, Edwin S.	Hugh Ford	tt0003301
1746487917	1918	1918	Cupid by Proxy	Cupid by Proxy	Bertram, William	William Bertram	tt0007831
1746583088	1939	1939	The Star Maker	The Star Maker	Del Ruth, Roy	Roy Del Ruth	tt0031974
1746453763	1945	1945	Hangover Square	Hangover Square	Brahm, John	John Brahm	tt0037761
1746577037	1959	1959	The Trap	The Trap	Panama, Norman	Norman Panama	tt0053374
1746581411	1928	1928	Saddle Mates	Saddle Mates	Thorpe, Richard	Richard Thorpe	tt0019343
1746582339	1928	1928	Buckskin Days	Buckskin Days	Fabian, Walter	Walter Fabian	tt0333193
1746581698	1928	1928	Broadway Daddies	Broadway Daddies	Windemere, Fred	Fred Windemere	tt0018723
1746410878	1994	1994	Blue Sky	Blue Sky	Richardson, Tony	Tony Richardson	tt0109306
1746218880	1977	1977	MacArthur	MacArthur	Sargent, Joseph	Joseph Sargent	tt0076342
1746520213	1926	1926	The Galloping Cowboy	The Galloping Cowboy	Craft, William J.	William James Craft	tt0016912
1746499849	1948	1948	Berlin Express	Berlin Express	Tourneur, Jacques	Jacques Tourneur	tt0040155
1746199246	1969	1969	Change of Mind	Change of Mind	Stevens, Robert	Robert Stevens	tt0064151
1746560344	1964	1964	Under Age	Under Age	Buchanan, Larry	Larry Buchanan	tt0057621
1746508058	1948	1948	The Mozart Story	The Mozart Story		Karl Hartl	tt0040612
1746583468	1931	1931	Always Goodbye	Always Goodbye	Menzies, William C.; MacKenna, Ken	Kenneth MacKenna	tt0021604
1746409486	1991	1991	Barton Fink	Barton Fink	Coen, Joel	Joel Coen	tt0101410
1746208282	1967	1967	The President's Analyst	The President's Analyst	Flicker, Theodore J.	Theodore J. Flicker	tt0062153
1746566003	1956	1956	The Flaming Teenage	The Flaming Teenage	Yeaworth, Irvin S.; Edwards, Charles	Charles Edwards	tt0125750
1746511766	1914	1914	The Valley of the Moon	The Valley of the Moon	Bosworth, Hobart	Hobart Bosworth	tt0004757
1746574715	1936	1936	Valley of the Lawless	Valley of the Lawless	Bradbury, Robert N.	Robert N. Bradbury	tt0028456
1746584234	1935	1935	Under Pressure	Under Pressure	Walsh, Raoul	Raoul Walsh	tt0027154
1746520702	1925	1925	The Demon Rider	The Demon Rider	Hurst, Paul	Paul Hurst	tt0015742
1746515044	1916	1916	Mrs. Dane's Danger	Mrs. Dane's Danger	North, Wilfrid	Wilfrid North	tt0163112
1746523576	1927	1927	Wandering Girls	Wandering Girls	Ince, Ralph	Ralph Ince	tt0018546
1746491966	1947	1947	Reet, Petite and Gone	Reet, Petite, and Gone	Crouch, William Forest	William Forest Crouch	tt0039759
1746515012	1914	1914	The Pawn of Fortune	The Pawn of Fortune	Wharton, Leopold	Leopold Wharton	tt0004459
1746584772	1929	1929	Love, Live and Laugh	Love, Live and Laugh	Howard, William K.	William K. Howard	tt0020118
1746393624	1983	1983	The Final Terror	The Final Terror	Davis, Andrew	Andrew Davis	tt0082379
1746500566	1926	1926	Alaskan Adventures	Alaskan Adventures	Robertson, Jack	Jack Robertson	tt0377347
1746580829	1939	1939	Winner Take All	Winner Take All	Brower, Otto	Otto Brower	tt0032131
1746562182	1956	1956	The Lieutenant Wore Skirts	The Lieutenant Wore Skirts	Tashlin, Frank	Frank Tashlin	tt0049443
1746584202	1938	1938	Personal Secretary	Personal Secretary	Garrett, Otis	Otis Garrett	tt0030574
1745357933	2008	2008	Harold & Kumar Escape from Guantanamo Bay	Harold & Kumar Escape from Guantanamo I	Hurwitz, Jon ; Schlossberg, Hayden	Jon Hurwitz	tt0481536
1745358064	2007	2007	Who Is Norman Lloyd?	Who Is Norman Lloyd?	Sussman, Matthew	Matthew Sussman	tt1159595
1746515740	1918	1918	Cyclone Higgins, D.D.	Cyclone Higgins, D.D.	Cabanne, William Christy	Christy Cabanne	tt0008987
1746583046	1935	1935	Maybe It's Love	Maybe It's Love	McGann, William	William C. McGann	tt0026691
1745282875	2006	2006	Saw III	Saw III	Bousman, Darren Lynn	Darren Lynn Bousman	tt0489270
1746574952	1937	1937	Boots of Destiny	Boots of Destiny	Rosson, Arthur	Arthur Rosson	tt0028647
1745360021	2001	2001	Startup.com	Startup.com	Noujaim, Jehane; Hegedus, Chris	Chris Hegedus	tt0256408

1745282653	2006	2006	Idlewild	Idlewild	Barber, Bryan	Bryan Barber	tt0417225
1745342792	2009	2008	Two Lovers	Two Lovers	Gray, James	James Gray	tt1103275
1746573506	1932	1932	Hot Saturday	Hot Saturday	Seiter, William	William A. Seiter	tt0023028
1746562386	1954	1954	The Outcast	The Outcast	Witney, William	William Witney	tt0047320
1746566199	1957	1957	The Brothers Rico	The Brothers Rico	Karlson, Phil	Phil Karlson	tt0050213
1746570975	1952	1952	Bal Tabarin	Bal Tabarin	Ford, Philip	Philip Ford	tt0044394
1746491725	1945	1945	Cornered	Cornered	Dmytryk, Edward	Edward Dmytryk	tt0037615
1746583200	1929	1929	Fast Life	Fast Life	Dillon, John Francis	John Francis Dillon	tt0019872
1746578757	1958	1958	The Toughest Gun in Tombstone	The Toughest Gun in Tombstone	Bellamy, Earl	Earl Bellamy	tt0052312
1746549850	1954	1954	The World Dances	The World Dances	Peters, Brooke L.	Boris Petroff	tt0131140
1746577698	1935	1935	Scream in the Night	A Scream in the Night	Newmeyer, Fred	Fred C. Newmeyer	tt0026971
1746508573	1926	1926	The Combat	The Combat	Reynolds, Lynn	Lynn Reynolds	tt0016736
1746208298	1969	1969	Changes	Changes	Bartlett, Hall	Hall Bartlett	tt0064152
1746410683	1985	1985	Clue	Clue	Lynn, Jonathan	Jonathan Lynn	tt0088930
1746408503	1988	1988	Working Girl	Working Girl	Nichols, Mike	Mike Nichols	tt0096463
1745272529	2000	2000	Miss Congeniality	Miss Congeniality	Petrie, Donald	Donald Petrie	tt0212346
1746584576	1928	1928	Bitter Sweets	Bitter Sweets	Hutchison, Charles	Charles Hutchison	tt0018702
1745303230	2007	2006	The Sasquatch Gang	The Sasquatch Gang	Skousen, Tim	Tim Skousen	tt0460925
1745282688	2005	2005	Sex Sells: The Making of Touche	Sex Sells: The Making of 'Touché'	Liebert, Jonathan	Jonathan Liebert	tt0372401
1746580590	1935	1935	The Old Homestead	The Old Homestead	Nigh, William	William Nigh	tt0026810
1746570320	1952	1952	Paula	Paula	Maté, Rudolph	Rudolph Maté	tt0045016
1746520928	1950	1950	The Iroquois Trail	The Iroquois Trail	Karlson, Phil	Phil Karlson	tt0042602
1769970495	1966	1966	Banned	Banned		Jerry Denby	tt0212800
1746578651	1961	1961	Master of the World	Master of the World	Witney, William	William Witney	tt0055152
1745272498	2000	2000	Snow Day	Snow Day	Koch, Chris	Chris Koch	tt0184907
1746491997	1947	1947	That Hagen Girl	That Hagen Girl	Godfrey, Peter	Peter Godfrey	tt0039892
1746508720	1949	1949	The Fighting Redhead	The Fighting Redhead	Collins, Lewis D.	Lewis D. Collins	tt0179809
1746578812	1961	1961	The Beast of Yucca Flats	The Beast of Yucca Flats	Francis, Coleman	Coleman Francis	tt0054673
1746465289	1942	1942	Once Upon a Honeymoon	Once Upon a Honeymoon	McCarey, Leo	Leo McCarey	tt0035151
1746509201	1926	1926	The Phantom Bullet	The Phantom Bullet	Smith, Clifford S.	Clifford Smith	tt0017263
1746561937	1957	1957	No Down Payment	No Down Payment	Ritt, Martin	Martin Ritt	tt0050771
1746464889	1941	1941	The Great American Broadcast	The Great American Broadcast	Mayo, Archie	Archie Mayo	tt0033674
1746502723	1917	1917	Fighting for Love	Fighting for Love	Wells, Raymond	Raymond Wells	tt0007940
1746570726	1933	1933	Disgraced	Disgraced	Kenton, Erle C.	Erle C. Kenton	tt0023951
1746533018	1923	1923	Oils Well!	Oil's Well	Goldaine, Mark	Mark Goldaine	tt0860478
1746582447	1937	1937	Ready, Willing and Able	Ready, Willing and Able	Enright, Ray	Ray Enright	tt0029467
1745272753	2000	2000	Road Trip	Road Trip	Phillips, Todd	Todd Phillips	tt0215129
1746503509	1920	1920	A Common Level	A Common Level	King, Burton	Burton L. King	tt0189433
1746475908	1948	1948	All My Sons	All My Sons	Reis, Irving	Irving Reis	tt0040087
1769973129	1925	1925	Tailoring	Tailoring	Davis, Jimmy	James D. Davis	tt0189135
1746584838	1930	1930	Raffles	Raffles	D'Arrast, Harry d'Abbadie; Fitzmauri	George Fitzmaurice	tt0021281
1746435208	1941	1941	Horror Island	Horror Island	Waggner, George	George Waggner	tt0033728
1746581368	1950	1950	The Miniver Story	The Miniver Story	Potter, H. C.; Saville, Victor	H.C. Potter	tt0042735
1746584503	1931	1931	Women of All Nations	Women of All Nations	Walsh, Raoul	Raoul Walsh	tt0022576
1746199487	1969	1969	Diaries, Notes, and Sketches	Diaries Notes and Sketches	Mekas, Jonas	Jonas Mekas	tt0196499
1746582940	1938	1938	Whirlwind Horseman	Whirlwind Horseman	Hill, Robert	Robert F. Hill	tt0030972
1746475523	1948	1948	California Firebrand	California Firebrand	Ford, Philip	Philip Ford	tt0040201
1746436343	1940	1940	Oh, Johnny, How You Can Love	Oh, Johnny, How You Can Love!	Lamont, Charles	Charles Lamont	tt0032857
1746497675	1921	1921	The Family Closet	The Family Closet	O'Brien, John B.	John B. O'Brien	tt0012152
1746491733	1944	1944	Lost in a Harem	Lost in a Harem	Riesner, Charles	Charles Reisner	tt0037027
1746200565	1970	1970	... Tick ... Tick ... Tick ...	...tick... tick... tick...	Nelson, Ralph	Ralph Nelson	tt0065360
1746533027	1923	1923	Don't Marry for Money	Don't Marry for Money	Brown, Clarence L.	Clarence Brown	tt0013999
1746577513	1933	1933	42nd Street	42nd Street	Bacon, Lloyd	Lloyd Bacon	tt0024034
1746435206	1940	1940	Strange Cargo	Strange Cargo	Borzage, Frank	Frank Borzage	tt0033105
1746514057	1916	1916	Driftwood	Driftwood	Farnum, Marshall	Marshall Farnum	tt0132102
1746499917	1948	1948	Fighting Father Dunne	Fighting Father Dunne	Tetzlaff, Ted	Ted Tetzlaff	tt0040355
1746493789	1910	1910	Michael Strogoff	Michael Strogoff	Dawley, J. Searle	J. Searle Dawley	tt0001318

1746583940	1930	1930	Captain Thunder	Captain Thunder	Crosland, Alan	Alan Crosland	tt0020740	
1746504216	1920	1920	Leave It to Me	Leave It to Me	Flynn, Emmett J.	Emmett J. Flynn	tt0011392	
1746513200	1916	1916	Ashes of Embers	Ashes of Embers	Kaufman, Joseph	Edward José	tt0006378	
1746520167	1925	1925	Heads Up	Heads Up	Garson, Harry	Harry Garson	tt0134707	
1746532831	1923	1923	You Can't Fool Your Wife	You Can't Fool Your Wife	Melford, George	George Melford	tt0014631	
1746515292	1919	1919	Let's Elope	Let's Elope	Robertson, John S.	John S. Robertson	tt0010350	
1746582956	1935	1935	Wilderness Mail	Wilderness Mail	Sheldon, Forrest	Forrest Sheldon	tt0027218	
1746574541	1952	1952	Border Saddlemates	Border Saddlemates	Witney, William	William Witney	tt0044439	
1745342208	2001	2001	Two Can Play that Game	Two Can Play That Game	Brown, Mark	Mark Brown	tt0269341	
1746577552	1935	1935	A Midsummer Night's Dream	A Midsummer Night's Dream	Reinhardt, Max; Dieterle, William	William Dieterle	tt0026714	
1746509259	1925	1925	The Texas Trail	The Texas Trail	Dunlap, Scott R.	Scott R. Dunlap	tt0016416	
1746516063	1921	1921	Enchantment	Enchantment	Vignola, Robert G.	Robert G. Vignola	tt0012136	
1746582700	1937	1937	On the Avenue	On the Avenue	Del Ruth, Roy	Roy Del Ruth	tt0029345	
1769970806	1914	1914	The Flames of Justice	The Flames of Justice	Billig, Irving		tt2773722	
1746523970	1915	1915	The Nigger	The Nigger	Lewis, Edgar	Edgar Lewis	tt0005809	
1746580229	1936	1936	The Rogues' Tavern	The Rogues' Tavern	Hill, Bob	Robert F. Hill	tt0028200	
**1746561328**	**1954**	**1954**	**They Rode West**	**They Rode West**	**Karlson, Phil**	**Phil Karlson**	**tt0047575**	1
1746409977	1986	1985	The Statue of Liberty	The Statue of Liberty	Burns, Ken	Ken Burns	tt0092011	
1745293640	2005	2005	The Exorcism of Emily Rose	The Exorcism of Emily Rose	Derrickson, Scott	Scott Derrickson	tt0404032	
1746502276	1916	1916	The Price of Happiness	The Price of Happiness	Lawrence, Edmond	Edmund Lawrence	tt0007210	
1745342804	2008	2008	Semi-Pro	Semi Pro	Alterman, Kent	Kent Alterman	tt0839980	
1746218767	1977	1977	The Incredible Melting Man	The Incredible Melting Man	Sachs, William	William Sachs	tt0076191	
1746582981	1939	1939	Across the Plains	Across the Plains	Bennett, Spencer	Spencer Gordon Bennet	tt0031018	
1746437328	1940	1940	Tear Gas Squad	Tear Gas Squad	Morse, Terry	Terry O. Morse	tt0033137	
**1746424227**	**1940**	**1940**	**Lights Out in Europe**	**Lights Out in Europe**	**Kline, Herbert**	**Herbert Kline**	**tt0195882**	1
1746570734	1933	1933	Her Resale Value	Her Resale Value	Eason, Breezy	B. Reeves Eason	tt0024112	
1746532845	1923	1923	Pleasure Mad	Pleasure Mad	Barker, Reginald	Reginald Barker	tt0014364	
1746208671	1969	1969	The Mad Room	The Mad Room	Girard, Bernard	Bernard Girard	tt0064617	
1746585199	1928	1928	Sins of the Fathers	Sins of the Fathers	Berger, Ludwig	Ludwig Berger	tt0019391	
1746496301	1920	1920	Fickle Women	Fickle Women	Butler, Frederick J.; McClung, Hugh	Fred J. Butler	tt0194875	
1746583592	1929	1929	Night Parade	Night Parade	St. Clair, Malcolm	Malcolm St. Clair	tt0020218	
1746235030	1980	1980	Caddyshack	Caddyshack	Ramis, Harold	Harold Ramis	tt0080487	
1746570577	1933	1933	Goldie Gets Along	Goldie Gets Along	St. Clair, Malcolm	Malcolm St. Clair	tt0024072	
1746486852	1919	1919	The Cinema Murder	The Cinema Murder	Baker, George D.	George D. Baker	tt0010008	
1746576713	1932	1932	The Son-Daughter	The Son Daughter	Brown, Clarence	Clarence Brown	tt0023495	
1746422286	1998	1998	3 Ninjas: High Noon at Mega Mountain	3 Ninjas: High Noon at Mega Mountain	McNamara, Sean	Sean McNamara	tt0118539	
1746566181	1953	1953	Split Second	Split Second	Powell, Dick	Dick Powell	tt0046353	
1746584922	1939	1939	...one third of a nation...	...One Third of a Nation...	Murphy, Dudley	Dudley Murphy	tt0031761	
1746475279	1948	1947	Heading for Heaven	Heading for Heaven	Collins, Lewis D.	Lewis D. Collins	tt0039451	
1746585365	1939	1939	Only Angels Have Wings	Only Angels Have Wings	Hawks, Howard	Howard Hawks	tt0031762	
1746569680	1955	1955	Count Three and Pray	Count Three and Pray	Sherman, George	George Sherman	tt0047954	
1746561097	1932	1932	False Faces	False Faces	Sherman, Lowell	Lowell Sherman	tt0022875	
1769969351	1964	1964	The Erotic Mr. Rose	The Erotic Mr. Rose	Millard, Nicholas	Nick Millard	tt0788069	
1746424180	1941	1941	Last of the Duanes	Last of the Duanes	Tinling, James	James Tinling	tt0033817	
1746584128	1938	1938	The Main Event	The Main Event	Dare, Danny	Daniel Dare	tt0030401	
1745272119	2000	2000	Tigerland	Tigerland	Schumacher, Joel	Joel Schumacher	tt0170691	
1746577036	1963	1963	Tammy and the Doctor	Tammy and the Doctor	Keller, Harry	Harry Keller	tt0057558	
1746487345	1917	1917	Pride	Pride	Ridgely, Richard	Richard Ridgely	tt0008455	
1746532752	1923	1923	Brass Commandments	Brass Commandments	Reynolds, Lynn F.	Lynn Reynolds	tt0013888	
1746583641	1931	1931	The Great Meadow	The Great Meadow	Brabin, Charles	Charles Brabin	tt0021928	
1746582410	1938	1938	Mystery House	Mystery House	Smith, Noel	Noel M. Smith	tt0030485	
1746569679	1953	1953	The President's Lady	The President's Lady	Levin, Henry	Henry Levin	tt0046204	
1746509028	1949	1949	Tulsa	Tulsa	Heisler, Stuart	Stuart Heisler	tt0041994	
1746410711	1991	1991	Necessary Roughness	Necessary Roughness	Dragoti, Stan	Stan Dragoti	tt0102517	
1746530728	1948	1948	Thunder in the Pines	Thunder in the Pines	Edwards, Robert	Robert Gordon	tt0040879	
1746515744	1920	1920	The Dark Mirror	The Dark Mirror	Giblyn, Charles	Charles Giblyn	tt0011094	
1746199523	1969	1969	Five the Hard Way	Five the Hard Way	Trikonis, Gus	Gus Trikonis	tt0061671	

1746520814	1926	1926	The Silent Lover	The Silent Lover	Archainbaud, George	George Archainbaud	tt0017388	
1746454304	1941	1941	Dynamite Canyon	Dynamite Canyon	Tansey, Robert	Robert Emmett Tansey	tt0033566	
1746581628	1910	1910	In the Season of Buds	In the Season of Buds	Griffith, D. W.	D.W. Griffith	tt0001268	
1746578700	1961	1961	Tomboy and the Champ	Tomboy and the Champ	Lyon, Francis D.	Francis D. Lyon	tt0055533	
1746410611	1988	1988	Casual Sex?	Casual Sex?	Robert, Genevieve	Geneviève Robert	tt0094846	
1746569764	1953	1953	Young Bess	Young Bess	Sidney, George	George Sidney	tt0046564	
1746582454	1936	1936	Ring Around the Moon	Ring Around the Moon	Lamont, Charles	Charles Lamont	tt0028186	
1746577981	1936	1936	Prison Shadows	Prison Shadows	Hill, Bob	Robert F. Hill	tt0028140	
1746520432	1926	1926	Jack o'Hearts	Jack O'Hearts	Hartford, David	David Hartford	tt0135478	
1746423469	1994	1994	Nell	Nell	Apted, Michael	Michael Apted	tt0110638	
1746574833	1936	1936	Ghost-Town Gold	Ghost Town Gold	Kane, Joseph	Joseph Kane	tt0027670	
1746584770	1930	1930	Son of the Gods	Son of the Gods	Lloyd, Frank	Frank Lloyd	tt0021400	
1746559869	1964	1964	The Masque of the Red Death	The Masque of the Red Death	Corman, Roger	Roger Corman	tt0058333	
1746497062	1917	1917	Sweetheart of the Doomed	Sweetheart of the Doomed	Barker, Reginald	Reginald Barker	tt0182477	
1746514281	1915	1915	A Gilded Fool	A Gilded Fool	Lewis, Edgar	Edgar Lewis	tt0005384	
1746580758	1935	1935	One New York Night	One New York Night	Conway, Jack	Jack Conway	tt0026823	
1746524810	1915	1915	The Island of Regeneration	The Island of Regeneration	Davenport, Harry	Harry Davenport	tt0005554	
1746434326	1995	1995	Species	Species	Donaldson, Roger	Roger Donaldson	tt0114508	
1746524139	1910	1910	The Unknown Claim	The Unknown Claim	Anderson, G. M.	Gilbert M. 'Broncho Billy' Anderson	tt0001436	
1746454405	1945	1945	The Phantom Speaks	The Phantom Speaks	English, John	John English	tt0037984	
1746503461	1919	1919	A Little Brother of the Rich	A Little Brother of the Rich	Reynolds, Lynn	Lynn Reynolds	tt0010367	
1746524337	1915	1915	My Madonna	My Madonna	Blaché, Alice	Alice Guy	tt0005790	
1745359484	2008	2008	Sex and the City	Sex and the City	King, Michael Patrick	Michael Patrick King	tt1000774	
1746503592	1919	1919	The Open Door	The Open Door	Fitzgerald, Dallas M.	Dallas M. Fitzgerald	tt0010531	
1746424427	1940	1940	She Couldn't Say No	She Couldn't Say No	Clemens, William	William Clemens	tt0034178	
1746516343	1920	1920	The Life of the Party	The Life of the Party	Henabery, Joseph	Joseph Henabery	tt0011401	
1746501591	1916	1916	The Stain in the Blood	The Stain in the Blood	MacQuarrie, Murdock	Murdock MacQuarrie	tt0158990	
1746394232	1992	1991	Lunatics: A Love Story	Lunatics: A Love Story	Becker, Josh	Josh Becker	tt0102357	
1746524723	1922	1922	Daring Danger	Daring Danger	Smith, Cliff	Clifford Smith	tt0013050	
1746581900	1910	1910	The Highbinders	The Highbinders			tt1840951	
1746508081	1927	1927	Rubber Tires	Rubber Tires	Hale, Alan	Alan Hale	tt0018350	
1746520467	1950	1950	Johnny One-Eye	Johnny One Eye	Florey, Robert	Robert Florey	tt0042618	
1746437171	1997	1997	Office Killer	Office Killer	Sherman, Cindy	Cindy Sherman	tt0119819	
1746467324	1917	1917	The Saintly Sinner	The Saintly Sinner	Wells, Raymond	Raymond Wells	tt0008533	
1746474825	1947	1947	Oregon Trail Scouts	Oregon Trail Scouts	Springsteen, R. G.	R.G. Springsteen	tt0039683	
1745272358	2000	2000	My Dog Skip	My Dog Skip	Russell, Jay	Jay Russell	tt0156812	
1746584506	1928	1928	The Good-Bye Kiss	The Good Bye Kiss	Sennett, Mack	Mack Sennett	tt0018951	
1745394319	2010	2010	Eat Pray Love	Eat Pray Love	Murphy, Ryan	Ryan Murphy	tt0879870	
1746555192	1956	1956	Love Me Tender	Love Me Tender	Webb, Robert D.	Robert D. Webb	tt0049452	
1746573499	1932	1932	Is My Face Red?	Is My Face Red?	Seiter, William	William A. Seiter	tt0023066	
1746581489	1950	1950	Rio Grande Patrol	Rio Grande Patrol	Selander, Lesley	Lesley Selander	tt0042896	
**1746573748**	**1957**	**1956**	**Full of Life**	**Full of Life**	**Quine, Richard**	**Richard Quine**	**tt0049240**	1
1745294010	2007	2007	Rush Hour 3	Rush Hour 3	Ratner, Brett	Brett Ratner	tt0293564	
1746465255	1944	1944	Partners of the Trail	Partners of the Trail	Hillyer, Lambert	Lambert Hillyer	tt0037165	
1746514731	1915	1915	Kreutzer Sonata	Kreutzer Sonata	Brenon, Herbert	Herbert Brenon	tt0005592	
1746577394	1961	1961	Eternal Summer	Eternal Summer	Wolk, Larry	Larry Wolk	tt0210030	
1746219000	1976	1976	Cannonball	Cannonball!	Bartel, Paul	Paul Bartel	tt0074279	
1746566147	1956	1955	No Place to Hide	No Place to Hide	Shaftel, Josef	Josef Shaftel	tt0049557	
1746582034	1951	1951	Father Takes the Air	Father Takes the Air	McDonald, Frank	Frank McDonald	tt0043525	
1746500509	1926	1926	The Love Thief	The Love Thief	McDermott, John	John McDermott	tt0017084	
1746567701	1933	1933	The Shadow Laughs	The Shadow Laughs	Hoerl, Arthur	Arthur Hoerl	tt0024545	
1746474970	1944	1944	The Very Thought of You	The Very Thought of You	Daves, Delmer	Delmer Daves	tt0037428	
1746581328	1959	1959	--30--	30	Webb, Jack	Jack Webb	tt0052526	
1746504204	1919	1919	It Pays to Advertise	It Pays to Advertise	Crisp, Donald	Donald Crisp	tt0010305	
1746561307	1955	1955	Headline Hunters	Headline Hunters	Witney, William	William Witney	tt0048150	
1746577489	1961	1961	The Two Little Bears	The Two Little Bears	Hood, Randall	Randall Hood	tt0055555	
1745357717	2008	2008	The Day the Earth Stood Still	The Day the Earth Stood Still	Derrickson, Scott	Scott Derrickson	tt0970416	

1746578044	1935	1935	George White's 1935 Scandals	George White's 1935 Scandals	Tinling, James	George White	tt0026403
1746523250	1927	1927	Wolf's Clothing	Wolf's Clothing	Del Ruth, Roy	Roy Del Ruth	tt0018585
1745309640	2006	2006	Let's Go to Prison	Let's Go to Prison	Odenkirk, Bob	Bob Odenkirk	tt0454987
1746569632	1957	1957	Jeanne Eagels	Jeanne Eagels	Sidney, George	George Sidney	tt0050560
1746508568	1927	1927	The Silent Rider	The Silent Rider	Reynolds, Lynn	Lynn Reynolds	tt0018401
1746454831	1942	1942	Joan of Ozark	Joan of Ozark	Stanley, Joseph	Joseph Santley	tt0034918
1746570302	1952	1952	Ma and Pa Kettle at the Fair	Ma and Pa Kettle at the Fair	Barton, Charles	Charles Barton	tt0044862
1746475245	1948	1948	The Big Punch	The Big Punch	Shourds, Sherry	Sherry Shourds	tt0040161
1746516357	1920	1920	The Woman of Mystery	The Woman of Mystery		Grace Cunard	tt0203212
1746393975	1989	1989	Born on the Fourth of July	Born on the Fourth of July	Stone, Oliver	Oliver Stone	tt0096969
1746435516	1940	1940	Golden Gloves	Golden Gloves	Dmytryk, Edward	Edward Dmytryk	tt0031378
1746182579	1910	1910	Capital vs. Labor	Capital vs. Labor		Van Dyke Brooke	tt0221051
1746520436	1926	1926	Beauty à la Mud	Beauty à la Mud	Beaudine, Harold	Harold Beaudine	tt0336010
1746491728	1946	1946	The El Paso Kid	The El Paso Kid	Carr, Thomas	Thomas Carr	tt0038507
1746524112	1910	1910	The Rummage Sale	The Rummage Sale		Sam Morris	tt1131693
1746577909	1938	1938	Reformatory	Reformatory	Collins, Lewis D.	Lewis D. Collins	tt0030664
1746501158	1915	1914	The Last Chapter	The Last Chapter	Taylor, William Desmond	William Desmond Taylor	tt0005604
1746410695	1983	1983	Bad Boys	Bad Boys	Rosenthal, Richard L.	Rick Rosenthal	tt0085210
1745341483	2010	2010	Letters to Juliet	Letters to Juliet	Winick, Gary	Gary Winick	tt0892318
1746438617	1940	1940	Danger on Wheels	Danger on Wheels	Cabanne, Christy	Christy Cabanne	tt0032380
1746185855	1969	1968	The Green Slime	The Green Slime	Fukasaku, Kinji	Kinji Fukasaku	tt0064393
1746520085	1926	1926	Winning the Futurity	Winning the Futurity	Dunlap, Scott	Scott R. Dunlap	tt0017568
1746583273	1929	1929	Frozen Justice	Frozen Justice	Dwan, Allan	Allan Dwan	tt0019907
1746520464	1927	1927	The Long Loop on the Pecos	The Long Loop on the Pecos	Maloney, Leo	Leo D. Maloney	tt0018101
1746584754	1928	1928	Hold 'Em Yale	Hold 'Em Yale	Griffith, Edward H.	Edward H. Griffith	tt0019009
1746200020	1969	1969	Shark!	Shark	Fuller, Samuel; Portillo, Rafael	Samuel Fuller	tt0066365
1746454192	1995	1995	Congo	Congo	Marshall, Frank	Frank Marshall	tt0112715
1746497289	1919	1919	Eastward Ho!	Eastward Ho!	Flynn, Emmett J.	Emmett J. Flynn	tt0011145
1746573870	1960	1960	Swiss Family Robinson	Swiss Family Robinson	Annakin, Ken	Ken Annakin	tt0054357
1746408479	1990	1990	Back to the Future Part III	Back to the Future Part III	Zemeckis, Robert	Robert Zemeckis	tt0099088
1746516294	1920	1920	Rio Grande	Rio Grande	Carewe, Edwin	Edwin Carewe	tt0011630
1745359277	2010	2010	Legion	Legion	Stewart, Scott	Scott Stewart	tt1038686
1746582582	1939	1939	The Cisco Kid and the Lady	The Cisco Kid and the Lady	Leeds, Herbert I.	Herbert I. Leeds	tt0032341
1746582267	1931	1931	Monkey Business	Donkey Business	McLeod, Norman	Arthur Hurley	tt0026290
1746524304	1923	1923	While Paris Sleeps	While Paris Sleeps	Tourneur, Maurice	Maurice Tourneur	tt0014602
1745360007	2008	2008	The Dark Knight	The Dark Knight	Nolan, Christopher	Christopher Nolan	tt0468569
2007988938	1982	1982	Basket Case	Basket Case	Henenlotter, Frank	Frank Henenlotter	tt0083624
1746497589	1921	1921	Too Wise Wives	Too Wise Wives	Weber, Lois	Lois Weber	tt0012769
1746454007	1943	1943	Mr. Lucky	Mr. Lucky	Potter, H. C.	H.C. Potter	tt0036174
1746496781	1919	1919	The Day She Paid	The Day She Paid	Ingram, Rex	Rex Ingram	tt0010056
1746524249	1923	1923	Our Hospitality	Our Hospitality	Keaton, Buster; Blystone, John	John G. Blystone	tt0014341
1746582767	1936	1936	Career Woman	Career Woman	Seiler, Lewis	Lewis Seiler	tt0027423
1746454815	1946	1946	Talk About a Lady	Talk About a Lady	Sherman, George	George Sherman	tt0039008
1746569904	1958	1958	Monster on the Campus	Monster on the Campus	Arnold, Jack	Jack Arnold	tt0051948
1746570376	1934	1934	The Man Who Reclaimed His Head	The Man Who Reclaimed His Head	Ludwig, Edward	Edward Ludwig	tt0025453
1746580962	1960	1960	The Rat Race	The Rat Race	Mulligan, Robert	Robert Mulligan	tt0054230
1746235120	1966	1966	The Black Klansman	The Black Klansman	Mikels, Ted V.	Ted V. Mikels	tt0060173
1746248838	1974	1974	Caged Heat!	Caged Heat	Demme, Jonathan	Jonathan Demme	tt0071266
1746575005	1933	1933	Best of Enemies	Best of Enemies	James, Rian; Craven, Frank	Rian James	tt0023796
1746555712	1956	1955	The Trouble with Harry	The Trouble with Harry	Hitchcock, Alfred	Alfred Hitchcock	tt0048750
1746235909	1976	1976	Futureworld	Futureworld	Heffron, Richard T.	Richard T. Heffron	tt0074559
1746585415	1940	1940	Money and the Woman	Money and the Woman	Howard, William K.	William K. Howard	tt0032806
1746499895	1946	1946	Notorious	Notorious	Hitchcock, Alfred	Alfred Hitchcock	tt0038787
1746500502	1924	1924	A Girl of the Limberlost	A Girl of the Limberlost	Meehan, James Leo	James Leo Meehan	tt0014947
1746184821	1965	1965	Bad Girls Go to Hell	Bad Girls Go to Hell		Doris Wishman	tt0058933
1745393784	2008	2008	Patti Smith: Dream of Life	Patti Smith: Dream of Life	Sebring, Steven	Steven Sebring	tt0940620
1746533179	1914	1914	The Kiss	The Kiss	Davis, Ulysses	Ulysses Davis	tt0004193

1746569807	1955	1955	The Private War of Major Benson	The Private War of Major Benson	Hopper, Jerry	Jerry Hopper	tt0048513
1746514242	1915	1915	The Senator	The Senator	Golden, Joseph A.	Joseph A. Golden	tt0006024
1746586162	1939	1939	Torchy Blane in Chinatown	Torchy Blane in Chinatown	Beaudine, William	William Beaudine	tt0030882
1746581856	1928	1928	The Valley of Hunted Men	The Valley of Hunted Men	Thorpe, Richard	Richard Thorpe	tt0019519
1746569990	1958	1958	Diamond Safari	Diamond Safari	Mayer, Gerald	Gerald Mayer	tt0205007
1746235824	1979	1979	H.O.T.S.	H.O.T.S.	Sindell, Gerald Seth	Gerald Seth Sindell	tt0079257
1746436197	1998	1998	U.S. Marshals	U.S. Marshals	Baird, Stuart	Stuart Baird	tt0120873
1746500685	1926	1926	Trumpin' Trouble	Trumpin' Trouble	Thorpe, Richard	Richard Thorpe	tt0174288
1746497590	1922	1922	The Lavender Bath Lady	The Lavender Bath Lady	Baggot, King	King Baggot	tt0013306
1746556030	1956	1956	The Opposite Sex	The Opposite Sex	Miller, David	David Miller	tt0049578
1746476200	1946	1946	The Razor's Edge	The Razor's Edge	Goulding, Edmund	Edmund Goulding	tt0038873
1746584056	1936	1936	They Met in a Taxi	They Met in a Taxi	Green, Alfred E.	Alfred E. Green	tt0028357
1746581265	1962	1962	Stagecoach to Dancers' Rock	Stagecoach to Dancers' Rock	Bellamy, Earl	Earl Bellamy	tt0056522
1746497292	1922	1922	Another Man's Boots	Another Man's Boots	Craft, William J.	William James Craft	tt0011927
1746582989	1938	1938	The Utah Trail	Utah Trail	Herman, Al	Albert Herman	tt0030921
1746532708	1923	1923	Other Men's Daughters	Other Men's Daughters	Wilson, Ben	Ben F. Wilson	tt0014340
1746475954	1943	1942	We Are the Marines	We Are the Marines	Rochemont, Louis de	Louis De Rochemont	tt0035534
1746440024	1997	1997	Dangerous Ground	Dangerous Ground	Roodt, Darrell James	Darrell Roodt	tt0118927
1746511771	1923	1923	The Scarlet Car	The Scarlet Car	Paton, Stuart	Stuart Paton	tt0014441
1746508939	1948	1948	The Olympic Cavalcade	Olympic Cavalcade			tt6241040
1769972172	1961	1961	Operation Bottleneck	Operation Bottleneck	Cahn, Edward L.	Edward L. Cahn	tt0055260
1746507571	1926	1926	Exclusive Rights	Exclusive Rights	O'Connor, Frank	Frank O'Connor	tt0016831
1746439460	1994	1994	Night of the Demons 2	Night of the Demons 2	Trenchard-Smith, Brian	Brian Trenchard	tt0110667
1746465337	1945	1945	Conflict	Conflict	Bernhardt, Curtis	Curtis Bernhardt	tt0037611
1746582063	1929	1929	The Devil's Apple Tree	The Devil's Apple Tree	Clifton, Elmer	Elmer Clifton	tt0019816
1746574030	1957	1957	Outlaw's Son	Outlaw's Son	Selander, Lesley	Lesley Selander	tt0050812
1746513046	1916	1916	Manhattan Madness	Manhattan Madness	Dwan, Allan	Allan Dwan	tt0007031
1746487041	1918	1918	Good Night, Paul	Good Night, Paul	Edwards, Walter	Walter Edwards	tt0009124
1745357982	2004	2004	Win a Date with Tad Hamilton!	Win a Date with Tad Hamilton!	Luketic, Robert	Robert Luketic	tt0335559
1746584765	1929	1929	The Iron Mask	The Iron Mask	Dwan, Allan	Allan Dwan	tt0020030
1746545346	1940	1940	One Man's Law	One Man's Law	Sherman, George	George Sherman	tt0032870
1746508189	1924	1924	K--The Unknown	K The Unknown	Pollard, Harry	Harry A. Pollard	tt0015030
1746524016	1916	1916	Whoso Findeth a Wife	Whoso Findeth a Wife	Crane, Frank	Frank Hall Crane	tt0007564
1746394703	1987	1987	Munchies	Munchies	Hirsch, Bettina	Tina Hirsch	tt0093582
1746583660	1928	1927	The Call of the Sea	The Call of the Sea	Hall, Harold L.	Harold R. Hall	tt0423934
1746582010	1929	1929	Redskin	Redskin	Schertzinger, Victor	Victor Schertzinger	tt0020320
1746422508	1996	1996	Original Gangstas	Original Gangstas	Cohen, Larry	Larry Cohen	tt0117260
1746393498	1980	1980	Heaven's Gate	Heaven's Gate	Cimino, Michael	Michael Cimino	tt0080855
1746581885	1929	1929	Bad Men's Money	Bad Men's Money	McGowan, J. P.	J.P. McGowan	tt0019672
1746438288	1943	1943	Man of Courage	Man of Courage	Thurn-Taxis, Alexis	Alexis Thurn	tt0036136
1746560661	1932	1932	Speak Easily	Sneak Easily	Sedgwick, Edward	Gus Meins	tt0023490
1746219562	1971	1971	A Gunfight	A Gunfight	Johnson, Lamont	Lamont Johnson	tt0067168
1746436685	1943	1943	Honeymoon Lodge	Honeymoon Lodge	Lilley, Edward	Edward C. Lilley	tt0036013
1746464959	1942	1942	The Big Shot	The Big Shot	Seiler, Lewis	Lewis Seiler	tt0034513
1746580718	1937	1937	Roll Along Cowboy	Roll Along, Cowboy	Meins, Gus	Gus Meins	tt0030688
1746232936	1969	1969	Marlowe	Marlowe	Bogart, Paul	Paul Bogart	tt0064638
1746573732	1961	1961	The Honeymoon Machine	The Honeymoon Machine	Thorpe, Richard	Richard Thorpe	tt0054989
1746453478	1941	1941	Sierra Sue	Sierra Sue	Morgan, William	William Morgan	tt0034187
1746282391	1980	1980	Any Which Way You Can	Any Which Way You Can	Van Horn, Buddy	Buddy Van Horn	tt0080377
1769970748	1915	1915	The Voice in the Wilderness	A Voice in the Wilderness		James W. Horne	tt3140184
1746248289	1966	1966	A Smell of Honey, a Swallow of Brine!	A Smell of Honey, a Swallow of Brine	Elliott, B. Ron	Byron Mabe	tt0180177
1746499943	1948	1948	Big City	Big City	Taurog, Norman	Norman Taurog	tt0040159
1746562303	1956	1956	I've Lived Before	I've Lived Before	Bartlett, Richard	Richard Bartlett	tt0049353
1746500505	1950	1951	Law of the Badlands	Law of the Badlands	Selander, Lesley	Lesley Selander	tt0042667
1746492230	1944	1944	End of the Road	End of the Road	Blair, George	George Blair	tt0036790
1746410568	1983	1983	Strange Brew	Strange Brew	Thomas, Dave; Moranis, Rick	Rick Moranis	tt0086373
1746532839	1914	1914	The Virginian	The Virginian		Cecil B. DeMille	tt0004766

1746501450	1915	1915	A Woman's Resurrection	A Woman's Resurrection	Edwards, J. Gordon	J. Gordon Edwards	tt0006308
1746574795	1933	1933	Unknown Valley	Unknown Valley	Hillyer, Lambert	Lambert Hillyer	tt0024722
1746409459	1986	1986	¡Three Amigos!	¡Three Amigos!	Landis, John	John Landis	tt0092086
1746514051	1915	1915	The Celebrated Scandal	The Celebrated Scandal	Durkin, James	James Durkin	tt0005071
1746437017	1943	1943	I Walked with a Zombie	I Walked with a Zombie	Tourneur, Jacques	Jacques Tourneur	tt0036027
1745372490	2001	2001	Evolution	Evolution	Reitman, Ivan	Ivan Reitman	tt0251075
1746409484	1988	1988	Tapeheads	Tapeheads	Fishman, Bill	Bill Fishman	tt0096223
1746394174	1992	1992	Light Sleeper	Light Sleeper	Schrader, Paul	Paul Schrader	tt0102307
1746233509	1968	1968	The Pick-up	The Pick Up	Frost, R. L.	Lee Frost	tt0127108
1746524820	1928	1928	A Night of Mystery	A Night of Mystery	Mendes, Lothar	Lothar Mendes	tt0019213
1769970812	1915	1915	The Leap From the Water Tower	The Leap from the Water Tower		J.P. McGowan	tt1316468
1746424384	1941	1941	Men of Boys Town	Men of Boys Town	Taurog, Norman	Norman Taurog	tt0033902
1746560319	1964	1964	One Potato, Two Potato	One Potato, Two Potato	Peerce, Larry	Larry Peerce	tt0058429
1746410748	1988	1988	Bat 21	Bat*21	Markle, Peter	Peter Markle	tt0094712
1746515269	1922	1922	The Old Homestead	The Cold Homestead	Cruze, James	Arthur D.V. Storey	tt3458410
1746465001	1942	1942	Rio Rita	Rio Rita	Simon, S. Sylvan	S. Sylvan Simon	tt0035259
1746580864	1935	1935	Fighting Youth	Fighting Youth	MacFadden, Hamilton	Hamilton MacFadden	tt0026356
1746199489	1965	1965	Rapture	Rapture	Guillermin, John	John Guillermin	tt0059633
1746235312	1978	1978	The Greek Tycoon	The Greek Tycoon	Thompson, J. Lee	J. Lee Thompson	tt0077636
1746513446	1916	1916	His Picture in the Papers	His Picture in the Papers	Emerson, John	John Emerson	tt0006809
1746573728	1956	1956	Foreign Intrigue	Foreign Intrigue	Reynolds, Sheldon	Sheldon Reynolds	tt0049224
1746584603	1930	1930	The Virtuous Sin	The Virtuous Sin	Cukor, George; Gasnier, Louis	George Cukor	tt0021525
1746574535	1933	1933	Peg O' My Heart	Peg o' My Heart	Leonard, Robert Z.	Robert Z. Leonard	tt0024433
1769976167	1928	1928	Racing Mad	Racing Mad	Roberts, Stephen	Stephen Roberts	tt0494538
1746454112	2000	1999	But I'm a Cheerleader	But I'm a Cheerleader	Babbit, Jamie	Jamie Babbit	tt0179116
1746394423	1990	1990	House Party	House Party	Hudlin, Reginald	Reginald Hudlin	tt0099800
1746454607	1945	1945	The Horn Blows at Midnight	The Horn Blows at Midnight	Walsh, Raoul	Raoul Walsh	tt0037790
1746524970	1915	1915	Chimmie Fadden	Chimmie Fadden	DeMille, Cecil B.	Cecil B. DeMille	tt0005086
1746515856	1920	1920	Love's Flame	Love's Flame	Gregory, Carl Louis	Carl Gregory	tt0196720
1746524848	1916	1916	The Men She Married	The Men She Married	Vale, Travers	Travers Vale	tt0007056
1746465296	1945	1945	I'll Tell the World	I'll Tell the World	Goodwins, Leslie	Leslie Goodwins	tt0037808
1746184378	1910	1910	The Affair of an Egg	The Affair of an Egg	Powell, Frank	Frank Powell	tt0001104
1746532634	1915	1915	The Voice in the Fog	The Voice in the Fog	McGowan, J. P.	J.P. McGowan	tt0006230
1746566306	1955	1955	Big House, U.S.A.	Big House, U.S.A.	Koch, Howard W.	Howard W. Koch	tt0047879
1746507621	1925	1925	Sky's the Limit	Sky's the Limit	Irving, I. W.	Harry L. Fraser	tt0015598
1746422622	1994	1994	Bad Girls	Bar Girls	Kaplan, Jonathan	Marita Giovanni	tt0109217
1746421679	1993	1993	The Age of Innocence	The Age of Innocence	Scorsese, Martin	Martin Scorsese	tt0106226
1746492218	1945	1945	The Chicago Kid	The Chicago Kid	McDonald, Frank	Frank McDonald	tt0037592
1746532486	1915	1915	The Man Trail	The Man Trail	Calvert, E. H.	E.H. Calvert	tt0005693
1746574536	1935	1935	Thunder in the Night	Thunder in the Night	Archainbaud, George	George Archainbaud	tt0027105
1746504419	1919	1919	Secret Service	Secret Service	Ford, Hugh	Hugh Ford	tt0010676
1746454214	1943	1943	A Gentle Gangster	A Gentle Gangster	Rosen, Phil	Phil Rosen	tt0035930
1746577420	1959	1959	A Hole in the Head	A Hole in the Head	Capra, Frank	Frank Capra	tt0052896
1746559875	1964	1965	Nightmare in the Sun	Nightmare in the Sun	Lawrence, Marc	John Derek	tt0059505
1746581492	1951	1951	Canyon Raiders	Canyon Raiders	Collins, Lewis	Lewis D. Collins	tt0043377
1746235673	1976	1976	The Premonition	The Premonition	Schnitzer, Robert Allen	Robert Allen Schnitzer	tt0075097
1746454379	1942	1942	Fly-by-Night	Fly By Night	Siodmak, Robert	Robert Siodmak	tt0034740
1746577633	1934	1934	Little Man, What Now?	Little Man, What Now?	Borzage, Frank	Frank Borzage	tt0025408
1746569582	1957	1957	Last Stagecoach West	The Last Stagecoach West	Kane, Joe	Joseph Kane	tt0050624
1746578992	1962	1962	Gypsy	Gypsy	LeRoy, Mervyn	Mervyn LeRoy	tt0056048
1746570265	1934	1934	The Notorious Sophie Lang	The Notorious Sophie Lang	Murphy, Ralph	Ralph Murphy	tt0025577
1746577923	1938	1938	Born to Be Wild	Born to Be Wild	Kane, Joe	Joseph Kane	tt0029939
1746235178	1968	1968	Fanny Hill Meets the Red Baron	Fanny Hill Meets the Red Baron	Mahon, Barry	Barry Mahon	tt0218204
1746580654	1938	1938	Stagecoach Days	Stagecoach Days	Levering, Joseph	Joseph Levering	tt0030788
1746500276	1924	1924	Gold Heels	Gold Heels	Van Dyke, William S.	W.S. Van Dyke	tt0014950
1746577959	1937	1937	Navy Spy	Navy Spy	Wilbur, Crane	Joseph H. Lewis	tt0125432
1746570136	1952	1952	Jumping Jacks	Jumping Jacks	Taurog, Norman	Norman Taurog	tt0044774

1746209584	1966	1966	W. I. A. (Wounded in Action)	W.I.A. Wounded in Action	Sunasky, Irving	Irving Sunasky	tt0170795
1746524840	1914	1914	The Man on the Box	The Man on the Box	Apfel, Oscar C.	Oscar Apfel	tt0004302
1746464976	1947	1947	The Bachelor and the Bobby Soxer	The Bachelor and the Bobby Soxer	Reis, Irving	Irving Reis	tt0039169
1746496572	1919	1919	The Little White Savage	The Little White Savage	Powell, Paul	Paul Powell	tt0010372
1746578994	1958	1958	Apache Territory	Apache Territory	Nazarro, Ray	Ray Nazarro	tt0051369
1746423334	1993	1993	The Music of Chance	The Music of Chance	Haas, Philip	Philip Haas	tt0107623
1745357615	2009	2009	Big River Man	Big River Man	Maringouin, John	John Maringouin	tt0956101
1746515072	1918	1918	The Fighting Grin	The Fighting Grin	De Grasse, Joseph	Joseph De Grasse	tt0009065
1746582944	1940	1940	Remedy for Riches	Remedy for Riches	Kenton, Erle C.	Erle C. Kenton	tt0032980
1746234967	1967	1967	Festival	Festival	Lerner, Murray	Murray Lerner	tt0061658
1746474459	1948	1948	Song of My Heart	Song of My Heart	Glazer, Benjamin	Benjamin Glazer	tt0039851
1746584574	1929	1928	In Old Arizona	In Old Arizona	Walsh, Raoul; Cummings, Irving	Irving Cummings	tt0020018
1746218701	1973	1973	The Spook Who Sat by the Door	The Spook Who Sat by the Door	Dixon, Ivan	Ivan Dixon	tt0070726
1746585033	1940	1940	Curtain Call	Curtain Call	Woodruff, Frank	Frank Woodruff	tt0032373
1746473898	1942	1942	Klondike Fury	Klondike Fury	Howard, William K.	William K. Howard	tt0034948
1746565888	1954	1954	Sitting Bull	Sitting Bull	Salkow, Sidney	Sidney Salkow	tt0047501
1746581777	1928	1927	That's My Daddy	That's My Daddy	Newmeyer, Fred	Fred C. Newmeyer	tt0019463
1746465360	1942	1942	Tomorrow We Live	Tomorrow We Live	Ulmer, Edgar G.	Edgar G. Ulmer	tt0035453
1746454481	1941	1941	King of the Zombies	King of the Zombies	Yarbrough, Jean	Jean Yarbrough	tt0033787
1746492228	1944	1943	Swing Fever	Swing Fever	Whelan, Tim	Tim Whelan	tt0037335
1746555723	1957	1957	The Monster that Challenged the World	The Monster That Challenged the World	Laven, Arnold	Arnold Laven	tt0050722
1746424170	1942	1942	Orchestra Wives	Orchestra Wives	Mayo, Archie; Brahm, John	Archie Mayo	tt0035157
1745359495	2008	2008	Beer for My Horses	Beer for My Horses	Salomon, Michael	Michael Salomon	tt1178640
1746574817	1933	1933	Strange People	Strange People	Thorpe, Richard	Richard Thorpe	tt0024619
1746520929	1950	1950	Frontier Outpost	Frontier Outpost	Nazarro, Ray	Ray Nazarro	tt0042484
1746500610	1926	1926	Dance Madness	Dance Madness	Leonard, Robert Z.	Robert Z. Leonard	tt0016759
1746512587	1927	1927	Dress Parade	Dress Parade	Crisp, Donald	Donald Crisp	tt0017829
1746568163	1933	1933	Hard to Handle	Hard to Handle	LeRoy, Mervyn	Mervyn LeRoy	tt0024090
1746513711	1916	1916	Behind Closed Doors	Behind Closed Doors	Golden, Joseph A.	Joseph A. Golden	tt0157369
1746520495	1926	1926	Dame Chance	Dame Chance	Bracken, Bertram	Bertram Bracken	tt0016758
1746454175	1997	1996	Box of Moonlight	Box of Moon Light	DiCillo, Tom	Tom DiCillo	tt0115738
1746569561	1954	1954	Athena	Athena	Thorpe, Richard	Richard Thorpe	tt0046728
1745272771	2001	2000	Before Night Falls	Before Night Falls	Schnabel, Julian	Julian Schnabel	tt0247196
1746235624	1978	1978	Starhops	Starhops	Peeters, Barbara	Barbara Peeters	tt0078318
1746585183	1928	1928	The Cloud Buster	The Cloud Buster	Moore, Vin	Vin Moore	tt0321725
1746563635	1952	1952	Hong Kong	Hong Kong	Foster, Lewis R.	Lewis R. Foster	tt0043654
1746487060	1918	1918	The Claim	The Claim	Reicher, Frank	Frank Reicher	tt0008965
1746420733	1988	1988	Bird	Bird	Eastwood, Clint	Clint Eastwood	tt0094747
1746583013	1935	1935	The Outlaw Deputy	The Outlaw Deputy	Brower, Otto	Otto Brower	tt0026836
1746571080	1934	1934	Here Comes the Navy	Here Comes the Navy	Bacon, Lloyd	Lloyd Bacon	tt0025238
1746423491	1998	1998	Meet the Deedles	Meet the Deedles	Boyum, Steve	Steve Boyum	tt0120645
1746511739	1915	1915	The Man Who Couldn't Beat God	The Man Who Couldn't Beat God	Costello, Maurice; Gaillard, Robert	Maurice Costello	tt0005695
1746393765	1987	1987	Gardens of Stone	Gardens of Stone	Coppola, Francis	Francis Ford Coppola	tt0093073
1746409080	1987	1987	Adventures in Babysitting	Adventures in Babysitting	Columbus, Chris	Chris Columbus	tt0092513
1746503064	1916	1916	The Patriot	The Patriot	Hart, William S.	William S. Hart	tt0007160
1746567809	1933	1933	Night of Terror	Night of Terror	Stoloff, Ben	Benjamin Stoloff	tt0024382
1746492491	1945	1945	The Return of the Durango Kid	The Return of the Durango Kid	Abrahams, Derwin	Derwin Abrahams	tt0037218
1746512066	1910	1910	St. Elmo	St. Elmo	O'Neil, Barry; Carleton, Lloyd B.	Lloyd B. Carleton	tt0344376
1746410882	1987	1987	Deadly Illusion	Deadly Illusion	Tannen, William; Cohen, Larry	Larry Cohen	tt0092847
1746565931	1954	1954	The Big Chase	The Big Chase	Hilton, Arthur D.	Arthur Hilton	tt0046774
1745341306	2001	2001	The Forsaken	The Forsaken	Cardone, J. S.	J.S. Cardone	tt0245120
1746577349	1963	1963	The Thrill of It All	The Thrill of It All	Jewison, Norman	Norman Jewison	tt0057581
1746570722	1952	1952	Rainbow 'Round My Shoulder	Rainbow 'Round My Shoulder	Quine, Richard	Richard Quine	tt0045068
1746585440	1940	1940	I Take This Woman	I Take This Woman	Van Dyke, W. S.; Sternberg, Josef von	W.S. Van Dyke	tt0031466
1746419390	1982	1981	Waitress!	Waitress!	Weil, Samuel; Herz, Michael	Michael Herz	tt0083312
1746574454	1934	1935	The Cactus Kid	The Cactus Kid	Webb, Harry S.	Harry S. Webb	tt0024942
1746524125	1910	1910	The Unchanging Sea	The Unchanging Sea	Griffith, D. W.	D.W. Griffith	tt0001431

1746584438	1931	1931	Platinum Blonde	Platinum Blonde	Capra, Frank R.	Frank Capra	tt0022268
1746492429	1945	1945	Behind City Lights	Behind City Lights	English, John	John English	tt0037532
1769970066	1912	1912	The Old Reporter	The Old Reporter	Miller, Ashley	Harold M. Shaw	tt0002403
1745342712	2008	2008	Meet Dave	Meet Dave	Robbins, Brian	Brian Robbins	tt0765476
1746566760	1952	1952	When in Rome	When in Rome	Brown, Clarence	Clarence Brown	tt0045324
1746577624	1936	1936	These Three	These Three	Wyler, William	William Wyler	tt0028356
1746474985	1944	1944	The Last Ride	The Last Ride	Lederman, D. Ross	D. Ross Lederman	tt0037007
1746475724	1946	1946	Mysterious Intruder	Mysterious Intruder	Castle, William	William Castle	tt0038766
1746586157	1940	1940	Military Academy	Military Academy	Lederman, D. Ross	D. Ross Lederman	tt0032793
1746585001	1939	1939	They All Come Out	They All Come Out	Tourneur, Jacques	Jacques Tourneur	tt0032019
1746453565	1943	1942	Journey into Fear	Journey Into Fear	Foster, Norman	Norman Foster	tt0034922
1746567251	1933	1933	Big Time or Bust	Big Time or Bust	Newfield, Sam	Sam Newfield	tt0024891
1746524497	1915	1915	The House of Tears	The House of Tears	Crane, Edwin	Edwin Carewe	tt0005516
1746503008	1910	1910	The Masher	The Masher	Powell, Frank	Frank Powell	tt0001309
1746581053	1962	1962	The Great Chase	The Great Chase			tt0057114
1746508903	1924	1924	Her Marriage Vow	Her Marriage Vow	Webb, Millard	Millard Webb	tt0014129
1746580975	1960	1960	Elmer Gantry	Elmer Gantry	Brooks, Richard	Richard Brooks	tt0053793
1746566748	1933	1933	Via Pony Express	Via Pony Express	Collins, Lew	Lewis D. Collins	tt0024730
1746573783	1958	1957	The Golden Age of Comedy	The Golden Age of Comedy		Robert Youngson	tt0129119
1746454319	1996	1996	The Adventures of Pinocchio	The Adventures of Pinocchio	Barron, Steve	Steve Barron	tt0115472
1746580596	1937	1937	The Shadow Strikes	The Shadow Strikes	Shores, Lynn	Lynn Shores	tt0029542
1746491726	1945	1945	Come Out Fighting	Come Out Fighting	Beaudine, William	William Beaudine	tt0037609
1746200220	1969	1969	Number One	Number One	Gries, Tom	Tom Gries	tt0064743
1746421252	1994	1994	Natural Born Killers	Natural Born Killers	Stone, Oliver	Oliver Stone	tt0110632
1746563670	1952	1952	Springfield Rifle	Springfield Rifle	DeToth, Andre	André De Toth	tt0045184
1746571178	1910	1910	For the Sunday Edition	For the Sunday Edition			tt1480766
1746520058	1925	1925	Fast Fightin'	Fast Fightin'	Thorpe, Richard	Richard Thorpe	tt0015795
1746453854	1941	1941	Sun Valley Serenade	Sun Valley Serenade	Humberstone, H. Bruce	H. Bruce Humberstone	tt0034241
1746423796	1941	1941	H. M. Pulham, Esq.	H.M. Pulham, Esq.	Vidor, King	King Vidor	tt0033686
1746574669	1936	1936	The Fugitive Sheriff	The Fugitive Sheriff	Bennett, Spencer Gordon	Spencer Gordon Bennet	tt0027650
1746561744	1957	1957	Lure of the Swamp	Lure of the Swamp	Cornfield, Hubert	Hubert Cornfield	tt0050661
1746583496	1951	1951	The First Legion	The First Legion	Sirk, Douglas	Douglas Sirk	tt0043537
1746576007	1910	1910	Faithful	Faithful	Griffith, D. W.	D.W. Griffith	tt0001207
1746500137	1925	1925	Broadway Lady	Broadway Lady	Ruggles, Wesley	Wesley Ruggles	tt0015647
1746574634	1935	1935	Break of Hearts	Break of Hearts	Moeller, Philip	Philip Moeller	tt0026134
1746419171	1994	1994	D2: The Mighty Ducks	D2: The Mighty Ducks	Weisman, Sam	Sam Weisman	tt0109520
1769970813	1912	1912	Is He Eligible	Is He Eligible?		C.J. Williams	tt0483689
1746502710	1916	1916	Hesper of the Mountains	Hesper of the Mountains	North, Wilfrid	Wilfrid North	tt0157752
1746583021	1935	1935	The Black Room	The Black Room	Neill, R. William	Roy William Neill	tt0026123
1745342049	2003	2003	View from the Top	View from the Top	Barreto, Bruno	Bruno Barreto	tt0264150
1746580911	1937	1937	The Holy Terror	The Holy Terror	Tinling, James	James Tinling	tt0029014
1746520840	1925	1925	The Road to Yesterday	The Road to Yesterday		Cecil B. DeMille	tt0016294
1746219362	1973	1973	The Cheerleaders	The Cheerleaders	Glicker, Paul	Paul Glickler	tt0068364
1746566241	1957	1957	Utah Blaine	Utah Blaine	Sears, Fred F.	Fred F. Sears	tt0051145
1746553944	1965	1965	War Party	War Party	Selander, Lesley	Lesley Selander	tt0229029
1746523430	1923	1923	Three Wise Fools	Three Wise Fools	Vidor, King	King Vidor	tt0014542
1746436898	1998	1997	Nightwatch	Nightwatch	Bornedal, Ole	Ole Bornedal	tt0119791
1746500058	1950	1950	The Jackpot	The Jackpot	Lang, Walter	Walter Lang	tt0042610
1746465244	1945	1945	Frisco Sal	Frisco Sal	Waggner, George	George Waggner	tt0037717
1746218680	1971	1971	something big	Something Big	McLaglen, Andrew V.	Andrew V. McLaglen	tt0068022
1746542663	1927	1927	Snowbound	Snowbound	Stone, Phil	Phil Goldstone	tt0018421
1746394514	1982	1982	The Seduction	The Seduction	Schmoeller, David	David Schmoeller	tt0084652
1746503761	1920	1920	A Thousand to One	A Thousand to One	Lee, Rowland V.	Rowland V. Lee	tt0012751
1746566134	1956	1956	Magic Fire	Magic Fire	Dieterle, William	William Dieterle	tt0049461
1745373168	2008	2008	Nothing Like the Holidays	Nothing Like the Holidays	De Villa, Alfredo	Alfredo Rodriguez de Villa	tt1151915
1746500006	1948	1948	Sundown in Santa Fe	Sundown in Santa Fe	Springsteen, R. G.	R.G. Springsteen	tt0040850
1746525031	1920	1920	Blind Love	Blind Love	Bailey, Oliver D.	Oliver D. Bailey	tt0186886

1769971965	1969	1969	Uta	Uta		Nick Millard	tt0260488
1746423558	1997	1997	Donnie Brasco	Donnie Brasco	Newell, Mike	Mike Newell	tt0119008
1746520300	1948	1948	The Vicious Circle	The Vicious Circle	Wilder, W. Lee	W. Lee Wilder	tt0040941
1745342282	2004	2004	The Whole Ten Yards	The Whole Ten Yards	Deutch, Howard	Howard Deutch	tt0327247
1746520892	1949	1949	Mighty Joe Young	Mighty Joe Young	Schoedsack, Ernest B.	Ernest B. Schoedsack	tt0041650
1746574968	1938	1938	Cassidy of Bar 20	Cassidy of Bar 20	Selander, Lesley	Lesley Selander	tt0029976
1746474468	1947	1947	The Unsuspected	The Unsuspected	Curtiz, Michael	Michael Curtiz	tt0039941
1746497004	1918	1918	The Eyes of Julia Deep	The Eyes of Julia Deep	Ingraham, Lloyd	Lloyd Ingraham	tt0009047
1746500178	1924	1924	In Fast Company	In Fast Company	Horne, James W.	James W. Horne	tt0123906
1746532628	1914	1914	Three Weeks	Three Weeks	Vekroff, Perry N.	Perry N. Vekroff	tt0004698
1746524823	1923	1923	The Six-Fifty	The Six Fifty	Ross, Nat	Nat Ross	tt0014479
1746584654	1929	1929	From Headquarters	From Headquarters	Bretherton, Howard	Howard Bretherton	tt0019906
1746577777	1938	1938	Arsène Lupin Returns	Arsène Lupin Returns	Fitzmaurice, Geo.	George Fitzmaurice	tt0029884
1746198730	1969	1969	The Girl Who Knew Too Much	The Girl Who Knew Too Much	Lyon, Francis D.	Francis D. Lyon	tt0064370
1746474834	1947	1947	Robin Hood of Texas	Robin Hood of Texas	Selander, Lesley	Lesley Selander	tt0039780
1746435336	1999	1999	Flawless	Flawless	Schumacher, Joel	Joel Schumacher	tt0155711
1745358527	2010	2010	The Crazies	The Crazies	Eisner, Breck	Breck Eisner	tt0455407
1746571576	1932	1932	A Successful Calamity	A Successful Calamity	Adolfi, John G.	John G. Adolfi	tt0023537
1746423562	1991	1991	The Pit and the Pendulum	The Pit and the Pendulum	Gordon, Stuart	Stuart Gordon	tt0100369
1746578680	1962	1962	The Cabinet of Caligari	The Cabinet of Caligari	Kay, Roger	Roger Kay	tt0055819
1746509264	1924	1924	The Perfect Flapper	The Perfect Flapper	Dillon, John Francis	John Francis Dillon	tt0015221
1746420806	1982	1981	Evilspeak	Evilspeak	Weston, Eric	Eric Weston	tt0082346
1746570213	1933	1933	Sailor's Luck	Sailor's Luck	Walsh, Raoul	Raoul Walsh	tt0024518
1746577779	1936	1936	Everyman's Law	Everyman's Law	Ray, Albert	Albert Ray	tt0027588
1746217332	1967	1967	The Girl, The Body, and The Pill	The Girl, the Body, and the Pill	Lewis, Herschell Gordon	Herschell Gordon Lewis	tt0061712
1746569579	1953	1953	Take Me to Town	Take Me to Town	Sirk, Douglas	Douglas Sirk	tt0046397
1746574066	1956	1956	Forbidden Planet	Forbidden Planet	Wilcox, Fred McLeod	Fred M. Wilcox	tt0049223
1746503467	1922	1922	The Five Dollar Baby	The Five Dollar Baby	Beaumont, Harry	Harry Beaumont	tt0013131
1746497218	1919	1919	Human Passions	Human Passions	Tyrol, Jacques	Jacques Tyrol	tt0190468
1746474519	1948	1948	Belle Starr's Daughter	Belle Starr's Daughter	Selander, Lesley	Lesley Selander	tt0040154
1746523814	1910	1910	A Sleep Walking Cure	A Sleep Walking Cure			tt1371147
1746509040	1950	1950	Girls' School	Girls' School	Landers, Lew	Lew Landers	tt0042507
1746503416	1918	1918	The World for Sale	The World for Sale	Blackton, J. Stuart	J. Stuart Blackton	tt0009840
1746515108	1920	1920	Nineteen and Phyllis	Nineteen and Phyllis	De Grasse, Joseph	Joseph De Grasse	tt0012503
1746465166	1947	1947	News Hounds	News Hounds	Beaudine, William	William Beaudine	tt0039656
1745341030	2002	2002	Far from Heaven	Far from Heaven	Haynes, Todd	Todd Haynes	tt0297884
1746583044	1936	1936	Smartest Girl in Town	Smartest Girl in Town	Santley, Joseph	Joseph Santley	tt0028270
1746570396	1934	1934	Sons of Steel	Sons of Steel	Lamont, Charles	Charles Lamont	tt0027026
1746501931	1915	1915	At the Flood-Tide	At the Flood Tide	Oliver, Guy	Guy Oliver	tt0413775
1746581241	1951	1951	Secrets of Beauty	Secrets of Beauty	Kenton, Erle C.	Erle C. Kenton	tt0228830
1746419791	1987	1987	Flowers in the Attic	Flowers in the Attic	Bloom, Jeffrey; Bloom, Jeffrey	Jeffrey Bloom	tt0093036
1746513793	1923	1923	Mighty Lak' a Rose	Mighty Lak' a Rose	Carewe, Edwin	Edwin Carewe	tt0014276
1746520728	1949	1949	Ranger of Cherokee Strip	Ranger of Cherokee Strip	Ford, Philip	Philip Ford	tt0041783
1746422119	1995	1995	Mallrats	Mallrats	Smith, Kevin	Kevin Smith	tt0113749
1746583832	1929	1929	Skin Deep	Skin Deep	Enright, Ray	Ray Enright	tt0020415
1746257935	1976	1976	Mother, Jugs & Speed	Mother, Jugs & Speed	Yates, Peter	Peter Yates	tt0074923
1746504025	1917	1917	'49-'17	'49 '17	Baldwin, Ruth Ann	Ruth Ann Baldwin	tt0007610
1746580380	1938	1938	Squadron of Honor	Squadron of Honor	Coleman, C. C.	Charles C. Coleman	tt0030785
1746562410	1953	1953	The Master of Ballantrae	The Master of Ballantrae	Keighley, William	William Keighley	tt0046054
1746419401	1982	1982	Smithereens	Smithereens	Seidelman, Susan	Susan Seidelman	tt0084698
1746424045	1940	1940	Earthbound	Earthbound	Pichel, Irving	Irving Pichel	tt0032427
1746514576	1916	1916	The Mediator	The Mediator	Turner, Otis	Otis Turner	tt0007051
1745373422	2008	2008	Baghead	Baghead	Duplass, Jay ; Duplass, Mark	Jay Duplass	tt0923600
1746574929	1938	1938	Air Devils	Air Devils	Rawlins, John	John Rawlins	tt0029847
1746583176	1936	1936	With Love and Kisses	With Love and Kisses	Goodwins, Les	Leslie Goodwins	tt0029787
1746581521	1951	1951	The Man with a Cloak	The Man with a Cloak	Markle, Fletcher	Fletcher Markle	tt0043782
1746200351	1966	1966	A Funny Thing Happened on the Way to the Foru	A Funny Thing Happened on the Way to the	Lester, Richard	Richard Lester	tt0060438

1746584480	1929	1929	Jealousy	Jealousy	De Limur, Jean	Jean de Limur	tt0020043
1746573758	1959	1959	Face of Fire	Face of Fire	Band, Albert	Albert Band	tt0052793
1746492712	1944	1944	Trocadero	Trocadero	Nigh, William	William Nigh	tt0037399
1746575020	1934	1934	Lazy River	Lazy River	Seitz, George B.	George B. Seitz	tt0025375
1746575541	1931	1931	Strangers May Kiss	Strangers May Kiss	Fitzmaurice, George	George Fitzmaurice	tt0022435
1746437412	1942	1942	Mexican Spitfire's Elephant	Mexican Spitfire's Elephant	Goodwins, Leslie	Leslie Goodwins	tt0035061
1746501774	1915	1915	Courtmartialed	Courtmartialed	Paton, Stuart	Stuart Paton	tt0005148
1746454581	1998	1998	Living Out Loud	Living Out Loud	LaGravenese, Richard	Richard LaGravenese	tt0120722
1746577228	1950	1950	The Next Voice You Hear	The Next Voice You Hear...	Wellman, William A.	William A. Wellman	tt0042786
1746485526	1919	1919	Bonds of Love	Bonds of Love	Barker, Reginald	Reginald Barker	tt0009952
1746453918	1996	1996	Mother	Mother	Brooks, Albert	Albert Brooks	tt0117091
1746571350	1932	1932	The Lost Squadron	The Lost Squadron	Archainbaud, George	George Archainbaud	tt0023151
1746501238	1915	1915	Over Night	Over Night	Young, James	James Young	tt0005856
1746577634	1936	1936	The Sky Parade	Sky Parade	Lovering, Otho	Otho Lovering	tt0028264
1746569806	1958	1958	Ride a Crooked Trail	Ride a Crooked Trail	Hibbs, Jesse	Jesse Hibbs	tt0052135
1746503617	1918	1918	The Rough Lover	The Rough Lover	De Grasse, Joseph	Joseph De Grasse	tt0009570
1746494244	1910	1910	Western Chivalry	Western Chivalry		Gilbert M. 'Broncho Billy' Anderson	tt0001448
1746575042	1936	1936	Love on the Run	Love on the Run	Van Dyke, W. S.	W.S. Van Dyke	tt0027914
1746475254	1947	1947	Deep Valley	Deep Valley	Negulesco, Jean	Jean Negulesco	tt0039308
1746392918	1984	1984	Cloak & Dagger	Cloak & Dagger	Franklin, Richard	Richard Franklin	tt0087065
1746509346	1926	1925	Red Blood	Red Blood	McGowan, J. P.	J.P. McGowan	tt0017316
1746584920	1940	1940	Wyoming	Wyoming	Thorpe, Richard	Richard Thorpe	tt0033276
1746571677	1931	1931	My Past	My Past	Del Ruth, Roy	Roy Del Ruth	tt0022177
1746562477	1955	1955	The Man from Bitter Ridge	The Man from Bitter Ridge	Arnold, Jack; Daniels, Jack	Jack Arnold	tt0048341
1746570825	1933	1933	Storm at Daybreak	Storm at Daybreak	Boleslavsky, Richard	Richard Boleslawski	tt0024616
1746582876	1936	1936	Song and Dance Man	Song and Dance Man	Dwan, Allan	Allan Dwan	tt0028280
1746464800	1942	1942	The Old Chisholm Trail	The Old Chisholm Trail	Clifton, Elmer	Elmer Clifton	tt0036232
1746570464	1934	1934	Murder in Trinidad	Murder in Trinidad	King, Louis	Louis King	tt0025526
1746576991	1963	1963	Harbor Lights	Harbor Lights	Dexter, Maury	Maury Dexter	tt0057125
1746583345	1951	1951	Pickup	Pickup	Haas, Hugo	Hugo Haas	tt0043919
1746582926	1935	1935	The Girl Who Came Back	The Girl Who Came Back	Lamont, Charles	Charles Lamont	tt0026412
1746567796	1952	1952	Here Come the Nelsons	Here Come the Nelsons	Cordova, Frederick de	Frederick De Cordova	tt0044701
1746435865	1991	1991	29th Street	29th Street	Gallo, George	George Gallo	tt0101252
1746500228	1946	1946	Landrush	Landrush	Keays, Vernon	Vernon Keays	tt0038681
1746582601	1936	1936	Reunion	Reunion	Taurog, Norman	Norman Taurog	tt0028173
1746508380	1926	1925	False Pride	False Pride	Dierker, Hugh	Hugh Dierker	tt0131380
1746454843	1946	1946	God's Country	God's Country	Tansey, Robert	Robert Emmett Tansey	tt0038567
1746422314	1998	1998	The Waterboy	The Waterboy	Coraci, Frank	Frank Coraci	tt0120484
1746508730	1925	1925	That Old Gang of Mine	That Old Gang of Mine	Tully, May	May Tully	tt0016419
1746577135	1961	1961	Voyage to the Bottom of the Sea	Voyage to the Bottom of the Sea	Allen, Irwin	Irwin Allen	tt0055608
1746454847	1941	1941	The Lone Rider Rides On	The Lone Rider Rides On	Newfield, Samuel	Sam Newfield	tt0033844
1746424044	1943	1943	Beyond the Last Frontier	Beyond the Last Frontier	Bretherton, Howard	Howard Bretherton	tt0035676
1746574689	1935	1935	Stormy	Stormy	Friedlander, Louis	Lew Landers	tt0027047
1746583304	1951	1951	Pride of Maryland	The Pride of Maryland	Ford, Philip	Philip Ford	tt0043933
1746583231	1951	1951	Quebec	Quebec	Templeton, George	George Templeton	tt0043946
1746572279	1931	1931	Maker of Men	Maker of Men	Sedgwick, Edward	Edward Sedgwick	tt0022109
1746508495	1924	1924	American Manners	American Manners	Horne, James W.	James W. Horne	tt0014673
1746572857	1931	1931	Puss in Boots	Puss in Boots		M.J. Weisfeldt	tt6427418
1745282819	2005	2005	The Producers	The Producers	Stroman, Susan	Susan Stroman	tt0395251
1746582501	1935	1935	The Whole Town's Talking	The Whole Town's Talking	Ford, John	John Ford	tt0027214
1746516623	1922	1922	Reckless Chances	Reckless Chances	McGowan, J. P.	J.P. McGowan	tt0013537
1746491873	1946	1946	Shadows Over Chinatown	Shadows Over Chinatown	Morse, Terry	Terry O. Morse	tt0038929
1746508374	1926	1926	A Trip to Chinatown	A Trip to Chinatown	Kerr, Robert P.	Robert P. Kerr	tt0017484
1746235002	1967	1967	The Love-Ins	The Love Ins	Dreifuss, Arthur	Arthur Dreifuss	tt0061922
1746570434	1934	1934	A Demon for Trouble	A Demon for Trouble	Hill, Bob	Robert F. Hill	tt0025041
1746573798	1960	1960	G.I. Blues	G.I. Blues	Taurog, Norman	Norman Taurog	tt0053848
1746583951	1930	1930	Mother's Cry	Mothers Cry	Henley, Hobart	Hobart Henley	tt0021159

1746565984	1954	1953	El Alamein	El Alaméin	Sears, Fred F.	Fred F. Sears	tt0045725
1746502404	1915	1915	The Runaway Wife	The Runaway Wife	Buel, Kenean	Kenean Buel	tt0005989
1746421071	1992	1992	Titanica	Titanica	Low, Stephen	Stephen Low	tt0105601
1746508376	1927	1927	The Last Trail	The Last Trail	Seiler, Lewis	Lewis Seiler	tt0018081
1746500089	1924	1924	So This Is Marriage	So This Is Marriage?	Henley, Hobart	Hobart Henley	tt0015348
1746555477	1955	1955	Murder Is My Beat	Murder Is My Beat	Ulmer, Edgar G.	Edgar G. Ulmer	tt0048396
1746578471	1963	1962	Young Guns of Texas	Young Guns of Texas	Dexter, Maury	Maury Dexter	tt0056710
1745341741	2003	2003	The Core	The Core	Amiel, Jon	Jon Amiel	tt0298814
1746570075	1952	1952	Fort Osage	Fort Osage	Selander, Lesley	Lesley Selander	tt0044630
1746504628	1917	1918	Cannibals and Carnivals	Cannibals and Carnivals	Moore, Vin	Vin Moore	tt0328928
1746533045	1914	1914	The Lust of the Red Man	The Lust of the Red Man	Hamilton, Gilbert P.	Gilbert P. Hamilton	tt0004276
1746393554	1986	1985	The Man Who Envied Women	The Man Who Envied Women	Rainer, Yvonne	Yvonne Rainer	tt0131467
1745358590	2008	2008	We Are Wizards	We Are Wizards	Koury, Josh	Josh Koury	tt1183705
1745358041	2008	2008	The Cool School	The Cool School	Neville, Morgan	Morgan Neville	tt1031225
1745293784	2007	2007	Year of the Dog	Year of the Dog	White, Mike	Kevin Lau	tt1035516
1745302519	2005	2004	Girl Play	Girl Play	Friedlander, Lee	Lee Friedlander	tt0416773
1745342653	2002	2001	Plan B	Plan B	Yaitanes, Greg		tt0391402
1746578474	1950	1950	Under Mexicali Stars	Under Mexicali Stars	Blair, George	George Blair	tt0043085
1746577286	1950	1949	Sons of New Mexico	Sons of New Mexico	English, John	John English	tt0041901
1746500362	1949	1950	The Kid from Gower Gulch	The Kid from Gower Gulch	Drake, Oliver	Oliver Drake	tt0041544
1746508280	1949	1949	Rusty's Birthday	Rusty's Birthday	Friedman, Seymour	Seymour Friedman	tt0041834
1746530666	1949	1949	Navajo Trail Raiders	Navajo Trail Raiders	Springsteen, R. G.	R.G. Springsteen	tt0041685
1746474959	1948	1948	Outlaw Brand	Outlaw Brand	Hillyer, Lambert	Lambert Hillyer	tt0040677
1746508459	1948	1948	Silver Trails	Silver Trails	Cabanne, Christy	Christy Cabanne	tt0040790
1746438325	1945	1945	Fighting Bill Carson	Fighting Bill Carson	Newfield, Sam	Sam Newfield	tt0037697
1746454249	1945	1945	A Game of Death	A Game of Death	Wise, Robert	Robert Wise	tt0038549
1746454398	1943	1943	Tornado	Tornado	Berke, William	William Berke	tt0036451
1746438219	1943	1943	Revenge of the Zombies	Revenge of the Zombies	Sekely, Steve	Steve Sekely	tt0036307
1746583793	1929	1929	Jazz Heaven	Jazz Heaven	Brown, Melville	Melville W. Brown	tt0020042
1746584846	1929	1929	The Laughing Lady	The Laughing Lady	Schertzinger, Victor	Victor Schertzinger	tt0020082
1746514086	1927	1927	I'll Be There	I'll Be There	Yaconelli, Frank	Frank Yaconelli	tt0361731
1746508467	1926	1926	Sheriff's Girl	Sheriff's Girl	Wilson, Ben	Ben F. Wilson	tt0339662
1746507793	1924	1924	Horse Shoe Luck	Horseshoe Luck	Franz, Joseph	Joseph Franz	tt0356703
1746585430	1940	1940	Dr. Cyclops	Dr. Cyclops	Schoedsack, Ernest B.	Ernest B. Schoedsack	tt0032412
1746580612	1937	1936	Death in the Air	Death in the Air	Clifton, Elmer	Elmer Clifton	tt0028776
1746577223	1962	1962	Hand of Death	Hand of Death	Nelson, Gene	Gene Nelson	tt0056055
1746569739	1957	1957	Voodoo Island	Voodoo Island	LeBorg, Reginald	Reginald Le Borg	tt0051173
1746566297	1955	1955	Five Guns West	Five Guns West	Corman, Roger	Roger Corman	tt0048078
1746563856	1952	1952	The Gunman	The Gunman	Collins, Lewis	Lewis D. Collins	tt0044678
1746571069	1952	1952	South Pacific Trail	South Pacific Trail	Witney, William	William Witney	tt0045182
1769971164	1969	1969	Snow Job	Snow Job	Shiffen, Arlo	Don Walters	tt3621648
1746208189	1969	1969	Charles Lloyd--Journey Within	Charles Lloyd: Journey Within	Sherman, Eric	Eric Sherman	tt0190297
1769970590	1968	1968	Diary of Knockers McCalla	The Diary of Knockers McCalla		Barry Mahon	tt0216702
1769970568	1968	1968	An Angle of Love	An Angle of Love	Hennigar, William K.	William K. Hennigar	tt0210509
1746186273	1967	1966	Skin Deep in Love	Skin Deep in Love	Sarno, Joe	Joseph W. Sarno	tt0060649
1769970410	1964	1964	Sextet	Sextet		Bud Irwin	tt0445705
1746410707	1993	1993	Family Prayers	Family Prayers	Rosenfelt, Scott	Scott M. Rosenfelt	tt0106860
1746577570	1933	1933	The Warrior's Husband	The Warrior's Husband	Lang, Walter	Walter Lang	tt0024753
1746575901	1932	1932	The Spirit of the West	Spirit of the West	Brower, Otto	Otto Brower	tt0023502
1746493453	1910	1910	A Texas Joke	A Texas Joke		Gaston Méliès	tt0822866
1746563019	1952	1952	African Treasure	African Treasure	Beebe, Ford	Ford Beebe	tt0044332
1746508931	1925	1925	Renegade Holmes, M. D.	Renegade Holmes, M.D.	Wilson, Ben	Ben F. Wilson	tt0156016
1746574567	1935	1935	Get That Man	Get That Man	Bennet, Spencer Gordon	Spencer Gordon Bennet	tt0026405
1746211224	1972	1972	Heat	Heat	Morrissey, Paul	Paul Morrissey	tt0068688
1746512375	1915	1915	Mary's Lamb	Mary's Lamb	MacKenzie, Donald	Donald MacKenzie	tt0005721
1746581877	1928	1928	Battling Bookworm	The Battling Bookworm			tt0315812
1746513234	1923	1923	When Law Comes to Hades	When Law Comes to Hades			tt0014597

1746582160	1929	1929	His Glorious Night	His Glorious Night	Barrymore, Lionel	Lionel Barrymore	tt0019988
1746410148	1989	1988	Fright Night: Part 2	Fright Night Part 2	Wallace, Tommy Lee	Tommy Lee Wallace	tt0097390
1746520480	1927	1927	The Unknown	The Unknown	Browning, Tod	Tod Browning	tt0018528
1746235887	1975	1974	Dark Star	Dark Star	Carpenter, John	John Carpenter	tt0069945
1746394376	1984	1984	Stop Making Sense	Stop Making Sense	Demme, Jonathan	Jonathan Demme	tt0088178
1746583991	1935	1935	The Last Days of Pompeii	The Last Days of Pompeii	Schoedsack, Ernest B.	Ernest B. Schoedsack	tt0026605
1746583114	1935	1935	Captain Hurricane	Captain Hurricane	Robertson, John	John S. Robertson	tt0026176
1746562399	1956	1955	The Phantom from 10,000 Leagues	The Phantom from 10,000 Leagues	Milner, Dan	Dan Milner	tt0049615
1746576731	1931	1931	Subway Express	Subway Express	Newmeyer, Fred	Fred C. Newmeyer	tt0022446
1746573747	1958	1958	In Love and War	In Love and War	Dunne, Philip	Philip Dunne	tt0051770
1746235217	1977	1977	The Turning Point	The Turning Point	Ross, Herbert	Herbert Ross	tt0076843
1746434930	1998	1998	Monument Ave.	Monument Ave.	Demme, Ted	Ted Demme	tt0119802
1746453479	1942	1942	Wings for the Eagle	Wings for the Eagle	Bacon, Lloyd	Lloyd Bacon	tt0035564
1746554364	1964	1964	Dark Purpose	Dark Purpose	Marshall, George	George Marshall	tt0058234
1746502040	1916	1916	The Blacklist	The Blacklist	Mille, William C. de	William C. de Mille	tt0006437
1746569809	1958	1958	The Brain Eaters	The Brain Eaters	VeSota, Bruno	Bruno VeSota	tt0051432
1746483448	1918	1918	Flower of the Dusk	Flower of the Dusk	Collins, John H.	John H. Collins	tt0009080
1746570345	1934	1934	Kentucky Kernels	Kentucky Kernels	Stevens, George	George Stevens	tt0025345
1746465148	1946	1946	Moon Over Montana	Moon Over Montana	Drake, Oliver	Oliver Drake	tt0038749
1746514050	1913	1913	The Count of Monte Cristo	The Count of Monte Cristo	Porter, Edwin S.; Golden, Joseph	Joseph A. Golden	tt0002767
1746574304	1950	1950	When Willie Comes Marching Home	When Willie Comes Marching Home	Ford, John	John Ford	tt0043129
1746474841	1945	1945	Wonder Man	Wonder Man	Humberstone, Bruce	H. Bruce Humberstone	tt0038260
1746507893	1950	1950	Crooked River	Crooked River	Carr, Thomas	Thomas Carr	tt0042356
1746574651	1935	1935	Alias John Law	Alias John Law	Bradbury, Robert N.	Robert N. Bradbury	tt0026052
1746584531	1929	1929	Riders of the Rio Grande	Riders of the Rio Grande	McGowan, J. P.	J.P. McGowan	tt0020329
1746513613	1927	1927	Ain't Love Funny?	Ain't Love Funny?	Andrews, Del	Del Andrews	tt0017595
1745358510	2002	2002	Insomnia	Insomnia	Nolan, Christopher	Christopher Nolan	tt0278504
1746503802	1919	1919	The Follies Girl	The Follies Girl	Dillon, Jack	John Francis Dillon	tt0010133
1746577946	1938	1938	The Lone Wolf in Paris	The Lone Wolf in Paris	Rogell, Albert S.	Albert S. Rogell	tt0030383
1746504208	1918	1918	Broken Ties	Broken Ties	Ashley, Arthur	Arthur Ashley	tt0184307
1746515013	1923	1923	The Red Warning	The Red Warning	Bradbury, Robert North	Robert N. Bradbury	tt0014400
1746566263	1953	1953	Private Eyes	Private Eyes	Bernds, Edward	Edward Bernds	tt0046210
1746465022	1942	1942	Bowery at Midnight	Bowery at Midnight	Fox, Wallace	Wallace Fox	tt0034546
1746580832	1939	1939	The Magnificent Fraud	The Magnificent Fraud	Florey, Robert	Robert Florey	tt0031604
1745293593	2005	2005	Shopgirl	Shopgirl	Tucker, Anand	Anand Tucker	tt0338427
1746520975	1949	1949	Special Agent	Special Agent	Thomas, William C.	William C. Thomas	tt0041910
1746501468	1915	1915	The White Pearl	The White Pearl	Porter, Edwin S.; Ford, Hugh	Hugh Ford	tt0006279
1746513164	1910	1910	The Pony Express Rider	The Pony Express Rider		Gilbert M. 'Broncho Billy' Anderson	tt0001365
1746549835	1955	1955	The Cobweb	The Cobweb	Minnelli, Vincente	Vincente Minnelli	tt0047944
1746454614	1941	1941	Man from Montana	Man from Montana	Taylor, Ray	Ray Taylor	tt0033872
1746580743	1935	1935	McFadden's Flats	McFadden's Flats	Murphy, Ralph	Ralph Murphy	tt0026693
1746436368	1943	1943	The Powers Girl	The Powers Girl	McLeod, Norman	Norman Z. McLeod	tt0035206
1746580833	1936	1936	Robin Hood of El Dorado	Robin Hood of El Dorado	Wellman, William A.	William A. Wellman	tt0028197
1746520423	1950	1950	No Way Out	No Way Out	Mankiewicz, Joseph L.	Joseph L. Mankiewicz	tt0042792
1746502217	1915	1915	What Happened to Father	What Happened to Father	Williams, C. Jay	C.J. Williams	tt0006252
1746577787	1935	1935	Trail's End	Trails End	Herman, Al	Albert Herman	tt0027129
1746584463	1931	1931	Night Life in Reno	Night Life in Reno	Cannon, Raymond	Raymond Cannon	tt0151751
1746504391	1920	1920	The Gilded Dream	The Gilded Dream	Sturgeon, Rollin	Rollin S. Sturgeon	tt0011225
1746566020	1955	1954	So This Is Paris	So This Is Paris	Quine, Richard	Richard Quine	tt0047508
1746582338	1928	1928	The Border Patrol	The Border Patrol	Hogan, James P.	James P. Hogan	tt0018715
1746508028	1949	1949	Roughshod	Roughshod	Robson, Mark	Mark Robson	tt0041827
1746500100	1946	1946	Dark Alibi	Dark Alibi	Karlson, Phil	Phil Karlson	tt0038452
1746186243	1967	1967	Clambake	Clambake	Nadel, Arthur H.	Arthur H. Nadel	tt0061489
1746500641	1950	1950	Experiment Alcatraz	Experiment Alcatraz	Cahn, Edward L.	Edward L. Cahn	tt0042442
1746491983	1948	1948	I Surrender Dear	I Surrender Dear	Dreifuss, Arthur	Arthur Dreifuss	tt0178610
1746474416	1948	1948	Pitfall	Pitfall	DeToth, Andre	André De Toth	tt0040695
1746199841	1967	1967	Hour of the Gun	Hour of the Gun	Sturges, John	John Sturges	tt0061787

1746583511	1931	1931	The Lady from Nowhere	The Lady from Nowhere	Thorpe, Richard	Richard Thorpe	tt0022044
1746421063	1986	1986	Wise Guys	Wise Guys	De Palma, Brian	Brian De Palma	tt0092226
1746523325	1915	1915	To Cherish and Protect	To Cherish and Protect	Humphrey, William	William Humphrey	tt0006164
1746496818	1918	1918	All Night	All Night	Powell, Paul	Paul Powell	tt0008822
1746574832	1933	1933	Trailing North	Trailing North	McCarthy, John P.	John P. McCarthy	tt0024690
1746559842	1964	1964	The Thin Red Line	The Thin Red Line	Marton, Andrew	Andrew Marton	tt0058648
1746495616	1919	1919	The Other Man's Wife	The Other Man's Wife	Harbaugh, Carl	Carl Harbaugh	tt0010535
1746503640	1919	1919	The Busher	The Busher	Storm, Jerome	Jerome Storm	tt0009976
1746561405	1932	1932	The Racing Strain	The Racing Strain	Storm, Jerome	Jerome Storm	tt0023366
1746507876	1925	1925	Big Pal	Big Pal	Adolfi, John G.	John G. Adolfi	tt0015623
1746564945	1952	1952	The Devil Makes Three	The Devil Makes Three	Marton, Andrew	Andrew Marton	tt0044547
1746438151	1943	1943	Hitler's Children	Hitler's Children	Dmytryk, Edward; Reis, Irving	Edward Dmytryk	tt0034856
1769969997	1911	1911	Over the Hills	Over the Hills	Smiley, Joseph W.; Tucker, George Lo	Joseph W. Smiley	tt0001824
1746583023	1935	1935	Seven Keys to Baldpate	Seven Keys to Baldpate	Hamilton, William; Killy, Edward	William Hamilton	tt0026978
1746584739	1929	1929	The Argyle Case	The Argyle Case	Bretherton, Howard	Howard Bretherton	tt0019647
1746409332	1990	1990	Cadillac Man	Cadillac Man	Donaldson, Roger	Roger Donaldson	tt0099204
1746584363	1939	1939	Legion of Lost Flyers	Legion of Lost Flyers	Cabanne, Christy	Christy Cabanne	tt0031562
1746583107	1937	1937	High Flyers	High Flyers	Cline, Edward	Edward F. Cline	tt0028998
1746199549	1967	1967	My Body Hungers	My Body Hungers	Sarno, Joe	Joseph W. Sarno	tt0057240
1746235037	1979	1979	Dracula	Dracula	Badham, John	John Badham	tt0079073
1746186086	1965	1965	Love and Kisses	Love and Kisses	Nelson, Ozzie	Ozzie Nelson	tt0059408
1746569684	1958	1958	The Geisha Boy	The Geisha Boy	Tashlin, Frank	Frank Tashlin	tt0051649
1746475540	1944	1944	The Pearl of Death	The Pearl of Death	Neill, Roy William	Roy William Neill	tt0037168
1746513814	1916	1916	My Country First	My Country First	Terriss, Tom	Tom Terriss	tt0007103
1745271907	2000	2000	What Planet Are You From?	What Planet Are You From?	Nichols, Mike	Mike Nichols	tt0181151
1746570233	1933	1933	The Important Witness	The Important Witness	Newfield, Sam	Sam Newfield	tt0024174
1746435109	1943	1943	The Chance of a Lifetime	The Chance of a Lifetime	Castle, William	William Castle	tt0035726
1746497453	1918	1918	Midnight Madness	Midnight Madness	Julian, Rupert	Rupert Julian	tt0009371
1746584881	1940	1940	George Washington Carver	George Washington Carver	Parker, Ben	Ben Parker	tt0032517
1746569692	1954	1954	The Long Wait	The Long Wait	Saville, Victor	Victor Saville	tt0047190
1746421266	1996	1996	Bottle Rocket	Bottle Rocket	Anderson, Wes	Wes Anderson	tt0115734
1746582828	1938	1938	Thunder in the Desert	Thunder in the Desert	Newfield, Sam	Sam Newfield	tt0030870
1746585441	1939	1939	Feud of the Range	Feud of the Range	Webb, Harry S.	Harry S. Webb	tt0031301
1746492239	1946	1946	I Ring Doorbells	I Ring Doorbells	Strayer, Frank	Frank R. Strayer	tt0038627
1746467515	1917	1917	Life's Whirlpool	Life's Whirlpool	Barrymore, Lionel	Lionel Barrymore	tt0008183
1746584231	1939	1939	The Girl from Mexico	The Girl from Mexico	Goodwins, Leslie	Leslie Goodwins	tt0031370
1769971120	1969	1969	Joys of Georgette	Joys of Georgette			tt0222074
1745271900	2000	2000	X-Men	X Men	Singer, Bryan	Bryan Singer	tt0120903
1746553435	1965	1965	My Blood Runs Cold	My Blood Runs Cold	Conrad, William	William Conrad	tt0059487
1746465292	1943	1943	Thumbs Up	Thumbs Up	Santley, Joseph	Joseph Santley	tt0036436
1746233383	1968	1968	Speedway	Speedway	Taurog, Norman	Norman Taurog	tt0063634
1746492062	1948	1948	That Lady in Ermine	That Lady in Ermine	Lubitsch, Ernst	Ernst Lubitsch	tt0040869
1746583596	1930	1930	In the Next Room	In the Next Room	Cline, Edward	Edward F. Cline	tt0020998
1746491780	1945	1945	The Valley of Decision	The Valley of Decision	Garnett, Tay	Tay Garnett	tt0038213
1746523069	1913	1913	A Sister to Carmen	A Sister to Carmen	Gaskill, Charles L.	Charles L. Gaskill	tt0003378
1746420156	1990	1989	The Unbelievable Truth	The Unbelievable Truth		Hal Hartley	tt0100842
1769969981	1912	1912	The Flirting Husband	The Flirting Husband	Sennett, Mack	Mack Sennett	tt0002184
1746235657	1973	1973	Battle for the Planet of the Apes	Battle for the Planet of the Apes	Thompson, J. Lee	J. Lee Thompson	tt0069768
1746578486	1959	1958	Some Came Running	Some Came Running	Minnelli, Vincente	Vincente Minnelli	tt0052218
1746584370	1940	1940	The Westerner	The Westerner	Wyler, William	William Wyler	tt0033253
1746554933	1965	1965	The Monkey's Uncle	The Monkey's Uncle	Stevenson, Robert	Robert Stevenson	tt0059462
1746573650	1931	1931	Lover Come Back	Lover Come Back	Kenton, Erle C.	Erle C. Kenton	tt0022094
1746578939	1960	1959	Cuban Rebel Girls	Cuban Rebel Girls	Mahon, Barry	Barry Mahon	tt0052717
1746497008	1918	1918	Brace Up	Brace Up	Clifton, Elmer	Elmer Clifton	tt0008917
1746533182	1928	1928	The Little Snob	The Little Snob	Adolfi, John G.	John G. Adolfi	tt0019105
1746569968	1958	1958	Crash Landing	Crash Landing	Sears, Fred F.	Fred F. Sears	tt0051497
1746579053	1951	1951	The Man from Planet X	The Man from Planet X	Ulmer, Edgar G.	Edgar G. Ulmer	tt0043778

1746583125	1936	1936	Trapped by Television	Trapped by Television	Lord, Del	Del Lord	tt0028405
1746512144	1916	1916	The Making of Maddalena	The Making of Maddalena	Lloyd, Frank	Frank Lloyd	tt0007011
1746570208	1934	1934	Paris Interlude	Paris Interlude	Marin, Edwin L.	Edwin L. Marin	tt0025629
1746464791	1941	1940	Victory	Victory	Cromwell, John	John Cromwell	tt0033220
1746524547	1916	1916	Little Meena's Romance	Little Meena's Romance	Powell, Paul	Paul Powell	tt0006933
1746584700	1930	1930	The Bad One	The Bad One	Fitzmaurice, George	George Fitzmaurice	tt0020663
1746566192	1956	1956	The Harder They Fall	The Harder They Fall	Robson, Mark	Mark Robson	tt0049291
1746198722	1965	1965	The Loved One	The Loved One	Richardson, Tony	Tony Richardson	tt0059410
1746492434	1947	1947	Two Blondes and a Redhead	Two Blondes and a Redhead	Dreifuss, Arthur	Arthur Dreifuss	tt0178956
1746574945	1935	1935	Bonnie Scotland	Bonnie Scotland	Horne, James W.	James W. Horne	tt0026126
1746504611	1918	1918	Beyond the Law	Beyond the Law	Marston, Theodore	Theodore Marston	tt0182787
1746433969	1997	1997	George of the Jungle	George of the Jungle	Weisman, Sam	Sam Weisman	tt0119190
1746464957	1945	1945	Pursuit to Algiers	Pursuit to Algiers	Neill, Roy William	Roy William Neill	tt0038008
1746501025	1922	1922	His Wife's Husband	His Wife's Husband	Webb, Kenneth	Kenneth S. Webb	tt0013240
1746185157	1965	1965	Joy in the Morning	Joy in the Morning	Segal, Alex	Alex Segal	tt0059339
1746418886	1940	1940	Susan and God	Susan and God	Cukor, George	George Cukor	tt0033117
1746571570	1931	1931	Woman Hungry	Woman Hungry	Badger, Clarence	Clarence G. Badger	tt0021728
1746570451	1934	1934	One Night of Love	One Night of Love	Schertzinger, Victor	Victor Schertzinger	tt0025601
1746584258	1936	1936	Sing, Baby, Sing	Sing, Baby, Sing	Lanfield, Sidney	Sidney Lanfield	tt0028255
1746502937	1923	1923	Woman-Proof	Woman Proof	Green, Alfred E.	Alfred E. Green	tt0014625
1746550126	1955	1955	The Looters	The Looters	Biberman, Abner	Abner Biberman	tt0048313
1746580646	1936	1936	Down to the Sea	Down to the Sea	Collins, Lewis D.	Lewis D. Collins	tt0027544
1746500035	1948	1948	Romance on the High Seas	Romance on the High Seas	Curtiz, Michael	Michael Curtiz	tt0040745
1746421556	1992	1992	A League of Their Own	A League of Their Own	Marshall, Penny	Penny Marshall	tt0104694
1746515361	1920	1920	Through Eyes of Men	Through Eyes of Men	Taylor, Charles A.	Charles A. Taylor	tt0011770
1745342912	2001	2001	Sweet November	Sweet November	O'Connor, Pat	Pat O'Connor	tt0230838
1746498295	1917	1917	When Love Was Blind	When Love Was Blind	Sullivan, Frederick	Frederick Sullivan	tt0184011
1746508078	1925	1925	The Call of Courage	The Call of Courage	Smith, Clifford S.	Clifford Smith	tt0015664
1746585280	1930	1930	The Utah Kid	The Utah Kid	Thorpe, Richard	Richard Thorpe	tt0021509
1746514429	1916	1916	Civilization's Child	Civilization's Child	Giblyn, Charles	Charles Giblyn	tt0157470
1745342694	2004	2004	Ladder 49	Ladder 49	Russell, Jay	Jay Russell	tt0349710
1746423037	1995	1995	The Doom Generation	The Doom Generation	Araki, Gregg	Gregg Araki	tt0112887
1746394249	1987	1987	Ishtar	Ishtar	May, Elaine	Elaine May	tt0093278
1746420106	1987	1987	Predator	Predator	McTiernan, John	John McTiernan	tt0093773
1746475651	1948	1947	The Lady from Shanghai	The Lady from Shanghai	Welles, Orson	Orson Welles	tt0040525
1746577551	1934	1934	The Thin Man	The Thin Man	Van Dyke, W. S.	W.S. Van Dyke	tt0025878
1746520483	1927	1926	The Little Firebrand	The Little Firebrand	Hutchison, Charles	Charles Hutchison	tt0127662
1746553653	1964	1964	Cheyenne Autumn	Cheyenne Autumn	Ford, John	John Ford	tt0057940
1746524013	1910	1910	A Self-Made Hero	A Self Made Hero		Harry Solter	tt0201076
1746578878	1951	1951	Mr. Belvedere Rings the Bell	Mr. Belvedere Rings the Bell	Koster, Henry	Henry Koster	tt0043820
1746408661	1988	1988	Young Guns	Young Guns	Cain, Christopher	Christopher Cain	tt0096487
1746438970	1942	1942	There's One Born Every Minute	There's One Born Every Minute	Young, Harold	Harold Young	tt0035427
1746574017	1961	1961	Shangri-La	Shangri La			tt0148786
1746559912	1965	1965	Shenandoah	Shenandoah	McLaglen, Andrew V.	Andrew V. McLaglen	tt0059711
1746555742	1957	1957	Island in the Sun	Island in the Sun	Rossen, Robert	Robert Rossen	tt0050549
1746574730	1934	1934	Here Is My Heart	Here Is My Heart	Tuttle, Frank	Frank Tuttle	tt0025239
1746582849	1937	1937	Girl Overboard	Girl Overboard	Salkow, Sidney	Sidney Salkow	tt0027674
1746563972	1953	1952	Ride the Man Down	Ride the Man Down	Kane, Joseph	Joseph Kane	tt0045086
1746585494	1939	1939	Texas Stampede	Texas Stampede	Nelson, Sam	Sam Nelson	tt0032013
1746218147	1966	1966	The Hot Pearl Snatch	The Hot Pearl Snatch		Ron Mart	tt0060517
1746569568	1954	1954	Johnny Guitar	Johnny Guitar	Ray, Nicholas	Nicholas Ray	tt0047136
1746501841	1922	1922	Broadway Rose	Broadway Rose	Leonard, Robert Z.	Robert Z. Leonard	tt0012979
1769970062	1912	1912	The Redemption of "Greek Joe"	The Redemption of 'Greek Joe'		William V. Mong	tt0002456
1746513849	1927	1927	The College Widow	The College Widow	Mayo, Archie L.	Archie Mayo	tt0017767
1745342702	2008	2008	Milk	Milk	Van Sant, Gus	Gus Van Sant	tt1013753
1746475512	1947	1947	They Won't Believe Me	They Won't Believe Me	Pichel, Irving	Irving Pichel	tt0039896
1746435165	1940	1940	Little Orvie	Little Orvie	McCarey, Ray	Ray McCarey	tt0032720

1745293709	2005	2005	In the Mix	In the Mix	Underwood, Ron	Ron Underwood	tt0426615
1746570590	1934	1934	Terror of the Plains	Terror of the Plains	Webb, Harry S.	Harry S. Webb	tt0027084
1746184812	1965	1965	Nude Scrapbook	Nude Scrapbook	Mahon, Barry	Barry Mahon	tt0145136
1746496106	1921	1921	They Shall Pay	They Shall Pay	Justine, Martin	Martin Justice	tt0169316
1746439133	1941	1941	Miss Polly	Miss Polly	Guiol, Fred	Fred Guiol	tt0033912
1746585282	1929	1929	The Last Warning	The Last Warning	Leni, Paul	Paul Leni	tt0020080
1746565948	1956	1956	Santiago	Santiago	Douglas, Gordon	Gordon Douglas	tt0049714
1746454387	1940	1940	Cherokee Strip	Cherokee Strip	Selander, Lesley	Lesley Selander	tt0032330
1746235053	1975	1975	Escape to Witch Mountain	Escape to Witch Mountain	Hough, John	John Hough	tt0072951
1746574385	1961	1961	Most Dangerous Man Alive	Most Dangerous Man Alive	Dwan, Allan	Allan Dwan	tt0055197
1746436043	1941	1941	Robin Hood of the Pecos	Robin Hood of the Pecos	Kane, Joseph	Joseph Kane	tt0034120
1746456072	1997	1997	Turbulence	Turbulence	Butler, Robert	Robert Butler	tt0120390
1746567751	1933	1933	I Cover the Waterfront	I Cover the Waterfront	Cruze, James	James Cruze	tt0024151
1746422290	1993	1993	Philadelphia	Philadelphia	Demme, Jonathan	Jonathan Demme	tt0107818
1746500567	1925	1925	Hurricane Horseman	The Hurricane Horseman	Eddy, Robert	Robert Eddy	tt0015933
1746570877	1952	1952	Harem Girl	Harem Girl	Bernds, Edward	Edward Bernds	tt0044689
1746496276	1917	1917	The Submarine Eye	The Submarine Eye	Kelley, Winthrop	Winthrop Kelley	tt0008638
1746464809	1941	1941	Death Valley Outlaws	Death Valley Outlaws	Sherman, George	George Sherman	tt0033521
1746578476	1958	1958	Saddle the Wind	Saddle the Wind	Parrish, Robert	Robert Parrish	tt0050923
1746566370	1958	1958	The Bride and the Beast	The Bride and the Beast	Weiss, Adrian	Adrian Weiss	tt0051434
1746581776	1930	1930	The Santa Fe Trail	The Santa Fe Trail	Brower, Otto; Knopf, Edwin H.	Otto Brower	tt0021055
1746484723	1918	1918	The Brass Check	The Brass Check	Davis, William S.	Will S. Davis	tt0008920
1746585403	1940	1940	Sporting Blood	Sporting Blood	Simon, S. Sylvan	S. Sylvan Simon	tt0033092
1746520430	1926	1926	Double Daring	Double Daring	Thorpe, Richard	Richard Thorpe	tt0016808
1746465065	1947	1947	When a Girl's Beautiful	When a Girl's Beautiful	McDonald, Frank	Frank McDonald	tt0039980
1746219360	1975	1975	Flash and the Firecat	Flash and the Firecat	Sebastian, Ferd; Sebastian, Beverly	Beverly Sebastian	tt0072995
1746492741	1944	1944	Follow the Boys	Follow the Boys	Sutherland, Eddie	A. Edward Sutherland	tt0036832
1746491769	1946	1946	Stars on Parade	Stars on Parade	Seiden, Joseph	Joseph Seiden	tt1457603
1746454462	1941	1941	A Very Young Lady	A Very Young Lady	Schuster, Harold	Harold D. Schuster	tt0034358
1746578404	1963	1963	The Quick and the Dead	The Quick and the Dead	Totten, Robert	Robert Totten	tt0057441
1746508205	1950	1950	Caged	Caged	Cromwell, John	John Cromwell	tt0042296
1746580359	1938	1937	Every Day's a Holiday	Every Day's a Holiday	Sutherland, A. Edward	A. Edward Sutherland	tt0028843
1746578634	1959	1958	Senior Prom	Senior Prom	Rich, David Lowell	David Lowell Rich	tt0052181
1746571329	1933	1933	No Other Woman	No Other Woman	Ruben, J. Walter	J. Walter Ruben	tt0024388
1746436570	1941	1941	I Was a Prisoner on Devil's Island	I Was a Prisoner on Devil's Island	Landers, Lew	Lew Landers	tt0033742
1746500177	1926	1926	My Old Dutch	My Old Dutch	Trimble, Lawrence	Laurence Trimble	tt0017186
1746437581	1995	1995	Halloween: The Curse of Michael Myers	Halloween: The Curse of Michael Myers	Chappelle, Joe	Joe Chappelle	tt0113253
1746579007	1960	1960	The Walking Target	The Walking Target	Cahn, Edward L.	Edward L. Cahn	tt0054459
1746578577	1959	1959	Plan 9 from Outer Space	Plan 9 from Outer Space	Wood, Edward D.	Edward D. Wood Jr.	tt0052077
1746501551	1916	1916	Betrayed	Betrayed	Mitchell, Howard M.	Howard M. Mitchell	tt0186870
1746198264	1969	1968	The Best of Laurel and Hardy	The Best of Laurel and Hardy		James L. Wolcott	tt0196372
1746217503	1967	1966	Savage Pampas	Savage Pampas	Fregonese, Hugo	Hugo Fregonese	tt0060942
1746208369	1970	1970	Beyond the Valley of the Dolls	Beyond the Valley of the Dolls	Meyer, Russ	Russ Meyer	tt0065466
1746433712	1996	1996	Independence Day	Independence Day	Emmerich, Roland	Roland Emmerich	tt0116629
1746504555	1920	1920	The Star Rover	The Star Rover	Sloman, Edward	Edward Sloman	tt0197913
1746566759	1933	1933	Ex-Lady	Ex Lady	Florey, Robert	Robert Florey	tt0023993
1746581427	1960	1960	The 3rd Voice	The 3rd Voice	Cornfield, Hubert	Hubert Cornfield	tt0054380
1746532444	1922	1922	Environment	Environment	Cummings, Irving	Irving Cummings	tt0013103
1746507607	1927	1927	The Love Thrill	The Love Thrill	Webb, Millard	Millard Webb	tt0018110
1746209553	1968	1968	Slow Run	Slow Run	Kardish, Larry	Laurence Kardish	tt0228873
1746500931	1916	1916	Tom Martin - A Man	Tom Martin: A Man	Nichols, George	George Nichols	tt0467283
1745341207	2004	2004	Wicker Park	Wicker Park	McGuigan, Paul	Paul McGuigan	tt0324554
1746465182	1947	1947	Wyoming	Wyoming	Kane, Joseph	Joseph Kane	tt0040007
1746509543	1950	1950	Lucky Losers	Lucky Losers	Beaudine, William	William Beaudine	tt0042693
1746572806	1910	1910	The Cowboy's Devotion	The Cowboy's Devotion			tt1799132
1746454577	1996	1996	High School High	High School High	Bochner, Hart	Hart Bochner	tt0116531
1746581339	1951	1951	Casa Mañana	Casa Manana	Yarbrough, Jean	Jean Yarbrough	tt0043383

1746580601	1937	1937	The Cherokee Strip	The Cherokee Strip	Smith, Noel	Noel M. Smith	tt0028711
1746571082	1934	1933	Eskimo	Eskimo	Van Dyke, W. S.	W.S. Van Dyke	tt0023990
1746578800	1963	1964	Ballad of a Gunfighter	The Ballad of a Gunfighter	Ward, Bill	Bill Ward	tt0057868
1746454389	1944	1944	Outlaw Trail	Outlaw Trail	Tansey, Robert	Robert Emmett Tansey	tt0037161
1746512905	1910	1910	Pardners	Pardners	McGlynn, Frank	Frank McGlynn Sr.	tt0132405
1746185889	1969	1969	The Arrangement	The Arrangement	Kazan, Elia	Elia Kazan	tt0064041
1746465305	1944	1944	Jamboree	Jamboree	Santley, Joseph	Joseph Santley	tt0036967
1746501949	1915	1915	Gambier's Advocate	Gambier's Advocate	Kirkwood, James	James Kirkwood	tt0005370
1745293243	2006	2006	A Good Year	A Good Year	Scott, Ridley	Ridley Scott	tt0401445
1746503527	1921	1921	Cupid's Brand	Cupid's Brand	Lee, Rowland V.	Rowland V. Lee	tt0012083
1746570169	1934	1934	The Last Round-Up	The Last Round Up	Hathaway, Henry	Henry Hathaway	tt0025370
1746567326	1933	1932	They Just Had to Get Married	They Just Had to Get Married	Ludwig, Edward	Edward Ludwig	tt0024654
1746198441	1968	1968	Villa Rides	Villa Rides	Kulik, Buzz	Buzz Kulik	tt0063775
1746566515	1933	1933	King of the Jungle	King of the Jungle	Humberstone, H. Bruce; Marcin, Ma	H. Bruce Humberstone	tt0024219
1746455074	1998	1998	Psycho	Psycho	Van Sant, Gus	Gus Van Sant	tt0155975
1746584660	1929	1929	New Orleans	New Orleans	Barker, Reginald	Reginald Barker	tt0020215
1746573842	1960	1960	Spring Affair	Spring Affair	Ray, Bernard "B. B."	Bernard B. Ray	tt0234789
1746570411	1933	1933	Golden Harvest	Golden Harvest	Murphy, Ralph	Ralph Murphy	tt0024071
1746422931	1994	1994	Bullets over Broadway	Bullets Over Broadway	Allen, Woody	Woody Allen	tt0109348
1746454037	1943	1943	Ghosts on the Loose	Ghosts on the Loose	Beaudine, William	William Beaudine	tt0035939
1769971187	1970	1970	Mondo Rocco	Mondo Rocco	Rocco, Pat	Pat Rocco	tt0206152
1745340983	2004	2004	Harold & Kumar Go to White Castle	Harold & Kumar Go to White Castle	Leiner, Danny	Danny Leiner	tt0366551
1769970818	1911	1911	The Higher Law	The Higher Law	Nichols, George	George Nichols	tt0410077
1746523380	1916	1916	The Gay Lord Waring	The Gay Lord Waring	Turner, Otis	Otis Turner	tt0006712
1746582907	1936	1936	Here Comes Carter	Here Comes Carter	Clemens, William	William Clemens	tt0025426
1746567005	1952	1952	The Congregation	The Congregation	Beaudine, William; Calihan, William	William Beaudine	tt0433538
1746583529	1930	1930	The Flirting Widow	The Flirting Widow	Seiter, William	William A. Seiter	tt0020890
1746438931	1941	1941	Moon Over Her Shoulder	Moon Over Her Shoulder	Werker, Alfred	Alfred L. Werker	tt0033917
1746524157	1922	1922	The Call of Home	The Call of Home	Gasnier, Louis J.	Louis J. Gasnier	tt0012994
1746437145	1999	1999	Inspector Gadget	Inspector Gadget	Kellogg, David	David Kellogg	tt0141369
1746433706	2000	2000	Turn It Up	Turn It Up	Adetuyi, Robert	Robert Adetuyi	tt0216772
1745340101	2004	2003	The Yes Men	The Yes Men	Smith, Chris; Ollman, Dan ; Price, Sar	Dan Ollman	tt0379593
1746502209	1915	1915	The Little Mademoiselle	The Little Mademoiselle	Eagle, Oscar	Oscar Eagle	tt0005632
1746582401	1937	1937	The Arizona Gunfighter	Arizona Gunfighter	Newfield, Sam	Sam Newfield	tt0028583
1746515260	1918	1918	Breakers Ahead	Breakers Ahead	Brabin, Charles J.	Charles Brabin	tt0008924
1769971122	1969	1969	Monica's Thing	Monica's Thing	Brand, Rex	Rex Brand	tt0794125
1746514826	1916	1916	Three Pals	The Three Pals	Berger, Rea	Rae Berger	tt0159023
1746419910	1989	1989	Wired	Wired	Peerce, Larry	Larry Peerce	tt0098660
1746581547	1930	1930	Three Faces East	Three Faces East	Del Ruth, Roy	Roy Del Ruth	tt0021472
1746464899	1946	1946	The Harvey Girls	The Harvey Girls	Sidney, George	George Sidney	tt0038589
1746574525	1935	1935	Circus Shadows	Circus Shadows	Hutchinson, Charles	Charles Hutchison	tt0026214
1746582078	1910	1910	A Gold Necklace	A Gold Necklace	Powell, Frank	Frank Powell	tt0001235
1746561383	1932	1932	No Man of Her Own	No Man of Her Own	Ruggles, Wesley	Wesley Ruggles	tt0023277
1746524101	1923	1923	Bag and Baggage	Bag and Baggage	Fox, Finis	Finis Fox	tt0013857
1746500084	1924	1924	Janice Meredith	Janice Meredith	Hopper, E. Mason	E. Mason Hopper	tt0015022
1746520793	1926	1926	Perils of the Coast Guard	Perils of the Coast Guard	Apfel, Oscar	Oscar Apfel	tt0017259
1746498308	1918	1918	The Crucible of Life	The Crucible of Life	Lambert, Captain Harry	Harry Lambart	tt0184365
1746455376	1995	1995	Bye Bye Love	Bye Bye Love	Weisman, Sam	Sam Weisman	tt0112606
1746499971	1945	1945	War Comes to America	War Comes to America		Frank Capra	tt0183997
1746453579	1942	1942	Madame Spy	Madame Spy	Neill, Roy William	Roy William Neill	tt0035010
1746500686	1949	1949	The Crime Doctor's Diary	The Crime Doctor's Diary	Friedman, Seymour	Seymour Friedman	tt0041267
1746508246	1949	1949	The Big Cat	The Big Cat	Karlson, Phil	Phil Karlson	tt0041175
1746454059	1943	1943	Henry Aldrich Gets Glamour	Henry Aldrich Gets Glamour	Bennett, Hugh	Hugh Bennett	tt0035983
1746474412	1943	1943	Wings Over the Pacific	Wings Over the Pacific	Rosen, Phil	Phil Rosen	tt0135055
1746577270	1960	1960	The Amazing Transparent Man	The Amazing Transparent Man	Ulmer, Edgar G.	Edgar G. Ulmer	tt0053593
1746583653	1930	1930	Extravagance	Extravagance	Rosen, Phil	Phil Rosen	tt0020866
1746393749	1987	1987	The Bedroom Window	The Bedroom Window	Hanson, Curtis	Curtis Hanson	tt0092627

1769970056	1914	1914	The Stain	The Stain	Powell, Frank	Frank Powell	tt0209347
1746574711	1933	1933	Corruption	Corruption	Roberts, C. Edward	Charles E. Roberts	tt0023909
1746573876	1957	1957	The Story of Mankind	The Story of Mankind	Allen, Irwin	Irwin Allen	tt0051016
1746525105	1920	1920	The Woman and the Puppet	The Woman and the Puppet	Barker, Reginald	Reginald Barker	tt0011873
1746577275	1950	1950	State Penitentiary	State Penitentiary	Landers, Lew	Lew Landers	tt0042999
1746514394	1910	1910	Marriage in Haste	Marriage in Haste			tt1800716
1746393465	1988	1988	Twins	Twins	Reitman, Ivan	Ivan Reitman	tt0096320
1746570815	1933	1933	Melody Cruise	Melody Cruise	Sandrich, Mark	Mark Sandrich	tt0024323
1745342328	2002	2001	The Grey Zone	The Grey Zone	Nelson, Tim Blake	Tim Blake Nelson	tt0252480
1746573918	1960	1960	Bells Are Ringing	Bells Are Ringing	Minnelli, Vincente	Vincente Minnelli	tt0053645
1746571340	1934	1934	I'll Tell the World	I'll Tell the World	Sedgwick, Edward	Edward Sedgwick	tt0025289
1745272504	2001	2001	The Brothers	The Brothers	Hardwick, Gary	Gary Hardwick	tt0250274
1746585335	1929	1929	Scandal	Scandal	Ruggles, Wesley	Wesley Ruggles	tt0020367
1746566075	1956	1955	There's Always Tomorrow	There's Always Tomorrow	Sirk, Douglas	Douglas Sirk	tt0049843
1746525038	1920	1920	Paris Green	Paris Green	Storm, Jerome	Jerome Storm	tt0196827
1746574448	1962	1962	Kid Galahad	Kid Galahad	Karlson, Phil	Phil Karlson	tt0056138
1746421964	1981	1981	Eyewitness	Eyewitness	Yates, Peter	Peter Yates	tt0082353
1746584501	1929	1929	Hard To Get	Hard to Get	Beaudine, William	William Beaudine	tt0019963
1746499915	1949	1949	Rose of the Yukon	Rose of the Yukon	Blair, George	George Blair	tt0174172
1746569672	1957	1957	Gun Duel in Durango	Gun Duel in Durango	Salkow, Sidney	Sidney Salkow	tt0050466
1746523707	1923	1923	The Spoilers	The Spoilers	Hillyer, Lambert	Lambert Hillyer	tt0014503
1746566357	1953	1953	A Slight Case of Larceny	A Slight Case of Larceny	Weis, Don	Don Weis	tt0046329
1746500259	1925	1925	Tricks	Tricks	Mitchell, Bruce	Bruce Mitchell	tt0016453
1746501082	1916	1916	The Moral Fabric	The Moral Fabric	Miller, Charles	Raymond B. West	tt0007091
1746514777	1915	1915	Colorado	Colorado	MacGregor, Norval	Norval MacGregor	tt0005123
1745303061	2007	2007	Great World of Sound	Great World of Sound	Zobel, Craig	Craig Zobel	tt0826547
1746508074	1925	1925	Silent Pal	Silent Pal	McCarty, Henry	Henry McCarty	tt0164169
1746211238	1973	1973	The Female Response	The Female Response	Kincaid, Tim	Tim Kincaid	tt0148130
1746500429	1925	1925	His Supreme Moment	His Supreme Moment	Fitzmaurice, George	George Fitzmaurice	tt0015920
1746500074	1925	1925	The Mansion of Aching Hearts	The Mansion of Aching Hearts	Hogan, James P.	James P. Hogan	tt0016089
1746422917	1993	1993	What's Eating Gilbert Grape	What's Eating Gilbert Grape	Hallström, Lasse	Lasse Hallström	tt0108550
1746454671	1946	1946	My Reputation	My Reputation	Bernhardt, Curtis	Curtis Bernhardt	tt0038765
1746503351	1920	1919	The Spenders	The Spender	Conway, Jack	Charles Swickard	tt0192627
1746582728	1937	1937	She Had to Eat	She Had to Eat	St. Clair, Malcolm	Malcolm St. Clair	tt0029549
1746577404	1951	1951	No Highway in the Sky	No Highway in the Sky	Koster, Henry	Henry Koster	tt0043859
1746570574	1934	1934	The Fighting Trooper	The Fighting Trooper	Taylor, Ray	Ray Taylor	tt0025114
1746465170	1942	1942	Valley of Hunted Men	Valley of Hunted Men	English, John	John English	tt0035500
1746583894	1930	1930	Fast and Loose	Fast and Loose	Newmeyer, Fred	Fred C. Newmeyer	tt0020873
1746436542	1941	1941	The Nurse's Secret	The Nurse's Secret	Smith, Noel M.	Noel M. Smith	tt0033966
1746583253	1928	1928	Forbidden Trails	Forbidden Trails	Horner, Robert J.	Robert J. Horner	tt0325411
1746437399	1943	1943	Gildersleeve on Broadway	Gildersleeve on Broadway	Douglas, Gordon	Gordon Douglas	tt0035940
1746409033	1984	1984	The Cotton Club	The Cotton Club	Coppola, Francis	Francis Ford Coppola	tt0087089
1746581435	1928	1928	The Wedding March	The Wedding March	Stroheim, Erich von	Erich von Stroheim	tt0019558
1746580694	1935	1935	Heir to Trouble	Heir to Trouble	Bennet, Spencer Gordon	Spencer Gordon Bennet	tt0027728
1746530797	1950	1950	Bandit Queen	Bandit Queen	Berke, William	William Berke	tt0042224
1746235345	1971	1971	The Incredible 2-Headed Transplant	The Incredible 2 Headed Transplant	Lanza, Anthony M.	Anthony M. Lanza	tt0067245
1746583326	1929	1929	West of the Rockies	West of the Rockies	Carpenter, H. B.	Horace B. Carpenter	tt0020576
1746559881	1963	1963	Lilies of the Field	Lilies of the Field	Nelson, Ralph	Ralph Nelson	tt0057251
1746581165	1951	1950	Hunt the Man Down	Hunt the Man Down	Archainbaud, George	George Archainbaud	tt0042582
1746581472	1910	1910	How Hubby Got a Raise	How Hubby Got a Raise	Powell, Frank	Frank Powell	tt0362736
1746508879	1926	1926	Tangled Herds	Tangled Herds	Bertram, William	William Bertram	tt0168209
1746512069	1914	1914	The Country Mouse	The Country Mouse	Bosworth, Hobart	Hobart Bosworth	tt0003798
1746565925	1953	1953	Iron Mountain Trail	Iron Mountain Trail	Witney, William	William Witney	tt0045918
1746497631	1919	1919	Sue of the South	Sue of the South	Moore, W. Eugene	Eugene Moore	tt0010743
1746454027	1942	1942	In This Our Life	In This Our Life	Huston, John	John Huston	tt0034890
1746234249	1966	1966	The Talisman	The Talisman	Carr, John	John Carr	tt0061064
1746515023	1916	1916	The Wild Girl of the Sierras	A Wild Girl of the Sierras	Powell, Paul	Paul Powell	tt0007571

1746503253	1920	1920	Honeymoon Ranch	Honeymoon Ranch	Townley, Robert	Robin H. Townley	tt0195796
1746520887	1950	1950	Fast on the Draw	Fast on the Draw	Carr, Thomas	Thomas Carr	tt0042448
1746574514	1934	1934	A Lost Lady	A Lost Lady	Green, Alfred E.	Alfred E. Green	tt0025422
1745293560	2007	2007	P2	P2	Khalfoun, Franck	Franck Khalfoun	tt0804516
1746499946	1923	1923	The Glimpses of the Moon	The Glimpses of the Moon	Dwan, Allan	Allan Dwan	tt0014086
1746513621	1916	1916	The Little Sister of the Poor	The Little Sister of the Poor	Mayo, Melvin	Melvin Mayo	tt0490641
1746508257	1927	1927	The Telephone Girl	The Telephone Girl	Brenon, Herbert	Herbert Brenon	tt0018469
1746574858	1935	1935	The Big Broadcast of 1936	The Big Broadcast of 1936	Taurog, Norman	Norman Taurog	tt0026113
1746210027	1970	1970	The Out-of-Towners	The Out of Towners	Hiller, Arthur	Arthur Hiller	tt0066193
1746393022	1985	1985	That Was Then...This Is Now	That Was Then... This Is Now	Cain, Christopher	Christopher Cain	tt0090151
1746524956	1916	1916	Land O' Lizards	Land o' Lizards	Borzage, Frank	Frank Borzage	tt0006907
1746437852	1941	1941	Saddle Mountain Roundup	Saddle Mountain Roundup	Luby, S. Roy	S. Roy Luby	tt0035280
1746516070	1921	1921	The Sport of the Gods	The Sport of the Gods	Vernot, Henry	Henry J. Vernot	tt0166362
1746424060	1940	1940	Always a Bride	Always a Bride	Smith, Noel M.	Noel M. Smith	tt0032198
1746514357	1927	1927	His First Flame	His First Flame	Edwards, Harry	Harry Edwards	tt0016971
1746466522	1917	1917	The Curse of Eve	The Curse of Eve	Beal, Frank	Frank Beal	tt0014052
1746420790	1982	1982	The Dark Crystal	The Dark Crystal	Henson, Jim; Oz, Frank	Jim Henson	tt0083791
1746514534	1923	1923	Purple Dawn	The Purple Dawn	Seeling, Charles R.	Charles R. Seeling	tt0014383
1746573947	1962	1962	The Nun and the Sergeant	The Nun and the Sergeant	Adreon, Franklin	Franklin Adreon	tt0056296
1746571281	1952	1952	Son of Ali Baba	Son of Ali Baba	Neumann, Kurt	Kurt Neumann	tt0045175
1746585318	1928	1928	Beggars of Life	Beggars of Life	Wellman, William A.	William A. Wellman	tt0018684
1746508393	1924	1924	Worldly Goods	Worldly Goods	Bern, Paul	Paul Bern	tt0015512
1746410010	1987	1987	Million Dollar Mystery	Million Dollar Mystery	Fleischer, Richard	Richard Fleischer	tt0093539
1746499959	1923	1923	The Girl of the Golden West	The Girl of the Golden West	Carewe, Edwin	Edwin Carewe	tt0014085
1746574218	1959	1958	The Naked Maja	The Naked Maja	Koster, Henry	Henry Koster	tt0051891
1746574447	1961	1961	The Roman Spring of Mrs. Stone	The Roman Spring of Mrs. Stone	Quintero, José	José Quintero	tt0055382
1745375174	2008	2007	The Air I Breathe	The Air I Breathe	Lee, Jieho	Jieho Lee	tt0485851
1746438140	1941	1941	Cracked Nuts	Cracked Nuts	Cline, Edward	Edward F. Cline	tt0033497
1746584204	1939	1939	Pride of the Blue Grass	Pride of the Blue Grass	McGann, William	William C. McGann	tt0031823
1746583855	1929	1929	Geraldine	Geraldine	Brown, Melville	Melville W. Brown	tt0019921
1746491724	1948	1948	Docks of New Orleans	Docks of New Orleans	Abrahams, Derwin	Derwin Abrahams	tt0040296
1769971202	1969	1969	Sex Circus	Sex Circus	Goetz, Tommy	Tommy Goetz	tt0257137
1746574250	1962	1961	Bachelor Flat	Bachelor Flat	Tashlin, Frank	Frank Tashlin	tt0054651
1746577880	1938	1938	The Law West of Tombstone	The Law West of Tombstone	Tryon, Glenn	Glenn Tryon	tt0030350
1746508429	1926	1925	The Golden Cocoon	The Golden Cocoon	Webb, Millard	Millard Webb	tt0015868
1746422050	1998	1997	Eden	Men	Goldberg, Howard	Zoe Clarke	tt0119653
1746568330	1934	1934	The Ferocious Pal	Ferocious Pal	Bennett, Spencer Gordon	Spencer Gordon Bennet	tt0167155
1746473735	1942	1942	Pardon My Sarong	Pardon My Sarong	Kenton, Erle C.	Erle C. Kenton	tt0035173
1746474119	1948	1947	The Bishop's Wife	The Bishop's Wife	Koster, Henry	Henry Koster	tt0039190
1746515008	1917	1917	The Girl in the Checkered Coat	The Girl in the Checkered Coat	Grasse, Joseph de	Joseph De Grasse	tt0008009
1746423560	1997	1997	Running Time	Running Time	Becker, Josh	Josh Becker	tt0120042
1746485231	1918	1918	For Freedom	For Freedom	Lloyd, Frank	Frank Lloyd	tt0009083
1746500261	1924	1924	After Dark	After Dark	Chapin, James	James Chapin	tt0014647
1746503372	1921	1921	The Girl From Nowhere	The Girl from Nowhere	Archainbaud, George	George Archainbaud	tt0012217
1745342043	2001	2001	L.I.E.	L.I.E.	Cuesta, Michael	Michael Cuesta	tt0242587
1746502439	1922	1922	The Inner Man	The Inner Man	Smith, Hamilton	Hamilton Smith	tt0124756
1746514333	1910	1910	The Stars and Stripes	The Stars and Stripes	Dawley, J. Searle	J. Searle Dawley	tt0234795
1746578845	1959	1959	Last Train from Gun Hill	Last Train from Gun Hill	Sturges, John	John Sturges	tt0052993
1769974391	1916	1916	His Wife Knew About It	His Wife Knew About It	Drew, Sidney	Sidney Drew	tt0154599
1746503337	1918	1918	Ace High	Ace High	Reynolds, Lynn	Lynn Reynolds	tt0008814
1745340329	2001	2001	Maze	Made	Morrow, Rob	Jon Favreau	tt0227005
1746476199	1946	1946	Beale Street Mama	Beale Street Mama	Williams, Spencer	Spencer Williams	tt0038337
1746496982	1920	1920	Flames of the Flesh	Flames of the Flesh	Le Saint, Edward J.	Edward LeSaint	tt0011190
1746583150	1940	1940	The Bank Dick	The Bank Dick	Cline, Edward	Edward F. Cline	tt0032234
1746580901	1935	1935	Transient Lady	Transient Lady	Buzzell, Edward	Edward Buzzell	tt0027132
1746577337	1959	1959	The Remarkable Mr. Pennypacker	The Remarkable Mr. Pennypacker	Levin, Henry	Henry Levin	tt0053214
1746453555	1943	1943	I Escaped From the Gestapo	I Escaped from the Gestapo	Young, Harold	Harold Young	tt0036026

1746421305	1999	1999	Universal Soldier: The Return	Universal Soldier: The Return	Rodgers, Mic	Mic Rodgers	tt0176269	
1746219556	1977	1977	Airport '77	Airport '77	Jameson, Jerry	Jerry Jameson	tt0075648	
1746438557	1996	1996	The Nutty Professor	The Nutty Professor	Shadyac, Tom	Tom Shadyac	tt0117218	
1746500365	1925	1925	The Man on the Box	The Man on the Box	Reisner, Charles	Charles Reisner	tt0016083	
1746515059	1921	1921	Diane of Star Hollow	Diane of Star Hollow	Sellers, Oliver L.	Oliver L. Sellers	tt0322018	
1746408837	1983	1983	Get Crazy	Get Crazy	Arkush, Allan	Allan Arkush	tt0085551	
1746584769	1929	1929	Smiling Irish Eyes	Smiling Irish Eyes	Seiter, William A.	William A. Seiter	tt0020421	
1746233135	1969	1969	The Big Cube	The Big Cube	Davison, Tito	Tito Davison	tt0064088	
1746437012	1940	1940	Third Finger, Left Hand	Third Finger, Left Hand	Leonard, Robert Z.	Robert Z. Leonard	tt0033153	
1746504779	1917	1916	On Dangerous Ground	On Dangerous Ground	Thornby, Robert	Murdock MacQuarrie	tt0325923	
1746491972	1944	1944	Marine Raiders	Marine Raiders	Schuster, Harold	Harold D. Schuster	tt0037048	
1746585095	1929	1929	The One Man Dog	The One Man Dog	d'Usseau, Leon	Leon D'Usseau	tt0145200	
1746582307	1950	1950	Perfect Strangers	Perfect Strangers	Windust, Bretaigne	Bretaigne Windust	tt0042841	
1746520617	1925	1925	Crack o' Dawn	Crack o' Dawn	Rogell, Albert	Albert S. Rogell	tt0015713	
1746419916	1994	1993	Blink	Blink	Apted, Michael	Michael Apted	tt0109297	
1746492249	1946	1946	Stagecoach to Denver	Stagecoach to Denver	Springsteen, R. G.	R.G. Springsteen	tt0038979	
1746473913	1944	1944	None Shall Escape	None Shall Escape	DeToth, Andre	André De Toth	tt0037136	
1746500354	1925	1925	Don X	Don X	Sheldon, Forrest	Forrest Sheldon	tt0130628	
1746500323	1924	1924	The Last of the Duanes	The Last of the Duanes	Reynolds, Lynn	Lynn Reynolds	tt0015052	
1745373742	2010	2009	Brooklyn's Finest	Brooklyn's Finest	Fuqua, Antoine	Antoine Fuqua	tt1210042	
1746574614	1937	1937	We're on the Jury	We're on the Jury	Holmes, Ben	Ben Holmes	tt0029750	
1746585043	1940	1939	Midnight Shadow	Midnight Shadow	Randol, George	George Randol	tt0031648	
1746584671	1930	1930	Recaptured Love	Recaptured Love	Adolfi, John G.	John G. Adolfi	tt0021289	
1746554845	1957	1957	The Lonely Man	The Lonely Man	Levin, Henry	Henry Levin	tt0050652	
1746565966	1953	1953	Saginaw Trail	Saginaw Trail	Archainbaud, George	George Archainbaud	tt0046266	
1746501752	1915	1915	The Strategist	The Strategist	MacGregor, Norval	Norval MacGregor	tt1276427	
1745358068	2003	2003	Gigli	Gigli	Brest, Martin	Martin Brest	tt0299930	
1745293370	2005	2005	The Comedians of Comedy	The Comedians of Comedy	Blieden, Michael	Michael Blieden	tt0451021	
1746199279	1966	1966	Santa's Christmas Circus	Santa's Christmas Circus	Wiziarde, Frank	Frank Wiziarde	tt0170547	
1746578566	1962	1962	House of Women	House of Women	Doniger, Walter	Walter Doniger	tt0056082	
1746545340	1940	1940	The Secret Seven	The Secret Seven	Moore, James	James Moore	tt0033032	
1746514378	1915	1915	The Man of Shame	The Man of Shame	Myers, Harry C.	Harry Myers	tt0005708	
1746572015	1910	1910	The Courtship of Miles Standish	The Courtship of Miles Standish		Otis Turner	tt0001166	
1746577046	1959	1959	Ride Lonesome	Ride Lonesome	Boetticher, Budd	Budd Boetticher	tt0053220	
1746453498	1941	1941	Adventure in Washington	Adventure in Washington	Green, Alfred E.	Alfred E. Green	tt0033315	
1746577577	1935	1935	Spring Tonic	Spring Tonic	Bruckman, Clyde; Brown, Melville	Clyde Bruckman	tt0027034	
1746570305	1934	1934	King Kelly of the U.S.A.	King Kelly of the U.S.A.	Fields, Leonard	Leonard Fields	tt0025349	
1746499844	1947	1947	Fiesta	Fiesta	Thorpe, Richard	Richard Thorpe	tt0039377	
1746465057	1945	1945	Eve Knew Her Apples	Eve Knew Her Apples	Jason, Will	Will Jason	tt0037685	
1746580897	1939	1939	King of Chinatown	King of Chinatown	Grinde, Nick	Nick Grinde	tt0031534	
**1746467009**	**1917**	**1917**	**Mother Love and the Law**	**Mother Love and the Law**	**Siegmann, George A.**	**George Siegmann**	**tt0008324**	1
1769969816	1911	1911	The Mexican	The Mexican			tt0364514	
1746209104	1965	1965	That Darn Cat	That Darn Cat!	Stevenson, Robert	Robert Stevenson	tt0059793	
1746218120	1967	1966	The Venetian Affair	The Venetian Affair	Thorpe, Jerry	Jerry Thorpe	tt0062432	
1746500607	1925	1925	A Thief in Paradise	A Thief in Paradise	Fitzmaurice, George	George Fitzmaurice	tt0015399	
1746423990	1943	1943	Bar 20	Bar 20	Selander, Lesley	Lesley Selander	tt0035663	
1745373801	2004	2004	Alfie	Alfie	Shyer, Charles	Charles Shyer	tt0375173	
1769976722	1929	1929	The Doll Shop	The Doll Shop	Lee, Sammy	Sammy Lee	tt0370475	
1746500841	1915	1915	The Avalanche	The Avalanche	Davis, Will S.	Will S. Davis	tt0004928	
1746583134	1937	1937	Dead End	Dead End	Wyler, William	William Wyler	tt0028773	
1746580267	1935	1935	Love in Bloom	Love in Bloom	Nugent, Elliott	Elliott Nugent	tt0026656	
1745302251	2006	2006	See No Evil	See No Evil	Dark, Gregory	Gregory Dark	tt0437179	
1746509501	1924	1924	The Torrent	Torment	Younger, A. P.; Doner, William	Maurice Tourneur	tt0015421	
1746491990	1948	1948	Man from Texas	The Man from Texas	Jason, Leigh	Leigh Jason	tt0040565	
1746559991	1963	1963	Wives and Lovers	Wives and Lovers	Rich, John	John Rich	tt0057688	
1746520884	1949	1949	Strange Bargain	Strange Bargain	Price, Will	Will Price	tt0041926	
1746574418	1951	1951	Cattle Drive	Cattle Drive	Neumann, Kurt	Kurt Neumann	tt0043389	

1746569991	1957	1957	The Tin Star	The Tin Star	Mann, Anthony	Anthony Mann	tt0051087	
1746584352	1936	1936	The Devil Is a Sissy	The Devil Is a Sissy	Van Dyke, W. S.; Brown, Rowland	W.S. Van Dyke	tt0027518	
1746585317	1930	1930	Bar L Ranch	Bar L Ranch	Webb, Harry	Harry S. Webb	tt0020665	
1746208372	1968	1968	Therese and Isabelle	Therese and Isabelle	Metzger, Radley H.	Radley Metzger	tt0063683	
1746566323	1957	1957	The Young Stranger	The Young Stranger	Frankenheimer, John	John Frankenheimer	tt0051214	
1746453528	1943	1942	China Girl	China Girl	Hathaway, Henry	Henry Hathaway	tt0034593	
1746219199	1980	1980	Melvin and Howard	Melvin and Howard	Demme, Jonathan	Jonathan Demme	tt0081150	
**1745340029**	**2002**	**2002**	**Two Weeks Notice**	**Two Weeks Notice**	**Lawrence, Marc**	**Marc Lawrence**	**tt0313737**	1
1746504051	1919	1919	The Red Viper	The Red Viper	Tyrol, Jacques	Jacques Tyrol	tt0010617	
1746574472	1952	1952	Red Planet Mars	Red Planet Mars	Horner, Harry	Harry Horner	tt0045073	
1746513831	1916	1916	Madame X	Madame X	Marion, George F.	George F. Marion	tt0007001	
1746424198	1942	1942	Moontide	Moontide	Mayo, Archie; Lang, Fritz	Archie Mayo	tt0035082	
1745341297	2001	2001	Joe Somebody	Joe Somebody	Pasquin, John	John Pasquin	tt0279889	
1746532930	1927	1927	The Shamrock and the Rose	The Shamrock and the Rose	Nelson, Jack	Jack Nelson	tt0018382	
1746565957	1954	1954	Jail Bait	Jail Bait	Wood, Edward D.	Edward D. Wood Jr.	tt0047127	
1746454633	1941	1941	Lydia	Lydia	Duvivier, Julien	Julien Duvivier	tt0033858	
1746583827	1930	1930	The Man Hunter	The Man Hunter	Lederman, Ross	D. Ross Lederman	tt0021113	
1746465261	1942	1942	The Night Before the Divorce	The Night Before the Divorce	Siodmak, Robert	Robert Siodmak	tt0035122	
1746574948	1937	1937	Idol of the Crowds	Idol of the Crowds	Lubin, Arthur	Arthur Lubin	tt0029044	
1746573188	1910	1910	Effecting a Cure	Effecting a Cure	Powell, Frank	Frank Powell	tt0001191	
1746509029	1925	1925	The Girl Who Wouldn't Work	The Girl Who Wouldn't Work	De Sano, Marcel	Marcel De Sano	tt0015860	
1746581383	1951	1951	Bowery Battalion	Bowery Battalion	Beaudine, William	William Beaudine	tt0043356	
1746393426	1987	1987	Hamburger Hill	Hamburger Hill	Irvin, John	John Irvin	tt0093137	
1746503984	1918	1918	The Still Alarm	The Still Alarm	Campbell, Colin	Colin Campbell	tt0009653	
1746584154	1938	1938	The Road to Reno	The Road to Reno	Simon, S. Sylvan	S. Sylvan Simon	tt0030685	
1746584098	1935	1935	The Eagle's Brood	The Eagle's Brood	Bretherton, Howard	Howard Bretherton	tt0026303	
1746453980	1995	1995	Ace Ventura: When Nature Calls	Ace Ventura: When Nature Calls	Oedekerk, Steve	Steve Oedekerk	tt0112281	
1746422913	1999	1999	Hard	Herd	Huckert, John	Mike Mitchell	tt0194006	
1746577141	1950	1950	A Ticket to Tomahawk	A Ticket to Tomahawk	Sale, Richard	Richard Sale	tt0043046	
1746570727	1952	1952	The Sniper	The Sniper	Dmytryk, Edward	Edward Dmytryk	tt0045161	
1746422542	1998	1997	Orgazmo	Orgazmo	Parker, Trey	Trey Parker	tt0124819	
1746503230	1919	1919	Crimson Shoals	Crimson Shoals	Ford, Francis	Francis Ford	tt0190330	
1746554032	1965	1965	Young Dillinger	Young Dillinger	Morse, Terry O.	Terry O. Morse	tt0059931	
1746410877	1987	1987	Three for the Road	Three for the Road	Norton, B. W. L.	Bill Norton	tt0094140	
1746492859	1946	1946	Vacation in Reno	Vacation in Reno	Goodwins, Leslie	Leslie Goodwins	tt0039075	
1746495604	1920	1920	You Never Can Tell	You Never Can Tell	Franklin, Chester M.	Chester M. Franklin	tt0011891	
1746573306	1931	1931	Many a Slip	Many a Sip	Moore, Vin	Mark Sandrich	tt0830923	
1746454915	1999	1998	SLC Punk!	SLC Punk!	Merendino, James	James Merendino	tt0133189	
1746454653	1946	1946	So Goes My Love	So Goes My Love	Ryan, Frank	Frank Ryan	tt0038959	
1746454613	1942	1942	Roxie Hart	Roxie Hart	Wellman, William A.	William A. Wellman	tt0035272	
1746563888	1953	1953	All Ashore	All Ashore	Quine, Richard	Richard Quine	tt0045491	
1746492425	1946	1945	The Red Dragon	The Red Dragon	Rosen, Phil	Phil Rosen	tt0038017	
1745342033	2008	2008	The Spirit	The Spirit	Miller, Frank	Frank Miller	tt0831887	
1746282289	1979	1979	The North Avenue Irregulars	The North Avenue Irregulars	Bilson, Bruce	Bruce Bilson	tt0079639	
1746487169	1918	1918	The Man Who Woke Up	The Man Who Woke Up	McLaughlin, J. W.	James McLaughlin	tt0009340	
1746577310	1963	1963	The Hook	The Hook	Seaton, George	George Seaton	tt0057152	
1746585002	1939	1939	Torchy Blane . . Playing with Dynamite	Torchy Blane.. Playing with Dynamite	Smith, Noel	Noel M. Smith	tt0032044	
1746492485	1945	1945	Strange Confession	Strange Confession	Hoffman, John	John Hoffman	tt0038124	
1746465259	1946	1946	The Big Sleep	The Big Sleep	Hawks, Howard	Howard Hawks	tt0038355	
1746504187	1918	1918	A Successful Adventure	A Successful Adventure	Franklin, Harry L.	Harry L. Franklin	tt0009665	
1746562469	1953	1953	The Man from Cairo	The Man from Cairo	Enright, Ray H.	Ray Enright	tt0045705	
1746585057	1929	1929	The Prince of Hearts	The Prince of Hearts	Wheeler, Cliff	Cliff Wheeler	tt0020290	
1746437140	1996	1996	That Thing You Do	That Thing You Do!	Hanks, Tom	Tom Hanks	tt0117887	
1745372416	2002	2002	Minority Report	Minority Report	Spielberg, Steven	Steven Spielberg	tt0181689	
1746433932	1994	1994	The Scout	The Scout	Ritchie, Michael	Michael Ritchie	tt0111094	
1746500102	1924	1924	The Mirage	The Mirage	Archainbaud, George	George Archainbaud	tt0015142	
1746581911	1910	1910	The Heart of Edna Leslie	The Heart of Edna Leslie		Sidney Olcott	tt0397450	

1746509446	1926	1926	Daniel Boone Thru the Wilderness	Daniel Boone Thru the Wilderness	Mattison, Frank S.; Bradbury, Robert	Robert N. Bradbury	tt0172295
1745359358	2002	2002	Joshua	Joshua	Purdy, Jon	Jon Purdy	tt0271582
1746571458	1931	1931	Rango	Rango		Ernest B. Schoedsack	tt0173105
1746581787	1950	1950	The Sleeping City	The Sleeping City	Sherman, George	George Sherman	tt0042976
1746579059	1960	1960	The Lost World	The Lost World	Allen, Irwin	Irwin Allen	tt0054038
1746574646	1933	1933	Love, Honor and Oh, Baby!	Love, Honor and Oh, Baby!	Buzzell, Eddie	Edward Buzzell	tt0024273
1746504569	1918	1918	The Crime of the Hour	The Crime of the Hour	Ricketts, Thomas R.	Tom Ricketts	tt0184361
1746492122	1947	1947	The Ghost and Mrs. Muir	The Ghost and Mrs. Muir	Mankiewicz, Joseph L.	Joseph L. Mankiewicz	tt0039420
1746500001	1948	1948	Smart Woman	Smart Woman	Blatt, Edward A.	Edward A. Blatt	tt0040804
1746585451	1939	1939	Women in the Wind	Women in the Wind	Farrow, John	John Farrow	tt0032142
1746438972	1943	1943	She's for Me	She's for Me	LeBorg, Reginald	Reginald Le Borg	tt0036347
1746584742	1928	1928	Glorious Betsy	Glorious Betsy	Crosland, Alan	Alan Crosland	tt0018945
1746580456	1936	1936	A Son Comes Home	A Son Comes Home	Dupont, E. A.	Ewald André Dupont	tt0028278
1746571144	1934	1934	Jane Eyre	Jane Eyre	Cabanne, Christy	Christy Cabanne	tt0025323
1746583998	1935	1935	The Judgement Book	The Judgement Book	Hutchinson, Charles	Charles Hutchison	tt0026554
1746269703	1977	1977	Short Eyes	Short Eyes	Young, Robert M.	Robert M. Young	tt0076706
1746566024	1956	1956	A Woman's Devotion	A Woman's Devotion	Henreid, Paul	Paul Henreid	tt0049961
1746577733	1936	1936	Make Way for a Lady	Make Way for a Lady	Burton, David	David Burton	tt0027932
1746580873	1935	1935	Orchids to You	Orchids to You	Seiter, William A.	William A. Seiter	tt0026829
1746409117	1990	1990	Nightbreed	Nightbreed	Barker, Clive	Clive Barker	tt0100260
1746574762	1935	1935	Captain Blood	Captain Blood	Curtiz, Michael	Michael Curtiz	tt0026174
1746433731	1999	1999	Liberty Heights	Liberty Heights	Levinson, Barry	Barry Levinson	tt0165859
1746508089	1925	1925	Double Fisted	Double Fisted	Webb, Harry	Harry S. Webb	tt0298839
1746409522	1983	1983	Cujo	Cujo	Teague, Lewis	Lewis Teague	tt0085382
1746487522	1918	1918	Vengeance	Vengeance	Vale, Travers	Travers Vale	tt0009758
1746514639	1923	1923	Soft Boiled	Soft Boiled	Blystone, J. G.	John G. Blystone	tt0014491
1746504592	1920	1920	Harriet and the Piper	Harriet and the Piper	Bracken, Bertram	Bertram Bracken	tt0011261
1746578571	1960	1960	The Leech Woman	The Leech Woman	Dein, Edward	Edward Dein	tt0054020
1769971960	1969	1969	Roommates Sociable	Roommates Sociable	Glick, Wizard	Wizard Glick	tt3631438
1746562440	1957	1957	Fury at Showdown	Fury at Showdown	Oswald, Gerd	Gerd Oswald	tt0050420
1746235477	1977	1977	Telefon	Telefon	Siegel, Don	Don Siegel	tt0076804
1746509224	1949	1949	Son of a Badman	Son of a Badman	Taylor, Ray	Ray Taylor	tt0041897
1746393352	1983	1983	Breathless	Breathless	McBride, Jim	Jim McBride	tt0085276
1746507614	1923	1923	In Search of a Thrill	In Search of a Thrill	Apfel, Oscar	Oscar Apfel	tt0014148
1746520412	1926	1926	In Borrowed Plumes	In Borrowed Plumes	Halperin, Victor Hugo	Victor Halperin	tt0016994
1746514841	1913	1913	The Rogues of Paris	The Rogues of Paris	Blaché, Alice	Alice Guy	tt0003339
1746454087	1944	1944	My Best Gal	My Best Gal	Mann, Anthony	Anthony Mann	tt0037107
1746501767	1915	1915	The Unknown	The Unknown	Melford, George	George Melford	tt0006201
1746581822	1930	1930	The Sea Wolf	The Sea Wolf	Santell, Alfred	Alfred Santell	tt0021348
1746501447	1910	1910	The Range Riders	The Range Riders	Reynolds, Lynn	Francis Boggs	tt0001375
1746524784	1915	1915	In the Shadow	In the Shadow	Handworth, Harry	Harry Handworth	tt0005543
1746571716	1931	1931	The Phantom	The Phantom	Neitz, Alvin J.	Alan James	tt0022265
1746513028	1916	1916	Spellbound	Spellbound	Harvey, Harry	Harry Harvey	tt0158986
1746500661	1926	1926	Transcontinental Limited	Transcontinental Limited	Ross, Nat	Nat Ross	tt0173352
1746419151	1989	1989	The Big Picture	The Big Picture	Guest, Christopher	Christopher Guest	tt0096926
1746555613	1955	1955	Wichita	Wichita	Tourneur, Jacques	Jacques Tourneur	tt0048806
1746182960	1910	1910	The Bad Man's Christmas Gift	The Bad Man's Christmas Gift		Gilbert M. 'Broncho Billy' Anderson	tt0001124
1746574117	1958	1958	Voice in the Mirror	Voice in the Mirror	Keller, Harry	Harry Keller	tt0052373
1746581237	1961	1961	The Boy Who Caught a Crook	Boy Who Caught a Crook	Cahn, Edward L.	Edward L. Cahn	tt0054695
1746513687	1916	1916	The Rack	Jack	Chautard, Emile	Frank Borzage	tt0484620
1746583130	1934	1934	Chloe Love Is Calling You	Chloe, Love Is Calling You	Neilan, Marshall	Marshall Neilan	tt0026209
1746410855	1987	1987	Radio Days	Radio Days	Allen, Woody	Woody Allen	tt0093818
1746464903	1940	1940	Texas Rangers Ride Again	Texas Rangers Ride Again	Hogan, James	James P. Hogan	tt0033140
1746523079	1910	1910	The Sheriff's Capture	The Sheriff's Capture			tt1822339
1769970680	1968	1967	Whip's Women	Whip's Women	Denby, Jerry	Jerry Denby	tt0260560
1746582666	1935	1935	Romance in Manhattan	Romance in Manhattan	Roberts, Stephen	Stephen Roberts	tt0025731
1746523853	1915	1915	The Commanding Officer	The Commanding Officer	Dwan, Allan	Allan Dwan	tt0005126

1746249766	1980	1980	Fade to Black	Fade to Black	Zimmerman, Vernon	Vernon Zimmerman	tt0080711
1746568150	1933	1933	Hot Pepper	Hot Pepper	Blystone, John	John G. Blystone	tt0024141
1746408700	1989	1989	Best of the Best	Best of the Best	Radler, Bob	Robert Radler	tt0096913
1746515810	1921	1921	Two Weeks With Pay	Two Weeks with Pay	Campbell, Maurice	Maurice Campbell	tt0012786
1746574652	1936	1936	Rogue of the Range	Rogue of the Range	Luby, S. Roy	S. Roy Luby	tt0028199
1746455817	1996	1996	Hype!	Hype!	Pray, Doug	Doug Pray	tt0116589
1746182345	1910	1910	An Arcadian Maid	An Arcadian Maid	Griffith, D. W.	D.W. Griffith	tt0001118
1746584741	1929	1929	The Last Performance	The Last Performance	Fejos, Paul	Pál Fejös	tt0020078
1746573805	1962	1962	It's Only Money	It's Only Money	Tashlin, Frank	Frank Tashlin	tt0056110
1746570105	1934	1934	The Lost Patrol	The Lost Patrol	Ford, John	John Ford	tt0025423
1746436655	1942	1942	Road to Morocco	Road to Morocco	Butler, David	David Butler	tt0035262
1746532854	1916	1916	The Dupe	The Dupe	Reicher, Frank	William Worthington	tt0325292
1746574819	1934	1934	Beyond Bengal	Beyond Bengal	Schenck, Harry	Harry Schenck	tt0126214
1746554901	1954	1954	Dragonfly Squadron	Dragonfly Squadron	Selander, Lesley	Lesley Selander	tt0045704
1745374725	2002	2002	Swimfan	Swimfan	Polson, John	John Polson	tt0283026
1769970739	1912	1912	The Cry of the Children	The Cry of the Children		George Nichols	tt0002122
1746520527	1925	1925	The Wild Girl	The Wild Girl	Bletcher, William	Billy Bletcher	tt0016531
1746512742	1923	1923	Adam's Rib	Adam's Rib	De Mille, Cecil B.	Cecil B. DeMille	tt0013817
1746198032	1969	1969	The Last of the Ski Bums	The Last of the Ski Bums	Barrymore, Dick	Dick Barrymore	tt0064575
1746581188	1951	1951	On Moonlight Bay	On Moonlight Bay	Del Ruth, Roy	Roy Del Ruth	tt0043880
1746580823	1937	1937	Two-Gun Law	Two Gun Law	Barsha, Leon	Leon Barsha	tt0134168
1746582150	1928	1928	Where the West Begins	Where the West Begins	Horner, Robert J.; De La Mothe, Leor	Robert J. Horner	tt0178987
1746256279	1977	1977	Sorcerer	Sorcerer	Friedkin, William	William Friedkin	tt0076740
1746580493	1937	1937	The Great Gambini	The Great Gambini	Vidor, Charles	Charles Vidor	tt0028952
1745357741	2009	2009	Not Easily Broken	Not Easily Broken	Duke, Bill	Bill Duke	tt0795438
1746217992	1967	1967	I, a Man	I, a Man	Warhol, Andy	Paul Morrissey	tt0220569
1746583113	1936	1936	Silks and Saddles	Silks and Saddles	Hill, Bob	Robert F. Hill	tt0029563
1746580583	1938	1938	Little Miss Thoroughbred	Little Miss Thoroughbred	Farrow, John	John Farrow	tt0030374
1746208586	1969	1969	Out of It	Out of It	Williams, Paul	Paul Williams	tt0063401
1746573971	1958	1958	Run Silent Run Deep	Run Silent Run Deep	Wise, Robert	Robert Wise	tt0052151
1746574337	1951	1951	I Want You	I Want You	Robson, Mark	Mark Robson	tt0043664
1746474978	1948	1948	Hills of Home	Hills of Home	Wilcox, Fred M.	Fred M. Wilcox	tt0040438
1745272617	2000	2000	What Women Want	What Women Want	Meyers, Nancy	Nancy Meyers	tt0207201
1746393055	1982	1982	Grease 2	Grease 2	Birch, Patricia	Patricia Birch	tt0084021
1746186319	1966	1966	The Singing Nun	The Singing Nun	Koster, Henry	Henry Koster	tt0060983
1745340012	2001	2000	The Debut	The Debut	Cajayon, Gene	Gene Cajayon	tt0163745
1746578943	1961	1961	Homicidal	Homicidal	Castle, William	William Castle	tt0054988
1746438472	1943	1943	Border Buckaroos	Border Buckaroos	Drake, Oliver	Oliver Drake	tt0035691
1746185842	1969	1969	House of a Thousand Dreams	House of a Thousand Dreams			tt0437235
1746185860	1968	1968	The Filthy Five	The Filthy Five	Milligan, Andy	Andy Milligan	tt0062972
1746585077	1928	1928	Ramona	Ramona	Carewe, Edwin	Edwin Carewe	tt0019305
1746234990	1966	1966	This Property Is Condemned	This Property Is Condemned	Pollack, Sydney	Sydney Pollack	tt0061089
1769970798	1911	1911	Lorna Doone	Lorna Doone		Theodore Marston	tt0410262
1746197865	1967	1967	Eros, o Basileus	Eros, O Basileus	Markopoulos, Gregory J.	Gregory J. Markopoulos	tt0195686
1746485927	1920	1920	The Yellow Typhoon	The Yellow Typhoon	José, Edward	Edward José	tt0011889
1746216744	1968	1968	The Wicked Die Slow	The Wicked Die Slow	Hennigar, William K.	William K. Hennigar	tt0261408
1746501684	1916	1916	The Smugglers	The Smugglers	Olcott, Sidney	Sidney Olcott	tt0007360
1746454597	1999	1998	Free Enterprise	Free Enterprise	Meyer Burnett, Robert	Robert Meyer Burnett	tt0141105
1746437930	1943	1943	Let's Have Fun	Let's Have Fun	Barton, Charles	Charles Barton	tt0183399
1746576608	1932	1932	45 Calibre Echo	45 Calibre Echo	Mitchell, Bruce	Bruce Mitchell	tt0193957
1746501154	1916	1916	The Hero of Submarine D-2	The Hero of Submarine D 2	Scardon, Paul	Paul Scardon	tt0157751
1746502676	1922	1922	Human Hearts	Human Hearts	Baggot, King	King Baggot	tt0013253
1746497683	1917	1917	Snap Judgement	Snap Judgment	Sloman, Edward	Edward Sloman	tt0008595
1746584034	1937	1937	The Toast of New York	The Toast of New York	Lee, Rowland V.; Hall, Alexander	Rowland V. Lee	tt0029675
1746583341	1930	1930	The Florodora Girl	The Florodora Girl	Beaumont, Harry	Harry Beaumont	tt0020891
1746577877	1935	1934	The Rawhide Terror	The Rawhide Terror	Nelson, Jack; Mitchell, Bruce	Bruce Mitchell	tt0173114
1746574249	1960	1960	The Facts of Life	The Facts of Life	Frank, Melvin	Melvin Frank	tt0053810

1746585212	1928	1928	The Chaser	The Chaser	Langdon, Harry	Harry Langdon	tt0018761	
1746574027	1960	1960	Studs Lonigan	Studs Lonigan	Lerner, Irving	Irving Lerner	tt0054348	
1746542699	1925	1925	Red Love	Red Love	Lewis, Edgar	Edgar Lewis	tt0016277	
1746516543	1920	1920	Headin' Home	Headin' Home	Windom, Lawrence	Lawrence C. Windom	tt0011267	
1746578644	1962	1961	Reptilicus	Reptilicus	Pink, Sidney; Bang, Poul	Sidney W. Pink	tt0056405	
1746578439	1963	1963	Nature's Sweethearts	Nature's Sweethearts	Wolk, Larry	Larry Wolk	tt0200894	
1746511823	1923	1923	Dead Game	Dead Game	Sedgwick, Edward	Edward Sedgwick	tt0013979	
1746513821	1915	1915	Right Off the Bat	Right Off the Bat	Reticker, Hugh	Hugh Reticker	tt0005967	
1746234257	1968	1968	Pretty Poison	Pretty Poison	Black, Noel	Noel Black	tt0063456	
1746437332	1940	1940	The Boys from Syracuse	The Boys from Syracuse	Sutherland, A. Edward	A. Edward Sutherland	tt0032276	
1746497895	1921	1921	Bob Hampton of Placer	Bob Hampton of Placer	Neilan, Marshall	Marshall Neilan	tt0011985	
1746570167	1934	1934	Cross Streets	Cross Streets	Strayer, Frank R.	Frank R. Strayer	tt0025016	
1746453757	1943	1943	What's Buzzin' Cousin?	What's Buzzin', Cousin?	Barton, Charles	Charles Barton	tt0036528	
1746454808	1941	1941	Power Dive	Power Dive	Hogan, James	James P. Hogan	tt0034056	
1746570420	1952	1952	The Turning Point	The Turning Point	Dieterle, William	William Dieterle	tt0045267	
1745341787	2010	2009	Don McKay	Don McKay	Goldberger, Jake	Jake Goldberger	tt1281374	
1746211227	1974	1974	The Longest Yard	The Longest Yard	Aldrich, Robert	Robert Aldrich	tt0071771	
1746272118	1967	1967	Bike Boy	Bike Boy	Warhol, Andy	Andy Warhol	tt0061403	
1746508477	1949	1948	The Valiant Hombre	The Valiant Hombre	Fox, Wallace	Wallace Fox	tt0040929	
1746520170	1926	1926	The Canadian	The Canadian	Beaudine, William	William Beaudine	tt0016706	
1746523356	1910	1910	A Ranchman's Wooing	A Ranchman's Wooing		Gilbert M. 'Broncho Billy' Anderson	tt0372485	
1746585481	1940	1940	While Thousands Cheer	While Thousands Cheer	Popkin, Leo C.	Leo C. Popkin	tt0033255	
1746409066	1990	1990	The Bonfire of the Vanities	The Bonfire of the Vanities	De Palma, Brian	Brian De Palma	tt0099165	
1746423529	1940	1940	One Crowded Night	One Crowded Night	Reis, Irving	Irving Reis	tt0032869	
1746495978	1920	1920	Duds	Suds	Mills, Thomas R.	John Francis Dillon	tt0011741	
1746454688	1941	1941	Look Who's Laughing	Look Who's Laughing	Dwan, Allan	Allan Dwan	tt0033850	
1746573743	1961	1961	Mr. Sardonicus	Mr. Sardonicus	Castle, William	William Castle	tt0055200	
1746420865	1998	1998	Apt Pupil	Apt Pupil	Singer, Bryan	Bryan Singer	tt0118636	
1746570127	1933	1933	Horseplay	Horse Play	Sedgwick, Edward	Edward Sedgwick	tt0024140	
1746423314	1990	1990	Disturbed	Disturbed	Winkler, Charles	Charles Winkler	tt0099441	
1746583883	1929	1929	Coquette	Coquette	Taylor, Sam	Sam Taylor	tt0019788	
1746574130	1961	1961	The Sergeant Was a Lady	The Sergeant Was a Lady	Glasser, Bernard	Bernard Glasser	tt0055434	
1746454819	1945	1945	Bandits of the Badland	Bandits of the Badlands	Carr, Thomas	Thomas Carr	tt0037525	
1746530674	1949	1949	I Shot Jesse James	I Shot Jesse James	Fuller, Samuel	Samuel Fuller	tt0041497	
**1746474946**	**1947**	**1947**	**Calendar Girl**	**Calendar Girl**	**Dwan, Allan**	**Allan Dwan**	**tt0039236**	1
1746436931	1995	1995	Jumanji	Jumanji	Johnston, Joe	Joe Johnston	tt0113497	
1746497880	1917	1917	Barnaby Lee	Barnaby Lee	Griffith, Edward H.	Edward H. Griffith	tt0176511	
1746565900	1954	1954	City Story	City Story	Beaudine, William	William Beaudine	tt0180605	
1746474528	1946	1946	Heldorado	Heldorado	Witney, William	William Witney	tt0038593	
1746578850	1959	1959	Holiday for Lovers	Holiday for Lovers	Levin, Henry	Henry Levin	tt0052897	
1746583730	1930	1930	The Devil's Holiday	The Devil's Holiday	Goulding, Edmund	Edmund Goulding	tt0020823	
1746576724	1931	1931	Soul of the Slums	Soul of the Slums	Strayer, Frank	Frank R. Strayer	tt0022416	
1746583448	1929	1929	The Jazz Age	The Jazz Age	Shores, Lynn	Lynn Shores	tt0020040	
1746422214	1989	1989	True Love	True Love	Savoca, Nancy	Nancy Savoca	tt0098528	
1746520611	1949	1949	Bad Men of Tombstone	Bad Men of Tombstone	Neumann, Kurt	Kurt Neumann	tt0041147	
1746569647	1953	1953	Son of the Renegade	Son of the Renegade	Browne, Reg	Reg Browne	tt0046347	
1746475937	1946	1946	The Dark Corner	The Dark Corner	Hathaway, Henry	Henry Hathaway	tt0038453	
1769976727	1929	1929	Music Hath Harms	Music Hath Charms	Graham, Walter	Murray Roth	tt0020191	
1746523369	1915	1915	Motherhood	Motherhood	Carleton, Lloyd B.	Lloyd B. Carleton	tt1261915	
1746584170	1936	1936	Wild Horse Round-Up	Wild Horse Round Up	James, Alan	Alan James	tt0134216	
1746581036	1960	1960	Oklahoma Territory	Oklahoma Territory	Cahn, Edward L.	Edward L. Cahn	tt0054138	
1746579023	1958	1958	Thunder Road	Thunder Road	Ripley, Arthur	Arthur Ripley	tt0052293	
1746495618	1917	1917	Money Madness	Money Madness	McRae, Henry	Henry MacRae	tt0008312	
1746570385	1934	1934	Tarzan and His Mate	Tarzan and His Mate	Gibbons, Cedric	Cedric Gibbons	tt0025862	
**1746524676**	**1917**	**1917**	**American Maid**	**American Maid**	**Capellani, Albert**	**Albert Capellani**	**tt0166970**	1
1746554728	1964	1964	Paris When It Sizzles	Paris When It Sizzles	Quine, Richard	Richard Quine	tt0058453	
1769975268	1916	1916	The Wonderful Wager	The Wonderful Wager	Plaissetty, Rene	René Plaissetty	tt0438644	

1746199537	1967	1967	Run Like a Thief	Run Like a Thief	Glasser, Bernard	Bernard Glasser	tt0062208
1746454630	1946	1946	Home in Oklahoma	Home in Oklahoma	Witney, William	William Witney	tt0038611
1746211215	1974	1974	Big Bad Mama	Big Bad Mama	Carver, Steve	Steve Carver	tt0071216
1746585229	1928	1928	The Cavalier	The Cavalier	Willat, Irvin	Irvin Willat	tt0018752
1746210480	1971	1971	Billy Jack	Billy Jack	Frank, T. C.	Tom Laughlin	tt0066832
1746520623	1926	1926	Baited Trap	The Baited Trap	Paton, Stuart	Stuart Paton	tt0016619
1746500639	1926	1926	Subway Sadie	Subway Sadie	Santell, Alfred	Alfred Santell	tt0017439
1746421284	1994	1994	The Crow	The Crow	Proyas, Alex	Alex Proyas	tt0109506
1745395698	2008	2007	Hollywood Chinese	Hollywood Chinese	Dong, Arthur	Arthur Dong	tt1052005
1746520162	1924	1924	Tongues of Flame	Tongues of Flame	Henabery, Joseph	Joseph Henabery	tt0016437
1746454092	1944	1944	The San Antonio Kid	The San Antonio Kid	Bretherton, Howard	Howard Bretherton	tt0037243
1745302798	2006	2006	Kettle of Fish	Kettle of Fish	Myers, Claudia	Claudia Myers	tt0473500
1746583388	1928	1928	Women Who Dare	Women Who Dare	King, Burton	Burton L. King	tt0019595
1746577436	1961	1961	Misty	Misty	Clark, James B.	James B. Clark	tt0055186
1746410740	1992	1992	The Bodyguard	The Bodyguard	Jackson, Mick	Mick Jackson	tt0103855
1746577316	1951	1951	Rawhide	Rawhide	Hathaway, Henry	Henry Hathaway	tt0043959
1746520819	1925	1925	Durand of the Bad Lands	Durand of the Bad Lands	Reynolds, Lynn	Lynn Reynolds	tt0015770
1746520345	1924	1924	Discontented Husbands	Discontented Husbands	Le Saint, Edward J.	Edward LeSaint	tt0014849
1746410593	1991	1991	Naked Gun 2 1/2: The Smell of Fear	The Naked Gun 2½: The Smell of Fear	Zucker, David	David Zucker	tt0102510
1746584364	1937	1937	Jim Hanvey, Detective	Jim Hanvey, Detective	Rosen, Phil	Phil Rosen	tt0029066
1746434263	1992	1992	Under Siege	Under Siege	Davis, Andrew	Andrew Davis	tt0105690
1746393062	1981	1981	They All Laughed	They All Laughed	Bogdanovich, Peter	Peter Bogdanovich	tt0083189
1746514604	1915	1915	Esmeralda	Esmeralda	Kirkwood, James	James Kirkwood	tt0005249
1746465303	1944	1944	An American Romance	An American Romance	Vidor, King	King Vidor	tt0036596
1745303121	2005	2004	Double Dare	Double Dare	Micheli, Amanda	Amanda Micheli	tt0365135
1746580461	1936	1936	The Drag-Net	The Drag Net	Moore, Vin	Vin Moore	tt0027546
1746583027	1936	1936	San Francisco	San Francisco	Van Dyke, W. S.	W.S. Van Dyke	tt0028216
1746574949	1937	1937	Stolen Holiday	Stolen Holiday	Curtiz, Michael	Michael Curtiz	tt0028310
1746578896	1950	1950	My Friend Irma Goes West	My Friend Irma Goes West	Walker, Hal	Hal Walker	tt0042769
1746235225	1971	1971	Sometimes a Great Notion	Sometimes a Great Notion	Newman, Paul; Colla, Richard A.	Paul Newman	tt0067774
1746508132	1950	1950	The Golden Gloves Story	The Golden Gloves Story	Feist, Felix	Felix E. Feist	tt0042511
1746580561	1938	1938	Hawaii Calls	Hawaii Calls	Cline, Edward F.	Edward F. Cline	tt0030217
1746256661	1979	1979	The Onion Field	The Onion Field	Becker, Harold	Harold Becker	tt0079668
1746218755	1973	1973	Beyond Atlantis	Beyond Atlantis	Romero, Eddie	Eddie Romero	tt0069783
1746582176	1930	1930	The Gorilla	The Gorilla	Foy, Bryan	Bryan Foy	tt0021923
1746474123	1948	1948	Appointment with Murder	Appointment with Murder	Bernhard, Jack	Jack Bernhard	tt0040106
1746566089	1953	1953	Prince of Pirates	Prince of Pirates	Salkow, Sidney	Sidney Salkow	tt0046208
1746211096	1971	1971	The Last Movie	The Last Movie	Hopper, Dennis	Dennis Hopper	tt0067327
1746581446	1910	1910	The Italian Sherlock Holmes	The Italian Sherlock Holmes			tt2288238
1746487024	1918	1918	Love Me	Love Me	Neill, R. William	Roy William Neill	tt0009319
1746568275	1933	1933	The Devil's Brother	The Devil's Brother	Roach, Hal	Hal Roach	tt0023943
1745282609	2007	2007	Vacancy	Vacancy	Antal, Nimród	Nimród Antal	tt0452702
1746574943	1937	1936	Shadow of Chinatown	Shadow of Chinatown	Hill, Robert S.	Robert F. Hill	tt0126659
1746567084	1933	1933	Dance Hall Hostess	Dance Hall Hostess	Eason, Breezy	B. Reeves Eason	tt0023924
1746507800	1926	1926	Without Orders	Without Orders	Maloney, Leo	Leo D. Maloney	tt0017570
1746475292	1948	1948	The Countess of Monte Cristo	The Countess of Monte Cristo	De Cordova, Frederick; Stone, Andre	Frederick De Cordova	tt0040251
1746512617	1928	1928	Jazz Mad	Jazz Mad	Weight, F. Harmon	F. Harmon Weight	tt0019041
1746410404	1983	1983	The Survivors	The Survivors	Ritchie, Michael	Michael Ritchie	tt0086397
1746423269	1998	1998	Woo	Woo	Mayer, Daisy von Scherler	Daisy von Scherler Mayer	tt0120531
1746423295	1994	1994	Corrina, Corrina	Corrina, Corrina	Nelson, Jessie	Jessie Nelson	tt0109484
1746577172	1963	1963	"X"--The Man With the X-Ray Eyes	X: The Man with the X Ray Eyes	Corman, Roger	Roger Corman	tt0057693
1746511751	1928	1928	Marry the Girl	Marry the Girl	Rosen, Philip	Phil Rosen	tt0019148
1746515406	1917	1917	Salt of the Earth	Salt of the Earth	Harrison, Saul	Saul Harrison	tt0127266
1746512423	1923	1923	Alias the Night Wind	Alias the Night Wind	Franz, Joseph	Joseph Franz	tt0013821
1746465230	1946	1946	Sun Valley Cyclone	Sun Valley Cyclone	Springsteen, R. G.	R.G. Springsteen	tt0038995
1746520761	1925	1925	The Unguarded Hour	The Unguarded Hour	Hillyer, Lambert	Lambert Hillyer	tt0016472
1746583322	1951	1951	Pardon My French	Pardon My French	Vorhaus, Bernard	Bernard Vorhaus	tt0043901

1746500417	1925	1925	Dangerous Fists	Dangerous Fists	Webb, Harry S.	Harry S. Webb	tt0246549
1745374117	2003	2003	A Mighty Wind	A Mighty Wind	Guest, Christopher	Christopher Guest	tt0310281
1746578993	1959	1959	The Naked Venus	The Naked Venus	Sehested, Ove H.	Edgar G. Ulmer	tt0144415
1746234266	1967	1967	The Further Perils of Laurel and Hardy	The Further Perils of Laurel and Hardy		Robert Youngson	tt0061692
1746216667	1969	1969	Me, Natalie	Me, Natalie	Coe, Fred	Fred Coe	tt0064651
1746454005	1999	1999	The Story of Us	The Story of Us	Reiner, Rob	Rob Reiner	tt0160916
1746574667	1938	1938	Where the West Begins	Where the West Begins	McGowan, J. P.	J.P. McGowan	tt0030970
1746574705	1933	1933	Reunion in Vienna	Reunion in Vienna	Franklin, Sidney	Sidney Franklin	tt0024496
1746475777	1948	1948	Rusty Leads the Way	Rusty Leads the Way	Jason, Will	Will Jason	tt0040750
1746574866	1936	1936	Colleen	Colleen	Green, Alfred E.	Alfred E. Green	tt0027455
1746566320	1954	1953	Crazylegs	Crazylegs	Lyon, Francis D.	Francis D. Lyon	tt0045656
1745272698	2001	2001	Baby Boy	Baby Boy	Singleton, John	John Singleton	tt0255819
1746423476	1996	1996	American Strays	American Strays	Covert, Michael	Michael Covert	tt0115531
1746453789	1943	1943	Du Barry Was a Lady	Du Barry Was a Lady	Del Ruth, Roy	Roy Del Ruth	tt0035829
1746585003	1939	1939	The Llano Kid	The Llano Kid	Venturini, Edward D.	Edward D. Venturini	tt0031581
1746439479	1943	1943	Pistol Packin' Mama	Pistol Packin' Mama	Woodruff, Frank	Frank Woodruff	tt0036266
1746500508	1926	1926	Sweet Daddies	Sweet Daddies	Santell, Alfred	Alfred Santell	tt0017444
1746503031	1910	1910	The Winter's Tale	The Winter's Tale		Theodore Marston	tt0131137
1746582633	1935	1935	Saddle Aces	Saddle Aces	Fraser, Harry	Harry L. Fraser	tt0028213
1746502421	1915	1915	Fighting Bob	Fighting Bob	Noble, John W.	John W. Noble	tt0128211
1746500330	1950	1950	The Capture	The Capture	Sturges, John	John Sturges	tt0042313
1746570244	1953	1952	The Bad and the Beautiful	The Bad and the Beautiful	Minnelli, Vincente	Vincente Minnelli	tt0044391
1746392883	1981	1981	The Hand	The Hand	Stone, Oliver	Oliver Stone	tt0082497
1746409449	1987	1987	The Running Man	The Running Man	Glaser, Paul Michael	Paul Michael Glaser	tt0093894
1745342793	2003	2003	Tupac: Resurrection	Tupac: Resurrection	Lazin, Lauren	Lauren Lazin	tt0343121
1746584393	1939	1939	The Girl and the Gambler	The Girl and the Gambler	Landers, Lew	Lew Landers	tt0031369
1746496331	1919	1919	Who Will Marry Me?	Who Will Marry Me?	Powell, Paul	Paul Powell	tt0010884
1746436545	1943	1943	Marching On!	Marching On!	Williams, Spencer	Spencer Williams	tt0135539
1746393955	1986	1986	Club Paradise	Club Paradise	Ramis, Harold	Harold Ramis	tt0090856
1746584064	1936	1935	The Human Adventure	The Human Adventure	Breasted, Charles	Charles Breasted	tt0026498
1746581396	1951	1951	Reunion in Reno	Reunion in Reno	Neumann, Kurt	Kurt Neumann	tt0043965
1746492586	1943	1943	Submarine Base	Submarine Base	Kelley, Albert	Albert H. Kelley	tt0036398
1746509463	1925	1925	North Star	North Star	Powell, Paul	Paul Powell	tt0017212
1746512172	1923	1923	The Extra Girl	The Extra Girl	Jones, F. Richard	F. Richard Jones	tt0014029
1746422256	1995	1995	Tom and Huck	Tom and Huck	Hewitt, Peter	Peter Hewitt	tt0112302
1746580808	1935	1935	The Case of the Missing Man	Case of the Missing Man	Lederman, D. Ross	D. Ross Lederman	tt0026186
1746485171	1919	1919	The Hushed Hour	The Hushed Hour	Mortimer, Edmund	Edmund Mortimer	tt0010277
1746574846	1933	1933	College Humor	College Humor	Ruggles, Wesley	Wesley Ruggles	tt0023900
1745282664	2005	2005	Constantine	Constantine	Lawrence, Francis	Francis Lawrence	tt0360486
1746504150	1918	1918	The Love Swindle	The Love Swindle	Dillon, Jack	John Francis Dillon	tt0009320
1745374354	2009	2009	The Answer Man	The Answer Man	Hindman, John	John Hindman	tt1187041
1746200496	1969	1969	The Great Bank Robbery	The Great Bank Robbery	Averback, Hy	Hy Averback	tt0064391
1746524897	1927	1927	East Side, West Side	East Side, West Side	Dwan, Allan	Allan Dwan	tt0017841
1746530678	1949	1949	State Department File 649	State Department: File 649	Stewart, Peter	Sam Newfield	tt0041918
1746569678	1957	1957	20 Million Miles to Earth	20 Million Miles to Earth	Juran, Nathan	Nathan Juran	tt0050084
1746582487	1937	1937	Battle of Greed	Battle of Greed	Higgin, Howard	Howard Higgin	tt0028614
1746560940	1932	1932	Gorilla Ship	Gorilla Ship	Strayer, Frank	Frank R. Strayer	tt0022957
1746578985	1960	1960	Ski Troop Attack	Ski Troop Attack	Corman, Roger	Roger Corman	tt0054315
1746498299	1918	1918	A Perfect 36	A Perfect 36	Giblyn, Charles	Charles Giblyn	tt0009485
1769970711	1915	1915	A Bush Ranger at Bay	A Bushranger at Bay		James W. Horne	tt3140194
1746577615	1937	1937	Land Beyond the Law	Land Beyond the Law	Eason, B. Reeves	B. Reeves Eason	tt0029109
1746583656	1931	1931	Women Love Once	Women Love Once	Goodman, Edward	Edward Goodman	tt0022574
1746492573	1944	1943	Tender Comrade	Tender Comrade	Dmytryk, Edward	Edward Dmytryk	tt0036418
1746574474	1934	1934	A Modern Hero	A Modern Hero	Pabst, G. W.	Georg Wilhelm Pabst	tt0025510
1746235324	1976	1976	Murder by Death	Murder by Death	Moore, Robert	Robert Moore	tt0074937
1746516108	1921	1921	After Your Own Heart	After Your Own Heart	Marshall, George E.	George Marshall	tt0011911
1746524907	1916	1916	According to Law	According to Law	Garrick, Richard	Richard Garrick	tt0006336

1746580841	1934	1934	Charlie Chan's Courage	Charlie Chan's Courage	Hadden, George; Forde, Eugene	Eugene Forde	tt0024969
1746580271	1936	1936	Gambling with Souls	Gambling with Souls	Clifton, Elmer	Elmer Clifton	tt0027654
1746198052	1966	1966	Lord Love a Duck	Lord Love a Duck	Axelrod, George	George Axelrod	tt0060636
1746248821	1976	1976	Dr. Black Mr. Hyde	Dr. Black, Mr. Hyde	Crain, William	William Crain	tt0074430
1746475527	1944	1944	Hi, Beautiful	Hi, Beautiful	Goodwins, Leslie	Leslie Goodwins	tt0036916
1746500969	1916	1916	The Soul of a Child	The Soul of a Child	Gorman, Jack	John Gorman	tt0158979
1746575037	1935	1935	Stranded	Stranded	Borzage, Frank	Frank Borzage	tt0027050
1746513055	1910	1910	Uncle Tom's Cabin	Uncle Tom's Cabin	O'Neil, Barry	Barry O'Neil	tt0348277
1746256266	1976	1976	The Pom Pom Girls	The Pom Pom Girls	Ruben, Joseph	Joseph Ruben	tt0075086
1769971128	1969	1969	Meeting on 69th Street	Meeting on 69th Street	Peacock, Kemper	Kemper Peacock	tt0251246
1746581733	1928	1928	Two Gun Murphy	Two Gun Murphy			tt0174297
1746574934	1935	1935	In Old Kentucky	In Old Kentucky	Marshall, George	George Marshall	tt0026524
1746464781	1942	1942	The Hidden Hand	The Hidden Hand	Stoloff, Ben	Benjamin Stoloff	tt0034850
1746584998	1939	1939	Broadway Serenade	Broadway Serenade	Leonard, Robert Z.	Robert Z. Leonard	tt0031121
1746508473	1925	1925	A Little Girl in a Big City	A Little Girl in a Big City	King, Burton	Burton L. King	tt0016031
1746570968	1934	1934	Tomorrow's Youth	Tomorrow's Youth	Lamont, Charles	Charles Lamont	tt0027121
1746500963	1914	1914	The Last Volunteer	The Last Volunteer	Apfel, Oscar C.	Oscar Apfel	tt0004208
1746465221	1947	1947	Gas House Kids Go West	Gas House Kids Go West	Beaudine, William	William Beaudine	tt0039411
1769970728	1915	1915	The Bride of Guadaloupe	The Bride of Guadaloupe	Edwards, Walter	Walter Edwards	tt0421809
1746185863	1966	1966	Sing a Song, for Heaven's Sake	Sing a Song for Heaven's Sake	Van Court, Ulf	Ulf van Court	tt1708436
1746584197	1935	1935	$10 Raise	$10 Raise	Marshall, George	George Marshall	tt0027083
1745359351	2009	2009	Surrogates	Surrogates	Mostow, Jonathan	Jonathan Mostow	tt0986263
1746584177	1939	1939	It's a Wonderful World	It's a Wonderful World	Van Dyke, W. S.	W.S. Van Dyke	tt0031500
1746219376	1973	1973	Hell Up in Harlem	Hell Up in Harlem	Cohen, Larry	Larry Cohen	tt0070169
1746532846	1915	1915	The Heart of Maryland	The Heart of Maryland	Brenon, Herbert	Herbert Brenon	tt0005449
1746500144	1924	1924	The Terror of Pueblo	The Terror of Pueblo			tt0169301
1746436200	1997	1997	Midnight in the Garden of Good and Evil	Midnight in the Garden of Good and Evil	Eastwood, Clint	Clint Eastwood	tt0119668
1746574097	1959	1959	The Lonely Sex	The Lonely Sex	Hilliard, Richard	Richard Hilliard	tt0132287
1746532711	1913	1913	The Shame of the Empire State	The Shame of the Empire State	Rubinstein, Leon J.	Leon J. Rubinstein	tt0003366
1746584786	1929	1929	The Rainbow Man	The Rainbow Man	Newmeyer, Fred	Fred C. Newmeyer	tt0020308
1746577736	1936	1936	Lady Be Careful	Lady Be Careful	Reed, Theodore	Theodore Reed	tt0027861
1746410166	1981	1981	Going Ape!	Going Ape!	Kronsberg, Jeremy Joe	Jeremy Joe Kronsberg	tt0082457
1746454684	1946	1946	Freddie Steps Out	Freddie Steps Out	Dreifuss, Arthur	Arthur Dreifuss	tt0038535
1746453551	1941	1941	New York Town	New York Town	Vidor, Charles	Charles Vidor	tt0033947
1746520642	1927	1927	Land of the Lawless	Land of the Lawless	Buckingham, Thomas	Tom Buckingham	tt0135518
1746578975	1951	1951	The Guy Who Came Back	The Guy Who Came Back	Newman, Joseph	Joseph M. Newman	tt0043609
1746492237	1944	1944	Girl in the Case	Girl in the Case	Berke, William	William Berke	tt0036867
1746577737	1935	1935	Party Wire	Party Wire	Kenton, Erle C.	Erle C. Kenton	tt0026852
1746583489	1928	1928	The Cheer Leader	The Cheer Leader	Neitz, Alvin J.	Alan James	tt0018762
1746584289	1938	1938	Block-Heads	Block Heads	Blystone, John G.	John G. Blystone	tt0029923
1746420225	1988	1988	Rain Man	Rain Man	Levinson, Barry	Barry Levinson	tt0095953
1745341233	2002	2002	The Adventures of Pluto Nash	The Adventures of Pluto Nash	Underwood, Ron	Ron Underwood	tt0180052
1746532728	1916	1916	The Hidden Law	The Hidden Law			tt0157753
1746569625	1958	1958	Hell Squad	Hell Squad	Topper, Burt	Burt Topper	tt0182218
1746583122	1936	1936	Roaming Lady	Roaming Lady	Rogell, Albert S.	Albert S. Rogell	tt0028193
1746500219	1925	1925	The Unnamed Woman	The Unnamed Woman	Hoyt, Harry O.	Harry O. Hoyt	tt0016476
1746435423	1989	1989	The Toxic Avenger Part III: The Last Temptation c	The Toxic Avenger Part III: The Last Temptati	Herz, Michael; Kaufman, Lloyd	Michael Herz	tt0098502
1746453886	1995	1995	Crimson Tide	Crimson Tide	Scott, Tony	Tony Scott	tt0112740
1746523705	1923	1923	Don Quickshot of the Rio Grande	Don Quickshot of the Rio Grande	Marshall, George E.	George Marshall	tt0013997
1746577903	1936	1936	Mind Your Own Business	Mind Your Own Business	McLeod, Norman Z.	Norman Z. McLeod	tt0029252
1746491788	1947	1947	Fear in the Night	Fear in the Night	Shane, Maxwell	Maxwell Shane	tt0039372
1745359156	2009	2009	Night at the Museum: Battle of the Smithsonian	Night at the Museum: Battle of the Smithsor	Levy, Shawn	Shawn Levy	tt1078912
1746570863	1952	1952	Love Island	Love Island	Pollard, Bud	Bud Pollard	tt0044853
1746553903	1965	1965	Two on a Guillotine	Two on a Guillotine	Conrad, William	William Conrad	tt0059837
1746533021	1916	1916	The Lights of New York	Lights of New York	Brooke, Van Dyke	Van Dyke Brooke	tt0163059
1746435383	1998	1998	Celebrity	Celebrity	Allen, Woody	Woody Allen	tt0120533
1746533185	1923	1923	The Broken Violin	The Broken Violin	Dillon, Jack	John Francis Dillon	tt0013897

1746409489	1982	1982	The Beast Within	The Beast Within	Mora, Philippe	Philippe Mora	tt0083629		
1746235546	1980	1980	Used Cars	Used Cars	Zemeckis, Robert	Robert Zemeckis	tt0081698		
1746565891	1956	1955	One Way Ticket to Hell	One Way Ticket to Hell	Price, B. Lawrence	Bamlet Lawrence Price Jr.	tt0045223		
1746585164	1929	1929	Prisoners	Prisoners	Seiter, William A.	William A. Seiter	tt0020291		
1745357728	2010	2010	The Spy Next Door	The Spy Next Door	Levant, Brian	Brian Levant	tt1273678		
1746501887	1915	1915	Keep Moving	Keep Moving	Myll, Louis	Louis Myll	tt0005585		
1746503494	1921	1920	Number 17	Number 17	Beranger, George A.	George Beranger	tt0011520		
1746464937	1945	1945	A Song to Remember	A Song to Remember	Vidor, Charles	Charles Vidor	tt0038104		
1746507741	1924	1924	Behind the Curtain	Behind the Curtain	Franklin, Chester M.	Chester M. Franklin	tt0014708		
1746199003	1969	1969	The Ice House	The Ice House	McGowan, Stuart E.	Stuart E. McGowan	tt0064466		
1746584045	1934	1934	Wings over Africa	Wings Over Africa			tt0130369		
1746585300	1929	1929	Not Quite Decent	Not Quite Decent	Cummings, Irving	Irving Cummings	tt0020226		
1746566255	1955	1955	You're Never Too Young	You're Never Too Young	Taurog, Norman	Norman Taurog	tt0048822		
1746454693	1942	1942	Dr. Renault's Secret	Dr. Renault's Secret	Lachman, Harry	Harry Lachman	tt0034681		
1746498476	1919	1919	The False Code	The False Code	Warde, Ernest C.	Ernest C. Warde	tt0190391		
1746512755	1915	1915	In the Tennessee Hills	In the Tennessee Hills	Vincent, James	James Vincent	tt0252542		
1746516150	1921	1921	A Cowboy Ace	The Cowboy Ace	Franchon, Leonard	Leonard Franchon	tt0012074		
1746508601	1926	1926	Home Sweet Home	Home Sweet Home	Gorman, John	John Gorman	tt1368094		
1746578957	1958	1957	Wild Is the Wind	Wild Is the Wind	Cukor, George	George Cukor	tt0051193		
1746524113	1923	1923	The Dancer of the Nile	The Dancer of the Nile	Earle, William P. S.	William P.S. Earle	tt0013966		
1746584888	1939	1939	Unborn Souls	Unborn Souls	Frazier, Del	Del Frazier	tt2519396		
1746454673	1942	1942	The Sombrero Kid	The Sombrero Kid	Sherman, George	George Sherman	tt0035355		
1746530695	1948	1948	Dead Man's Gold	Dead Man's Gold	Taylor, Ray	Ray Taylor	tt0040277		
1746574498	1934	1934	Hold That Girl	Hold That Girl	MacFadden, Hamilton	Hamilton MacFadden	tt0025259		
1746504049	1920	1920	Heritage	Heritage	Roubert, William L.	William L. Roubert	tt0195779		
1746435219	1942	1942	Overland Stagecoach	Overland Stagecoach	Newfield, Sam	Sam Newfield	tt0035166		
1746464913	1946	1946	Do You Love Me	Do You Love Me	Ratoff, Gregory	Gregory Ratoff	tt0038484		
1746566080	1957	1957	Tomahawk Trail	Tomahawk Trail	Selander, Lesley	Lesley Selander	tt0165990		
1746235474	1971	1972	Angels' Wild Women	Angels' Wild Women	Adamson, Al	Al Adamson	tt0068211		
1746577525	1933	1933	Lone Cowboy	Lone Cowboy	Sloane, Paul	Paul Sloane	tt0025416		
1746454062	1941	1941	Nine Lives Are Not Enough	Nine Lives Are Not Enough	Sutherland, A. Edward	A. Edward Sutherland	tt0033955		
1746485152	1919	1919	Crooked Straight	Crooked Straight	Storm, Jerome	Jerome Storm	tt0189453		
1746496249	1919	1919	The Forfeit	The Forfeit	Powell, Frank	Frank Powell	tt0010143		
1746235463	1973	1973	Idaho Transfer	Idaho Transfer	Fonda, Peter; Matthiesen, Tom	Peter Fonda	tt0071647		
1746464779	1944	1944	Maisie Goes to Reno	Maisie Goes to Reno	Beaumont, Harry	Harry Beaumont	tt0037040		
1746561160	1932	1932	The Widow in Scarlet	The Widow in Scarlet	Seitz, George B.	George B. Seitz	tt0023698		
1746508501	1924	1924	The Woman on the Jury	The Woman on the Jury	Hoyt, Harry O.	Harry O. Hoyt	tt0015507		
1745358912	2003	2003	Something's Gotta Give	Something's Gotta Give	Meyers, Nancy	Nancy Meyers	tt0337741		
1746465105	1942	1942	The Mystery of Marie Roget	Mystery of Marie Roget	Rosen, Phil	Phil Rosen	tt0035107		
**1746570002**	**1962**	**1962**	**The Three Stooges in Orbit**	**The Three Stooges in Orbit**	**Bernds, Edward**	**Edward Bernds**	**tt0056580**		1
1746473758	1942	1942	The Girl from Alaska	The Girl from Alaska	Grinde, Nick	Nick Grinde	tt0034795		
1746520667	1949	1949	And Baby Makes Three	And Baby Makes Three	Levin, Henry	Henry Levin	tt0041122		
1746571662	1931	1931	Stepping Out	Stepping Out	Riesner, Charles F.	Charles Reisner	tt0022432		
1746583171	1940	1940	Midnight Limited	Midnight Limited	Bretherton, Howard	Howard Bretherton	tt0032790		
1746555170	1956	1956	Giant	Giant	Stevens, George	George Stevens	tt0049261		
1746503341	1922	1922	When the Devil Drives	When the Devil Drives	Scardon, Paul	Paul Scardon	tt0013755		
1746210495	1972	1971	Women in Revolt	Women in Revolt	Morrissey, Paul; Warhol, Andy	Paul Morrissey	tt0129631		
1746508905	1924	1924	Pioneer's Gold	Pioneer's Gold	Dixon, Denver	Victor Adamson	tt0015234		
1746499894	1924	1924	Midnight Secrets	Midnight Secrets	Nelson, Jack	Jack Nelson	tt0015134		
1746513080	1923	1923	Danger	Danger	Elfelt, Clifford S.	Clifford S. Elfelt	tt0013967		
1746516282	1921	1921	Her Mad Bargain	Her Mad Bargain	Carewe, Edwin	Edwin Carewe	tt0012270		
1746584694	1930	1930	Such Men Are Dangerous	Such Men Are Dangerous	Hawks, Kenneth	Kenneth Hawks	tt0021432		
1746501919	1916	1916	The Ransom	The Ransom	Lawrence, Edmund	Edmund Lawrence	tt0158885		
1746577731	1936	1936	F-Man	F Man	Cline, Edward F.	Edward F. Cline	tt0027596		
1746580834	1936	1936	Hideaway Girl	Hideaway Girl	Archainbaud, George	George Archainbaud	tt0027740		
1746562232	1956	1956	Seven Wonders of the World	Seven Wonders of the World	Garnett, Tay; Mantz, Paul; Marton, A	Tay Garnett	tt0049744		
1746434978	1998	1998	Deep Rising	Deep Rising	Sommers, Stephen	Stephen Sommers	tt0118956		

1746583714	1931	1931	Doctors' Wives	Doctors' Wives	Borzage, Frank	Frank Borzage	tt0021804
1746520553	1926	1926	You'd Be Surprised	You'd Be Surprised	Rosson, Arthur	Arthur Rosson	tt0017585
1746585202	1930	1930	The Unholy Three	The Unholy Three	Conway, Jack	Jack Conway	tt0021505
1746554076	1964	1964	Georg	Georg	Kaye, Stanton	Stanton Kaye	tt4883026
1746574828	1936	1936	Hitch Hike to Heaven	Hitch Hike to Heaven	Strayer, Frank R.	Frank R. Strayer	tt0027751
1746502702	1914	1914	Judith of Bethulia	Judith of Bethulia	Griffith, D. W.	D.W. Griffith	tt0004181
1746503733	1921	1921	The Wolverine	The Wolverine	Bertram, William	William Bertram	tt0180313
1746491800	1944	1944	Stagecoach to Monterey	Stagecoach to Monterey	Selander, Lesley	Lesley Selander	tt0037309
1746524207	1916	1916	Powder	Powder	Maude, Arthur	Arthur Maude	tt0007205
1746525123	1919	1919	The Unpardonable Sin	The Unpardonable Sin	Neilan, Marshall	Marshall Neilan	tt0010825
1746560212	1963	1963	The Old Dark House	The Old Dark House	Castle, William	William Castle	tt0057379
1746570228	1933	1933	Silent Men	Silent Men	Lederman, D. Ross	D. Ross Lederman	tt0024558
1746523279	1928	1928	Let 'Er Go Gallegher	Let 'Er Go Gallegher	Clifton, Elmer	Elmer Clifton	tt0019085
1746569794	1958	1958	China Doll	China Doll	Borzage, Frank	Frank Borzage	tt0051476
1745293943	2005	2005	Dreamer: Inspired by a True Story	Dreamer: Inspired by a True Story	Gatins, John	John Gatins	tt0418647
1746573750	1960	1960	The 3 Worlds of Gulliver	The 3 Worlds of Gulliver	Sher, Jack	Jack Sher	tt0053882
1746464766	1946	1946	Susie Steps Out	Susie Steps Out	LeBorg, Reginald	Reginald Le Borg	tt0038999
1746499846	1948	1948	Homecoming	Homecoming	LeRoy, Mervyn	Mervyn LeRoy	tt0040446
1746500495	1925	1925	The Lawful Cheater	The Lawful Cheater	O'Connor, Frank	Frank O'Connor	tt0016011
1746576009	1931	1931	Their Mad Moment	Their Mad Moment	Sprague, Chandler	Hamilton MacFadden	tt0022476
1746394691	1991	1991	Thelma & Louise	Thelma & Louise	Scott, Ridley	Ridley Scott	tt0103074
1746584207	1937	1936	One in a Million	One in a Million	Lanfield, Sidney	Sidney Lanfield	tt0028063
1746234877	1968	1968	Attack on the Iron Coast	Attack on the Iron Coast	Wendkos, Paul	Paul Wendkos	tt0062688
1746578891	1961	1961	Days of Thrills and Laughter	Days of Thrills and Laughter		Robert Youngson	tt0054791
1746437855	1941	1940	Dark Streets of Cairo	Dark Streets of Cairo	Kardos, Leslie	László Kardos	tt0033514
1746582901	1938	1938	The Man from Music Mountain	Man from Music Mountain	Kane, Joe	Joseph Kane	tt0030409
1746586163	1939	1939	Unexpected Father	Unexpected Father	Lamont, Charles	Charles Lamont	tt0032078
1746520354	1950	1950	Love That Brute	Love That Brute	Hall, Alexander	Alexander Hall	tt0042688
1746570977	1933	1933	I Loved You Wednesday	I Loved You Wednesday	King, Henry; Menzies, William Camer	Henry King	tt0024159
1746504043	1919	1919	My Little Sister	My Little Sister	Buel, Kenean	Kenean Buel	tt0010496
1746424007	1942	1942	The Mysterious Rider	The Mysterious Rider	Scott, Sherman	Sam Newfield	tt0035106
1769971949	1969	1969	Monique My Love	Monique, My Love	Woodcock, Peter	Peter Woodcock	tt1043859
1746409685	1987	1987	Superman IV: The Quest for Peace	Superman IV: The Quest for Peace	Furie, Sidney J.	Sidney J. Furie	tt0094074
1746408902	1984	1984	Grandview, U.S.A.	Grandview, U.S.A.	Kleiser, Randal	Randal Kleiser	tt0087359
1746580230	1936	1936	Lady of Secrets	Lady of Secrets	Gering, Marion	Marion Gering	tt0027865
1746436081	1942	1942	Girl Trouble	Girl Trouble	Schuster, Harold	Harold D. Schuster	tt0034794
1746523954	1916	1916	The Sex Lure	The Sex Lure	Abramson, Ivan	Ivan Abramson	tt0158939
1746503797	1918	1918	Up the Road with Sallie	Up the Road with Sallie	Taylor, William D.	William Desmond Taylor	tt0009749
1746436252	1999	1999	The Minus Man	The Minus Man	Fancher, Hampton	Hampton Fancher	tt0151582
1769970721	1912	1912	The Man They Scorned	The Man They Scorned		Reginald Barker	tt0806763
1746508497	1924	1924	The Confidence Man	The Confidence Man	Heerman, Victor	Victor Heerman	tt0014798
1746577924	1937	1937	Girls Can Play	Girls Can Play	Hillyer, Lambert	Lambert Hillyer	tt0028933
1746580289	1939	1939	The Long Shot	Long Shot	Lamont, Charles	Charles Lamont	tt0031590
1769971913	1969	1969	The Divorcee	The Divorcee	Stephen, A. C.	Stephen C. Apostolof	tt0124410
1746438299	1941	1941	Playmates	Playmates	Butler, David	David Butler	tt0034033
1746453538	1943	1943	London Blackout Murders	London Blackout Murders	Sherman, George	George Sherman	tt0034988
1746499930	1925	1925	A Man of Nerve	A Man of Nerve	Chaudet, Louis	Louis Chaudet	tt0016082
1746410210	1984	1984	The Ice Pirates	The Ice Pirates	Raffill, Stewart	Stewart Raffill	tt0087451
1746410218	1989	1989	Next of Kin	Next of Kin	Irvin, John	John Irvin	tt0097967
1746500015	1946	1945	Secrets of a Sorority Girl	Secrets of a Sorority Girl	Landers, Lew; Wisbar, Frank	Frank Wisbar	tt0038918
1746410848	1993	1993	Mad Dog and Glory	Mad Dog and Glory	McNaughton, John	John McNaughton	tt0107473
1746563831	1952	1952	The Kid from Broken Gun	The Kid from Broken Gun	Sears, Fred F.	Fred F. Sears	tt0044795
1746583127	1939	1939	Navy Secrets	Navy Secrets	Bretherton, Howard	Howard Bretherton	tt0031715
1746564450	1952	1952	Carrie	Carrie	Wyler, William	William Wyler	tt0044486
1746583420	1951	1951	Lightning Strikes Twice	Lightning Strikes Twice	Vidor, King	King Vidor	tt0043744
1746424217	1941	1941	Steel Against the Sky	Steel Against the Sky	Sutherland, A. Edward	A. Edward Sutherland	tt0034232
1746520907	1949	1950	Side Street	Side Street	Mann, Anthony	Anthony Mann	tt0042960

1746485213	1917	1917	Forbidden Paths	Forbidden Paths	Thornby, Robert T.	Robert Thornby	tt0007968	
1746454054	1943	1943	The Hard Way	The Hard Way	Sherman, Vincent	Vincent Sherman	tt0034828	
1745341999	2003	2003	The Singing Detective	The Singing Detective	Gordon, Keith	Keith Gordon	tt0314676	
1746574935	1936	1936	The Gorgeous Hussy	The Gorgeous Hussy	Brown, Clarence	Clarence Brown	tt0027690	
1746566230	1955	1955	Rage at Dawn	Rage at Dawn	Whelan, Tim	Tim Whelan	tt0048535	
1746585375	1939	1939	Thou Shalt Not Kill	Thou Shalt Not Kill	Auer, John H.	John H. Auer	tt0033156	
1746555848	1955	1955	Moonfleet	Moonfleet	Lang, Fritz	Fritz Lang	tt0048387	
1746497609	1921	1921	The Queen of Sheba	The Queen of Sheba	Edwards, J. Gordon	J. Gordon Edwards	tt0012600	
1746503770	1918	1918	Little Orphant Annie	Little Orphant Annie	Campbell, Colin	Colin Campbell	tt0009306	
1746455397	1997	1997	City of Industry	City of Industry	Irvin, John	John Irvin	tt0118859	
1746420762	1989	1989	sex, lies, and videotape	Sex, Lies, and Videotape	Soderbergh, Steven	Steven Soderbergh	tt0098724	
1746199540	1967	1967	The Gnome-Mobile	The Gnome Mobile	Stevenson, Robert	Robert Stevenson	tt0061715	
1746582586	1938	1938	The Last Express	The Last Express	Garrett, Otis	Otis Garrett	tt0030345	
1746501123	1915	1915	Sealed Valley	Sealed Valley	McGill, Lawrence B.	Lawrence B. McGill	tt0006015	
1746578842	1960	1960	The Subterraneans	The Subterraneans	MacDougall, Ranald	Ranald MacDougall	tt0054351	
1746574799	1933	1933	Curtain at Eight	Curtain at Eight	Hopper, E. Mason	E. Mason Hopper	tt0025025	
1746500080	1945	1945	Mama Loves Papa	Mama Loves Papa	Strayer, Frank	Frank R. Strayer	tt0037891	
1746410759	1990	1990	Sibling Rivalry	Sibling Rivalry	Reiner, Carl	Carl Reiner	tt0100611	
1746582365	1930	1930	Lucky Larkin	Lucky Larkin	Brown, Harry J.	Harry Joe Brown	tt0021104	
1746250331	1975	1975	Doc Savage...The Man of Bronze	Doc Savage: The Man of Bronze	Anderson, Michael	Michael Anderson	tt0072886	
1746435690	1942	1942	Counter-Espionage	Counter Espionage	Dmytryk, Edward	Edward Dmytryk	tt0034617	
1746574809	1936	1936	Brilliant Marriage	Brilliant Marriage	Rosen, Phil	Phil Rosen	tt0027399	
1746584688	1930	1930	The Last of the Lone Wolf	The Last of the Lone Wolf	Boleslavsky, Richard	Richard Boleslawski	tt0021052	
1746217606	1968	1968	Private Relations	Private Relations	Crane, Larry	Larry Crane	tt0128422	
1745272583	2000	2000	Pollock	Pollock	Harris, Ed	Ed Harris	tt0183659	
1746572568	1932	1932	Cheaters at Play	Cheaters at Play	MacFadden, Hamilton	Hamilton MacFadden	tt0022757	
1746514789	1916	1916	Chickens	Chickens		Bobby Burns	tt0006503	
1746516511	1920	1920	The Testing Block	The Testing Block	Hillyer, Lambert	Lambert Hillyer	tt0011756	
1746577962	1938	1938	Phantom Gold	Phantom Gold	Levering, Joseph	Joseph Levering	tt0030580	
1746576944	1951	1951	The Golden Horde	The Golden Horde	Sherman, George	George Sherman	tt0043593	
1746574053	1963	1963	The Sadist	The Sadist	Landis, James	James Landis	tt0057465	
1746410844	1993	1993	Father Hood	Father Hood	Roodt, Darrell James	Darrell Roodt	tt0106877	
1746562316	1953	1953	East of Sumatra	East of Sumatra	Boetticher, Budd	Budd Boetticher	tt0045716	
1746578895	1959	1959	The Tingler	The Tingler	Castle, William	William Castle	tt0053363	
1746570993	1935	1935	Annie Oakley	Annie Oakley	Stevens, George	George Stevens	tt0026073	
1746584121	1935	1935	When a Man's a Man	When a Man's a Man	Cline, Edward F.	Edward F. Cline	tt0027202	
1746409466	1989	1989	Kickboxer	Kickboxer	DiSalle, Mark; Worth, David	Mark DiSalle	tt0097659	
1746500020	1950	1949	There's a Girl in My Heart	There's a Girl in My Heart	Dreifuss, Arthur	Arthur Dreifuss	tt0041957	
**1746581344**	**1960**	**1960**	**The Hound That Thought He Was a Raccoon**	**The Hound That Thought He Was a Raccoon**	**McGowan, Tom**	**Tom McGowan**	**tt0940822**	1
1746198454	1965	1965	The Bedford Incident	The Bedford Incident	Harris, James B.	James B. Harris	tt0058962	
1746582368	1930	1930	Roaring Ranch	Roaring Ranch	Eason, Reaves	B. Reeves Eason	tt0021306	
1746555686	1956	1956	Carousel	Carousel	King, Henry	Henry King	tt0049055	
1746475714	1948	1948	Deadline	Deadline	Drake, Oliver	Oliver Drake	tt0040278	
1746393142	1981	1980	The Watcher in the Woods	The Watcher in the Woods	Hough, John	John Hough	tt0081738	
1746566123	1958	1958	Flood Tide	Flood Tide	Biberman, Abner	Abner Biberman	tt0051620	
1746570045	1952	1952	The Prisoner of Zenda	The Prisoner of Zenda	Thorpe, Richard	Richard Thorpe	tt0045053	
1746520583	1926	1926	The Son of the Sheik	The Son of the Sheik	Fitzmaurice, George	George Fitzmaurice	tt0017416	
1746577103	1959	1959	Okefenokee	Okefenokee	Haig, Roul	Roul Haig	tt0053135	
1746433957	1995	1995	Casino	Casino	Scorsese, Martin	Martin Scorsese	tt0112641	
1746583740	1929	1929	The Phantom of the North	The Phantom of the North	Webb, Harry	Harry S. Webb	tt0020268	
1746514844	1923	1923	The Acquittal	The Acquittal	Brown, Clarence	Clarence Brown	tt0013815	
1746499892	1948	1948	The Arkansas Swing	The Arkansas Swing	Nazarro, Ray	Ray Nazarro	tt0309299	
1746578677	1960	1960	Let No Man Write My Epitaph	Let No Man Write My Epitaph	Leacock, Philip	Philip Leacock	tt0054021	
1746499860	1945	1945	The Kid Sister	The Kid Sister	Newfield, Sam	Sam Newfield	tt0037847	
1746585080	1929	1929	Grace Johnston and the Indiana Five	Grace Johnston and The Indiana Five		Murray Roth	tt1538485	
1746584792	1930	1930	The Rogue Song	The Rogue Song	Barrymore, Lionel	Lionel Barrymore	tt0021307	
1746514818	1917	1917	The Barker	The Barker	Richmond, J. A.	J.A. Richmond	tt0167024	

1746561678	1965	1965	Ski Party	Ski Party	Rafkin, Alan	Alan Rafkin	tt0059726
1746523423	1916	1916	Life's Blind Alley	Life's Blind Alley		Tom Ricketts	tt0006919
1746566976	1934	1934	Forsaking All Others	Forsaking All Others	Van Dyke, W. S.	W.S. Van Dyke	tt0025132
1746566830	1932	1932	The Scarlet Brand	The Scarlet Brand	McGowan, J. P.	J.P. McGowan	tt0023428
1746533184	1916	1916	The Immortal Flame	The Immortal Flame	Abramson, Ivan	Ivan Abramson	tt0157795
1745309220	2007	2007	Sweeney Todd The Demon Barber of Fleet Street	Sweeney Todd: The Demon Barber of Fleet S	Burton, Tim	Tim Burton	tt0408236
1746210369	1971	1971	Cry Uncle	Cry Uncle	Avildsen, John G.	John G. Avildsen	tt0066960
1746474958	1944	1944	Mademoiselle Fifi	Mademoiselle Fifi	Wise, Robert	Robert Wise	tt0037034
1746524042	1917	1917	Durand of the Bad Lands	Durand of the Bad Lands	Stanton, Richard	Richard Stanton	tt0007876
1746434029	1992	1992	Pet Sematary II	Pet Sematary II	Lambert, Mary	Mary Lambert	tt0105128
1746578604	1959	1959	Escort West	Escort West	Lyon, Francis D.	Francis D. Lyon	tt0051591
1746582708	1938	1938	Cattle Raiders	Cattle Raiders	Nelson, Sam	Sam Nelson	tt0029979
1745342782	2004	2003	Los Angeles Plays Itself	Los Angeles Plays Itself	Andersen, Thom	Thom Andersen	tt0379357
1746424376	1940	1940	Buzzy Rides the Range	Buzzy Rides the Range	Kahn, Richard C.	Richard C. Kahn	tt0041216
1746408513	1982	1982	Swamp Thing	Swamp Thing	Craven, Wes	Wes Craven	tt0084745
1746584909	1939	1939	The Jones Family in Hollywood	The Jones Family in Hollywood	St. Clair, Malcolm	Malcolm St. Clair	tt0031512
1746502915	1916	1916	A Reformation Delayed	A Reformation Delayed	Mayo, Melvin	Edward Sloman	tt0495414
1746218738	1971	1971	Sweet Sweetback's Baadasssss Song	Sweet Sweetback's Baadasssss Song	Van Peebles, Melvin	Melvin Van Peebles	tt0067810
1746582248	1931	1931	Sweepstakes	Sweepstakes	Rogell, Albert	Albert S. Rogell	tt0022456
1746520665	1948	1948	Frontier Revenge	Frontier Revenge	Taylor, Ray	Ray Taylor	tt0041393
1745293830	2005	2005	Happy Endings	Happy Endings	Roos, Don	Don Roos	tt0361693
1746435113	1943	1943	In Old Oklahoma	In Old Oklahoma	Rogell, Albert S.	Albert S. Rogell	tt0036038
1746581447	1928	1928	Satan and the Woman	Satan and the Woman	King, Burton	Burton L. King	tt0019352
1746491700	1944	1944	Henry Aldrich Plays Cupid	Henry Aldrich Plays Cupid	Bennett, Hugh	Hugh Bennett	tt0036907
1769969930	1912	1912	Flying to Fortune	Flying to Fortune	Nichols, George O.	George Nichols	tt0414018
1746514116	1914	1914	Ill Starred Babbie	Ill Starred Babbie	MacDonald, Sherwood	Sherwood MacDonald	tt0004140
1746436561	1943	1943	The Falcon and the Co-eds	The Falcon and the Co eds	Clemens, William	William Clemens	tt0035855
1746561580	1954	1954	Dangerous Mission	Dangerous Mission	King, Louis	Louis King	tt0046891
1745293159	2004	2004	The Grudge	The Grudge	Shimizu, Takashi	Takashi Shimizu	tt0391198
1746582938	1935	1936	Burning Gold	Burning Gold	Newfield, Sam	Sam Newfield	tt0027410
1746580319	1937	1938	Here's Flash Casey	Here's Flash Casey	Shores, Lynn	Lynn Shores	tt0028994
1746524963	1915	1915	The Incorrigible Dukane	The Incorrigible Dukane	Durkin, James	James Durkin	tt0005545
1746436088	1940	1940	Dr. Kildare Goes Home	Dr. Kildare Goes Home	Bucquet, Harold S.	Harold S. Bucquet	tt0032414
1746217663	1966	1966	The Glass Bottom Boat	The Glass Bottom Boat	Tashlin, Frank	Frank Tashlin	tt0060463
1746235934	1975	1975	At Long Last Love	At Long Last Love	Bogdanovich, Peter	Peter Bogdanovich	tt0072665
1746514225	1923	1924	Treasure Canyon	Treasure Canyon		B. Reeves Eason	tt0174278
1746492053	1948	1948	Cowboy Cavalier	Cowboy Cavalier	Abrahams, Derwin M.	Derwin Abrahams	tt0040253
1746423744	1998	1998	Baseketball	BASEketball	Zucker, David	David Zucker	tt0131857
1746520294	1927	1927	A Man's Past	A Man's Past	Melford, George	George Melford	tt0018137
1746524519	1922	1922	The Flirt	The Flirt	Henley, Hobart	Hobart Henley	tt0013138
1745373691	2002	2002	Personal Velocity	Personal Velocity	Miller, Rebecca	Rebecca Miller	tt0295238
1746569513	1954	1954	The Atomic Kid	The Atomic Kid	Martinson, Leslie H.	Leslie H. Martinson	tt0046729
1746575649	1931	1931	The Runaround	The Runaround	Craft, Wm. James	William James Craft	tt0022332
1746566007	1954	1954	Vera Cruz	Vera Cruz	Aldrich, Robert	Robert Aldrich	tt0047647
1746424243	1943	1943	Behind the Rising Sun	Behind the Rising Sun	Dmytryk, Edward	Edward Dmytryk	tt0035669
1746515051	1916	1916	Love's Sacrifice	Love's Sacrifice		William H. Clifford	tt0163075
1746583925	1929	1929	Hardboiled	Hardboiled	Ince, Ralph	Ralph Ince	tt0133841
1746576729	1931	1931	Young Donovan's Kid	Young Donovan's Kid	Niblo, Fred	Fred Niblo	tt0022589
1746562361	1954	1954	Gorilla at Large	Gorilla at Large	Jones, Harmon	Harmon Jones	tt0047041
1746514096	1916	1916	The Apostle of Vengeance	The Apostle of Vengeance	Hart, William S.	William S. Hart	tt0006367
1746439701	1995	1995	The Usual Suspects	The Usual Suspects	Singer, Bryan	Bryan Singer	tt0114814
1746577505	1933	1933	Out All Night	Out All Night	Taylor, Sam	Sam Taylor	tt0024422
1746578748	1951	1951	Prairie Roundup	Prairie Roundup	Sears, Fred F.	Fred F. Sears	tt0043932
1746520249	1926	1926	The White Black Sheep	The White Black Sheep	Olcott, Sidney	Sidney Olcott	tt0017552
1769970619	1968	1968	Four Kinds of Love	Four Kinds of Love	Carse, Shannon	William Rotsler	tt0173862
1746580631	1937	1937	Nobody's Baby	Nobody's Baby	Meins, Gus	Gus Meins	tt0029318
1746491866	1948	1948	Beyond Glory	Beyond Glory	Farrow, John	John Farrow	tt0040158

1746514261	1914	1914	Such a Little Queen	Such a Little Queen	Porter, Edwin S.; Ford, Hugh	Hugh Ford	tt0004654
1746583540	1930	1930	Georgia Rose	Georgia Rose	Gant, Harry A.	Harry A. Gant	tt0020916
1746409773	1990	1990	Predator 2	Predator 2	Hopkins, Stephen	Stephen Hopkins	tt0100403
1746501880	1916	1916	20,000 Leagues Under the Sea	20,000 Leagues Under the Sea	Paton, Stuart	Stuart Paton	tt0006333
1746520824	1925	1925	The Trouble Buster	The Trouble Buster	Maloney, Leo	Leo D. Maloney	tt0016456
1746585073	1929	1929	Bulldog Drummond	Bulldog Drummond	Jones, F. Richard	F. Richard Jones	tt0019735
1746582992	1935	1935	Vagabond Lady	Vagabond Lady	Taylor, Sam	Sam Taylor	tt0025176
1746584298	1940	1940	Music in My Heart	Music in My Heart	Santley, Joseph	Joseph Santley	tt0032823
1746473730	1946	1946	House-Rent Party	House Rent Party	Newfield, Sam	Sam Newfield	tt0135460
1746394214	1991	1991	Ernest Scared Stupid	Ernest Scared Stupid	Cherry, John	John R. Cherry III	tt0101821
1746503831	1920	1920	Life's Twist	Life's Twist	Cabanne, William Christy	Christy Cabanne	tt0011402
1746483839	1917	1917	Outwitted	Outwitted	Baker, George D.	George D. Baker	tt0008400
1746574301	1958	1958	Thundering Jets	Thundering Jets	Dantine, Helmut	Helmut Dantine	tt0052294
1746524896	1916	1916	The Reapers	The Reapers	King, Burton	Burton L. King	tt0007252
1746566824	1952	1952	Red Snow	Red Snow	Petroff, Boris L.; Franklin, Harry S.	Harry S. Franklin	tt0045075
1745359392	2002	2002	The Truth About Charlie	The Truth About Charlie	Demme, Jonathan	Jonathan Demme	tt0270707
1746555659	1955	1955	Spy Chasers	Spy Chasers	Bernds, Edward	Edward Bernds	tt0048653
1746567944	1933	1933	Get That Venus	Get That Venus	Lee, Grover	Arthur Varney	tt0024052
1745302788	2006	2006	Talladega Nights: The Ballad of Ricky Bobby	Talladega Nights: The Ballad of Ricky Bobby	McKay, Adam	Adam McKay	tt0415306
1746219219	1979	1979	Love at First Bite	Love at First Bite	Dragoti, Stan	Stan Dragoti	tt0079489
1746578064	1935	1935	Law Beyond the Range	Law Beyond the Range	Beebe, Ford	Ford Beebe	tt0026611
1746577881	1935	1935	Hooray for Love	Hooray for Love	Lang, Walter	Walter Lang	tt0026491
1746473943	1946	1946	My Darling Clementine	My Darling Clementine	Ford, John	John Ford	tt0038762
1746410535	1987	1987	Like Father Like Son	Like Father Like Son	Daniel, Rod	Rod Daniel	tt0093418
1746569749	1954	1954	Brigadoon	Brigadoon	Minnelli, Vincente	Vincente Minnelli	tt0046807
1746582812	1936	1936	The Leavenworth Case	The Leavenworth Case	Collins, Lewis D.	Lewis D. Collins	tt0027878
1746209097	1966	1966	Follow Me, Boys!	Follow Me, Boys!	Tokar, Norman	Norman Tokar	tt0060420
1746435545	1943	1943	How's About It?	How's About It	Kenton, Erle C.	Erle C. Kenton	tt0036021
1769970624	1968	1968	The Muthers	The Muthers		Donald A. Davis	tt0144371
1746272146	1966	1966	The Fat Spy	The Fat Spy	Cates, Joseph	Joseph Cates	tt0059171
1746566227	1958	1958	The Big Beat	The Big Beat	Cowan, Will	Will Cowan	tt0051410
1746554761	1964	1963	Lust for Ecstasy	Lust for Ecstasy	Kuchar, George; Kuchar, Mike	George Kuchar	tt0252659
1746420870	1996	1996	Curdled	Curdled	Braddock, Reb	Reb Braddock	tt0115994
1769971172	1969	1969	She Came on the Bus	She Came on the Bus	Ledger, Curt	Curt Ledger	tt0064972
1746580504	1935	1935	Hold 'Em Yale	Hold 'Em Yale	Lanfield, Sidney	Sidney Lanfield	tt0026480
1746454267	1941	1941	Here Comes Mr. Jordan	Here Comes Mr. Jordan	Hall, Alexander	Alexander Hall	tt0033712
1746570822	1934	1934	Glamour	Glamour	Wyler, William	William Wyler	tt0025181
1746580415	1935	1935	Reckless	Reckless	Fleming, Victor	Victor Fleming	tt0026914
1746577856	1935	1935	The Hawk	The Hawk	Dmytryk, Edward	Edward Dmytryk	tt0026456
1746582905	1938	1938	Sing You Sinners	Sing, You Sinners	Ruggles, Wesley	Wesley Ruggles	tt0030752
1746553239	1963	1963	Diary of a Madman	Diary of a Madman	Le Borg, Reginald	Reginald Le Borg	tt0056993
1746501373	1916	1916	The Chain Invisible	The Chain Invisible	Powell, Frank E.	Frank Powell	tt0160125
1746574124	1957	1957	Battle Hymn	Battle Hymn	Sirk, Douglas	Douglas Sirk	tt0050171
1746418690	1940	1940	Street of Memories	Street of Memories	Traube, Shepard	Shepard Traube	tt0033109
1746504055	1918	1918	The Cast-Off	The Cast Off	West, Raymond B.	Raymond B. West	tt0184331
1746584360	1937	1937	Big Town Girl	Big Town Girl	Werker, Alfred	Alfred L. Werker	tt0028631
1746578004	1936	1936	Men of the Plains	Men of the Plains	Hill, Robert	Robert F. Hill	tt0027956
1745359352	2003	2003	A Guy Thing	A Guy Thing	Koch, Chris	Chris Koch	tt0295289
1746566110	1955	1954	The Bridges at Toko-Ri	The Bridges at Toko Ri	Robson, Mark	Mark Robson	tt0046806
1746582321	1929	1929	Flight	Flight	Capra, Frank R.	Frank Capra	tt0019884
1746555967	1956	1956	Indestructible Man	Indestructible Man	Pollexfen, Jack	Jack Pollexfen	tt0049363
1746525100	1921	1921	Life's Greatest Question	Life's Greatest Question	Revier, Harry	Harry Revier	tt0012388
1769970479	1968	1968	Someone	Someone	Rocco, Pat	Pat Rocco	tt0258189
1746420959	1997	1997	Fire Down Below	Fire Down Below	Alcala, Felix Enrique	Félix Enríquez Alcalá	tt0119123
1746524941	1923	1923	The Westbound Limited	The West~Bound Limited	Johnson, Emory	Emory Johnson	tt0348369
1746524000	1913	1913	An Hour Before Dawn	An Hour Before Dawn	Dawley, J. Searle	J. Searle Dawley	tt0002981
1746583559	1930	1930	Outside the Law	Outside the Law	Browning, Tod	Tod Browning	tt0021224

1746580408	1937	1937	Thoroughbreds Don't Cry	Thoroughbreds Don't Cry	Green, Alfred E.	Alfred E. Green	tt0029664
1746492582	1945	1946	The Great Morgan	The Great Morgan	Perrin, Nat	Nat Perrin	tt0038575
1769969412	1964	1964	The Block	The Block	Orlando, Tony	Tony Orlando	tt0211272
1746184384	1965	1965	Return From the Ashes	Return from the Ashes	Thompson, J. Lee	J. Lee Thompson	tt0059649
1746569624	1954	1954	Executive Suite	Executive Suite	Wise, Robert	Robert Wise	tt0046963
1746465358	1946	1946	My Dog Shep	My Dog Shep	Beebe, Ford	Ford Beebe	tt0038763
1746582301	1931	1931	Safe in Hell	Safe in Hell	Wellman, William A.	William A. Wellman	tt0022335
1746584043	1938	1938	Sally, Irene and Mary	Sally, Irene and Mary	Seiter, William A.	William A. Seiter	tt0030712
1746532818	1916	1916	The Cossack Whip	The Cossack Whip	Collins, John H.	John H. Collins	tt0006547
1746503805	1921	1921	The Ranger and the Law	The Ranger and the Law	Kelly, Robert	Robert Kelly	tt0012609
1746581244	1951	1951	Ridin' the Outlaw Trail	Ridin' the Outlaw Trail	Sears, Fred F.	Fred F. Sears	tt0043970
1745357596	2009	2009	My Life in Ruins	My Life in Ruins	Petrie, Donald	Donald Petrie	tt0865559
1746513794	1915	1915	Trilby	Trilby	Tourneur, Maurice	Maurice Tourneur	tt0006182
1746583359	1928	1928	Bare Knees	Bare Knees	Kenton, Erle C.	Erle C. Kenton	tt0018673
1746483471	1917	1917	Red, White and Blue Blood	Red, White and Blue Blood	Brabin, Charles J.	Charles Brabin	tt0008504
1746508524	1924	1924	Fast and Fearless	Fast and Fearless	Thorpe, Richard	Richard Thorpe	tt0014878
1746578967	1951	1951	The Great Missouri Raid	The Great Missouri Raid	Douglas, Gordon	Gordon Douglas	tt0043600
1746501784	1913	1913	From Dusk to Dawn	From Dusk to Dawn	Wolfe, Frank E.	Frank E. Wolfe	tt0002885
1745309714	2006	2006	Nightmare Man	Nightmare Man	Kanefsky, Rolfe	Rolfe Kanefsky	tt0478216
1746578504	1963	1963	Sin You Sinners	Sin You Sinners	Farrar, Anthony	Anthony Farrar	tt0055450
1745340982	2001	2001	From Hell	From Hell	Hughes, Allen ; Hughes, Albert	Albert Hughes	tt0120681
1746235023	1972	1972	1776	1776	Hunt, Peter H.	Peter H. Hunt	tt0068156
1746533016	1927	1927	The Cat and the Canary	Fat and the Canary	Leni, Paul		tt1027041
1746512190	1927	1927	Closed Gates	Closed Gates	Rosen, Phil	Phil Rosen	tt0017760
1746576619	1932	1932	The Big Broadcast	The Big Broadcast	Tuttle, Frank	Frank Tuttle	tt0022675
1746515380	1918	1918	The Heart of Rachael	The Heart of Rachael	Hickman, Howard	Howard C. Hickman	tt0184555
1746233023	1969	1969	A Boy ... A Girl	A Boy... a Girl	Derek, John	John Derek	tt0173689
1746577692	1933	1933	Cocktail Hour	Cocktail Hour	Schertzinger, Victor	Victor Schertzinger	tt0023897
1746437968	1943	1943	Never a Dull Moment	Never a Dull Moment	Lilley, Edward	Edward C. Lilley	tt0036200
1746567986	1952	1952	Steel Town	Steel Town	Sherman, George	George Sherman	tt0045189
1746566358	1953	1953	Savage Mutiny	Savage Mutiny	Bennet, Spencer G.	Spencer Gordon Bennet	tt0046276
1746504209	1920	1920	The Round-Up	The Round Up	Melford, George H.	George Melford	tt0011647
1746282287	1980	1980	Oh Heavenly Dog	Oh Heavenly Dog	Camp, Joe	Joe Camp	tt0081269
1745373429	2002	2002	Gangs of New York	Gangs of New York	Scorsese, Martin	Martin Scorsese	tt0217505
1746503704	1922	1922	Watch Him Step	Watch Him Step	Nelson, Jack	Jack Nelson	tt0177357
1745374747	2002	2002	Jackass the Movie	Jackass: The Movie	Tremaine, Jeff	Jeff Tremaine	tt0322802
1769970074	1912	1912	The Heart of a Tramp	The Heart of a Tramp			tt0405008
1746569615	1956	1956	It Conquered the World	It Conquered the World	Corman, Roger	Roger Corman	tt0049370
1746554658	1964	1964	Tarzan and Jane Regained Sort Of	Tarzan and Jane Regained... Sort of	Warhol, Andy	Andy Warhol	tt0244833
1746495837	1917	1917	Like Wildfire	Like Wildfire	Paton, Stuart	Stuart Paton	tt0008187
1746185907	1967	1967	White Lightnin' Road	White Lightnin' Road	Ormond, Ron	Ron Ormond	tt0059907
1746424203	1942	1942	Down Texas Way	Down Texas Way	Bretherton, Howard P.	Howard Bretherton	tt0034677
1746524677	1916	1916	The Foundling	The Foundling	O'Brien, John D.	John B. O'Brien	tt0006699
1746584563	1929	1929	High Voltage	High Voltage	Higgin, Howard	Howard Higgin	tt0019983
1746235416	1977	1977	The Deep	The Deep	Yates, Peter	Peter Yates	tt0075925
1746475653	1946	1946	Till the End of Time	Till the End of Time	Dmytryk, Edward	Edward Dmytryk	tt0039036
1746453512	1941	1941	The Lone Rider Crosses the Rio	The Lone Rider Crosses the Rio	Newfield, Sam	Sam Newfield	tt0033842
1746524517	1923	1923	Breaking Into Society	Breaking Into Society	Stromberg, Hunt	Hunt Stromberg	tt0013889
1746570051	1934	1934	Dancing Man	Dancing Man	Ray, Albert	Albert Ray	tt0025030
1746503832	1922	1922	Oliver Twist	Oliver Twist	Lloyd, Frank	Frank Lloyd	tt0013450
1746500434	1926	1926	Her Own Story	Her Own Story	Ford, Francis	Francis Ford	tt0327858
1746500076	1924	1924	The Bandolero	The Bandolero	Terriss, Tom	Tom Terriss	tt0014695
1746420253	1992	1992	Laws of Gravity	Laws of Gravity	Gomez, Nick	Nick Gomez	tt0104693
1746218920	1972	1972	The Getaway	The Getaway	Peckinpah, Sam	Sam Peckinpah	tt0068638
1746520841	1949	1949	I Cheated the Law!	I Cheated the Law	Cahn, Edward L.	Edward L. Cahn	tt0041494
1746566337	1958	1958	Island Women	Island Women	Berke, William	William Berke	tt0122119
1746575004	1933	1933	Headline Shooter	Headline Shooter	Brower, Otto	Otto Brower	tt0024097

1745373410	2008	2008	The Wackness	The Wackness	Levine, Jonathan	Jonathan Levine	tt1082886
1746500142	1949	1949	Master Minds	Master Minds	Yarbrough, Jean	Jean Yarbrough	tt0041644
1746497258	1919	1919	Human Desire	Human Desire	North, Wilfrid	Wilfrid North	tt0010276
1746566737	1932	1932	Virgins of Bali	Virgins of Bali		Deane H. Dickason	tt1268922
1746438152	1942	1942	Baby Face Morgan	Baby Face Morgan	Dreifuss, Arthur	Arthur Dreifuss	tt0034486
1746503459	1921	1921	Ashamed of Parents	Ashamed of Parents	Plympton, Horace G.	Horace G. Plympton	tt0011933
1746577277	1962	1962	13 West Street	13 West Street	Leacock, Philip	Philip Leacock	tt0055718
1746584256	1935	1935	Million Dollar Haul	Million Dollar Haul	Herman, Albert	Albert Herman	tt0312963
1746515000	1923	1923	Canyon of the Fools	Canyon of the Fools	Paul, Val	Val Paul	tt0013911
1746584159	1940	1940	My Little Chickadee	My Little Chickadee	Cline, Edward F.	Edward F. Cline	tt0032828
1746497483	1920	1920	Unseen Forces	Unseen Forces	Franklin, Sidney A.	Sidney Franklin	tt0157139
1746492210	1946	1946	The Dark Horse	The Dark Horse	Jason, Will	Will Jason	tt0038454
1746454051	1942	1942	Henry and Dizzy	Henry and Dizzy	Bennett, Hugh	Hugh Bennett	tt0034844
1746573923	1957	1957	The Strange One	The Strange One	Garfein, Jack	Jack Garfein	tt0051019
1746583079	1937	1937	Sea Devils	Sea Devils	Stoloff, Ben	Benjamin Stoloff	tt0029527
1746566214	1955	1955	The Last Command	The Last Command	Lloyd, Frank	Frank Lloyd	tt0048287
1746436649	1941	1941	Hello Sucker	Hello, Sucker	Cline, Edward	Edward F. Cline	tt0033703
1745373919	2002	2002	Family Fundamentals	Family Fundamentals	Dong, Arthur	Arthur Dong	tt0303334
1746454022	1945	1944	The Fighting Lady	The Fighting Lady		Edward Steichen	tt0036823
1746580568	1938	1938	Spirit of Youth	Spirit of Youth	Fraser, Harry	Harry L. Fraser	tt0030781
1746584486	1930	1930	Born Reckless	Born Reckless	Ford, John	Andrew Bennison	tt0020702
1746465370	1945	1945	The Enchanted Cottage	The Enchanted Cottage	Cromwell, John	John Cromwell	tt0037671
1746581739	1951	1951	Rhythm Inn	Rhythm Inn	Landres, Paul	Paul Landres	tt0224010
1746583910	1930	1930	Under Montana Skies	Under Montana Skies	Thorpe, Richard	Richard Thorpe	tt0021500
1746512629	1915	1915	Gladiola	Gladiola	Collins, John H.	John H. Collins	tt0005396
1746514657	1915	1915	Snobs	Snobs	Apfel, Oscar	Oscar Apfel	tt0006060
1746523284	1916	1916	Tapped Wires	Tapped Wires	Davis, James	J. Gunnis Davis	tt1534512
1746420145	1986	1986	F/X	F/X	Mandel, Robert	Robert Mandel	tt0089118
1746582133	1928	1928	Phantom of the Range	Phantom of the Range	Dugan, James	James Dugan	tt0019270
1746438623	1943	1943	Edge of Darkness	Edge of Darkness	Milestone, Lewis	Lewis Milestone	tt0034694
1746567801	1933	1933	The Song of Songs	The Song of Songs	Mamoulian, Rouben	Rouben Mamoulian	tt0024598
1746582867	1937	1937	Big City	Big City	Borzage, Frank	Frank Borzage	tt0028628
1746577023	1960	1960	The Girl in Lovers Lane	The Girl in Lovers Lane	Rondeau, Charles R.	Charles R. Rondeau	tt0052849
1746454072	1941	1941	Western Union	Western Union	Lang, Fritz	Fritz Lang	tt0034384
1746501616	1915	1915	The Secret Sin	The Secret Sin	Reicher, Frank	Frank Reicher	tt0006020
1746532739	1914	1914	The Better Man	The Better Man		William Powers	tt0003677
1746581770	1928	1928	Trial Marriage	Trial Marriage	Curran, William	William Hughes Curran	tt0174280
1746491843	1948	1947	Secret Beyond the Door	Secret Beyond the Door...	Lang, Fritz	Fritz Lang	tt0040766
1746504011	1920	1920	The Luck of Geraldine Laird	The Luck of Geraldine Laird	Sloman, Edward	Edward Sloman	tt0011420
1746580365	1937	1937	Meet the Boyfriend	Meet the Boy Friend	Staub, Ralph	Ralph Staub	tt0029223
1769969463	1911	1911	The Lonely Range	The Lonely Range		Allan Dwan	tt0492158
1746585251	1930	1930	The Oklahoma Sheriff	The Oklahoma Sheriff	McGowan, J. P.	J.P. McGowan	tt0145178
1746583809	1929	1929	The Racketeer	The Racketeer	Higgin, Howard	Howard Higgin	tt0020305
1746582014	1929	1929	The Sacred Flame	The Sacred Flame	Mayo, Archie L.	Archie Mayo	tt0020355
1746512081	1927	1927	Hidden Aces	Hidden Aces	Mitchell, Howard	Howard M. Mitchell	tt0017990
1746465155	1946	1946	Crime of the Century	Crime of the Century	Ford, Philip	Philip Ford	tt0038431
1746424235	1941	1942	Small Town Deb	Small Town Deb	Schuster, Harold	Harold D. Schuster	tt0034202
1746508584	1926	1926	Western Pluck	Western Pluck	Vale, Travers	Travers Vale	tt0017536
1746433735	1997	1997	Inventing the Abbotts	Inventing the Abbotts	O'Connor, Pat	Pat O'Connor	tt0119381
1746583185	1929	1929	True Heaven	True Heaven	Tinling, James	James Tinling	tt0020520
1746567957	1952	1952	The Greatest Show on Earth	The Greatest Show on Earth	DeMille, Cecil B.	Cecil B. DeMille	tt0044672
1746508469	1923	1923	Scaramouche	Scaramouche	Ingram, Rex	Rex Ingram	tt0014440
1746235227	1976	1976	Rocky	Rocky	Avildsen, John G.	John G. Avildsen	tt0075148
1746218751	1973	1973	Sssssss	Sssssss	Kowalski, Bernard L.	Bernard L. Kowalski	tt0070622
1746545311	1940	1940	The Grapes of Wrath	The Grapes of Wrath	Ford, John	John Ford	tt0032551
1746561342	1954	1954	Johnny Dark	Johnny Dark	Sherman, George	George Sherman	tt0047135
1746523545	1915	1915	Out of the Darkness	Out of the Darkness	Melford, George	George Melford	tt0005847

1746454095	1943	1943	Cowboy in Manhattan	Cowboy in Manhattan	Woodruff, Frank	Frank Woodruff	tt0035761
1746454026	1944	1943	Yellow Canary	Yellow Canary	Wilcox, Herbert	Herbert Wilcox	tt0036549
1746585123	1929	1929	Synthetic Sin	Synthetic Sin	Seiter, William A.	William A. Seiter	tt0020471
1746583627	1929	1929	Wall Street	Wall Street	Neill, R. William	Roy William Neill	tt0020564
1746514771	1914	1914	La Belle Russe	La Belle Russe	Hanley, William J.	William J. Hanley	tt0003672
1746465246	1946	1946	Smooth as Silk	Smooth as Silk	Barton, Charles	Charles Barton	tt0038956
1746574184	1959	1959	The Young Captives	The Young Captives	Kershner, Irvin	Irvin Kershner	tt0053460
1746503447	1922	1922	Turn to the Right	Turn to the Right	Ingram, Rex	Rex Ingram	tt0013716
1746582735	1938	1938	Strange Faces	Strange Faces	Taggart, Errol	Errol Taggart	tt0030804
1746504073	1919	1919	Help! Help! Police!	Help! Help! Police!	Dillon, Edward	Edward Dillon	tt0010236
1746424053	1941	1941	Man Hunt	Man Hunt	Lang, Fritz	Fritz Lang	tt0033873
1746453542	1942	1942	The Fleet's In	The Fleet's In	Schertzinger, Victor	Victor Schertzinger	tt0034736
1745293664	2007	2007	Look	Loop	Rifkin, Adam	Pericles Lewnes	tt1155062
1746503678	1922	1922	A Tailor Made Man	A Tailor Made Man	De Grasse, Joseph	Joseph De Grasse	tt0013654
1746233693	1967	1967	The Happening	The Happening	Silverstein, Elliot	Elliot Silverstein	tt0061748
1746503321	1922	1922	Riders of the Law	Riders of the Law	Bradbury, Robert North	Robert N. Bradbury	tt0265646
1746503851	1920	1920	White Youth	White Youth	Dawn, Norman	Norman Dawn	tt0011863
1746572195	1932	1932	Shanghai Express	Shanghai Express	Sternberg, Josef von	Josef von Sternberg	tt0023458
1746584507	1930	1930	Kismet	Kismet	Dillon, John Francis	John Francis Dillon	tt0021029
1745372812	2010	2010	Scott Pilgrim vs. The World	Scott Pilgrim vs. the World	Wright, Edgar	Edgar Wright	tt0446029
1746501577	1922	1922	The Ghost Breaker	The Ghost Breaker	Green, Alfred E.	Alfred E. Green	tt0013170
1746567601	1932	1932	Down to Earth	Down to Earth	Butler, David	David Butler	tt0022833
1746584379	1936	1936	Strike Me Pink	Strike Me Pink	Taurog, Norman	Norman Taurog	tt0028321
1746507575	1925	1925	Forty Winks	Forty Winks	Urson, Frank; Iribe, Paul	Paul Iribe	tt0015835
1746561468	1932	1932	Hotel Continental	Hotel Continental	Cabanne, Christy	Christy Cabanne	tt0023030
1746561906	1956	1956	Flame of the Islands	Flame of the Islands	Ludwig, Edward	Edward Ludwig	tt0048079
1746454628	1941	1940	Four Shall Die	Four Shall Die	Popkin, Leo C.	William Beaudine	tt0032351
1746532841	1927	1927	The Yankee Clipper	The Yankee Clipper	Julian, Rupert	Rupert Julian	tt0018601
1746561952	1954	1954	Men of the Fighting Lady	Men of the Fighting Lady	Marton, Andrew	Andrew Marton	tt0047230
1746570380	1952	1952	Horizons West	Horizons West	Boetticher, Budd	Budd Boetticher	tt0044722
1746513783	1916	1916	The Crisis	The Crisis	Campbell, Colin	Colin Campbell	tt0006557
1746197984	1970	1970	Hercules in New York	Hercules in New York	Seidelman, Arthur A.	Arthur Allan Seidelman	tt0065832
1746571123	1934	1934	The Gay Divorcee	The Gay Divorcee	Sandrich, Mark	Mark Sandrich	tt0025164
1746578882	1951	1951	The Basketball Fix	The Basketball Fix	Feist, Felix	Felix E. Feist	tt0043320
1746570382	1934	1934	Monte Carlo Nights	Monte Carlo Nights	Nigh, William	William Nigh	tt0025514
1746419924	1988	1988	Dominick and Eugene	Dominick and Eugene	Young, Robert M.	Robert M. Young	tt0095050
1746502564	1916	1916	The Habit of Happiness	The Habit of Happiness	Dwan, Allan	Allan Dwan	tt0006751
1746250392	1976	1976	Marathon Man	Marathon Man	Schlesinger, John	John Schlesinger	tt0074860
1746584008	1935	1935	College Scandal	College Scandal	Nugent, Elliott	Elliott Nugent	tt0026225
1746410597	1986	1986	Little Shop of Horrors	Little Shop of Horrors	Oz, Frank	Frank Oz	tt0091419
1746234803	1966	1966	Lost Command	Lost Command	Robson, Mark	Mark Robson	tt0060637
1746211055	1970	1970	The Dunwich Horror	The Dunwich Horror	Haller, Daniel	Daniel Haller	tt0065669
1746582604	1936	1936	Public Enemy's Wife	Public Enemy's Wife	Grinde, Nick	Nick Grinde	tt0028144
1746464870	1942	1942	Jail House Blues	Jail House Blues	Rogell, Albert S.	Albert S. Rogell	tt0034911
1746567756	1934	1934	Twisted Rails	Twisted Rails	Herman, Albert	Albert Herman	tt0202640
1746208247	1967	1967	Gunn	Gunn	Edwards, Blake	Blake Edwards	tt0061739
1746499993	1948	1948	The Hawk of Powder River	The Hawk of Powder River	Taylor, Ray	Ray Taylor	tt0179209
1746501246	1915	1915	Don Caesar De Bazan	Don Caesar de Bazan	Vignola, Robert G.	Robert G. Vignola	tt0005208
1746569818	1954	1954	Operation Manhunt	Operation Manhunt	Alexander, Jack	Jack Alexander	tt0254632
1746509243	1924	1924	When a Girl Loves	When a Girl Loves	Halperin, Victor Hugo; Wallace, C. R.	Victor Halperin	tt0015486
1746516462	1920	1920	39 East	39 East	Robertson, John S.	John S. Robertson	tt0010945
1746583476	1950	1950	Pygmy Island	Pygmy Island	Berke, William	William Berke	tt0042865
1746562470	1957	1957	Monster from Green Hell	Monster from Green Hell	Crane, Kenneth G.	Kenneth G. Crane	tt0050723
1746210470	1970	1970	Jenny	Jenny	Bloomfield, George	George Bloomfield	tt0064510
1746498283	1917	1916	His Mother's Boy	His Mother's Boy	Schertzinger, Victor L.	Fred Kelsey	tt0335153
1746508394	1925	1925	Man and Maid	Man and Maid	Schertzinger, Victor	Victor Schertzinger	tt0016079
1746582349	1950	1950	Timber Fury	Timber Fury	Ray, Bernard B.	Bernard B. Ray	tt0043049

1746514313	1923	1923	The Ne'er-Do-Well	The Ne'er Do Well	Green, Alfred E.	Alfred E. Green	tt0014306
1746454772	1997	1997	Titanic	Titanic	Cameron, James	James Cameron	tt0120338
1745373794	2002	2002	Death to Smoochy	Death to Smoochy	DeVito, Danny	Danny DeVito	tt0266452
1746514536	1915	1915	The Despoiler	The Despoiler	Barker, Reginald	Reginald Barker	tt0005187
1745395619	2010	2010	The Social Network	The Social Network	Fincher, David	David Fincher	tt1285016
1746569556	1954	1954	The Black Pirates	The Black Pirates	Miner, Allen H.	Allen H. Miner	tt0046787
1746577815	1935	1935	The Wanderer of the Wasteland	Wanderer of the Wasteland	Lovering, Otho	Otho Lovering	tt0027182
1746584157	1935	1935	Two Sinners	Two Sinners	Lubin, Arthur	Arthur Lubin	tt0027146
1746574758	1934	1934	Cheaters	Cheaters	Rosen, Phil	Phil Rosen	tt0024971
1746503598	1920	1920	Sweet Lavender	Sweet Lavender	Powell, Paul	Paul Powell	tt0011745
1746582652	1937	1937	Under the Red Robe	Under the Red Robe	Seastrom, Victor	Victor Sjöström	tt0029712
1746515580	1920	1920	The False Road	The False Road	Niblo, Fred	Fred Niblo	tt0011170
1746474466	1945	1945	Trail to Vengeance	Trail to Vengeance	Fox, Wallace W.	Wallace Fox	tt0038186
1746580618	1937	1937	Manhattan Merry-Go-Round	Manhattan Merry Go Round	Riesner, Charles F.	Charles Reisner	tt0029209
1745302261	2007	2007	Dead Silence	Dead Silence	Wan, James	James Wan	tt0455760
1746475509	1945	1945	Marshal of Laredo	Marshal of Laredo	Springsteen, R. G.	R.G. Springsteen	tt0037902
1746582870	1935	1935	It's a Small World	It's a Small World	Cummings, Irving	Irving Cummings	tt0026539
1746408697	1985	1985	Target	Target	Penn, Arthur	Arthur Penn	tt0090130
1746525069	1921	1921	A Wife's Awakening	A Wife's Awakening	Gasnier, Louis	Louis J. Gasnier	tt0012848
1746438624	1941	1941	Road Agent	Road Agent	Lamont, Charles	Charles Lamont	tt0034114
1746423753	1997	1997	Commandments	Commandments	Taplitz, Daniel	Daniel Taplitz	tt0115927
1746574268	1958	1958	White Wilderness	White Wilderness	Algar, James	James Algar	tt0052389
1746524419	1910	1910	A Lucky Toothache	A Lucky Toothache	Powell, Frank	Frank Powell	tt0001298
1746574888	1935	1935	Little Big Shot	Little Big Shot	Curtiz, Michael	Michael Curtiz	tt0026632
1746578612	1960	1960	For the Love of Mike	For the Love of Mike	Sherman, George	George Sherman	tt0053831
1746569628	1953	1953	Roman Holiday	Roman Holiday	Wyler, William	William Wyler	tt0046250
1746574723	1938	1938	Bulldog Drummond in Africa	Bulldog Drummond in Africa	King, Louis	Louis King	tt0029951
1745342769	2002	2002	Paid in Full	Paid in Full	Stone , Charles	Charles Stone III	tt0259484
1746513712	1923	1923	The Purple Highway	The Purple Highway	Kolker, Henry	Henry Kolker	tt0014384
1746532620	1916	1916	The Abandonment	The Abandonment	MacDonald, Donald	Donald MacDonald	tt0006335
1746271831	1978	1978	The Bad News Bears Go to Japan	The Bad News Bears Go to Japan	Berry, John	John Berry	tt0077199
1746580474	1938	1938	Riders of the Black Hills	Riders of the Black Hills	Sherman, George	George Sherman	tt0030681
1746436658	1943	1943	Leather Burners	Leather Burners	Henabery, Joseph E.	Joseph Henabery	tt0036103
1746272402	1966	1966	The Appaloosa	The Appaloosa	Furie, Sidney J.	Sidney J. Furie	tt0060120
1746580594	1935	1935	Forced Landing	Forced Landing	Brown, Melville	Melville W. Brown	tt0026376
1746571355	1934	1934	The Band Plays On	The Band Plays On	Mack, Russell	Russell Mack	tt0024862
1746584291	1939	1939	Wuthering Heights	Wuthering Heights	Wyler, William	William Wyler	tt0032145
1746566137	1955	1955	Good Morning, Miss Dove	Good Morning, Miss Dove	Koster, Henry	Henry Koster	tt0048130
1746509340	1926	1926	Broadway Billy	Broadway Billy	Brown, Harry J.	Harry Joe Brown	tt0016681
1746533176	1915	1915	A Daughter of the City	A Daughter of the City	Calvert, E. H.	E.H. Calvert	tt0005174
1746570463	1933	1933	Lucky Devils	Lucky Devils	Ince, Ralph	Ralph Ince	tt0024276
1746410160	1986	1986	The Hitcher	The Hitcher	Harmon, Robert	Robert Harmon	tt0091209
1746515806	1921	1921	Love's Plaything	Love's Plaything	Coyle, Walter V.	Walter V. Coyle	tt0220625
1746514601	1916	1916	A Corner in Cotton	A Corner in Cotton	Balshofer, Fred J.	Fred J. Balshofer	tt0157485
1746500721	1925	1925	Fear-Bound	Fear Bound	Nigh, Will	William Nigh	tt0015797
1746578013	1935	1935	Calm Yourself	Calm Yourself	Seitz, George B.	George B. Seitz	tt0026166
1746583142	1940	1940	Little Old New York	Little Old New York	King, Henry	Henry King	tt0032719
1746584924	1940	1940	Those Were the Days!	Those Were the Days!	Reed, Jay Theodore	Theodore Reed	tt0033155
1746524686	1915	1915	Samson	Samson	Lewis, Edgar	Edgar Lewis	tt0006002
1745342027	2001	2001	Town & Country	Town & Country	Chelsom, Peter	Peter Chelsom	tt0141907
1746438312	1945	1945	Blonde Ransom	Blonde Ransom	Beaudine, William	William Beaudine	tt0037546
1746513064	1923	1922	The Prairie Mystery	The Prairie Mystery	Hall, George Edward	George Edwardes	tt0013508
1746577342	1961	1961	Breakfast at Tiffany's	Breakfast at Tiffany's	Edwards, Blake	Blake Edwards	tt0054698
1745342446	2002	2001	Kissing Jessica Stein	Kissing Jessica Stein	Herman-Wurmfeld, Charles	Charles Herman	tt0264761
1746512588	1923	1923	Ruggles of Red Gap	Ruggles of Red Gap	Cruze, James	James Cruze	tt0014423
1746484435	1917	1917	Alias Mrs. Jessop	Alias Mrs. Jessop	Davis, William S.	Will S. Davis	tt0007621
1745309203	2007	2007	The Mist	The Mist	Darabont, Frank	Frank Darabont	tt0884328

1746514814	1916	1916	Stranded	Stranded	Ingraham, Lloyd	Will Louis	tt0269917	
1746486459	1917	1917	The Bar Sinister	The Bar Sinister	Lewis, Edgar	Edgar Lewis	tt0007677	
1746577750	1937	1937	Thunder Trail	Thunder Trail	Barton, Charles	Charles Barton	tt0029669	
1746464890	1941	1941	The Smiling Ghost	The Smiling Ghost	Seiler, Lewis	Lewis Seiler	tt0034204	
1746502870	1916	1916	The Selfish Woman	The Selfish Woman	Hopper, E. Mason; Melford, George	E. Mason Hopper	tt0007322	
1746515048	1917	1917	The Pride of the Clan	The Pride of the Clan	Tourneur, Maurice	Maurice Tourneur	tt0008458	
1746467489	1917	1917	Betsy's Burglar	Betsy's Burglar	Powell, Paul	Paul Powell	tt0007699	
1746555272	1954	1954	Port of Hell	Port of Hell	Schuster, Harold	Harold D. Schuster	tt0047359	
1746523702	1915	1915	The Millionaire Baby	The Millionaire Baby	Marston, Lawrence	Lawrence Marston	tt0005746	
1746585013	1940	1940	I Was an Adventuress	I Was an Adventuress	Ratoff, Gregory	Gregory Ratoff	tt0032620	
1746234180	1967	1966	The Drifter	The Drifter	Matter, Alex	Alex Matter	tt0060347	
1746565561	1932	1932	The Last Man	The Last Man	Higgin, Howard	Howard Higgin	tt0023116	
1746584712	1928	1928	Forbidden Grass	Forbidden Grass	Eldridge, E. M.	Evelyn Nicholas	tt0132971	
1746562455	1953	1953	Walking My Baby Back Home	Walking My Baby Back Home	Bacon, Lloyd	Lloyd Bacon	tt0046531	
1746410530	1984	1984	Heartbreakers	Heartbreakers	Roth, Bobby	Bobby Roth	tt0087397	
1746439090	1999	1999	A Walk on the Moon	A Walk on the Moon	Goldwyn, Tony	Tony Goldwyn	tt0120613	
1746583978	1935	1935	I Live for Love	I Live for Love	Berkeley, Busby	Busby Berkeley	tt0026512	
1745271947	2001	2001	Antitrust	Antitrust	Howitt, Peter	Peter Howitt	tt0218817	
1746491767	1943	1943	The Blocked Trail	The Blocked Trail	Clifton, Elmer	Elmer Clifton	tt0035683	
1746456198	1996	1996	Fly Away Home	Fly Away Home	Ballard, Carroll	Carroll Ballard	tt0116329	
1769970732	1911	1911	Cinderella	Cinderella	Nichols, George O.	George Nichols	tt0001545	
1746583115	1938	1938	Rebecca of Sunnybrook Farm	Rebecca of Sunnybrook Farm	Dwan, Allan	Allan Dwan	tt0030657	
1746504060	1918	1918	Miss Mischief Maker	Miss Mischief Maker	MacDonald, Sherwood	Sherwood MacDonald	tt0009381	
1746584253	1935	1935	Miss Pacific Fleet	Miss Pacific Fleet	Enright, Ray	Ray Enright	tt0026721	
1746249982	1978	1978	Invasion of the Body Snatchers	Invasion of the Body Snatchers	Kaufman, Philip	Philip Kaufman	tt0077745	
1746491876	1947	1947	That's My Man	That's My Man	Borzage, Frank	Frank Borzage	tt0039022	
1746580949	1961	1961	Eve and the Handyman	Eve and the Handyman	Meyer, Russ	Russ Meyer	tt0054859	
1746583836	1929	1929	Dangerous Curves	Dangerous Curves	Mendes, Lothar	Lothar Mendes	tt0019800	
1746584275	1935	1935	Kentucky Blue Streak	Kentucky Blue Streak	Johnson, Raymond K.	Bernard B. Ray	tt0026563	
1746504535	1918	1918	Shackled	Shackled	Barker, Reginald	Reginald Barker	tt0009599	
1746509205	1949	1949	Slightly French	Slightly French	Sirk, Douglas	Douglas Sirk	tt0041885	
**1746562358**	**1953**	**1953**	**Down Laredo Way**	**Down Laredo Way**	**Witney, William**	**William Witney**	**tt0045703**	1
1746532493	1927	1927	Dearie	Dearie	Mayo, Archie	Archie Mayo	tt0017795	
1746550042	1955	1955	Battle Cry	Battle Cry	Walsh, Raoul	Raoul Walsh	tt0047860	
1746208967	1968	1968	Head	Head	Rafelson, Bob	Bob Rafelson	tt0063049	
1746492424	1948	1948	An Innocent Affair	An Innocent Affair	Bacon, Lloyd	Lloyd Bacon	tt0040473	
1746581123	1961	1961	Blueprint for Robbery	Blueprint for Robbery	Hopper, Jerry	Jerry Hopper	tt0054693	
1769970677	1968	1968	Sugar Daddy	Sugar Daddy	Crane, Larry	Larry Crane	tt0291497	
1746466638	1917	1917	The Crab	The Crab	Edwards, Walter	Walter Edwards	tt0168613	
1746393243	1988	1988	Brain Damage	Brain Damage	Henenlotter, Frank	Frank Henenlotter	tt0094793	
1746408465	1987	1987	Some Kind of Wonderful	Some Kind of Wonderful	Deutch, Howard	Howard Deutch	tt0094006	
1746491775	1944	1944	Summer Storm	Summer Storm	Sirk, Douglas	Douglas Sirk	tt0037325	
1746500497	1924	1924	Being Respectable	Being Respectable	Rosen, Philip	Phil Rosen	tt0014710	
1746497065	1919	1919	When Men Desire	When Men Desire	Edwards, J. Gordon	J. Gordon Edwards	tt0010876	
1746514328	1914	1914	Classmates	Classmates	Kirkwood, James	James Kirkwood	tt0003777	
1746584476	1930	1930	Scarlet Pages	Scarlet Pages	Enright, Ray	Ray Enright	tt0021337	
1745373324	2008	2008	Bedtime Stories	Bedtime Stories	Shankman, Adam	Adam Shankman	tt0960731	
1746525018	1919	1919	The Woman Under Cover	The Woman Under Cover	Seigmann, George	George Siegmann	tt0010914	
1746464859	1944	1943	The Spider Woman	The Spider Woman	Neill, Roy William	Roy William Neill	tt0037303	
1746530671	1949	1949	Mr. Soft Touch	Mr. Soft Touch	Levin, Henry; Douglas, Gordon	Gordon Douglas	tt0041663	
1746509530	1926	1926	Lone Hand Saunders	Lone Hand Saunders	Eason, B. Reeves	B. Reeves Eason	tt0017076	
1746514092	1914	1914	A Fight for Freedom; or, Exiled to Siberia	A Fight for Freedom; Or, Exiled to Siberia			tt0003957	
1746577500	1933	1933	Adorable	Adorable	Dieterle, Wilhelm	William Dieterle	tt0023740	
1746453798	1941	1941	Mr. District Attorney in the Carter Case	Mr. District Attorney in the Carter Case	Vorhaus, Bernard	Bernard Vorhaus	tt0035090	
1746508051	1924	1924	The Reckless Age	The Reckless Age	Pollard, Harry	Harry A. Pollard	tt0015263	
1746585039	1940	1940	Rollin' Home to Texas	Rollin' Home to Texas	Herman, Al	Albert Herman	tt0032997	
1746500563	1927	1927	The Swift Shadow	The Swift Shadow	Storm, Jerome	Jerome Storm	tt0167433	

1746439476	1941	1941	Hurry, Charlie, Hurry	Hurry, Charlie, Hurry	Roberts, Charles E.	Charles E. Roberts	tt0033733
1746514943	1916	1916	The Island of Surprise	The Island of Surprise	Scardon, Paul	Paul Scardon	tt0006873
1746409939	1985	1985	Brewster's Millions	Brewster's Millions	Hill, Walter	Walter Hill	tt0088850
1746574428	1959	1959	Tarzan, the Ape Man	Tarzan, the Ape Man	Newman, Joseph	Joseph M. Newman	tt0053335
1746465266	1947	1947	Seven Were Saved	Seven Were Saved	Pine, William H.	William H. Pine	tt0039816
1746583428	1928	1928	The Cameraman	The Cameraman	Sedgwick, Edward	Edward Sedgwick	tt0018742
1746572550	1932	1932	No Greater Love	No Greater Love	Seiler, Lewis	Lewis Seiler	tt0023275
1746498273	1918	1918	Heart of the Sunset	Heart of the Sunset	Powell, Frank	Frank Powell	tt0184556
1746569822	1959	1959	Imitation of Life	Imitation of Life	Sirk, Douglas	Douglas Sirk	tt0052918
1746524414	1927	1927	Hell Hounds of the Plains	Hell Hounds of the Plains	Wilson, Ben	Jacques Jaccard	tt0127583
1746394690	1986	1986	Troll	Troll	Buechler, John Carl	John Carl Buechler	tt0092115
1746578898	1951	1951	I Was a Communist for the F.B.I.	I Was a Communist for the F.B.I.	Douglas, Gordon	Gordon Douglas	tt0043665
1746575021	1936	1936	Libeled Lady	Libeled Lady	Conway, Jack	Jack Conway	tt0027884
1746574059	1960	1960	Because They're Young	Because They're Young	Wendkos, Paul	Paul Wendkos	tt0053641
1746566043	1954	1953	Give a Girl a Break	Give a Girl a Break	Donen, Stanley	Stanley Donen	tt0045822
1769971188	1969	1969	Submission	Submission	Savage, Allen	Allen Savage	tt0258997
1745357626	2008	2008	The X-Files: I Want to Believe	The X Files: I Want to Believe	Carter, Chris	Chris Carter	tt0443701
1746520771	1949	1949	The Mysterious Desperado	The Mysterious Desperado	Selander, Lesley	Lesley Selander	tt0041675
1746570878	1933	1933	Wild Boys of the Road	Wild Boys of the Road	Wellman, William A.	William A. Wellman	tt0024772
1746584764	1930	1930	War Nurse	War Nurse	Selwyn, Edgar	Edgar Selwyn	tt0021531
1746584726	1928	1928	Crooks Can't Win	Crooks Can't Win	Arthur, George M.	George M. Arthur	tt0018805
1746216883	1968	1968	Guns for San Sebastian	Guns for San Sebastian	Verneuil, Henri	Henri Verneuil	tt0062713
1746585180	1929	1929	The Cohens and Kellys in Atlantic City	The Cohens and Kellys in Atlantic City	Craft, William James	William James Craft	tt0019779
1746580915	1935	1935	King Solomon of Broadway	King Solomon of Broadway	Crosland, Alan	Alan Crosland	tt0026570
1745294019	2007	2006	Kurt Cobain About a Son	Kurt Cobain About a Son	Schnack, A. J.	AJ Schnack	tt0790769
1746513465	1923	1923	Daddy	Daddy	Hopper, E. Mason	E. Mason Hopper	tt0013965
1746515675	1922	1922	Timothy's Quest	Timothy's Quest	Olcott, Sidney	Sidney Olcott	tt0013682
1746483867	1919	1919	His Father's Wife	His Father's Wife	Crane, Frank	Frank Hall Crane	tt0205129
1746410698	1993	1993	Rookie of the Year	Rookie of the Year	Stern, Daniel	Daniel Stern	tt0107985
1746474535	1946	1946	Tall, Tan and Terrific	Tall, Tan, and Terrific	Pollard, Bud	Bud Pollard	tt0139634
1745359827	2010	2010	Secretariat	Secretariat	Wallace, Randall	Randall Wallace	tt1028576
1746498687	1917	1917	The Dazzling Miss Davison	The Dazzling Miss Davison	Powell, Frank	Frank Powell	tt0007843
1746408719	1985	1985	Invasion U.S.A.	Invasion U.S.A.	Zito, Joseph	Joseph Zito	tt0089348
1746577204	1959	1959	The Monster of Piedras Blancas	The Monster of Piedras Blancas	Berwick, Irvin	Irvin Berwick	tt0051947
1746502362	1916	1916	Dulcie's Adventure	Dulcie's Adventure	Kirkwood, James	James Kirkwood	tt0125069
1746508997	1926	1926	Doubling With Danger	Doubling with Danger	Dunlap, Scott R.	Scott R. Dunlap	tt0130634
1746503566	1918	1918	For Sale	For Sale	Wright, Fred	Fred E. Wright	tt0009085
1746583896	1930	1930	Crazy That Way	Crazy That Way	MacFadden, Hamilton	Hamilton MacFadden	tt0020797
1746543063	1916	1916	Her Father's Gold	Her Father's Gold	Moore, W. Eugene	Eugene Moore	tt0157745
1746585470	1940	1940	Florian	Florian	Marin, Edwin L.	Edwin L. Marin	tt0032479
1746574687	1936	1936	White Fang	White Fang	Butler, David	David Butler	tt0028501
1746553597	1964	1964	Lilith	Lilith	Rossen, Robert	Robert Rossen	tt0058294
1746474394	1945	1945	How Doooo You Do!!!	How DOooo You Do	Murphy, Ralph	Ralph Murphy	tt0037796
1746583594	1950	1950	Rider from Tucson	Rider from Tucson	Selander, Lesley	Lesley Selander	tt0042891
1746393251	1981	1981	Sharky's Machine	Sharky's Machine	Reynolds, Burt	Burt Reynolds	tt0083064
1746394422	1981	1981	Halloween II	Halloween II	Rosenthal, Rick	Rick Rosenthal	tt0082495
1746561974	1953	1953	The Maze	The Maze		William Cameron Menzies	tt0046057
1746577222	1951	1950	The Man Who Cheated Himself	The Man Who Cheated Himself	Feist, Felix E.	Felix E. Feist	tt0042707
1746464801	1944	1944	It Happened Tomorrow	It Happened Tomorrow	Clair, René	René Clair	tt0036962
1746524335	1915	1915	The Lily of Poverty Flat	The Lily of Poverty Flat	Middleton, George E.	George E. Middleton	tt0005623
1746508594	1925	1925	The Love Gamble	The Love Gamble	Le Saint, Edward	Edward LeSaint	tt0016042
1746524806	1916	1916	Diplomacy	Diplomacy	Olcott, Sidney	Sidney Olcott	tt0006595
1746584600	1929	1930	Midnight Daddies	Midnight Daddies	Sennett, Mack	Mack Sennett	tt0020169
1746516273	1921	1921	Outlawed	Outlawed	Neitz, Alvin J.	Alan James	tt0145951
1746422916	2000	2000	Lucky Numbers	Lucky Numbers	Ephron, Nora	Nora Ephron	tt0219952
1746582425	1935	1935	The Outlaw Tamer	The Outlaw Tamer	McGowan, J. P.	J.P. McGowan	tt0025613
1746583897	1931	1931	City Lights	City Lights	Chaplin, Charles	Charles Chaplin	tt0021749

1746475654	1947	1947	Song of Love	Song of Love	Brown, Clarence	Clarence Brown	tt0039850
1746583871	1928	1928	The Four-Footed Ranger	The Four Footed Ranger	Paton, Stuart	Stuart Paton	tt0018911
1746581369	1961	1961	The Errand Boy	The Errand Boy	Lewis, Jerry	Jerry Lewis	tt0054853
1746394389	1984	1984	2010	2010	Hyams, Peter	Peter Hyams	tt0086837
1746577970	1938	1938	Angels with Dirty Faces	Angels with Dirty Faces	Curtiz, Michael	Michael Curtiz	tt0029870
1746422597	1999	1999	The Green Mile	The Green Mile	Darabont, Frank	Frank Darabont	tt0120689
1746492501	1945	1945	The Great John L.	The Great John L.	Tuttle, Frank	Frank Tuttle	tt0037750
1746577729	1938	1938	The Adventures of Marco Polo	The Adventures of Marco Polo	Mayo, Archie; Cromwell, John	Archie Mayo	tt0029842
1746419291	1940	1940	The Captain Is a Lady	The Captain Is a Lady	Sinclair, Robert B.	Robert B. Sinclair	tt0032305
1746512114	1927	1927	The Haunted Ship	The Haunted Ship	Sheldon, Forrest K.	Forrest Sheldon	tt0017969
1746437832	1942	1942	White Cargo	White Cargo	Thorpe, Richard	Richard Thorpe	tt0035553
1746422212	1994	1994	3 Ninjas Kick Back	3 Ninjas Kick Back	Kanganis, Charles T.	Charles T. Kanganis	tt0109015
1746513848	1910	1910	The Mexican's Faith	The Mexican's Faith	Hamilton (?), Gilbert P.	Gilbert M. 'Broncho Billy' Anderson	tt0001317
1746575081	1935	1935	Metropolitan	Metropolitan	Boleslawski, Richard	Richard Boleslawski	tt0026705
1746520175	1927	1927	A Kiss in a Taxi	A Kiss in a Taxi	Badger, Clarence	Clarence G. Badger	tt0018058
1746577363	1950	1950	Outlaw Gold	Outlaw Gold	Fox, Wallace W.	Wallace Fox	tt0042822
1746500133	1924	1924	Another Man's Wife	Another Man's Wife	Mitchell, Bruce	Bruce Mitchell	tt0014677
1746514287	1916	1916	The Pearl of Paradise	The Pearl of Paradise	Pollard, Harry	Harry A. Pollard	tt0163146
1746584143	1936	1936	Yellow Cargo	Yellow Cargo	Wilbur, Crane	Crane Wilbur	tt0028521
1746584749	1929	1929	Stairs of Sand	Stairs of Sand	Brower, Otto	Otto Brower	tt0020448
1746249488	1975	1975	Rooster Cogburn	Rooster Cogburn	Millar, Stuart	Stuart Millar	tt0073636
1746516013	1922	1922	A Wide-Open Town	A Wide Open Town	Ince, Ralph	Ralph Ince	tt0013764
1746584886	1940	1940	The Ape	The Ape	Nigh, William	William Nigh	tt0032215
1746555111	1956	1956	The Oklahoma Woman	The Oklahoma Woman	Corman, Roger	Roger Corman	tt0049570
1746523841	1916	1916	The Pursuing Vengeance	The Pursuing Vengeance	Sabine, Martin	Martin Sabine	tt0158867
1746520534	1926	1926	Western Trails	Western Trails	Carpenter, H. B.	Horace B. Carpenter	tt0177390
1746419774	1989	1989	Harlem Nights	Harlem Nights	Murphy, Eddie	Eddie Murphy	tt0097481
1746580952	1958	1958	War of the Satellites	War of the Satellites	Corman, Roger	Roger Corman	tt0052379
1745309150	2007	2007	Broken English	Broken English	Cassavetes, Zoe	Zoe R. Cassavetes	tt0772157
1746570261	1934	1934	The Silver Streak	The Silver Streak	Atkins, Tommy	Thomas Atkins	tt0027000
1746422261	1998	1998	Buffalo '66	Buffalo '66	Gallo, Vincent	Vincent Gallo	tt0118789
1746514986	1927	1927	The Slaver	The Slaver	Revier, Harry	Harry Revier	tt0164894
1746583992	1940	1940	Behind the News	Behind the News	Santley, Joseph	Joseph Santley	tt0032246
1746514941	1927	1927	Irish Hearts	Irish Hearts	Haskin, Byron	Byron Haskin	tt0018028
1746583327	1930	1930	Song of the Flame	The Song of the Flame	Crosland, Alan	Alan Crosland	tt0021404
1746569734	1954	1954	Flame and the Flesh	Flame and the Flesh	Brooks, Richard	Richard Brooks	tt0046982
1746580930	1961	1961	The Choppers	The Choppers	Jason, Leigh	Leigh Jason	tt0054744
1746436034	1940	1940	Murder over New York	Murder Over New York	Lachman, Harry	Harry Lachman	tt0032819
1746564761	1952	1952	Target	Target	Gilmore, Stuart	Stuart Gilmore	tt0045218
1746198068	1967	1966	The Crazy World of Laurel and Hardy	The Crazy World of Laurel and Hardy			tt0061528
1746248750	1973	1973	Oklahoma Crude	Oklahoma Crude	Kramer, Stanley	Stanley Kramer	tt0070472
1746218663	1972	1972	Silent Running	Silent Running	Trumbull, Douglas	Douglas Trumbull	tt0067756
1746575051	1934	1934	Madame Spy	Madame Spy	Freund, Karl	Karl Freund	tt0025444
1746583209	1931	1931	Red Fork Range	Red Fork Range	Neitz, Alvin J.	Alan James	tt0022299
1746583560	1930	1929	The Great Gabbo	The Great Gabbo	Cruze, James	James Cruze	tt0019946
1746574665	1938	1938	Gold Is Where You Find It	Gold Is Where You Find It	Curtiz, Michael	Michael Curtiz	tt0030192
1746507886	1949	1949	Bride of Vengeance	Bride of Vengeance	Leisen, Mitchell	Mitchell Leisen	tt0041209
1746501050	1916	1916	The Two Edged Sword	The Two Edged Sword	Baker, George D.	George D. Baker	tt0159059
1746580284	1935	1935	One Way Ticket	One Way Ticket	Biberman, Herbert	Herbert J. Biberman	tt0026825
1746486508	1918	1918	Hearts or Diamonds?	Hearts or Diamonds?	King, Henry	Henry King	tt0009151
1746574123	1956	1956	Johnny Concho	Johnny Concho	McGuire, Don	Don McGuire	tt0049384
1746583517	1928	1928	Black Butterflies	Black Butterflies	Horne, James W.	James W. Horne	tt0018704
1746501048	1915	1915	The Barnstormers	The Barnstormers	Horne, James W.	James W. Horne	tt0004939
1746567961	1933	1933	Penthouse	Penthouse	Van Dyke, W. S.	W.S. Van Dyke	tt0024435
1746497295	1922	1922	The Trail of the Axe	Trail of the Axe	Warde, Ernest C.	Ernest C. Warde	tt0173348
1746573847	1957	1957	Voodoo Woman	Voodoo Woman	Cahn, Edward L.	Edward L. Cahn	tt0051174
1746574487	1935	1935	Becky Sharp	Becky Sharp	Mamoulian, Rouben	Rouben Mamoulian	tt0026104

1746577938	1939	1939	Second Fiddle	Second Fiddle	Lanfield, Sidney	Sidney Lanfield	tt0031907
1746574940	1933	1933	When Ladies Meet	When Ladies Meet	Beaumont, Harry	Harry Beaumont	tt0024763
1746393755	1982	1982	Conan the Barbarian	Conan the Barbarian	Milius, John	John Milius	tt0082198
1746580527	1937	1937	Hopalong Rides Again	Hopalong Rides Again	Selander, Lesley	Lesley Selander	tt0029020
1746581183	1960	1960	Five Bold Women	Five Bold Women	López-Portillo, Jorge	Jorge López Portillo	tt0052807
1746533038	1923	1923	The Devil's Dooryard	The Devil's Dooryard	King, Lewis	Louis King	tt0013988
1746423493	1992	1992	Ladybugs	Ladybugs	Furie, Sidney J.	Sidney J. Furie	tt0104670
1746570459	1933	1933	The Silver Cord	The Silver Cord	Cromwell, John	John Cromwell	tt0024560
1746208138	1969	1969	Hard Contract	Hard Contract	Pogostin, S. Lee	S. Lee Pogostin	tt0064406
1746465160	1947	1947	Love from a Stranger	Love from a Stranger	Whorf, Richard	Richard Whorf	tt0039586
1746464986	1944	1944	The Falcon in Hollywood	The Falcon in Hollywood	Douglas, Gordon	Gordon Douglas	tt0036810
1746567113	1952	1952	Montana Belle	Montana Belle	Dwan, Allan	Allan Dwan	tt0044919
1746184582	1910	1910	Blasted Hopes	Blasted Hopes			tt0221025
1746437850	1943	1943	Riders of the Deadline	Riders of the Deadline	Selander, Lesley	Lesley Selander	tt0037224
1746560773	1932	1932	Son of Oklahoma	Son of Oklahoma	Bradbury, Robert N.	Robert N. Bradbury	tt0023494
1746520327	1948	1948	Walk a Crooked Mile	Walk a Crooked Mile	Douglas, Gordon	Gordon Douglas	tt0040947
1746553584	1964	1964	Mary Poppins	Mary Poppins	Stevenson, Robert	Robert Stevenson	tt0058331
1746578841	1951	1951	Along the Great Divide	Along the Great Divide	Walsh, Raoul	Raoul Walsh	tt0043276
1746497897	1920	1920	The Unfortunate Sex	The Unfortunate Sex	La Maie, Elsier	Elsier La Maie	tt0202646
1746583180	1938	1938	Sinners in Paradise	Sinners in Paradise	Whale, James	James Whale	tt0030754
1746500462	1927	1927	On Your Toes	On Your Toes	Newmeyer, Fred	Fred C. Newmeyer	tt0018220
1746487838	1917	1917	The Small Town Guy	The Small Town Guy	Windom, L. C.	Lawrence C. Windom	tt0182430
1746574855	1933	1933	Parachute Jumper	Parachute Jumper	Green, Alfred E.	Alfred E. Green	tt0024426
1746524477	1910	1910	The Piece of Lace	The Piece of Lace	Miller, Ashley	Ashley Miller	tt1542761
1746584230	1936	1936	Mister Cinderella	Mister Cinderella	Sedgwick, Edward	Edward Sedgwick	tt0027975
1746410891	1990	1990	Bad Influence	Bad Influence	Hanson, Curtis	Curtis Hanson	tt0099091
1746455400	1996	1996	Citizen Ruth	Citizen Ruth	Payne, Alexander	Alexander Payne	tt0115906
1746514135	1923	1923	Speed King	Speed King	Jones, Grover	Grover Jones	tt0166357
1746210300	1970	1970	The Psycho Lover	The Psycho Lover	O'Neil, Robert Vincent	Robert Vincent O'Neill	tt0168137
1746524168	1914	1914	The Yellow Traffic	The Yellow Traffic		Olaf Skavlan	tt0004840
1746580905	1935	1936	Sunset of Power	Sunset of Power	Taylor, Ray	Ray Taylor	tt0028326
1746394733	1986	1986	Heartbreak Ridge	Heartbreak Ridge	Eastwood, Clint	Clint Eastwood	tt0091187
1746569852	1958	1958	The Missouri Traveler	The Missouri Traveler	Hopper, Jerry	Jerry Hopper	tt0051936
1746491785	1945	1945	Lady on a Train	Lady on a Train	David, Charles	Charles David	tt0037859
1745374028	2008	2008	First Sunday	First Sunday	Talbert, David E.	David E. Talbert	tt0486578
1746569948	1956	1956	Man from Del Rio	Man from Del Rio	Horner, Harry	Harry Horner	tt0049472
1746419762	1991	1992	Tribulation 99: Alien Anomalies Under America	Tribulation 99: Alien Anomalies Under Ame	Baldwin, Craig	Craig Baldwin	tt0105639
1746504760	1921	1921	Broadway Buckaroo	Broadway Buckaroo		Phil Goldstone	tt3355294
1746492753	1946	1946	The Well Groomed Bride	The Well Groomed Bride	Lanfield, Sidney	Sidney Lanfield	tt0039098
1746508523	1924	1924	The Next Corner	The Next Corner	Wood, Sam	Sam Wood	tt0015173
1746507754	1924	1924	Judgment of the Storm	The Judgment of the Storm	Andrews, Del	Del Andrews	tt0015027
1746579013	1960	1960	Desire in the Dust	Desire in the Dust	Claxton, William F.	William F. Claxton	tt0053762
1746508697	1926	1926	The Better Man	The Better Man	Dunlap, Scott R.	Scott R. Dunlap	tt0016644
1746573811	1957	1957	The Guns of Fort Petticoat	The Guns of Fort Petticoat	Marshall, George	George Marshall	tt0050470
1746487284	1918	1918	The Accidental Honeymoon	The Accidental Honeymoon	Perret, Léonce	Léonce Perret	tt0008813
1746573291	1932	1932	Lady with a Past	Lady with a Past	Griffith, Edward H.	Edward H. Griffith	tt0023110
1746486133	1917	1917	A Roadside Impresario	A Roadside Impresario	Crisp, Donald	Donald Crisp	tt0182392
1746524500	1927	1927	What Happened to Father	What Happened to Father?	Adolfi, John G.	John G. Adolfi	tt0018559
1746512314	1922	1922	Boy Crazy	Boy Crazy	Seiter, William A.	William A. Seiter	tt0012969
1746520387	1924	1924	For Another Woman	For Another Woman	Kirkland, David	David Kirkland	tt0014922
1746584254	1935	1935	Adventurous Knights	Adventurous Knights	Roberts, C. Edward	Charles E. Roberts	tt0026039
1746566189	1957	1956	The Naked Eye	The Naked Eye	Stoumen, Louis Clyde	Louis Clyde Stoumen	tt0049538
1746566099	1957	1957	Satchmo the Great	Satchmo the Great			tt0135642
1746209273	1966	1966	Frankie and Johnny	Frankie and Johnny	De Cordova, Frederick	Frederick De Cordova	tt0060429
1746585495	1939	1939	Code of the Secret Service	Code of the Secret Service	Smith, Noel	Noel M. Smith	tt0031166
1746554643	1963	1963	The Haunting	The Haunting	Wise, Robert	Robert Wise	tt0057129
1746583179	1935	1935	The Keeper of the Bees	The Keeper of the Bees	Cabanne, Christy	Christy Cabanne	tt0026562

1746572021	1931	1931	Sea Ghost	The Sea Ghost	Nigh, William	William Nigh	tt0022352
1745340902	2002	2002	Queen of the Damned	Queen of the Damned	Rymer, Michael	Michael Rymer	tt0238546
1746533051	1927	1927	Held by the Law	Held by the Law	Laemmle, Edward	Ernst Laemmle	tt0017978
1746475524	1947	1946	Lady in the Lake	Lady in the Lake	Montgomery, Robert	Robert Montgomery	tt0039545
1746562013	1953	1953	Mister Scoutmaster	Mister Scoutmaster	Levin, Henry	Henry Levin	tt0046081
1746410375	1986	1986	Deadly Friend	Deadly Friend	Craven, Wes	Wes Craven	tt0090917
1746562414	1957	1957	Ghost Diver	Ghost Diver	Einfeld, Richard; White, Merrill G.	Richard Einfeld	tt0050431
1746509267	1924	1924	Missing Daughters	Missing Daughters	Clifford, William H.	William H. Clifford	tt0015143
1769970501	1967	1967	Hip, Hot and 21	Hip Hot and 21	Berry, Dale	Dale Berry	tt0140168
1746563639	1953	1953	Appointment in Honduras	Appointment in Honduras	Tourneur, Jacques	Jacques Tourneur	tt0045512
1746514988	1916	1916	A Knight of the Range	A Knight of the Range	Jaccard, Jacques	Jacques Jaccard	tt0006901
1746582574	1937	1937	God's Country and the Woman	God's Country and the Woman	Keighley, William	William Keighley	tt0028941
1746520411	1949	1949	Renegades of the Sage	Renegades of the Sage	Nazarro, Ray	Ray Nazarro	tt0041801
1746420996	1983	1983	Never Cry Wolf	Never Cry Wolf	Ballard, Carroll	Carroll Ballard	tt0086005
1746211080	1974	1974	The Taking of Pelham One Two Three	The Taking of Pelham One Two Three	Sargent, Joseph	Joseph Sargent	tt0072251
1745303075	2005	2005	Kicking & Screaming	Kicking & Screaming	Dylan, Jesse	Jesse Dylan	tt0384642
1746409370	1983	1983	To Be or Not to Be	To Be or Not to Be	Johnson, Alan	Alan Johnson	tt0086450
1769970701	1968	1968	In Hot Blood	In Hot Blood	Rhewdnal, Leo J.	Joel Landwehr	tt0221263
1746583975	1935	1935	We're in the Money	We're in the Money	Enright, Ray	Ray Enright	tt0027188
1746499936	1945	1945	Wildfire	Wildfire	Tansey, Robert	Robert Emmett Tansey	tt0038254
1746500447	1949	1949	You're My Everything	You're My Everything	Lang, Walter	Walter Lang	tt0042060
1746501744	1915	1915	The Garden of Lies	Garden of Lies	Pratt, John H.	Jack Pratt	tt0005374
1746464823	1941	1941	Road Show	Road Show	Roach, Hal	Hal Roach	tt0034115
1746585428	1940	1940	Granny Get Your Gun	Granny Get Your Gun	Amy, George	George Amy	tt0032550
1746435196	1943	1943	Frontier Badmen	Frontier Badmen	Beebe, Ford; McGann, William	Ford Beebe	tt0035904
1746577030	1962	1962	Almost Angels	Almost Angels	Previn, Steven	Steve Previn	tt0055740
1746570413	1933	1933	Luxury Liner	Luxury Liner	Mendes, Lothar	Lothar Mendes	tt0024281
1746581302	1951	1951	Target Unknown	Target Unknown	Sherman, George	George Sherman	tt0044108
1746583400	1928	1928	The Awakening	The Awakening	Fleming, Victor	Victor Fleming	tt0018666
1746580662	1937	1937	Two Fisted Sheriff	Two Fisted Sheriff	Barsha, Leon	Leon Barsha	tt0029703
1746408828	1991	1991	The Rocketeer	The Rocketeer	Johnston, Joe	Joe Johnston	tt0102803
1745395648	2009	2009	Brief Interviews with Hideous Men	Brief Interviews with Hideous Men	Krasinski, John	John Krasinski	tt0790627
1745359539	2010	2010	Stonewall Uprising	Stonewall Uprising	Davis, Kate ; Heilbroner, David	Kate Davis	tt1562450
1746568327	1934	1934	We're Not Dressing	We're Not Dressing	Taurog, Norman	Norman Taurog	tt0025965
1746438321	1941	1941	Our Wife	Our Wife	Stahl, John M.	John M. Stahl	tt0033986
1746509540	1926	1926	Private Izzy Murphy	Private Izzy Murphy	Bacon, Lloyd	Lloyd Bacon	tt0017290
1769969891	1912	1912	The Legend of the Lost Arrow	The Legend of the Lost Arrow	Bosworth, Hobart	Hobart Bosworth	tt0489169
1746248762	1974	1974	The Bat People	The Bat People	Jameson, Jerry	Jerry Jameson	tt0071198
1769971176	1969	1969	The Bedspread	The Bed Spread			tt0211234
1769970783	1915	1915	The Patent Food Conveyor	Patent Food Conveyor	Williams, C. Jay	C.J. Williams	tt0437411
1746583652	1930	1930	The Way of All Men	The Way of All Men	Lloyd, Frank	Frank Lloyd	tt0021535
1746474084	1945	1945	The Big Show-Off	The Big Show Off	Bretherton, Howard	Howard Bretherton	tt0037541
1746504006	1921	1921	Little Miss Hawkshaw	Little Miss Hawkshaw	Harbaugh, Carl	Carl Harbaugh	tt0012399
1746515033	1910	1910	Muggsy's First Sweetheart	Muggsy's First Sweetheart	Griffith, D. W.	D.W. Griffith	tt0001333
1746410743	1991	1991	McBain	McBain	Glickenhaus, James	James Glickenhaus	tt0102422
1746581468	1950	1950	Trail of the Rustlers	Trail of the Rustlers	Nazarro, Ray	Ray Nazarro	tt0043062
1746495877	1918	1918	Pals First	Pals First	Carewe, Edwin	Edwin Carewe	tt0009476
1746559814	1964	1964	Send Me No Flowers	Send Me No Flowers	Jewison, Norman	Norman Jewison	tt0058571
1746567269	1933	1933	Under Secret Orders	Under Secret Orders	Newfield, Sam	Sam Newfield	tt0024717
1746513787	1915	1915	The Wily Chaperon	The Wily Chaperon	Ricketts, Thomas	Tom Ricketts	tt0437590
1746542664	1950	1950	Covered Wagon Raid	Covered Wagon Raid	Springsteen, R. G.	R.G. Springsteen	tt0179737
1746566005	1955	1954	Young at Heart	Young at Heart	Douglas, Gordon	Gordon Douglas	tt0047688
1746422179	1997	1997	Rosewood	Rosewood	Singleton, John	John Singleton	tt0120036
1746502649	1922	1922	The Girl Who Ran Wild	The Girl Who Ran Wild	Julian, Rupert	Rupert Julian	tt0013173
1746562319	1953	1953	No Escape	No Escape	Bennett, Charles	Charles Bennett	tt0046130
1746584569	1931	1931	Millie	Millie	Dillon, John Francis	John Francis Dillon	tt0022149
1746235704	1974	1974	Sugar Hill	Sugar Hill	Maslansky, Paul	Paul Maslansky	tt0072225

1745302608	2007	2007	August Rush	August Rush	Sheridan, Kirsten	Kirsten Sheridan	tt0426931
1746575064	1934	1934	Now I'll Tell	Now I'll Tell		Edwin J. Burke	tt0025579
1746474810	1948	1948	Mr. Blandings Builds His Dream House	Mr. Blandings Builds His Dream House	Potter, H. C.	H.C. Potter	tt0040613
1746270151	1980	1980	Willie & Phil	Willie & Phil	Mazursky, Paul	Paul Mazursky	tt0081758
1746555949	1956	1956	Secrets of Life	Secrets of Life	Algar, James	James Algar	tt0049733
1746574314	1959	1959	A Stranger in My Arms	A Stranger in My Arms	Kautner, Helmut	Helmut Käutner	tt0051358
1746409981	1991	1991	Scenes from a Mall	Scenes from a Mall	Mazursky, Paul	Paul Mazursky	tt0102849
1746562300	1957	1957	Outlaw Queen	Outlaw Queen	Greene, Herbert S.	Herbert S. Greene	tt0050811
1746260547	1980	1980	Somewhere in Time	Somewhere in Time	Szwarc, Jeannot	Jeannot Szwarc	tt0081534
1746580652	1936	1936	The Lion's Den	The Lion's Den	Newfield, Sam	Sam Newfield	tt0027890
1746393232	1988	1988	Night of the Demons	Night of the Demons	Tenney, Kevin S.	Kevin Tenney	tt0093624
1746424188	1943	1943	The Avenging Rider	The Avenging Rider	Nelson, Sam	Sam Nelson	tt0035655
1746570113	1933	1933	What--No Beer?	What No Beer?	Sedgwick, Edward	Edward Sedgwick	tt0024762
1746409061	1981	1981	The Funhouse	The Funhouse	Hooper, Tobe	Tobe Hooper	tt0082427
1746474527	1946	1946	Gunning for Vengeance	Gunning for Vengeance	Nazarro, Ray	Ray Nazarro	tt0038581
1746533003	1915	1915	The Luring Lights	The Luring Lights		Robert G. Vignola	tt0005668
1746509034	1925	1925	The Mad Marriage	The Mad Marriage	Donovan, Frank P.	Frank P. Donovan	tt0016062
1746218725	1973	1972	Sisters	Sisters	De Palma, Brian	Brian De Palma	tt0070698
1746553793	1965	1965	The Bounty Killer	The Bounty Killer	Bennet, Spencer Gordon	Spencer Gordon Bennet	tt0058988
1746419180	1983	1983	The Right Stuff	The Right Stuff	Kaufman, Philip	Philip Kaufman	tt0086197
1746496274	1917	1917	The Gun Fighter	The Gun Fighter	Hart, William S.	William S. Hart	tt0008036
1746582419	1937	1937	Counsel for Crime	Counsel for Crime	Brahm, John	John Brahm	tt0028743
1746573968	1957	1957	The Pride and the Passion	The Pride and the Passion	Kramer, Stanley	Stanley Kramer	tt0050858
1746453533	1943	1943	Sagebrush Law	Sagebrush Law	Nelson, Sam	Sam Nelson	tt0035282
1746419489	1940	1940	Gallant Sons	Gallant Sons		George B. Seitz	tt0032503
1746574923	1937	1937	A Doctor's Diary	A Doctor's Diary	Vidor, Charles	Charles Vidor	tt0028793
1746566674	1934	1934	Two Heads on a Pillow	Two Heads on a Pillow	Nigh, William	William Nigh	tt0025924
1746583333	1929	1929	Sea Fury	Sea Fury	Melford, George	George Melford	tt0020379
1746562383	1957	1957	God Is My Partner	God Is My Partner	Claxton, William F.	William F. Claxton	tt0050447
1746520595	1926	1926	West of the Rainbow's End	West of the Rainbow's End	Cohn, Bennett	Bennett Cohen	tt0017535
1746563218	1951	1950	Stage to Tucson	Stage to Tucson	Murphy, Ralph	Ralph Murphy	tt0042995
1745357727	2002	2002	Showtime	Showtime	Dey, Tom	Tom Dey	tt0284490
1746453486	1941	1941	Robbers of the Range	Robbers of the Range	Killy, Edward	Edward Killy	tt0034119
1746583177	1935	1935	A Night at the Opera	A Night at the Opera	Wood, Sam	Sam Wood	tt0026778
1769970580	1968	1968	A Thousand Pleasures	A Thousand Pleasures	Marsh, Julian	Michael Findlay	tt0063690
1746554243	1965	1965	Young Fury	Young Fury	Nyby, Christian	Christian Nyby	tt0059932
1746504745	1917	1917	Paradise Garden	Paradise Garden	Balshofer, Fred J.	Fred J. Balshofer	tt0008407
1746578052	1937	1937	Portia on Trial	Portia on Trial	Nicholls, George	George Nichols Jr.	tt0029433
1746424594	1942	1942	On the Sunny Side	On the Sunny Side	Schuster, Harold	Harold D. Schuster	tt0035150
1746584266	1935	1935	Reckless Roads	Reckless Roads	Lynwood, Burt	Burt P. Lynwood	tt0026915
1746440080	1999	1999	The Underground Comedy Movie	The Underground Comedy Movie	Offer, Vince	Vince Offer	tt0201290
1746578033	1938	1938	Stablemates	Stablemates	Wood, Sam	Sam Wood	tt0030786
1746474842	1948	1947	For You I Die	For You I Die	Reinhardt, John	John Reinhardt	tt0039389
1746509310	1927	1927	The Opening Night	The Opening Night	Griffith, Edward H.	Edward H. Griffith	tt0018234
1746562142	1956	1956	Fury at Gunsight Pass	Fury at Gunsight Pass	Sears, Fred F.	Fred F. Sears	tt0049245
1746495900	1920	1920	Love	Love	Ruggles, Wesley	Wesley Ruggles	tt0196715
1746574951	1933	1933	Secrets	Secrets	Borzage, Frank	Frank Borzage	tt0024539
1746454252	1941	1941	Blossoms in the Dust	Blossoms in the Dust	LeRoy, Mervyn	Mervyn LeRoy	tt0033407
1746475953	1948	1948	Embraceable You	Embraceable You	Jacoves, Felix	Felix Jacoves	tt0040315
1746582720	1937	1937	Valley of Terror	Valley of Terror	Herman, Al	Albert Herman	tt0169357
1746573195	1932	1932	A Parisian Romance	A Parisian Romance	Franklin, Chester M.	Chester M. Franklin	tt0023319
1746574637	1937	1937	You Can't Have Everything	You Can't Have Everything	Taurog, Norman	Norman Taurog	tt0029806
1746409424	1984	1983	My Breakfast With Blassie	My Breakfast with Blassie	Legend, Johnny; Lautrec, Linda	Linda Lautrec	tt0085979
1746580275	1937	1937	It's All Yours	It's All Yours	Nugent, Elliott	Elliott Nugent	tt0029057
1746514645	1917	1917	Black Orchids	Black Orchids	Ingram, Rex	Rex Ingram	tt0006434
1746410396	1987	1987	Born in East L. A.	Born in East L.A.	Marin, Cheech	Cheech Marin	tt0092690
1746574552	1933	1933	The Sweetheart of Sigma Chi	The Sweetheart of Sigma Chi	Marin, Edwin L.	Edwin L. Marin	tt0024635

1746453530	1941	1941	Hard Guy	Hard Guy	Clifton, Elmer	Elmer Clifton	tt0033688	
1746423333	1997	1997	Free Willy 3: The Rescue	Free Willy 3: The Rescue	Pillsbury, Sam	Sam Pillsbury	tt0119152	
1745358703	2010	2010	Our Family Wedding	Our Family Wedding	Famuyiwa, Rick	Rick Famuyiwa	tt1305583	
1769977749	1981	1981	This Is Elvis	This Is Elvis	Leo, Malcolm; Solt, Andrew	Malcolm Leo	tt0083193	
1746577314	1951	1951	As You Were!	As You Were	Guiol, Fred L.	Bernard Girard	tt0043298	
1746434673	1999	1999	She's All That	She's All That	Iscove, Robert	Robert Iscove	tt0160862	
1745293999	2007	2007	The Great Debaters	The Great Debaters	Washington, Denzel	Denzel Washington	tt0427309	
1746582962	1936	1935	Defying the Law	Defying the Law	Horner, Robert J.	Robert J. Horner	tt0214622	
1746409949	1989	1989	Always	Always	Spielberg, Steven	Steven Spielberg	tt0096794	
1746504084	1921	1921	Across the Divide	Across the Divide	Holloway, John	John Holloway	tt0011905	
1746499963	1925	1925	The Live Wire	The Live Wire	Hines, Charles	Charles Hines	tt0016034	
1746583092	1936	1936	Human Cargo	Human Cargo	Dwan, Allan	Allan Dwan	tt0027769	
1746488042	1920	1920	Trumpet Island	Trumpet Island	Terriss, Tom	Tom Terriss	tt0011793	
1746562192	1953	1953	Easy to Love	Easy to Love	Walters, Charles	Charles Walters	tt0045718	
1746524926	1916	1916	A Child of the Paris Streets	A Child of the Paris Streets	Ingraham, Lloyd	Lloyd Ingraham	tt0006505	
1769970816	1912	1912	The Spanish Cavalier	The Spanish Cavalier		J. Searle Dawley	tt0794354	
1746438181	1942	1942	Sin Town	Sin Town	Enright, Ray	Ray Enright	tt0035337	
1769976136	1918	1918	Give Her Gas	Give Her Gas	Lyons, Eddie; Moran, Lee	Eddie Lyons	tt0332631	
1746532990	1914	1914	The Eagle's Mate	The Eagle's Mate	Kirkwood, James	James Kirkwood	tt0003871	
1746434368	1989	1989	Miss Firecracker	Miss Firecracker	Schlamme, Thomas	Thomas Schlamme	tt0097892	
1746582760	1936	1936	Vengeance of Rannah	Vengeance of Rannah	Samuels, Raymond; Shamray, Frankl	Bernard B. Ray	tt0168239	
1746216812	1967	1967	Blast-Off Girls	Blast Off Girls	Lewis, Herschell Gordon	Herschell Gordon Lewis	tt0061408	
1746475748	1945	1945	Hollywood and Vine	Hollywood and Vine	Thurn-Taxis, Alexis	Alexis Thurn	tt0037786	
1746394037	1993	1993	Beethoven's 2nd	Beethoven's 2nd	Daniel, Rod	Rod Daniel	tt0106375	
1746233134	1969	1969	What Ever Happened to Aunt Alice?	What Ever Happened to Aunt Alice?	Katzin, Lee H.	Lee H. Katzin	tt0065206	
1746464799	1944	1944	Nine Girls	Nine Girls	Jason, Leigh	Leigh Jason	tt0037131	
1746584774	1929	1929	The Oklahoma Kid	The Oklahoma Kid	McGowan, J. P.	J.P. McGowan	tt0020236	
**1746503827**	**1918**	**1918**	**Marriage**	**Marriage**	**Kirkwood, James**	**James Kirkwood**	**tt0009352**	1
1746420240	1993	1992	Wishman	Wishman	Marvin, Michael	Mike Marvin	tt0103273	
1746569777	1957	1957	Untamed Youth	Untamed Youth	Koch, Howard W.	Howard W. Koch	tt0051139	
1746491777	1947	1947	The Story of Mr. Hobbs	The Story of Mr. Hobbs	Alagia, Lorenzo	Arthur Varney	tt1282478	
1746512199	1923	1923	The Shriek of Araby	The Shriek of Araby	Jones, F. Richard	F. Richard Jones	tt0014467	
1746420232	1987	1987	Ernest Goes to Camp	Ernest Goes to Camp	Cherry, John	John R. Cherry III	tt0092974	
1746474537	1944	1944	Minstrel Man	Minstrel Man	Lewis, Joseph H.; Fox, Wallace	Joseph H. Lewis	tt0037076	
1746560313	1964	1964	Point of Order!	Point of Order!	de Antonio, Emile	Emile de Antonio	tt0058481	
1746234474	1968	1968	You Are What You Eat	You Are What You Eat	Feinstein, Barry	Barry Feinstein	tt0142005	
1746453848	1943	1943	Cinderella Swings It	Cinderella Swings It	Cabanne, Christy	Christy Cabanne	tt0035738	
1746453593	1940	1940	Tugboat Annie Sails Again	Tugboat Annie Sails Again	Seiler, Lewis	Lewis Seiler	tt0033187	
1746525166	1921	1921	Uncharted Seas	Uncharted Seas	Ruggles, Wesley	Wesley Ruggles	tt0012788	
1746583454	1930	1930	The Border Legion	The Border Legion	Brower, Otto; Knopf, Edwin H.	Otto Brower	tt0020700	
1746561597	1957	1957	Decision at Sundown	Decision at Sundown	Boetticher, Budd	Budd Boetticher	tt0050296	
1746491863	1944	1944	Rogues Gallery	Rogues' Gallery	Herman, Albert	Albert Herman	tt0038034	
1746512882	1916	1916	The Last Act	The Last Act	Edwards, Walter	Walter Edwards	tt0163047	
1746578480	1959	1959	The Last Angry Man	The Last Angry Man	Mann, Daniel	Daniel Mann	tt0052990	
1746424250	1941	1941	Accent on Love	Accent on Love	McCarey, Ray	Ray McCarey	tt0033311	
1745357916	2002	2002	Undercover Brother	Undercover Brother	Lee, Malcolm D.	Malcolm D. Lee	tt0279493	
1746580838	1938	1938	Newsboys' Home	Newsboys' Home	Young, Harold	Harold Young	tt0031720	
1746582203	1931	1931	Monsters of the Deep	Monsters of the Deep		Harold Austin	tt0151624	
1746577589	1937	1937	Melody of the Plains	Melody of the Plains	Newfield, Sam	Sam Newfield	tt0029230	
1746454805	1998	1998	Slappy & the Stinkers	Slappy and the Stinkers	Kellman, Barnet	Barnet Kellman	tt0120213	
1746574473	1933	1933	To the Last Man	To the Last Man	Hathaway, Henry	Henry Hathaway	tt0024674	
1746578433	1958	1958	She Demons	She Demons	Cunha, Richard E.	Richard E. Cunha	tt0052187	
1746465096	1946	1946	The Desert Horseman	The Desert Horseman	Nazarro, Ray	Ray Nazarro	tt0038467	
1746467363	1917	1917	The Food Gamblers	The Food Gamblers	Parker, Albert	Albert Parker	tt0007962	
1746435619	1942	1942	Juke Box Jenny	Juke Box Jenny	Young, Harold	Harold Young	tt0034927	
1746569640	1954	1954	The Adventures of Hajji Baba	The Adventures of Hajji Baba	Weis, Don	Don Weis	tt0046683	
1746577215	1963	1963	Heavenly Bodies	Heavenly Bodies!	Meyer, Russ	Russ Meyer	tt0057133	

1746492756	1948	1948	Julia Misbehaves	Julia Misbehaves	Conway, Jack	Jack Conway	tt0040498
1745358583	2008	2008	Strange Wilderness	Strange Wilderness	Wolf, Fred	Fred Wolf	tt0489282
1746581935	1910	1910	The Highlander's Defiance	The Highlander's Defiance			tt0352401
1746504001	1920	1920	Neptune's Bride	Neptune's Bride	Peacocke, Leslie T.	Leslie T. Peacocke	tt0196786
1746198758	1966	1966	Brighty of the Grand Canyon	Brighty of the Grand Canyon	Foster, Norman	Norman Foster	tt0061424
1746200298	1969	1969	Marooned	Marooned	Sturges, John	John Sturges	tt0064639
1746509170	1927	1927	The Land Beyond the Law	The Land Beyond the Law	Brown, Harry J.	Harry Joe Brown	tt0018076
1769970466	1967	1967	The Lusting Hours	Lusting Hours		John Amero	tt0242625
1746502202	1910	1910	The Two Brothers	The Two Brothers	Griffith, D. W.	D.W. Griffith	tt0001428
1746508981	1925	1925	The Handicap	The Handicap	O`Brien, John B.	John B. O'Brien	tt4481158
1746584547	1929	1929	Three Live Ghosts	Three Live Ghosts	Freeland, Thornton	Thornton Freeland	tt0020496
1746500992	1916	1916	The Law's Injustice	The Law's Injustice	Sloman, Edward	Edward Sloman	tt0489153
1746501045	1910	1910	Mr. Bumptious on Birds	Mr. Bumptious on Birds	Miller, Ashley	Ashley Miller	tt1499675
1746582838	1937	1937	When Love Is Young	When Love Is Young	Mohr, Hal	Hal Mohr	tt0029759
1746515787	1922	1922	Under Two Flags	Under Two Flags	Browning, Tod	Tod Browning	tt0013719
1746497480	1919	1919	Forbidden	Forbidden	Weber, Lois; Smalley, Phillips	Phillips Smalley	tt0010140
1746584690	1930	1930	Paradise Island	Paradise Island	Glennon, Bert	Bert Glennon	tt0021231
1746579037	1959	1959	Verboten!	Verboten!	Fuller, Samuel	Samuel Fuller	tt0052354
1746497673	1920	1920	Silk Husbands and Calico Wives	Silk Husbands and Calico Wives	Green, Alfred E.	Alfred E. Green	tt0011694
1746200297	1968	1968	Dayton's Devils	Dayton's Devils	Shea, Jack	Jack Shea	tt0062866
1746454044	1943	1943	Above Suspicion	Above Suspicion	Thorpe, Richard	Richard Thorpe	tt0035605
1746554316	1963	1963	The Cardinal	The Cardinal	Preminger, Otto	Otto Preminger	tt0056907
1746491968	1944	1944	Dragon Seed	Dragon Seed	Conway, Jack; Bucquet, Harold S.	Harold S. Bucquet	tt0036777
1746474127	1946	1946	Valley of the Zombies	Valley of the Zombies	Ford, Philip	Philip Ford	tt0039076
1746569571	1955	1955	Three Stripes in the Sun	Three Stripes in the Sun	Murphy, Richard	Richard Murphy	tt0049257
1746424390	1942	1942	Tish	Tish	Simon, S. Sylvan	S. Sylvan Simon	tt0035445
1769970627	1968	1968	Mid-Day Mistress	Mid Day Mistress	Emyl, Rolf	Rolf Emyl	tt0246781
1745293703	2007	2006	The TV Set	The TV Set	Kasdan, Jake	Jake Kasdan	tt0473709
1746500619	1924	1924	Yolanda	Yolanda	Vignola, Robert G.	Robert G. Vignola	tt0015518
1769970645	1968	1968	The Specialists	The Specialists	Moser, Clem	Nick Millard	tt0258199
1746520852	1925	1925	Beauty and the Bad Man	Beauty and the Bad Man	Worthington, William	William Worthington	tt0015609
1746393968	1987	1987	Steel Dawn	Steel Dawn	Hool, Lance	Lance Hool	tt0094033
1746577323	1951	1951	Missing Women	Missing Women	Ford, Philip	Philip Ford	tt0043810
1746211271	1973	1973	Super Fly T.N.T.	Super Fly T.N.T.	O'Neal, Ron	Ron O'Neal	tt0070753
1746524598	1916	1916	The Salamander	The Salamander	Donaldson, Arthur	Arthur Donaldson	tt0007281
1746585386	1939	1939	Remember?	Remember?	McLeod, Norman Z.	Norman Z. McLeod	tt0031845
1746572317	1932	1932	Week-end Marriage	Week End Marriage	Freeland, Thornton	Thornton Freeland	tt0023677
1746582314	1931	1931	Touchdown!	Touchdown!	McLeod, Norman	Norman Z. McLeod	tt0022493
1746454929	1946	1946	The Phantom Thief	The Phantom Thief	Lederman, D. Ross	D. Ross Lederman	tt0038839
1746574830	1934	1934	The Fighting Rookie	The Fighting Rookie	Bennet, Spencer Gordon	Spencer Gordon Bennet	tt0025112
1746583788	1931	1931	The Royal Bed	The Royal Bed	Sherman, Lowell	Lowell Sherman	tt0022326
1746421949	1992	1992	Glengarry Glen Ross	Glengarry Glen Ross	Foley, James	James Foley	tt0104348
1746504223	1917	1917	A Stormy Knight	A Stormy Knight	Clifton, Elmer	Elmer Clifton	tt0008633
1746499992	1924	1924	Birthright	Birthright	Micheaux, Oscar	Oscar Micheaux	tt0014717
1746421255	1996	1996	Girl 6	Girl 6	Lee, Spike	Spike Lee	tt0116414
1746566254	1954	1954	Untamed Heiress	Untamed Heiress	Lamont, Charles	Charles Lamont	tt0047635
1746577476	1962	1962	All Fall Down	All Fall Down	Frankenheimer, John	John Frankenheimer	tt0055738
1746454094	1942	1942	The Man Who Wouldn't Die	The Man Who Wouldn't Die	Leeds, Herbert I.	Herbert I. Leeds	tt0035027
1746555407	1956	1956	Wetbacks	Wetbacks	McCune, Hank	Hank McCune	tt0049946
1746483853	1917	1917	The Circus of Life	The Circus of Life	Julian, Rupert	Rupert Julian	tt0007798
1746455131	1996	1996	My Fellow Americans	My Fellow Americans	Segal, Peter	Peter Segal	tt0117119
1746584402	1940	1940	Maryland	Maryland	King, Henry	Henry King	tt0032766
1746232995	1968	1968	Counterpoint	Counterpoint	Nelson, Ralph	Ralph Nelson	tt0062829
1746504596	1922	1922	Sky High	Sky High	Reynolds, Lynn	Lynn Reynolds	tt0013607
1746209071	1966	1966	Gunpoint	Gunpoint	Bellamy, Earl	Earl Bellamy	tt0060483
1746576877	1932	1932	It's Tough to Be Famous	It's Tough to Be Famous	Green, Alfred E.	Alfred E. Green	tt0021919
1746257387	1976	1976	Assault on Precinct 13	Assault on Precinct 13	Carpenter, John	John Carpenter	tt0074156

1746583249	1929	1929	Frozen River	Frozen River	Weight, F. Harmon	F. Harmon Weight	tt0019908	
1746520042	1926	1926	Laddie	Laddie	Meehan, James Leo	James Leo Meehan	tt0017051	
1746514143	1916	1916	The Redemption of Dave Darcey	The Redemption of Dave Darcey	Scardon, Paul	Paul Scardon	tt0158891	
1746577796	1938	1938	Paradise for Three	Paradise for Three	Buzzell, Edward	Edward Buzzell	tt0030554	
1746583972	1936	1936	Black Gold	Black Gold	Hopton, Russell	Russell Hopton	tt0027366	
1746584372	1936	1936	The Singing Kid	The Singing Kid	Keighley, William	William Keighley	tt0028257	
1745309175	2005	2005	Beauty Shop	Beauty Shop	Woodruff, Bille	Bille Woodruff	tt0388500	
**1746475950**	**1948**	**1948**	**Raw Deal**	**Raw Deal**	**Mann, Anthony**	**Anthony Mann**	**tt0040723**	1
1745293637	2005	2005	Kiss Kiss, Bang Bang	Kiss Kiss Bang Bang	Black, Shane	Shane Black	tt0373469	
1746464832	1941	1941	Sunny	Sunny	Wilcox, Herbert	Herbert Wilcox	tt0034243	
1746508106	1925	1925	Galloping Vengeance	Galloping Vengeance	Craft, William James	William James Craft	tt0133788	
1746423463	1989	1989	Dead-Bang	Dead Bang	Frankenheimer, John	John Frankenheimer	tt0097166	
1746568207	1932	1932	Prosperity	Prosperity	Wood, Sam	Sam Wood	tt0023358	
1746582022	1928	1928	Riding for Fame	Riding for Fame	Eason, Reaves	B. Reeves Eason	tt0019324	
1746567955	1933	1933	Olsen's Big Moment	Olsen's Big Moment	St. Clair, Malcolm	Malcolm St. Clair	tt0024406	
1746514800	1910	1910	The Wonderful Wizard of Oz	The Wonderful Wizard of Oz	Turner, Otis	Otis Turner	tt0001463	
1746409694	1987	1987	Three O'Clock High	Three O'Clock High	Joanou, Phil	Phil Joanou	tt0094138	
1746555836	1956	1956	The Killing	The Killing	Kubrick, Stanley	Stanley Kubrick	tt0049406	
1746586167	1939	1939	Heritage of the Desert	Heritage of the Desert	Selander, Lesley	Lesley Selander	tt0031419	
1746410617	1988	1988	Killer Klowns from Outer Space	Killer Klowns from Outer Space	Chiodo, Stephen	Stephen Chiodo	tt0095444	
1746502837	1915	1915	The End of the Road	The End of the Road	Ricketts, Thomas	Tom Ricketts	tt0005244	
1746410550	1988	1988	I'm Gonna Git You Sucka	I'm Gonna Git You Sucka	Wayans, Keenen Ivory	Keenen Ivory Wayans	tt0095348	
1746497553	1920	1920	The White Rider	The White Rider	Craft, William J.	William James Craft	tt0203196	
1746583777	1930	1930	So This Is London	So This Is London	Blystone, John	John G. Blystone	tt0021395	
1746410696	1991	1991	The People Under the Stairs	The People Under the Stairs	Craven, Wes	Wes Craven	tt0105121	
1746520969	1949	1949	Riders of the Dusk	Riders of the Dusk	Hillyer, Lambert	Lambert Hillyer	tt0041808	
1746500709	1948	1948	Smoky Mountain Melody	Smoky Mountain Melody	Nazarro, Ray	Ray Nazarro	tt0041888	
1746582761	1936	1936	Dimples	Dimples	Seiter, William A.	William A. Seiter	tt0027527	
1746566042	1953	1953	From Here to Eternity	From Here to Eternity	Zinnemann, Fred	Fred Zinnemann	tt0045793	
1746219159	1980	1980	The Island	The Island	Ritchie, Michael	Michael Ritchie	tt0080934	
1745272652	2001	2001	Bully	Bully	Clark, Larry	Larry Clark	tt0242193	
1746508385	1927	1927	One Glorious Scrap	One Glorious Scrap	Lewis, Edgar	Edgar Lewis	tt0018225	
1746250019	1976	1976	Drum	Drum	Carver, Steve	Steve Carver	tt0074437	
1746520719	1926	1925	The Silent Guardian	The Silent Guardian	Bletcher, William	Billy Bletcher	tt0017387	
1746575027	1934	1934	Coming Out Party	Coming Out Party	Blystone, John	John G. Blystone	tt0024996	
1746520856	1948	1948	Will It Happen Again?	Will It Happen Again?		Dwain Esper	tt0204454	
1746473925	1947	1947	Raiders of the South	Raiders of the South	Hillyer, Lambert	Lambert Hillyer	tt0038868	
1746514911	1923	1923	Dundee-Criqui Boxing Exhibition	Dundee Criqui Boxing Exhibition			tt1032865	
1745309513	2005	2005	Kids in America	Kids in America	Stolberg, Josh	Josh Stolberg	tt0408961	
1746491985	1947	1947	Land of the Lawless	Land of the Lawless	Hillyer, Lambert	Lambert Hillyer	tt0039548	
1746524923	1923	1923	The Lone Wagon	The Lone Wagon	Mattison, Frank S.	Frank S. Mattison	tt0133933	
1746453781	1941	1940	San Francisco Docks	San Francisco Docks	Lubin, Arthur	Arthur Lubin	tt0034149	
1746438200	1996	1996	Black Sheep	Black Sheep	Spheeris, Penelope	Penelope Spheeris	tt0115697	
1745293654	2005	2005	Brokeback Mountain	Brokeback Mountain	Lee, Ang	Ang Lee	tt0388795	
1746578565	1963	1963	For Love or Money	For Love or Money	Gordon, Michael	Michael Gordon	tt0057067	
1745309119	2007	2007	Disturbia	Disturbia	Caruso, D. J.	D.J. Caruso	tt0486822	
1746520438	1949	1949	The Last Bandit	The Last Bandit	Kane, Joseph	Joseph Kane	tt0041578	
1746585235	1930	1930	Africa Speaks	Africa Speaks!		Walter Futter	tt0174450	
1746569503	1955	1955	Death Tide	Death Tide	Komow, Victor H.	Victor Komow	tt0301172	
1746582967	1935	1935	Les Misérables	Les Misérables	Boleslawski, Richard	Richard Boleslawski	tt0026725	
1746410705	1986	1986	Police Academy 3: Back in Training	Police Academy 3: Back in Training	Paris, Jerry	Jerry Paris	tt0091777	
1746566988	1933	1933	Hello, Everybody!	Hello, Everybody!	Seiter, William A.	William A. Seiter	tt0024103	
1769970681	1968	1968	See How They Come	See How They Come	Hennigar, William K.	William K. Hennigar	tt0257126	
1746184150	1910	1910	Big Elk's Turndown	Big Elk's Turndown		Kenean Buel	tt1930293	
1746467186	1917	1917	The Charmer	The Charmer	Conway, Jack	Jack Conway	tt0007784	
1746524975	1915	1915	Sold	Sold	Porter, Edwin S.; Ford, Hugh	Hugh Ford	tt0006064	
1746575060	1936	1936	Snowed Under	Snowed Under	Enright, Raymond	Ray Enright	tt0028272	

1746503435	1920	1920	Rouge and Riches	Rouge and Riches	Franklin, Harry L.	Harry L. Franklin	tt0011646
1745394109	2008	2008	Doomsday	Doomsday	Marshall, Neil	Neil Marshall	tt0483607
1746582037	1928	1928	Red Gold	Red Gold		Ben F. Wilson	tt0021290
1746514744	1922	1922	The Cowboy King	The Cowboy King	Seeling, Charles R.	Charles R. Seeling	tt0013029
1746524867	1916	1916	The Clarion	The Clarion	Durkin, James	James Durkin	tt0157471
1746422639	1993	1993	Money for Nothing	Money for Nothing	Menendez, Ramon	Ramón Menéndez	tt0107594
1746509292	1925	1925	Morals for Men	Morals for Men	Hyman, Bernard	Bernard H. Hyman	tt0016125
1746498265	1919	1919	The Hoodlum	The Hoodlum	Franklin, Sidney A.	Sidney Franklin	tt0010267
1746500009	1924	1924	The Sword of Valor	The Sword of Valor	Worne, Duke	Duke Worne	tt0015383
1746423490	1998	1998	The Replacement Killers	The Replacement Killers	Fuqua, Antoine	Antoine Fuqua	tt0120008
1746578942	1961	1961	Erotica	Erotica	Meyer, Russ	Russ Meyer	tt0054852
1746219011	1974	1974	A Woman Under the Influence	A Woman Under the Influence	Cassavetes, John	John Cassavetes	tt0072417
1746420881	1993	1993	CB4	CB4	Davis, Tamra	Tamra Davis	tt0106500
1746511764	1916	1916	The Path of Happiness	The Path of Happiness		Elaine S. Carrington	tt0007159
1746491714	1945	1945	Utah	Utah	English, John	John English	tt0038212
1746575015	1935	1935	Speed Limited	Speed Limited	Herman, Al	Albert Herman	tt0033087
1746438950	1943	1943	Sarong Girl	Sarong Girl	Dreifuss, Arthur	Arthur Dreifuss	tt0035289
1746504393	1918	1918	You Can't Believe Everything	You Can't Believe Everything	Conway, Jack	Jack Conway	tt0009843
1746475979	1948	1948	Moonrise	Moonrise	Borzage, Frank	Frank Borzage	tt0040607
1746453458	1943	1943	Dr. Gillespie's Criminal Case	Dr. Gillespie's Criminal Case	Goldbeck, Willis	Willis Goldbeck	tt0035826
1746561029	1932	1932	With Williamson Beneath the Sea	With Williamson Beneath the Sea	Williamson, J. E.	J.E. Williamson	tt1270705
1746520529	1950	1950	Call of the Klondike	Call of the Klondike	McDonald, Frank	Frank McDonald	tt0042300
1746394239	1987	1987	Cold Steel	Cold Steel	Puzo, Dorothy Ann	Dorothy Ann Puzo	tt0092767
1746581527	1928	1928	Show Girl	Show Girl	Santell, Alfred	Alfred Santell	tt0019378
1746582793	1938	1938	Arson Gang Busters	Arson Gang Busters	Kane, Joe	Joseph Kane	tt0029882
1746475730	1945	1945	Eadie Was a Lady	Eadie Was a Lady	Dreifuss, Arthur	Arthur Dreifuss	tt0037663
1746577874	1938	1937	Lady Behave!	Lady Behave!	Corrigan, Lloyd	Lloyd Corrigan	tt0029102
1745373547	2001	2001	The Fluffer	The Fluffer	Glatzer, Richard; West, Wash	Richard Glatzer	tt0245115
1745359034	2003	2003	Honey	Honey	Woodruff, Bille	Bille Woodruff	tt0322589
1746583953	1930	1930	Song of the Caballero	Song of the Caballero	Brown, Harry J.	Harry Joe Brown	tt0021403
1745359553	2001	2001	Monster's Ball	Monster's Ball	Forster, Marc	Marc Forster	tt0285742
1746474134	1948	1948	The Dude Goes West	The Dude Goes West	Neumann, Kurt	Kurt Neumann	tt0040305
1769975762	1918	1918	In and Out	In and Out	Seiter, William A.	William A. Seiter	tt1312977
1746573685	1931	1931	Husband's Holiday	Husband's Holiday	Milton, Robert	Robert Milton	tt0023039
1746584357	1939	1939	The Lady's from Kentucky	The Lady's from Kentucky	Hall, Alexander	Alexander Hall	tt0031550
1745340442	2001	2001	The Curse of the Jade Scorpion	The Curse of the Jade Scorpion	Allen, Woody	Woody Allen	tt0256524
1746584605	1931	1931	Mystery Train	The Mystery Train	Whitman, Phil	Phil Whitman	tt0022181
1746555813	1954	1954	Shield for Murder	Shield for Murder	O'Brien, Edmond; Koch, Howard W.	Howard W. Koch	tt0047479
1746464863	1942	1942	Bullets for Bandits	Bullets for Bandits	Fox, Wallace W.	Wallace Fox	tt0034557
1746520760	1925	1925	Old Clothes	Old Clothes	Cline, Edward	Edward F. Cline	tt0016174
1746585088	1930	1930	Monte Carlo	Monte Carlo	Lubitsch, Ernst	Ernst Lubitsch	tt0021153
1746585402	1940	1940	Hired Wife	Hired Wife	Seiter, William A.	William A. Seiter	tt0032596
1746582000	1928	1928	The Boss of Rustler's Roost	The Boss of Rustler's Roost	Maloney, Leo	Leo D. Maloney	tt0018716
1746508887	1925	1925	Lady Robinhood	Lady Robinhood	Ince, Ralph	Ralph Ince	tt0016002
1746423052	1994	1994	Color of Night	Color of Night	Rush, Richard	Richard Rush	tt0109456
1746454833	1944	1944	Bowery to Broadway	Bowery to Broadway	Lamont, Charles	Charles Lamont	tt0036668
1746584130	1934	1934	The Countess of Monte Cristo	The Countess of Monte Cristo	Freund, Karl	Karl Freund	tt0025005
1746565969	1957	1957	Duel at Apache Wells	Duel at Apache Wells	Kane, Joe	Joseph Kane	tt0050343
1746583458	1931	1931	Mounted Fury	Mounted Fury	Paton, Stuart	Stuart Paton	tt0022166
1746573620	1931	1931	Just a Gigolo	Just a Gigolo	Conway, Jack	Jack Conway	tt0021780
1746393956	1985	1985	Lust in the Dust	Lust in the Dust	Bartel, Paul	Paul Bartel	tt0089523
1746500406	1925	1925	The Desert Demon	The Desert Demon	Thorpe, Richard	Richard Thorpe	tt0015744
1746467839	1917	1917	The Boy Girl	The Boy Girl	Stevens, Edwin	Edwin Stevens	tt0007743
1746454039	1941	1941	Pacific Blackout	Pacific Blackout	Murphy, Ralph	Ralph Murphy	tt0033993
1746454464	1943	1943	Campus Rhythm	Campus Rhythm	Dreifuss, Arthur	Arthur Dreifuss	tt0035710
1746583380	1930	1930	The Golf Specialist	The Golf Specialist	Brice, Monte	Monte Brice	tt0020927
1746424248	1941	1941	Gauchos of Eldorado	Gauchos of El Dorado	Orlebeck, Les	Lester Orlebeck	tt0033649

1745357896	2008	2008	Sex Drive	Sex Drive	Anders, Sean	Sean Anders	tt1135985
1746584684	1928	1928	The Barker	The Barker	Fitzmaurice, George	George Fitzmaurice	tt0018674
1746567520	1932	1932	The Night of June 13	The Night of June 13	Roberts, Stephen	Stephen Roberts	tt0023272
1746571602	1932	1932	Hell-Fire Austin	Hell Fire Austin	Sheldon, Forrest	Forrest Sheldon	tt0022988
1746466951	1917	1916	The Final Payment	The Final Payment	Powell, Frank	Wilbert Melville	tt0921809
1746568020	1933	1933	Tomorrow at Seven	Tomorrow at Seven	Enright, Ray	Ray Enright	tt0024679
1746503415	1918	1918	Her Boy	Her Boy	Irving, George	George Irving	tt0009160
1746582174	1928	1928	The Water Hole	The Water Hole	Jones, F. Richard	F. Richard Jones	tt0019550
1746585107	1929	1929	Chasing Through Europe	Chasing Through Europe	Butler, David; Werker, Alfred L.	David Butler	tt0019759
1746567619	1932	1932	Wild Girl	Wild Girl	Walsh, Raoul	Raoul Walsh	tt0023700
1746208605	1969	1969	Goodbye, Mr. Chips	Goodbye, Mr. Chips	Ross, Herbert	Herbert Ross	tt0064382
1745293941	2006	2006	10th & Wolf	10th & Wolf	Moresco, Bobby	Bobby Moresco	tt0360323
1746492551	1948	1948	Luxury Liner	Luxury Liner	Whorf, Richard	Richard Whorf	tt0040556
1746585353	1929	1929	The Trial of Mary Dugan	The Trial of Mary Dugan	Veiller, Bayard	Bayard Veiller	tt0020516
1746580476	1935	1935	The Arizonian	The Arizonian	Vidor, Charles	Charles Vidor	tt0026082
1746235607	1974	1974	Claudine	Claudine	Berry, John	John Berry	tt0071334
1746392764	1982	1982	Some Kind of Hero	Some Kind of Hero	Pressman, Michael	Michael Pressman	tt0083107
1746581218	1951	1951	Fixed Bayonets!	Fixed Bayonets!	Fuller, Samuel	Samuel Fuller	tt0043540
1746423458	1997	1997	In & Out	In & Out	Oz, Frank	Frank Oz	tt0119360
1746520773	1926	1926	The Nervous Wreck	The Nervous Wreck	Sidney, Scott	Scott Sidney	tt0017201
1746438291	1943	1943	The Ghost Rider	The Ghost Rider	Fox, Wallace W.	Wallace Fox	tt0035936
1746234764	1966	1966	Incident at Phantom Hill	Incident at Phantom Hill	Bellamy, Earl	Earl Bellamy	tt0060537
1746555466	1957	1956	On the Bowery	On the Bowery	Rogosin, Lionel	Lionel Rogosin	tt0050800
1746438324	1943	1943	Silver Spurs	Silver Spurs	Kane, Joseph	Joseph Kane	tt0036357
1746509242	1926	1926	The Call of the Klondike	The Call of the Klondike	Apfel, Oscar	Oscar Apfel	tt0016701
1746579019	1960	1960	The Mountain Road	The Mountain Road	Mann, Daniel	Daniel Mann	tt0054095
1746584855	1929	1929	One Stolen Night	One Stolen Night	Dunlap, Scott R.	Scott R. Dunlap	tt0020243
1746525146	1920	1920	The Cyclone	The Cyclone	Smith, Cliff	Clifford Smith	tt0011086
1746570395	1952	1952	The Girl in White	The Girl in White	Sturges, John	John Sturges	tt0044663
1746439724	1999	1999	Lost & Found	Lost & Found	Pollack, Jeff	Jeff Pollack	tt0120836
1746563485	1952	1952	Shadow in the Sky	Shadow in the Sky	Wilcox, Fred M.	Fred M. Wilcox	tt0044027
1746500095	1924	1924	Black Gold	Black Gold	Sheldon, Forrest	Forrest Sheldon	tt0014719
1746585480	1940	1940	Blondie on a Budget	Blondie on a Budget	Strayer, Frank R.	Frank R. Strayer	tt0032263
1746394669	1985	1985	Death Wish 3	Death Wish 3	Winner, Michael	Michael Winner	tt0089003
1746561019	1932	1932	Million Dollar Legs	Million Dollar Legs	Cline, Edward	Edward F. Cline	tt0023225
1746584893	1940	1940	Rainbow over the Range	Rainbow Over the Range	Herman, Al	Albert Herman	tt0032966
1746532717	1916	1916	Lovely Mary	Lovely Mary	Jones, Edgar	Edgar Jones	tt0006958
1746210513	1972	1972	"Everything You Always Wanted to Know About	Everything You Always Wanted to Know Abo	Allen, Woody	Woody Allen	tt0068555
1746562061	1954	1954	Rose Marie	Rose Marie	LeRoy, Mervyn	Mervyn LeRoy	tt0047429
1746433889	1995	1995	Top Dog	Top Dog	Norris, Aaron	Aaron Norris	tt0114697
1746532761	1923	1923	Souls in Bondage	Souls in Bondage	Clifford, William H.	William H. Clifford	tt0165952
1746581640	1928	1928	United States Smith	United States Smith	Henabery, Joseph	Joseph Henabery	tt0019512
1746409512	1990	1990	Bird on a Wire	Bird on a Wire	Badham, John	John Badham	tt0099141
1746584961	1940	1940	East Side Kids	East Side Kids	Hill, Robert	Robert F. Hill	tt0032428
1746574790	1937	1937	She's No Lady	She's No Lady	Vidor, Charles	Charles Vidor	tt0029552
1746525052	1920	1920	Roman Candles	Roman Candles	Pratt, Jack	Jack Pratt	tt0013793
1746574506	1935	1935	The New Frontier	The New Frontier	Pierson, Carl L.	Carl Pierson	tt0026774
1746523057	1916	1916	Behind the Lines	Behind the Lines	McRae, Henry	Henry MacRae	tt0006413
1746465191	1947	1947	The Fighting Vigilantes	The Fighting Vigilantes	Taylor, Ray	Ray Taylor	tt0039380
1746497940	1921	1921	Footlights	Footlights	Robertson, John S.	John S. Robertson	tt0012185
1745359938	2002	2002	Big Fat Liar	Big Fat Liar	Levy, Shawn	Shawn Levy	tt0265298
1746582978	1938	1938	Adventure in Sahara	Adventure in Sahara	Lederman, D. Ross	D. Ross Lederman	tt0029841
1746454042	1943	1943	Clancy Street Boys	Clancy Street Boys	Beaudine, William	William Beaudine	tt0035741
1746570131	1934	1934	Belle of the Nineties	Belle of the Nineties	McCarey, Leo	Leo McCarey	tt0024873
1746503654	1919	1919	The Law of Nature	The Law of Nature	Fischer, David G.	David G. Fischer	tt0190523
1746569983	1956	1956	Gun Brothers	Gun Brothers	Salkow, Sidney	Sidney Salkow	tt0049283
1746574675	1937	1938	Assassin of Youth	Assassin of Youth	Clifton, Elmer	Elmer Clifton	tt0028589

1746499858	1947	1947	Ride the Pink Horse	Ride the Pink Horse	Montgomery, Robert	Robert Montgomery	tt0039768
1746571592	1932	1932	Harlem Is Heaven	Harlem Is Heaven	Franklyn, Irwin R.	Irwin Franklyn	tt0022976
1746523043	1915	1915	Chimmie Fadden Out West	Chimmie Fadden Out West	DeMille, Cecil B.	Cecil B. DeMille	tt0005087
1746453495	1943	1943	Keep 'Em Slugging	Keep 'Em Slugging	Cabanne, Christy	Christy Cabanne	tt0036069
1746438938	1943	1943	Fired Wife	Fired Wife	Lamont, Charles	Charles Lamont	tt0035880
1746524811	1916	1916	The Wheel of the Law	The Wheel of the Law	Baker, George D.	George D. Baker	tt0159123
1746502657	1915	1915	The Bulldogs of the Trail	The Bulldogs of the Trail	MacDougall, Kenneth	Kenneth MacDougall	tt0005035
1746210199	1971	1971	The Brotherhood of Satan	The Brotherhood of Satan	McEveety, Bernard	Bernard McEveety	tt0066863
1746465082	1947	1947	Northwest Outpost	Northwest Outpost	Dwan, Allan	Allan Dwan	tt0039667
1746454850	1941	1941	One Foot in Heaven	One Foot in Heaven	Rapper, Irving	Irving Rapper	tt0033980
1746508144	1925	1925	Ermine and Rhinestones	Ermine and Rhinestones	King, Burton	Burton L. King	tt0015782
1746545285	1940	1940	Riders from Nowhere	Riders from Nowhere	Johnson, Raymond K.	Bernard B. Ray	tt0032988
1746508924	1926	1926	The Fighting Marine	The Fighting Marine	Bennett, Spencer Gordon	Spencer Gordon Bennet	tt0016864
1746420251	1989	1989	When Harry Met Sally...	When Harry Met Sally...	Reiner, Rob	Rob Reiner	tt0098635
1746409978	1989	1988	Miracle Mile	Miracle Mile	De Jarnatt, Steve	Steve De Jarnatt	tt0097889
1746585356	1940	1940	Flight Command	Flight Command	Borzage, Frank	Frank Borzage	tt0032477
1746525091	1919	1919	Beckoning Roads	Beckoning Roads	Hickman, Howard	Howard C. Hickman	tt0186861
1746424222	1943	1943	The Amazing Mrs. Holliday	The Amazing Mrs. Holliday	Manning, Bruce; Renoir, Jean	Bruce Manning	tt0035631
1746561262	1932	1932	The Devil Is Driving	The Devil Is Driving	Stoloff, Ben	Benjamin Stoloff	tt0022815
1746492454	1946	1946	Strange Triangle	Strange Triangle	McCarey, Ray	Ray McCarey	tt0038989
1746583539	1951	1951	The Hoodlum	The Hoodlum	Nosseck, Max	Max Nosseck	tt0043655
1746572413	1932	1932	Chandu the Magician	Chandu the Magician	Varnel, Marcel; Menzies, William C.	William Cameron Menzies	tt0022753
1746570140	1934	1934	Smarty	Smarty	Florey, Robert	Robert Florey	tt0025803
1746559938	1964	1964	Kiss Me, Stupid	Kiss Me, Stupid	Wilder, Billy	Billy Wilder	tt0058265
1746577463	1959	1959	Riot in Juvenile Prison	Riot in Juvenile Prison	Cahn, Edward L.	Edward L. Cahn	tt0053222
1746453492	1945	1945	Gaslight Follies	Gaslight Follies		Albert Herman	tt0037732
1746569921	1957	1957	Calypso Joe	Calypso Joe	Dein, Edward	Edward Dein	tt0050223
1746219338	1972	1972	Beware! the Blob	Beware! The Blob	Hagman, Larry	Larry Hagman	tt0068271
1746491718	1948	1948	River Lady	River Lady	Sherman, George	George Sherman	tt0040738
1746532611	1923	1923	The Sunshine Trail	The Sunshine Trail	Horne, James W.	James W. Horne	tt0013647
1746577295	1962	1962	Carnival of Souls	Carnival of Souls	Harvey, Herk	Herk Harvey	tt0055830
1746583737	1931	1931	I Like Your Nerve	I Like Your Nerve	McGann, William	William C. McGann	tt0021986
1746487346	1917	1917	To-Day	To Day	Ince, Ralph W.	Ralph Ince	tt0183901
1746583041	1936	1936	Adventure in Manhattan	Adventure in Manhattan	Ludwig, Edward	Edward Ludwig	tt0027258
1746497069	1919	1919	Behind the Door	Behind the Door	Willat, Irvin V.	Irvin Willat	tt0009915
1746583348	1931	1931	The Hard Hombre	The Hard Hombre	Brower, Otto	Otto Brower	tt0021940
1746435180	1943	1943	Murder on the Waterfront	Murder on the Waterfront	Eason, B. Reeves	B. Reeves Eason	tt0036181
1746500205	1924	1924	Barbara Frietchie	Barbara Frietchie	Hillyer, Lambert	Lambert Hillyer	tt0014696
1746497231	1917	1916	The Dummy	The Rummy	Grandon, Francis J.	Paul Powell	tt0007277
1746464814	1941	1941	Billy the Kid in Santa Fe	Billy the Kid in Santa Fe	Scott, Sherman	Sam Newfield	tt0033392
1746563217	1952	1952	Yukon Gold	Yukon Gold	McDonald, Frank	Frank McDonald	tt0122324
1746576310	1931	1931	Quick Trigger Lee	Quick Trigger Lee	McGowan, J. P.	J.P. McGowan	tt0022291
1746565998	1956	1956	Singing in the Dark	Singing in the Dark	Nosseck, Max	Max Nosseck	tt0049760
1746573989	1959	1959	Green Mansions	Green Mansions	Ferrer, Mel	Mel Ferrer	tt0052864
1746524798	1910	1910	The Romance of Circle Ranch	The Romance of Circle Ranch		William F. Haddock	tt2239178
1746566310	1953	1953	Vicki	Vicki	Horner, Harry	Harry Horner	tt0046515
1746532812	1923	1923	Souls for Sale	Souls for Sale	Hughes, Rupert	Rupert Hughes	tt0014497
1746503858	1917	1917	The Promise	The Promise	Balshofer, Fred J.	Jay Hunt	tt0008468
1746455802	1998	1998	Caught Up	Caught Up	Scott, Darin	Darin Scott	tt0119988
1745374980	2009	2009	Race to Witch Mountain	Race to Witch Mountain	Fickman, Andy	Andy Fickman	tt1075417
1746578880	1951	1951	My Outlaw Brother	My Outlaw Brother	Nugent, Elliott	Elliott Nugent	tt0043829
1746532813	1927	1927	Cross Breed	Cross Breed	Smith, Noel Mason	Noel M. Smith	tt0017782
1745393671	2010	2010	Beneath the Dark	Beneath the Dark	Feehan, Chad	Chad Feehan	tt1422800
1746514113	1915	1915	The Pitfall	The Pitfall	Horne, James W.	James W. Horne	tt0005900
1745357708	2002	2002	The Time Machine	The Time Machine	Wells, Simon	Simon Wells	tt0268695
1746502935	1923	1923	Broadway Broke	Broadway Broke	Dawley, J. Searle	J. Searle Dawley	tt0013894
1746500443	1949	1950	Riders of the Range	Riders of the Range	Selander, Lesley	Lesley Selander	tt0042892

1746497571	1917	1917	A Wife on Trial	A Wife on Trial	Baldwin, Ruth Ann	Ruth Ann Baldwin	tt0008773
1746500598	1948	1948	Strike It Rich	Strike It Rich	Selander, Leslie	Lesley Selander	tt0040845
1746566004	1957	1957	Sweet Smell of Success	Sweet Smell of Success	Mackendrick, Alexander	Alexander Mackendrick	tt0051036
1746422518	1996	1996	Dear God	Dear God	Marshall, Garry	Garry Marshall	tt0116059
1746438296	1944	1944	Law of the Valley	Law of the Valley	Bretherton, Howard	Howard Bretherton	tt0037011
1746580822	1936	1936	Gun Grit	Gun Grit		William Berke	tt0027706
1746584892	1939	1939	Within the Law	Within the Law	Machaty, Gustav	Gustav Machatý	tt0032135
1746567615	1932	1932	It Happened in Paris	It Happened in Paris	Weisfeldt, M. J.	M.J. Weisfeldt	tt0945569
1746582899	1938	1938	Numbered Woman	Numbered Woman	Brown, Karl	Karl Brown	tt0030513
1746572531	1910	1910	A Flirty Affliction	A Flirty Affliction		Gilbert M. 'Broncho Billy' Anderson	tt0372284
1746582886	1936	1936	The Glory Trail	The Glory Trail	Shores, Lynn	Lynn Shores	tt0027682
1746474143	1946	1946	Swamp Fire	Swamp Fire	Pine, William H.	William H. Pine	tt0039001
1746567317	1952	1952	Outlaw Women	Outlaw Women	Newfield, Sam; Ormond, Ron	Sam Newfield	tt0045004
1746492079	1944	1944	Dead or Alive	Dead or Alive	Clifton, Elmer	Elmer Clifton	tt0036750
1746520964	1949	1949	Make Believe Ballroom	Make Believe Ballroom	Santley, Joseph	Joseph Santley	tt0041620
1746200243	1966	1966	Maya	Maya	Berry, John	John Berry	tt0060681
1746509558	1926	1926	Puppets	Puppets	Archainbaud, George	George Archainbaud	tt0017297
1746496993	1918	1918	The Light Within	The Light Within	Trimble, Larry	Laurence Trimble	tt0009300
1746580689	1938	1938	Rio Grande	Rio Grande	Nelson, Sam	Sam Nelson	tt0031864
1746508037	1926	1926	Paths of Flame	Paths of Flame			tt0147155
1746523994	1910	1910	A New Divorce Cure	A New Divorce Cure			tt0352633
1746577919	1937	1937	The Life of the Party	The Life of the Party	Seiter, William A.	William A. Seiter	tt0029145
1746504413	1919	1919	The Broken Melody	The Broken Melody	Earle, William P. S.	William P.S. Earle	tt0187759
1746465012	1944	1944	Rainbow Island	Rainbow Island	Murphy, Ralph; Tuttle, Frank	Ralph Murphy	tt0037208
1746410452	1983	1983	Brainstorm	Brainstorm	Trumbull, Douglas	Douglas Trumbull	tt0085271
1746574167	1963	1963	A New Kind of Love	A New Kind of Love	Shavelson, Melville	Melville Shavelson	tt0057360
1746554073	1963	1963	The Ceremony	The Ceremony	Harvey, Laurence	Laurence Harvey	tt0056918
1746217856	1967	1967	Hot Rods to Hell	Hot Rods to Hell	Brahm, John	John Brahm	tt0061784
1746555033	1955	1955	Double Jeopardy	Double Jeopardy	Springsteen, R. G.	R.G. Springsteen	tt0046927
1746218242	1967	1967	A Covenant With Death	A Covenant with Death	Johnson, Lamont	Lamont Johnson	tt0061525
1746583275	1951	1951	Insurance Investigator	Insurance Investigator	Blair, George	George Blair	tt0128271
1746381966	1981	1981	Blow Out	Blow Out	De Palma, Brian	Brian De Palma	tt0082085
1746516274	1919	1919	The She Wolf	The She Wolf	Smith, Cliff	Clifford Smith	tt0192597
1746576898	1932	1932	Letty Lynton	Letty Lynton	Brown, Clarence	Clarence Brown	tt0023132
1746520844	1925	1925	Soul-Fire	Soul Fire	Robertson, John S.	John S. Robertson	tt0016377
1746487140	1920	1920	What Women Want	What Women Want	Archainbaud, George	George Archainbaud	tt0011849
1746583474	1931	1930	Viennese Nights	Viennese Nights	Crosland, Alan	Alan Crosland	tt0021522
1746249059	1973	1973	Running with the Devil	Running with the Devil	Sullivan, Ron	Henri Pachard	tt0314595
1746569576	1958	1958	Juvenile Jungle	Juvenile Jungle	Witney, William	William Witney	tt0051805
1746580625	1938	1938	The Jury's Secret	The Jury's Secret	Sloman, Edward	Edward Sloman	tt0030301
1746524136	1923	1923	Why Women Remarry	Why Women Remarry	Gorman, John	John Gorman	tt0014610
1746492713	1948	1948	Michael O'Halloran	Michael O'Halloran	Rawlins, John	John Rawlins	tt0040584
1746520286	1924	1924	Baffled	Baffled	McGowan, J. P.	J.P. McGowan	tt0014693
1746566329	1958	1958	Ghost of the China Sea	Ghost of the China Sea	Sears, Fred F.	Fred F. Sears	tt0051653
1746571569	1931	1931	The Hot Heiress	The Hot Heiress	Badger, Clarence	Clarence G. Badger	tt0021973
1746582868	1937	1937	A Star Is Born	A Star Is Born	Wellman, William A.	William A. Wellman	tt0029606
1746514375	1915	1915	Up from the Depths	Up from the Depths	Powell, Paul	Paul Powell	tt0146334
1746570606	1933	1933	Sailor Be Good!	Sailor Be Good	Cruze, James	James Cruze	tt0024517
1746186197	1967	1967	Who's Minding the Mint?	Who's Minding the Mint?	Morris, Howard	Howard Morris	tt0062490
1746473667	1942	1942	Land of the Open Range	Land of the Open Range	Killy, Edward	Edward Killy	tt0033810
1746574761	1934	1934	The Prescott Kid	The Prescott Kid	Selman, David	David Selman	tt0028134
1746560029	1964	1964	The Naked Kiss	The Naked Kiss	Fuller, Samuel	Samuel Fuller	tt0058390
1746500582	1925	1925	Love on the Rio Grande	Love on the Rio Grande	Craft, William J.	William James Craft	tt0016046
1746567357	1932	1932	The Strange Love of Molly Louvain	The Strange Love of Molly Louvain	Curtiz, Michael	Michael Curtiz	tt0023525
1746582205	1930	1930	Dumbbells in Ermine	Dumbbells in Ermine	Adolfi, John G.	John G. Adolfi	tt0020848
1746435223	1943	1943	Canyon City	Canyon City	Bennet, Spencer	Spencer Gordon Bennet	tt0035711
1746582971	1938	1938	Sharpshooters	Sharpshooters	Tinling, James	James Tinling	tt0030738

1746514516	1916	1916	The Wasted Years	The Wasted Years	Broadwell, Robert B.	Robert Broadwell	tt0159114
1769975280	1917	1917	In Treason's Grasp	In Treason's Grasp		Francis Ford	tt0312806
1746582494	1939	1939	Blackmail	Blackmail	Potter, H. C.	H.C. Potter	tt0031102
1746582862	1935	1935	Every Night at Eight	Every Night at Eight	Walsh, Raoul	Raoul Walsh	tt0026325
1745358053	2010	2010	You Again	You Again	Fickman, Andy	Andy Fickman	tt1414382
1746508908	1925	1925	Unrestrained Youth	Unrestrained Youth	Levering, Joseph	Joseph Levering	tt0175283
1746582684	1938	1938	Cipher Bureau	Cipher Bureau	Lamont, Charles	Charles Lamont	tt0029994
1746570716	1934	1934	Murder at the Vanities	Murder at the Vanities	Leisen, Mitchell	Mitchell Leisen	tt0025529
1746520763	1948	1948	Jungle Jim	Jungle Jim	Berke, William	William Berke	tt0040501
1746553904	1964	1964	The Horror of Party Beach	The Horror of Party Beach	Tenney, Del	Del Tenney	tt0058208
1745302765	2005	2005	Going Shopping	Going Shopping	Jaglom, Henry	Henry Jaglom	tt0295605
1746582712	1935	1935	Rendezvous at Midnight	Rendezvous at Midnight	Cabanne, Christy	Christy Cabanne	tt0026923
1746503255	1917	1917	The Man Without a Country	The Man Without a Country	Warde, Ernest C.	Ernest C. Warde	tt0008259
1746524994	1923	1923	The Unknown Purple	The Unknown Purple	West, Roland	Roland West	tt0014569
1746567250	1933	1933	Private Jones	Private Jones	Mack, Russell	Russell Mack	tt0024472
1746454024	1943	1943	Buckskin Frontier	Buckskin Frontier	Selander, Lesley	Lesley Selander	tt0035700
1746509011	1925	1925	The Splendid Road	The Splendid Road		Frank Lloyd	tt0016386
2007986195	2004	2004	Bridget Jones: The Edge of Reason	Bridget Jones: The Edge of Reason	Kidron, Beeban	Beeban Kidron	tt0317198
1746581234	1960	1960	The Story of Ruth	The Story of Ruth	Koster, Henry	Henry Koster	tt0054343
1746409294	1991	1991	One Good Cop	One Good Cop	Gould, Heywood	Heywood Gould	tt0102593
1746217055	1966	1966	Arabesque	Arabesque	Donen, Stanley	Stanley Donen	tt0060121
1746420936	1940	1940	West of Carson City	West of Carson City	Taylor, Ray	Ray Taylor	tt0033250
1746583303	1931	1931	Leftover Ladies	Left Over Ladies	Kenton, Erle C.	Erle C. Kenton	tt0022066
1746524296	1915	1915	The Curious Conduct of Judge Legarde	The Curious Conduct of Judge Legarde	Davis, Will	Will S. Davis	tt0005167
1746437117	2000	2000	Love and Basketball	Love & Basketball	Prince-Bythewood, Gina	Gina Prince	tt0199725
1746211082	1973	1973	Dillinger	Dillinger	Milius, John	John Milius	tt0069976
1746497665	1919	1919	Red Hot Dollars	Red Hot Dollars	Storm, Jerome	Jerome Storm	tt0010615
1746474104	1946	1946	Terror by Night	Terror by Night	Neill, Roy William	Roy William Neill	tt0039017
1746419367	1990	1989	Blind Fury	Blind Fury	Noyce, Phillip	Phillip Noyce	tt0096945
1746435886	2000	2000	Center Stage	Center Stage	Hytner, Nicholas	Nicholas Hytner	tt0210616
1746530686	1949	1949	Anna Lucasta	Anna Lucasta	Rapper, Irving	Irving Rapper	tt0041125
1745309124	2005	2005	Be Cool	Be Cool	Gray, F. Gary	F. Gary Gray	tt0377471
1746501323	1915	1915	The Woman Next Door	The Woman Next Door	Edwin, Walter	Walter Edwin	tt0006302
1746508455	1949	1949	The Crooked Way	The Crooked Way	Florey, Robert	Robert Florey	tt0041269
1745342930	2003	2002	Cabin Fever	Cabin Fever	Roth, Eli	Eli Roth	tt0303816
1745359355	2008	2008	War, Inc.	War, Inc.	Seftel, Joshua	Joshua Seftel	tt0884224
1746574803	1933	1933	Take a Chance	Take a Chance	Brice, Monte; Schwab, Laurence	Monte Brice	tt0024640
1746486137	1919	1919	Love, Honor and -Xxx ?	Love, Honor and ?	Miller, Charles	Charles Miller	tt0191259
1746585203	1929	1929	A Single Man	A Single Man	Beaumont, Harry	Harry Beaumont	tt0020409
1746566750	1933	1933	Cradle Song	Cradle Song	Leisen, Mitchell	Mitchell Leisen	tt0023913
1746584697	1930	1930	Abraham Lincoln	Abraham Lincoln	Griffith, D. W.	D.W. Griffith	tt0020620
1745359810	2001	2000	The Independent	The Independent	Kessler, Stephen	Stephen Kessler	tt0160403
1746492555	1948	1948	My Dear Secretary	My Dear Secretary	Martin, Charles	Charles Martin	tt0040626
1746574794	1936	1936	Brides Are Like That	Brides Are Like That	McGann, William	William C. McGann	tt0027396
1746567124	1932	1932	War Correspondent	War Correspondent	Sloane, Paul	Paul Sloane	tt0023670
1746473938	1948	1948	So This Is New York	So This Is New York	Fleischer, Richard O.	Richard Fleischer	tt0040810
1746514852	1910	1910	Up a Tree	Up a Tree	Powell, Frank	Frank Powell	tt0001438
1746234523	1967	1967	Sweet Love, Bitter	Sweet Love, Bitter	Danska, Herbert	Herbert Danska	tt0062325
1746574045	1959	1959	The Horse Soldiers	The Horse Soldiers	Ford, John	John Ford	tt0052902
1745374329	2003	2002	Poolhall Junkies	Poolhall Junkies	Callahan, Mars	Mars Callahan	tt0273982
1746509031	1924	1924	Ragged Robin	Ragged Robin	Mattison, Frank S.	Frank S. Mattison	tt0015257
1746580349	1938	1938	Of Human Hearts	Of Human Hearts	Brown, Clarence	Clarence Brown	tt0030517
1745395609	2010	2010	Morning Glory	Morning Glory	Michell, Roger	Roger Michell	tt1126618
1746580707	1935	1935	Redheads on Parade	Redheads on Parade	McLeod, Norman	Norman Z. McLeod	tt0026920
1746584252	1940	1940	Stranger on the Third Floor	Stranger on the Third Floor	Ingster, Boris	Boris Ingster	tt0033107
1746474531	1946	1946	Anna and the King of Siam	Anna and the King of Siam	Cromwell, John	John Cromwell	tt0038303
1746497614	1919	1919	In Old Kentucky	In Old Kentucky	Neilan, Marshall	Marshall Neilan	tt0010291

1746153058	1910	1910	An Affair of Hearts	An Affair of Hearts	Griffith, D. W.	Frank Powell	tt0001103
1746186231	1967	1967	Caprice	Caprice	Tashlin, Frank	Frank Tashlin	tt0061447
1746248011	1966	1966	The Wild World of Batwoman	The Wild World of Batwoman	Warren, Jerry	Jerry Warren	tt0061191
1746532636	1923	1923	Bell Boy 13	Bell Boy 13	Seiter, William	William A. Seiter	tt0013866
1746437002	1942	1942	The Silver Bullet	The Silver Bullet	Lewis, Joseph	Joseph H. Lewis	tt0035334
1746576848	1931	1931	The Sheriff's Secret	The Sheriff's Secret	Hogan, James P.	James P. Hogan	tt0022372
1746582480	1936	1936	The Old Corral	The Old Corral	Kane, Joseph	Joseph Kane	tt0028054
1746186017	1969	1969	That Tender Touch	That Tender Touch	Vincent, Russel	Russel Vincent	tt0153000
1746473727	1943	1943	Top Man	Top Man	Lamont, Charles	Charles Lamont	tt0036450
1746520173	1924	1924	The Man Without a Heart	The Man Without a Heart	King, Burton	Burton L. King	tt0015112
1745394013	2009	2008	Walt & El Grupo	Walt & El Grupo	Thomas, Theodore	Theodore Thomas	tt1223150
1746421578	1997	1997	The Devil's Own	The Devil's Own	Pakula, Alan J.	Alan J. Pakula	tt0118972
1746453627	1943	1943	Larceny with Music	Larceny with Music	Lilley, Edward	Edward C. Lilley	tt0036097
1746491774	1947	1947	Bells of San Angelo	Bells of San Angelo	Witney, William	William Witney	tt0039180
1746508505	1924	1924	Another Scandal	Another Scandal	Griffith, Edward H.	Edward H. Griffith	tt0014678
1746465107	1944	1944	The Canterville Ghost	The Canterville Ghost	Dassin, Jules; McLeod, Norman Z.	Jules Dassin	tt0036696
1746497645	1920	1920	Up in Mary's Attic	Up in Mary's Attic	Watson, William H.	William Watson	tt0180276
1746585273	1928	1928	There It Is	There It Is	Muller, H. L.	Harold L. Muller	tt0019466
1745309284	2005	2005	Dark Water	Dark Water	Salles, Walter	Walter Salles	tt0382628
1746508760	1924	1924	Ramshackle House	Ramshackle House	Weight, Harmon	F. Harmon Weight	tt0015259
1745395966	2010	2010	Going the Distance	Going the Distance	Burstein, Nanette	Nanette Burstein	tt1322312
1746584129	1938	1938	Convicts at Large	Convicts at Large	Beal, Scott; Friedman, David A.	Scott R. Beal	tt0165696
1746513459	1915	1915	In the Palace of the King	In the Palace of the King	Wright, Fred	Fred E. Wright	tt0005541
1746438289	1943	1943	Raiders of Sunset Pass	Raiders of Sunset Pass	English, John	John English	tt0036291
1746586159	1940	1940	Rhythm on the River	Rhythm on the River	Schertzinger, Victor	Victor Schertzinger	tt0032986
1746502336	1916	1916	The Phantom	The Phantom	Giblyn, Charles	Charles Giblyn	tt0007180
1746580435	1935	1935	Never Too Late	Never Too Late	Shamray, Franklin	Bernard B. Ray	tt0026771
1769971077	1969	1969	Mnasidika	Mnasidika		Michael Findlay	tt0064677
1746566361	1958	1958	The Hunters	The Hunters	Powell, Dick	Dick Powell	tt0051750
1746508020	1925	1926	Trapped	Trapped		William A. Crinley	tt0159800
1746580351	1938	1938	California Frontier	California Frontier	Clifton, Elmer	Elmer Clifton	tt0029959
1746580812	1936	1936	Neighborhood House	Neighborhood House	Parrott, Charles; Law, Harold; Hale, A	Charley Chase	tt0028024
1746514772	1913	1913	The Tonopah Stampede for Gold	The Tonopah Stampede for Gold	Pryor, Charles A.	Charles A. Pryor	tt0003464
1746569633	1954	1954	Drums of Tahiti	Drums of Tahiti	Castle, William	William Castle	tt0046938
1746582223	1951	1951	Queen for a Day	Queen for a Day	Lubin, Arthur	Arthur Lubin	tt0043947
1746436270	1990	1990	Texasville	Texasville	Bogdanovich, Peter	Peter Bogdanovich	tt0103069
1746454674	1942	1942	The Living Ghost	The Living Ghost	Beaudine, William	William Beaudine	tt0034985
1746464892	1942	1942	Flying with Music	Flying with Music	Archainbaud, George	George Archainbaud	tt0034743
1746574520	1953	1952	Limelight	Limelight	Chaplin, Charles	Charles Chaplin	tt0044837
1746393176	1988	1988	Short Circuit 2	Short Circuit 2	Johnson, Kenneth	Kenneth Johnson	tt0096101
1746569725	1953	1953	Beneath the 12-Mile Reef	Beneath the 12 Mile Reef	Webb, Robert D.	Robert D. Webb	tt0045551
1746250536	1977	1977	American Tickler	American Tickler	Vincent, Chuck	Chuck Vincent	tt0074135
1746504411	1920	1920	Woman's Man	A Woman's Man	Gordon, Warren	Warren Gordon	tt0202688
1746500939	1915	1915	The Mystery of Room 13	The Mystery of Room 13	Ridgwell, George	George Ridgwell	tt0005795
1746393434	1983	1983	My Tutor	My Tutor	Bowers, George	George Bowers	tt0085980
1746520865	1949	1949	Easy Living	Easy Living	Tourneur, Jacques	Jacques Tourneur	tt0041328
1746583712	1928	1928	Breed of the Sunsets	Breed of the Sunsets	Fox, Wallace W.	Wallace Fox	tt0018719
1745359151	2009	2009	Bandslam	Bandslam	Graff, Todd	Todd Graff	tt0976222
1746584866	1930	1930	The Life of the Party	The Life of the Party	Del Ruth, Roy	Roy Del Ruth	tt0021069
1746282242	1980	1980	Cruising	Cruising	Friedkin, William	William Friedkin	tt0080569
1746533225	1915	1915	Bred in the Bone	Bred in the Bone	Powell, Paul	Paul Powell	tt0004999
1746513182	1915	1915	The White Scar	The White Scar	Davis, Ulysses	Hobart Bosworth	tt0006280
1746503818	1922	1922	Wolf Pack	Wolf Pack	Craft, William J.	William James Craft	tt0180312
1746453770	1941	1941	Glamour Boy	Glamour Boy	Murphy, Ralph; Tetzlaff, Ted	Ralph Murphy	tt0033666
1746218845	1972	1972	Deliverance	Deliverance	Boorman, John	John Boorman	tt0068473
1746516528	1921	1921	Man--Woman--Marriage	Man Woman Marriage	Holubar, Allen	Allen Holubar	tt0012445
1746408433	1984	1984	Night of the Comet	Night of the Comet	Eberhardt, Thom	Thom Eberhardt	tt0087799

1746583926	1931	1931	Near the Trail's End	Near the Trail's End	Fox, Wallace	Wallace Fox	tt0022192
1745302604	2006	2005	Trust the Man	Trust the Man	Freundlich, Bart	Bart Freundlich	tt0427968
1746514110	1914	1914	The Unwelcome Mrs. Hatch	The Unwelcome Mrs. Hatch		Allan Dwan	tt0004754
1745342880	2002	2002	Slackers	Slackers	Nicks, Dewey	Dewey Nicks	tt0240900
1746569778	1954	1954	Suddenly	Suddenly	Allen, Lewis	Lewis Allen	tt0047542
1746567966	1934	1934	Born to Be Bad	Born to Be Bad	Sherman, Lowell	Lowell Sherman	tt0024906
1746513650	1923	1923	The Love Trap	The Love Trap	Ince, John	John Ince	tt0014221
1769971165	1969	1969	Felicia	Felicia	Shiffen, Arlo	Don Walters	tt0218221
1746524888	1928	1928	The Night Flyer	The Night Flyer	Lang, Walter	Walter Lang	tt0019211
1746578051	1937	1937	Smoke Tree Range	Smoke Tree Range	Selander, Lesley	Lesley Selander	tt0029582
1746581028	1951	1951	Call Me Mister	Call Me Mister	Bacon, Lloyd	Lloyd Bacon	tt0043370
1745358069	2008	2008	Wanted	Wanted	Bekmambetov, Timur	Timur Bekmambetov	tt0493464
1746560013	1965	1965	Convict Stage	Convict Stage	Selander, Lesley	Lesley Selander	tt0122027
1746530691	1949	1949	Henry, the Rainmaker	Henry, the Rainmaker	Yarbrough, Jean	Jean Yarbrough	tt0041457
1746578859	1963	1963	It Happened at the World's Fair	It Happened at the World's Fair	Taurog, Norman	Norman Taurog	tt0057191
1746514949	1916	1916	A Prince in a Pawnshop	A Prince in a Pawnshop	Scardon, Paul	Paul Scardon	tt0007219
1746509526	1948	1948	Mark of the Lash	Mark of the Lash	Taylor, Ray	Ray Taylor	tt0040571
1746566143	1958	1958	Damn Yankees	Damn Yankees	Abbott, George; Donen, Stanley	George Abbott	tt0051516
1769973126	1924	1924	Andy's Stump Speech	Andy's Stump Speech	Taurog, Norman	Norman Taurog	tt0321231
1746455365	1994	1994	Naked Gun 33 1/3: The Final Insult	Naked Gun 33 1/3: The Final Insult	Segal, Peter	Peter Segal	tt0110622
1746513841	1923	1923	Ridin' Thru	Ridin' Thru		Dick Hatton	tt0436719
1746234962	1967	1967	I Was a Man	I Was a Man	Mahon, Barry	Barry Mahon	tt0190473
1746524698	1915	1915	The Frame-Up	The Frame Up	Turner, Otis	Otis Turner	tt0005356
1745282864	2006	2006	RV	RV	Sonnenfeld, Barry	Barry Sonnenfeld	tt0449089
1746502088	1917	1917	Conscience	Conscience	Bracken, Bertram	Bertram Bracken	tt0005130
1746570329	1933	1933	Hell Below	Hell Below	Conway, Jack	Jack Conway	tt0024100
1746217288	1966	1966	An Eye for an Eye	An Eye for an Eye	Moore, Michael	Michael D. Moore	tt0060386
1746465097	1945	1945	The Crime Doctor's Warning	The Crime Doctor's Warning	Castle, William	William Castle	tt0037621
1746573167	1910	1910	For a Woman's Honor	For a Woman's Honor		Sidney Olcott	tt1912395
1746210494	1973	1973	Little Cigars	Little Cigars	Christenberry, Chris	Chris Christenberry	tt0070323
1745309404	2006	2006	The Second Chance	The Second Chance	Taylor, Steve	Steve Taylor	tt0429068
1746583632	1929	1929	Western Methods	Western Methods			tt0177386
1746423309	1995	1995	Father of the Bride Part II	Father of the Bride Part II	Shyer, Charles	Charles Shyer	tt0113041
1746584771	1928	1928	The Cowboy Cavalier	The Cowboy Cavalier	Thorpe, Richard	Richard Thorpe	tt0018798
1746508432	1950	1950	Captain China	Captain China	Foster, Lewis R.	Lewis R. Foster	tt0041227
1746570981	1934	1934	The Man from Utah	The Man from Utah	Bradbury, Robert	Robert N. Bradbury	tt0025455
1746580303	1937	1937	Left-Handed Law	Left Handed Law	Selander, Lesley	Lesley Selander	tt0029129
1745309471	2007	2007	Balls of Fury	Balls of Fury	Garant, Robert Ben	Robert Ben Garant	tt0424823
1746555280	1956	1956	Manfish	Manfish	Wilder, W. Lee	W. Lee Wilder	tt0049477
1746464911	1946	1946	In Fast Company	In Fast Company	Lord, Del	Del Lord	tt0038636
1746570999	1934	1934	Evelyn Prentice	Evelyn Prentice	Howard, William K.	William K. Howard	tt0025091
1746501384	1916	1916	Atta Boy's Last Race	Atta Boy's Last Race	Siegmann, George	George Siegmann	tt0006381
1746523969	1927	1927	Hula	Hula	Fleming, Victor	Victor Fleming	tt0018016
1746487372	1918	1918	Till I Come Back to You	Till I Come Back to You	DeMille, Cecil B.	Cecil B. DeMille	tt0009702
1745302302	2007	2007	Into the Wild	Into the Wild	Penn, Sean	Sean Penn	tt0758758
1746556001	1954	1954	Taza, Son of Cochise	Taza, Son of Cochise	Sirk, Douglas	Douglas Sirk	tt0047562
1769970839	1915	1915	Beyond All Is Love	Beyond All Is Love	Greene, Clay M.	Clay M. Greene	tt0416544
1746475977	1945	1945	Escape in the Desert	Escape in the Desert	Blatt, Edward A.	Edward A. Blatt	tt0037677
1746583397	1930	1930	Lightnin'	Lightnin'	King, Henry	Henry King	tt0021071
1746421712	1993	1993	A Perfect World	A Perfect World	Eastwood, Clint	Clint Eastwood	tt0107808
1746234787	1974	1974	The Swinging Cheerleaders	The Swinging Cheerleaders	Hill, Jack	Jack Hill	tt0072236
1746583888	1931	1931	Hell Bent for Frisco	Hell Bent for Frisco	Paton, Stuart	Stuart Paton	tt0021948
1746261214	1979	1979	The Wanderers	The Wanderers	Kaufman, Philip	Philip Kaufman	tt0080117
1746584280	1935	1935	The Case of the Lucky Legs	The Case of the Lucky Legs	Mayo, Archie L.	Archie Mayo	tt0026185
1746394748	1985	1985	American Ninja	American Ninja	Firstenberg, Sam	Sam Firstenberg	tt0088708
1746184516	1910	1910	Chief Blackfoot's Vindication	Chief Blackfoot's Vindication		Kenean Buel	tt1914227
1746508371	1949	1949	The Threat	The Threat	Feist, Felix	Felix E. Feist	tt0041963

1746577516	1938	1938	The Young in Heart	The Young in Heart	Wallace, Richard	Richard Wallace	tt0031002
1746524506	1916	1916	My Partner	My Partner	Sanger, Mr.	Mr. Sanger	tt0007106
1746583147	1935	1935	The Drunkard	The Drunkard	Herman, Albert	Albert Herman	tt0132929
1746394766	1991	1991	What About Bob?	What About Bob?	Oz, Frank	Frank Oz	tt0103241
1746464923	1941	1941	Repent at Leisure	Repent at Leisure	Woodruff, Frank	Frank Woodruff	tt0034094
1746199538	1969	1969	Putney Swope	Putney Swope		Robert Downey Sr.	tt0064855
1746501150	1914	1914	Tess of the Storm Country	Tess of the Storm Country		Edwin S. Porter	tt0004681
1746507583	1926	1926	Skinner's Dress Suit	Skinner's Dress Suit	Seiter, William A.	William A. Seiter	tt0017401
1745372376	2008	2008	Zombie Strippers	Zombie Strippers	Lee, Jay	Jay Lee	tt0960890
1746507872	1925	1925	Hearts and Spurs	Hearts and Spurs	Van Dyke, William S.	W.S. Van Dyke	tt0015903
1746475505	1943	1943	Swing Shift Maisie	Swing Shift Maisie	McLeod, Norman Z.	Norman Z. McLeod	tt0036407
1746438616	1941	1941	Sing for Your Supper	Sing for Your Supper	Barton, Charles	Charles Barton	tt0035339
1746474405	1944	1944	San Fernando Valley	San Fernando Valley	English, John	John English	tt0037245
1746514486	1927	1927	Cheating Cheaters	Cheating Cheaters	Laemmle, Edward	Edward Laemmle	tt0017746
1746502679	1916	1916	What Love Can Do	What Love Can Do	Hunt, Jay	Jay Hunt	tt0007551
1746500037	1946	1946	Santa Fe Uprising	Santa Fe Uprising	Springsteen, R. G.	R.G. Springsteen	tt0038907
1746208531	1969	1969	The April Fools	The April Fools	Rosenberg, Stuart	Stuart Rosenberg	tt0064036
1746507811	1926	1926	The Cowboy and the Countess	The Cowboy and the Countess	Neill, R. William	Roy William Neill	tt0016746
1746582192	1951	1950	The Sound of Fury	The Sound of Fury	Endfield, Cyril	Cy Endfield	tt0043075
1746197908	1968	1968	A Lovely Way To Die	A Lovely Way to Die	Rich, David Lowell	David Lowell Rich	tt0063246
1746514580	1917	1917	Infidelity	Infidelity	Miller, Ashley	Ashley Miller	tt0176852
1746545349	1940	1940	Phantom Submarine	The Phantom Submarine	Barton, Charles	Charles Barton	tt0034021
1746504199	1920	1920	Something Different	Something Different	Neill, R. William	Roy William Neill	tt0012712
1746410208	1984	1984	Best Defense	Best Defense	Huyck, Willard	Willard Huyck	tt0086955
1746584004	1939	1939	Yes, My Darling Daughter	Yes, My Darling Daughter	Keighley, William	William Keighley	tt0032149
1746582991	1935	1935	Powdersmoke Range	Powdersmoke Range	Fox, Wallace	Wallace Fox	tt0026886
1746219004	1974	1974	Like Father, Like Son	Like Father, Like Son	Mitchell, Duke	Duke Mitchell	tt0077525
1746492120	1948	1948	Tarzan and the Mermaids	Tarzan and the Mermaids	Florey, Robert	Robert Florey	tt0040862
1746492563	1946	1946	The Falcon's Adventure	The Falcon's Adventure	Berke, William	William Berke	tt0038518
1746578698	1950	1950	Tea for Two	Tea for Two	Butler, David	David Butler	tt0043030
1746578765	1959	1959	The Miracle of the Hills	The Miracle of the Hills	Landres, Paul	Paul Landres	tt0053068
1746453622	1943	1943	Raiders of San Joaquin	Raiders of San Joaquin	Collins, Lewis D.	Lewis D. Collins	tt0036290
1746578726	1961	1961	All in a Night's Work	All in a Night's Work	Anthony, Joseph	Joseph Anthony	tt0054615
1746530683	1950	1950	Holiday Rhythm	Holiday Rhythm	Scholl, Jack	Jack Scholl	tt0042568
1746569613	1953	1953	San Antone	San Antone	Kane, Joseph	Joseph Kane	tt0046271
1746579057	1960	1960	The Last Voyage	The Last Voyage	Stone, Andrew L.	Andrew L. Stone	tt0054016
1746475263	1948	1948	Feudin', Fussin and A-Fightin'	Feudin', Fussin' and A Fightin'	Sherman, George	George Sherman	tt0040349
1746577568	1933	1933	Convention City	Convention City	Mayo, Archie	Archie Mayo	tt0023906
1746582431	1939	1939	Harlem Rides the Range	Harlem Rides the Range	Kahn, Richard C.	Richard C. Kahn	tt0031406
1746580364	1938	1938	Double Danger	Double Danger	Landers, Lew	Lew Landers	tt0030074
1746524505	1923	1923	Merry-Go-Round	Merry Go Round	Julian, Rupert	Rupert Julian	tt0013386
1746572975	1931	1931	The Devil Plays	The Devil Plays	Thorpe, Richard	Richard Thorpe	tt0021796
1769973314	1970	1970	I Am Curious--Tahiti	I Am Curious Tahiti	Tobalina, Carlos	Carlos Tobalina	tt0189596
1746421323	1994	1994	Little Giants	Little Giants	Dunham, Duwayne	Duwayne Dunham	tt0110364
1746233305	1968	1968	Revolution	Revolution	O'Connell, Jack	Jack O'Connell	tt0063503
1769970650	1966	1966	Caught in the Act!	Caught in the Act!	Nehemiah, J.	Jerald Intrator	tt0212850
1746500002	1945	1945	San Antonio	San Antonio	Butler, David	David Butler	tt0038048
1746576930	1961	1961	Frontier Uprising	Frontier Uprising	Cahn, Edward L.	Edward L. Cahn	tt0054899
1746248498	1967	1967	Thunder Alley	Thunder Alley	Rush, Richard	Richard Rush	tt0062365
1746465256	1947	1947	Return of the Lash	Return of the Lash	Taylor, Ray	Ray Taylor	tt0039764
1746508266	1927	1927	South Sea Love	South Sea Love	Ince, Ralph	Ralph Ince	tt0018432
1746566775	1952	1952	Plymouth Adventure	Plymouth Adventure	Brown, Clarence	Clarence Brown	tt0045039
1746578478	1951	1950	Appointment with Danger	Appointment with Danger	Allen, Lewis	Lewis Allen	tt0043292
1746561422	1932	1932	Downstairs	Downstairs	Bell, Monta	Monta Bell	tt0022834
1746550071	1954	1954	Salt of the Earth	Salt of the Earth	Biberman, Herbert J.	Herbert J. Biberman	tt0047443
1746525020	1920	1920	The Notorious Mrs. Sands	The Notorious Mrs. Sands	Cabanne, William Christy	Christy Cabanne	tt0011519
1746581193	1910	1910	In the Wilderness	In the Wilderness			tt1056076

1746501629	1915	1915	A Woman's Past	A Woman's Past	Powell, Frank	Frank Powell	tt0006307	
1745340798	2001	2000	Punks	Punks	Polk, Patrik-Ian	Patrik	tt0160710	
1746500579	1949	1949	The Lady Gambles	The Lady Gambles	Gordon, Michael	Michael Gordon	tt0041572	
1746585012	1940	1940	Five Little Peppers in Trouble	Five Little Peppers in Trouble	Barton, Charles	Charles Barton	tt0032473	
1746439433	1998	1998	My Giant	My Giant	Lehmann, Michael	Michael Lehmann	tt0120765	
1746421330	1992	1992	The Gun in Betty Lou's Handbag	The Gun in Betty Lou's Handbag	Moyle, Allan	Allan Moyle	tt0104376	
1746487575	1919	1919	The Indestructible Wife	The Indestructible Wife	Maigne, Charles	Charles Maigne	tt0010296	
1745342624	2002	2002	Stealing Harvard	Stealing Harvard	McCulloch, Bruce	Bruce McCulloch	tt0265808	
1746578010	1937	1937	Sudden Bill Dorn	Sudden Bill Dorn	Taylor, Ray	Ray Taylor	tt0030809	
1746520265	1925	1925	Kentucky Pride	Kentucky Pride	Ford, John	John Ford	tt0015973	
1746421276	1993	1993	Surf Ninjas	Surf Ninjas	Israel, Neal	Neal Israel	tt0108258	
1746566062	1957	1957	The Phantom Stagecoach	The Phantom Stagecoach	Nazarro, Ray	Ray Nazarro	tt0050840	
1746408839	1987	1987	Dolls	Dolls	Gordon, Stuart	Stuart Gordon	tt0092906	
1746501222	1923	1923	Men in the Raw	Men in the Raw	Marshall, George E.	George Marshall	tt0014268	
1746208845	1968	1968	The Other Side of Bonnie and Clyde	The Other Side of Bonnie and Clyde	Buchanan, Larry	Larry Buchanan	tt0063399	
1746532478	1923	1923	Adam and Eva	Adam and Eva	Vignola, Robert	Robert G. Vignola	tt0012879	
1746514615	1910	1910	The Twisted Trail	The Twisted Trail	Griffith, D. W.	D.W. Griffith	tt0001427	
1746501012	1914	1914	The Education of Mr. Pipp	The Education of Mr. Pipp		William F. Haddock	tt0003875	
1746453650	1941	1940	Little Men	Little Men	McLeod, Norman Z.	Norman Z. McLeod	tt0032717	
1746525187	1921	1921	Anne of Little Smoky	Anne of Little Smoky	Connor, Edward	Edward Connor	tt0011926	
1746581001	1959	1959	Day of the Outlaw	Day of the Outlaw	DeToth, Andre	André De Toth	tt0052724	
1746503587	1920	1920	The Invisible Divorce	The Invisible Divorce	Mills, Thomas R.; Deverich, Nat C.	Nat G. Deverich	tt0011334	
1746585340	1930	1930	The Office Wife	The Office Wife	Bacon, Lloyd	Lloyd Bacon	tt0021197	
1746583612	1931	1931	Anybody's Blonde	Anybody's Blonde	Strayer, Frank	Frank R. Strayer	tt0021613	
1746503944	1919	1918	The Nature Girl	The Nature Girl	Lund, O. A. C.	O.A.C. Lund	tt0009421	
1746259879	1978	1978	Interiors	Interiors	Allen, Woody	Woody Allen	tt0077742	
1745374008	2010	2009	Chain Letter	Chain Letter	Taylor , Deon	Deon Taylor	tt1148200	
1746508079	1923	1923	His Last Race	His Last Race	Eason, Reeves; Mitchell, Howard	B. Reeves Eason	tt0014135	
1746574615	1933	1933	Only Yesterday	Only Yesterday	Stahl, John M.	John M. Stahl	tt0024418	
1746509245	1925	1925	Too Many Kisses	Too Many Kisses	Sloane, Paul	Paul Sloane	tt0016439	
1746582704	1935	1935	Annapolis Farewell	Annapolis Farewell	Hall, Alexander	Alexander Hall	tt0026072	
1746435587	1941	1941	The Cowboy and the Blonde	The Cowboy and the Blonde	McCarey, Ray	Ray McCarey	tt0033495	
1746524526	1915	1915	Money	Money	Keane, James	James Keane	tt0005763	
1746502568	1916	1916	The Woman Who Dared	The Woman Who Dared	Middleton, George E.	George E. Middleton	tt0159135	
1746585205	1929	1929	The Vagabond Cub	The Vagabond Cub	King, Louis	Louis King	tt0348301	
1746520381	1949	1949	The Big Steal	The Big Steal	Siegel, Don	Don Siegel	tt0041178	
1746582077	1930	1930	They Learned About Women	They Learned About Women	Conway, Jack; Wood, Sam	Jack Conway	tt0021464	
1746565913	1956	1956	Thunder over Arizona	Thunder Over Arizona	Kane, Joe	Joseph Kane	tt0049851	
1746502338	1917	1917	The Debt	The Debt	Powell, Frank	Frank Powell	tt0007844	
1746491745	1948	1948	The Gay Ranchero	The Gay Ranchero	Witney, William	William Witney	tt0040385	
1746575045	1937	1937	Green Light	Green Light	Borzage, Frank	Frank Borzage	tt0028958	
1746495703	1920	1920	The Amazing Woman	The Amazing Woman	Adolfi, John G.	John G. Adolfi	tt0184203	
1745358075	2010	2010	Cats & Dogs: The Revenge of Kitty Galore	Cats & Dogs: The Revenge of Kitty Galore	Peyton, Brad	Brad Peyton	tt1287468	
1746454468	1942	1942	Mrs. Wiggs of the Cabbage Patch	Mrs. Wiggs of the Cabbage Patch	Murphy, Ralph	Ralph Murphy	tt0035094	
1746520740	1925	1925	The Shadow on the Wall	The Shadow on the Wall	Eason, Reeves	B. Reeves Eason	tt0016337	
**1746504561**	**1921**	**1921**	**Crossing Trails**	**Crossing Trails**	**Smith, Cliff**	**Clifford Smith**	**tt0012080**	1
1746581287	1910	1910	Liz's Career	Liz's Career			tt1480814	
1746435694	1942	1942	The Bashful Bachelor	The Bashful Bachelor	St. Clair, Malcolm	Malcolm St. Clair	tt0033379	
1746422594	1999	1999	Office Space	Office Space	Judge, Mike	Mike Judge	tt0151804	
1746574982	1936	1936	Poor Little Rich Girl	Poor Little Rich Girl	Cummings, Irving	Irving Cummings	tt0028118	
1746507721	1927	1926	Savage Passions	Savage Passions	Allen, Fred S.	Fred Allen	tt0018360	
1746500643	1924	1924	Alimony	Alimony	Horne, James W.	James W. Horne	tt0014666	
1746525068	1921	1921	The Kingdom of Human Hearts	The Kingdom of Human Hearts	Cosper, Wilbert Leroy	Wilbert Leroy Cosper	tt0330526	
1746582465	1936	1936	King of Hockey	King of Hockey	Smith, Noel	Noel M. Smith	tt0027848	
1746562105	1954	1954	Day of Triumph	Day of Triumph	Pichel, Irving; Coyle, John T.	John T. Coyle	tt0046895	
1745342914	2002	2002	Jane White Is Sick & Twisted	Jane White Is Sick & Twisted	Latt, David Michael	David Michael Latt	tt0271018	
1746570754	1934	1935	Home on the Range	Home on the Range	Jacobson, Arthur	Arthur Jacobson	tt0026484	

1746454680	1941	1940	Life with Henry	Life with Henry	Reed, Jay Theodore	Theodore Reed	tt0033834
1746524494	1927	1927	Born To Battle	Born to Battle	Neitz, Alvin J.	Alan James	tt0355282
1746578675	1961	1961	Gun Street	Gun Street	Cahn, Edward L.	Edward L. Cahn	tt0056043
1746582562	1936	1936	The President's Mystery	The President's Mystery	Rosen, Phil	Phil Rosen	tt0028135
1746409346	1985	1985	A Chorus Line	A Chorus Line	Attenborough, Richard	Richard Attenborough	tt0088915
1746581742	1928	1928	Our Dancing Daughters	Our Dancing Daughters	Beaumont, Harry	Harry Beaumont	tt0019237
1746434002	1996	1996	Two If by Sea	Two If by Sea	Bennett, Bill	Bill Bennett	tt0118002
1746185144	1965	1965	Sylvia's Girls	Sylvia's Girls	Dempsey, Al	William Kerwin	tt0259024
1746199974	1969	1969	The Wonderful Land of Oz	The Wonderful Land of Oz	Mahon, Barry	Barry Mahon	tt0065223
1746500666	1925	1925	Kiss Me Again	Miss Me Again	Lubitsch, Ernst	Wesley Ruggles	tt0425256
1746572258	1931	1931	Trader Horn	Trader Horn	Van Dyke, W. S.	W.S. Van Dyke	tt0022495
1746582134	1951	1951	The Brave Bulls	The Brave Bulls	Rossen, Robert	Robert Rossen	tt0043359
1746393999	1989	1989	New York Stories	New York Stories		Woody Allen	tt0097965
1746514470	1922	1922	Chain Lightning	Chain Lightning	Wilson, Ben	Ben F. Wilson	tt0013005
1746409696	1986	1986	One More Saturday Night	One More Saturday Night	Klein, Dennis	Dennis Klein	tt0091681
1746419158	1986	1986	From Beyond	From Beyond	Gordon, Stuart	Stuart Gordon	tt0091083
1746584559	1931	1931	Born to Love	Born to Love	Stein, Paul L.	Paul L. Stein	tt0021684
1746516295	1922	1922	The Lying Truth	The Lying Truth	Fairfax, Marion	Marion Fairfax	tt0013348
1746410604	1988	1988	Moving	Moving	Metter, Alan	Alan Metter	tt0095662
1746437429	1942	1942	Raiders of the West	Raiders of the West	Stewart, Peter	Sam Newfield	tt0035237
1746578068	1938	1938	The Storm	The Storm	Young, Harold	Harold Young	tt0030800
1746583722	1930	1930	See America Thirst	See America Thirst	Craft, William James	William James Craft	tt0021354
1746509550	1925	1925	West of Arizona	West of Arizona	Gibson, Tom	Tom Gibson	tt0177371
1746561417	1932	1932	Strangers of the Evening	Strangers of the Evening	Humberstone, H. Bruce	H. Bruce Humberstone	tt0023528
1746235199	1976	1975	The Giant Spider Invasion	The Giant Spider Invasion	Rebane, Bill	Bill Rebane	tt0073043
1746408897	1982	1982	A Midsummer Night's Sex Comedy	A Midsummer Night's Sex Comedy	Allen, Woody	Woody Allen	tt0084329
1746580851	1938	1938	Little Miss Roughneck	Little Miss Roughneck	Scotto, Aubrey	Aubrey Scotto	tt0030373
1746465331	1947	1947	The Sin of Harold Diddlebock	The Sin of Harold Diddlebock	Sturges, Preston	Preston Sturges	tt0039825
1746409931	1990	1990	Awakenings	Awakenings	Marshall, Penny	Penny Marshall	tt0099077
1746581778	1930	1930	The Social Lion	The Social Lion	Sutherland, A. Edward	A. Edward Sutherland	tt0021396
1746501292	1913	1913	The Inside of the White Slave Traffic	The Inside of the White Slave Traffic	Beal, Frank	Frank Beal	tt0003016
1746524777	1928	1928	Old Age Handicap	Old Age Handicap	Mattison, Frank S.	Frank S. Mattison	tt0144533
1746584277	1939	1939	Western Caravans	Western Caravans	Nelson, Sam	Sam Nelson	tt0032120
1746433734	1999	1999	Austin Powers: The Spy Who Shagged Me	Austin Powers: The Spy Who Shagged Me	Roach, Jay	Jay Roach	tt0145660
1746502992	1916	1916	The Ninety and Nine	The Ninety and Nine	Ince, Ralph W.	Ralph Ince	tt0007129
1746420392	1981	1981	Friday the 13th Part 2	Friday the 13th Part 2	Miner, Steve	Steve Miner	tt0082418
1746186405	1969	1969	From Nashville With Music	From Nashville with Music	Crandall, Eddie; Patrick, Robert	Eddie Crandall	tt0064348
1746509253	1927	1927	The Silver Slave	The Silver Slave	Bretherton, Howard	Howard Bretherton	tt0018405
1746563807	1951	1951	Teresa	Teresa	Zinnemann, Fred	Fred Zinnemann	tt0044112
1746500311	1950	1950	Colorado Ranger	Colorado Ranger	Carr, Thomas	Thomas Carr	tt0042337
1746514895	1927	1927	Code of the Cow Country	Code of the Cow Country	Apfel, Oscar	Oscar Apfel	tt0017763
1746565471	1952	1952	Man from the Black Hills	Man from the Black Hills	Carr, Thomas	Thomas Carr	tt0044873
1746581808	1951	1951	The Tanks Are Coming	The Tanks Are Coming	Seiler, Lewis	Lewis Seiler	tt0044106
1746584722	1929	1929	The Hole in the Wall	The Hole in the Wall	Florey, Robert	Robert Florey	tt0019992
1746408855	1986	1986	The Wraith	The Wraith	Marvin, Mike	Mike Marvin	tt0092240
1746520837	1950	1950	American Guerrilla in the Philippines	American Guerrilla in the Philippines	Lang, Fritz	Fritz Lang	tt0042195
1745375188	2010	2010	Iron Man 2	Iron Man 2	Favreau, Jon	Jon Favreau	tt1228705
1746256141	1975	1975	Mitchell	Mitchell	McLaglen, Andrew V.	Andrew V. McLaglen	tt0073396
1746455168	1995	1995	Jury Duty	Jury Duty	Fortenberry, John	John Fortenberry	tt0113500
1746520764	1950	1950	Francis	Francis	Lubin, Arthur	Arthur Lubin	tt0041387
1746570833	1933	1933	Smoky	Smoky	Forde, Eugene	Eugene Forde	tt0024577
1746580219	1938	1938	Lightning Carson Rides Again	Lightning Carson Rides Again	Newfield, Sam	Sam Newfield	tt0030366
1746565325	1932	1932	The Fighting Gentleman	The Fighting Gentleman	Newmeyer, Fred	Fred C. Newmeyer	tt0022885
1746208833	1968	1968	The Horse in the Gray Flannel Suit	The Horse in the Gray Flannel Suit	Tokar, Norman	Norman Tokar	tt0063091
1746436049	1943	1943	A Lady Takes a Chance	A Lady Takes a Chance	Seiter, William A.	William A. Seiter	tt0036092
1746584773	1929	1929	They Had To See Paris	They Had to See Paris	Borzage, Frank	Frank Borzage	tt0020490
1746509230	1927	1927	One-Round Hogan	One Round Hogan	Bretherton, Howard	Howard Bretherton	tt0018231

1746577995	1937	1937	Cheyenne Rides Again	Cheyenne Rides Again	Hill, Bob	Robert F. Hill	tt0028712
1746502856	1915	1915	June Friday	June Friday	McRae, Duncan	Duncan McRae	tt0005580
1746210573	1970	1970	Husbands	Husbands	Cassavetes, John	John Cassavetes	tt0065867
1746422245	1993	1993	Swing Kids	Swing Kids	Carter, Thomas	Thomas Carter	tt0108265
1746555229	1955	1955	Oklahoma!	Oklahoma!	Zinnemann, Fred	Fred Zinnemann	tt0048445
1746514755	1915	1915	The Lonesome Heart	The Lonesome Heart		William Desmond Taylor	tt0005645
1746583443	1930	1930	Beyond the Law	Beyond the Law	McGowan, J. P.	J.P. McGowan	tt0020681
1746491950	1944	1943	Destination Tokyo	Destination Tokyo	Daves, Delmer	Delmer Daves	tt0035799
1746581420	1950	1950	Walk Softly, Stranger	Walk Softly, Stranger	Stevenson, Robert	Robert Stevenson	tt0043118
1746553743	1964	1964	The World of Henry Orient	The World of Henry Orient	Hill, George Roy	George Roy Hill	tt0058756
1745373076	2004	2004	The Hillside Strangler	The Hillside Strangler	Parello, Chuck	Chuck Parello	tt0376649
1746508460	1926	1926	Midnight Limited	The Midnight Limited	Apfel, Oscar	Oscar Apfel	tt0017141
1746576329	1931	1931	The Ridin' Fool	The Ridin' Fool	McCarthy, J. P.	John P. McCarthy	tt0022316
1746583184	1930	1930	Breed of the West	Breed of the West	Neitz, Alvin J.	Alan James	tt0020709
1746219067	1980	1980	Midnight Madness	Midnight Madness	Wechter, David; Nankin, Michael	Michael Nankin	tt0081159
1746580254	1936	1936	Girl of the Ozarks	Girl of the Ozarks	Shea, William	William Shea	tt0027675
1746582170	1928	1928	The Stronger Will	The Stronger Will	McEveety, Bernard	Bernard McEveety	tt0019433
1746500376	1924	1924	Laughing at Danger	Laughing at Danger	Horne, James W.	James W. Horne	tt0015053
1746408899	1990	1990	Side Out	Side Out	Israelson, Peter	Peter Israelson	tt0100613
1746508166	1950	1950	A Modern Marriage	A Modern Marriage	Landres, Paul	Paul Landres	tt0042743
1746233240	1968	1968	The Name of the Game Is Kill!	The Name of the Game Is Kill!	Hellström, Gunnar	Gunnar Hellström	tt0063335
1746569878	1960	1960	Seven Thieves	Seven Thieves	Hathaway, Henry	Henry Hathaway	tt0054295
1746583691	1928	1928	Confessions of a Wife	Confessions of a Wife	Kelly, Albert	Albert H. Kelley	tt0018788
1746197968	1965	1965	The Hallelujah Trail	The Hallelujah Trail	Sturges, John	John Sturges	tt0059250
1745372399	2010	2010	All Good Things	All Good Things	Jarecki, Andrew	Andrew Jarecki	tt1175709
1746515270	1920	1920	Into the Light	Into the Light	Bradbury, Robert North	Robert N. Bradbury	tt0012749
1745358055	2002	2002	Deuces Wild	Deuces Wild	Kalvert, Scott	Scott Kalvert	tt0231448
1746581709	1930	1930	Shooting Straight	Shooting Straight	Archainbaud, George	George Archainbaud	tt0021369
1746520168	1926	1926	The Broadway Boob	The Broadway Boob	Henabery, Joseph	Joseph Henabery	tt0016682
1746198425	1969	1969	Paint Your Wagon	Paint Your Wagon	Logan, Joshua	Joshua Logan	tt0064782
1746574312	1960	1960	Violent Women	Violent Women	Mahon, Barry	Barry Mahon	tt0053424
1746582513	1937	1937	Once a Doctor	Once a Doctor	Clemens, William	William Clemens	tt0029346
1746500629	1926	1926	Rocking Moon	Rocking Moon	Melford, George	George Melford	tt0017331
1746219064	1972	1972	Hammer	Hammer	Clark, Bruce	Bruce D. Clark	tt0068673
1746555216	1955	1955	Stranger on Horseback	Stranger on Horseback	Tourneur, Jacques	Jacques Tourneur	tt0048666
1746561120	1932	1932	Western Limited	The Western Limited	Cabanne, Christy	Christy Cabanne	tt0023683
1745373185	2003	2003	Camp	Camp	Graff, Todd	Todd Graff	tt0342167
1746520711	1926	1926	Salt Lake Trail	Salt Lake Trail			tt0161954
1746581573	1928	1928	The Road to Ruin	The Road to Ruin	Parker, Norton S.	Dorothy Davenport	tt0019332
1746199198	1966	1966	The Bed and How To Make It!	The Bed and How to Make It!	Sarno, Joe	Joseph W. Sarno	tt0058961
1746422221	1999	1999	Life	Life	Demme, Ted	Ted Demme	tt0123964
1746492747	1948	1948	My Girl Tisa	My Girl Tisa	Nugent, Elliott	Elliott Nugent	tt0040628
1769971987	1969	1969	Dare the Devil	Dare the Devil	Emery, Robert J.	Robert J. Emery	tt0216660
1746434602	1997	1997	The Pest	The Pest	Miller, Paul	Paul Miller	tt0119887
1746495799	1919	1920	Puppy Love	Puppy Love	Neill, R. William		tt1666062
1746520879	1948	1948	The Rangers Ride	The Rangers Ride	Abrahams, Derwin	Derwin Abrahams	tt0179444
1746454685	1942	1942	Holiday Inn	Holiday Inn	Sandrich, Mark	Mark Sandrich	tt0034862
1746563481	1952	1952	Cattle Town	Cattle Town	Smith, Noel	Noel M. Smith	tt0044490
1746580790	1935	1935	Gun Play	Gun Play	Herman, Al	Albert Herman	tt0026445
1746533205	1915	1915	York State Folks	York State Folks	Jackson, Harry	Harry Jackson	tt0006325
1746523039	1916	1916	The Combat	The Combat	Ince, Ralph W.	Ralph Ince	tt0006537
1746584293	1939	1939	Everything Happens at Night	Everything Happens at Night	Cummings, Irving	Irving Cummings	tt0031285
1746507805	1926	1926	The Warning Signal	The Warning Signal	Hunt, Charles	Charles J. Hunt	tt0017528
1746508559	1949	1949	Top O' the Morning	Top o' the Morning	Miller, David	David Miller	tt0041969
1746422026	1998	1998	Small Soldiers	Small Soldiers	Dante, Joe	Joe Dante	tt0122718
1746249250	1980	1980	Coal Miner's Daughter	Coal Miner's Daughter	Apted, Michael	Michael Apted	tt0080549
1746258479	1979	1978	The Legacy	The Legacy	Marquand, Richard	Richard Marquand	tt0079450

1746217888	1966	1966	Born Free	Born Free	Hill, James	James Hill	tt0060182
1746502356	1915	1915	The Lion's Mate	The Lion's Mate	Santschi, Thomas	Tom Santschi	tt0490608
1746523553	1916	1916	Should a Baby Die?	Should a Baby Die?	Vekroff, Perry N.	Perry N. Vekroff	tt0158952
1746565980	1953	1953	Raiders of the Seven Seas	Raiders of the Seven Seas	Salkow, Sidney	Sidney Salkow	tt0046223
1746454043	1941	1941	The Strawberry Blonde	The Strawberry Blonde	Walsh, Raoul	Raoul Walsh	tt0034236
1746581103	1951	1951	Cry Danger	Cry Danger	Parrish, Robert	Robert Parrish	tt0043435
1746573407	1932	1932	Penguin Pool Murder	The Penguin Pool Murder	Archainbaud, George	George Archainbaud	tt0023327
1746567121	1932	1932	Forbidden Trail	Forbidden Trail	Hillyer, Lambert	Lambert Hillyer	tt0024029
1745359635	2004	2004	Kinsey	Kinsey	Condon, Bill	Bill Condon	tt0362269
1746583032	1939	1939	Ninotchka	Ninotchka	Lubitsch, Ernst	Ernst Lubitsch	tt0031725
1745394096	2010	2009	Winnebago Man	Winnebago Man	Steinbauer, Ben	Ben Steinbauer	tt1396557
1746513441	1927	1927	The Clown	The Clown	Craft, William James	William James Craft	tt0017761
1746525011	1915	1915	The Making of Crooks	The Making of Crooks	Daly, William Robert	William Robert Daly	tt0812283
1769970553	1968	1968	Michelene and the Device	Michelene and the Device	Roberts, R. Jack	Robert L. Roberts	tt0251264
1746423285	1996	1996	The Birdcage	The Birdcage	Nichols, Mike	Mike Nichols	tt0115685
1746582491	1935	1935	Red Salute	Red Salute	Lanfield, Sidney	Sidney Lanfield	tt0026919
1746570744	1934	1934	Flirtation	Flirtation	Birinski, Leo	Leo Birinsky	tt0026367
1746394527	1982	1982	Jimmy the Kid	Jimmy the Kid	Nelson, Gary	Gary Nelson	tt0085756
1746499913	1923	1923	The French Doll	The French Doll	Leonard, Robert Z.	Robert Z. Leonard	tt0014060
1746509531	1925	1925	The Rag Man	The Rag Man	Cline, Edward F.	Edward F. Cline	tt0016258
1769969933	1911	1911	Desperate Desmond Abducts Rosamond	Desperate Desmond Abducts Rosamond		Tom Ricketts	tt0213603
1746562281	1954	1954	King Richard and the Crusaders	King Richard and the Crusaders	Butler, David	David Butler	tt0047150
1746584937	1940	1940	Vigil in the Night	Vigil in the Night	Stevens, George	George Stevens	tt0033221
1746498680	1920	1920	April Folly	April Folly	Leonard, Robert Z.	Robert Z. Leonard	tt0010964
1746520970	1949	1949	Bagdad	Bagdad	Lamont, Charles	Charles Lamont	tt0041149
1769972174	1962	1962	The Wild and the Naked	The Wild and the Naked	Roberts, Stan	Stan Roberts	tt0153836
1746569947	1958	1958	The Proud Rebel	The Proud Rebel	Curtiz, Michael	Michael Curtiz	tt0052097
1746422543	1989	1989	The Dream Team	The Dream Team	Zieff, Howard	Howard Zieff	tt0097235
1746503326	1918	1918	The Return of Mary	The Return of Mary	Lucas, Wilfred	Wilfred Lucas	tt0009542
1746584813	1929	1929	Dynamite	Dynamite	De Mille, Cecil B.	Cecil B. DeMille	tt0019843
1746514593	1923	1923	Crimson Gold	Crimson Gold	Elfelt, Clifford S.	Clifford S. Elfelt	tt0013957
1746422583	1989	1988	976-EVIL	976 EVIL	Englund, Robert	Robert Englund	tt0094597
1746235425	1972	1972	Super Fly	Super Fly	Parks, Gordon	Gordon Parks Jr.	tt0069332
1746233985	1968	1968	The Brotherhood	The Brotherhood	Ritt, Martin	Martin Ritt	tt0062760
1746421325	1992	1992	Venice/Venice	Venice/Venice	Jaglom, Henry	Henry Jaglom	tt0105729
1746434997	1991	1991	Dice Rules	Dice Rules	Dubin, Jay	Jay Dubin	tt0101726
1746580964	1960	1960	CinderFella	Cinderfella	Tashlin, Frank	Frank Tashlin	tt0053716
1746567338	1933	1933	Stage Mother	Stage Mother	Brabin, Charles	Charles Brabin	tt0024609
1746435726	1940	1940	The Saint Takes Over	The Saint Takes Over	Hively, Jack	Jack Hively	tt0033013
1746465363	1942	1942	Flight Lieutenant	Flight Lieutenant	Salkow, Sidney	Sidney Salkow	tt0034739
1746502939	1922	1922	Forsaking All Others	Forsaking All Others	Chautard, Emile	Emile Chautard	tt0013147
1746581125	1910	1910	The Light in the Window	The Light in the Window		Ray Myers	tt0489196
1746584719	1928	1928	The Cowboy Prince	The Cowboy Prince		Ben F. Wilson	tt0321775
1769976729	1929	1929	The Framing Of The Shrew	The Framing of the Shrew	Gillstrom, Arvid E.	Arvid E. Gillstrom	tt0363619
1746218986	1973	1973	Bummer	Bummer	Castleman, William Allen	William Allen Castleman	tt0068324
1746434377	1991	1991	Hearts of Darkness: A Filmmaker's Apocalypse	Hearts of Darkness: A Filmmaker's Apocalyp:	Bahr , Fax ; Hickenlooper, George	Fax Bahr	tt0102015
1746233994	1966	1966	The Plainsman	The Plainsman	Rich, David Lowell	David Lowell Rich	tt0060842
1746574931	1934	1934	A Woman's Man	A Woman's Man	Ludwig, Edward T.	Edward Ludwig	tt0026005
1746573808	1960	1960	Visit to a Small Planet	Visit to a Small Planet	Taurog, Norman	Norman Taurog	tt0054446
1746503424	1922	1922	Slim Shoulders	Slim Shoulders	Crosland, Alan	Alan Crosland	tt0013609
1746574119	1960	1960	Pay or Die	Pay or Die!	Wilson, Richard	Richard Wilson	tt0054164
1746583105	1940	1940	The Kid from Santa Fe	The Kid from Santa Fe	Johnson, Raymond K.	Bernard B. Ray	tt0032664
1746423279	1993	1993	Dangerous Game	Dangerous Game	Ferrara, Abel	Abel Ferrara	tt0106660
1746513647	1927	1927	American Beauty	The American Beauty	Wallace, Richard	Richard Wallace	tt0017626
1746423813	1940	1940	Lone Star Raiders	Lone Star Raiders	Sherman, George	George Sherman	tt0032724
1746423970	1942	1942	The Lone Star Vigilantes	The Lone Star Vigilantes	Fox, Wallace W.	Wallace Fox	tt0034995
1746508204	1925	1925	Timber Wolf	The Timber Wolf	Van Dyke, William S.	W.S. Van Dyke	tt0016433

1746514545	1915	1915	The Money Master	The Money Master	Fitzmaurice, George	George Fitzmaurice	tt0005764
1746423329	1990	1990	Impulse	Impulse	Locke, Sondra	Sondra Locke	tt0099832
1746578492	1951	1951	Meet Me After the Show	Meet Me After the Show	Sale, Richard	Richard Sale	tt0043795
1746584812	1929	1929	Where East Is East	Where East Is East	Browning, Tod	Tod Browning	tt0020581
1746545150	1940	1940	Youth Will Be Served	Youth Will Be Served	Brower, Otto	Otto Brower	tt0033291
1746525131	1919	1919	The Midnight Stage	The Midnight Stage	Warde, Ernest C.	Ernest C. Warde	tt0010462
1746520054	1924	1924	The Fire Patrol	The Fire Patrol	Stromberg, Hunt	Hunt Stromberg	tt0014902
1746500079	1950	1949	Samson and Delilah	Samson and Delilah	DeMille, Cecil B.	Cecil B. DeMille	tt0041838
1746437031	1940	1940	Safari	Safari	Griffith, Edward H.	Edward H. Griffith	tt0033009
1746563344	1952	1952	The Raiders	The Raiders	Selander, Lesley	Lesley Selander	tt0045067
1746210502	1971	1971	Revenge Is My Destiny	Revenge Is My Destiny	Adler, Joseph	Joseph Adler	tt0067674
1746561567	1956	1956	Naked Gun	Naked Gun	Dew, Edward	Eddie Dew	tt0049539
1746581972	1910	1910	He Met the Champion	He Met the Champion		Gilbert M. 'Broncho Billy' Anderson	tt1644591
1746491881	1944	1944	Silent Partner	Silent Partner	Blair, George	George Blair	tt0037277
1746393310	1985	1985	Better Off Dead...	Better Off Dead...	Holland, Savage Steve	Savage Steve Holland	tt0088794
1746420242	1990	1990	Child's Play 2	Child's Play 2	Lafia, John	John Lafia	tt0099253
1746581681	1929	1929	Wonder of Women	Wonder of Women	Brown, Clarence	Clarence Brown	tt0020603
1746577413	1951	1951	Callaway Went Thataway	Callaway Went Thataway	Panama, Norman; Frank, Melvin	Melvin Frank	tt0043371
1746580393	1939	1939	Star Reporter	Star Reporter	Bretherton, Howard	Howard Bretherton	tt0031975
1746409337	1981	1981	Graduation Day	Graduation Day	Freed, Herb	Herb Freed	tt0082467
1746453803	1940	1940	Three Men from Texas	Three Men from Texas	Selander, Lesley	Lesley Selander	tt0033160
1746554638	1963	1963	Cairo	Cairo	Rilla, Wolf	Wolf Rilla	tt0056894
1746585017	1940	1940	The Mark of Zorro	The Mark of Zorro	Mamoulian, Rouben	Rouben Mamoulian	tt0032762
1746218769	1972	1972	Bonnie's Kids	Bonnie's Kids	Marks, Arthur	Arthur Marks	tt0083677
1746485172	1918	1918	Conquered Hearts	Conquered Hearts	Grandon, Francis J.	Francis J. Grandon	tt0184355
1746503756	1918	1918	The Yellow Dog	The Yellow Dog	Campbell, Colin	Colin Campbell	tt0009841
1746455868	1996	1996	Mr. Wrong	Mr. Wrong	Castle, Nick	Nick Castle	tt0117102
1746574990	1934	1934	Song of the Islands	Song of the Islands		George Tahara	tt6843588
1746532633	1916	1916	Extravagance	Extravagance	King, Burton L.	Burton L. King	tt0157607
1746516288	1921	1921	The Stampede	The Stampede	Ford, Francis	Francis Ford	tt0166816
1746576609	1932	1932	Come On, Tarzan	Come On, Tarzan	James, Alan	Alan James	tt0022773
1746496832	1920	1920	The Web of Deceit	The Web of Deceit	Carewe, Edwin	Edwin Carewe	tt0011842
1746580934	1961	1960	Pepe	Pepe	Sidney, George	George Sidney	tt0054172
1746570333	1934	1934	Imitation of Life	Imitation of Life	Stahl, John M.	John M. Stahl	tt0025301
1746497000	1919	1919	Fruits of Passion	Fruits of Passion	Ridgwell, George	George Ridgwell	tt0162336
1746503478	1920	1920	My Lady's Garter	My Lady's Garter	Tourneur, Maurice	Maurice Tourneur	tt0011493
1746474533	1944	1944	The Navy Way	The Navy Way	Berke, William	William Berke	tt0037121
1746219645	1974	1973	The Outfit	The Outfit	Flynn, John	John Flynn	tt0071960
1745374158	2004	2003	National Lampoon's Gold Diggers	National Lampoon's Gold Diggers	Preisler, Gary	Gary Preisler	tt0376717
1746437340	1942	1942	Texas Trouble Shooters	Texas Trouble Shooters	Luby, S. Roy	S. Roy Luby	tt0158267
1746508993	1925	1925	Blood and Steel	Blood and Steel	McGowan, J. P.	J.P. McGowan	tt0015629
1746453574	1944	1944	Broadway Rhythm	Broadway Rhythm	Del Ruth, Roy	Roy Del Ruth	tt0036673
1746569891	1956	1956	The Flesh Merchant	The Flesh Merchant	Connell, W. Merle	W. Merle Connell	tt0124578
1746500480	1925	1925	Blue Blood	Blue Blood	Dunlap, Scott	Scott R. Dunlap	tt0015631
1746454242	1941	1941	Affectionately Yours	Affectionately Yours	Bacon, Lloyd	Lloyd Bacon	tt0033319
1746582213	1930	1929	General Crack	General Crack	Crosland, Alan	Alan Crosland	tt0020915
1746571331	1935	1935	Stars over Broadway	Stars Over Broadway	Keighley, William	William Keighley	tt0027041
1746567556	1952	1952	Stolen Face	Stolen Face	Fisher, Terence	Terence Fisher	tt0045191
1769969938	1912	1912	Linked Together	Linked Together		C.J. Williams	tt0490607
1746585380	1939	1939	What a Life	What a Life	Reed, Jay Theodore	Theodore Reed	tt0032123
1746582833	1935	1935	Traveling Saleslady	Traveling Saleslady	Enright, Ray	Ray Enright	tt0027133
1746514548	1914	1914	The Circus Man	The Circus Man	Apfel, Oscar C.	Oscar Apfel	tt0003774
1746534801	1922	1922	Thorns and Orange Blossoms	Thorns and Orange Blossoms	Gasnier, Louis J.	Louis J. Gasnier	tt0013671
1746572045	1910	1910	The Deacon's Daughter	The Deacon's Daughter		Sidney Olcott	tt0397385
1746394387	1986	1986	A Fine Mess	A Fine Mess	Edwards, Blake	Blake Edwards	tt0091051
1746464997	1945	1945	The Jade Mask	The Jade Mask	Rosen, Phil	Phil Rosen	tt0037826
1746502719	1922	1922	The Flaming Hour	The Flaming Hour	Sedgwick, Edward	Edward Sedgwick	tt0013133

1746464901	1942	1942	The Forest Rangers	The Forest Rangers	Marshall, George	George Marshall	tt0034749	
1746566363	1957	1957	Affair in Reno	Affair in Reno	Springsteen, R. G.	R.G. Springsteen	tt0050104	
1746515572	1918	1918	The Forbidden Path	The Forbidden Path	Edwards, J. Gordon	J. Gordon Edwards	tt0009087	
1769977748	1981	1980	Hardly Working	Hardly Working	Lewis, Jerry	Jerry Lewis	tt0082501	
1746533001	1916	1916	The Law Decides	The Law Decides	Bertsch, Marguerite; Earle, William F	Marguerite Bertsch	tt0163048	
1746393089	1988	1988	Beetle Juice	Beetlejuice	Burton, Tim	Tim Burton	tt0094721	
1746475915	1943	1943	Tahiti Honey	Tahiti Honey	Auer, John H.	John H. Auer	tt0140592	
**1746580872**	**1935**	**1935**	**Eight Bells**	**Eight Bells**	**Neill, Roy William**	**Roy William Neill**	**tt0026307**	1
1746582873	1935	1935	Charlie Chan in Paris	Charlie Chan in Paris	Seiler, Lewis	Lewis Seiler	tt0026198	
1746520766	1926	1926	Men Women Love	The Men Women Love	Dierker, Hugh		tt1690533	
1746512598	1927	1927	The Broken Violin	The Broken Violin	Micheaux, Oscar	Oscar Micheaux	tt0017708	
1746508039	1949	1949	A Dangerous Profession	A Dangerous Profession	Tetzlaff, Ted	Ted Tetzlaff	tt0041278	
1746465034	1942	1942	One Thrilling Night	One Thrilling Night	Beaudine, William	William Beaudine	tt0035152	
1746516334	1921	1921	Making the Grade	Making the Grade	Butler, Fred J.	Fred J. Butler	tt0133082	
1746578588	1963	1963	Red Runs the River	Red Runs the River	Stenholm, Katherine	Katherine Stenholm	tt0435318	
1746583486	1951	1951	Strangers on a Train	Strangers on a Train	Hitchcock, Alfred	Alfred Hitchcock	tt0044079	
1769971976	1969	1969	The Erotic Circus	The Erotic Circus	Sullivan, Ron	Henri Pachard	tt0217442	
1746561415	1932	1932	No One Man	No One Man	Corrigan, Lloyd	Lloyd Corrigan	tt0023279	
1746584199	1936	1936	Laughing Irish Eyes	Laughing Irish Eyes	Santley, Joseph	Joseph Santley	tt0027871	
1746572013	1931	1931	His Woman	His Woman	Sloman, Edward	Edward Sloman	tt0021963	
1769970605	1966	1966	Mondo Freudo	Mondo Freudo	Frost, R. L.	Lee Frost	tt0063321	
1746569796	1958	1958	Let's Rock	Let's Rock	Foster, Harry	Harry Foster	tt0051856	
1746567330	1952	1952	The Rose Bowl Story	The Rose Bowl Story	Beaudine, William	William Beaudine	tt0045104	
1746532965	1927	1927	Black Tears	Black Tears	Gorman, John	John Gorman	tt0017680	
1746182716	1910	1910	The Call	The Call	Griffith, D. W.	D.W. Griffith	tt0001144	
1746581914	1928	1928	Silent Trail	Silent Trail	McGowan, J. P.	J.P. McGowan	tt0019384	
1745309416	2005	2005	Charlie and the Chocolate Factory	Charlie and the Chocolate Factory	Burton, Tim	Tim Burton	tt0367594	
1769971181	1969	1969	The Ramrodder	The Ramrodder	Guylder, Van	Ed Forsyth	tt0127207	
1746524615	1923	1923	Nobody's Bride	Nobody's Bride	Blaché, Herbert	Herbert Blaché	tt0014311	
1746495806	1919	1919	Peg O' My Heart	Peg o' My Heart	Mille, William C. de	William C. de Mille	tt0338339	
1746581576	1961	1961	Man-Trap	Man Trap	O'Brien, Edmond	Edmond O'Brien	tt0055134	
1746543067	1915	1915	The House of a Thousand Candles	The House of a Thousand Candles	Heffron, Thomas N.	Thomas N. Heffron	tt0005517	
1746500149	1925	1925	Flattery	Flattery	Forman, Tom	Tom Forman	tt0015822	
1746211092	1973	1973	Executive Action	Executive Action	Miller, David	David Miller	tt0070046	
1746573950	1960	1960	The Alamo	The Alamo	Wayne, John	John Wayne	tt0053580	
1746574455	1933	1933	One Man's Journey	One Man's Journey	Robertson, John	John S. Robertson	tt0024411	
1746525017	1921	1921	The Smart Sex	The Smart Sex	Granville, Fred Leroy	Fred LeRoy Granville	tt0012703	
1746218765	1975	1975	Dr. Minx	Dr. Minx	Avedis, Hikmet	Howard Avedis	tt0072905	
1746553466	1964	1964	Diary of a Bachelor	Diary of a Bachelor	Howard, Sandy	Sandy Howard	tt0058012	
1745282695	2007	2006	The Last Winter	The Last Winter	Fessenden, Larry	Larry Fessenden	tt0454864	
1746573782	1958	1958	The Return of Dracula	The Return of Dracula	Landres, Paul	Paul Landres	tt0052131	
1746582296	1930	1930	Big Boy	Big Boy	Crosland, Alan	Alan Crosland	tt0020683	
1745341770	2001	2001	The Last Castle	The Last Castle	Lurie, Rod	Rod Lurie	tt0272020	
1746516143	1920	1920	The Blooming Angel	The Blooming Angel	Schertzinger, Victor L.	Victor Schertzinger	tt0122407	
1746574246	1959	1959	A Private's Affair	A Private's Affair	Walsh, Raoul	Raoul Walsh	tt0053192	
1746465190	1944	1944	The Heavenly Body	The Heavenly Body	Hall, Alexander	Alexander Hall	tt0035980	
1746554025	1964	1964	Dr. Strangelove or: How I Learned to Stop Worry	Dr. Strangelove or: How I Learned to Stop W	Kubrick, Stanley	Stanley Kubrick	tt0057012	
1746424366	1941	1941	Law of the Tropics	Law of the Tropics	Enright, Ray	Ray Enright	tt0033821	
1746514216	1910	1910	The Missing Bridegroom	The Missing Bridegroom			tt0001324	
1746584269	1938	1938	Swiss Miss	Swiss Miss	Blystone, John G.	John G. Blystone	tt0030824	
1746577263	1962	1962	Tender Is the Night	Tender Is the Night	King, Henry	Henry King	tt0056566	
1746455804	1996	1996	Set It Off	Set It Off	Gray, F. Gary	F. Gary Gray	tt0117603	
1745303186	2006	2006	Basic Instinct 2	Basic Instinct 2	Caton-Jones, Michael	Michael Caton	tt0430912	
1746581191	1962	1962	Incident in an Alley	Incident in an Alley	Cahn, Edward L.	Edward L. Cahn	tt0056098	
1746498478	1920	1920	The Daughter of Dawn	The Daughter of Dawn	Myles, Norbert	Norbert A. Myles	tt0191066	
1746570397	1934	1934	The Meanest Gal in Town	The Meanest Gal in Town	Mack, Russell	Russell Mack	tt0025481	
1746567611	1933	1933	Today We Live	Today We Live	Hawks, Howard	Howard Hawks	tt0024675	

1746235133	1967	1967	It's a Bikini World	It's a Bikini World	Rothman, Stephanie	Stephanie Rothman	tt0061827
1746410693	1986	1986	The Whoopee Boys	The Whoopee Boys	Byrum, John	John Byrum	tt0092210
1746454380	1945	1945	Between Two Women	Between Two Women	Goldbeck, Willis	Willis Goldbeck	tt0036640
1746511805	1923	1923	The Barefoot Boy	The Barefoot Boy	Kirkland, David	David Kirkland	tt0013860
1746577381	1960	1960	Spartacus	Spartacus	Kubrick, Stanley	Stanley Kubrick	tt0054331
1769970637	1968	1968	The Wild Females	The Wild Females	Samoya, Carlos	Carlos Samoya	tt0067986
1746421318	1982	1982	Blade Runner	Blade Runner	Scott, Ridley	Ridley Scott	tt0083658
1746580924	1951	1950	The Sun Sets at Dawn	The Sun Sets at Dawn	Sloane, Paul H.	Paul Sloane	tt0044088
1746574650	1936	1936	Road Gang	Road Gang	King, Louis	Louis King	tt0028190
1746583119	1936	1936	Desert Gold	Desert Gold	Hogan, James	James P. Hogan	tt0027511
1746577941	1937	1937	Hotel Haywire	Hotel Haywire	Archainbaud, George	George Archainbaud	tt0029024
1746554185	1963	1963	Promises! Promises!	Promises..... Promises!	Donovan, King	King Donovan	tt0057428
1746501127	1917	1917	Strife	Strife	Hillyer, Lambert	Lambert Hillyer	tt0182466
1746569655	1957	1957	April Love	April Love	Levin, Henry	Henry Levin	tt0050135
1746566374	1953	1952	The Jazz Singer	The Jazz Singer	Curtiz, Michael	Michael Curtiz	tt0044765
1746234815	1979	1979	The Warriors	The Warriors	Hill, Walter	Walter Hill	tt0080120
1746574686	1934	1934	Carolina	Carolina	King, Henry	Henry King	tt0024956
1746419624	1988	1987	World Gone Wild	World Gone Wild	Katzin, Lee H.	Lee H. Katzin	tt0096465
1746492051	1947	1947	The Devil Thumbs a Ride	The Devil Thumbs a Ride	Feist, Felix	Felix E. Feist	tt0039317
1746465272	1947	1947	That's My Gal	That's My Gal	Blair, George	George Blair	tt0039894
1746571708	1932	1932	Union Depot	Union Depot	Green, Alfred E.	Alfred E. Green	tt0023642
1745302687	2005	2005	Hide and Seek	Hide and Seek	Polson, John	John Polson	tt0382077
1746580453	1937	1937	Where Trails Divide	Where Trails Divide	Bradbury, Robert N.	Robert N. Bradbury	tt0029766
1746210488	1970	1969	Wild, Free and Hungry	Wild, Free & Hungry	Edwards, H. P.	Paul Hunt	tt0134215
1746514673	1914	1913	A Lady of Quality	A Lady of Quality		J. Searle Dawley	tt0003048
1745374746	2010	2010	The Town	The Town	Affleck, Ben	Ben Affleck	tt0840361
1746216740	1969	1969	Like Mother Like Daughter	Like Mother Like Daughter	O'Neil, Robert V.	Robert Vincent O'Neill	tt0151338
1746532817	1923	1923	Racing Hearts	Racing Hearts	Powell, Paul	Paul Powell	tt0014389
1746520898	1950	1949	Dear Wife	Dear Wife	Haydn, Richard	Richard Haydn	tt0041286
1746486844	1919	1919	Widow by Proxy	Widow by Proxy	Edward, Walter	Walter Edwards	tt0010891
1746465372	1944	1944	The Missing Juror	The Missing Juror	Boetticher, Oscar	Budd Boetticher	tt0037078
1746500075	1924	1924	The Bedroom Window	The Bedroom Window	de Mille, William	William C. de Mille	tt0014706
1745340774	2003	2003	Marci X	Marci X	Benjamin, Richard	Richard Benjamin	tt0266747
1746583103	1935	1935	The Littlest Rebel	The Littlest Rebel	Butler, David	David Butler	tt0026641
1746562221	1953	1953	Jennifer	Jennifer	Newton, Joel; Girard, Bernard	Joel Newton	tt0045931
1746507870	1924	1924	The House of Youth	The House of Youth	Ince, Ralph	Ralph Ince	tt0135459
1746566212	1958	1958	Machine-Gun Kelly	Machine Gun Kelly	Corman, Roger	Roger Corman	tt0051887
1746210712	1971	1971	Evel Knievel	Evel Knievel	Chomsky, Marvin	Marvin J. Chomsky	tt0067069
1746574602	1933	1933	Life in the Raw	Life in the Raw	King, Louis	Louis King	tt0024256
1746583796	1929	1929	The Battle of Paris	Battle of Paris	Florey, Robert	Robert Florey	tt0019679
1746514488	1923	1923	Riders of the Range	Riders of the Range	Thayer, Otis B.	Otis Thayer	tt0156021
1746585162	1928	1928	Across the Atlantic	Across the Atlantic	Bretherton, Howard	Howard Bretherton	tt0018616
1746570985	1934	1934	Cleopatra	Cleopatra	DeMille, Cecil B.	Cecil B. DeMille	tt0024991
1746520891	1949	1949	Daughter of the Jungle	Daughter of the Jungle	Blair, George	George Blair	tt0041281
1746585351	1929	1929	Laughing at Death	Laughing at Death	Fox, Wallace W.	Wallace Fox	tt0020083
1746422276	1999	1999	Man on the Moon	Man on the Moon	Forman, Milos	Milos Forman	tt0125664
1745359781	2008	2008	Righteous Kill	Righteous Kill	Avnet, Jon	Jon Avnet	tt1034331
1746582472	1935	1935	Private Worlds	Private Worlds	La Cava, Gregory	Gregory La Cava	tt0026893
1746585459	1940	1940	Down Argentine Way	Down Argentine Way	Cummings, Irving	Irving Cummings	tt0032410
1746564528	1951	1951	The Last Outpost	The Last Outpost	Foster, Lewis R.	Lewis R. Foster	tt0043725
1746582754	1939	1939	Let Us Live	Let Us Live	Brahm, John	John Brahm	tt0031566
1746498491	1918	1918	Hobbs in a Hurry	Hobbs in a Hurry	King, Henry	Henry King	tt0010260
1746585091	1930	1930	Anybody's Woman	Anybody's Woman	Arzner, Dorothy	Dorothy Arzner	tt0020645
1746423853	1940	1940	Pop Always Pays	Pop Always Pays	Goodwins, Leslie	Leslie Goodwins	tt0032925
1746583558	1930	1930	Just Imagine	Just Imagine	Butler, David	David Butler	tt0021016
1746504557	1918	1918	A Soul in Trust	A Soul in Trust	Hamilton, G. P.	Gilbert P. Hamilton	tt0009636
1746577377	1960	1960	Under Ten Flags	Under Ten Flags	Coletti, Duilio	Duilio Coletti	tt0054328

1746496996	1921	1921	The Inside of the Cup	The Inside of the Cup	Capellani, Albert	Albert Capellani	tt0012318
1746186170	1968	1968	Come Back Baby	Come Back Baby	Greene, David Allen	David Allen Greene	tt0181394
1769971937	1969	1969	Her Odd Tastes	Her Odd Tastes		Donald A. Davis	tt0140144
1746419166	1994	1994	Trapped in Paradise	Trapped in Paradise	Gallo, George	George Gallo	tt0111477
1746580407	1935	1935	Born to Gamble	Born to Gamble	Rosen, Phil	Phil Rosen	tt0026131
1745359797	2004	2003	Greendale	Greendale	Shakey, Bernard	Neil Young	tt0379307
1746504365	1920	1920	Romance	Romance	Withey, Chet	Chester Withey	tt0011639
1746394439	1986	1986	Playing for Keeps	Playing for Keeps	Weinstein, Bob; Weinstein, Harvey	Bob Weinstein	tt0091767
1746512876	1916	1916	Bluff	Bluff	Berger, Rea	Rae Berger	tt0157416
1746454927	1946	1946	Tumbleweed Trail	Tumbleweed Trail	Tansey, Robert Emmett	Robert Emmett Tansey	tt0039052
1746509506	1924	1924	Galloping Gallagher	Galloping Gallagher	Rogell, Albert	Albert S. Rogell	tt0128228
1746569811	1957	1957	The Wings of Eagles	The Wings of Eagles	Ford, John	John Ford	tt0051198
1746585189	1929	1929	Blue Skies	Blue Skies	Werker, Alfred L.	Alfred L. Werker	tt0019705
1746454284	1943	1943	The Mystery of the 13th Guest	The Mystery of the 13th Guest	Beaudine, William	William Beaudine	tt0036189
1746553683	1964	1964	The Americanization of Emily	The Americanization of Emily	Hiller, Arthur	Arthur Hiller	tt0057840
1746583250	1931	1931	The Hurricane Horseman	Hurricane Horseman	Schaeffer, Armand	Armand Schaefer	tt0021982
1746508964	1926	1926	The Golden Web	The Golden Web	Lang, Walter	Walter Lang	tt0016929
1746507748	1949	1949	Look for the Silver Lining	Look for the Silver Lining	Butler, David	David Butler	tt0041599
1746408920	1986	1986	Legal Eagles	Legal Eagles	Reitman, Ivan	Ivan Reitman	tt0091396
1746496573	1918	1918	Denny from Ireland	Denny from Ireland	Clifford, W. H.	William H. Clifford	tt0161491
1746208663	1966	1966	My Brother's Wife	My Brother's Wife	Wishman, Doris	Doris Wishman	tt0138636
1746582642	1938	1938	Code of the Rangers	Code of the Rangers	Newfield, Sam	Sam Newfield	tt0030001
1746574538	1934	1934	Are We Civilized?	Are We Civilized?		Edwin Carewe	tt0024834
1746201190	1970	1970	Darling Lili	Darling Lili	Edwards, Blake	Blake Edwards	tt0065611
1746394505	1984	1984	Tank	Tank	Chomsky, Marvin J.	Marvin J. Chomsky	tt0088224
1745302738	2007	2007	Bratz	Bratz	McNamara, Sean	Sean McNamara	tt0804452
1746408858	1986	1986	Just Between Friends	Just Between Friends	Burns, Allan	Allan Burns	tt0091310
1746232972	1966	1966	Made in Paris	Made in Paris	Sagal, Boris	Boris Sagal	tt0060646
1746574991	1935	1935	Suicide Squad	Suicide Squad	Johnson, Raymond K.	Bernard B. Ray	tt0027057
1746454150	1995	1995	Nixon	Nixon	Stone, Oliver	Oliver Stone	tt0113987
1746465239	1947	1947	The Hucksters	The Hucksters	Conway, Jack	Jack Conway	tt0039477
1746503765	1921	1921	Hearts o' the Range	Hearts o' the Range	Morante, Milburn	Milburn Morante	tt0134711
1745294009	2007	2007	Why Did I Get Married?	Why Did I Get Married?	Perry, Tyler	Tyler Perry	tt0906108
1769970538	1966	1966	Suburbia Confidential	Suburbia Confidential		Stephen C. Apostolof	tt0128640
1745340794	2002	2002	Juwanna Mann	Juwanna Mann	Vaughan, Jesse	Jesse Vaughan	tt0247444
1745342520	2010	2009	Middle Men	Middle Men	Gallo, George	George Gallo	tt1251757
1745282679	2007	2006	First Snow	First Snow	Fergus, Mark	Mark Fergus	tt0432289
1745293443	2007	2007	Halloween	Halloween	Zombie, Rob	Rob Zombie	tt0373883
1746584968	1940	1940	Son of Ingagi	Son of Ingagi	Kahn, Richard C.	Richard C. Kahn	tt0033075
1746198502	1968	1968	Buckskin	Buckskin	Moore, Michael	Michael D. Moore	tt0062764
1746508671	1949	1949	The Girl from Jones Beach	The Girl from Jones Beach	Godfrey, Peter	Peter Godfrey	tt0041415
1746580338	1938	1938	Boy Meets Girl	Boy Meets Girl	Bacon, Lloyd	Lloyd Bacon	tt0029940
1746465258	1943	1943	Something to Shout About	Something to Shout About	Ratoff, Gregory	Gregory Ratoff	tt0036375
1746491711	1948	1948	April Showers	April Showers	Kern, James V.	James V. Kern	tt0040107
1746520322	1926	1925	The Ace of Clubs	Ace of Clubs	McGowan, J. P.	J.P. McGowan	tt0016573
1746508110	1927	1927	Perch of the Devil	Perch of the Devil	Baggot, King	King Baggot	tt0018256
1745302333	2005	2005	Little Manhattan	Little Manhattan	Levin, Mark	Mark Levin	tt0412922
1746572246	1931	1931	Up for Murder	Up for Murder	Bell, Monta	Monta Bell	tt0021868
1746583083	1939	1939	Undercover Agent	Undercover Agent	Bretherton, Howard	Howard Bretherton	tt0032076
1746455613	1998	1998	Bulworth	Bulworth	Beatty, Warren	Warren Beatty	tt0118798
1746554082	1964	1964	Surf Party	Surf Party	Dexter, Maury	Maury Dexter	tt0058626
1746512656	1915	1915	The Flying Twins	The Flying Twins		Jack Harvey	tt0005335
1746571292	1933	1933	Mr. Broadway	Mr. Broadway	Walker, Johnnie	Johnnie Walker	tt0024356
1746513098	1927	1927	Home Struck	Home Struck	Ince, Ralph	Ralph Ince	tt0135450
1746394230	1983	1983	The Being	The Being	Kong, Jackie	Jackie Kong	tt0085224
1746217068	1965	1965	A Patch of Blue	A Patch of Blue	Green, Guy	Guy Green	tt0059573
1746555581	1956	1956	Toward the Unknown	Toward the Unknown	LeRoy, Mervyn	Mervyn LeRoy	tt0049870

1746393685	1982	1982	Silent Rage	Silent Rage	Miller, Michael	Michael Miller	tt0084684
1746501167	1910	1910	A Touching Affair	A Touching Affair	Morris, Sam	Sam Morris	tt1570533
1769970450	1965	1965	Flesh and Lace	Flesh and Lace	Sarno, Joe	Joseph W. Sarno	tt0058102
1746514199	1910	1910	Love in Quarantine	Love in Quarantine	Powell, Frank	Frank Powell	tt0362828
1746567554	1952	1951	Westward the Women	Westward the Women	Wellman, William A.	William A. Wellman	tt0044205
1746493501	1910	1910	Sisters	Sisters		Bannister Merwin	tt0001397
1746465376	1947	1947	It's a Joke, Son!	It's a Joke, Son!	Stoloff, Ben	Benjamin Stoloff	tt0039503
1746585084	1929	1929	Night Club	Night Club	Florey, Robert	Robert Florey	tt0019210
1746493366	1910	1910	The Usurper	The Usurer		D.W. Griffith	tt0001439
1746524467	1916	1916	The Net	The Net	Platt, George Foster	George Foster Platt	tt0007120
1746183365	1910	1910	The Call to Arms	The Call to Arms	Griffith, D. W.	D.W. Griffith	tt0001143
1746567758	1933	1933	Scandal	Scandal		Jack Goldberg	tt0135643
1746583600	1929	1929	The Mighty	The Mighty	Cromwell, John	John Cromwell	tt0021147
1746577354	1960	1960	All the Fine Young Cannibals	All the Fine Young Cannibals	Anderson, Michael	Michael Anderson	tt0053582
1746574449	1961	1961	The Right Approach	The Right Approach	Butler, David	David Butler	tt0055373
1746561391	1932	1932	Amateur Daddy	Amateur Daddy	Blystone, John	John G. Blystone	tt0022625
1745302994	2006	2005	Hard Candy	Hard Candy	Slade, David	David Slade	tt0424136
1746570371	1952	1952	The Atomic City	The Atomic City	Hopper, Jerry	Jerry Hopper	tt0044382
1746500649	1949	1950	The Blonde Bandit	The Blonde Bandit	Keller, Harry	Harry Keller	tt0042261
1746410372	1982	1982	Eating Raoul	Eating Raoul	Bartel, Paul	Paul Bartel	tt0083869
1745293145	2007	2007	The Girl Next Door	The Girl Next Door	Wilson, Gregory M.	Gregory Wilson	tt0830558
1746581490	1928	1928	13 Washington Square	13 Washington Square	Brown, Melville W.	Melville W. Brown	tt0018611
1745358085	2004	2004	Spider-Man 2	Spider Man 2	Raimi, Sam	Sam Raimi	tt0316654
1746576191	1932	1932	Whistlin' Dan	Whistlin' Dan	Rosen, Phil	Phil Rosen	tt0023692
1746562129	1953	1953	Girls in the Night	Girls in the Night	Arnold, Jack	Jack Arnold	tt0045818
1746497078	1918	1918	His Robe of Honor	His Robe of Honor	Ingram, Rex	Rex Ingram	tt0009184
1746568147	1933	1933	Chance at Heaven	Chance at Heaven	Seiter, William	William A. Seiter	tt0023880
1746410438	1986	1986	Children of a Lesser God	Children of a Lesser God	Haines, Randa	Randa Haines	tt0090830
1746454494	1942	1942	Strictly in the Groove	Strictly in the Groove	Keays, Vernon	Vernon Keays	tt0035389
1746464919	1944	1944	Music in Manhattan	Music in Manhattan	Auer, John H.	John H. Auer	tt0037105
1746514688	1915	1915	The Dictator	The Dictator	Eagle, Oscar	Oscar Eagle	tt0005196
1746410460	1986	1986	American Anthem	American Anthem	Magnoli, Albert	Albert Magnoli	tt0090631
1746530740	1949	1949	Pinky	Pinky	Kazan, Elia	Elia Kazan	tt0041746
1746423009	2000	2000	Dracula 2000	Dracula 2000	Lussier, Patrick	Patrick Lussier	tt0219653
1746409092	1992	1992	Of Mice and Men	Of Mice and Men	Sinise, Gary	Gary Sinise	tt0105046
1746565919	1955	1955	Ten Wanted Men	Ten Wanted Men	Humberstone, Bruce	H. Bruce Humberstone	tt0048703
1746584725	1929	1929	The Awful Truth	The Awful Truth	Neilan, Marshall	Marshall Neilan	tt0019666
1746553385	1964	1964	Roustabout	Roustabout	Rich, John	John Rich	tt0058534
1746523560	1916	1916	The Secret of the Swamp	The Secret of the Swamp	Reynolds, Lynn	Lynn Reynolds	tt0007318
1746577258	1951	1951	Wanted Dead or Alive	Wanted: Dead or Alive	Carr, Thomas	Thomas Carr	tt0162760
1746502388	1915	1915	The Vow	The Vow	Taylor, S. E. V.	Stanner E.V. Taylor	tt0006235
1746235220	1980	1980	Flash Gordon	Flash Gordon	Hodges, Mike	Mike Hodges	tt0080745
1746566338	1954	1954	The Law vs. Billy the Kid	The Law vs. Billy the Kid	Castle, William	William Castle	tt0047163
1769969544	1965	1965	The Sexploiters	The Sexploiters	Ruban, Al C.	Al Ruban	tt0148746
1746574205	1959	1959	Battle Flame	Battle Flame	Springsteen, R. G.	R.G. Springsteen	tt0052605
1746581233	1960	1960	Seven Ways from Sundown	Seven Ways from Sundown	Keller, Harry; Sherman, George	Harry Keller	tt0054296
1746409103	1984	1984	Bachelor Party	Bachelor Party	Israel, Neal	Neal Israel	tt0086927
1746453540	1944	1944	The Uninvited	The Uninvited	Allen, Lewis	Lewis Allen	tt0037415
1746520253	1926	1926	The Great K & A Train Robbery	The Great K & A Train Robbery	Seiler, Lewis	Lewis Seiler	tt0016939
1746393329	1984	1984	Choose Me	Choose Me	Rudolph, Alan	Alan Rudolph	tt0087054
1745373162	2004	2004	The Village	The Village	Shyamalan, M. Night	M. Night Shyamalan	tt0368447
1746561335	1956	1956	Dance with Me, Henry	Dance with Me, Henry	Barton, Charles	Charles Barton	tt0049120
1746513838	1917	1917	Beware of Strangers	Beware of Strangers	Campbell, Colin	Colin Campbell	tt0008883
1746421960	1997	1997	Latin Boys Go to Hell	Latin Boys Go to Hell	Troyano, Ela	Ela Troyano	tt0124777
1746465120	1944	1944	Heavenly Days	Heavenly Days	Estabrook, Howard	Howard Estabrook	tt0036902
1746410896	1991	1991	Nothing But Trouble	Nothing But Trouble	Aykroyd, Dan	Dan Aykroyd	tt0102558
1746563795	1951	1951	The Mark of the Renegade	The Mark of the Renegade	Fregonese, Hugo	Hugo Fregonese	tt0043787

1746436071	1941	1941	Golden Hoofs	Golden Hoofs	Shores, Lynn	Lynn Shores	tt0033670
1746219377	1972	1972	Elvis on Tour	Elvis on Tour	Adidge, Pierre; Abel, Robert	Robert Abel	tt0068537
1746524570	1915	1915	The Rosary	The Rosary	Campbell, Colin	Colin Campbell	tt0005979
1746520342	1926	1926	Monte Carlo	Monte Carlo	Cabanne, Christy	Christy Cabanne	tt0017166
1746582529	1938	1938	Men Are Such Fools	Men Are Such Fools	Berkeley, Busby	Busby Berkeley	tt0030433
1746454886	1999	1999	Girl, Interrupted	Girl, Interrupted	Mangold, James	James Mangold	tt0172493
1746486482	1919	1919	The Sheriff's Son	The Sheriff's Son	Schertzinger, Victor L.	Victor Schertzinger	tt0010691
1745340964	2001	2001	Porn Star: The Legend of Ron Jeremy	Porn Star: The Legend of Ron Jeremy	Gill, Scott J.	Scott J. Gill	tt0282856
1746525194	1921	1921	Shams of Society	Shams of Society	Walsh, Thomas B.	Thomas B. Walsh	tt0012673
1746581633	1929	1929	Clear the Decks	Clear the Decks	Henabery, Joseph E.	Joseph Henabery	tt0019773
1746503313	1918	1918	Everywoman's Husband	Everywoman's Husband	Hamilton, Gilbert P.	Gilbert P. Hamilton	tt0009042
1746583268	1951	1950	Revenue Agent	Revenue Agent	Landers, Lew	Lew Landers	tt0042887
1746393272	1987	1987	The Secret of My Success	The Secret of My Succe$s	Ross, Herbert	Herbert Ross	tt0093936
1746209441	1966	1966	A Man Called Adam	A Man Called Adam	Penn, Leo	Leo Penn	tt0060660
1745339943	2002	2002	Reign of Fire	Reign of Fire	Bowman, Rob	Rob Bowman	tt0253556
1746566264	1956	1956	Red Sundown	Red Sundown	Arnold, Jack	Jack Arnold	tt0049667
1746585061	1929	1929	The Younger Generation	The Younger Generation	Capra, Frank R.	Frank Capra	tt0020613
1746509316	1924	1924	A Desperate Adventure	A Desperate Adventure	McGowan, J. P.	J.P. McGowan	tt0014845
1746464996	1947	1947	Six-Gun Serenade	Six Gun Serenade	Beebe, Ford	Ford Beebe	tt0163244
1746421607	1989	1989	Police Academy 6: City Under Siege	Police Academy 6: City Under Siege	Bonerz, Peter	Peter Bonerz	tt0098105
1746582188	1951	1951	The Lady from Texas	The Lady from Texas	Pevney, Joseph	Joseph Pevney	tt0043723
1746569972	1957	1957	Mister Cory	Mister Cory	Edwards, Blake	Blake Edwards	tt0050710
1746465385	1945	1945	The Woman in Green	The Woman in Green	Neill, Roy William	Roy William Neill	tt0038259
1746492707	1948	1947	Design for Death	Design for Death			tt0040285
1745309716	2006	2006	Waist Deep	Waist Deep	Hall, Vondie Curtis	Vondie Curtis	tt0456020
1746524617	1927	1927	Broadway After Midnight	Broadway After Midnight	Windermere, Fred	Fred Windemere	tt0017700
1746500040	1948	1948	Close-Up	Close Up	Donohue, Jack	Jack Donohue	tt0040237
1746525046	1920	1920	Number 99	Number 99	Warde, Ernest C.	Ernest C. Warde	tt0196805
1745360018	2001	2001	Haiku Tunnel	Haiku Tunnel	Kornbluth, Jacob ; Kornbluth, Josh	Jacob Kornbluth	tt0273253
1745341262	2002	2002	Dahmer	Dahmer	Jacobson, David	David Jacobson	tt0285728
1746186299	1965	1965	Vinyl	Vinyl	Warhol, Andy	Andy Warhol	tt0059880
1746491874	1946	1946	Janie Gets Married	Janie Gets Married	Sherman, Vincent	Vincent Sherman	tt0038652
1746218898	1972	1972	Lenny Bruce Without Tears	Lenny Bruce Without Tears	Baker, Fred	Fred Baker	tt0151327
1746475768	1944	1944	When Strangers Marry	When Strangers Marry	Castle, William	William Castle	tt0037458
1745342055	2002	2002	The Salton Sea	The Salton Sea	Caruso, D. J.	D.J. Caruso	tt0235737
1746581515	1928	1928	Speedy	Speedy	Wilde, Ted	Ted Wilde	tt0019412
1746571361	1933	1932	The Bitter Tea of General Yen	The Bitter Tea of General Yen	Capra, Frank R.	Frank Capra	tt0023814
1769970418	1965	1965	Watch the Birdie	Watch the Birdie	Alti, Giulio	Giulio Alti	tt0260531
1746584781	1930	1930	Rogue of the Rio Grande	Rogue of the Rio Grande	Bennett, Spencer Gordon	Spencer Gordon Bennet	tt0021308
1746408665	1987	1987	Overboard	Overboard	Marshall, Garry	Garry Marshall	tt0093693
1746454934	1941	1941	Blues in the Night	Blues in the Night	Litvak, Anatole	Anatole Litvak	tt0033409
1746580757	1935	1935	The Raven	The Raven	Friedlander, Louis	Lew Landers	tt0026912
1746570970	1934	1934	The Man from Hell	The Man from Hell	Collins, Lew	Lewis D. Collins	tt0025454
1746578448	1959	1959	Speed Crazy	Speed Crazy	Hole, William	William J. Hole Jr.	tt0053300
1746513622	1927	1927	The Final Extra	The Final Extra	Hogan, James P.	James P. Hogan	tt0017882
1746581363	1950	1950	Outcast of Black Mesa	Outcasts of Black Mesa	Nazarro, Ray	Ray Nazarro	tt0042821
1746574557	1935	1935	Hong Kong Nights	Hong Kong Nights	Hopper, E. Mason	E. Mason Hopper	tt0026489
1746436680	1941	1941	Singapore Woman	Singapore Woman	Negulesco, Jean	Jean Negulesco	tt0034191
1746580309	1939	1939	The Great Victor Herbert	The Great Victor Herbert	Stone, Andrew L.	Andrew L. Stone	tt0031393
1746219545	1974	1974	Golden Needles	Golden Needles	Clouse, Robert	Robert Clouse	tt0071568
1746497474	1920	1920	Young Mrs. Winthrop	Young Mrs. Winthrop	Edwards, Walter	Walter Edwards	tt0011892
1746209091	1968	1968	Girl in Gold Boots	Girl in Gold Boots	Mikels, Ted V.	Ted V. Mikels	tt0174685
1746488043	1917	1917	On Trial	On Trial	Young, James	James Young	tt0008383
1746580898	1937	1937	Youth on Parole	Youth on Parole	Rosen, Phil	Phil Rosen	tt0029812
1746435320	1998	1998	Hush	Hush	Darby, Jonathan	Jonathan Darby	tt0118744
1746569869	1955	1955	Women's Prison	Women's Prison	Seiler, Lewis	Lewis Seiler	tt0048811
1746577118	1959	1959	The Giant Gila Monster	The Giant Gila Monster	Kellogg, Ray	Ray Kellogg	tt0052846

1746424375	1940	1940	Glamour for Sale	Glamour for Sale	Lederman, D. Ross	D. Ross Lederman	tt0032534
1746435028	1998	1998	The Impostors	The Impostors	Tucci, Stanley	Stanley Tucci	tt0120823
1746520682	1925	1925	Thundering Through	Thundering Through	Bain, Fred	Fred Bain	tt0016431
1746566828	1952	1952	The San Francisco Story	The San Francisco Story	Parrish, Robert	Robert Parrish	tt0045119
1746235286	1976	1976	Family Plot	Family Plot	Hitchcock, Alfred	Alfred Hitchcock	tt0074512
1746570005	1958	1958	The Mugger	The Mugger	Berke, William	William Berke	tt0151666
1745272520	2000	2000	Scream 3	Scream 3	Craven, Wes	Wes Craven	tt0134084
1745309446	2006	2006	Hoot	Hoot	Shriner, Wil	Wil Shriner	tt0453494
1746577747	1937	1937	Partners in Crime	Partners in Crime	Murphy, Ralph	Ralph Murphy	tt0029380
1746464894	1945	1945	Enemy of the Law	Enemy of the Law	Fraser, Harry	Harry L. Fraser	tt0037673
1746569903	1954	1954	The Glenn Miller Story	The Glenn Miller Story	Mann, Anthony	Anthony Mann	tt0047030
1746577912	1936	1936	The Walking Dead	The Walking Dead	Curtiz, Michael	Michael Curtiz	tt0028478
1746583498	1929	1929	The Talk of Hollywood	The Talk of Hollywood	Sandrich, Mark	Mark Sandrich	tt0021449
1746520848	1949	1949	In the Good Old Summertime	In the Good Old Summertime	Leonard, Robert Z.	Robert Z. Leonard	tt0041507
1746578611	1951	1951	Montana Desperado	Montana Desperado	Fox, Wallace W.	Wallace Fox	tt0043817
1745302344	2006	2006	The Pursuit of Happyness	The Pursuit of Happyness	Muccino, Gabriele	Gabriele Muccino	tt0454921
1746583286	1930	1930	The Case of Sergeant Grischa	The Case of Sergeant Grischa	Brenon, Herbert	Herbert Brenon	tt0020744
1746525177	1919	1919	More Deadly Than the Male	More Deadly Than the Male	Vignola, Robert G.	Robert G. Vignola	tt0010483
1746583055	1935	1935	Fighting Pioneers	Fighting Pioneers	Fraser, Harry	Harry L. Fraser	tt0026354
1746507733	1925	1925	Ridin' Comet	The Riding Comet	Wilson, Ben	Ben F. Wilson	tt0125999
1746394649	1984	1984	The Muppets Take Manhattan	The Muppets Take Manhattan	Oz, Frank	Frank Oz	tt0087755
1746498488	1919	1919	That's Good	That's Good	Franklin, Harry L.	Harry L. Franklin	tt0194443
1746574273	1961	1961	After Mein Kampf	After Mein Kampf	Porter, Ralph	Ralph Porter	tt0054606
1746524513	1913	1913	Sapho	Sapho	Henderson, Lucius J.	Lucius Henderson	tt0003351
1745374147	2004	2004	Ella Enchanted	Ella Enchanted	O'Haver, Tommy	Tommy O'Haver	tt0327679
1746580587	1938	1938	Under Western Stars	Under Western Stars	Kane, Joe	Joseph Kane	tt0030910
1746465269	1942	1942	Not a Ladies' Man	Not a Ladies' Man	Landers, Lew	Lew Landers	tt0035137
1745359834	2010	2010	Step Up 3D	Step Up 3D	Chu, Jon M.	Jon M. Chu	tt1193631
1746574616	1936	1936	Mad Holiday	Mad Holiday	Seitz, George B.	George B. Seitz	tt0027923
1746503257	1921	1921	The Call of the North	The Call of the North	Henabery, Joseph	Joseph Henabery	tt0012025
1746570088	1934	1934	Fashions of 1934	Fashions of 1934	Dieterle, William	William Dieterle	tt0025101
1746584183	1936	1936	Anything Goes	Anything Goes	Milestone, Lewis	Lewis Milestone	tt0027302
1746580701	1938	1938	Penrod and His Twin Brother	Penrod and His Twin Brother	McGann, William	William C. McGann	tt0030571
1769970401	1967	1967	Myra's Bed	Myra's Bed	Gaston, William	William Gaston	tt3624008
1746408684	1992	1992	Scent of a Woman	Scent of a Woman	Brest, Martin	Martin Brest	tt0105323
1746508884	1923	1923	The Gunfighter	The Gunfighter	Reynolds, Lynn F.	Lynn Reynolds	tt0014107
1746533006	1915	1915	The Boss	The Boss	Chautard, Emile	Emile Chautard	tt0004992
1746434929	1996	1996	I'm Not Rappaport	I'm Not Rappaport	Gardner, Herb	Herb Gardner	tt0116601
1746487592	1917	1917	The Hidden Spring	The Hidden Spring	Hopper, E. Mason	E. Mason Hopper	tt0008091
1746507832	1926	1926	Melodies	Melodies	Ford, Francis	Francis Ford	tt0333904
1746566159	1957	1957	The Seventh Sin	The Seventh Sin	Neame, Ronald	Ronald Neame	tt0050954
1746585305	1930	1930	The Silent Enemy	The Silent Enemy	Carver, H. P.	H.P. Carver	tt0020405
1746487591	1918	1918	The Unbeliever	The Unbeliever	Crosland, Alan	Alan Crosland	tt0009737
1746555443	1955	1955	Not As a Stranger	Not as a Stranger	Kramer, Stanley	Stanley Kramer	tt0048432
1746524302	1917	1917	Daughter of Maryland	Daughter of Maryland	O'Brien, John B.	John B. O'Brien	tt0191067
1746509248	1924	1924	Shadows of Paris	Shadows of Paris	Brenon, Herbert	Herbert Brenon	tt0015320
1746584918	1939	1939	Convict's Code	Convict's Code	Hillyer, Lambert	Lambert Hillyer	tt0031179
1746565907	1954	1954	The Outlaw's Daughter	The Outlaw's Daughter	Barry, Wesley	Wesley Barry	tt0047322
1769970646	1968	1968	Too Much Too Often!	Too Much Too Often!	Silverman, Louis	Doris Wishman	tt0128776
1745359390	2009	2009	Avatar	Avatar	Cameron, James	James Cameron	tt0499549
1746574519	1952	1952	The WAC from Walla Walla	The WAC from Walla Walla	Witney, William	William Witney	tt0045303
1746475906	1947	1947	Under Colorado Skies	Under Colorado Skies	Springsteen, R. G.	R.G. Springsteen	tt0039933
1746473748	1944	1944	The Falcon Out West	The Falcon Out West	Clemens, William	William Clemens	tt0036809
1746525015	1914	1914	A Good Little Devil	A Good Little Devil	Porter, Edwin S.	Edwin S. Porter	tt0004027
1746394022	1992	1992	Mistress	Mistress	Primus, Barry	Barry Primus	tt0104892
1746563375	1952	1952	Apache War Smoke	Apache War Smoke	Kress, Harold	Harold F. Kress	tt0044369
1746582953	1936	1936	Sitting on the Moon	Sitting on the Moon	Staub, Ralph	Ralph Staub	tt0028260

1746394526	1986	1986	Poltergeist II: The Other Side	Poltergeist II: The Other Side	Gibson, Brian	Brian Gibson	tt0091778
1746564768	1953	1953	Captain Scarface	Captain Scarface	Guilfoyle, Paul	Paul Guilfoyle	tt0045604
1746523696	1915	1915	A Matrimonial Boomerang	A Matrimonial Boomerang	Mix, Tom	Tom Mix	tt0005729
1746501309	1922	1922	Deserted at the Altar	Deserted at the Altar	Howard, William K.	William K. Howard	tt0013062
1746581725	1910	1910	The Kid	The Kid	Powell, Frank	Frank Powell	tt0361798
1746464831	1942	1942	Sunset on the Desert	Sunset on the Desert	Kane, Joseph	Joseph Kane	tt0035397
1746464944	1945	1945	Captain Tugboat Annie	Captain Tugboat Annie	Rosen, Phil	Phil Rosen	tt0037577
1746453637	1941	1941	The Gay Vagabond	The Gay Vagabond	Morgan, William	William Morgan	tt0033652
1746577370	1958	1958	Showdown at Boot Hill	Showdown at Boot Hill	Fowler, Gene	Gene Fowler Jr.	tt0052194
1746218697	1972	1972	What's Up, Doc?	What's Up, Doc?	Bogdanovich, Peter	Peter Bogdanovich	tt0069495
1746514521	1916	1916	Man and His Angel	Man and His Angel	King, Burton	Burton L. King	tt0163088
1746272379	1979	1979	King Frat	King Frat	Wiederhorn, Ken	Ken Wiederhorn	tt0079406
1746520441	1949	1949	Range Land	Range Land	Hillyer, Lambert	Lambert Hillyer	tt0041782
1746523823	1916	1916	The Election Bet	The Election Bet	Metcalfe, Earl	Earl Metcalfe	tt0444044
1746582916	1937	1937	Love and Hisses	Love and Hisses	Lanfield, Sidney	Sidney Lanfield	tt0029170
1746492731	1944	1944	Week-End Pass	Week End Pass	Yarbrough, Jean	Jean Yarbrough	tt0037452
1746565903	1953	1953	Star of Texas	Star of Texas	Carr, Thomas	Thomas Carr	tt0046363
1746248463	1966	1966	Johnny Reno	Johnny Reno	Springsteen, R. G.	R.G. Springsteen	tt0060564
1746581981	1929	1929	Spite Marriage	Spite Marriage	Sedgwick, Edward	Edward Sedgwick	tt0020442
1746487828	1919	1919	Turning the Tables	Turning the Tables	Clifton, Elmer	Elmer Clifton	tt0010808
1746492560	1945	1945	The Cheaters	The Cheaters	Kane, Joseph	Joseph Kane	tt0037588
1746566009	1955	1955	Yellowneck	Yellowneck	Hugh, R. John	R. John Hugh	tt0048818
1746555451	1956	1956	Bundle of Joy	Bundle of Joy	Taurog, Norman	Norman Taurog	tt0049034
1746584191	1936	1936	The Dark Hour	The Dark Hour	Lamont, Charles	Charles Lamont	tt0027501
1746486787	1919	1919	The Phantom Honeymoon	The Phantom Honeymoon	Dawley, J. Searle	J. Searle Dawley	tt0010565
1746508268	1950	1949	Intruder in the Dust	Intruder in the Dust	Brown, Clarence	Clarence Brown	tt0041513
1746251481	1980	1980	Friday the 13th	Friday the 13th	Cunningham, Sean S.	Sean S. Cunningham	tt0080761
1746583786	1929	1929	Pointed Heels	Pointed Heels	Sutherland, A. Edward	A. Edward Sutherland	tt0020278
1746500646	1925	1925	A Gentleman Roughneck	Gentleman Roughneck	Jones, Grover	Grover Jones	tt0015856
1745358911	2002	2001	Monsoon Wedding	Monsoon Wedding	Nair, Mira	Mira Nair	tt0265343
1746554999	1964	1964	Topkapi	Topkapi	Dassin, Jules	Jules Dassin	tt0058672
1746198964	1968	1968	The Mini-Skirt Mob	The Mini Skirt Mob	Dexter, Maury	Maury Dexter	tt0063306
1746486185	1919	1919	Atonement	Atonement	Humphrey, William	William Humphrey	tt0009891
1746464940	1946	1946	Roaring Rangers	Roaring Rangers	Nazarro, Ray	Ray Nazarro	tt0038887
1746524734	1915	1915	When We Were Twenty-One	When We Were Twenty One	Porter, Edwin S.; Ford, Hugh	Hugh Ford	tt0006266
1746392955	1984	1984	Ghostbusters	Ghostbusters	Reitman, Ivan	Ivan Reitman	tt0087332
1745282807	2007	2006	Be My Oswald	Be My Oswald	Cato, Don	Don Cato	tt0907620
1745293202	2007	2007	Lions for Lambs	Lions for Lambs	Redford, Robert	Robert Redford	tt0891527
1746584934	1940	1940	Gaucho Serenade	Gaucho Serenade	McDonald, Frank	Frank McDonald	tt0032513
1746574241	1959	1958	Submarine Seahawk	Submarine Seahawk	Bennet, Spencer G.	Spencer Gordon Bennet	tt0052256
1746524118	1914	1914	Wolfe; or, the Conquest of Quebec	Wolfe; Or, The Conquest of Quebec	Buel, Kenean	Kenean Buel	tt0004830
1746572783	1931	1931	The Fighting Sheriff	The Fighting Sheriff	King, Louis	Louis King	tt0021863
1746574402	1951	1951	Sunny Side of the Street	Sunny Side of the Street	Quine, Richard	Richard Quine	tt0044090
1746218871	1975	1975	Cooley High	Cooley High	Schultz, Michael	Michael Schultz	tt0072820
1746524602	1916	1916	Bettina Loved a Soldier	Bettina Loved a Soldier	Julian, Rupert	Rupert Julian	tt0006424
1746475918	1945	1945	Where Do We Go from Here?	Where Do We Go from Here?	Ratoff, Gregory	Gregory Ratoff	tt0038245
1746509203	1926	1926	Money Talks	Money Talks	Mayo, Archie	Archie Mayo	tt0017164
1746465264	1945	1945	Hitchhike to Happiness	Hitchhike to Happiness	Santley, Joseph	Joseph Santley	tt0037782
1746582379	1931	1931	Ex-Bad Boy	Ex Bad Boy	Moore, Vin	Vin Moore	tt0021841
1745340816	2001	2001	Panic	Manic	Bromell, Henry	Jordan Melamed	tt0252684
1746502149	1923	1923	King's Creek Law	King's Creek Law	Maloney, Leo; Williamson, Bob	Leo D. Maloney	tt0014180
1746580276	1936	1936	My American Wife	My American Wife	Young, Harold	Harold Young	tt0028009
1746585454	1940	1940	The Old Swimmin' Hole	The Old Swimmin' Hole	McGowan, Robert	Robert F. McGowan	tt0032864
1746504017	1919	1919	A Regular Fellow	A Regular Fellow	Cabanne, Christy	Christy Cabanne	tt0010620
1746514426	1914	1914	The Floor Above	The Floor Above	Kirkwood, James	James Kirkwood	tt0003972
1746487859	1920	1920	The Deep Purple	The Deep Purple	Walsh, R. A.	Raoul Walsh	tt0011102
1746500608	1924	1924	Virtue's Revolt	Virtue's Revolt	Chapin, James	James Chapin	tt0015463

1746577032	1950	1950	711 Ocean Drive	711 Ocean Drive	Newman, Joseph M.	Joseph M. Newman	tt0042176
1746576955	1963	1963	Around the World With Nothing On	Around the World with Nothing On	Knight, Arthur	Arthur Knight	tt2358893
1746566884	1933	1933	Tugboat Annie	Tugboat Annie	LeRoy, Mervyn	Mervyn LeRoy	tt0024701
1746569861	1957	1957	Hidden Fear	Hidden Fear	DeToth, Andre	André De Toth	tt0050506
1746474960	1944	1944	Sergeant Mike	Sergeant Mike	Levin, Henry	Henry Levin	tt0038074
1746410805	1994	1994	Trading Mom	Trading Mom	Brelis, Tia	Tia Brelis	tt0111470
1746433751	1996	1996	Foxfire	Foxfire	Haywood-Carter, Annette	Annette Haywood	tt0116353
1746523083	1923	1923	Bella Donna	Bella Donna	Fitzmaurice, George	George Fitzmaurice	tt0013867
1746475274	1946	1946	The Green Years	The Green Years	Saville, Victor	Victor Saville	tt0038578
1746520062	1925	1925	The Man Without a Country	The Man Without a Country	Lee, Rowland V.	Rowland V. Lee	tt0016078
1746508918	1949	1949	Sheriff of Wichita	Sheriff of Wichita	Springsteen, R. G.	R.G. Springsteen	tt0041869
1746525176	1921	1921	A Parisian Scandal	A Parisian Scandal	Cox, George L.	George L. Cox	tt0012550
1746465396	1944	1944	Till We Meet Again	Till We Meet Again	Borzage, Frank	Frank Borzage	tt0037379
1746584138	1938	1938	There's Always a Woman	There's Always a Woman	Hall, Alexander	Alexander Hall	tt0030857
1746200319	1968	1968	How Sweet It Is!	How Sweet It Is!	Paris, Jerry	Jerry Paris	tt0063098
1746580413	1938	1938	Romance in the Dark	Romance in the Dark	Potter, H. C.	H.C. Potter	tt0030691
1745293163	2006	2006	Wordplay	Wordplay	Creadon, Patrick	Patrick Creadon	tt0492506
1746566144	1954	1954	The Barefoot Contessa	The Barefoot Contessa	Mankiewicz, Joseph L.	Joseph L. Mankiewicz	tt0046754
1746436559	1941	1941	The Pioneers	The Pioneers	Herman, Al	Albert Herman	tt0034029
1746508230	1949	1949	Souls of Sin	Souls of Sin	Lindsay, Powell	Powell Lindsay	tt0041903
1746571011	1934	1934	The Age of Innocence	The Age of Innocence	Moeller, Philip	Philip Moeller	tt0024819
1746573810	1957	1957	Up In Smoke	Up in Smoke	Beaudine, William	William Beaudine	tt0051143
1746580886	1937	1937	On Such a Night	On Such a Night	Dupont, E. A.	Ewald André Dupont	tt0029344
1746438300	1943	1943	The Constant Nymph	The Constant Nymph	Goulding, Edmund	Edmund Goulding	tt0035751
1746533222	1915	1915	Jewel	Jewel	Smalley, Phillips	Phillips Smalley	tt0005568
1746219153	1975	1976	Adios Amigo	Adiós Amigo	Williamson, Fred	Fred Williamson	tt0072604
1746578589	1960	1960	The Unforgiven	The Unforgiven	Huston, John	John Huston	tt0054428
1746508177	1925	1925	The Making of O'Malley	The Making of O'Malley	Hillyer, Lambert	Lambert Hillyer	tt0016071
1746235237	1971	1971	200 Motels	200 Motels		Tony Palmer	tt0066732
1746500316	1927	1927	The Prairie King	The Prairie King	Eason, Reeves	B. Reeves Eason	tt0018275
1746565898	1953	1953	Sea Devils	Sea Devils	Walsh, Raoul	Raoul Walsh	tt0046286
1746199794	1968	1968	The Young Runaways	The Young Runaways	Dreifuss, Arthur	Arthur Dreifuss	tt0063827
1746219192	1979	1979	Breaking Away	Breaking Away	Yates, Peter	Peter Yates	tt0078902
1746211498	1972	1972	The New Centurions	The New Centurions	Fleischer, Richard	Richard Fleischer	tt0068997
1746567740	1933	1933	Infernal Machine	Infernal Machine	Varnel, Marcel	Marcel Varnel	tt0024180
1746580552	1938	1938	Freshman Year	Freshman Year	McDonald, Frank	Frank McDonald	tt0030158
1746422574	1995	1995	Devil in a Blue Dress	Devil in a Blue Dress	Franklin, Carl	Carl Franklin	tt0112857
1746570741	1934	1934	The Ninth Guest	The Ninth Guest	Neill, R. William	Roy William Neill	tt0025566
1746582181	1928	1928	The Speed Classic	The Speed Classic	Mitchell, Bruce	Bruce Mitchell	tt0019411
1746219658	1974	1974	Chinatown	Chinatown	Polanski, Roman	Roman Polanski	tt0071315
1745342845	2008	2008	Drillbit Taylor	Drillbit Taylor	Brill, Steven	Steven Brill	tt0817538
1746583016	1939	1939	My Son Is a Criminal	My Son Is a Criminal	Coleman, C. C.	Charles C. Coleman	tt0031699
1746573803	1960	1960	All the Young Men	All the Young Men	Bartlett, Hall	Hall Bartlett	tt0053583
1746216705	1969	1969	The Fantastic Plastic Machine	The Fantastic Plastic Machine	Blum, Eric; Blum, Lowell	Eric Blum	tt0233702
1746577867	1938	1938	Ladies in Distress	Ladies in Distress	Meins, Gus	Gus Meins	tt0030339
1746584030	1940	1940	New Moon	New Moon	Leonard, Robert Z.; Van Dyke, W. S.	Robert Z. Leonard	tt0032840
1746577061	1951	1951	According to Mrs. Hoyle	According to Mrs. Hoyle	Yarbrough, Jean	Jean Yarbrough	tt0043260
1746464806	1941	1941	Richest Man in Town	The Richest Man in Town	Barton, Charles	Charles Barton	tt0034099
1746499989	1949	1949	The Big Wheel	The Big Wheel	Ludwig, Edward	Edward Ludwig	tt0041179
1769971950	1969	1969	Lady Godiva Rides	Lady Godiva Rides	Stephen, A. C.	Stephen C. Apostolof	tt0125332
1746503011	1915	1915	What Happened to Jones	What Happened to Jones	Mace, Fred	Fred Mace	tt0006253
1746466980	1917	1917	The High Sign	The High Sign	Clifton, Elmer	Elmer Clifton	tt0008095
1746473920	1944	1944	The Last Horseman	The Last Horseman	Berke, William	William Berke	tt0037006
1745341845	2002	2002	Welcome to Collinwood	Welcome to Collinwood	Russo, Anthony ; Russo, Joe	Anthony Russo	tt0271259
1746394200	1983	1983	Easy Money	Easy Money	Signorelli, James	James Signorelli	tt0085470
1746234120	1965	1964	The Pawnbroker	The Pawnbroker	Lumet, Sidney	Sidney Lumet	tt0059575
1746514791	1915	1915	The Turn of the Road	The Turn of the Road	Johnson, Tefft	Tefft Johnson	tt0006187

1746580720	1938	1938	Sunset Trail	Sunset Trail	Selander, Lesley	Lesley Selander	tt0030812
1746498312	1921	1921	The Snob	The Snob	Wood, Sam	Sam Wood	tt0012705
1746570274	1934	1934	Sing Sing Nights	Sing Sing Nights	Collins, Lewis D.	Lewis D. Collins	tt0027003
1769970776	1912	1912	The Ghost of Sulphur Mountain	The Ghost of Sulphur Mountain		Gaston Méliès	tt0403068
1746567299	1934	1934	Mystery Liner	Mystery Liner	Nigh, William	William Nigh	tt0025543
1745394542	2010	2010	Gulliver's Travels	Gulliver's Travels	Letterman, Rob	Rob Letterman	tt1320261
1746491772	1948	1948	Night Has a Thousand Eyes	Night Has a Thousand Eyes	Farrow, John	John Farrow	tt0040643
1746582877	1938	1938	Vivacious Lady	Vivacious Lady	Stevens, George	George Stevens	tt0030944
1746572249	1931	1931	The Easiest Way	The Easiest Way	Conway, Jack	Jack Conway	tt0021825
1746499871	1948	1948	Tenth Avenue Angel	Tenth Avenue Angel	Rowland, Roy	Roy Rowland	tt0040865
1746573986	1958	1958	The Heart Is a Rebel	The Heart Is a Rebel	Ross, Dick	Dick Ross	tt0288042
1746423713	1940	1940	Heroes of the Saddle	Heroes of the Saddle	Witney, William	William Witney	tt0032584
1745359168	2004	2004	Friday Night Lights	Friday Night Lights	Berg, Peter	Peter Berg	tt0390022
1746574335	1963	1964	Twice a Man	Twice a Man	Markopoulos, Gregory J.	Gregory J. Markopoulos	tt0129470
1746574086	1956	1956	The Revolt of Mamie Stover	The Revolt of Mamie Stover	Walsh, Raoul	Raoul Walsh	tt0049672
1745293831	2007	2007	Borderland	Borderland	Berman, Zev	Zev Berman	tt0452592
1746485069	1919	1919	His Divorced Wife	His Divorced Wife	Gerrard, Douglas	Douglas Gerrard	tt0010254
1746534800	1921	1921	The Man Worth While	The Man Worthwhile	Fielding, Romaine	Romaine Fielding	tt0012440
1746583623	1930	1930	Chasing Rainbows	Chasing Rainbows	Reisner, Charles F.	Charles Reisner	tt0020336
1746584592	1931	1931	Once a Sinner	Once a Sinner	McClintic, Guthrie	Guthrie McClintic	tt0021211
1746218927	1979	1979	Tourist Trap	Tourist Trap	Schmoeller, David	David Schmoeller	tt0080040
1746523224	1915	1915	The Victory of Virtue	The Victory of Virtue	Webster, Harry McRae	Harry McRae Webster	tt0006221
1746576847	1931	1931	Side Show	Side Show	Del Ruth, Roy	Roy Del Ruth	tt0022381
1746533054	1927	1927	With Sitting Bull at the Spirit Lake Massacre	With Sitting Bull at the Spirit Lake Massacre	Bradbury, Robert North	Robert N. Bradbury	tt0017399
1746455629	2000	2000	Gladiator	Gladiator	Scott, Ridley	Ridley Scott	tt0172495
1746582581	1938	1938	Merrily We Live	Merrily We Live	McLeod, Norman Z.	Norman Z. McLeod	tt0030442
1746578758	1959	1959	The Man Who Understood Women	The Man Who Understood Women	Johnson, Nunnally	Nunnally Johnson	tt0053043
1746578547	1958	1958	Tank Battalion	Tank Battalion	Rose, Sherman A.	Sherman A. Rose	tt0051052
1746454127	2000	2000	High Fidelity	High Fidelity	Frears, Stephen	Stephen Frears	tt0146882
1746502414	1916	1915	Between Men	Between Men	Hart, William S.	William S. Hart	tt0004962
1746566251	1958	1958	The Old Man and the Sea	The Old Man and the Sea	Sturges, John; Zinnemann, Fred	John Sturges	tt0052027
1746532712	1915	1915	The Song of the Wage Slave	The Song of the Wage Slave		Herbert Blaché	tt0006072
1746554264	1965	1965	Echoes of Silence	Echoes of Silence	Goldman, Peter Emanuel	Peter Emmanuel Goldman	tt0208927
1746561056	1932	1932	The Devil on Deck	Devil on Deck	Fox, Wallace W.	Wallace Fox	tt0022813
1746582951	1935	1935	After Office Hours	After Office Hours	Leonard, Robert Z.	Robert Z. Leonard	tt0026043
1746555872	1956	1956	Tension at Table Rock	Tension at Table Rock	Warren, Charles Marquis	Charles Marquis Warren	tt0049834
1746581845	1929	1929	Love in the Desert	Love in the Desert	Melford, George	George Melford	tt0020116
1746512419	1922	1922	Conquering the Woman	Conquering the Woman	Vidor, King	King Vidor	tt0013024
1746582498	1938	1938	Penrod's Double Trouble	Penrod's Double Trouble	Seiler, Lewis	Lewis Seiler	tt0030572
1746219195	1976	1976	Grizzly	Grizzly	Girdler, William	William Girdler	tt0074593
1746583330	1950	1950	Trail of Robin Hood	Trail of Robin Hood	Witney, William	William Witney	tt0043061
1746524114	1927	1927	Becky	Becky	McCarthy, John P.	John P. McCarthy	tt0017666
1746571342	1934	1934	The Black Cat	The Black Cat	Ulmer, Edgar G.	Edgar G. Ulmer	tt0024894
1746568012	1932	1932	Too Busy to Work	Too Busy to Work	Blystone, John	John G. Blystone	tt0023606
1746582987	1940	1940	Dark Command	Dark Command	Walsh, Raoul	Raoul Walsh	tt0032383
1746486864	1920	1920	The Isle of Destiny	The Isle of Destiny	Lane, Tamar	Tamar Lane	tt0195841
1746235653	1974	1974	Blazing Saddles	Blazing Saddles	Brooks, Mel	Mel Brooks	tt0071230
1746434937	1996	1996	Daylight	Daylight	Cohen, Rob	Rob Cohen	tt0116040
1746578618	1963	1963	Showdown	Showdown	Springsteen, R. G.	R.G. Springsteen	tt0057497
1746581688	1928	1928	Lost in the Arctic	Lost in the Arctic	Snow, Sydney; Snow, H. A.	H.A. Snow	tt0020110
1746502930	1915	1915	Infatuation	Infatuation	Pollard, Harry	Harry A. Pollard	tt0005548
1746420146	1994	1994	Lassie	Lassie	Petrie, Daniel	Daniel Petrie	tt0110305
1746503000	1915	1915	The Menace of the Mute	The Menace of the Mute	Miller, Ashley; Daly, Arnold	Ashley Miller	tt0005738
1746437000	1940	1940	The Leatherpushers	The Leather Pushers	Rawlins, John	John Rawlins	tt0032695
1746584589	1929	1929	The White Outlaw	The White Outlaw	Horner, Robert J.	Robert J. Horner	tt0020584
1746409054	1983	1983	WarGames	WarGames	Badham, John	John Badham	tt0086567
1745282681	2007	2007	Lars and the Real Girl	Lars and the Real Girl	Gillespie, Craig	Craig Gillespie	tt0805564

1746420578	1940	1940	Thundering Frontier	Thundering Frontier	Lederman, D. Ross	D. Ross Lederman	tt0033163
1746582231	1931	1931	Illicit	Illicit	Mayo, Archie	Archie Mayo	tt0021992
1746465132	1942	1942	You're Telling Me	You're Telling Me	Lamont, Charles	Charles Lamont	tt0035584
1746474133	1947	1947	Cheyenne	Cheyenne	Walsh, Raoul	Raoul Walsh	tt0039260
1745340028	2003	2003	Darkness Falls	Darkness Falls	Liebesman, Jonathan	Jonathan Liebesman	tt0282209
1746574959	1937	1937	Women Men Marry	The Women Men Marry	Taggart, Errol	Errol Taggart	tt0029793
1746514703	1916	1916	Pillars of Society	Pillars of Society		Raoul Walsh	tt0007184
1746500126	1925	1925	Wandering Footsteps	Wandering Footsteps	Rosen, Phil	Phil Rosen	tt0016502
1746434289	1997	1997	Fast, Cheap & Out of Control	Fast, Cheap & Out of Control	Morris, Errol	Errol Morris	tt0119107
1746475949	1947	1946	Going to Glory, Come to Jesus	Going to Glory... Come to Jesus		T. Meyer	tt0038568
1746420402	1992	1992	Freejack	Freejack	Murphy, Geoff	Geoff Murphy	tt0104299
1746476207	1947	1947	Philo Vance's Secret Mission	Philo Vance's Secret Mission	LeBorg, Reginald	Reginald Le Borg	tt0039703
1746500736	1924	1924	The Law and the Lady	The Law and the Lady	McCutcheon, John L.	John L. McCutcheon	tt0015057
1746424419	1940	1940	Moon over Burma	Moon Over Burma	King, Louis	Louis King	tt0032809
1746508210	1927	1927	Streets of Shanghai	Streets of Shanghai	Gasnier, Louis	Louis J. Gasnier	tt0019432
1746219647	1972	1972	Now You See Him, Now You Don't	Now You See Him, Now You Don't	Butler, Robert	Robert Butler	tt0069031
1746584944	1939	1939	Private Detective	Private Detective	Smith, Noel	Noel M. Smith	tt0031825
1746208293	1969	1969	How To Commit Marriage	How to Commit Marriage	Panama, Norman	Norman Panama	tt0064449
1746556036	1955	1954	The Violent Men	The Violent Men	Maté, Rudolph	Rudolph Maté	tt0048789
1745272882	2000	2000	Shaft	Shaft	Singleton, John	John Singleton	tt0162650
1746454701	1940	1940	Andy Hardy Meets Debutante	Andy Hardy Meets Debutante	Seitz, George B.	George B. Seitz	tt0032206
1746573879	1960	1960	The Apartment	The Apartment	Wilder, Billy	Billy Wilder	tt0053604
1745359477	2004	2004	Secret Window	Secret Window	Koepp, David	David Koepp	tt0363988
1746496566	1918	1918	Brown of Harvard	Brown of Harvard	Beaumont, Harry	Harry Beaumont	tt0008932
1746440010	1996	1996	The People vs. Larry Flynt	The People vs. Larry Flynt	Forman, Milos	Milos Forman	tt0117318
1746576227	1932	1932	Scarface	Scarface	Hawks, Howard	Howard Hawks	tt0023427
1746567825	1933	1933	Obey the Law	Obey the Law	Stoloff, Benjamin	Benjamin Stoloff	tt0024398
1746502272	1915	1915	Du Barry	DuBarry	Bencivenga, Edoardo	Edoardo Bencivenga	tt0005229
1745358016	2002	2002	Dragonfly	Dragonfly	Shadyac, Tom	Tom Shadyac	tt0259288
1769970643	1968	1968	Orgy Girls '69	Orgy Girls '69	Canton, Robert	Robert Canton	tt0130164
1746393566	1984	1984	Finders Keepers	Finders Keepers	Lester, Richard	Richard Lester	tt0087260
1746504330	1920	1920	Birthright	Birthright	Hemmer, Edward	Edward L. Hemmer	tt0186881
1746433914	1991	1991	JFK	JFK	Stone, Oliver	Oliver Stone	tt0102138
1746585250	1929	1929	Sally	Sally	Dillon, John Francis	John Francis Dillon	tt0020358
1769976194	1918	1918	Beauties and Bombs	Beauties and Bombs	Seiter, William A.	William A. Seiter	tt1315929
1746581452	1950	1950	Trigger, Jr.	Trigger, Jr.	Witney, William	William Witney	tt0043069
1746500043	1949	1949	Arson--Inc.	Arson, Inc.	Berke, William	William Berke	tt0041133
1746574950	1933	1933	Strictly Personal	Strictly Personal	Murphy, Ralph	Ralph Murphy	tt0024622
1746580920	1959	1958	Slippery When Wet	Slippery When Wet	Brown, Bruce A.	Bruce Brown	tt0298155
1746497521	1917	1917	Twin Kiddies	Twin Kiddies	King, Henry	Henry King	tt0008706
1746530791	1950	1950	Whirlpool	Whirlpool	Preminger, Otto	Otto Preminger	tt0042039
1746497055	1918	1918	The Law That Divides	The Law That Divides	Mitchell, Howard M.	Howard M. Mitchell	tt0186289
1746569548	1958	1958	Desert Hell	Desert Hell	Warren, Charles Marquis	Charles Marquis Warren	tt0051531
1746422924	1993	1993	Cool Runnings	Cool Runnings	Turteltaub, Jon	Jon Turteltaub	tt0106611
1746542707	1926	1925	Hard Boiled	Hard Boiled	Blystone, J. G.	Leo McCarey	tt0209012
1746514562	1923	1923	Three O'Clock in the Morning	Three O'Clock in the Morning	Webb, Kenneth	Kenneth S. Webb	tt0014540
1746500465	1926	1926	The Country Beyond	The Country Beyond	Cummings, Irving	Irving Cummings	tt0016744
1746208222	1969	1968	Last Summer Won't Happen	Last Summer Won't Happen	Gessner, Peter; Hurwitz, Tom	Peter Gessner	tt2221727
1746581162	1961	1961	When the Clock Strikes	When the Clock Strikes	Cahn, Edward L.	Edward L. Cahn	tt0055617
1769971156	1969	1969	The Filth Shop	The Filth Shop		Looney Bear	tt0218238
1746582630	1937	1937	Flight from Glory	Flight from Glory	Landers, Lew	Lew Landers	tt0028880
1746185677	1910	1910	An Advertisement Answered	An Advertisement Answered	Ricketts, Thomas	Tom Ricketts	tt1661349
1746568015	1932	1932	The Broken Wing	The Broken Wing	Corrigan, Lloyd	Lloyd Corrigan	tt0022726
1746570163	1952	1952	A Yank in Indo-China	A Yank in Indo China	Grissell, Wallace A.	Wallace Grissell	tt0045342
1746576971	1962	1962	Surftide 77	Surftide 77	Frost, R. L.	Lee Frost	tt0056538
1746573817	1958	1958	The Case Against Brooklyn	The Case Against Brooklyn	Wendkos, Paul	Paul Wendkos	tt0051456
1746582453	1937	1937	Sky Racket	Sky Racket	Katzman, Sam	Sam Katzman	tt0140551

1746507569	1926	1926	The Imposter	The Impostor	Withey, Chet	Chester Withey	tt0016993
1746520199	1925	1925	Lovers in Quarantine	Lovers in Quarantine	Tuttle, Frank	Frank Tuttle	tt0016048
1746583846	1931	1931	Kid from Arizona	The Kid from Arizona	Horner, Robert J.	Robert J. Horner	tt0022024
1746512912	1915	1915	Ranson's Folly	Ranson's Folly	Ridgley, Richard	Richard Ridgely	tt0005950
1746487490	1919	1919	The Misleading Widow	The Misleading Widow	Robertson, John S.	John S. Robertson	tt0010469
1746393459	1986	1986	Back to School	Back to School	Metter, Alan	Alan Metter	tt0090685
1746409680	1985	1985	The Return of the Living Dead	The Return of the Living Dead	O'Bannon, Dan	Dan O'Bannon	tt0089907
1745272370	2000	2000	The Replacements	The Replacements	Deutch, Howard	Howard Deutch	tt0191397
1746532428	1915	1915	A Butterfly on the Wheel	A Butterfly on the Wheel	Tourneur, Maurice	Maurice Tourneur	tt0005041
1746585337	1930	1930	Man Trouble	Man Trouble	Viertel, Berthold	Berthold Viertel	tt0021114
1746520140	1925	1925	The Circle	The Circle	Borzage, Frank	Frank Borzage	tt0015684
1746561211	1932	1932	The Animal Kingdom	The Animal Kingdom	Griffith, Edward H.	Edward H. Griffith	tt0022628
1746577534	1934	1934	The Lost Jungle	The Lost Jungle	Schaefer, Armand; Howard, David	David Howard	tt0025420
1746453641	1942	1942	Johnny Doughboy	Johnny Doughboy	Auer, John H.	John H. Auer	tt0034921
1769970497	1966	1965	The Love Statue	The Love Statue	Durston, David	David E. Durston	tt0061030
1746515812	1920	1920	The Virgin of Stamboul	The Virgin of Stamboul	Browning, Tod	Tod Browning	tt0011830
1746491814	1944	1944	Up in Arms	Up in Arms	Nugent, Elliott	Elliott Nugent	tt0037420
1746570593	1934	1934	Heat Lightning	Heat Lightning	LeRoy, Mervyn	Mervyn LeRoy	tt0025228
1746422242	1990	1989	Robot Jox	Robot Jox	Gordon, Stuart	Stuart Gordon	tt0102800
1746577768	1938	1938	The Little Adventuress	The Little Adventuress	Lederman, D. Ross	D. Ross Lederman	tt0030369
1746583452	1930	1930	East Is West	East Is West	Bell, Monta	Monta Bell	tt0020850
1746423263	1992	1992	Batman Returns	Batman Returns	Burton, Tim	Tim Burton	tt0103776
1746493825	1910	1910	The Troublesome Baby	The Troublesome Baby	Powell, Frank	Frank Powell	tt0194472
1746437959	1941	1941	Law of the Timber	Law of the Timber	Ray, Bernard B.	Bernard B. Ray	tt0033820
1746577600	1933	1933	Savage Gold	Savage Gold	Dyott, George M.		tt0195233
1746570591	1952	1952	Kid Monk Baroni	Kid Monk Baroni	Schuster, Harold	Harold D. Schuster	tt0044796
1746501394	1914	1914	The Ordeal	The Ordeal	Davis, William S.	Tom Santschi	tt0313434
1746475911	1948	1948	King of the Gamblers	King of the Gamblers	Blair, George	George Blair	tt0040511
1746565943	1953	1953	A Lion Is in the Streets	A Lion Is in the Streets	Walsh, Raoul	Raoul Walsh	tt0046002
1746474455	1945	1945	Strange Illusion	Strange Illusion	Ulmer, Edgar G.	Edgar G. Ulmer	tt0038126
1746512420	1915	1915	Zaza	Zaza	Porter, Edwin S.; Ford, Hugh	Hugh Ford	tt0006330
1746574927	1933	1933	Sons of the Desert	Sons of the Desert	Seiter, William A.	William A. Seiter	tt0024601
1746424557	1942	1942	Heart of the Rio Grande	Heart of the Rio Grande	Morgan, William	William Morgan	tt0034833
1746576700	1931	1931	Murder at Midnight	Murder at Midnight	Strayer, Frank	Frank R. Strayer	tt0022172
1746475258	1944	1944	Mr. Skeffington	Mr. Skeffington	Sherman, Vincent	Vincent Sherman	tt0037094
1746439410	1942	1942	War Dogs	War Dogs	Luby, S. Roy	S. Roy Luby	tt0035532
1746585433	1939	1939	Island of Lost Men	Island of Lost Men	Neumann, Kurt	Kurt Neumann	tt0031497
1746572189	1910	1910	A Child's Impulse	A Child's Impulse	Griffith, D. W.	D.W. Griffith	tt0001152
1746520784	1926	1926	The Midnight Message	The Midnight Message	Hurst, Paul	Paul Hurst	tt0017143
1746571631	1932	1932	Come On Danger!	Come on Danger!	Hill, Robert	Robert F. Hill	tt0022774
1746584882	1939	1939	Home on the Prairie	Home on the Prairie	Townley, Jack	Jack Townley	tt0031437
1746465268	1942	1942	A Yank on the Burma Road	A Yank on the Burma Road	Seitz, George B.	George B. Seitz	tt0035574
1746419191	1987	1987	The Whales of August	The Whales of August	Anderson, Lindsay	Lindsay Anderson	tt0094315
1746520516	1949	1949	Tension	Tension	Berry, John	John Berry	tt0041954
1746584070	1939	1939	The Roaring Twenties	The Roaring Twenties	Walsh, Raoul	Raoul Walsh	tt0031867
1746508379	1925	1925	Double Action Daniels	Double Action Daniels	Thorpe, Richard	Richard Thorpe	tt0130632
1746583417	1931	1931	West of Cheyenne	West of Cheyenne	Webb, Harry S.	Harry S. Webb	tt0022554
1746507612	1925	1925	Up the Ladder	Up the Ladder	Sloman, Edward	Edward Sloman	tt0016478
1746573599	1931	1931	Two Gun Caballero	Two Gun Caballero	Nelson, Jack	Jack Nelson	tt0022512
1746575036	1938	1938	Desert Patrol	Desert Patrol	Neufield, Sam	Sam Newfield	tt0030051
1746532989	1914	1914	The Woman of Mystery	The Woman of Mystery	Blaché, Alice	Alice Guy	tt0004833
1746485199	1919	1919	Lure of Ambition	The Lure of Ambition	Lawrence, Edmund	Edmund Lawrence	tt0010407
1746520934	1948	1948	Singin' Spurs	Singin' Spurs	Nazarro, Ray	Ray Nazarro	tt0040792
1746582554	1935	1935	Living on Velvet	Living on Velvet	Borzage, Frank	Frank Borzage	tt0026644
1746578608	1959	1959	That Kind of Woman	That Kind of Woman	Lumet, Sidney	Sidney Lumet	tt0053349
1746454080	1943	1943	Dixie Dugan	Dixie Dugan	Brower, Otto; McCarey, Ray	Otto Brower	tt0035811
1746455781	1998	1998	Why Do Fools Fall in Love	Why Do Fools Fall in Love	Nava, Gregory	Gregory Nava	tt0123324

1746200290	1966	1966	Gambit	Gambit	Neame, Ronald	Ronald Neame	tt0060445
1746488011	1921	1921	The Trail to Red Dog	Trail to Red Dog	Franchon, Leonard	Leonard Franchon	tt0173349
1746542762	1925	1925	Shattered Lives	Shattered Lives	McCarty, Henry	Henry McCarty	tt0163827
1746573822	1957	1957	The Unearthly	The Unearthly	Peters, Brooke L.	Boris Petroff	tt0051134
1746583575	1951	1951	Weekend with Father	Week End with Father	Sirk, Douglas	Douglas Sirk	tt0044199
1746474518	1945	1944	The Thin Man Goes Home	The Thin Man Goes Home	Thorpe, Richard	Richard Thorpe	tt0037365
1746507719	1924	1924	The Virgin	The Virgin	Neitz, Alvin J.	Alan James	tt0015461
1746500663	1950	1950	Paid in Full	Paid in Full	Dieterle, William	William Dieterle	tt0042830
1746525021	1920	1920	Old Dad	Old Dad	Ingraham, Lloyd	Lloyd Ingraham	tt0011529
1746453772	1943	1943	The Man from the Rio Grande	The Man from the Rio Grande	Bretherton, Howard	Howard Bretherton	tt0036133
1746584939	1939	1939	Blackwell's Island	Blackwell's Island	McGann, William	William C. McGann	tt0031103
1746581367	1910	1910	The Little Spreewald Maiden	The Little Spreewald Maiden		Sidney Olcott	tt0255326
1746198428	1969	1969	This Sporting House	This Sporting House	Sullivan, Ron	Henri Pachard	tt0259643
1745373933	2010	2010	The Expendables	The Expendables	Stallone, Sylvester	Sylvester Stallone	tt1320253
1746533177	1927	1927	The Valley of Hell	The Valley of Hell	Smith, Clifford S.	Clifford Smith	tt0017511
1746514733	1915	1915	The Ingratitude of Liz Taylor	Ingratitude of Liz Taylor	Le Saint, E. J.	Edward LeSaint	tt0142403
1746514346	1916	1916	Playing with Fire	Playing with Fire	Grandon, Francis J.	Francis J. Grandon	tt0007190
1746423988	1943	1943	Behind Prison Walls	Behind Prison Walls	Sekely, Steve	Steve Sekely	tt0035668
1746509323	1925	1925	A Man of Iron	A Man of Iron	Bennett, Whitman	Whitman Bennett	tt0134810
1746566982	1952	1952	Kansas City Confidential	Kansas City Confidential	Karlson, Phil	Phil Karlson	tt0044789
1746561980	1956	1956	The Lone Ranger	The Lone Ranger	Heisler, Stuart	Stuart Heisler	tt0048310
1746423336	1997	1997	Nick and Jane	Nick and Jane	Mauro, Rich	Richard Mauro	tt0117172
1746509213	1926	1926	Flying High	Flying High	Hutchinson, Charles	Charles Hutchison	tt0016890
1746393052	1985	1985	Too Scared to Scream	Too Scared to Scream	Lo Bianco, Tony	Tony Lo Bianco	tt0090186
1746500013	1949	1948	Whispering Smith	Whispering Smith	Fenton, Leslie	Leslie Fenton	tt0040965
1746555000	1964	1964	Behind the Nudist Curtain	Behind the Nudist Curtain	Wishman, Doris	Doris Wishman	tt0186864
1746566208	1958	1957	The Astounding She-Monster	The Astounding She Monster	Ashcroft, Ronnie	Ronald V. Ashcroft	tt0050143
1746486275	1919	1919	A Sporting Chance	A Sporting Chance	Melford, George	Henry King	tt0010729
1746453586	1941	1941	Nice Girl?	Nice Girl?	Seiter, William A.	William A. Seiter	tt0033950
1746577131	1951	1951	Hard, Fast and Beautiful	Hard, Fast and Beautiful	Lupino, Ida	Ida Lupino	tt0043619
1746562415	1955	1955	The Glass Slipper	The Glass Slipper	Walters, Charles	Charles Walters	tt0048124
1746583214	1951	1951	Royal Wedding	Royal Wedding	Donen, Stanley	Stanley Donen	tt0043983
1746565952	1953	1953	Fighter Attack	Fighter Attack	Selander, Lesley	Lesley Selander	tt0045765
1745271919	2001	2001	Moulin Rouge!	Moulin Rouge!	Luhrmann, Baz	Baz Luhrmann	tt0203009
1746575008	1937	1937	Navy Blues	Navy Blues	Staub, Ralph	Ralph Staub	tt0029298
1746219234	1980	1980	Serial	Serial	Persky, Bill	Bill Persky	tt0081485
1746560517	1965	1965	The Great Sioux Massacre	The Great Sioux Massacre	Salkow, Sidney	Sidney Salkow	tt0059244
1746499921	1949	1949	Western Renegades	Western Renegades	Fox, Wallace	Wallace Fox	tt0042037
1746524691	1914	1914	A Million Bid	A Million Bid	Ince, Ralph W.	Ralph Ince	tt0004335
1746439143	1942	1942	George Washington Slept Here	George Washington Slept Here	Keighley, William	William Keighley	tt0034780
1746566333	1957	1957	The Green-Eyed Blonde	The Green Eyed Blonde	Girard, Bernard	Bernard Girard	tt0050457
1746438951	1943	1943	Riding High	Riding High	Marshall, George	George Marshall	tt0036313
1746573672	1931	1931	Bad Sister	The Bad Sister	Henley, Hobart	Hobart Henley	tt0021636
1746584899	1940	1940	Men Without Souls	Men Without Souls	Grindé, Nick	Nick Grinde	tt0032783
1746514838	1910	1910	A Simple Mistake	A Simple Mistake		Theodore Wharton	tt1225735
1746574122	1959	1959	The Big Fisherman	The Big Fisherman	Borzage, Frank	Frank Borzage	tt0052627
1746197927	1967	1967	Warning Shot	Warning Shot	Kulik, Buzz	Buzz Kulik	tt0062473
1746464926	1944	1944	Dixie Jamboree	Dixie Jamboree	Cabanne, Christy	Christy Cabanne	tt0036762
1746525160	1920	1920	His Pajama Girl	His Pajama Girl	Edwards, Donald	Donald Edwards	tt0012286
1746574274	1963	1962	Black Gold	Black Gold	Martinson, Leslie H.	Leslie H. Martinson	tt0056871
1746513201	1916	1916	The Ballet Girl	The Ballet Girl		George Irving	tt0006398
1746584399	1936	1936	Everybody's Old Man	Everybody's Old Man	Flood, James	James Flood	tt0027587
1746501213	1914	1914	The Dancer and the King	The Dancer and the King	Arnaud, E.	Étienne Arnaud	tt0003820
1746475718	1948	1948	Sinister Journey	Sinister Journey	Archainbaud, George	George Archainbaud	tt0040793
1746574555	1936	1936	Beware of Ladies	Beware of Ladies	Pichel, Irving	Irving Pichel	tt0027354
1746578977	1958	1958	Terror from the Year 5,000	Terror from the Year 5000	Gurney, Robert J.	Robert J. Gurney Jr.	tt0052286
1746504151	1921	1921	The Conflict	The Conflict	Paton, Stuart	Stuart Paton	tt0012066

1746217947	1968	1969	The Virgin President	The Virgin President	Ferguson, Graeme	Graeme Ferguson	tt0063783	
1746437374	1943	1943	Law of the Saddle	Law of the Saddle	De Lay, Melville	Melville De Lay	tt0037010	
1746421293	1994	1993	Mi Vida Loca	Mi vida loca	Anders, Allison	Allison Anders	tt0107566	
1746566025	1954	1954	Susan Slept Here	Susan Slept Here	Tashlin, Frank	Frank Tashlin	tt0047550	
1746561575	1956	1956	Massacre	Massacre	King, Louis	Louis King	tt0049485	
1746491752	1946	1946	People Are Funny	People Are Funny	White, Sam	Sam White	tt0038831	
1746475519	1946	1946	I've Always Loved You	I've Always Loved You	Borzage, Frank	Frank Borzage	tt0038629	
1746474807	1944	1944	Shadow of Suspicion	Shadow of Suspicion	Beaudine, William	William Beaudine	tt0037265	
1746500693	1926	1926	Devil's Island	Devil's Island	O'Connor, Frank	Frank O'Connor	tt0016787	
1769976129	1926	1926	The Battling Kangaroo	The Battling Kangaroo	White, Jules	Jules White	tt0367533	
1746566825	1933	1933	Wine, Women and Song	Wine, Women and Song	Brenon, Herbert	Herbert Brenon	tt0024776	
1746577050	1960	1960	Portrait in Black	Portrait in Black	Gordon, Michael	Michael Gordon	tt0054197	
1746564734	1953	1953	Dangerous Crossing	Dangerous Crossing	Newman, Joseph M.	Joseph M. Newman	tt0045669	
1745373305	2009	2009	The Fourth Kind	The Fourth Kind	Osunsanmi, Olatunde	Olatunde Osunsanmi	tt1220198	
1746210371	1970	1970	Black Angels	Black Angels	Merrick, Laurence	Laurence Merrick	tt0065474	
1746393418	1983	1983	Blue Thunder	Blue Thunder	Badham, John	John Badham	tt0085255	
**1746503349**	**1920**	**1920**	**Miss Hobbs**	**Miss Hobbs**	**Crisp, Donald**	**Donald Crisp**	**tt0011469**	1
1746555044	1955	1955	Paris Follies of 1956	Paris Follies of 1956	Goodwins, Leslie	Leslie Goodwins	tt0048094	
1746186057	1969	1969	They Shoot Horses, Don't They?	They Shoot Horses, Don't They?	Pollack, Sydney	Sydney Pollack	tt0065088	
1746581750	1929	1929	The Tip-Off	The Tip Off	Jason, Leigh	Leigh Jason	tt0020506	
1746419757	1994	1994	Above the Rim	Above the Rim	Pollack, Jeff	Jeff Pollack	tt0109035	
1769970151	1912	1912	Help! Help!	Help! Help!		Mack Sennett	tt0002233	
1746568340	1934	1934	Here Comes the Groom	Here Comes the Groom	Sedgwick, Edward	Edward Sedgwick	tt0025237	
1746423276	1997	1997	The Race to Save 100 Years	The Race to Save 100 Years	Benson, Scott	Scott Benson	tt0128429	
1746210872	1971	1971	Bananas	Bananas	Allen, Woody	Woody Allen	tt0066808	
1746454855	1945	1945	The Naughty Nineties	The Naughty Nineties	Yarbrough, Jean	Jean Yarbrough	tt0037939	
1746500110	1924	1924	Three Women	Three Women	Lubitsch, Ernst	Ernst Lubitsch	tt0015409	
1746580600	1939	1939	Panama Patrol	Panama Patrol	Lamont, Charles	Charles Lamont	tt0031778	
1746409090	1991	1991	Bill & Ted's Bogus Journey	Bill & Ted's Bogus Journey	Hewitt, Pete	Peter Hewitt	tt0101452	
1746198227	1969	1969	The Happy Ending	The Happy Ending	Brooks, Richard	Richard Brooks	tt0064405	
1746423296	1989	1989	New Year's Day	New Year's Day	Jaglom, Henry	Henry Jaglom	tt0097964	
1746502323	1922	1922	Forget-Me-Not	Forget Me Not	Van Dyke, W. S.	W.S. Van Dyke	tt0013145	
1746581901	1930	1930	River's End	River's End	Curtiz, Michael	Michael Curtiz	tt0021303	
1746584609	1929	1929	The Very Idea	The Very Idea	Rosson, Richard	Frank Craven	tt0020549	
1746454945	1945	1945	River Gang	River Gang	David, Charles	Charles David	tt0038030	
1746512605	1915	1915	The Marriage of Kitty	The Marriage of Kitty	Melford, George	George Melford	tt0005715	
1746393463	1983	1983	Two of a Kind	Two of a Kind	Herzfeld, John	John Herzfeld	tt0086494	
1746514807	1928	1928	The Midnight Taxi	The Midnight Taxi	Adolfi, John	John G. Adolfi	tt0019165	
1746524127	1922	1922	Bulldog Courage	Bulldog Courage	Kull, Edward	Edward A. Kull	tt0015652	
1746577835	1937	1937	The Crime Nobody Saw	The Crime Nobody Saw	Barton, Charles	Charles Barton	tt0028748	
1746563964	1952	1952	Macao	Macao	Sternberg, Josef von	Josef von Sternberg	tt0044863	
1746464974	1947	1947	The Hat-box Mystery	The Hat Box Mystery	Hillyer, Lambert	Lambert Hillyer	tt0038590	
1746514402	1915	1915	The Flash of an Emerald	The Flash of an Emerald	Capellani, Albert	Albert Capellani	tt0005331	
1745293864	2005	2005	Broken Flowers	Broken Flowers	Jarmusch, Jim	Jim Jarmusch	tt0412019	
1745374953	2010	2010	How Do You Know	How Do You Know	Brooks, James L.	James L. Brooks	tt1341188	
1746578042	1935	1935	The Great Hotel Murder	The Great Hotel Murder	Forde, Eugene	Eugene Forde	tt0026437	
1746198168	1965	1965	Angel's Flight	Angel's Flight	Nassour, Raymond; Richardson, K. W	Raymond Nassour	tt0316601	
1746501219	1916	1916	The Catspaw	The Catspaw	Wright, George A.	George A. Wright	tt0157447	
1746219383	1976	1976	At the Earth's Core	At the Earth's Core	Connor, Kevin	Kevin Connor	tt0074157	
1746574329	1958	1957	Oregon Passage	Oregon Passage	Landres, Paul	Paul Landres	tt0052034	
1746500546	1926	1926	The Ridin' Rascal	The Ridin' Rascal	Smith, Clifford S.	Clifford Smith	tt0017323	
1746574384	1951	1951	The Sword of Monte Cristo	The Sword of Monte Cristo	Geraghty, Maurice	Maurice Geraghty	tt0044094	
1746453887	1997	1997	Meet Wally Sparks	Meet Wally Sparks	Baldwin, Peter	Peter Baldwin	tt0119644	
1746583172	1936	1936	Sutter's Gold	Sutter's Gold	Cruze, James	James Cruze	tt0028329	
1746561379	1932	1932	Jewel Robbery	Jewel Robbery	Dieterle, William	William Dieterle	tt0023074	
1746577442	1959	1959	Attack of the Jungle Women	Attack of the Jungle Women	Juliano, Joseph R.	Joseph R. Juliano	tt0052588	
1746577601	1936	1936	Anthony Adverse	Anthony Adverse	LeRoy, Mervyn	Mervyn LeRoy	tt0027300	

1746533165	1915	1915	The Cotton King	The Cotton King	Eagle, Oscar	Oscar Eagle	tt0005140
1746584923	1940	1940	Our Town	Our Town	Wood, Sam	Sam Wood	tt0032881
1746569959	1957	1957	12 Angry Men	12 Angry Men	Lumet, Sidney	Sidney Lumet	tt0050083
1746530714	1950	1950	I'll Get By	I'll Get By	Sale, Richard	Richard Sale	tt0042589
1746583164	1937	1937	Quality Street	Quality Street	Stevens, George	George Stevens	tt0029454
1746492725	1947	1947	Sepia Cinderella	Sepia Cinderella	Leonard, Arthur	Arthur H. Leonard	tt0039812
1746512192	1915	1915	The Ivory Snuff Box	The Ivory Snuff Box	Tourneur, Maurice	Maurice Tourneur	tt0005558
1746486838	1918	1918	The Wine Girl	The Wine Girl	Paton, Stuart	Stuart Paton	tt0009815
1746570594	1934	1934	Twenty Million Sweethearts	Twenty Million Sweethearts	Enright, Ray	Ray Enright	tt0025920
1746569803	1956	1956	Anything Goes	Anything Goes	Lewis, Robert	Robert Lewis	tt0048954
1746524237	1916	1916	Green Stockings	Green Stockings	North, Wilfrid	Wilfrid North	tt0006742
1746410454	1991	1991	Queens Logic	Queens Logic	Rash, Steve	Steve Rash	tt0102741
1746570280	1952	1952	Wait Till the Sun Shines, Nellie	Wait Till the Sun Shines, Nellie	King, Henry	Henry King	tt0045307
1746566353	1956	1955	Bride of the Monster	Bride of the Monster	Wood, Edward D.	Edward D. Wood Jr.	tt0047898
1746583284	1931	1931	Law of the Rio Grande	Law of the Rio Grande	Sheldon, Forrest	Forrest Sheldon	tt0022060
1746511790	1916	1916	The Honor of Mary Blake	The Honor of Mary Blake	Stevens, Edwin	Edwin Stevens	tt0006823
1745373770	2002	2002	The Emperor's Club	The Emperor's Club	Hoffman, Michael	Michael Hoffman	tt0283530
1746453611	1940	1940	Flight Angels	Flight Angels	Seiler, Lewis	Lewis Seiler	tt0032476
1746584457	1929	1929	His First Command	His First Command	La Cava, Gregory	Gregory La Cava	tt0019987
1746577882	1936	1936	Federal Agent	Federal Agent	Newfield, Sam	Sam Newfield	tt0027613
1746422035	1999	1999	200 Cigarettes	200 Cigarettes	Bramon Garcia, Risa	Risa Bramon Garcia	tt0137338
1746571642	1932	1932	Love Affair	Love Affair	Freeland, Thornton	Thornton Freeland	tt0023153
1746581705	1951	1951	The Kid from Amarillo	The Kid from Amarillo	Nazarro, Ray	Ray Nazarro	tt0043708
1746491835	1947	1947	Forever Amber	Forever Amber	Preminger, Otto	Otto Preminger	tt0039391
1746210069	1970	1969	The Belt and Suspenders Man	The Belt and Suspenders Man	Levy, Donald J.	Donald J. Levy	tt2431590
1746563810	1952	1952	The Merry Widow	The Merry Widow	Bernhardt, Curtis	Curtis Bernhardt	tt0044900
1746576512	1931	1931	Shipmates	Shipmates	Pollard, Harry	Harry A. Pollard	tt0022376
1746562028	1953	1953	The Great Jesse James Raid	The Great Jesse James Raid	Le Borg, Reginald	Reginald Le Borg	tt0045840
1746583036	1939	1939	Mesquite Buckaroo	Mesquite Buckaroo	Webb, Harry S.	Harry S. Webb	tt0031639
1746555091	1954	1953	Hondo	Hondo	Farrow, John	John Farrow	tt0045883
1746576499	1932	1932	One Hour with You	One Hour with You	Lubitsch, Ernst	George Cukor	tt0023303
1746577320	1961	1961	The Parent Trap	The Parent Trap	Swift, David	David Swift	tt0055277
1746581354	1910	1910	The Girl of the Northern Woods	The Girl of the Northern Woods	O'Neil, Barry	Barry O'Neil	tt0343721
1746584648	1930	1930	Rain or Shine	Rain or Shine	Capra, Frank	Frank Capra	tt0021282
1769969562	1911	1911	The Angel of Paradise Ranch	The Angel of Paradise Ranch		Allan Dwan	tt0001478
1746453801	1943	1943	Fighting Frontier	Fighting Frontier	Hillyer, Lambert	Lambert Hillyer	tt0035874
1746208686	1968	1968	Duffy	Duffy	Parrish, Robert	Robert Parrish	tt0062917
1746423557	1996	1996	Hamlet	Hamlet	Branagh, Kenneth	Kenneth Branagh	tt0116477
1746570759	1933	1933	Thrill Hunter	The Thrill Hunter	Seitz, George B.	George B. Seitz	tt0024665
1746576927	1962	1962	The Seducers	The Seducers	Ferguson, Graeme	Graeme Ferguson	tt0056462
1746232925	1967	1967	Casino Royale	Casino Royale	Huston, John; Hughes, Ken; Guest, V	Ken Hughes	tt0061452
1746577664	1933	1933	Myrt and Marge	Myrt and Marge	Boasberg, Al	Al Boasberg	tt0025542
1746491755	1946	1946	Dirty Gertie from Harlem, U.S.A.	Dirty Gertie from Harlem U.S.A.	Williams, Spencer	Spencer Williams	tt0038480
1746436380	1941	1941	Lucky Devils	Lucky Devils	Landers, Lew	Lew Landers	tt0033855
1746514889	1922	1922	Hills of Missing Men	Hills of Missing Men	McGowan, J. P.	J.P. McGowan	tt0013236
1746492085	1948	1948	Lulu Belle	Lulu Belle	Fenton, Leslie	Leslie Fenton	tt0040555
1746569599	1957	1957	The Wayward Girl	The Wayward Girl	Selander, Lesley	Lesley Selander	tt0051183
1745357699	2008	2008	The Rocker	The Rocker	Cattaneo, Peter	Peter Cattaneo	tt1031969
1746561665	1932	1932	No Living Witness	No Living Witness	Hopper, E. Mason	E. Mason Hopper	tt0023276
1746438308	1944	1944	My Gal Loves Music	My Gal Loves Music	Lilley, Edward	Edward C. Lilley	tt0037110
1746567360	1932	1932	But the Flesh Is Weak	But the Flesh Is Weak	Conway, Jack	Jack Conway	tt0022731
1746582353	1929	1928	Ned McCobb's Daughter	Ned McCobb's Daughter	Cowen, William J.	William J. Cowen	tt0019205
1746582363	1950	1950	The Underworld Story	The Underworld Story	Endfield, Cyril	Cy Endfield	tt0043088
1746474981	1944	1944	Henry Aldrich, Boy Scout	Henry Aldrich, Boy Scout	Bennett, Hugh	Hugh Bennett	tt0036909
1746235701	1977	1977	Ruby	Ruby	Harrington, Curtis	Curtis Harrington	tt0076644
1746584395	1940	1940	Torrid Zone	Torrid Zone	Keighley, William	William Keighley	tt0033175
1746584330	1940	1940	The Ghost Comes Home	The Ghost Comes Home	Thiele, William	Wilhelm Thiele	tt0032521

1746580290	1938	1938	Goodbye Broadway	Goodbye Broadway	McCarey, Ray	Ray McCarey	tt0030197
1746504353	1918	1918	A Daughter of the West	A Daughter of the West	Bertram, William	William Bertram	tt0191068
1746419760	1993	1993	The Last Party	The Last Party	Benjamin, Mark ; Levin, Marc	Mark Benjamin	tt0107372
1746566031	1953	1953	Seminole	Seminole	Boetticher, Budd	Budd Boetticher	tt0046294
1746436547	1943	1943	Hail to the Rangers	Hail to the Rangers	Berke, William	William Berke	tt0035961
1746501438	1915	1915	His Wife	His Wife	Platt, George Foster	George Foster Platt	tt0005493
1745302773	2007	2007	Starting Out in the Evening	Starting Out in the Evening	Wagner, Andrew	Andrew Wagner	tt0758784
1746578989	1950	1950	Western Pacific Agent	Western Pacific Agent	Newfield, Sam	Sam Newfield	tt0043126
1746573821	1960	1960	The Dark at the Top of the Stairs	The Dark at the Top of the Stairs	Mann, Delbert	Delbert Mann	tt0053750
1746555041	1957	1957	Footsteps in the Night	Footsteps in the Night	Yarbrough, Jean	Jean Yarbrough	tt0050404
1746525196	1920	1920	The Bromley Case	The Bromley Case	Wall, David	David Wall	tt0187760
1746523344	1923	1923	Anna Christie	Anna Christie	Wray, John Griffith	John Griffith Wray	tt0013834
1746455299	1997	1996	Hard Eight	Hard Eight	Anderson, Paul Thomas	Paul Thomas Anderson	tt0119256
1746580475	1935	1935	Without Regret	Without Regret	Young, Harold	Harold Young	tt0027225
1746580778	1939	1939	The Flying Deuces	The Flying Deuces	Sutherland, A. Edward	A. Edward Sutherland	tt0031322
1746584347	1936	1936	Phantom Patrol	Phantom Patrol	Hutchison, Charles	Charles Hutchison	tt0028098
1746582801	1935	1935	The Girl from 10th Avenue	The Girl from 10th Avenue	Green, Alfred E.	Alfred E. Green	tt0026413
1746235618	1973	1973	Wattstax	Wattstax	Stuart, Mel	Mel Stuart	tt0070902
1746465247	1947	1947	Heartaches	Heartaches	Wrangell, Basil	Basil Wrangell	tt0039452
1746520972	1950	1950	Armored Car Robbery	Armored Car Robbery	Fleischer, Richard	Richard Fleischer	tt0042206
1746453502	1940	1940	The Return of Wild Bill	The Return of Wild Bill	Lewis, Joseph H.	Joseph H. Lewis	tt0032984
1746219578	1971	1971	The Hard Ride	The Hard Ride	Topper, Burt	Burt Topper	tt0065815
1746581392	1961	1961	Pagan Island	Pagan Island	Mahon, Barry	Barry Mahon	tt0054154
1746524498	1910	1910	Rose O' Salem-Town	Rose o' Salem Town	Griffith, D. W.	D.W. Griffith	tt0001388
1745309389	2005	2005	Racing Stripes	Racing Stripes	Du Chau, Frederik	Frederik Du Chau	tt0376105
1746420895	1996	1996	Joe's Apartment	Joe's Apartment	Payson, John	John Payson	tt0116707
1746492247	1948	1948	On an Island with You	On an Island with You	Thorpe, Richard	Richard Thorpe	tt0040665
1746486840	1918	1918	Once to Every Man	Once to Every Man	Hunter, T. Hayes	T. Hayes Hunter	tt0189862
1746577347	1951	1951	Half Angel	Half Angel	Sale, Richard; Dassin, Jules	Richard Sale	tt0043615
1745359638	2008	2008	Hellboy II: The Golden Army	Hellboy II: The Golden Army	del Toro, Guillermo	Guillermo del Toro	tt0411477
1746454437	1940	1940	A Night at Earl Carroll's	A Night at Earl Carroll's	Neumann, Kurt	Kurt Neumann	tt0032843
1746393644	1988	1988	Phantasm II	Phantasm II	Coscarelli, Don	Don Coscarelli	tt0095863
1746583355	1930	1930	Those Who Dance	Those Who Dance	Beaudine, William	William Beaudine	tt0021471
1746424585	1942	1942	Street of Chance	Street of Chance	Hively, Jack	Jack Hively	tt0035388
1746562461	1953	1953	The Moonlighter	The Moonlighter	Rowland, Roy	Roy Rowland	tt0046095
1746532731	1916	1916	The Girl Who Doesn't Know	The Girl Who Doesn't Know	Bartlett, Charles E.	Charles Bartlett	tt0157698
1746554762	1965	1965	Across the River	Across the River	Sharff, Stefan	Stefan Sharff	tt0164319
1746574461	1934	1934	Servants' Entrance	Servants' Entrance	Lloyd, Frank	Frank Lloyd	tt0025766
1746571071	1934	1934	Sing and Like It	Sing and Like It	Seiter, William A.	William A. Seiter	tt0025797
1746584156	1935	1935	Three Kids and a Queen	Three Kids and a Queen	Ludwig, Edward	Edward Ludwig	tt0027096
1746571831	1932	1932	Freaks	Freaks	Browning, Tod	Tod Browning	tt0022913
1746577026	1963	1963	The Best of Cinerama	Best of Cinerama			tt0055789
1746474507	1944	1944	Youth Runs Wild	Youth Runs Wild	Robson, Mark	Mark Robson	tt0037476
1746419395	1992	1992	3 Ninjas	3 Ninjas	Turteltaub, Jon	Jon Turteltaub	tt0103596
1746584283	1935	1935	Texas Terror	Texas Terror	Bradbury, R. N.	Robert N. Bradbury	tt0027087
1746582540	1936	1936	Hell-Ship Morgan	Hell Ship Morgan	Lederman, D. Ross	D. Ross Lederman	tt0027731
1746437395	1941	1941	Blood and Sand	Blood and Sand	Mamoulian, Rouben	Rouben Mamoulian	tt0033405
1746198261	1969	1969	Frustrated Cherie	Frustrated Cherie	Dean, Arthur	Arthur Dean	tt0137619
1746524026	1912	1912	At It Again	At It Again	Sennett, Mack	Mack Sennett	tt0002036
1746582950	1935	1935	Broadway Melody of 1936	Broadway Melody of 1936	Del Ruth, Roy	Roy Del Ruth	tt0026144
1746491981	1945	1945	Marked for Murder	Marked for Murder	Clifton, Elmer	Elmer Clifton	tt0037901
1746467707	1917	1917	The Clock	The Clock	Worthington, William	William Worthington	tt0007805
1746408631	1989	1988	Red Scorpion	Red Scorpion	Zito, Joseph	Joseph Zito	tt0098180
1746233004	1970	1970	Fandango	Fandango	Hayes, John	John Hayes	tt0121266
1769969883	1911	1911	The Pied Piper of Hamelin	The Pied Piper of Hamelin		Theodore Marston	tt0001831
1746523070	1914	1914	The Marked Woman	The Marked Woman	Lund, O. A. C.	O.A.C. Lund	tt0004304
1746574429	1951	1951	Hollywood Story	Hollywood Story	Castle, William	William Castle	tt0043646

1746584868	1939	1939	Television Spy	Television Spy	Dmytryk, Edward	Edward Dmytryk	tt0032009	
1746582911	1936	1936	Don't Gamble with Love	Don't Gamble with Love	Murphy, Dudley	Dudley Murphy	tt0027534	
1746576902	1932	1932	Hidden Valley	Hidden Valley	Bradbury, Robert N.	Robert N. Bradbury	tt0023005	
1746577025	1961	1961	The Misfits	The Misfits	Huston, John	John Huston	tt0055184	
1746584446	1931	1931	The Mad Parade	The Mad Parade	Beaudine, William	William Beaudine	tt0148436	
1746581888	1951	1951	Submarine Command	Submarine Command	Farrow, John	John Farrow	tt0044085	
1746512845	1916	1916	The Man from Nowhere	The Man from Nowhere	Otto, Henry	Henry Otto	tt0007025	
**1746577629**	**1935**	**1935**	**The Three Musketeers**	**The Three Musketeers**	**Lee, Rowland V.**	**Rowland V. Lee**	**tt0027099**	1
1746580426	1935	1935	Happiness C.O.D.	Happiness C.O.D.	Lamont, Charles	Charles Lamont	tt0026453	
1769971139	1969	1969	Swingtail	Swingtail	Shane, Dave	Dave Shane	tt0259021	
1746574725	1935	1935	Here Comes the Band	Here Comes the Band	Sloane, Paul	Paul Sloane	tt0026466	
1746583442	1928	1928	The Wright Idea	The Wright Idea	Hines, Charles	Charles Hines	tt0019597	
1746233631	1968	1968	Planet of the Apes	Planet of the Apes	Schaffner, Franklin J.	Franklin J. Schaffner	tt0063442	
1746235385	1972	1972	Dealing: Or the Berkeley-to-Boston Forty-Brick L	Dealing: Or the Berkeley to Boston Forty Bri	Williams, Paul	Paul Williams	tt0068455	
1746577861	1937	1937	The Red Rope	The Red Rope	Luby, S. Roy	S. Roy Luby	tt0029473	
1746562050	1956	1956	Strange Intruder	Strange Intruder	Rapper, Irving	Irving Rapper	tt0049805	
1746520960	1950	1950	Dynamite Pass	Dynamite Pass	Landers, Lew	Lew Landers	tt0042425	
1746435163	1940	1940	Laddie	Laddie	Hively, Jack	Jack Hively	tt0032683	
1746574835	1937	1937	Empty Holsters	Empty Holsters	Eason, B. Reeves	B. Reeves Eason	tt0028830	
1746582787	1936	1936	Desert Justice	Desert Justice	Williams, Lester	William Berke	tt0027513	
1746583479	1951	1951	Wells Fargo Gunmaster	Wells Fargo Gunmaster	Ford, Philip	Philip Ford	tt0044203	
1746577611	1937	1937	As Good As Married	As Good as Married	Buzzell, Edward	Edward Buzzell	tt0028588	
1746569828	1958	1958	Andy Hardy Comes Home	Andy Hardy Comes Home	Koch, Howard W.	Howard W. Koch	tt0051360	
1746566726	1932	1931	Manhattan Parade	Manhattan Parade	Bacon, Lloyd	Lloyd Bacon	tt0022120	
1746474803	1948	1948	Night Time in Nevada	Night Time in Nevada	Witney, William	William Witney	tt0040644	
1746569800	1956	1956	The First Traveling Saleslady	The First Traveling Saleslady	Lubin, Arthur	Arthur Lubin	tt0049212	
1746583579	1930	1930	The Dancers	The Dancers	Sprague, Chandler	Chandler Sprague	tt0020804	
1746508606	1950	1950	Comanche Territory	Comanche Territory	Sherman, George	George Sherman	tt0042339	
1746582263	1930	1930	Paramount on Parade	Paramount on Parade	Arzner, Dorothy; Brower, Otto; Goul	Dorothy Arzner	tt0021232	
1746421317	1995	1995	Desperado	Desperado	Rodriguez, Robert	Robert Rodriguez	tt0112851	
1746474136	1945	1945	Shadow of Terror	Shadow of Terror	Landers, Lew	Lew Landers	tt0038077	
1746513805	1916	1916	Madame La Presidente	Madame la Presidente	Lloyd, Frank	Frank Lloyd	tt0007000	
1746579060	1950	1950	Where Danger Lives	Where Danger Lives	Farrow, John	John Farrow	tt0043131	
1746574757	1935	1935	Rough Riding Ranger	Rough Riding Ranger	Clifton, Elmer	Elmer Clifton	tt0026952	
1746394416	1993	1993	Bopha!	Bopha!	Freeman, Morgan	Morgan Freeman	tt0106464	
1746424608	1940	1940	West of Pinto Basin	West of Pinto Basin	Luby, S. Roy	S. Roy Luby	tt0033251	
1746582888	1938	1938	Dramatic School	Dramatic School	Sinclair, Robert B.	Robert B. Sinclair	tt0030079	
1746500279	1948	1948	Escape	Escape	Mankiewicz, Joseph L.	Joseph L. Mankiewicz	tt0040325	
1746464975	1946	1946	Temptation	Temptation	Pichel, Irving	Irving Pichel	tt0039015	
1746560792	1932	1932	The Reckoning	The Reckoning	Fraser, Harry	Harry L. Fraser	tt0023381	
1746553454	1964	1964	The Shepherd of the Hills	The Shepherd of the Hills	Parker, Ben	Ben Parker	tt0057492	
1746454078	1941	1941	Tillie the Toiler	Tillie the Toiler	Salkow, Sidney	Sidney Salkow	tt0034294	
1746466834	1917	1917	High Speed	High Speed	Sargent, George L.; Clifton, Elmer	George L. Sargent	tt0008096	
1746580967	1910	1910	Happy Jack, a Hero	Happy Jack, a Hero	Powell, Frank	Frank Powell	tt0362703	
1745309128	2005	2005	Son of the Mask	Son of the Mask	Guterman, Lawrence	Lawrence Guterman	tt0362165	
1746513193	1922	1922	I Am the Law	I Am the Law	Carewe, Edwin	Edwin Carewe	tt0013259	
1746501196	1915	1915	The Impostor	The Impostor	Capellani, Albert	Albert Capellani	tt0005537	
1746524490	1922	1922	For Big Stakes	For Big Stakes	Reynolds, Lynn	Lynn Reynolds	tt0013143	
1746235576	1976	1976	Hot Potato	Hot Potato	Williams, Oscar	Oscar Williams	tt0074646	
1746439990	1995	1995	Something to Talk About	Something to Talk About	Hallström, Lasse	Lasse Hallström	tt0114496	
1745374721	2004	2003	Frankie and Johnny Are Married	Frankie and Johnny Are Married	Pressman, Michael	Michael Pressman	tt0400446	
1746564495	1952	1952	Storm over Tibet	Storm Over Tibet	Marton, Andrew	Andrew Marton	tt0045196	
1746577155	1950	1950	Right Cross	Right Cross	Sturges, John	John Sturges	tt0042894	
1746580324	1937	1937	John Meade's Woman	John Meade's Woman	Wallace, Richard	Richard Wallace	tt0029069	
1746502311	1915	1915	Regeneration	Regeneration	Walsh, R. A.	Raoul Walsh	tt0005960	
1746580884	1936	1936	The Bohemian Girl	The Bohemian Girl	Horne, James W.; Rogers, Charles; Ro	James W. Horne	tt0027376	
1746198125	1966	1965	Kitchen	Kitchen	Warhol, Andy	Andy Warhol	tt0190508	

1746499866	1947	1947	Brute Force	Brute Force	Dassin, Jules	Jules Dassin	tt0039224	
1745372869	2003	2003	Dreamcatcher	Dreamcatcher	Kasdan, Lawrence	Lawrence Kasdan	tt0285531	
1746409505	1990	1990	My Blue Heaven	My Blue Heaven	Ross, Herbert	Herbert Ross	tt0100212	
1746504137	1921	1921	The Highest Law	The Highest Law	Ince, Ralph	Ralph Ince	tt0012280	
1746508504	1925	1925	My Lady's Lips	My Lady's Lips	Hogan, James P.	James P. Hogan	tt0016134	
1746565893	1955	1955	The Prodigal	The Prodigal	Thorpe, Richard	Richard Thorpe	tt0048517	
1745342450	2004	2004	L.A. Twister	L.A. Twister	Pape, Sven	Sven Pape	tt0347368	
1746509553	1925	1925	Introduce Me	Introduce Me	Crone, George J.	George Crone	tt0015949	
1746574930	1935	1935	Skull and Crown	Skull and Crown	Clifton, Elmer	Elmer Clifton	tt0027007	
1746235046	1973	1973	Invasion of the Bee Girls	Invasion of the Bee Girls	Sanders, Denis	Denis Sanders	tt0070222	
1746235012	1980	1980	The Last Married Couple in America	The Last Married Couple in America	Cates, Gilbert	Gilbert Cates	tt0081032	
1746198719	1969	1969	Medium Cool	Medium Cool	Wexler, Haskell	Haskell Wexler	tt0064652	
1746564755	1952	1952	The Pace That Thrills	The Pace That Thrills	Barsha, Leon	Leon Barsha	tt0045006	
1746507736	1927	1927	Stolen Pleasures	Stolen Pleasures	Rosen, Philip E.	Phil Rosen	tt0018448	
1746455566	1998	1998	The Faculty	The Faculty	Rodriguez, Robert	Robert Rodriguez	tt0133751	
1746555906	1955	1955	Foxfire	Foxfire	Pevney, Joseph	Joseph Pevney	tt0048091	
1746513630	1914	1914	Captain Swift	Captain Swift	Lewis, Edgar	Edgar Lewis	tt0003752	
1746580446	1938	1938	Outside of Paradise	Outside of Paradise	Auer, John H.	John H. Auer	tt0030540	
1745342041	2008	2008	The Express	The Express	Fleder, Gary	Gary Fleder	tt0469903	
1746584659	1929	1929	Idaho Red	Idaho Red	De Lacey, Robert	Robert De Lacey	tt0020013	
1746209629	1967	1967	Hell on Wheels	Hell on Wheels	Zens, Will	Will Zens	tt0061757	
1746434852	1998	1997	Niagara, Niagara	Niagara, Niagara	Gosse, Bob	Bob Gosse	tt0119780	
1745271666	2001	2001	Crazy/Beautiful	Crazy/Beautiful	Stockwell, John	John Stockwell	tt0250224	
1746570391	1934	1934	What Every Woman Knows	What Every Woman Knows	La Cava, Gregory	Gregory La Cava	tt0025973	
1746568342	1952	1952	Carbine Williams	Carbine Williams	Thorpe, Richard	Richard Thorpe	tt0044480	
1769971147	1969	1969	Sisters in Leather	Sisters in Leather	Crilly, Spence	Zoltan G. Spencer	tt0064991	
1746508103	1950	1950	Jiggs and Maggie Out West	Jiggs and Maggie Out West	Beaudine, William	William Beaudine	tt0042615	
1746566105	1954	1954	Man with a Million	Man with a Million	Neame, Ronald	Ronald Neame	tt0046072	
1746579049	1960	1960	Why Must I Die?	Why Must I Die?	Del Ruth, Roy	Roy Del Ruth	tt0054474	
1746571967	1931	1931	The White Renegade	White Renegade	Irwin, Jack	Jack Irwin	tt0022557	
1746583647	1928	1928	Freckles	Freckles	Meehan, Leo	James Leo Meehan	tt0018915	
1746501290	1916	1916	Overalls	Overalls	Halloway, Jack	Jack Halloway	tt0163135	
1746566078	1953	1953	Scandal at Scourie	Scandal at Scourie	Negulesco, Jean	Jean Negulesco	tt0046279	
1746585354	1930	1930	The Man From Blankley's	The Man from Blankley's	Green, Alfred E.	Alfred E. Green	tt0021115	
1746520247	1925	1925	Warrior Gap	Warrior Gap	Neitz, Alvin J.	Alan James	tt0177353	
1746454111	1998	1998	Can't Hardly Wait	Can't Hardly Wait	Elfont, Harry ; Kaplan, Deborah	Harry Elfont	tt0127723	
1746563873	1952	1951	Another Man's Poison	Another Man's Poison	Rapper, Irving	Irving Rapper	tt0044364	
1746420234	1988	1988	Bull Durham	Bull Durham	Shelton, Ron	Ron Shelton	tt0094812	
1746532740	1928	1928	Ladies of the Night Club	Ladies of the Night Club	Archainbaud, George	George Archainbaud	tt0019063	
1746555713	1954	1954	Pride of the Blue Grass	Pride of the Blue Grass	Beaudine, William	William Beaudine	tt0047363	
1746569874	1958	1958	Gigi	Gigi	Minnelli, Vincente	Vincente Minnelli	tt0051658	
1746582890	1938	1938	Orphans of the Street	Orphans of the Street	Auer, John H.	John H. Auer	tt0030532	
1746464839	1945	1945	Man Alive	Man Alive	Enright, Ray	Ray Enright	tt0037892	
1746501638	1910	1910	The Marked Time-Table	The Marked Time Table	Griffith, D. W.	D.W. Griffith	tt0001307	
1746566291	1953	1953	Port Sinister	Port Sinister	Daniels, Harold	Harold Daniels	tt0046199	
1746514654	1922	1922	In the Name of the Law	In the Name of the Law	Johnson, Emory	Emory Johnson	tt0013266	
1746439092	1995	1995	Forget Paris	Forget Paris	Crystal, Billy	Billy Crystal	tt0113097	
1746577313	1958	1957	Uncle Vanya	Uncle Vanya	Goetz, John	John Goetz	tt0052335	
1746454939	1995	1995	Waiting to Exhale	Waiting to Exhale	Whitaker, Forest	Forest Whitaker	tt0114885	
1746569844	1957	1957	The Halliday Brand	The Halliday Brand	Lewis, Joseph H.	Joseph H. Lewis	tt0050481	
**1746571065**	**1934**	**1934**	**The White Parade**	**The White Parade**	**Cummings, Irving**	**Irving Cummings**	**tt0025986**	1
1746453792	1943	1943	The Purple V	The Purple V	Sherman, George	George Sherman	tt0036281	
1746578702	1951	1950	Counterspy Meets Scotland Yard	Counterspy Meets Scotland Yard	Friedman, Seymour	Seymour Friedman	tt0178348	
1746570982	1952	1952	The Lion and the Horse	The Lion and the Horse	King, Louis	Louis King	tt0044838	
1745342703	2001	2001	Domestic Disturbance	Domestic Disturbance	Becker, Harold	Harold Becker	tt0249478	
1746509522	1925	1925	Flash o' Lightning	Flash o' Lightning	Maloney, Leo	Leo D. Maloney	tt0132961	
1746514790	1923	1922	Thirty Days	Thirty Days	Cruze, James	James Cruze	tt0013669	

1746393782	1991	1991	Rush	Rush	Zanuck, Lili Fini	Lili Fini Zanuck	tt0102820
1746507744	1950	1950	Gun Crazy	Gun Crazy	Lewis, Joseph H.	Joseph H. Lewis	tt0042530
1746583475	1931	1931	Dugan of the Bad Lands	Dugan of the Badlands	Bradbury, Robert North	Robert N. Bradbury	tt0021822
1746474818	1945	1945	Pan-Americana	Pan Americana	Auer, John H.	John H. Auer	tt0037970
1746577094	1959	1959	Yellowstone Kelly	Yellowstone Kelly	Douglas, Gordon	Gordon Douglas	tt0053457
1746492740	1947	1947	Joe Palooka in the Knockout	Joe Palooka in the Knockout	LeBorg, Reginald	Reginald Le Borg	tt0039514
1746561651	1932	1933	He Learned About Women	He Learned About Women	Corrigan, Lloyd	Lloyd Corrigan	tt0024095
1746433938	1991	1991	Robin Hood: Prince of Thieves	Robin Hood: Prince of Thieves	Reynolds, Kevin	Kevin Reynolds	tt0102798
1746454472	1999	1999	The Deep End of the Ocean	The Deep End of the Ocean	Grosbard, Ulu	Ulu Grosbard	tt0120646
1746566172	1954	1953	Cease Fire	Cease Fire!	Crump, Owen	Owen Crump	tt0045616
1746467827	1917	1917	Alimony	Alimony	Flynn, Emmett J.	Emmett J. Flynn	tt0007623
1746419315	1940	1940	Carolina Moon	Carolina Moon	McDonald, Frank	Frank McDonald	tt0032311
1746423750	1989	1989	Little Monsters	Little Monsters	Greenberg, Richard Alan	Richard Greenberg	tt0097758
1745293167	2006	2006	Conversations with God	Conversations with God	Simon, Stephen	Stephen Deutsch	tt0489682
1746568004	1933	1933	College Coach	College Coach	Wellman, William A.	William A. Wellman	tt0023899
1746486861	1918	1918	Which Woman?	Which Woman?	Browning, Tod; Pollard, Harry	Tod Browning	tt0009793
1746436568	1943	1943	Aerial Gunner	Aerial Gunner	Pine, William H.	William H. Pine	tt0035614
1746198685	1969	1969	The Reivers	The Reivers	Rydell, Mark	Mark Rydell	tt0064886
1746520830	1950	1950	The Big Hangover	The Big Hangover	Krasna, Norman	Norman Krasna	tt0042247
1746580253	1934	1934	Wild Gold	Wild Gold	Marshall, George	George Marshall	tt0025992
1746578813	1962	1962	Premature Burial	Premature Burial	Corman, Roger	Roger Corman	tt0056368
1746499916	1945	1945	You Came Along	You Came Along	Farrow, John	John Farrow	tt0038263
1746524819	1915	1915	The Wheels of Justice	The Wheels of Justice	Marston, Theodore	Theodore Marston	tt0006257
1746424430	1943	1943	The North Star	The North Star	Milestone, Lewis	Lewis Milestone	tt0036217
1745271781	2000	2000	Trixie	Trixie	Rudolph, Alan	Alan Rudolph	tt0162711
1746523436	1910	1910	The Sorrows of the Unfaithful	The Sorrows of the Unfaithful	Griffith, D. W.	D.W. Griffith	tt0001401
1746420883	1996	1996	To Gillian on Her 37th Birthday	To Gillian on Her 37th Birthday	Pressman, Michael	Michael Pressman	tt0117924
1746410739	1994	1994	In the Army Now	In the Army Now	Petrie, Daniel	Daniel Petrie Jr.	tt0110123
1746454011	1943	1943	The Return of the Vampire	The Return of the Vampire	Landers, Lew	Lew Landers	tt0037219
1746498472	1921	1921	The Supreme Passion	The Supreme Passion	Bradley, Samuel	Samuel R. Bradley	tt0167428
1746578764	1960	1960	September Storm	September Storm	Haskin, Byron	Byron Haskin	tt0054290
1746575067	1938	1938	Law of the Texan	Law of the Texan	Clifton, Elmer	Elmer Clifton	tt0030352
1746487852	1918	1918	The Rainbow Trail	The Rainbow Trail	Lloyd, Frank	Frank Lloyd	tt0009532
1745309463	2007	2007	Darfur Now	Darfur Now	Braun, Theodore	Ted Braun	tt0988102
1746394004	1989	1989	Worth Winning	Worth Winning	Mackenzie, Will	Will Mackenzie	tt0098678
1746583757	1931	1931	A Connecticut Yankee	A Connecticut Yankee	Butler, David	David Butler	tt0021759
1746455861	1999	1998	Desert Blue	Desert Blue	Freeman, Morgan J.	Morgan J. Freeman	tt0126261
1746550192	1955	1955	The Return of Jack Slade	The Return of Jack Slade	Schuster, Harold	Harold D. Schuster	tt0048552
1746423325	1999	1999	Ed TV	Edtv	Howard, Ron	Ron Howard	tt0131369
1746582730	1935	1935	Mississippi	Mississippi	Sutherland, A. Edward	A. Edward Sutherland	tt0026723
1746271146	1980	1980	The Shining	The Shining	Kubrick, Stanley	Stanley Kubrick	tt0081505
1746436333	1943	1942	Hitler--Dead or Alive	Hitler Dead or Alive	Grinde, Nick	Nick Grinde	tt0034857
1746421004	1993	1993	Sleepless in Seattle	Sleepless in Seattle	Ephron, Nora	Nora Ephron	tt0108160
1746454950	1941	1941	Where Did You Get That Girl?	Where Did You Get That Girl?	Lubin, Arthur	Arthur Lubin	tt0034388
1746582415	1937	1937	Doomed at Sundown	Doomed at Sundown	Newfield, Sam	Sam Newfield	tt0028802
1745272199	2000	2000	The Way of the Gun	The Way of the Gun	McQuarrie, Christopher	Christopher McQuarrie	tt0202677
1746582456	1937	1937	Wild Money	Wild Money	King, Louis	Louis King	tt0029777
1746258156	1976	1976	The Gumball Rally	The Gumball Rally	Bail, Chuck	Charles Bail	tt0074597
1746567982	1933	1933	Murder on the Campus	Murder on the Campus	Thorpe, Richard	Richard Thorpe	tt0025533
1746580566	1937	1936	Polygamy	Polygamy	Carlyle, Patrick	Pat Carlyle	tt0031475
1746580760	1937	1937	The Law Commands	The Law Commands	Nigh, William	William Nigh	tt0029122
1746420120	1988	1988	Ernest Saves Christmas	Ernest Saves Christmas	Cherry, John	John R. Cherry III	tt0095107
1746464932	1943	1943	Stage Door Canteen	Stage Door Canteen	Borzage, Frank	Frank Borzage	tt0036384
1746424458	1941	1941	Scattergood Meets Broadway	Scattergood Meets Broadway	Cabanne, Christy	Christy Cabanne	tt0033401
1746514763	1916	1916	Silas Marner	Silas Marner	Warde, Ernest	Ernest C. Warde	tt0012684
1746513651	1915	1915	The Climbers	The Climbers	O'Neil, Barry	Barry O'Neil	tt0005101
1746475985	1945	1945	High Powered	High Powered	Berke, William	William Berke	tt0037777

1746582689	1934	1934	Beyond the Law	Beyond the Law	Lederman, D. Ross	D. Ross Lederman	tt0024884
1769971088	1969	1968	Odd Triangle	Odd Triangle	Sarno, Joseph W.	Joseph W. Sarno	tt0063375
1745282670	2006	2005	The Mostly Unfabulous Social Life of Ethan Greei	The Mostly Unfabulous Social Life of Ethan (	Bamber, George	George Bamber	tt0424287
1746260483	1980	1980	The Nude Bomb	The Nude Bomb	Donner, Clive	Clive Donner	tt0081249
1746580478	1935	1935	Special Agent	Special Agent	Keighley, William	William Keighley	tt0027029
1746502270	1917	1917	The Rainbow Girl	The Rainbow Girl	Sturgeon, Rollin S.	Rollin S. Sturgeon	tt0008492
1746560491	1932	1932	Roar of the Dragon	Roar of the Dragon	Ruggles, Wesley	Wesley Ruggles	tt0023407
1746394177	1985	1984	The Mutilator	The Mutilator	Cooper, Buddy	Buddy Cooper	tt0124014
1746439216	1997	1997	Breakdown	Breakdown	Mostow, Jonathan	Jonathan Mostow	tt0118771
1746435857	2000	2000	Frequency	Frequency	Hoblit, Gregory	Gregory Hoblit	tt0186151
1746248850	1974	1974	The Take	Axe	Hartford-Davis, Robert	Frederick R. Friedel	tt0075710
1746570828	1934	1934	The Mystery of Mr. X	The Mystery of Mr. X	Selwyn, Edgar	Edgar Selwyn	tt0025546
1746580307	1935	1935	Paradise Canyon	Paradise Canyon	Pierson, Carl L.	Carl Pierson	tt0026846
1745303097	2007	2007	Illegal Tender	Illegal Tender	Reyes, Franc.	Franc. Reyes	tt0775488
1769970757	1912	1912	The Rivals	The Rivals		Mack Sennett	tt0002464
1746516314	1922	1922	Wildcat Jordan	Wildcat Jordan	Santell, Al	Alfred Santell	tt0013767
1746582067	1928	1928	The Price of Fear	The Price of Fear	Jason, Leigh	Leigh Jason	tt0019294
1746199815	1966	1966	Paradise--Hawaiian Style	Paradise, Hawaiian Style	Moore, Michael	Michael D. Moore	tt0059563
1746576962	1959	1959	Cast a Long Shadow	Cast a Long Shadow	Carr, Thomas	Thomas Carr	tt0052681
1746574937	1935	1935	Harmony Lane	Harmony Lane	Santley, Joseph	Joseph Santley	tt0026455
1746465329	1944	1944	The Negro Soldier	The Negro Soldier	Heisler, Stuart	Stuart Heisler	tt0139491
1745374932	2010	2010	Remember Me	Remember Me	Coulter, Allen	Allen Coulter	tt1403981
1746503665	1922	1922	White Shoulders	White Shoulders	Forman, Tom	Tom Forman	tt0013761
1746561015	1932	1932	The Roadhouse Murder	The Roadhouse Murder	Ruben, J. Walter	J. Walter Ruben	tt0023406
1745309528	2007	2007	Saw IV	Saw IV	Bousman, Darren Lynn	Darren Lynn Bousman	tt0890870
1746570000	1956	1956	Teenage Rebel	Teenage Rebel	Goulding, Edmund	Edmund Goulding	tt0049831
1746514727	1916	1916	The Scarlet Woman	The Scarlet Woman	Lawrence, Edmund	Edmund Lawrence	tt0007304
1746572365	1932	1932	Sky Devils	Sky Devils	Sutherland, Edward	A. Edward Sutherland	tt0023485
1746584735	1928	1928	Gang War	Gang War	Glennon, Bert	Bert Glennon	tt0018926
1746219426	1976	1976	The Outlaw Josey Wales	The Outlaw Josey Wales	Eastwood, Clint	Clint Eastwood	tt0075029
1746555729	1955	1955	The Americano	The Americano	Castle, William	William Castle	tt0047820
1746500005	1924	1924	Blasted Hopes	Blasted Hopes	Rosson, Arthur	Arthur Rosson	tt0014722
1746584984	1940	1940	Doomed to Die	Doomed to Die	Nigh, William	William Nigh	tt0032404
1746580234	1935	1935	The Mystery Man	The Mystery Man	McCarey, Raymond	Ray McCarey	tt0026756
1746465123	1942	1942	Pierre of the Plains	Pierre of the Plains	Seitz, George B.	George B. Seitz	tt0035190
1746423729	1940	1940	Girl from God's Country	Girl from God's Country	Salkow, Sidney	Sidney Salkow	tt0032526
1746580536	1935	1935	The Melody Lingers On	The Melody Lingers On	Burton, David	David Burton	tt0026696
1746578656	1959	1959	The Last Mile	The Last Mile	Koch, Howard W.	Howard W. Koch	tt0052992
1746563490	1952	1952	Sound Off	Sound Off		Richard Quine	tt0045181
1746435176	1942	1942	Wild Bill Hickok Rides	Wild Bill Hickok Rides	Enright, Ray	Ray Enright	tt0034392
1746577980	1936	1936	The Gun Ranger	The Gun Ranger	Bradbury, Robert N.	Robert N. Bradbury	tt0028968
1746574560	1933	1933	Blood Money	Blood Money	Brown, Rowland	Rowland Brown	tt0023818
1746497242	1919	1919	The Roaring Road	The Roaring Road	Cruze, James	James Cruze	tt0010635
1746577350	1960	1960	Heller in Pink Tights	Heller in Pink Tights	Cukor, George	George Cukor	tt0053902
1746577963	1935	1935	We're Only Human	We're Only Human	Flood, James	James Flood	tt0027187
1746491816	1947	1947	The Secret Life of Walter Mitty	The Secret Life of Walter Mitty	McLeod, Norman Z.	Norman Z. McLeod	tt0039808
1746487161	1919	1919	All of a Sudden Norma	All of a Sudden Norma	Hickman, Howard	Howard C. Hickman	tt0140800
1746492054	1945	1945	See My Lawyer	See My Lawyer	Cline, Eddie	Edward F. Cline	tt0038065
1746491888	1948	1948	The Black Arrow	The Black Arrow	Douglas, Gordon; Hiecke, Carl	Gordon Douglas	tt0040166
1746474406	1945	1945	Roughly Speaking	Roughly Speaking	Curtiz, Michael	Michael Curtiz	tt0038038
1746409929	1986	1986	Down by Law	Down by Law	Jarmusch, Jim	Jim Jarmusch	tt0090967
1746585016	1939	1939	Sky Patrol	Sky Patrol	Bretherton, Howard	Howard Bretherton	tt0033060
1746512212	1917	1917	An Even Break	An Even Break	Hillyer, Lambert	Lambert Hillyer	tt0007903
1746507819	1925	1925	That Man Jack	That Man Jack!	Craft, William J.	William James Craft	tt0016418
1746553975	1964	1964	Ensign Pulver	Ensign Pulver	Logan, Joshua	Joshua Logan	tt0058060
1746393098	1986	1986	The Clan of the Cave Bear	The Clan of the Cave Bear	Chapman, Michael	Michael Chapman	tt0090848
1746582895	1937	1937	The Great Hospital Mystery	The Great Hospital Mystery	Tinling, James	James Tinling	tt0028774

1746582182	1951	1951	You're in the Navy Now	You're in the Navy Now	Hathaway, Henry	Henry Hathaway	tt0044224
1746585018	1939	1939	Calling Dr. Kildare	Calling Dr. Kildare	Bucquet, Harold S.	Harold S. Bucquet	tt0031133
1746438157	1943	1942	Commandos Strike at Dawn	Commandos Strike at Dawn	Farrow, John	John Farrow	tt0035746
1746437838	1942	1942	Twin Beds	Twin Beds	Whelan, Tim	Tim Whelan	tt0035479
1746515583	1922	1922	Wildness of Youth	Wildness of Youth	Abramson, Ivan	Ivan Abramson	tt0013768
1746394383	1985	1985	My Science Project	My Science Project	Betuel, Jonathan R.	Jonathan R. Betuel	tt0089652
1746218791	1970	1970	Myra Breckinridge	Myra Breckinridge	Sarne, Michael	Michael Sarne	tt0066115
1746408826	1985	1985	Volunteers	Volunteers	Meyer, Nicholas	Nicholas Meyer	tt0090274
1746501592	1922	1922	The Infidel	The Infidel	Young, James	James Young	tt0013267
1746438785	1995	1995	Canadian Bacon	Canadian Bacon	Moore, Michael	Michael Moore	tt0109370
1746208423	1969	1969	Turn On to Love	Turn on to Love	Avildsen, John G.	John G. Avildsen	tt0065131
1746523328	1927	1927	Down the Stretch	Down the Stretch	Baggot, King	King Baggot	tt0017824
1746504545	1919	1919	The Ace of the Saddle	Ace of the Saddle	Ford, Jack	John Ford	tt0009859
1746585274	1928	1928	Divine Sinner	The Divine Sinner	Pembroke, Scott	Scott Pembroke	tt0018835
1746564442	1952	1952	Chained for Life	Chained for Life	Fraser, Harry L.	Harry L. Fraser	tt0043397
1746523710	1914	1914	Threads of Destiny	Threads of Destiny	Smiley, Joseph W.	Joseph W. Smiley	tt0004695
1745359603	2001	2001	The Bread, My Sweet	The Bread, My Sweet	Martin, Melissa	Melissa Martin	tt0281680
1746502396	1916	1916	Saint, Devil and Woman	Saint, Devil and Woman	Sullivan, Frederic	Frederick Sullivan	tt0158909
1746514441	1910	1910	The Tide of Fortune	The Tide of Fortune		Harry Solter	tt0329684
1746578974	1958	1958	The Roots of Heaven	The Roots of Heaven	Huston, John	John Huston	tt0052148
1746582502	1937	1937	Range Defenders	Range Defenders	Wright, Mack V.	Mack V. Wright	tt0029462
1746580454	1937	1937	Round-Up Time in Texas	Round Up Time in Texas	Kane, Joseph	Joseph Kane	tt0029501
1746532755	1914	1914	The Truth Wagon	The Truth Wagon	Figman, Max	Max Figman	tt0004734
1746248436	1968	1968	A Time To Sing	A Time to Sing	Dreifuss, Arthur	Arthur Dreifuss	tt0063698
1746409807	1982	1982	48 Hrs.	48 Hrs.	Hill, Walter	Walter Hill	tt0083511
1746464871	1944	1944	Swing in the Saddle	Swing in the Saddle	Landers, Lew	Lew Landers	tt0037338
1746516526	1920	1920	In the Shadow of the Dome	In the Shadow of the Dome	Fischer, David G.	David G. Fischer	tt0195831
1746514824	1915	1915	An Affair of Three Nations	An Affair of Three Nations	Daly, Arnold; Miller, Ashley	Ashley Miller	tt0004861
1746408650	1986	1986	8 Million Ways to Die	8 Million Ways to Die	Ashby, Hal	Hal Ashby	tt0090568
1746435166	1941	1941	Bowery Blitzkrieg	Bowery Blitzkrieg	Fox, Wallace	Wallace Fox	tt0033428
1746574502	1952	1952	Million Dollar Mermaid	Million Dollar Mermaid	LeRoy, Mervyn	Mervyn LeRoy	tt0044903
1746436996	1942	1942	Panama Hattie	Panama Hattie	McLeod, Norman Z.	Norman Z. McLeod	tt0035170
1746520577	1926	1926	The Arizona Sweepstakes	Arizona Sweepstakes	Smith, Clifford S.	Clifford Smith	tt0016610
1746269696	1976	1975	Psychic Killer	Psychic Killer	Danton, Raymond	Ray Danton	tt0073586
1746235470	1978	1978	Coming Home	Coming Home	Ashby, Hal	Hal Ashby	tt0077362
1746486849	1920	1920	The Blue Moon	The Blue Moon	Cox, George L.	George L. Cox	tt0186889
1746235458	1980	1980	The Big Red One	The Big Red One	Fuller, Samuel	Samuel Fuller	tt0080437
1746571004	1952	1952	The Belle of New York	The Belle of New York	Walters, Charles	Charles Walters	tt0044408
1746410139	1990	1990	Another 48 Hrs.	Another 48 Hrs.	Hill, Walter	Walter Hill	tt0099044
1745282630	2005	2005	You Think You Really Know Me: The Gary Wilson	You Think You Really Know Me: The Gary Wi	Wolk, Michael	Michael Wolk	tt0458529
1746569623	1954	1954	Three Coins in the Fountain	Three Coins in the Fountain	Negulesco, Jean	Jean Negulesco	tt0047580
1746200348	1968	1967	Countdown	Countdown	Altman, Robert	Robert Altman	tt0062827
1746571002	1933	1933	Hoop-la	Hoopla	Lloyd, Frank	Frank Lloyd	tt0024139
1746575069	1933	1933	Easy Millions	Easy Millions	Newmeyer, Fred	Fred C. Newmeyer	tt0023976
1745340302	2001	2001	Exit Wounds	Exit Wounds	Bartkowiak, Andrzej	Andrzej Bartkowiak	tt0242445
1746514817	1910	1910	Never Again	Never Again	Powell, Frank	Frank Powell	tt0001334
1746520685	1950	1950	I Was a Shoplifter	I Was a Shoplifter	Lamont, Charles	Charles Lamont	tt0042588
1746584328	1936	1936	Two in Revolt	Two in Revolt	Tryon, Glenn	Glenn Tryon	tt0028431
1746567279	1932	1932	If I Had a Million	If I Had a Million		James Cruze	tt0023049
1746393254	1988	1988	The Last Temptation of Christ	The Last Temptation of Christ	Scorsese, Martin	Martin Scorsese	tt0095497
1746183411	1910	1910	Blue Horse Mine	The Blue Horse Mine			tt0348552
1746440003	1996	1995	The Celluloid Closet	The Celluloid Closet	Epstein, Rob; Friedman, Jeffrey	Rob Epstein	tt0112651
1746583453	1950	1950	Outside the Wall	Outside the Wall	Wilbur, Crane	Crane Wilbur	tt0042826
1746454041	1942	1942	Always in My Heart	Always in My Heart	Graham, Jo	Jo Graham	tt0034454
1746562234	1953	1953	Desert Legion	Desert Legion	Pevney, Joseph	Joseph Pevney	tt0045678
1746523306	1916	1916	I Accuse	I Accuse	Haddock, William F.	William F. Haddock	tt0145829
1746464842	1941	1941	Unholy Partners	Unholy Partners	LeRoy, Mervyn	Mervyn LeRoy	tt0034342

1746409706	1991	1991	The Dark Backward	The Dark Backward	Rifkin, Adam	Adam Rifkin	tt0101660
1746583467	1931	1931	Trails of the Golden West	Trails of the Golden West	De Cordova, Leander	Leander De Cordova	tt0022498
1746409808	1985	1985	Code of Silence	Code of Silence	Davis, Andrew	Andrew Davis	tt0088936
1746560065	1965	1965	Rat Fink	Rat Fink	Landis, James	James Landis	tt0059636
1746497041	1922	1922	Kid Canfield the Reform Gambler	Kid Canfield, the Reform Gambler		Kid Canfield	tt0332670
1746487870	1921	1921	The Simp	The Simp		Ferris Hartman	tt0977827
1746487301	1918	1918	Shifting Sands	Shifting Sands	Parker, Albert		tt2823094
1746524180	1915	1915	The Magic Toy Maker	The Magic Toy Maker	Kingsley, Pierce	Pierce Kingsley	tt0005686
1746408638	1984	1983	Signals Through the Flames	Signals Through the Flames	Rochlin, Sheldon; Harris, Maxine	Sheldon Rochlin	tt0235764
1746218918	1976	1976	The Amazing Dobermans	The Amazing Dobermans	Chudnow, Byron Ross	Byron Chudnow	tt0074130
1746258204	1975	1974	TNT Jackson	TNT Jackson	Santiago, Cirio	Cirio H. Santiago	tt0072245
1746583364	1951	1951	St. Benny the Dip	St. Benny the Dip	Ulmer, Edgar G.	Edgar G. Ulmer	tt0044067
1746530786	1949	1948	The Quiet One	The Quiet One	Meyers, Sidney	Sidney Meyers	tt0040717
1746520851	1949	1949	The Mutineers	The Mutineers	Yarbrough, Jean	Jean Yarbrough	tt0041670
1746520941	1949	1949	Frontier Investigator	Frontier Investigator	Brannon, Fred C.	Fred C. Brannon	tt0041392
1746435156	1943	1943	Find the Blackmailer	Find the Blackmailer	Lederman, D. Ross	D. Ross Lederman	tt0035879
1746453497	1942	1942	The World at War	The World at War		Lowell Mellett	tt0185016
1746523815	1927	1928	Framed	Framed	Brabin, Charles J.	Bruce Mitchell	tt0349464
1746500729	1925	1925	On the Go	On the Go	Thorpe, Richard	Benjamin Stoloff	tt0867373
1746509360	1924	1924	Trouble Trail	Trouble Trail	Holt, George	George Holt	tt0174285
1746584089	1940	1940	The Last Alarm	The Last Alarm	West, William	William West	tt0032690
1746454469	1940	1940	Ridin' the Trail	Ridin' the Trail	Johnson, Raymond K.	Bernard B. Ray	tt0134056
1746584375	1940	1940	Land of the Six Guns	Land of the Six Guns	Johnson, Raymond K.	Bernard B. Ray	tt0032689
1746582887	1939	1939	Straight to Heaven	Straight to Heaven	Leonard, Arthur	Arthur H. Leonard	tt0031984
1746580738	1938	1938	Making the Headlines	Making the Headlines	Collins, Lewis D.	Lewis D. Collins	tt0030405
1746577917	1937	1937	Whistling Bullets	Whistling Bullets	English, John W.	John English	tt0029768
1746580636	1937	1938	Race Suicide	Race Suicide	Luby, S. Roy	S. Roy Luby	tt0030936
1746580427	1936	1936	Tango	Tango	Rosen, Phil	Phil Rosen	tt0028343
1746574732	1936	1936	West of Nevada	West of Nevada	Hill, Robert	Robert F. Hill	tt0028491
1746577763	1936	1936	Rebellion	Rebellion	Shores, Lynn	Lynn Shores	tt0028162
1746576957	1958	1958	Street of Darkness	Street of Darkness	Walker, Robert	Robert G. Walker	tt0152608
1746199519	1969	1969	Journey Into Self	Journey Into Self		Bill McGaw	tt0063160
1769970489	1967	1967	Diary of a Swinger	Diary of a Swinger	Amero, John; Amero, Lem	John Amero	tt0135921
1746185901	1967	1967	Himself as Herself	Himself as Herself	Markopoulos, Gregory J.	Gregory J. Markopoulos	tt0195782
1746185142	1965	1964	Goldstein	Goldstein	Kaufman, Philip; Manaster, Benjami	Philip Kaufman	tt0144947
1746571242	1935	1935	Border Vengeance	Border Vengeance	Heinz, Ray	Ray Heinz	tt0176564
1746394546	1992	1991	American Fabulous	American Fabulous	Dakota, Reno	Reno Dakota	tt0103669
1746433683	1989	1989	The 'Burbs	The 'Burbs	Dante, Joe	Joe Dante	tt0096734
1746198931	1968	1967	Good Morning, and Goodbye!	Good Morning... and Goodbye!	Meyer, Russ	Russ Meyer	tt0061719
1746465294	1945	1945	My Name Is Julia Ross	My Name Is Julia Ross	Lewis, Joseph H.	Joseph H. Lewis	tt0037932
1746585166	1930	1930	The Matrimonial Bed	The Matrimonial Bed	Curtiz, Michael	Michael Curtiz	tt0021132
1746554123	1963	1963	The Raven	The Raven	Corman, Roger	Roger Corman	tt0057449
1746509358	1924	1924	The Valley of Vanishing Men	Valley of Vanishing Men	Hart, Neal	Neal Hart	tt0176279
1769975293	1916	1916	A Telegraphic Tangle	A Telegraphic Tangle	Drew, Sidney	Sidney Drew	tt0155248
1746580763	1935	1935	Smokey Smith	Smokey Smith	Bradbury, Robert N.	Robert N. Bradbury	tt0027015
1746582826	1936	1936	The Amazing Exploits of the Clutching Hand	The Amazing Exploits of the Clutching Hand	Herman, Albert	Albert Herman	tt0027452
1746500026	1948	1948	Son of God's Country	Son of God's Country	Springsteen, R. G.	R.G. Springsteen	tt0040819
1746566202	1954	1954	Beachhead	Beachhead	Heisler, Stuart	Stuart Heisler	tt0046757
1746569864	1958	1958	The Fly	The Fly	Neumann, Kurt	Kurt Neumann	tt0051622
1746436187	1995	1995	Mortal Kombat	Mortal Kombat	Anderson, Paul	Paul W.S. Anderson	tt0113855
1746524212	1910	1910	Pressed Roses	Pressed Roses		Harry Solter	tt0200973
1746583948	1930	1930	Dangerous Nan McGrew	Dangerous Nan McGrew	St. Clair, Malcolm	Malcolm St. Clair	tt0020809
1746500271	1926	1925	Here He Comes	Here He Comes	Corby, Travers	Travers Colby	tt0356688
1746580317	1938	1938	Mr. Chump	Mr. Chump	Clemens, William	William Clemens	tt0030467
1746500615	1926	1926	The Road to Broadway	The Road to Broadway	Mitchell, Howard	Howard M. Mitchell	tt0156968
1746217016	1968	1968	Prowl Girls	Prowl Girls		Barry Mahon	tt0255519
1746504744	1921	1921	School Days	School Days	Nigh, William	William Nigh	tt0162635

1746581519	1910	1910	The Girls of the Range	The Girls of the Range		Francis Boggs	tt1390944
1746435863	1999	1999	Cruel Intentions	Cruel Intentions	Kumble, Roger	Roger Kumble	tt0139134
1746543065	1915	1915	The Captive	The Captive	DeMille, Cecil B.	Cecil B. DeMille	tt0005059
1746565918	1953	1953	Glen or Glenda?	Glen or Glenda	Wood, Edward D.	Edward D. Wood Jr.	tt0045826
1746439222	1998	1998	Another Day in Paradise	Another Day in Paradise	Clark, Larry	Larry Clark	tt0127722
1745342472	2008	2008	Burn After Reading	Burn After Reading	Coen, Ethan; Coen, Joel	Ethan Coen	tt0887883
1746573767	1956	1956	Navy Wife	Navy Wife	Bernds, Edward L.	Edward Bernds	tt0049544
1746577518	1938	1938	I'm from the City	I'm from the City	Holmes, Ben	Ben Holmes	tt0030262
1746485592	1918	1919	Angel Child	Angel Child	Otto, Henry	Henry Otto	tt0182714
1746550310	1955	1955	White Feather	White Feather	Webb, Robert	Robert D. Webb	tt0048805
1746580197	1937	1937	Hot Water	Hot Water	Strayer, Frank R.	Frank R. Strayer	tt0029023
1746208528	1966	1965	Adam Lost His Apple	Adam Lost His Apple	Wainwright, Earl	Earl Wainwright	tt0058874
1746438630	1941	1941	West Point Widow	West Point Widow	Siodmak, Robert	Robert Siodmak	tt0034381
1746453672	1998	1998	Velvet Goldmine	Velvet Goldmine	Haynes, Todd	Todd Haynes	tt0120879
1746491732	1944	1944	Cry of the Werewolf	Cry of the Werewolf	Levin, Henry	Henry Levin	tt0036731
1746584962	1939	1939	The Witness Vanishes	The Witness Vanishes	Garrett, Otis	Otis Garrett	tt0032137
1746218443	1966	1966	Swamp Country	Swamp Country	Patrick, Robert	Robert Patrick	tt0160937
1746410525	1987	1987	Creepshow 2	Creepshow 2	Gornick, Michael	Michael Gornick	tt0092796
1746577250	1951	1951	Kentucky Jubilee	Kentucky Jubilee	Ormond, Ron	Ron Ormond	tt0043705
1746409121	1987	1987	Enemy Territory	Enemy Territory	Manoogian, Peter	Peter Manoogian	tt0092969
1746584429	1939	1939	Spoilers of the Range	Spoilers of the Range	Coleman, C. C.	Charles C. Coleman	tt0031963
1746502548	1917	1917	The Hater of Men	The Hater of Men	Miller, Charles	Charles Miller	tt0008051
1746499854	1947	1947	The Woman on the Beach	The Woman on the Beach	Renoir, Jean	Jean Renoir	tt0040000
1746235387	1973	1973	Coffy	Coffy	Hill, Jack	Jack Hill	tt0069897
1746532737	1923	1923	The Wild Party	The Wild Party	Blaché, Herbert	Herbert Blaché	tt0014616
1746578029	1935	1935	Bars of Hate	Bars of Hate	Herman, Al	Albert Herman	tt0027335
1746584063	1935	1935	Here's to Romance	Here's to Romance	Green, Alfred E.	Alfred E. Green	tt0026467
1746578373	1950	1950	The Vanishing Westerner	The Vanishing Westerner	Ford, Philip	Philip Ford	tt0043098
1746453646	1942	1942	Here We Go Again	Here We Go Again	Dwan, Allan	Allan Dwan	tt0034847
1745309499	2007	2007	Unborn in the USA: Inside the War on Abortion	Unborn in the USA: Inside the War on Abort	Fell, Stephen ; Thompson, Will	Stephen Fell	tt1059238
1746394229	1990	1990	Wild at Heart	Wild at Heart	Lynch, David	David Lynch	tt0100935
1746465197	1945	1944	The Keys of the Kingdom	The Keys of the Kingdom	Stahl, John M.	John M. Stahl	tt0036983
1745302578	2005	2005	The Devil and Daniel Johnston	The Devil and Daniel Johnston	Feuerzeig, Jeff	Jeff Feuerzeig	tt0436231
1746211073	1970	1970	Soldier Blue	Soldier Blue	Nelson, Ralph	Ralph Nelson	tt0066390
1746235066	1972	1972	Get to Know Your Rabbit	Get to Know Your Rabbit	De Palma, Brian	Brian De Palma	tt0068637
1746580624	1938	1938	Too Hot to Handle	Too Hot to Handle	Conroy, Jack	Jack Conway	tt0030879
1746422020	1989	1989	The Fabulous Baker Boys	The Fabulous Baker Boys	Kloves, Steve	Steve Kloves	tt0097322
1746520326	1926	1926	Kosher Kitty Kelly	Kosher Kitty Kelly	Horne, James W.	James W. Horne	tt0017044
1746492460	1947	1947	Cynthia	Cynthia	Leonard, Robert Z.	Robert Z. Leonard	tt0039293
1746578488	1951	1950	Gambling House	Gambling House	Tetzlaff, Ted	Ted Tetzlaff	tt0042496
1746436206	1995	1995	Leaving Las Vegas	Leaving Las Vegas	Figgis, Mike	Mike Figgis	tt0113627
1746585475	1939	1939	Mystery Plane	Mystery Plane	Waggner, George	George Waggner	tt0031703
1745372491	2008	2008	Step Brothers	Step Brothers	McKay, Adam	Adam McKay	tt0838283
1746508692	1925	1925	The Man from Red Gulch	The Man from Red Gulch	Mortimer, Edmund	Edmund Mortimer	tt0016072
1746575050	1936	1936	Secret Patrol	Secret Patrol	Selman, David	David Selman	tt0028232
1746410894	1993	1993	Ghost in the Machine	Ghost in the Machine	Talalay, Rachel	Rachel Talalay	tt0107009
1746574780	1934	1934	Grand Canary	Grand Canary	Cummings, Irving	Irving Cummings	tt0025196
1746408518	1986	1986	Labyrinth	Labyrinth	Henson, Jim	Jim Henson	tt0091369
1746561997	1956	1956	The Great American Pastime	The Great American Pastime	Hoffman, Herman	Herman Hoffman	tt0049277
1746508212	1926	1926	The Lone Wolf Returns	The Lone Wolf Returns	Ince, Ralph	Ralph Ince	tt0017077
1746507908	1927	1927	One Hour of Love	One Hour of Love	Florey, Robert	Robert Florey	tt0018227
1746454038	1942	1942	North to the Klondike	North to the Klondike	Kenton, Erle C.	Erle C. Kenton	tt0035133
1746530787	1949	1949	Crashing Thru	Crashing Thru	Taylor, Ray	Ray Taylor	tt0041265
1746500732	1924	1924	The Millionaire Cowboy	The Millionaire Cowboy	Garson, Harry	Harry Garson	tt0015139
1746208481	1966	1966	Rat Pfink A Boo Boo	Rat Pfink a Boo Boo	Steckler, Ray Dennis	Ray Dennis Steckler	tt0059637
1746561632	1932	1932	Unholy Love	Unholy Love	Ray, Albert	Albert Ray	tt0023641
1746585369	1939	1939	Unmarried	Unmarried	Neumann, Kurt	Kurt Neumann	tt0032081

1746566275	1957	1957	Gunfight at the O.K. Corral	Gunfight at the O.K. Corral	Sturges, John	John Sturges	tt0050468
1746545142	1940	1940	Dancing on a Dime	Dancing on a Dime	Santley, Joseph	Joseph Santley	tt0032377
1746421721	1994	1994	The Chase	The Chase	Rifkin, Adam	Adam Rifkin	tt0109402
1745342834	2009	2008	Under Our Skin	Under Our Skin	Wilson, Andy Abrahams	Andy Abrahams Wilson	tt1202579
1746577097	1958	1958	Twilight for the Gods	Twilight for the Gods	Pevney, Joseph	Joseph Pevney	tt0052326
1746455219	2000	2000	Meet the Parents	Meet the Parents	Roach, Jay	Jay Roach	tt0212338
1746515802	1922	1922	Lure of Gold	Lure of Gold	Hart, Neal	Neal Hart	tt0013346
1746549812	1954	1954	Drive a Crooked Road	Drive a Crooked Road	Quine, Richard	Richard Quine	tt0046935
1746578714	1951	1951	The Day the Earth Stood Still	The Day the Earth Stood Still	Wise, Robert	Robert Wise	tt0043456
1746203027	1970	1970	A Man Called Horse	A Man Called Horse	Silverstein, Elliot	Elliot Silverstein	tt0066049
1746580300	1936	1936	The Captain's Kid	The Captain's Kid	Grinde, Nick	Nick Grinde	tt0028688
1745309113	2006	2005	Pizza	Pizza	Christopher, Mark	Mark Christopher	tt0376827
1746524527	1923	1923	Wolf Tracks	Wolf Tracks	North Bradbury, Robert	Robert N. Bradbury	tt0011871
1746516316	1921	1921	Be My Wife	Be My Wife	Linder, Max	Max Linder	tt0011948
1746584732	1929	1929	Fox Movietone Follies of 1929	Fox Movietone Follies of 1929	Butler, David	David Butler	tt0019896
1746454160	1999	1999	My Favorite Martian	My Favorite Martian	Petrie, Donald	Donald Petrie	tt0120764
1746199025	1969	1969	The Model Shop	Model Shop	Demy, Jacques	Jacques Demy	tt0064679
1746475260	1947	1947	The Fabulous Dorseys	The Fabulous Dorseys	Green, Alfred E.	Alfred E. Green	tt0039365
1746437400	1940	1940	Dulcy	Dulcy	Simon, S. Sylvan	S. Sylvan Simon	tt0032422
1746507831	1925	1925	Scandal Street	Scandal Street	Bennett, Whitman	Whitman Bennett	tt0016316
1746524199	1915	1915	Camille	Camille	Capellani, Albert	Albert Capellani	tt0005049
1746422149	1994	1994	Baby's Day Out	Baby's Day Out	Johnson, Patrick Read	Patrick Read Johnson	tt0109190
1745303023	2007	2006	Walking to Werner	Walking to Werner	Phillips, Linas	Linas Phillips	tt0798898
1746549734	1955	1955	An Annapolis Story	An Annapolis Story	Siegel, Don	Don Siegel	tt0047835
1746455710	1998	1997	Inside/Out	Inside Out	Tregenza, Rob	Jason Gould	tt0119372
1746576188	1932	1932	Escapade	Escapade	Thorpe, Richard	Richard Thorpe	tt0022861
1746584033	1937	1937	Riding on Air	Riding on Air	Sedgwick, Edward	Edward Sedgwick	tt0029488
1746508072	1948	1948	Strange Gamble	Strange Gamble	Archainbaud, George	George Archainbaud	tt0040837
1746248387	1966	1966	Music City U. S. A.	Music City U.S.A.	Collins, Preston; Dinet, James	Preston Collins	tt1487981
1746437543	2000	1999	Hot Wax Zombies on Wheels	Hot Wax Zombies on Wheels	Roush, Mike J.	Michael Roush	tt0160376
1746513036	1915	1915	The Girl of the Golden West	The Girl of the Golden West	DeMille, Cecil B.	Cecil B. DeMille	tt0005393
1746465002	1947	1947	Last Days of Boot Hill	Last Days of Boot Hill	Nazarro, Ray	Ray Nazarro	tt0039551
1746584107	1938	1938	Law of the Plains	Law of the Plains	Nelson, Sam	Sam Nelson	tt0030351
1746573957	1958	1958	Satan's Satellites	Satan's Satellites	Brannon, Fred C.	Fred C. Brannon	tt0052160
1746209809	1970	1969	Mondo Trasho	Mondo Trasho	Waters, John	John Waters	tt0064683
1746566987	1932	1932	Stowaway	Stowaway	Whitman, Phil	Phil Whitman	tt0023518
1746410185	1992	1992	Captain Ron	Captain Ron	Eberhardt, Thom	Thom Eberhardt	tt0103924
1746582149	1930	1930	A Devil With Women	A Devil with Women	Cummings, Irving	Irving Cummings	tt0020822
1745342265	2009	2009	Laid to Rest	Laid to Rest	Hall, Robert	Robert Hall	tt1228933
1769970409	1965	1965	Nympho	Nympho	Nicola, Alfredo	Nick Millard	tt1468867
1746573584	1932	1932	Polly of the Circus	Polly of the Circus	Santell, Alfred	Alfred Santell	tt0023349
1746218678	1974	1974	Black Belt Jones	Black Belt Jones	Clouse, Robert	Robert Clouse	tt0071221
1746495629	1918	1918	Old Wives for New	Old Wives for New	DeMille, Cecil B.	Cecil B. DeMille	tt0009440
1746572976	1931	1931	Sporting Chance	Sporting Chance	Herman, Albert	Albert Herman	tt0129392
1746438184	1942	1942	Get Hep to Love	Get Hep to Love	Lamont, Charles	Charles Lamont	tt0034784
1746500887	1916	1916	The Eternal Grind	The Eternal Grind	O'Brien, John B.	John B. O'Brien	tt0006637
1746501351	1915	1915	The Slim Princess	The Slim Princess	Calvert, E. H.	E.H. Calvert	tt0006059
1746584915	1940	1940	Escape	Escape	LeRoy, Mervyn	Mervyn LeRoy	tt0032447
1746454294	1942	1942	Prisoner of Japan	Prisoner of Japan	Ripley, Arthur	Arthur Ripley	tt0035217
1746585443	1939	1939	Charlie Chan in Reno	Charlie Chan in Reno	Foster, Norman	Norman Foster	tt0031149
1746484087	1918	1918	Her Decision	Her Decision	Conway, Jack	Jack Conway	tt0009161
1746235664	1974	1974	The Parallax View	The Parallax View	Pakula, Alan J.	Alan J. Pakula	tt0071970
1746582920	1938	1938	The Black Doll	The Black Doll	Garrett, Otis	Otis Garrett	tt0029918
1746554885	1957	1957	Carnival Rock	Carnival Rock	Corman, Roger	Roger Corman	tt0050230
1746555217	1956	1956	Battle Stations	Battle Stations	Seiler, Lewis	Lewis Seiler	tt0048989
1746584453	1929	1929	The Unholy Night	The Unholy Night	Barrymore, Lionel	Lionel Barrymore	tt0020534
1745282668	2005	2005	Harry Potter and the Goblet of Fire	Harry Potter and the Goblet of Fire	Newell, Mike	Mike Newell	tt0330373

1746562207	1954	1954	The Egyptian	The Egyptian	Curtiz, Michael	Michael Curtiz	tt0046949	
1746454029	1941	1941	Lone Star Law Men	Lone Star Law Men	Tansey, Robert	Robert Emmett Tansey	tt0033847	
1746584186	1940	1940	A Dispatch from Reuters	A Dispatch from Reuters	Dieterle, William	William Dieterle	tt0032396	
1746572453	1931	1931	The Reckless Hour	The Reckless Hour	Dillon, John Francis	John Francis Dillon	tt0022297	
1746453756	1941	1941	Riot Squad	Riot Squad	Finney, Edward	Edward Finney	tt0034111	
1746271328	1980	1979	The Day Time Ended	The Day Time Ended	Cardos, John "Bud"	John 'Bud' Cardos	tt0080596	
1746577396	1960	1960	Cimarron	Cimarron	Mann, Anthony	Anthony Mann	tt0053715	
1769969511	1911	1911	Bar Z's New Cook	Bar Z's New Cook		Thomas H. Ince	tt0001807	
1746410402	1990	1990	I Come in Peace	I Come in Peace	Baxley, Craig R.	Craig R. Baxley	tt0099817	
1769970640	1968	1968	Like It Is	Like It Is	Rotsler, William	William Rotsler	tt0143392	
1746508406	1925	1925	A Fight to the Finish	A Fight to the Finish	Eason, Reeves	B. Reeves Eason	tt0015805	
1745395382	2008	2008	Appaloosa	Appaloosa	Harris, Ed	Ed Harris	tt0800308	
1746574760	1933	1933	The Billion Dollar Scandal	The Billion Dollar Scandal	Brown, Harry Joe	Harry Joe Brown	tt0023811	
1746465103	1945	1945	Without Love	Without Love	Bucquet, Harold S.	Harold S. Bucquet	tt0038256	
1746582220	1928	1928	A Son of the Desert	A Son of the Desert	McCormick, William Merrill	Merrill McCormick	tt0165487	
1745302898	2006	2006	Flannel Pajamas	Flannel Pajamas	Lipsky, Jeff	Jeff Lipsky	tt0432290	
1746484436	1917	1917	The Spindle of Life	The Spindle of Life	Cochrane, George	George Cochrane	tt0008616	
1746474945	1944	1944	Experiment Perilous	Experiment Perilous	Tourneur, Jacques	Jacques Tourneur	tt0036807	
1746580542	1937	1937	Oh, Doctor	Oh, Doctor	McCarey, Raymond B.	Ray McCarey	tt0029333	
1746580371	1937	1937	Harlem on the Prairie	Harlem on the Prairie	Newfield, Sam	Sam Newfield	tt0028978	
1746503949	1918	1918	De Luxe Annie	De Luxe Annie	West, Roland	Roland West	tt0008998	
1746454662	1941	1941	Harvard, Here I Come	Harvard, Here I Come!	Landers, Lew	Lew Landers	tt0034831	
1746524609	1928	1928	Lady Raffles	Lady Raffles	Neill, R. William	Roy William Neill	tt0019066	
1746455194	1994	1994	Miracle on 34th Street	Miracle on 34th Street	Mayfield, Les	Les Mayfield	tt0110527	
1746184096	1910	1910	A Change of Heart	A Change of Heart			tt1809217	
1746520911	1950	1950	Gunfire	Gunfire	Berke, William	William Berke	tt0042532	
1746199227	1967	1967	Doctor Dolittle	Doctor Dolittle	Fleischer, Richard	Richard Fleischer	tt0061584	
1746420114	1994	1994	A Great Day in Harlem	A Great Day in Harlem		Jean Bach	tt0109934	
1746497079	1919	1919	Injustice	Injustice	Peacocke, Leslie T.	Leslie T. Peacocke	tt4725198	
1746523357	1915	1915	The Yankee Girl	The Yankee Girl	Clark, J. J.	J.J. Clark	tt0006321	
1746577464	1963	1962	Stark Fear	Stark Fear	Hockman, Ned	Ned Hockman	tt0055478	
1746420229	1994	1994	Airheads	Airheads	Lehmann, Michael	Michael Lehmann	tt0109068	
1746500674	1924	1925	Soiled	Soiled	Windemere, Fred	Fred Windemere	tt0015350	
1746235234	1974	1974	Buster and Billie	Buster and Billie	Petrie, Daniel	Daniel Petrie	tt0071258	
1746495826	1919	1919	John Petticoats	John Petticoats	Hillyer, Lambert	Lambert Hillyer	tt0010314	
1746560292	1964	1964	Sleep	Sleep	Warhol, Andy	Andy Warhol	tt0187513	
1769970611	1968	1968	Scare Their Pants Off	Scare Their Pants Off!	Maddox, John	John Maddox	tt0063559	
1746568173	1952	1952	At Sword's Point	At Sword's Point	Allen, Lewis	Lewis Allen	tt0044380	
1746501672	1915	1915	Dora Thorne	Dora Thorne		Lawrence Marston	tt0005212	
1746219212	1973	1973	Blood Orgy of the She-Devils	Blood Orgy of the She Devils	Mikels, Ted V.	Ted V. Mikels	tt0068288	
1746566053	1953	1953	Shadows of Tombstone	Shadows of Tombstone	Witney, William	William Witney	tt0046302	
**1746437421**	**1944**	**1944**	**Atlantic City**	**Atlantic City**	**McCarey, Ray**	**Ray McCarey**	**tt0036619**	1
1746580397	1937	1937	Three Legionnaires	Three Legionnaires	MacFadden, Hamilton	Hamilton MacFadden	tt0029666	
1746565932	1954	1954	Security Risk	Security Risk	Schuster, Harold	Harold D. Schuster	tt0047465	
1746569780	1958	1958	Merry Andrew	Merry Andrew	Kidd, Michael	Michael Kidd	tt0051924	
1746509476	1926	1926	Mantrap	Mantrap	Fleming, Victor	Victor Fleming	tt0017119	
1746580860	1935	1935	The Good Fairy	The Good Fairy	Wyler, William	William Wyler	tt0026424	
1746574824	1933	1933	Fury of the Jungle	Fury of the Jungle	Neill, Roy William	Roy William Neill	tt0025155	
1746566165	1954	1953	Bad for Each Other	Bad for Each Other	Rapper, Irving	Irving Rapper	tt0045534	
1746523238	1915	1915	Temptation	Temptation	DeMille, Cecil B.	Cecil B. DeMille	tt0006140	
1745375201	2010	2010	Clash of the Titans	Clash of the Titans	Leterrier, Louis	Louis Leterrier	tt0800320	
1746438307	1942	1942	Her Cardboard Lover	Her Cardboard Lover	Cukor, George	George Cukor	tt0034846	
1746574621	1933	1933	Mama Loves Papa	Mama Loves Papa	McLeod, Norman	Norman Z. McLeod	tt0024292	
1746454659	1946	1946	The Dark Mirror	The Dark Mirror	Siodmak, Robert	Robert Siodmak	tt0038455	
1746464858	1941	1941	A Woman's Face	A Woman's Face	Cukor, George	George Cukor	tt0034399	
1746520555	1950	1950	Beauty on Parade	Beauty on Parade	Landers, Lew	Lew Landers	tt0042234	
1746199211	1966	1966	Love--My Way	Love: My Way	Hudson, Arch	Arch Hudson	tt0250538	

1746569832	1958	1958	Harry Black and the Tiger	Harry Black and the Tiger	Fregonese, Hugo	Hugo Fregonese	tt0051702
1746569763	1954	1954	Down Three Dark Streets	Down Three Dark Streets	Laven, Arnold	Arnold Laven	tt0046928
1746577669	1936	1936	I'll Name the Murderer	I'll Name the Murderer	Johnson, Raymond K.	Bernard B. Ray	tt0182242
1746454790	1998	1998	Dance with Me	Dance with Me	Haines, Randa	Randa Haines	tt0120576
1746571367	1933	1933	What Price Innocence?	What Price Innocence?	Mack, Willard	Willard Mack	tt0024761
1746503280	1919	1919	Love's Prisoner	Love's Prisoner	Dillon, Jack	John Francis Dillon	tt0010402
1746574169	1959	1959	Counterplot	Counterplot	Neumann, Kurt	Kurt Neumann	tt0052707
1746219231	1975	1975	Moonrunners	Moonrunners	Waldron, Gy	Gy Waldron	tt0071854
1745341563	2001	2001	The Shipping News	The Shipping News	Hallström, Lasse	Lasse Hallström	tt0120824
1746577854	1937	1937	Talent Scout	Talent Scout	Clemens, William	William Clemens	tt0029636
1746520769	1949	1949	Stagecoach Kid	Stagecoach Kid	Landers, Lew	Lew Landers	tt0041915
1746580282	1936	1936	Hearts Divided	Hearts Divided	Borzage, Frank	Frank Borzage	tt0027726
1746577214	1959	1959	Never So Few	Never So Few	Sturges, John	John Sturges	tt0053108
1746525005	1923	1923	Blood Test	Blood Test	Marquis, Don	Don Marquis	tt0013875
1746508251	1927	1927	Tearin' Into Trouble	Tearin' Into Trouble	Thorpe, Richard	Richard Thorpe	tt0019449
1746497493	1917	1917	The Warfare of the Flesh	The Warfare of the Flesh	Warren, Edward	Edward Warren	tt0008750
1746235369	1974	1974	Dirty Mary Crazy Larry	Dirty Mary Crazy Larry	Hough, John	John Hough	tt0071424
1746502470	1915	1915	Marrying Money	Marrying Money	Young, James	James Young	tt0005716
1746520369	1925	1925	Flaming Waters	Flaming Waters	Weight, F. Harmon	F. Harmon Weight	tt0132960
1746514993	1910	1910	The Musical Ranch	The Musical Ranch			tt1080758
1746533207	1915	1915	Body and Soul	Body and Soul	Irving, George	George Irving	tt0004988
1746394190	1982	1982	The Slumber Party Massacre	The Slumber Party Massacre	Jones, Amy	Amy Holden Jones	tt0084695
1746501361	1913	1913	The Daughter of the Hills	The Daughter of the Hills	Dawley, J. Searle	J. Searle Dawley	tt0002787
1746424231	1942	1942	Larceny, Inc.	Larceny, Inc.	Bacon, Lloyd	Lloyd Bacon	tt0034965
1746524007	1910	1910	The Smoker	The Smoker	Powell, Frank	Frank Powell	tt0001399
1746577672	1933	1933	Jaws of Justice	Jaws of Justice	Bennet, Spencer Gordon	Spencer Gordon Bennet	tt0024192
1746580581	1935	1935	Manhattan Butterfly	Manhattan Butterfly	Collins, Lewis D.	Lewis D. Collins	tt0025462
1746438212	1944	1944	Barbary Coast Gent	Barbary Coast Gent	Del Ruth, Roy	Roy Del Ruth	tt0036625
1746210231	1971	1971	Believe in Me	Believe in Me	Hagmann, Stuart; Avildsen, John G.	Stuart Hagmann	tt0066822
1746566135	1954	1954	Rear Window	Rear Window	Hitchcock, Alfred	Alfred Hitchcock	tt0047396
1746438101	1998	1997	Lolita	Lolita	Lyne, Adrian	Adrian Lyne	tt0119558
1746491812	1946	1946	The Walls Came Tumbling Down	The Walls Came Tumbling Down	Mendes, Lothar	Lothar Mendes	tt0039094
1746198553	1969	1969	Rascal	Rascal	Tokar, Norman	Norman Tokar	tt0064875
1746530758	1949	1949	The Lady Takes a Sailor	The Lady Takes a Sailor	Curtiz, Michael	Michael Curtiz	tt0041573
1746503549	1919	1919	The Golden Shower	The Golden Shower	Noble, John W.	John W. Noble	tt0010183
1746497866	1920	1920	The Eternal Mother	The Eternal Mother	Davis, William	Will S. Davis	tt0012137
1746410385	1984	1984	Racing With the Moon	Racing with the Moon	Benjamin, Richard	Richard Benjamin	tt0087968
1746503470	1921	1921	The Greater Claim	The Greater Claim	Ruggles, Wesley	Wesley Ruggles	tt0012241
1746569593	1957	1957	Motorcycle Gang	Motorcycle Gang	Cahn, Edward L.	Edward L. Cahn	tt0050728
1746269632	1976	1976	Lipstick	Lipstick	Johnson, Lamont	Lamont Johnson	tt0074802
1746520488	1927	1927	The Tired Business Man	The Tired Business Man	Dale, Allan	Allen Dale	tt0018490
1746569933	1955	1954	Masterson of Kansas	Masterson of Kansas	Castle, William	William Castle	tt0048358
1746577564	1937	1937	Tovarich	Tovarich	Litvak, Anatole	Anatole Litvak	tt0029685
1746493842	1910	1910	The Sanitarium	The Sanitarium		Tom Santschi	tt0132490
1746523240	1915	1915	The Devil	The Devil	Barker, Reginald; Ince, Thomas H.	Reginald Barker	tt0005193
1746208217	1965	1965	Lord Jim	Lord Jim	Brooks, Richard	Richard Brooks	tt0059399
1746562237	1953	1953	The Farmer Takes a Wife	The Farmer Takes a Wife	Levin, Henry	Henry Levin	tt0045752
1745342868	2003	2003	Pieces of April	Pieces of April	Hedges, Peter	Peter Hedges	tt0311648
1746409940	1986	1986	Deadtime Stories	Deadtime Stories	Delman, Jeffrey S.	Jeffrey Delman	tt0092850
1745293248	2007	2007	The Assassination of Jesse James by the Coward F	The Assassination of Jesse James by the Cow	Dominik, Andrew	Andrew Dominik	tt0443680
1746583077	1935	1935	Mystery Woman	Mystery Woman	Forde, Eugene	Eugene Forde	tt0026757
1746474090	1946	1946	Mr. Ace	Mr. Ace	Marin, Edwin L.	Edwin L. Marin	tt0038752
1746569532	1953	1953	Shane	Shane	Stevens, George	George Stevens	tt0046303
1746502996	1915	1915	The Heart of Jennifer	The Heart of Jennifer	Kirkwood, James	James Kirkwood	tt0005448
1746508544	1927	1927	Naughty Nanette	Naughty Nanette	Meehan, James Leo	James Leo Meehan	tt0018195
1746585407	1940	1940	Pals of the Silver Sage	Pals of the Silver Sage	Herman, Al	Albert Herman	tt0032885
1746570386	1933	1933	My Lips Betray	My Lips Betray	Blystone, John	John G. Blystone	tt0024363

1746497676	1920	1920	The Good-Bad Wife	The Good Bad Wife	DeVonde, Chester	Vera McCord	tt0012233
1746394535	1985	1984	Trancers	Trancers	Band, Charles	Charles Band	tt0090192
1746569622	1958	1958	The Barbarian and the Geisha	The Barbarian and the Geisha	Huston, John	John Huston	tt0051398
1746523979	1910	1910	The Ranch Girl's Legacy	The Ranch Girl's Legacy		Gilbert M. 'Broncho Billy' Anderson	tt0001372
1745358663	2003	2003	Big Fish	Big Fish	Burton, Tim	Tim Burton	tt0319061
1746439156	1942	1942	The Palm Beach Story	The Palm Beach Story	Sturges, Preston	Preston Sturges	tt0035169
1746454237	1942	1941	The Shanghai Gesture	The Shanghai Gesture	Sternberg, Josef von	Josef von Sternberg	tt0034175
1746580256	1938	1938	Battle of Broadway	Battle of Broadway	Marshall, George	George Marshall	tt0029902
1746256825	1979	1979	Summer Camp	Summer Camp	Vincent, Chuck	Chuck Vincent	tt0079965
1746561305	1955	1955	The Night of the Hunter	The Night of the Hunter	Laughton, Charles	Charles Laughton	tt0048424
1745293454	2006	2006	Miami Vice	Miami Vice	Mann, Michael	Michael Mann	tt0430357
1746515831	1921	1921	The Gunsaulus Mystery	The Gunsaulus Mystery	Micheaux, Oscar	Oscar Micheaux	tt0133825
1746580363	1936	1936	Along Came Love	Along Came Love	Lytell, Bert	Bert Lytell	tt0027279
1746513635	1910	1910	A Mohawk's Way	A Mohawk's Way	Griffith, D. W.	D.W. Griffith	tt0001327
1746423348	2000	2000	The Gift	The Gift	Raimi, Sam	Sam Raimi	tt0219699
1746503679	1921	1921	Hail the Woman	Hail the Woman	Wray, John Griffith	John Griffith Wray	tt0012248
1746219623	1971	1971	Pretty Maids All in a Row	Pretty Maids All in a Row	Vadim, Roger	Roger Vadim	tt0067610
1746497005	1920	1920	Just Pals	Just Pals	Ford, Jack	John Ford	tt0011358
1746467695	1917	1917	The Raggedy Queen	The Raggedy Queen	Marston, Theodore	Theodore Marston	tt0008490
1746579008	1950	1950	The Showdown	The Showdown	McGowan, Dorrell; McGowan, Stuar	Dorrell McGowan	tt0042957
1746584136	1935	1935	Loser's End	Loser's End	Ray, B. B.	Bernard B. Ray	tt0026650
1746210350	1971	1971	McCabe & Mrs. Miller	McCabe & Mrs. Miller	Altman, Robert	Robert Altman	tt0067411
1746500045	1924	1924	The Whirlwind Ranger	The Whirlwind Ranger	Hatton, Richard	Dick Hatton	tt0179515
1746550000	1954	1954	The Other Woman	The Other Woman	Haas, Hugo	Hugo Haas	tt0047317
1746514906	1927	1927	Alias the Lone Wolf	Alias the Lone Wolf	Griffith, Edward H.	Edward H. Griffith	tt0017599
1746500536	1923	1923	Haldane of the Secret Service	Haldane of the Secret Service	Houdini, Harry	Harry Houdini	tt0014112
1746503683	1920	1920	Thou Art the Man	Thou Art the Man	Heffron, Thomas N.	Thomas N. Heffron	tt0011766
1746420523	1988	1988	The Seventh Sign	The Seventh Sign	Schultz, Carl	Carl Schultz	tt0096073
1746520669	1925	1925	Heartless Husbands	Heartless Husbands	Bracken, Bertram	Bertram Bracken	tt0134709
1746419376	1993	1993	Lost in Yonkers	Lost in Yonkers	Coolidge, Martha	Martha Coolidge	tt0107443
1746578050	1935	1935	Lottery Lover	The Lottery Lover	Thiele, William	Wilhelm Thiele	tt0026653
1746580781	1938	1938	Radio City Revels	Radio City Revels	Stoloff, Ben	Benjamin Stoloff	tt0030651
1746485936	1918	1918	The Wolf and His Mate	The Wolf and His Mate	LeSaint, E. J.	Edward LeSaint	tt0008784
1745374321	2002	2002	K-19: The Widowmaker	K 19: The Widowmaker	Bigelow, Kathryn	Kathryn Bigelow	tt0267626
1746454490	1942	1942	The Strange Case of Doctor Rx	The Strange Case of Doctor Rx	Nigh, William	William Nigh	tt0035386
1746576986	1958	1958	Wolf Larsen	Wolf Larsen	Jones, Harmon	Harmon Jones	tt0052402
1746455175	1998	1998	Deep Impact	Deep Impact	Leder, Mimi	Mimi Leder	tt0120647
1745272333	2000	1999	The Woman Chaser	The Woman Chaser	Devor, Robinson	Robinson Devor	tt0217894
1746578830	1963	1963	Europe in the Raw	Europe in the Raw	Meyer, Russ	Russ Meyer	tt0057047
1746523414	1916	1916	Love's Pilgrimage to America	Love's Pilgrimage to America	Marston, Lawrence	Lawrence Marston	tt0005658
1746503046	1923	1923	Finger Prints	Finger Prints	Levering, Joseph	Joseph Levering	tt0325380
1746199827	1969	1969	Popi	Popi	Hiller, Arthur	Arthur Hiller	tt0064827
1746235864	1975	1975	The Black Gestapo	The Black Gestapo	Frost, Lee	Lee Frost	tt0169620
1746570315	1952	1952	It Grows on Trees	It Grows on Trees	Lubin, Arthur	Arthur Lubin	tt0044758
1746474137	1945	1945	The Shanghai Cobra	The Shanghai Cobra	Karlson, Phil	Phil Karlson	tt0038080
1746438314	1942	1942	In Old California	In Old California	McGann, William	William C. McGann	tt0034889
1746514524	1910	1910	That Chink at Golden Gulch	That Chink at Golden Gulch	Griffith, D. W.	D.W. Griffith	tt0001414
1746581205	1910	1910	A Game of Hearts	A Game of Hearts			tt0449270
1746233823	1966	1966	Blood Bath	Blood Bath	Hill, Jack; Rothman, Stephanie	Jack Hill	tt0060174
1746499994	1924	1924	The Desert Hawk	The Desert Hawk	De La Mothe, Leon	Leon De La Mothe	tt0014842
1746408699	1984	1983	Cold Feet	Cold Feet	Van Dusen, Bruce	Bruce Van Dusen	tt0087070
1746508381	1925	1925	Tearin' Loose	Tearin' Loose	Thorpe, Richard	Richard Thorpe	tt0016412
1746560144	1964	1964	Get Yourself a College Girl	Get Yourself a College Girl	Miller, Sidney	Sidney Miller	tt0161612
1746585271	1930	1930	Parade of the West	Parade of the West	Brown, Harry J.	Harry Joe Brown	tt0021230
1746520361	1924	1924	Icebound	Icebound	de Mille, William	William C. de Mille	tt0015008
1746584015	1939	1939	Stagecoach	Stagecoach	Ford, John	John Ford	tt0031971
1746569979	1960	1960	The Crowded Sky	The Crowded Sky	Pevney, Joseph	Joseph Pevney	tt0053742

1746512206	1927	1927	Aflame in the Sky	Aflame in the Sky	McGowan, J. P.	J.P. McGowan	tt0017591
1746555342	1955	1955	Running Wild	Running Wild	Biberman, Abner	Abner Biberman	tt0048571
1746497924	1920	1920	The Thirteenth Commandment	The Thirteenth Commandment	Vignola, Robert G.	Robert G. Vignola	tt0011764
1746566068	1953	1952	Off Limits	Off Limits	Marshall, George	George Marshall	tt0046138
1746503052	1922	1922	The Golden Gallows	The Golden Gallows	Scardon, Paul	Paul Scardon	tt0013190
1746582618	1937	1937	Rich Relations	Rich Relations	Sanforth, Clifford	Clifford Sanforth	tt0029483
1746504368	1919	1919	A Man's Duty	A Man's Duty	Gant, Harry A.; Thomas, D. Ireland	Harry A. Gant	tt0010431
1745359793	2002	2002	Hart's War	Hart's War	Hoblit, Gregory	Gregory Hoblit	tt0251114
1746575022	1933	1933	Revenge at Monte Carlo	Revenge at Monte Carlo	Eason, Breezy	B. Reeves Eason	tt0024497
1746508481	1925	1925	Back to Life	Back to Life	Bennett, Whitman	Whitman Bennett	tt0015591
1745341861	2010	2010	Yeardley	Yeardley	Michaels, Heath C.	Heath C. Michaels	tt1329330
1746248794	1974	1974	Black Samson	Black Samson	Bail, Charles	Charles Bail	tt0071226
1746560870	1932	1932	The Phantom Express	The Phantom Express	Johnson, Emory	Emory Johnson	tt0023333
1746486305	1917	1917	The Barrier	The Barrier	Lewis, Edgar	Edgar Lewis	tt0007684
1746496070	1920	1920	Other Men's Shoes	Other Men's Shoes	Lewis, Edgar	Edgar Lewis	tt0196815
1746570372	1934	1934	The House of Mystery	House of Mystery	Nigh, William	William Nigh	tt0025271
1746561598	1954	1954	Out of This World	Out of This World	Thomas, Lowell; Thomas, Lowell		tt0282822
1746512926	1913	1913	Ivanhoe	Ivanhoe	Brenon, Herbert	Herbert Brenon	tt0003022
1746410602	1983	1983	Abuse	Abuse	Bressan, Arthur J.	Arthur J. Bressan Jr.	tt0083517
1746270046	1980	1980	The Exterminator	The Exterminator	Glickenhaus, James	James Glickenhaus	tt0080707
1746569555	1958	1958	Onionhead	Onionhead	Taurog, Norman	Norman Taurog	tt0052030
1746465321	1942	1941	Sullivan's Travels	Sullivan's Travels	Sturges, Preston	Preston Sturges	tt0034240
1746186079	1969	1969	Last Summer	Last Summer	Perry, Frank	Frank Perry	tt0064573
1746235193	1975	1975	Race with the Devil	Race with the Devil	Starrett, Jack	Jack Starrett	tt0073600
1746233485	1967	1967	Chuka	Chuka	Douglas, Gordon	Gordon Douglas	tt0061477
1745272746	2000	2000	Reindeer Games	Reindeer Games	Frankenheimer, John	John Frankenheimer	tt0184858
1746420978	1995	1995	Dracula: Dead and Loving It	Dracula: Dead and Loving It	Brooks, Mel	Mel Brooks	tt0112896
1746577205	1951	1951	Silver City	Silver City	Haskin, Byron	Byron Haskin	tt0044036
1746569621	1953	1953	Song of the Land	Song of the Land	Kesler, Henry S.	Henry S. Kesler	tt0358707
1746523333	1916	1916	The Weakness of Strength	The Weakness of Strength	Revier, Harry	Harry Revier	tt0007546
1745372646	2008	2008	Choke	Choke	Gregg, Clark	Clark Gregg	tt1024715
1746487730	1921	1921	A Perfect Crime	A Perfect Crime	Dwan, Allan	Allan Dwan	tt0012565
1746507825	1925	1925	The Beautiful City	The Beautiful City	Webb, Kenneth	Kenneth S. Webb	tt0015608
1746514431	1915	1915	The Moth and the Flame	The Moth and the Flame	Olcott, Sidney	Sidney Olcott	tt0005773
1746566120	1958	1958	The Female Animal	The Female Animal	Keller, Harry	Harry Keller	tt0050385
1746574228	1960	1959	The Atomic Submarine	The Atomic Submarine	Bennet, Spencer G.	Spencer Gordon Bennet	tt0052587
1746408457	1989	1989	The Mighty Quinn	The Mighty Quinn	Schenkel, Carl	Carl Schenkel	tt0097880
1746509548	1926	1926	Eve's Leaves	Eve's Leaves	Sloane, Paul	Paul Sloane	tt0016827
1746501788	1916	1916	Black Friday	Black Friday	Carleton, Lloyd B.	Lloyd B. Carleton	tt0006433
1746575006	1934	1934	Crime Without Passion	Crime Without Passion	Hecht, Ben; MacArthur, Charles	Ben Hecht	tt0025009
1746423322	1999	1999	Sleepy Hollow	Sleepy Hollow	Burton, Tim	Tim Burton	tt0162661
1746520730	1949	1949	Challenge of the Range	Challenge of the Range	Nazarro, Ray	Ray Nazarro	tt0041237
1746533214	1916	1916	Is Any Girl Safe?	Is Any Girl Safe?	Jaccard, Jacques	Jacques Jaccard	tt0006871
1746574156	1963	1963	Wall of Noise	Wall of Noise	Wilson, Richard	Richard Wilson	tt0057667
1746569542	1955	1955	Tennessee's Partner	Tennessee's Partner	Dwan, Allan	Allan Dwan	tt0048707
1746483874	1919	1919	Love Insurance	Love Insurance	Crisp, Donald	Donald Crisp	tt0010396
1746491797	1946	1946	Renegade Girl	Renegade Girl	Berke, William	William Berke	tt0038877
1769969352	1964	1964	Olga's Girls	Olga's Girls	Mawra, Joseph P.	Joseph P. Mawra	tt0058427
1746514428	1914	1914	The Corsair	The Corsair	Powell, Frank	Frank Powell	tt0003795
1746553708	1965	1965	The Art of Love	The Art of Love	Jewison, Norman	Norman Jewison	tt0058920
1746501160	1916	1916	Her Greatest Story	Her Greatest Story		Lynn Reynolds	tt0326931
1746423826	1941	1941	Outlaws of the Desert	Outlaws of the Desert	Bretherton, Howard	Howard Bretherton	tt0033989
1746562456	1954	1954	Yankee Pasha	Yankee Pasha	Pevney, Joseph	Joseph Pevney	tt0047684
1746524031	1910	1910	The Millionaire and the Ranch Girl	The Millionaire and the Ranch Girl		Gilbert M. 'Broncho Billy' Anderson	tt0001322
1746582250	1931	1931	Night Nurse	Night Nurse	Wellman, William A.	William A. Wellman	tt0022208
1746578047	1937	1937	Life Begins in College	Life Begins in College	Seiter, William A.	William A. Seiter	tt0029144
1746513402	1910	1910	Stung!	Stung!			tt0449403

1746543066	1916	1916	The Measure of a Man	The Measure of a Man	Conway, Jack	Jack Conway	tt0007050
1746453493	1941	1941	Riders of the Badlands	Riders of the Badlands	Bretherton, Howard	Howard Bretherton	tt0034103
1746523052	1916	1916	The Captive God	The Captive God	Swickard, Charles	Charles Swickard	tt0006489
1746583879	1930	1930	Hunted Men	The Hunted Men	McGowan, J. P.	J.P. McGowan	tt0020991
1746582330	1930	1930	The Poor Millionaire	The Poor Millionaire	Melford, George	George Melford	tt0021266
1746424598	1941	1941	Flying Blind	Flying Blind	McDonald, Frank	Frank McDonald	tt0033611
1746585050	1928	1928	Flash of the Forest	The Flash of the Forest			tt0169910
1746578652	1963	1963	Bell, Bare and Beautiful	Bell, Bare and Beautiful	Gordon, Lewis H.	Herschell Gordon Lewis	tt0056863
1746585145	1930	1930	Strictly Modern	Strictly Modern	Seiter, William A.	William A. Seiter	tt0021421
1746570997	1934	1935	The Silver Bullet	The Silver Bullet	Ray, B. B.	Bernard B. Ray	tt0026999
1746573047	1932	1932	Central Park	Central Park	Adolfi, John G.	John G. Adolfi	tt0022751
1746409803	1988	1988	Married to the Mob	Married to the Mob	Demme, Jonathan	Jonathan Demme	tt0095593
1746210018	1971	1971	The Beguiled	The Beguiled	Siegel, Donald	Don Siegel	tt0066819
1746563025	1951	1951	The Great Caruso	The Great Caruso	Thorpe, Richard	Richard Thorpe	tt0043599
1746561322	1956	1956	World Without End	World Without End	Bernds, Edward	Edward Bernds	tt0049964
1769970770	1912	1912	The Jocular Winds of Fate	The Jocular Winds of Fate		Van Dyke Brooke	tt0222066
1746580766	1938	1937	Thrill of a Lifetime	Thrill of a Lifetime	Archainbaud, George	George Archainbaud	tt0029668
1746570820	1934	1934	Babes in Toyland	Babes in Toyland	Meins, Gus; Rogers, Charles	Gus Meins	tt0024852
1746577628	1937	1937	Nancy Steele Is Missing!	Nancy Steele Is Missing!	Marshall, George	George Marshall	tt0029292
1746509223	1925	1925	The Crimson Runner	The Crimson Runner	Forman, Tom	Tom Forman	tt0015715
1746216754	1967	1967	The Reluctant Astronaut	The Reluctant Astronaut	Montagne, Edward J.	Edward Montagne	tt0062190
1746567244	1952	1952	No Holds Barred	No Holds Barred	Beaudine, William	William Beaudine	tt0044969
1746501166	1923	1923	The Nth Commandment	The Nth Commandment	Borzage, Frank	Frank Borzage	tt0014322
1746500055	1923	1923	Golden Silence	Golden Silence	Hurst, Paul	Paul Hurst	tt0013192
1746574133	1962	1962	A Girl Named Tamiko	A Girl Named Tamiko	Sturges, John	John Sturges	tt0056021
1746491993	1948	1948	Caged Fury	Caged Fury	Berke, William	William Berke	tt0040196
1746184852	1965	1965	Scream of the Butterfly	Scream of the Butterfly	Lobato, Ebar	Eber Lobato	tt0059688
1746585401	1939	1939	Frontier Marshal	Frontier Marshal	Dwan, Allan	Allan Dwan	tt0031346
1746233008	1969	1969	The Satin Mushroom	The Satin Mushroom	Brown, Don	Don Brown	tt0138076
1746453516	1944	1944	The Ghost That Walks Alone	The Ghost That Walks Alone	Landers, Lew	Lew Landers	tt0036863
1746216690	1968	1968	Lady in Cement	Lady in Cement	Douglas, Gordon	Gordon Douglas	tt0063210
1746219705	1976	1976	Midway	Midway	Smight, Jack	Jack Smight	tt0074899
1746571079	1935	1934	West of the Pecos	West of the Pecos	Rosen, Phil	Phil Rosen	tt0025971
1746409029	1982	1982	Pandemonium	Pandemonium	Sole, Alfred	Alfred Sole	tt0084464
1746581959	1930	1930	What a Widow	What a Widow!	Dwan, Allan	Allan Dwan	tt0021545
1746394395	1983	1983	Gorky Park	Gorky Park	Apted, Michael	Michael Apted	tt0085615
1746568263	1952	1952	Babes in Bagdad	Babes in Bagdad	Ulmer, Edgar G.	Jerónimo Mihura	tt0044389
1746453811	1943	1943	Two Senoritas from Chicago	Two Señoritas from Chicago	Woodruff, Frank	Frank Woodruff	tt0036466
1746503403	1919	1919	The Echo of Youth	The Echo of Youth	Abramson, Ivan	Ivan Abramson	tt0010081
1746584431	1939	1939	For Love or Money	For Love or Money	Rogell, Albert S.	Albert S. Rogell	tt0031328
1746393982	1984	1984	The Natural	The Natural	Levinson, Barry	Barry Levinson	tt0087781
1746520402	1925	1925	Western Promise	Western Promise			tt0177388
1746520088	1949	1949	Bride for Sale	Bride for Sale	Russell, William D.	William D. Russell	tt0041208
1769976141	1927	1927	Campus Romeos	Campus Romeos	Herman, Albert	Gilbert Pratt	tt0423942
1746582336	1928	1928	Across to Singapore	Across to Singapore	Nigh, William	William Nigh	tt0018618
1746570147	1933	1933	Fast Workers	Fast Workers	Browning, Tod	Tod Browning	tt0024005
1746492050	1947	1947	The Spirit of West Point	The Spirit of West Point	Murphy, Ralph	Ralph Murphy	tt0039858
1746524962	1915	1915	The Wonderful Adventure	The Wonderful Adventure	Thomson, Frederick	Frederick A. Thomson	tt0006311
1746419359	1990	1990	Alice	Alice	Allen, Woody	Woody Allen	tt0099012
1746466696	1917	1917	Pay Me	Pay Me!	De Grasse, Joseph	Joseph De Grasse	tt0008417
1746583571	1931	1931	Trapped	Trapped	Mitchell, Bruce	Kurt Neumann	tt0134160
1746578945	1962	1962	Nature's Playmates	Nature's Playmates	Gordon, Lewis H.	Herschell Gordon Lewis	tt0056273
1746520656	1927	1927	Night Life	Night Life	Archainbaud, George	George Archainbaud	tt0018202
1746576862	1931	1931	The Virtuous Husband	The Virtuous Husband	Moore, Vin	Vin Moore	tt0022539
1746580312	1934	1934	Let's Be Ritzy	Let's Be Ritzy	Ludwig, Edward	Edward Ludwig	tt0025380
1746524610	1922	1922	Don't Write Letters	Don't Write Letters	Baker, George D.	George D. Baker	tt0013082
1746575809	1931	1930	Under Suspicion	Under Suspicion	Erickson, A. F.	A.F. Erickson	tt0021501

1745358520	2009	2009	Watchmen	Watchmen	Snyder, Zack	Zack Snyder	tt0409459
1746566360	1954	1954	Duel in the Jungle	Duel in the Jungle	Marshall, George	George Marshall	tt0046942
1746577717	1933	1933	Christopher Strong	Christopher Strong	Arzner, Dorothy	Dorothy Arzner	tt0023891
1746491746	1947	1947	The Brasher Doubloon	The Brasher Doubloon	Brahm, John	John Brahm	tt0039217
1746487150	1919	1919	The Lone Wolf's Daughter	The Lone Wolf's Daughter	Earle, William P. S.	William P.S. Earle	tt0010378
1746421742	1993	1993	Short Cuts	Short Cuts	Altman, Robert	Robert Altman	tt0108122
1746503345	1920	1920	The Week-End	The Week End	Cox, George L.	George L. Cox	tt0011844
1746571129	1952	1952	This Woman Is Dangerous	This Woman Is Dangerous	Feist, Felix	Felix E. Feist	tt0045232
1746503995	1920	1920	Her Story	Her Story	Samuelson, G. B.; Carrick, Allyn B.	Alexander Butler	tt0140146
1745342637	2009	2009	Humpday	Humpday	Shelton, Lynn	Lynn Shelton	tt1334537
1746566088	1958	1958	Paris Holiday	Paris Holiday	Oswald, Gerd	Gerd Oswald	tt0052047
1746578542	1960	1960	Sergeant Rutledge	Sergeant Rutledge	Ford, John	John Ford	tt0054292
1746514421	1916	1916	The Diamond Runners	The Diamond Runners	McGowan, J. P.	J.P. McGowan	tt0157538
1746464994	1947	1947	The Romance of Rosy Ridge	The Romance of Rosy Ridge	Rowland, Roy	Roy Rowland	tt0039783
1746577395	1951	1951	Sugarfoot	Sugarfoot	Marin, Edwin L.	Edwin L. Marin	tt0044087
1746573949	1963	1963	The Caretakers	The Caretakers	Bartlett, Hall	Hall Bartlett	tt0056908
1746251182	1976	1976	Norman...Is That You?	Norman... Is That You?	Schlatter, George	George Schlatter	tt0074973
1746485237	1917	1917	The Slave Market	The Slave Market	Ford, Hugh	Hugh Ford	tt0182427
1746554452	1964	1964	Your Cheatin' Heart	Your Cheatin' Heart	Nelson, Gene	Gene Nelson	tt0058765
1746577726	1934	1934	Murder on the Blackboard	Murder on the Blackboard	Archainbaud, George	George Archainbaud	tt0025532
1746578065	1938	1938	Josette	Josette	Dwan, Allan	Allan Dwan	tt0030291
1746573937	1958	1958	South Seas Adventure	South Seas Adventure		Carl Dudley	tt0052226
1746569901	1954	1954	The Forty-Niners	The Forty Niners	Carr, Thomas	Thomas Carr	tt0122072
1746569573	1957	1957	Love in the Afternoon	Love in the Afternoon	Wilder, Billy	Billy Wilder	tt0050658
1746499852	1950	1950	A Lady Without Passport	A Lady Without Passport	Lewis, Joseph H.	Joseph H. Lewis	tt0042664
1746409454	1981	1981	Four Friends	Four Friends	Penn, Arthur	Arthur Penn	tt0082404
1746524736	1916	1916	The Silent Battle	The Silent Battle	Conway, Jack	Jack Conway	tt0007346
1746560527	1932	1932	Lone Trail	The Lone Trail	Webb, Harry S.; Douglas, Forrest	Forrest Sheldon	tt0303359
1746566354	1954	1954	The Lawless Rider	The Lawless Rider	Canutt, Yakima	Yakima Canutt	tt0047164
1746453596	1943	1943	Action in the North Atlantic	Action in the North Atlantic	Bacon, Lloyd	Lloyd Bacon	tt0035608
1746570861	1934	1934	The Mysterious Mr. Wong	The Mysterious Mr. Wong	Nigh, William	William Nigh	tt0026755
1746434278	1999	1999	The Mod Squad	The Mod Squad	Silver, Scott	Scott Silver	tt0120757
1746209564	1965	1965	The Reward	The Reward	Bourguignon, Serge	Serge Bourguignon	tt0059651
1746520373	1926	1926	The Fighting Peacemaker	The Fighting Peacemaker	Smith, Clifford S.	Clifford Smith	tt0016865
1746454162	1999	1999	Light It Up	Light It Up	Bolotin, Craig	Craig Bolotin	tt0172726
1746394668	1988	1988	Dangerous Liaisons	Dangerous Liaisons	Frears, Stephen	Stephen Frears	tt0094947
1746248154	1967	1967	The Taming of the Shrew	The Taming of the Shrew	Zeffirelli, Franco	Franco Zeffirelli	tt0061407
1746422128	1998	1998	Rush Hour	Rush Hour	Ratner, Brett	Brett Ratner	tt0120812
1746583989	1939	1939	Keep Punching	Keep Punching	Clein, John	John Clein	tt0135501
1746584832	1929	1929	The Carnation Kid	The Carnation Kid	Hopper, E. Mason	E. Mason Hopper	tt0019753
1746508686	1924	1924	Fighter's Paradise	Fighter's Paradise	Neitz, Alvin J.	Alan James	tt0014890
1769976188	1927	1927	Crazy to Act	Crazy to Act	Rodney, Earle	Earle Rodney	tt0246538
1746507794	1926	1926	Memory Lane	Memory Lane	Stahl, John M.	John M. Stahl	tt0017132
1745359530	2003	2003	Girls Will Be Girls	Girls Will Be Girls	Day, Richard	Richard Day	tt0339071
1746516145	1922	1922	The Song of Life	The Song of Life	Stahl, John M.	John M. Stahl	tt0013311
1746515265	1918	1918	Two-Gun Betty	Two Gun Betty	Hickman, Howard	Howard C. Hickman	tt0190100
1746578666	1963	1963	Drums of Africa	Drums of Africa	Clark, James B.	James B. Clark	tt0057015
1746465074	1942	1942	Down Rio Grande Way	Down Rio Grande Way	Berke, William	William Berke	tt0034676
1746515298	1921	1921	Blind Hearts	Blind Hearts	Lee, Rowland V.	Rowland V. Lee	tt0011978
1746584174	1936	1936	End of the Trail	End of the Trail	Kenton, Erle C.	Erle C. Kenton	tt0027573
1746520179	1925	1925	Beyond the Border	Beyond the Border	Dunlap, Scott R.	Scott R. Dunlap	tt0015620
1746520482	1925	1925	Two Fisted Jones	Two Fisted Jones	Sedgwick, Edward	Edward Sedgwick	tt0016466
1746584341	1936	1936	The Arizona Raiders	The Arizona Raiders	Hogan, James	James P. Hogan	tt0027308
1745357619	2008	2008	Yes Man	Yes Man	Reed, Peyton	Peyton Reed	tt1068680
1746574796	1933	1933	Flying Down to Rio	Flying Down to Rio	Freeland, Thornton	Thornton Freeland	tt0024025
1746581871	1951	1951	Only the Valiant	Only the Valiant	Douglas, Gordon	Gordon Douglas	tt0043885
1746217384	1967	1967	Guess Who's Coming to Dinner	Guess Who's Coming to Dinner	Kramer, Stanley	Stanley Kramer	tt0061735

1746501256	1914	1913	Uncle Tom's Cabin	Uncle Tom's Cabin	Daly, William Robert	Otis Turner	tt0201287
1746562433	1954	1954	The Human Jungle	The Human Jungle	Newman, Joseph M.	Joseph M. Newman	tt0047102
1746532498	1916	1916	The Return of Eve	The Return of Eve	Berthelet, Arthur	Arthur Berthelet	tt0158894
1745303184	2006	2006	Vajra Sky Over Tibet	Vajra Sky Over Tibet	Bush, John	John Bush	tt0840019
1746422037	1997	1996	Ratchet	Ratchet	Johnson, John S.	John Johnson	tt0117443
1746420871	1983	1982	The Entity	The Entity	Furie, Sidney J.	Sidney J. Furie	tt0082334
1746566179	1957	1957	My Man Godfrey	My Man Godfrey	Koster, Henry	Henry Koster	tt0050738
1746570224	1934	1934	Spitfire	Spitfire	Cromwell, John	John Cromwell	tt0025822
1745282911	2006	2006	Blood Diamond	Blood Diamond	Zwick, Edward	Edward Zwick	tt0450259
1746499975	1925	1925	Lorraine of the Lions	Lorraine of the Lions	Sedgwick, Edward	Edward Sedgwick	tt0016038
1746410352	1990	1990	Stella	Stella	Erman, John	John Erman	tt0100691
1746512368	1914	1914	The Boer War	The Boer War	Melford, George	George Melford	tt0003686
1746577535	1933	1933	She Done Him Wrong	She Done Him Wrong	Sherman, Lowell	Lowell Sherman	tt0024548
1746492593	1946	1946	Renegades	Renegades	Sherman, George	George Sherman	tt0038878
1746436533	1941	1941	Free and Easy	Free and Easy	Sidney, George; Buzzell, Edward	George Sidney	tt0033634
1746424593	1943	1943	Dangerous Blondes	Dangerous Blondes	Jason, Leigh	Leigh Jason	tt0035779
1746572572	1910	1910	The Eternal Triangle	The Eternal Triangle		Harry Solter	tt0200620
1746560725	1932	1932	The Riding Tornado	The Riding Tornado	Lederman, D. Ross	D. Ross Lederman	tt0023402
1746270781	1980	1980	Without Warning	Without Warning	Clark, Greydon	Greydon Clark	tt0081764
1746210857	1973	1973	White Lightning	White Lightning	Sargent, Joseph	Joseph Sargent	tt0070915
1746465139	1944	1944	The Black Parachute	The Black Parachute	Landers, Lew	Lew Landers	tt0036648
1746453570	1943	1943	Hit Parade of 1943	Hit Parade of 1943	Rogell, Albert S.	Albert S. Rogell	tt0035727
1746533171	1915	1915	The Disciple	The Disciple	Hart, William S.	William S. Hart	tt0005200
1746496817	1920	1920	The Girl in the Web	The Girl in the Web	Thornby, Robert	Robert Thornby	tt0011231
1746560010	1963	1963	The Victors	The Victors	Foreman, Carl	Carl Foreman	tt0057652
1746582434	1938	1938	Under the Big Top	Under the Big Top	Brown, Karl	Karl Brown	tt0030911
1746516352	1919	1919	The Lord Loves the Irish	The Lord Loves the Irish	Warde, Ernest C.	Ernest C. Warde	tt0191251
1746569816	1957	1957	Desk Set	Desk Set	Lang, Walter	Walter Lang	tt0050307
1746514811	1915	1915	Always in the Way	Always in the Way	Dawley, J. Searle	J. Searle Dawley	tt0004885
1746420522	1992	1992	Incident at Oglala	Incident at Oglala	Apted, Michael	Michael Apted	tt0104504
1746509299	1927	1927	Painted Ponies	Painted Ponies	Eason, Reeves	B. Reeves Eason	tt0018248
1746261332	1978	1978	Grease	Grease	Kleiser, Randal	Randal Kleiser	tt0077631
1746584195	1939	1939	Blind Alley	Blind Alley	Vidor, Charles	Charles Vidor	tt0031104
1746420983	1997	1997	The Apostle	The Apostle	Duvall, Robert	Robert Duvall	tt0118632
1746183906	1910	1910	The Bad Man and the Preacher	The Bad Man and the Preacher		Gilbert M. 'Broncho Billy' Anderson	tt0372159
1746532461	1916	1916	The Lash of Destiny	The Lash of Destiny	Terwilliger, George	George Terwilliger	tt0163046
1769971964	1969	1969	Gutter Trash	Gutter Trash	Milligan, Andy	Andy Milligan	tt0219724
1745303083	2007	2006	Jack Smith and the Destruction of Atlantis	Jack Smith and the Destruction of Atlantis	Jordan, Mary	Mary Jordan	tt0795406
1746454009	1944	1944	Between Two Worlds	Between Two Worlds	Blatt, Edward A.	Edward A. Blatt	tt0036641
1746583898	1930	1930	Double Cross Roads	Double Cross Roads	Werker, Alfred L.	George E. Middleton	tt0020837
1746583742	1930	1930	Code of Honor	Code of Honor	McGowan, J. P.	J.P. McGowan	tt0020774
1746273708	1978	1978	Jennifer	Jennifer	Mack, Brice	Brice Mack	tt0077769
1746577140	1958	1957	Escape from Red Rock	Escape from Red Rock	Bernds, Edward	Edward Bernds	tt0051590
1746500431	1949	1949	Miss Grant Takes Richmond	Miss Grant Takes Richmond	Bacon, Lloyd	Lloyd Bacon	tt0041652
1746565939	1954	1953	War Arrow	War Arrow	Sherman, George	George Sherman	tt0046532
1746496120	1919	1919	A Midnight Romance	A Midnight Romance	Weber, Lois	Lois Weber	tt0010461
1746475534	1947	1947	The Flame	The Flame	Auer, John H.	John H. Auer	tt0039386
1746580379	1938	1938	The Last Stand	The Last Stand	Lewis, Joseph H.	Joseph H. Lewis	tt0030346
1746508942	1926	1926	While London Sleeps	While London Sleeps	Bretherton, H. P.	Howard Bretherton	tt0017548
1746475998	1946	1946	Affairs of Geraldine	Affairs of Geraldine	Blair, George	George Blair	tt0038290
1746577820	1938	1938	Fugitives for a Night	Fugitives for a Night	Goodwins, Leslie	Leslie Goodwins	tt0030162
1746455324	1995	1995	Unzipped	Unzipped	Keeve, Douglas	Douglas Keeve	tt0114805
1746554581	1965	1965	Once Upon a Coffee House	Once Upon a Coffee House	Traube, Shepherd	Shepard Traube	tt0234388
1746514603	1917	1917	Draft 258	Draft 258	Cabanne, William Christy	Christy Cabanne	tt0009024
1746219185	1972	1972	Night of the Lepus	Night of the Lepus	Claxton, William F.	William F. Claxton	tt0069005
1769971150	1969	1969	The Ecstasies of Women	The Ecstasies of Women	Hansen, Mark	Herschell Gordon Lewis	tt0064278
1746500272	1925	1925	Braveheart	Braveheart	Hale, Alan	Alan Hale	tt0015643

1746394472	1987	1987	Rumpelstiltskin	Rumpelstiltskin	Irving, David	David Irving	tt0093891	
1745294002	2006	2006	16 Blocks	16 Blocks	Donner, Richard	Richard Donner	tt0450232	
1746474809	1948	1948	The Girl from Manhattan	The Girl from Manhattan	Green, Alfred E.	Alfred E. Green	tt0040388	
1746555644	1954	1954	Rogue Cop	Rogue Cop	Rowland, Roy	Roy Rowland	tt0047424	
1746503956	1922	1922	Through the Storm	Through the Storm	Plympton, Horace G.	Horace G. Plympton	tt0170681	
1746576992	1961	1961	Honeymoon of Terror	Honeymoon of Terror	Peri	Peter Perry Jr.	tt0173908	
1746500161	1924	1923	Love Letters	The Love Letter	Soloman, David	King Baggot	tt0014217	
1769970063	1912	1912	The Lie	The Lie	Baggot, King; Daly, W. R.	King Baggot	tt0339297	
1746579029	1951	1951	The Thundering Trail	The Thundering Trail	Ormond, Ron	Ron Ormond	tt0044129	
1746455323	1998	1998	The Opposite of Sex	The Opposite of Sex	Roos, Don	Don Roos	tt0120777	
1745359982	2002	2002	The Hot Chick	The Hot Chick	Brady, Tom	Tom Brady	tt0302640	
1746584181	1938	1938	Dangerous to Know	Dangerous to Know	Florey, Robert	Robert Florey	tt0030039	
1746562214	1954	1954	Border River	Border River	Sherman, George	George Sherman	tt0046800	
**1746520602**	**1925**	**1925**	**White Thunder**	**White Thunder**	**Wilson, Ben**	**Ben F. Wilson**	**tt0127974**	1
1746234408	1967	1967	Hells Angels on Wheels	Hells Angels on Wheels	Rush, Richard	Richard Rush	tt0061758	
1746567760	1933	1933	Let's Fall in Love	Let's Fall in Love	Burton, David	David Burton	tt0025381	
1746573851	1960	1960	Comanche Station	Comanche Station	Boetticher, Budd	Budd Boetticher	tt0053729	
1746574825	1938	1938	The Duke Is Tops	The Duke Is Tops	Nolte, William	William L. Nolte	tt0030089	
1746503813	1920	1921	Greater Than Love	Greater Than Love	Stahl, John M.	Fred Niblo	tt0012242	
1746422633	1993	1993	What's Love Got to Do with It	What's Love Got to Do with It	Gibson, Brian	Brian Gibson	tt0108551	
1746570118	1933	1933	Her Splendid Folly	Her Splendid Folly	O'Connor, William	William A. O'Connor	tt0024114	
1746584381	1940	1940	All This, and Heaven Too	All This, and Heaven Too	Litvak, Anatole	Anatole Litvak	tt0032194	
1745293198	2006	2006	Unaccompanied Minors	Unaccompanied Minors	Feig, Paul	Paul Feig	tt0488658	
1746509356	1949	1949	The Fighting Kentuckian	The Fighting Kentuckian	Waggner, George	George Waggner	tt0041361	
1746580421	1937	1937	Something to Sing About	Something to Sing About	Schertzinger, Victor	Victor Schertzinger	tt0029588	
1746514123	1923	1923	The Covered Schooner	The Covered Schooner	Edwards, Harry	Harry Edwards	tt0246534	
1746455237	2000	1999	Held Up	Held Up	Rash, Steve	Steve Rash	tt0165831	
1746573787	1956	1956	Crime in the Streets	Crime in the Streets	Siegel, Don	Don Siegel	tt0049107	
1746500815	1914	1914	A Gentleman from Mississippi	A Gentleman from Mississippi	Sargent, George L.	George L. Sargent	tt0004006	
1746573781	1958	1958	The Cool and the Crazy	The Cool and the Crazy	Witney, William	William Witney	tt0051488	
1746582856	1936	1936	Born to Dance	Born to Dance	Del Ruth, Roy	Roy Del Ruth	tt0027387	
1746583100	1935	1935	Wild Mustang	Wild Mustang	Fraser, Harry	Harry L. Fraser	tt0027217	
1746584425	1939	1939	On Your Toes	On Your Toes	Enright, Ray	Ray Enright	tt0031756	
1746504162	1922	1922	One Wonderful Night	One Wonderful Night	Paton, Stuart	Stuart Paton	tt0013464	
1746508091	1925	1925	Kit Carson Over the Great Divide	Kit Carson Over the Great Divide	Mattison, Frank S.	Frank S. Mattison	tt0015981	
1746569602	1958	1958	Jet Attack	Jet Attack	Cahn, Edward L.	Edward L. Cahn	tt0051797	
1746530705	1949	1949	Madame Bovary	Madame Bovary	Minnelli, Vincente	Vincente Minnelli	tt0041615	
1746436371	1943	1943	Corvette K-225	Corvette K 225	Rosson, Richard	Richard Rosson	tt0035757	
1745358902	2009	2008	Phoebe in Wonderland	Phoebe in Wonderland	Barnz, Daniel	Daniel Barnz	tt1034325	
1746503440	1917	1917	The Lad and the Lion	The Lad and the Lion	Green, Alfred	Alfred E. Green	tt0008165	
1746523849	1917	1917	Greed	Greed	Marston, Theodore	Theodore Marston	tt0175685	
1746208260	1969	1969	Once You Kiss a Stranger	Once You Kiss a Stranger...	Sparr, Robert	Robert Sparr	tt0064760	
1745272043	2000	2000	The Next Best Thing	The Next Best Thing	Schlesinger, John	John Schlesinger	tt0156841	
1746524984	1915	1915	The Final Judgment	The Final Judgment	Carewe, Edwin	Edwin Carewe	tt0005326	
1746570439	1933	1933	Hell and High Water	Hell and High Water	Jones, Grover; McNutt, William Slav	Grover Jones	tt0024101	
1745372899	2008	2008	The Love Guru	The Love Guru	Schnabel, Marco	Marco Schnabel	tt0811138	
1746532423	1917	1917	The Heir of the Ages	Heir of the Ages	Le Saint, Edward J.	Edward LeSaint	tt0008064	
1746499950	1948	1948	Who Killed Doc Robbin	Who Killed Doc Robbin?	Carr, Bernard	Bernard Carr	tt0040966	
1746520040	1924	1924	Safe and Sane	Safe and Sane	Beaudine, Harold	Harold Beaudine	tt0229693	
1746523848	1916	1916	The Sunbeam	The Sunbeam	Carewe, Edwin	Edwin Carewe	tt0159006	
1746508719	1927	1927	Rookies	Rookies	Wood, Sam	Sam Wood	tt0018338	
1746502819	1915	1915	Carmen	Carmen	Walsh, Raoul A.	Cecil B. DeMille	tt0005060	
1746581139	1961	1961	X-15	X 15	Donner, Richard D.	Richard Donner	tt0055627	
1746577742	1936	1936	Absolute Quiet	Absolute Quiet	Seitz, George B.	George B. Seitz	tt0027250	
1746433703	1995	1995	Born to Be Wild	Born to Be Wild	Gray, John	John Gray	tt0113533	
1746524511	1910	1910	Love and Money	Love and Money		Fred J. Balshofer	tt0359620	
1746584019	1936	1937	Galloping Dynamite	Galloping Dynamite	Fraser, Harry	Harry L. Fraser	tt0132990	

1746502581	1915	1915	A Mother's Confession	A Mother's Confession	Abramson, Ivan	Ivan Abramson	tt0005775
1745373726	2003	2003	A Good Night to Die	A Good Night to Die	Singer, Craig	Craig Singer	tt0203536
1745359318	2004	2004	Before Sunset	Before Sunset	Linklater, Richard	Richard Linklater	tt0381681
1746533230	1915	1915	The Ploughshare	The Ploughshare	Collins, John H.	John H. Collins	tt0005904
1746583063	1937	1937	Mile-a-Minute-Love	Mile a Minute Love	Clifton, Elmer	Elmer Clifton	tt0029244
1746474465	1948	1948	Campus Sleuth	Campus Sleuth	Jason, Will	Will Jason	tt0040208
1746577465	1962	1962	The Shameless	The Shameless	Martin, Jay	Joel Holt	tt0230763
1745372558	2010	2010	When in Rome	When in Rome	Johnson, Mark Steven	Mark Steven Johnson	tt1185416
1746422294	1998	1998	Jack Frost	Jack Frost	Miller, Troy	Troy Miller	tt0141109
1746509185	1926	1926	Speeding Through	Speeding Through	Bracken, Bertram	Bertram Bracken	tt0166359
1746520776	1925	1925	Fair Play	Fair Play	Crane, Frank	Frank Hall Crane	tt0131376
1745358504	2010	2010	It's Kind of a Funny Story	It's Kind of a Funny Story	Fleck, Ryan; Boden, Anna	Anna Boden	tt0804497
1746424444	1940	1940	Wildcat Bus	Wildcat Bus	Woodruff, Frank	Frank Woodruff	tt0033265
1746520284	1927	1927	Out of the Past	Out of the Past	Fitzgerald, Dallas M.	Dallas M. Fitzgerald	tt0018241
1746453793	1942	1942	The Falcon's Brother	The Falcon's Brother	Logan, Stanley	Stanley Logan	tt0034717
1746574967	1936	1935	Widow from Monte Carlo	The Widow from Monte Carlo	Collins, Arthur Greville	Arthur Greville Collins	tt0027215
1745340054	2002	2001	The Lost Skeleton of Cadavra	The Lost Skeleton of Cadavra	Blamire, Larry	Larry Blamire	tt0307109
1746496767	1919	1919	Gates of Brass	Gates of Brass	Warde, Ernest C.	Ernest C. Warde	tt0010156
1746235083	1977	1977	Annie Hall	Annie Hall	Allen, Woody	Woody Allen	tt0075686
1746409925	1990	1990	Far Out Man	Far Out Man	Chong, Tommy	Tommy Chong	tt0099546
1746580487	1935	1935	Women Must Dress	Women Must Dress	Barker, Reginald	Reginald Barker	tt0027229
1746501894	1910	1910	Thief	The Thief		Tom Ricketts	tt1705958
1746453580	1940	1940	Saturday's Children	Saturday's Children	Sherman, Vincent	Vincent Sherman	tt0033024
1746581304	1910	1910	John Halifax, Gentleman	John Halifax, Gentleman		Theodore Marston	tt0339224
1746274205	1980	1980	Bronco Billy	Bronco Billy	Eastwood, Clint	Clint Eastwood	tt0080472
1746501217	1915	1915	The High Road	The High Road	Noble, John W.	John W. Noble	tt0137814
1746583775	1930	1930	Love Comes Along	Love Comes Along	Julian, Rupert	Rupert Julian	tt0021097
1746504566	1917	1917	The Little Samaritan	The Little Samaritan	Levering, Joseph	Joseph Levering	tt0219134
1746520171	1949	1949	Make Mine Laughs	Make Mine Laughs	Fleischer, Richard O.	Richard Fleischer	tt0041621
1746465062	1945	1945	Mexicana	Mexicana	Santell, Alfred	Alfred Santell	tt0037909
1746497243	1918	1918	The Deciding Kiss	The Deciding Kiss	Browning, Tod	Tod Browning	tt0009001
1746545314	1940	1940	Girls Under 21	Girls Under 21	Nosseck, Max	Max Nosseck	tt0032529
1746524053	1923	1923	Salomy Jane	Salomy Jane	Melford, George	George Melford	tt0014431
1746492444	1944	1944	Trail to Gunsight	Trail to Gunsight	Keays, Vernon	Vernon Keays	tt0037391
1745372583	2010	2010	The Next Three Days	The Next Three Days	Haggis, Paul	Paul Haggis	tt1458175
1746582932	1935	1935	False Pretenses	False Pretenses	Lamont, Charles	Charles Lamont	tt0026333
1746554308	1964	1964	Vengeance	Vengeance	Hilyard, Dene	Dene Hilyard	tt0058717
1746584396	1939	1939	Whispering Enemies	Whispering Enemies	Collins, Lewis D.	Lewis D. Collins	tt0032127
1745341587	2010	2010	Predators	Predators	Antal, Nimród	Nimród Antal	tt1424381
1746574747	1935	1935	Show Them No Mercy!	Show Them No Mercy!	Marshall, George	George Marshall	tt0026992
1746514554	1914	1914	Forgiven; or, the Jack of Diamonds	Forgiven; Or, The Jack of Diamonds	Daly, William Robert	William Robert Daly	tt0003987
1746573692	1910	1910	A Colonial Belle	A Colonial Belle		Sidney Olcott	tt0393164
1746514362	1916	1916	Cured	Cured	Metcalfe, Earl	Earl Metcalfe	tt0429922
1746562039	1955	1955	Creature with the Atom Brain	Creature with the Atom Brain	Cahn, Edward L.	Edward L. Cahn	tt0047960
1746524492	1915	1915	The Reward	The Reward	Barker, Reginald	Reginald Barker	tt0005964
1746577992	1938	1938	Flight to Fame	Flight to Fame	Coleman, C. C.	Charles C. Coleman	tt0030141
1746454852	1944	1944	Adventure in Music	Adventure in Music	Matray, Ernst; Winston, S. K.; LeBorg	Reginald Le Borg	tt0240323
1746514570	1914	1914	Thirty Leagues Under the Sea	Thirty Leagues Under the Sea	Gregory, Carl L.	Carl Gregory	tt0004690
1746438297	1941	1941	The Bad Man	The Bad Man	Thorpe, Richard	Richard Thorpe	tt0033368
1746455645	1996	1996	Big Bully	Big Bully	Miner, Steve	Steve Miner	tt0115676
1746424388	1942	1942	Today I Hang	Today I Hang	Merrick, Geo. M.; Drake, Oliver	Oliver Drake	tt0035448
1746582703	1938	1937	Mama Runs Wild	Mama Runs Wild	Staub, Ralph	Ralph Staub	tt0029195
1746569512	1954	1954	Bengal Brigade	Bengal Brigade	Benedek, Laslo	Laslo Benedek	tt0046767
1746256208	1980	1980	Falling in Love Again	Falling in Love Again	Paul, Steven	Steven Paul	tt0080714
1746572964	1932	1932	Secrets of the French Police	Secrets of the French Police	Sutherland, Edward	A. Edward Sutherland	tt0023447
1746504406	1918	1918	Heredity	Heredity	Earle, William P. S.	William P.S. Earle	tt0184565
1746436370	1940	1940	Ellery Queen, Master Detective	Ellery Queen, Master Detective	Neumann, Kurt	Kurt Neumann	tt0032438

1769969338	1931	1931	House of Mystery	House of Mystery	Neumann, Kurt	Kurt Neumann	tt0135457
1746574022	1962	1962	Advise & Consent	Advise & Consent	Preminger, Otto	Otto Preminger	tt0055728
1746474953	1947	1947	The Law Comes to Gunsight	The Law Comes to Gunsight	Hillyer, Lambert	Lambert Hillyer	tt0039557
1746474083	1947	1947	Philo Vance's Gamble	Philo Vance's Gamble	Wrangell, Basil	Basil Wrangell	tt0039702
1746500714	1926	1926	The Two-Gun Man	The Two Gun Man	Kirkland, David	David Kirkland	tt0017495
1746504070	1920	1920	The Miracle of Money	The Miracle of Money	Henley, Hobart	Hobart Henley	tt0011466
1746520786	1949	1949	The Lawton Story	The Lawton Story	Beaudine, William	William Beaudine	tt0043936
1746555744	1955	1955	Lord of the Jungle	Lord of the Jungle	Beebe, Ford	Ford Beebe	tt0048314
1746530717	1949	1949	Home of the Brave	Home of the Brave	Robson, Mark	Mark Robson	tt0041481
1746580376	1935	1935	Outlaw Rule	Outlaw Rule	Luby, S. Roy	S. Roy Luby	tt0026837
1746465193	1942	1942	Sleepytime Gal	Sleepytime Gal	Rogell, Albert S.	Albert S. Rogell	tt0035344
1746569594	1953	1953	Three Sailors and a Girl	Three Sailors and a Girl	Del Ruth, Roy	Roy Del Ruth	tt0046424
1746504109	1921	1921	The Invisible Fear	The Invisible Fear	Carewe, Edwin	Edwin Carewe	tt0012319
1746581376	1910	1910	The Little Preacher	The Little Preacher		William F. Haddock	tt2239164
1746503305	1919	1919	Coax Me	Coax Me	Hamilton, Gilbert	Gilbert P. Hamilton	tt0247253
1746583567	1931	1931	The Single Sin	The Single Sin	Nigh, William	William Nigh	tt0022388
1745357703	2008	2008	Good Dick	Good Dick	Palka, Marianna	Marianna Palka	tt0944101
1746582570	1938	1938	Shine on Harvest Moon	Shine on Harvest Moon	Kane, Joe	Joseph Kane	tt0030742
1746576965	1963	1962	Daughter of the Sun God	Daughter of the Sun God	Herts, Kenneth	Kenneth Hartford	tt0055891
1746465397	1947	1947	Killer Dill	Killer Dill	Collins, Lewis D.	Lewis D. Collins	tt0039530
1746453642	1941	1941	The Trial of Mary Dugan	The Trial of Mary Dugan	McLeod, Norman Z.	Norman Z. McLeod	tt0034311
1746520544	1927	1927	Uneasy Payments	Uneasy Payments	Kirkland, David	David Kirkland	tt0175281
1746578959	1951	1951	Leave It to the Marines	Leave It to the Marines	Newfield, Samuel	Sam Newfield	tt0043730
1746453632	1941	1941	Kansas Cyclone	Kansas Cyclone	Sherman, George	George Sherman	tt0033779
1746502547	1910	1910	The Navajo's Bride	The Navajo's Bride		Sidney Olcott	tt1975292
1769976731	1929	1929	Oft in the Silly Night	Oft in the Silly Night	Gillstrom, Arvid E.	Arvid E. Gillstrom	tt0254614
1746567106	1932	1932	The Impatient Maiden	The Impatient Maiden	Whale, James	James Whale	tt0023055
1746514962	1915	1915	A Girl of Yesterday	A Girl of Yesterday	Dwan, Allan	Allan Dwan	tt0005392
1746509450	1950	1950	Copper Canyon	Copper Canyon	Farrow, John	John Farrow	tt0042344
1746574423	1951	1951	Bird of Paradise	Bird of Paradise	Daves, Delmer	Delmer Daves	tt0043343
1746409431	1988	1988	Punchline	Punchline	Seltzer, David	David Seltzer	tt0095927
1745374460	2002	2002	Punch-Drunk Love	Punch Drunk Love	Anderson, Paul Thomas	Paul Thomas Anderson	tt0272338
1746233167	1966	1966	Lt. Robin Crusoe, U.S.N.	Lt. Robin Crusoe, U.S.N.	Paul, Byron	Byron Paul	tt0060640
1746409442	1989	1989	Say Anything...	Say Anything...	Crowe, Cameron	Cameron Crowe	tt0098258
1746580529	1939	1939	Good Girls Go to Paris	Good Girls Go to Paris	Hall, Alexander	Alexander Hall	tt0031383
1746421985	1997	1997	G. I. Jane	G.I. Jane	Scott, Ridley	Ridley Scott	tt0119173
1746199842	1968	1968	For Love of Ivy	For Love of Ivy	Mann, Daniel	Daniel Mann	tt0062985
1746409359	1984	1984	Hot Dog...The Movie	Hot Dog... The Movie	Markle, Peter	Peter Markle	tt0087425
1746574958	1933	1933	Bye-Gones	Bye Gones	Goulding, Alf	Alfred J. Goulding	tt0023861
1745341319	2008	2008	Indiana Jones and the Kingdom of the Crystal Sk	Indiana Jones and the Kingdom of the Cryst	Spielberg, Steven	Steven Spielberg	tt0367882
1746567747	1933	1933	Terror Trail	Terror Trail	Schaefer, Armand	Armand Schaefer	tt0024650
1746524439	1916	1916	The Iron Woman	The Iron Woman	Harbaugh, Carl	Carl Harbaugh	tt0006870
1746420397	1993	1993	Menace II Society	Menace II Society	Hughes, Allen ; Hughes, Albert	Albert Hughes	tt0107554
1746501501	1916	1916	The Girl with the Green Eyes	The Girl with the Green Eyes	Blaché, Herbert	Herbert Blaché	tt0006722
1746568337	1934	1934	Private Scandal	Private Scandal	Murphy, Ralph	Ralph Murphy	tt0025682
1746581627	1951	1951	Crosswinds	Crosswinds	Foster, Lewis R.	Lewis R. Foster	tt0043434
1746410128	1982	1982	Diner	Diner	Levinson, Barry	Barry Levinson	tt0083833
1746583708	1928	1928	Her Summer Hero	Her Summer Hero	Dugan, James	James Dugan	tt0134719
1746421154	1991	1991	Homicide	Homicide	Mamet, David	David Mamet	tt0102048
1746583668	1931	1931	The Mad Genius	The Mad Genius	Curtiz, Michael	Michael Curtiz	tt0022103
1746564454	1951	1951	Double Crossbones	Double Crossbones	Barton, Charles T.	Charles Barton	tt0042411
1746512866	1916	1916	Rose of the Alley	Rose of the Alley	Horan, Charles	Charles Horan	tt0004544
1746439145	1944	1944	Henry Aldrich's Little Secret	Henry Aldrich's Little Secret	Bennett, Hugh	Hugh Bennett	tt0036908
1769970420	1967	1967	Free Love Confidential	Free Love Confidential	Heller, Gordon	Gordon Heller	tt0062992
1746554648	1964	1964	Passion Street, U. S. A.	Passion Street, U.S.A.	Daley, Oscar	Oscar Daley	tt0223888
1746501067	1914	1914	The Chechako	The Chechako	Bosworth, Hobart	Hobart Bosworth	tt0003765
1746409070	1984	1984	Breakin'	Breakin'	Silberg, Joel	Joel Silberg	tt0086998

1746562191	1953	1953	Fort Algiers	Fort Algiers	Selander, Lesley	Lesley Selander	tt0045783
1746569494	1957	1957	Young and Dangerous	Young and Dangerous	Claxton, William F.	William F. Claxton	tt0051215
1746216864	1968	1968	A Face of War	A Face of War	Jones, Eugene S.	Eugene S. Jones	tt0169885
1746571321	1933	1933	Secrets of Hollywood	Secrets of Hollywood	Todd, Holbrook	George M. Merrick	tt0024540
1746560018	1964	1964	633 Squadron	633 Squadron	Grauman, Walter	Walter Grauman	tt0057811
1746569653	1953	1953	Taxi	Taxi	Ratoff, Gregory	Gregory Ratoff	tt0046405
1746584426	1940	1940	Green Hell	Green Hell	Whale, James	James Whale	tt0032558
1746504361	1920	1920	The Devil's Claim	The Devil's Claim	Swickard, Charles	Charles Swickard	tt0011112
1746504195	1918	1918	Revenge	Revenge	Browning, Tod	Tod Browning	tt0009544
1746584916	1939	1939	The Middleton Family at the New York World's F	The Middleton Family at the New York Worl	Snody, Robert R.	Robert R. Snody	tt0031645
1745293153	2005	2004	My Date with Drew	My Date with Drew	Gunn, Jon ; Herzlinger, Brian ; Winn,	Jon Gunn	tt0378407
1746554379	1964	1964	Iron Angel	Iron Angel	Kennedy, Ken	Ken Kennedy	tt0228438
1746502726	1913	1913	The Legend of Provence	The Legend of Provence		Eugene Moore	tt0003058
1746581806	1910	1910	Jean and the Calico Doll	Jean and the Calico Doll		Laurence Trimble	tt0340183
1746217516	1967	1967	Don't Make Waves	Don't Make Waves	Mackendrick, Alexander	Alexander Mackendrick	tt0061590
1746235490	1974	1974	A Very Natural Thing	A Very Natural Thing	Larkin, Christopher	Christopher Larkin	tt0072362
1746582939	1938	1938	Mars Attacks the World	Mars Attacks the World	Beebe, Ford; Hill, Robert	Ford Beebe	tt0030423
1746513157	1916	1915	The Test	The Test	Fitzmaurice, George	James W. Castle	tt0343086
1746509498	1927	1927	Love	The Dove	Goulding, Edmund	Roland West	tt0017822
1746569745	1958	1958	Indiscreet	Indiscreet	Donen, Stanley	Stanley Donen	tt0051773
1746574831	1935	1935	A Notorious Gentleman	A Notorious Gentleman	Laemmle, Edward	Edward Laemmle	tt0026792
1746578627	1962	1961	The Big Wave	The Big Wave	Danielewski, Tad	Tad Danielewski	tt0189391
1746574463	1935	1935	His Night Out	His Night Out	Nigh, William	William Nigh	tt0026477
1746515823	1922	1922	Foolish Lives	Foolish Lives		Arvid E. Gillstrom	tt0332623
1746585265	1928	1928	Hit of the Show	Hit of the Show	Ince, Ralph	Ralph Ince	tt0019006
1746577887	1937	1937	Woman Against the World	Woman Against the World	Selman, David	David Selman	tt0030984
1746573912	1963	1962	The Raiders of Leyte Gulf	The Raiders of Leyte Gulf	Romero, Eddie	Eddie Romero	tt0057445
1769970576	1968	1968	The Night Hustlers	The Night Hustlers	O'Donald, Bobby	Bobby O'Donald	tt0253321
1746585182	1928	1928	The Apache	The Apache	Rosen, Philip	Phil Rosen	tt0018649
1746491891	1946	1946	The Kid from Brooklyn	The Kid from Brooklyn	McLeod, Norman Z.	Norman Z. McLeod	tt0038668
1746507798	1926	1926	Her Big Night	Her Big Night	Brown, Melville W.	Melville W. Brown	tt0016957
1746583813	1929	1929	The House of Secrets	The House of Secrets	Lawrence, Edmund	Edmund Lawrence	tt0123108
1746580269	1938	1938	It's All in Your Mind	It's All in Your Mind	Ray, Bernard B.	Bernard B. Ray	tt0030281
1746504374	1919	1919	The Mayor of Filbert	The Mayor of Filbert	Cabanne, William Christy	Christy Cabanne	tt0010447
1746578558	1960	1960	Noose for a Gunman	Noose for a Gunman	Cahn, Edward L.	Edward L. Cahn	tt0054126
1746502447	1916	1916	A Million a Minute	A Million a Minute	Noble, John W.	John W. Noble	tt0007065
1746584332	1937	1937	Clarence	Clarence	Archainbaud, George	George Archainbaud	tt0028722
1746235759	1976	1976	Squirm	Squirm	Lieberman, Jeff	Jeff Lieberman	tt0075261
1746585252	1929	1929	The Donovan Affair	The Donovan Affair	Capra, Frank R.	Frank Capra	tt0019831
1746186192	1965	1965	Eve and the Merman	Eve and the Merman	Royton, Chev	Roy P. Cheverton	tt0136814
1746453776	1943	1943	Smart Guy	Smart Guy	Hillyer, Lambert	Lambert Hillyer	tt0036366
1746453465	1941	1942	Thundering Hoofs	Thundering Hoofs	Selander, Lesley	Lesley Selander	tt0034290
1746394670	1990	1990	Taking Care of Business	Taking Care of Business	Hiller, Arthur	Arthur Hiller	tt0103035
1746577586	1937	1937	Make a Wish	Make a Wish	Neumann, Kurt	Kurt Neumann	tt0029193
1746581701	1910	1910	The Greater Love	The Greater Love		Edwin S. Porter	tt1064757
1746209157	1968	1968	Salt & Pepper	Salt and Pepper	Donner, Richard D.	Richard Donner	tt0063534
1746494362	1911	1911	When Love Was Blind	When Love Was Blind	Henderson, Lucius J.	Lucius Henderson	tt0409478
1746581037	1951	1951	Purple Heart Diary	Purple Heart Diary	Quine, Richard	Richard Quine	tt0043942
1746496384	1918	1918	Riders of the Night	Riders of the Night	Collins, John H.	John H. Collins	tt0009551
1746420512	1993	1993	Dazed and Confused	Dazed and Confused	Linklater, Richard	Richard Linklater	tt0106677
1746555232	1954	1954	It Should Happen to You	It Should Happen to You	Cukor, George	George Cukor	tt0047123
1746456098	1994	1994	Serial Mom	Serial Mom	Waters, John	John Waters	tt0111127
1746464822	1942	1942	A Desperate Chance for Ellery Queen	A Desperate Chance for Ellery Queen	Hogan, James	James P. Hogan	tt0034645
1746581964	1928	1928	Tempest	Tempest	Taylor, Sam	Sam Taylor	tt0019451
1746569631	1955	1955	The Tender Trap	The Tender Trap	Walters, Charles	Charles Walters	tt0048705
1746435925	1996	1996	It's My Party	It's My Party	Kleiser, Randal	Randal Kleiser	tt0113443
1746454177	1997	1996	Suburbia	SubUrbia	Linklater, Richard	Richard Linklater	tt0120238

1746219591	1972	1972	Play It Again, Sam	Play It Again, Sam	Ross, Herbert	Herbert Ross	tt0069097
1746475935	1947	1947	Lost Honeymoon	Lost Honeymoon	Jason, Leigh	Leigh Jason	tt0039582
1746533029	1923	1923	The Abysmal Brute	Abysmal Brute	Henley, Hobart	Hobart Henley	tt0013814
1746500098	1946	1946	Prairie Badmen	Prairie Badmen	Newfield, Sam	Sam Newfield	tt0038856
1746482898	1917	1917	Betty Be Good	Betty Be Good	MacDonald, Sherwood	Sherwood MacDonald	tt0167041
1745341020	2008	2007	Kabluey	Kabluey	Prendergast, Scott	Scott Prendergast	tt0816545
1746512658	1927	1927	Desert Dust	Desert Dust	Wyler, William	William Wyler	tt0017802
1746465213	1944	1944	Rationing	Rationing	Goldbeck, Willis	Willis Goldbeck	tt0037211
1746583162	1939	1939	First Love	First Love	Koster, Henry	Henry Koster	tt0031311
1746524765	1916	1916	The Bruiser	The Bruiser	Bartlett, Charles	Charles Bartlett	tt0006473
1746248259	1968	1968	Three Guns for Texas	Three Guns for Texas	Rich, David Lowell; Stanley, Paul; Be	Earl Bellamy	tt0063691
1746515696	1918	1918	Closin' in	Closin' In	McLaughlin, J. W.	James McLaughlin	tt0184345
1746570352	1934	1934	David Harum	David Harum	Cruze, James	James Cruze	tt0025035
1746574977	1936	1935	Just My Luck	Just My Luck	Heinz, Russell Ray	Ray Heinz	tt0027832
1746495917	1918	1918	Together	Together	Lund, O. A. C.	O.A.C. Lund	tt0009708
1746525162	1920	1920	Dad's Girl	Dad's Girl	Fischer, David G.	David G. Fischer	tt0190343
1746572132	1932	1932	Men Are Such Fools	Men Are Such Fools	Nigh, William	William Nigh	tt0023206
1746515776	1922	1922	The Woman He Loved	The Woman He Loved	Sloman, Edward	Edward Sloman	tt0013779
1746575927	1932	1932	One Way Passage	One Way Passage	Garnett, Tay	Tay Garnett	tt0023305
1769970827	1915	1915	Syd and His Sweethearts	Syd and His Sweethearts	De Grey, Sidney	Sidney De Gray	tt1456629
1746561982	1955	1955	Wine of Morning	Wine of Morning	Stenholm, Katherine	Katherine Stenholm	tt0497057
1746513819	1916	1916	Other People's Money	Other People's Money	Parke, William	William Parke	tt0163132
1746584344	1939	1939	Chip of the Flying U	Chip of the Flying U	Staub, Ralph	Ralph Staub	tt0032336
1746565292	1932	1932	Dangers of the Arctic	Dangers of the Arctic	Rossman, Earl	Earl Rossman	tt1270623
1746465032	1946	1946	'Neath Canadian Skies	'Neath Canadian Skies	Eason, B. Reeves	B. Reeves Eason	tt0038277
1746577170	1951	1951	A Millionaire for Christy	A Millionaire for Christy	Marshall, George	George Marshall	tt0043807
1746524342	1916	1915	Cross Currents	Cross Currents	Grandon, Francis	Francis J. Grandon	tt0005160
1746582691	1936	1936	Half Angel	Half Angel	Lanfield, Sidney	Sidney Lanfield	tt0027713
1769973935	1925	1926	Raisin-Trouble!!	Raisin' Trouble	Pembroke, "Perc"	Scott Pembroke	tt1074590
1745272314	2000	2000	Nutty Professor II: The Klumps	Nutty Professor II: The Klumps	Segal, Peter	Peter Segal	tt0144528
1745342898	2004	2004	Starsky & Hutch	Starsky & Hutch	Phillips, Todd	Todd Phillips	tt0335438
1746512743	1913	1913	A Princess of Bagdad	A Princess of Bagdad	Gaskill, Charles L.	Charles L. Gaskill	tt0003298
1746248604	1967	1967	Mondo Mod	Mondo Mod	Perry, Peter	Peter Perry Jr.	tt0174030
1746585165	1930	1930	Under Texas Skies	Under Texas Skies	McGowan, J. P.	J.P. McGowan	tt0021502
1746393270	1987	1987	Fatal Attraction	Fatal Attraction	Lyne, Adrian	Adrian Lyne	tt0093010
1746555218	1957	1957	The Dalton Girls	The Dalton Girls	LeBorg, Reginald	Reginald Le Borg	tt0050285
1746584305	1939	1939	Westbound Stage	Westbound Stage	Bennett, Spencer Gordon	Spencer Gordon Bennet	tt0033252
1746437851	1940	1940	I Can't Give You Anything but Love, Baby	I Can't Give You Anything But Love, Baby	Rogell, Albert S.	Albert S. Rogell	tt0032616
1746235703	1978	1978	Bloodbrothers	Bloodbrothers	Mulligan, Robert	Robert Mulligan	tt0078878
1746584423	1940	1940	The Letter	The Letter	Wyler, William	William Wyler	tt0032701
1746410753	1994	1994	Getting Even with Dad	Getting Even with Dad	Deutch, Howard	Howard Deutch	tt0109891
1746582719	1936	1936	The Bridge of Sighs	The Bridge of Sighs	Rosen, Phil	Phil Rosen	tt0027398
1745302305	2006	2006	Maple Palm	Maple Palm	Torjan, Ralph	Ralph Torjan	tt0481572
1746581652	1951	1951	Cuban Fireball	Cuban Fireball	Beaudine, William	William Beaudine	tt0043437
1746493391	1910	1910	The Stolen Fortune	The Stolen Fortune	Ricketts, Thomas	Tom Ricketts	tt1661444
1746410856	1993	1993	Hexed	Hexed	Spencer, Alan	Alan Spencer	tt0107107
1746491778	1944	1944	Tucson Raiders	Tucson Raiders	Bennet, Spencer	Spencer Gordon Bennet	tt0037402
1746504021	1921	1921	White and Unmarried	White and Unmarried	Forman, Tom	Tom Forman	tt0012844
1746409494	1985	1985	Out of Africa	Out of Africa	Pollack, Sydney	Sydney Pollack	tt0089755
1746520659	1927	1927	Not for Publication	Not for Publication	Ince, Ralph	Ralph Ince	tt0142704
1746584560	1931	1931	Hell's Valley	Hell's Alley	Neitz, Alvin J.	Thomas Moseley	tt0366563
1746566276	1955	1955	Kentucky Rifle	Kentucky Rifle	Hittleman, Carl K.	Carl K. Hittleman	tt0049403
1746570570	1933	1933	Shadows of Sing Sing	Shadows of Sing Sing	Rosen, Phil	Phil Rosen	tt0025769
1746491750	1946	1946	The Cockeyed Miracle	The Cockeyed Miracle	Simon, S. Sylvan	S. Sylvan Simon	tt0038420
1746561315	1954	1954	Witness to Murder	Witness to Murder	Rowland, Roy	Roy Rowland	tt0047679
1746564757	1953	1953	By the Light of the Silvery Moon	By the Light of the Silvery Moon	Butler, David	David Butler	tt0045586
1745372853	2003	2003	Anything Else	Anything Else	Allen, Woody	Woody Allen	tt0313792

1746464837	1945	1944	Meet Me in St. Louis	Meet Me in St. Louis	Minnelli, Vincente	Vincente Minnelli	tt0037059
1746568136	1934	1934	Hat, Coat, and Glove	Hat, Coat, and Glove	Miner, Worthington	Worthington Miner	tt0025222
1746524585	1928	1928	Man from Headquarters	The Man from Headquarters	Worne, Duke	Duke Worne	tt0019132
1746464802	1942	1942	Beyond the Blue Horizon	Beyond the Blue Horizon	Santell, Alfred	Alfred Santell	tt0034510
1746504122	1920	1920	The Girl in Number 29	The Girl in Number 29	Ford, Jack	John Ford	tt0011228
1746582923	1939	1939	Six-Gun Rhythm	Six Gun Rhythm	Newfield, Sam	Sam Newfield	tt0031929
1746423500	1991	1991	All I Want for Christmas	All I Want for Christmas	Lieberman, Robert	Robert Lieberman	tt0101301
1746577449	1951	1951	Lullaby of Broadway	Lullaby of Broadway	Butler, David	David Butler	tt0043762
1746559892	1965	1965	Requiem for a Gunfighter	Requiem for a Gunfighter	Bennet, Spencer Gordon	Spencer Gordon Bennet	tt0059647
1746410450	1987	1987	Nuts	Nuts	Ritt, Martin	Martin Ritt	tt0093660
1745393799	2008	2008	The Bank Job	The Bank Job	Donaldson, Roger	Roger Donaldson	tt0200465
1746453783	1940	1940	Trailing Double Trouble	Trailing Double Trouble	Luby, S. Roy	S. Roy Luby	tt0033181
1746210157	1971	1971	Catlow	Catlow	Wanamaker, Sam	Sam Wanamaker	tt0066900
1746514559	1916	1916	A Corner in Colleens	A Corner in Colleens	Miller, Charles	Charles Miller	tt0157484
1746514871	1923	1923	Tipped Off	Tipped Off	Fox, Finis	Finis Fox	tt0014551
1746562328	1956	1956	Gaby	Gaby	Bernhardt, Curtis	Curtis Bernhardt	tt0049247
1746524690	1927	1927	The Beloved Rogue	The Beloved Rogue	Crosland, Alan	Alan Crosland	tt0017667
1746555392	1954	1954	The Scarlet Spear	The Scarlet Spear	Breakston, George; Stahl, Ray	George P. Breakston	tt0047452
1746465339	1944	1944	Frontier Outlaws	Frontier Outlaws	Newfield, Sam	Sam Newfield	tt0036842
1746465028	1947	1947	Jiggs and Maggie in Society	Jiggs and Maggie in Society	Cline, Eddie	Edward F. Cline	tt0039513
1746454834	1942	1942	Private Buckaroo	Private Buckaroo	Cline, Edward F.	Edward F. Cline	tt0035218
1746574435	1958	1958	The World Was His Jury	The World Was His Jury	Sears, Fred F.	Fred F. Sears	tt0052407
1746515725	1922	1922	Spitfire	Spitfire			tt0896863
1746488023	1918	1918	A Bachelor's Children	A Bachelor's Children	Scardon, Paul	Paul Scardon	tt0008858
1746497034	1920	1920	That Something	That Something	Wilson, Margery; Underwood, Lawre	Margery Wilson	tt0011759
1745341608	2002	2002	Ballistic: Ecks vs. Sever	Ballistic: Ecks vs. Sever	Kaos	Wych Kaosayananda	tt0308208
1746502003	1914	1914	The Tangle	The Tangle	Lambart, Harry	Harry Lambart	tt0004668
1746497889	1919	1919	The Unknown Love	Unknown Love	Perret, Léonce	Léonce Perret	tt0010822
1746516272	1920	1920	Love Without Question	Love Without Question	Rolfe, B. A.	B.A. Rolfe	tt0011417
1746509017	1949	1949	The Green Promise	The Green Promise	Russell, William D.	William D. Russell	tt0041431
1746582695	1935	1935	Four Hours to Kill!	Four Hours to Kill!	Leisen, Mitchell	Mitchell Leisen	tt0026378
1746410886	1989	1989	Relentless	Relentless	Lustig, William	William Lustig	tt0098184
1746409250	1987	1987	3 Men and a Baby	3 Men and a Baby	Nimoy, Leonard	Leonard Nimoy	tt0094137
1746569737	1953	1953	Mesa of Lost Women	Mesa of Lost Women	Tevos, Herbert; Ormond, Ron	Ron Ormond	tt0046066
1746233960	1966	1965	Taylor Mead's Ass	Taylor Mead's Ass	Warhol, Andy	Andy Warhol	tt0244835
1746578054	1938	1938	Romance of the Limberlost	Romance of the Limberlost	Nigh, William	William Nigh	tt0030692
1746435484	1943	1943	Mr. Muggs Steps Out	Mr. Muggs Steps Out	Beaudine, William	William Beaudine	tt0036175
1746574904	1935	1935	Hard Rock Harrigan	Hard Rock Harrigan	Howard, David	David Howard	tt0026454
1746497572	1919	1919	The House of Intrigue	The House of Intrigue	Ingraham, Lloyd	Lloyd Ingraham	tt0010272
1746438292	1945	1945	Boston Blackie Booked on Suspicion	Boston Blackie Booked on Suspicion	Dreifuss, Arthur	Arthur Dreifuss	tt0037553
1746570285	1934	1934	The Road to Ruin	The Road to Ruin	Reid, Wallace; Shyer, Melville	Dorothy Davenport	tt0025725
1746574056	1961	1961	Everything's Ducky	Everything's Ducky	Taylor, Don	Don Taylor	tt0054860
1746486156	1919	1919	Counterfeit	Counterfeit	Fitzmaurice, George	George Fitzmaurice	tt0144845
1746250600	1980	1980	American Gigolo	American Gigolo	Schrader, Paul	Paul Schrader	tt0080365
1746198210	1969	1969	Pit Stop	Pit Stop	Hill, Jack	Jack Hill	tt0064819
1746410527	1992	1992	Diggstown	Diggstown	Ritchie, Michael	Michael Ritchie	tt0104107
1746420154	1981	1981	Hell Night	Hell Night	De Simone, Tom	Tom DeSimone	tt0082511
1746423714	1940	1940	Rangers of Fortune	Rangers of Fortune	Wood, Sam	Sam Wood	tt0032973
1746438469	1941	1941	Double Date	Double Date	Tryon, Glenn	Glenn Tryon	tt0033548
1746232996	1968	1968	The Bamboo Saucer	The Bamboo Saucer	Telford, Frank	Frank Telford	tt0062704
1746581272	1961	1961	The Ruined Bruin	The Ruined Bruin	McCarthy, John K.	John K. McCarthy	tt0445684
1746420898	1995	1995	Angus	Angus	Johnson, Patrick Read	Patrick Read Johnson	tt0112368
1746571768	1910	1910	The Course of True Love	The Course of True Love	Powell, Frank	D.W. Griffith	tt0001165
1746583562	1951	1951	Navy Bound	Navy Bound	Landres, Paul	Paul Landres	tt0043843
1745272077	2000	2000	Whipped	Whipped	Cohen, Peter M.	Peter M. Cohen	tt0174336
1746525128	1921	1921	The Ten Dollar Raise	The Ten Dollar Raise	Sloman, Edward	Edward Sloman	tt0012744
1746438608	1944	1944	Jam Session	Jam Session	Barton, Charles	Charles Barton	tt0036966

1746513659	1916	1916	The Dark Silence	The Dark Silence	Capellani, Albert	Albert Capellani	tt0157508
1746584434	1930	1930	Manslaughter	Manslaughter	Abbott, George	George Abbott	tt0021121
1746485839	1920	1920	The Wonderful Chance	The Wonderful Chance	Archainbaud, George	George Archainbaud	tt0011881
1746542643	1924	1924	Huntin' Trouble	Huntin' Trouble	Maloney, Leo D.; Williamson, Bob	Leo D. Maloney	tt0135464
1746577795	1933	1933	She Had to Say Yes	She Had to Say Yes	Berkeley, Busby; Amy, George	George Amy	tt0024549
1746567612	1933	1933	The Women in His Life	The Women in His Life	Seitz, George B.	George B. Seitz	tt0026006
1746209739	1971	1971	The Barefoot Executive	The Barefoot Executive	Butler, Robert	Robert Butler	tt0066811
1745359857	2001	2001	Dogtown and Z-Boys	Dogtown and Z Boys	Peralta, Stacy	Stacy Peralta	tt0275309
1769969440	1964	1964	Dr. Sex	Dr. Sex		Ted V. Mikels	tt0058037
1746433772	1998	1998	I Still Know What You Did Last Summer	I Still Know What You Did Last Summer	Cannon, Danny	Danny Cannon	tt0130018
1746498318	1919	1919	Hearts Asleep	Hearts Asleep	Hickman, Howard	Howard C. Hickman	tt0203559
1746503615	1920	1920	Beautifully Trimmed	Beautifully Trimmed	De Sano, Marcel	Marcel De Sano	tt0010984
1746524046	1916	1916	The World and the Woman	The World and the Woman	Moore, Eugene	Frank Lloyd	tt0007590
1746184678	1965	1965	Psycho A Go-Go!	Psycho a Go Go	Adamson, Al	Al Adamson	tt0134915
1746410217	1990	1990	The Invisible Maniac	The Invisible Maniac	Coogan, Rif	Adam Rifkin	tt0099856
1746580252	1936	1936	I Conquer the Sea!	I Conquer the Sea!	Halperin, Victor	Victor Halperin	tt0027774
1746454626	1944	1944	Since You Went Away	Since You Went Away	Cromwell, John	John Cromwell	tt0037280
1746580938	1910	1910	Jane Eyre	Jane Eyre		Theodore Marston	tt0290677
1746435913	1992	1991	There's Nothing Out There	There's Nothing Out There	Kanefsky, Rolfe	Rolfe Kanefsky	tt0103077
1746577725	1933	1933	Going Hollywood	Going Hollywood	Walsh, Raoul	Raoul Walsh	tt0024067
1746532468	1914	1914	His Last Dollar	His Last Dollar		Frank Powell	tt0004097
1746583993	1936	1936	A Man Betrayed	A Man Betrayed	Auer, John H.	John H. Auer	tt0029199
1746577739	1935	1935	I Dream Too Much	I Dream Too Much	Cromwell, John	John Cromwell	tt0026507
1746503567	1920	1920	Oh, Lady, Lady	Oh, Lady, Lady	Campbell, Maurice	Maurice Campbell	tt0011527
1746569709	1957	1957	The Midnight Story	The Midnight Story	Pevney, Joseph	Joseph Pevney	tt0050704
1746500738	1927	1927	Loco Luck	Loco Luck	Smith, Cliff	Clifford Smith	tt0018096
1746453826	1944	1944	Thundering Gun Slingers	Thundering Gun Slingers	Newfield, Sam	Sam Newfield	tt0037375
1746583966	1939	1939	Ambush	Ambush	Neumann, Kurt	Kurt Neumann	tt0031039
1745309637	2007	2007	The Reaping	The Reaping	Hopkins, Stephen	Stephen Hopkins	tt0444682
1746570579	1934	1933	Dinner at Eight	Dinner at Eight	Cukor, George	George Cukor	tt0023948
1746555526	1955	1955	The Vanishing American	The Vanishing American	Kane, Joe	Joseph Kane	tt0048773
1746500960	1913	1913	Victory	Victory	Read, J. Parker	J. Parker Read Jr.	tt0003508
1746198473	1966	1967	Road to Nashville	The Road to Nashville	Zens, Will	Will Zens	tt0268582
1746520708	1925	1925	Never Too Late	Never Too Late	Sheldon, Forrest	Forrest Sheldon	tt0016149
1746503570	1918	1918	By Right of Purchase	By Right of Purchase	Miller, Charles	Charles Miller	tt0184314
1746516478	1920	1920	The River's End	The River's End	Neilan, Marshall; Heerman, Victor	Victor Heerman	tt0011632
1746438471	1942	1942	Call of the Canyon	Call of the Canyon	Santley, Joseph	Joseph Santley	tt0034568
1746508070	1948	1948	Tornado Range	Tornado Range	Taylor, Ray	Ray Taylor	tt0040889
1746503692	1921	1921	The Speed Girl	The Speed Girl	Campbell, Maurice	Maurice Campbell	tt0012718
1746454363	1941	1941	Tuxedo Junction	Tuxedo Junction	McDonald, Frank	Frank McDonald	tt0034319
1746273672	1980	1980	Humanoids from the Deep	Humanoids from the Deep	Peeters, Barbara	Barbara Peeters	tt0080904
1746423685	1940	1940	Dr. Ehrlich's Magic Bullet	Dr. Ehrlich's Magic Bullet	Dieterle, William	William Dieterle	tt0032413
1746554339	1965	1965	Space	Space	Warhol, Andy	Andy Warhol	tt0196966
1746514632	1916	1916	Why Love Is Blind	Why Love Is Blind	Nichols, George	George Nichols	tt0436969
1746424381	1941	1941	The Lone Rider Ambushed	The Lone Rider Ambushed	Newfield, Sam	Sam Newfield	tt0033841
1746530782	1950	1950	Father Makes Good	Father Makes Good	Yarbrough, Jean	Jean Yarbrough	tt0042450
1746570159	1952	1952	Breakdown	Breakdown	Angelo, Edmond	Edmond Angelo	tt0044445
1746562310	1955	1955	Revenge of the Creature	Revenge of the Creature	Arnold, Jack	Jack Arnold	tt0048554
1746509232	1925	1925	Easy Money	Easy Money	Rogell, Albert	Albert S. Rogell	tt0015777
1745282663	2006	2006	Mini's First Time	Mini's First Time	Guthe, Nick	Nick Guthe	tt0425253
1746583111	1934	1934	White Heat	White Heat	Weber, Lois	Lois Weber	tt0025985
1746508440	1926	1926	The Hidden Way	The Hidden Way	De Grasse, Joseph	Joseph De Grasse	tt0016964
1746584757	1928	1928	Free Lips	Free Lips	MacDonald, Wallace	Wallace MacDonald	tt0018916
1746584678	1930	1930	Lotus Lady	Lotus Lady	Rosen, Phil	Phil Rosen	tt0021095
1746566234	1955	1955	The Shrike	The Shrike	Ferrer, José	José Ferrer	tt0048614
1746500125	1924	1924	Driftwood	Driftwood		Roland West	tt0356556
1746524856	1928	1928	The Olympic Hero	The Olympic Hero	Neill, R. William	Roy William Neill	tt0019232

1746419799	1993	1993	Six Degrees of Separation	Six Degrees of Separation	Schepisi, Fred	Fred Schepisi	tt0108149	
1746508742	1926	1926	Meet the Prince	Meet the Prince	Henabery, Joseph	Joseph Henabery	tt0017131	
1746580523	1938	1938	Kentucky	Kentucky	Butler, David	David Butler	tt0030317	
1746583193	1931	1932	One Man Law	One Man Law	Hillyer, Lambert	Lambert Hillyer	tt0023304	
1746504009	1922	1922	No Trespassing	No Trespassing	Hollywood, Edwin L.	Edwin L. Hollywood	tt0013439	
1746424232	1940	1940	The Light of Western Stars	The Light of Western Stars	Selander, Lesley	Lesley Selander	tt0032706	
1746197975	1969	1969	Daddy's Gone A-Hunting	Daddy's Gone A Hunting	Robson, Mark	Mark Robson	tt0064209	
1746503749	1917	1917	The Valentine Girl	The Valentine Girl	Dawley, J. Searle	J. Searle Dawley	tt0008726	
1745340530	2002	2001	American Adobo	American Adobo	Guillen, Laurice	Laurice Guillen	tt0294289	
1769970072	1912	1912	The Non-Commissioned Officer	The Non Commissioned Officer	Edwin, Walter	Charles Brabin	tt0002393	
1746584940	1940	1940	Convicted Woman	Convicted Woman	Grinde, Nick	Nick Grinde	tt0032358	
1746514108	1914	1914	The Voice at the Telephone	The Voice at the Telephone	Giblyn, Charles	Charles Giblyn	tt0004769	
1746582438	1936	1936	A Woman Rebels	A Woman Rebels	Sandrich, Mark	Mark Sandrich	tt0028517	
1746566331	1958	1958	The Bravados	The Bravados	King, Henry	Henry King	tt0051433	
1746533204	1923	1923	Cyclone Jones	Cyclone Jones	Seeling, Charles R.	Charles R. Seeling	tt0013964	
1746408664	1990	1990	Die Hard 2	Die Hard 2	Harlin, Renny	Renny Harlin	tt0099423	
1746563812	1953	1953	Affair with a Stranger	Affair with a Stranger	Rowland, Roy	Roy Rowland	tt0045477	
1746500549	1926	1926	The Outlaw Express	The Outlaw Express	Maloney, Leo D.	Leo D. Maloney	tt0017239	
1746577873	1938	1938	Penitentiary	Penitentiary	Brahm, John	John Brahm	tt0030567	
1746218776	1973	1973	Bad Charleston Charlie	Bad Charleston Charlie	Nagy, Ivan	Ivan Nagy	tt0069758	
1746219193	1980	1980	Smokey and the Bandit II	Smokey and the Bandit II	Needham, Hal	Hal Needham	tt0081529	
1746516459	1920	1920	Red Foam	Red Foam	Ince, Ralph	Ralph Ince	tt0011615	
1746578624	1961	1961	By Love Possessed	By Love Possessed	Sturges, John	John Sturges	tt0054710	
1769969385	1930	1930	Crazy House	Crazy House	Cummings, Jack	Jack Cummings	tt0020796	
1746582955	1937	1937	King of Gamblers	King of Gamblers	Florey, Robert	Robert Florey	tt0029083	
**1746584421**	**1940**	**1940**	**Lillian Russell**	**Lillian Russell**	**Cummings, Irving**	**Irving Cummings**	**tt0032710**	1
1746577630	1933	1933	Design for Living	Design for Living	Lubitsch, Ernst	Ernst Lubitsch	tt0023940	
1746503355	1918	1918	Dolly Does Her Bit	Dolly Does Her Bit	Bertram, William	William Bertram	tt0184410	
1746498669	1918	1918	The Guilty Man	The Guilty Man	Willat, Irvin V.	Irvin Willat	tt0184539	
1746473716	1947	1947	King of the Bandits	King of the Bandits	Cabanne, Christy	Christy Cabanne	tt0039533	
1746493904	1910	1910	The Modern Prodigal	The Modern Prodigal	Griffith, D. W.	D.W. Griffith	tt0001326	
1746581945	1910	1910	How Bumptious Papered the Parlor	How Bumptious Papered the Parlor	Miller, Ashley	Ashley Miller	tt0477316	
1746508758	1927	1927	Ladies at Ease	Ladies at Ease	Storm, Jerome	Jerome Storm	tt0018073	
1746574695	1935	1935	The Spanish Cape Mystery	The Spanish Cape Mystery	Collins, Lewis D.	Lewis D. Collins	tt0027028	
1746453704	1998	1998	Free Tibet	Free Tibet	Pirozek, Sarah	Sarah Pirozek	tt0169921	
1746235363	1972	1972	A Sense of Loss	A Sense of Loss	Ophuls, Marcel	Marcel Ophüls	tt0069247	
1746499881	1948	1948	Daredevils of the Clouds	Daredevils of the Clouds	Blair, George	George Blair	tt0040269	
1746209491	1967	1967	The Happiest Millionaire	The Happiest Millionaire	Tokar, Norman	Norman Tokar	tt0061749	
1746422319	1996	1996	Jack	Jack	Coppola, Francis Ford	Francis Ford Coppola	tt0116669	
1746580237	1938	1938	Heart of Arizona	Heart of Arizona	Selander, Lesley	Lesley Selander	tt0030224	
1746235837	1977	1977	Exorcist II: The Heretic	Exorcist II: The Heretic	Boorman, John	John Boorman	tt0076009	
1746570262	1933	1933	Under the Tonto Rim	Under the Tonto Rim	Hathaway, Henry	Henry Hathaway	tt0024718	
1746520493	1927	1927	Thumbs Down	Thumbs Down	Rosen, Phil	Phil Rosen	tt0170684	
1745372561	2001	2001	One Night at McCool's	One Night at McCool's	Zwart, Harald	Harald Zwart	tt0203755	
1746424165	1940	1940	Devil Bat	The Devil Bat	Yarbrough, Jean	Jean Yarbrough	tt0032390	
1746465050	1945	1945	Blood on the Sun	Blood on the Sun	Lloyd, Frank	Frank Lloyd	tt0037547	
1746583715	1931	1931	Hell Bound	Hell Bound	Lang, Walter	Walter Lang	tt0021949	
1746574443	1951	1951	Spoilers of the Plains	Spoilers of the Plains	Witney, William	William Witney	tt0044064	
1746577858	1935	1935	Mystery of Edwin Drood	The Mystery of Edwin Drood	Walker, Stuart	Stuart Walker	tt0026758	
1746514660	1917	1917	The Dark Road	The Dark Road	Miller, Charles	Charles Miller	tt0181443	
1746514833	1916	1916	The Secret Seven	The Secret Seven	Humphrey, William	William Humphrey	tt0765203	
1746578811	1958	1958	Return to Warbow	Return to Warbow	Nazarro, Ray	Ray Nazarro	tt0052132	
1745309250	2006	2006	Beerfest	Beerfest	Chandrasekhar, Jay	Jay Chandrasekhar	tt0486551	
1746508536	1925	1925	Winds of Chance	Winds of Chance	Lloyd, Frank	Frank Lloyd	tt0016541	
1746502046	1914	1914	Lord Chumley	Lord Chumley	Kirkwood, James	James Kirkwood	tt0004254	
1746555756	1956	1956	Fighting Trouble	Fighting Trouble	Blair, George	George Blair	tt0049208	
1746422218	1991	1991	Poison	Poison	Haynes, Todd	Todd Haynes	tt0102687	

1746570399	1934	1934	Bright Eyes	Bright Eyes	Butler, David	David Butler	tt0024914
1746508223	1927	1927	The Lunatic at Large	The Lunatic at Large	Newmeyer, Fred	Fred C. Newmeyer	tt0018122
1746409276	1987	1987	Made in Heaven	Made in Heaven	Rudolph, Alan	Alan Rudolph	tt0093467
1746394675	1988	1988	A Nightmare on Elm Street 4: The Dream Master	A Nightmare on Elm Street 4: The Dream Ma	Harlin, Renny	Renny Harlin	tt0095742
1746580765	1935	1935	The Hoosier Schoolmaster	The Hoosier Schoolmaster	Collins, Lewis D.	Lewis D. Collins	tt0026492
1769970657	1967	1967	The Old Man's Bride	The Old Man's Bride	Gunter, George	George Gunter	tt0062071
1746580223	1936	1936	Winds of the Wasteland	Winds of the Wasteland	Wright, Mack V.	Mack V. Wright	tt0028510
1746585149	1928	1928	Four Sons	Four Sons	Ford, John	John Ford	tt0018909
1746499839	1950	1950	Law of the Panhandle	Law of the Panhandle	Collins, Lewis	Lewis D. Collins	tt0042668
1746465055	1945	1945	Nob Hill	Nob Hill	Hathaway, Henry	Henry Hathaway	tt0037946
1746578538	1959	1959	Juke Box Rhythm	Juke Box Rhythm	Dreifuss, Arthur	Arthur Dreifuss	tt0052951
1746486273	1917	1917	The Gift Girl	The Gift Girl	Julian, Rupert	Rupert Julian	tt0007999
1746561296	1955	1955	How To Be Very, Very Popular	How to Be Very, Very Popular	Johnson, Nunnally	Nunnally Johnson	tt0048183
1746578586	1951	1951	The Whistle at Eaton Falls	The Whistle at Eaton Falls	Siodmak, Robert	Robert Siodmak	tt0044212
1746501627	1923	1923	The Lone Horseman	The Lone Horseman	Caldwell, Fred	Fred Caldwell	tt0175857
1746566177	1954	1953	Miss Sadie Thompson	Miss Sadie Thompson	Bernhardt, Curtis	Curtis Bernhardt	tt0046076
1746532505	1910	1910	Making a Man of Him	Making a Man of Him			tt0348670
1746394207	1989	1989	Major League	Major League	Ward, David S.	David S. Ward	tt0097815
1746514136	1927	1927	In a Moment of Temptation	In a Moment of Temptation	Carle, Philip	Philip Carle	tt0018026
1746563629	1951	1951	Cavalry Scout	Cavalry Scout	Selander, Leslie	Lesley Selander	tt0043391
1769970564	1968	1968	The Secret Files of Detective "X"	The Secret Files of Detective 'X'	Hilliard, Richard	Richard Hilliard	tt0257116
1746410758	1994	1994	The Favor	The Favor	Petrie, Donald	Donald Petrie	tt0109783
1746454839	1944	1944	The Falcon in Mexico	The Falcon in Mexico	Berke, William	William Berke	tt0036811
1746520614	1926	1926	Blue Blazes	Blue Blazes	Franz, Joseph	Joseph Franz	tt0016663
1746437847	1943	1943	Hi' Ya, Chum	Hi'Ya Chum	Young, Harold	Harold Young	tt0035997
1746465049	1944	1944	Cheyenne Wildcat	Cheyenne Wildcat	Selander, Lesley	Lesley Selander	tt0036707
1746438322	1942	1942	Shut My Big Mouth	Shut My Big Mouth	Barton, Charles	Charles Barton	tt0035324
1746572797	1932	1932	The Big Stampede	The Big Stampede	Wright, Tenny	Tenny Wright	tt0022681
1746513198	1915	1915	The Galloper	The Galloper	MacKenzie, Donald	Donald MacKenzie	tt0005368
1746182925	1910	1910	A Cheyenne Brave	A Cheyenne Brave	Young Deer, James	James Young Deer	tt0233435
1746553953	1963	1963	Love Is a Ball	Love Is a Ball	Swift, David	David Swift	tt0057262
1746582573	1938	1938	Reckless Living	Reckless Living	McDonald, Frank	Frank McDonald	tt0030660
1746419921	1990	1990	Presumed Innocent	Presumed Innocent	Pakula, Alan J.	Alan J. Pakula	tt0100404
1746577428	1951	1951	Gene Autry and the Mounties	Gene Autry and The Mounties	English, John	John English	tt0043579
1746424602	1940	1940	I Want a Divorce	I Want a Divorce	Murphy, Ralph	Ralph Murphy	tt0032619
1745293262	2007	2007	Slipstream	Slipstream	Hopkins, Anthony	Anthony Hopkins	tt0499570
1746455338	1997	1997	I Know What You Did Last Summer	I Know What You Did Last Summer	Gillespie, Jim	Jim Gillespie	tt0119345
1746514987	1916	1916	Revelations	Revelation	Maude, Arthur	Arthur Maude	tt0248361
1746561177	1932	1932	Ride Him, Cowboy	Ride Him, Cowboy	Allen, Fred	Fred Allen	tt0023396
1746553902	1965	1965	The Raw Ones	The Raw Ones	Lamb, John	John Lamb	tt0189959
1746567087	1933	1933	Trouble Busters	Trouble Busters	Collins, Lew	Lewis D. Collins	tt0023621
1746475955	1946	1946	The Postman Always Rings Twice	The Postman Always Rings Twice	Garnett, Tay	Tay Garnett	tt0038854
1746514090	1915	1915	The Adventures of a Madcap	The Adventures of a Madcap			tt0004860
1746582172	1929	1929	Devil-May-Care	Devil May Care	Franklin, Sidney	Sidney Franklin	tt0019815
1746581873	1930	1930	Night Work	Night Work	Mack, Russell	Russell Mack	tt0021184
1746570287	1934	1934	These Thirty Years	These Thirty Years	Pincus, David	Dave Pincus	tt0157108
1746515394	1918	1918	Daybreak	Daybreak	Capellani, Albert	Albert Capellani	tt0184385
1746394201	1987	1987	Heaven	Heaven	Keaton, Diane	Diane Keaton	tt0093166
1746199530	1965	1965	Town Tamer	Town Tamer	Selander, Lesley	Lesley Selander	tt0059824
1746513417	1928	1928	The Last Command	The Last Command	Sternberg, Josef von	Josef von Sternberg	tt0019071
1746553697	1963	1963	Come Blow Your Horn	Come Blow Your Horn	Yorkin, Bud	Bud Yorkin	tt0056940
1746233515	1966	1966	The Chelsea Girls	Chelsea Girls	Warhol, Andy	Paul Morrissey	tt0061465
1746571325	1936	1936	Fury	Fury	Lang, Fritz	Fritz Lang	tt0027652
1746409513	1987	1987	Planes, Trains and Automobiles	Planes, Trains & Automobiles	Hughes, John	John Hughes	tt0093748
1746475496	1946	1946	Two Guys from Milwaukee	Two Guys from Milwaukee	Butler, David	David Butler	tt0039053
1746549974	1954	1954	Jungle Man-Eaters	Jungle Man Eaters	Sholem, Lee	Lee Sholem	tt0047139
1746572410	1932	1932	Flames	Flames	Brown, Karl	Karl Brown	tt0022895

1746583313	1929	1929	Thunderbolt	Thunderbolt	Sternberg, Josef von	Josef von Sternberg	tt0020499
1746409368	1982	1982	Star Trek II: The Wrath of Khan	Star Trek II: The Wrath of Khan	Meyer, Nicholas	Nicholas Meyer	tt0084726
1746209414	1965	1964	The Brig	The Brig	Mekas, Jonas; Mekas, Adolfas	Jonas Mekas	tt0057899
1746491783	1945	1945	The Beautiful Cheat	The Beautiful Cheat	Barton, Charles	Charles Barton	tt0038339
1746524502	1915	1915	The Woman	A Woman	Melford, George	Charles Chaplin	tt0006309
1746580245	1938	1938	The Texans	The Texans	Hogan, James	James P. Hogan	tt0030850
1746250163	1979	1979	Screams of a Winter Night	Screams of a Winter Night	Wilson, James L.	James L. Wilson	tt0079869
1746438554	1996	1996	I Shot Andy Warhol	I Shot Andy Warhol	Harron, Mary	Mary Harron	tt0116594
1746576998	1959	1959	Edge of Eternity	Edge of Eternity	Siegel, Don	Don Siegel	tt0052771
1746515998	1920	1920	Hairpins	Hairpins	Niblo, Fred	Fred Niblo	tt0011257
1746501496	1916	1916	The Man Who Would Not Die	The Man Who Would Not Die	Russell, William; Prescott, John	Jack Prescott	tt0007018
1746524017	1915	1915	The Earl of Pawtucket	The Earl of Pawtucket	Myers, Harry C.	Harry Myers	tt0005234
1746584094	1938	1938	Fast Company	Fast Company	Buzzell, Edward	Edward Buzzell	tt0030120
1746502210	1916	1916	Out of the Drifts	Out of the Drifts	Dawley, J. Searle	J. Searle Dawley	tt0007150
1746569810	1960	1960	The Hypnotic Eye	The Hypnotic Eye	Blair, George	George Blair	tt0053931
1746235294	1972	1972	The King of Marvin Gardens	The King of Marvin Gardens	Rafelson, Bob	Bob Rafelson	tt0068805
1746559873	1963	1963	The Haunted Palace	The Haunted Palace	Corman, Roger	Roger Corman	tt0057128
1746581175	1960	1959	Jazz on a Summer's Day	Jazz on a Summer's Day	Stern, Bert	Bert Stern	tt0052942
1746524850	1915	1915	The College Widow	The College Widow	O'Neil, Barry	Barry O'Neil	tt0005106
1746507795	1925	1925	Go Straight	Go Straight	O'Connor, Frank	Frank O'Connor	tt0015862
1746454841	1945	1945	The Crime Doctor's Courage	The Crime Doctor's Courage	Sherman, George	George Sherman	tt0037620
1745357623	2001	2001	A Knight's Tale	A Knight's Tale	Helgeland, Brian	Brian Helgeland	tt0183790
1746440315	1999	1999	julien donkey-boy	Julien Donkey Boy	Korine, Harmony	Harmony Korine	tt0192194
1746569558	1953	1953	She's Back on Broadway	She's Back on Broadway	Douglas, Gordon	Gordon Douglas	tt0046306
1746501338	1916	1916	Gretchen, the Greenhorn	Gretchen the Greenhorn	Franklin, C. M.; Franklin, S. A.	Chester M. Franklin	tt0006745
1746514448	1916	1916	Reclamation	The Reclamation	Sloman, Edward	Edward Sloman	tt0209273
1746577334	1950	1950	The Return of Jesse James	The Return of Jesse James	Hilton, Arthur David	Arthur Hilton	tt0042885
1746565889	1953	1953	Girl on the Run	Girl on the Run	Lee, Joseph; Beckhard, Arthur J.	Arthur J. Beckhard	tt0054930
1745359735	2008	2008	Baby Mama	Baby Mama	McCullers, Michael	Michael McCullers	tt0871426
1746578788	1958	1958	Gunman's Walk	Gunman's Walk	Karlson, Phil	Phil Karlson	tt0051690
1746514874	1915	1915	The Silent Voice	The Silent Voice	Bowman, William J.	William Bowman	tt0006043
1746440009	1998	1997	Chicago Cab	Chicago Cab	Cybulski, Mary; Tintori, John	Mary Cybulski	tt0119278
1746394506	1991	1991	Stone Cold	Stone Cold	Baxley, Craig R.	Craig R. Baxley	tt0102984
1746473933	1946	1946	Three Wise Fools	Three Wise Fools	Buzzell, Edward	Edward Buzzell	tt0039031
1746209479	1968	1968	The Lustful Turk	The Lustful Turk	Elliott, B. Ron	Byron Mabe	tt0063252
1746508570	1925	1925	Away in the Lead	Away in the Lead			tt0015588
1746421227	1994	1994	Jimmy Hollywood	Jimmy Hollywood	Levinson, Barry	Barry Levinson	tt0110197
1746580980	1950	1950	The West Point Story	The West Point Story	Del Ruth, Roy	Roy Del Ruth	tt0043123
1745271693	2000	2000	Urban Legends: Final Cut	Urban Legends: Final Cut	Ottman, John	John Ottman	tt0192731
1746503314	1918	1918	Chase Me Charlie	Chase Me Charlie	Chaplin, Charles	Charles Chaplin	tt0175524
1746520333	1926	1926	Ella Cinders	Ella Cinders	Green, Alfred E.	Alfred E. Green	tt0016822
1746530756	1950	1950	Dark City	Dark City	Dieterle, William	William Dieterle	tt0042379
1746582799	1935	1935	Man of Iron	Man of Iron	McGann, William	William C. McGann	tt0026674
1746474828	1948	1948	Canon City	Canon City	Wilbur, Crane	Crane Wilbur	tt0040210
1746554501	1963	1963	Hothead	Hothead	Mann, Edward	Edward Mann	tt0057158
1745303228	2006	2006	An Inconvenient Truth	An Inconvenient Truth	Guggenheim, Davis	Davis Guggenheim	tt0497116
1746497450	1918	1918	Her One Mistake	Her One Mistake	Le Saint, Edward J.	Edward LeSaint	tt0009167
1746435861	1992	1992	Bad Lieutenant	Bad Lieutenant	Ferrara, Abel	Abel Ferrara	tt0103759
1746566773	1932	1932	Self-Defense	Self Defense	Rosen, Phil	Phil Rosen	tt0024542
1746523713	1916	1916	A Night Out	A Night Out	Baker, George D.	George D. Baker	tt0005811
1746570025	1934	1934	The Return of Chandu	The Return of Chandu		Ray Taylor	tt0025712
1746577144	1960	1960	Cage of Evil	Cage of Evil	Cahn, Edward L.	Edward L. Cahn	tt0053689
1746516121	1922	1922	Across the Dead-Line	Across the Deadline	Conway, Jack	Jack Conway	tt0012878
1746514350	1917	1917	The Secret Man	The Secret Man	Ford, Jack	John Ford	tt0008551
1746520499	1927	1927	The Lure of the Night Club	Lure of the Night Club	Buckingham, Thomas	Tom Buckingham	tt0018125
1746499984	1924	1924	The Beautiful Sinner	The Beautiful Sinner	Van Dyke, W. S.	W.S. Van Dyke	tt0014703
1745375265	2008	2008	Witless Protection	Witless Protection	Carner, Charles Robert	Charles Robert Carner	tt1001562

1745342832	2003	2003	Calendar Girls	Calendar Girls	Cole, Nigel	Nigel Cole	tt0337909
1746566132	1955	1955	We're No Angels	We're No Angels	Curtiz, Michael	Michael Curtiz	tt0048801
1745359475	2004	2004	Team America: World Police	Team America: World Police	Parker, Trey	Trey Parker	tt0372588
1746574554	1935	1935	Courageous Avenger	The Courageous Avenger	Bradbury, Robert N.	Robert N. Bradbury	tt0026241
1746498673	1918	1918	The Model's Confession	A Model's Confession	Park, Ida May	Ida May Park	tt0009383
1746555403	1955	1955	Canyon Crossroads	Canyon Crossroads	Werker, Alfred L.	Alfred L. Werker	tt0047917
1746580902	1936	1936	The Last Outlaw	The Last Outlaw	Cabanne, Christy	Christy Cabanne	tt0027868
1746578597	1962	1962	Four for the Morgue	Four for the Morgue	Sledge, John	John Sledge	tt0212949
1746422292	1992	1992	Hugh Hefner: Once Upon a Time	Hugh Hefner: Once Upon a Time	Heath, Robert	Robert Heath	tt0104461
1746514515	1916	1916	The Other Girl	The Other Girl	Winter, Percy	Percy Winter	tt0163131
1746434893	1993	1993	Another Stakeout	Another Stakeout	Badham, John	John Badham	tt0106292
1769970422	1967	1967	I, Marquis de Sade	I, Marquis de Sade	Hilliard, Richard	Richard Hilliard	tt0220568
1746584933	1940	1940	Babies for Sale	Babies for Sale	Barton, Charles	Charles Barton	tt0032231
1746580574	1937	1937	Blake of Scotland Yard	Blake of Scotland Yard	Hill, Bob	Robert F. Hill	tt0028637
1769974254	1916	1916	A Duel in the Desert	The Duel in the Desert	Horne, James W.	James W. Horne	tt3140210
1745303206	2006	2005	The Great New Wonderful	The Great New Wonderful	Leiner, Danny	Danny Leiner	tt0402230
1746532727	1916	1916	The Red Mouse	The Red Mouse	Noble, John W.	John W. Noble	tt0158890
1746524361	1927	1927	The Cherokee Kid	The Cherokee Kid	De Lacy, Robert	Robert De Lacey	tt0017749
1746393013	1984	1984	Up the Creek	Up the Creek	Butler, Robert	Robert Butler	tt0088333
1746582804	1936	1936	Hollywood Boulevard	Hollywood Boulevard	Florey, Robert	Robert Florey	tt0027757
1746421631	1940	1940	Adventure in Diamonds	Adventure in Diamonds	Fitzmaurice, Geo.	George Fitzmaurice	tt0032183
1746493989	1910	1910	The Way of the World	The Way of the World	Griffith, D. W.	D.W. Griffith	tt0001446
1746534792	1920	1920	The Family Honor	The Family Honor	Vidor, King W.	King Vidor	tt0011172
1746520655	1948	1948	Whirlwind Raiders	Whirlwind Raiders	Keays, Vernon	Vernon Keays	tt0040964
1746579026	1963	1963	Goldilocks and the Three Bares	Goldilocks and the Three Bares	Gordon, Lewis H.	Herschell Gordon Lewis	tt0057111
1769970673	1968	1968	Acapulco Uncensored	Acapulco Uncensored		Donald A. Davis	tt0209886
1746584565	1931	1931	The Road to Reno	The Road to Reno	Wallace, Richard	Richard Wallace	tt0022320
1746454484	1941	1941	The Kid from Kansas	The Kid from Kansas	Nigh, William	William Nigh	tt0033783
1746410155	1987	1987	Police Academy 4: Citizens On Patrol	Police Academy 4: Citizens on Patrol	Drake, Jim	Jim Drake	tt0093756
1746571380	1932	1932	Huddle	Huddle		Sam Wood	tt0023034
1746410687	1986	1986	Out of Bounds	Out of Bounds	Tuggle, Richard	Richard Tuggle	tt0091706
1746574770	1935	1935	Unconquered Bandit	Unconquered Bandit	Webb, Harry S.	Harry S. Webb	tt0027153
1746513667	1923	1923	The Last Moment	The Last Moment		J. Parker Read Jr.	tt0014188
1746582778	1938	1938	Crime Takes a Holiday	Crime Takes a Holiday	Collins, Lewis D.	Lewis D. Collins	tt0030027
1746562177	1953	1953	Murder Without Tears	Murder Without Tears	Beaudine, William	William Beaudine	tt0046105
1746507720	1926	1927	The Fighting Stallion	The Fighting Stallion	Wilson, Ben	Ben F. Wilson	tt0016867
1746453466	1944	1944	Strangers in the Night	Strangers in the Night	Mann, Anthony	Anthony Mann	tt0037318
1746467639	1917	1917	Happiness	Happiness	Barker, Reginald	Reginald Barker	tt0008044
1746583893	1928	1928	Driftin' Sands	Driftin' Sands	Fox, Wallace W.	Wallace Fox	tt0018852
1746580535	1936	1936	Yellowstone	Yellowstone	Lubin, Arthur	Arthur Lubin	tt0028523
1746520715	1950	1949	My Foolish Heart	My Foolish Heart	Robson, Mark	Mark Robson	tt0041672
1746436281	1998	1998	How Stella Got Her Groove Back	How Stella Got Her Groove Back	Sullivan, Kevin Rodney	Kevin Rodney Sullivan	tt0120703
1746219753	1977	1977	Chatterbox!	Chatterbox!	De Simone, Tom	Tom DeSimone	tt0075830
1746492562	1944	1944	Something for the Boys	Something for the Boys	Seiler, Lewis	Lewis Seiler	tt0037295
1746586170	1940	1940	The Great Profile	The Great Profile	Lang, Walter	Walter Lang	tt0032556
1746514619	1915	1915	Blackbirds	Blackbirds	McGowan, J. P.	J.P. McGowan	tt0004978
1746487827	1918	1918	The Whirlpool	The Whirlpool	Crosland, Alan	Alan Crosland	tt0009795
1746569845	1958	1957	Old Yeller	Old Yeller	Stevenson, Robert	Robert Stevenson	tt0050798
1746394509	1982	1982	The Beach Girls	The Beach Girls	Townsend, Pat	Bud Townsend	tt0083628
1746492507	1947	1947	The Fabulous Texan	The Fabulous Texan	Ludwig, Edward	Edward Ludwig	tt0039367
1746410363	1985	1985	Movers & Shakers	Movers & Shakers	Asher, William	William Asher	tt0089628
1746581740	1928	1928	Undressed	Undressed	Rosen, Philip	Phil Rosen	tt0019510
1746474396	1948	1948	Key Largo	Key Largo	Huston, John	John Huston	tt0040506
1746574645	1934	1934	Once to Every Woman	Once to Every Woman	Hillyer, Lambert	Lambert Hillyer	tt0025596
1746454401	1942	1942	Parachute Nurse	Parachute Nurse	Barton, Charles	Charles Barton	tt0035172
1746497643	1917	1917	The Haunted Pajamas	The Haunted Pajamas	Balshofer, Fred J.	Fred J. Balshofer	tt0008053
1746582417	1938	1938	Hawaiian Buckaroo	Hawaiian Buckaroo	Taylor, Ray	Ray Taylor	tt0030218

1746577700	1933	1933	Notorious but Nice	Notorious But Nice	Thorpe, Richard	Richard Thorpe	tt0024394
1746465020	1944	1944	Louisiana Hayride	Louisiana Hayride	Barton, Charles	Charles Barton	tt0037028
1746501677	1923	1923	Michael O'Halloran	Michael O'Halloran	Meehan, James Leo	James Leo Meehan	tt0014271
1746492709	1947	1947	The Arnelo Affair	The Arnelo Affair	Oboler, Arch	Arch Oboler	tt0039160
1746420216	1991	1991	Hook	Hook	Spielberg, Steven	Steven Spielberg	tt0102057
1746218828	1973	1973	Manson	Manson	Merrick, Laurence	Robert Hendrickson	tt0068918
1746566104	1958	1958	The Naked and the Dead	The Naked and the Dead	Walsh, Raoul	Raoul Walsh	tt0051978
1746492493	1948	1948	The Paleface	The Paleface	McLeod, Norman Z.	Norman Z. McLeod	tt0040679
1746197936	1966	1966	Crazy Quilt	The Crazy Quilt	Korty, John	John Korty	tt0060260
1746453517	1941	1941	The People vs. Dr. Kildare	The People vs. Dr. Kildare	Bucquet, Harold S.	Harold S. Bucquet	tt0034013
1746474125	1945	1945	Zombies on Broadway	Zombies on Broadway	Douglas, Gordon	Gordon Douglas	tt0038270
1746393762	1981	1981	Nighthawks	Nighthawks	Malmuth, Bruce	Bruce Malmuth	tt0082817
1746574118	1957	1957	The Sun Also Rises	The Sun Also Rises	King, Henry	Henry King	tt0051028
1746393294	1981	1980	The Earthling	The Earthling	Collinson, Peter	Peter Collinson	tt0080671
1746185703	1965	1964	The Train	The Train	Frankenheimer, John; Penn, Arthur	John Frankenheimer	tt0059825
1746232937	1969	1969	The Learning Tree	The Learning Tree	Parks, Gordon	Gordon Parks	tt0064579
1746436375	1943	1942	Eyes of the Underworld	Eyes of the Underworld	Neill, Roy William	Roy William Neill	tt0035850
1746524236	1915	1915	Hearts in Exile	Hearts in Exile	Young, James	James Young	tt0005460
1746570137	1934	1934	Guilty Parents	Guilty Parents	Townley, Jack	Jack Townley	tt0025210
1746577824	1939	1938	Gang Smashers	Gang Smashers	Popkin, Leo C.	Leo C. Popkin	tt0135426
1746464922	1941	1941	The Forgotten Village	The Forgotten Village	Kline, Herbert	Herbert Kline	tt0033623
1746553737	1965	1965	Crack in the World	Crack in the World	Marton, Andrew	Andrew Marton	tt0059065
1746514263	1910	1910	Sunshine Sue	Sunshine Sue	Griffith, D. W.	D.W. Griffith	tt0001407
1746434034	1997	1997	Fathers' Day	Fathers' Day	Reitman, Ivan	Ivan Reitman	tt0119109
1746203223	1970	1970	Watermelon Man	Watermelon Man	Van Peebles, Melvin	Melvin Van Peebles	tt0066550
1746583834	1930	1930	Children of Pleasure	Children of Pleasure	Beaumont, Harry	Harry Beaumont	tt0020763
1746498677	1922	1922	Bing Bang Boom	Bing Bang Boom	Butler, Fred J.	Fred J. Butler	tt0012942
1746571300	1935	1935	The Law of 45's	The Law of 45's	McCarthy, John P.	John P. McCarthy	tt0026612
1746577432	1950	1950	The Strip-Tease Murder Case	The Strip Tease Murder Case	Prince, Hugh	Hugh Prince	tt0043009
1746520686	1925	1925	Tearing Through	Tearing Through	Rosson, Arthur	Arthur Rosson	tt0168213
1746514127	1915	1915	Ghosts	Ghosts	Nicholls, George	George Nichols	tt0005381
1746509460	1925	1925	Sporting Life	Sporting Life	Tourneur, Maurice	Maurice Tourneur	tt0016389
1746198887	1969	1969	The Activist	The Activist	Napoleon, Art	Art Napoleon	tt0063984
1746581271	1950	1950	Sierra Passage	Sierra Passage	McDonald, Frank	Frank McDonald	tt0042964
1746569727	1954	1954	Tennessee Champ	Tennessee Champ	Wilcox, Fred M.	Fred M. Wilcox	tt0047566
1746573065	1932	1932	Big City Blues	Big City Blues	LeRoy, Mervyn	Mervyn LeRoy	tt0022676
1746499914	1923	1923	Gimme	Gimme	Hughes, Rupert	Rupert Hughes	tt0014082
1746485620	1918	1918	Riders of the Purple Sage	Riders of the Purple Sage	Lloyd, Frank	Frank Lloyd	tt0009552
1746410190	1985	1985	Turk 182!	Turk 182!	Clark, Bob	Bob Clark	tt0090217
1746467070	1917	1917	The Mate of the Sally Ann	The Mate of the Sally Ann	King, Henry	Henry King	tt0008278
1746475920	1946	1946	Adventure	Adventure	Fleming, Victor	Leonard Anderson	tt1144467
1746503348	1920	1919	Vengeance and the Girl	Vengeance and the Girl	De La Mothe, Leon	Leon De La Mothe	tt0010838
1745341869	2010	2010	Cool It	Cool It	Timoner, Ondi	Ondi Timoner	tt1694015
1746465035	1942	1942	United We Stand	United We Stand			tt0954353
1746566224	1953	1953	Ambush at Tomahawk Gap	Ambush at Tomahawk Gap	Sears, Fred F.	Fred F. Sears	tt0045502
1746496129	1919	1919	Wanted: A Husband	Wanted: A Husband	Windom, Lawrence C.	Lawrence C. Windom	tt0010856
1746523324	1915	1915	The Buzzard's Shadow	The Buzzard's Shadow	Ricketts, Thomas	Tom Ricketts	tt0005043
1746435272	1942	1942	Tombstone: The Town Too Tough to Die	Tombstone: The Town Too Tough to Die	McGann, William	William C. McGann	tt0035450
1746516037	1920	1920	His House in Order	His House in Order	Ford, Hugh	Hugh Ford	tt0011296
1746561233	1932	1932	Tomorrow and Tomorrow	Tomorrow and Tomorrow	Wallace, Richard	Richard Wallace	tt0022487
1746475912	1945	1944	Thirty Seconds Over Tokyo	Thirty Seconds Over Tokyo	LeRoy, Mervyn	Mervyn LeRoy	tt0037366
1746576841	1932	1932	The Golden West	The Golden West	Howard, David	David Howard	tt0022953
1746186302	1968	1968	Hellfighters	Hellfighters	McLaglen, Andrew V.	Andrew V. McLaglen	tt0063060
1746500583	1925	1925	Share and Share Alike	Share and Share Alike	Bennett, Whitman	Whitman Bennett	tt0163825
1745359040	2004	2004	Little Black Book	Little Black Book	Hurran, Nick	Nick Hurran	tt0361841
1746567228	1932	1932	The Final Edition	The Final Edition	Higgin, Howard	Howard Higgin	tt0022888
1746575351	1932	1932	Devil and the Deep	Devil and the Deep	Gering, Marion	Marion Gering	tt0022814

1746500409	1924	1924	The Law Forbids	The Law Forbids	Robbins, Jesse	Jess Robbins	tt0015056
1746514411	1910	1910	The Sheriff	The Sheriff		Francis Boggs	tt0354912
1746421972	1996	1996	Ghosts of Mississippi	Ghosts of Mississippi	Reiner, Rob	Rob Reiner	tt0116410
1746502065	1922	1922	The Curse of Drink	The Curse of Drink	Hoyt, Harry O.	Harry O. Hoyt	tt0013044
1746497221	1918	1918	A Weaver of Dreams	A Weaver of Dreams	Collins, John H.	John H. Collins	tt0009783
1746409463	1985	1985	Silverado	Silverado	Kasdan, Lawrence	Lawrence Kasdan	tt0090022
1746583869	1929	1929	Tonight at Twelve	Tonight at Twelve	Pollard, Harry A.	Harry A. Pollard	tt0020511
1746500712	1927	1927	She's My Baby	She's My Baby	Windemere, Fred	Fred Windemere	tt0018385
1746563864	1953	1953	The Affairs of Dobie Gillis	The Affairs of Dobie Gillis	Weis, Don	Don Weis	tt0045479
1746435117	1940	1940	The Quarterback	The Quarterback	Humberstone, H. Bruce	H. Bruce Humberstone	tt0032957
1746574222	1959	1959	The Big Operator	The Big Operator	Haas, Charles	Charles F. Haas	tt0052629
1746532409	1927	1927	A Gentleman of Paris	A Gentleman of Paris	D'Arrast, Harry d'Abbadie	Harry d'Abbadie d'Arrast	tt0017926
1746565974	1953	1953	Remains to Be Seen	Remains to Be Seen	Weis, Don	Don Weis	tt0046235
1746466900	1917	1917	The Price of Her Soul	The Price of Her Soul	Apfel, Oscar	Oscar Apfel	tt0008451
1746554576	1963	1963	The Ugly American	The Ugly American	Englund, George	George Englund	tt0056632
1746584155	1938	1938	Swing That Cheer	Swing That Cheer	Schuster, Harold	Harold D. Schuster	tt0030819
1746424556	1943	1943	Passport to Suez	Passport to Suez	DeToth, Andre	André De Toth	tt0036253
1746419206	1990	1990	The First Power	The First Power	Resnikoff, Robert	Robert Resnikoff	tt0099578
1746419610	1986	1986	Off Beat	Off Beat	Dinner, Michael	Michael Dinner	tt0091668
1746465378	1947	1947	Fall Guy	Fall Guy	Le Borg, Reginald	Reginald Le Borg	tt0039368
1746570736	1934	1934	Viva Villa	Viva Villa!	Conway, Jack; Hawks, Howard	Jack Conway	tt0025948
1746474114	1948	1948	Bodyguard	Bodyguard	Fleischer, Richard O.	Richard Fleischer	tt0040178
1746497037	1920	1920	It's a Great Life	It's a Great Life	Hopper, E. Mason	E. Mason Hopper	tt0143313
1746560633	1932	1932	Behind Jury Doors	Behind Jury Doors	Eason, Breezy	B. Reeves Eason	tt0023792
1746566994	1952	1952	Lone Star	Lone Star	Sherman, Vincent	Vincent Sherman	tt0043751
1746584653	1929	1929	The Four Feathers	The Four Feathers	Cooper, Merian C.; Schoedsack, Erne	Merian C. Cooper	tt0018908
1746234380	1968	1968	The Scalphunters	The Scalphunters	Pollack, Sydney	Sydney Pollack	tt0063557
1746571333	1931	1931	Cimarron	Cimarron	Ruggles, Wesley	Wesley Ruggles	tt0021746
1746581245	1951	1951	Desert of Lost Men	Desert of Lost Men	Keller, Harry	Harry Keller	tt0043462
1746565968	1953	1953	Topeka	Topeka	Carr, Thomas	Thomas Carr	tt0046445
1746581755	1910	1910	The Girl Spy Before Vicksburg	The Girl Spy Before Vicksburg	Olcott, Sidney	Sidney Olcott	tt0255193
1746504371	1917	1917	The War of the Tongs	The War of the Tongs		H.O. Davis	tt0008749
1746584385	1940	1940	The Howards of Virginia	The Howards of Virginia	Lloyd, Frank	Frank Lloyd	tt0032612
1769971149	1969	1969	The Screentest Girls	The Screentest Girls	S., Zoltan	Zoltan G. Spencer	tt0127807
1746424052	1940	1940	Up in the Air	Up in the Air	Bretherton, Howard	Howard Bretherton	tt0033214
1746512457	1917	1917	As Man Made Her	As Man Made Her	Archainbaud, George	George Archainbaud	tt0007652
1746514855	1916	1916	The Valiants of Virginia	The Valiants of Virginia	Heffron, T. N.	Thomas N. Heffron	tt0007508
1746577114	1951	1951	The Redhead and the Cowboy	The Redhead and the Cowboy	Fenton, Leslie	Leslie Fenton	tt0042881
1746562457	1957	1957	The Night Runner	The Night Runner	Biberman, Abner	Abner Biberman	tt0050764
1746562450	1954	1954	Track of the Cat	Track of the Cat	Wellman, William A.	William A. Wellman	tt0047603
1746508466	1925	1925	An Enemy Of Men	An Enemy of Men	Strayer, Frank R.	Frank R. Strayer	tt0016824
1746492453	1944	1944	The Pinto Bandit	The Pinto Bandit	Clifton, Elmer	Elmer Clifton	tt0037176
1746583573	1930	1930	Remote Control	Remote Control	St. Clair, Malcolm; Grinde, Nick; Sed	Nick Grinde	tt0021294
1746555925	1954	1954	Hell and High Water	Hell and High Water	Fuller, Samuel	Samuel Fuller	tt0047073
1746501011	1916	1916	Jane's Husband	Jane's Husband	Baker, George D.	George D. Baker	tt0223565
1746487045	1918	1918	Life or Honor?	Life or Honor?	Lawrence, Edmund	Edmund Lawrence	tt0009299
1746453970	1998	1998	Armageddon	Armageddon	Bay, Michael	Michael Bay	tt0120591
1746574673	1935	1935	The County Chairman	The County Chairman	Blystone, John	John G. Blystone	tt0026239
1746584378	1939	1939	Beware, Spooks!	Beware Spooks!	Sedgwick, Edward	Edward Sedgwick	tt0031095
1746420355	1988	1988	Nightfall	Nightfall	Mayersberg, Paul	Paul Mayersberg	tt0095738
1746216674	1967	1967	The Dirty Dozen	The Dirty Dozen	Aldrich, Robert	Robert Aldrich	tt0061578
1745293902	2006	2006	Poseidon	Poseidon	Petersen, Wolfgang	Wolfgang Petersen	tt0409182
1746464769	1946	1946	Child of Divorce	Child of Divorce	Fleischer, Richard O.	Richard Fleischer	tt0038411
1746210532	1971	1971	The Murder of Fred Hampton	The Murder of Fred Hampton	Gray, Mike	Howard Alk	tt0067456
1746532981	1927	1927	Code of the Range	Code of the Range	Cohn, Bennett; Schlank, Morris R.	Bennett Cohen	tt0312576
1746453823	1941	1941	I'll Sell My Life	I'll Sell My Life	Clifton, Elmer	Elmer Clifton	tt0033747
1746570576	1952	1952	Red Ball Express	Red Ball Express	Boetticher, Budd	Budd Boetticher	tt0045072

1746504222	1920	1920	High Speed	High Speed	Miller, Charles	Charles Miller	tt0195781
1746500688	1926	1926	Obey the Law	Obey the Law	Raboch, Alfred	Alfred Raboch	tt0017217
1746583096	1938	1938	Outlaws of Sonora	Outlaws of Sonora	Sherman, George	George Sherman	tt0030538
1746582768	1936	1936	Border Flight	Border Flight	Lovering, Otho	Otho Lovering	tt0027383
1746584482	1930	1930	Sinners' Holiday	Sinners' Holiday	Adolfi, John G.	John G. Adolfi	tt0021381
1746408486	1986	1986	Invaders from Mars	Invaders from Mars	Hooper, Tobe	Tobe Hooper	tt0091276
1746520684	1926	1926	Lightning Bill	Lightning Bill	Chaudet, Louis	Louis Chaudet	tt0017066
1746269637	1980	1980	A Small Circle of Friends	A Small Circle of Friends	Cohen, Rob	Rob Cohen	tt0081528
1746436636	1941	1941	Ride, Kelly, Ride	Ride, Kelly, Ride	Foster, Norman	Norman Foster	tt0034101
1746249270	1975	1975	Johnny Firecloud	Johnny Firecloud	Castleman, William A.	William Allen Castleman	tt0073210
1746555426	1954	1954	Silver Lode	Silver Lode	Dwan, Allan	Allan Dwan	tt0047495
1746567989	1933	1933	Ace of Aces	Ace of Aces	Ruben, J. Walter	J. Walter Ruben	tt0023737
1746542651	1924	1924	Who's Cheating?	Who's Cheating?	Levering, Joseph	Joseph Levering	tt0015496
1745374755	2009	2009	Confessions of a Shopaholic	Confessions of a Shopaholic	Hogan, P. J.	P.J. Hogan	tt1093908
1746503969	1919	1919	Daring Hearts	Daring Hearts	Houry, Henry	Henry Houry	tt0010047
1746509319	1926	1926	The Checkered Flag	The Checkered Flag	Adolfi, John G.	John G. Adolfi	tt0016720
1746492729	1947	1947	Last Frontier Uprising	Last Frontier Uprising	Selander, Lesley	Lesley Selander	tt0039552
1746465394	1947	1947	The Long Night	The Long Night	Litvak, Anatole	Anatole Litvak	tt0039581
1746424204	1942	1942	Heart of the Golden West	Heart of the Golden West	Kane, Joseph	Joseph Kane	tt0034832
1746582440	1938	1938	White Banners	White Banners	Goulding, Edmund	Edmund Goulding	tt0030973
1746235393	1974	1974	Dynamite Brothers	Dynamite Brothers	Adamson, Al	Al Adamson	tt0132933
1746393360	1984	1984	Sheena	Sheena	Guillermin, John	John Guillermin	tt0088103
1746564784	1953	1953	Confidentially Connie	Confidentially Connie	Buzzell, Edward	Edward Buzzell	tt0045644
1746453915	2000	2000	Wonder Boys	Wonder Boys	Hanson, Curtis	Curtis Hanson	tt0185014
1746524538	1927	1927	After Midnight	After Midnight	Bell, Monta	Monta Bell	tt0017593
1746581680	1928	1928	Romance of a Rogue	Romance of a Rogue	Baggot, King	King Baggot	tt0019334
1746532440	1910	1910	You Saved My Life	You Saved My Life			tt1896861
1746574421	1963	1963	The Man From the Diners' Club	The Man from the Diners' Club	Tashlin, Frank	Frank Tashlin	tt0057284
1746512615	1916	1916	The Last Man	The Last Man	Wolbert, William	William Wolbert	tt0006910
1746453531	1943	1942	Sherlock Holmes and the Secret Weapon	Sherlock Holmes and the Secret Weapon	Neill, Roy William	Roy William Neill	tt0035317
1746525061	1920	1920	Wings of Pride	Wings of Pride	Rolfe, B. A.	B.A. Rolfe	tt0203205
1746454931	1942	1942	Secrets of the Underground	Secrets of the Underground	Morgan, William	William Morgan	tt0036338
1746497873	1919	1919	Easy to Make Money	Easy to Make Money	Carewe, Edwin	Edwin Carewe	tt0010080
1746566639	1952	1952	Without Warning!	Without Warning!	Laven, Arnold	Arnold Laven	tt0045334
1746574350	1961	1961	The Big Gamble	The Big Gamble	Fleischer, Richard	Richard Fleischer	tt0054679
1746580332	1937	1937	Nothing Sacred	Nothing Sacred	Wellman, William A.	William A. Wellman	tt0029322
1746454502	1945	1945	Crime, Inc.	Crime, Inc.	Landers, Lew	Lew Landers	tt0037622
1746453631	1941	1941	The Phantom Cowboy	The Phantom Cowboy	Sherman, George	George Sherman	tt0034020
1746496092	1917	1917	The Eyes of the World	The Eyes of the World	Crisp, Donald	Donald Crisp	tt0007909
1746504008	1921	1921	Room and Board	Room and Board	Crosland, Alan	Alan Crosland	tt0012635
1745358554	2009	2009	The Ugly Truth	The Ugly Truth	Luketic, Robert	Robert Luketic	tt1142988
1745293645	2006	2006	World Trade Center	World Trade Center	Stone, Oliver	Oliver Stone	tt0469641
1746491784	1948	1948	Half Past Midnight	Half Past Midnight	Claxton, William F.	William F. Claxton	tt0040414
1746424558	1941	1941	Roar of the Press	Roar of the Press	Rosen, Phil	Phil Rosen	tt0034117
1746524804	1923	1923	Danger Ahead	Danger Ahead	Howard, William K.	William K. Howard	tt0013968
1746438964	1943	1943	It Comes Up Love	It Comes Up Love	Lamont, Charles	Charles Lamont	tt0036048
1746497063	1920	1920	The Money-Changers	The Money Changers	Conway, Jack	Jack Conway	tt0011475
1746436269	1997	1997	Sick: The Life and Death of Bob Flanagan Superm	Sick: The Life & Death of Bob Flanagan, Supe	Dick, Kirby	Kirby Dick	tt0120126
1745341633	2002	2002	Windtalkers	Windtalkers	Woo, John	John Woo	tt0245562
1769971975	1969	1969	Erika/One	Erika/One	Denby, Jerry	Jerry Denby	tt0217441
1746499944	1923	1923	A Gentleman of Leisure	A Gentleman of Leisure	Henabery, Joseph	Joseph Henabery	tt0131407
1746554280	1965	1965	Mutiny in Outer Space	Mutiny in Outer Space	Grimaldi, Hugo	Hugo Grimaldi	tt0059486
1745359637	2001	2000	Citizen Toxie: The Toxic Avenger IV	Citizen Toxie: The Toxic Avenger IV	Kaufman, Lloyd	Lloyd Kaufman	tt0212879
1745372325	2004	2004	Fade to Black	Fade to Black	Paulson, Patrick ; Warren, Michael J	Patrick Paulson	tt0428518
1745340292	2001	2001	Planet of the Apes	Planet of the Apes	Burton, Tim	Tim Burton	tt0133152
1746555348	1954	1954	The Command	The Command	Butler, David	David Butler	tt0046865
1746524281	1910	1910	Renunciation	Renunciation		Wilbert Melville	tt0339571

1746514329	1923	1923	The Enemies of Women	Enemies of Women	Crosland, Alan	Alan Crosland	tt0014021
1746502061	1923	1923	The Meanest Man in the World	The Meanest Man in the World	Cline, Edward F.	Edward F. Cline	tt0014266
1746582243	1930	1930	Hot Curves	Hot Curves	Taurog, Norman	Norman Taurog	tt0020988
1745359937	2001	2001	Freddy Got Fingered	Freddy Got Fingered	Green, Tom	Tom Green	tt0240515
1746249452	1980	1980	The Blues Brothers	The Blues Brothers	Landis, John	John Landis	tt0080455
1746475736	1947	1947	Little Miss Broadway	Little Miss Broadway	Dreifuss, Arthur	Arthur Dreifuss	tt0039571
1746474122	1948	1948	Apartment for Peggy	Apartment for Peggy	Seaton, George	George Seaton	tt0040104
1746504169	1917	1917	The Voice of Conscience	The Voice of Conscience	Carewe, Edwin	Edwin Carewe	tt0008741
1746577699	1935	1935	Fighting Pilot	The Fighting Pilot	Mason, Noel	Noel M. Smith	tt0026353
1746496983	1919	1919	A Man's Country	A Man's Country	Kolker, Henry	Henry Kolker	tt0010429
1746524704	1916	1916	The Daring of Diana	The Daring of Diana	Drew, S. Rankin	S. Rankin Drew	tt0006566
1746583406	1951	1951	Grounds for Marriage	Grounds for Marriage	Leonard, Robert Z.	Robert Z. Leonard	tt0042526
1746409506	1987	1987	Death Wish 4: The Crackdown	Death Wish 4: The Crackdown	Thompson, J. Lee	J. Lee Thompson	tt0092857
1746464803	1941	1941	Riders of the Purple Sage	Riders of the Purple Sage	Tinling, James	James Tinling	tt0034104
1746525019	1920	1920	The Sins of St. Anthony	The Sins of St. Anthony	Cruze, James	James Cruze	tt0011697
1746582099	1910	1910	In Old California	In Old California	Griffith, D. W.	D.W. Griffith	tt0001264
1745341799	2004	2004	Blade: Trinity	Blade: Trinity	Goyer, David S.	David S. Goyer	tt0359013
1769976186	1920	1920	The Cameraman	The Cameraman	Jones, Grover	Grover Jones	tt0313851
1746573915	1963	1962	Sodom and Gomorrah	Sodom and Gomorrah	Aldrich, Robert; Leone, Sergio	Robert Aldrich	tt0056504
1746581543	1910	1910	Her Father's Pride	Her Father's Pride	Griffith, D. W.	D.W. Griffith	tt0001243
1746272148	1968	1968	The Private Navy of Sgt. O'Farrell	The Private Navy of Sgt. O'Farrell	Tashlin, Frank	Frank Tashlin	tt0063459
1746422232	1996	1996	Happy Gilmore	Happy Gilmore	Dugan, Dennis	Dennis Dugan	tt0116483
1746524937	1923	1923	Desert Rider	Desert Rider	Bradbury, Robert North	Robert N. Bradbury	tt0013984
1746453851	1941	1941	That Uncertain Feeling	That Uncertain Feeling	Lubitsch, Ernst	Ernst Lubitsch	tt0034274
1746496115	1920	1920	The Red Lane	The Red Lane	Reynolds, Lynn F.	Lynn Reynolds	tt0011616
1746504591	1921	1921	Diamonds Adrift	Diamonds Adrift	Bennett, Chester	Chester Bennett	tt0322011
1746524936	1915	1915	The Long Chance	The Long Chance	Le Saint, Edward J.	Edward LeSaint	tt0005648
1746423327	1999	1998	Frogs for Snakes	Frogs for Snakes	Poe, Amos	Amos Poe	tt0120680
1746583719	1929	1929	Slim Fingers	Slim Fingers	Levigard, Josef	Joseph Levigard	tt0020418
1746574297	1958	1958	She Gods of Shark Reef	She Gods of Shark Reef	Corman, Roger	Roger Corman	tt0052188
1746436537	1942	1942	Professor Creeps	Professor Creeps	Beaudine, William	William Beaudine	tt0035221
1746199984	1969	1968	In the Year of the Pig	In the Year of the Pig	de Antonio, Emile	Emile de Antonio	tt0064482
1746496236	1920	1920	Life	Life	Vale, Travers	Travers Vale	tt0195880
1746503611	1921	1921	Kazan	Kazan	Bracken, Bertram	Bertram Bracken	tt0012343
1746562387	1953	1953	Destination Gobi	Destination Gobi	Wise, Robert	Robert Wise	tt0045682
1746577895	1935	1935	Hands Across the Table	Hands Across the Table	Leisen, Mitchell	Mitchell Leisen	tt0026452
1746570342	1934	1934	The Mighty Barnum	The Mighty Barnum	Lang, Walter	Walter Lang	tt0025501
1746434606	2000	2000	George Washington	George Washington	Green, David Gordon	David Gordon Green	tt0262432
1745309448	2006	2005	The Outsider	The Outsider	Jarecki, Nicholas	Nicholas Jarecki	tt0455989
1746520133	1923	1923	Gossip	Gossip	Baggot, King	King Baggot	tt0014095
1746422045	1982	1982	The Soldier	The Soldier	Glickenhaus, James	James Glickenhaus	tt0084704
1746501294	1916	1916	Peck O' Pickles	Peck o' Pickles	Heffron, T. N.	Thomas N. Heffron	tt0163148
1746516261	1922	1922	The Trap	The Trap	Thornby, Robert	Robert Thornby	tt0013704
1746513649	1923	1923	The White Rose	The White Rose	Griffith, D. W.	D.W. Griffith	tt0014604
1746437380	1942	1942	The Big Street	The Big Street	Reis, Irving	Irving Reis	tt0034514
1746571532	1932	1932	Uptown New York	Uptown New York	Schertzinger, Victor	Victor Schertzinger	tt0023646
1746515061	1919	1919	Why Smith Left Home	Why Smith Left Home	Crisp, Donald	Donald Crisp	tt0010889
1746422645	1993	1993	Striking Distance	Striking Distance	Herrington, Rowdy	Rowdy Herrington	tt0108238
1746583031	1939	1939	Missing Daughters	Missing Daughters	Coleman, C. C.	Charles C. Coleman	tt0031661
1746393671	1985	1985	Marie	Maxie	Donaldson, Roger	Paul Aaron	tt0089569
1746235533	1973	1973	Sweet Jesus, Preacherman	Sweet Jesus, Preacherman	Schellerup, Henning	Henning Schellerup	tt0167431
1746569682	1954	1954	World for Ransom	World for Ransom	Aldrich, Robert	Robert Aldrich	tt0047681
1746466721	1917	1917	The Phantom's Secret	The Phantom's Secret	Swickard, Charles	Charles Swickard	tt0008429
1746584462	1931	1931	The Cuban Love Song	The Cuban Love Song	Van Dyke, W. S.	W.S. Van Dyke	tt0021772
1746577153	1960	1961	Kipling's Women	Kipling's Women	Hudson, Fred; Smith, Harry	Fred Hudson	tt0142453
1746464951	1945	1945	A Sporting Chance	A Sporting Chance	Blair, George	George Blair	tt0038112
1746584672	1928	1928	A Bit of Heaven	A Bit of Heaven	Wheeler, Cliff	Cliff Wheeler	tt0018701

1746555566	1954	1954	Seven Brides for Seven Brothers	Seven Brides for Seven Brothers	Donen, Stanley	Stanley Donen	tt0047472
1746577227	1963	1963	13 Frightened Girls	13 Frightened Girls	Castle, William	William Castle	tt0056796
1746576861	1932	1932	Boiling Point	The Boiling Point	Melford, George	George Melford	tt0022702
1746508762	1925	1925	His Master's Voice	His Master's Voice	Hoffman, Renaud	Renaud Hoffman	tt0015917
1746582142	1928	1928	A Woman's Way	A Woman's Way	Mortimer, Edmund	Edmund Mortimer	tt0019592
1746419954	1993	1993	Coneheads	Coneheads	Barron, Steve	Steve Barron	tt0106598
1745359894	2009	2009	My One and Only	My One and Only	Loncraine, Richard	Richard Loncraine	tt1185431
1746423989	1941	1941	Mutiny in the Arctic	Mutiny in the Arctic	Rawlins, John	John Rawlins	tt0033930
1746501670	1910	1910	A True Indian Brave	A True Indian Brave		Fred J. Balshofer	tt1371151
1746217996	1967	1967	Round Trip	Round Trip	Gaisseau, Pierre Dominique	Pierre	tt0257060
1746515560	1922	1922	Thorobred	Thorobred	Halligan, George	George Halligan	tt0170675
1746487594	1917	1917	The Cold Deck	The Cold Deck	Hart, William S.	William S. Hart	tt0007811
1746184683	1910	1910	As the Bells Rang Out!	As the Bells Rang Out!	Griffith, D. W.	D.W. Griffith	tt0001121
1746435220	1942	1942	Careful Soft Shoulder	Careful, Soft Shoulders	Garrett, Oliver H. P.	Oliver H.P. Garrett	tt0034579
1746520923	1949	1949	Law of the Golden West	Law of the Golden West	Ford, Philip	Philip Ford	tt0041583
1746573883	1962	1962	Geronimo	Geronimo	Laven, Arnold	Arnold Laven	tt0056016
1746575028	1933	1933	Trail Drive	The Trail Drive	James, Alan	Alan James	tt0025904
1746524601	1916	1916	The Closed Road	The Closed Road	Tourneur, Maurice	Maurice Tourneur	tt0006519
1746581292	1910	1910	The Legend of Scar-Face	The Legend of Scar Face		Kenean Buel	tt2012074
1746185864	1966	1966	Dimension 5	Dimension 5	Adreon, Franklin	Franklin Adreon	tt0060311
1746233031	1967	1967	Hillbillys in a Haunted House	Hillbillys in a Haunted House	Yarbrough, Jean	Jean Yarbrough	tt0061765
1746576950	1961	1961	Pirates of Tortuga	Pirates of Tortuga	Webb, Robert D.	Robert D. Webb	tt0055302
1746570383	1934	1934	Ever Since Eve	Ever Since Eve	Marshall, George; St. Clair, Malcolm	George Marshall	tt0025093
1746545174	1940	1940	Bowery Boy	Bowery Boy	Morgan, William	William Morgan	tt0033429
1746582863	1937	1937	Between Two Women	Between Two Women	Seitz, George B.	George B. Seitz	tt0028623
1746503504	1919	1919	Shadows of Suspicion	Shadows of Suspicion	Carewe, Edwin	Edwin Carewe	tt0010687
1746454184	2000	2000	Down from the Mountain	Down from the Mountain	Doob, Nick; Hegedus, Chris; Penneba	Nick Doob	tt0284067
1746574511	1933	1933	Midshipman Jack	Midshipman Jack	Cabanne, Christy	Christy Cabanne	tt0024336
1746578541	1962	1962	Sweet Bird of Youth	Sweet Bird of Youth	Brooks, Richard	Richard Brooks	tt0056541
1746583644	1931	1931	Tarnished Lady	Tarnished Lady	Cukor, George	George Cukor	tt0022463
1746585323	1928	1928	The Butter and Egg Man	The Butter and Egg Man	Wallace, Richard	Richard Wallace	tt0018736
1746509047	1925	1925	The Half-Way Girl	The Half Way Girl	Dillon, John Francis	John Francis Dillon	tt0015889
1746249033	1980	1980	Inside Moves	Inside Moves	Donner, Richard	Richard Donner	tt0080928
1746422238	1999	1999	Baby Geniuses	Baby Geniuses	Clark, Bob	Bob Clark	tt0118665
1746574787	1935	1936	Wildcat Saunders	Wildcat Saunders	Fraser, Harry	Harry L. Fraser	tt0028507
1746574048	1956	1956	Three for Jamie Dawn	Three for Jamie Dawn	Carr, Thomas	Thomas Carr	tt0049850
1746503037	1917	1917	The Slacker's Heart	The Slacker's Heart	Ireland, Frederick J.	Frederick J. Ireland	tt0008589
1746577942	1935	1935	Roaring Roads	Roaring Roads	Roberts, C. Edward; Nazarro, Raymor	Ray Nazarro	tt0136492
1746208287	1969	1969	Sam Whiskey	Sam Whiskey	Laven, Arnold	Arnold Laven	tt0064923
1746577916	1938	1938	This Marriage Business	This Marriage Business	Cabanne, Christy	Christy Cabanne	tt0030862
1746456084	1996	1996	2 Days in the Valley	2 Days in the Valley	Herzfeld, John	John Herzfeld	tt0115438
1746562164	1954	1954	The Immortal City	The Immortal City	Cappi, Jerome	Jerome Cappi	tt0459406
1746566064	1953	1953	Column South	Column South	Cordova, Frederick de	Frederick De Cordova	tt0045637
1745293903	2006	2006	Shut Up & Sing	Shut Up & Sing	Kopple, Barbara; Peck, Cecilia	Barbara Kopple	tt0811136
1746423459	1996	1996	Bogus	Bogus	Jewison, Norman	Norman Jewison	tt0115725
1745302666	2006	2005	Lassie	Lassie	Sturridge, Charles	Charles Sturridge	tt0431213
1746454266	1998	1998	Knock Off	Knock Off	Hark, Tsui	Hark Tsui	tt0120724
1746394547	1985	1985	D.A.R.Y.L.	D.A.R.Y.L.	Wincer, Simon	Simon Wincer	tt0088979
1746435900	1998	1998	Rounders	Rounders	Dahl, John	John Dahl	tt0128442
1745359506	2003	2003	Dysfunktional Family	DysFunktional Family	Gallo, George	George Gallo	tt0337996
1746503688	1918	1918	And the Children Pay	And the Children Pay	Tyrol, Jacques	Jacques Tyrol	tt0181293
1745342677	2010	2009	Best Worst Movie	Best Worst Movie	Stephenson, Michael Paul	Michael Paul Stephenson	tt1144539
1746503057	1914	1914	The County Chairman	The County Chairman		Allan Dwan	tt0003799
1746585163	1929	1929	Sunnyside Up	Sunnyside Up	Butler, David	David Butler	tt0020466
1746496545	1919	1919	Oh, You Women	Oh, You Women!	Emerson, John	John Emerson	tt0010518
1746582609	1937	1937	Dangerous Number	Dangerous Number	Thorpe, Richard	Richard Thorpe	tt0028764
1746578877	1962	1962	Follow That Dream	Follow That Dream	Douglas, Gordon	Gordon Douglas	tt0055992

1746520650	1926	1926	Danger Ahead	Danger Ahead	Pembroke, Perc	Scott Pembroke	tt0251037
1746574607	1933	1933	Midnight Club	Midnight Club	Hall, Alexander; Somnes, George	Alexander Hall	tt0024333
1746208508	1969	1969	The Illustrated Man	The Illustrated Man	Smight, Jack	Jack Smight	tt0064473
1769970849	1915	1915	No Smoking	No Smoking	Metcalfe, Earl		tt0844735
1746533042	1914	1914	Soldiers of Fortune	Soldiers of Fortune	Haddock, William F.	William F. Haddock	tt0004613
1746182446	1910	1910	The Broken Oath	The Broken Oath		Harry Solter	tt0200505
1746578681	1959	1959	Career	Career	Anthony, Joseph	Joseph Anthony	tt0052673
1746542696	1926	1926	Queen o' Diamonds	Queen o'Diamonds	Withey, Chet	Chester Withey	tt0017301
1746514641	1915	1915	The Carpet from Bagdad	The Carpet from Bagdad	Campbell, Colin	Colin Campbell	tt0005062
1746578804	1951	1951	Go for Broke!	Go for Broke!	Pirosh, Robert	Robert Pirosh	tt0043590
1746248844	1980	1980	Roadie	Roadie	Rudolph, Alan	Alan Rudolph	tt0081433
1746257252	1979	1979	Norma Rae	Norma Rae	Ritt, Martin	Martin Ritt	tt0079638
1746437973	1940	1940	South to Karanga	South to Karanga	Schuster, Harold	Harold D. Schuster	tt0033085
1746532726	1914	1914	The Little Gray Lady	The Little Gray Lady	Powers, Francis	Francis Powers	tt0004236
1746585098	1930	1930	Prince of Diamonds	Prince of Diamonds	Brown, Karl; Van Buren, A. H.	Karl Brown	tt0021271
1745341572	2009	2009	The Soloist	The Soloist	Wright, Joe	Joe Wright	tt0821642
1746583135	1936	1936	Oh, Susanna!	Oh, Susanna!	Kane, Joseph	Joseph Kane	tt0029334
1746584996	1939	1939	Charlie Chan at Treasure Island	Charlie Chan at Treasure Island	Foster, Norman	Norman Foster	tt0031147
1746508202	1926	1926	The Bells	The Bells	Young, James	James Young	tt0016640
1745340017	2002	2002	Blade II	Blade II	del Toro, Guillermo	Guillermo del Toro	tt0187738
1746500136	1925	1925	Lilies of the Streets	Lilies of the Streets	Levering, Joseph	Joseph Levering	tt0016024
1746483472	1917	1917	Over There	Over There	Kirkwood, James	James Kirkwood	tt0181747
1746453719	1995	1995	Empire Records	Empire Records	Moyle, Allan	Allan Moyle	tt0112950
1746498289	1922	1922	The Lane That Had No Turning	The Lane That Had No Turning	Fleming, Victor	Victor Fleming	tt0013303
1746408905	1989	1989	Indiana Jones and the Last Crusade	Indiana Jones and the Last Crusade	Spielberg, Steven	Steven Spielberg	tt0097576
1746567776	1933	1933	The Kennel Murder Case	The Kennel Murder Case	Curtiz, Michael	Michael Curtiz	tt0024210
1746491734	1946	1946	Fear	Fear	Zeisler, Alfred	Alfred Zeisler	tt0038524
1769970778	1912	1912	Just Like a Woman	Just Like a Woman		D.W. Griffith	tt0002287
1746583483	1929	1929	The Lady Lies	The Lady Lies	Henley, Hobart	Hobart Henley	tt0020072
1746511793	1916	1916	The Quest of Life	The Quest of Life	Miller, Ashley	Ashley Miller	tt0158877
1746578988	1962	1962	Eegah!	Eegah	Merriwether, Nicholas	Arch Hall Sr.	tt0055946
1746574042	1956	1956	Lust for Life	Lust for Life	Minnelli, Vincente	Vincente Minnelli	tt0049456
1746455744	1995	1995	Clockers	Clockers	Lee, Spike	Spike Lee	tt0112688
1746513041	1916	1916	Seventeen	Seventeen	Vignola, Robert G.	Robert G. Vignola	tt0007326
1746583464	1951	1951	When Worlds Collide	When Worlds Collide	Maté, Rudolph	Rudolph Maté	tt0044207
1746465124	1945	1945	Molly and Me	Molly and Me	Seiler, Lewis	Lewis Seiler	tt0037923
1746218885	1972	1972	Cool Breeze	Cool Breeze	Pollack, Barry	Barry Pollack	tt0068409
1746464961	1941	1941	Never Give a Sucker an Even Break	Never Give a Sucker an Even Break	Cline, Edward	Edward F. Cline	tt0033945
1746571783	1931	1931	Annabelle's Affairs	Annabelle's Affairs	Werker, Alfred	Alfred L. Werker	tt0021610
1746520310	1925	1925	That Devil Quemado	That Devil Quemado	Andrews, Del	Del Andrews	tt0169310
1746494240	1910	1910	Two Little Waifs	Two Little Waifs	Griffith, D. W.	D.W. Griffith	tt0001430
1746453847	1941	1941	Moon Over Miami	Moon Over Miami	Lang, Walter	Walter Lang	tt0033918
1746419570	1986	1986	Quiet Cool	Quiet Cool	Borris, Clay	Clay Borris	tt0091815
1746504188	1917	1917	A Bit O' Heaven	A Bit o' Heaven	Warrenton, Lule	Lule Warrenton	tt0168513
1746465039	1944	1944	The Old Texas Trail	The Old Texas Trail	Collins, Lewis D.	Lewis D. Collins	tt0037148
1746410001	1984	1984	Sixteen Candles	Sixteen Candles	Hughes, John	John Hughes	tt0088128
1746581642	1928	1929	The Saddle King	The Saddle King	Wilson, Benjamin Franklin	Ben F. Wilson	tt0159718
1746566346	1958	1958	Darby's Rangers	Darby's Rangers	Wellman, William A.	William A. Wellman	tt0051519
1746500082	1945	1945	Santa Fe Saddlemates	Santa Fe Saddlemates	Carr, Thomas	Thomas Carr	tt0038050
1746574852	1936	1936	Border Caballero	Border Caballero	Newfield, Sam	Sam Newfield	tt0027382
1745372702	2003	2003	Kangaroo Jack	Kangaroo Jack	McNally, David	David McNally	tt0257568
1746580732	1938	1938	The Colorado Trail	The Colorado Trail	Nelson, Sam	Sam Nelson	tt0030003
1746515302	1918	1918	Human Clay	Human Clay	Physioc, Wray	Wray Bartlett Physioc	tt0184594
1746577541	1938	1939	Birthright	Birthright	Micheaux, Oscar	Oscar Micheaux	tt0135163
1746438327	1945	1945	The Daltons Ride Again	The Daltons Ride Again	Taylor, Ray	Ray Taylor	tt0037628
1746235043	1971	1971	Women in Cages	Women in Cages	De Leon, Gerry	Gerardo de Leon	tt0067995
1746520956	1949	1948	Highway 13	Highway 13	Berke, William	William Berke	tt0040437

1746436051	1940	1940	Little Nellie Kelly	Little Nellie Kelly	Taurog, Norman	Norman Taurog	tt0032718
1746410663	1986	1986	Soul Man	Soul Man	Miner, Steve	Steve Miner	tt0091991
1746436702	1943	1943	The Heat's On	The Heat's On	Ratoff, Gregory	Gregory Ratoff	tt0035978
1746408654	1984	1984	Star Trek III: The Search for Spock	Star Trek III: The Search for Spock	Nimoy, Leonard	Leonard Nimoy	tt0088170
1746394160	1992	1992	Husbands and Wives	Husbands and Wives	Allen, Woody	Woody Allen	tt0104466
1746582678	1935	1935	Remember Last Night?	Remember Last Night?	Whale, James	James Whale	tt0026921
1746473686	1942	1942	Billy the Kid Trapped	Billy the Kid Trapped	Scott, Sherman	Sam Newfield	tt0033390
1746566330	1956	1956	1984	1984	Anderson, Michael	Michael Anderson	tt0048918
1746583538	1928	1928	Annapolis	Annapolis	Cabanne, Christy	Christy Cabanne	tt0018646
1745293656	2006	2006	Children of Men	Children of Men	Cuarón, Alfonso	Alfonso Cuarón	tt0206634
1746584090	1940	1940	Girls of the Road	Girls of the Road	Grinde, Nick	Nick Grinde	tt0032530
1746410867	1991	1991	Bugsy	Bugsy	Levinson, Barry	Barry Levinson	tt0101516
1746420793	1981	1981	Dead & Buried	Dead & Buried	Sherman, Gary A.	Gary Sherman	tt0082242
1746437923	1945	1945	Girls of the Big House	Girls of the Big House	Archainbaud, George	George Archainbaud	tt0037742
1746454637	1942	1942	Almost Married	Almost Married	Lamont, Charles	Charles Lamont	tt0034451
1746585128	1928	1928	Happiness Ahead	Happiness Ahead	Seiter, William A.	William A. Seiter	tt0018974
1769969985	1912	1912	The Female of the Species	The Female of the Species	Griffith, D. W.	D.W. Griffith	tt0002176
1746492203	1946	1946	Frontier Gun Law	Frontier Gunlaw	Abrahams, Derwin	Derwin Abrahams	tt0038538
1746582392	1928	1928	Gypsy of the North	Gypsy of the North	Pembroke, Scott	Scott Pembroke	tt0018969
1746508736	1926	1926	Beverly of Graustark	Beverly of Graustark	Franklin, Sidney	Sidney Franklin	tt0016646
1746580796	1939	1939	Lucky Night	Lucky Night	Taurog, Norman	Norman Taurog	tt0031595
1746530701	1948	1948	El Dorado Pass	El Dorado Pass	Nazarro, Ray	Ray Nazarro	tt0041331
1746553649	1964	1964	Dead Ringer	Dead Ringer	Henreid, Paul	Paul Henreid	tt0057997
1746513192	1923	1923	Cameo Kirby	Cameo Kirby	Ford, John	John Ford	tt0013909
1746514708	1910	1910	Red Girl's Romance	Red Girl's Romance		Fred J. Balshofer	tt1390977
1746573722	1932	1932	Two Seconds	Two Seconds	LeRoy, Mervyn	Mervyn LeRoy	tt0023629
1746503352	1921	1921	Get-Rich-Quick Wallingford	Get Rich Quick Wallingford	Borzage, Frank	Frank Borzage	tt0012209
1746584436	1929	1929	The Wild Party	The Wild Party	Arzner, Dorothy	Dorothy Arzner	tt0020590
1746502461	1914	1914	The Squaw Man	The Squaw Man	Apfel, Oscar C.; DeMille, Cecil B.	Oscar Apfel	tt0004635
1746566292	1958	1958	Cowboy	Cowboy	Daves, Delmer	Delmer Daves	tt0051496
1746574050	1960	1960	Sunrise at Campobello	Sunrise at Campobello	Donehue, Vincent J.	Vincent J. Donehue	tt0054354
1769976709	1929	1929	Hot Lemonade	Hot Lemonade	Gillstrom, Arvid E.	Arvid E. Gillstrom	tt0254390
1746487749	1918	1918	The Light of Western Stars	The Light of Western Stars	Swickard, Charles	Charles Swickard	tt0009301
1746550224	1954	1954	There's No Business Like Show Business	There's No Business Like Show Business	Lang, Walter	Walter Lang	tt0047574
1746454278	1942	1942	Obliging Young Lady	Obliging Young Lady	Wallace, Richard	Richard Wallace	tt0033968
1746568351	1952	1952	Barbed Wire	Barbed Wire	Archainbaud, George	George Archainbaud	tt0044396
1746532457	1927	1927	The Enchanted Island	The Enchanted Island	Crosby, William G.	William G. Crosby	tt0017849
1746487148	1920	1920	The Blue Pearl	The Blue Pearl	Irving, George	George Irving	tt0011006
1746569635	1955	1955	Broadway Jungle	Broadway Jungle	Tucker, Phil	Phil Tucker	tt0047900
1746584943	1939	1939	Pardon Our Nerve	Pardon Our Nerve	Humberstone, H. Bruce	H. Bruce Humberstone	tt0031785
1746465320	1947	1947	Living in a Big Way	Living in a Big Way	La Cava, Gregory	Gregory La Cava	tt0039574
1746464930	1947	1947	Second Chance	Second Chance	Tinling, James S.	James Tinling	tt0039807
1746569563	1953	1953	Shoot First	Shoot First	Parrish, Robert	Robert Parrish	tt0046307
1746520906	1950	1950	Abbott & Costello in the Foreign Legion	Abbott and Costello in the Foreign Legion	Lamont, Charles	Charles Lamont	tt0042179
1746566162	1954	1954	Riding Shotgun	Riding Shotgun	DeToth, Andre	André De Toth	tt0047413
1746524438	1923	1923	The Dangerous Maid	The Dangerous Maid	Heerman, Victor	Victor Heerman	tt0013971
1746574979	1938	1937	Clipped Wings	Clipped Wings	Paton, Stuart	Stuart Paton	tt0028725
1746585155	1928	1928	The Battle of the Sexes	The Battle of the Sexes		D.W. Griffith	tt0018678
1746492094	1945	1945	Sing Me a Song of Texas	Sing Me a Song of Texas	Keays, Vernon	Vernon Keays	tt0038090
1746260935	1980	1980	Fatso	Fatso	Bancroft, Anne	Anne Bancroft	tt0080724
1746520549	1926	1926	Two Can Play	Two Can Play	Ross, Nat	Nat Ross	tt0017494
1746454399	1941	1941	Virginia	Virginia	Griffith, Edward H.	Edward H. Griffith	tt0034364
1746553968	1964	1964	Bunny Yeager's Nude Las Vegas	Bunny Yeager's Nude Las Vegas	Mahon, Barry	Barry Mahon	tt0157429
1746198232	1969	1970	The Curious Female	The Curious Female	Rapp, Paul	Paul Rapp	tt0123770
1746409286	1987	1987	Roxanne	Roxanne	Schepisi, Fred	Fred Schepisi	tt0093886
1746454499	1941	1941	Manpower	Manpower	Walsh, Raoul	Raoul Walsh	tt0035034
1746583806	1929	1929	The Little Savage	The Little Savage	King, Louis	Louis King	tt0331557

1746454155	1995	1995	Gold Diggers: The Secret of Bear Mountain	Gold Diggers: The Secret of Bear Mountain	Dobson, Kevin James	Kevin James Dobson	tt0113188
1746509419	1924	1924	On Time	On Time	Lehrman, Henry	Henry Lehrman	tt0015191
1746438591	1996	1996	Striptease	Striptease	Bergman, Andrew	Andrew Bergman	tt0117765
1746500134	1946	1946	Bad Bascomb	Bad Bascomb	Simon, S. Sylvan	S. Sylvan Simon	tt0038319
1746508757	1924	1923	Black Oxen	Black Oxen	Lloyd, Frank	Frank Lloyd	tt0014721
1745357697	2010	2010	Sex and the City 2	Sex and the City 2	King, Michael Patrick	Michael Patrick King	tt1261945
1746455147	1994	1994	Safe Passage	Safe Passage	Ackerman, Robert Allan	Robert Allan Ackerman	tt0111054
1746560616	1932	1932	Forbidden Company	Forbidden Company	Thorpe, Richard	Richard Thorpe	tt0022906
1746516258	1919	1919	Here Comes the Bride	Here Comes the Bride	Robertson, John S.	John S. Robertson	tt0010244
1746464998	1944	1943	A Guy Named Joe	A Guy Named Joe	Fleming, Victor	Victor Fleming	tt0035959
1746422513	1998	1998	Mercury Rising	Mercury Rising	Becker, Harold	Harold Becker	tt0120749
1746584302	1935	1935	Death from a Distance	Death from a Distance	Strayer, Frank R.	Frank R. Strayer	tt0027504
1746183155	1910	1910	The Blind Man's Tact	The Blind Man's Tact		Harry Solter	tt0200495
1746496872	1917	1917	The Door Between	The Door Between	Julian, Rupert	Rupert Julian	tt0007868
1746571073	1935	1935	One Frightened Night	One Frightened Night	Cabanne, Christy	Christy Cabanne	tt0026819
1746569646	1954	1954	The Gambler from Natchez	The Gambler from Natchez	Levin, Henry	Henry Levin	tt0047011
1745357724	2001	2001	Not Another Teen Movie	Not Another Teen Movie	Gallen, Joel	Joel Gallen	tt0277371
1746409064	1990	1990	Heart Condition	Heart Condition	Parriott, James D.	James D. Parriott	tt0099750
1746438725	1998	1998	Billy's Hollywood Screen Kiss	Billy's Hollywood Screen Kiss	O'Haver, Tommy	Tommy O'Haver	tt0137386
1746248809	1979	1979	Skatetown U.S.A.	Skatetown, U.S.A.	Levey, William A.	William A. Levey	tt0079912
1746577920	1934	1934	The Crosby Case	The Crosby Case	Marin, Edward L.	Edwin L. Marin	tt0025014
1746516111	1921	1921	The Cave Girl	The Cave Girl	Franz, Joseph J.	Joseph Franz	tt0012034
1746216770	1967	1967	Don't Look Back	Dont Look Back	Pennebaker, D. A.	D.A. Pennebaker	tt0061589
1746565054	1952	1952	Wagon Team	Wagon Team	Archainbaud, George	George Archainbaud	tt0045305
1746578928	1951	1951	Pier 23	Pier 23	Berke, William	William Berke	tt0043920
1745303063	2005	2005	Waiting ...	Waiting...	McKittrick, Rob	Rob McKittrick	tt0348333
1746573647	1932	1932	Bachelor's Affairs	Bachelor's Affairs	Werker, Alfred	Alfred L. Werker	tt0022649
1746453930	1995	1995	The Perez Family	The Perez Family	Nair, Mira	Mira Nair	tt0114113
1746424382	1942	1942	The Mayor of 44th Street	The Mayor of 44th Street	Green, Alfred E.	Alfred E. Green	tt0035048
1746198180	1966	1966	Johnny Tiger	Johnny Tiger	Wendkos, Paul	Paul Wendkos	tt0060565
1746579021	1951	1951	Blazing Bullets	Blazing Bullets	Fox, Wallace W.	Wallace Fox	tt0043348
1746580414	1938	1938	Sweethearts	Sweethearts	Van Dyke, W. S.	W.S. Van Dyke	tt0030817
1746435419	1995	1995	Major Payne	Major Payne	Castle, Nick	Nick Castle	tt0110443
1746573863	1958	1958	The Screaming Skull	The Screaming Skull	Nicol, Alex	Alex Nicol	tt0052169
1746473922	1945	1945	Club Havana	Club Havana	Ulmer, Edgar G.	Edgar G. Ulmer	tt0037605
1746580816	1936	1936	Three Men on a Horse	Three Men on a Horse	LeRoy, Mervyn	Mervyn LeRoy	tt0028371
1746520609	1925	1925	Fighting Youth	Fighting Youth	Eason, Reeves	B. Reeves Eason	tt0015815
1745271798	2000	2000	Pitch Black	Pitch Black	Twohy, David	David Twohy	tt0134847
1746573208	1931	1931	Up Pops the Devil	Up Pops the Devil	Sutherland, A. Edward	A. Edward Sutherland	tt0022525
1746578693	1962	1962	The Legend of Lobo	The Legend of Lobo	Algar, James		tt0056173
1746233280	1966	1966	The Trouble With Angels	The Trouble with Angels	Lupino, Ida	Ida Lupino	tt0061122
1746423576	1995	1995	Sense and Sensibility	Sense and Sensibility	Lee, Ang	Ang Lee	tt0114388
1746504140	1921	1921	Bits of Life	Bits of Life	Neilan, Marshall	James Flood	tt0011972
1746567819	1952	1952	The Thief	The Thief	Rouse, Russell	Russell Rouse	tt0045230
1746509238	1925	1925	Rose of the World	Rose of the World	Beaumont, Harry	Harry Beaumont	tt0016299
1746269693	1980	1980	Little Darlings	Little Darlings	Maxwell, Ronald F.	Ron Maxwell	tt0081060
1746584023	1936	1936	Timothy's Quest	Timothy's Quest	Barton, Charles	Charles Barton	tt0028384
1746520209	1926	1926	The Dixie Flyer	The Dixie Flyer	Hunt, Charles J.	Charles J. Hunt	tt0126265
1746514325	1923	1923	Chastity	Chastity	Schertzinger, Victor	Victor Schertzinger	tt0013919
1746569924	1959	1959	Happy Anniversary	Happy Anniversary	Miller, David	David Miller	tt0052878
1746520308	1925	1925	Straight Through	Straight Through	Rosson, Arthur	Arthur Rosson	tt0016402
1746500449	1926	1925	The Train Wreckers	The Train Wreckers	McGowan, J. P.	J.P. McGowan	tt0016450
1746580669	1938	1938	Knight of the Plains	Knight of the Plains	Newfield, Sam	Sam Newfield	tt0030329
1746453620	1943	1943	Gals, Incorporated	Gals, Incorporated	Goodwins, Leslie	Leslie Goodwins	tt0035915
1769970620	1968	1968	The Sex Shuffle	The Sex Shuffle	Scott, Ron	Ron Scott	tt0257149
1746574590	1937	1937	Expensive Husbands	Expensive Husbands	Connolly, Bobby	Bobby Connolly	tt0030111
1746211467	1973	1973	Breezy	Breezy	Eastwood, Clint	Clint Eastwood	tt0069822

1745309510	2007	2006	Air Guitar Nation	Air Guitar Nation	Lipsitz, Alexandra	Alexandra Lipsitz	tt0799915
1746532849	1928	1928	Land of the Silver Fox	Land of the Silver Fox	Enright, Ray	Ray Enright	tt0019070
1746585333	1928	1929	Headin' Westward	Headin' Westward	McGowan, J. P.	J.P. McGowan	tt0019970
1746566077	1953	1953	Tropic Zone	Tropic Zone	Foster, Lewis R.	Lewis R. Foster	tt0046462
1745293564	2006	2006	Madea's Family Reunion	Madea's Family Reunion	Perry, Tyler	Tyler Perry	tt0455612
1746248478	1968	1968	Beware The Black Widow	Beware the Black Widow	Crane, Larry	Larry Crane	tt0211247
1746566256	1954	1954	Three Young Texans	Three Young Texans	Levin, Henry	Henry Levin	tt0047583
1746520254	1925	1925	His People	His People	Sloman, Edward	Edward Sloman	tt0015918
1746496034	1920	1920	The Thirtieth Piece of Silver	The Thirtieth Piece of Silver	Cox, George L.	George L. Cox	tt0202624
1745341868	2002	2002	My Big Fat Greek Wedding	My Big Fat Greek Wedding	Zwick, Joel	Joel Zwick	tt0259446
1746581618	1910	1910	His Last Burglary	His Last Burglary	Griffith, D. W.	D.W. Griffith	tt0001248
1746533053	1923	1923	Pure Grit	Pure Grit	Ross, Nat	Nat Ross	tt0014381
1746454100	1942	1942	Secrets of a Co-ed	Secrets of a Co Ed	Lewis, Joseph H.	Joseph H. Lewis	tt0165938
1745357968	2009	2009	The Cove	Come	Psihoyos, Louie	Kirk Chastain	tt1710955
1746453694	1995	1994	Rave Review	Rave Review	Seymour, Jeff	Jeff Seymour	tt0110946
1745341851	2009	2008	Ice People	Ice People	Aghion, Anne	Anne Aghion	tt1234433
1746465312	1944	1944	Crazy Knights	Crazy Knights	Beaudine, William	William Beaudine	tt0036728
1746570824	1934	1934	There's Always Tomorrow	There's Always Tomorrow	Sloman, Edward	Edward Sloman	tt0025876
1746498481	1919	1919	The World Aflame	The World Aflame	Warde, Ernest C.	Ernest C. Warde	tt0010919
1746585463	1940	1940	Passport to Alcatraz	Passport to Alcatraz	Collins, Lewis D.	Lewis D. Collins	tt0032893
1746524311	1916	1916	The Supreme Temptation	The Supreme Temptation	Davenport, Harry	Harry Davenport	tt0007412
1746508148	1925	1925	The Blood Bond	The Blood Bond	Maloney, Leo	Leo D. Maloney	tt0015628
1746580209	1937	1937	Blonde Trouble	Blonde Trouble	Archainbaud, George	George Archainbaud	tt0028641
1746520716	1925	1925	Santa Fe Pete	Santa Fe Pete	Webb, Harry S.	Harry S. Webb	tt0161958
1746571968	1931	1931	Men of the Sky	Men of the Sky	Green, Alfred E.	Alfred E. Green	tt0022138
1746578902	1962	1962	The Horizontal Lieutenant	The Horizontal Lieutenant	Thorpe, Richard	Richard Thorpe	tt0056079
1746579063	1961	1961	The Big Show	The Big Show	Clark, James B.	James B. Clark	tt0054681
1746466941	1917	1917	The Public Be Damned	Public Be Damned	Taylor, S. E. V.	Stanner E.V. Taylor	tt0008471
1746508084	1925	1925	Graustark	Graustark	Buchowetzki, Dimitri	Dimitri Buchowetzki	tt0015874
1746394171	1987	1987	Moonstruck	Moonstruck	Jewison, Norman	Norman Jewison	tt0093565
1746563007	1952	1951	On Dangerous Ground	On Dangerous Ground	Ray, Nicholas	Nicholas Ray	tt0043879
1746454723	1999	1999	The Wood	The Wood	Famuyiwa, Rick	Rick Famuyiwa	tt0161100
1746524773	1915	1915	Bella Donna	Bella Donna	Porter, Edwin S.; Ford, Hugh	Hugh Ford	tt0004956
1746210820	1973	1973	Shaft in Africa	Shaft in Africa	Guillermin, John	John Guillermin	tt0070679
1746520825	1949	1949	The Red Danube	The Red Danube	Sidney, George	George Sidney	tt0041788
1745341539	2010	2010	The Extra Man	The Extra Man	Berman, Shari Springer; Pulcini, Rob	Shari Springer Berman	tt1361313
1746584525	1929	1929	The Sophomore	The Sophomore	McCarey, Leo	Leo McCarey	tt0020436
1746582516	1939	1939	Girl from Rio	Girl from Rio	Hillyer, Lambert	Lambert Hillyer	tt0031371
1746436214	1990	1990	Night of the Living Dead	Night of the Living Dead	Savini, Tom	Tom Savini	tt0100258
1746454015	1943	1943	Calling Dr. Death	Calling Dr. Death	LeBorg, Reginald	Reginald Le Borg	tt0035706
1746436264	1992	1992	Poison Ivy	Poison Ivy	Ruben, Katt Shea	Katt Shea	tt0105156
1746560971	1932	1932	Sally of the Subway	Sally of the Subway	Seitz, George B.	George B. Seitz	tt0023421
1746514077	1916	1916	Casey at the Bat	Casey at the Bat	Ingraham, Lloyd	Lloyd Ingraham	tt0006491
1745359146	2001	2001	Sugar & Spice	Sugar & Spice	McDougall, Francine	Francine McDougall	tt0186589
1746513698	1928	1928	The Last Moment	The Last Moment	Fejos, Paul	Pál Fejös	tt0019072
1746269652	1978	1978	Piranha	Piranha	Dante, Joe	Joe Dante	tt0078087
1746581316	1910	1910	The Hobble Skirt	The Hobble Skirt			tt1195745
1746500328	1926	1927	The Night Owl	Night Owls	Brown, Harry J.	Mark Sandrich	tt0349888
1746573586	1932	1932	Blessed Event	Blessed Event	Del Ruth, Roy	Roy Del Ruth	tt0022696
1746250733	1978	1978	Big Wednesday	Big Wednesday	Milius, John	John Milius	tt0077235
1746494195	1913	1913	In the Stretch	In the Stretch	Davis, Will S.	Will S. Davis	tt0003008
1746580212	1938	1938	Listen, Darling	Listen, Darling	Marin, Edwin L.	Edwin L. Marin	tt0030368
1746487051	1918	1918	Social Hypocrites	Social Hypocrites	Capellani, Albert	Albert Capellani	tt0009627
1746500683	1924	1924	Coyote Fangs	Coyote Fangs	Webb, Harry	Harry S. Webb	tt0014805
1746500433	1926	1926	The Great Deception	The Great Deception	Higgin, Howard	Howard Higgin	tt0016937
1746514747	1917	1917	The Adventurer	The Adventurer	Blaché, Alice	Alice Guy	tt0007614
1746582262	1928	1928	Stocks and Blondes	Stocks and Blondes	Murphy, Dudley	Dudley Murphy	tt0019423

1746581488	1960	1960	The Touch of Flesh	The Touch of Flesh	Hugh, R. John	R. John Hugh	tt0054397
1746272240	1979	1979	The Dark	The Dark	Cardos, John "Bud"	John 'Bud' Cardos	tt0079027
1746581977	1928	1928	Souls Aflame	Souls Aflame	Wells, Raymond	Raymond Wells	tt0165951
1746419798	1994	1994	Leprechaun 2	Leprechaun 2	Flender, Rodman	Rodman Flender	tt0110329
1746491865	1948	1948	A Date with Judy	A Date with Judy	Thorpe, Richard	Richard Thorpe	tt0040271
1746219196	1972	1972	The Final Comedown	The Final Comedown	Williams, Oscar	Oscar Williams	tt0068588
1746581764	1910	1910	The Golden Secret	The Golden Secret		William F. Haddock	tt2239166
1746475975	1948	1948	13 Lead Soldiers	13 Lead Soldiers	McDonald, Frank	Frank McDonald	tt0040063
1746576616	1932	1932	Cabin in the Cotton	The Cabin in the Cotton	Curtiz, Michael	Michael Curtiz	tt0022735
1746486501	1921	1922	The Jolt	The Jilt	Marshall, George E.	Irving Cummings	tt0013277
1746234153	1966	1966	Teenage Gang Debs	Teenage Gang Debs	Johnsen, Sande	Sande N. Johnsen	tt0061070
1746583985	1938	1938	Prison Farm	Prison Farm	King, Louis	Louis King	tt0030626
1746581729	1950	1950	Six Gun Mesa	Six Gun Mesa	Fox, Wallace W.	Wallace Fox	tt0271190
1746493763	1914	1914	Fire and Sword	Fire and Sword	Hunter, T. Hayes	T. Hayes Hunter	tt0003965
1746475728	1946	1946	Don't Gamble with Strangers	Don't Gamble with Strangers	Beaudine, William	William Beaudine	tt0038485
1746566348	1954	1954	The Boy from Oklahoma	The Boy from Oklahoma	Curtiz, Michael	Michael Curtiz	tt0046804
1746502820	1914	1914	The House Next Door	The House Next Door	O'Neil, Barry	Barry O'Neil	tt0004125
1769971898	1969	1969	How Many Times	How Many Times	Shiffen, Arlo	Don Walters	tt0141248
1746554541	1964	1964	A Shot in the Dark	A Shot in the Dark	Edwards, Blake	Blake Edwards	tt0058586
1746475961	1944	1944	Guns of the Law	Guns of the Law	Clifton, Elmer	Elmer Clifton	tt0036888
1746492231	1944	1944	The Invisible Man's Revenge	The Invisible Man's Revenge	Beebe, Ford	Ford Beebe	tt0036959
1746567970	1933	1933	Footlight Parade	Footlight Parade	Bacon, Lloyd	Lloyd Bacon	tt0024028
1769974371	1916	1916	A Daughter of the Woods	A Daughter of the Woods			tt3333326
1746584955	1939	1939	Flaming Lead	Flaming Lead	Newfield, Sam	Sam Newfield	tt0031318
1746577225	1950	1950	Surrender	Surrender	Dwan, Allan	Allan Dwan	tt0043017
1746492726	1945	1945	The True Glory	The True Glory	Reed, Carol; Kanin, Garson	Garson Kanin	tt0038192
1746486798	1919	1919	The Rough Neck	The Rough Neck	Apfel, Oscar	Oscar Apfel	tt0010644
1746455355	1995	1995	Live Nude Girls	Live Nude Girls	Lavin, Julianna	Julianna Lavin	tt0113674
1746565922	1953	1953	Julius Caesar	Julius Caesar	Mankiewicz, Joseph L.	Joseph L. Mankiewicz	tt0045943
1746530764	1949	1949	Deputy Marshal	Deputy Marshal	Berke, William	William Berke	tt0041289
1746560034	1963	1963	The Three Stooges Go Around the World in a Daz	The Three Stooges Go Around the World in a	Maurer, Norman	Norman Maurer	tt0057580
1746584322	1939	1939	The Mysterious Miss X	The Mysterious Miss X	Meins, Gus	Gus Meins	tt0031701
1745341542	2002	2002	Sweet Home Alabama	Sweet Home Alabama	Tennant, Andy	Andy Tennant	tt0256415
1769971205	1969	1969	The Ultimate Voyeur	The Ultimate Voyeur		Cosmo Vance	tt0153409
1746420118	1993	1993	Geronimo: An American Legend	Geronimo: An American Legend	Hill, Walter	Walter Hill	tt0107004
1746502216	1916	1916	The Shadow of a Doubt	The Shadow of a Doubt		Wray Bartlett Physioc	tt0158946
1746475514	1946	1946	Fight That Ghost	Fight That Ghost	Newfield, Sam	Sam Newfield	tt0135420
1746524444	1916	1916	The Market of Vain Desire	The Market of Vain Desire	Barker, Reginald	Reginald Barker	tt0007040
1746394745	1987	1987	American Ninja 2: The Confrontation	American Ninja 2: The Confrontation	Firstenberg, Sam	Sam Firstenberg	tt0092548
1746197904	1966	1966	The "Imp"probable Mr. Wee Gee	The 'Imp'probable Mr. Wee Gee	Price, Sherman	Sherman Price	tt0220948
1746491999	1948	1948	The Tender Years	The Tender Years	Schuster, Harold	Harold D. Schuster	tt0039890
1746584551	1931	1931	Dragnet Patrol	Dragnet Patrol	Strayer, Frank	Frank R. Strayer	tt0022836
1746571815	1910	1910	Cyclone Pete's Matrimony	Cyclone Pete's Matrimony		William F. Haddock	tt0001174
1746453643	1943	1943	Bordertown Gun Fighters	Bordertown Gun Fighters	Bretherton, Howard	Howard Bretherton	tt0035693
1746583000	1936	1936	Step on It	Step on It	Samuels, Henrí	Harry S. Webb	tt0140570
1746584058	1940	1940	Tin Pan Alley	Tin Pan Alley	Lang, Walter	Walter Lang	tt0033167
1746512732	1915	1915	The Reform Candidate	The Reform Candidate	Lloyd, Frank	Frank Lloyd	tt0005959
1746392776	1983	1983	Scarface	Scarface	DePalma, Brian	Brian De Palma	tt0086250
1746585042	1939	1939	Ex-Champ	Ex Champ	Rosen, Philip	Phil Rosen	tt0031287
1746454604	1999	1998	Naturally Native	Naturally Native	Red-Horse, Valerie ; Farmer, Jennifer	Jennifer Wynne Farmer	tt0133117
1746502588	1917	1917	The Dancer's Peril	The Dancer's Peril	Vale, Travers	Travers Vale	tt0007834
1746581578	1910	1910	The Impalement	The Impalement	Griffith, D. W.	D.W. Griffith	tt0001260
1746580494	1938	1938	A Trip to Paris	A Trip to Paris	St. Clair, Mal	Malcolm St. Clair	tt0030894
1746467711	1917	1917	Society's Driftwood	Society's Driftwood	Chaudet, Louis William	Louis Chaudet	tt0008597
1746580592	1936	1936	Riffraff	Riffraff	Ruben, J. Walter	J. Walter Ruben	tt0026932
1746524076	1927	1927	Alias the Deacon	Alias the Deacon	Sloman, Edward	Edward Sloman	tt0018639
1746520811	1926	1926	The Last Chance	The Last Chance	Carpenter, H. B.	Horace B. Carpenter	tt0160445

1746570442	1934	1934	Return of the Terror	Return of the Terror	Bretherton, Howard	Howard Bretherton	tt0025714
1746574933	1936	1936	Marihuana	Marihuana	Esper, Dwain	Dwain Esper	tt0026683
1746584858	1930	1930	Leathernecking	Leathernecking	Cline, Edward	Edward F. Cline	tt0021057
1746584182	1935	1935	Cyclone of the Saddle	Cyclone of the Saddle	Clifton, Elmer	Elmer Clifton	tt0026254
1746420971	1994	1994	Ready to Wear	Ready to Wear	Altman, Robert	Robert Altman	tt0110907
1746585327	1929	1929	Thundering Thompson	Thundering Thompson	Wilson, B. Frank	Ben F. Wilson	tt0020500
1746496812	1919	1919	Yankee Doodle in Berlin	Yankee Doodle in Berlin	Jones, Richard	F. Richard Jones	tt0010925
1746530733	1950	1949	Sands of Iwo Jima	Sands of Iwo Jima	Dwan, Allan	Allan Dwan	tt0041841
1746583580	1928	1928	The Crimson City	The Crimson City	Mayo, Archie	Archie Mayo	tt0018804
1746421310	1993	1993	Son-In-Law	Son in Law	Rash, Steve	Steve Rash	tt0108186
1746585035	1939	1939	The Fighting Renegade	The Fighting Renegade	Newfield, Sam	Sam Newfield	tt0031306
1746434244	1990	1990	Basket Case 2	Basket Case 2	Henenlotter, Frank	Frank Henenlotter	tt0099108
1746542761	1924	1924	Sundown	Sundown	Trimble, Laurence; Hoyt, Harry O.	Harry O. Hoyt	tt0015379
1746501525	1914	1914	The Will O' the Wisp	The Will o' the Wisp	Otto, Henry	Henry Otto	tt0004817
1746508734	1924	1924	The Measure of a Man	The Measure of a Man	Rosson, Arthur	Arthur Rosson	tt0015127
1746436790	1998	1997	Men with Guns	Men with Guns	Sayles, John	John Sayles	tt0119657
1746567994	1933	1932	After the Ball	After the Ball	Rosmer, Milton	Milton Rosmer	tt0023743
1746508409	1927	1926	Smashed Back	Smashed Back		Forrest Sheldon	tt1694101
1746584481	1930	1930	Temple Tower	Temple Tower	Gallaher, Donald	Donald Gallaher	tt0021457
1746555670	1956	1956	The Catered Affair	The Catered Affair	Brooks, Richard	Richard Brooks	tt0049061
1746553796	1965	1965	The World of Abbott and Costello	The World of Abbott and Costello		Charles Barton	tt0059921
1746500525	1949	1949	Dynamite	Dynamite	Pine, William H.	William H. Pine	tt0041325
1746561985	1954	1954	Overland Pacific	Overland Pacific	Sears, Fred F.	Fred F. Sears	tt0047323
1769970176	1912	1912	War's Havoc	War's Havoc		Kenean Buel	tt0002568
1746392673	1981	1981	Continental Divide	Continental Divide	Apted, Michael	Michael Apted	tt0082200
1746502037	1916	1916	Hidden Valley	Hidden Valley	Warde, Ernest	Ernest C. Warde	tt0157754
1746582518	1935	1935	Lawless Riders	Lawless Riders	Bennet, Spencer Gordon	Spencer Gordon Bennet	tt0026615
1746513657	1916	1916	The Twin Triangle	The Twin Triangle	Harvey, Harry	Harry Harvey	tt0007484
1746186196	1965	1965	Once a Thief	Once a Thief	Nelson, Ralph	Ralph Nelson	tt0059540
1746508899	1926	1926	The Blackbird	The Blackbird	Browning, Tod	Tod Browning	tt0016655
1746582623	1935	1935	I'll Love You Always	I'll Love You Always	Bulgakov, Leo	Leo Bulgakov	tt0026514
1746569510	1957	1957	Stopover Tokyo	Stopover Tokyo	Breen, Richard L.	Richard L. Breen	tt0051011
1746437158	1999	1999	Molly	Molly	Duigan, John	John Duigan	tt0143746
1746582152	1929	1929	Daughters of Desire	Daughters of Desire	King, Burton	Burton L. King	tt0019807
1746566139	1958	1958	Joy Ride	Joy Ride	Bernds, Edward	Edward Bernds	tt0051803
1746570454	1933	1933	The Life of Jimmy Dolan	The Life of Jimmy Dolan	Mayo, Archie	Archie Mayo	tt0024257
1746485567	1918	1918	Beauty and the Rogue	Beauty and the Rogue	King, Henry	Henry King	tt0008869
1746409933	1991	1990	Lionheart	Lionheart	Lettich, Sheldon	Sheldon Lettich	tt0100029
1746542676	1948	1948	Triggerman	Triggerman	Bretherton, Howard	Howard Bretherton	tt0040903
1746577387	1958	1958	The Whole Truth	The Whole Truth	Guillermin, John	John Guillermin	tt0052391
1746421583	1995	1994	Don Juan DeMarco	Don Juan DeMarco	Leven, Jeremy	Jeremy Leven	tt0112883
1746423337	1992	1992	This Is My Life	This Is My Life	Ephron, Nora	Nora Ephron	tt0105577
1745341998	2008	2008	Forgetting Sarah Marshall	Forgetting Sarah Marshall	Stoller, Nicholas	Nicholas Stoller	tt0800039
1746420361	1988	1988	Rambo III	Rambo III	MacDonald, Peter	Peter MacDonald	tt0095956
1746504764	1918	1918	The Lady of the Dug-Out	The Lady of the Dugout	Van Dyke, W. S.	W.S. Van Dyke	tt0009273
1746573824	1958	1958	Imitation General	Imitation General	Marshall, George	George Marshall	tt0051767
1746554275	1964	1964	Babo 73	Babo 73	Downey, Robert	Robert Downey Sr.	tt0138311
1746438600	1945	1945	China Sky	China Sky	Enright, Ray	Ray Enright	tt0037593
1746584259	1935	1935	Mister Dynamite	Mister Dynamite	Crosland, Alan	Alan Crosland	tt0026724
1746584635	1929	1929	The Voice Within	The Voice Within	Archainbaud, George	George Archainbaud	tt0020559
1746582384	1931	1931	Two Gun Man	The Two Gun Man	Rosen, Phil	Phil Rosen	tt0022514
1746453853	1941	1941	A Dangerous Game	A Dangerous Game	Rawlins, John	John Rawlins	tt0033509
1746485621	1920	1920	Silk Hosiery	Silk Hosiery	Niblo, Fred	Fred Niblo	tt0012687
1746394702	1983	1983	Jaws 3-D	Jaws 3 D	Alves, Joe	Joe Alves	tt0085750
1746582194	1928	1928	While the City Sleeps	While the City Sleeps	Conway, Jack	Jack Conway	tt0019571
1746422010	1990	1990	Almost an Angel	Almost an Angel	Cornell, John	John Cornell	tt0099018
1746249987	1975	1975	Milestones	Milestones	Douglas, John; Kramer, Robert	John Douglas	tt0073389

1746575077	1935	1935	East of Java	East of Java	Melford, George	George Melford	tt0026304
1746576327	1931	1931	Expensive Women	Expensive Women	Henley, Hobart	Hobart Henley	tt0021845
1746504031	1920	1919	Hidden Charms	Hidden Charms	Brodsky, Samuel	Samuel Brodsky	tt0179847
1746434877	1999	1999	Tequila Body Shots	Tequila Body Shots	Shyu, Tony	Tony Shyu	tt0144637
1746580753	1938	1938	Starlight over Texas	Starlight Over Texas	Herman, Al	Albert Herman	tt0030791
1746571370	1935	1935	Accent on Youth	Accent on Youth	Ruggles, Wesley	Wesley Ruggles	tt0026034
1746514612	1916	1916	The Shop Girl	The Shop Girl	Baker, George D.	George D. Baker	tt0007342
1746576585	1931	1931	Local Boy Makes Good	Local Boy Makes Good	LeRoy, Mervyn	Mervyn LeRoy	tt0022085
1746454212	1943	1943	Paris After Dark	Paris After Dark	Moguy, Leonide	Léonide Moguy	tt0036251
1746422091	1940	1940	Young Bill Hickok	Young Bill Hickok	Kane, Joseph	Joseph Kane	tt0033286
1746570161	1933	1933	Master of Men	Master of Men	Hillyer, Lambert	Lambert Hillyer	tt0024309
1746570007	1958	1958	Macabre	Macabre	Castle, William	William Castle	tt0051885
1746218432	1967	1967	Gentle Giant	Gentle Giant	Neilson, James	James Neilson	tt0061703
1746467828	1917	1917	House of Cards	House of Cards	Blaché, Alice Guy	Alice Guy	tt0008123
1746422176	1995	1995	Vampire in Brooklyn	Vampire in Brooklyn	Craven, Wes	Wes Craven	tt0114825
1746584134	1940	1940	High School	High School	Nicholls, George	George Nichols Jr.	tt0032595
1746566158	1957	1957	Taming Sutton's Gal	Taming Sutton's Gal	Selander, Lesley	Lesley Selander	tt0051050
1746571896	1931	1931	The Beloved Bachelor	Beloved Bachelor	Corrigan, Lloyd	Lloyd Corrigan	tt0021651
1769969500	1911	1911	Love Heeds Not Showers	Love Heeds Not Showers	Moore, Owen	Owen Moore	tt0001749
1746500042	1924	1924	Shootin' Square	Shootin' Square			tt0132511
1746499955	1950	1950	The Flying Saucer	The Flying Saucer	Conrad, Mikel	Mikel Conrad	tt0042469
1746525065	1920	1920	Sherry	Sherry	Lewis, Edgar	Edgar Lewis	tt0197875
1746500891	1916	1915	Ambition	Ambition	Vincent, James	Howard M. Mitchell	tt0412360
1746585004	1940	1940	Christmas in July	Christmas in July	Sturges, Preston	Preston Sturges	tt0032338
1745374399	2002	2001	Circuit	Circuit	Shafer, Dirk	Dirk Shafer	tt0218107
1745293318	2007	2007	You Kill Me	You Kill Me	Dahl, John	John Dahl	tt0796375
1769970455	1967	1967	The Hookers	The Hookers	Horthy, Jalo Miklos	Jalo Miklos Horthy	tt0219808
1746567004	1932	1932	The Washington Masquerade	The Washington Masquerade	Brabin, Charles	Charles Brabin	tt0023672
1746583199	1929	1929	Mother's Boy	Mother's Boy	Barker, Bradley	Bradley Barker	tt0020187
1746408702	1987	1987	Morgan Stewart's Coming Home	Morgan Stewart's Coming Home	Smithee, Alan; Winsor , Terry ; Aaron	Paul Aaron	tt0093567
1746543073	1916	1916	The Plow Girl	The Plow Girl	Leonard, Robert	Robert Z. Leonard	tt0007192
1746577735	1936	1936	Three on the Trail	Three on the Trail	Bretherton, Howard	Howard Bretherton	tt0028377
1746582643	1938	1938	Two Gun Justice	Two Gun Justice	James, Alan	Alan James	tt0030903
1746486285	1919	1919	The Poppy Girl's Husband	The Poppy Girl's Husband	Hart, William S.; Hillyer, Lambert	William S. Hart	tt0010581
1746211076	1971	1971	Carnal Knowledge	Carnal Knowledge	Nichols, Mike	Mike Nichols	tt0066892
1746454736	1996	1996	Love Is All There Is	Love Is All There Is	Bologna, Joseph	Joseph Bologna	tt0116928
1746566226	1953	1953	Ride, Vaquero!	Ride, Vaquero!	Farrow, John	John Farrow	tt0046239
1746515839	1922	1922	The Milky Way	The Milky Way		W.S. Van Dyke	tt0013391
1769970558	1968	1968	Darling, Are You Bored With Men?	Darling, Are You Bored with Men?	Meyer, John	Nick Millard	tt0216661
1746454851	1941	1941	The Blonde from Singapore	The Blonde from Singapore	Dmytryk, Edward	Edward Dmytryk	tt0033402
1746508434	1950	1950	The Second Woman	The Second Woman	Kern, James V.	James V. Kern	tt0044013
1746520183	1949	1949	Caught	Caught	Opuls, Max	Max Ophüls	tt0040221
1746583187	1950	1950	So Young, So Bad	So Young So Bad	Vorhaus, Bernard	Bernard Vorhaus	tt0042982
1746497428	1918	1918	Her Mistake	Her Mistake	Steger, Julius	Julius Steger	tt0184563
1746584537	1931	1931	Susan Lenox (Her Fall and Rise)	Susan Lenox <Her Fall and Rise>	Leonard, Robert Z.	Robert Z. Leonard	tt0022453
1746583404	1950	1950	Three Came Home	Three Came Home	Negulesco, Jean	Jean Negulesco	tt0043041
1746210743	1973	1973	Save the Tiger	Save the Tiger	Avildsen, John G.	John G. Avildsen	tt0070640
1746563657	1953	1953	All the Brothers Were Valiant	All the Brothers Were Valiant	Thorpe, Richard	Richard Thorpe	tt0045494
1746584047	1938	1938	Secrets of a Nurse	Secrets of a Nurse	Lubin, Arthur	Arthur Lubin	tt0030726
1746501004	1914	1914	Imar the Servitor	Imar the Servitor		John B. O'Brien	tt0004142
1746583856	1930	1930	Madonna of the Streets	Madonna of the Streets	Robertson, John S.	John S. Robertson	tt0021108
1746584642	1929	1929	45 Calibre War	.45 Calibre War	Maloney, Leo	Leo D. Maloney	tt0019894
1769970659	1968	1968	The Taming	The Taming	Arkless, Robert	Robert Arkless	tt0131048
1746455202	1999	1999	The Suburbans	The Suburbans	Ward, Donal Lardner	Donal Lardner Ward	tt0157075
1746585242	1930	1930	Ladies Must Play	Ladies Must Play	Cannon, Raymond	Raymond Cannon	tt0021038
1746584249	1940	1940	Gangs of Chicago	Gangs of Chicago	Lubin, Arthur	Arthur Lubin	tt0032509
1745293738	2005	2005	Coach Carter	Coach Carter	Carter, Thomas	Thomas Carter	tt0393162

1746233872	1968	1968	Wild in the Streets	Wild in the Streets	Shear, Barry	Barry Shear	tt0063808
1746576913	1960	1959	Take a Giant Step	Take a Giant Step	Leacock, Philip	Philip Leacock	tt0053331
1746580950	1951	1951	Anne of the Indies	Anne of the Indies	Tourneur, Jacques	Jacques Tourneur	tt0043288
1746574947	1935	1935	North of Arizona	North of Arizona	Ray, B. B.	Harry S. Webb	tt0026790
1746569584	1953	1953	Salome	Salome	Dieterle, William	William Dieterle	tt0046269
1746516469	1920	1920	Smiling All the Way	Smiling All the Way	Butler, Fred J.	Fred J. Butler	tt0011710
1746523428	1923	1923	A Clouded Name	A Clouded Name	Huhn, Austin O.	Austin O. Huhn	tt0013930
1746583155	1937	1937	Git Along Little Dogies	Git Along Little Dogies	Kane, Joseph	Joseph Kane	tt0028934
1746499880	1945	1945	Identity Unknown	Identity Unknown	Colmes, Walter	Walter Colmes	tt0037811
1746572206	1932	1932	Law and Lawless	Law and Lawless	Schaefer, Armand	Armand Schaefer	tt0023120
1746503982	1920	1920	The Wrong Woman	The Wrong Woman	Abramson, Ivan	Ivan Abramson	tt0012867
1746570455	1933	1933	Smoke Lightning	Smoke Lightning	Howard, David	David Howard	tt0024576
1746582319	1929	1929	Wolves of the City	Wolves of the City	Jason, Leigh	Leigh Jason	tt0020597
1746524939	1914	1914	The Escape	The Escape	Griffith, David W.	D.W. Griffith	tt0003890
1746502995	1910	1910	The Tattooed Arm	The Tattooed Arm			tt1793962
1746576285	1931	1931	Strictly Dishonorable	Strictly Dishonorable	Stahl, John M.	John M. Stahl	tt0022437
1746566311	1955	1955	Son of Sinbad	Son of Sinbad	Tetzlaff, Ted	Ted Tetzlaff	tt0048642
1746454656	1942	1942	Dawn on the Great Divide	Dawn on the Great Divide	Bretherton, Howard	Howard Bretherton	tt0034638
1746584221	1939	1939	Jeepers Creepers	Jeepers Creepers	McDonald, Frank	Frank McDonald	tt0151123
1746475265	1944	1944	The Racket Man	The Racket Man	Lederman, D. Ross	D. Ross Lederman	tt0036287
1746520745	1949	1949	Abandoned	Abandoned	Newman, Joe	Joseph M. Newman	tt0041084
1746566668	1933	1933	The Emperor Jones	The Emperor Jones	Murphy, Dudley	Dudley Murphy	tt0023985
1746574914	1938	1938	Cowboy from Brooklyn	Cowboy from Brooklyn	Bacon, Lloyd	Lloyd Bacon	tt0030019
1746523852	1927	1927	The Gay Retreat	The Gay Retreat	Stoloff, Ben	Benjamin Stoloff	tt0017921
1746393289	1983	1983	Deal of the Century	Deal of the Century	Friedkin, William	William Friedkin	tt0085412
1746520615	1949	1949	Angels in Disguise	Angels in Disguise	Yarbrough, Jean	Jean Yarbrough	tt0041124
1746465315	1944	1944	Call of the Jungle	Call of the Jungle	Rosen, Phil	Phil Rosen	tt0036688
1746235376	1979	1979	C.H.O.M.P.S.	C.H.O.M.P.S.	Chaffey, Don	Don Chaffey	tt0078924
1746507749	1925	1925	Bashful Buccaneer	Bashful Buccaneer	Brown, Harry J.	Harry Joe Brown	tt0015604
1746584682	1929	1929	Whispering Winds	Whispering Winds	Flood, James	James Flood	tt0020583
1746435722	1940	1940	Arizona Frontier	Arizona Frontier	Herman, Al	Albert Herman	tt0032222
1746574558	1935	1935	Toll of the Desert	Toll of the Desert	Williams, Lester	William Berke	tt0028393
1746198302	1970	1970	The Super Fight	The Super Fight	Woroner, Murry	Murray Woroner	tt0065050
1746507853	1924	1924	Lone Hand Texas	The Lone Hand Texan		W. Adcook	tt0246043
1746577765	1938	1938	Boys Town	Boys Town	Taurog, Norman	Norman Taurog	tt0029942
1746423268	1992	1992	American Me	American Me	Olmos, Edward James	Edward James Olmos	tt0103671
1746514456	1915	1915	My Valet	My Valet	Sennett, Mack	Mack Sennett	tt0005792
1746553561	1965	1965	Git!	Git!	Kadison, Ellis	Ellis Kadison	tt0169953
1746500725	1925	1925	The Handsome Brute	The Handsome Brute	Eddy, Robert	Robert Eddy	tt0015890
1746475246	1948	1948	Big Town Scandal	Big Town Scandal	Thomas, William C.	William C. Thomas	tt0040162
1746581732	1928	1927	Chicago	Chicago	Urson, Frank; DeMille, Cecil B.	Frank Urson	tt0017750
1746514504	1923	1923	Can a Woman Love Twice?	Can a Woman Love Twice?	Horne, James W.	James W. Horne	tt0013910
1746421604	1993	1993	Hocus Pocus	Hocus Pocus	Ortega, Kenny	Kenny Ortega	tt0107120
1746455897	1997	1997	Conspiracy Theory	Conspiracy Theory	Donner, Richard	Richard Donner	tt0118883
1746424364	1942	1942	Somewhere I'll Find You	Somewhere I'll Find You	Ruggles, Wesley	Wesley Ruggles	tt0035356
1746567968	1933	1933	Rusty Rides Alone	Rusty Rides Alone	Lederman, D. Ross	D. Ross Lederman	tt0024512
1746585383	1939	1939	The Law Comes to Texas	The Law Comes to Texas	Levering, Joseph	Joseph Levering	tt0031556
1746209359	1968	1968	The Impossible Years	The Impossible Years	Gordon, Michael	Michael Gordon	tt0063128
1746582865	1935	1935	Midnight Phantom	Midnight Phantom	Ray, B. B.	Bernard B. Ray	tt0026711
1746569652	1957	1957	War Drums	War Drums	LeBorg, Reginald	Reginald Le Borg	tt0051179
1746515065	1922	1922	Angel Citizens	Angel Citizens	Ford, Francis	Francis Ford	tt0013664
1746523366	1922	1922	Gun Shy	Gun Shy	Neitz, Alvin J.	Alan James	tt0013208
1746520299	1925	1925	Peacock Feathers	Peacock Feathers	Gade, Svend	Svend Gade	tt0016206
1746561051	1932	1932	The Midnight Lady	The Midnight Lady	Thorpe, Richard	Richard Thorpe	tt0023218
1746524611	1915	1915	Judy Forgot	Judy Forgot	Hunter, T. Hayes	T. Hayes Hunter	tt0005578
1746419285	1940	1940	Gold Rush Maisie	Gold Rush Maisie	Marin, Edwin L.	Edwin L. Marin	tt0032539
1746513611	1916	1916	Life's Shadows	Life's Shadows	Nigh, William	William Nigh	tt0006921

1746566911	1934	1934	Dr. Monica	Dr. Monica	Keighley, William	William Keighley	tt0025068
1746248753	1980	1979	Smokey and the Hotwire Gang	Smokey and the Hotwire Gang	Cardoza, Anthony	Anthony Cardoza	tt0188203
1746497504	1917	1917	The Flashlight	The Flashlight	Park, Ida May	Ida May Park	tt0007955
1746574197	1963	1963	Operation Bikini	Operation Bikini	Carras, Anthony	Anthony Carras	tt0057387
1746584709	1929	1929	Silent Sentinel	Silent Sentinel	Neitz, Alvin J.	Alan James	tt0019383
1746560628	1932	1932	Street of Women	Street of Women	Mayo, Archie	Archie Mayo	tt0023529
1769973216	1970	1969	Once Upon a Body	Once Upon a Body		Sam S. Catah	tt0064759
1746507789	1924	1924	California in '49	California in '49	Jaccard, Jacques; Wilson, Ben	Jacques Jaccard	tt0014756
1746565928	1953	1953	The Nebraskan	The Nebraskan	Sears, Fred F.	Fred F. Sears	tt0046122
1746585104	1929	1929	Kid Gloves	Kid Gloves	Enright, Ray	Ray Enright	tt0020057
1746419801	1994	1994	Hoop Dreams	Hoop Dreams	James, Steve	Steve James	tt0110057
1746574662	1933	1933	Secret of the Blue Room	Secret of the Blue Room	Neumann, Kurt	Kurt Neumann	tt0024538
1746533014	1928	1928	Manhattan Knights	Manhattan Knights	King, Burton	Burton L. King	tt0019138
1746208319	1967	1967	Waterhole #3	Waterhole #3	Graham, William	William A. Graham	tt0062477
1746582595	1938	1938	Sons of the Legion	Sons of the Legion	Hogan, James	James P. Hogan	tt0030771
1746567742	1933	1933	Man's Castle	Man's Castle	Borzage, Frank	Frank Borzage	tt0024302
1746524406	1914	1914	The Dream Woman	The Dream Woman	Blaché, Alice	Alice Guy	tt0003869
1746571932	1910	1910	A Child's Stratagem	A Child's Stratagem	Griffith, D. W.	D.W. Griffith	tt0001153
1746533180	1915	1915	The Ringtailed Rhinoceros	The Ringtailed Rhinoceros	Terwilliger, George	George Terwilliger	tt0005971
1746574559	1933	1933	This Is America	This Is America			tt0138872
1746584958	1939	1939	The Man Who Dared	The Man Who Dared	Wilbur, Crane	Crane Wilbur	tt0031461
1746566233	1954	1954	Valley of the Kings	Valley of the Kings	Pirosh, Robert	Robert Pirosh	tt0047641
1746583646	1931	1931	The Viking	The Viking	Melford, George	Varick Frissell	tt0022537
1746577734	1938	1938	Songs and Bullets	Songs and Bullets	Newfield, Sam	Sam Newfield	tt0030770
1745359207	2002	2002	The Crocodile Hunter: Collision Course	The Crocodile Hunter: Collision Course	Stainton, John	John Stainton	tt0305396
1746566086	1956	1957	Gun Girls	Gun Girls	Dertano, Robert C.	Robert C. Dertano	tt0049284
1746523359	1916	1916	Sorrows of Happiness	Sorrows of Happiness	Kaufman, Joseph	Joseph Kaufman	tt0158976
1746565501	1932	1931	Hell Divers	Hell Divers	Hill, George	George W. Hill	tt0022987
1746567997	1952	1952	Bela Lugosi Meets a Brooklyn Gorilla	Bela Lugosi Meets a Brooklyn Gorilla	Beaudine, William	William Beaudine	tt0044406
1746423530	1941	1941	Barnacle Bill	Barnacle Bill	Thorpe, Richard	Richard Thorpe	tt0033378
1746248495	1967	1967	The Graduate	The Graduate	Nichols, Mike	Mike Nichols	tt0061722
1746454700	1942	1942	The Great Gildersleeve	The Great Gildersleeve	Douglas, Gordon	Gordon Douglas	tt0034810
1746569714	1953	1953	Those Redheads from Seattle	Those Redheads from Seattle	Foster, Lewis R.	Lewis R. Foster	tt0046422
1746219425	1978	1978	F.I.S.T.	F.I.S.T.	Jewison, Norman	Norman Jewison	tt0077531
1746577929	1935	1935	Sweepstake Annie	Sweepstake Annie	Nigh, William	William Nigh	tt0027063
1746507865	1926	1926	Fighting Thorobreds	Fighting Thorobreds	Brown, Harry J.	Harry Joe Brown	tt0016868
1746569954	1957	1957	Funny Face	Funny Face	Donen, Stanley	Stanley Donen	tt0050419
1746419704	1940	1940	Billy the Kid in Texas	Billy the Kid in Texas	Stewart, Peter	Sam Newfield	tt0032253
1746566307	1954	1954	Beau Brummell	Beau Brummell	Bernhardt, Curtis	Curtis Bernhardt	tt0046759
1746454082	1942	1942	The Panther's Claw	The Panther's Claw	Beaudine, William	William Beaudine	tt0035171
1746580988	1950	1950	Never a Dull Moment	Never a Dull Moment	Marshall, George	George Marshall	tt0042784
1746500994	1916	1916	The Price of Malice	The Price of Malice	Lund, O. A. C.	O.A.C. Lund	tt0007211
1746566503	1952	1953	Born to the Saddle	Born to the Saddle	Beaudine, William	William Beaudine	tt0045573
1746563899	1952	1952	The Brigand	The Brigand	Karlson, Phil	Phil Karlson	tt0044449
1746566277	1955	1955	The Left Hand of God	The Left Hand of God	Dmytryk, Edward	Edward Dmytryk	tt0048291
1746581153	1960	1959	Come Back, Africa	Come Back, Africa	Rogosin, Lionel	Lionel Rogosin	tt0049087
1746569583	1955	1954	The Far Country	The Far Country	Mann, Anthony	Anthony Mann	tt0048055
1746435158	1943	1943	No Time for Love	No Time for Love	Leisen, Mitchell	Mitchell Leisen	tt0036208
1746516470	1920	1920	Bubbles	Bubbles	Mack, Wayne	Wayne Mack	tt0187763
1746584585	1927	1928	His Last Bullet	His Last Bullet	Wilson, B. Frank	Ben F. Wilson	tt0264685
1746473906	1946	1946	My Pal Trigger	My Pal Trigger	McDonald, Frank	Frank McDonald	tt0038764
1746512776	1915	1915	The Edge of the Abyss	The Edge of the Abyss	Edwards, Walter	Walter Edwards	tt0005237
1746581534	1910	1910	The Irish Boy	The Irish Boy			tt0233951
1746437361	1943	1943	Riders of the Northwest Mounted	Riders of the Northwest Mounted	Berke, William	William Berke	tt0166777
1746454018	1942	1942	My Sister Eileen	My Sister Eileen	Hall, Alexander	Alexander Hall	tt0035105
1746209783	1971	1971	Dirty Harry	Dirty Harry	Siegel, Don	Don Siegel	tt0066999
1746394191	1991	1991	A Kiss Before Dying	A Kiss Before Dying	Dearden, James	James Dearden	tt0102220

1745359314	2002	2001	Harvard Man	Harvard Man	Toback, James	James Toback	tt0242508
1746577689	1936	1936	The Green Pastures	The Green Pastures	Connelly, Marc; Keighley, William	Marc Connelly	tt0027700
1746582433	1936	1936	Poppy	Poppy	Sutherland, A. Edward	A. Edward Sutherland	tt0028120
1746574016	1957	1957	I Was a Teenage Werewolf	I Was a Teenage Werewolf	Fowler, Gene	Gene Fowler Jr.	tt0050530
1746524860	1927	1927	The Warning	The Warning	Seitz, George B.	George B. Seitz	tt0018549
1746580767	1938	1938	Pals of the Saddle	Pals of the Saddle	Sherman, George	George Sherman	tt0030550
1746567234	1932	1932	Under-Cover Man	Under Cover Man	Flood, James	James Flood	tt0023638
1746583704	1931	1931	Three Girls Lost	Three Girls Lost	Lanfield, Sidney	Sidney Lanfield	tt0022479
1746566301	1954	1954	The Miami Story	The Miami Story	Sears, Fred F.	Fred F. Sears	tt0047232
1746584646	1928	1927	Hot Heels	Hot Heels	Craft, William James	William James Craft	tt0019023
1746473883	1946	1946	Bedlam	Bedlam	Robson, Mark	Mark Robson	tt0038343
1746219015	1980	1979	The Hitter	The Hitter	Leitch, Christopher	Christopher Leitch	tt0179219
1746569950	1958	1958	Sing, Boy, Sing	Sing Boy Sing	Ephron, Henry	Henry Ephron	tt0052198
1746454165	1997	1997	Star Maps	Star Maps	Arteta, Miguel	Miguel Arteta	tt0120197
1746424625	1940	1940	City of Chance	City of Chance	Cortez, Ricardo	Ricardo Cortez	tt0032343
1746570729	1934	1934	Hide-Out	Hide Out	Van Dyke, W. S.	W.S. Van Dyke	tt0025251
1746574444	1951	1951	No Questions Asked	No Questions Asked	Kress, Harold F.	Harold F. Kress	tt0043861
1746504580	1919	1919	23 1/2 Hours' Leave	23 1/2 Hours' Leave	King, Henry	Henry King	tt0009855
1746586168	1939	1939	The Covered Trailer	The Covered Trailer	Meins, Gus	Gus Meins	tt0031187
1746499893	1948	1948	Angel on the Amazon	Angel on the Amazon	Auer, John H.	John H. Auer	tt0040095
1746556009	1954	1954	White Christmas	White Christmas	Curtiz, Michael	Michael Curtiz	tt0047673
1746467987	1917	1917	The Gentle Intruder	The Gentle Intruder	Kirkwood, James	James Kirkwood	tt0007995
1746514954	1914	1914	Your Girl and Mine: a Woman Suffrage Play	Your Girl and Mine: A Woman Suffrage Play	Warren, Giles	Giles Warren	tt0004842
1746392868	1982	1982	Tootsie	Tootsie	Pollack, Sydney	Sydney Pollack	tt0084805
1746577171	1960	1960	Strangers When We Meet	Strangers When We Meet	Quine, Richard	Richard Quine	tt0054345
1746259111	1980	1980	Where the Buffalo Roam	Where the Buffalo Roam	Linson, Art	Art Linson	tt0081748
1746581406	1961	1961	Fanny	Fanny	Logan, Joshua	Joshua Logan	tt0054866
1746568172	1934	1934	'Neath the Arizona Skies	'Neath the Arizona Skies	Fraser, Harry	Harry L. Fraser	tt0024805
1746561976	1954	1954	Pushover	Pushover	Quine, Richard	Richard Quine	tt0047377
1746582937	1935	1934	Cowboy Holiday	Cowboy Holiday	Hill, Bob	Robert F. Hill	tt0025006
1746394173	1982	1982	Dead Men Don't Wear Plaid	Dead Men Don't Wear Plaid	Reiner, Carl	Carl Reiner	tt0083798
1746580987	1962	1961	The Festival Girls	The Festival Girls	Jason, Leigh	Leigh Jason	tt0053822
1746520864	1925	1925	Alias Mary Flynn	Alias Mary Flynn	Ince, Ralph	Ralph Ince	tt0015550
1746199035	1969	1969	Hot Rod Action	Hot Rod Action	McCabe, Gene	Gene McCabe	tt2807330
1746433739	1991	1991	The Silence of the Lambs	The Silence of the Lambs	Demme, Jonathan	Jonathan Demme	tt0102926
1745359879	2008	2008	What Happens in Vegas	What Happens in Vegas	Vaughan, Tom	Tom Vaughan	tt1033643
1746524319	1915	1915	The Shooting of Dan Mcgrew	The Shooting of Dan McGrew	Blaché, Herbert	Herbert Blaché	tt0006037
1746585143	1929	1929	Strange Cargo	Strange Cargo		Benjamin Glazer	tt0020456
1769971161	1970	1970	Threesome	Threesome	Beale, Lee	Brandon Chase	tt0127939
1769975282	1916	1916	The Honor of the Road	The Honor of the Road		James W. Horne	tt3140200
1746421258	1995	1995	The Cure	The Cure	Horton, Peter	Peter Horton	tt0112757
1746467627	1917	1917	Melissa of the Hills	Melissa of the Hills	Kirkwood, James	James Kirkwood	tt0125397
1746422595	1993	1993	Last Action Hero	Last Action Hero	McTiernan, John	John McTiernan	tt0107362
1746576942	1963	1963	Blood Feast	Blood Feast	Lewis, Herschell G.	Herschell Gordon Lewis	tt0056875
1746520151	1924	1924	Conductor 1492	Conductor 1492	Hines, Charles; Griffin, Frank	Frank Griffin	tt0014797
1746504896	1921	1921	Discontented Wives	Discontented Wives	McGowan, J. P.	J.P. McGowan	tt0012110
1746582112	1951	1951	Arizona Manhunt	Arizona Manhunt	Brannon, Fred C.	Fred C. Brannon	tt0043295
1745359150	2003	2003	Biker Boyz	Biker Boyz	Bythewood, Reggie Rock	Reggie Rock Bythewood	tt0326769
1746200477	1969	1969	Nightmare in Wax	Nightmare in Wax	Townsend, Bud	Bud Townsend	tt0064729
1746474409	1946	1946	Blondie's Lucky Day	Blondie's Lucky Day	Berlin, Abby	Abby Berlin	tt0038368
1746585342	1929	1929	The Flying Marine	The Flying Marine	Rogell, Albert	Albert S. Rogell	tt0019888
1745303177	2007	2007	Fantastic Four: Rise of the Silver Surfer	Fantastic Four: Rise of the Silver Surfer	Story, Tim	Tim Story	tt0486576
1746502230	1915	1915	An Eye for an Eye	An Eye for an Eye		William Desmond Taylor	tt0005260
1746555277	1955	1955	Daddy Long Legs	Daddy Long Legs	Negulesco, Jean	Jean Negulesco	tt0047969
1769970413	1967	1967	Professor Lust	Professor Lust	Rose, Warner	William Rose	tt0242226
1746577291	1950	1950	Prisoners in Petticoats	Prisoners in Petticoats	Ford, Philip	Philip Ford	tt0127163
1745359973	2001	2000	Jails, Hospitals & Hip-Hop	Jails, Hospitals & Hip Hop	Benjamin, Mark ; Hoch, Danny	Mark Benjamin	tt0210130

1746409331	1986	1985	House	House	Miner, Steve	Steve Miner	tt0091223
1746580794	1935	1935	All the King's Horses	All the King's Horses	Tuttle, Frank	Frank Tuttle	tt0024823
1746500329	1924	1924	In High Gear	In High Gear	Bradbury, Robert North	Robert N. Bradbury	tt0123907
1746580420	1935	1935	The Singing Vagabond	The Singing Vagabond	Pierson, Carl	Carl Pierson	tt0028258
1746569557	1957	1956	Revolt at Fort Laramie	Revolt at Fort Laramie	Selander, Lesley	Lesley Selander	tt0050895
1746509568	1923	1923	Her Accidental Husband	Her Accidental Husband	Fitzgerald, Dallas M.	Dallas M. Fitzgerald	tt0014126
1746585034	1940	1940	Fugitive from a Prison Camp	Fugitive from a Prison Camp	Collins, Lewis D.	Lewis D. Collins	tt0032500
1745341474	2009	2009	Good Hair	Good Hair	Stilson, Jeff	Jeff Stilson	tt1213585
1769977350	1977	1977	Female Fever	Female Fever	Patrick, Alain	Alain Patrick	tt0849439
1746199997	1966	1966	Tarzan and the Valley of Gold	Tarzan and the Valley of Gold	Day, Robert	Robert Day	tt0061067
1746514567	1916	1916	The Marriage Bond	The Marriage Bond	Marston, Lawrence	Lawrence Marston	tt0007041
1746581275	1960	1960	Juke Box Racket	Juke Box Racket	Barris, George; Geallis, Jim	George Barris	tt0053972
1746583569	1929	1929	The Cock-Eyed World	The Cock Eyed World	Walsh, Raoul	Raoul Walsh	tt0019776
1745374965	2009	2009	Transformers: Revenge of the Fallen	Transformers: Revenge of the Fallen	Bay, Michael	Michael Bay	tt1055369
1746562293	1953	1953	The Golden Blade	The Golden Blade	Juran, Nathan	Nathan Juran	tt0045828
1746584999	1940	1940	Arizona Gang Busters	Arizona Gang Busters	Stewart, Peter	Sam Newfield	tt0032223
1746584272	1936	1936	The Mandarin Mystery	The Mandarin Mystery	Staub, Ralph	Ralph Staub	tt0029207
1746492375	1948	1948	Larceny	Larceny	Sherman, George	George Sherman	tt0039550
1746420973	1983	1983	The Man Who Loved Women	The Man Who Loved Women	Edwards, Blake	Blake Edwards	tt0085891
1746420988	1995	1995	Judge Dredd	Judge Dredd	Cannon, Danny	Danny Cannon	tt0113492
1746581108	1960	1960	Get Outta Town	Get Outta Town	Davis, Charles	Charles Davis	tt0179823
1746437840	1944	1944	In the Meantime, Darling	In the Meantime, Darling	Preminger, Otto	Otto Preminger	tt0036955
1746435344	1996	1996	Executive Decision	Executive Decision	Baird, Stuart	Stuart Baird	tt0116253
1746520648	1926	1927	Racing Romance	Racing Romance	Brown, Harry J.	Fred J. Balshofer	tt4578182
1746508492	1926	1926	What Happened to Jones	What Happened to Jones	Seiter, William A.	William A. Seiter	tt0017539
1746514115	1910	1910	The Mystery of the Torn Note	The Mystery of the Torn Note			tt1080760
1746577533	1935	1935	Silent Valley	Silent Valley	Ray, B. B.	Bernard B. Ray	tt0026997
1769970446	1967	1967	The Love Rebellion	The Love Rebellion	Sarno, Joe	Joseph W. Sarno	tt0059407
1746566727	1932	1932	Rebecca of Sunnybrook Farm	Rebecca of Sunnybrook Farm	Santell, Alfred	Alfred Santell	tt0023378
1746566913	1932	1932	High Speed	High Speed	Lederman, D. Ross	D. Ross Lederman	tt0023008
1746465117	1944	1944	Block Busters	Block Busters	Fox, Wallace	Wallace Fox	tt0036650
1746440075	1995	1995	The Jerky Boys	The Jerky Boys	Melkonian, James	James Melkonian	tt0110189
1746498482	1918	1918	The Phantom Riders	The Phantom Riders	Ford, Jack	John Ford	tt0009494
1746437856	1941	1941	Footsteps in the Dark	Footsteps in the Dark	Bacon, Lloyd	Lloyd Bacon	tt0033616
1746514813	1915	1915	Emmy of Stork's Nest	Emmy of Stork's Nest	Nigh, William	William Nigh	tt0005243
1746209277	1967	1967	Oddo	Oddo	Davis, Joe	Nick Millard	tt0253368
1746454050	1944	1944	The National Barn Dance	National Barn Dance	Bennett, Hugh	Hugh Bennett	tt0037119
1746573850	1956	1956	Jubal	Jubal	Daves, Delmer	Delmer Daves	tt0048233
1746393645	1988	1988	Child's Play	Child's Play	Holland, Tom	Tom Holland	tt0094862
1746569521	1953	1953	Tarzan and the She-Devil	Tarzan and the She Devil	Neumann, Kurt	Kurt Neumann	tt0046404
1746520949	1949	1949	Sword in the Desert	Sword in the Desert	Sherman, George	George Sherman	tt0041940
1746235008	1968	1968	Stranger in Hollywood	Stranger in Hollywood	Slipyj, Rodion	Rodion Slipyj	tt0284520
1746574110	1961	1961	No Return Address	No Return Address	Grattan, Alexander	Alejandro Grattan	tt0251302
1746184199	1965	1965	The Tarts	The Tarts	Davis, Joe	Nick Millard	tt0259040
1746467766	1917	1917	Reaching for the Moon	Reaching for the Moon	Emerson, John	John Emerson	tt0008498
1746562108	1953	1953	Man of Conflict	Man of Conflict	Makelim, Hal R.	Hal R. Makelim	tt0046039
1746577243	1958	1958	Teacher's Pet	Teacher's Pet	Seaton, George	George Seaton	tt0052278
1746583451	1950	1950	Please Believe Me	Please Believe Me	Taurog, Norman	Norman Taurog	tt0042848
1746566364	1958	1958	Giant from the Unknown	Giant from the Unknown	Cunha, Richard E.	Richard E. Cunha	tt0051654
1745293143	2005	2005	Heights	Heights	Terrio, Chris	Chris Terrio	tt0382073
1746420223	1993	1993	The War Room	The War Room		Chris Hegedus	tt0108515
1746583009	1940	1940	The Man with Nine Lives	The Man with Nine Lives	Grinde, Nick	Nick Grinde	tt0032753
1746572227	1931	1931	It Pays to Advertise	It Pays to Advertise	Tuttle, Frank	Frank Tuttle	tt0022006
1746501483	1910	1910	Thou Shalt Not	Thou Shalt Not	Griffith, D. W.	D.W. Griffith	tt0001415
1746507776	1926	1926	It Must Be Love	It Must Be Love	Green, Alfred E.	Alfred E. Green	tt0017002
1746513704	1923	1923	The Huntress	The Huntress	Reynolds, Lynn	John Francis Dillon	tt0014143
1746420778	1993	1993	Jack the Bear	Jack the Bear	Herskovitz, Marshall	Marshall Herskovitz	tt0107247

1746582622	1937	1937	Some Blondes Are Dangerous	Some Blondes Are Dangerous	Carruth, Milton	Milton Carruth	tt0029587
1746566327	1953	1953	Thunder over the Plains	Thunder Over the Plains	DeToth, Andre	André De Toth	tt0046426
1746483462	1917	1917	Even As You and I	Even As You and I	Weber, Lois	Lois Weber	tt0007902
1746578733	1962	1962	Long Day's Journey Into Night	Long Day's Journey Into Night	Lumet, Sidney	Sidney Lumet	tt0056196
1745342684	2009	2009	The Missing Person	The Missing Person	Buschel, Noah	Noah Buschel	tt1105512
1746582729	1937	1937	Blossoms on Broadway	Blossoms on Broadway	Wallace, Richard	Richard Wallace	tt0028642
1746503310	1922	1922	The Man Who Waited	The Man Who Waited	Luddy, Edward I.	Edward Ludwig	tt0013365
1746566286	1958	1958	The Beast of Budapest	The Beast of Budapest	Jones, Harmon C.	Harmon Jones	tt0051403
1769969970	1911	1911	The Water War	The Water War		Allan Dwan	tt0433199
1746437423	1942	1942	Mexican Spitfire Sees a Ghost	Mexican Spitfire Sees a Ghost	Goodwins, Leslie	Leslie Goodwins	tt0035059
1746256646	1975	1975	Bucktown	Bucktown	Marks, Arthur	Arthur Marks	tt0072747
1746578645	1958	1958	Seven Guns to Mesa	Seven Guns to Mesa	Dein, Edward	Edward Dein	tt0050953
1746514277	1916	1916	Diane of the Follies	Diane of the Follies	Cabanne, William Christy	Christy Cabanne	tt0006592
1746577666	1935	1935	The Woman in Red	The Woman in Red	Florey, Robert	Robert Florey	tt0027228
1746520269	1926	1926	The Outsider	The Outsider	Lee, Rowland V.	Rowland V. Lee	tt0017240
1746410821	1994	1994	Double Dragon	Double Dragon	Yukich, James	James Yukich	tt0106761
1746583920	1930	1930	The Bishop Murder Case	The Bishop Murder Case		David Burton	tt0020695
1746437160	1998	1998	Ronin	Ronin	Frankenheimer, John	John Frankenheimer	tt0122690
1746467326	1917	1917	The Terror	The Terror	Wells, Raymond	Raymond Wells	tt0008664
1746533202	1927	1927	Cheaters	Cheaters	Apfel, Oscar	Oscar Apfel	tt0017745
1746550020	1955	1955	The Racers	The Racers	Hathaway, Henry	Henry Hathaway	tt0048531
1746542702	1926	1926	Fort Frayne	Fort Frayne	Wilson, Ben	Ben F. Wilson	tt0132978
1746566239	1957	1957	Crime of Passion	Crime of Passion	Oswald, Gerd	Gerd Oswald	tt0050271
1746565937	1953	1953	Lili	Lili	Walters, Charles	Charles Walters	tt0046000
1746524354	1916	1916	The Book Agent's Romance	The Book Agent's Romance	Anderson, G. M.	Gilbert M. 'Broncho Billy' Anderson	tt0419552
1746500153	1925	1925	The Early Bird	The Early Bird	Hines, Charles	Charles Hines	tt0015773
1746393367	1989	1989	Black Rain	Black Rain	Scott, Ridley	Ridley Scott	tt0096933
1746514385	1916	1916	Joy and the Dragon	Joy and the Dragon	King, Henry	Henry King	tt0006884
1746233141	1967	1967	Surfari	Surfari	Blair, Milton	Milton Blair	tt0134124
1746582692	1935	1935	Circumstantial Evidence	Circumstantial Evidence	Lamont, Charles	Charles Lamont	tt0026213
1746393220	1981	1981	True Confessions	True Confessions	Grossbard, Ulu	Ulu Grosbard	tt0083232
1746577249	1960	1960	The Wild Ride	The Wild Ride	Berman, Harvey	Harvey Berman	tt0054475
1746474398	1944	1944	Hollywood Canteen	Hollywood Canteen	Daves, Delmer	Delmer Daves	tt0036922
1746513868	1914	1914	A Suspicious Wife	A Suspicious Wife	Frank, Alexander F.	Alexander F. Frank	tt0004660
1746566510	1952	1952	Everything I Have Is Yours	Everything I Have Is Yours	Leonard, Robert Z.	Robert Z. Leonard	tt0044599
1746581847	1910	1910	The Fugitive	The Fugitive	Griffith, D. W.	D.W. Griffith	tt0001227
1746584179	1940	1940	Johnny Apollo	Johnny Apollo	Hathaway, Henry	Henry Hathaway	tt0032651
1746483144	1917	1917	Little Miss Nobody	Little Miss Nobody	Millarde, Harry	Harry F. Millarde	tt0008191
1746574537	1935	1935	Sunset Range	Sunset Range	McCarey, Ray	Ray McCarey	tt0027058
1769969547	1911	1911	Jolly Bill of the Rocking R	Jolly Bill of the Rocking R		Allan Dwan	tt0486367
1746433972	1996	1996	The Rock	The Rock	Bay, Michael	Michael Bay	tt0117500
1746476202	1947	1947	Hoppy's Holiday	Hoppy's Holiday	Archainbaud, George	George Archainbaud	tt0039473
1746251488	1977	1977	Smokey and the Bandit	Smokey and the Bandit	Needham, Hal	Hal Needham	tt0076729
1746570748	1933	1933	Little Women	Little Women	Cukor, George	George Cukor	tt0024264
1746437150	1997	1996	Visiting Desire	Visiting Desire	B, Beth	Beth B	tt0118094
1746569685	1956	1956	A Day of Fury	A Day of Fury	Jones, Harmon	Harmon Jones	tt0049126
1746474962	1947	1947	Sweet Genevieve	Sweet Genevieve	Dreifuss, Arthur	Arthur Dreifuss	tt0039876
1746434346	1995	1995	Mr. Holland's Opus	Mr. Holland's Opus	Herek, Stephen	Stephen Herek	tt0113862
1746569620	1958	1958	Curse of the Faceless Man	Curse of the Faceless Man	Cahn, Edward L.	Edward L. Cahn	tt0051507
1746561525	1956	1956	The Black Whip	The Black Whip	Warren, Charles Marquis	Charles Marquis Warren	tt0049015
1769969346	1933	1933	Knee Deep in Music	Knee Deep in Music	Goulding, Alf	Alfred J. Goulding	tt0024227
1746499842	1947	1947	The Foxes of Harrow	The Foxes of Harrow	Stahl, John M.	John M. Stahl	tt0039394
1746532432	1916	1916	The Dream Girl	The Dream Girl	DeMille, Cecil B.	Cecil B. DeMille	tt0006605
1746434283	1996	1996	Hellraiser: Bloodline	Hellraiser: Bloodline	Smithee, Alan	Kevin Yagher	tt0116514
1746499951	1945	1945	Under Western Skies	Under Western Skies	Yarbrough, Jean	Jean Yarbrough	tt0038203
1746513380	1914	1914	Sins of the Parents	Sins of the Parents		Ivan Abramson	tt0004598
1746434634	1999	1999	Summer of Sam	Summer of Sam	Lee, Spike	Spike Lee	tt0162677

1745322670	2006	2006	Babel	Babel	González Iñárritu, Alejandro	Alejandro G. Iñárritu	tt0449467	
1746585260	1929	1929	Gun Law	Gun Law	De Lacey, Robert	Robert De Lacey	tt0019954	
1746421585	1996	1996	Matilda	Matilda	DeVito, Danny	Danny DeVito	tt0117008	
1746416233	1917	1917	The Little Pirate	The Little Pirate	Wilson, Elsie Jane	Elsie Jane Wilson	tt0008195	
1746585010	1939	1939	In Old Caliente	In Old Caliente	Kane, Joseph	Joseph Kane	tt0031478	
1746580657	1938	1938	Three Blind Mice	Three Blind Mice	Seiter, William A.	William A. Seiter	tt0030864	
1746504588	1919	1919	The Gay Old Dog	A Gay Old Dog	Henley, Hobart	Hobart Henley	tt0010158	
1746514250	1916	1916	God's Country and the Woman	God's Country and the Woman	Sturgeon, Rollin S.	Rollin S. Sturgeon	tt0006728	
1746501318	1916	1916	The Red Widow	The Red Widow	Durkin, James	James Durkin	tt0007254	
1746576588	1931	1931	Fifty Fathoms Deep	Fifty Fathoms Deep	Neill, Roy William	Roy William Neill	tt0021858	
1746585089	1929	1929	One Hysterical Night	One Hysterical Night	Craft, William J.	William James Craft	tt0020241	
1746572054	1931	1931	A Holy Terror	A Holy Terror	Cummings, Irving	Irving Cummings	tt0021966	
1746500335	1924	1924	Battling Mason	Battling Mason	Nelson, Jack; Craft, William James	William James Craft	tt0014700	
1746583230	1930	1930	The Melody Man	The Melody Man	Neill, R. William	Roy William Neill	tt0021136	
1746577507	1935	1935	Guard That Girl	Guard That Girl	Hillyer, Lambert	Lambert Hillyer	tt0026443	
1746571327	1934	1934	Uncertain Lady	Uncertain Lady	Freund, Karl	Karl Freund	tt0025932	
1746577543	1937	1937	Park Avenue Logger	Park Avenue Logger	Howard, David	David Howard	tt0029376	
1746491821	1946	1945	Go Down, Death!	Go Down, Death!	Williams, Spencer	Spencer Williams	tt0036870	
1746197928	1968	1968	The Power	The Power	Haskin, Byron	Byron Haskin	tt0063452	
1746583999	1935	1935	Trails of the Wild	Trails of the Wild	Newfield, Sam	Sam Newfield	tt0027130	
1746503559	1918	1918	The Ghosts of Yesterday	The Ghosts of Yesterday	Miller, Charles	Charles Miller	tt0009106	
1746218162	1966	1966	Hawaii	Hawaii	Hill, George Roy	George Roy Hill	tt0060491	
1746550153	1954	1954	Living It Up	Living It Up	Taurog, Norman	Norman Taurog	tt0047184	
1746580558	1935	1935	The Night Is Young	The Night Is Young	Murphy, Dudley	Dudley Murphy	tt0026775	
1746569882	1960	1960	Macumba Love	Macumba Love	Fowley, Douglas V.	Douglas Fowley	tt0054045	
1746511781	1922	1922	Perils of the West	Perils of the West		Patrick Sylvester McGeeney	tt0008422	
1746524818	1922	1922	Hunting Big Game in Africa With Gun and Camer	Hunting Big Game in Africa with Gun and Camera		H.A. Snow	tt1314195	
1746465172	1944	1944	Action in Arabia	Action in Arabia	Moguy, Leonide	Léonide Moguy	tt0036579	
1746577306	1959	1959	The Oregon Trail	The Oregon Trail	Fowler, Gene	Gene Fowler Jr.	tt0053145	
1746499945	1950	1950	Hills of Oklahoma	Hills of Oklahoma	Springsteen, R. G.	R.G. Springsteen	tt0042556	
1745358080	2009	2009	Drag Me to Hell	Drag Me to Hell	Raimi, Sam	Sam Raimi	tt1127180	
1746418840	1940	1940	West of Abilene	West of Abilene	Ceder, Ralph	Ralph Ceder	tt0033249	
1746564751	1953	1953	City Beneath the Sea	City Beneath the Sea	Boetticher, Budd	Budd Boetticher	tt0045630	
1746235850	1974	1974	The Groove Tube	The Groove Tube	Shapiro, Ken	Ken Shapiro	tt0071583	
1746420754	1981	1981	Smokey Bites the Dust	Smokey Bites the Dust	Griffith, Charles B.	Charles B. Griffith	tt0083097	
1746574140	1963	1963	Beauty and the Body	Beauty and the Body	Mart, Paul	Paul Mart	tt0292962	
1746500517	1925	1925	Speed	Speed	Le Saint, Edward J.	Edward LeSaint	tt0016380	
1746583437	1930	1930	Madam Satan	Madam Satan	De Mille, Cecil B.	Cecil B. DeMille	tt0021106	
1746235307	1975	1975	Mahogany	Mahogany	Gordy, Berry	Berry Gordy	tt0073335	
1745342065	2002	2002	Barbershop	Barbershop	Story, Tim	Tim Story	tt0303714	
**1746582784**	**1938**	**1938**	**Call the Mesquiteers**	**Call the Mesquiteers**	**English, John**	**John English**	**tt0029962**	1
1746199746	1968	1968	Brides of Blood	Brides of Blood	Romero, Eddie; de Leon, Gerardo	Gerardo de Leon	tt0062758	
1746433696	1999	1999	Mumford	Mumford	Kasdan, Lawrence	Lawrence Kasdan	tt0140397	
1746554171	1964	1964	Girlsapoppin	Girlsapoppin	Good, Knott	Knott Good	tt0173878	
1746530698	1949	1949	Prison Warden	Prison Warden	Friedman, Seymour	Seymour Friedman	tt0041768	
1746501872	1915	1915	Poor Schmaltz	Poor Schmaltz	Ford, Hugh	Hugh Ford	tt0005911	
1746249656	1975	1975	Bite the Bullet	Bite the Bullet	Brooks, Richard	Richard Brooks	tt0072705	
1746574349	1962	1962	Babes in the Woods	Babes in the Woods		A.A. Krovek	tt0437081	
1746580602	1938	1938	My Lucky Star	My Lucky Star	Del Ruth, Roy	Roy Del Ruth	tt0030481	
1745342531	2010	2010	Inside Job	Inside Job	Ferguson, Charles	Charles Ferguson	tt1645089	
1746520881	1925	1925	Time, the Comedian	Time, the Comedian	Leonard, Robert Z.	Robert Z. Leonard	tt0016435	
1746577584	1937	1937	Boy of the Streets	Boy of the Streets	Nigh, William	William Nigh	tt0029941	
1746581180	1910	1910	His New Lid	His New Lid	Powell, Frank	Frank Powell	tt0001251	
1746520245	1924	1924	Wandering Husbands	Wandering Husbands	Beaudine, William	William Beaudine	tt0015468	
1746561228	1932	1932	Sunset Trail	The Sunset Trail	Eason, B. Reeves	B. Reeves Eason	tt0023539	
1746584376	1937	1937	Ali Baba Goes to Town	Ali Baba Goes to Town	Butler, David	David Butler	tt0028566	
1769970845	1915	1915	The Call of the Sea	The Call of the Sea	Otto, Henry	Frank Montgomery	tt1284611	

1746492867	1944	1944	Hidden Valley Outlaws	Hidden Valley Outlaws	Bretherton, Howard	Howard Bretherton	tt0036918
1746502709	1915	1915	The Battle Cry of Peace	The Battle Cry of Peace	North, Wilfred	J. Stuart Blackton	tt0004942
1746580394	1934	1934	A Man's Game	A Man's Game	Lederman, D. Ross	D. Ross Lederman	tt0025460
1746494129	1910	1910	The Road to Happiness	The Road to Happiness			tt0348736
1746562089	1955	1955	East of Eden	East of Eden	Kazan, Elia	Elia Kazan	tt0048028
1746520416	1926	1925	Typhoon Love	Typhoon Love	Dawn, Norman	Norman Dawn	tt0175269
1746438209	1943	1943	The Man from Down Under	The Man from Down Under	Leonard, Robert Z.	Robert Z. Leonard	tt0031612
1746496067	1917	1917	One Hour	One Hour	McAllister, Paul; Hollywood, Edwin I	Edwin L. Hollywood	tt0008387
1746217208	1966	1966	Birds Do It	Birds Do It	Marton, Andrew	Andrew Marton	tt0060169
1746565213	1952	1952	Hiawatha	Hiawatha	Neumann, Kurt	Kurt Neumann	tt0044705
1746530793	1950	1950	Beyond the Purple Hills	Beyond the Purple Hills	English, John	John English	tt0042244
1746499998	1924	1924	Open All Night	Open All Night	Bern, Paul	Paul Bern	tt0015200
1746584208	1935	1935	One More Spring	One More Spring	King, Henry	Henry King	tt0026822
1746503614	1917	1917	The Spirit of '76	The Spirit of '76	Montgomery, Frank	George Siegmann	tt0182444
1746520953	1950	1950	Killer Shark	Killer Shark	Boetticher, Oscar	Budd Boetticher	tt0042642
1746422322	1940	1940	Ladies Must Live	Ladies Must Live	Smith, Noel	Noel M. Smith	tt0032684
1746474990	1947	1947	The Lone Wolf in London	The Lone Wolf in London	Goodwins, Leslie	Leslie Goodwins	tt0039579
1746169541	1910	1910	Abraham Lincoln's Clemency	Abraham Lincoln's Clemency	Wharton, Theodore	Theodore Wharton	tt0001101
1746582120	1950	1950	Rock Island Trail	Rock Island Trail	Kane, Joseph	Joseph Kane	tt0041816
1746424241	1941	1941	Redhead	Redhead	Cahn, Edward	Edward L. Cahn	tt0034087
1746520211	1949	1949	The Pilgrimage Play	The Pilgrimage Play	Strayer, Frank	Frank R. Strayer	tt0282842
1746582694	1936	1937	Play! Girls	Play! Girls	Graham, Walter	Walter Graham	tt0328264
1746486348	1919	1919	Home	Home	Weber, Lois	Lois Weber	tt0010261
1746509421	1926	1926	The Gay Deceiver	The Gay Deceiver	Stahl, John M.	John M. Stahl	tt0016913
1746515860	1921	1921	Under Western Skies	Under Western Skies	Martin, George	George Martin	tt0175279
1746501865	1910	1910	Settled Out of Court	Settled Out of Court			tt1376449
1746577636	1938	1938	Marie Antoinette	Marie Antoinette	Van Dyke, W. S.	W.S. Van Dyke	tt0030418
1745342058	2004	2004	A Home at the End of the World	A Home at the End of the World	Mayer, Michael	Michael Mayer	tt0359423
1746555598	1956	1956	Beyond a Reasonable Doubt	Beyond a Reasonable Doubt	Lang, Fritz	Fritz Lang	tt0049006
1746410734	1994	1994	Little Big League	Little Big League	Scheinman, Andrew	Andrew Scheinman	tt0110363
1746512756	1914	1914	An Odyssey of the North	An Odyssey of the North	Bosworth, Hobart	Hobart Bosworth	tt0004415
1746580621	1936	1936	Happy Go Lucky	Happy Go Lucky	Scotto, Aubrey	Aubrey Scotto	tt0027718
1746513425	1915	1915	Stop Thief!	Stop Thief!	Fitzmaurice, George	George Fitzmaurice	tt0006104
1746485278	1918	1918	A Petticoat Pilot	A Petticoat Pilot	Sturgeon, Rollin S.	Rollin S. Sturgeon	tt0009492
1746567248	1933	1933	Her Bodyguard	Her Bodyguard	Beaudine, William	William Beaudine	tt0024107
1746582713	1938	1938	Highway Patrol	Highway Patrol	Coleman, C. C.	Charles C. Coleman	tt0030234
1746409692	1991	1991	Popcorn	Popcorn	Herrier, Mark	Mark Herrier	tt0102690
1746465164	1946	1946	If I'm Lucky	If I'm Lucky	Seiler, Lewis	Lewis Seiler	tt0038632
1746503932	1921	1921	Straight Is the Way	Straight Is the Way	Vignola, Robert G.	Robert G. Vignola	tt0012728
1746532997	1915	1915	The Iron Strain	The Iron Strain	Barker, Reginald	Reginald Barker	tt0005553
1745302732	2007	2007	Ocean's Thirteen	Ocean's Thirteen	Soderbergh, Steven	Steven Soderbergh	tt0496806
1746464849	1941	1941	Double Cross	Double Cross	Kelley, Albert	Albert H. Kelley	tt0033547
1746566287	1956	1955	The Cockleshell Heroes	The Cockleshell Heroes	Ferrer, José	José Ferrer	tt0049083
1746503966	1918	1918	Fame and Fortune	Fame and Fortune	Reynolds, Lynn F.	Lynn Reynolds	tt0009053
1746580519	1936	1936	The Return of Jimmy Valentine	The Return of Jimmy Valentine	Collins, Lewis D.	Lewis D. Collins	tt0028171
1746437913	1942	1942	Bullet Scars	Bullet Scars	Lederman, D. Ross	D. Ross Lederman	tt0034555
1745342484	2002	2002	The Scorpion King	The Scorpion King	Russell, Chuck	Chuck Russell	tt0277296
1745272748	2000	2000	Mission: Impossible II	Mission: Impossible II	Woo, John	John Woo	tt0120755
1746582563	1938	1938	There's That Woman Again	There's That Woman Again	Hall, Alexander	Alexander Hall	tt0032017
1746500247	1949	1949	Ma and Pa Kettle	Ma and Pa Kettle	Lamont, Charles	Charles Lamont	tt0041613
1746583687	1930	1930	Hide-Out	Hide Out	Barker, Reginald	Reginald Barker	tt0020971
1746585471	1940	1940	Millionaires in Prison	Millionaires in Prison	McCarey, Ray	Ray McCarey	tt0032796
1746500432	1926	1926	Under Western Skies	Under Western Skies	Sedgwick, Edward	Edward Sedgwick	tt0017497
1746393957	1992	1992	Medicine Man	Medicine Man	McTiernan, John	John McTiernan	tt0104839
1746465000	1942	1942	Jesse James, Jr.	Jesse James, Jr.	Sherman, George	George Sherman	tt0034915
1746454473	1945	1945	Too Young to Know	Too Young to Know	Cordova, Frederick de	Frederick De Cordova	tt0038179
1746503732	1920	1920	The Dream Cheater	The Dream Cheater	Warde, Ernest C.	Ernest C. Warde	tt0011135

1746257428	1976	1975	The Adventures of the Wilderness Family	The Adventures of the Wilderness Family	Raffill, Stewart	Stewart Raffill	tt0072610
1746512920	1927	1926	The General	The General	Keaton, Buster; Bruckman, Clyde	Clyde Bruckman	tt0017925
1746497850	1919	1919	The Hornet's Nest	Hornet's Nest	Young, James	James Young	tt0010270
1746497561	1918	1918	The Forbidden City	The Forbidden City	Franklin, Sidney A.	Sidney Franklin	tt0009086
1746453626	1942	1942	My Heart Belongs to Daddy	My Heart Belongs to Daddy	Siodmak, Robert	Robert Siodmak	tt0035104
1746410176	1992	1992	The Last of the Mohicans	The Last of the Mohicans	Mann, Michael	Michael Mann	tt0104691
1746585147	1929	1929	The Black Watch	The Black Watch	Ford, John	John Ford	tt0019699
1746566156	1957	1957	The Lawless Eighties	The Lawless Eighties	Kane, Joe	Joseph Kane	tt0050627
1745358648	2003	2002	7th Street	7th Street	Pais, Josh	Josh Pais	tt0362390
1746569627	1956	1956	The Mountain	The Mountain	Dmytryk, Edward	Edward Dmytryk	tt0049523
1746577403	1962	1962	If a Man Answers	If a Man Answers	Levin, Henry	Henry Levin	tt0056093
1746453589	1944	1944	The Princess and the Pirate	The Princess and the Pirate	Butler, David	David Butler	tt0037193
1746435584	1943	1943	Girls in Chains	Girls in Chains	Ulmer, Edgar G.	Edgar G. Ulmer	tt0035944
1746508431	1923	1923	His Children's Children	His Children's Children	Wood, Sam	Sam Wood	tt0014134
1746211085	1973	1973	Schlock	Schlock	Landis, John	John Landis	tt0067716
1746234363	1966	1966	Murderers' Row	Murderers' Row	Levin, Henry	Henry Levin	tt0060728
1746555538	1954	1954	The Outlaw Stallion	The Outlaw Stallion	Sears, Fred F.	Fred F. Sears	tt0047321
1746492584	1946	1946	Drifting Along	Drifting Along	Abrahams, Derwin M.	Derwin Abrahams	tt0038496
1746197960	1968	1968	The Road Hustlers	The Road Hustlers	Jackson, Larry E.	Larry E. Jackson	tt0063512
1746523087	1915	1915	Simon, the Jester	Simon, the Jester	José, Edward	Edward José	tt0006048
1746485155	1918	1918	The Romance of Tarzan	The Romance of Tarzan	Lucas, Wilfred	Wilfred Lucas	tt0009560
1746464827	1942	1942	Jacaré	Jacaré	Ford, Charles E.	Charles E. Ford	tt0034908
1746553550	1964	1964	Panic Button	Panic Button	Sherman, George	George Sherman	tt0058446
1746474514	1945	1945	Sing Your Way Home	Sing Your Way Home	Mann, Anthony	Anthony Mann	tt0038091
1746578610	1960	1960	Tormented	Tormented	Gordon, Bert I.	Bert I. Gordon	tt0054393
1746578556	1961	1961	The Police Dog Story	Police Dog Story	Cahn, Edward L.	Edward L. Cahn	tt0055316
1746454084	1942	1942	Trail Riders	Trail Riders	Tansey, Robert	Robert Emmett Tansey	tt0035464
1746503002	1928	1928	Man-Made Women	Man Made Women	Stein, Paul L.	Paul L. Stein	tt0019135
1746576486	1931	1930	The Devil's Cabaret	The Devil's Cabaret	Grindé, Nick	Nick Grinde	tt0022817
1746580633	1938	1938	The Beloved Brat	The Beloved Brat	Lubin, Arthur	Arthur Lubin	tt0029906
1746569507	1953	1953	The Glass Wall	The Glass Wall	Shane, Maxwell	Maxwell Shane	tt0045824
1746515574	1920	1920	Food for Scandal	Food for Scandal	Cruze, James	James Cruze	tt0012180
1746577149	1959	1959	The Last Blitzkrieg	The Last Blitzkrieg	Dreifuss, Arthur	Arthur Dreifuss	tt0051844
1746575017	1936	1936	The First Baby	The First Baby	Seiler, Lewis	Lewis Seiler	tt0027621
1746420151	1987	1987	Prince of Darkness	Prince of Darkness	Carpenter, John	John Carpenter	tt0093777
1746584599	1930	1930	Ingagi	Ingagi		William Campbell	tt0164686
1746555335	1954	1954	Prince Valiant	Prince Valiant	Hathaway, Henry	Henry Hathaway	tt0047365
1746571530	1932	1932	Bird of Paradise	Bird of Paradise	Vidor, King	King Vidor	tt0022689
1746567319	1952	1952	Tarzan's Savage Fury	Tarzan's Savage Fury	Endfield, Cyril	Cy Endfield	tt0045220
1746566761	1933	1933	Her First Mate	Her First Mate	Wyler, William	William Wyler	tt0024109
1746585446	1939	1939	Rio	Rio	Brahm, John	John Brahm	tt0031863
1746584246	1936	1936	And So They Were Married	And So They Were Married	Nugent, Elliott	Elliott Nugent	tt0027293
1746503865	1922	1922	Rounding Up the Law	Rounding Up the Law	Seeling, Charles R.	Charles R. Seeling	tt0013562
1746566142	1956	1956	The Kettles in the Ozarks	The Kettles in the Ozarks	Lamont, Charles	Charles Lamont	tt0049404
1746579054	1963	1963	55 Days at Peking	55 Days at Peking	Ray, Nicholas	Nicholas Ray	tt0056800
1746565994	1953	1953	Man in the Attic	Man in the Attic	Fregonese, Hugo	Hugo Fregonese	tt0047209
1746574710	1936	1936	Navy Born	Navy Born	Watt, Nate	Nate Watt	tt0028023
1746424460	1940	1940	King of the Lumberjacks	King of the Lumberjacks	Clemens, William	William Clemens	tt0032668
1746420092	1994	1994	The Getaway	The Getaway	Donaldson, Roger	Roger Donaldson	tt0109890
1746570865	1934	1934	No Ransom	No Ransom	Newmeyer, Fred	Fred C. Newmeyer	tt0025573
1746574057	1959	1959	Face of a Fugitive	Face of a Fugitive	Wendkos, Paul	Paul Wendkos	tt0052794
1746424387	1943	1943	Hostages	Hostages	Tuttle, Frank	Frank Tuttle	tt0036017
1769970464	1967	1967	Venus in Furs	Venus in Furs	Marzano, Joe	Joseph Marzano	tt0062434
1746581624	1928	1928	Branding Fire	Branding Fire		Ben F. Wilson	tt0301068
1746424587	1940	1940	In Old Missouri	In Old Missouri	McDonald, Frank	Frank McDonald	tt0032629
1746513049	1914	1914	The Tigress	The Tigress		Alice Guy	tt0004706
1746508752	1925	1925	Two Fisted Thompson	Two Fisted Thompson		W. Adcook	tt0174296

1746583501	1928	1928	The Docks of New York	The Docks of New York	Sternberg, Josef von	Josef von Sternberg	tt0018839
1746577943	1937	1937	The Last Gangster	The Last Gangster	Ludwig, Edward	Edward Ludwig	tt0029118
1746408673	1981	1981	Deadly Blessing	Deadly Blessing	Craven, Wes	Wes Craven	tt0082245
1746218860	1978	1978	Hi-Riders	Hi Riders	Clark, Greydon	Greydon Clark	tt0076140
1746524707	1927	1927	The Broadway Drifter	The Broadway Drifter	McEveety, Bernard	Bernard McEveety	tt0017701
1746465413	1944	1944	She's a Sweetheart	She's a Sweetheart	Lord, Del	Del Lord	tt0037269
1746524854	1923	1923	Mary of the Movies	Mary of the Movies	McDermott, John	John McDermott	tt0014262
1746524386	1927	1927	When Danger Calls	When Danger Calls	Hutchison, Charles	Charles Hutchison	tt0018563
1746500296	1925	1925	The Dressmaker From Paris	The Dressmaker from Paris	Bern, Paul	Paul Bern	tt0015765
1746580386	1939	1939	S.O.S.--Tidal Wave	S.O.S. Tidal Wave	Auer, John H.	John H. Auer	tt0031886
1746554116	1964	1964	Bullet for a Badman	Bullet for a Badman	Springsteen, R. G.	R.G. Springsteen	tt0057905
1746575076	1933	1933	The Masquerader	The Masquerader	Wallace, Richard	Richard Wallace	tt0024308
1746500018	1924	1924	Sagebrush Gospel	Sagebrush Gospel	Hatton, Richard	Dick Hatton	tt0159719
1746433719	1999	1999	Friends & Lovers	Friends & Lovers	Haas, George	George Haas	tt0143261
1746570388	1934	1934	A Wicked Woman	A Wicked Woman	Brabin, Charles	Charles Brabin	tt0025989
1746497509	1920	1920	The Soul of Youth	The Soul of Youth	Taylor, William D.	William Desmond Taylor	tt0011721
1746496865	1918	1917	Parentage	Parentage	Henley, Hobart	Hobart Henley	tt0161907
1746582590	1936	1936	The Texas Rangers	The Texas Rangers	Vidor, King	King Vidor	tt0028352
1746474980	1944	1944	Trigger Trail	Trigger Trail	Collins, Lewis D.	Lewis D. Collins	tt0037398
1746516278	1920	1921	Below the Deadline	Below the Deadline	McGowan, J. P.	J.P. McGowan	tt0011959
1746219033	1973	1973	Pat Garrett & Billy the Kid	Pat Garrett & Billy the Kid	Peckinpah, Sam	Sam Peckinpah	tt0070518
1746235233	1973	1973	Sleeper	Sleeper	Allen, Woody	Woody Allen	tt0070707
1745293897	2007	2007	Mr. Untouchable	Mr. Untouchable	Levin, Marc	Marc Levin	tt1086340
1746570243	1934	1934	Let's Talk It Over	Let's Talk It Over	Neumann, Kurt	Kurt Neumann	tt0025154
1746475897	1946	1946	Her Sister's Secret	Her Sister's Secret	Ulmer, Edgar G.	Edgar G. Ulmer	tt0038598
1746542637	1924	1924	Romance of the Wasteland	Romance of the Wasteland		Victor Adamson	tt0015287
1746573892	1958	1958	Fort Massacre	Fort Massacre	Newman, Joseph M.	Joseph M. Newman	tt0051629
1746419790	1990	1990	Jacob's Ladder	Jacob's Ladder	Lyne, Adrian	Adrian Lyne	tt0099871
1746577834	1938	1938	Western Trails	Western Trails	Waggner, George	George Waggner	tt0030961
1746216714	1966	1966	Let's Kill Uncle	Let's Kill Uncle	Castle, William	William Castle	tt0060626
1746574103	1959	1959	Ask Any Girl	Ask Any Girl	Walters, Charles	Charles Walters	tt0052583
2007987181	1922	1922	Silas Marner	Silas Marner	Donovan, Frank P.; Donovan, Frank I	Frank P. Donovan	tt0163234
1746503771	1919	1919	Love and the Law	Love and the Law	Lewis, Edgar	Edgar Lewis	tt0191256
1746585190	1930	1930	The Mounted Stranger	The Mounted Stranger	Rosson, Arthur	Arthur Rosson	tt0021161
1746464810	1943	1943	Tonight We Raid Calais	Tonight We Raid Calais	Brahm, John	John Brahm	tt0036449
1746567025	1932	1932	The Fourth Horseman	The Fourth Horseman	MacFadden, Hamilton	Hamilton MacFadden	tt0022909
1746464795	1944	1944	Meet the People	Meet the People	Riesner, Charles	Charles Reisner	tt0037062
1746420969	1989	1989	Tango & Cash	Tango & Cash	Konchalovsky, Andrei	Andrey Konchalovskiy	tt0098439
1746500116	1948	1948	Night Wind	Night Wind	Tinling, James	James Tinling	tt0040645
1746503524	1921	1921	Sheltered Daughters	Sheltered Daughters	Dillon, Edward	Edward Dillon	tt0012676
1769970440	1966	1966	Male Service	Male Service	Hudson, Arch	Arch Hudson	tt0243401
1746550285	1954	1954	Jivaro	Jivaro	Ludwig, Edward	Edward Ludwig	tt0047133
1746577662	1933	1933	War of the Range	War of the Range	McGowan, J. P.	J.P. McGowan	tt0024752
1746409074	1988	1988	Lady in White	Lady in White	LaLoggia, Frank	Frank LaLoggia	tt0095484
1746574007	1958	1958	Spy in the Sky!	Spy in the Sky!	Wilder, W. Lee	W. Lee Wilder	tt0144620
1746423290	1991	1991	Frankie and Johnny	Frankie and Johnny	Marshall, Garry	Garry Marshall	tt0101912
1746437981	1941	1941	Highway West	Highway West	McGann, William	William C. McGann	tt0033718
1746503291	1919	1919	The Bluffer	The Bluffer	Vale, Travers	Travers Vale	tt0009941
1746562006	1957	1957	The Prince and the Showgirl	The Prince and the Showgirl	Olivier, Laurence	Laurence Olivier	tt0050861
1746497656	1922	1922	Wild Honey	Wild Honey	Ruggles, Wesley	Wesley Ruggles	tt0013766
1746514837	1922	1922	The Game Chicken	A Game Chicken	Franklin, Chester M.	Chester M. Franklin	tt0013162
1746393628	1986	1986	King Kong Lives	King Kong Lives	Guillermin, John	John Guillermin	tt0091344
1746578532	1959	1959	On the Beach	On the Beach	Kramer, Stanley	Stanley Kramer	tt0053137
1746408881	1989	1989	Uncle Buck	Uncle Buck	Hughes, John	John Hughes	tt0098554
1746500250	1924	1924	Wages of Virtue	Wages of Virtue		Allan Dwan	tt0015465
1746520439	1924	1924	Waterfront Wolves	Waterfront Wolves	Gibson, Tom	Tom Gibson	tt0015472
1746580398	1937	1937	Trouble in Texas	Trouble in Texas	Bradbury, R. N.	Robert N. Bradbury	tt0029694

1746508130	1925	1925	The Light of Western Stars	The Light of Western Stars	Howard, William K.	William K. Howard	tt0016021	
1746499872	1948	1948	The Search	The Search	Zinnemann, Fred	Fred Zinnemann	tt0040765	
1746584997	1939	1939	The Man They Could Not Hang	The Man They Could Not Hang	Grinde, Nick	Nick Grinde	tt0031614	
1746437173	1998	1997	Mrs. Dalloway	Mrs Dalloway	Gorris, Marleen	Marleen Gorris	tt0119723	
1746579052	1961	1961	The Little Shepherd of Kingdom Come	The Little Shepherd of Kingdom Come	McLaglen, Andrew V.	Andrew V. McLaglen	tt0055091	
1746423532	1940	1940	Prairie Law	Prairie Law	Howard, David	David Howard	tt0032939	
1746570730	1934	1934	6 Day Bike Rider	6 Day Bike Rider	Bacon, Lloyd	Lloyd Bacon	tt0024809	
1746582521	1935	1935	The Vanishing Riders	The Vanishing Riders	Hill, Bob	Robert F. Hill	tt0027167	
1769976189	1927	1927	Listen Lena	Listen Lena	Beauchamp, Clem	Clem Beauchamp	tt0249734	
1746565926	1955	1955	Guys and Dolls	Guys and Dolls	Mankiewicz, Joseph L.	Joseph L. Mankiewicz	tt0048140	
1746578039	1939	1939	The Cat and the Canary	The Cat and the Canary	Nugent, Elliott	Elliott Nugent	tt0031143	
1746580514	1937	1937	Black Aces	Black Aces	Jones, Buck	Buck Jones	tt0028635	
1746211242	1971	1971	Preacherman	Preacherman	Viola, Albert T.	Albert T. Viola	tt0067606	
1746555596	1954	1954	Garden of Evil	Garden of Evil	Hathaway, Henry	Henry Hathaway	tt0047013	
1769969991	1912	1912	O'Brien's Busy Day	O'Brien's Busy Day		Otis Turner	tt0339462	
1746574922	1937	1936	That Girl from Paris	That Girl from Paris	Jason, Leigh	Leigh Jason	tt0028354	
1746524381	1914	1914	A Florida Enchantment	A Florida Enchantment	Drew, Sidney	Sidney Drew	tt0003973	
1746580203	1935	1935	Storm over the Andes	Storm Over the Andes	Cabanne, Christy	Christy Cabanne	tt0027046	
1746577453	1962	1962	State Fair	State Fair	Ferrer, José	José Ferrer	tt0056526	
1746570816	1934	1934	Sadie McKee	Sadie McKee	Brown, Clarence	Clarence Brown	tt0025740	
1746454758	1997	1997	Dante's Peak	Dante's Peak	Donaldson, Roger	Roger Donaldson	tt0118928	
1746454489	1942	1941	Kathleen	Kathleen	Bucquet, Harold S.	Harold S. Bucquet	tt0033780	
1746497075	1919	1919	When My Ship Comes in	When My Ship Comes In	Thornby, Robert T.	Robert Thornby	tt0135051	
1769976176	1928	1928	Spook Spoofing	Spook Spoofing	McGowan, Robert	Robert F. McGowan	tt0234787	
1746393308	1981	1981	The Prowler	The Prowler	Zito, Joseph	Joseph Zito	tt0082951	
1746217906	1966	1966	You're a Big Boy Now	You're a Big Boy Now	Coppola, Francis Ford	Francis Ford Coppola	tt0061209	
1746503279	1919	1918	The Son-of-A-Gun!	Son of a Gun	Robbins, Jesse J.	F. Richard Jones	tt1341269	
1746553706	1964	1964	Muscle Beach Party	Muscle Beach Party	Asher, William	William Asher	tt0058384	
1745293721	2007	2007	Confessions of a Superhero	Confessions of a Superhero	Ogens, Matt	Matthew Ogens	tt1016164	
1746218330	1965	1965	The Slender Thread	The Slender Thread	Pollack, Sydney	Sydney Pollack	tt0059729	
1746473705	1947	1947	Rainbow Over the Rockies	Rainbow Over the Rockies	Drake, Oliver	Oliver Drake	tt0163171	
1746419795	1987	1987	Programmed to Kill	Programmed to Kill	Holzman, Allan	Allan Holzman	tt0093790	
1746511737	1923	1922	Shifting Sands	Shifting Sands	Granville, Fred Leroy	Fred LeRoy Granville	tt0014462	
1746500407	1926	1926	The Fighting Edge	The Fighting Edge	Lehrman, Henry	Henry Lehrman	tt0016860	
1746578844	1962	1961	The Devil's Messenger	The Devil's Messenger	Strock, Herbert L.	Herbert L. Strock	tt0054807	
1745372622	2002	2002	Star Wars Episode II: Attack of the Clones	Star Wars: Episode II Attack of the Clones	Lucas, George	George Lucas	tt0121765	
1746500678	1927	1928	The Riddle Trail	The Riddle Trail			tt0339584	
1746198923	1967	1967	Woman Times Seven	Woman Times Seven	De Sica, Vittorio	Vittorio De Sica	tt0062502	
1746507837	1949	1949	Massacre River	Massacre River	Rawlins, John	John Rawlins	tt0041643	
1746436210	1992	1992	Unlawful Entry	Unlawful Entry	Kaplan, Jonathan	Jonathan Kaplan	tt0105699	
1746512624	1927	1927	The Irresistible Lover	The Irresistible Lover	Beaudine, William	William Beaudine	tt0018029	
1746433780	1997	1997	Rhyme & Reason	Rhyme & Reason	Spirer, Peter	Peter Spirer	tt0120014	
1746582227	1931	1931	The Squaw Man	The Squaw Man	DeMille, Cecil B.	Cecil B. DeMille	tt0022428	
1746581875	1928	1928	Tracy the Outlaw	Tracy the Outlaw	Thayer, Otis B.	Otis Thayer	tt0171839	
1746216886	1968	1968	Fever Heat	Fever Heat	Doughten, Russell S.	Russell S. Doughten Jr.	tt0061660	
1746497219	1919	1919	The Price Woman Pays	The Price Woman Pays	Terwilliger, George	George Terwilliger	tt0010586	
1746496998	1918	1918	Borrowed Clothes	Borrowed Clothes	Weber, Lois	Lois Weber	tt0008913	
1746584575	1928	1928	The Gate Crasher	The Gate Crasher	Craft, William James	William James Craft	tt0018928	
1746423046	1994	1994	Mary Shelley's Frankenstein	Mary Shelley's Frankenstein	Branagh, Kennth	Kenneth Branagh	tt0109836	
1746585176	1929	1929	Come Across	Come Across	Taylor, Ray	Ray Taylor	tt0019783	
**1746554319**	**1965**	**1965**	**A Swingin' Summer**	**A Swingin' Summer**	**Sparr, Robert**	**Robert Sparr**	**tt0059771**	1
1746574289	1963	1963	Palm Springs Weekend	Palm Springs Weekend	Taurog, Norman	Norman Taurog	tt0057396	
1746501313	1923	1922	Making a Man	Making a Man	Henabery, Joseph	Joseph Henabery	tt0132296	
1746585398	1939	1939	Laugh It Off	Laugh It Off	Rogell, Albert S.	Albert S. Rogell	tt0031555	
1746580807	1935	1935	Red Hot Tires	Red Hot Tires	Lederman, D. Ross	D. Ross Lederman	tt0026917	
1746508208	1927	1927	Three Miles Up	Three Miles Up	Mitchell, Bruce	Bruce Mitchell	tt0018483	
1745282865	2007	2007	Pride	Pride	Gonera, Sunu	Sunu Gonera	tt0475355	

1745395414	2009	2009	Star Trek	Star Trek	Abrams, J. J.	J.J. Abrams	tt0796366
1746438966	1944	1944	Ministry of Fear	Ministry of Fear	Lang, Fritz	Fritz Lang	tt0037075
1746234222	1968	1968	Angels From Hell	Angels from Hell	Kessler, Bruce	Bruce Kessler	tt0062669
1746409993	1987	1987	84 Charing Cross Road	84 Charing Cross Road	Jones, David	David Hugh Jones	tt0090570
1746423020	1993	1993	Manhattan Murder Mystery	Manhattan Murder Mystery	Allen, Woody	Woody Allen	tt0107507
1746520050	1924	1924	Empty Hearts	Empty Hearts	Santell, Al	Alfred Santell	tt0014867
1746566141	1953	1953	The Robe	The Robe	Koster, Henry	Henry Koster	tt0046247
1746574490	1934	1934	The Cat and the Fiddle	The Cat and the Fiddle	Howard, William K.	William K. Howard	tt0024960
1746520191	1926	1926	The Magician	The Magician	Ingram, Rex	Rex Ingram	tt0017103
1746500251	1948	1947	The Pearl	The Pearl	Fernández, Emilio	Emilio Fernández	tt0037981
1746569978	1959	1959	Night of the Quarter Moon	Night of the Quarter Moon	Haas, Hugo	Hugo Haas	tt0053113
1746464900	1946	1945	The Sailor Takes a Wife	The Sailor Takes a Wife	Whorf, Richard	Richard Whorf	tt0038045
1746566083	1958	1958	This Is Russia	This Is Russia!		Sid Feder	tt0051075
1746487946	1918	1918	The Risky Road	The Risky Road	Park, Ida May	Ida May Park	tt0009554
1746524603	1928	1928	Ladies' Night in a Turkish Bath	Ladies' Night in a Turkish Bath	Cline, Edward	Edward F. Cline	tt0019064
1746578781	1963	1963	Thunder Island	Thunder Island	Leewood, Jack	Jack Leewood	tt0057582
1746580729	1936	1936	Boulder Dam	Boulder Dam	McDonald, Frank	Frank McDonald	tt0027391
1746420147	1987	1987	From the Hip	From the Hip	Clark, Bob	Bob Clark	tt0093051
1746577384	1951	1951	Blue Blood	Blue Blood	Landers, Lew	Lew Landers	tt0043349
1746583779	1930	1930	The Cat Creeps	The Cat Creeps	Julian, Rupert	Rupert Julian	tt0020748
1746567811	1932	1932	The All American	The All American	Mack, Russell	Russell Mack	tt0022620
1746580431	1936	1936	Cavalcade of the West	Cavalcade of the West	Fraser, Harry	Harry L. Fraser	tt0027434
1746209474	1968	1968	Ice Station Zebra	Ice Station Zebra	Sturges, John	John Sturges	tt0063121
1746577550	1937	1937	That's My Story	That's My Story!	Salkow, Sidney	Sidney Salkow	tt0029653
1746582819	1937	1937	Alcatraz Island	Alcatraz Island	McGann, William	William C. McGann	tt0028564
1746235642	1974	1973	Badlands	Badlands	Malick, Terrence	Terrence Malick	tt0069762
1746574870	1935	1935	I Live My Life	I Live My Life	Van Dyke, W. S.	W.S. Van Dyke	tt0026417
1746507604	1926	1926	A Horse on Broadway	A Horse on Broadway	Mattison, Frank S.		tt0356702
1746455778	1997	1997	Murder at 1600	Murder at 1600	Little, Dwight	Dwight H. Little	tt0119731
1746514406	1916	1916	The Kid	The Kid	North, Wilfrid	Wilfrid North	tt0163036
1746487577	1919	1919	The Profiteers	The Profiteer	Fitzmaurice, George	John K. Holbrook	tt0010594
1745358685	2002	2002	The Kid Stays in the Picture	The Kid Stays in the Picture	Morgen, Brett ; Burstein, Nanette	Nanette Burstein	tt0303353
1746584350	1939	1939	Stunt Pilot	Stunt Pilot	Waggner, George	George Waggner	tt0031989
1746465231	1942	1942	Crossroads	Crossroads	Conway, Jack	Jack Conway	tt0034622
1746524315	1916	1916	Broken Fetters	Broken Fetters	Ingram, Rex	Rex Ingram	tt0006470
1746454301	1943	1943	Slightly Dangerous	Slightly Dangerous	Ruggles, Wesley	Wesley Ruggles	tt0036365
1746424436	1943	1943	Old Acquaintance	Old Acquaintance	Sherman, Vincent	Vincent Sherman	tt0036230
1746561088	1932	1932	The Kid from Spain	The Kid from Spain	McCarey, Leo	Leo McCarey	tt0023088
1746424205	1941	1941	Charley's Aunt	Charley's Aunt	Mayo, Archie; Rosenberg, Aaron	Archie Mayo	tt0033457
1745358978	2008	2008	Rambo	Rambo	Stallone, Sylvester	Sylvester Stallone	tt0462499
1746436350	1943	1943	Hi Diddle Diddle	Hi Diddle Diddle	Stone, Andrew L.	Andrew L. Stone	tt0035996
1746524503	1915	1915	A Texas Steer	A Texas Steer	Warren, Giles R.	Giles Warren	tt0006144
1746577984	1937	1937	Hold 'Em Navy!	Hold 'Em Navy	Neumann, Kurt	Kurt Neumann	tt0029008
1746578784	1963	1963	Violent Midnight	Violent Midnight	Hilliard, Richard	Richard Hilliard	tt0057434
1746504164	1921	1921	Jesse James as the Outlaw	Jesse James as the Outlaw	Coates, Franklin B.	Franklin B. Coates	tt0135482
1746501410	1910	1910	Young Deer's Return	Young Deer's Return		Fred J. Balshofer	tt0360222
1745357937	2002	2002	High Crimes	High Crimes	Franklin, Carl	Carl Franklin	tt0257756
1746584554	1928	1928	Crashing Through	Crashing Through	Buckingham, Thomas	Tom Buckingham	tt0018802
1746475257	1947	1947	Blondie's Anniversary	Blondie's Anniversary	Berlin, Abby	Abby Berlin	tt0039201
1746515565	1917	1917	Stagestruck	Stage Struck	Morrisey, Edward	Edward Morrissey	tt0138832
1746585243	1929	1929	River of Romance	The River of Romance	Wallace, Richard	Richard Wallace	tt0020334
1746500373	1927	1927	Slightly Used	Slightly Used	Mayo, Archie L.	Archie Mayo	tt0018414
1746438439	1942	1942	Wrecking Crew	Wrecking Crew	MacDonald, Frank	Frank McDonald	tt0035569
1746584804	1928	1928	Arizona Days	Arizona Days	McGowan, J. P.	J.P. McGowan	tt0018654
1746584753	1928	1928	Freedom of the Press	Freedom of the Press	Melford, George	George Melford	tt0018917
1746476194	1944	1944	Andy Hardy's Blonde Trouble	Andy Hardy's Blonde Trouble	Seitz, George B.	George B. Seitz	tt0036602
1746433755	1997	1997	Critical Care	Critical Care	Lumet, Sidney	Sidney Lumet	tt0118901

1746502212	1917	1917	Hell Morgan's Girl	Tell Morgan's Girl	De Grasse, Joseph	Louis Chaudet	tt0332771	
1746581347	1910	1910	The Further Adventures of the Girl Spy	The Further Adventures of the Girl Spy	Olcott, Sidney	Sidney Olcott	tt0397435	
1746581940	1951	1951	Footlight Varieties	Footlight Varieties	Yates, Hal	D.W. Griffith	tt0043551	
1746423442	1941	1941	Double Trouble	Double Trouble	West, William	William West	tt0033549	
1746491794	1948	1948	Saigon	Saigon	Fenton, Leslie	Leslie Fenton	tt0040753	
1746465362	1947	1947	Road to Rio	Road to Rio	McLeod, Norman Z.	Norman Z. McLeod	tt0039776	
1769969817	1912	1912	The Letter with the Black Seals	The Letter with the Black Seals		Étienne Arnaud	tt0002304	
1745374182	2002	2002	Drumline	Drumline	Stone , Charles	Charles Stone III	tt0303933	
1746509193	1925	1925	Starlight, the Untamed	Starlight, the Untamed	Webb, Harry	Harry S. Webb	tt0166821	
1746473902	1942	1942	Boston Blackie Goes Hollywood	Boston Blackie Goes Hollywood	Gordon, Michael	Michael Gordon	tt0034545	
1746409030	1987	1987	Raising Arizona	Raising Arizona	Coen, Joel	Joel Coen	tt0093822	
1746219604	1973	1973	The Laughing Policeman	The Laughing Policeman	Rosenberg, Stuart	Stuart Rosenberg	tt0070292	
1746497074	1918	1918	Treasure of the Sea	The Treasure of the Sea	Reicher, Frank	Frank Reicher	tt0009722	
1746560662	1932	1932	This Reckless Age	This Reckless Age	Tuttle, Frank	Frank Tuttle	tt0023380	
1745373533	2002	2001	The Believer	The Believer	Bean, Henry	Henry Bean	tt0247199	
1746485173	1918	1917	Sauce for the Goose	Sauce for the Goose	Edwards, Walter	Al Christie	tt0759682	
1746580559	1937	1937	This Is My Affair	This Is My Affair	Seiter, William A.	William A. Seiter	tt0029662	
1746582869	1935	1935	The Silent Code	The Silent Code	Paton, Stuart	Stuart Paton	tt0240897	
1746578032	1938	1938	Youth Takes a Fling	Youth Takes a Fling	Mayo, Archie	Archie Mayo	tt0031003	
1746524886	1916	1916	Miss George Washington	Miss George Washington	Dawley, J. Searle	J. Searle Dawley	tt0007074	
1746465402	1947	1947	The Adventures of Don Coyote	The Adventures of Don Coyote	LeBorg, Reginald	Reginald Le Borg	tt0039134	
1746577652	1935	1935	Frisco Kid	Frisco Kid	Bacon, Lloyd	Lloyd Bacon	tt0026385	
1746509577	1924	1924	Bluff	Bluff	Wood, Sam	Sam Wood	tt0014725	
1745309516	2007	2007	The Final Season	The Final Season	Evans, David Mickey	David Mickey Evans	tt0449018	
1746486174	1919	1919	Mind the Paint Girl	The Mind the Paint Girl	North, Wilfrid	Wilfrid North	tt0010464	
1746465048	1947	1947	New Orleans	New Orleans	Lubin, Arthur	Arthur Lubin	tt0039655	
1769970463	1966	1966	The House of Cats	The House of Cats		Arch Hudson	tt0220539	
1746508892	1926	1926	Sin Cargo	Sin Cargo	Gasnier, Louis J.	Louis J. Gasnier	tt0017395	
1745373505	2008	2008	Street Kings	Street Kings	Ayer, David	David Ayer	tt0421073	
1746507582	1949	1949	Arctic Fury	Arctic Fury	Dawn, Norman; Feitshans, Fred R.	Norman Dawn	tt0041131	
1746410852	1994	1994	Clear and Present Danger	Clear and Present Danger	Noyce, Phillip	Phillip Noyce	tt0109444	
1746582753	1937	1937	Varsity Show	Varsity Show	Keighley, William	William Keighley	tt0029725	
1746566278	1953	1953	Prisoners of the Casbah	Prisoners of the Casbah	Bare, Richard	Richard L. Bare	tt0046209	
1746437133	2000	2000	Men of Honor	Men of Honor	Tillman, George	George Tillman Jr.	tt0203019	
1745340492	2003	2003	Final Destination 2	Final Destination 2	Ellis, David R.	David R. Ellis	tt0309593	
1746573645	1931	1931	Sporting Blood	Sporting Blood	Brabin, Charles	Charles Brabin	tt0022425	
1746523473	1923	1923	Shell Shocked Sammy	Shell Shocked Sammy	Mattison, Frank S.	Frank S. Mattison	tt0163829	
1769970018	1911	1911	Falsely Accused	Falsely Accused		Thomas H. Ince	tt0358469	
1746197966	1966	1966	How to Steal a Million	How to Steal a Million	Wyler, William	William Wyler	tt0060522	
1746569826	1960	1960	The Little Shop of Horrors	The Little Shop of Horrors	Corman, Roger	Roger Corman	tt0054033	
1746409702	1986	1986	Under the Cherry Moon	Under the Cherry Moon	Prince	Prince	tt0092133	
1746560871	1932	1932	Sinister Hands	Sinister Hands	Schaefer, Armand	Armand Schaefer	tt0023478	
1746585449	1940	1940	Jennie	Jennie	Burton, David	David Burton	tt0032648	
1746584460	1930	1930	Sweet Mama	Sweet Mama	Cline, Edward	Edward F. Cline	tt0021440	
1746422529	1998	1998	Patch Adams	Patch Adams	Shadyac, Tom	Tom Shadyac	tt0129290	
1746501325	1916	1916	The Crucial Test	The Crucial Test	Thornby, Robert; Ince, John	John Ince	tt0006558	
1746198460	1968	1968	How To Save a Marriage--And Ruin Your Life	How to Save a Marriage and Ruin Your Life	Cook, Fielder	Fielder Cook	tt0063102	
1746567987	1933	1933	The Mysterious Rider	The Mysterious Rider	Allen, Fred	Fred Allen	tt0024366	
1746567775	1933	1933	It's Great to Be Alive	It's Great to Be Alive	Werker, Alfred	Alfred L. Werker	tt0024189	
**1769969371**	**1965**	**1965**	**The Secret Society**	**The Secret Society**	**Johnsen, S. N.**	**Sande N. Johnsen**	**tt0138084**	1
1746508391	1925	1925	The Knockout Kid	The Knockout Kid	Rogell, Albert	Albert S. Rogell	tt0126372	
1746465275	1944	1944	The Seventh Cross	The Seventh Cross	Zinnemann, Fred	Fred Zinnemann	tt0037263	
1745342187	2002	2002	About Schmidt	About Schmidt	Payne, Alexander	Alexander Payne	tt0257360	
1746560459	1932	1932	Lena Rivers	Lena Rivers	Rosen, Phil	Phil Rosen	tt0023128	
1746570829	1933	1933	The Dude Bandit	The Dude Bandit	Melford, George	George Melford	tt0023970	
1746235390	1980	1980	Ordinary People	Ordinary People	Redford, Robert	Robert Redford	tt0081283	
1746250818	1977	1977	Valentino	Valentino	Russell, Ken	Ken Russell	tt0076868	

1746465420	1946	1946	Ding Dong Williams	Ding Dong Williams	Berke, William	William Berke	tt0038479
1746514424	1915	1915	Life Without Soul	Life Without Soul	Smiley, Joseph W.	Joseph W. Smiley	tt0005615
1746474508	1947	1947	The Pilgrim Lady	The Pilgrim Lady	Selander, Lesley	Lesley Selander	tt0039707
1746571830	1931	1931	The Secret Call	The Secret Call	Walker, Stuart	Stuart Walker	tt0022355
1746513180	1923	1923	The White Sister	The White Sister	King, Henry	Henry King	tt0014605
1746217967	1968	1968	The Legend of Lylah Clare	The Legend of Lylah Clare	Aldrich, Robert	Robert Aldrich	tt0063219
1746573954	1960	1960	This Rebel Breed	This Rebel Breed	Bare, Richard L.	Richard L. Bare	tt0054381
1746514230	1915	1915	The Primrose Path	The Primrose Path	Marston, Theodore	Lawrence Marston	tt0005927
1746574944	1936	1936	Come Closer, Folks	Come Closer, Folks	Lederman, D. Ross	D. Ross Lederman	tt0027458
1746560534	1932	1932	Swing High	Swing High	Cummings, Jack	Jack Cummings	tt0152771
1746572276	1910	1910	The District Attorney's Triumph	The District Attorney's Triumph			tt0348588
1746584930	1940	1940	You Can't Fool Your Wife	You Can't Fool Your Wife	McCarey, Ray	Ray McCarey	tt0033279
1746573921	1956	1956	A Kiss Before Dying	A Kiss Before Dying	Oswald, Gerd	Gerd Oswald	tt0049414
1746465108	1942	1942	Shadows on the Sage	Shadows on the Sage	Orlebeck, Les	Lester Orlebeck	tt0035311
1746555172	1955	1954	The Green Buddha	The Green Buddha	Lemont, John	John Lemont	tt0048135
1746217860	1967	1967	Gunfight in Abilene	Gunfight in Abilene	Hale, William	William Hale	tt0061738
1746577318	1959	1959	The Hangman	The Hangman	Curtiz, Michael	Michael Curtiz	tt0052877
1746575891	1931	1931	Beyond Victory	Beyond Victory	Robertson, John	John S. Robertson	tt0021656
1746569812	1956	1956	Lum and Abner Abroad	Lum and Abner Abroad	Kern, James V.	James V. Kern	tt0049454
1746572041	1931	1931	Delicious	Delicious	Butler, David	David Butler	tt0021793
1746567320	1934	1934	The Lucky Texan	The Lucky Texan	Bradbury, Robert N.	Robert N. Bradbury	tt0025440
1746584389	1936	1936	Don't Get Personal	Don't Get Personal	Nigh, William	Charles Lamont	tt0027535
1745309186	2005	2005	Ellie Parker	Ellie Parker	Coffey, Scott	Scott Coffey	tt0436262
1746516476	1920	1920	The Law of the Yukon	The Law of the Yukon	Miller, Charles	Charles Miller	tt0011390
1746219344	1976	1976	The Next Man	The Next Man	Sarafian, Richard C.	Richard C. Sarafian	tt0074962
1746410177	1992	1992	Passenger 57	Passenger 57	Hooks, Kevin	Kevin Hooks	tt0105104
1746499840	1945	1945	Pillow to Post	Pillow to Post	Sherman, Vincent	Vincent Sherman	tt0037990
1745302257	2007	2007	Girl 27	Girl 27	Stenn, David	David Stenn	tt0912586
1746581708	1910	1910	How Rastus Gets His Turkey	How Rastus Gets His Turkey		Theodore Wharton	tt0135462
1746578725	1958	1957	All Mine to Give	All Mine to Give	Reisner, Allen	Allen Reisner	tt0050112
1746419977	1993	1993	Needful Things	Needful Things	Heston, Fraser C.	Fraser C. Heston	tt0107665
1746523550	1914	1914	Paid in Full	Paid in Full	Thomas, Augustus	Augustus E. Thomas	tt0004451
1746498342	1917	1917	Sunshine and Gold	Sunshine and Gold	King, Henry	Henry King	tt0008644
1746533047	1915	1915	A Man's Prerogative	A Man's Prerogative	Nichols, George	George Nichols	tt0005711
1746509282	1924	1924	Wanderer of the Wasteland	Wanderer of the Wasteland	Willat, Irvin	Irvin Willat	tt0015467
1769970597	1968	1969	The Love Clinic	The Love Clinic	Sebastian, Ferdinand	Ferd Sebastian	tt0241684
1746408842	1990	1990	Roger Corman's Frankenstein Unbound	Roger Corman's Frankenstein Unbound	Corman, Roger	Roger Corman	tt0099612
1746580375	1936	1936	California Mail	California Mail	Smith, Noel	Noel M. Smith	tt0027414
1746574752	1934	1934	Thunder over Texas	Thunder Over Texas	Warner [Edgar G. Ulmer], John	Edgar G. Ulmer	tt0025891
1746210774	1972	1972	Portnoy's Complaint	Portnoy's Complaint	Lehman, Ernest	Ernest Lehman	tt0069112
1746574323	1961	1961	Parrish	Parrish	Daves, Delmer	Delmer Daves	tt0055279
1746566103	1958	1958	Invisible Avenger	Invisible Avenger	Howe, James Wong; Sledge, John	James Wong Howe	tt0051783
1746585374	1939	1939	Water Rustlers	Water Rustlers	Diege, Samuel	Samuel Diege	tt0032112
1746495602	1917	1917	The Argyle Case	The Argyle Case	Ince, Ralph W.	Ralph Ince	tt0007649
1746497852	1918	1918	The Magic Eye	The Magic Eye	Berger, Rea	Rae Berger	tt0009332
1746524059	1913	1913	The Girl of the Sunny South	The Girl of the Sunny South		Travers Vale	tt0002901
1746483903	1917	1917	Sylvia of the Secret Service	Sylvia of the Secret Service	Fitzmaurice, George	George Fitzmaurice	tt0145508
1746566842	1933	1933	The Silk Express	The Silk Express	Enright, Ray	Ray Enright	tt0024559
1746571005	1933	1933	The Circus Queen Murder	The Circus Queen Murder	Neill, Roy William	Roy William Neill	tt0023893
1745293365	2007	2007	Joshua	Joshua	Ratliff, George	George Ratliff	tt0808331
1746568022	1933	1933	Employee's Entrance	Employees' Entrance	Del Ruth, Roy	Roy Del Ruth	tt0023986
1746500668	1926	1926	Ten Nights in a Barroom	Ten Nights in a Barroom		Roy Calnek	tt0139641
1746496579	1921	1921	The Plaything of Broadway	The Plaything of Broadway	Dillon, Jack	John Francis Dillon	tt0012573
1746235100	1976	1976	Obsession	Obsession	De Palma, Brian	Brian De Palma	tt0074991
1746576864	1932	1932	Riders of the Desert	Riders of the Desert	Bradbury, Robert N.	Robert N. Bradbury	tt0023399
1746562449	1957	1957	Interlude	Interlude	Sirk, Douglas	Douglas Sirk	tt0050544
1746500317	1925	1925	Playing With Souls	Playing with Souls	Ince, Ralph	Ralph Ince	tt0016228

1746583435	1929	1929	Navy Blues	Navy Blues	Brown, Clarence	Clarence Brown	tt0020210	
1746455309	1999	1999	trick	Dick	Fall, Jim	Andrew Fleming	tt0144168	
1746520610	1949	1949	The Set-Up	The Set Up	Wise, Robert	Robert Wise	tt0041859	
1746573211	1932	1931	Prestige	Prestige	Garnett, Tay	Tay Garnett	tt0023352	
1746272119	1966	1965	Sting of Death	Sting of Death	Grefé, William	William Grefé	tt0059756	
1746420843	1940	1940	Village Barn Dance	Village Barn Dance	McDonald, Frank	Frank McDonald	tt0033223	
1746513428	1915	1915	The Secret Orchard	The Secret Orchard	Reicher, Frank	Frank Reicher	tt0006017	
1745341829	2001	2001	How High	How High	Dylan, Jesse	Jesse Dylan	tt0278488	
1746577573	1937	1937	She Loved a Fireman	She Loved a Fireman	Farrow, John	John Farrow	tt0030739	
1746475762	1948	1948	Casbah	Casbah	Berry, John	John Berry	tt0040214	
1746566282	1954	1953	Beat the Devil	Beat the Devil	Huston, John	John Huston	tt0046414	
1746520690	1949	1949	Alias the Champ	Alias the Champ	Blair, George	George Blair	tt0041108	
1746420139	1983	1984	Almonds and Raisins	Almonds and Raisins	Karel, Russ	David Elstein	tt0086875	
1746502038	1916	1916	Her Debt of Honor	Her Debt of Honor	Nigh, William	William Nigh	tt0006787	
1769972678	1963	1963	Hollywood Nudes Report	Hollywood Nudes Report	Mahon, Barry	Barry Mahon	tt0291246	
1746501750	1910	1910	The Woman in the Case	A Woman in the Case		William F. Haddock	tt2239170	
1746581351	1910	1910	Her Terrible Peril	Her Terrible Peril	Balshofer, Fred J.	Fred J. Balshofer	tt0358513	
1746435864	1995	1995	Boys On the Side	Boys on the Side	Ross, Herbert	Herbert Ross	tt0112571	
1746515066	1921	1921	The Blazing Trail	The Blazing Trail	Thornby, Robert	Robert Thornby	tt0011977	
1746582715	1936	1936	Rose of the Rancho	Rose of the Rancho	Gering, Marion	Marion Gering	tt0028208	
1746454474	1943	1943	Bombardier	Bombardier	Wallace, Richard	Richard Wallace	tt0035689	
1745374752	2010	2010	Ticked-Off Trannies with Knives	Ticked Off Trannies with Knives	Luna, Israel	Israel Luna	tt1587431	
1745358984	2009	2009	Orphan	Orphan	Collet-Serra, Jaume	Jaume Collet	tt1148204	
1746569975	1958	1958	Man of the West	Man of the West	Mann, Anthony	Anthony Mann	tt0051899	
1746492220	1948	1948	Million Dollar Weekend	Million Dollar Weekend	Raymond, Gene	Gene Raymond	tt0040590	
1746577682	1935	1935	Cappy Ricks Returns	Cappy Ricks Returns	Wright, Mack	Mack V. Wright	tt0026173	
1746492561	1945	1945	Wagon Wheels Westward	Wagon Wheels Westward	Springsteen, R. G.	R.G. Springsteen	tt0038234	
1746209436	1968	1968	Mondo Daytona	Mondo Daytona	Willard, Frank	Frank Willard	tt0188910	
1746475936	1948	1948	Jungle Goddess	Jungle Goddess	Collins, Lewis D.	Lewis D. Collins	tt0040500	
1746514422	1922	1922	A Homespun Vamp	A Homespun Vamp	O'Connor, Frank	Frank O'Connor	tt0013246	
1746572452	1932	1932	Red Dust	Red Dust	Fleming, Victor	Victor Fleming	tt0023382	
1746577283	1950	1950	Square Dance Katy	Square Dance Katy	Yarbrough, Jean	Jean Yarbrough	tt0042992	
1746488018	1920	1920	The Unseen Witness	The Unseen Witness	Collins, Tom	Tom Collins	tt0188265	
1746393769	1982	1982	The Thing	The Thing	Carpenter, John	John Carpenter	tt0084787	
1746578432	1959	1959	The Story on Page One	The Story on Page One	Odets, Clifford	Clifford Odets	tt0053308	
**1746577985**	**1937**	**1937**	**Two Wise Maids**	**Two Wise Maids**	**Rosen, Phil**	**Phil Rosen**	**tt0029702**	1
1746516546	1920	1920	The Fourteenth Man	The Fourteenth Man	Henabery, Joseph	Joseph Henabery	tt0011207	
1746491857	1947	1947	Pirates of Monterey	Pirates of Monterey	Werker, Alfred	Alfred L. Werker	tt0039709	
1746423343	1991	1991	For the Boys	For the Boys	Rydell, Mark	Mark Rydell	tt0101902	
1746435154	1940	1940	Lightning Strikes West	Lightning Strikes West	Fraser, Harry	Harry L. Fraser	tt0032707	
1745342617	2003	2003	Prey for Rock and Roll	Prey for Rock & Roll	Steyermark, Alex	Alex Steyermark	tt0307351	
1746561605	1932	1932	Riding for Justice	Ridin' for Justice	Lederman, D. Ross	D. Ross Lederman	tt0023401	
1746487908	1921	1921	Moonlight Follies	Moonlight Follies	Baggot, King	King Baggot	tt0012472	
1746524201	1910	1910	Under Both Flags	Under Both Flags	Young Deer, James	James Young Deer	tt1225769	
1746258464	1978	1978	Eyes of Laura Mars	Eyes of Laura Mars	Kershner, Irvin	Irvin Kershner	tt0077530	
1746584900	1940	1940	Hi-Yo Silver	Hi Yo Silver	Witney, William; English, John	John English	tt0032590	
1746577653	1933	1933	Gabriel over the White House	Gabriel Over the White House	LaCava, Gregory	Gregory La Cava	tt0024044	
1746570392	1933	1933	The Nuisance	The Nuisance	Conway, Jack	Jack Conway	tt0024396	
1746513703	1927	1927	A Hero on Horseback	A Hero on Horseback	Andrews, Del	Del Andrews	tt0017985	
1746574708	1933	1933	Elmer, the Great	Elmer, the Great	LeRoy, Mervyn	Mervyn LeRoy	tt0023982	
1746504096	1922	1922	The Three Buckaroos	The Three Buckaroos	Balshofer, Fred J.	Fred J. Balshofer	tt0170677	
1746583389	1930	1930	Captain of the Guard	Captain of the Guard	Robertson, John S.; Fejos, Paul	John S. Robertson	tt0020741	
1746473942	1942	1942	To Be or Not to Be	To Be or Not to Be	Lubitsch, Ernst	Ernst Lubitsch	tt0035446	
1746582758	1935	1935	Red Blood of Courage	The Red Blood of Courage	English, Jack	John English	tt0026916	
1746436133	1941	1941	Pot O' Gold	Pot o' Gold	Marshall, George	George Marshall	tt0034055	
1746475532	1947	1947	Border Feud	Border Feud	Taylor, Ray	Ray Taylor	tt0039210	
1746474976	1947	1947	Valley of Fear	Valley of Fear	Hillyer, Lambert	Lambert Hillyer	tt0039950	

1746433562	1989	1989	Phantom of the Mall: Eric's Revenge	Phantom of the Mall: Eric's Revenge	Friedman, Richard	Richard Friedman	tt0098089
1746514257	1923	1923	The Town Scandal	The Town Scandal	Baggot, King	King Baggot	tt0014556
1746556010	1954	1954	The Bounty Hunter	The Bounty Hunter	DeToth, Andre	André De Toth	tt0046801
1746218707	1974	1974	The Great Gatsby	The Great Gatsby	Clayton, Jack	Jack Clayton	tt0071577
1746520264	1948	1948	Shep Comes Home	Shep Comes Home	Beebe, Ford	Ford Beebe	tt0038935
1746512379	1915	1915	The Man Who Found Himself	The Man Who Found Himself	Crane, Frank H.	Frank Hall Crane	tt0005696
1746513382	1910	1910	The Vicar of Wakefield	The Vicar of Wakefield		Theodore Marston	tt0409435
1746582257	1930	1930	The Danger Man	The Danger Man	Pollard, Bud	Charles Hutchison	tt0020807
1745293348	2006	2005	Harsh Times	Harsh Times	Ayer, David	David Ayer	tt0433387
1746217058	1966	1966	Dead Heat on a Merry-Go-Round	Dead Heat on a Merry Go Round	Girard, Bernard	Bernard Girard	tt0060287
1746217033	1967	1967	The Way West	The Way West	McLaglen, Andrew V.	Andrew V. McLaglen	tt0062479
1746574903	1935	1935	Escapade	Escapade	Leonard, Robert Z.	Robert Z. Leonard	tt0026319
1746584806	1928	1928	The Yellow Lily	Yellow Lily	Korda, Alexander	Alexander Korda	tt0019603
1746258155	1978	1978	The Driver	The Driver	Hill, Walter	Walter Hill	tt0077474
1746508042	1926	1926	The Texas Streak	The Texas Streak	Reynolds, Lynn	Lynn Reynolds	tt0017455
1746576918	1958	1958	Rock-a-Bye Baby	Rock a Bye Baby	Tashlin, Frank	Frank Tashlin	tt0052141
1746523441	1916	1915	The Beast	The Beast	Stanton, Richard	Edgar Jones	tt0415626
1746501028	1917	1917	Fighting Mad	Fighting Mad	Le Saint, Edward J.	Edward LeSaint	tt0007943
1746422265	1994	1994	Reality Bites	Reality Bites	Stiller, Ben	Ben Stiller	tt0110950
1746582593	1935	1935	Men Without Names	Men Without Names	Murphy, Ralph	Ralph Murphy	tt0026699
1745342933	2002	2002	OT: Our Town	OT: Our Town	Kennedy, Scott Hamilton	Scott Hamilton Kennedy	tt0334029
1746502058	1915	1915	The Moonstone	The Moonstone	Crane, Frank	Frank Hall Crane	tt0005769
1746574653	1935	1935	The Dawn Rider	The Dawn Rider	Bradbury, R. N.	Robert N. Bradbury	tt0026267
1746573899	1963	1963	Gunfight at Comanche Creek	Gunfight at Comanche Creek	McDonald, Frank	Frank McDonald	tt0058165
1746424606	1943	1943	So Proudly We Hail!	So Proudly We Hail!	Sandrich, Mark	Mark Sandrich	tt0036367
1746520594	1925	1925	The Goose Woman	The Goose Woman	Brown, Clarence	Clarence Brown	tt0015872
1746464928	1942	1942	Eagle Squadron	Eagle Squadron	Lubin, Arthur	Arthur Lubin	tt0034691
1746500527	1924	1924	Wanted by the Law	Wanted by the Law	Bradbury, Robert N.	Robert N. Bradbury	tt0015470
1746437407	1941	1941	Road to Happiness	Road to Happiness	Rosen, Phil	Phil Rosen	tt0035261
1746573812	1957	1957	Hell Canyon Outlaws	Hell Canyon Outlaws	Landres, Paul	Paul Landres	tt0050496
1746474516	1948	1948	The Golden Eye	The Golden Eye	Beaudine, William	William Beaudine	tt0040394
1746219577	1980	1980	Don't Answer the Phone	Don't Answer the Phone!	Hammer, Robert	Robert Hammer	tt0080645
1746582705	1937	1937	Personal Property	Personal Property	Van Dyke, W. S.	W.S. Van Dyke	tt0029395
1746271952	1980	1980	The Gong Show Movie	The Gong Show Movie	Barris, Chuck	Chuck Barris	tt0080808
1746520150	1925	1925	The New Commandment	The New Commandment	Higgin, Howard	Howard Higgin	tt0016153
1746198198	1966	1966	Girl on a Chain Gang	Girl on a Chain Gang	Gross, Jerry	Jerry Gross	tt0059226
1746454109	1998	1998	Simon Birch	Simon Birch	Johnson, Mark Steven	Mark Steven Johnson	tt0124879
1746465122	1947	1947	Range Beyond the Blue	Range Beyond the Blue	Taylor, Ray	Ray Taylor	tt0039751
1746464816	1942	1942	Nightmare	Nightmare	Whelan, Tim	Tim Whelan	tt0035127
1746580331	1935	1935	Frontier Justice	Frontier Justice	McGowan, Robert	Robert F. McGowan	tt0027647
1746219071	1971	1971	The Gang That Couldn't Shoot Straight	The Gang That Couldn't Shoot Straight	Goldstone, James	James Goldstone	tt0067124
1746571675	1931	1931	Ladies' Man	Ladies' Man	Mendes, Lothar	Lothar Mendes	tt0022043
1746434258	1991	1991	Boyz N the Hood	Boyz n the Hood	Singleton, John	John Singleton	tt0101507
1746419618	1981	1981	Modern Romance	Modern Romance	Brooks, Albert	Albert Brooks	tt0082764
1746573705	1910	1910	The Early Settlers	The Early Settlers			tt1390938
1746554522	1964	1964	Handle With Care	Handle with Care	McCarthy, John K.	John K. McCarthy	tt0218319
1746433725	1993	1993	Addams Family Values	Addams Family Values	Sonnenfeld, Barry	Barry Sonnenfeld	tt0106220
1746578408	1959	1959	Some Like It Hot	Some Like It Hot	Wilder, Billy	Billy Wilder	tt0053291
1746561079	1932	1932	A Bill of Divorcement	A Bill of Divorcement	Cukor, George	George Cukor	tt0022685
1746575010	1935	1935	The Gilded Lily	The Gilded Lily	Ruggles, Wesley	Wesley Ruggles	tt0026409
1746570068	1952	1952	The World in His Arms	The World in His Arms	Walsh, Raoul	Raoul Walsh	tt0045339
1746584042	1940	1940	American Gang Busters	The American Gang Busters	Dion, A. F.	A.F. Dion	tt0189349
1746513636	1923	1923	The Darling of New York	The Darling of New York	Baggot, King	King Baggot	tt0013975
1746569706	1958	1958	I Accuse!	I Accuse!	Ferrer, José	José Ferrer	tt0051754
1746465423	1946	1946	Dangerous Millions	Dangerous Millions	Tinling, James	James Tinling	tt0038448
1746491856	1943	1943	Unknown Guest	The Unknown Guest	Neumann, Kurt	Kurt Neumann	tt0036480
1746563977	1951	1950	Frenchie	Frenchie	King, Louis	Louis King	tt0042482

1746503792	1920	1920	The Amateur Wife	The Amateur Wife	Dillon, Edward	Edward Dillon	tt0184200
1769970447	1967	1967	Marked for Love	Marked for Love	Clay, Adam	Adam Clay	tt0251227
1746504781	1917	1917	The Secret of Black Mountain	The Secret of Black Mountain	Hoffman, Otto	Otto Hoffman	tt0008552
1746198119	1968	1968	Bye Bye Braverman	Bye Bye Braverman	Lumet, Sidney	Sidney Lumet	tt0062768
1746464841	1940	1940	La Conga Nights	La Conga Nights	Landers, Lew	Lew Landers	tt0032682
1746234445	1965	1965	The Flight of the Phoenix	The Flight of the Phoenix	Aldrich, Robert	Robert Aldrich	tt0059183
1746581969	1910	1910	In the Great Northwest	In the Great Northwest		Francis Boggs	tt0352434
1746483842	1917	1917	The Spotted Lily	The Spotted Lily	Solter, Harry	Harry Solter	tt0008621
1746569564	1954	1954	Varietease	Varietease	Klaw, Irving	Irving Klaw	tt0047643
1769971194	1969	1969	Alimony Lovers	Alimony Lovers	Perkins, Harold	John Hayes	tt0154127
1746570401	1952	1952	Jungle Jim in the Forbidden Land	Jungle Jim in the Forbidden Land	Landers, Lew	Lew Landers	tt0044777
1746583862	1929	1929	The Careless Age	The Careless Age	Wray, John Griffith	John Griffith Wray	tt0019752
1769973192	1970	1970	Big Beaver	Big Beaver	Lavender, Semore	Semore Lavender	tt0211250
1746198131	1969	1969	Hang Your Hat on the Wind	Hang Your Hat on the Wind	Lansburgh, Larry	Larry Lansburgh	tt0218321
1746570740	1952	1952	Thunderbirds	Thunderbirds	Auer, John H.	John H. Auer	tt0045237
1746582154	1951	1951	Dear Brat	Dear Brat	Seiter, William	William A. Seiter	tt0043457
1746496058	1918	1918	Smashing Through	Smashing Through	Clifton, Elmer	Elmer Clifton	tt0009624
1746475244	1947	1947	The Return of Rin Tin Tin	The Return of Rin Tin Tin	Nosseck, Max	Max Nosseck	tt0039763
1746465245	1947	1947	Slave Girl	Slave Girl	Lamont, Charles	Charles Lamont	tt0039836
1746274940	1979	1979	Escape from Alcatraz	Escape from Alcatraz	Siegel, Donald	Don Siegel	tt0079116
1746235814	1978	1978	Movie Movie	Movie Movie	Donen, Stanley	Stanley Donen	tt0077952
1746467929	1917	1917	Beloved Jim	Beloved Jim	Paton, Stuart	Stuart Paton	tt0007695
1746491834	1948	1948	Money Madness	Money Madness	Stewart, Peter	Sam Newfield	tt0040604
1746584355	1940	1940	Pride of the Bowery	Pride of the Bowery	Lewis, Joseph H.	Joseph H. Lewis	tt0034064
1746520538	1927	1927	Rich Men's Sons	Rich Men's Sons	Graves, Ralph	Ralph Graves	tt0018323
1746554125	1964	1964	My Fair Lady	My Fair Lady	Cukor, George	George Cukor	tt0058385
1745293398	2006	2006	The Shaggy Dog	The Shaggy Dog	Robbins, Brian	Brian Robbins	tt0393735
1746251222	1976	1976	Embryo	Embryo	Nelson, Ralph	Ralph Nelson	tt0074475
1746501630	1915	1915	The Alster Case	The Alster Case	Haydon, J. Charles	J. Charles Haydon	tt0004884
1769970473	1967	1967	Come Ride the Wild Pink Horse	Come Ride the Wild Pink Horse	Sarno, Joe	Joseph W. Sarno	tt0060252
1746574060	1957	1957	From Hell It Came	From Hell It Came	Milner, Dan	Dan Milner	tt0050414
1746454835	1944	1944	Hey, Rookie	Hey, Rookie	Barton, Charles	Charles Barton	tt0036915
1746508970	1925	1925	Private Affairs	Private Affairs	Hoffman, Renaud	Renaud Hoffman	tt0016247
1746456046	1997	1997	All Over Me	All Over Me	Sichel, Alex	Alex Sichel	tt0118586
1746514706	1927	1927	Topsy and Eva	Topsy and Eva	Lord, Del	Del Lord	tt0173340
1746219365	1980	1980	One-Trick Pony	One Trick Pony	Young, Robert M.	Robert M. Young	tt0081280
1746542631	1925	1925	The Great Love	The Great Love	Neilan, Marshall	Marshall Neilan	tt0015878
1746454706	1995	1995	Houseguest	Houseguest	Miller, Randall	Randall Miller	tt0110066
1746574661	1933	1933	Scarlet River	Scarlet River	Brower, Otto	Otto Brower	tt0024524
1746532629	1915	1915	The Bridge of Sighs	The Bridge of Sighs		Bertram Bracken	tt0421814
1746573114	1931	1931	Lariats and Six Shooters	Lariats and Six Shooters	Neitz, Alvin J.	Alan James	tt0022052
1746562366	1955	1955	Treasure of Ruby Hills	Treasure of Ruby Hills	McDonald, Frank	Frank McDonald	tt0048745
1746566094	1954	1954	Playgirl	Playgirl	Pevney, Joseph	Joseph Pevney	tt0047355
1746567696	1952	1952	Laramie Mountains	Laramie Mountains	Nazarro, Ray	Ray Nazarro	tt0044824
1746465199	1942	1942	A Close Call for Ellery Queen	A Close Call for Ellery Queen	Hogan, James	James P. Hogan	tt0034602
1746503962	1922	1922	Love Is an Awful Thing	Love Is an Awful Thing	Heerman, Victor	Victor Heerman	tt0013334
1746581720	1951	1951	I Was an American Spy	I Was an American Spy	Selander, Lesley	Lesley Selander	tt0043666
1746507590	1924	1924	Love's Whirlpool	Love's Whirlpool	Mitchell, Bruce	Bruce Mitchell	tt0015089
1746581157	1910	1910	Go West, Young Woman, Go West	Go West, Young Woman, Go West			tt0345339
1769975751	1918	1918	Cave Man Stuff	Cave Man Stuff	Curtis, Allen	Allen Curtis	tt0328935
1746486774	1918	1918	Hungry Eyes	Hungry Eyes	Julian, Rupert	Rupert Julian	tt0009213
1746584936	1940	1940	Parole Fixer	Parole Fixer	Florey, Robert	Robert Florey	tt0032890
1746423176	1940	1940	Captain Caution	Captain Caution	Wallace, Richard	Richard Wallace	tt0032304
1746583804	1930	1930	The Doorway to Hell	The Doorway to Hell	Mayo, Archie	Archie Mayo	tt0020836
1769973298	1970	1971	Room 11	Room 11	Irwin, Bud	Bud Irwin	tt0257053
1769970460	1967	1968	Hot Erotic Dreams	Hot Erotic Dreams	Shuman, Mort	Mort Shuman	tt0220529
1746217522	1966	1966	Fireball 500	Fireball 500	Asher, William	William Asher	tt0060415

1746585364	1939	1939	Pirates of the Skies	Pirates of the Skies	McDonough, Joseph A.	Joseph A. McDonough	tt0031801
1746584403	1940	1940	Li'l Abner	Li'l Abner	Rogell, Albert S.	Albert S. Rogell	tt0032703
1746562462	1955	1955	The Phenix City Story	The Phenix City Story	Karlson, Phil	Phil Karlson	tt0048488
1746583503	1951	1951	Starlift	Starlift	Del Ruth, Roy	Roy Del Ruth	tt0044070
1746578819	1962	1962	Two Weeks in Another Town	Two Weeks in Another Town	Minnelli, Vincente	Vincente Minnelli	tt0056625
1746492708	1948	1948	Station West	Station West	Lanfield, Sidney	Sidney Lanfield	tt0040835
1746409963	1983	1983	Twilight Zone: The Movie	Twilight Zone: The Movie		Joe Dante	tt0086491
1746583237	1929	1929	The Ghost Talks	The Ghost Talks	Seiler, Lewis	Lewis Seiler	tt0019923
1746421069	1999	1999	The Out-of-Towners	The Out of Towners	Weisman, Sam	Sam Weisman	tt0129280
1746515575	1918	1918	The Widow's Might	Widow's Might	de Mille, William C.		tt0437563
1746249224	1975	1975	The Apple Dumpling Gang	The Apple Dumpling Gang	Tokar, Norman	Norman Tokar	tt0072653
1746562436	1955	1955	Jail Busters	Jail Busters	Beaudine, William	William Beaudine	tt0048222
1746433693	1996	1996	Paradise Lost: The Child Murders at Robin Hood	Paradise Lost: The Child Murders at Robin H	Berlinger, Joe ; Sinofsky, Bruce	Joe Berlinger	tt0117293
1745340034	2004	2004	13 Going on 30	13 Going on 30	Winick, Gary	Gary Winick	tt0337563
1746508049	1925	1925	Passionate Youth	Passionate Youth	Fitzgerald, Dallas M.	Dallas M. Fitzgerald	tt0016204
1746234564	1966	1966	The Navy vs. the Night Monsters	The Navy vs. the Night Monsters	Hoey, Michael A.	Michael A. Hoey	tt0060741
1746421075	1992	1992	Buffy the Vampire Slayer	Buffy the Vampire Slayer	Kuzui, Fran Rubel	Fran Rubel Kuzui	tt0103893
1746569722	1953	1953	Son of Belle Starr	Son of Belle Starr	McDonald, Frank	Frank McDonald	tt0046346
1746584536	1929	1929	The Glad Rag Doll	Glad Rag Doll	Curtiz, Michael	Michael Curtiz	tt0019932
1746583325	1931	1931	Five Star Final	Five Star Final	LeRoy, Mervyn	Mervyn LeRoy	tt0021873
1746273824	1980	1980	Private Benjamin	Private Benjamin	Zieff, Howard	Howard Zieff	tt0081375
1746454316	1996	1996	Sunchaser	The Sunchaser	Cimino, Michael	Michael Cimino	tt0117781
1746409036	1988	1988	School Daze	School Daze	Lee, Spike	Spike Lee	tt0096054
1746492866	1948	1948	Tap Roots	Tap Roots	Marshall, George	George Marshall	tt0040861
1746578785	1960	1960	The Music Box Kid	The Music Box Kid	Cahn, Edward L.	Edward L. Cahn	tt0054103
1746564271	1951	1951	Fort Defiance	Fort Defiance	Rawlins, John	John Rawlins	tt0043554
1746520104	1925	1925	Satan in Sables	Satan in Sables	Flood, James	James Flood	tt0016313
1746454961	1945	1944	House of Frankenstein	House of Frankenstein	Kenton, Erle C.	Erle C. Kenton	tt0036931
1746573746	1957	1957	The Shadow on the Window	The Shadow on the Window	Asher, William	William Asher	tt0049748
1746492585	1944	1944	Passage to Marseille	Passage to Marseille	Curtiz, Michael	Michael Curtiz	tt0037166
1746523929	1915	1915	A Little Brother of the Rich	A Little Brother of the Rich	Turner, Otis	Otis Turner	tt0005624
1746566058	1955	1955	Dial Red O	Dial Red O	Ullman, Daniel B.	Daniel B. Ullman	tt0047993
1746520395	1925	1925	The Night Club	The Night Club	Urson, Frank; Iribe, Paul	Paul Iribe	tt0016156
1746524027	1923	1923	Red Lights	Red Lights	Badger, Clarence G.	Clarence G. Badger	tt0014398
1746585153	1928	1928	The Garden of Eden	The Garden of Eden	Milestone, Lewis	Lewis Milestone	tt0018927
1746502715	1915	1915	A Bunch of Keys	A Bunch of Keys	Baker, Richard Foster	Richard Foster Baker	tt0005036
1745374462	2009	2009	Nine	Nine	Marshall, Rob	Rob Marshall	tt0875034
1746495881	1918	1918	Caught in the Act	Caught in the Act	Millarde, Harry	Harry F. Millarde	tt0008953
1746583979	1939	1939	Streets of New York	Streets of New York	Nigh, William; Mintz, Jack	William Nigh	tt0031987
1746581615	1930	1930	The Arizona Kid	The Arizona Kid	Santell, Alfred	Alfred Santell	tt0020649
1746524486	1910	1910	A Rose of the Philippines	A Rose of the Philippines			tt1889362
1746497894	1917	1917	The Man Who Forgot	The Man Who Forgot	Chautard, Emile	Emile Chautard	tt0008255
1746577143	1961	1961	Ring of Fire	Ring of Fire	Stone, Andrew L.	Andrew L. Stone	tt0055374
1746500481	1924	1924	Ace of the Law	Ace of the Law	King, Lewis	Louis King	tt0220959
1746218597	1972	1972	Northeast of Seoul	Northeast of Seoul	Rich, David Lowell	David Lowell Rich	tt0069022
1746501113	1915	1915	The Running Fight	The Running Fight	Durkin, James	James Durkin	tt0005990
1746465016	1942	1942	Random Harvest	Random Harvest	LeRoy, Mervyn	Mervyn LeRoy	tt0035238
1746474833	1945	1945	Sheriff of Cimarron	Sheriff of Cimarron	Canutt, Yakima	Yakima Canutt	tt0038085
1746577704	1935	1935	Arizona Badman	Arizona Bad Man	Luby, S. Roy	S. Roy Luby	tt0026080
1746508956	1950	1950	Captain Carey, U.S.A.	Captain Carey, U.S.A.	Leisen, Mitchell	Mitchell Leisen	tt0042310
1769970535	1968	1968	Two Girls for a Madman	Two Girls for a Madman	Brasloff, Stanley H.	Stanley H. Brassloff	tt0062620
1746198128	1969	1969	Topaz	Topaz	Hitchcock, Alfred	Alfred Hitchcock	tt0065112
1746465248	1946	1946	The Mysterious Mr. Valentine	The Mysterious Mr. Valentine	Ford, Philip	Philip Ford	tt0038768
1746561324	1957	1957	That Night!	That Night!	Newland, John	John Newland	tt0051070
1746577082	1961	1961	Mad Dog Coll	Mad Dog Coll	Balaban, Burt	Burt Balaban	tt0055118
1769970582	1966	1966	Hot Nights on the Campus	Hot Nights on the Campus	Orlando, Tony	Tony Orlando	tt0125171
1746533161	1915	1915	The Sins of Society	The Sins of Society	Eagle, Oscar	Oscar Eagle	tt0006051

1746578979	1963	1963	Beach Party	Beach Party	Asher, William	William Asher	tt0056860
1746420230	1988	1988	Funny Farm	Funny Farm	Hill, George Roy	George Roy Hill	tt0095188
1746572419	1910	1910	The Cowboy's Stratagem	The Cowboy's Stratagem			tt0345082
1746554158	1963	1963	Donovan's Reef	Donovan's Reef	Ford, John	John Ford	tt0057007
1746508754	1925	1925	Daring Days	Daring Days	O'Brien, John B.	John B. O'Brien	tt0015734
1746408431	1985	1985	Creator	Creator	Passer, Ivan	Ivan Passer	tt0088960
1746580443	1937	1937	Forlorn River	Forlorn River	Barton, Charles	Charles Barton	tt0028890
1746574586	1937	1937	Springtime in the Rockies	Springtime in the Rockies	Kane, Joe	Joseph Kane	tt0029602
1746420861	1994	1994	True Lies	True Lies	Cameron, James	James Cameron	tt0111503
1746421577	1981	1981	The Decline of Western Civilization	The Decline of Western Civilization	Spheeris, Penelope	Penelope Spheeris	tt0082252
1746581346	1961	1961	Sanctuary	Sanctuary	Richardson, Tony	Tony Richardson	tt0055402
1746496245	1918	1918	$5,000 Reward	$5,000 Reward	Gerrard, Douglas	Douglas Gerrard	tt0008808
1746513427	1923	1923	Mr. Billings Spends His Dime	Mr. Billings Spends His Dime	Ruggles, Wesley	Wesley Ruggles	tt0014296
1746520754	1925	1925	The Fool	The Fool	Millarde, Harry	Harry F. Millarde	tt0015833
1745272129	2000	2000	Spin the Bottle	Spin the Bottle	Yerkes, Jamie	Jamie Yerkes	tt0131596
1746504136	1918	1918	Lend Me Your Name	Lend Me Your Name	Balshofer, Fred J.	Fred J. Balshofer	tt0009285
1746434932	1998	1998	Wild Things	Wild Things	McNaughton, John	John McNaughton	tt0120890
1746580438	1938	1938	Trade Winds	Trade Winds	Garnett, Tay	Tay Garnett	tt0030888
1746580917	1939	1939	The Man from Sundown	The Man from Sundown	Nelson, Sam	Sam Nelson	tt0031613
1746569718	1958	1958	Quantrill's Raiders	Quantrill's Raiders	Bernds, Edward	Edward Bernds	tt0052103
1746438160	1944	1944	Men on Her Mind	Men on Her Mind	Fox, Wallace W.	Wallace Fox	tt0037067
1746393741	1985	1985	Rappin'	Rappin'	Silberg, Joel	Joel Silberg	tt0089883
1745293270	2006	2006	A Prairie Home Companion	A Prairie Home Companion	Altman, Robert	Robert Altman	tt0420087
1746202534	1970	1970	Promise at Dawn	Promise at Dawn	Dassin, Jules	Jules Dassin	tt0066251
1746454704	1946	1946	Murder in the Music Hall	Murder in the Music Hall	English, John	John English	tt0038758
1746208633	1968	1968	The Swimmer	The Swimmer	Perry, Frank	Frank Perry	tt0063663
1746583029	1937	1937	Dangerously Yours	Dangerously Yours	St. Clair, Mal	Malcolm St. Clair	tt0028765
1746580335	1939	1939	Fugitive at Large	Fugitive at Large	Collins, Lewis D.	Lewis D. Collins	tt0031352
1746584890	1939	1939	Tropic Fury	Tropic Fury	Cabanne, Christy	Christy Cabanne	tt0032057
1746574891	1937	1937	Racing Lady	Racing Lady	Fox, Wallace	Wallace Fox	tt0028153
1746520586	1950	1950	The Flame and the Arrow	The Flame and the Arrow	Tourneur, Jacques	Jacques Tourneur	tt0042464
1746257987	1978	1978	The Fury	The Fury	De Palma, Brian	Brian De Palma	tt0077588
1745340897	2002	2002	Unfaithful	Unfaithful	Lyne, Adrian	Adrian Lyne	tt0250797
1746269507	1980	1980	The Children	The Children	Kalmanowicz, Max	Max Kalmanowicz	tt0080527
1746421849	1940	1940	Wild Horse Valley	Wild Horse Valley	Webb, Ira	Ira Webb	tt0033262
1746542748	1925	1925	The Awful Truth	The Awful Truth	Powell, Paul	Paul Powell	tt0015589
1746491846	1944	1944	Lifeboat	Lifeboat	Hitchcock, Alfred	Alfred Hitchcock	tt0037017
1746555064	1955	1955	Cinerama Holiday	Cinerama Holiday	Bendick, Robert; De Lacy, Philippe	Robert L. Bendick	tt0047939
1746198060	1969	1969	Where It's At	Where It's At	Kanin, Garson	Garson Kanin	tt0065208
1746569658	1955	1955	The Naked Dawn	The Naked Dawn	Ulmer, Edgar G.	Edgar G. Ulmer	tt0048411
1746524318	1923	1923	Crossed Wires	Crossed Wires	Baggot, King	King Baggot	tt0013960
1769974259	1916	1916	Going Up	Going Up	Myll, Louis	Milton J. Fahrney	tt0460139
1746584274	1940	1940	Earl of Puddlestone	Earl of Puddlestone	Meins, Gus	Gus Meins	tt0032425
1746584334	1939	1939	Susannah of the Mounties	Susannah of the Mounties	Seiter, William A.; Lang, Walter	William A. Seiter	tt0031995
1746394202	1991	1990	Superstar: The Life and Times of Andy Warhol	Superstar: The Life and Times of Andy Warho	Workman, Chuck	Chuck Workman	tt0103008
1746577504	1937	1937	Murder in Greenwich Village	Murder in Greenwich Village	Rogell, Albert S.	Albert S. Rogell	tt0029274
1746453835	1941	1941	Buy Me That Town	Buy Me That Town	Forde, Eugene	Eugene Forde	tt0033442
1746566258	1953	1953	Last of the Comanches	Last of the Comanches	DeToth, Andre	André De Toth	tt0044828
1746574975	1935	1935	Public Opinion	Public Opinion	Strayer, Frank R.	Frank R. Strayer	tt0026898
1746520076	1925	1925	Go West	Go West	Keaton, Buster	Buster Keaton	tt0015863
1746496050	1918	1918	Men	Men	Vekroff, Perry N.	Perry N. Vekroff	tt0186343
1746491737	1946	1946	Badman's Territory	Badman's Territory	Whelan, Tim	Tim Whelan	tt0038321
1746532456	1916	1916	Arms and the Woman	Arms and the Woman	Fitzmaurice, George	George Fitzmaurice	tt0006371
1746211013	1971	1971	Play Misty for Me	Play Misty for Me	Eastwood, Clint	Clint Eastwood	tt0067588
1746574668	1936	1936	Little Miss Nobody	Little Miss Nobody	Blystone, John	John G. Blystone	tt0027894
1746217333	1966	1966	The Gentle Rain	The Gentle Rain	Balaban, Burt	Burt Balaban	tt0060452
1746465425	1945	1945	Captain Kidd	Captain Kidd	Lee, Rowland V.	Rowland V. Lee	tt0037576

1746584591	1930	1929	Happy Days	Happy Days	Stoloff, Benjamin	Benjamin Stoloff	tt0020949
1746578920	1958	1958	The Light in the Forest	The Light in the Forest	Daugherty, Herschel	Herschel Daugherty	tt0051864
1746512767	1910	1910	Saved from the Redmen	Saved from the Redmen		Fred J. Balshofer	tt0359939
1746584474	1928	1928	We Americans	We Americans	Sloman, Edward	Edward Sloman	tt0019555
1746581229	1910	1910	Her Indian Mother	Her Indian Mother		Sidney Olcott	tt0195776
1746198691	1969	1969	Big Daddy	Big Daddy	Hittleman, Carl K.	Carl K. Hittleman	tt0058968
1746475944	1948	1948	Texas, Brooklyn & Heaven	Texas, Brooklyn & Heaven	Castle, William	William Castle	tt0040868
1745309238	2007	2007	Firehouse Dog	Firehouse Dog	Holland, Todd	Todd Holland	tt0476995
1746520620	1926	1926	Fools of Fashion	Fools of Fashion	McKay, James C.	James C. McKay	tt0016892
1746514412	1927	1927	Where Trails Begin	Where Trails Begin	Smith, Noel Mason	Noel M. Smith	tt0179513
1746584335	1936	1936	We're in the Legion Now	We're in the Legion Now	Wilbur, Crane	Crane Wilbur	tt0028170
1746516325	1921	1921	The Fighting Breed	The Fighting Breed	Lucas, Wilfred	Wilfred Lucas	tt0012163
1746524152	1913	1913	Shadows of the Moulin Rouge	Shadows of the Moulin Rouge	Blaché, Alice	Alice Guy	tt0003365
1746208439	1969	1969	The Secret of Santa Vittoria	The Secret of Santa Vittoria	Kramer, Stanley	Stanley Kramer	tt0064952
1746520460	1927	1927	The Understanding Heart	The Understanding Heart	Conway, Jack	Jack Conway	tt0018525
1746394391	1992	1992	Straight Talk	Straight Talk	Kellman, Barnet	Barnet Kellman	tt0105481
1746509331	1924	1924	The Rejected Woman	The Rejected Woman	Parker, Albert	Albert Parker	tt0015270
1746495875	1921	1921	Partners of the Tide	Partners of the Tide	Jefferson, L. V.	L.V. Jefferson	tt0012553
1745357698	2010	2010	Dahmer vs. Gacy	Dahmer vs. Gacy	Austin, Ford	Ford Austin	tt1335986
1769970388	1964	1964	SINderella and the Golden Bra	Sinderella and the Golden Bra	Minardi, Loel	Loel Minardi	tt0058590
1746484769	1919	1919	Her Game	Her Game	Crane, Frank H.	Frank Hall Crane	tt0010240
1746581796	1929	1929	Is Everybody Happy?	Is Everybody Happy?	Mayo, Archie L.	Archie Mayo	tt0020031
1746581820	1929	1929	Show Boat	Show Boat	Pollard, Harry	Harry A. Pollard	tt0020402
1746581465	1928	1928	San Francisco Nights	San Francisco Nights	Neill, R. William	Roy William Neill	tt0019351
1746500337	1927	1927	The Rambling Ranger	The Rambling Ranger	Henderson, Del	Dell Henderson	tt0018303
1746516072	1919	1919	His Debt	His Debt	Worthington, William	William Worthington	tt0205128
1746454827	1946	1946	Gun Town	Gun Town	Fox, Wallace W.	Wallace Fox	tt0038579
1746520263	1949	1948	She's Too Mean for Me	She's Too Mean for Me			tt0135648
1746583405	1930	1930	Going Wild	Going Wild	Seiter, William A.	William A. Seiter	tt0020924
1769970796	1912	1912	Under Mexican Skies	Under Mexican Skies	Anderson, G. M.	Gilbert M. 'Broncho Billy' Anderson	tt0376290
1746569906	1958	1958	The Long, Hot Summer	The Long, Hot Summer	Ritt, Martin	Martin Ritt	tt0051878
1746574220	1961	1960	The Wackiest Ship in the Army?	The Wackiest Ship in the Army	Murphy, Richard	Richard Murphy	tt0054453
1746555931	1956	1956	The Benny Goodman Story	The Benny Goodman Story	Davies, Valentine	Valentine Davies	tt0047873
1746503058	1917	1917	Modern Mother Goose	A Modern Mother Goose	Betz, Matthew von	Matthew Betz	tt0008308
1746584989	1940	1940	Queen of the Yukon	Queen of the Yukon	Rosen, Phil	Phil Rosen	tt0032959
1746261333	1981	1981	All Night Long	All Night Long	Tramont, Jean-Claude	Jean	tt0082001
1746408830	1989	1989	Ministry of Vengeance	Ministry of Vengeance	Maris, Peter	Peter Maris	tt0097887
1746578941	1958	1958	Screaming Mimi	Screaming Mimi	Oswald, Gerd	Gerd Oswald	tt0052168
1746500911	1910	1910	The Sheriff's Sacrifice	The Sheriff's Sacrifice		Gilbert M. 'Broncho Billy' Anderson	tt0001394
1746573927	1962	1962	The Miracle Worker	The Miracle Worker	Penn, Arthur	Arthur Penn	tt0056241
1745271823	2000	2000	Trois	Trois	Hardy, Rob	Rob Hardy	tt0217107
1745271780	2000	2000	The Skulls	The Skulls	Cohen, Rob	Rob Cohen	tt0192614
1746569501	1953	1953	The Silver Whip	The Silver Whip	Jones, Harmon	Harmon Jones	tt0046317
1746435325	1992	1992	Stop! Or My Mom Will Shoot	Stop! Or My Mom Will Shoot	Spottiswoode, Roger	Roger Spottiswoode	tt0105477
1746434340	1996	1996	Down Periscope	Down Periscope	Ward, David S.	David S. Ward	tt0116130
1746498270	1920	1920	The Scoffer	The Scoffer	Dwan, Allan	Allan Dwan	tt0012654
1746438302	1944	1944	Timber Queen	Timber Queen	McDonald, Frank	Frank McDonald	tt0036440
1746515053	1910	1910	The Tenderfoot Messenger	The Tenderfoot Messenger		Gilbert M. 'Broncho Billy' Anderson	tt0001412
1746578020	1939	1939	Babes in Arms	Babes in Arms	Berkeley, Busby	Busby Berkeley	tt0031066
1746524145	1927	1927	The Broncho Buster	The Broncho Buster	Laemmle, Ernst	Ernst Laemmle	tt0017710
1746580465	1936	1936	The Moon's Our Home	The Moon's Our Home	Seiter, William A.	William A. Seiter	tt0027979
1746493451	1914	1914	The $5,000,000 Counterfeiting Plot	The $5,000,000 Counterfeiting Plot	Harrison, Bertram	Bertram Harrison	tt0003584
1746508022	1927	1927	The Ladybird	The Ladybird	Lang, Walter	Walter Lang	tt0018075
1746574451	1933	1933	Should Ladies Behave	Should Ladies Behave	Beaumont, Harry	Harry Beaumont	tt0024553
1746507751	1925	1925	Bustin' Thru	Bustin' Thru	Smith, Clifford S.	Clifford Smith	tt0015656
1746210246	1971	1971	The Omega Man	The Omega Man	Sagal, Boris	Boris Sagal	tt0067525
1746585420	1939	1939	Dancing Co-Ed	Dancing Co Ed	Simon, S. Sylvan	S. Sylvan Simon	tt0031203

1746509239	1949	1949	Challenge to Lassie	Challenge to Lassie	Thorpe, Richard	Richard Thorpe	tt0041238
1746571000	1934	1934	The Merry Widow	The Merry Widow	Lubitsch, Ernst	Ernst Lubitsch	tt0025493
1746217091	1966	1966	Deux Voix	Deux Voix	Stevenson, Rosalind A.	Rosalind Stevenson	tt4666168
1746573844	1956	1956	Hidden Guns	Hidden Guns	Gannaway, Al	Albert C. Gannaway	tt0049311
1746491731	1946	1946	The Strange Woman	The Strange Woman	Ulmer, Edgar	Edgar G. Ulmer	tt0038990
1769969465	1911	1911	The Imposter	The Imposter	Henderson, Lucius J.	Lucius Henderson	tt0408904
1746555127	1955	1954	Green Fire	Green Fire	Marton, Andrew	Andrew Marton	tt0047050
1746567793	1952	1952	Mara Maru	Mara Maru	Douglas, Gordon	Gordon Douglas	tt0044884
1746486467	1921	1921	Skirts	Skirts	Del Ruth, Hampton	Hampton Del Ruth	tt0012698
1746486280	1921	1921	Why Girls Leave Home	Why Girls Leave Home	Nigh, William	William Nigh	tt0012846
1746581211	1961	1961	Capture That Capsule!	Capture That Capsule	Zens, Will	Will Zens	tt0054723
1746436376	1940	1940	Half a Sinner	Half a Sinner	Christie, Al	Al Christie	tt0032566
1746577195	1951	1951	Flame of Stamboul	Flame of Stamboul	Nazzaro, Ray	Ray Nazarro	tt0043543
1746235751	1978	1978	Coma	Coma	Crichton, Michael	Michael Crichton	tt0077355
1746467791	1917	1917	Paws of the Bear	Paws of the Bear	Barker, Reginald	Reginald Barker	tt0008415
1746578030	1939	1939	Hotel Imperial	Hotel Imperial	Florey, Robert	Robert Florey	tt0031445
1746492221	1948	1948	I Remember Mama	I Remember Mama	Stevens, George	George Stevens	tt0040458
1746465176	1941	1941	Smilin' Through	Smilin' Through	Borzage, Frank	Frank Borzage	tt0034203
1746508129	1925	1925	The Price of Pleasure	The Price of Pleasure	Sloman, Edward	Edward Sloman	tt0016242
1746584952	1940	1940	The Carson City Kid	The Carson City Kid	Kane, Joseph	Joseph Kane	tt0032312
1746533178	1915	1915	Stolen Goods	Stolen Goods	Melford, George	George Melford	tt0006098
1746507862	1923	1923	Is Money Everything?	Is Money Everything?	Lyons, Glen	Glen Lyons	tt0014157
1746465243	1946	1945	The Windjammer	The Windjammer	Landers, Lew	Lew Landers	tt1572174
1746574926	1934	1934	Wheels of Destiny	Wheels of Destiny	James, Alan	Alan James	tt0025978
1769969543	1965	1965	It's a Sick, Sick, Sick World	It's a Sick, Sick, Sick World	Scarpati, Antonio	Antonio Scarpati	tt0222047
1746465267	1943	1943	Border Patrol	Border Patrol	Selander, Lesley	Lesley Selander	tt0035692
1746554156	1964	1964	Wild and Wonderful	Wild and Wonderful	Anderson, Michael	Michael Anderson	tt0058750
1746501396	1916	1916	A Woman's Power	A Woman's Power	Thornby, Robert	Robert Thornby	tt0159138
1746580333	1934	1934	No Greater Glory	No Greater Glory	Borzage, Frank	Frank Borzage	tt0025569
1746569587	1954	1954	Four Guns to the Border	Four Guns to the Border	Carlson, Richard	Richard Carlson	tt0046994
1746554685	1963	1963	Take Her, She's Mine	Take Her, She's Mine	Koster, Henry	Henry Koster	tt0057556
1746574656	1935	1935	The Throwback	The Throwback	Taylor, Ray	Ray Taylor	tt0027103
1746569577	1958	1958	New Orleans After Dark	New Orleans After Dark	Sledge, John	John Sledge	tt0051988
1746581113	1960	1960	Heroes Die Young	Heroes Die Young	Shepard, Gerald S.	Gerald Shepard	tt0220513
1746235509	1976	1976	Sparkle	Sparkle	O'Steen, Sam	Sam O'Steen	tt0075249
1769970837	1915	1915	The Mysterious Bride	The Mysterious Bride			tt3333296
1746580280	1934	1934	Bombay Mail	Bombay Mail	Marin, Edwin L.	Edwin L. Marin	tt0024904
1746574685	1935	1935	A Night at the Ritz	A Night at the Ritz	McGann, William	William C. McGann	tt0026779
1746577496	1933	1933	Mazie	Mazie	Fitzgerald, Dallas M.	Dallas M. Fitzgerald	tt0427512
1746520826	1925	1925	The Pinch Hitter	The Pinch Hitter	Henabery, Joseph	Joseph Henabery	tt0016224
1746523724	1916	1916	The Woman in the Case	The Woman in the Case	Ford, Hugh	Hugh Ford	tt0007580
1746208118	1969	1968	Kenner	Kenner	Sekely, Steve	Steve Sekely	tt0064539
1769972178	1961	1961	The Touchables	The Touchables	Mann, Monte; Manning, Monroe; Sh	Monte Mann	tt0055537
1746216942	1967	1967	What Am I Bid?	What Am I Bid?	Nash, Gene	Gene Nash	tt0062487
1746571086	1934	1934	The Firebird	The Firebird	Dieterle, William	William Dieterle	tt0025119
1746419912	1993	1993	The Dark Half	The Dark Half	Romero, George A.	George A. Romero	tt0106664
1746500520	1924	1924	The Left Hand Brand	The Left Hand Brand	Hart, Neal	Neal Hart	tt0135521
1746474086	1945	1945	Blazing the Western Trail	Blazing the Western Trail	Keays, Vernon	Vernon Keays	tt0191833
1746561303	1956	1956	The Come On	The Come On	Birdwell, Russell	Russell Birdwell	tt0049089
1746423755	1996	1996	When We Were Kings	When We Were Kings	Gast, Leon	Leon Gast	tt0118147
1746569781	1953	1953	The Charge at Feather River	The Charge at Feather River	Douglas, Gordon	Gordon Douglas	tt0045621
1746582244	1928	1928	On Trial	On Trial	Mayo, Archie	Archie Mayo	tt0019233
1746583838	1929	1929	Twin Beds	Twin Beds	Santell, Alfred	Alfred Santell	tt0020524
1746583343	1930	1930	The Big Pond	The Big Pond	Henley, Hobart	Hobart Henley	tt0020690
1746500687	1924	1924	The Uninvited Guest	The Uninvited Guest	Ince, Ralph	Ralph Ince	tt0015447
1746580262	1937	1937	Over the Goal	Over the Goal	Smith, Noel M.	Noel M. Smith	tt0029364
1746393655	1986	1986	Say Yes	Say Yes	Yust, Larry	Larry Yust	tt0091896

1746465392	1943	1943	The Strange Death of Adolf Hitler	The Strange Death of Adolf Hitler	Hogan, James	James P. Hogan	tt0036393
1746503256	1922	1922	The Law and the Woman	The Law and the Woman	Stanlaws, Penrhyn	Penrhyn Stanlaws	tt0013307
1746424041	1941	1941	Hold Back the Dawn	Hold Back the Dawn	Leisen, Mitchell	Mitchell Leisen	tt0033722
1746466802	1917	1917	The Cook of Canyon Camp	The Cook of Canyon Camp	Crisp, Donald	Donald Crisp	tt0168605
1746502165	1917	1917	The Bad Boy	The Bad Boy	Withey, Chester	Chester Withey	tt0007673
1746565964	1955	1955	The Eternal Sea	The Eternal Sea	Auer, John H.	John H. Auer	tt0048047
1746575047	1933	1933	Below the Sea	Below the Sea	Rogell, Albert	Albert S. Rogell	tt0023793
1746509575	1924	1924	Behind Two Guns	Behind Two Guns	Bradbury, Robert N.	Robert N. Bradbury	tt0014707
1746570762	1934	1934	Going Spanish	Going Spanish	Christie, Al	Al Christie	tt0025187
1746569547	1957	1957	Chicago Confidential	Chicago Confidential	Salkow, Sidney	Sidney Salkow	tt0050249
1746520056	1924	1924	Shackles of Fear	Shackles of Fear	Ferguson, Al	Al Ferguson	tt0015317
1746410214	1989	1989	Gross Anatomy	Gross Anatomy	Eberhardt, Thom	Thom Eberhardt	tt0097458
1746500487	1923	1923	Homeward Bound	Homeward Bound	Ince, Ralph	Ralph Ince	tt0014138
1746438180	1996	1996	Barb Wire	Barb Wire	Hogan, David	David Hogan	tt0115624
1745340053	2004	2004	Dodgeball: A True Underdog Story	Dodgeball: A True Underdog Story	Thurber, Rawson Marshall	Rawson Marshall Thurber	tt0364725
1746501380	1917	1917	Wild and Woolly	Wild and Woolly	Emerson, John	John Emerson	tt0008775
1746578570	1951	1951	Rhubarb	Rhubarb	Lubin, Arthur	Arthur Lubin	tt0043967
1746545272	1940	1940	Out West with the Peppers	Out West with the Peppers	Barton, Charles	Charles Barton	tt0032882
1746410171	1987	1986	Blood Hook	Blood Hook	Mallon, James	Jim Mallon	tt0090750
1746574019	1960	1960	One Foot in Hell	One Foot in Hell	Clark, James B.	James B. Clark	tt0054142
1745293815	2005	2005	King's Ransom	King's Ransom	Byrd, Jeff	Jeffrey W. Byrd	tt0388183
1746578843	1951	1951	The 13th Letter	The 13th Letter	Preminger, Otto	Otto Preminger	tt0043251
1746574359	1963	1963	Captain Sinbad	Captain Sindbad	Haskin, Byron	Byron Haskin	tt0056904
1746524456	1923	1923	Outlaws of the Sea	Outlaws of the Sea	Okey, Jack	Jack Okey	tt0145952
1746495601	1918	1918	Come on in	Come on In	Emerson, John	John Emerson	tt0008973
1746217739	1967	1967	Barefoot in the Park	Barefoot in the Park	Saks, Gene	Gene Saks	tt0061385
1745340271	2004	2004	Along Came Polly	Along Came Polly	Hamburg, John	John Hamburg	tt0343135
1746560595	1932	1932	The Man Called Back	The Man Called Back	Florey, Robert	Robert Florey	tt0023179
1745271893	2000	2000	Ready to Rumble	Ready to Rumble	Robbins, Brian	Brian Robbins	tt0217756
1746583076	1935	1934	Western Racketeers	Western Racketeers	Horner, Robert J.	Robert J. Horner	tt0240204
1746502164	1914	1914	The School for Scandal	The School for Scandal	Buel, Keanan	Kenean Buel	tt0004565
1769970441	1966	1965	Censored	Censored	Frost, R. L.	Barry Mahon	tt0171161
1746569514	1957	1957	The Hired Gun	The Hired Gun	Nazarro, Ray	Ray Nazarro	tt0050510
1746566318	1953	1953	Thunder Bay	Thunder Bay	Mann, Anthony	Anthony Mann	tt0046425
1746569988	1958	1957	Bitter Victory	Bitter Victory	Ray, Nicholas	Nicholas Ray	tt0050126
1746577965	1936	1936	Wife Vs. Secretary	Wife vs. Secretary	Brown, Clarence	Clarence Brown	tt0028505
1746577454	1962	1962	Invasion of the Star Creatures	Invasion of the Star Creatures	Ve Sota, Bruno	Bruno VeSota	tt0128274
1746453578	1945	1945	And Then There Were None	And Then There Were None	Clair, René	René Clair	tt0037515
1746465236	1941	1941	Up Jumped the Devil	Up Jumped the Devil		William Beaudine	tt0316838
1746434621	1992	1992	Mr. Baseball	Mr. Baseball	Schepisi, Fred	Fred Schepisi	tt0104926
1746564740	1952	1952	Bomba and the Jungle Girl	Bomba and the Jungle Girl	Beebe, Ford	Ford Beebe	tt0044435
1746491701	1945	1945	The Fatal Witness	The Fatal Witness	Selander, Lesley	Lesley Selander	tt0037693
1745293719	2006	2005	Tweek City	Tweek City	Johnson, Eric G.	Eric G. Johnson	tt0351885
1746497241	1919	1919	Happiness À La Mode	Happiness a la Mode	Edwards, Walter	Walter Edwards	tt0010207
1746576960	1959	1959	Street Fighter	Street Fighter	Savage, Vic	Joseph Sargent	tt0053311
1746513395	1914	1914	The Sign of the Cross	The Sign of the Cross	Thomson, Frederick	Frederick A. Thomson	tt0004593
1746530706	1949	1949	Streets of Laredo	Streets of Laredo	Fenton, Leslie	Leslie Fenton	tt0041929
1746585406	1940	1940	They Drive by Night	They Drive by Night	Walsh, Raoul	Raoul Walsh	tt0033149
1746232950	1966	1966	Who's Afraid of Virginia Woolf?	Who's Afraid of Virginia Woolf?	Nichols, Mike	Mike Nichols	tt0061184
1746423289	1990	1990	Quick Change	Quick Change	Franklin, Howard; Murray, Bill	Howard Franklin	tt0100449
1746516514	1919	1918	A Woman's Experience	A Woman's Experience	Vekroff, Perry N.	Perry N. Vekroff	tt0009836
1746524566	1916	1916	True Nobility	True Nobility	MacDonald, Donald	Donald MacDonald	tt0159054
1746578901	1959	1959	These Thousand Hills	These Thousand Hills	Fleischer, Richard	Richard Fleischer	tt0053350
1746495977	1919	1919	Girls	Girls	Edwards, Walter	Walter Edwards	tt0010175
1746436276	2000	2000	Bait	Bait	Fuqua, Antoine	Antoine Fuqua	tt0211938
1746465388	1946	1946	Colonel Effingham's Raid	Colonel Effingham's Raid	Pichel, Irving	Irving Pichel	tt0037607
1746577355	1961	1961	The Purple Hills	The Purple Hills	Dexter, Maury	Maury Dexter	tt0055343

1746434005	1997	1996	The Mouse	The House	Adams, Daniel	Keane Groisman	tt6443944
1746421959	1994	1994	Milk Money	Milk Money	Benjamin, Richard	Richard Benjamin	tt0110516
1746491859	1946	1946	Murder Is My Business	Murder Is My Business	Newfield, Sam	Sam Newfield	tt0038757
1746574768	1937	1937	True Confession	True Confession	Ruggles, Wesley	Wesley Ruggles	tt0029695
1746562209	1953	1953	Killer Ape	Killer Ape	Bennet, Spencer G.	Spencer Gordon Bennet	tt0045959
1746507585	1924	1924	Monsieur Beaucaire	Monsieur Beaucaire	Olcott, Sidney	Sidney Olcott	tt0015145
1746582957	1939	1939	Death of a Champion	Death of a Champion	Florey, Robert	Robert Florey	tt0031221
1746492422	1946	1946	Girl on the Spot	Girl on the Spot	Beaudine, William	William Beaudine	tt0038073
1746565876	1953	1953	The Living Desert	The Living Desert	Algar, James	James Algar	tt0046008
1746437287	1940	1940	The Farmer's Daughter	The Farmer's Daughter	Hogan, James	James P. Hogan	tt0032456
1746500155	1949	1948	Command Decision	Command Decision	Wood, Sam	Sam Wood	tt0040242
1746410757	1992	1992	Passion Fish	Passion Fish	Sayles, John	John Sayles	tt0105107
1746566917	1953	1952	Bwana Devil	Bwana Devil	Oboler, Arch	Arch Oboler	tt0044462
1746525198	1920	1920	Peaceful Valley	Peaceful Valley	Storm, Jerome	Jerome Storm	tt0011563
1746571910	1932	1932	Doctor X	Doctor X	Curtiz, Michael	Michael Curtiz	tt0022827
1746504342	1918	1918	Confession	Confession	Franklin, S. A.	Sidney Franklin	tt0008974
1746409708	1992	1992	Feed	Feed	Rafferty, Kevin; Ridgeway, James	Kevin Rafferty	tt0104244
1746503961	1921	1921	Closed Doors	Closed Doors	Seyffertitz, G. V.	Gustav von Seyffertitz	tt0012056
1769970570	1968	1968	The Kiss of Her Flesh	The Kiss of Her Flesh	Marsh, Julian	Michael Findlay	tt0125316
1746578427	1962	1962	Air Patrol	Air Patrol	Dexter, Maury	Maury Dexter	tt0055731
1746455277	1996	1995	Welcome to the Dollhouse	Welcome to the Dollhouse	Solondz, Todd	Todd Solondz	tt0114906
1746583515	1930	1930	Call of the Flesh	Call of the Flesh	Brabin, Charles	Charles Brabin	tt0020724
1745302274	2005	2005	Reel Paradise	Reel Paradise	James, Steve	Steve James	tt0405271
1746580328	1938	1938	Say It in French	Say It in French	Stone, Andrew L.	Andrew L. Stone	tt0030718
1746453609	1943	1943	Idaho	Idaho	Kane, Joseph	Joseph Kane	tt0036033
1745358640	2010	2010	She's Out of My League	She's Out of My League	Smith, Jim Field	Jim Field Smith	tt0815236
1746422074	1995	1995	Strange Days	Strange Days	Bigelow, Kathryn	Kathryn Bigelow	tt0114558
1746512135	1916	1916	When the Circus Came to Town	When the Circus Came to Town	Smith, Sidney	Sidney Smith	tt0434636
1746524241	1917	1917	Bringing Home Father	Bringing Home Father	Worthington, William	William Worthington	tt0007749
1746571102	1935	1935	Hop-Along Cassidy	Hop a Long Cassidy	Bretherton, Howard	Howard Bretherton	tt0026493
1746514393	1916	1916	The Fear of Poverty	The Fear of Poverty	Sullivan, Frederic	Frederick Sullivan	tt0157622
1746568130	1934	1934	Flirting with Danger	Flirting with Danger	Moore, Vin	Vin Moore	tt0025125
1746533166	1915	1915	The Cowboy and the Lady	The Cowboy and the Lady	Carewe, Edwin	Edwin Carewe	tt0005151
1746584270	1939	1939	Pride of the Navy	Pride of the Navy	Lamont, Charles	Charles Lamont	tt0031824
1746566073	1956	1956	Hilda Crane	Hilda Crane	Dunne, Philip	Philip Dunne	tt0049317
1746503808	1920	1920	Beyond the Great Wall	Beyond the Great Wall	Heath, A. B.	Arch Heath	tt0193767
1746491885	1944	1944	The Battle of China	The Battle of China		Frank Capra	tt0184254
1745293313	2006	2006	Man of the Year	Man of the Year	Levinson, Barry	Barry Levinson	tt0483726
1746465119	1947	1947	The Peanut Man	The Peanut Man	Paton, Tony		tt0135595
1746578947	1963	1963	Monstrosity	Monstrosity	Mascelli, Joseph V.	Joseph V. Mascelli	tt0057859
1746454654	1940	1940	Private Affairs	Private Affairs	Rogell, Albert S.	Albert S. Rogell	tt0032947
1746219620	1972	1972	FTA	FTA	Parker, Francine	Francine Parker	tt0068562
1746583069	1935	1935	Baby Face Harrington	Baby Face Harrington	Walsh, Raoul	Raoul Walsh	tt0026092
1746507774	1927	1926	The Scarlet Letter	The Scarlet Letter	Seastrom, Victor	Victor Sjöström	tt0017350
1746474820	1945	1945	The House on 92nd Street	The House on 92nd Street	Hathaway, Henry	Henry Hathaway	tt0037795
1746520895	1948	1948	To the Ends of the Earth	To the Ends of the Earth	Stevenson, Robert; Buchman, Sidney	Robert Stevenson	tt0040887
1746423278	1994	1994	Shrunken Heads	Shrunken Heads	Elfman, Richard	Richard Elfman	tt0111175
1746562342	1953	1953	The Hitch-Hiker	The Hitch Hiker	Lupino, Ida	Ida Lupino	tt0045877
2007990110	1993	1993	The Thing Called Love	The Thing Called Love	Bogdanovich, Peter	Peter Bogdanovich	tt0108327
1745359670	2004	2004	Agent Cody Banks 2: Destination London	Agent Cody Banks 2: Destination London	Allen, Kevin	Kevin Allen	tt0358349
1746523315	1922	1922	Isle of Doubt	Isle of Doubt	Smith, Hamilton	Hamilton Smith	tt0013273
1746567464	1933	1933	Thunder over Mexico	Thunder Over Mexico	Eisenstein, Sergei M.	Sergei M. Eisenstein	tt0024668
1746574791	1935	1935	It Happened in New York	It Happened in New York	Crosland, Alan	Alan Crosland	tt0026535
1746393970	1986	1986	The Morning After	The Morning After	Lumet, Sidney	Sidney Lumet	tt0091554
1746210472	1972	1972	Ben	Ben	Karlson, Phil	Phil Karlson	tt0068264
1746580221	1939	1939	The Hunchback of Notre Dame	The Hunchback of Notre Dame	Dieterle, William	William Dieterle	tt0031455
1746570309	1934	1934	Beggar's Holiday	Beggar's Holiday	Newfield, Sam	Sam Newfield	tt0024869

1746565909	1954	1954	The Iron Glove	The Iron Glove	Castle, William	William Castle	tt0047121	
1746582718	1935	1935	Border Brigands	Border Brigands	Grinde, Nick	Nick Grinde	tt0026127	
1746562073	1954	1954	Silent Raiders	Silent Raiders	Bartlett, Richard	Richard Bartlett	tt0047492	
1746571934	1931	1931	The Spy	The Spy	Viertel, Berthold	Berthold Viertel	tt0022427	
1746491872	1946	1946	Sweetheart of Sigma Chi	Sweetheart of Sigma Chi	Bernhard, Jack	Jack Bernhard	tt0039002	
1746209253	1967	1967	How to Succeed in Business Without Really Tryi	How to Succeed in Business Without Really	Swift, David	David Swift	tt0061791	
1746438166	1941	1941	Border Vigilantes	Border Vigilantes	Abrahams, Derwin	Derwin Abrahams	tt0033425	
1746532983	1915	1915	The Cave Man	The Cave Man	Marston, Theodore	Theodore Marston	tt0005070	
1746513389	1913	1913	The Battle of Gettysburg	The Battle of Gettysburg	Ince, Thomas H.	Charles Giblyn	tt0002669	
1746582925	1937	1937	The Legion of Missing Men	The Legion of Missing Men	MacFadden, Hamilton	Hamilton MacFadden	tt0029130	
1746495955	1921	1921	The Passion Flower	Passion Flower	Brenon, Herbert	Herbert Brenon	tt0012555	
1746570048	1933	1933	The Mind Reader	The Mind Reader	Del Ruth, Roy	Roy Del Ruth	tt0024339	
1746419617	1992	1992	Roadside Prophets	Roadside Prophets	Wool, Abbe	Abbe Wool	tt0105267	
1769970544	1966	1966	Unholy Matrimony	Unholy Matrimony	John, Arthur	Arthur John	tt0153446	
1746455289	1996	1996	Bound	Bound	Wachowski, Larry; Wachowski, Andy	Lana Wachowski	tt0115736	
1746257773	1980	1980	Times Square	Times Square	Moyle, Alan	Allan Moyle	tt0081635	
1746453834	1944	1944	Thoroughbreds	Thoroughbreds	Blair, George	George Blair	tt0037369	
1746581522	1928	1928	West of Zanzibar	West of Zanzibar	Browning, Tod	Tod Browning	tt0019563	
1746492052	1948	1948	The Return of the Whistler	The Return of the Whistler	Lederman, D. Ross	D. Ross Lederman	tt0040733	
1746454677	1942	1942	Stagecoach Buckaroo	Stagecoach Buckaroo	Taylor, Ray	Ray Taylor	tt0035376	
1746513803	1915	1915	The Case of Becky	The Case of Becky	Reicher, Frank	Frank Reicher	tt0005063	
1746500604	1925	1925	Pursued	Pursued	Henderson, Dell	Dell Henderson	tt0016254	
1745339983	2003	2003	School of Rock	School of Rock	Linklater, Richard	Richard Linklater	tt0332379	
1746574881	1936	1936	Freshman Love	Freshman Love	McGann, William	William C. McGann	tt0027645	
1746585411	1939	1939	El Diablo Rides	El Diablo Rides	Webb, Ira S.	Ira Webb	tt0031267	
1746185598	1966	1966	Namu, the Killer Whale	Namu, the Killer Whale	Benedek, Laslo	Laslo Benedek	tt0060737	
1746513031	1910	1910	On the Reef	On the Reef	Griffith, D. W.	D.W. Griffith	tt0001345	
1746523329	1923	1923	The Law Rustlers	The Law Rustlers	King, Lewis	Louis King	tt0126996	
1746503456	1919	1919	The Great Air Robbery	The Great Air Robbery	Jaccard, Jacques	Jacques Jaccard	tt0011241	
1746420221	1994	1994	Surviving the Game	Surviving the Game	Dickerson, Ernest	Ernest R. Dickerson	tt0111323	
1746570028	1934	1934	Big Hearted Herbert	Big Hearted Herbert	Keighley, William	William Keighley	tt0024892	
1745340039	2003	2003	The In-Laws	The In Laws	Fleming, Andrew	Andrew Fleming	tt0314786	
1746568126	1933	1933	Jungle Bride	Jungle Bride	Hoyt, Harry O.; Kelley, Albert	Harry O. Hoyt	tt0024199	
1745272342	2000	1999	The Sky Is Falling	The Sky Is Falling	Laurence, Florrie	Florrie Laurence	tt0120829	
1746577538	1936	1936	A Face in the Fog	A Face in the Fog	Hill, Bob	Robert F. Hill	tt0144191	
1746578864	1951	1951	Texas Lawmen	Texas Lawmen	Collins, Lewis	Lewis D. Collins	tt0180243	
1746563634	1952	1952	The Snows of Kilimanjaro	The Snows of Kilimanjaro	King, Henry	Henry King	tt0045162	
**1746532723**	**1927**	**1927**	**Combat**	**Combat**	**Hiatt, Albert**	**Albert Hiatt**	**tt0017768**	1
1746532985	1923	1923	Safety Last!	Safety Last!	Newmeyer, Fred; Taylor, Sam	Fred C. Newmeyer	tt0014429	
1746504005	1922	1922	Lucky Dan	Lucky Dan	Howard, William K.	William K. Howard	tt0013340	
1746503600	1918	1918	The Cabaret Girl	The Cabaret Girl	Gerrard, Douglas	Douglas Gerrard	tt0008943	
1746498306	1920	1920	Drag Harlan	Drag Harlan	Edwards, J. Gordon	J. Gordon Edwards	tt0011133	
1746573852	1958	1957	Appointment with a Shadow	Appointment with a Shadow	Carlson, Richard	Richard Carlson	tt0051370	
1746574153	1961	1961	The Cat Burglar	The Cat Burglar	Witney, William	William Witney	tt0054733	
1746583056	1939	1939	Mystery of the White Room	Mystery of the White Room	Garrett, Otis	Otis Garrett	tt0031705	
1746500713	1924	1924	Try and Get It	Try and Get It	Tate, Cullen	Cullen Tate	tt0015439	
1746438620	1943	1943	Ladies' Day	Ladies' Day	Goodwins, Leslie	Leslie Goodwins	tt0036091	
1746218744	1976	1976	Revenge of the Cheerleaders	Revenge of the Cheerleaders	Lerner, Richard	Richard Lerner	tt0075137	
1746520855	1925	1925	The Unknown Lover	The Unknown Lover	Halperin, Victor Hugo	Victor Halperin	tt0016475	
1746501775	1915	1915	The Stubbornness of Geraldine	The Stubbornness of Geraldine	Mervale, Gaston	Gaston Mervale	tt0006112	
1746523075	1914	1914	The Key to Yesterday	The Key to Yesterday	Dillon, J. Francis	John Francis Dillon	tt0004188	
1746492571	1948	1948	The Gentleman from Nowhere	The Gentleman from Nowhere	Castle, William	William Castle	tt0040386	
1746514569	1927	1927	Yours To Command	Yours to Command	Kirkland, David	David Kirkland	tt0018605	
1746584595	1931	1931	Rich Man's Folly	Rich Man's Folly	Cromwell, John	John Cromwell	tt0022309	
1746567616	1933	1933	Marriage on Approval	Marriage on Approval	Higgin, Howard	Howard Higgin	tt0129226	
1746465030	1943	1943	Wintertime	Wintertime	Brahm, John	John Brahm	tt0036540	
1746580792	1935	1935	Murder on a Honeymoon	Murder on a Honeymoon	Corrigan, Lloyd	Lloyd Corrigan	tt0026744	

1746584029	1935	1935	Clive of India	Clive of India	Boleslawski, Richard	Richard Boleslawski	tt0026221
1746562284	1953	1953	The Fighting Lawman	Fighting Lawman	Carr, Thomas	Thomas Carr	tt0045766
1746464965	1945	1945	Sagebrush Heroes	Sagebrush Heroes	Kline, Benjamin	Benjamin H. Kline	tt0037241
1746504736	1922	1922	Between Two Husbands	Between Two Husbands			tt0012934
1746577282	1958	1958	Frontier Gun	Frontier Gun	Landres, Paul	Paul Landres	tt0051639
1746454620	1940	1940	Bad Man from Red Butte	Bad Man from Red Butte	Taylor, Ray	Ray Taylor	tt0032232
1746420363	1990	1990	Goodfellas	Goodfellas	Scorsese, Martin	Martin Scorsese	tt0099685
1746513426	1916	1916	The Summer Girl	The Summer Girl	August, Edwin	Edwin August	tt0007408
1746455048	1999	1999	End of Days	End of Days	Hyams, Peter	Peter Hyams	tt0146675
1746583536	1929	1929	Hunting Tigers in India	Hunting Tigers in India	Dyott, George M.; Meehan, Leo	James Leo Meehan	tt0295998
1746500215	1925	1925	Bobbed Hair	Bobbed Hair	Crosland, Alan	Alan Crosland	tt0015632
1746492407	1948	1948	The Babe Ruth Story	The Babe Ruth Story	Del Ruth, Roy	Roy Del Ruth	tt0040142
1746584473	1931	1931	Graft	Graft	Cabanne, Christy	Christy Cabanne	tt0021926
1746437021	1941	1941	Skylark	Skylark	Sandrich, Mark	Mark Sandrich	tt0034198
1746502446	1910	1910	A Woman's Wit	A Woman's Wit		Joseph A. Golden	tt0291568
1746560975	1932	1932	Texas Cyclone	Texas Cyclone	Lederman, D. Ross	D. Ross Lederman	tt0023567
1746514622	1915	1915	M'liss	M'Liss	Lund, O. A. C.	O.A.C. Lund	tt0005671
1746421669	1999	1999	The Other Sister	The Other Sister	Marshall, Garry	Garry Marshall	tt0123209
1746269675	1979	1979	Starting Over	Starting Over	Pakula, Alan J.	Alan J. Pakula	tt0079948
1746580693	1938	1938	Storm over Bengal	Storm Over Bengal	Salkow, Sidney	Sidney Salkow	tt0030798
1746573838	1957	1957	Hell on Devil's Island	Hell on Devil's Island	Nyby, Christian	Christian Nyby	tt0050498
1746571672	1931	1931	The Forgotten Frontier	The Forgotten Frontier		Marvin Breckinridge	tt0021881
1746514815	1923	1923	None So Blind	None So Blind	King, Burton	Burton L. King	tt0014315
1746582505	1939	1939	The Little Princess	The Little Princess	Lang, Walter	Walter Lang	tt0031580
1746516067	1921	1921	Hush	Hush	Garson, Harry	Harry Garson	tt0012299
1746584644	1928	1928	The Girl He Didn't Buy	The Girl He Didn't Buy	Fitzgerald, Dallas M.	Dallas M. Fitzgerald	tt0133801
1746582278	1930	1930	The Midnight Special	Midnight Special	Worne, Duke	Duke Worne	tt0022148
1746504014	1922	1922	Minnie	Minnie		Marshall Neilan	tt0013392
1769974306	1916	1916	Billie's Headache	Billie's Headache	Metcalfe, Earl	Earl Metcalfe	tt0416576
1746565927	1957	1957	The Careless Years	The Careless Years	Hiller, Arthur	Arthur Hiller	tt0050229
1745341803	2001	2001	Sidewalks of New York	Sidewalks of New York	Burns, Edward	Edward Burns	tt0239986
1746560620	1932	1932	The King Murder	The King Murder	Thorpe, Richard	Richard Thorpe	tt0023092
1769971972	1969	1969	Switcheroo!	Switcheroo!	Donne, John	Donn Greer	tt0259022
1745395420	2010	2009	Solitary Man	Solitary Man	Koppelman, Brian; Levien, David	Brian Koppelman	tt1294213
1746497227	1921	1921	Dawn of the East	Dawn of the East	Griffith, E. H.	Edward H. Griffith	tt0012097
1746500451	1924	1924	Sinners in Heaven	Sinners in Heaven	Crosland, Alan	Alan Crosland	tt0015335
1746583770	1930	1930	The Convict's Code	Convict's Code	Revier, Harry	Harry Revier	tt0020788
1746583295	1929	1929	A Song of Kentucky	A Song of Kentucky	Seiler, Lewis	Lewis Seiler	tt0020433
1746218246	1968	1967	Will Penny	Will Penny	Gries, Tom	Tom Gries	tt0063811
1746465308	1945	1945	An Angel Comes to Brooklyn	An Angel Comes to Brooklyn	Goodwins, Leslie	Leslie Goodwins	tt0037516
1746234083	1967	1967	C'mon, Let's Live a Little	C'mon, Let's Live a Little	Butler, David	David Butler	tt0061434
1746524022	1927	1927	The Devil Dancer	The Devil Dancer	Niblo, Fred; Raboch, Alfred; Shores, I	Fred Niblo	tt0017806
1746554190	1964	1964	The Night of the Iguana	The Night of the Iguana	Huston, John	John Huston	tt0058404
1746508482	1926	1926	The Temptress	The Temptress	Stiller, Mauritz	Fred Niblo	tt0017449
1746502193	1915	1915	The Hungarian Nabob	The Hungarian Nabob	Vale, Travers	Travers Vale	tt0005525
1746453832	1942	1942	Cowboy Serenade	Cowboy Serenade	Morgan, William	William Morgan	tt0034619
1745293506	2007	2007	Things We Lost in the Fire	Things We Lost in the Fire	Bier, Susanne	Susanne Bier	tt0469623
1746569544	1957	1957	Something of Value	Something of Value	Brooks, Richard	Richard Brooks	tt0050993
1746513816	1923	1923	Thru the Flames	Thru the Flames	Nelson, Jack	Jack Nelson	tt0170683
1746513780	1915	1915	The Fixer	The Fixer		Edward Morrissey	tt0447151
1746473703	1946	1946	Shadowed	Shadowed	Sturges, John	John Sturges	tt0038928
1769969388	1965	1965	The Girl With the Magic Box	The Girl with the Magic Box	Mahon, Barry	Barry Mahon	tt0262434
1746501671	1915	1915	Dr. Rameau	Dr. Rameau	Davis, Will S.	Will S. Davis	tt0005216
1746500166	1949	1949	Prince of Foxes	Prince of Foxes	King, Henry	Henry King	tt0041767
1746453839	1944	1944	Wing and a Prayer	Wing and a Prayer	Hathaway, Henry	Henry Hathaway	tt0037466
1746524630	1910	1910	Senator's Double	The Senator's Double		Harry Solter	tt0201077
1746454842	1941	1941	The Chocolate Soldier	The Chocolate Soldier	Del Ruth, Roy	Roy Del Ruth	tt0033464

1746408626	1986	1986	Hannah and Her Sisters	Hannah and Her Sisters	Allen, Woody	Woody Allen	tt0091167	
1746578405	1959	1959	The Diary of Anne Frank	The Diary of Anne Frank	Stevens, George	George Stevens	tt0052738	
1746533189	1927	1927	Grinning Guns	Grinning Guns	Rogell, Albert	Albert S. Rogell	tt0017951	
1746585285	1928	1929	The Wages of Sin	Wages of Sin	Micheaux, Oscar	Oscar Micheaux	tt0139731	
1745272133	2001	2001	Corky Romano	Corky Romano	Pritts, Rob	Rob Pritts	tt0250310	
1746394170	1986	1986	The Best of Times	The Best of Times	Spottiswoode, Roger	Roger Spottiswoode	tt0090713	
1746454857	1995	1995	Three Wishes	Three Wishes	Coolidge, Martha	Martha Coolidge	tt0114663	
1746583500	1930	1930	Courage	Courage	Mayo, Archie	Archie Mayo	tt0020794	
1746464854	1941	1941	Come Live with Me	Come Live with Me	Brown, Clarence	Clarence Brown	tt0033477	
1746515383	1920	1920	The Flaming Clue	The Flaming Clue	Hollywood, Edwin L.	Edwin L. Hollywood	tt0195711	
1746504599	1919	1919	The Black Gate	The Black Gate	Marston, Theodore	Theodore Marston	tt0186883	
1746453815	1943	1943	For Whom the Bell Tolls	For Whom the Bell Tolls	Wood, Sam	Sam Wood	tt0035896	
1746435834	1990	1990	White Palace	White Palace	Mandoki, Luis	Luis Mandoki	tt0103251	
1746577766	1935	1935	It's a Great Life!	It's a Great Life	Cline, Edward F.	Edward F. Cline	tt0027812	
1746408860	1982	1982	Deathtrap	Deathtrap	Lumet, Sidney	Sidney Lumet	tt0083806	
1746464846	1940	1940	Meet the Wildcat	Meet the Wildcat	Lubin, Arthur	Arthur Lubin	tt0032774	
1746545202	1940	1940	The Fight for Life	The Fight for Life	Lorentz, Pare	Pare Lorentz	tt0032465	
1746576958	1961	1961	Judgment at Nuremberg	Judgment at Nuremberg	Kramer, Stanley	Stanley Kramer	tt0055031	
1746501674	1914	1914	In Mizzoura	In Mizzoura	McGill, Lawrence B.	Lawrence B. McGill	tt0138515	
1746512100	1917	1917	The Soul of Satan	The Soul of Satan	Turner, Otis	Otis Turner	tt0182439	
1746455962	1998	1997	Passion in the Desert	Passion in the Desert	Currier, Lavinia	Lavinia Currier	tt0125980	
1746439202	1999	1999	Never Been Kissed	Never Been Kissed	Gosnell, Raja	Raja Gosnell	tt0151738	
1746566108	1956	1956	Forever, Darling	Forever, Darling	Hall, Alexander	Alexander Hall	tt0049225	
**1746503738**	**1918**	**1918**	**The Sea Flower**	**The Sea Flower**	**Campbell, Colin**	**Colin Campbell**	**tt0009587**	1
1746564431	1952	1952	The Wild North	The Wild North	Marton, Andrew	Andrew Marton	tt0045329	
1745309329	2006	2006	The Black Dahlia	The Black Dahlia	De Palma, Brian	Brian De Palma	tt0387877	
1746533026	1916	1916	The Flower of Faith	The Flower of Faith	King, Burton L.	Burton L. King	tt0162328	
1746583222	1930	1929	Hell's Heroes	Hell's Heroes	Wyler, William	William Wyler	tt0019976	
1746219589	1974	1974	The Texas Chain Saw Massacre	The Texas Chain Saw Massacre	Hooper, Tobe	Tobe Hooper	tt0072271	
1746218839	1972	1972	The Limit	The Limit	Kotto, Yaphet	Yaphet Kotto	tt0068861	
1746560538	1932	1932	The County Fair	The County Fair	King, Louis	Louis King	tt0022781	
1746570456	1934	1934	The Life of Vergie Winters	The Life of Vergie Winters	Santell, Alfred	Alfred Santell	tt0025393	
1746473927	1947	1947	Railroaded!	Railroaded!	Mann, Anthony	Anthony Mann	tt0039748	
1746578807	1960	1960	The Sundowners	The Sundowners	Zinnemann, Fred	Fred Zinnemann	tt0054353	
1746503033	1915	1915	Wormwood	Wormwood	Farnum, Marshall	Marshall Farnum	tt0006317	
1746423759	1998	1998	The Wedding Singer	The Wedding Singer	Coraci, Frank	Frank Coraci	tt0120888	
1746520314	1925	1925	Ranchers and Rascals	Ranchers and Rascals		Leo D. Maloney	tt0016260	
1745395125	2008	2007	Remember the Daze	Remember the Daze	Manafort, Jess	Jess Manafort	tt0790618	
1746570158	1934	1934	The Crime of Helen Stanley	The Crime of Helen Stanley	Lederman, D. Ross	D. Ross Lederman	tt0025010	
1746523364	1927	1927	The Fightin' Comeback	The Fightin' Comeback	Wright, Tenny	Tenny Wright	tt0017876	
1746573975	1959	1959	Teenagers from Outer Space	Teenagers from Outer Space	Graeff, Tom	Tom Graeff	tt0053337	
1746436578	1940	1940	Congo Maisie	Congo Maisie	Potter, Henry C.	H.C. Potter	tt0032353	
1746582525	1935	1935	Wolf Riders	Wolf Riders	Webb, Harry S.	Harry S. Webb	tt0141994	
1746520301	1949	1949	Shockproof	Shockproof	Sirk, Douglas	Douglas Sirk	tt0041871	
1746520232	1925	1925	Speed Madness	Speed Madness	Mitchell, Bruce	Bruce Mitchell	tt0016382	
1746501153	1916	1916	The Purple Lady	The Purple Lady	Lessey, George A.	George Lessey	tt0007230	
1746475677	1947	1947	Born to Kill	Born to Kill	Wise, Robert	Robert Wise	tt0039211	
1746577812	1935	1935	Cardinal Richelieu	Cardinal Richelieu	Lee, Rowland V.	Rowland V. Lee	tt0026180	
1746585036	1940	1940	Melody Ranch	Melody Ranch	Santley, Joseph	Joseph Santley	tt0032779	
1746409992	1992	1992	Wayne's World	Wayne's World	Spheeris, Penelope	Penelope Spheeris	tt0105793	
1746491801	1948	1948	The Secret Land	The Secret Land			tt0040767	
1746520925	1949	1948	The Kissing Bandit	The Kissing Bandit	Benedek, Laslo	Laslo Benedek	tt0040513	
1745341467	2003	2002	The Year That Trembled	The Year That Trembled	Craven, Jay	Jay Craven	tt0288324	
1746211249	1973	1973	A Warm December	A Warm December	Poitier, Sidney	Sidney Poitier	tt0070898	
1746514336	1916	1916	Thou Shalt Not Covet	Thou Shalt Not Covet	Campbell, Colin	Colin Campbell	tt0007451	
1746577022	1951	1951	G. I. Jane	G.I. Jane	Le Borg, Reginald	Reginald Le Borg	tt0043572	
1746580518	1935	1935	Nevada	Nevada	Barton, Charles	Charles Barton	tt0028026	

1746577252	1950	1950	Tarnished	Tarnished	Keller, Harry	Harry Keller	tt0043024
1746533011	1915	1915	The Deathlock	The Deathlock		Fred J. Butler	tt0005184
1746434334	1989	1989	Dream a Little Dream	Dream a Little Dream	Rocco, Marc	Marc Rocco	tt0097236
1746583332	1930	1930	Mammy	Mammy	Curtiz, Michael	Michael Curtiz	tt0021110
1746532763	1914	1914	Salomy Jane	Salomy Jane	Henderson, Lucius; Nigh, William	Lucius Henderson	tt0004555
1746501470	1916	1916	Oliver Twist	Oliver Twist	Young, James	James Young	tt0007142
1746218872	1974	1974	The Lords of Flatbush	The Lords of Flatbush	Verona, Stephen F.; Davidson, Marti	Martin Davidson	tt0071772
1746582651	1935	1935	The Bishop Misbehaves	The Bishop Misbehaves	Dupont, E. A.	Ewald André Dupont	tt0026120
1745342829	2008	2008	The Ruins	The Ruins	Smith, Carter	Carter Smith	tt0963794
1746394030	1989	1989	Enemies, A Love Story	Enemies: A Love Story	Mazursky, Paul	Paul Mazursky	tt0097276
1746392656	1982	1982	Alone in the Dark	Alone in the Dark	Sholder, Jack	Jack Sholder	tt0083542
1746577807	1936	1936	Outlaws of the Range	Outlaws of the Range	Herman, Al	Albert Herman	tt0028072
1746492490	1947	1946	Cross My Heart	Cross My Heart	Berry, John	John Berry	tt0038438
1746420508	1982	1982	I, the Jury	I, the Jury	Heffron, Richard T.	Richard T. Heffron	tt0084112
1746578631	1962	1962	Confessions of an Opium Eater	Confessions of an Opium Eater	Zugsmith, Albert	Albert Zugsmith	tt0055864
1746574436	1962	1962	Freud	Freud	Huston, John	John Huston	tt0055998
1746567614	1933	1933	Girl Without a Room	Girl Without a Room	Murphy, Ralph	Ralph Murphy	tt0024063
1746584723	1928	1928	The Grain of Dust	The Grain of Dust	Archainbaud, George	George Archainbaud	tt0018953
1746573763	1958	1958	A Nice Little Bank That Should Be Robbed	A Nice Little Bank That Should Be Robbed	Levin, Henry	Henry Levin	tt0051990
1745342910	2003	2003	The Rundown	The Rundown	Berg, Peter	Peter Berg	tt0327850
1746582536	1935	1935	G-Men	'G' Men	Keighley, William	William Keighley	tt0026393
1746410544	1985	1984	The Dungeonmaster	The Dungeonmaster		David Allen	tt0089060
1746584430	1939	1939	The Ice Follies of 1939	The Ice Follies of 1939	Schunzel, Reinhold	Reinhold Schünzel	tt0031470
1746439089	1996	1995	From the Journals of Jean Seberg	From the Journals of Jean Seberg	Rappaport, Mark	Mark Rappaport	tt0113125
1746514284	1923	1923	Where Is This West?	Where Is This West?	Marshall, George E.	George Marshall	tt0014599
1746423971	1943	1943	At the Front in North Africa with the U.S. Army	At the Front in North Africa with the U.S. Army		John Ford	tt2491546
1746501665	1916	1916	Husband and Wife	Husband and Wife	O'Neil, Barry	Barry O'Neil	tt0006838
1745340767	2002	2002	Tuck Everlasting	Tuck Everlasting	Russell, Jay	Jay Russell	tt0283084
1746565910	1953	1953	One Girl's Confession	One Girl's Confession	Haas, Hugo	Hugo Haas	tt0046143
1746408509	1988	1988	Vibes	Vibes	Kwapis, Ken	Ken Kwapis	tt0096378
1746453828	1941	1941	Ziegfeld Girl	Ziegfeld Girl	Leonard, Robert Z.	Robert Z. Leonard	tt0034415
1746583430	1931	1930	Playthings of Hollywood	Playthings of Hollywood	O'Connor, William	William A. O'Connor	tt0022269
1746483868	1918	1918	A Japanese Nightingale	A Japanese Nightingale	Fitzmaurice, George	George Fitzmaurice	tt0009237
1745309645	2006	2006	The Gravedancers	The Gravedancers	Mendez, Mike	Mike Mendez	tt0435653
1746497555	1917	1917	The Web of Life	The Web of Life	Rolands, George K.	George K. Rolands	tt0184002
1746569641	1958	1958	Live Fast, Die Young	Live Fast, Die Young	Henreid, Paul	Paul Henreid	tt0051871
1746574112	1958	1958	Space Master X-7	Space Master X 7	Bernds, Edward	Edward Bernds	tt0052228
1746486304	1920	1920	Water, Water Everywhere	Water, Water, Everywhere	Badger, Clarence G.	Clarence G. Badger	tt0011840
1746409078	1982	1982	Zapped!	Zapped!	Rosenthal, Robert J.	Robert J. Rosenthal	tt0084945
1746562474	1953	1953	Fear and Desire	Fear and Desire	Kubrick, Stanley	Stanley Kubrick	tt0045758
1746580392	1939	1939	Society Lawyer	Society Lawyer	Marin, Edwin L.	Edwin L. Marin	tt0031946
1746514243	1916	1916	The Prince Chap	The Prince Chap	Neilan, Marshall	Marshall Neilan	tt0007218
1746492380	1945	1945	Gangs of the Waterfront	Gangs of the Waterfront	Blair, George	George Blair	tt0037727
1745373077	2010	2010	Howl	The Owls	Epstein, Rob; Friedman, Jeffrey	Cheryl Dunye	tt1507369
1746423308	1997	1997	Slaves to the Underground	Slaves to the Underground	Peterson, Kristine	Kristine Peterson	tt0117664
1746409950	1989	1989	The Wizard	The Wizard	Holland, Todd	Todd Holland	tt0098663
1746583163	1935	1935	On Probation	On Probation	Hutchison, Charles	Charles Hutchison	tt0026817
1746408472	1986	1986	Nomads	Nomads	McTiernan, John	John McTiernan	tt0091647
1746580472	1937	1937	The Fighting Texan	The Fighting Texan	Abbott, Charles	Charles Abbott	tt0132951
1769969520	1911	1911	The Fallin' Out	The Fallin' Out			tt0359252
1746577921	1937	1937	What Price Vengeance	What Price Vengeance	Lord, Del	Del Lord	tt0029728
1746409502	1985	1985	Into the Night	Into the Night	Landis, John	John Landis	tt0089346
1746581462	1962	1962	Strangers in the City	Strangers in the City	Carrier, Rick	Rick Carrier	tt0056529
1746573815	1957	1957	Panama Sal	Panama Sal	Witney, William	William Witney	tt0050816
1746523812	1923	1923	The Shock	The Shock	Hillyer, Lambert	Lambert Hillyer	tt0014463
1746485898	1920	1920	Terror Island	Terror Island	Cruze, James	James Cruze	tt0011753
1746580360	1935	1935	Death Flies East	Death Flies East	Rosen, Phil	Phil Rosen	tt0026268

1746562180	1957	1957	Not of This Earth	Not of This Earth	Corman, Roger	Roger Corman	tt0050778
1746571752	1932	1932	Tiger Shark	Tiger Shark	Hawks, Howard	Howard Hawks	tt0023594
1746581107	1960	1960	Last Woman on Earth	Last Woman on Earth	Corman, Roger	Roger Corman	tt0054017
1746574703	1933	1933	The Right to Romance	The Right to Romance	Santell, Alfred	Alfred Santell	tt0024501
1746512622	1910	1910	The Winning of Miss Langdon	The Winning of Miss Langdon		Edwin S. Porter	tt1064824
1746219342	1978	1978	Moment by Moment	Moment by Moment	Wagner, Jane	Jane Wagner	tt0077942
1746197930	1969	1969	The Lost Man	The Lost Man	Aurthur, Robert Alan	Robert Alan Aurthur	tt0064602
1746550313	1957	1957	Love Slaves of the Amazons	Love Slaves of the Amazons	Siodmak, Curt	Curt Siodmak	tt0050656
1746580423	1938	1938	God's Step Children	God's Step Children	Micheaux, Oscar	Oscar Micheaux	tt0028942
1746504909	1921	1921	Don't Neglect Your Wife	Don't Neglect Your Wife	Worsley, Wallace	Wallace Worsley	tt0012116
1746436360	1940	1940	Father Is a Prince	Father Is a Prince	Smith, Noel	Noel M. Smith	tt0032458
1746580277	1938	1938	Rollin' Plains	Rollin' Plains	Herman, Al	Albert Herman	tt0030689
1746408910	1992	1992	Sneakers	Sneakers	Robinson, Phil Alden	Phil Alden Robinson	tt0105435
1746580885	1934	1934	Embarrassing Moments	Embarrassing Moments	Laemmle, Edward	Edward Laemmle	tt0025081
1746424442	1941	1941	Borrowed Hero	Borrowed Hero	Collins, Lewis D.	Lewis D. Collins	tt0033426
1746513638	1915	1915	Graustark	Graustark	Wright, Fred E.	Fred E. Wright	tt0005411
1746567993	1934	1934	Frontier Days	Frontier Days	Hill, Robert	Robert F. Hill	tt0025143
1746584185	1938	1938	If I Were King	If I Were King	Lloyd, Frank	Frank Lloyd	tt0030265
1746484432	1919	1919	Someone Must Pay	Someone Must Pay	Abramson, Ivan	Ivan Abramson	tt0168180
1746534781	1921	1921	The Double O	The Double O	Clements, Roy	Roy Clements	tt0012119
1746569846	1955	1955	Kismet	Kismet	Minnelli, Vincente	Vincente Minnelli	tt0048260
1746514698	1927	1927	The Interferin' Gent	The Interferin' Gent	Thorpe, Richard	Richard Thorpe	tt0124760
1746454384	1943	1943	Death Valley Rangers	Death Valley Rangers	Tansey, Robert	Robert Emmett Tansey	tt0035788
1746581927	1928	1928	Under the Black Eagle	Under the Black Eagle	Van Dyke, W. S.	W.S. Van Dyke	tt0019507
1746504170	1918	1918	The Green God	The Green God	Scardon, Paul	Paul Scardon	tt0010194
1746525134	1921	1921	The Woman God Changed	The Woman God Changed	Vignola, Robert G.	Robert G. Vignola	tt0012860
1746491841	1947	1947	The Devil on Wheels	The Devil on Wheels	Wilbur, Crane	Crane Wilbur	tt0039318
1746583197	1930	1930	The Kibitzer	The Kibitzer	Sloman, Edward	Edward Sloman	tt0020056
1745309208	2006	2006	Al Franken: God Spoke	Al Franken: God Spoke	Doob, Nick; Hegedus, Chris	Nick Doob	tt0799916
1746585319	1930	1930	Romance	Romance	Brown, Clarence	Clarence Brown	tt0021310
1746520982	1948	1948	Urubu	Urubu	Breakston, George; Coplen, Yorke	George P. Breakston	tt0040927
1746219065	1980	1980	Hopscotch	Hopscotch	Neame, Ronald	Ronald Neame	tt0080889
1746580883	1939	1939	Stanley and Livingstone	Stanley and Livingstone	King, Henry	Henry King	tt0031973
1746419187	1993	1993	The Man Without a Face	The Man Without a Face	Gibson, Mel	Mel Gibson	tt0107501
1745302899	2005	2005	Pretty Persuasion	Pretty Persuasion	Siega, Marcos	Marcos Siega	tt0381505
1746261218	1979	1979	Manhattan	Manhattan	Allen, Woody	Woody Allen	tt0079522
1746562157	1953	1953	Law and Order	Law and Order	Juran, Nathan	Nathan Juran	tt0045991
1746394729	1989	1989	She's Out of Control	She's Out of Control	Dragoti, Stan	Stan Dragoti	tt0098308
1746570310	1934	1934	Gambling Lady	Gambling Lady	Mayo, Archie	Archie Mayo	tt0025161
1746584166	1939	1939	Captain Fury	Captain Fury	Roach, Hal	Hal Roach	tt0031137
1746524657	1927	1927	We're All Gamblers	We're All Gamblers	Cruze, James	James Cruze	tt0018550
1746524890	1915	1915	The Great Divide	The Great Divide	Lewis, Edgar	Edgar Lewis	tt0005416
1746504207	1921	1921	The Kiss	The Kiss	Conway, Jack	Jack Conway	tt0012356
1746520454	1926	1926	The Whole Town's Talking	The Whole Town's Talking	Laemmle, Edward	Edward Laemmle	tt0017554
1746503945	1919	1919	The Life Line	The Life Line	Tourneur, Maurice	Maurice Tourneur	tt0010352
1746532463	1916	1916	The Target	The Target	MacGregor, Norval	Norval MacGregor	tt0007430
1746580682	1939	1939	Blondie Brings Up Baby	Blondie Brings Up Baby	Strayer, Frank R.	Frank R. Strayer	tt0031106
1746504403	1920	1920	A Child for Sale	A Child for Sale	Abramson, Ivan	Ivan Abramson	tt0167826
1746436550	1943	1943	The Man from Music Mountain	Man from Music Mountain	Kane, Joseph	Joseph Kane	tt0036132
1746523591	1916	1916	The Light That Failed	The Light That Failed	José, Edward	Edward José	tt0005620
1746520762	1925	1925	The Cactus Cure	The Cactus Cure	Hayes, Ward	Ward Hayes	tt0015660
1746524066	1910	1910	Mazeppa; or, The Wild Horse of Tartary	Mazeppa, or the Wild Horse of Tartary		Francis Boggs	tt0001311
1745309287	2007	2007	There Will Be Blood	There Will Be Blood	Anderson, Paul Thomas	Paul Thomas Anderson	tt0469494
1746475272	1947	1947	The Corpse Came C.O.D.	The Corpse Came C.O.D.	Levin, Henry	Henry Levin	tt0039279
1746585144	1928	1928	Devil Dogs	Devil Dogs	Windermere, Fred	Fred Windemere	tt0018827
1746580861	1935	1935	Ruggles of Red Gap	Ruggles of Red Gap	McCarey, Leo	Leo McCarey	tt0026955
1746582840	1936	1936	The Blackmailer	Blackmailer	Wiles, Gordon	Gordon Wiles	tt0027368

1746185892	1969	1969	The Scavengers	The Scavengers	Frost, R. L.	Lee Frost	tt0067714
1746434011	2000	2000	Gossip	Gossip	Guggenheim, Davis	Davis Guggenheim	tt0176783
1746575007	1936	1936	Arizona Mahoney	Arizona Mahoney	Hogan, James	James P. Hogan	tt0028584
1746465136	1946	1946	Our Hearts Were Growing Up	Our Hearts Were Growing Up	Russell, William D.	William D. Russell	tt0038815
1746503354	1919	1919	The Speed Maniac	The Speed Maniac	LeSainte, Edward J.	Edward LeSaint	tt0010725
1746218417	1966	1965	The Naked Prey	The Naked Prey	Wilde, Cornel	Cornel Wilde	tt0060736
1746568360	1933	1933	Man of the Forest	Man of the Forest	Hathaway, Henry	Henry Hathaway	tt0024301
1746564806	1953	1953	Cruisin' Down the River	Cruisin' Down the River	Quine, Richard	Richard Quine	tt0045660
1746420249	1987	1987	Hiding Out	Hiding Out	Giraldi, Bob	Bob Giraldi	tt0093186
1769970498	1965	1965	Raw Love	Raw Love	Osborne, Kent	Ken Osborne	tt0194296
1746578952	1959	1959	This Earth Is Mine	This Earth Is Mine	King, Henry	Henry King	tt0053355
1746420528	1993	1993	Grumpy Old Men	Grumpy Old Men	Petrie, Donald	Donald Petrie	tt0107050
1746566207	1956	1956	Rock Rock Rock!	Rock Rock Rock!	Price, Will	Will Price	tt0049684
1745359697	2002	2003	Astoria	Æstoria	Efteriades, Nick	Leo Fialho	tt0410948
1746566222	1953	1953	Yesterday and Today	Yesterday and Today	Greshler, Abner J.	Abner J. Greshler	tt0358778
1746500769	1913	1913	Chelsea 7750	Chelsea 7750	Dawley, J. Searle	J. Searle Dawley	tt0002748
1746474982	1948	1948	The Bride Goes Wild	The Bride Goes Wild	Taurog, Norman	Norman Taurog	tt0040187
1746454101	1943	1943	The Battle of Britain	The Battle of Britain		Frank Capra	tt0182766
1746582974	1938	1938	The Gladiator	The Gladiator	Sedgwick, Edward	Edward Sedgwick	tt0030186
1746435611	1941	1941	Mob Town	Mob Town	Nigh, William	William Nigh	tt0033914
1746578709	1962	1962	Five Finger Exercise	Five Finger Exercise	Mann, Daniel	Daniel Mann	tt0055987
1746569940	1959	1959	North by Northwest	North by Northwest	Hitchcock, Alfred	Alfred Hitchcock	tt0053125
1746454634	1945	1945	Phantom of the Plains	Phantom of the Plains	Selander, Lesley	Lesley Selander	tt0037986
1746509287	1924	1924	Black Lightning	Black Lightning	Hogan, James P.	James P. Hogan	tt0014720
1746453787	1944	1944	Sweet and Low-Down	Sweet and Low Down	Mayo, Archie	Archie Mayo	tt0037333
1746393236	1984	1984	Lassiter	Lassiter	Young, Roger	Roger Young	tt0087589
1746503796	1918	1918	That Devil, Bateese	That Devil, Bateese	Wolbert, William	William Wolbert	tt0009688
1746392749	1984	1984	Splash	Splash	Howard, Ron	Ron Howard	tt0088161
1746502007	1916	1916	Dollars and the Woman	Dollars and the Woman	Kaufman, Joseph	Joseph Kaufman	tt0157549
1746508900	1927	1927	Naughty	Naughty	Del Ruth, Hampton	Hampton Del Ruth	tt0018193
1746571251	1935	1935	A Shot in the Dark	A Shot in the Dark	Lamont, Charles	Charles Lamont	tt0026991
1746491813	1946	1946	The People's Choice	The People's Choice	Fraser, Harry	Harry L. Fraser	tt0295512
1746439698	1999	1999	Instinct	Instinct	Turteltaub, Jon	Jon Turteltaub	tt0128278
1746520679	1925	1925	Shore Leave	Shore Leave	Robertson, John S.	John S. Robertson	tt0016346
1746503993	1918	1918	Painted Lips	Painted Lips	Le Saint, Edward J.	Edward LeSaint	tt0009473
1746424555	1940	1940	An Angel from Texas	An Angel from Texas	Enright, Ray	Ray Enright	tt0032207
1745341437	2002	2002	Like Mike	Like Mike	Schultz, John	John Schultz	tt0308506
1746582681	1935	1935	The Casino Murder Case	The Casino Murder Case	Marin, Edwin L.	Edwin L. Marin	tt0026187
1746570294	1933	1933	Lilly Turner	Lilly Turner	Wellman, William A.	William A. Wellman	tt0024258
1746574984	1936	1936	Murder at Glen Athol	Murder at Glen Athol	Strayer, Frank R.	Frank R. Strayer	tt0028000
1746497906	1918	1918	The House of Glass	The House of Glass	Chautard, Emile	Emile Chautard	tt0009202
1746523463	1915	1915	The Fighting Hope	The Fighting Hope	Melford, George	George Melford	tt0005323
1746569683	1957	1957	Four Boys and a Gun	Four Boys and a Gun	Berke, William	William Berke	tt0127562
1746504746	1922	1922	Beware of the Law	Beware of the Law	Douglas, W. A. S.	W.A.S. Douglas	tt0012935
1746218824	1980	1980	The Man with Bogart's Face	The Man with Bogart's Face	Day, Robert	Robert Day	tt0081110
1746394516	1986	1985	Zone Troopers	Zone Troopers	Bilson, Danny	Danny Bilson	tt0092298
1746524437	1915	1915	The Deep Purple	The Deep Purple	Young, James	James Young	tt0005185
1746410202	1984	1984	Mike's Murder	Mike's Murder	Bridges, James	James Bridges	tt0087722
1746565981	1953	1953	Houdini	Houdini	Marshall, George	George Marshall	tt0045886
1746438320	1942	1941	Paris Calling	Paris Calling	Marin, Edwin L.	Edwin L. Marin	tt0034002
1746581676	1950	1950	Radar Secret Service	Radar Secret Service	Newfield, Sam	Sam Newfield	tt0042874
1746465047	1942	1942	Dr. Gillespie's New Assistant	Dr. Gillespie's New Assistant	Goldbeck, Willis	Willis Goldbeck	tt0034680
1746573995	1962	1962	The Man Who Shot Liberty Valance	The Man Who Shot Liberty Valance	Ford, John	John Ford	tt0056217
1746393479	1983	1983	10 to Midnight	10 to Midnight	Thompson, J. Lee	J. Lee Thompson	tt0085121
1746500660	1925	1925	The Home Maker	The Home Maker	Baggot, King	King Baggot	tt0015924
1745358683	2010	2010	Extraordinary Measures	Extraordinary Measures	Vaughan, Tom	Tom Vaughan	tt1244659
1746454390	1941	1941	Man at Large	Man at Large	Forde, Eugene	Eugene Forde	tt0033876

1746564490	1951	1951	The Raging Tide	The Raging Tide	Sherman, George	George Sherman	tt0043957
1746513147	1915	1915	On the Night Stage	On the Night Stage	Barker, Reginald	Reginald Barker	tt0005832
1746583531	1931	1931	First Aid	First Aid	Paton, Stuart	Stuart Paton	tt0021869
1745342028	2010	2010	MacGruber	MacGruber	Taccone, Jorma	Jorma Taccone	tt1470023
1746575797	1932	1932	Men of America	Men of America	Ince, Ralph	Ralph Ince	tt0023208
1746582186	1929	1929	Speakeasy	Speakeasy	Stoloff, Benjamin	Benjamin Stoloff	tt0020438
1746248143	1966	1966	The Undertaker and His Pals	The Undertaker and His Pals	Swicegood, T. L. P.	T.L.P. Swicegood	tt0061140
1746582358	1929	1929	Pleasure Crazed	Pleasure Crazed	Gallaher, Donald	Donald Gallaher	tt0020275
1746251197	1975	1975	A Boy and His Dog	A Boy and His Dog	Jones, L. Q.	L.Q. Jones	tt0072730
1746465056	1942	1942	Footlight Serenade	Footlight Serenade	Ratoff, Gregory	Gregory Ratoff	tt0034745
1746524766	1914	1914	The Line-Up at Police Headquarters	The Line Up at Police Headquarters	Beal, Frank	Frank Beal	tt0004230
1746574405	1961	1961	Two Loves	Two Loves	Walters, Charles	Charles Walters	tt0055557
1745359567	2001	2001	The Royal Tenenbaums	The Royal Tenenbaums	Anderson, Wes	Wes Anderson	tt0265666
1746270017	1979	1979	Rocky II	Rocky II	Stallone, Sylvester	Sylvester Stallone	tt0079817
1746582367	1929	1929	Fancy Baggage	Fancy Baggage	Adolfi, John G.	John G. Adolfi	tt0019867
1746514816	1923	1923	Are You a Failure?	Are You a Failure?	Forman, Tom	Tom Forman	tt0013838
1746232953	1968	1968	Rosemary's Baby	Rosemary's Baby	Polanski, Roman	Roman Polanski	tt0063522
1746577953	1938	1938	Accidents Will Happen	Accidents Will Happen	Clemens, William	William Clemens	tt0029836
1746550169	1955	1955	The Seven Year Itch	The Seven Year Itch	Wilder, Billy	Billy Wilder	tt0048605
1746513472	1916	1916	The Victory of Conscience	The Victory of Conscience	Reicher, Frank	Frank Reicher	tt0007517
1746584244	1935	1935	Swell-Head	Swellhead	Stoloff, Ben	Benjamin Stoloff	tt0027066
1746410741	1982	1982	First Blood	First Blood	Kotcheff, Ted	Ted Kotcheff	tt0083944
1746570035	1934	1934	Manhattan Love Song	Manhattan Love Song	Fields, Leonard	Leonard Fields	tt0025463
1746561696	1932	1932	Tangled Fortunes	Tangled Fortunes	McGowan, J. P.	J.P. McGowan	tt0023550
1769970093	1912	1912	God's Unfortunate	God's Unfortunates		Allan Dwan	tt1326980
1746580742	1937	1937	Ever Since Eve	Ever Since Eve	Bacon, Lloyd	Lloyd Bacon	tt0028842
1746542647	1924	1924	Dynamite Dan	Dynamite Dan	Mitchell, Bruce	Bruce Mitchell	tt0014857
1746504395	1920	1920	The Furnace	The Furnace	Taylor, William D.	William Desmond Taylor	tt0011215
1746584639	1930	1930	What Men Want	What Men Want	Laemmle, Ernst	Ernst Laemmle	tt0021543
1746422034	1992	1992	Zebrahead	Zebrahead	Drazan, Anthony	Anthony Drazan	tt0105885
1746475976	1948	1948	Music Man	Music Man	Jason, Will	Will Jason	tt0040621
1746217850	1966	1966	Rings Around the World	Rings Around the World	Cates, Gilbert	Gilbert Cates	tt0060902
1746581256	1910	1910	Gold Is Not All	Gold Is Not All	Griffith, D. W.	D.W. Griffith	tt0001234
1746570427	1933	1933	Tarzan the Fearless	Tarzan the Fearless	Hill, Robert F.	Robert F. Hill	tt0024645
1746454036	1941	1941	The Monster and the Girl	The Monster and the Girl	Heisler, Stuart	Stuart Heisler	tt0033916
1746578655	1959	1959	The Crimson Kimono	The Crimson Kimono	Fuller, Samuel	Samuel Fuller	tt0052713
1746422237	1998	1998	The Players Club	The Players Club	Cube, Ice	Ice Cube	tt0119905
1746555196	1956	1955	The Beast with a Million Eyes	The Beast with a Million Eyes	Kramarsky, David	David Kramarsky	tt0048991
1745293563	2004	2004	Final Cut: The Making and Unmaking of Heaven's	Final Cut: The Making and Unmaking of Heav	Epstein, Michael	Michael Epstein	tt0424089
1746475927	1945	1945	That Night with You	That Night with You	Seiter, William A.	William A. Seiter	tt0038156
1746566098	1956	1956	The Bold and the Brave	The Bold and the Brave	Foster, Lewis R.	Lewis R. Foster	tt0049022
1746508511	1925	1925	A Kiss in the Dark	A Kiss in the Dark	Tuttle, Frank	Frank Tuttle	tt0015980
1769971151	1969	1969	The Layout	The Layout	Sarno, Joe	Joseph W. Sarno	tt0063213
1746574524	1934	1934	Many Happy Returns	Many Happy Returns	McLeod, Norman	Norman Z. McLeod	tt0025468
1746554691	1964	1964	A Tiger Walks	A Tiger Walks	Tokar, Norman	Norman Tokar	tt0058657
1746500411	1926	1926	When the Wife's Away	When the Wife's Away	Strayer, Frank R.	Frank R. Strayer	tt0017547
1746502369	1916	1916	Bobbie of the Ballet	Bobbie of the Ballet	De Grasse, Joseph	Joseph De Grasse	tt0006443
1746409973	1981	1981	Excalibur	Excalibur	Boorman, John	John Boorman	tt0082348
1746502326	1915	1915	The Winged Idol	The Winged Idol	Sidney, Scott	Scott Sidney	tt0006294
1746499941	1945	1944	Resisting Enemy Interrogation	Resisting Enemy Interrogation		Bernard Vorhaus	tt0037215
1746208678	1967	1967	The Trip	The Trip	Corman, Roger	Roger Corman	tt0062395
1746582964	1937	1937	Midnight Madonna	Midnight Madonna	Flood, James	James Flood	tt0029239
1746577027	1960	1960	The Prime Time	The Prime Time	Weisenborn, Gordon	Gordon Weisenborn	tt0054206
1746509237	1925	1925	The Mad Dancer	The Mad Dancer	King, Burton	Burton L. King	tt0016061
1745341835	2008	2008	Redbelt	Redbelt	Mamet, David	David Mamet	tt1012804
1746584410	1940	1940	'Til We Meet Again	'Til We Meet Again	Goulding, Edmund	Edmund Goulding	tt0032176
1746433564	1998	1998	The Big Lebowski	The Big Lebowski	Coen, Joel	Joel Coen	tt0118715

1746500211	1926	1926	The Sea Wolf	The Sea Wolf	Ince, Ralph	Ralph Ince	tt0017358
1746576874	1932	1932	The Vanishing Frontier	The Vanishing Frontier	Rosen, Philip E.	Phil Rosen	tt0023650
1746566071	1958	1958	Hot Rod Gang	Hot Rod Gang	Landers, Lew	Lew Landers	tt0051741
1746580881	1937	1937	Super-Sleuth	Super Sleuth	Stoloff, Ben	Benjamin Stoloff	tt0029622
1746584331	1940	1940	Men with Steel Faces	Men with Steel Faces	Brower, Otto; Eason, Breezy	Otto Brower	tt0032782
1746515097	1922	1922	Ridin' Wild	Ridin' Wild	Ross, Nat	Nat Ross	tt0013552
1746581598	1951	1951	Best of the Badmen	Best of the Badmen	Russell, William D.	William D. Russell	tt0043335
1746233863	1967	1967	The Incident	The Incident	Peerce, Larry	Larry Peerce	tt0061814
1746565878	1957	1957	Loving You	Loving You	Kanter, Hal	Hal Kanter	tt0050659
1746578956	1963	1963	Dementia 13	Dementia 13	Coppola, Francis	Francis Ford Coppola	tt0056983
1746465265	1941	1941	Two Latins from Manhattan	Two Latins from Manhattan	Barton, Charles	Charles Barton	tt0034325
1746565874	1953	1953	Jack Slade	Jack Slade	Schuster, Harold	Harold D. Schuster	tt0045925
1746584150	1935	1935	The Florentine Dagger	The Florentine Dagger	Florey, Robert	Robert Florey	tt0026369
1746566041	1958	1959	Guns, Girls and Gangsters	Guns Girls and Gangsters	Cahn, Edward L.	Edward L. Cahn	tt0052870
1746424011	1942	1942	The Omaha Trail	The Omaha Trail	Buzzell, Edward; Cahn, Edward	Edward Buzzell	tt0035149
1746577483	1950	1950	Nancy Goes to Rio	Nancy Goes to Rio	Leonard, Robert Z.	Robert Z. Leonard	tt0042779
1746216813	1968	1968	Live a Little, Love a Little	Live a Little, Love a Little	Taurog, Norman	Norman Taurog	tt0063231
1746524758	1922	1922	Confidence	Confidence	Pollard, Harry	Harry A. Pollard	tt0013023
1746437925	1942	1942	Below the Border	Below the Border	Bretherton, Howard P.	Howard Bretherton	tt0034505
1746584608	1929	1929	Wedding Rings	Wedding Rings	Beaudine, William	William Beaudine	tt0021537
1746584606	1929	1929	Rich People	Rich People	Griffith, Edward H.	Edward H. Griffith	tt0020328
1746184030	1910	1910	Billy's Sister	Billy's Sister			tt0833426
1746492718	1948	1948	The Return of October	The Return of October	Lewis, Joseph H.	Joseph H. Lewis	tt0040730
1746507600	1924	1924	Passion's Pathway	Passion's Pathway	Bracken, Bertram	Bertram Bracken	tt0015216
1746507785	1925	1925	Three Weeks in Paris	Three Weeks in Paris	Del Ruth, Roy	Roy Del Ruth	tt0017466
1746508383	1949	1949	Johnny Stool Pigeon	Johnny Stool Pigeon	Castle, William	William Castle	tt0041529
1746473718	1944	1944	Abroad with Two Yanks	Abroad with Two Yanks	Dwan, Allan	Allan Dwan	tt0036578
1746507743	1926	1926	The Hurricane	The Hurricane	Caldwell, Fred	Fred Caldwell	tt0327907
1746542754	1950	1949	Bodyhold	Bodyhold	Friedman, Seymour	Seymour Friedman	tt0041191
1746503449	1921	1921	His Brother's Keeper	His Brother's Keeper	North, Wilfrid	Wilfrid North	tt0011295
1746581049	1950	1950	Quicksand	Quicksand	Pichel, Irving	Irving Pichel	tt0042869
1746453638	1942	1942	Enemy Agents Meet Ellery Queen	Enemy Agents Meet Ellery Queen	Hogan, James	James P. Hogan	tt0034703
1746418489	1940	1940	On Their Own	On Their Own	Brower, Otto	Otto Brower	tt0032867
1746453559	1943	1942	Northwest Rangers	Northwest Rangers	Newman, Joe	Joseph M. Newman	tt0035134
1746248879	1979	1979	Scavenger Hunt	Scavenger Hunt	Schultz, Michael	Michael Schultz	tt0079858
1746422011	1997	1997	Mr. Magoo	Mr. Magoo	Tong, Stanley	Stanley Tong	tt0119718
1746574507	1934	1934	Side Streets	Side Streets	Green, Alfred E.	Alfred E. Green	tt0025788
1746581168	1910	1910	Her Terrible Ordeal	Her Terrible Ordeal	Griffith, D. W.	D.W. Griffith	tt0001245
1746217889	1967	1967	In the Heat of the Night	In the Heat of the Night	Jewison, Norman	Norman Jewison	tt0061811
1746582106	1950	1950	Night and the City	Night and the City	Dassin, Jules	Jules Dassin	tt0042788
1746474835	1946	1946	Sheriff of Redwood Valley	Sheriff of Redwood Valley	Springsteen, R. G.	R.G. Springsteen	tt0038936
1746500044	1946	1946	The Caravan Trail	The Caravan Trail	Emmett, Robert	Robert Emmett Tansey	tt0038397
1746584675	1929	1929	This Thing Called Love	This Thing Called Love	Stein, Paul L.	Paul L. Stein	tt0020494
1746573858	1960	1960	Stop! Look! and Laugh!	Stop! Look! and Laugh!	White, Jules	Don Appell	tt0173256
1746566845	1933	1933	Grand Slam	Grand Slam	Dieterle, William	William Dieterle	tt0024078
1746495805	1918	1918	Just a Woman	Just a Woman	Steger, Julius	Julius Steger	tt0184630
1746464888	1942	1942	Affairs of Jimmy Valentine	The Affairs of Jimmy Valentine	Vorhaus, Bernard	Bernard Vorhaus	tt0034432
1745357739	2004	2004	Dawn of the Dead	Dawn of the Dead	Snyder, Zack	Zack Snyder	tt0363547
1746514820	1913	1913	Caprice	Caprice		J. Searle Dawley	tt0002736
1746185161	1965	1965	The Fool Killer	The Fool Killer	González, Servando	Servando González	tt0059190
1746503307	1918	1918	She Hired a Husband	She Hired a Husband	Dillon, Jack	John Francis Dillon	tt0009602
1746520543	1927	1927	Underworld	Underworld	Sternberg, Josef von	Josef von Sternberg	tt0018526
1746514347	1927	1927	Ginsberg the Great	Ginsberg the Great	Haskin, Byron	Byron Haskin	tt0017932
1745359736	2004	2004	Saw	Saw	Wan, James	James Wan	tt0387564
1746467259	1917	1917	All for a Husband	All for a Husband	Harbaugh, Carl	Carl Harbaugh	tt0007625
1746500300	1924	1924	The Lone Chance	The Lone Chance	Mitchell, Howard	Howard M. Mitchell	tt0015078
1746438287	1941	1941	Shadows on the Stairs	Shadows on the Stairs	Lederman, D. Ross	D. Ross Lederman	tt0034173

1746563330	1952	1951	Oriental Evil	Oriental Evil	Breakston, George; Stahl, C. Ray	George P. Breakston	tt0042815
1746577508	1938	1938	Policy Man	Policy Man			tt0030596
1746234781	1973	1973	Your Three Minutes Are Up	Your Three Minutes Are Up	Schwartz, Douglas N.	Douglas Schwartz	tt0070945
1746503633	1920	1920	Voices	Voices	De Vonde, Chester	Chester De Vonde	tt0011832
1746453758	1943	1943	Princess O'Rourke	Princess O'Rourke	Krasna, Norman	Norman Krasna	tt0036277
1746564429	1953	1953	Angel Face	Angel Face	Preminger, Otto	Otto Preminger	tt0044357
1746257216	1978	1978	Jaws 2	Jaws 2	Szwarc, Jeannot	Jeannot Szwarc	tt0077766
1746574847	1935	1935	Doubting Thomas	Doubting Thomas	Butler, David	David Butler	tt0026292
1746584965	1940	1940	Pier 13	Pier 13	Forde, Eugene	Eugene Forde	tt0032908
1746218773	1973	1973	American Graffiti	American Graffiti	Lucas, George	George Lucas	tt0069704
1769970002	1911	1911	His Vacation	His Vacation		Tom Ricketts	tt1277433
1746501567	1915	1915	The Puppet Crown	The Puppet Crown	Melford, George	George Melford	tt0005935
1746473900	1945	1945	Northwest Trail	Northwest Trail	Abrahams, Derwin	Derwin Abrahams	tt0037949
1746436047	1941	1941	We Go Fast	We Go Fast	McGann, William	William C. McGann	tt0034375
1746475017	1947	1946	Big Town	Big Town	Thomas, William C.	William C. Thomas	tt0039186
1746503032	1914	1914	In the Days of the Thundering Herd	In the Days of the Thundering Herd	Campbell, Colin	Colin Campbell	tt0003724
1746569755	1958	1958	Johnny Rocco	Johnny Rocco	Landres, Paul	Paul Landres	tt0051800
1746392880	1985	1985	Fool for Love	Fool for Love	Altman, Robert	Robert Altman	tt0089160
1746543064	1916	1916	The Courtesan	The Courtesan	Maude, Arthur	Arthur Maude	tt0157487
1746580224	1936	1935	Sylvia Scarlett	Sylvia Scarlett	Cukor, George	George Cukor	tt0027067
1746582484	1938	1938	Prison Break	Prison Break	Lubin, Arthur	Arthur Lubin	tt0030625
1746437836	1944	1944	Laura	Laura	Preminger, Otto	Otto Preminger	tt0037008
1746408872	1984	1984	Moscow on the Hudson	Moscow on the Hudson	Mazursky, Paul	Paul Mazursky	tt0087747
1746435836	1992	1992	Unforgiven	Unforgiven	Eastwood, Clint	Clint Eastwood	tt0105695
1746585315	1929	1929	The Charlatan	The Charlatan	Melford, George	George Melford	tt0019757
1746210574	1973	1973	Terminal Island	Terminal Island	Rothman, Stephanie	Stephanie Rothman	tt0070782
1746574581	1937	1937	Roaring Six Guns	Roaring Six Guns	McGowan, J. P.	J.P. McGowan	tt0030686
1746577546	1934	1934	Laughing Boy	Laughing Boy	Van Dyke, W. S.	W.S. Van Dyke	tt0025371
1746503575	1920	1920	Honest Hutch	Honest Hutch	Badger, Clarence G.	Clarence G. Badger	tt0011303
1745302746	2005	2005	The Hitchhiker's Guide to the Galaxy	The Hitchhiker's Guide to the Galaxy	Jennings, Garth	Garth Jennings	tt0371724
1746433987	1998	1998	Six-String Samurai	Six String Samurai	Mungia, Lance	Lance Mungia	tt0118736
1746465233	1942	1942	The Falcon Takes Over	The Falcon Takes Over	Reis, Irving	Irving Reis	tt0034716
1746582291	1930	1930	Bright Lights	Bright Lights	Curtiz, Michael	Michael Curtiz	tt0020713
1746475657	1948	1948	Stage Struck	Stage Struck	Nigh, William	William Nigh	tt0040832
1746409279	1987	1987	The Pick-Up Artist	The Pick up Artist	Toback, James	James Toback	tt0093737
1746576938	1960	1960	Thunder in Carolina	Thunder in Carolina	Helmick, Paul	Paul Helmick	tt0054385
1746584883	1939	1939	Daughter of the Tong	Daughter of the Tong	Johnson, Raymond K.	Bernard B. Ray	tt0031211
1745302544	2005	2005	The Fog	The Fog	Wainwright, Rupert	Rupert Wainwright	tt0432291
1746524986	1927	1927	The Angel of Broadway	The Angel of Broadway	Weber, Lois	Lois Weber	tt0017630
1746524370	1914	1914	The Bargain	The Bargain	Barker, Reginald	Reginald Barker	tt0003657
1746503714	1917	1917	Her Sister	Her Sister	O'Brien, John B.	John B. O'Brien	tt0175728
1746582847	1935	1935	The Ghost Rider	The Ghost Rider	Jevne, Jack	Jack Jevne	tt0026407
1746562071	1956	1956	Screaming Eagles	Screaming Eagles	Haas, Charles	Charles F. Haas	tt0049728
1746501403	1914	1914	The Opened Shutters	The Opened Shutters	Turner, Otis	Otis Turner	tt0004435
1746585022	1940	1940	Chad Hanna	Chad Hanna	King, Henry	Henry King	tt0032321
1746209176	1967	1967	Beach Red	Beach Red	Wilde, Cornel	Cornel Wilde	tt0061389
1746424441	1941	1941	Navy Blues	Navy Blues	Bacon, Lloyd	Lloyd Bacon	tt0033942
1746512773	1912	1912	Alkali Ike's Boarding House	Alkali Ike's Boarding House	Anderson, G. M.	Gilbert M. 'Broncho Billy' Anderson	tt0002007
1746508010	1926	1926	Readin' 'Ritin' 'Rithmetic	Readin', 'Ritin', 'Rithmetic	Eddy, Robert	Robert Eddy	tt0017313
1746582449	1935	1935	Thanks a Million	Thanks a Million	Del Ruth, Roy	Roy Del Ruth	tt0027088
1746455166	1998	1997	The Only Thrill	The Only Thrill	Masterson, Peter	Peter Masterson	tt0119837
1746583408	1929	1929	False Fathers	False Fathers	Carpenter, Horace B.	Horace B. Carpenter	tt0019866
1746524988	1914	1914	The Quest of the Sacred Jewel	The Quest of the Sacred Jewel	Fitzmaurice, George	George Fitzmaurice	tt0004507
1746580417	1935	1935	Welcome Home	Welcome Home	Tinling, James	James Tinling	tt0027191
1746454821	1945	1945	The Southerner	The Southerner	Renoir, Jean	Jean Renoir	tt0038107
1745282564	2005	2005	Are We There Yet?	Are We There Yet?	Levant, Brian	Brian Levant	tt0368578
1746475959	1946	1946	Live Wires	Live Wires	Karlson, Phil	Phil Karlson	tt0038699

1746497523	1922	1922	Red Hot Romance	Red Hot Romance	Fleming, Victor	Victor Fleming	tt0013540
1746570983	1933	1933	Before Dawn	Before Dawn	Pichel, Irving	Irving Pichel	tt0023790
1746248229	1966	1965	The Devil's Mistress	The Devil's Mistress	Wanzer, Orville	Orville Wanzer	tt0126865
1746409517	1991	1991	Doc Hollywood	Doc Hollywood	Caton-Jones, Michael	Michael Caton	tt0101745
1746566072	1955	1955	Strange Lady in Town	Strange Lady in Town	LeRoy, Mervyn	Mervyn LeRoy	tt0048665
1746502791	1915	1915	The Price	The Price	Golden, Joseph A.	Joseph A. Golden	tt0005926
1746571305	1910	1910	A Dog on Business	A Dog on Business		Gilbert M. 'Broncho Billy' Anderson	tt0372242
1746566250	1953	1953	Terror on a Train	Terror on a Train	Tetzlaff, Ted	Ted Tetzlaff	tt0046432
1746560829	1932	1932	The Sport Parade	The Sport Parade	Murphy, Dudley	Dudley Murphy	tt0022918
1746584938	1940	1940	Typhoon	Typhoon	King, Louis	Louis King	tt0033196
1746580462	1939	1939	Buried Alive	Buried Alive	Halperin, Victor	Victor Halperin	tt0031126
1746420219	1983	1983	Getting It On	Getting It On	Olsen, William	William Olsen	tt0085588
1746562438	1956	1956	Comanche	Comanche	Sherman, George	George Sherman	tt0049086
1746440170	1998	1998	Fallen	Fallen	Hoblit, Gregory	Gregory Hoblit	tt0119099
1746569597	1953	1953	The Twonky	The Twonky	Oboler, Arch	Arch Oboler	tt0046475
1746563912	1952	1952	Against All Flags	Against All Flags	Sherman, George	George Sherman	tt0044333
1746485549	1917	1917	Fighting Odds	Fighting Odds	Dwan, Allan	Allan Dwan	tt0007944
1745282599	2005	2005	Protocols of Zion	Protocols of Zion	Levin, Marc	Marc Levin	tt0436686
1745396225	2010	2010	The Runaways	The Runaways	Sigismondi, Floria	Floria Sigismondi	tt1017451
1746198662	1969	1969	Midas Run	Midas Run	Kjellin, Alf	Alf Kjellin	tt0064664
1746578375	1959	1959	John Paul Jones	John Paul Jones	Farrow, John	John Farrow	tt0052946
1746524735	1914	1914	Called Back	Called Back	Turner, Otis	Henry Otto	tt3418528
1746394198	1989	1989	The Fly II	The Fly II	Walas, Chris	Chris Walas	tt0097368
1746523700	1916	1916	The Highest Bid	The Highest Bid	Prescott, Jack; Russell, William	William Russell	tt0157755
1746583190	1929	1929	The Winged Horseman	The Winged Horseman	Rosson, Arthur; Eason, Reeves	B. Reeves Eason	tt0020592
1746578469	1962	1962	The Broken Land	The Broken Land	Bushelman, John	John A. Bushelman	tt0055811
1746465079	1947	1947	Miracle on 34th Street	Miracle on 34th Street	Seaton, George	George Seaton	tt0039628
1746514683	1927	1927	The Girl in the Pullman	The Girl in the Pullman	Kenton, Erle	Erle C. Kenton	tt0017936
1746475993	1948	1947	Captain from Castile	Captain from Castile	King, Henry	Henry King	tt0039243
1746235622	1975	1975	The Master Gunfighter	The Master Gunfighter	Laughlin, Frank	Tom Laughlin	tt0073357
1746566182	1953	1953	Sins of Jezebel	Sins of Jezebel	Le Borg, Reginald	Reginald Le Borg	tt0046320
1746270726	1978	1978	Avalanche	Avalanche	Allen, Corey	Corey Allen	tt0077189
1746583905	1930	1930	Along Came Youth	Along Came Youth	Corrigan, Lloyd; McLeod, Norman	Lloyd Corrigan	tt0020634
1746560467	1965	1965	Monster A Go-Go!	Monster a Go Go	Seymour, Sheldon	Bill Rebane	tt0059464
1746524788	1928	1928	Nameless Men	Nameless Men	Cabanne, Christy	Christy Cabanne	tt0019202
1746514923	1916	1915	Dizzy Heights and Daring Hearts	Dizzy Heights and Daring Hearts	Wright, Walter	Walter Wright	tt0005204
1746514245	1922	1922	Clarence	Clarence	de Mille, William C.	William C. de Mille	tt0013016
1746567259	1932	1932	The Texas Bad Man	The Texas Bad Man	Laemmle, Edward	Edward Laemmle	tt0023565
1746533227	1915	1915	The Pearl of the Antilles	The Pearl of the Antilles	Terriss, Tom	Tom Terriss	tt0005876
1746583331	1951	1951	Cave of Outlaws	Cave of Outlaws	Castle, William	William Castle	tt0043392
1746492592	1948	1948	Song of the Drifter	Song of the Drifter	Hillyer, Lambert	Lambert Hillyer	tt0163265
1746583864	1931	1931	Laugh and Get Rich	Laugh and Get Rich	La Cava, Gregory	Gregory La Cava	tt0022056
1746393959	1988	1988	Cocoon: The Return	Cocoon: The Return	Petrie, Daniel	Daniel Petrie	tt0094890
1746500423	1949	1949	Colorado Territory	Colorado Territory	Walsh, Raoul	Raoul Walsh	tt0041253
1746585224	1930	1930	Journey's End	Journey's End	Whale, James	James Whale	tt0021013
1746465149	1944	1944	Ghost Catchers	Ghost Catchers	Cline, Eddie	Edward F. Cline	tt0036861
1746582286	1930	1930	The Big Trail	The Big Trail	Walsh, Raoul	Raoul Walsh	tt0020691
1746503029	1916	1916	Salvation Joan	Salvation Joan	North, Wilfred	Wilfrid North	tt0007284
1746218760	1973	1973	Scarecrow	Scarecrow	Schatzberg, Jerry	Jerry Schatzberg	tt0070643
1746571362	1934	1934	City Limits	City Limits	Nigh, William	William Nigh	tt0024984
1746409083	1988	1988	Clara's Heart	Clara's Heart	Mulligan, Robert	Robert Mulligan	tt0094882
1746218005	1966	1966	Nevada Smith	Nevada Smith	Hathaway, Henry	Henry Hathaway	tt0060748
1746573856	1960	1959	Teenage Zombies	Teenage Zombies	Warren, Jerry	Jerry Warren	tt0051064
1746211471	1974	1974	Newman's Law	Newman's Law	Heffron, Richard	Richard T. Heffron	tt0071902
1746570757	1952	1952	The Outcasts of Poker Flat	The Outcasts of Poker Flat	Newman, Joseph M.	Joseph M. Newman	tt0045003
1746578743	1961	1961	The Naked Edge	The Naked Edge	Anderson, Michael	Michael Anderson	tt0055214
1746259648	1978	1978	The Swarm	The Swarm	Allen, Irwin	Irwin Allen	tt0078350

1746576254	1931	1931	Compromised	Compromised	Adolfi, John G.	John G. Adolfi	tt0021757
1746465234	1946	1945	Leave Her to Heaven	Leave Her to Heaven	Stahl, John M.	John M. Stahl	tt0037865
1746497012	1920	1920	Who's Your Servant?	Who's Your Servant?			tt0011864
1746437095	2000	2000	Little Nicky	Little Nicky	Brill, Steven	Steven Brill	tt0185431
1746570441	1933	1933	Dance Girl Dance	Dance, Girl, Dance	Strayer, Frank R.	Frank R. Strayer	tt0023925
1746584189	1937	1937	Atlantic Flight	Atlantic Flight	Nigh, William	William Nigh	tt0028592
1746570444	1952	1952	The Duel at Silver Creek	The Duel at Silver Creek	Siegel, Don	Don Siegel	tt0044573
1746436940	1995	1995	Nine Months	Nine Months	Columbus, Chris	Chris Columbus	tt0113986
1746435352	1992	1992	Thunderheart	Thunderheart	Apted, Michael	Michael Apted	tt0105585
1746566097	1958	1958	How to Make a Monster	How to Make a Monster	Strock, Herbert L.	Herbert L. Strock	tt0051746
1745340151	2001	2001	Serendipity	Serendipity	Chelsom, Peter	Peter Chelsom	tt0240890
1746422580	1998	1998	Pi	Pi	Aronofsky, Darren	Darren Aronofsky	tt0138704
1746515011	1910	1910	The Wife of Marcius	The Wife of Marcius			tt0306232
1745293908	2005	2005	Rent	Rent	Columbus, Chris	Chris Columbus	tt0294870
1746533024	1915	1915	Sealed Lips	Sealed Lips	Ince, John	John Ince	tt0006014
1746578518	1959	1959	The Devil's Disciple	The Devil's Disciple	Hamilton, Guy; Mackendrick, Alexan	Guy Hamilton	tt0052735
1746473689	1946	1945	Kitty	Kitty	Leisen, Mitchell	Mitchell Leisen	tt0037849
1746420356	1990	1990	Desperate Hours	Desperate Hours	Cimino, Michael	Michael Cimino	tt0099409
1746554147	1964	1964	Walk a Tightrope	Walk a Tightrope	Nesbitt, Frank	Frank Nesbitt	tt0260526
1746584941	1939	1938	Zaza	Zaza	Cukor, George	George Cukor	tt0032157
1746577690	1933	1933	Russia Today	Russia Today	Bryan, Julien	Mrs. Carveth Wells	tt1270683
1746474515	1948	1948	Back Trail	Back Trail	Cabanne, Christy	Christy Cabanne	tt0040144
1746514629	1923	1923	The Silent Partner	The Silent Partner	Maigne, Charles	Charles Maigne	tt0014470
1746422266	1990	1990	Ghost	Ghost	Zucker, Jerry	Jerry Zucker	tt0099653
1746233014	1967	1967	The Wild Rebels	The Wild Rebels	Grefé, William	William Grefé	tt0062493
1746585083	1930	1930	The Man From Nowhere	The Man from Nowhere	McGowan, J. P.	J.P. McGowan	tt0021116
1746575798	1931	1931	Aloha	Aloha	Rogell, Albert	Albert S. Rogell	tt0021602
1746573942	1960	1960	Dalton That Got Away	The Dalton That Got Away	Salvador, Jaime	Jaime Salvador	tt0202843
1746211218	1970	1970	The Great White Hope	The Great White Hope	Ritt, Martin	Martin Ritt	tt0065797
1746455102	1998	1998	Lenny Bruce: Swear to Tell the Truth	Lenny Bruce: Swear to Tell the Truth	Weide, Robert B.	Robert B. Weide	tt0175844
1746500958	1914	1914	Mcveagh of the South Seas	McVeagh of the South Seas	Carey, Harry; Bruce, Cyril	Cyril Bruce	tt0004319
1745302534	2006	2005	Boynton Beach Club	Boynton Beach Club	Seidelman, Susan	Susan Seidelman	tt0439478
1746512043	1915	1915	The Beachcomber	The Beachcomber		Phil Rosen	tt0004950
1746394713	1984	1984	Teachers	Teachers	Hiller, Arthur	Arthur Hiller	tt0088242
1746209161	1967	1967	Ski on the Wild Side	Ski on the Wild Side	Miller, Warren	Warren Miller	tt0062274
1746520346	1926	1926	Gasoline Cowboy	Gasoline Cowboy	Reel, Frederick	Frederick Reel Jr.	tt0325450
1746409079	1985	1985	The Goonies	The Goonies	Donner, Richard	Richard Donner	tt0089218
1746454610	1940	1940	Three Faces West	Three Faces West	Vorhaus, Bernard	Bernard Vorhaus	tt0033159
1746508545	1925	1925	The Desert Flower	The Desert Flower	Cummings, Irving	Irving Cummings	tt0015745
1746566079	1955	1955	Love Is a Many-Splendored Thing	Love Is a Many Splendored Thing	King, Henry	Henry King	tt0048316
1746410164	1989	1989	Parenthood	Parenthood	Howard, Ron	Ron Howard	tt0098067
1746394658	1983	1983	Mausoleum	Mausoleum	Dugan, Michael	Michael Dugan	tt0085918
1746500253	1926	1926	Pals First	Pals First	Carewe, Edwin	Edwin Carewe	tt0017245
1746499948	1923	1923	The Forbidden Trail	The Forbidden Trail	Bradbury, Robert North	Robert N. Bradbury	tt0132973
1746523418	1910	1910	Love Among the Roses	Love Among the Roses	Griffith, D. W.	D.W. Griffith	tt0001292
1746408679	1984	1984	Cheech & Chong's The Corsican Brothers	Cheech & Chong's The Corsican Brothers	Chong, Thomas	Tommy Chong	tt0087042
1746501214	1911	1911	An Arizona Romance	An Arizona Romance	Beal, Frank	Frank Beal	tt1164547
1746584835	1929	1929	Trent's Last Case	Trent's Last Case	Hawks, Howard	Howard Hawks	tt0020513
1746453476	1942	1942	Red River Robin Hood	Red River Robin Hood	Selander, Lesley	Lesley Selander	tt0036296
1746532850	1914	1914	The Governor's Ghost	The Governor's Ghost	Davis, Will S.	Will S. Davis	tt0004031
1746424420	1940	1940	Alias the Deacon	Alias the Deacon	Cabanne, Christy	Christy Cabanne	tt0032191
1746492714	1944	1944	Going My Way	Going My Way	McCarey, Leo	Leo McCarey	tt0036872
1746574201	1960	1959	A Dog's Best Friend	A Dog's Best Friend	Cahn, Edward L.	Edward L. Cahn	tt0053776
1746473931	1943	1943	Yanks Ahoy	Yanks Ahoy	Neumann, Kurt	Kurt Neumann	tt0036548
1746582960	1938	1938	Passport Husband	Passport Husband	Tinling, James	James Tinling	tt0030562
1746503718	1922	1922	The World's a Stage	The World's a Stage	Campbell, Colin	Colin Campbell	tt0013791
1746562280	1953	1953	Gun Fury	Gun Fury	Walsh, Raoul	Raoul Walsh	tt0045846

1746574613	1935	1935	The Man from Guntown	The Man from Guntown	Beebe, Ford	Ford Beebe	tt0026673
1746466559	1917	1917	A Modern Monte Cristo	A Modern Monte Cristo	Moore, W. Eugene	Eugene Moore	tt0178759
1746464902	1941	1941	Mystery Ship	Mystery Ship	Landers, Lew	Lew Landers	tt0033934
1746575055	1934	1934	A Very Honorable Guy	A Very Honorable Guy	Bacon, Lloyd	Lloyd Bacon	tt0025945
1746455958	1995	1995	Sabrina	Sabrina	Pollack, Sydney	Sydney Pollack	tt0114319
1746514365	1927	1927	A Hero for a Night	A Hero for a Night	Craft, William James	William James Craft	tt0014985
1746585055	1929	1929	Meet The Missus	Meet the Missus	Gillstrom, Arvid E.	Arvid E. Gillstrom	tt0447285
1746208295	1967	1967	Bikini Paradise	Bikini Paradise	Tallas, Gregg	Gregg G. Tallas	tt0058970
1746500224	1924	1924	Western Feuds	Western Feuds	Ford, Francis	Francis Ford	tt0232931
1746584232	1935	1935	Behind the Evidence	Behind the Evidence	Hillyer, Lambert	Lambert Hillyer	tt0024871
1746577627	1937	1937	Reckless Ranger	Reckless Ranger	Bennet, Spencer Gordon	Spencer Gordon Bennet	tt0029468
1746582658	1937	1937	Amateur Crook	Amateur Crook	Katzman, Sam	Sam Katzman	tt0028574
1746436637	1941	1941	Here Comes Happiness	Here Comes Happiness	Smith, Noel M.	Noel M. Smith	tt0033711
1746580430	1935	1935	Obeah!	Obeah	Herrick, F. Herrick	F. Herrick Herrick	tt0026800
1746393423	1984	1984	The Philadelphia Experiment	The Philadelphia Experiment	Raffill, Stewart	Stewart Raffill	tt0087910
1746574533	1935	1935	Arizona Trails	Arizona Trails	James, Alan	Victor Adamson	tt0026081
1746420133	1993	1993	Three of Hearts	Three of Hearts	Bogayevicz, Yurek	Yurek Bogayevicz	tt0108334
1746199807	1966	1966	Hot Rod Hullabaloo	Hot Rod Hullabaloo	Naud, William T.	William T. Naud	tt0060518
1746504203	1918	1918	A Broadway Scandal	A Broadway Scandal	De Grasse, Joseph	Joseph De Grasse	tt0008931
1746564497	1951	1950	One Too Many	One Too Many	Kenton, Erle C.	Erle C. Kenton	tt0042808
1746509192	1924	1924	Traffic in Hearts	Traffic in Hearts	Dunlap, Scott	Scott R. Dunlap	tt0015424
1746573607	1932	1932	Between Fighting Men	Between Fighting Men	Sheldon, Forrest	Forrest Sheldon	tt0022673
1746438459	1944	1943	Around the World	Around the World	Dwan, Allan	Allan Dwan	tt0035648
1746570440	1933	1933	Riders of Destiny	Riders of Destiny	Bradbury, Robert N.	Robert N. Bradbury	tt0024500
1746554427	1964	1964	Moonshine Mountain	Moonshine Mountain	Lewis, Herschell Gordon	Herschell Gordon Lewis	tt0058372
1746574851	1934	1934	Love Past Thirty	Love Past Thirty	Moore, Vin	Vin Moore	tt0025430
1746524599	1913	1913	Hoodman Blind	Hoodman Blind	Gordon, James	James Gordon	tt0002978
1746503426	1917	1917	Who Shall Take My Life?	Who Shall Take My Life?	Campbell, Colin	Colin Campbell	tt0009800
1746525158	1921	1921	The Range Pirate	The Range Pirate	Franchon, Leonard	Leonard Franchon	tt0012608
1746583244	1951	1951	Two Gals and a Guy	Two Gals and a Guy	Green, Alfred E.	Alfred E. Green	tt0044156
1746439131	1941	1941	The Eternal Gift	The Eternal Gift		Hugh Calkins	tt0033579
1746560622	1932	1932	Madame Racketeer	Madame Racketeer	Hall, Alexander; Gribble, Harry Wags	Harry Wagstaff Gribble	tt0023170
1746563631	1953	1953	Bandits of the West	Bandits of the West	Keller, Harry	Harry Keller	tt0045540
1746500131	1946	1946	Death Valley	Death Valley	Landers, Lew	Lew Landers	tt0038460
1746577944	1938	1938	Crashing Hollywood	Crashing Hollywood	Landers, Lew	Lew Landers	tt0030022
1746486187	1918	1918	A Pair of Silk Stockings	A Pair of Silk Stockings	Edwards, Walter	Walter Edwards	tt0009475
1746491806	1946	1946	Shadow of a Woman	Shadow of a Woman	Santley, Joseph	Joseph Santley	tt0038927
1746515824	1920	1920	Occasionally Yours	Occasionally Yours	Horne, James W.	James W. Horne	tt0196807
1746410548	1990	1990	State of Grace	State of Grace	Joanou, Phil	Phil Joanou	tt0100685
1746418704	1940	1939	A Child Is Born	A Child Is Born	Bacon, Lloyd	Lloyd Bacon	tt0032333
1745359743	2002	2002	Serving Sara	Serving Sara	Hudlin, Reginald	Reginald Hudlin	tt0261289
1745357922	2003	2003	Capturing the Friedmans	Capturing the Friedmans	Jarecki, Andrew	Andrew Jarecki	tt0342172
1746512747	1923	1923	Yesterday's Wife	Yesterday's Wife	Le Saint, Edward J.	Edward LeSaint	tt0014628
1746525066	1920	1920	Shod with Fire	Shod with Fire	Flynn, Emmett J.	Emmett J. Flynn	tt0011688
1746467859	1917	1917	The Mad Lover	The Mad Lover	Perret, Léonce	Léonce Perret	tt0008239
1746569775	1954	1954	Alaska Seas	Alaska Seas	Hopper, Jerry	Jerry Hopper	tt0046692
1746524205	1917	1917	The Candy Girl	The Candy Girl	Moore, W. Eugene	Eugene Moore	tt0168565
1746524807	1923	1923	The Broad Road	The Broad Road	Mortimer, Edmund	Edmund Mortimer	tt0013893
1746422628	1993	1993	The Crush	The Crush	Shapiro, Alan	Alan Shapiro	tt0106627
1746520152	1926	1926	The Midnight Sun	The Midnight Sun	Buchowetski, Dimitri	Dimitri Buchowetzki	tt0017144
1745293245	2006	2005	Edmond	Edmond	Gordon, Stuart	Stuart Gordon	tt0443496
1746580586	1937	1937	Wallaby Jim of the Islands	Wallaby Jim of the Islands	Lamont, Charles	Charles Lamont	tt0029745
1746219207	1972	1972	Joe Kidd	Joe Kidd	Sturges, John	John Sturges	tt0068768
1746436367	1941	1940	Michael Shayne, Private Detective	Michael Shayne: Private Detective	Forde, Eugene	Eugene Forde	tt0032788
1746410012	1992	1992	The Babe	The Babe	Hiller, Arthur	Arthur Hiller	tt0103747
1746583195	1930	1930	Bride of the Regiment	Bride of the Regiment	Dillon, John Francis	John Francis Dillon	tt0020712
1746508155	1949	1949	Trails End	Trails End	Hillyer, Lambert	Lambert Hillyer	tt0122288

1745282633	2006	2005	Commune	Commune	Berman, Jonathan	Jonathan Berman	tt0439511
1746584039	1935	1935	Here Comes Cookie	Here Comes Cookie	McLeod, Norman	Norman Z. McLeod	tt0026465
1746574199	1960	1960	Exodus	Exodus	Preminger, Otto	Otto Preminger	tt0053804
1746248848	1980	1980	Carny	Carny	Kaylor, Robert	Robert Kaylor	tt0080500
1746270417	1978	1978	Magic	Magic	Attenborough, Richard	Richard Attenborough	tt0077889
1746567111	1952	1952	Just Across the Street	Just Across the Street	Pevney, Joseph	Joseph Pevney	tt0044780
1746577642	1935	1935	After the Dance	After the Dance	Bulgakov, Leo	Leo Bulgakov	tt0026044
1746394005	1985	1985	Barbarian Queen	Barbarian Queen	Olivera, Héctor	Héctor Olivera	tt0088771
1746493535	1910	1910	What the Daisy Said	What the Daisy Said	Griffith, D. W.	D.W. Griffith	tt0001452
1746464835	1941	1941	Pals of the Pecos	Pals of the Pecos	Orlebeck, Les	Lester Orlebeck	tt0033995
1746500242	1924	1924	The Breed of the Border	Breed of the Border	Garson, Harry	Harry Garson	tt0014741
1746456079	1997	1997	Gone Fishin'	Gone Fishin'	Cain, Christopher	Christopher Cain	tt0119214
1746393660	1988	1988	Colors	Colors	Hopper, Dennis	Dennis Hopper	tt0094894
1746570438	1934	1933	Twin Husbands	Twin Husbands	Strayer, Frank R.	Frank R. Strayer	tt0025921
1746493616	1912	1912	Cleopatra	Cleopatra	Gaskill, Charles L.	Charles L. Gaskill	tt0002101
1746532814	1916	1916	The Crippled Hand	The Crippled Hand	Leonard, Robert; Kirkland, David	David Kirkland	tt0006556
1746453927	1997	1997	Vegas Vacation	Vegas Vacation	Kessler, Stephen	Stephen Kessler	tt0120434
1746569757	1954	1954	Betrayed	Betrayed	Reinhardt, Gottfried	Gottfried Reinhardt	tt0046770
1746574618	1935	1935	Confidential	Confidential	Cahn, Edward L.	Edward L. Cahn	tt0026231
1746573565	1932	1932	The Phantom President	The Phantom President	Taurog, Norman	Norman Taurog	tt0023334
1746569614	1953	1953	Tumbleweed	Tumbleweed	Juran, Nathan	Nathan Juran	tt0046469
1746483490	1917	1917	Her Secret	Her Secret	Vekroff, Perry N.	Perry N. Vekroff	tt0175727
1769970616	1968	1968	Key Club Wives	Key Club Wives	Clay, Adam	Adam Clay	tt0222106
1746581833	1928	1928	Taxi 13	Taxi 13	Neilan, Marshall	Marshall Neilan	tt0019448
1746500405	1925	1925	The Business of Love	The Business of Love	Robbins, Jesse	Irving Reis	tt0015655
1746570884	1933	1933	Good-bye Love	Goodbye Love	Humberstone, H. Bruce	H. Bruce Humberstone	tt0025194
1746392855	1985	1985	The Breakfast Club	The Breakfast Club	Hughes, John	John Hughes	tt0088847
1746524830	1923	1923	Your Friend and Mine	Your Friend and Mine	Badger, Clarence G.	Clarence G. Badger	tt0014633
1746570856	1934	1934	Fugitive Lovers	Fugitive Lovers	Boleslavsky, Richard	Richard Boleslawski	tt0025151
1746496422	1918	1918	Berlin Via America	Berlin Via America	Ford, Francis	Francis Ford	tt0182783
1746554318	1963	1963	Bye Bye Birdie	Bye Bye Birdie	Sidney, George	George Sidney	tt0056891
1746562193	1956	1956	Calling Homicide	Calling Homicide	Bernds, Edward	Edward Bernds	tt0049044
1746423461	1991	1991	Shattered	Shattered	Petersen, Wolfgang	Wolfgang Petersen	tt0102900
1746570571	1934	1934	I Can't Escape	I Can't Escape	Brower, Otto	Otto Brower	tt0165322
1746454477	1942	1942	The Great Man's Lady	The Great Man's Lady	Wellman, William A.	William A. Wellman	tt0034812
1746524794	1917	1917	The Bondage of Fear	The Bondage of Fear	Vale, Travers	Travers Vale	tt0168531
1746513629	1916	1916	Feathertop	Feathertop	Vernot, Henry	Henry J. Vernot	tt0157623
1746186340	1967	1966	Chappaqua	Chappaqua	Rooks, Conrad	Conrad Rooks	tt0059025
1746409126	1988	1988	Willow	Willow	Howard, Ron	Ron Howard	tt0096446
1746216725	1968	1968	Maryjane	Maryjane	Dexter, Maury	Maury Dexter	tt0063281
1746423262	1997	1997	The Devil's Advocate	The Devil's Advocate	Hackford, Taylor	Taylor Hackford	tt0118971
1746577609	1938	1938	Brother Rat	Brother Rat	Keighley, William	William Keighley	tt0029949
1746420529	1994	1994	Beverly Hills Cop III	Beverly Hills Cop III	Landis, John	John Landis	tt0109254
1746218819	1973	1973	Black Mama, White Mama	Black Mama White Mama	Romero, Eddie	Eddie Romero	tt0068282
1746569985	1958	1958	The Restless Years	The Restless Years	Kautner, Helmut	Helmut Käutner	tt0052129
1746560730	1932	1932	Manhattan Tower	Manhattan Tower	Strayer, Frank	Frank R. Strayer	tt0023187
1746474944	1945	1945	Thrill of a Romance	Thrill of a Romance	Thorpe, Richard	Richard Thorpe	tt0038170
1746563846	1953	1953	The Beast from 20,000 Fathoms	The Beast from 20,000 Fathoms	Lourié, Eugene	Eugène Lourié	tt0045546
1746499906	1923	1923	The Go-Getter	The Go Getter	Griffith, E. H.	Edward H. Griffith	tt0014087
1746582757	1935	1935	The Irish in Us	The Irish in Us	Bacon, Lloyd	Lloyd Bacon	tt0026534
1746585352	1930	1930	Near the Rainbow's End	Near the Rainbow's End	McGowan, J. P.	J.P. McGowan	tt0021176
1746424428	1941	1941	The Devil Commands	The Devil Commands	Dmytryk, Edward	Edward Dmytryk	tt0033530
1746420952	1940	1940	The Man Who Talked Too Much	The Man Who Talked Too Much	Sherman, Vincent	Vincent Sherman	tt0032747
1746218758	1974	1974	Thunderbolt and Lightfoot	Thunderbolt and Lightfoot	Cimino, Michael	Michael Cimino	tt0072288
1746569857	1957	1957	Undersea Girl	Undersea Girl	Peyser, John	John Peyser	tt0051131
1746574841	1933	1933	Above the Clouds	Above the Clouds	Neill, Roy William	Roy William Neill	tt0024811
1746497891	1922	1922	Shattered Dreams	Shattered Dreams	Scardon, Paul	Paul Scardon	tt0013594

1746496045	1918	1918	The Legion of Death	The Legion of Death	Browning, Tod	Tod Browning	tt0009284	
1746503729	1921	1921	The Last Door	The Last Door	Earle, William P. S.	William P.S. Earle	tt0012378	
1746570324	1934	1934	Sweet Adeline	Sweet Adeline	Le Roy, Mervyn	Mervyn LeRoy	tt0025850	
1746393179	1986	1985	Pray for Death	Pray for Death	Hessler, Gordon	Gordon Hessler	tt0089835	
1746421709	1999	1999	Wild Wild West	Wild Wild West	Sonnenfeld, Barry	Barry Sonnenfeld	tt0120891	
1746523694	1916	1916	The Ne'er-Do-Well	The Ne'er Do Well	Campbell, Colin	Colin Campbell	tt0007117	
1746422270	1999	1999	Mystery Men	Mystery Men	Usher, Kinka	Kinka Usher	tt0132347	
1746562435	1957	1957	The Invisible Boy	The Invisible Boy	Hoffman, Herman	Herman Hoffman	tt0050546	
1746491749	1947	1947	The Sea of Grass	The Sea of Grass	Kazan, Elia	Elia Kazan	tt0039806	
1745293368	2007	2007	The Darjeeling Limited	The Darjeeling Limited	Anderson, Wes	Wes Anderson	tt0838221	
1746574759	1937	1937	Man of the People	Man of the People	Marin, Edwin L.	Edwin L. Marin	tt0029206	
1746500349	1924	1924	Floodgates	Floodgates	Irving, George	George Irving	tt0014913	
1746423743	1992	1992	We're Talkin' Serious Money	We're Talkin' Serious Money	Lemmo, James	James Lemmo	tt0108528	
1746578393	1959	1959	Date with Death	Date with Death	Daniels, Harold	Harold Daniels	tt0124336	
1746473930	1947	1947	Road to the Big House	Road to the Big House	Colmes, Walter	Walter Colmes	tt0039777	
1746582002	1929	1929	Outlawed	Outlawed	Forde, Eugene	Eugene Forde	tt0020249	
1746498347	1920	1920	In Folly's Trail	In Folly's Trail	Sturgeon, Rollin	Rollin S. Sturgeon	tt0011328	
1746186223	1968	1968	Sol Madrid	Sol Madrid	Hutton, Brian G.	Brian G. Hutton	tt0063621	
1746503272	1919	1919	A White Man's Chance	A White Man's Chance	Warde, Ernest C.	Ernest C. Warde	tt0194536	
1746532643	1916	1916	Divorce and the Daughter	Divorce and the Daughter	Sullivan, Frederick	Frederick Sullivan	tt0157543	
1746574733	1937	1937	Wings over Honolulu	Wings Over Honolulu	Potter, H. C.	H.C. Potter	tt0029784	
1746566049	1957	1957	Kiss Them for Me	Kiss Them for Me	Donen, Stanley	Stanley Donen	tt0050599	
1746575787	1931	1931	Command Performance	Command Performance	Lang, Walter	Walter Lang	tt0020780	
1746520869	1925	1925	Savages of the Sea	Savages of the Sea	Mitchell, Bruce	Bruce Mitchell	tt0016314	
1746515678	1920	1920	Crooked Streets	Crooked Streets	Powell, Paul	Paul Powell	tt0011079	
1746581130	1960	1960	I Passed for White	I Passed for White	Wilcox, Fred M.	Fred M. Wilcox	tt0053933	
1746433573	1995	1995	Party Girl	Party Girl	Mayer, Daisy von Scherler	Daisy von Scherler Mayer	tt0114095	
1746508451	1925	1925	Winning a Woman	Winning a Woman		Harry S. Webb	tt0180307	
1746508238	1949	1949	The Sun Comes Up	The Sun Comes Up	Thorpe, Richard	Richard Thorpe	tt0040849	
1746392747	1985	1985	Perfect	Perfect	Bridges, James	James Bridges	tt0089798	
**1746474110**	**1946**	**1946**	**Of Human Bondage**	**Of Human Bondage**	**Goulding, Edmund**	**Edmund Goulding**	**tt0038795**	1
1746464813	1941	1941	A Girl, a Guy and a Gob	A Girl, a Guy, and a Gob	Wallace, Richard	Richard Wallace	tt0033664	
1746454643	1946	1946	Behind the Mask	Behind the Mask	Karlson, Phil	Phil Karlson	tt0038347	
1746497884	1921	1921	His Nibs	His Nibs	La Cava, Gregory	Gregory La Cava	tt0012284	
1746582578	1938	1938	Young Dr. Kildare	Young Dr. Kildare	Bucquet, Harold S.	Harold S. Bucquet	tt0031000	
1746464977	1941	1940	The Philadelphia Story	The Philadelphia Story	Cukor, George	George Cukor	tt0032904	
1746545237	1940	1940	Star Dust	Star Dust	Lang, Walter	Walter Lang	tt0033100	
1746501893	1916	1916	A Huntress of Men	The Huntress of Men	Henderson, Lucius	Lucius Henderson	tt0006837	
1746570984	1933	1933	The Whirlwind	The Whirlwind	Lederman, D. Ross	D. Ross Lederman	tt0024767	
1746582963	1936	1936	Captain Calamity	Captain Calamity	Reinhardt, John	John Reinhardt	tt0027420	
1746582128	1929	1930	Fighting Cowboy	Fighting Cowboy			tt0325373	
1746258713	1980	1980	Schizoid	Schizoid	Paulsen, David	David Paulsen	tt0081459	
1745395387	2010	2009	Today's Special	Today's Special	Kaplan, David	David Kaplan	tt1153053	
1746561341	1957	1957	The Disembodied	The Disembodied	Grauman, Walter	Walter Grauman	tt0050317	
1746514289	1922	1922	The Crossroads of New York	The Crossroads of New York	Jones, F. Richard	F. Richard Jones	tt0013038	
1746198101	1968	1968	What's So Bad About Feeling Good?	What's So Bad About Feeling Good?	Seaton, George	George Seaton	tt0063799	
1746394519	1985	1985	The Journey of Natty Gann	The Journey of Natty Gann	Kagan, Jeremy	Jeremy Kagan	tt0089385	
1746507878	1923	1923	The Hero	The Hero	Gasnier, Louis J.	Louis J. Gasnier	tt0014132	
1746507821	1927	1927	The Nest	The Nest	Nigh, William	William Nigh	tt0018198	
1746439179	1999	1999	Angela's Ashes	Angela's Ashes	Parker, Alan	Alan Parker	tt0145653	
1746491804	1946	1946	Partners in Time	Partners in Time	Nigh, William	William Nigh	tt0038827	
1746567426	1933	1933	Berkeley Square	Berkeley Square	Lloyd, Frank	Frank Lloyd	tt0023794	
1746563790	1951	1951	The Steel Helmet	The Steel Helmet	Fuller, Samuel	Samuel Fuller	tt0044072	
1745282720	2006	2006	Charlotte's Web	Charlotte's Web	Winick, Gary	Gary Winick	tt0413895	
1746582977	1936	1936	Under Your Spell	Under Your Spell	Preminger, Otto Ludwig	Otto Preminger	tt0028443	
1746585167	1929	1929	Linda	Linda	Reid, Wallace	Dorothy Davenport	tt0020099	
1746408880	1990	1990	Eating	Eating	Jaglom, Henry	Henry Jaglom	tt0099480	

1746577338	1962	1962	Sergeants 3	Sergeants 3	Sturges, John	John Sturges	tt0056470
1746495582	1917	1917	The Corner Grocer	The Corner Grocer	Cowl, George	George Cowl	tt0007821
1746235028	1971	1971	Chandler	Chandler	Magwood, Paul	Paul Magwood	tt0068356
1746435705	1943	1943	Deerslayer	The Deerslayer	Landers, Lew	Lew Landers	tt0035791
1746569717	1956	1956	The Man Who Never Was	The Man Who Never Was	Neame, Ronald	Ronald Neame	tt0049471
1746514068	1916	1916	The Social Secretary	The Social Secretary	Emerson, John	John Emerson	tt0007367
1746508235	1924	1924	The Pell Street Mystery	The Pell Street Mystery	Franz, Joseph	Joseph Franz	tt0148593
1746393388	1989	1989	Bert Rigby, You're a Fool	Bert Rigby, You're a Fool	Reiner, Carl	Carl Reiner	tt0096911
1746500704	1924	1924	Not Built for Runnin'	Not Built for Runnin'	Maloney, Leo	Leo D. Maloney	tt0142702
1746486294	1917	1917	The Beautiful Lie	The Beautiful Lie	Noble, John W.	John W. Noble	tt0167033
1746453563	1941	1941	The Saint's Vacation	The Saint's Vacation	Fenton, Leslie	Leslie Fenton	tt0034146
1769970588	1966	1966	The Girl From S.I.N.	The Girl from S.I.N.	Smith, C. Davis	C. Davis Smith	tt0196588
1746581773	1928	1928	The Sky Rider	The Sky Rider	Neitz, Alvin J.	Alan James	tt0019396
1746583290	1929	1929	New York Nights	New York Nights	Milestone, Lewis	Lewis Milestone	tt0020217
1746419593	1981	1980	Blood Beach	Blood Beach	Bloom, Jeffrey	Jeffrey Bloom	tt0082083
1746513072	1910	1910	Simpson's Skate	Simpson's Skate			tt1717684
1746454378	1946	1946	Canyon Passage	Canyon Passage	Tourneur, Jacques	Jacques Tourneur	tt0038395
1746514873	1916	1916	A Modern Paul	A Modern Paul	Mayo, Melvin	Melvin Mayo	tt0817221
1746423778	1940	1940	Castle on the Hudson	Castle on the Hudson	Litvak, Anatole	Anatole Litvak	tt0032315
1746501220	1916	1916	Peggy	Peggy	Ince, Thomas H.; Giblyn, Charles	Charles Giblyn	tt0007167
1746497546	1918	1918	Wild Honey	Wild Honey	Grandon, Francis J.	Francis J. Grandon	tt0190142
1746233298	1966	1966	That Tennessee Beat	That Tennessee Beat	Brill, Richard	Richard Brill	tt0232791
1746582189	1928	1928	Phyllis of the Follies	Phyllis of the Follies	Laemmle, Ernst	Ernst Laemmle	tt0019272
1746583565	1950	1950	Once a Thief	Once a Thief	Wilder, W. Lee	W. Lee Wilder	tt0042807
1769971956	1969	1969	The Stud Farm	The Stud Farm	Zacha, Jac	Jac Zacha	tt0065044
1746408426	1990	1990	Avalon	Avalon	Levinson, Barry	Barry Levinson	tt0099073
1746520531	1927	1926	Oh, Baby	Oh, Baby!	Knoles, Harley	Harley Knoles	tt0017224
1746467158	1917	1917	Chicken Casey	Chicken Casey	West, Raymond B.	Raymond B. West	tt0168577
1769974349	1916	1916	Jerry's Double Header	Jerry's Double Header	Fahrney, Milton	Milton J. Fahrney	tt0484681
1746530676	1949	1949	Everybody Does It	Everybody Does It	Goulding, Edmund	Edmund Goulding	tt0041341
1745372652	2003	2003	Seabiscuit	Seabiscuit	Ross, Gary	Gary Ross	tt0329575
1746524933	1923	1923	Puritan Passions	Puritan Passions	Tuttle, Frank	Frank Tuttle	tt0014382
1746473732	1942	1942	'Neath Brooklyn Bridge	'Neath Brooklyn Bridge	Fox, Wallace	Wallace Fox	tt0034420
1745342676	2002	2002	The Transporter	The Transporter	Yuen, Cory	Louis Leterrier	tt0293662
1745293311	2006	2006	ATL	ATL	Robinson, Chris	Chris Robinson	tt0466856
1746409360	1982	1982	Six Pack	Six Pack	Petrie, Daniel	Daniel Petrie	tt0084690
1745293983	2005	2005	The Squid and the Whale	The Squid and the Whale	Baumbach, Noah	Noah Baumbach	tt0367089
1746572434	1931	1931	City Streets	City Streets	Mamoulian, Rouben	Rouben Mamoulian	tt0021750
1746508604	1924	1924	The Hellion	The Hellion	Mitchell, Bruce	Bruce Mitchell	tt0014979
1746580553	1935	1935	Love Me Forever	Love Me Forever	Schertzinger, Victor	Victor Schertzinger	tt0026654
1746501302	1910	1910	Winning Back His Love	Winning Back His Love	Griffith, D. W.	D.W. Griffith	tt0001459
1746574292	1958	1958	Suicide Battalion	Suicide Battalion	Cahn, Edward L.	Edward L. Cahn	tt0052257
1746492752	1946	1946	O.S.S.	O.S.S.	Pichel, Irving	Irving Pichel	tt0038794
1746574566	1937	1937	Outlaws of the Orient	Outlaws of the Orient	Schoedsack, Ernest B.	Ernest B. Schoedsack	tt0029363
1746574837	1935	1935	The Nitwits	The Nitwits	Stevens, George	George Stevens	tt0026781
1746573828	1960	1960	Song Without End	Song Without End	Vidor, Charles; Cukor, George	Charles Vidor	tt0054324
1746577847	1939	1939	Days of Jesse James	Days of Jesse James	Kane, Joseph	Joseph Kane	tt0031216
1745358528	2010	2010	My Soul to Take	My Soul to Take	Craven, Wes	Wes Craven	tt0872230
1746570384	1934	1934	The Girl from Missouri	The Girl from Missouri	Conway, Jack; Wood, Sam	Jack Conway	tt0025173
1746453524	1943	1943	The Ghost Ship	The Ghost Ship	Robson, Mark	Mark Robson	tt0035937
1746208393	1967	1967	The Flim-Flam Man	The Flim Flam Man	Kershner, Irvin	Irvin Kershner	tt0061678
1746530679	1949	1949	Across the Rio Grande	Across the Rio Grande	Drake, Oliver	Oliver Drake	tt0041087
1746494169	1910	1910	The Taming of Wild Bill	The Taming of Wild Bill			tt1816694
1746555881	1957	1957	The Amazing Colossal Man	The Amazing Colossal Man	Gordon, Bert I.	Bert I. Gordon	tt0050118
1746497564	1921	1921	Wet Gold	Wet Gold	Ince, Ralph	Ralph Ince	tt0012826
1746453605	1941	1941	Bad Man of Deadwood	Bad Man of Deadwood	Kane, Joseph	Joseph Kane	tt0033367
1769969354	1964	1963	Six Shes and a He	Six She's and a He		Richard S. Flink	tt0304355

1746553966	1964	1964	Two Thousand Maniacs!	Two Thousand Maniacs!	Lewis, Herschell G.	Herschell Gordon Lewis	tt0058694
1746560638	1965	1965	Harlow	Harlow	Segal, Alex	Gordon Douglas	tt0059252
1746577278	1961	1961	Susan Slade	Susan Slade	Daves, Delmer	Delmer Daves	tt0055490
1746578996	1961	1961	Tammy Tell Me True	Tammy Tell Me True	Keller, Harry	Harry Keller	tt0055503
1746496508	1919	1919	The Darkest Hour	The Darkest Hour	Scardon, Paul	Paul Scardon	tt0190350
1746219640	1976	1976	All the President's Men	All the President's Men	Pakula, Alan J.	Alan J. Pakula	tt0074119
1746474135	1948	1948	Racing Luck	Racing Luck	Berke, William	William Berke	tt0126630
1746409303	1989	1989	Mutant on the Bounty	Mutant on the Bounty	Torrance, Robert	Robert Torrance	tt0097934
1746524539	1915	1915	Are You a Mason?	Are You a Mason?	Heffron, Thomas N.	Thomas N. Heffron	tt0004907
1746583287	1931	1931	Lonely Wives	Lonely Wives	Mack, Russell	Russell Mack	tt0022086
1746565038	1953	1952	Botany Bay	Botany Bay	Farrow, John	John Farrow	tt0045574
1746508024	1927	1927	The Mojave Kid	The Mojave Kid	Bradbury, Robert North	Robert N. Bradbury	tt0018169
1746582126	1930	1930	Isle of Escape	Isle of Escape	Bretherton, Howard	Howard Bretherton	tt0021003
1746500473	1949	1949	Leave It to Henry	Leave It to Henry	Yarbrough, Jean	Jean Yarbrough	tt0041586
1746421288	1999	1999	Splendor	Splendor	Araki, Gregg	Gregg Araki	tt0127296
1746569866	1956	1956	The Man Is Armed	The Man Is Armed	Adreon, Franklin	Franklin Adreon	tt0049469
1745341231	2008	2007	My Name Is Bruce	My Name Is Bruce	Campbell, Bruce	Bruce Campbell	tt0489235
1746393994	1988	1988	Big	Big	Marshall, Penny	Penny Marshall	tt0094737
1746520847	1925	1925	The Human Tornado	The Human Tornado	Wilson, Ben	Ben F. Wilson	tt0127592
1746523312	1914	1914	The Royal Box	The Royal Box	Eagle, Oscar	Oscar Eagle	tt0004548
1746582748	1936	1936	Fugitive in the Sky	Fugitive in the Sky	Grinde, Nick	Nick Grinde	tt0028907
1746520424	1925	1925	The Lucky Devil	The Lucky Devil	Tuttle, Frank	Frank Tuttle	tt0016051
1746570587	1933	1933	Devil's Mate	The Devil's Mate	Rosen, Phil	Phil Rosen	tt0023944
1746573941	1959	1959	Jet over the Atlantic	Jet Over the Atlantic	Haskin, Byron	Byron Haskin	tt0052943
1746515772	1917	1917	The Understudy	The Understudy	Bertram, William	William Bertram	tt0183945
1746465025	1946	1946	The Undercover Woman	The Undercover Woman	Carr, Thomas	Thomas Carr	tt0039065
1746585447	1939	1939	Boy Slaves	Boy Slaves	Wolfson, P. J.	P.J. Wolfson	tt0031117
1746571797	1931	1931	The Right of Way	The Right of Way	Lloyd, Frank	Frank Lloyd	tt0022317
1746498287	1918	1918	Suspicion	Suspicion	Stahl, John M.	John M. Stahl	tt0009674
1746553483	1965	1965	Camp	Camp	Warhol, Andy	Andy Warhol	tt0173709
1745282676	2007	2007	Because I Said So	Because I Said So	Lehmann, Michael	Michael Lehmann	tt0490084
1746485588	1917	1917	The Courage of the Common Place	The Courage of the Common Place	Turbett, Ben	Ben Turbett	tt0168611
1746498337	1919	1919	The White Heather	The White Heather	Tourneur, Maurice	Maurice Tourneur	tt0010882
1746532815	1915	1915	The Patriot and the Spy	The Patriot and the Spy		Jack Harvey	tt0005874
1746530773	1948	1948	Three Daring Daughters	Three Daring Daughters	Wilcox, Fred M.	Fred M. Wilcox	tt0040875
1746524068	1923	1923	The Lonely Road	The Lonely Road	Schertzinger, Victor	Victor Schertzinger	tt0014210
1746520957	1949	1949	The Secret of St. Ives	The Secret of St. Ives	Rosen, Philip	Phil Rosen	tt0041856
1746410700	1993	1993	Only the Strong	Only the Strong	Lettich, Sheldon	Sheldon Lettich	tt0107750
1746393277	1984	1984	The Lonely Guy	The Lonely Guy	Hiller, Arthur	Arthur Hiller	tt0087635
1746577809	1937	1937	One Hundred Men and a Girl	One Hundred Men and a Girl	Koster, Henry	Henry Koster	tt0029347
1746475293	1947	1947	Jewels of Brandenburg	Jewels of Brandenburg	Forde, Eugene	Eugene Forde	tt0179249
1746475712	1944	1944	Four Jills in a Jeep	Four Jills in a Jeep	Seiter, William A.	William A. Seiter	tt0036836
1746562117	1953	1953	Half a Hero	Half a Hero	Weis, Don	Don Weis	tt0045854
1746581057	1960	1960	Hell to Eternity	Hell to Eternity	Karlson, Phil	Phil Karlson	tt0053901
1746502712	1910	1910	Red Eagle's Love Affair	Red Eagle's Love Affair			tt0286913
1746436634	1941	1941	The Round Up	The Roundup	Selander, Lesley	Lesley Selander	tt0034133
1746507740	1924	1924	The Covered Trail	The Covered Trail	Nelson, Jack	Jack Nelson	tt0014803
1746492550	1947	1946	Fool's Gold	Fool's Gold	Archainbaud, George	George Archainbaud	tt0038532
1746234607	1968	1968	Blackbeard's Ghost	Blackbeard's Ghost	Stevenson, Robert	Robert Stevenson	tt0062737
1746509024	1924	1924	The Snob	The Snob	Bell, Monta	Monta Bell	tt0015346
1746424169	1943	1943	Bad Men of Thunder Gap	Bad Men of Thunder Gap	Herman, Albert	Albert Herman	tt0039900
1745393807	2008	2008	You Don't Mess with the Zohan	You Don't Mess with the Zohan	Dugan, Dennis	Dennis Dugan	tt0960144
1746513615	1922	1922	Flesh and Spirit	Flesh and Spirit	Levering, Joseph	Joseph Levering	tt0013135
1746561826	1956	1956	The Broken Star	The Broken Star	Selander, Lesley	Lesley Selander	tt0049031
1746582549	1938	1938	Holiday	Holiday	Cukor, George	George Cukor	tt0030241
1746499885	1949	1949	Always Leave Them Laughing	Always Leave Them Laughing	Del Ruth, Roy	Roy Del Ruth	tt0041115
1746577408	1950	1950	The Outriders	The Outriders	Rowland, Roy	Roy Rowland	tt0042825

1746520808	1925	1925	The Unchastened Woman	The Unchastened Woman	Young, James	James Young	tt0016469
1746420136	1994	1994	Martin Lawrence: You So Crazy	Martin Lawrence: You So Crazy	Schlamme, Thomas	Thomas Schlamme	tt0111804
1746186174	1967	1967	First To Fight	First to Fight	Nyby, Christian	Christian Nyby	tt0061667
1746582945	1937	1937	Bulldog Drummond Comes Back	Bulldog Drummond Comes Back	King, Louis	Louis King	tt0028667
1746218840	1979	1979	Buck Rogers in the 25th Century	Buck Rogers in the 25th Century	Haller, Daniel	Daniel Haller	tt0077278
1746453510	1943	1943	Taxi, Mister	Taxi, Mister	Neumann, Kurt	Kurt Neumann	tt0036416
1746438973	1944	1944	My Pal Wolf	My Pal Wolf	Werker, Alfred	Alfred L. Werker	tt0037111
1746577868	1939	1939	Slightly Honorable	Slightly Honorable	Garnett, Tay	Tay Garnett	tt0033062
1746410726	1992	1992	Far and Away	Far and Away	Howard, Ron	Ron Howard	tt0104231
1746249913	1978	1978	California Suite	California Suite	Ross, Herbert	Herbert Ross	tt0077289
1746520458	1926	1925	Iron Fist	The Iron Fist	McGowan, J. P.	J.P. McGowan	tt0327963
1746410380	1983	1983	Staying Alive	Staying Alive	Stallone, Sylvester	Sylvester Stallone	tt0086361
1746454086	1943	1943	Return of the Rangers	The Return of the Rangers	Clifton, Elmer	Elmer Clifton	tt0036305
1745358544	2004	2004	First Daughter	First Daughter	Whitaker, Forest	Forest Whitaker	tt0361620
1746421970	1997	1997	Sprung	Sprung	Cundieff, Rusty	Rusty Cundieff	tt0120190
1746577519	1938	1938	I Am a Criminal	I Am a Criminal	Nigh, William	William Nigh	tt0030253
1746560548	1932	1932	High Pressure	High Pressure	Le Roy, Mervyn	Mervyn LeRoy	tt0023007
1746504106	1918	1918	Fast Company	Fast Company	Reynolds, Lynn F.	Lynn Reynolds	tt0009058
1746234993	1971	1971	The American Dreamer	The American Dreamer	Schiller, Lawrence; Carson, L. M. Kit	L.M. Kit Carson	tt0177516
1745373325	2002	2001	Human Nature	Human Nature	Gondry, Michel	Michel Gondry	tt0219822
1746524454	1915	1915	After Dark	After Dark	Thomson, Frederic	Frederick A. Thomson	tt0004865
1746496244	1921	1921	Ladies Must Live	Ladies Must Live	Tucker, George Loane	George Loane Tucker	tt0012366
1746583439	1951	1951	Bedtime For Bonzo	Bedtime for Bonzo	Cordova, Frederick de	Frederick De Cordova	tt0043325
1746566249	1956	1956	The Maverick Queen	The Maverick Queen	Kane, Joe	Joseph Kane	tt0049487
1746512907	1917	1917	Charity Castle	Charity Castle	Ingraham, Lloyd	Lloyd Ingraham	tt0007783
1746525090	1920	1920	Her First Elopement	Her First Elopement	Wood, Sam	Sam Wood	tt0011285
1746216732	1969	1969	Smith!	Smith!	O'Herlihy, Michael	Michael O'Herlihy	tt0065003
1746577819	1936	1936	Tarzan Escapes	Tarzan Escapes	Thorpe, Richard; Farrow, John; Seitz	Richard Thorpe	tt0028345
1746508763	1927	1927	Spuds	Spuds	Semon, Larry	Edward Ludwig	tt0166363
1746577618	1934	1934	It's a Gift	It's a Gift	McLeod, Norman	Norman Z. McLeod	tt0025318
1746581784	1950	1950	Sideshow	Sideshow	Yarbrough, Jean	Jean Yarbrough	tt0042961
1746508056	1925	1925	The Spaniard	The Spaniard	Walsh, Raoul	Raoul Walsh	tt0016379
1746580859	1938	1938	West of Rainbow's End	West of Rainbow's End	James, Alan	Alan James	tt0030959
1746580878	1935	1935	Tumbling Tumbleweeds	Tumbling Tumbleweeds	Kane, Joseph	Joseph Kane	tt0027139
1746574307	1958	1958	Ambush at Cimarron Pass	Ambush at Cimarron Pass	Copelan, Jodie	Jodie Copelan	tt0051349
1746410222	1991	1990	Pastime	Pastime	Armstrong, Robin B.	Robin B. Armstrong	tt0105109
1746562149	1953	1953	Fort Vengeance	Fort Vengeance	Selander, Lesley	Lesley Selander	tt0045785
1746577495	1935	1935	The Return of Peter Grimm	The Return of Peter Grimm	Nicholls, George	George Nichols Jr.	tt0026926
1746454465	1943	1943	Wagon Tracks West	Wagon Tracks West	Bretherton, Howard	Howard Bretherton	tt0036509
1746568283	1933	1933	Black Beauty	Black Beauty	Rosen, Phil	Phil Rosen	tt0023815
1746516501	1920	1920	Mothers of Men	Mothers of Men	José, Edward	Edward José	tt0011483
1745359986	2003	2003	Kill Bill -- Vol. 1	Kill Bill: Vol. 1	Tarantino, Quentin	Quentin Tarantino	tt0266697
1745293557	2007	2007	I Think I Love My Wife	I Think I Love My Wife	Rock, Chris	Chris Rock	tt0770772
1746438956	1942	1942	Dr. Broadway	Dr. Broadway	Mann, Anthony	Anthony Mann	tt0034678
1746582875	1936	1936	King of the Pecos	King of the Pecos	Kane, Joseph	Joseph Kane	tt0027849
1746561220	1932	1932	Young America	Young America	Borzage, Frank	Frank Borzage	tt0023719
1746504054	1922	1922	Step On It	Step on It!	Conway, Jack	Jack Conway	tt0013636
1745359117	2008	2008	Cloverfield	Cloverfield	Reeves, Matt	Matt Reeves	tt1060277
1746569835	1957	1957	The Vintage	The Vintage	Hayden, Jeffrey	Jeffrey Hayden	tt0051167
1746561607	1965	1965	White Hunter	White Hunter	Michael, George	George Michael	tt0386876
1746580457	1938	1938	Ghost Town Riders	Ghost Town Riders	Waggner, George	George Waggner	tt0030179
1746419946	1993	1993	Schindler's List	Schindler's List	Spielberg, Steven	Steven Spielberg	tt0108052
1746574489	1952	1952	Skirts Ahoy!	Skirts Ahoy!	Lanfield, Sidney	Sidney Lanfield	tt0045155
1769972849	1963	1963	Hollywood's World of Flesh	Hollywood's World of Flesh	Frost, R. L.	Lee Frost	tt0125157
1746569750	1957	1957	The Enemy Below	The Enemy Below	Powell, Dick	Dick Powell	tt0050356
1746409108	1991	1990	Book of Love	Book of Love	Shaye, Robert	Robert Shaye	tt0099166
1746514054	1914	1914	Lola	Lola	Young, James	James Young	tt0004249

1746520787	1925	1925	A Slave of Fashion	A Slave of Fashion	Henley, Hobart	Hobart Henley	tt0016358		
1746569862	1958	1958	Teenage Cave Man	Teenage Cave Man	Corman, Roger	Roger Corman	tt0052279		
1746561162	1932	1932	Love in High Gear	Love in High Gear	Strayer, Frank	Frank R. Strayer	tt0022093		
1746580584	1938	1938	South of Arizona	South of Arizona	Nelson, Sam	Sam Nelson	tt0134109		
1746474837	1948	1948	Sons of Adventure	Sons of Adventure	Canutt, Yakima	Yakima Canutt	tt0040822		
1746503292	1920	1920	Curtain	Curtain	Young, James	James Young	tt0145732		
1746235442	1979	1979	Walk Proud	Walk Proud	Collins, Robert	Robert L. Collins	tt0080114		
1746419198	1989	1989	She-Devil	She Devil	Seidelman, Susan	Susan Seidelman	tt0098309		
1746503054	1915	1915	I'm Glad My Boy Grew Up to Be a Soldier	I'm Glad My Boy Grew Up to Be a Soldier	Beal, Frank	Frank Beal	tt0005533		
1746186208	1969	1969	This Is My Alaska	This Is My Alaska	Shebal, Leroy		tt1180350		
1746578802	1962	1962	The Wacky World of Dr. Morgus	The Wacky World of Dr. Morgus	Haig, Roul	Roul Haig	tt0251470		
1746533031	1923	1923	The Call of the Canyon	The Call of the Canyon	Fleming, Victor	Victor Fleming	tt0013906		
1746524564	1910	1910	The Mystery of Lonely Gulch	The Mystery of Lonely Gulch		Theodore Wharton	tt1498876		
1746422969	1990	1990	Spaced Invaders	Spaced Invaders	Johnson, Patrick Read	Patrick Read Johnson	tt0100666		
1746580445	1934	1935	Helldorado	Helldorado	Cruze, James	James Cruze	tt0026462		
1746474977	1948	1948	Last of the Wild Horses	Last of the Wild Horses	Lippert, Robert L.	Robert L. Lippert	tt0040530		
1746583159	1939	1939	Should a Girl Marry?	Should a Girl Marry?	Hillyer, Lambert	Lambert Hillyer	tt0031923		
1746578626	1962	1962	The Spiral Road	The Spiral Road	Mulligan, Robert	Robert Mulligan	tt0056516		
1746584205	1937	1937	They Won't Forget	They Won't Forget	LeRoy, Mervyn	Mervyn LeRoy	tt0029658		
1746436041	1942	1942	Sweetheart of the Fleet	Sweetheart of the Fleet	Barton, Charles	Charles Barton	tt0035401		
1746424057	1941	1941	Louisiana Purchase	Louisiana Purchase	Cummings, Irving	Irving Cummings	tt0033851		
1746574704	1936	1936	Empty Saddles	Empty Saddles	Selander, Lesley	Lesley Selander	tt0027571		
1746216661	1966	1966	The Street Is My Beat	The Street Is My Beat	Berwick, Irvin	Irvin Berwick	tt0216244		
1746583964	1936	1935	Magnificent Obsession	Magnificent Obsession	Stahl, John M.	John M. Stahl	tt0026667		
1746555333	1957	1957	Man on Fire	Man on Fire	MacDougall, Ranald	Ranald MacDougall	tt0050682		
1746208128	1969	1969	The Trouble with Girls	The Trouble with Girls	Tewksbury, Peter	Peter Tewksbury	tt0065125		
1746577331	1959	1959	Never Steal Anything Small	Never Steal Anything Small	Lederer, Charles	Charles Lederer	tt0053109		
1746580810	1937	1937	Merry-Go-Round of 1938	Merry Go Round of 1938	Cummings, Irving	Irving Cummings	tt0029234		
1746524276	1915	1915	The Green Cloak	The Green Cloak	Edwin, Walter	Walter Edwin	tt0005426		
1746500273	1925	1925	Why Women Love	Why Women Love		Edwin Carewe	tt0016528		
1746523559	1910	1910	The Tenderfoot's Triumph	The Tenderfoot's Triumph	Powell, Frank	Frank Powell	tt0001413		
1746210948	1971	1971	The Todd Killings	The Todd Killings	Shear, Barry	Barry Shear	tt0066463		
1746257247	1975	1974	Act of Vengeance	Act of Vengeance	Kelljchian, Robert	Bob Kelljan	tt0072067		
1746568346	1952	1952	Walk East on Beacon	Walk East on Beacon!	Werker, Alfred	Alfred L. Werker	tt0045309		
1746410879	1993	1993	The Pickle	The Pickle	Mazursky, Paul	Paul Mazursky	tt0107824		
1746525168	1920	1920	Run 'em Ragged	Run 'Em Ragged	Goulding, Alf	Alfred J. Goulding	tt0142813		
1746248837	1974	1975	Boss Nigger	Boss Nigger	Arnold, Jack	Jack Arnold	tt0072725		
1746530692	1949	1949	Shamrock Hill	Shamrock Hill	Dreifuss, Arthur	Arthur Dreifuss	tt0041863		
1746569999	1958	1958	Blood Arrow	Blood Arrow	Warren, Charles Marquis	Charles Marquis Warren	tt0051420		
1745341617	2004	2004	The Butterfly Effect	The Butterfly Effect	Bress, Eric ; Gruber, J. Mackye	Eric Bress	tt0289879		
1746473912	1946	1946	Plainsman and the Lady	Plainsman and the Lady	Kane, Joe	Joseph Kane	tt0038846		
1746475742	1946	1946	The Secret Heart	The Secret Heart	Leonard, Robert Z.	Robert Z. Leonard	tt0038916		
1746487602	1919	1919	Captain Kidd, Jr.	Captain Kidd, Jr.	Taylor, William D.	William Desmond Taylor	tt0009987		
1746454948	1941	1941	Arizona Bound	Arizona Bound	Bennet, Spencer	Spencer Gordon Bennet	tt0033351		
1746566040	1953	1953	Violated	Violated	Strate, Walter	Walter Strate	tt0046520		
1746422922	1989	1989	Slaves of New York	Slaves of New York	Ivory, James	James Ivory	tt0098347		
1746466705	1917	1917	The Lair of the Wolf	The Lair of the Wolf	Swickard, Charles	Charles Swickard	tt0008167		
1746199295	1966	1966	The Bubble	The Bubble	Oboler, Arch	Arch Oboler	tt0060396		
1746582057	1928	1928	The River Pirate	The River Pirate	Howard, William K.	William K. Howard	tt0019329		
1746465371	1946	1946	Night Train to Memphis	Night Train to Memphis	Selander, Lesley	Lesley Selander	tt0038775		
**1746513628**	**1923**	**1923**	**Crooked Alley**	**Crooked Alley**	**Hill, Robert F.**	**Robert F. Hill**	**tt0013959**		1
1745302893	2007	2006	Alone with Her	Alone with Her	Nicholas, Eric	Eric Nicholas	tt0472259		
1746553539	1964	1964	Moro Witch Doctor	Moro Witch Doctor	Romero, Eddie	Eddie Romero	tt0058373		
1745293951	2006	2006	Mad Cowgirl	Mad Cowgirl	Hatanaka, Gregory	Gregory Hatanaka	tt0438204		
1746513789	1910	1910	Ranson's Folly	Ranson's Folly		Edwin S. Porter	tt1515891		
1746582324	1928	1928	Fangs of the Wild	Fangs of the Wild	Storm, Jerome	Jerome Storm	tt0018874		
1746585289	1929	1929	The Air Legion	The Air Legion	Glennon, Bert	Bert Glennon	tt0019628		

1746453616	1942	1941	Confessions of Boston Blackie	Confessions of Boston Blackie	Dmytryk, Edward	Edward Dmytryk	tt0033484
1746554377	1965	1965	John Goldfarb, Please Come Home!	John Goldfarb, Please Come Home!	Thompson, J. Lee	J. Lee Thompson	tt0059336
1746583264	1928	1928	The Fleet's In	The Fleet's In	St. Clair, Malcolm	Malcolm St. Clair	tt0018896
1746410669	1988	1988	Gorillas in the Mist	Gorillas in the Mist	Apted, Michael	Michael Apted	tt0095243
1746574517	1935	1935	Motive for Revenge	Motive for Revenge	Lynwood, Burt	Burt P. Lynwood	tt0026731
1746584116	1937	1937	That Certain Woman	That Certain Woman	Goulding, Edmund	Edmund Goulding	tt0029650
1745375163	2009	2008	American Swing	American Swing	Kaufman, Mat ; Hart, Jon	Jon Hart	tt1058058
1746421602	1993	1993	Ruby in Paradise	Ruby in Paradise	Nunez, Victor	Victor Nunez	tt0108000
1746566136	1955	1955	The Far Horizons	The Far Horizons	Maté, Rudolph	Rudolph Maté	tt0048056
1746524466	1913	1913	The Fight for Millions	The Fight for Millions	Blaché, Herbert	Herbert Blaché	tt0002859
1746583157	1936	1936	King of the Royal Mounted	King of the Royal Mounted	Bretherton, Howard	Howard Bretherton	tt0027850
1746197919	1969	1969	Finney	Finney	Hare, Bill	Bill Hare	tt0192024
1746523345	1910	1910	The Regeneration of Father	The Regeneration of Father			tt1824239
1746562290	1957	1957	The Night the World Exploded	The Night the World Exploded	Sears, Fred F.	Fred F. Sears	tt0050767
1746524383	1917	1917	The Devil's Assistant	The Devil's Assistant	Pollard, Harry	Harry A. Pollard	tt0007854
1746454183	1995	1995	Under Siege 2: Dark Territory	Under Siege 2: Dark Territory	Murphy, Geoff	Geoff Murphy	tt0114781
1746566728	1933	1933	My Woman	My Woman	Schertzinger, Victor	Victor Schertzinger	tt0024365
1746423272	1993	1993	Fearless	Fearless	Weir, Peter	Peter Weir	tt0106881
1746508902	1926	1925	The Wildcat	The Wildcat	Fraser, Harry L.	Harry L. Fraser	tt0141987
1746491771	1947	1947	Key Witness	Key Witness	Lederman, D. Ross	D. Ross Lederman	tt0039529
1746500257	1924	1924	Mile a Minute Morgan	Mile a Minute Morgan	Mattison, Frank S.	Frank S. Mattison	tt0015137
1746566260	1957	1957	Until They Sail	Until They Sail	Wise, Robert	Robert Wise	tt0051141
1746550170	1954	1954	Go Man Go	Go Man Go	Howe, James Wong	James Wong Howe	tt0047032
1746573109	1932	1932	Devil's Lottery	Devil's Lottery	Taylor, Sam	Sam Taylor	tt0022816
1746475288	1944	1944	Winged Victory	Winged Victory	Cukor, George	George Cukor	tt0037467
1746580610	1938	1938	Law of the Underworld	Law of the Underworld	Landers, Lew	Lew Landers	tt0030353
1746583423	1930	1930	Talking It Over	Talking It Over		Murray Roth	tt1546026
1746578501	1961	1961	A Thunder of Drums	A Thunder of Drums	Newman, Joseph M.	Joseph M. Newman	tt0055524
1746474142	1945	1945	Saddle Serenade	Saddle Serenade	Drake, Oliver	Oliver Drake	tt0163189
1746508687	1926	1926	Just Suppose	Just Suppose	Webb, Kenneth	Kenneth S. Webb	tt0017014
1746523049	1916	1916	New York	New York	Fitzmaurice, George	George Fitzmaurice	tt0145119
1746210271	1970	1970	Something for Everyone	Something for Everyone	Prince, Harold	Harold Prince	tt0066392
1746218900	1972	1972	Blacula	Blacula	Crain, William	William Crain	tt0068284
1746504424	1922	1922	Arabian Love	Arabian Love	Storm, Jerome	Jerome Storm	tt0012903
1746509216	1925	1925	Makers of Men	Makers of Men	Sheldon, Forrest	Forrest Sheldon	tt0016070
1746555653	1955	1955	Two-Gun Lady	Two Gun Lady	Bartlett, Richard H.	Richard Bartlett	tt0049897
1746560744	1932	1932	Vanishing Men	Vanishing Men	Fraser, Harry	Harry L. Fraser	tt0023651
1746584479	1931	1931	Svengali	Svengali	Mayo, Archie	Archie Mayo	tt0022454
1746499909	1948	1948	Marshal of Amarillo	Marshal of Amarillo	Ford, Philip	Philip Ford	tt0040574
1746580926	1961	1961	You Have To Run Fast	You Have to Run Fast	Cahn, Edward L.	Edward L. Cahn	tt0055631
1746566117	1958	1958	The Lineup	The Lineup	Siegel, Don	Don Siegel	tt0051866
1746507833	1926	1926	Forest Havoc	Forest Havoc	Paton, Stuart	Stuart Paton	tt0132975
1746584897	1939	1939	Code of the Cactus	Code of the Cactus	Newfield, Sam	Sam Newfield	tt0031164
1746474950	1944	1944	The Eve of St. Mark	The Eve of St. Mark	Stahl, John M.	John M. Stahl	tt0036803
1746532472	1910	1910	The Tout's Remembrance	The Tout's Remembrance		Gilbert M. 'Broncho Billy' Anderson	tt0001419
1746582058	1910	1910	The House with Closed Shutters	The House with Closed Shutters	Griffith, D. W.	D.W. Griffith	tt0001256
1746438286	1941	1941	Scattergood Baines	Scattergood Baines	Cabanne, Christy	Christy Cabanne	tt0034154
1746584304	1936	1936	The Broken Coin	The Broken Coin	Herman, Albert	Albert Herman	tt0202277
1769970559	1967	1967	The Singles	The Singles	Johnsen, S. N.	Sande N. Johnsen	tt0138102
1746514234	1923	1923	Trimmed in Scarlet	Trimmed in Scarlet	Conway, Jack	Jack Conway	tt0014563
1746508231	1927	1927	The Range Raiders	The Range Raiders	Hurst, Paul	Paul Hurst	tt0155096
1746474968	1947	1947	I Wonder Who's Kissing Her Now?	I Wonder Who's Kissing Her Now	Bacon, Lloyd	Lloyd Bacon	tt0039483
1746409023	1982	1982	Jinxed!	Jinxed!	Siegel, Don	Don Siegel	tt0084173
1746514064	1923	1923	The Silent Command	The Silent Command	Edwards, J. Gordon	J. Gordon Edwards	tt0014469
1746424168	1941	1941	Aloma of the South Seas	Aloma of the South Seas	Santell, Alfred	Alfred Santell	tt0033331
1746583820	1930	1930	Hook, Line and Sinker	Hook Line and Sinker	Cline, Edward	Edward F. Cline	tt0020987
1746530668	1950	1950	Battling Marshal	Battling Marshal	Drake, Oliver	Oliver Drake	tt0042232

1746435709	1943	1943	Hitler's Madman	Hitler's Madman	Sirk, Douglas	Douglas Sirk	tt0036005
1746208469	1968	1968	Kona Coast	Kona Coast	Johnson, Lamont	Lamont Johnson	tt0063194
1746492084	1947	1947	It Had to Be You	It Had to Be You	Hartman, Don; Maté, Rudolph	Don Hartman	tt0039500
1746491978	1945	1945	Earl Carroll Vanities	Earl Carroll Vanities	Santley, Joseph	Joseph Santley	tt0037664
1746520781	1950	1949	Twelve O'Clock High	Twelve O'Clock High	King, Henry	Henry King	tt0041996
1746584078	1940	1940	North West Mounted Police	North West Mounted Police	De Mille, Cecil B.	Cecil B. DeMille	tt0032850
1746453654	1943	1943	Mister Big	Mister Big	Lamont, Charles	Charles Lamont	tt0036167
1746198778	1969	1968	The Love Bug	The Love Bug	Stevenson, Robert	Robert Stevenson	tt0064603
1746562113	1957	1957	Sayonara	Sayonara	Logan, Joshua	Joshua Logan	tt0050933
1746570328	1952	1952	She's Working Her Way Through College	She's Working Her Way Through College	Humberstone, Bruce	H. Bruce Humberstone	tt0045144
1746570247	1934	1934	Lightning Strikes Twice	Lightning Strikes Twice	Holmes, Ben	Ben Holmes	tt0025395
1746520275	1926	1926	Whispering Canyon	Whispering Canyon	Forman, Tom	Tom Forman	tt0017549
1746502815	1916	1916	The World Against Him	The World Against Him	Crane, Frank Hall	Frank Hall Crane	tt0007589
1746577246	1963	1963	Marilyn	Marilyn		Henry Koster	tt0057290
1746524638	1928	1928	Just Married	Just Married	Strayer, Frank	Frank R. Strayer	tt0019044
1746465014	1942	1942	Home in Wyomin'	Home in Wyomin'	Morgan, William	William Morgan	tt0034866
1746500965	1915	1915	The Builder of Bridges	The Builder of Bridges	Irving, George	George Irving	tt0005034
1746574534	1937	1937	West of Shanghai	West of Shanghai	Farrow, John	John Farrow	tt0029754
1746569711	1954	1954	Cry Vengeance	Cry Vengeance	Stevens, Mark	Mark Stevens	tt0046885
1746435375	2000	2000	Bless the Child	Bless the Child	Russell, Chuck	Chuck Russell	tt0163983
1746251263	1976	1976	Shout at the Devil	Shout at the Devil	Hunt, Peter	Peter R. Hunt	tt0075214
1746235574	1974	1974	Madhouse	Madhouse	Clark, Jim	Jim Clark	tt0071790
1746574070	1963	1963	None but the Brave	None But the Brave	Richardson, Ken	Kenneth W. Richardson	tt0251305
1746583432	1931	1931	Women Go on Forever	Women Go on Forever	Lang, Walter	Walter Lang	tt0022573
1746525104	1920	1920	The Common Sin	The Common Sin	King, Burton	Burton L. King	tt0189435
1746503794	1922	1922	West vs. East	West vs. East	Perez, Marcel	Marcel Perez	tt0177377
1746507766	1924	1924	Sporting Youth	Sporting Youth	Pollard, Harry A.	Harry A. Pollard	tt0015359
1746584420	1939	1939	When Tomorrow Comes	When Tomorrow Comes	Stahl, John M.	John M. Stahl	tt0032124
1746437390	1940	1940	Son of Roaring Dan	Son of Roaring Dan	Beebe, Ford	Ford Beebe	tt0033077
1746520563	1925	1925	The Desert's Price	The Desert's Price	Van Dyke, W. S.	W.S. Van Dyke	tt0015746
1745359313	2009	2008	The Great Buck Howard	The Great Buck Howard	McGinly, Sean	Sean McGinly	tt0460810
1746520328	1926	1926	Roaring Rider	The Roaring Rider	Thorpe, Richard	Richard Thorpe	tt0017330
1746248271	1968	1968	Blue	Blue	Narizzano, Silvio	Silvio Narizzano	tt0062742
1746584104	1936	1936	The Music Goes 'Round	The Music Goes 'Round	Schertzinger, Victor	Victor Schertzinger	tt0028005
1746577260	1961	1961	Guns of the Trees	Guns of the Trees	Mekas, Jonas	Jonas Mekas	tt0058168
1746512365	1915	1915	Cohen's Luck	Cohen's Luck	Collins, John H.	John H. Collins	tt0005104
1746581047	1961	1960	I'll Give My Life	I'll Give My Life	Claxton, William F.	William F. Claxton	tt0055001
1746520110	1950	1950	Chain Gang	Chain Gang	Landers, Lew	Lew Landers	tt0042323
1746585435	1939	1939	Mutiny on the Blackhawk	Mutiny on the Blackhawk	Cabanne, Christy	Christy Cabanne	tt0031691
1746574756	1933	1933	Shanghai Madness	Shanghai Madness	Blystone, John	John G. Blystone	tt0024546
1746569923	1958	1958	Dreamland Capers	Dreamland Capers			tt0261663
1746496769	1918	1918	Tongues of Flame	Tongues of Flame	Campbell, Colin	Colin Campbell	tt0009709
1746566850	1933	1932	The Sign of the Cross	The Sign of the Cross	De Mille, Cecil B.	Cecil B. DeMille	tt0023470
1746509537	1926	1926	Blue Streak O'Neil	Blue Streak O'Neil	Hurst, Paul	Paul Hurst	tt0016665
1746256114	1977	1977	I Never Promised You a Rose Garden	I Never Promised You a Rose Garden	Page, Anthony	Anthony Page	tt0076172
1746500444	1926	1926	The Timid Terror	The Timid Terror	Andrews, Del	Del Andrews	tt0017471
1746524413	1914	1914	The Pit	The Pit	Tourneur, Maurice	Maurice Tourneur	tt0004473
1746508065	1926	1926	Bardelys the Magnificent	Bardelys the Magnificent	Vidor, King	King Vidor	tt0016627
1746508958	1949	1949	The Secret Garden	The Secret Garden	Wilcox, Fred M.	Fred M. Wilcox	tt0041855
1746524787	1916	1916	The Libertine	The Libertine	Steger, Julius; Golden, Joseph A.	Joseph A. Golden	tt0163056
1746582412	1935	1935	This Is the Life	This Is the Life	Neilan, Marshall	Marshall Neilan	tt0027093
1746520078	1924	1924	So Big	So Big	Brabin, Charles	Charles Brabin	tt0015347
1746583121	1939	1939	The Night of Nights	The Night of Nights	Milestone, Lewis	Lewis Milestone	tt0031724
1746516260	1920	1919	Fighting Cressy	Fighting Cressy	Thornby, Robert T.	Robert Thornby	tt0010122
1746257688	1978	1978	Who'll Stop the Rain	Who'll Stop the Rain	Reisz, Karel	Karel Reisz	tt0078490
1746408643	1981	1981	Sphinx	Sphinx	Schaffner, Franklin J.	Franklin J. Schaffner	tt0083113
1745342040	2004	2004	Metallica: Some Kind of Monster	Metallica: Some Kind of Monster	Berlinger, Joe ; Sinofsky, Bruce	Joe Berlinger	tt0387412

1746218317	1968	1968	The Thomas Crown Affair	The Thomas Crown Affair	Jewison, Norman	Norman Jewison	tt0063688
1746501583	1914	1914	A Factory Magdalen	A Factory Magdalen	Bostwick, Elwood Fleet	Elwood Fleet Bostwick	tt0003925
1746579035	1959	1959	Mustang	Mustang!	Stephens, Peter	Tom Gries	tt0127038
1746507897	1924	1924	Changing Husbands	Changing Husbands	Urson, Frank; Iribe, Paul	Paul Iribe	tt0014767
1746475923	1945	1945	The Body Snatcher	The Body Snatcher	Wise, Robert	Robert Wise	tt0037549
1746570990	1952	1952	Just for You	Just for You	Nugent, Elliott	Elliott Nugent	tt0044782
1746438323	1940	1940	Murder on the Yukon	Murder on the Yukon	Gasnier, Louis	Louis J. Gasnier	tt0032821
1746501512	1915	1915	The Supreme Test	The Supreme Test	Le Saint, Edward J.	Edward LeSaint	tt0006120
1746393437	1984	1984	Beverly Hills Cop	Beverly Hills Cop	Brest, Martin	Martin Brest	tt0086960
1746584419	1939	1939	Reno	Reno	Farrow, John	John Farrow	tt0031848
1746524957	1915	1914	The Man Behind the Door	The Man Behind the Door	Van, Wally	Wally Van	tt0005692
1769971101	1969	1969	Teresa ... Darling	Teresa... Darling	Stylianou, Michel	Michel Stylianou	tt0259634
1746217439	1965	1965	The Third Day	The Third Day	Smight, Jack	Jack Smight	tt0059795
1746533192	1916	1916	The Hidden Scar	The Hidden Scar	O'Neil, Barry	Barry O'Neil	tt0006798
1745293681	2007	2006	The Astronaut Farmer	The Astronaut Farmer	Polish, Michael	Michael Polish	tt0469263
1746453576	1942	1942	Joan of Paris	Joan of Paris	Stevenson, Robert	Robert Stevenson	tt0034919
1746520194	1925	1925	Silent Sanderson	Silent Sanderson	Dunlap, Scott R.	Scott R. Dunlap	tt0016350
1746234581	1966	1966	The Quiller Memorandum	The Quiller Memorandum	Anderson, Michael	Michael Anderson	tt0060880
1746465219	1947	1947	The Beginning or the End	The Beginning or the End	Taurog, Norman	Norman Taurog	tt0039178
1746524364	1923	1923	The Destroying Angel	Destroying Angel	Van Dyke, W. S.	W.S. Van Dyke	tt0013986
1746524288	1923	1923	Mysterious Goods	Mysterious Goods	Seeling, Charles R.	Charles R. Seeling	tt0014300
1746455082	1995	1995	Billy Madison	Billy Madison	Davis, Tamra	Tamra Davis	tt0112508
1746523572	1927	1927	Cactus Trails	Cactus Trails	Pembroke, Percy	Scott Pembroke	tt0319133
1746435331	1990	1990	Problem Child	Problem Child	Dugan, Dennis	Dennis Dugan	tt0100419
1746524899	1923	1923	Poor Men's Wives	Poor Men's Wives	Gasnier, Louis J.	Louis J. Gasnier	tt0014368
1746202092	1970	1970	Black Roots	Black Roots	Rogosin, Lionel	Lionel Rogosin	tt1196121
1746565881	1955	1955	Interrupted Melody	Interrupted Melody	Bernhardt, Curtis	Curtis Bernhardt	tt0048210
1746566739	1933	1933	Gun Justice	Gun Justice	James, Alan	Alan James	tt0025212
1746508169	1926	1926	Men of the Night	Men of the Night	Rogell, Albert	Albert S. Rogell	tt0017134
1746553756	1964	1964	The Patsy	The Patsy	Lewis, Jerry	Jerry Lewis	tt0058456
1746211275	1974	1974	Hearts and Minds	Hearts and Minds	Davis, Peter	Peter Davis	tt0071604
1746581027	1960	1960	The Boy and the Pirates	The Boy and the Pirates	Gordon, Bert I.	Bert I. Gordon	tt0053672
1746581882	1928	1928	The Pinto Kid	The Pinto Kid	King, Louis	Louis King	tt0019275
1746421587	1996	1996	Me & My Matchmaker	Me and My Matchmaker	Wexler, Mark	Mark Wexler	tt0117013
1746234996	1977	1977	Greased Lightning	Greased Lightning	Schultz, Michael	Michael Schultz	tt0076106
1746583679	1929	1929	Words and Music	Words and Music	Tinling, James	James Tinling	tt0020604
1746580534	1938	1938	Call of the Rockies	Call of the Rockies	James, Alan	Alan James	tt0029960
1746513462	1917	1917	Brand's Daughter	Brand's Daughter	Harvey, Harry	Harry Harvey	tt0007745
1746574869	1933	1933	Christopher Bean	Christopher Bean	Wood, Sam	Sam Wood	tt0023890
1746504530	1921	1921	The Devil	The Devil	Young, James	James Young	tt0012107
1746520672	1925	1925	Soul Mates	Soul Mates	Conway, Jack	Jack Conway	tt0017420
1746574677	1934	1934	Journal of a Crime	Journal of a Crime	Keighley, William	William Keighley	tt0025333
1746582352	1931	1931	Indiscreet	Indiscreet	McCarey, Leo	Leo McCarey	tt0022000
1746525060	1921	1921	Without Benefit of Clergy	Without Benefit of Clergy	Young, James	James Young	tt0012856
1746200597	1965	1965	Orgy of the Dead	Orgy of the Dead		Stephen C. Apostolof	tt0054240
1746437308	1941	1941	Sergeant York	Sergeant York	Hawks, Howard	Howard Hawks	tt0034167
1746577522	1937	1937	Flying Fists	Flying Fists	Hill, Bob	Robert F. Hill	tt0030143
1746570116	1952	1952	Strip Tease Girl	Striptease Girl			tt0160921
1746464824	1941	1941	Ice-Capades	Ice Capades	Santley, Joseph	Joseph Santley	tt0033749
1745395785	2010	2010	Tiny Furniture	Tiny Furniture	Dunham, Lena	Lena Dunham	tt1570989
1746583586	1930	1929	The Love Parade	The Love Parade	Lubitsch, Ernst	Ernst Lubitsch	tt0020112
1746582216	1951	1950	The Flying Missile	The Flying Missile	Levin, Henry	Henry Levin	tt0042468
1746566148	1957	1957	Istanbul	Istanbul	Pevney, Joseph	Joseph Pevney	tt0050552
1746580900	1938	1938	Life Goes On	Life Goes On	Nolte, William	William L. Nolte	tt0030365
1746559940	1964	1964	For Those Who Think Young	For Those Who Think Young	Martinson, Leslie H.	Leslie H. Martinson	tt0058110
1746269714	1978	1978	Sweater Girls	Sweater Girls	Jones, Don	Don Jones	tt0081583
1746574073	1961	1961	Go Naked in the World	Go Naked in the World	MacDougall, Ranald	Ranald MacDougall	tt0054933

1746455774	1999	1998	Lucinda's Spell	Lucinda's Spell	Jacobs, Jon	Jon Jacobs	tt0165866
1746524843	1917	1917	Bondage	Bondage	Park, Ida May	Ida May Park	tt0007736
1746408721	1986	1986	Solarbabies	Solarbabies	Johnson, Alan	Alan Johnson	tt0091981
1746502571	1917	1917	Young Mother Hubbard	Young Mother Hubbard	Berthelet, Arthur	Arthur Berthelet	tt0008797
1746514976	1910	1910	She's Done It Again	She's Done It Again	Carleton, Lloyd B.	Lloyd B. Carleton	tt0344327
1746574213	1959	1959	Compulsion	Compulsion	Fleischer, Richard	Richard Fleischer	tt0052700
1746577192	1959	1959	The Bat	The Bat	Wilbur, Crane	Crane Wilbur	tt0052602
1746393363	1987	1987	Living on Tokyo Time	Living on Tokyo Time	Okazaki, Steven	Steven Okazaki	tt0093429
1746562398	1954	1954	Trouble in the Glen	Trouble in the Glen	Wilcox, Herbert	Herbert Wilcox	tt0046465
1746584981	1940	1940	Pride and Prejudice	Pride and Prejudice	Leonard, Robert Z.	Robert Z. Leonard	tt0032943
1746420884	1995	1995	How to Make an American Quilt	How to Make an American Quilt	Moorhouse, Jocelyn	Jocelyn Moorhouse	tt0113347
1746567123	1932	1932	Without Honor	Without Honors	Nigh, William	William Nigh	tt0023703
1746584095	1935	1935	The Cowboy and the Bandit	The Cowboy and the Bandit	Herman, Al	Albert Herman	tt0026243
1746581604	1929	1929	The Show of Shows	The Show of Shows	Adolfi, John G.	John G. Adolfi	tt0020403
1746508134	1925	1925	The Dark Angel	The Dark Angel	Fitzmaurice, George	George Fitzmaurice	tt0015735
1745341016	2003	2003	Bringing Down the House	Bringing Down the House	Shankman, Adam	Adam Shankman	tt0305669
1746218925	1980	1980	Altered States	Altered States	Russell, Ken	Ken Russell	tt0080360
1745282686	2007	2007	Evan Almighty	Evan Almighty	Shadyac, Tom	Tom Shadyac	tt0413099
1746566129	1953	1953	Pack Train	Pack Train	Archainbaud, George	George Archainbaud	tt0046156
1746582672	1938	1938	Topa Topa	Topa Topa	Moore, Vin; Hutchison, Charles	Charles Hutchison	tt0032666
1746578395	1963	1963	Kings of the Sun	Kings of the Sun	Thompson, J. Lee	J. Lee Thompson	tt0057225
1746496761	1918	1918	Nobody's Wife	Nobody's Wife	Le Saint, Edward J.	Edward LeSaint	tt0009432
1746523711	1913	1913	The Sea Wolf	The Sea Wolf	Bosworth, Hobart	Hobart Bosworth	tt0003362
1746567976	1933	1933	Song of the Eagle	Song of the Eagle	Murphy, Ralph	Ralph Murphy	tt0024599
1746563886	1952	1952	The Quiet Man	The Quiet Man	Ford, John	John Ford	tt0045061
1746582861	1937	1937	The Lady Fights Back	The Lady Fights Back	Carruth, Milton	Milton Carruth	tt0029104
1746439411	1942	1942	Canal Zone	Canal Zone	Landers, Lew	Lew Landers	tt0034572
1746583721	1929	1929	Big News	Big News	La Cava, Gregory	Gregory La Cava	tt0019690
1746474973	1948	1948	The Fighting Ranger	The Fighting Ranger	Hillyer, Lambert	Lambert Hillyer	tt0040357
1746574783	1935	1935	A Dog of Flanders	A Dog of Flanders	Sloman, Edward	Edward Sloman	tt0026285
1746409510	1988	1988	The Wash	The Wash	Uno, Michael Toshiyuki	Michael Toshiyuki Uno	tt0096424
1746200559	1970	1969	The Computer Wore Tennis Shoes	The Computer Wore Tennis Shoes	Butler, Robert	Robert Butler	tt0065566
1746574648	1935	1934	The Brand of Hate	The Brand of Hate	Collins, Lew	Lewis D. Collins	tt0024911
1746507750	1926	1926	The Torrent	Torrent	Bell, Monta	Monta Bell	tt0017480
1746208717	1969	1969	Strategy of Terror	Strategy of Terror	Smight, Jack	Jack Smight	tt0065042
1746508895	1924	1924	The Street of Tears	The Street of Tears	Vale, Travers	Travers Vale	tt0015374
1769969340	1930	1930	Some Babies	Some Babies	Fox, Wallace	Wallace Fox	tt0830637
1746509268	1926	1926	Partners Again	Partners Again	King, Henry	Henry King	tt0017254
1746580762	1938	1938	West of Cheyenne	West of Cheyenne	Nelson, Sam	Sam Nelson	tt0132622
1746561894	1954	1954	Secret of the Incas	Secret of the Incas	Hopper, Jerry	Jerry Hopper	tt0047464
1746577677	1933	1933	Lightning Range	Lightning Range	Dixon, Denver	Victor Adamson	tt0132279
1746508243	1924	1924	Hit and Run	Hit and Run	Sedgwick, Edward	Edward Sedgwick	tt0014994
1746424462	1942	1942	Sheriff of Sage Valley	Sheriff of Sage Valley	Scott, Sherman	Sam Newfield	tt0035316
1746504389	1918	1918	Dolly's Vacation	Dolly's Vacation	Bertram, William	William Bertram	tt0184411
1746409426	1984	1983	Android	Android	Lipstadt, Aaron	Aaron Lipstadt	tt0083557
1746571816	1931	1931	Confessions of a Co-ed	Confessions of a Co Ed	Burton, David; Murphy, Dudley	David Burton	tt0021758
1746503759	1917	1917	Zollenstein	Zollenstein	Jones, Edgar	Edgar Jones	tt0008802
1746584696	1929	1929	The Phantom Rider	The Phantom Rider	McGowan, J. P.	J.P. McGowan	tt0020266
1746582533	1935	1935	Condemned to Live	Condemned to Live	Strayer, Frank R.	Frank R. Strayer	tt0026230
1746500622	1925	1925	North of Nome	North of Nome	Johnston, Raymond K.	Raymond Johnson	tt0142701
1746574129	1961	1961	Snow White and the Three Stooges	Snow White and the Three Stooges	Lang, Walter	Walter Lang	tt0055458
1746583053	1939	1939	Torture Ship	Torture Ship	Halperin, Victor	Victor Halperin	tt0032047
1746453554	1940	1939	Geronimo	Geronimo	Sloane, Paul H.	Paul Sloane	tt0031365
1745309522	2006	2006	Final Destination 3	Final Destination 3	Wong, James	James Wong	tt0414982
1746502484	1913	1913	The Third Degree	The Third Degree	O'Neil, Barry	Barry O'Neil	tt0003449
1746393499	1983	1983	Strange Invaders	Strange Invaders	Laughlin, Michael	Michael Laughlin	tt0086374
1746525095	1920	1920	The Fighting Chance	The Fighting Chance	Maigne, Charles	Charles Maigne	tt0162324

1746508745	1924	1924	Rarin' To Go	Rarin' to Go	Thorpe, Richard	Richard Thorpe	tt0015261
1746512853	1922	1922	Fools First	Fools First	Neilan, Marshall	Marshall Neilan	tt0013141
1746410157	1986	1986	Star Trek IV: The Voyage Home	Star Trek IV: The Voyage Home	Nimoy, Leonard	Leonard Nimoy	tt0092007
1746514968	1927	1927	Driven From Home	Driven from Home	Young, James	James Young	tt0017831
1746581022	1960	1960	Date Bait	Date Bait	Ireland, O'Dale	O'Dale Ireland	tt0053751
1746575066	1937	1937	Top of the Town	Top of the Town	Murphy, Ralph; Lang, Walter	Ralph Murphy	tt0029680
1746583778	1930	1930	Jazz Cinderella	The Jazz Cinderella	Pembroke, Scott	Scott Pembroke	tt0021011
1746438741	1995	1995	First Knight	First Knight	Zucker, Jerry	Jerry Zucker	tt0113071
1746421590	1991	1991	Dogfight	Dogfight	Savoca, Nancy	Nancy Savoca	tt0101748
1746583645	1930	1930	Breezy Bill	Breezy Bill	McGowan, J. P.	J.P. McGowan	tt0020710
1746422991	1991	1991	Star Trek VI: The Undiscovered Country	Star Trek VI: The Undiscovered Country	Meyer, Nicholas	Nicholas Meyer	tt0102975
1746578385	1960	1960	Tall Story	Tall Story	Logan, Joshua	Joshua Logan	tt0054367
1746584499	1930	1930	Last of the Duanes	The Last of the Duanes	Werker, Alfred L.	Alfred L. Werker	tt0021051
1746580216	1935	1935	The Perfect Clue	The Perfect Clue	Vignola, Robert G.	Robert G. Vignola	tt0026863
1746419938	1990	1990	Flashback	Flashback	Amurri, Franco	Franco Amurri	tt0099581
1746507889	1924	1924	Pot Luck Pards	Pot Luck Pards		Forrest Sheldon	tt0015246
1746580442	1938	1938	Gambling Ship	Gambling Ship	Scotto, Aubrey	Aubrey Scotto	tt0030167
1746577810	1937	1937	Outcast	Outcast	Florey, Robert	Robert Florey	tt0029360
1746578507	1958	1958	tom thumb	Tom Thumb	Pal, George	George Pal	tt0052427
1746578801	1961	1961	Come September	Come September	Mulligan, Robert	Robert Mulligan	tt0054759
1746567246	1933	1933	The Gallant Fool	The Gallant Fool	Bradbury, Robert N.	Robert N. Bradbury	tt0024045
1746454016	1941	1941	Belle Starr	Belle Starr	Cummings, Irving	Irving Cummings	tt0033383
1746583510	1930	1930	Divorce Among Friends	Divorce Among Friends	Del Ruth, Roy	Roy Del Ruth	tt0020826
1746455109	2000	2000	Bedazzled	Bedazzled	Ramis, Harold	Harold Ramis	tt0230030
1746508444	1926	1926	Made for Love	Made for Love	Sloane, Paul	Paul Sloane	tt0017100
1746514410	1916	1916	The Vagabond Prince	The Vagabond Prince	Giblyn, Charles	Charles Giblyn	tt0007506
1746475930	1946	1946	Cowboy Blues	Cowboy Blues	Nazarro, Ray	Ray Nazarro	tt0038428
1746570891	1935	1935	The Scoundrel	The Scoundrel	Hecht, Ben; MacArthur, Charles	Ben Hecht	tt0026970
1746409462	1991	1991	Suburban Commando	Suburban Commando	Kennedy, Burt	Burt Kennedy	tt0103003
1745340100	2003	2002	The Secret Lives of Dentists	The Secret Lives of Dentists	Rudolph, Alan	Alan Rudolph	tt0314630
1746436055	1941	1941	Unexpected Uncle	Unexpected Uncle	Godfrey, Peter	Peter Godfrey	tt0034339
1746491765	1946	1946	Rainbow Over Texas	Rainbow Over Texas	McDonald, Frank	Frank McDonald	tt0038869
1746500851	1915	1915	Just Jim	Just Jim	Lund, O. A. C.	O.A.C. Lund	tt0005581
1746507887	1925	1925	The Rip Snorter	The Rip Snorter	Hayes, Ward	Ward Hayes	tt0156965
1746500179	1949	1949	Lust for Gold	Lust for Gold	Simon, S. Sylvan	S. Sylvan Simon	tt0041610
1746577158	1961	1961	Ada	Ada	Mann, Daniel	Daniel Mann	tt0054601
1769970389	1965	1965	The Sweet Smell of Sex	Sweet Smell of Sex	Downey, Robert	Robert Downey Sr.	tt0259014
1746584518	1929	1929	Gold Diggers of Broadway	Gold Diggers of Broadway	Del Ruth, Roy	Roy Del Ruth	tt0019936
1746560120	1965	1965	Genghis Khan	Genghis Khan	Levin, Henry	Henry Levin	tt0059219
1746580486	1937	1937	The Devil Is Driving	The Devil Is Driving	Lachman, Harry	Harry Lachman	tt0028782
1746582355	1930	1930	Rough Waters	Rough Waters	Daumery, John	John Daumery	tt0021320
1746584119	1936	1936	Doughnuts and Society	Doughnuts and Society	Collins, Lewis D.	Lewis D. Collins	tt0027541
1746561541	1954	1954	Broken Lance	Broken Lance	Dmytryk, Edward	Edward Dmytryk	tt0046808
1746423472	1996	1996	Harriet the Spy	Harriet the Spy	Hughes, Bronwen	Bronwen Hughes	tt0116493
1746453477	1941	1941	Flying Wild	Flying Wild	West, William	William West	tt0033613
1746499937	1946	1946	The Bachelor's Daughters	The Bachelor's Daughters	Stone, Andrew	Andrew L. Stone	tt0039168
1746581300	1910	1910	In the Serpent's Power	In the Serpent's Power		Frank Beal	tt0352437
1746582821	1938	1938	Two Gun Man from Harlem	Two Gun Man from Harlem	Kahn, Richard C.	Richard C. Kahn	tt0135694
1746233778	1967	1967	Exposure!	Exposure		Joel L. Freedman	tt0221161
1746584629	1929	1929	Careers	Careers	Dillon, John Francis	John Francis Dillon	tt0019751
1746574565	1933	1933	Rainbow Ranch	Rainbow Ranch	Fraser, Harry	Harry L. Fraser	tt0132461
1746419806	1987	1987	Zombie High	Zombie High	Link, Ron	Ron Link	tt0094383
1746487926	1917	1917	Sister Against Sister	Sister Against Sister	Vincent, James R.	James Vincent	tt0008580
1746584991	1940	1940	The Ramparts We Watch	The Ramparts We Watch	Rochemont, Louis de	Louis De Rochemont	tt0032968
1746454240	2000	2000	Bring It On	Bring It On	Reed, Peyton	Peyton Reed	tt0204946
1746409131	1982	1982	An Officer and a Gentleman	An Officer and a Gentleman	Hackford, Taylor	Taylor Hackford	tt0084434
1746501186	1916	1916	Then I'll Come Back to You	Then I'll Come Back to You	Irving, George	George Irving	tt0007445

1746424054	1941	1941	Thunder over the Prairie	Thunder Over the Prairie	Hillyer, Lambert	Lambert Hillyer	tt0034289	
1746465189	1942	1942	Broadway	Broadway	Seiter, William A.	William A. Seiter	tt0034549	
1746513124	1916	1916	If My Country Should Call	If My Country Should Call	De Grasse, Joseph	Joseph De Grasse	tt0006847	
1746585211	1928	1928	The Gun Runner	The Gun Runner	Lewis, Edgar	Edgar Lewis	tt0018965	
1746555802	1955	1955	The Silver Star	The Silver Star	Bartlett, Richard	Richard Bartlett	tt0048619	
1746584621	1929	1929	Dark Streets	Dark Streets	Lloyd, Frank	Frank Lloyd	tt0019805	
1746454083	1944	1944	Address Unknown	Address Unknown	Menzies, William Cameron	William Cameron Menzies	tt0036581	
1746520365	1925	1925	Counsel for the Defense	Counsel for the Defense	King, Burton	Burton L. King	tt0015707	
1746516055	1921	1921	West of the Rio Grande	West of the Rio Grande	Townley, Robert H.	Robin H. Townley	tt0177376	
1746504586	1918	1918	Winning Grandma	Winning Grandma	Bertram, William	William Bertram	tt0190151	
1746574204	1957	1957	The Bridge on the River Kwai	The Bridge on the River Kwai	Lean, David	David Lean	tt0050212	
1746533217	1915	1915	The Stolen Voice	The Stolen Voice	Crane, Frank H.	Frank Hall Crane	tt0006101	
1746581332	1951	1951	Storm Warning	Storm Warning	Heisler, Stuart	Stuart Heisler	tt0044075	
1746583667	1930	1930	The Furies	The Furies	Crosland, Alan	Alan Crosland	tt0020910	
1746500459	1924	1924	Sandra	Sandra	Sawyer, Arthur H.; Campbell, Webst	Arthur H. Sawyer	tt0015302	
1746584519	1929	1929	The Mysterious Dr. Fu Manchu	The Mysterious Dr. Fu Manchu	Lee, Rowland V.	Rowland V. Lee	tt0020197	
1746583335	1930	1930	Min and Bill	Min and Bill	Hill, George	George W. Hill	tt0021148	
**1746566261**	**1957**	**1957**	**The Garment Jungle**	**The Garment Jungle**	**Sherman, Vincent; Aldrich, Robert**	**Vincent Sherman**	**tt0050424**	1
1746508240	1924	1924	Riders Up	Riders Up	Cummings, Irving	Irving Cummings	tt0015276	
1745359955	2010	2010	Black Swan	Black Swan	Aronofsky, Darren	Darren Aronofsky	tt0947798	
1746219110	1976	1976	The Missouri Breaks	The Missouri Breaks	Penn, Arthur	Arthur Penn	tt0074906	
1746577755	1936	1936	Charlie Chan at the Circus	Charlie Chan at the Circus	Lachman, Harry	Harry Lachman	tt0027439	
1746530687	1950	1950	The Torch	The Torch	Fernández, Emilio	Emilio Fernández	tt0041971	
1746566138	1958	1958	Cole Younger, Gunfighter	Cole Younger, Gunfighter	Springsteen, R. G.	R.G. Springsteen	tt0051482	
1746509298	1925	1925	Lena Rivers	Lena Rivers	Bennett, Whitman	Whitman Bennett	tt0127648	
1746464825	1945	1945	The Strange Affair of Uncle Harry	The Strange Affair of Uncle Harry	Siodmak, Robert	Robert Siodmak	tt0038123	
1746565945	1957	1956	Written on the Wind	Written on the Wind	Sirk, Douglas	Douglas Sirk	tt0049966	
1745374325	2003	2003	The Real Cancun	The Real Cancun	De Oliveira, Rick	Rick de Oliveira	tt0360916	
1746524874	1915	1915	At Bay	At Bay	Fitzmaurice, George	George Fitzmaurice	tt0004919	
1746520372	1949	1949	Bad Boy	Bad Boy	Neumann, Kurt	Kurt Neumann	tt0041144	
1746211079	1972	1972	The Poseidon Adventure	The Poseidon Adventure	Neame, Ronald	Ronald Neame	tt0069113	
1745395113	2009	2009	Hannah Montana: The Movie	Hannah Montana: The Movie	Chelsom, Peter	Peter Chelsom	tt1114677	
1746474971	1945	1945	Snafu	Snafu	Moss, Jack	Jack Moss	tt0038957	
1746408521	1988	1988	The Wrong Guys	The Wrong Guys	Bilson, Danny	Danny Bilson	tt0096466	
**1746549944**	**1955**	**1955**	**Bengazi**	**Bengazi**	**Brahm, John**	**John Brahm**	**tt0047872**	1
1746577475	1960	1960	Too Soon to Love	Too Soon to Love	Rush, Richard	Richard Rush	tt0054392	
1746525008	1915	1915	Fatherhood	Fatherhood	Bosworth, Hobart	Hobart Bosworth	tt0005308	
1746520248	1924	1924	Sherlock Jr.	Sherlock Jr.	Keaton, Buster	Buster Keaton	tt0015324	
1746492222	1948	1948	The Fight Never Ends	The Fight Never Ends	Lerner, Joe	Joseph Lerner	tt0041359	
1746581772	1929	1929	Thunder	Thunder	Nigh, William	William Nigh	tt0020498	
1746496232	1917	1917	The Bronze Bride	The Bronze Bride	McRae, Henry	Henry MacRae	tt0007753	
1746553589	1965	1965	That Funny Feeling	That Funny Feeling	Thorpe, Richard	Richard Thorpe	tt0059794	
1745372892	2009	2009	The Invention of Lying	The Invention of Lying	Gervais, Ricky ; Robinson, Matthew	Ricky Gervais	tt1058017	
1746514608	1915	1915	Marse Covington	Marse Covington	Carewe, Edwin	Edwin Carewe	tt0005717	
1746485524	1918	1918	The Dream Lady	The Dream Lady	Wilson, Elsie Jane	Elsie Jane Wilson	tt0009026	
1746553703	1964	1964	The Last Man on Earth	The Last Man on Earth	Salkow, Sidney	Ubaldo Ragona	tt0058700	
1746570580	1953	1952	April in Paris	April in Paris	Butler, David	David Butler	tt0044370	
1746574546	1935	1935	The Cowboy Millionaire	The Cowboy Millionaire	Cline, Edward F.	Edward F. Cline	tt0026242	
1745340092	2002	2002	Auto Focus	Auto Focus	Schrader, Paul	Paul Schrader	tt0298744	
1745293190	2005	2004	David Hockney: The Colors of Music	David Hockney: The Colors of Music	Kavaliauskas, Maryte ; Schneidman, S	Maryte Kavaliauskas	tt0424935	
1746516090	1922	1922	One Moment's Temptation	One Moment's Temptation	Rooke, A. J.	Arthur Rooke	tt0013460	
1746485825	1918	1918	The Bride's Awakening	The Bride's Awakening	Leonard, Robert Z.	Robert Z. Leonard	tt0008927	
1746497578	1918	1918	Vive La France!	Vive la France!	Neill, R. William	Roy William Neill	tt0009766	
1746503639	1918	1918	Hugon, the Mighty	Hugon, the Mighty	Sturgeon, Rollin S.	Rollin S. Sturgeon	tt0009210	
1746454438	1995	1995	Restoration	Restoration	Hoffman, Michael	Michael Hoffman	tt0114272	
1746393654	1981	1980	Caboblanco	Caboblanco	Thompson, J. Lee	J. Lee Thompson	tt0080485	
1746410579	1984	1984	Streets of Fire	Streets of Fire	Hill, Walter	Walter Hill	tt0088194	

1746492579	1944	1944	That's My Baby	That's My Baby!	Berke, William	William Berke	tt0037361
1746507775	1925	1925	If Marriage Fails	If Marriage Fails	Ince, John	John Ince	tt0015940
1746210831	1972	1972	The Legend of Boggy Creek	The Legend of Boggy Creek	Pierce, Charles B.	Charles B. Pierce	tt0068837
1769970539	1967	1967	Depraved!	Depraved!	Milligan, Andy	Andy Milligan	tt0216681
1746475709	1948	1948	The Big Clock	The Big Clock	Farrow, John	John Farrow	tt0040160
1746583561	1931	1931	The Champ	The Champ	Vidor, King	King Vidor	tt0021730
1746507595	1949	1949	Big Jack	Big Jack	Thorpe, Richard	Richard Thorpe	tt0041176
1746585469	1939	1939	Bad Little Angel	Bad Little Angel	Thiele, William	Wilhelm Thiele	tt0031072
1746561811	1957	1956	Rock, Pretty Baby	Rock, Pretty Baby	Bartlett, Richard	Richard Bartlett	tt0049683
1746524179	1915	1915	The Pretty Sister of Jose	The Pretty Sister of Jose	Dwan, Allan	Allan Dwan	tt0005922
1746554085	1963	1963	The Birds	The Birds	Hitchcock, Alfred	Alfred Hitchcock	tt0056869
1746545138	1940	1940	Billy the Kid Outlawed	Billy the Kid Outlawed	Stewart, Peter	Sam Newfield	tt0162197
1746585279	1930	1930	Way Out West	Way Out West	Niblo, Fred	Fred Niblo	tt0021533
1746392961	1985	1984	Ghoulies	Ghoulies	Bercovici, Luca	Luca Bercovici	tt0089200
1746438610	1942	1942	The Bugle Sounds	The Bugle Sounds	Simon, S. Sylvan	S. Sylvan Simon	tt0033438
1745309157	2005	2005	Guess Who	Guess Who	Sullivan, Kevin Rodney	Kevin Rodney Sullivan	tt0372237
1746561172	1932	1932	The Expert	The Expert	Mayo, Archie	Archie Mayo	tt0022864
1746453841	1941	1941	A Shot in the Dark	A Shot in the Dark	McGann, William	William C. McGann	tt0034185
1746583968	1939	1939	Wall Street Cowboy	Wall Street Cowboy	Kane, Joseph	Joseph Kane	tt0032107
1746258003	1980	1980	Stardust Memories	Stardust Memories	Allen, Woody	Woody Allen	tt0081554
1746492092	1947	1947	Michigan Kid	The Michigan Kid	Taylor, Ray	Ray Taylor	tt0039622
1746562334	1955	1955	The View from Pompey's Head	The View from Pompey's Head	Dunne, Philip	Philip Dunne	tt0048788
1746573337	1932	1932	Hell's Highway	Hell's Highway	Brown, Rowland	Rowland Brown	tt0022990
1745340252	2002	2002	Heaven	Heaven	Tykwer, Tom	Tom Tykwer	tt0246677
1746393777	1985	1985	Just One of the Guys	Just One of the Guys	Gottlieb, Lisa	Lisa Gottlieb	tt0089393
1746578041	1935	1935	Hitch Hike Lady	Hitch Hike Lady	Scotto, Aubrey	Aubrey Scotto	tt0027750
1745341452	2009	2009	The Slammin' Salmon	The Slammin' Salmon	Heffernan, Kevin	Kevin Heffernan	tt1135525
1746508403	1924	1924	The Last White Man	The Last White Man	Mattison, Frank S.	Frank S. Mattison	tt0330547
1746584745	1930	1930	Big Money	Big Money	Mack, Russell	Russell Mack	tt0020688
1746562102	1953	1953	Jeopardy	Jeopardy	Sturges, John	John Sturges	tt0045932
1746514259	1915	1915	The Politicians	The Politicians	Angeles, Bert	Bert Angeles	tt0005908
1746418506	1940	1940	My Love Came Back	My Love Came Back	Bernhardt, Kurt	Curtis Bernhardt	tt0032829
1746439708	1998	1997	George B	George B.	Lea, Eric	Eric Lea	tt0119188
1746492728	1944	1944	The Mummy's Ghost	The Mummy's Ghost	LeBorg, Reginald	Reginald Le Borg	tt0037099
1746436033	1943	1943	The Fighting Buckaroo	The Fighting Buckaroo	Berke, William	William Berke	tt0177770
1746495792	1920	1920	Risky Business	Risky Business	Harris, Harry; Sturgeon, Rollin	Harry B. Harris	tt0011631
1746584264	1936	1936	The Murder of Dr. Harrigan	The Murder of Dr. Harrigan	McDonald, Frank	Frank McDonald	tt0028002
1746583610	1931	1931	Desert Vengeance	Desert Vengeance	King, Louis	Louis King	tt0021795
1746523479	1923	1922	The Power of a Lie	The Power of a Lie	Archainbaud, George	George Archainbaud	tt0013507
1746573875	1958	1958	Life Begins at 17	Life Begins at 17	Dreifuss, Arthur	Arthur Dreifuss	tt0051862
1746554741	1964	1964	Circus World	Circus World	Hathaway, Henry	Henry Hathaway	tt0057952
1746235278	1973	1973	Wicked, Wicked	Wicked, Wicked	Bare, Richard L.	Richard L. Bare	tt0070916
1746563664	1952	1951	Decision Before Dawn	Decision Before Dawn	Litvak, Anatole	Anatole Litvak	tt0043459
1746421184	1990	1990	Teenage Mutant Ninja Turtles	Teenage Mutant Ninja Turtles	Barron, Steve	Steve Barron	tt0100758
1746574647	1937	1937	Borderland	Borderland	Watt, Nate	Nate Watt	tt0028650
1746509054	1925	1925	The Keeper of the Bees	The Keeper of the Bees	Meehan, James Leo	James Leo Meehan	tt0015972
1746496237	1920	1920	The Dancin' Fool	The Dancin' Fool	Wood, Sam	Sam Wood	tt0011089
1746198130	1966	1966	10:30 P.M. Summer	10:30 P.M. Summer	Dassin, Jules	Jules Dassin	tt0060045
1746497259	1919	1919	The Challenge of Chance	The Challenge of Chance	Revier, Harry	Harry Revier	tt0009997
1746582587	1938	1938	Up the River	Up the River	Werker, Alfred	Alfred L. Werker	tt0030920
1746580225	1935	1935	Too Tough to Kill	Too Tough to Kill	Lederman, D. Ross	D. Ross Lederman	tt0027122
1746491807	1946	1946	Hot Cargo	Hot Cargo	Landers, Lew	Lew Landers	tt0038618
1746580388	1938	1938	Girls' School	Girls' School	Brahm, John	John Brahm	tt0030184
1746504610	1921	1920	The Witch's Lure	Witch's Lure		Nat G. Deverich	tt0203207
1746585309	1929	1929	This Is Heaven	This Is Heaven	Santell, Alfred	Alfred Santell	tt0020493
1745374772	2008	2008	High School Musical 3: Senior Year	High School Musical 3: Senior Year	Ortega, Kenny	Kenny Ortega	tt0962726
1746574390	1961	1961	The Second Time Around	The Second Time Around	Sherman, Vincent	Vincent Sherman	tt0055421

1746500700	1925	1925	Paint and Powder	Paint and Powder		Hunt Stromberg	tt0016195
1746473672	1946	1946	Song of the Sierras	Song of the Sierras	Drake, Oliver	Oliver Drake	tt0281208
1746584738	1930	1930	Roadhouse Nights	Roadhouse Nights	Henley, Hobart	Hobart Henley	tt0021305
1746503898	1919	1919	Thou Shalt Not	Thou Shalt Not	Brabin, Charles J.	Charles Brabin	tt0010773
1746515067	1920	1920	The Fear Market	The Fear Market	Webb, Kenneth	Kenneth S. Webb	tt0011177
1746434616	1999	1999	Mr. Death: The Rise and Fall of Fred A. Leuchter,	Mr. Death: The Rise and Fall of Fred A. Leuch	Morris, Errol	Errol Morris	tt0192335
1746514661	1915	1915	Pearls of Temptation	Pearls of Temptation			tt0005877
1746422512	1994	1994	Blankman	Blankman	Binder, Mike	Mike Binder	tt0109288
1746501117	1916	1916	The Innocent Lie	The Innocent Lie	Olcott, Sidney	Sidney Olcott	tt0006860
1746520955	1948	1948	When My Baby Smiles at Me	When My Baby Smiles at Me	Lang, Walter	Walter Lang	tt0040962
1746419195	1987	1987	The Believers	The Believers	Schlesinger, John	John Schlesinger	tt0092632
1746580329	1935	1935	The Informer	The Informer	Ford, John	John Ford	tt0026529
1745340503	2004	2004	You Got Served	You Got Served	Stokes, Christopher B.	Chris Stokes	tt0365957
1746508118	1926	1926	Flame of the Argentine	Flame of the Argentine	Dillon, Edward	Edward Dillon	tt0016877
1746566504	1933	1932	Robbers' Roost	Robbers' Roost	King, Louis	David Howard	tt0024505
1745341250	2010	2010	Just Wright	Just Wright	Hamri, Sanaa	Sanaa Hamri	tt1407061
1746582086	1929	1929	Taming of the Shrew	The Taming of the Shrew	Taylor, Sam	Sam Taylor	tt0020479
1746581093	1951	1951	The Lady and the Bandit	The Lady and the Bandit	Murphy, Ralph	Ralph Murphy	tt0043722
1746571698	1910	1910	The Debt Repaid	The Debt Repaid		William F. Haddock	tt2236908
1746496578	1918	1918	Back to the Woods	Back to the Woods	Irving, George	George Irving	tt0008861
1746465222	1945	1945	First Yank into Tokyo	First Yank Into Tokyo	Douglas, Gordon	Gordon Douglas	tt0037701
1746473650	1944	1944	West of the Rio Grande	West of the Rio Grande	Hillyer, Lambert	Lambert Hillyer	tt0037454
1746508870	1926	1926	The Devil's Circus	The Devil's Circus	Christensen, Benjamin	Benjamin Christensen	tt0016783
1745271882	2001	2001	Behind Enemy Lines	Behind Enemy Lines	Moore, John	John Moore	tt0159273
1746584839	1930	1930	The Cohens and the Kellys in Scotland	The Cohens and the Kellys in Scotland	Craft, William James	William James Craft	tt0020777
1746520871	1925	1925	Drug Store Cowboy	The Drug Store Cowboy	Frame, Park	Park Frame	tt0015766
1746422528	1994	1994	The Ref	The Ref	Demme, Ted	Ted Demme	tt0110955
1746420917	1940	1940	Bitter Sweet	Bitter Sweet	Van Dyke, W. S.	W.S. Van Dyke	tt0032256
1746580404	1938	1938	Hold That Kiss	Hold That Kiss	Marin, Edwin L.	Edwin L. Marin	tt0030240
1746569938	1957	1957	The Three Faces of Eve	The Three Faces of Eve	Johnson, Nunnally	Nunnally Johnson	tt0051077
1745293444	2006	2006	John Tucker Must Die	John Tucker Must Die	Thomas, Betty	Betty Thomas	tt0455967
1746524442	1910	1910	Method in His Madness	Method in His Madness		Gilbert M. 'Broncho Billy' Anderson	tt0372421
1745293972	2007	2007	Perfect Stranger	Perfect Stranger	Foley, James	James Foley	tt0457433
1746503680	1918	1918	Leap to Fame	Leap to Fame	Blackwell, Carlyle	Carlyle Blackwell	tt0009283
1746501298	1915	1915	A Royal Family	A Royal Family	Nigh, William	William Nigh	tt0005983
1746464852	1942	1941	Fighting Bill Fargo	Fighting Bill Fargo	Taylor, Ray	Ray Taylor	tt0033601
1746235265	1976	1975	Winterhawk	Winterhawk	Pierce, Charles B.	Charles B. Pierce	tt0075432
1746572467	1931	1931	Range Law	Range Law	Rosen, Phil	Phil Rosen	tt0022294
1746583987	1936	1936	Here Comes Trouble	Here Comes Trouble	Seiler, Lewis	Lewis Seiler	tt0027736
1746216713	1969	1969	Midnight Cowboy	Midnight Cowboy	Schlesinger, John	John Schlesinger	tt0064665
1746516042	1921	1921	Little Lord Fauntleroy	Little Lord Fauntleroy	Green, Alfred E.; Pickford, Jack	Alfred E. Green	tt0012397
1746582214	1928	1928	Wickedness Preferred	Wickedness Preferred	Henley, Hobart	Hobart Henley	tt0019578
1746198529	1967	1967	The Legend of the Boy and the Eagle	The Legend of the Boy and the Eagle	Couffer, Jack	Jack Couffer	tt0061904
1746584527	1930	1930	Young Man of Manhattan	Young Man of Manhattan	Bell, Monta	Monta Bell	tt0021568
1745293804	2007	2007	Oswald's Ghost	Oswald's Ghost	Stone, Robert	Robert Stone	tt0938321
1746453590	1943	1943	He Hired the Boss	He Hired the Boss	Loring, Thomas Z.	Thomas Z. Loring	tt0035974
1746423351	1996	1996	Caught	Caught	Young, Robert M.	Robert M. Young	tt0115847
1746438615	1943	1943	Captive Wild Woman	Captive Wild Woman	Dmytryk, Edward	Edward Dmytryk	tt0035713
1769969699	1911	1911	The Portrait	The Portrait		Thomas H. Ince	tt0358646
1746513070	1927	1927	Finnegan's Ball	Finnegan's Ball	Hogan, James P.	James P. Hogan	tt0017884
1746569523	1957	1957	Portland Exposé	Portland Exposé	Schuster, Harold	Harold D. Schuster	tt0124076
1745340452	2002	2002	Equilibrium	Equilibrium	Wimmer, Kurt	Kurt Wimmer	tt0238380
1746584312	1936	1936	Dangerous Waters	Dangerous Waters	Hillyer, Lambert	Lambert Hillyer	tt0027498
1746577691	1933	1933	The Barbarian	The Barbarian	Wood, Sam	Sam Wood	tt0023778
1746567810	1952	1952	California Conquest	California Conquest	Landers, Lew	Lew Landers	tt0044466
1769970419	1967	1967	Bachelor's Dream	The Bachelor's Dreams		Stephen C. Apostolof	tt0123398
1745358879	2002	2002	Kung Pow: Enter the Fist	Kung Pow: Enter the Fist	Oedekerk, Steve	Steve Oedekerk	tt0240468

1746409500	1981	1981	Endless Love	Endless Love	Zeffirelli, Franco	Franco Zeffirelli	tt0082329
1746574202	1956	1956	Ransom!	Ransom!	Segal, Alex	Alex Segal	tt0049656
1746523216	1917	1917	The Girl Without a Soul	The Girl Without a Soul	Collins, John H.	John H. Collins	tt0008004
1746503252	1918	1918	Me Und Gott	Me und Gott	Gittens, Wyndham	Wyndham Gittens	tt0186338
1746582469	1938	1938	Mr. Boggs Steps Out	Mr. Boggs Steps Out	Wiles, Gordon	Gordon Wiles	tt0029266
1746514452	1923	1923	Scarred Hands	Scarred Hands	Smith, Cliff	Clifford Smith	tt0014443
1746503451	1922	1922	Living Lies	Living Lies	Chautard, Emile	Emile Chautard	tt0013325
1746513408	1922	1922	Guilty	Guilty		Ranger Bill Miller	tt0361680
1746502412	1916	1916	The Little Girl Next Door	The Little Girl Next Door	Baker, Richard Foster; Coan, M. Blair	Richard Foster Baker	tt0163061
1746533224	1916	1916	Indiana	The Indian	Beal, Frank	David Miles	tt0483414
1769974357	1916	1916	The Wraith of Haddon Towers	The Wraith of Haddon Towers		Arthur Maude	tt0437649
1746514681	1916	1916	The Fable of the Two Philanthropic Sons	The Fable of the Two Philanthropic Sons		Richard Foster Baker	tt0006659
1746502555	1915	1915	The Daughter of the People	The Daughter of the People	Dawley, J. Searle	J. Searle Dawley	tt0005176
1769970840	1915	1915	Is Christmas a Bore?	Is Christmas a Bore?	Drew, Sidney	Sidney Drew	tt0154652
1746513790	1915	1915	Pool Sharks	Pool Sharks	Middleton, Edwin	Edwin Middleton	tt0005910
1746532646	1914	1914	The Policewoman	The Policewoman			tt0177121
1746512410	1913	1913	The Seed of the Fathers	The Seed of the Fathers	Taylor, S. E. V.	Stanner E.V. Taylor	tt0003363
1746184138	1910	1910	Across the Plains	Across the Plains	Boggs, Frank	Francis Boggs	tt0001102
1746394032	1983	1983	Anarchism in America?	Anarchism in America	Sucher, Joel; Fischler, Steven	Steven Fischler	tt0186820
1746248877	1980	1980	Today Is for the Championship	Today Is for the Championship	Weisburd, Dan	Dan E. Weisburd	tt0365849
1745341025	2002	2002	I Don't Know Jack	I Don't Know Jack	Leavens, Chris	Chris Leavens	tt0292553
1746578944	1951	1951	Abilene Trail	Abilene Trail	Collins, Lewis	Lewis D. Collins	tt0043257
1746578799	1950	1950	Vienna Art Treasures	Vienna Art Treasures			tt0335524
1746578646	1950	1950	Stella	Stella	Binyon, Claude	Claude Binyon	tt0043000
1746508073	1949	1949	The Devil's Henchman	The Devil's Henchman	Friedman, Seymour	Seymour Friedman	tt0041293
1746465291	1944	1944	Return of the Ape Man	Return of the Ape Man	Rosen, Philip	Phil Rosen	tt0037217
1746512923	1928	1928	Last Lap	The Last Lap	Mitchell, Bruce	Bruce Mitchell	tt0329280
1746542758	1927	1927	The Mystery Brand	The Mystery Brand	Wilson, Ben	Ben F. Wilson	tt0018186
1746509332	1926	1926	The Fighting Doctor	The Fighting Doctor	Bradbury, Robert North	Robert N. Bradbury	tt0016859
1746500457	1925	1925	Fun's Fun	Fun's Fun	Ray, Albert	Albert Ray	tt0248025
1746520733	1925	1925	Hurricane Hal	Hurricane Hal		John P. McCarthy	tt0356715
1746520145	1924	1924	Lawless Men	Lawless Men	Hart, Neal	Neal Hart	tt0330549
1746500617	1924	1924	Western Fate	Western Fate	Holt, George	George Holt	tt0177383
1746464761	1941	1941	The Gang's All Here	The Gang's All Here	Yarbrough, Jean	Jean Yarbrough	tt0033646
1746464970	1941	1941	Ladies in Retirement	Ladies in Retirement	Vidor, Charles	Charles Vidor	tt0033802
1746436358	1940	1940	Calling All Husbands	Calling All Husbands	Smith, Noel	Noel M. Smith	tt0032297
1746580769	1938	1938	The Land of Fighting Men	Land of Fighting Men	James, Alan	Alan James	tt0030343
1746580520	1938	1938	Return to Life	Return to Life	Cartier, Henri	Henri Cartier	tt0030676
1746582503	1936	1936	Ghost Town	Ghost Town	Fraser, Harry	Harry L. Fraser	tt0028927
1746580641	1936	1936	Chatterbox	Chatterbox	Nicholls, George	George Nichols Jr.	tt0027443
1746576964	1959	1959	Roadracers	Roadracers	Swerdloff, Arthur	Arthur Swerdloff	tt0230702
1746550237	1956	1956	Walk the Dark Street	Walk the Dark Street	Ordung, Wyott	Wyott Ordung	tt0049932
1746566314	1954	1954	Jungle Gents	Jungle Gents	Bernds, Edward	Edward Bernds	tt0047138
1769971132	1969	1969	More Head	More Head	Glick, Wizard; Ruttenberg, Robert	Wizard Glick	tt0251860
1746216652	1967	1967	Sadismo	Sadismo			tt0217778
1746235163	1967	1967	Anything for Money	Anything for Money	Sarno, Joe	Joseph W. Sarno	tt0060113
1746200259	1967	1967	The Strange Fetishes	The Strange Fetishes	Blancocello, Enrico	Enrico Blancocello	tt0246956
1769970390	1967	1967	Sex Club International	Sex Club International	Mahon, Barry	Barry Mahon	tt0147455
1769970439	1967	1967	Candy's Lustful Nature	Candy's Lustful Nature	Millard, Nicholas	Nick Millard	tt1467247
1746553651	1965	1964	Dog Star Man	Dog Star Man	Brakhage, Stan	Stan Brakhage	tt5896468
1769969442	1965	1965	The Hot Bed	The Hot Bed	Epstein, Harry	Harry Epstein	tt0219814
1746577065	1963	1963	Flipper	Flipper	Clark, James B.	James B. Clark	tt0057063
1746578752	1963	1963	The Young and the Brave	The Young and the Brave	Lyon, Francis D.	Francis D. Lyon	tt0057709
1746577794	1935	1935	Kid Courageous	Kid Courageous	Bradbury, Robert N.	Robert N. Bradbury	tt0026567
1746576022	1931	1930	Mystery of Life	The Mystery of Life	Cochrane, George	George Cochrane	tt0151699
1746585085	1930	1930	Leave It to Lester	Leave It to Lester	Cambria, Frank; Cozine, Ray	Frank Cambria	tt0021058
1746513044	1922	1922	F. O. B. Africa	F.O.B. Africa	Warner, Jack L.	Jack L. Warner	tt1402398

1745395686	2011	2010	The Woodmans	The Woodmans	Willis, Scott	Scott Willis	tt1610516
1746210525	1970	1970	The Kremlin Letter	The Kremlin Letter	Huston, John	John Huston	tt0065950
1746583219	1950	1950	Pagan Love Song	Pagan Love Song	Alton, Robert	Robert Alton	tt0042829
1746584861	1929	1929	Melody Lane	Melody Lane	Hill, Robert F.	Robert F. Hill	tt0020160
1746520344	1950	1950	Boy from Indiana	The Boy from Indiana	Rawlins, John	John Rawlins	tt0042278
1746573420	1931	1931	Secrets of a Secretary	Secrets of a Secretary	Abbott, George	George Abbott	tt0022359
1746581566	1910	1910	An Interrupted Courtship	An Interrupted Courtship			tt0256831
1746475517	1948	1948	The Strawberry Roan	The Strawberry Roan	English, John	John English	tt0040840
1746532994	1916	1916	Embers	Embers	Maude, Arthur	Arthur Maude	tt0157574
1746581924	1928	1929	The Sideshow	The Side Show	Kenton, Erle C.	Doc Salomon	tt1706507
1746486830	1920	1920	Girls Don't Gamble	Girls Don't Gamble	Butler, Fred J.; McClung, Hugh C.	Fred J. Butler	tt0012220
1746520720	1925	1925	As Man Desires	As Man Desires	Cummings, Irving	Irving Cummings	tt0015579
1746542630	1926	1926	Behind the Front	Behind the Front	Sutherland, Edward	A. Edward Sutherland	tt0016638
1746491805	1948	1948	Adventures of Gallant Bess	Adventures of Gallant Bess	Landers, Lew	Lew Landers	tt0040078
1746454672	1946	1946	Danger Woman	Danger Woman	Collins, Lewis D.	Lewis D. Collins	tt0038446
1746509445	1926	1926	The Boy Friend	The Boy Friend	Bell, Monta	Monta Bell	tt0016677
1746508249	1924	1924	Roulette	Roulette	Taylor, S. E. V.	Stanner E.V. Taylor	tt0015293
1746562033	1953	1953	Inferno	Inferno	Baker, Roy	Roy Ward Baker	tt0045911
1746503026	1927	1926	The Fire Brigade	The Fire Brigade	Nigh, William	William Nigh	tt0016874
1746514302	1916	1916	The Aryan	The Aryan	Hart, William S.	Reginald Barker	tt0006375
1746584327	1936	1936	Petticoat Fever	Petticoat Fever	Fitzmaurice, Geo.	George Fitzmaurice	tt0028097
1746581303	1961	1961	20,000 Eyes	20,000 Eyes	Leewood, Jack	Jack Leewood	tt0054585
1746515793	1921	1921	Cold Steel	Cold Steel	MacDonald, Sherwood	Sherwood MacDonald	tt0012058
1746475268	1948	1948	Sitting Pretty	Sitting Pretty	Lang, Walter	Walter Lang	tt0040795
1746580783	1934	1934	Judge Priest	Judge Priest	Ford, John	John Ford	tt0025335
1746269917	1980	1980	Borderline	Borderline	Freedman, Jerrold	Jerrold Freedman	tt0080465
1746584135	1935	1935	Sweet Music	Sweet Music	Green, Alfred E.	Alfred E. Green	tt0027064
1746532758	1915	1915	Her Reckoning	Her Reckoning	Horan, Charles	Charles Horan	tt0005476
1746581562	1950	1950	Sierra	Sierra	Green, Alfred E.	Alfred E. Green	tt0042963
1746492474	1945	1945	Here Come the Co-Eds	Here Come the Co eds	Yarbrough, Jean	Jean Yarbrough	tt0037771
1746453769	1943	1943	The Dancing Masters	The Dancing Masters	St. Clair, Mal	Malcolm St. Clair	tt0035777
1746523589	1923	1923	The Eternal Struggle	The Eternal Struggle	Barker, Reginald	Reginald Barker	tt0014024
1746569595	1958	1958	Country Music Holiday	Country Music Holiday	Ganzer, Alvin	Alvin Ganzer	tt0051493
1746219022	1979	1979	Butch and Sundance: The Early Days	Butch and Sundance: The Early Days	Lester, Richard	Richard Lester	tt0078919
1746585270	1929	1929	It Can Be Done	It Can Be Done	Newmeyer, Fred	Fred C. Newmeyer	tt0020033
1746453795	1941	1941	Hurricane Smith	Hurricane Smith	Vorhaus, Bernard	Bernard Vorhaus	tt0034878
1746509197	1925	1925	School for Wives	School for Wives	Halperin, Victor Hugo	Victor Halperin	tt0016323
1746420143	1983	1983	The Big Chill	The Big Chill	Kasdan, Lawrence	Lawrence Kasdan	tt0085244
1746523280	1923	1923	Ashes of Vengeance	Ashes of Vengeance		Frank Lloyd	tt0013841
1746500270	1924	1924	What Three Men Wanted	What Three Men Wanted	Burns, Paul	Paul Burns	tt0015483
1746523314	1910	1910	Love, Luck and Gasoline	Love, Luck and Gasoline		Wilfrid North	tt0338215
1746584842	1930	1930	Second Choice	Second Choice	Bretherton, Howard	Howard Bretherton	tt0021350
1746394380	1985	1985	Dim Sum: A Little Bit of Heart	Dim Sum: A Little Bit of Heart	Wang, Wayne	Wayne Wang	tt0087150
1746569716	1954	1954	Ma and Pa Kettle at Home	Ma and Pa Kettle at Home	Lamont, Charles	Charles Lamont	tt0047197
1746500581	1926	1926	Mannequin	Mannequin	Cruze, James	James Cruze	tt0017117
1746520721	1948	1948	Unknown Island	Unknown Island	Bernhard, Jack	Jack Bernhard	tt0040920
1746520043	1925	1925	Parisian Love	Parisian Love	Gasnier, Louis	Louis J. Gasnier	tt0016201
1745393662	2010	2010	Life As We Know It	Life as We Know It	Berlanti, Greg	Greg Berlanti	tt1055292
1746573490	1931	1931	Shanghaied Love	Shanghaied Love	Seitz, George B.	George B. Seitz	tt0022369
1746575554	1931	1931	Flying High	Flying High	Riesner, Charles F.	Charles Reisner	tt0021879
1746410528	1987	1987	House II: The Second Story	House II: The Second Story	Wiley, Ethan	Ethan Wiley	tt0093220
1746453506	1942	1942	Sealed Lips	Sealed Lips	Waggner, George	George Waggner	tt0034163
1746487132	1920	1920	Chains of Evidence	Chains of Evidence	Fitzgerald, Dallas M.	Dallas M. Fitzgerald	tt0011047
1746570029	1934	1934	Money Means Nothing	Money Means Nothing	Cabanne, Christy	Christy Cabanne	tt0025513
1746578938	1962	1962	Tonight for Sure!	Tonight for Sure	Coppola, Francis	Francis Ford Coppola	tt0153167
1746562077	1955	1955	Duel on the Mississippi	Duel on the Mississippi	Castle, William	William Castle	tt0048023
1769971089	1968	1968	Candy	Candy	Marquand, Christian	Christian Marquand	tt0062776

1746520639	1927	1927	Million Dollar Mystery	Million Dollar Mystery	Hunt, Charles J.	Charles J. Hunt	tt0018160
1746583382	1951	1951	He Ran All the Way	He Ran All the Way	Berry, John; Berry, John	John Berry	tt0043625
1746474141	1944	1944	I Love a Soldier	I Love a Soldier	Sandrich, Mark	Mark Sandrich	tt0036938
1746580378	1937	1937	Westbound Mail	Westbound Mail	Blangsted, Folmer	Folmar Blangsted	tt0134210
1746563361	1951	1951	Show Boat	Show Boat	Sidney, George	George Sidney	tt0044030
1746408657	1988	1988	Big Top Pee-wee	Big Top Pee wee	Kleiser, Randal	Randal Kleiser	tt0094744
1746421290	1990	1990	The Exorcist III	The Exorcist III	Blatty, William Peter	William Peter Blatty	tt0099528
1769970392	1966	1966	Brothel	Brothel	Vehr, Bill	Bill Vehr	tt0212003
1746420893	1990	1990	Miami Blues	Miami Blues	Armitage, George	George Armitage	tt0100143
1746218703	1971	1971	Directed by John Ford	Directed by John Ford	Bogdanovich, Peter	Peter Bogdanovich	tt0066997
1746434272	1989	1989	Tap	Tap	Castle, Nick	Nick Castle	tt0098442
1746409455	1991	1991	The Hard Way	The Hard Way	Badham, John	John Badham	tt0102004
1746437420	1943	1943	The Seventh Victim	The Seventh Victim	Robson, Mark	Mark Robson	tt0036341
1746453647	1941	1941	Moonlight in Hawaii	Moonlight in Hawaii	Lamont, Charles	Charles Lamont	tt0033919
1746573475	1932	1932	The First Year	The First Year	Howard, William K.	William K. Howard	tt0022892
1745294051	2007	2007	Delta Farce	Delta Farce	Harding, C. B.	C.B. Harding	tt0800003
1746492088	1946	1946	They Made Me a Killer	They Made Me a Killer	Thomas, William C.	William C. Thomas	tt0039023
1746577549	1934	1934	Limehouse Blues	Limehouse Blues	Hall, Alexander	Alexander Hall	tt0025399
1746453942	2000	2000	Duets	Duets	Paltrow, Bruce	Bruce Paltrow	tt0134630
1746524721	1910	1910	Over Silent Paths	Over Silent Paths	Griffith, D. W.	D.W. Griffith	tt0001351
1746438317	1944	1944	Roger Touhy, Gangster	Roger Touhy, Gangster	Florey, Robert	Robert Florey	tt0036846
1745341476	2004	2004	The Future of Food	The Future of Food	Garcia, Deborah Koons	Deborah Koons	tt0427276
1746584279	1937	1937	Kid Galahad	Kid Galahad	Curtiz, Michael	Michael Curtiz	tt0029080
1746184544	1910	1910	Branding a Thief	Branding a Thief		William F. Haddock	tt0835750
1746436773	1992	1992	Mr. Saturday Night	Mr. Saturday Night	Crystal, Billy	Billy Crystal	tt0104928
1746554406	1964	1964	The Chalk Garden	The Chalk Garden	Neame, Ronald	Ronald Neame	tt0057933
1746235896	1974	1974	The Sugarland Express	The Sugarland Express	Spielberg, Steven	Steven Spielberg	tt0072226
1746574729	1934	1934	Riding Speed	Riding Speed		Jay Wilsey	tt0133179
1746560026	1965	1965	Who Killed Teddy Bear?	Who Killed Teddy Bear	Cates, Joseph	Joseph Cates	tt0059909
1746561000	1965	1965	Tickle Me	Tickle Me	Taurog, Norman	Norman Taurog	tt0056585
1746500436	1926	1926	The Millionaire Policeman	The Millionaire Policeman	Le Saint, Edward J.	Edward LeSaint	tt0017153
1746436579	1941	1941	Two Gun Sheriff	Two Gun Sheriff	Sherman, George	George Sherman	tt0034324
1746492723	1943	1943	Spy Train	Spy Train	Young, Harold	Harold Young	tt0036383
1746578048	1938	1938	Gateway	Gateway	Werker, Alfred	Alfred L. Werker	tt0030173
1746585099	1930	1930	Men Without Women	Men Without Women	Ford, John	John Ford	tt0021140
1746500398	1926	1925	The Feud Woman	The Feud Woman	Williamson, R. E.	Robin Williamson	tt0357658
1745302720	2005	2005	Four Brothers	Four Brothers	Singleton, John	John Singleton	tt0430105
1746550227	1955	1955	City of Shadows	City of Shadows	Witney, William	William Witney	tt0047942
1746583696	1931	1931	The Sky Spider	The Sky Spider	Thorpe, Richard	Richard Thorpe	tt0022399
1746584458	1928	1928	Wheel of Chance	Wheel of Chance	Santell, Alfred	Alfred Santell	tt0019569
1746574165	1961	1961	Maeva	Maeva	Bonsignori, Umberto	Umberto Bonsignori	tt0055120
1746464887	1945	1945	They Were Expendable	They Were Expendable	Ford, John	John Ford	tt0038160
1746485877	1919	1919	The Volcano	The Volcano	Irving, George	George Irving	tt0010851
1746569505	1955	1955	It's a Dog's Life	It's a Dog's Life	Hoffman, Herman	Herman Hoffman	tt0048217
1746233537	1968	1968	The Girl Grabbers	The Girl Grabbers	Nuchtern, Simon	Simon Nuchtern	tt0125765
1746520753	1926	1926	Law of the Snow Country	The Law of the Snow Country	Hurst, Paul	Paul Hurst	tt0017061
1746438162	1942	1942	Let's Get Tough!	Let's Get Tough!	Fox, Wallace	Wallace Fox	tt0034973
1746439474	1941	1941	Rise and Shine	Rise and Shine	Dwan, Allan	Allan Dwan	tt0034112
1746585152	1928	1928	The Bantam Cowboy	The Bantam Cowboy	King, Louis	Louis King	tt0377453
1746500735	1926	1926	The Night Watch	The Night Watch	Caldwell, Fred	Fred Caldwell	tt0017208
1745375004	2008	2008	Sleepwalking	Sleepwalking	Maher, William	Bill Maher	tt0888693
1746573945	1956	1956	Yaqui Drums	Yaqui Drums	Yarbrough, Jean	Jean Yarbrough	tt0049970
1746524153	1914	1914	The Merchant of Venice	The Merchant of Venice	Smalley, Phillips; Weber, Lois	Phillips Smalley	tt0004325
1746576961	1961	1961	Blue Hawaii	Blue Hawaii	Taurog, Norman	Norman Taurog	tt0054692
1746520569	1926	1926	Josselyn's Wife	Josselyn's Wife	Thorpe, Richard	Richard Thorpe	tt0017011
1746561395	1932	1932	Two Fisted Law	Two Fisted Law	Lederman, D. Ross	D. Ross Lederman	tt0023631
1746394029	1984	1984	Just the Way You Are	Just the Way You Are	Molinaro, Edouard	Édouard Molinaro	tt0087525

1745359816	2004	2004	Johnson Family Vacation	Johnson Family Vacation	Erskin, Christopher	Christopher Erskin	tt0359517
1746503700	1917	1917	The Inspirations of Harry Larrabee	The Inspirations of Harry Larrabee	Bracken, Bertram	Bertram Bracken	tt0137023
1746570431	1934	1934	Orient Express	Orient Express	Martin, Paul	Paul Martin	tt0025608
1746582408	1937	1937	The Sheik Steps Out	The Sheik Steps Out	Pichel, Irving	Irving Pichel	tt0029553
1769969403	1911	1911	The Derelict	The Derelict		Phillips Smalley	tt1284458
1746424050	1942	1942	Wake Island	Wake Island	Farrow, John	John Farrow	tt0035530
1746500038	1945	1945	Swingin' on a Rainbow	Swingin' on a Rainbow	Beaudine, William	William Beaudine	tt0038144
1746578833	1959	1958	Gideon of Scotland Yard	Gideon of Scotland Yard	Ford, John	John Ford	tt0051655
1746235036	1977	1977	The Car	The Car	Silverstein, Elliot	Elliot Silverstein	tt0075809
1746577974	1938	1938	The Marines Are Here	The Marines Are Here	Rosen, Phil	Phil Rosen	tt0030419
1746467185	1917	1917	Pots-and-Pans Peggy	Pots and Pans Peggy	Moore, Eugene	Eugene Moore	tt0008445
1746534788	1922	1922	The Woman Conquers	The Woman Conquers	Forman, Tom	Tom Forman	tt0013778
1746580231	1938	1938	The Dawn Patrol	The Dawn Patrol	Goulding, Edmund	Edmund Goulding	tt0030044
1746465037	1942	1942	A-Haunting We Will Go	A Haunting We Will Go	Werker, Alfred	Alfred L. Werker	tt0034424
1746512613	1927	1927	White Flannels	White Flannels	Bacon, Lloyd	Lloyd Bacon	tt0018570
1746523292	1916	1916	Fate's Boomerang	Fate's Boomerang	Crane, Frank H.	Frank Hall Crane	tt0157618
1746475277	1947	1947	The Wistful Widow of Wagon Gap	The Wistful Widow of Wagon Gap	Barton, Charles T.	Charles Barton	tt0039998
1746273823	1979	1979	Cuba	Cuba	Lester, Richard	Richard Lester	tt0079013
1746578739	1960	1960	The Angel Wore Red	The Angel Wore Red	Johnson, Nunnally	Nunnally Johnson	tt0053601
1746567229	1933	1933	Too Much Harmony	Too Much Harmony	Sutherland, Edward	A. Edward Sutherland	tt0024683
1746219372	1974	1973	Road Movie	Road Movie	Strick, Joseph	Joseph Strick	tt0072089
1746582327	1951	1951	Three Desperate Men	Three Desperate Men	Newfield, Sam	Sam Newfield	tt0044124
1769977813	1982	1982	Tron	TRON	Lisberger, Steven	Steven Lisberger	tt0084827
1746513394	1914	1914	Ready Money	Ready Money	Apfel, Oscar C.	Oscar Apfel	tt0004514
1746500380	1926	1926	The Arizona Streak	The Arizona Streak	De Lacy, Robert	Robert De Lacey	tt0016609
1746524300	1923	1923	The Tiger's Claw	The Tiger's Claw	Henabery, Joseph	Joseph Henabery	tt0014548
1746571359	1934	1934	Hi Nellie!	Hi, Nellie!	LeRoy, Mervyn	Mervyn LeRoy	tt0025250
1746393620	1986	1986	Raw Deal	Raw Deal	Irvin, John	John Irvin	tt0091828
1746219049	1973	1973	The Mack	The Mack	Campus, Michael	Michael Campus	tt0070350
1745375272	2008	2007	Then She Found Me	Then She Found Me	Hunt, Helen	Helen Hunt	tt0455805
1746582508	1937	1937	Mr. Dodd Takes the Air	Mr. Dodd Takes the Air	Green, Alfred E.	Alfred E. Green	tt0029267
1746583004	1939	1939	Zenobia	Zenobia	Douglas, Gordon	Gordon Douglas	tt0032158
1746577499	1936	1936	The Kid Ranger	The Kid Ranger	Bradbury, Robert N.	Robert N. Bradbury	tt0027842
1746219128	1973	1973	The Day of the Dolphin	The Day of the Dolphin	Nichols, Mike	Mike Nichols	tt0069946
1746578484	1961	1960	Girl Fever	Girl Fever	Petrushansky, Yevsie; Price, Shermar	Yevsie S. Petrushansky	tt0266586
1746580353	1939	1939	Juarez	Juarez	Dieterle, William	William Dieterle	tt0031516
1746515797	1920	1920	Greater Than Fame	Greater Than Fame	Crosland, Alan	Alan Crosland	tt0011249
1746578011	1938	1938	The Shining Hour	The Shining Hour	Borzage, Frank	Frank Borzage	tt0030743
1746410682	1988	1988	Running on Empty	Running on Empty	Lumet, Sidney	Sidney Lumet	tt0096018
1746577598	1937	1937	Wise Girl	Wise Girl	Jason, Leigh	Leigh Jason	tt0029786
1746532844	1915	1915	The Juggernaut	The Juggernaut	Ince, Ralph W.	Ralph Ince	tt0005579
1746562048	1956	1956	Diane	Diane	Miller, David	David Miller	tt0049140
1746475256	1947	1947	Vigilantes of Boomtown	Vigilantes of Boomtown	Springsteen, R. G.	R.G. Springsteen	tt0039959
1746578031	1937	1937	The Fighting Deputy	The Fighting Deputy	Newfield, Sam	Sam Newfield	tt0028870
1746563932	1953	1953	All I Desire	All I Desire	Sirk, Douglas	Douglas Sirk	tt0045492
1746567932	1934	1934	Down to Their Last Yacht	Down to Their Last Yacht	Sloane, Paul	Paul Sloane	tt0025066
1746438284	1942	1942	Outlaws of Boulder Pass	Outlaws of Boulder Pass	Newfield, Sam	Sam Newfield	tt0035162
1746496873	1918	1918	Ruling Passions	Ruling Passions	Schomer, Abraham S.	Abraham S. Schomer	tt0187469
1745302749	2007	2007	Waitress	Waitress	Shelly, Adrienne	Adrienne Shelly	tt0473308
1746453953	1998	1997	The Alarmist	The Alarmist	Dunsky, Evan	Evan Dunsky	tt0119534
1746210728	1972	1972	Where Does It Hurt?	Where Does It Hurt?	Amateau, Rod	Rod Amateau	tt0069497
1746585291	1928	1928	The Black Feather	Black Feather	Ince, John E.	John Ince	tt0018705
1746453597	1945	1945	Fog Island	Fog Island	Morse, Terry	Terry O. Morse	tt0037711
1769969491	1965	1965	"Rent-A-Girl"	Rent a Girl	Rose, William L.	William Rose	tt0146230
1746554332	1964	1964	The Guns of August	The Guns of August	Kroll, Nathan	Nathan Kroll	tt0132998
1746585172	1930	1930	Shadow Ranch	Shadow Ranch	King, Louis	Louis King	tt0021358
1746507771	1948	1948	Blondie's Secret	Blondie's Secret	Bernds, Edward	Edward Bernds	tt0040174

1746503407	1922	1922	Smudge	Smudge	Ray, Charles	Charles Ray	tt0013613
1746576906	1931	1931	Heaven on Earth	Heaven on Earth	Mack, Russell	Russell Mack	tt0021947
1746530755	1949	1949	Miss Mink of 1949	Miss Mink of 1949	Tryon, Glenn	Glenn Tryon	tt0041653
1746520127	1926	1926	The Runaway Express	The Runaway Express	Sedgwick, Edward	Edward Sedgwick	tt0017339
1746578026	1938	1938	The Sisters	The Sisters	Litvak, Anatole	Anatole Litvak	tt0030755
1746491721	1948	1948	Concert Magic	Concert Magic	Gordon, Paul	Paul Gordon	tt0220370
1746566032	1957	1957	The Bachelor Party	The Bachelor Party	Mann, Delbert	Delbert Mann	tt0050156
1746504753	1917	1917	The Yellow Bullet	The Yellow Bullet	Harvey, Harry	Harry Harvey	tt0008796
1746465130	1947	1947	Dear Ruth	Dear Ruth	Russell, William D.	William D. Russell	tt0039307
1746466887	1917	1917	The Lincoln Cycle	The Lincoln Cycle	Stahl, John M.	John M. Stahl	tt0176932
1745293693	2005	2005	The Longest Yard	The Longest Yard	Segal, Peter	Peter Segal	tt0398165
1746514472	1915	1915	Help Wanted	Help Wanted	Bosworth, Hobart	Hobart Bosworth	tt0005465
1746503885	1922	1922	Two-fisted Jefferson	Two Fisted Jefferson	Clements, Roy	Roy Clements	tt0175267
1746577808	1935	1935	Steamboat Round the Bend	Steamboat Round the Bend	Ford, John	John Ford	tt0027042
1746577540	1937	1937	The Hit Parade	The Hit Parade	Meins, Gus	Gus Meins	tt0029003
1746504211	1918	1918	The Ghost of Rosy Taylor	The Ghost of Rosy Taylor	Sloman, Edward S.	Edward Sloman	tt0009104
1746570177	1934	1934	Wednesday's Child	Wednesday's Child	Robertson, John	John S. Robertson	tt0025967
1746581870	1928	1928	Sharp Shooters	Sharp Shooters	Blystone, J. G.	John G. Blystone	tt0019370
1746454703	1941	1941	You Belong to Me	You Belong to Me	Ruggles, Wesley	Wesley Ruggles	tt0034407
1746569534	1957	1957	The Deerslayer	The Deerslayer	Neumann, Kurt	Kurt Neumann	tt0050299
1746567258	1932	1932	Vanity Street	Vanity Street	Grinde, Nicholas	Nick Grinde	tt0023653
1746555655	1956	1956	Shake, Rattle & Rock!	Shake, Rattle & Rock!	Cahn, Edward L.	Edward L. Cahn	tt0049749
1746502889	1910	1910	Taming a Husband	Taming a Husband	Griffith, D. W.	D.W. Griffith	tt0001410
1746574395	1962	1962	Boys' Night Out	Boys' Night Out	Gordon, Michael	Michael Gordon	tt0055809
1746497266	1922	1922	What's Wrong With the Women?	What's Wrong with the Women?	Neill, R. William	Roy William Neill	tt0013747
1745341597	2008	2008	Be Kind Rewind	Be Kind Rewind	Gondry, Michel	Michel Gondry	tt0799934
1746530722	1950	1950	Black Hand	Black Hand	Thorpe, Richard	Richard Thorpe	tt0041181
1745309555	2007	2006	DOA: Dead or Alive	DOA: Dead or Alive	Yuen, Corey	Corey Yuen	tt0398913
1746423261	1997	1997	Nothing to Lose	Nothing to Lose	Oedekerk, Steve	Steve Oedekerk	tt0119807
1746580700	1936	1936	The Prisoner of Shark Island	The Prisoner of Shark Island	Ford, John	John Ford	tt0028141
1746578059	1936	1936	Isle of Fury	Isle of Fury	McDonald, Frank	Frank McDonald	tt0027805
1746504560	1920	1920	The Branding Iron	The Branding Iron	Barker, Reginald	Reginald Barker	tt0011021
1746503520	1921	1921	Seven Years Bad Luck	Seven Years Bad Luck	Linder, Max	Max Linder	tt0127834
1746584471	1928	1928	Baby Mine	Baby Mine	Leonard, Robert Z.	Robert Z. Leonard	tt0018668
1745303161	2005	2005	Dirty Love	Dirty Love	Asher, John	John Asher	tt0327643
1746508088	1926	1926	The Man in the Saddle	The Man in the Saddle	Smith, Clifford S.	Lynn Reynolds	tt0017112
1746520680	1925	1925	The Red Rider	The Red Rider	Smith, Clifford S.	Clifford Smith	tt0016278
1746464856	1944	1944	Janie	Janie	Curtiz, Michael	Michael Curtiz	tt0036970
1746514514	1914	1914	The Spy	The Spy	Turner, Otis	Otis Turner	tt0004633
1745359965	2001	2001	Wet Hot American Summer	Wet Hot American Summer	Wain, David	David Wain	tt0243655
1746565493	1932	1932	Virtue	Virtue	Buzzell, Edward	Edward Buzzell	tt0023659
1746572026	1931	1931	Cracked Nuts	Cracked Nuts	Cline, Edward	Edward F. Cline	tt0021768
1746580580	1937	1937	It Can't Last Forever	It Can't Last Forever	MacFadden, Hamilton	Hamilton MacFadden	tt0029053
1746435278	1940	1940	Margie	Margie	Garrett, Otis; Smith, Paul Gerard	Otis Garrett	tt0032759
1746465092	1947	1947	Bulldog Drummond at Bay	Bulldog Drummond at Bay	Salkow, Sidney	Sidney Salkow	tt0039229
1746572835	1910	1910	Cupid and the Motor Boat	Cupid and the Motor Boat		J. Stuart Blackton	tt0298819
1746409819	1988	1988	High Spirits	High Spirits	Jordan, Neil	Neil Jordan	tt0095304
1746569798	1953	1953	Thy Neighbor's Wife	Thy Neighbor's Wife	Haas, Hugo	Hugo Haas	tt0046428
1746464844	1944	1944	The Hour Before the Dawn	The Hour Before the Dawn	Tuttle, Frank	Frank Tuttle	tt0036930
1746582422	1937	1937	Saturday's Heroes	Saturday's Heroes	Killy, Edward	Edward Killy	tt0029517
1746409714	1988	1987	Dudes	Dudes	Spheeris, Penelope	Penelope Spheeris	tt0092933
1746583750	1931	1931	Range Feud	The Range Feud	Lederman, D. Ross	D. Ross Lederman	tt0022293
1746577582	1933	1933	My Weakness	My Weakness	Butler, David	David Butler	tt0024364
1746582999	1937	1937	Girl Loves Boy	Girl Loves Boy	Mansfield, Duncan	Duncan Mansfield	tt0028930
1746464893	1942	1942	The Affairs of Martha	The Affairs of Martha	Dassin, Jules	Jules Dassin	tt0034433
1746584716	1930	1930	Behind the Make-Up	Behind the Make Up	Milton, Robert	Robert Milton	tt0020677
1746571289	1934	1934	Registered Nurse	Registered Nurse	Florey, Robert	Robert Florey	tt0025709

1746584387	1939	1939	The Fighting Gringo	The Fighting Gringo	Howard, David	David Howard	tt0031304	
1746584386	1939	1939	Five Little Peppers and How They Grew	Five Little Peppers and How They Grew	Barton, Charles	Charles Barton	tt0031315	
1746524659	1915	1915	The Master of the House	The Master of the House	Golden, Joseph A.	Joseph A. Golden	tt0005725	
1746500544	1949	1949	Tale of the Navajos	Tale of the Navajos			tt0041946	
1746577956	1938	1938	Extortion	Extortion	Hillyer, Lambert	Lambert Hillyer	tt0030114	
1746582492	1935	1935	Circle of Death	The Circle of Death	Glendon, J. Frank	J. Frank Glendon	tt0125701	
**1746455657**	**1995**	**1995**	**Wigstock: The Movie**	**Wigstock: The Movie**	**Shils, Barry**	**Barry Shils**	**tt0114937**	1
1746549869	1955	1955	Desert Sands	Desert Sands	Selander, Lesley	Lesley Selander	tt0047983	
1746492447	1946	1946	Abie's Irish Rose	Abie's Irish Rose	Sutherland, A. Edward	A. Edward Sutherland	tt0038283	
1746436567	1942	1942	Mr. & Mrs. North	Mr. and Mrs. North	Sinclair, Robert B.	Robert B. Sinclair	tt0033926	
1746198414	1968	1968	The Savage Seven	The Savage Seven	Rush, Richard	Richard Rush	tt0063553	
1746577189	1963	1963	California	California	Petroff, Hamil	Hamil Petroff	tt0056896	
1746562282	1956	1956	The Best Things in Life Are Free	The Best Things in Life Are Free	Curtiz, Michael	Michael Curtiz	tt0049002	
1746516300	1920	1921	Women Men Love	Women Men Love	Bradley, Samuel R.	Samuel R. Bradley	tt0012863	
1746473771	1944	1944	The Mask of Dimitrios	The Mask of Dimitrios	Negulesco, Jean	Jean Negulesco	tt0037055	
1746483545	1917	1917	Efficiency Edgar's Courtship	Efficiency Edgar's Courtship	Windom, L. C.	Lawrence C. Windom	tt0007882	
1746584190	1939	1939	Boy Friend	Boy Friend	Tinling, James	James Tinling	tt0031115	
1746512140	1917	1917	The Inevitable	The Inevitable	Goetz, Ben	Ben Goetz	tt0176851	
1746251507	1978	1978	Goin' South	Goin' South	Nicholson, Jack	Jack Nicholson	tt0077621	
1746520990	1949	1949	Red Rock Outlaw	Red Rock Outlaw	Pond, Elmer S.	Elmer Clifton	tt0180089	
1746583136	1940	1940	Grand Ole Opry	Grand Ole Opry	McDonald, Frank	Frank McDonald	tt0032546	
1746570263	1933	1933	Blondie Johnson	Blondie Johnson	Enright, Ray	Ray Enright	tt0023817	
1746573465	1932	1932	Westward Passage	Westward Passage	Milton, Robert	Robert Milton	tt0023684	
1746577816	1936	1936	Trail Dust	Trail Dust	Watt, Nate	Nate Watt	tt0028400	
1746573483	1932	1932	Law of the North	Law of the North	Fraser, Harry	Harry L. Fraser	tt0023122	
1746584126	1936	1936	The Law in Her Hands	The Law in Her Hands	Clemens, William	William Clemens	tt0027875	
1746475510	1943	1943	Two-Fisted Justice	Two Fisted Justice	Tansey, Robert	Robert Emmett Tansey	tt0036468	
1746574591	1933	1933	The Return of Casey Jones	The Return of Casey Jones	McCarthy, J. P.	John P. McCarthy	tt0025711	
1746464767	1942	1942	Seven Days' Leave	Seven Days' Leave	Whelan, Tim	Tim Whelan	tt0035307	
1746198648	1968	1968	Finian's Rainbow	Finian's Rainbow	Coppola, Francis Ford	Francis Ford Coppola	tt0062974	
1746575817	1932	1932	Almost Married	Almost Married	Menzies, William Cameron; Varnel, M	William Cameron Menzies	tt0022622	
1746509231	1926	1926	Secret Orders	Secret Orders	Withey, Chet	Chester Withey	tt0017361	
1746464875	1945	1945	South of the Rio Grande	South of the Rio Grande	Hillyer, Lambert	Lambert Hillyer	tt0038106	
1746561574	1954	1954	The Vanishing Prairie	The Vanishing Prairie	Algar, James	James Algar	tt0047642	
1746523045	1916	1916	A Million for Mary	A Million for Mary	Berger, Rea	Rae Berger	tt0007066	
1746465284	1947	1947	Robin Hood of Monterey	Robin Hood of Monterey	Cabanne, Christy	Christy Cabanne	tt0039779	
1745309425	2006	2006	Crank	Crank	Neveldine, Mark ; Taylor, Brian	Mark Neveldine	tt0479884	
1746516144	1921	1921	Penny of Top Hill Trail	Penny of Top Hill Trail	Berthelet, Arthur	Arthur Berthelet	tt0011566	
1746500578	1926	1926	A Man of Quality	A Man of Quality	Ruggles, Wesley	Wesley Ruggles	tt0017114	
1746502416	1916	1916	Sweet Kitty Bellairs	Sweet Kitty Bellairs	Young, James	James Young	tt0007421	
1746409474	1992	1992	One False Move	One False Move	Franklin, Carl	Carl Franklin	tt0102592	
1746235215	1972	1972	Dr. Phibes Rises Again	Dr. Phibes Rises Again	Fuest, Robert	Robert Fuest	tt0068503	
1746584296	1939	1939	Sundown on the Prairie	Sundown on the Prairie	Herman, Al	Albert Herman	tt0031993	
1746504192	1921	1921	Who Am I?	Who Am I?	Kolker, Henry	Henry Kolker	tt0012845	
1746508242	1925	1924	Secrets of the Night	Secrets of the Night	Blaché, Herbert	Herbert Blaché	tt0015313	
1746555299	1956	1956	Helen of Troy	Helen of Troy	Wise, Robert	Robert Wise	tt0049301	
1746234524	1967	1967	Banning	Banning	Winston, Ron	Ron Winston	tt0061383	
1746419779	1994	1994	Car 54, Where Are You?	Car 54, Where Are You?	Fishman, Bill	Bill Fishman	tt0109376	
1746578918	1950	1950	Streets of Ghost Town	Streets of Ghost Town	Nazarro, Ray	Ray Nazarro	tt0043007	
**1746583374**	**1930**	**1930**	**Young Desire**	**Young Desire**	**Collins, Lew**	**Lewis D. Collins**	**tt0021566**	1
1769975297	1917	1918	Busted Hearts and Buttermilk	Busted Hearts and Buttermilk		Al Christie	tt0344981	
1746499988	1948	1947	A Double Life	A Double Life	Cukor, George	George Cukor	tt0039335	
1746583293	1951	1951	Follow the Sun	Follow the Sun	Lanfield, Sidney	Sidney Lanfield	tt0043550	
1746499979	1924	1924	Man from God's Country	Man from God's Country	Neitz, Alvin J.	Alan James	tt0015113	
1746507730	1926	1926	Fightin' Jack	Fightin' Jack	Chaudet, Louis	Louis Chaudet	tt0016862	
1746453771	1944	1944	Cobra Woman	Cobra Woman	Siodmak, Robert	Robert Siodmak	tt0036716	
1745309153	2005	2005	Cheaper by the Dozen 2	Cheaper by the Dozen 2	Shankman, Adam	Adam Shankman	tt0452598	

1746584491	1931	1931	Three Who Loved	Three Who Loved	Archainbaud, George	George Archainbaud	tt0022481
1746234797	1967	1966	Death Curse of Tartu	Death Curse of Tartu	Grefé, William	William Grefé	tt0060290
1746533198	1915	1915	The White Sister	The White Sister	Wright, Fred E.	Fred E. Wright	tt0006281
1746571485	1932	1932	Rockabye	Rockabye	Cukor, George	George Cukor	tt0024506
1746497236	1918	1918	The Whispering Chorus	The Whispering Chorus	DeMille, Cecil B.	Cecil B. DeMille	tt0009796
1746570745	1933	1933	The Kiss Before the Mirror	The Kiss Before the Mirror	Whale, James	James Whale	tt0024222
1746500199	1925	1925	Sealed Lips	Sealed Lips	Gaudio, Antonio	Tony Gaudio	tt0016326
1746217659	1966	1966	The Cat	The Cat	Kadison, Ellis	Ellis Kadison	tt0060221
1746570414	1934	1934	Bachelor Bait	Bachelor Bait	Stevens, George	George Stevens	tt0024855
1746257210	1977	1977	Black Oak Conspiracy	Black Oak Conspiracy	Kelljan, Bob	Bob Kelljan	tt0075763
1746577748	1933	1933	Deadwood Pass	Deadwood Pass	McGowan, J. P.	J.P. McGowan	tt0023934
1769970449	1967	1967	The Playpen	The Playpen	St. Thomas, Warren	Warren St. Thomas	tt0148621
1746500306	1924	1924	The Storm Daughter	The Storm Daughter	Archainbaud, George	George Archainbaud	tt0015370
1746520353	1925	1925	The Mystic	The Mystic	Browning, Tod	Tod Browning	tt0016142
1746570718	1933	1933	The Solitaire Man	The Solitaire Man	Conway, Jack	Jack Conway	tt0024590
1746532419	1922	1922	The Fighting Ranger	The Fighting Ranger			tt0325374
1746422146	1994	1994	Sleep with Me	Sleep with Me	Kelly, Rory	Rory Kelly	tt0111218
1746435333	1998	1998	Chairman of the Board	Chairman of the Board	Zamm, Alex	Alex Zamm	tt0118836
1746585467	1939	1939	Irish Luck	Irish Luck	Bretherton, Howard	Howard Bretherton	tt0031495
1746583003	1940	1940	Women Without Names	Women Without Names	Florey, Robert	Robert Florey	tt0033272
1746474821	1945	1944	Of One Blood	Of One Blood	Williams, Spencer	Spencer Williams	tt0029330
1746576726	1932	1932	While Paris Sleeps	While Paris Sleeps	Dwan, Allan	Allan Dwan	tt0023691
1746524308	1923	1923	Little Church Around the Corner	Little Church Around the Corner	Seiter, William A.	William A. Seiter	tt0014202
1746572315	1932	1932	Bring 'Em Back Alive	Bring 'Em Back Alive	Elliott, Clyde E.	Clyde E. Elliott	tt0023843
1746257446	1978	1978	Renaldo & Clara	Renaldo and Clara	Dylan, Bob	Bob Dylan	tt0078151
1746199284	1969	1969	The Royal Hunt of the Sun	The Royal Hunt of the Sun	Lerner, Irving	Irving Lerner	tt0064907
1746497271	1920	1920	The City of Masks	The City of Masks	Heffron, Thomas N.	Thomas N. Heffron	tt0011060
1746550259	1955	1955	Violent Saturday	Violent Saturday	Fleischer, Richard	Richard Fleischer	tt0048790
1746491719	1946	1946	Rolling Home	Rolling Home	Berke, William	William Berke	tt0039782
1746573868	1958	1958	Man or Gun	Man or Gun	Gannaway, Albert C.	Albert C. Gannaway	tt0051901
1746578015	1937	1937	Bulldog Drummond Escapes	Bulldog Drummond Escapes	Hogan, James	James P. Hogan	tt0028668
1746434328	1999	1998	Relax... It's Just Sex	Relax... It's Just Sex	Castellaneta, P. J.	P.J. Castellaneta	tt0159696
1746577211	1950	1950	No Sad Songs for Me	No Sad Songs for Me	Maté, Rudolph	Rudolph Maté	tt0042790
1746567440	1932	1932	Cornered	Cornered	Eason, Reeves	B. Reeves Eason	tt0023908
1746520688	1925	1925	Rough Stuff	Rough Stuff	Henderson, Dell	Dell Henderson	tt0158903
1746549847	1954	1954	Woman's World	Woman's World	Negulesco, Jean	Jean Negulesco	tt0047680
1746492480	1948	1948	The Main Street Kid	The Main Street Kid	Springsteen, R. G.	R.G. Springsteen	tt0040563
1746475898	1945	1945	The Jungle Captive	The Jungle Captive	Young, Harold	Harold Young	tt0037837
1746550114	1955	1955	Man with the Gun	Man with the Gun	Wilson, Richard	Richard Wilson	tt0048339
1746553659	1964	1964	Bikini Beach	Bikini Beach	Asher, William	William Asher	tt0057887
1746504759	1920	1920	Jack Straw	Jack Straw	de Mille, William C.	William C. de Mille	tt0011343
1746574467	1952	1952	Lydia Bailey	Lydia Bailey	Negulesco, Jean	Jean Negulesco	tt0044861
1746219133	1971	1971	The Steagle	The Steagle	Sylbert, Paul	Paul Sylbert	tt0067792
1746438780	1998	1998	Hi-Life	Hi Life	Hedden, Roger	Roger Hedden	tt0132213
1745302681	2007	2007	The Savages	The Savages	Jenkins, Tamara	Tamara Jenkins	tt0775529
1746582530	1939	1939	The Bronze Buckaroo	The Bronze Buckaroo	Kahn, Richard C.	Richard C. Kahn	tt0031122
1746569847	1959	1959	The Man in the Net	The Man in the Net	Curtiz, Michael	Michael Curtiz	tt0053045
1746513383	1914	1914	The Man from Mexico	The Man from Mexico	Heffron, Thomas N.	Thomas N. Heffron	tt0004295
1746579036	1964	1963	The Comedy of Terrors	The Comedy of Terrors	Tourneur, Jacques	Jacques Tourneur	tt0056943
1746524750	1923	1923	Deceit	Deceit	Micheaux, Oscar	Oscar Micheaux	tt0013980
1746581954	1928	1928	The Smart Set	The Smart Set	Conway, Jack	Jack Conway	tt0019400
1746532640	1915	1915	Heritage	Heritage	Leonard, Robert	Robert Z. Leonard	tt0005480
1746484203	1917	1918	Whose Wife?	Whose Wife?	Sturgeon, Rollin S.	Al Christie	tt0436959
1746571114	1935	1935	Shadow of Doubt	Shadow of Doubt	Seitz, George B.	George B. Seitz	tt0026980
1746583328	1928	1928	Faithless Lover	Faithless Lover	Windom, Lawrence	Lawrence C. Windom	tt0018872
1746564328	1953	1953	Calamity Jane	Calamity Jane	Butler, David	David Butler	tt0045591
1746503573	1920	1919	Haunting Shadows	Haunting Shadows	King, Henry	Henry King	tt0010216

1746576321	1931	1931	Men Call It Love	Men Call It Love	Selwyn, Edgar	Edgar Selwyn	tt0022135
1746504338	1918	1918	Hitting the High Spots	Hitting the High Spots	Swickard, Charles	Charles Swickard	tt0184577
1746409460	1987	1987	A Nightmare on Elm Street 3: Dream Warriors	A Nightmare on Elm Street 3: Dream Warrio	Russell, Chuck	Chuck Russell	tt0093629
1746577126	1961	1961	The Last Time I Saw Archie	The Last Time I Saw Archie	Webb, Jack	Jack Webb	tt0055074
1746509053	1949	1949	Sand	Sand	King, Louis	Louis King	tt0041840
1746421348	1990	1990	Cry-Baby	Cry Baby	Waters, John	John Waters	tt0099329
1746584196	1936	1936	Shakedown	Shakedown	Selman, David	David Selman	tt0028244
1746392827	1982	1982	The Sword and the Sorcerer	The Sword and the Sorcerer	Pyun, Albert	Albert Pyun	tt0084749
1746570318	1934	1934	Gift of Gab	Gift of Gab	Freund, Karl	Karl Freund	tt0025170
1746578749	1951	1951	The Lady Pays Off	The Lady Pays Off	Sirk, Douglas	Douglas Sirk	tt0043720
1746394771	1991	1991	Oscar	Oscar	Landis, John	John Landis	tt0102603
1746569894	1958	1958	The Man Who Died Twice	The Man Who Died Twice	Kane, Joe	Joseph Kane	tt0051895
1746439087	1996	1996	Larger Than Life	Larger Than Life	Franklin, Howard	Howard Franklin	tt0116823
1746434372	1989	1989	Cousins	Cousins	Schumacher, Joel	Joel Schumacher	tt0097116
1746580735	1937	1937	She's Dangerous	She's Dangerous	Foster, Lewis R.; Carruth, Milton	Milton Carruth	tt0028246
1746218743	1976	1976	Death Machines	Death Machines	Kyriazi, Paul	Paul Kyriazi	tt0074383
1746574456	1935	1935	Old Man Rhythm	Old Man Rhythm	Ludwig, Edward	Edward Ludwig	tt0026811
1746453829	1943	1943	After Midnight with Boston Blackie	After Midnight with Boston Blackie	Landers, Lew	Lew Landers	tt0035615
1769973132	1924	1924	Her Boy Friend	Her Boy Friend	Smith, Noel Mason	Larry Semon	tt0248078
1746436253	1997	1997	Masterminds	Masterminds	Christian, Roger	Roger Christian	tt0119630
1746393392	1984	1984	Beat Street	Beat Street	Lathan, Stan	Stan Lathan	tt0086946
1746211502	1972	1972	Sleuth	Sleuth	Mankiewicz, Joseph L.	Joseph L. Mankiewicz	tt0069281
1746453773	1943	1943	Flight for Freedom	Flight for Freedom	Mendes, Lothar	Lothar Mendes	tt0035888
1746500541	1925	1925	Jimmie's Millions	Jimmie's Millions	Hogan, James P.	James P. Hogan	tt0135483
1746569805	1958	1958	The Quiet American	The Quiet American	Mankiewicz, Joseph L.	Joseph L. Mankiewicz	tt0052106
1745342280	2002	2001	Never Again	Never Again	Schaeffer, Eric	Eric Schaeffer	tt0244094
1746581694	1928	1928	The Showdown	The Showdown	Schertzinger, Victor	Victor Schertzinger	tt0019380
1746498297	1922	1922	A Pasteboard Crown	A Pasteboard Crown	Vale, Travers	Travers Vale	tt0013483
1746256783	1977	1977	The Kentucky Fried Movie	The Kentucky Fried Movie	Landis, John	John Landis	tt0076257
1746422262	1997	1997	Rocketman	RocketMan	Gillard, Stuart	Stuart Gillard	tt0120029
1746553881	1964	1964	Rio Conchos	Rio Conchos	Douglas, Gordon	Gordon Douglas	tt0058525
1769970843	1915	1915	Mixed and Fixed	Mixed and Fixed		Bobby Burns	tt0269558
1746208445	1969	1969	Scream, Baby, Scream	Scream Baby Scream	Adler, Joseph	Joseph Adler	tt0064950
1746454369	1941	1941	Confirm or Deny	Confirm or Deny	Mayo, Archie; Lang, Fritz	Archie Mayo	tt0033486
1746580768	1938	1938	Little Tough Guys in Society	Little Tough Guys in Society	Kenton, Erle C.	Erle C. Kenton	tt0030379
1746502118	1914	1914	Aftermath	The Aftermath		Sydney Ayres	tt0214456
1746504057	1921	1921	Puppets of Fate	Puppets of Fate	Fitzgerald, Dallas M.	Dallas M. Fitzgerald	tt0012597
1746572308	1932	1932	Sin's Pay Day	Sin's Pay Day	Seitz, George B.	George B. Seitz	tt0023475
1746422031	1994	1994	Forrest Gump	Forrest Gump	Zemeckis, Robert	Robert Zemeckis	tt0109830
1746393649	1986	1986	Peggy Sue Got Married	Peggy Sue Got Married	Coppola, Francis	Francis Ford Coppola	tt0091738
1746542633	1925	1925	The Hurricane Kid	The Hurricane Kid	Sedgwick, Edward	Edward Sedgwick	tt0015934
1746436381	1940	1940	A Fugitive from Justice	A Fugitive from Justice	Morse, Terry	Terry O. Morse	tt0032499
1746486808	1920	1920	The Perfect Woman	The Perfect Woman	Kirkland, David	David Kirkland	tt0011567
1746573855	1959	1959	The Rebel Set	The Rebel Set	Fowler, Gene	Gene Fowler Jr.	tt0053213
1746520517	1927	1927	A Sailor's Sweetheart	A Sailor's Sweetheart	Bacon, Lloyd	Lloyd Bacon	tt0018355
1745342070	2009	2009	The Men Who Stare at Goats	The Men Who Stare at Goats	Heslov, Grant	Grant Heslov	tt1234548
1746475529	1947	1947	Bandits of Dark Canyon	Bandits of Dark Canyon	Ford, Philip	Philip Ford	tt0039173
1746436652	1943	1943	Sherlock Holmes Faces Death	Sherlock Holmes Faces Death	Neill, Roy William	Roy William Neill	tt0036348
1746257161	1975	1976	Apple Pie	Apple Pie	Goldberg, Howard	Howard Goldberg	tt0074149
1746475278	1946	1946	Shock	Shock	Werker, Alfred	Alfred L. Werker	tt0038937
1746578056	1938	1938	Swing Sister Swing	Swing, Sister, Swing	Santley, Joseph	Joseph Santley	tt0030821
1746583686	1929	1929	Paris	Paris	Badger, Clarence	Clarence G. Badger	tt0020257
1746496073	1919	1919	For a Woman's Honor	For a Woman's Honor	Frame, Park	Park Frame	tt0010139
1746500413	1926	1926	The Fighting Failure	The Fighting Failure	Boyle, E. G.	Edward G. Boyle	tt0016861
1746582241	1929	1929	The Saturday Night Kid	The Saturday Night Kid	Sutherland, Edward	A. Edward Sutherland	tt0020364
1746453471	1943	1943	West of Texas	West of Texas	Drake, Oliver	Oliver Drake	tt0036522
1746520671	1950	1950	Fortunes of Captain Blood	Fortunes of Captain Blood	Douglas, Gordon	Gordon Douglas	tt0042474

1746515816	1921	1921	Dead or Alive	Dead or Alive	Henderson, Dell	Dell Henderson	tt0264517
1746198055	1969	1969	Tell Them Willie Boy Is Here	Tell Them Willie Boy Is Here	Polonsky, Abraham	Abraham Polonsky	tt0065079
1746486492	1918	1918	Men Who Have Made Love to Me	Men Who Have Made Love to Me	Berthelet, Arthur	Arthur Berthelet	tt0186344
1769973336	1970	1970	Substitution	Substitution	Davis, Walt	Walt Davis	tt0149207
1746501393	1916	1916	The Eagle's Wing	The Eagle's Wings	Steele, Rufus	Robert Z. Leonard	tt0006620
1746483434	1917	1917	Bucking Broadway	Bucking Broadway	Ford, Jack	John Ford	tt0007755
1745359877	2008	2008	Beautiful Losers	Beautiful Losers	Rose, Aaron	Aaron Rose	tt0430916
1746512408	1922	1922	The Desert's Crucible	The Desert's Crucible	Clements, Roy	Roy Clements	tt0013061
1746554657	1964	1964	Voice of the Hurricane	Voice of the Hurricane	Fraser, George	George Fraser	tt0180291
1746210031	1970	1970	Rio Lobo	Rio Lobo	Hawks, Howard	Howard Hawks	tt0066301
1746515585	1921	1921	Chickens	Chickens	Nelson, Jack	Jack Nelson	tt0012044
1746500858	1916	1916	The Faded Flower	The Faded Flower	Abramson, Ivan	Ivan Abramson	tt0157609
1746198536	1966	1966	The Embracers	The Embracers	Graver, Gary	Gary Graver	tt0178403
1746500448	1925	1925	The Golden Princess	The Golden Princess	Badger, Clarence	Clarence G. Badger	tt0015869
1746453550	1942	1942	King of the Stallions	King of the Stallions	Finney, Edward	Edward Finney	tt0034945
1746582380	1928	1928	Golf Widows	Golf Widows	Kenton, Erle C.	Erle C. Kenton	tt0018949
1746423819	1940	1940	Oklahoma Renegades	Oklahoma Renegades	Watt, Nate	Nate Watt	tt0032859
1746455783	1996	1996	Sgt. Bilko	Sgt. Bilko	Lynn, Jonathan	Jonathan Lynn	tt0117608
1769970780	1912	1912	The Dumb Wooing	The Dumb Wooing		Will Louis	tt0002150
1746577999	1937	1937	Tough to Handle	Tough to Handle	Luby, Roy	S. Roy Luby	tt0165992
1746464999	1947	1947	Hard Boiled Mahoney	Hard Boiled Mahoney	Beaudine, William	William Beaudine	tt0039447
1746555276	1955	1955	The Rains of Ranchipur	The Rains of Ranchipur	Negulesco, Jean	Jean Negulesco	tt0048538
1746523276	1915	1915	The Sable Lorcha	The Sable Lorcha	Ingraham, Lloyd	Lloyd Ingraham	tt0005997
1746422312	1993	1993	Bodies, Rest & Motion	Bodies, Rest & Motion	Steinberg, Michael	Michael Steinberg	tt0106447
1746453504	1944	1944	Nevada	Nevada	Killy, Edward	Edward Killy	tt0037124
1746530754	1949	1948	3 Godfathers	3 Godfathers	Ford, John	John Ford	tt0040064
1746508693	1949	1949	Mississippi Rhythm	Mississippi Rhythm	Abrahams, Derwin	Derwin Abrahams	tt0041656
1746573882	1960	1961	Runaway	The Runaway	Kone, Elliot H.	Claudio Guzmán	tt0201048
1746584849	1929	1929	Illusion of Love	Illusion of Love			tt0361737
1746520212	1924	1924	Reckless Riding Bill	Reckless Riding Bill	Morrow, Frank	Frank Morrow	tt0015264
1746583886	1930	1930	Love in the Rough	Love in the Rough	Reisner, Charles F.	Charles Reisner	tt0021100
1746464876	1942	1942	Riders of the Northland	Riders of the Northland	Berke, William	William Berke	tt0035253
1746585208	1928	1928	Fandango	Fandango	George, Henry W.	Lupino Lane	tt0256021
1746575039	1934	1934	The Great Flirtation	The Great Flirtation	Murphy, Ralph	Ralph Murphy	tt0025203
1746219397	1975	1975	Crazy Mama	Crazy Mama	Demme, Jonathan	Jonathan Demme	tt0072829
1746455537	1999	1999	Trippin'	Trippin'	Raynr, David	David Raynr	tt0160298
1746503875	1918	1918	The Panther Woman	The Panther Woman	Ince, Ralph	Ralph Ince	tt0009477
1746465086	1944	1944	Faces in the Fog	Faces in the Fog	English, John	John English	tt0036808
1746211243	1971	1971	Private Duty Nurses	Private Duty Nurses	Armitage, George	George Armitage	tt0067618
1746184904	1965	1965	The Satan Bug	The Satan Bug	Sturges, John	John Sturges	tt0059678
1746572046	1932	1932	Guilty As Hell	Guilty as Hell	Kenton, Erle C.	Erle C. Kenton	tt0022967
1746492755	1948	1948	Let's Live a Little	Let's Live a Little	Wallace, Richard	Richard Wallace	tt0040535
1746514720	1913	1913	Buried Alive in a Coal Mine	Buried Alive in a Coal Mine		Albert W. Hale	tt0002725
1746571639	1910	1910	The Cowboy's Mother-in-Law	A Cowboy's Mother in Law		Gilbert M. 'Broncho Billy' Anderson	tt0001168
1746198432	1969	1969	The Candy Man	The Candy Man		Herbert J. Leder	tt0064125
1746208545	1966	1965	The Spy With My Face	The Spy with My Face	Newland, John	John Newland	tt0058610
1746520523	1924	1924	Hot Water	Hot Water	Taylor, Sam; Newmeyer, Fred	Fred C. Newmeyer	tt0015002
1746577637	1935	1935	Six Gun Justice	Six Gun Justice		Robert F. Hill	tt0027006
1746542652	1924	1924	Inez From Hollywood	Inez from Hollywood	Green, Alfred E.	Alfred E. Green	tt0015013
1746583220	1951	1951	Overland Telegraph	Overland Telegraph	Selander, Lesley	Lesley Selander	tt0043891
1746576728	1932	1932	Divorce in the Family	Divorce in the Family	Riesner, Charles F.	Charles Reisner	tt0022824
1746584067	1937	1937	That Man's Here Again	That Man's Here Again	King, Louis	Louis King	tt0029652
1746497060	1919	1919	The Lady of Red Butte	The Lady of Red Butte	Schertzinger, Victor L.	Victor Schertzinger	tt0190518
1746487866	1919	1919	The Undercurrent	The Undercurrent	North, Wilfrid	Wilfrid North	tt0010819
1746570323	1934	1934	Shoot the Works	Shoot the Works	Ruggles, Wesley	Wesley Ruggles	tt0025784
1746474987	1945	1945	Over 21	Over 21	Vidor, Charles	Charles Vidor	tt0037966
1746576867	1931	1931	Children of Dreams	Children of Dreams	Crosland, Alan	Alan Crosland	tt0021740

1746571024	1910	1910	The Education of Elizabeth	The Education of Elizabeth		Sidney Olcott	tt0397408
1746524654	1914	1914	Neptune's Daughter	Neptune's Daughter	Brenon, Herbert	Herbert Brenon	tt0004391
1745342498	2004	2004	Welcome to Mooseport	Welcome to Mooseport	Petrie, Donald	Donald Petrie	tt0361925
1746580919	1962	1962	Days of Wine and Roses	Days of Wine and Roses	Edwards, Blake	Blake Edwards	tt0055895
1746484081	1918	1918	The Sunset Princess	The Sunset Princess	Hitchcock, Charles Webster; Cooper	William S. Cooper	tt0152660
1746502397	1916	1916	The Strength of Donald Mckenzie	The Strength of Donald McKenzie	Russell, William; Prescott, Jack	Jack Prescott	tt0158997
1746508684	1926	1926	Sweet Rosie O'Grady	Sweet Rosie O'Grady	Strayer, Frank R.	Frank R. Strayer	tt0017445
1746577517	1933	1933	Sitting Pretty	Sitting Pretty	Brown, Harry Joe	Harry Joe Brown	tt0024570
1746582070	1951	1951	South of Caliente	South of Caliente	Witney, William	William Witney	tt0044063
1746574453	1935	1935	Charlie Chan in Egypt	Charlie Chan in Egypt	King, Louis	Louis King	tt0026197
1746577574	1936	1936	Earthworm Tractors	Earthworm Tractors	Enright, Raymond	Ray Enright	tt0027562
1746408692	1990	1990	Ghost Dad	Ghost Dad	Poitier, Sidney	Sidney Poitier	tt0099654
1746500402	1925	1925	The Last Edition	The Last Edition	Johnson, Emory	Emory Johnson	tt0016008
1746569961	1958	1958	The Deep Six	The Deep Six	Maté, R.	Rudolph Maté	tt0050298
1746524520	1916	1916	Wanted -A Home	Wanted: A Home	Smalley, Phillips	Phillips Smalley	tt0007535
1746453714	1995	1995	Bad Boys	Bad Boys	Bay, Michael	Michael Bay	tt0112442
1746582673	1936	1936	The Crooked Trail	The Crooked Trail	Luby, S. Roy	S. Roy Luby	tt0132909
1746583240	1929	1929	Back From Shanghai	Back from Shanghai	Mason, Noel	Noel M. Smith	tt0019669
1746502450	1915	1915	The Wolf Man	The Wolf Man	Powell, Paul	Paul Powell	tt0006299
1746454077	1940	1940	Santa Fe Trail	Santa Fe Trail	Curtiz, Michael	Michael Curtiz	tt0033021
1746580828	1936	1936	Lawless Land	Lawless Land	Ray, Albert	Albert Ray	tt0029125
1746419382	1982	1982	Shoot the Moon	Shoot the Moon	Parker, Alan	Alan Parker	tt0084675
1769974396	1916	1916	Guardian Angels	Guardian Angels	Beaudine, William	William Beaudine	tt0460218
1746577870	1936	1936	The Luckiest Girl in the World	The Luckiest Girl in the World	Buzzell, Eddie	Edward Buzzell	tt0027915
1746508547	1924	1924	Daring Youth	Daring Youth	Beaudine, William	William Beaudine	tt0014829
1746454611	1941	1941	Murder on Lenox Avenue	Murder on Lenox Avenue	Dreifuss, Arthur	Arthur Dreifuss	tt0033929
1746508535	1924	1924	Fires Of Youth	Fires of Youth			tt0325383
1746210596	1970	1970	The Landlord	The Landlord	Ashby, Hal	Hal Ashby	tt0065963
1746577892	1939	1939	Gentleman from Arizona	The Gentleman from Arizona	Haley, Earl	Earl Haley	tt0031363
1746515593	1920	1920	Everybody's Sweetheart	Everybody's Sweetheart	Trimble, Laurence	Alan Crosland	tt0143240
1746500267	1949	1949	Jiggs and Maggie in Jackpot Jitters	Jiggs and Maggie in Jackpot Jitters	Beaudine, William	William Beaudine	tt0041516
1769973127	1924	1924	Fast and Furious	Fast and Furious	Taurog, Norman	Norman Taurog	tt0247334
1746574908	1936	1936	Cattle Thief	The Cattle Thief	Bennet, Spencer Gordon	Spencer Gordon Bennet	tt0027433
1746516497	1920	1920	Judy of Rogue's Harbor	Judy of Rogue's Harbor	Taylor, William D.	William Desmond Taylor	tt0011356
1746422274	1999	1999	Blast from the Past	Blast from the Past	Wilson, Hugh	Hugh Wilson	tt0124298
1746438940	1943	1943	The Sky's the Limit	The Sky's the Limit	Griffith, Edward H.	Edward H. Griffith	tt0036363
1746507742	1926	1926	Easy Going	Easy Going	Thorpe, Richard	Richard Thorpe	tt0016818
1746218721	1974	1974	It's Alive	It's Alive	Cohen, Larry	Larry Cohen	tt0071675
1746580460	1935	1935	Cheyenne Tornado	The Cheyenne Tornado	O'Connor, William	William A. O'Connor	tt0132900
1746502910	1914	1914	Joseph in the Land of Egypt	Joseph in the Land of Egypt	Moore, Eugene	Eugene Moore	tt0004179
1746584932	1939	1939	I Was a Convict	I Was a Convict	Scotto, Aubrey	Aubrey Scotto	tt0031467
1746578922	1958	1958	Stage Struck	Stage Struck	Lumet, Sidney	Sidney Lumet	tt0052235
1746581581	1961	1961	Flight of the Lost Balloon	Flight of the Lost Balloon		Nathan Juran	tt0054883
1746576928	1951	1950	Cyrano de Bergerac	Cyrano de Bergerac	Gordon, Michael	Michael Gordon	tt0042367
1745359573	2003	2003	Lara Croft Tomb Raider: The Cradle of Life	Lara Croft Tomb Raider: The Cradle of Life	de Bont, Jan	Jan de Bont	tt0325703
1746250582	1975	1975	One Flew Over the Cuckoo's Nest	One Flew Over the Cuckoo's Nest	Forman, Milos	Milos Forman	tt0073486
1746509309	1925	1925	The White Desert	The White Desert	Barker, Reginald	Reginald Barker	tt0016523
1746570869	1933	1933	The Working Man	The Working Man	Adolfi, John G.	John G. Adolfi	tt0024785
1746569612	1957	1956	Nightfall	Nightfall	Tourneur, Jacques	Jacques Tourneur	tt0049552
1746513406	1923	1923	Love's Old Sweet Song	Love's Old Sweet Song	Lund, Oscar	J. Searle Dawley	tt0014222
1746577843	1936	1936	Don't Turn 'Em Loose	Don't Turn 'em Loose	Stoloff, Ben	Benjamin Stoloff	tt0027537
1746582200	1950	1950	Stars in My Crown	Stars in My Crown	Tourneur, Jacques	Jacques Tourneur	tt0042998
1746438090	1997	1997	Austin Powers: International Man of Mystery	Austin Powers: International Man of Myster	Roach, Jay	Jay Roach	tt0118655
1745302599	2006	2005	Looking for Comedy in the Muslim World	Looking for Comedy in the Muslim World	Brooks, Albert	Albert Brooks	tt0433116
1745341017	2009	2009	District 9	District 9	Blomkamp, Neill	Neill Blomkamp	tt1136608
1746491877	1946	1946	Dressed to Kill	Dressed to Kill	Neill, Roy William	Roy William Neill	tt0038494
1746562005	1953	1953	Martin Luther	Martin Luther	Pichel, Irving	Irving Pichel	tt0046051

1746507815	1926	1926	Fascinating Youth	Fascinating Youth	Wood, Sam	Sam Wood	tt0016844
1746439125	1941	1941	Arizona Cyclone	Arizona Cyclone	Lewis, Joseph H.; Taylor, Ray	Joseph H. Lewis	tt0033352
1746525070	1920	1920	The Mysteries of Paris	The Mysteries of Paris	Cornell, Ed	Ed Cornell	tt0196774
1746585207	1930	1930	Mamba	Mamba	Rogell, Albert	Albert S. Rogell	tt0021109
1745358901	2008	2008	Flash of Genius	Flash of Genius	Abraham, Marc	Marc Abraham	tt1054588
1746497281	1918	1918	Uncle Tom's Cabin	Uncle Tom's Cabin	Dawley, J. Searle	J. Searle Dawley	tt0009741
1746582470	1935	1935	Bulldog Courage	Bulldog Courage	Newfield, Sam	Sam Newfield	tt0026157
1746465067	1942	1942	The Adventures of Martin Eden	The Adventures of Martin Eden	Salkow, Sidney	Sidney Salkow	tt0034431
1746577761	1933	1933	Gambling Ship	Gambling Ship	Gasnier, Louis; Marcin, Max	Louis J. Gasnier	tt0024048
1746569524	1958	1958	Party Girl	Party Girl	Ray, Nicholas	Nicholas Ray	tt0052050
1746583068	1940	1940	Colorado	Colorado	Kane, Joseph	Joseph Kane	tt0032346
1746197980	1969	1969	Butch Cassidy and the Sundance Kid	Butch Cassidy and the Sundance Kid	Hill, George Roy	George Roy Hill	tt0064115
1746473659	1944	1944	Dark Mountain	Dark Mountain	Berke, William	William Berke	tt0036743
1746198660	1966	1966	Run, Appaloosa, Run	Run, Appaloosa, Run	Lansburgh, Larry	Larry Lansburgh	tt0249129
1746509457	1927	1927	Matinee Ladies	Matinee Ladies	Haskin, Byron	Byron Haskin	tt0018148
1746410727	1986	1986	Gung Ho	Gung Ho	Howard, Ron	Ron Howard	tt0091159
1746259897	1978	1978	Malibu Beach	Malibu Beach	Rosenthal, Robert J.	Robert J. Rosenthal	tt0077895
1746475249	1947	1947	The Vigilantes Return	The Vigilantes Return	Taylor, Ray	Ray Taylor	tt0039958
1746563369	1951	1951	Adventures of Captain Fabian	Adventures of Captain Fabian	Marshall, William; Florey, Robert	William Marshall	tt0043264
1746502553	1915	1915	Kilmeny	Kilmeny	Apfel, Oscar	Oscar Apfel	tt0005588
1746577905	1935	1935	Your Uncle Dudley	Your Uncle Dudley	Forde, Eugene	Eugene Forde	tt0027236
1746419933	1994	1994	The War	The War	Avnet, Jon	Jon Avnet	tt0111667
1746574295	1960	1960	A Touch of Larceny	A Touch of Larceny	Hamilton, Guy	Guy Hamilton	tt0053371
1746585179	1929	1929	Charming Sinners	Charming Sinners	Milton, Robert	Robert Milton	tt0019758
1746571109	1933	1933	The Big Brain	The Big Brain	Archainbaud, George	George Archainbaud	tt0023806
1745373784	2009	2009	The Pink Panther 2	The Pink Panther 2	Zwart, Harald	Harald Zwart	tt0838232
1746184062	1910	1910	The Big Scoop	The Big Scoop	McGlynn, Frank	Frank McGlynn Sr.	tt1520398
1746513823	1915	1915	Mother's Roses	Mother's Roses	Marston, Theodore	Theodore Marston	tt0005776
1746530802	1950	1950	Belle of Old Mexico	Belle of Old Mexico	Springsteen, R. G.	R.G. Springsteen	tt0042237
1746582293	1930	1930	Second Honeymoon	Second Honeymoon	Rosen, Phil	Phil Rosen	tt0021349
1746577845	1938	1938	Her Jungle Love	Her Jungle Love	Archainbaud, George	George Archainbaud	tt0030229
1745302382	2005	2005	The Cave	The Cave	Hunt, Bruce	Bruce Hunt	tt0402901
1746585474	1939	1939	Pack Up Your Troubles	Pack Up Your Troubles	Humberstone, H. Bruce	H. Bruce Humberstone	tt0031775
1746571218	1910	1910	The Face at the Window	The Face at the Window	Griffith, D. W.	D.W. Griffith	tt0001206
1745340107	2004	2004	Catch That Kid	Catch That Kid	Freundlich, Bart	Bart Freundlich	tt0337917
1746492233	1948	1948	Isn't It Romantic	Isn't It Romantic?	McLeod, Norman Z.	Norman Z. McLeod	tt0040479
1746561834	1956	1956	Great Day in the Morning	Great Day in the Morning	Tourneur, Jacques	Jacques Tourneur	tt0049278
1746573762	1961	1961	Atlas	Atlas	Corman, Roger	Roger Corman	tt0053610
1746420128	1993	1993	Hot Shots! Part Deux	Hot Shots! Part Deux	Abrahams, Jim	Jim Abrahams	tt0107144
1746483488	1917	1917	The Hero of the Hour	The Hero of the Hour	Wells, Raymond	Raymond Wells	tt0008088
1746562452	1953	1953	Never Let Me Go	Never Let Me Go	Daves, Delmer	Delmer Daves	tt0046124
1769970104	1912	1912	A Doctor For an Hour	A Doctor for an Hour	Merwin, Bannister	C.J. Williams	tt0441223
1746581992	1928	1928	So This Is Love	So This Is Love	Capra, Frank	Frank Capra	tt0019401
1746465344	1946	1946	Gunman's Code	Gunman's Code	Fox, Wallace W.	Wallace Fox	tt0038580
1746454943	1941	1941	Citadel of Crime	Citadel of Crime	Sherman, George	George Sherman	tt0033992
1746583097	1935	1935	Big Calibre	Big Calibre	Bradbury, R. N.	Robert N. Bradbury	tt0026114
1746393631	1986	1986	Native Son	Native Son	Freedman, Jerrold	Jerrold Freedman	tt0091613
1746574044	1961	1961	West Side Story	West Side Story	Wise, Robert; Robbins, Jerome	Jerome Robbins	tt0055614
1746500873	1910	1910	Red Fern and the Kid	Red Fern and the Kid		Fred J. Balshofer	tt1392217
1746514966	1915	1915	The Governor's Boss	The Governor's Boss		Charles E. Davenport	tt0005406
1746573974	1963	1963	The Slime People	The Slime People	Hutton, Robert	Robert Hutton	tt0056499
1746554665	1963	1963	Summer Magic	Summer Magic	Neilson, James	James Neilson	tt0057542
1746562451	1953	1953	Escape from Fort Bravo	Escape from Fort Bravo	Sturges, John	John Sturges	tt0045737
1746454686	1946	1946	Under Arizona Skies	Under Arizona Skies	Hillyer, Lambert	Lambert Hillyer	tt0039063
1746580591	1939	1939	My Wife's Relatives	My Wife's Relatives	Meins, Gus	Gus Meins	tt0031700
1746435173	1941	1941	Honky Tonk	Honky Tonk	Conway, Jack	Jack Conway	tt0033726
1746503625	1919	1919	Victory	Victory	Tourneur, Maurice	Maurice Tourneur	tt0010844

1746585214	1930	1930	The Swellhead	The Swellhead	Flood, James	James Flood	tt0021444
1746569589	1958	1958	Buchanan Rides Alone	Buchanan Rides Alone	Boetticher, Budd	Budd Boetticher	tt0051437
1746508411	1949	1949	The Devil's Sleep	The Devil's Sleep	Connell, W. Merle	W. Merle Connell	tt0043466
1746524604	1914	1914	Richelieu	Richelieu	Dwan, Allan	Allan Dwan	tt0004532
1746569560	1957	1957	The Helen Morgan Story	The Helen Morgan Story	Curtiz, Michael	Michael Curtiz	tt0050494
1746577333	1960	1960	The Threat	The Threat	Rondeau, Charles R.	Charles R. Rondeau	tt0054383
1746466669	1917	1917	Mayblossom	Mayblossom	José, Edward	Edward José	tt0008282
1746509037	1925	1925	Cheaper To Marry	Cheaper to Marry	Leonard, Robert Z.	Robert Z. Leonard	tt0015678
1746513066	1916	1916	One Day	One Day	Clarendon, Hal	Hal Clarendon	tt0007146
1746582039	1928	1928	Thundergod	Thundergod	Hunt, Charles J.	Charles J. Hunt	tt0019475
1746438316	1941	1941	The Face Behind the Mask	The Face Behind the Mask	Florey, Robert	Robert Florey	tt0033582
1769974392	1916	1916	Houses of Mystery	The House of Mystery			tt0479422
1746566332	1955	1955	Dementia	Dementia	Parker, John	John Parker	tt0047976
1746420555	1991	1991	Problem Child 2	Problem Child 2	Levant, Brian	Brian Levant	tt0102719
1746520146	1949	1949	Act of Violence	Act of Violence	Zinnemann, Fred	Fred Zinnemann	tt0041088
1769976173	1928	1928	Deaf, Dumb and Blonde	Deaf, Dumb and Blonde		Leslie Goodwins	tt1660525
1746568335	1933	1933	He Couldn't Take It	He Couldn't Take It	Nigh, William	William Nigh	tt0024094
1746530763	1950	1950	The Lost Volcano	The Lost Volcano	Beebe, Ford	Ford Beebe	tt0042686
1746503266	1919	1919	Under Suspicion	Under Suspicion	Dowlan, William C.	William C. Dowlan	tt0010817
1746574649	1935	1935	Big Boy Rides Again	Big Boy Rides Again	Herman, Albert	Albert Herman	tt0026112
1746464784	1946	1946	Riverboat Rhythm	Riverboat Rhythm	Goodwins, Leslie	Leslie Goodwins	tt0038886
1746439077	1998	1997	Star Kid	Star Kid	Coto, Manny	Manny Coto	tt0120478
1746561059	1932	1932	Tom Brown of Culver	Tom Brown of Culver	Wyler, William	William Wyler	tt0023601
1745272405	2000	2000	Unbreakable	Unbreakable	Shyamalan, M. Night	M. Night Shyamalan	tt0217869
1746235272	1972	1972	Shaft's Big Score!	Shaft's Big Score!	Parks, Gordon	Gordon Parks	tt0069257
1746584284	1935	1935	The New Adventures of Tarzan	The New Adventures of Tarzan	Kull, Edward; McGaugh, Wilbur	Edward A. Kull	tt0026773
1746257378	1979	1979	Time After Time	Time After Time	Meyer, Nicholas	Nicholas Meyer	tt0080025
1746483844	1917	1917	Little Miss Optimist	Little Miss Optimist	Thornby, Robert	Robert Thornby	tt0008192
1746582611	1938	1938	Nurse from Brooklyn	The Nurse from Brooklyn	Simon, S. Sylvan	S. Sylvan Simon	tt0030515
1746502667	1915	1915	The White Terror	The White Terror	Paton, Stuart	Stuart Paton	tt0006282
1746439151	1942	1942	Romance on the Range	Romance on the Range	Kane, Joseph	Joseph Kane	tt0035266
1746584203	1940	1940	Too Many Girls	Too Many Girls	Abbott, George	George Abbott	tt0033173
1746475508	1948	1948	Scudda Hoo! Scudda Hay!	Scudda Hoo! Scudda Hay!	Herbert, F. Hugh	F. Hugh Herbert	tt0040762
1746232945	1969	1969	Hell's Angels '69	Hell's Angels '69	Madden, Lee	Lee Madden	tt0064415
1745341735	2003	2003	Down with Love	Down with Love	Reed, Peyton	Peyton Reed	tt0309530
1745341229	2001	2001	Pearl Harbor	Pearl Harbor	Bay, Michael	Michael Bay	tt0213149
1746581331	1959	1959	Middle of the Night	Middle of the Night	Mann, Delbert	Delbert Mann	tt0053065
1746566281	1958	1958	Outcasts of the City	Outcasts of the City	Petroff, Boris L.	Boris Petroff	tt0052039
1746491754	1944	1944	The Soul of a Monster	The Soul of a Monster	Jason, Will	Will Jason	tt0037301
1746525193	1921	1921	Scrap Iron	Scrap Iron	Ray, Charles	Charles Ray	tt0012656
1746410872	1986	1985	Screamplay	Screamplay	Seder, Rufus Butler	Rufus Butler Seder	tt0088067
1746542673	1924	1924	The Back Trail	The Back Trail	Smith, Clifford	George Marshall	tt0014692
1745293675	2005	2005	The Deal	The Deal	Kahn, Harvey	Harvey Kahn	tt0405861
1746525059	1921	1921	Keeping Up With Lizzie	Keeping Up with Lizzie	Ingraham, Lloyd	Lloyd Ingraham	tt0012345
1746584731	1930	1930	The Parting of the Trails	The Parting of the Trails	McGowan, J. P.	J.P. McGowan	tt0021238
1746576707	1931	1931	Sob Sister	Sob Sister	Santell, Alfred	Alfred Santell	tt0022407
1746585477	1939	1939	Big Town Czar	Big Town Czar	Lubin, Arthur	Arthur Lubin	tt0031098
1746453862	2000	2000	Finding Forrester	Finding Forrester	Van Sant, Gus	Gus Van Sant	tt0181536
1746563906	1952	1952	Retreat, Hell!	Retreat, Hell!	Lewis, Joseph H.	Joseph H. Lewis	tt0045082
1745374560	2009	2009	Everybody's Fine	Everybody's Fine	Jones, Kirk	Kirk Jones	tt0780511
1746198007	1969	1969	Charro!	Charro!	Warren, Charles Marquis	Charles Marquis Warren	tt0064155
1746574283	1962	1962	Zotz!	Zotz!	Castle, William	William Castle	tt0056725
1746455284	1998	1998	Sphere	Sphere	Levinson, Barry	Barry Levinson	tt0120184
1746420385	1981	1981	Fort Apache, The Bronx	Fort Apache the Bronx	Petrie, Daniel	Daniel Petrie	tt0082402
1746524928	1914	1914	When Broadway Was a Trail	When Broadway Was a Trail	Lund, O. A. C.	O.A.C. Lund	tt0004794
1746235231	1972	1972	The Life and Times of Judge Roy Bean	The Life and Times of Judge Roy Bean	Huston, John	John Huston	tt0068853
1746508499	1950	1950	Destination Moon	Destination Moon	Pichel, Irving	Irving Pichel	tt0042393

1769970719	1912	1912	East Lynne	East Lynne	Nichols, George O.	Theodore Marston	tt0338947
1746569909	1960	1960	Natchez Trace	Natchez Trace	Crosland, Alan	Alan Crosland Jr.	tt0054112
1746198514	1967	1967	Bonnie and Clyde	Bonnie and Clyde	Penn, Arthur	Arthur Penn	tt0061418
1746198152	1968	1968	Robby	Robby	Bluemke, Ralph C.	Ralph C. Bluemke	tt0063513
1746582746	1938	1938	Test Pilot	Test Pilot	Fleming, Victor	Victor Fleming	tt0030848
1746497903	1920	1920	The Desert Scorpion	The Desert Scorpion	Thayer, Otis B.	Otis Thayer	tt0191079
1746583873	1931	1931	The Deceiver	The Deceiver	King, Louis	Louis King	tt0021507
1746524929	1915	1915	The Rug Maker's Daughter	The Rug Maker's Daughter	Apfel, Oscar	Oscar Apfel	tt0005984
1746475974	1948	1948	Speed to Spare	Speed to Spare	Berke, William	William Berke	tt0040827
1746503555	1921	1922	The Fast Freight	The Fast Freight	Cruze, James	James Cruze	tt0012157
1746421190	1992	1991	Rock Soup	Rock Soup	Kowalski, Lech	Lech Kowalski	tt0105269
1746420570	1940	1940	The Durango Kid	The Durango Kid	Hillyer, Lambert	Lambert Hillyer	tt0032423
1746582548	1938	1938	International Crime	International Crime	Lamont, Charles	Charles Lamont	tt0030273
1746465209	1945	1945	I'll Remember April	I'll Remember April	Young, Harold	Harold Young	tt0037807
1746454385	1943	1943	Northern Pursuit	Northern Pursuit	Walsh, Raoul	Raoul Walsh	tt0036218
1746491740	1948	1948	French Leave	French Leave	McDonald, Frank	Frank McDonald	tt0040372
1746502733	1914	1914	The Thief	The Thief	Lewis, Edgar	Edgar Lewis	tt0004688
1746575057	1933	1934	The Big Race	The Big Race	Newmeyer, Fred	Fred C. Newmeyer	tt0023810
1746585404	1939	1939	Mutiny in the Big House	Mutiny in the Big House	Nigh, William	William Nigh	tt0031690
1746574753	1936	1936	Dangerous Intrigue	Dangerous Intrigue	Selman, David	David Selman	tt0027497
1746580608	1938	1938	My Old Kentucky Home	My Old Kentucky Home	Hillyer, Lambert	Lambert Hillyer	tt0030482
1746260907	1979	1979	Bush Mama	Bush Mama	Gerima, Haile	Haile Gerima	tt0074259
1746508116	1949	1949	Fighting Fools	Fighting Fools	Le Borg, Reginald	Reginald Le Borg	tt0041360
1745340055	2001	2001	Ocean's Eleven	Ocean's Eleven	Soderbergh, Steven	Steven Soderbergh	tt0240772
1746409437	1989	1989	Friday the 13th Part VIII -- Jason Takes Manhatta	Friday the 13th Part VIII: Jason Takes Manha	Hedden, Rob	Rob Hedden	tt0097388
1746582402	1937	1937	Breezing Home	Breezing Home	Carruth, Milton	Milton Carruth	tt0028660
1746436266	1991	1991	He Said, She Said	He Said, She Said	Kwapis, Ken; Silver, Marisa	Ken Kwapis	tt0102011
1746454142	1997	1997	Hijacking Hollywood	Hijacking Hollywood	Mandt, Neil	Neil Mandt	tt0119292
1746565880	1953	1953	Plunder of the Sun	Plunder of the Sun	Farrow, John	John Farrow	tt0046196
1746508484	1925	1925	Spook Ranch	Spook Ranch	Laemmle, Edward	Edward Laemmle	tt0016387
1746576866	1931	1931	Daddy Long Legs	Daddy Long Legs	Santell, Alfred	Alfred Santell	tt0021775
1746573466	1910	1910	The Duke's Plan	The Duke's Plan	Griffith, D. W.	D.W. Griffith	tt0001187
1746508944	1924	1924	Those Who Judge	Those Who Judge	King, Burton	Burton L. King	tt0015405
1746577002	1963	1963	Ladybug Ladybug	Ladybug Ladybug	Perry, Frank	Frank Perry	tt0057242
1746394714	1983	1983	Risky Business	Risky Business	Brickman, Paul	Paul Brickman	tt0086200
1769970746	1915	1915	Perkin's Pep Producer	Perkin's Pep Producer	Smith, Sidney	Sidney Smith	tt0219245
1746496987	1919	1919	A Girl at Bay	A Girl at Bay	Mills, Tom	Thomas R. Mills	tt0008006
1746438965	1942	1942	The Lady Is Willing	The Lady Is Willing		Mitchell Leisen	tt0034961
1745339934	2002	2002	Big Trouble	Big Trouble	Sonnenfeld, Barry	Barry Sonnenfeld	tt0246464
1745341245	2008	2007	Choose Connor	Choose Connor	Eberl, Luke	Lucas Elliot Eberl	tt0495026
1746574741	1937	1937	Guns in the Dark	Guns in the Dark	Newfield, Sam	Sam Newfield	tt0028969
1746524488	1912	1912	His Secretary	His Secretary	Merwin, Bannister	Bannister Merwin	tt0002249
1746514898	1910	1910	There, Little Girl, Don't Cry	There, Little Girl, Don't Cry			tt1389569
1745309496	2007	2007	Mr. Brooks	Mr. Brooks	Evans, Bruce A.	Bruce A. Evans	tt0780571
1769970848	1915	1915	Hartney Merwin's Adventure	Hartney Merwin's Adventure		E.A. Martin	tt0470331
1746508947	1924	1924	Broken Laws	The Broken Law	Neill, R. William	Paul Hurst	tt0016687
1746578008	1938	1938	Touchdown, Army	Touchdown, Army	Neumann, Kurt	Kurt Neumann	tt0030886
1746475285	1948	1948	Check Your Guns	Check Your Guns	Taylor, Ray	Ray Taylor	tt0039259
1746583639	1929	1929	The Last Roundup	The Last Round up	McGowan, J. P.	J.P. McGowan	tt0020079
1746585049	1929	1929	Honky Tonk	Honky Tonk	Bacon, Lloyd	Lloyd Bacon	tt0019997
1746494011	1910	1910	The Recreation of an Heiress	The Recreation of an Heiress	Powell, Frank	Frank Powell	tt0363017
1746569788	1956	1955	The Square Jungle	The Square Jungle	Hopper, Jerry	Jerry Hopper	tt0048654
1746524884	1916	1915	The Come-Back	The Comeback	Balshofer, Fred J.	Raoul Walsh	tt0005125
1746533037	1915	1915	How Molly Malone Made Good	How Molly Malone Made Good	McGill, Lawrence B.	Lawrence B. McGill	tt0005522
1746512093	1917	1917	The Duchess of Doubt	The Duchess of Doubt	Baker, George D.	George D. Baker	tt4830834
1746580660	1935	1935	Stolen Harmony	Stolen Harmony	Werker, Alfred	Alfred L. Werker	tt0027043
1746501233	1915	1915	The Italian	The Italian	Barker, Reginald	Reginald Barker	tt0005557

1746514332	1927	1927	Papa's Boy	Papa's Boy	Taurog, Norman	Norman Taurog	tt0248288
1746491756	1945	1945	Counter-Attack	Counter Attack	Korda, Zoltan	Zoltan Korda	tt0037618
1746409046	1984	1984	Silent Night, Deadly Night	Silent Night, Deadly Night	Sellier, Jr., Charles E.	Charles E. Sellier Jr.	tt0088117
1746465093	1946	1946	West of the Alamo	West of the Alamo	Drake, Oliver	Oliver Drake	tt0039099
1746436203	1997	1996	Sudden Manhattan	Sudden Manhattan	Shelly, Adrienne	Adrienne Shelly	tt0117775
1746419383	1993	1992	Rich in Love	Rich in Love	Beresford, Bruce	Bruce Beresford	tt0105256
1746454396	1941	1941	Week-End in Havana	Week End in Havana	Lang, Walter	Walter Lang	tt0034379
1746584373	1940	1941	Mr. Washington Goes to Town	Mr. Washington Goes to Town	Buell, Jed	Jed Buell	tt0033925
1746465242	1944	1944	Gunsmoke Mesa	Gunsmoke Mesa	Fraser, Harry	Harry L. Fraser	tt0036889
1746394522	1985	1985	Gymkata	Gymkata	Clouse, Robert	Robert Clouse	tt0089243
1746569687	1954	1954	The Bob Mathias Story	The Bob Mathias Story	Lyon, Francis D.	Francis D. Lyon	tt0046795
1746486871	1920	1920	The Great Redeemer	The Great Redeemer	Brown, Clarence	Clarence Brown	tt0011246
1746524754	1916	1916	What Will People Say?	What Will People Say?	Blaché, Alice	Alice Guy	tt0007552
1746568003	1932	1931	Stranger in Town	Stranger in Town	Kenton, Erle C.	Erle C. Kenton	tt0023526
1746567574	1952	1952	Invasion U.S.A.	Invasion, U.S.A.	Green, Alfred E.	Alfred E. Green	tt0044750
1746581646	1928	1928	Top Sergeant Mulligan	Top Sergeant Mulligan	Hogan, James P.	James P. Hogan	tt0019484
1746576133	1932	1932	Mason of the Mounted	Mason of the Mounted	Fraser, Harry	Harry L. Fraser	tt0023195
1746570893	1935	1935	Bad Boy	Bad Boy	Blystone, John	John G. Blystone	tt0026093
1746439420	2000	2000	Gone in Sixty Seconds	Gone in Sixty Seconds	Sena, Dominic	Dominic Sena	tt0187078
1746582285	1930	1930	Phantom of the Desert	Phantom of the Desert	Webb, Harry S.	Harry S. Webb	tt0021248
1746198291	1966	1966	A Man Could Get Killed	A Man Could Get Killed	Neame, Ronald; Owen, Cliff	Ronald Neame	tt0060662
1746419928	1991	1991	The Doors	The Doors	Stone, Oliver	Oliver Stone	tt0101761
1745340033	2002	2001	e-Dreams	Dream	Chin, Wonsuk	Leo Fialho	tt0279792
1746569880	1958	1958	The Flame Barrier	The Flame Barrier	Landres, Paul	Paul Landres	tt0051618
1769971969	1969	1969	Infrasexum	Infrasexum		Carlos Tobalina	tt0170054
1746219364	1974	1974	The Towering Inferno	The Towering Inferno	Guillermin, John	John Guillermin	tt0072308
1746483826	1917	1917	Her Beloved Enemy	Her Beloved Enemy	Warde, Ernest	Ernest C. Warde	tt0175719
1746438936	1942	1942	Kid Glove Killer	Kid Glove Killer	Zinnemann, Fred	Fred Zinnemann	tt0034941
1746408446	1986	1986	Dangerously Close	Dangerously Close	Pyun, Albert	Albert Pyun	tt0090904
1746492055	1948	1948	Disaster	Disaster	Pine, William H.	William H. Pine	tt0040293
1746235072	1975	1975	Bug	Bug	Szwarc, Jeannot	Jeannot Szwarc	tt0072750
1745271957	2001	2001	The Affair of the Necklace	The Affair of the Necklace	Shyer, Charles	Charles Shyer	tt0242252
1746580485	1937	1937	Champagne Waltz	Champagne Waltz	Sutherland, A. Edward	A. Edward Sutherland	tt0028705
1746502693	1915	1915	Tainted Money	Tainted Money	Davis, Ulysses	Ulysses Davis	tt0006133
1746542665	1950	1950	Customs Agent	Customs Agent	Friedman, Seymour	Seymour Friedman	tt0042366
1769976710	1929	1929	A Bird In The Hand	A Bird in the Hand	Graham, Walter	Walter Graham	tt0019694
1746410457	1985	1985	Moving Violations	Moving Violations	Israel, Neal	Neal Israel	tt0089629
1746492734	1947	1947	West to Glory	West to Glory	Taylor, Ray	Ray Taylor	tt0039979
1746565322	1932	1932	The Seventh Commandment	The Seventh Commandment	Hogan, James P.	Dwain Esper	tt0121730
1746473890	1942	1942	Iceland	Iceland	Humberstone, Bruce	H. Bruce Humberstone	tt0034886
1746520628	1926	1926	Beyond All Odds	Beyond All Odds	Neitz, Alvin J.	Alan James	tt0015619
1746570817	1952	1952	The Happy Time	The Happy Time	Fleischer, Richard	Richard Fleischer	tt0044687
1746410645	1994	1994	Disclosure	Disclosure	Levinson, Barry	Barry Levinson	tt0109635
1746409350	1984	1984	Exterminator 2	Exterminator 2	Buntzman, Mark	Mark Buntzman	tt0087229
1746515766	1917	1917	Cleopatra	Cleopatra	Edwards, J. Gordon	J. Gordon Edwards	tt0007801
1769974304	1916	1916	The Desert Calls Its Own	The Desert Calls Its Own	Mix, Tom	Tom Mix	tt0006578
1745302266	2007	2007	Kickin' It Old Skool	Kickin' It Old Skool	Glazer, Harv	Harvey Glazer	tt0772178
1746453461	1943	1942	The Pay Off	The Pay Off	Dreifuss, Arthur	Arthur Dreifuss	tt0036255
1746581641	1929	1929	Pals of the Prairie	Pals of the Prairie	King, Louis	Louis King	tt0020256
1746577039	1951	1951	Native Son	Native Son	Chenal, Pierre	Pierre Chenal	tt0042781
1746500555	1950	1950	The Cariboo Trail	The Cariboo Trail	Marin, Edwin L.	Edwin L. Marin	tt0042315
1746577210	1950	1950	No Man of Her Own	No Man of Her Own	Leisen, Mitchell	Mitchell Leisen	tt0041694
1745282533	2007	2007	War	War	Atwell, Philip G.	Philip G. Atwell	tt0499556
1746583296	1951	1951	Comin' Round the Mountain	Comin' Round the Mountain	Lamont, Charles	Charles Lamont	tt0043425
1746455377	1996	1996	Kids in the Hall Brain Candy	Kids in the Hall: Brain Candy	Makin, Kelly	Kelly Makin	tt0116768
1746492087	1948	1948	Kidnapped	Kidnapped	Beaudine, William	William Beaudine	tt0040507
1746454946	1996	1996	The Line King: The Al Hirschfeld Story	The Line King: The Al Hirschfeld Story	Dryfoos, Susan W.	Susan Warms Dryfoos	tt0116886

1746571078	1934	1934	The Dragon Murder Case	The Dragon Murder Case	Humberstone, H. Bruce	H. Bruce Humberstone	tt0025069
1746465069	1947	1946	Gallant Bess	Gallant Bess	Marton, Andrew	Andrew Marton	tt0038547
1769976124	1926	1926	Back Fire	Back Fire		Scott Pembroke	tt0415543
1746581658	1929	1929	Untamed	Untamed	Conway, Jack	Jack Conway	tt0020538
1746582743	1935	1935	The Ivory Handled Gun	The Ivory Handled Gun	Taylor, Ray	Ray Taylor	tt0026541
1746585450	1940	1940	Rancho Grande	Rancho Grande	McDonald, Frank	Frank McDonald	tt0032970
1746520799	1949	1949	Trail of the Yukon	Trail of the Yukon	Crowley, William X.	William Beaudine	tt0041981
1746576721	1932	1932	The Conquerors	The Conquerors	Wellman, William A.	William A. Wellman	tt0022778
1746582101	1930	1929	Hit the Deck	Hit the Deck	Reed, Luther	Luther Reed	tt0020977
1746423323	1992	1992	Deep Cover	Deep Cover	Duke, Bill	Bill Duke	tt0104073
1746234621	1968	1968	The Queen	The Queen	Simon, Frank	Frank Simon	tt0063477
1746569795	1954	1954	Twist of Fate	Twist of Fate	Miller, David	David Miller	tt0047625
1746545254	1940	1940	Yesterday's Heroes	Yesterday's Heroes	Leeds, Herbert I.	Herbert I. Leeds	tt0033278
1746512146	1917	1917	Chris and His Wonderful Lamp	Chris and His Wonderful Lamp	Crosland, Alan	Alan Crosland	tt0168582
1746410205	1987	1987	The Witches of Eastwick	The Witches of Eastwick	Miller, George	George Miller	tt0094332
1769969347	1930	1930	Two Plus Fours	Two Plus Fours	McCarey, Raymond	Ray McCarey	tt0021496
1746409062	1992	1992	White Men Can't Jump	White Men Can't Jump	Shelton, Ron	Ron Shelton	tt0105812
1746570428	1952	1952	Colorado Sundown	Colorado Sundown	Witney, William	William Witney	tt0044506
1746583042	1938	1938	Illegal Traffic	Illegal Traffic	King, Louis	Louis King	tt0030266
1746520304	1925	1925	A Streak of Luck	A Streak of Luck	Thorpe, Richard	Richard Thorpe	tt0166827
1746560961	1932	1932	The Drifter	The Drifter	O'Connor, Wm.	William A. O'Connor	tt0022840
1746578017	1935	1935	Streamline Express	Streamline Express	Fields, Leonard	Leonard Fields	tt0027053
1745357710	2002	2002	Blue Crush	Blue Crush	Stockwell, John	John Stockwell	tt0300532
1746524024	1922	1922	Caught Bluffing	Caught Bluffing	Hillyer, Lambert	Lambert Hillyer	tt0013003
1746574372	1951	1951	The Enforcer	The Enforcer	Windust, Bretaigne	Bretaigne Windust	tt0043503
1746583202	1931	1931	Sea Devils	Sea Devils	Levering, Joseph	Joseph Levering	tt0022351
1746584994	1939	1939	6,000 Enemies	6,000 Enemies	Seitz, George B.	George B. Seitz	tt0031014
1746585445	1940	1940	The Villain Still Pursued Her	The Villain Still Pursued Her	Cline, Edward	Edward F. Cline	tt0033224
1746235502	1976	1976	Taxi Driver	Taxi Driver	Scorsese, Martin	Martin Scorsese	tt0075314
1746455725	1998	1998	A Soldier's Daughter Never Cries	A Soldier's Daughter Never Cries	Ivory, James	James Ivory	tt0120835
1746234823	1972	1972	The Doberman Gang	The Doberman Gang	Chudnow, Byron Ross	Byron Chudnow	tt0068491
1746580857	1935	1935	Fighting Shadows	Fighting Shadows	Selman, David	David Selman	tt0026355
1746571116	1934	1934	Father Brown, Detective	Father Brown, Detective	Sedgwick, Edward	Edward Sedgwick	tt0026341
1746509260	1927	1927	The Slingshot Kid	The Slingshot Kid	King, Louis	Louis King	tt0165476
1746184607	1965	1965	The Agony and the Ecstasy	The Agony and the Ecstasy	Reed, Carol	Carol Reed	tt0058886
1746410840	1989	1988	Criminal Law	Criminal Law	Campbell, Martin	Martin Campbell	tt0097125
1746578659	1963	1963	The Shotgun Wedding	Shotgun Wedding	Petroff, Boris L.	Boris Petroff	tt0057496
1746437604	1995	1995	Tank Girl	Tank Girl	Talalay, Rachel	Rachel Talalay	tt0114614
1746524824	1915	1915	The Dawn of a Tomorrow	The Dawn of a Tomorrow	Kirkwood, James	James Kirkwood	tt0005180
1746555391	1955	1955	Robbers' Roost	Robbers' Roost	Salkow, Sidney	Sidney Salkow	tt0048558
1746574457	1933	1933	The Barber Shop	The Barber Shop	Ripley, Arthur	Arthur Ripley	tt0023779
1746562424	1954	1954	Black Horse Canyon	Black Horse Canyon	Hibbs, Jesse	Jesse Hibbs	tt0046785
1746437020	1943	1943	The Lone Star Trail	The Lone Star Trail	Taylor, Ray	Ray Taylor	tt0036118
1745341776	2002	2002	Scooby-Doo	Scooby Doo	Gosnell, Raja	Raja Gosnell	tt0267913
1746560282	1963	1963	The Prize	The Prize	Robson, Mark	Mark Robson	tt0057426
1746501585	1915	1915	The Crimson Wing	The Crimson Wing	Calvert, E. H.	E.H. Calvert	tt0005158
1746576712	1932	1932	Me and My Gal	Me and My Gal	Walsh, Raoul	Raoul Walsh	tt0023202
1746560646	1932	1932	Cock of the Air	Cock of the Air	Buckingham, Tom	Tom Buckingham	tt0022769
1746435215	1942	1942	Desperate Journey	Desperate Journey	Walsh, Raoul	Raoul Walsh	tt0034646
1746576929	1951	1951	Roadblock	Roadblock	Daniels, Harold	Harold Daniels	tt0043973
1769969417	1911	1911	The Eastern Cowboy	The Eastern Cowboy		Allan Dwan	tt0444785
1745359942	2001	2001	Session 9	Session 9	Anderson, Brad	Brad Anderson	tt0261983
1746562123	1956	1956	The Last Hunt	The Last Hunt	Brooks, Richard	Richard Brooks	tt0049432
1746211455	1971	1971	Werewolves on Wheels	Werewolves on Wheels	Levesque, Michel	Michel Levesque	tt0067972
1746554794	1957	1957	Monkey on My Back	Monkey on My Back	DeToth, Andre; Post, Ted	André De Toth	tt0050719
1746534812	1920	1920	Eyes of the Heart	Eyes of the Heart	Powell, Paul	Paul Powell	tt0011166
1746453613	1940	1940	Honeymoon Deferred	Honeymoon Deferred	Landers, Lew	Lew Landers	tt0032607

1746500297	1925	1925	Below the Line	Below the Line	Raymaker, Herman	Herman C. Raymaker	tt0015616
1746580578	1936	1936	Tundra	Tundra	Dawn, Norman	Norman Dawn	tt0028422
1746581838	1928	1928	Outcast Souls	Outcast Souls	Chaudet, Louis	Louis Chaudet	tt0019240
1746184521	1910	1910	Aunt Maria's Substitute	Aunt Maria's Substitute			tt1914206
1745272497	2000	2000	South of Heaven, West of Hell	South of Heaven, West of Hell	Yoakam, Dwight	Dwight Yoakam	tt0179473
1746567255	1933	1933	Samarang	Samarang	Wing, Ward	Ward Wing	tt0034177
1746583912	1930	1930	Sweet Kitty Bellairs	Sweet Kitty Bellairs	Green, Alfred E.	Alfred E. Green	tt0021439
1746576854	1932	1932	Little Orphan Annie	Little Orphan Annie	Robertson, John	John S. Robertson	tt0023142
1746580409	1938	1938	Tom Sawyer, Detective	Tom Sawyer, Detective	King, Louis	Louis King	tt0030878
1746523985	1915	1915	John Glayde's Honor	John Glayde's Honor	Irving, George	George Irving	tt0005572
1746433954	1998	1998	Star Trek: Insurrection	Star Trek: Insurrection	Frakes, Jonathan	Jonathan Frakes	tt0120844
1746514229	1916	1916	John Needham's Double	John Needham's Double	Weber, Lois; Smalley, Phillips	Phillips Smalley	tt0006883
1746584016	1938	1938	Change of Heart	Change of Heart	Tinling, James	James Tinling	tt0029983
1746217174	1967	1967	Valley of the Dolls	Valley of the Dolls	Robson, Mark	Mark Robson	tt0062430
1746419458	1940	1940	Shooting High	Shooting High	Green, Alfred E.	Alfred E. Green	tt0033044
1746565185	1953	1953	Striporama	Striporama	Intrator, Jerald	Jerald Intrator	tt0047535
1746420331	1940	1940	Knights of the Range	Knights of the Range	Selander, Lesley	Lesley Selander	tt0032674
1746454491	1945	1945	Strange Holiday	Strange Holiday	Oboler, Arch	Arch Oboler	tt0038125
1746581832	1929	1929	The Trespasser	The Trespasser	Goulding, Edmund	Edmund Goulding	tt0020514
1746500484	1924	1924	The Gaiety Girl	The Gaiety Girl	Baggot, King	King Baggot	tt0014930
1745309127	2006	2006	Lady in the Water	Lady in the Water	Shyamalan, M. Night	M. Night Shyamalan	tt0452637
1746436847	1999	1999	Dogma	Dogma	Smith, Kevin	Kevin Smith	tt0120655
1746199922	1970	1970	Tobacco Roody	Tobacco Roody	Buckalew	Bethel Buckalew	tt0128769
1746581226	1959	1959	The Giant Behemoth	The Giant Behemoth	Lourie, Eugene	Douglas Hickox	tt0052611
1746577136	1950	1950	Pretty Baby	Pretty Baby	Windust, Bretaigne	Bretaigne Windust	tt0042858
1746534807	1920	1920	Love's Protegé	Love's Protegé	Wright, Walter	Walter Wright	tt0196721
1746486159	1918	1918	The Eleventh Commandment	The Eleventh Commandment	Ince, Ralph W.	Ralph Ince	tt0009034
1746249816	1976	1976	Scorchy	Scorchy	Avedis, Hikmet	Howard Avedis	tt0075174
1746202641	1970	1969	Land Raiders	Land Raiders	Juran, Nathan	Nathan Juran	tt0065962
1745372675	2008	2008	Marley & Me	Marley & Me	Frankel, David	David Frankel	tt0822832
1746507907	1950	1950	The Gunfighter	The Gunfighter	King, Henry	Henry King	tt0042531
1746561585	1956	1956	While the City Sleeps	While the City Sleeps	Lang, Fritz	Fritz Lang	tt0049949
1746524792	1916	1916	Romeo and Juliet	Romeo and Juliet	Noble, John W.	Francis X. Bushman	tt0007272
1746423012	1998	1997	Plump Fiction	Plump Fiction	Koherr, Bob	Bob Koherr	tt0117350
1746435590	1942	1942	Rolling Down the Great Divide	Rolling Down the Great Divide	Stewart, Peter	Sam Newfield	tt0035265
1746507916	1924	1924	Galloping Fish	The Galloping Fish	Andrews, Del	Del Andrews	tt0014934
1746409952	1982	1982	The Beastmaster	The Beastmaster	Coscarelli, Don	Don Coscarelli	tt0083630
1746583930	1929	1929	Lucky Star	Lucky Star	Borzage, Frank	Frank Borzage	tt0020122
1746580299	1938	1938	Rebellious Daughters	Rebellious Daughters	Yarborough, Jean	Jean Yarbrough	tt0030659
1746532632	1914	1914	The Daughters of Men	The Daughters of Men	Terwilliger, George	George Terwilliger	tt0003825
1746483465	1918	1918	Secret Code	The Secret Code	Parker, Albert	Albert Parker	tt0009593
1746420791	1994	1994	Ed Wood	Ed Wood	Burton, Tim	Tim Burton	tt0109707
1746437933	1943	1942	Time to Kill	Time to Kill	Leeds, Herbert I.	Herbert I. Leeds	tt0035443
1746393966	1989	1989	Troop Beverly Hills	Troop Beverly Hills	Kanew, Jeff	Jeff Kanew	tt0098519
1746454397	1941	1941	You're the One	You're the One	Murphy, Ralph	Ralph Murphy	tt0034412
1746580619	1936	1936	Lightnin' Bill Carson	Lightnin' Bill Carson	Newfield, Sam	Sam Newfield	tt0027886
1746218779	1971	1971	The Projectionist	The Projectionist	Hurwitz, Harry	Harry Hurwitz	tt0067622
1746248084	1966	1966	Walk, Don't Run	Walk Don't Run	Walters, Charles	Charles Walters	tt0061170
1745372553	2004	2004	Spanglish	Spanglish	Brooks, James L.	James L. Brooks	tt0371246
1746200465	1968	1968	The Devil's Brigade	The Devil's Brigade	McLaglen, Andrew V.	Andrew V. McLaglen	tt0062886
1745340321	2003	2003	Runaway Jury	Runaway Jury	Fleder, Gary	Gary Fleder	tt0313542
1746520036	1924	1924	Daring Love	Daring Love	Edwards, Roland G.	Rowland G. Edwards	tt0014828
1746501498	1910	1910	The Road to Richmond	The Road to Richmond			tt1389547
1746454504	1946	1946	Lover Come Back	Lover Come Back	Seiter, William A.	William A. Seiter	tt0038705
1746423318	1989	1989	The Karate Kid Part III	The Karate Kid Part III	Avildsen, John G.	John G. Avildsen	tt0097647
1746570226	1934	1934	She Made Her Bed	She Made Her Bed	Murphy, Ralph	Ralph Murphy	tt0025775
1746569787	1953	1952	The Member of the Wedding	The Member of the Wedding	Zinnemann, Fred	Fred Zinnemann	tt0044896

1746523561	1915	1915	The Mating	The Mating	West, Raymond B.	Raymond B. West	tt0005728
1746530681	1949	1949	Law of the West	Law of the West	Taylor, Ray	Ray Taylor	tt0041584
1746581146	1959	1959	The FBI Story	The FBI Story	LeRoy, Mervyn	Mervyn LeRoy	tt0052792
1746219335	1971	1971	The Hospital	The Hospital	Hiller, Arthur	Arthur Hiller	tt0067217
1746453543	1942	1942	Mexican Spitfire at Sea	Mexican Spitfire at Sea	Goodwins, Leslie	Leslie Goodwins	tt0035060
1746561090	1932	1932	South Sea Adventures	South Sea Adventures		Zane Grey	tt0216228
1746435266	1941	1940	Stolen Paradise	Stolen Paradise	Gasnier, Louis	Louis J. Gasnier	tt0221585
1746583523	1951	1951	The Prince Who Was a Thief	The Prince Who Was a Thief	Maté, Rudolph	Rudolph Maté	tt0043935
1746580605	1938	1938	Peck's Bad Boy with the Circus	Peck's Bad Boy with the Circus	Cline, Edward F.	Edward F. Cline	tt0030565
1746571358	1935	1935	West Point of the Air	West Point of the Air	Rosson, Richard	Richard Rosson	tt0027196
1746580811	1939	1939	Bad Lands	Bad Lands	Landers, Lew	Lew Landers	tt0031071
1746582716	1936	1936	The Mine with the Iron Door	The Mine with the Iron Door	Howard, David	David Howard	tt0027973
1746520186	1924	1924	Notch Number One	Notch Number One		Ben F. Wilson	tt0338293
1746508740	1924	1924	Do It Now	Do It Now	Worne, Duke	Duke Worne	tt0014850
1746566012	1957	1957	Four Girls in Town	Four Girls in Town	Sher, Jack	Jack Sher	tt0049228
1746514634	1927	1927	The Golden Stallion	The Golden Stallion	Webb, Harry	Harry S. Webb	tt0017943
1746580428	1937	1937	Fit for a King	Fit for a King	Sedgwick, Edward	Edward Sedgwick	tt0028876
1746562103	1956	1956	The Sharkfighters	The Sharkfighters	Hopper, Jerry	Jerry Hopper	tt0049751
1745373939	2002	2002	Stone Reader	Stone Reader	Moskowitz, Mark	Mark Moskowitz	tt0324080
1746512133	1916	1916	The Haunted Station	The Haunted Station	Davis, James	J. Gunnis Davis	tt1534526
1746524002	1916	1916	Kinkaid, Gambler	Kinkaid, Gambler	Wells, Raymond	Raymond Wells	tt0006898
1746184129	1910	1910	After the Ball	After the Ball	Powell, Frank	Frank Powell	tt0001472
1746485615	1918	1918	The Birth of a Race	The Birth of a Race	Noble, John W.	John W. Noble	tt0133418
1746580634	1937	1937	Night of Mystery	Night of Mystery	Dupont, E. A.	Ewald André Dupont	tt0029312
1746582969	1937	1937	Charlie Chan at the Olympics	Charlie Chan at the Olympics	Humberstone, H. Bruce	H. Bruce Humberstone	tt0028708
1746491730	1947	1947	Merton of the Movies	Merton of the Movies	Alton, Robert	Robert Alton	tt0039619
1746474495	1944	1944	Outlaws of Santa Fe	Outlaws of Santa Fe	Bretherton, Howard	Howard Bretherton	tt0037162
1769970563	1967	1967	Teach Me How To Do It!	Teach Me How to Do It!	Meola, Michael	Michael Meola	tt0259043
1746465110	1947	1947	The Millerson Case	The Millerson Case	Archainbaud, George	George Archainbaud	tt0039625
1746208504	1967	1967	Monkeys, Go Home!	Monkeys, Go Home!	McLaglen, Andrew V.	Andrew V. McLaglen	tt0061989
1746560917	1932	1932	Fireman Save My Child	Fireman, Save My Child	Bacon, Lloyd	Lloyd Bacon	tt0022890
1746409496	1988	1987	Twisted Nightmare	Twisted Nightmare	Hunt, Paul	Paul Hunt	tt0134167
1746199022	1969	1969	Guns of the Magnificent Seven	Guns of the Magnificent Seven	Wendkos, Paul	Paul Wendkos	tt0064395
1746216671	1968	1967	Custer of the West	Custer of the West	Siodmak, Robert	Robert Siodmak	tt0062844
1746465116	1942	1942	The Vanishing Virginian	The Vanishing Virginian	Borzage, Frank	Frank Borzage	tt0035504
1746393324	1988	1988	Dakota	Dakota	Holmes, Fred	Fred Holmes	tt0094940
1746423460	1990	1990	Mountains of the Moon	Mountains of the Moon	Rafelson, Bob	Bob Rafelson	tt0100196
1746508682	1924	1924	Stay Single	Stay Single	Sidney, Scott	Scott Sidney	tt0830661
1746249984	1975	1975	The Man in the Glass Booth	The Man in the Glass Booth	Hiller, Arthur	Arthur Hiller	tt0073345
1746520097	1927	1927	The Missing Link	The Missing Link	Reisner, Charles F.	Charles Reisner	tt0018164
1746393319	1985	1985	Wizards of the Lost Kingdom	Wizards of the Lost Kingdom	Olivera, Hector	Héctor Olivera	tt0090333
1746578860	1951	1951	Goodbye, My Fancy	Goodbye, My Fancy	Sherman, Vincent; Mattison, Frank	Vincent Sherman	tt0043595
1746582362	1951	1951	The Longhorn	The Longhorn	Collins, Lewis	Lewis D. Collins	tt0043753
1746454655	1946	1946	The Devil's Playground	The Devil's Playground	Archainbaud, George	George Archainbaud	tt0038472
1746504624	1922	1922	Blind Circumstances	Blind Circumstances	Morante, Milburn	Milburn Morante	tt0012950
1746437931	1941	1941	City of Missing Girls	City of Missing Girls	Clifton, Elmer	Elmer Clifton	tt0033469
1746475286	1948	1948	Homicide for Three	Homicide for Three	Blair, George	George Blair	tt0040447
1746568148	1933	1933	Central Airport	Central Airport	Wellman, William A.	William A. Wellman	tt0023878
1746435688	1940	1940	Escape to Glory	Escape to Glory	Brahm, John	John Brahm	tt0032448
1745293528	2007	2007	No Reservations	No Reservations	Hicks, Scott	Scott Hicks	tt0481141
1746545372	1940	1940	Billy the Kid's Gun Justice	Billy the Kid's Gun Justice	Stewart, Peter	Sam Newfield	tt0135805
1746424049	1940	1940	I'm Still Alive	I'm Still Alive	Reis, Irving	Irving Reis	tt0032622
1746467506	1917	1917	The Flame of Youth	The Flame of Youth	Clifton, Elmer	Elmer Clifton	tt0007952
1746583725	1928	1928	The Ballyhoo Buster	The Ballyhoo Buster	Thorpe, Richard	Richard Thorpe	tt0018672
1746508443	1925	1925	The Shock Punch	The Shock Punch	Sloane, Paul	Paul Sloane	tt0016345
1746486784	1919	1919	Eyes of Youth	Eyes of Youth	Parker, Albert	Albert Parker	tt0010103
1746420220	1993	1993	Cop & 1/2	Cop & ½	Winkler, Henry	Henry Winkler	tt0106613

1746567813	1952	1952	The Ring	The Ring	Neumann, Kurt	Kurt Neumann	tt0045091	
1746524149	1917	1917	The Clean-Up	The Clean Up	Worthington, William	William Worthington	tt0007800	
1746582791	1934	1934	One Is Guilty	One Is Guilty	Hillyer, Lambert	Lambert Hillyer	tt0025599	
1746570871	1933	1933	Ann Vickers	Ann Vickers	Cromwell, John	John Cromwell	tt0023759	
1746515017	1923	1923	Fog Bound	Fog Bound	Willat, Irvin	Irvin Willat	tt0014050	
1746559986	1964	1964	Goodbye Charlie	Goodbye Charlie	Minnelli, Vincente	Vincente Minnelli	tt0058154	
1746507570	1926	1926	Paris at Midnight	Paris at Midnight	Hopper, E. Mason	E. Mason Hopper	tt0017252	
1746576904	1931	1931	Not Exactly Gentlemen	Not Exactly Gentlemen	Stoloff, Benjamin	Benjamin Stoloff	tt0022480	
1746495802	1920	1920	The Forbidden Thing	The Forbidden Thing	Dwan, Allan	Allan Dwan	tt0011198	
1746408511	1984	1984	Under the Volcano	Under the Volcano	Huston, John	John Huston	tt0088322	
1769970789	1913	1913	How to Live 100 Years	How to Live 100 Years	Miles, David	David Miles	tt0233896	
1746185684	1965	1965	Faster, Pussycat! Kill! Kill!	Faster, Pussycat! Kill! Kill!	Meyer, Russ	Russ Meyer	tt0059170	
1746515372	1918	1918	Whatever the Cost	Whatever the Cost	Ensminger, Robert	Robert Ensminger	tt0190133	
1746554787	1964	1964	Magic Christmas Tree	Magic Christmas Tree	Parish, Richard C.	Richard C. Parish	tt0195039	
1746453487	1945	1945	Back to Bataan	Back to Bataan	Dmytryk, Edward	Edward Dmytryk	tt0037522	
1746583852	1929	1928	Lucky Boy	Lucky Boy	Taurog, Norman; Wilson, Charles C.	Norman Taurog	tt0020121	
1746555955	1955	1955	Underwater!	Underwater!	Sturges, John	John Sturges	tt0048764	
1746555855	1956	1956	Disneyland, U.S.A.	Disneyland, U.S.A.	Luske, Hamilton S.	Hamilton Luske	tt0472588	
1746520508	1949	1949	On the Town	On the Town	Kelly, Gene; Donen, Stanley	Stanley Donen	tt0041716	
1746580869	1936	1936	General Spanky	General Spanky	Newmeyer, Fred; Douglas, Gordon	Gordon Douglas	tt0027665	
1746501377	1915	1915	The Explorer	The Explorer	Melford, George	George Melford	tt0126898	
1746525084	1920	1920	A Lady in Love	A Lady in Love	Edwards, Walter	Walter Edwards	tt0011381	
1746435134	1940	1940	Charlie Chan in Panama	Charlie Chan in Panama	Foster, Norman	Norman Foster	tt0032325	
1746560759	1932	1932	What Price Hollywood?	What Price Hollywood?	Cukor, George	George Cukor	tt0023686	
1746574540	1952	1952	Somebody Loves Me	Somebody Loves Me	Brecher, Irving	Irving Brecher	tt0045171	
1746420366	1993	1993	Poetic Justice	Poetic Justice	Singleton, John	John Singleton	tt0107840	
1746573958	1963	1963	Critic's Choice	Critic's Choice	Weis, Don	Don Weis	tt0056964	
1746524908	1915	1915	The Closing Net	The Closing Net	José, Edward	Edward José	tt0005102	
1746580793	1935	1935	Unknown Woman	Unknown Woman	Rogell, Albert	Albert S. Rogell	tt0027161	
1746520662	1925	1925	Border Vengeance	Border Vengeance	Webb, Harry	Harry S. Webb	tt0015639	
1746567794	1952	1952	Lure of the Wilderness	Lure of the Wilderness	Negulesco, Jean	Jean Negulesco	tt0044859	
1746420995	1982	1982	Still of the Night	Still of the Night	Benton, Robert	Robert Benton	tt0084732	
1746508263	1924	1924	The Bowery Bishop	The Bowery Bishop	Campbell, Colin	Colin Campbell	tt0014732	
1746437985	1942	1942	The Black Swan	The Black Swan	King, Henry	Henry King	tt0034522	
1746453614	1943	1943	Colt Comrades	Colt Comrades	Selander, Lesley	Lesley Selander	tt0035744	
1746410851	1994	1994	8 Seconds	8 Seconds	Avildsen, John G.	John G. Avildsen	tt0109021	
1745372323	2003	2003	Willard	Willard	Morgan, Glen	Glen Morgan	tt0310357	
1746487290	1918	1918	Her American Husband	Her American Husband	Hopper, E. Mason	E. Mason Hopper	tt0148271	
**1746581925**	**1930**	**1930**	**Montana Moon**	**Montana Moon**	**St. Clair, Malcolm**	**Malcolm St. Clair**	**tt0021152**	1
1746520966	1948	1948	The Winner's Circle	The Winner's Circle	Feist, Felix E.	Felix E. Feist	tt0040969	
1746585192	1929	1929	Code of the West	Code of the West	McGowan, J. P.	J.P. McGowan	tt0019778	
1746415810	1917	1917	Lorelei of the Sea	Lorelei of the Sea	Otto, Henry	Henry Otto	tt0008215	
1746575031	1936	1936	Without Orders	Without Orders	Landers, Lew	Lew Landers	tt0028512	
1746520323	1924	1925	The Girl on the Stairs	The Girl on the Stairs	Worthington, William	William Worthington	tt0014948	
1746578060	1938	1938	Wanted by the Police	Wanted by the Police	Bretherton, Howard	Howard Bretherton	tt0030954	
1746561778	1956	1956	Attack	Attack	Aldrich, Robert	Robert Aldrich	tt0048966	
1746512444	1910	1910	Spoony Sam	Spoony Sam			tt0348747	
1746483862	1917	1917	Blue Jeans	Blue Jeans	Collins, John H.	John H. Collins	tt0007711	
1746498262	1917	1917	The Defeat of the City	The Defeat of the City	Mills, Thomas R.	Thomas R. Mills	tt0007846	
1746453535	1942	1942	Thunder River Feud	Thunder River Feud	Luby, S. Roy	S. Roy Luby	tt0035439	
1746420553	1992	1992	Patriot Games	Patriot Games	Noyce, Phillip	Phillip Noyce	tt0105112	
1746566894	1934	1934	Enlighten Thy Daughter	Enlighten Thy Daughter	Varley, John	John Varley	tt0023988	
1746497478	1921	1921	The Bride's Confession	The Bride's Confession	Abramson, Ivan	Ivan Abramson	tt0012974	
1746503414	1921	1921	Two Minutes To Go	Two Minutes to Go	Ray, Charles	Charles Ray	tt0012785	
1746581689	1929	1928	Hoofbeats of Vengeance	Hoofbeats of Vengeance	MacRae, Henry	Henry MacRae	tt0019998	
1746583936	1931	1931	In Line of Duty	In Line of Duty	Glennon, Bert	Bert Glennon	tt0021995	
1746578885	1963	1963	The Right Hand of the Devil	The Right Hand of the Devil		Aram Katcher	tt0268576	

1746509189	1925	1924	Always Ridin' To Win	Always Ridin' to Win	Sheldon, Forrest	Forrest Sheldon	tt0015567
1746504046	1918	1918	The Firebrand	The Firebrand	Lawrence, Edmund	Edmund Lawrence	tt0009069
1746532638	1923	1923	The Self-Made Wife	The Self Made Wife	Dillon, Jack	John Francis Dillon	tt0014457
1746465207	1942	1942	Men of Texas	Men of Texas	Enright, Ray	Ray Enright	tt0035058
1746561977	1956	1956	The Power and the Prize	The Power and the Prize	Koster, Henry	Henry Koster	tt0049635
1746555148	1955	1955	Pirates of Tripoli	Pirates of Tripoli	Feist, Felix	Felix E. Feist	tt0048493
1746566349	1954	1953	Wicked Woman	Wicked Woman	Rouse, Russell	Russell Rouse	tt0047676
1746453955	1998	1998	Dead Man on Campus	Dead Man on Campus	Cohn, Alan	Alan Cohn	tt0118301
1745359161	2002	2002	The Four Feathers	The Four Feathers	Kapur, Shekhar	Shekhar Kapur	tt0240510
1746454057	1941	1941	The Return of Daniel Boone	The Return of Daniel Boone	Hillyer, Lambert	Lambert Hillyer	tt0034096
1746509241	1949	1949	Jigsaw	Jigsaw	Markle, Fletcher	Fletcher Markle	tt0041523
1746573981	1959	1959	Westbound	Westbound	Boetticher, Budd	Budd Boetticher	tt0053441
1746507781	1925	1925	His Greatest Battle	His Greatest Battle	Horner, Robert J.	Robert J. Horner	tt0230295
1746585138	1928	1929	The Cop	Copy	Crisp, Donald	Norman Houston	tt0129011
1746421187	1994	1994	Cabin Boy	Cabin Boy	Resnick, Adam	Adam Resnick	tt0109361
1746421347	1993	1992	Visions of Light	Visions of Light	Glassman, Arnold ; McCarthy, Todd;	Arnold Glassman	tt0105764
1746554194	1965	1965	Laurel and Hardy's Laughing 20's	Laurel and Hardy's Laughing 20's		Robert Youngson	tt0059379
1746394213	1985	1985	The Stuff	The Stuff	Cohen, Larry	Larry Cohen	tt0090094
1746584101	1939	1939	Three Sons	Three Sons	Hively, Jack	Jack Hively	tt0032031
1746583394	1931	1931	The Deadline	The Deadline	Hillyer, Lambert	Lambert Hillyer	tt0021789
1746439132	1944	1944	I'm from Arkansas	I'm from Arkansas	Landers, Lew	Lew Landers	tt0036941
1746393666	1987	1987	Wanted: Dead or Alive	Wanted: Dead or Alive	Sherman, Gary	Gary Sherman	tt0094293
1746574527	1953	1952	The Four Poster	The Four Poster	Reis, Irving	Irving Reis	tt0044631
1746420394	1987	1987	Revenge of the Nerds II: Nerds in Paradise	Revenge of the Nerds II: Nerds in Paradise	Roth, Joe	Joe Roth	tt0093857
1746503710	1920	1920	The Woman in the Suitcase	The Woman in the Suitcase	Niblo, Fred	Fred Niblo	tt0011877
1746514481	1912	1912	At Coney Island	At Coney Island	Sennett, Mack	Mack Sennett	tt0002034
1746424226	1941	1941	The Gunman from Bodie	The Gunman from Bodie	Bennet, Spencer	Spencer Gordon Bennet	tt0033683
1746584925	1939	1939	The Kid from Texas	The Kid from Texas	Simon, S. Sylvan	S. Sylvan Simon	tt0031532
1746582866	1937	1937	Seventh Heaven	Seventh Heaven	King, Henry	Henry King	tt0029539
1746393273	1987	1987	Lethal Weapon	Lethal Weapon	Donner, Richard	Richard Donner	tt0093409
1746491878	1948	1948	Louisiana Story	Louisiana Story	Flaherty, Robert	Robert J. Flaherty	tt0040550
1769969406	1965	1965	Chained Girls	Chained Girls	Mawra, Joseph P.	Joseph P. Mawra	tt0123652
1746454768	2000	1999	The Big Kahuna	The Big Kahuna	Swanbeck, John	John Swanbeck	tt0189584
1746582647	1939	1939	Conspiracy	Conspiracy	Landers, Lew	Lew Landers	tt0031177
1746574095	1959	1959	Tokyo After Dark	Tokyo After Dark	Herman, Norman T.	Norman T. Herman	tt0053364
1746582275	1931	1931	So This Is Arizona	So This Is Arizona	Kirkland, David	J.P. McGowan	tt0267936
1746200171	1969	1969	Generation	Generation	Schaefer, George	George Schaefer	tt0064365
1746580490	1938	1938	Always in Trouble	Always in Trouble	Santley, Joseph	Joseph Santley	tt0029862
1769969383	1930	1930	Under the Cock-Eyed Moon	Under the Cock Eyed Moon	Fox, Wallace	Wallace Fox	tt0830690
1746585373	1939	1939	Man from Texas	The Man from Texas	Herman, Al	Albert Herman	tt0031618
1769973209	1970	1970	Hot Line	Hot Line	Goetz, Tommy	Tommy Goetz	tt0220532
1746200332	1969	1968	The Song and the Silence	The Song and the Silence	Cohen, Nathan	Nathan Cohen	tt0065018
1746574624	1937	1937	Love in a Bungalow	Love in a Bungalow	McCarey, Raymond B.	Ray McCarey	tt0029172
1746570889	1935	1935	Grand Old Girl	Grand Old Girl	Robertson, John	John S. Robertson	tt0026432
1746492862	1944	1944	Are These Our Parents	Are These Our Parents?	Nigh, William	William Nigh	tt0036609
1746525172	1920	1920	Out of the Dust	Out of the Dust	McCarthy, John P.	John P. McCarthy	tt0196818
1745375178	2010	2010	Is It Just Me?	Is It Just Me?	Calciano, J C	J.C. Calciano	tt1533053
1746554483	1964	1965	Country Music on Broadway	Country Music on Broadway	Duncan, Victor	Victor Duncan	tt0337947
1746555696	1957	1957	The Spirit of St. Louis	The Spirit of St. Louis	Wilder, Billy	Billy Wilder	tt0051003
1746584093	1936	1936	Times Square Playboy	Times Square Playboy	McGann, William	William C. McGann	tt0028383
1746394031	1984	1984	The Terminator	The Terminator	Cameron, James	James Cameron	tt0088247
1746577657	1938	1938	Billy the Kid Returns	Billy the Kid Returns	Kane, Joe	Joseph Kane	tt0029915
1746520303	1925	1925	The Scarlet West	The Scarlet West	Adolfi, John G.	John G. Adolfi	tt0016320
1746569697	1954	1954	Two Guns and a Badge	Two Guns and a Badge	Collins, Lewis D.	Lewis D. Collins	tt0047626
1746492227	1945	1945	Song of the Sarong	Song of the Sarong	Young, Harold	Harold Young	tt0038103
1746572105	1910	1910	The Egret Hunter	The Egret Hunter		Sidney Olcott	tt0393255
1746270763	1980	1980	No Nukes	No Nukes	Schlossberg, Julian; Goldberg, Dann	Danny Goldberg	tt0081242

1746573869	1959	1959	But Not for Me	But Not for Me	Lang, Walter	Walter Lang	tt0052662
1746576915	1961	1961	The Young Savages	The Young Savages	Frankenheimer, John	John Frankenheimer	tt0055633
1746561979	1953	1953	Here Come the Girls	Here Come the Girls	Binyon, Claude	Claude Binyon	tt0045866
1746455492	1996	1996	The Craft	The Craft	Fleming, Andrew	Andrew Fleming	tt0115963
1746454994	1998	1998	I Got the Hook-Up	I Got the Hook Up	Martin, Michael	Michael Martin	tt0131436
1746483824	1917	1917	The Girl Glory	The Girl, Glory	Neill, R. William	Roy William Neill	tt0175666
1746584548	1929	1929	Red Hot Rhythm	Red Hot Rhythm	McCarey, Leo	Leo McCarey	tt0020315
1746582068	1930	1930	Around the Corner	Around the Corner	Glennon, Bert	Bert Glennon	tt0020650
1746584311	1940	1940	Lucky Cisco Kid	Lucky Cisco Kid	Humberstone, H. Bruce	H. Bruce Humberstone	tt0032732
1746454522	1995	1995	Dangerous Minds	Dangerous Minds	Smith, John N.	John N. Smith	tt0112792
1746234411	1968	1968	Yours, Mine, and Ours	Yours, Mine and Ours	Shavelson, Melville	Melville Shavelson	tt0063829
1746524086	1923	1923	Lone Fighter	Lone Fighter	Russell, Albert	Albert Russell	tt0129206
1746574785	1937	1937	Another Dawn	Another Dawn	Dieterle, William	William Dieterle	tt0028579
1746542694	1950	1950	The Fighting Stallion	The Fighting Stallion	Tansey, Robert	Robert Emmett Tansey	tt0179810
1746497017	1918	1918	Petticoats and Politics	Petticoats and Politics	Mitchell, Howard M.	Howard M. Mitchell	tt0009493
1746583356	1951	1951	The Company She Keeps	The Company She Keeps	Cromwell, John	John Cromwell	tt0042340
1746577302	1961	1961	Babes in Toyland	Babes in Toyland	Donohue, Jack	Jack Donohue	tt0054649
1746235379	1971	1970	I Drink Your Blood	I Drink Your Blood	Durston, David	David E. Durston	tt0067229
1746434017	1989	1989	National Lampoon's Christmas Vacation	National Lampoon's Christmas Vacation	Chechik, Jeremiah	Jeremiah S. Chechik	tt0097958
1746532748	1915	1915	Little Miss Brown	The Little Miss Brown	Young, James	James Young	tt0005634
1746435873	1998	1998	Practical Magic	Practical Magic	Dunne, Griffin	Griffin Dunne	tt0120791
1746567746	1933	1932	Rasputin and the Empress	Rasputin and the Empress	Boleslavsky, Richard; Brabin, Charles	Richard Boleslawski	tt0023374
1746574476	1934	1934	Baby Take a Bow	Baby, Take a Bow	Lachman, Harry	Harry Lachman	tt0024854
1746574265	1959	1959	Darby O'Gill and the Little People	Darby O'Gill and the Little People	Stevenson, Robert	Robert Stevenson	tt0052722
1746569870	1960	1960	Gunfighters of Abilene	Gunfighters of Abilene	Cahn, Edward L.	Edward L. Cahn	tt0053883
1746409067	1984	1984	Micki + Maude	Micki + Maude	Edwards, Blake	Blake Edwards	tt0087718
1746577372	1962	1962	Merrill's Marauders	Merrill's Marauders	Fuller, Samuel	Samuel Fuller	tt0056234
1746584365	1936	1936	Caryl of the Mountains	Caryl of the Mountains	Ray, Bernard B.	Bernard B. Ray	tt0027425
1746585367	1940	1940	Isle of Destiny	Isle of Destiny	Clifton, Elmer	Elmer Clifton	tt0032642
1746569519	1955	1954	Quest for the Lost City	Quest for the Lost City		Dana Lamb	tt0374984
1746582388	1929	1929	Seven Faces	Seven Faces	Viertel, Berthold	Berthold Viertel	tt0020387
1746421726	1999	1999	Drop Dead Gorgeous	Drop Dead Gorgeous	Jann, Michael Patrick	Michael Patrick Jann	tt0157503
1746577511	1933	1933	Trick for Trick	Trick for Trick	MacFadden, Hamilton	Hamilton MacFadden	tt0024698
1746574915	1937	1936	Romeo and Juliet	Romeo and Juliet	Cukor, George	George Cukor	tt0028203
1746583822	1929	1929	Riders of the Storm	Riders of the Storm	McGowan, J. P.	J.P. McGowan	tt0020330
1746542668	1925	1925	Canyon Rustlers	Canyon Rustlers	Webb, Harry	Harry S. Webb	tt0298783
1746508033	1926	1926	The Gypsy Romance	The Gypsy Romance			tt0133827
1746584818	1929	1929	Half Marriage	Half Marriage	Cowen, William J.	William J. Cowen	tt0019957
1746569608	1955	1955	Mad at the World	Mad at the World	Essex, Harry	Harry Essex	tt0048329
1746580495	1937	1937	Way Out West	Way Out West	Horne, James W.	James W. Horne	tt0029747
1746455784	1995	1995	Higher Learning	Higher Learning	Singleton, John	John Singleton	tt0113305
1746533215	1915	1915	Eugene Aram	Eugene Aram	Ridgely, Richard	Richard Ridgely	tt0005252
1746507904	1926	1926	The Adorable Deceiver	The Adorable Deceiver	Rosen, Phil	Phil Rosen	tt0016577
1746500596	1926	1926	The Escape	The Escape	Morante, Milburn	Milburn Morante	tt0016825
1746574010	1961	1961	Scanty Panties	Scanty Panties	Hornick, Jay	Jay Hornick	tt7255934
1746201132	1970	1970	Hi, Mom!	Hi, Mom!	De Palma, Brian	Brian De Palma	tt0065836
1746524649	1928	1928	The Law's Lash	The Law's Lash	Smith, Noel Mason	Noel M. Smith	tt0127645
1746202363	1970	1969	The Appointment	The Appointment	Lumet, Sidney	Sidney Lumet	tt0064035
1746507780	1925	1925	Souls for Sables	Souls for Sables	McKay, James C.	James C. McKay	tt0016378
1746584348	1939	1939	Wolf Call	Wolf Call	Waggner, George	George Waggner	tt0032139
1746499927	1950	1950	Duchess of Idaho	Duchess of Idaho	Leonard, Robert Z.	Robert Z. Leonard	tt0042417
1746577632	1933	1933	The Eagle and the Hawk	The Eagle and the Hawk	Walker, Stuart	Stuart Walker	tt0023973
1746520142	1926	1926	Prisoners of the Storm	Prisoners of the Storm	Reynolds, Lynn	Lynn Reynolds	tt0017289
1746524716	1927	1927	The Western Whirlwind	The Western Whirlwind	Rogell, Albert	Albert S. Rogell	tt0018557
1746475972	1948	1948	The Gay Intruders	The Gay Intruders	McCarey, Ray	Ray McCarey	tt0040384
1746492238	1944	1944	Casanova in Burlesque	Casanova in Burlesque	Goodwins, Leslie	Leslie Goodwins	tt0036700
1746474388	1944	1944	Knickerbocker Holiday	Knickerbocker Holiday	Brown, Harry Joe	Harry Joe Brown	tt0036988

1746584904	1940	1940	I Love You Again	I Love You Again	Van Dyke, W. S.	W.S. Van Dyke	tt0032617	
1746576520	1931	1931	Girls About Town	Girls About Town	Cukor, George	George Cukor	tt0021910	
1746492246	1945	1945	The Unseen	The Unseen	Allen, Lewis	Lewis Allen	tt0038205	
1746580296	1937	1937	Heidi	Heidi	Dwan, Allan	Allan Dwan	tt0028988	
1746573220	1932	1932	Madame Butterfly	Madame Butterfly	Gering, Marion	Marion Gering	tt0023169	
1746569867	1959	1958	Daddy-O	Daddy O	Place, Lou	Lou Place	tt0052719	
1746420795	1996	1996	Tin Cup	Tin Cup	Shelton, Ron	Ron Shelton	tt0117918	
1746564518	1953	1953	A Blueprint for Murder	A Blueprint for Murder	Stone, Andrew	Andrew L. Stone	tt0045566	
1746503938	1922	1922	Across the Border	Across the Border	Seeling, Charles R.	Charles R. Seeling	tt0012876	
1746567602	1933	1933	A Lady's Profession	A Lady's Profession	McLeod, Norman	Norman Z. McLeod	tt0024241	
1746438304	1943	1943	The Black Raven	The Black Raven	Newfield, Sam	Sam Newfield	tt0035679	
1746577253	1962	1962	The 300 Spartans	The 300 Spartans	Maté, Rudolph	Rudolph Maté	tt0055719	
1746583745	1930	1930	Maybe It's Love	Maybe It's Love	Wellman, William	William A. Wellman	tt0021133	
1746553973	1965	1965	The Girls on the Beach	The Girls on the Beach	Witney, William	William Witney	tt0059227	
1746576982	1960	1960	The Rise and Fall of Legs Diamond	The Rise and Fall of Legs Diamond	Boetticher, Budd	Budd Boetticher	tt0054243	
1746574043	1960	1960	Ma Barker's Killer Brood	Ma Barker's Killer Brood	Karn, Bill	Bill Karn	tt0054041	
1746502186	1915	1915	Princess Romanoff	Princess Romanoff	Powell, Frank	Frank Powell	tt0005930	
1746500068	1948	1947	Unconquered	Unconquered	DeMille, Cecil B.	Cecil B. DeMille	tt0039931	
1746574960	1937	1937	Midnight Court	Midnight Court	McDonald, Frank	Frank McDonald	tt0029238	
1746579043	1951	1951	His Kind of Woman	His Kind of Woman	Farrow, John	John Farrow	tt0043643	
1746492554	1945	1945	Leave It to Blondie	Leave It to Blondie	Berlin, Abby	Abby Berlin	tt0037866	
1746185682	1965	1965	Mme. Olga's Massage Parlor	Mme. Olga's Massage Parlor	Mawra, Joseph P.	Joseph P. Mawra	tt0126448	
1746495709	1920	1920	Huckleberry Finn	Huckleberry Finn	Taylor, William D.	William Desmond Taylor	tt0011313	
1746454151	1997	1997	Private Parts	Private Parts	Thomas, Betty	Betty Thomas	tt0119951	
1746508170	1924	1924	Manhattan	Manhattan	Burnside, R. H.	R.H. Burnside	tt0015117	
1746520180	1924	1924	Yankee Speed	Yankee Speed	Bradbury, Robert N.	Robert N. Bradbury	tt0015516	
1746497582	1919	1919	Are You Legally Married?	Are You Legally Married?	Thornby, Robert T.	Robert Thornby	tt0185160	
1746580909	1938	1938	Slander House	Slander House	Lamont, Charles	Charles Lamont	tt0030763	
1745341625	2003	2003	Intolerable Cruelty	Intolerable Cruelty	Coen, Joel	Joel Coen	tt0138524	
1746574912	1936	1936	Rio Grande Romance	Rio Grande Romance	Hill, Bob	Robert F. Hill	tt0028150	
1746585065	1929	1929	Darkened Rooms	Darkened Rooms	Gasnier, Louis	Louis J. Gasnier	tt0019806	
1746394195	1988	1988	Mississippi Burning	Mississippi Burning	Parker, Alan	Alan Parker	tt0095647	
1746500547	1926	1926	The High Flyer	The High Flyer	Brown, Harry J.	Harry Joe Brown	tt0016965	
1746502156	1915	1915	Pennington's Choice	Pennington's Choice	Bowman, William	William Bowman	tt0005884	
1746577088	1959	1959	Gunmen from Laredo	Gunmen from Laredo	MacDonald, Wallace	Wallace MacDonald	tt0052869	
1746582934	1937	1937	Larceny on the Air	Larceny on the Air	Pichel, Irving	Irving Pichel	tt0029112	
1746581563	1951	1951	Badman's Gold	Badman's Gold	Tansey, Robert	Robert Emmett Tansey	tt0167783	
1746393480	1988	1988	Switching Channels	Switching Channels	Kotcheff, Ted	Ted Kotcheff	tt0096203	
1746555618	1956	1956	Friendly Persuasion	Friendly Persuasion	Wyler, William	William Wyler	tt0049233	
1746513860	1914	1914	Shannon of the Sixth	Shannon of the Sixth	Melford, George	George Melford	tt0004581	
**1745309570**	**2007**	**2007**	**Mr. Magorium's Wonder Emporium**	**Mr. Magorium's Wonder Emporium**	**Helm, Zach**	**Zach Helm**	**tt0457419**	1
1746424619	1941	1941	Badlands of Dakota	Badlands of Dakota	Green, Alfred E.	Alfred E. Green	tt0033370	
1746492574	1947	1947	Trail to San Antone	Trail to San Antone	English, John	John English	tt0039909	
1746584627	1930	1930	Soldiers and Women	Soldiers and Women	Sloman, Edward	Edward Sloman	tt0021398	
1746421351	1993	1993	Wayne's World 2	Wayne's World 2	Surjik, Stephen	Stephen Surjik	tt0108525	
1746565296	1932	1932	Red-Headed Woman	Red Headed Woman	Conway, Jack	Jack Conway	tt0023385	
1745372519	2010	2010	Killers	Killers	Luketic, Robert	Robert Luketic	tt1103153	
1746500096	1950	1950	Father Is a Bachelor	Father Is a Bachelor	Foster, Norman; Berlin, Abby	Abby Berlin	tt0042449	
1746394019	1981	1981	Harry's War	Harry's War	Merrill, Kieth	Kieth Merrill	tt0082503	
1746561068	1932	1932	Law and Order	Law and Order	Cahn, Edward L.	Edward L. Cahn	tt0023121	
1746504418	1918	1918	Cupid's Roundup	Cupid's Roundup	Le Saint, Edward J.	Edward LeSaint	tt0008985	
1746570325	1934	1934	The Key	The Key	Curtiz, Michael	Michael Curtiz	tt0025346	
1745374194	2001	2000	Lisa Picard Is Famous	Lisa Picard Is Famous	Dunne, Griffin	Griffin Dunne	tt0233699	
1746533186	1916	1916	Prudence the Pirate	Prudence, the Pirate	Parke, William	William Parke	tt0007225	
1746248561	1967	1967	In Cold Blood	In Cold Blood	Brooks, Richard	Richard Brooks	tt0061809	
1746578777	1950	1950	Summer Stock	Summer Stock	Walters, Charles	Charles Walters	tt0043012	
1746507784	1949	1949	Edward, My Son	Edward, My Son	Cukor, George	George Cukor	tt0041329	

1746436355	1940	1939	Of Mice and Men	Of Mice and Men	Milestone, Lewis	Lewis Milestone	tt0031742	
**1746567003**	**1933**	**1933**	**State Fair**	**State Fair**	**King, Henry**	**Henry King**	**tt0024610**	1
1746520952	1950	1950	Crisis	Crisis	Brooks, Richard	Richard Brooks	tt0042352	
1746501647	1916	1916	The Bond Within	The Bond Within	Sloman, Edward	Edward Sloman	tt0319089	
1746256833	1979	1979	All That Jazz	All That Jazz	Fosse, Bob	Bob Fosse	tt0078754	
1746408645	1992	1992	Blame It on the Bellboy	Blame It on the Bellboy	Herman, Mark	Mark Herman	tt0103827	
1746516486	1920	1920	Blackbirds	Blackbirds	Dillon, Jack	John Francis Dillon	tt0010998	
1745373535	2004	2004	The Chronicles of Riddick	The Chronicles of Riddick	Twohy, David	David Twohy	tt0296572	
1746566663	1952	1952	Bonzo Goes to College	Bonzo Goes to College	De Cordova, Frederick	Frederick De Cordova	tt0044436	
1746524284	1923	1923	Counterfeit Love	Counterfeit Love	Sheldon, Roy; Ince, Ralph	Ralph Ince	tt0013947	
1746496269	1919	1919	The Common Cause	The Common Cause	Blackton, J. Stuart	J. Stuart Blackton	tt0007817	
1746504780	1922	1922	The Snowshoe Trail	The Snowshoe Trail	Bennett, Chester	Chester Bennett	tt0013615	
1746542669	1927	1927	Shanghaied	Shanghaied	Ince, Ralph	Ralph Ince	tt0018384	
1746563661	1952	1953	Meet Me at the Fair	Meet Me at the Fair	Sirk, Douglas	Douglas Sirk	tt0046059	
1746583616	1950	1950	The Sundowners	The Sundowners	Templeton, George	George Templeton	tt0043013	
1746509521	1949	1949	Follow Me Quietly	Follow Me Quietly	Fleischer, Richard O.	Richard Fleischer	tt0041378	
1746583462	1931	1931	The Common Law	The Common Law	Stein, Paul L.	Paul L. Stein	tt0021756	
**1746435174**	**1941**	**1941**	**The Stork Pays Off**	**The Stork Pays Off**	**Landers, Lew**	**Lew Landers**	**tt0034234**	1
1746574862	1936	1936	It's Up to You	It's Up to You	Cabanne, Christy	Christy Cabanne	tt1825825	
1746584210	1937	1937	Lancer Spy	Lancer Spy	Ratoff, Gregory	Gregory Ratoff	tt0029108	
1746525078	1921	1921	Three Word Brand	Three Word Brand	Hillyer, Lambert	Lambert Hillyer	tt0012754	
1746524314	1916	1916	The Velvet Paw	The Velvet Paw	Tourneur, Maurice	Maurice Tourneur	tt0007510	
1769970448	1965	1965	The Incredible Sex Revolution	The Incredible Sex Revolution	Zugsmith, Albert	Albert Zugsmith	tt0059310	
1746514084	1916	1916	The Half Million Bribe	The Half Million Bribe	Jones, Edgar	Edgar Jones	tt0006754	
1746218859	1977	1977	Islands in the Stream	Islands in the Stream	Schaffner, Franklin J.	Franklin J. Schaffner	tt0076211	
1746512427	1916	1916	The Suspect	The Suspect	Drew, S. Rankin	S. Rankin Drew	tt0007417	
1746504010	1917	1917	The Avenging Trail	The Avenging Trail	Ford, Francis	Francis Ford	tt0007661	
1746500523	1927	1927	The Swell-Head	The Swell Head	Graves, Ralph	Ralph Graves	tt0018458	
1746497541	1917	1917	Bab's Matinee Idol	Bab's Matinee Idol	Dawley, J. Searle	J. Searle Dawley	tt0007668	
1746492081	1948	1948	Angels' Alley	Angels' Alley	Beaudine, William	William Beaudine	tt0040097	
1746487072	1921	1921	Sure Fire	Sure Fire	Ford, Jack	John Ford	tt0012734	
1746532811	1915	1915	The House of Fear	The House of Fear	Daly, Arnold; Miller, Ashley	John Ince	tt0005515	
1746503538	1917	1917	Her Fighting Chance	Her Fighting Chance	Carewe, Edwin	Edwin Carewe	tt0008073	
1746582857	1938	1938	The Headleys at Home	The Headleys at Home	Beute, Chris	Chris Beute	tt0030222	
1745374803	2009	2009	The Final Destination	The Final Destination	Ellis, David R.	David R. Ellis	tt1144884	
1769971941	1969	1969	Dr. Masher	Dr. Masher	Maxwell, Alexander		tt0217393	
1746508919	1927	1927	One Increasing Purpose	One Increasing Purpose	Beaumont, Harry	Harry Beaumont	tt0018228	
1746578828	1963	1963	Hootenanny Hoot	Hootenanny Hoot	Nelson, Gene	Gene Nelson	tt0057153	
1746219334	1973	1973	This Is a Hijack	This Is a Hijack	Pollack, Barry	Barry Pollack	tt0072277	
1745272042	2000	2000	Price of Glory	Price of Glory	Avila, Carlos	Carlos Ávila	tt0188160	
1746496779	1921	1922	Stardust	Stardust	Henley, Hobart	Hobart Henley	tt0166819	
1746202597	1970	1970	Bloody Mama	Bloody Mama	Corman, Roger	Roger Corman	tt0065481	
1746503300	1917	1917	A Doll's House	A Doll's House	De Grasse, Joseph	Joseph De Grasse	tt0007867	
1746434588	1997	1997	Alien Resurrection	Alien: Resurrection	Jeunet, Jean-Pierre	Jean	tt0118583	
1746515095	1921	1921	Extravagance	Extravagance	Rosen, Philip E.	Phil Rosen	tt0012145	
1746475247	1946	1946	Boys' Ranch	Boys' Ranch	Rowland, Roy	Roy Rowland	tt0038381	
1746492075	1948	1948	Out of the Storm	Out of the Storm	Springsteen, R. G.	R.G. Springsteen	tt0040676	
1746520835	1925	1925	If I Marry Again	If I Marry Again	Dillon, John Francis	John Francis Dillon	tt0015009	
1746576896	1932	1932	The Most Dangerous Game	The Most Dangerous Game	Schoedsack, Ernest B.; Pichel, Irving	Irving Pichel	tt0023238	
1746435248	1943	1943	Kid Dynamite	Kid Dynamite	Fox, Wallace	Wallace Fox	tt0036073	
1746503016	1917	1917	The Weaker Sex	The Weaker Sex	West, Raymond B.	Raymond B. West	tt0184001	
1746423980	1941	1941	In the Navy	In the Navy	Lubin, Arthur	Arthur Lubin	tt0033754	
1746513155	1916	1916	The Sorrows of Love	The Sorrows of Love	Giblyn, Charles	Charles Giblyn	tt0158977	
1746509579	1926	1926	False Friends	False Friends	Ford, Francis	Francis Ford	tt0325347	
1746583391	1950	1950	The Palomino	The Palomino	Nazarro, Ray	Ray Nazarro	tt0042831	
1746454361	1943	1943	Stormy Weather	Stormy Weather	Stone, Andrew	Andrew L. Stone	tt0036391	
1746585230	1929	1929	The Desert Song	The Desert Song	Del Ruth, Roy	Roy Del Ruth	tt0019813	

1746574660	1933	1932	Crashin' Broadway	Crashin' Broadway	McCarthy, John P.	John P. McCarthy	tt0023914
1746234648	1967	1967	The King's Pirate	The King's Pirate	Weis, Don	Don Weis	tt0061867
1745359582	2001	2001	Chop Suey	Chop Suey	Weber, Bruce	Bruce Weber	tt0276860
1746516544	1920	1920	Whispering Devils	Whispering Devils	Voshell, John M.	Harry Garson	tt0011858
1746509478	1927	1927	The Return of Boston Blackie	The Return of Boston Blackie	Hoyt, Harry O.	Harry O. Hoyt	tt0018319
1745293936	2005	2005	Proof	Proof	Madden, John	John Madden	tt0377107
1745302617	2006	2006	Another Gay Movie	Another Gay Movie	Stephens, Todd	Todd Stephens	tt0443431
1745360048	2004	2004	Hellboy	Hellboy	del Toro, Guillermo	Guillermo del Toro	tt0167190
1746500557	1949	1949	Rimfire	Rimfire	Eason, B. Reeves	B. Reeves Eason	tt0041811
1746248748	1980	1980	The Blue Lagoon	The Blue Lagoon	Kleiser, Randal	Randal Kleiser	tt0080453
1746555982	1955	1955	Escape to Burma	Escape to Burma	Dwan, Allan	Allan Dwan	tt0048044
1746503843	1921	1921	Father Tom	Father Tom	O'Brien, John B.	John B. O'Brien	tt0131387
1746486846	1918	1918	The Finger of Justice	The Finger of Justice	Chaudet, Louis William	Louis Chaudet	tt0009068
1746485565	1919	1919	Everybody's Business	Everybody's Business	Dawley, J. Searle	J. Searle Dawley	tt0010092
1746455261	1997	1997	Cold Around the Heart	Cold Around the Heart	Ridley, John	John Ridley	tt0118870
1746566304	1956	1956	Julie	Julie	Stone, Andrew L.	Andrew L. Stone	tt0049388
1746582717	1935	1935	Gigolette	Gigolette	Lamont, Charles	Charles Lamont	tt0026408
1746512892	1915	1915	The Great Ruby	The Great Ruby	O'Neil, Barry	Barry O'Neil	tt0005418
1746423499	1995	1995	Operation Dumbo Drop	Operation Dumbo Drop	Wincer, Simon	Simon Wincer	tt0114048
1746555606	1957	1957	Johnny Tremain	Johnny Tremain	Stevenson, Robert	Robert Stevenson	tt0050567
1746559872	1963	1963	Soldier in the Rain	Soldier in the Rain	Nelson, Ralph	Ralph Nelson	tt0057517
1746438449	1941	1941	The Shepherd of the Hills	The Shepherd of the Hills	Hathaway, Henry	Henry Hathaway	tt0034182
1746504026	1919	1919	Brothers Divided	Brothers Divided	Keenan, Frank	Frank Keenan	tt0187762
1746570251	1933	1933	Man of Action	Man of Action	Melford, George	George Melford	tt0024299
1746504735	1922	1922	Kindred of the Dust	Kindred of the Dust	Walsh, R. A.	Raoul Walsh	tt0013293
1746577553	1938	1938	A Christmas Carol	A Christmas Carol	Marin, Edwin L.	Edwin L. Marin	tt0029992
1746454282	1941	1941	Night of January 16th	The Night of January 16th	Clemens, William	William Clemens	tt0033954
1746524701	1915	1915	Her Mother's Secret	Her Mother's Secret	Thomson, Frederick A.		tt0312757
1746503784	1918	1918	The Unchastened Woman	The Unchastened Woman	Humphrey, William	William Humphrey	tt0009739
1746424550	1943	1943	Wolves of the Range	Wolves of the Range	Newfield, Sam	Sam Newfield	tt0036543
1746574941	1936	1936	Silly Billies	Silly Billies	Guiol, Fred	Fred Guiol	tt0028250
1746585074	1929	1929	The Last of Mrs. Cheyney	The Last of Mrs. Cheyney	Franklin, Sidney	Sidney Franklin	tt0020081
1746434603	1992	1992	Dr. Giggles	Dr. Giggles	Coto, Manny	Manny Coto	tt0104139
1746580562	1938	1938	Outlaw Express	Outlaw Express	Waggner, George	George Waggner	tt0030537
1746569895	1956	1956	The Toy Tiger	The Toy Tiger	Hopper, Jerry	Jerry Hopper	tt0049873
1746584225	1939	1939	The Hound of the Baskervilles	The Hound of the Baskervilles	Lanfield, Sidney	Sidney Lanfield	tt0031448
1746554922	1965	1965	Sergeant Deadhead	Sergeant Dead Head	Taurog, Norman	Norman Taurog	tt0059704
1746464873	1944	1944	Hands Across the Border	Hands Across the Border	Kane, Joseph	Joseph Kane	tt0035965
1746532422	1915	1915	The Wolf of Debt	The Wolf of Debt	Harvey, Jack	Jack Harvey	tt0006300
1746520897	1949	1949	Roseanna McCoy	Roseanna McCoy	Reis, Irving	Irving Reis	tt0041824
1746394770	1985	1985	Friday the 13th: A New Beginning	Friday the 13th: A New Beginning	Steinmann, Danny	Danny Steinmann	tt0089173
1746503044	1915	1914	The Sins of the Mothers	The Sins of the Mothers	Ince, Ralph W.	Ralph Ince	tt0006052
1746520944	1949	1948	Loaded Pistols	Loaded Pistols	English, John	John English	tt0040543
1745395979	2008	2007	Meet Bill	Meet Bill	Wallack, Melisa ; Goldmann, Bernie	Bernie Goldmann	tt0790623
1746501417	1910	1910	The Way of the Red Man	The Way of the Red Man	Turner, Otis	Otis Turner	tt0346134
1746466567	1917	1917	The Birth of Patriotism	The Birth of Patriotism	Ingleton, E. Magnus	E. Magnus Ingleton	tt0007704
1746580597	1937	1937	Ridin' the Lone Trail	Ridin' the Lone Trail	Newfield, Sam	Sam Newfield	tt0029487
1746573867	1960	1960	Where the Boys Are	Where the Boys Are	Levin, Henry	Henry Levin	tt0054469
1746504385	1920	1920	The Cost	The Cost	Knoles, Harley	Harley Knoles	tt0189446
1746550309	1955	1955	Wiretapper	Wiretapper	Ross, Dick	Dick Ross	tt0049957
1746422273	1992	1992	Home Alone 2: Lost in New York	Home Alone 2: Lost in New York	Columbus, Chris	Chris Columbus	tt0104431
1746576587	1932	1932	The Age of Consent	The Age of Consent	LaCava, Gregory	Gregory La Cava	tt0022614
1746474392	1946	1946	Galloping Thunder	Galloping Thunder	Nazarro, Ray	Ray Nazarro	tt0038548
1746410884	1994	1994	Timecop	Timecop	Hyams, Peter	Peter Hyams	tt0111438
1746583323	1929	1929	Lucky In Love	Lucky in Love	Webb, Kenneth	Kenneth S. Webb	tt0020123
1746584616	1929	1929	Fashions in Love	Fashions in Love	Schertzinger, Victor	Victor Schertzinger	tt0019870
1745302880	2006	2006	Dreamgirls	Dreamgirls	Condon, Bill	Bill Condon	tt0443489

1746578490	1960	1960	A Dog of Flanders	A Dog of Flanders	Clark, James B.	James B. Clark	tt0052745
1746583732	1930	1930	Sons of the Saddle	Sons of the Saddle	Brown, Harry J.	Harry Joe Brown	tt0021407
1746533049	1914	1914	The Light Unseen	The Light Unseen	Taylor, Stanley E. V.	Stanner E.V. Taylor	tt0004225
1745358890	2001	2001	Rush Hour 2	Rush Hour 2	Ratner, Brett	Brett Ratner	tt0266915
1746454938	1941	1941	Sheriff of Tombstone	Sheriff of Tombstone	Kane, Joseph	Joseph Kane	tt0034183
1746508410	1927	1927	The Notorious Lady	The Notorious Lady	Baggot, King	King Baggot	tt0018210
1746393174	1989	1989	Bill & Ted's Excellent Adventure	Bill & Ted's Excellent Adventure	Herek, Stephen	Stephen Herek	tt0096928
1746494396	1910	1910	The Woman from Mellon's	The Woman from Mellon's	Griffith, D. W.	D.W. Griffith	tt0001460
1746514219	1910	1910	Lost in the Soudan	Lost in the Soudan	Turner, Otis	Otis Turner	tt0345588
1746234518	1967	1967	Divorce American Style	Divorce American Style	Yorkin, Bud	Bud Yorkin	tt0061581
1746409797	1984	1983	The Prodigal	The Prodigal	Collier, James F.	James F. Collier	tt0086146
1746435891	1989	1989	Shirley Valentine	Shirley Valentine	Gilbert, Lewis	Lewis Gilbert	tt0098319
1746423567	1991	1991	Backdraft	Backdraft	Howard, Ron	Ron Howard	tt0101393
1746576932	1960	1960	The Half Pint	The Half Pint	Jourdan, Erven	Erven Jourdan	tt0168844
1746565576	1933	1933	Thundering Herd	The Thundering Herd	Hathaway, Henry	Henry Hathaway	tt0025893
1746503977	1917	1917	Trooper 44	Trooper 44	Gahris, Roy	Roy Gahris	tt0178079
1746580750	1937	1937	Nudist Land	Nudist Land	Horter, A. Royal	A. Royal Horter	tt5710644
1746508119	1925	1925	The Trouble With Wives	The Trouble with Wives	St. Clair, Malcolm	Malcolm St. Clair	tt0016457
1746500023	1924	1924	The White Moth	The White Moth	Tourneur, Maurice	Maurice Tourneur	tt0015492
1746583748	1931	1931	Yankee Don	Yankee Don	Mason, Noel	Noel M. Smith	tt0022581
1746219537	1971	1971	Vanishing Point	Vanishing Point	Sarafian, Richard C.	Richard C. Sarafian	tt0067927
1746582517	1935	1935	Don't Bet on Blondes	Don't Bet on Blondes	Florey, Robert	Robert Florey	tt0026289
1746420896	1996	1995	Angela	Angela	Miller, Rebecca	Rebecca Miller	tt0112364
1746422233	1991	1991	House Party 2	House Party 2	McHenry, Doug; Jackson, George	George Jackson	tt0102065
1746584896	1940	1939	The Light That Failed	The Light That Failed	Wellman, William A.	William A. Wellman	tt0031572
1745293389	2007	2006	Behind the Mask: The Rise of Leslie Vernon	Behind the Mask: The Rise of Leslie Vernon	Glosserman, Scott	Scott Glosserman	tt0437857
1746465351	1942	1942	The Boogie Man Will Get You	The Boogie Man Will Get You	Landers, Lew	Lew Landers	tt0034540
1746248811	1973	1973	The Naked Ape	The Naked Ape	Driver, Donald	Donald Driver	tt0070433
1746530752	1950	1950	The Admiral Was a Lady	The Admiral Was a Lady	Rogell, Albert S.	Albert S. Rogell	tt0042182
1746576893	1932	1932	White Eagle	White Eagle	Hillyer, Lambert	Lambert Hillyer	tt0023693
1746581199	1958	1958	Tarawa Beachhead	Tarawa Beachhead	Wendkos, Paul	Paul Wendkos	tt0052272
1746573779	1956	1956	Baby Doll	Baby Doll	Kazan, Elia	Elia Kazan	tt0048973
1769971125	1969	1969	A Taste of Hot Lead	A Taste of Hot Lead	Rotsler, William	William Rotsler	tt0174260
1746491953	1948	1948	Macbeth	Macbeth	Welles, Orson	Orson Welles	tt0040558
1746573658	1932	1932	Washington Merry-Go-Round	Washington Merry Go Round	Cruze, James	James Cruze	tt0023673
1746419951	1981	1981	Reds	Reds	Beatty, Warren	Warren Beatty	tt0082979
1746578704	1962	1962	Hands of a Stranger	Hands of a Stranger	Arnold, Newt	Newt Arnold	tt0056056
1745372409	2002	2002	The Santa Clause 2	The Santa Clause 2	Lembeck, Michael	Michael Lembeck	tt0304669
1746410692	1983	1982	Vigilante	Vigilante	Lustig, William	William Lustig	tt0084867
1746438453	1943	1943	Young Ideas	Young Ideas	Dassin, Jules	Jules Dassin	tt0036552
1746577123	1961	1961	The Outsider	The Outsider	Mann, Delbert	Delbert Mann	tt0055270
1746530690	1948	1948	The Tioga Kid	The Tioga Kid	Taylor, Ray	Ray Taylor	tt0178065
1746500420	1950	1950	Double Deal	Double Deal	Berlin, Abby	Abby Berlin	tt0042412
1746560844	1932	1932	The Misleading Lady	The Misleading Lady	Walker, Stuart	Stuart Walker	tt0023229
1746574598	1935	1935	Melody Trail	Melody Trail	Kane, Joseph	Joseph Kane	tt0026697
1745372507	2004	2004	Taxi	Taxi	Story, Tim	Tim Story	tt0316732
1746491883	1948	1948	Ruthless	Ruthless	Ulmer, Edgar G.	Edgar G. Ulmer	tt0040751
1746484196	1917	1917	Magda	Magda	Chautard, Emile	Emile Chautard	tt0008244
1746500731	1926	1926	Stepping Along	Stepping Along	Hines, Charles	Charles Hines	tt0017429
1746555942	1957	1957	Man of a Thousand Faces	Man of a Thousand Faces	Pevney, Joseph	Joseph Pevney	tt0050681
1746186015	1966	1966	What's Up Tiger Lily?	What's Up, Tiger Lily?		Woody Allen	tt0061177
1746577607	1936	1936	It Had to Happen	It Had to Happen	Del Ruth, Roy	Roy Del Ruth	tt0027808
1746513840	1913	1913	The Star of India	The Star of India	Blaché, Herbert	Herbert Blaché	tt0003404
1746235080	1971	1971	Shaft	Shaft	Parks, Gordon	Gordon Parks	tt0067741
1746559848	1964	1964	The Gallant One	The Gallant One	Stell, Aaron	Aaron Stell	tt0213674
1746503244	1920	1920	The Spirit of Good	The Spirit of Good	Cazeneuve, Paul	Paul Cazeneuve	tt0011724
1746581510	1928	1928	When the Law Rides	When the Law Rides	De Lacy, Robert	Robert De Lacey	tt0019570

1746580764	1939	1940	Mad Youth	Mad Youth	Shyer, Melville	Melville Shyer	tt0032741
1746513087	1914	1914	The Idler	The Idler	Carleton, Lloyd B.	Lloyd B. Carleton	tt0004137
1746233371	1967	1967	Double Trouble	Double Trouble	Taurog, Norman	Norman Taurog	tt0061595
1746218812	1976	1976	All This and World War II	All This and World War II	Winslow, Susan	Susan Winslow	tt0132679
1746270391	1978	1978	Heaven Can Wait	Heaven Can Wait	Beatty, Warren; Henry, Buck	Warren Beatty	tt0077663
1746503499	1920	1920	The Night Riders	The Night Rider	Butler, Alexander	Jay Hunt	tt0807011
1746484700	1917	1917	Mrs. Balfame	Mrs. Balfame	Powell, Frank	Frank Powell	tt0008334
1746508873	1924	1924	Battlin' Buckaroo	Battlin' Buckaroo	Neitz, Alvin J.		tt0315811
1769971421	1922	1922	Pardon Me	Pardon Me		Ralph Ceder	tt0323580
1746208548	1969	1969	The Girl With the Fabulous Box	The Girl with the Fabulous Box	Nisbet, Charles	Charles Nisbet	tt3619386
1746424588	1941	1941	Week-End for Three	Weekend for Three	Reis, Irving	Irving Reis	tt0034378
1746500200	1925	1925	The Crackerjack	The Crackerjack	Hines, Charles	Charles Hines	tt0015714
1746580270	1937	1936	Fury and the Woman	Fury and the Woman	Collins, Lewis D.	Lewis D. Collins	tt0028910
1746501340	1914	1914	The Spitfire	The Spitfire	Porter, Edwin Stanton; Thomson, Fr	Edwin S. Porter	tt0004628
1746197993	1966	1966	Once Before I Die	Once Before I Die	Derek, John	John Derek	tt0059539
1746582624	1938	1938	Kidnapped	Kidnapped	Werker, Alfred; Preminger, Otto	Alfred L. Werker	tt0030321
1745341823	2004	2003	Baadasssss!	Baadasssss!	Van Peebles, Mario	Mario Van Peebles	tt0367790
1746453990	1999	1999	Sweet and Lowdown	Sweet and Lowdown	Allen, Woody	Woody Allen	tt0158371
1746465071	1944	1944	Our Hearts Were Young and Gay	Our Hearts Were Young and Gay	Allen, Lewis	Lewis Allen	tt0037158
1746514466	1916	1916	Vengeance Is Mine	Vengeance Is Mine!	Broadwell, Robert B.	Robert Broadwell	tt0007511
1746438953	1941	1941	Cavalcade of Faith	Cavalcade of Faith		Allyn Butterfield	tt0447887
1746578460	1951	1951	Pals of the Golden West	Pals of the Golden West	Witney, William	William Witney	tt0043898
1746394180	1990	1990	Havana	Havana	Pollack, Sydney	Sydney Pollack	tt0099747
1746567275	1952	1952	Montana Territory	Montana Territory	Nazarro, Ray	Ray Nazarro	tt0044921
1746410823	1986	1986	Ruthless People	Ruthless People	Abrahams, Jim; Zucker, David; Zucke	Jim Abrahams	tt0091877
1746453761	1944	1944	Murder in the Blue Room	Murder in the Blue Room	Goodwins, Leslie	Leslie Goodwins	tt0037100
1745309114	2006	2006	Cocaine Cowboys	Cocaine Cowboys	Corben, Billy	Billy Corben	tt0380268
1746436774	1999	1999	The Mating Habits of the Earthbound Human	The Mating Habits of the Earthbound Huma	Abugov, Jeff	Jeff Abugov	tt0165874
1745309618	2007	2007	Fired!	Fired!	Bradley, Chris; LaBrache, Kyle	Chris Bradley	tt0816238
1746436921	1997	1997	McHale's Navy	McHale's Navy	Spicer, Bryan	Bryan Spicer	tt0119640
1746454813	1946	1946	A Night in Paradise	Night in Paradise	Lubin, Arthur	Arthur Lubin	tt0038778
1746577484	1959	1959	The Hanging Tree	The Hanging Tree	Daves, Delmer; Malden, Karl; Sherma	Delmer Daves	tt0052876
1746577078	1960	1960	Sex Kittens Go to College	Sex Kittens Go to College	Zugsmith, Albert	Albert Zugsmith	tt0054298
1746574910	1933	1933	Diplomaniacs	Diplomaniacs	Seiter, William A.	William A. Seiter	tt0023949
1746524176	1922	1922	Defying the Law	Defying the Law	Horner, Robert J.	Robert J. Horner	tt0321972
1746514738	1910	1910	A Midnight Cupid	A Midnight Cupid	Griffith, D. W.	D.W. Griffith	tt0001319
1746520959	1949	1949	Joe Palooka in the Counterpunch	Joe Palooka in The Counterpunch	LeBorg, Reginald	Reginald Le Borg	tt0041525
1746562434	1953	1953	Devil's Canyon	Devil's Canyon	Werker, Alfred	Alfred L. Werker	tt0045684
1746218676	1970	1970	Beneath the Planet of the Apes	Beneath the Planet of the Apes	Post, Ted	Ted Post	tt0065462
1746424252	1940	1940	We Who Are Young	We Who Are Young	Bucquet, Harold S.	Harold S. Bucquet	tt0033246
1769969705	1911	1911	The Way of the West	The Way of the West		Allan Dwan	tt0433209
1746520498	1949	1949	Alias Nick Beal	Alias Nick Beal	Farrow, John	John Farrow	tt0041107
1746577139	1958	1958	Ten Days to Tulara	Ten Days to Tulara	Sherman, George	George Sherman	tt0052282
1746513438	1922	1922	The Broadway Madonna	The Broadway Madonna	Revier, Harry	Harry Revier	tt0012977
1746186229	1910	1910	The Actor's Children	The Actor's Children	O'Neil, Barry	Barry O'Neil	tt0233154
1746577782	1935	1935	Dangerous	Dangerous	Green, Alfred E.	Alfred E. Green	tt0026261
1746581016	1910	1910	Her First Long Dress	Her First Long Dress			tt1390947
1746509281	1926	1926	The Jade Cup	The Jade Cup	Crane, Frank Hall	Frank Hall Crane	tt0017006
1746509519	1925	1925	The Verdict of the Desert	The Verdict of the Desert	Hart, Neal	Neal Hart	tt0016485
1746454501	1945	1945	Grissly's Millions	Grissly's Millions	English, John	John English	tt0037752
1746492861	1947	1947	Sarge Goes to College	Sarge Goes to College	Jason, Will	Will Jason	tt0039799
1746185535	1965	1965	A Very Special Favor	A Very Special Favor	Gordon, Michael	Michael Gordon	tt0059868
1746500962	1910	1910	Simple Charity	Simple Charity	Griffith, D. W.	D.W. Griffith	tt0001396
1746409307	1986	1986	Haunted Honeymoon	Haunted Honeymoon	Wilder, Gene	Gene Wilder	tt0091178
1746499907	1950	1950	Mrs. O'Malley and Mr. Malone	Mrs. O'Malley and Mr. Malone	Taurog, Norman	Norman Taurog	tt0042755
1746520810	1926	1926	Pleasures of the Rich	Pleasures of the Rich	Gasnier, Louis	Louis J. Gasnier	tt0017270
1746582497	1937	1937	Broadway Melody of 1938	Broadway Melody of 1938	Del Ruth, Roy	Roy Del Ruth	tt0028665

1746574654	1934	1934	Gambling	Gambling	Lee, Rowland V.	Rowland V. Lee	tt0025160
1746585277	1929	1929	The Smiling Terror	The Smiling Terror	Levigard, Josef	Joseph Levigard	tt0020422
1746570858	1934	1934	Brides of Sulu	Brides of Sulu	Nelson, Jack	John Nelson	tt0024913
1746574413	1959	1959	Forbidden Island	Forbidden Island	Griffith, Charles B.	Charles B. Griffith	tt0052818
1746583643	1928	1928	Don Juan of the West	Don Juan of the West		Ben F. Wilson	tt0356544
1746523051	1923	1923	Legally Dead	Legally Dead	Parke, William	William Parke	tt0014194
1746574971	1936	1936	Forgotten Faces	Forgotten Faces	Dupont, E. A.	Ewald André Dupont	tt0027636
1746569965	1962	1962	Married Too Young	Married Too Young	Moskov, George	George Moskov	tt0056225
1746420379	1994	1994	Wolf	Wolf	Nichols, Mike	Mike Nichols	tt0111742
1746454661	1942	1942	Who Is Hope Schuyler?	Who Is Hope Schuyler?	Loring, Thos. Z.	Thomas Z. Loring	tt0035556
1746509334	1927	1927	Sky-High Saunders	Sky High Saunders	Mitchell, Bruce	Bruce Mitchell	tt0018411
1746210136	1970	1970	The Christine Jorgensen Story	The Christine Jorgensen Story	Rapper, Irving	Irving Rapper	tt0065549
1746393661	1985	1985	The Purple Rose of Cairo	The Purple Rose of Cairo	Allen, Woody	Woody Allen	tt0089853
1746569642	1958	1958	The Lady Takes a Flyer	The Lady Takes a Flyer	Arnold, Jack	Jack Arnold	tt0051839
1746523058	1916	1916	The Decoy	The Decoy	Lederer, George W.	George W. Lederer	tt0006574
1746582135	1931	1931	The Public Enemy	The Public Enemy	Wellman, William A.	William A. Wellman	tt0022286
1746435323	1989	1989	Shocker	Shocker	Craven, Wes	Wes Craven	tt0098320
1746520501	1925	1925	The Man in Blue	The Man in Blue	Laemmle, Edward	Edward Laemmle	tt0016081
1746577555	1936	1936	And Sudden Death	And Sudden Death	Barton, Charles	Charles Barton	tt0027294
1745293325	2005	2005	Deuce Bigalow: European Gigolo	Deuce Bigalow: European Gigolo	Bigelow, Mike	Mike Bigelow	tt0367652
1746507884	1926	1926	Blarney	Blarney	De Sano, Marcel	Marcel De Sano	tt0016657
1746218589	1971	1971	The Big Doll House	The Big Doll House	Hill, Jack	Jack Hill	tt0066830
1746580470	1937	1937	A Family Affair	A Family Affair	Seitz, George B.	George B. Seitz	tt0028850
1746465260	1947	1947	Over the Santa Fe Trail	Over the Santa Fe Trail	Nazzaro, Ray	Ray Nazarro	tt0039690
1746569885	1957	1957	Rock Baby-Rock It	Rock Baby Rock It	Sporup, Murray Douglas	Murray Douglas Sporup	tt0050908
1746511738	1923	1923	The Call of the Hills	The Call of the Hills	Hornby, Fred	Fred Hornby	tt0013907
1745342436	2001	2001	Shallow Hal	Shallow Hal	Farrelly, Peter; Farrelly, Bobby	Bobby Farrelly	tt0256380
1746571274	1933	1933	Laughing at Life	Laughing at Life	Beebe, Ford	Ford Beebe	tt0024245
1746585023	1939	1939	Bad Boy	Bad Boy	Meyer, Herbert	Herbert Meyer	tt0031070
1746508953	1927	1927	The Terror of Bar X	The Terror of Bar X	Pembroke, Scott	Scott Pembroke	tt0169300
1746574813	1935	1935	The Lone Bandit	The Lone Bandit	McGowan, J. P.	J.P. McGowan	tt0200817
1746582931	1935	1935	Alibi Ike	Alibi Ike	Enright, Ray	Ray Enright	tt0026055
1746573983	1963	1963	Living Between Two Worlds	Living Between Two Worlds	Johnson, Bobby	Bobby Johnson	tt0057256
1746216839	1968	1968	No Way To Treat a Lady	No Way to Treat a Lady	Smight, Jack	Jack Smight	tt0063356
1746569638	1956	1956	Frontier Gambler	Frontier Gambler	Newfield, Sam	Sam Newfield	tt0049235
1746475270	1946	1946	The Bandit of Sherwood Forest	The Bandit of Sherwood Forest	Sherman, George; Levin, Henry	Henry Levin	tt0038326
1746439469	1943	1943	Gildersleeve's Bad Day	Gildersleeve's Bad Day	Douglas, Gordon	Gordon Douglas	tt0035941
1746487600	1918	1918	The Midnight Burglar	The Midnight Burglar	Ensminger, Bert	Robert Ensminger	tt0009370
1746520818	1926	1926	The Old Soak	The Old Soak	Sloman, Edward	Edward Sloman	tt0017229
1746578446	1960	1959	The Incredible Petrified World	The Incredible Petrified World	Warren, Jerry	Jerry Warren	tt0053944
1746524120	1915	1915	The Clemenceau Case	The Clemenceau Case	Brenon, Herbert	Herbert Brenon	tt0005100
1746583619	1929	1929	Rarin' to Go	Rarin' to Go		Bryan Foy	tt1538517
1746523936	1912	1912	Becky Gets a Husband	Becky Gets a Husband			tt1126599
1746492749	1948	1947	I Walk Alone	I Walk Alone	Haskin, Byron	Byron Haskin	tt0039482
1746497030	1917	1917	Sadie Goes to Heaven	Sadie Goes to Heaven	Van Dyke, W. S.	W.S. Van Dyke	tt0182401
1746492589	1948	1948	Fort Apache	Fort Apache	Ford, John	John Ford	tt0040369
1746562099	1957	1957	Spook Chasers	Spook Chasers	Blair, George	George Blair	tt0051005
1746507572	1924	1924	The Courageous Coward	The Courageous Coward	Hurst, Paul	Paul Hurst	tt0014802
1745282876	2005	2005	Boogeyman	Boogeyman	Kay, Stephen	Stephen Kay	tt0357507
1746514440	1923	1923	Slow as Lightning	Slow as Lightning	Jones, Grover	Grover Jones	tt0165479
1746491996	1944	1944	Passport to Destiny	Passport to Destiny	McCarey, Ray	Ray McCarey	tt0037167
1746559798	1964	1964	Pardon My Brush	Pardon My Brush	McCarthy, John K.	John K. McCarthy	tt0254657
1746567991	1952	1952	Flat Top	Flat Top	Selander, Lesley	Lesley Selander	tt0044621
1746583256	1928	1928	The Danger Rider	The Danger Rider	MacRae, Henry	Henry MacRae	tt0018813
1746235368	1979	1979	The Concorde...Airport '79	The Concorde... Airport '79	Rich, David Lowell	David Lowell Rich	tt0078740
1746563905	1952	1952	About Face	About Face	Del Ruth, Roy	Roy Del Ruth	tt0044323
1746474949	1947	1947	The Wild Frontier	The Wild Frontier	Ford, Philip	Philip Ford	tt0039995

1746235362	1975	1975	Jaws	Jaws	Spielberg, Steven	Steven Spielberg	tt0073195
1746486819	1918	1918	The Two-Soul Woman	The Two Soul Woman	Clifton, Elmer	Elmer Clifton	tt0009733
1746419389	1991	1991	Mobsters	Mobsters	Karbelnikoff, Michael	Michael Karbelnikoff	tt0102460
1746571259	1933	1933	Son of a Sailor	Son of a Sailor	Bacon, Lloyd	Lloyd Bacon	tt0024594
1746565904	1953	1953	The Steel Lady	The Steel Lady	Dupont, E. A.	Ewald André Dupont	tt0046368
1746584132	1940	1940	Abe Lincoln in Illinois	Abe Lincoln in Illinois	Cromwell, John	John Cromwell	tt0032181
1746561294	1956	1956	Hot Rod Girl	Hot Rod Girl	Martinson, Leslie T.	Leslie H. Martinson	tt0049335
1746208771	1966	1966	Feelin' Good	Feelin' Good	Pike, James A.	James A. Pike	tt0200634
1746514918	1928	1928	The Lone Patrol	The Lone Patrol		Fred Caldwell	tt0019108
1746435707	1940	1940	Women in War	Women in War	Auer, John H.	John H. Auer	tt0033274
1746420802	1983	1983	A Christmas Story	A Christmas Story	Clark, Bob	Bob Clark	tt0085334
1746500184	1924	1924	Leave It to Gerry	Leave It to Gerry	Gillstrom, Arvid E.	Arvid E. Gillstrom	tt0020089
1746570746	1934	1934	The Witching Hour	The Witching Hour	Hathaway, Henry	Henry Hathaway	tt0025998
1746577296	1963	1963	Follow the Boys	Follow the Boys	Thorpe, Richard	Richard Thorpe	tt0057066
1746520182	1925	1925	Compromise	Compromise	Crosland, Alan	Alan Crosland	tt0015701
1746409119	1985	1985	American Flyers	American Flyers	Badham, John	John Badham	tt0088707
1746520916	1950	1950	Destination Murder	Destination Murder	Cahn, Edward L.	Edward L. Cahn	tt0042394
1746520392	1926	1926	For Heaven's Sake	For Heaven's Sake	Taylor, Sam	Sam Taylor	tt0016895
1746560015	1964	1963	The Cool World	The Cool World	Clarke, Shirley	Shirley Clarke	tt0056952
1746512409	1914	1914	Tillie's Punctured Romance	Tillie's Punctured Romance	Sennett, Mack	Mack Sennett	tt0004707
1745374168	2001	2001	Valentine	Valentine	Blanks, Jamie	Jamie Blanks	tt0242998
1745340908	2002	2002	The Cockettes	The Cockettes	Weber, Bill; Weissman, David	Bill Weber	tt0303321
1746434632	2000	2000	Coyote Ugly	Coyote Ugly	McNally, David	David McNally	tt0200550
1746585008	1939	1939	Almost a Gentleman	Almost a Gentleman	Goodwins, Leslie	Leslie Goodwins	tt0031035
1745359380	2008	2007	Charlie Bartlett	Charlie Bartlett	Poll, Jon	Jon Poll	tt0423977
1746563057	1951	1951	Hurricane Island	Hurricane Island	Landers, Lew	Lew Landers	tt0043662
1746422234	1989	1989	Honey, I Shrunk the Kids	Honey, I Shrunk the Kids	Johnston, Joe	Joe Johnston	tt0097523
1746542625	1927	1926	Pirates of the Sky	Pirates of the Sky	Andrews, Charles	Charles Andrews	tt0148611
1746570601	1952	1952	Here Come the Marines	Here Come the Marines	Beaudine, William	William Beaudine	tt0044700
1745309572	2006	2005	Adam & Steve	Adam & Steve	Chester, Craig	Craig Chester	tt0372122
1746569770	1953	1953	Project Moon Base	Project Moon Base	Talmadge, Richard	Richard Talmadge	tt0046213
1745373929	2009	2008	Lymelife	Lymelife	Martini, Derick	Derick Martini	tt0363780
1746473743	1941	1941	Tumbledown Ranch in Arizona	Tumbledown Ranch in Arizona	Luby, S. Roy	S. Roy Luby	tt0034315
1746567267	1952	1952	Pony Soldier	Pony Soldier	Newman, Joseph M.	Joseph M. Newman	tt0045041
1746210845	1971	1971	What's the Matter with Helen?	What's the Matter with Helen?	Harrington, Curtis	Curtis Harrington	tt0067975
1746577166	1960	1960	Ice Palace	Ice Palace	Sherman, Vincent	Vincent Sherman	tt0053936
1746514492	1916	1916	The Flames of Johannis	The Flames of Johannis	Lewis, Edgar	Edgar Lewis	tt0133769
1746217777	1966	1966	Moment to Moment	Moment to Moment	LeRoy, Mervyn	Mervyn LeRoy	tt0060711
1746584309	1939	1939	The Adventures of Jane Arden	The Adventures of Jane Arden	Morse, Terry	Terry O. Morse	tt0031021
1746198012	1967	1966	Chafed Elbows	Chafed Elbows	Downey, Robert	Robert Downey Sr.	tt0138355
1746585416	1940	1940	Four Sons	Four Sons	Mayo, Archie	Archie Mayo	tt0032490
1746582210	1928	1928	Thanks for the Buggy Ride	Thanks for the Buggy Ride	Seiter, William A.	William A. Seiter	tt0019460
1746465127	1946	1946	Strange Conquest	Strange Conquest	Rawlins, John	John Rawlins	tt0038985
1746577732	1933	1933	Treason	Treason	Seitz, George B.	George B. Seitz	tt0024694
1745359305	2008	2007	Operation Filmmaker	Operation Filmmaker	Davenport, Nina	Nina Davenport	tt0984213
1746496826	1920	1920	The Flame of Hellgate	The Flame of Hellgate	Middleton, George E.	George E. Middleton	tt0195710
1746507910	1926	1926	Silken Shackles	Silken Shackles	Morosco, Walter	Walter Morosco	tt0017391
1769970699	1968	1968	Scarlet Negligee	Scarlet Négligée	Scott, Ron	Ron Scott	tt0257105
1746570437	1934	1934	Men in White	Men in White	Boleslavsky, Richard	Richard Boleslawski	tt0025489
1746465357	1941	1941	Kisses for Breakfast	Kisses for Breakfast	Seiler, Lewis	Lewis Seiler	tt0033792
1746435869	1996	1995	Palookaville	Palookaville	Taylor, Alan	Alan Taylor	tt0117284
1746581828	1929	1929	An Oklahoma Cowboy	An Oklahoma Cowboy	Carpenter, H. B.	J.P. McGowan	tt0020235
1746256221	1976	1975	Grey Gardens	Grey Gardens	Maysles, David; Maysles, Albert; Hov	Ellen Hovde	tt0073076
1746473740	1942	1942	Prairie Gunsmoke	Prairie Gunsmoke	Hillyer, Lambert	Lambert Hillyer	tt0035207
1746584490	1931	1931	Street Scene	Street Scene	Vidor, King	King Vidor	tt0022436
1746197882	1968	1968	Skidoo	Skidoo	Preminger, Otto	Otto Preminger	tt0063612
1745302919	2007	2007	Reno 911!: Miami	Reno 911!: Miami	Garant, Robert Ben	Robert Ben Garant	tt0499554

1746564131	1952	1952	The Story of Robin Hood and His Merrie Men	The Story of Robin Hood and His Merrie Mer	Annakin, Ken	Ken Annakin	tt0045197
1746514494	1910	1910	When the Cat's Away	When the Cat's Away			tt1822370
1746436573	1943	1943	The Law Rides Again	The Law Rides Again	James, Alan	Alan James	tt0036101
1746575998	1931	1931	No Limit	No Limit	Tuttle, Frank	Frank Tuttle	tt0022211
1746574996	1935	1935	Kind Lady	Kind Lady	Seitz, George B.	George B. Seitz	tt0027845
1746509453	1950	1950	The Great Jewel Robber	The Great Jewel Robber	Godfrey, Peter	Peter Godfrey	tt0042521
1746569855	1958	1958	Murder By Contract	Murder by Contract	Lerner, Irving	Irving Lerner	tt0051959
1746503263	1921	1921	The Bell Hop	The Bell Hop		Larry Semon	tt0011958
1746454812	1944	1943	The Desert Song	The Desert Song	Florey, Robert	Robert Florey	tt0036755
1746570061	1933	1933	Somewhere in Sonora	Somewhere in Sonora	Wright, Mack V.	Mack V. Wright	tt0024592
1746580699	1934	1934	The Human Side	The Human Side	Buzzell, Edward	Edward Buzzell	tt0025276
1746524637	1914	1914	The Walls of Jericho	The Walls of Jericho	Carleton, Lloyd B.	Lloyd B. Carleton	tt0004776
1769970471	1967	1967	Mundo Depravados	Mundo depravados	Jeffries, Herb	Herb Jeffries	tt0062008
1746577095	1951	1951	Flight to Mars	Flight to Mars	Selander, Lesley	Lesley Selander	tt0043545
1746409677	1982	1981	The Boogens	The Boogens	Conway, James L.	James L. Conway	tt0082094
1769977777	1983	1983	Lianna	Lianna	Sayles, John	John Sayles	tt0085838
1769974390	1916	1916	Destiny	Destiny	Beaumont, Harry	Harry Beaumont	tt0006582
1745342811	2002	2002	Signs	Signs	Shyamalan, M. Night	M. Night Shyamalan	tt0286106
1746516271	1920	1920	Her Beloved Villain	Her Beloved Villain	Wood, Sam	Sam Wood	tt0011282
1746500485	1925	1925	The Four From Nowhere	The Four from Nowhere	Ford, Francis	Francis Ford	tt0132980
1746566671	1952	1952	Wild Stallion	Wild Stallion	Collins, Lewis D.	Lewis D. Collins	tt0045330
1746514679	1915	1915	A Price for Folly	A Price for Folly	Baker, George D.	George D. Baker	tt0005923
1746514692	1910	1910	The Boy City	The Boy City			tt1954893
1769976138	1918	1917	The Slave	The Slave	Gillstrom, Arvid E.	Arvid E. Gillstrom	tt0258969
1746454864	2000	2000	Boys and Girls	Boys and Girls	Iscove, Robert	Robert Iscove	tt0204175
1746422956	1997	1997	love jones	Love Jones	Witcher, Theodore	Theodore Witcher	tt0119572
1746498249	1920	1920	Thoughtless Women	Thoughtless Women	Goodman, Daniel Carson	Daniel Carson Goodman	tt0011767
1769971157	1969	1969	Wild Outtakes	Wild Outtakes		Donald A. Davis	tt0261412
1746578557	1950	1950	International Burlesque	International Burlesque	Connell, W. Merle	W. Merle Connell	tt0126355
1746508113	1925	1925	Cold Nerve	Cold Nerve	McGowan, J. P.	J.P. McGowan	tt0015696
1746585400	1940	1940	The Courageous Dr. Christian	The Courageous Dr. Christian	Vorhaus, Bernard	Bernard Vorhaus	tt0032361
1746516309	1920	1920	What's Your Husband Doing?	What's Your Husband Doing?	Ingraham, Lloyd	Lloyd Ingraham	tt0011852
1746514652	1914	1914	Trapped in the Great Metropolis	Trapped in the Great Metropolis		George K. Rolands	tt0004725
1746503360	1918	1918	A Son of Strife	A Son of Strife	Trinchera, Paul	Paolo Trinchera	tt0190022
1746574556	1933	1933	Reform Girl	Reform Girl	Neufeld, Sam	Sam Newfield	tt0024491
1746577393	1951	1951	Bullfighter and the Lady	Bullfighter and the Lady	Boetticher, Budd	Budd Boetticher	tt0043363
1746577894	1938	1938	Girls on Probation	Girls on Probation	McGann, William	William C. McGann	tt0030183
1746438932	1941	1941	Mercy Island	Mercy Island	Morgan, William	William Morgan	tt0033906
1746438737	1995	1995	Hideaway	Hideaway	Leonard, Brett	Brett Leonard	tt0113303
1746581215	1959	1959	The Wild and the Innocent	The Wild and the Innocent	Sher, Jack	Jack Sher	tt0053445
1745272248	2000	2000	What Lies Beneath	What Lies Beneath	Zemeckis, Robert	Robert Zemeckis	tt0161081
1746523831	1915	1915	Inspiration	Inspiration	Platt, George Foster	George Foster Platt	tt0005551
1746582490	1937	1937	Night Key	Night Key	Corrigan, Lloyd	Lloyd Corrigan	tt0029309
1745359993	2003	2003	Agent Cody Banks	Agent Cody Banks	Zwart, Harald	Harald Zwart	tt0313911
1746512925	1914	1914	The Littlest Rebel	The Littlest Rebel	Lewis, Edgar	Edgar Lewis	tt0004244
1746504040	1920	1920	Love, Honor and Obey	Love, Honor and Obey	Cordova, Leander de	Leander De Cordova	tt0196722
1746513625	1927	1927	A Boy of the Streets	A Boy of the Streets	Hunt, Charles J.	Charles J. Hunt	tt0017695
1746562083	1953	1953	Jalopy	Jalopy	Beaudine, William	William Beaudine	tt0045926
1746508201	1949	1949	The Blazing Trail	The Blazing Trail	Nazarro, Ray	Ray Nazarro	tt0041186
1746439194	1998	1998	Pecker	Pecker	Waters, John	John Waters	tt0126604
1746571263	1933	1933	Torch Singer	Torch Singer	Hall, Alexander; Somnes, George	Alexander Hall	tt0024685
1746465288	1945	1945	She Went to the Races	She Went to the Races	Goldbeck, Willis	Willis Goldbeck	tt0038083
1746503500	1917	1917	The Amazons	The Amazons	Kaufman, Joseph	Joseph Kaufman	tt0007631
1746524234	1927	1927	Back to God's Country	Back to God's Country	Willat, Irvin	Irvin Willat	tt0017655
1746574894	1933	1933	Sing Sinner Sing	Sing Sinner Sing	Christy, Howard	Howard Christie	tt0024564
1746577935	1935	1935	Age of Indiscretion	Age of Indiscretion	Ludwig, Edward	Edward Ludwig	tt0026045
1746554994	1964	1964	Island of the Blue Dolphins	Island of the Blue Dolphins	Clark, James B.	James B. Clark	tt0058241

1746582859	1938	1938	The Port of Missing Girls	Port of Missing Girls	Brown, Karl	Karl Brown	tt0030614
1746503278	1921	1921	A Heart To Let	A Heart to Let	Dillon, Edward	Edward Dillon	tt0012260
1746561502	1955	1955	The Sea Chase	The Sea Chase	Farrow, John	John Farrow	tt0048593
1769970428	1967	1967	The Game People Play	The Game People Play	Johnsen, S. N.	Sande N. Johnsen	tt0137644
1746235049	1974	1974	Phantom of the Paradise	Phantom of the Paradise	De Palma, Brian	Brian De Palma	tt0071994
1746394524	1988	1987	Big Bad Mama II	Big Bad Mama II	Wynorski, Jim	Jim Wynorski	tt0092652
1746436346	1941	1941	Bullets for O'Hara	Bullets for O'Hara	Howard, William K.	William K. Howard	tt0033439
1746554594	1963	1963	Papa's Delicate Condition	Papa's Delicate Condition	Marshall, George	George Marshall	tt0057400
1746423979	1940	1940	Ghost Valley Raiders	Ghost Valley Raiders	Sherman, George	George Sherman	tt0032522
1746575533	1910	1910	Ferdie's Vacation	Ferdie's Vacation			tt0348612
1746574409	1962	1962	The Three Stooges Meet Hercules	The Three Stooges Meet Hercules	Bernds, Edward	Edward Bernds	tt0056579
1746581554	1951	1951	Oh! Susanna	Oh! Susanna	Kane, Joseph	Joseph Kane	tt0043874
1746576863	1932	1932	Ladies of the Jury	Ladies of the Jury	Sherman, Lowell	Lowell Sherman	tt0023108
1746565915	1953	1953	The Man from the Alamo	The Man from the Alamo	Boetticher, Budd	Budd Boetticher	tt0046035
1746559952	1964	1964	McHale's Navy	McHale's Navy	Montagne, Edward J.	Edward Montagne	tt0058336
1746584036	1940	1940	The Mortal Storm	The Mortal Storm	Borzage, Frank	Frank Borzage	tt0032811
1746577186	1960	1960	Scent of Mystery	Scent of Mystery	Cardiff, Jack	Jack Cardiff	tt0054271
1746532454	1915	1915	The Warning	The Warning	Lawrence, Edmund	Edmund Lawrence	tt0006242
1746542627	1925	1925	Silent Sheldon	Silent Sheldon	Webb, Harry	Harry S. Webb	tt0016351
1746582385	1930	1930	Sisters	Sisters	Flood, James	James Flood	tt0021383
1746508974	1924	1924	A Pair of Hellions	A Pair of Hellions	Willis, Walter	Walter Willis	tt0015210
1746435300	1989	1989	Do the Right Thing	Do the Right Thing	Lee, Spike	Spike Lee	tt0097216
1746420511	1983	1983	Frightmare	Frightmare	Vane, Norman Thaddeus	Norman Thaddeus Vane	tt0083973
1746218152	1967	1967	Hotter After Dark	Hotter After Dark		Mort Shuman	tt0141209
1745293757	2007	2007	Music and Lyrics	Music and Lyrics	Lawrence, Marc	Marc Lawrence	tt0758766
1769971939	1969	1969	And Five Makes Jason	And Five Makes Jason	Stagg, William	William Stagg	tt0210503
1746513120	1916	1916	The Innocence of Lizette	The Innocence of Lizette	Kirkwood, James	James Kirkwood	tt0125242
1746524973	1927	1927	Galloping Fury	Galloping Fury	Eason, Reaves	B. Reeves Eason	tt0017913
1769969910	1912	1912	The Arab's Bride	The Arab's Bride	Nichols, George O.	George Nichols	tt0002028
1746502387	1915	1915	The Clue	The Clue	Neill, James; Reicher, Frank	James Neill	tt0005103
1746454480	1941	1941	The Miracle Kid	The Miracle Kid	Beaudine, William	William Beaudine	tt0035067
1746584966	1939	1939	Desperate Trails	Desperate Trails	Ray, Albert	Albert Ray	tt0031224
1746581413	1928	1928	The Sheik of Mojave	The Sheik of Mojave			tt0339660
1746565882	1953	1953	The 49th Man	The 49th Man	Sears, Fred F.	Fred F. Sears	tt0045463
1746574627	1933	1933	The Constant Woman	The Constant Woman	Schertzinger, Victor	Victor Schertzinger	tt0023904
1746410434	1984	1984	The Woman in Red	The Woman in Red	Wilder, Gene	Gene Wilder	tt0088414
1746573724	1959	1959	They Came to Cordura	They Came to Cordura	Rossen, Robert	Robert Rossen	tt0053351
1746487149	1917	1917	Youth	Youth	Fielding, Romaine	Romaine Fielding	tt0008799
1746509468	1926	1926	Hands Across the Border	Hands Across the Border	Kirkland, David	David Kirkland	tt0016946
1745342687	2003	2003	My Boss's Daughter	My Boss's Daughter	Zucker, David	David Zucker	tt0270980
1746211256	1973	1973	The Seven-Ups	The Seven Ups	D'Antoni, Philip	Philip D'Antoni	tt0070672
1746520933	1949	1948	My Own True Love	My Own True Love	Bennett, Compton	Compton Bennett	tt0040629
1746424004	1940	1940	Before I Hang	Before I Hang	Grinde, Nick	Nick Grinde	tt0032245
1746218864	1974	1973	The Last Detail	The Last Detail	Ashby, Hal	Hal Ashby	tt0070290
1746512051	1914	1914	The Jungle	The Jungle	Thomas, Augustus; Irving, George He	George Irving	tt0004182
1746555313	1955	1955	Air Strike	Air Strike	Roth, Cy	Cy Roth	tt0047805
1746583663	1931	1931	Clearing the Range	Clearing the Range	Brower, Otto	Otto Brower	tt0021751
1746562443	1953	1953	White Witch Doctor	White Witch Doctor	Hathaway, Henry	Henry Hathaway	tt0046547
1746575002	1937	1937	Live, Love and Learn	Live, Love and Learn	Fitzmaurice, Geo.	George Fitzmaurice	tt0029157
1746501489	1916	1916	A Youth of Fortune	A Youth of Fortune	Turner, Otis	Otis Turner	tt0007602
1746583932	1928	1928	Dead Man's Curve	Dead Man's Curve	Rosson, Richard	Richard Rosson	tt0018818
1746435724	1940	1940	And One Was Beautiful	And One Was Beautiful	Sinclair, Robert B.	Robert B. Sinclair	tt0032205
1746409713	1990	1990	Mo' Better Blues	Mo' Better Blues	Lee, Spike	Spike Lee	tt0100168
1746502225	1910	1910	Romance of the Lazy K Ranch	Romance of the Lazy K Ranch			tt0348738
1745372513	2003	2003	Cold Mountain	Cold Mountain	Minghella, Anthony	Anthony Minghella	tt0159365
1746507814	1949	1949	Wild Weed	Wild Weed	Scott, Sherman	Sam Newfield	tt0041865
1746454487	1944	1944	Three Little Sisters	Three Little Sisters	Santley, Joseph	Joseph Santley	tt0037372

1746524055	1923	1923	The Knock on the Door	The Knock on the Door	Curran, William Hughes	William Hughes Curran	tt0328036
1746573744	1958	1958	My World Dies Screaming	My World Dies Screaming	Daniels, Harold	Harold Daniels	tt0051962
1746583015	1940	1940	Anne of Windy Poplars	Anne of Windy Poplars	Hively, Jack	Jack Hively	tt0032211
1746569726	1957	1957	Apache Warrior	Apache Warrior	Williams, Elmo	Elmo Williams	tt0050131
1746500684	1925	1925	Accused	Accused	Henderson, Dell	Dell Henderson	tt0015536
1746584863	1930	1930	Be Yourself!	Be Yourself!	Freeland, Thornton	Thornton Freeland	tt0020670
1746562118	1957	1957	Hell Ship Mutiny	Hell Ship Mutiny	Sholem, Lee; Williams, Elmo	Lee Sholem	tt0050497
1746512650	1917	1917	Heart Strings	Heart Strings	Holubar, Allan	Allen Holubar	tt0008063
1746574754	1938	1938	Rawhide	Rawhide	Taylor, Ray	Ray Taylor	tt0030655
1746455605	1996	1996	Star Trek: First Contact	Star Trek: First Contact	Frakes, Jonathan	Jonathan Frakes	tt0117731
1746585231	1928	1928	Five and Ten Cent Annie	Five and Ten Cent Annie	Del Ruth, Roy	Roy Del Ruth	tt0018894
1746497622	1919	1919	Red Blood and Yellow	Red Blood and Yellow	Robbins, Jesse J.	Gilbert M. 'Broncho Billy' Anderson	tt0192517
1746487869	1917	1917	Passion	Passion	Ridgely, Richard	Richard Ridgely	tt0181755
1746533211	1914	1914	Too Late	Too Late		Marshall Farnum	tt1198662
1746583980	1939	1939	Rose of Washington Square	Rose of Washington Square	Ratoff, Gregory	Gregory Ratoff	tt0031877
1746524516	1923	1923	Why Worry?	Why Worry?	Newmeyer, Fred; Taylor, Sam	Fred C. Newmeyer	tt0014611
1746423760	1992	1992	The Power of One	The Power of One	Avildsen, John G.	John G. Avildsen	tt0105159
1746577458	1958	1958	Young and Wild	Young and Wild	Witney, William	William Witney	tt0052416
1746497593	1917	1917	The Planter	The Planter	Heffron, T. N.	Thomas N. Heffron	tt0008437
1746562218	1955	1955	Tarzan's Hidden Jungle	Tarzan's Hidden Jungle	Schuster, Harold	Harold D. Schuster	tt0048699
1746585240	1930	1930	Under a Texas Moon	Under a Texas Moon	Curtiz, Michael	Michael Curtiz	tt0021503
1746574381	1962	1962	Tales of Terror	Tales of Terror	Corman, Roger	Roger Corman	tt0056552
1746577321	1963	1963	Common Law Wife	Common Law Wife	Sayers, Eric	Eric Sayers	tt0123722
1746584862	1929	1929	Unmasked	Unmasked	Lewis, Edgar	Edgar Lewis	tt0020537
1745309270	2006	2006	The Hills Have Eyes	The Hills Have Eyes	Aja, Alexandre	Alexandre Aja	tt0454841
1746492588	1944	1944	Song of the Open Road	Song of the Open Road	Simon, S. Sylvan	S. Sylvan Simon	tt0037297
1745309228	2005	2005	Saw II	Saw II	Bousman, Darren Lynn	Darren Lynn Bousman	tt0432348
1746583140	1938	1938	In Old Chicago	In Old Chicago	King, Henry	Henry King	tt0029047
1746514111	1916	1916	The Hunted Woman	The Hunted Woman	Drew, S. Rankin	S. Rankin Drew	tt0006836
1746500029	1946	1946	Behind Green Lights	Behind Green Lights	Brower, Otto	Otto Brower	tt0038346
1746184541	1965	1965	Ship of Fools	Ship of Fools	Kramer, Stanley	Stanley Kramer	tt0059712
1746524791	1914	1914	Burning Daylight: the Adventures of "Burning Da	Burning Daylight: The Adventures of 'Burnir	Bosworth, Hobart	Hobart Bosworth	tt0003732
1769970635	1968	1968	Deep Inside	Deep Inside	Sarno, Joe	Joseph W. Sarno	tt0060294
1746567945	1933	1933	I Loved a Woman	I Loved a Woman	Green, Alfred E.	Alfred E. Green	tt0024160
1746475938	1945	1945	Great Stagecoach Robbery	Great Stagecoach Robbery	Selander, Lesley	Lesley Selander	tt0217521
1746578058	1935	1935	Enchanted April	Enchanted April	Beaumont, Harry	Harry Beaumont	tt0026313
1746570201	1952	1952	Captain Pirate	Captain Pirate	Murphy, Ralph	Ralph Murphy	tt0044475
1745359425	2003	2003	Daddy Day Care	Daddy Day Care	Carr, Steve	Steve Carr	tt0317303
1746507851	1924	1924	The Siren of Seville	The Siren of Seville	Storm, Jerome; Stromberg, Hunt	Jerome Storm	tt0015337
1746198217	1969	1969	The Mafia Girls	Mafia Girls	Ross, Ed	Ed Ross	tt0243398
1746500201	1924	1924	Listen Lester	Listen Lester	Seiter, William A.	William A. Seiter	tt0015073
1746570170	1933	1933	From Headquarters	From Headquarters	Dieterle, William	William Dieterle	tt0024040
1746585426	1939	1939	Inside Story	Inside Story	Cortez, Ricardo	Ricardo Cortez	tt0031488
1746576910	1951	1951	Gold Raiders	Gold Raiders	Bernds, Edward	Edward Bernds	tt0043591
1746583494	1930	1930	Cameo Kirby	Cameo Kirby	Cummings, Irving	Irving Cummings	tt0020728
1745293695	2006	2006	She's the Man	She's the Man	Fickman, Andy	Andy Fickman	tt0454945
1746455155	1998	1998	Out of Sight	Out of Sight	Soderbergh, Steven	Steven Soderbergh	tt0120780
1746520513	1927	1927	Tea for Three	Tea for Three	Leonard, Robert Z.	Robert Z. Leonard	tt0018467
1746512194	1915	1915	The Woman Pays	The Woman Pays	Jones, Edgar	Edgar Jones	tt0006303
1746465204	1943	1943	A Stranger in Town	A Stranger in Town	Rowland, Roy	Roy Rowland	tt0036395
1746575670	1932	1932	Igloo	Igloo	Scott, Ewing	Ewing Scott	tt0023050
1746584821	1930	1930	Check and Double Check	Check and Double Check	Brown, Melville	Melville W. Brown	tt0020758
1746423541	1940	1940	Keeping Company	Keeping Company	Simon, S. Sylvan	S. Sylvan Simon	tt0033782
1746219598	1977	1976	Eaten Alive!	Eaten Alive	Hooper, Tobe	Tobe Hooper	tt0074455
1745358030	2008	2008	Shine a Light	Shine a Light	Scorsese, Martin	Martin Scorsese	tt0893382
1746569829	1956	1956	The Young Guns	The Young Guns	Band, Albert	Albert Band	tt0049974
1746437392	1943	1943	Coney Island	Coney Island	Lang, Walter	Walter Lang	tt0035749

1746500226	1927	1927	The Reckless Mollycoddle	The Reckless Mollycoddle		Tony D'Algy	tt0018309
1745272300	2001	2000	Big Eden	Big Eden	Bezucha, Thomas	Thomas Bezucha	tt0212815
1746393328	1982	1982	The Atomic Cafe	The Atomic Cafe	Rafferty, Kevin; Loader, Jayne; Raffer	Jayne Loader	tt0083590
1746208530	1968	1968	Nobody's Perfect	Nobody's Perfect	Rafkin, Alan	Alan Rafkin	tt0063361
1746504028	1919	1919	The Lottery Man	The Lottery Man	Cruze, James	James Cruze	tt0010389
1746394421	1985	1985	Fright Night	Fright Night	Holland, Tom	Tom Holland	tt0089175
1746560147	1964	1964	The Trial of Lee Harvey Oswald	The Trial of Lee Harvey Oswald	Buchanan, Larry	Larry Buchanan	tt0058687
1746508675	1925	1925	Before Midnight	Before Midnight	Adolfi, John	John G. Adolfi	tt0015611
1745340057	2004	2004	Mean Girls	Mean Girls	Waters, Mark	Mark Waters	tt0377092
1746523078	1915	1915	The Eternal City	The Eternal City	Porter, Edwin S.; Ford, Hugh	Hugh Ford	tt0005250
1746516482	1920	1921	Bachelor Apartments	Bachelor Apartments	Walker, Johnny	Johnnie Walker	tt0010977
1746454732	2000	2000	Hanging Up	Hanging Up	Keaton, Diane	Diane Keaton	tt0162983
1746574765	1936	1935	Professional Soldier	Professional Soldier	Garnett, Tay	Tay Garnett	tt0028143
1746234905	1968	1968	Moonshiner's Woman	Moonshiner's Woman	Davison, D. E.	Donn Davison	tt0202479
1746455600	1999	1999	Lake Placid	Lake Placid	Miner, Steve	Steve Miner	tt0139414
1769970744	1912	1912	The Broken Lease	The Broken Lease	Walsh, F. S.	F.S. Walsh	tt0338815
1746533160	1915	1915	The Woman Who Lied	The Woman Who Lied	Henderson, Lucius	Lucius Henderson	tt0006305
1746503927	1918	1918	The Velvet Hand	The Velvet Hand	Gerrard, Douglas	Douglas Gerrard	tt0009756
1746560905	1932	1932	Klondike	Klondike	Rosen, Phil	Phil Rosen	tt0023096
1746502125	1916	1916	Her Husband's Wife	Her Husband's Wife	Abramson, Ivan	Ivan Abramson	tt0006793
1746501409	1910	1910	Seth's Temptation	Seth's Temptation	Olcott, Sidney	Sidney Olcott	tt0397627
1746410455	1989	1988	Heathers	Heathers	Lehmann, Michael	Michael Lehmann	tt0097493
1746515764	1918	1918	Jack Spurlock, Prodigal	Jack Spurlock, Prodigal	Harbaugh, Carl	Carl Harbaugh	tt0009236
1746561553	1932	1932	70,000 Witnesses	70,000 Witnesses	Murphy, Ralph	Ralph Murphy	tt0022603
1746583718	1930	1930	Follow the Leader	Follow the Leader	Taurog, Norman	Norman Taurog	tt0020895
1746585185	1929	1929	Her Private Affair	Her Private Affair	Stein, Paul	Paul L. Stein	tt0019979
1746199053	1967	1967	Lupe	Lupe	Rodriguez-Soltero, José	José Rodriguez	tt0242623
1746577119	1958	1958	Step Down to Terror	Step Down to Terror	Keller, Harry	Harry Keller	tt0052245
1746219567	1979	1979	The Seduction of Joe Tynan	The Seduction of Joe Tynan	Schatzberg, Jerry	Jerry Schatzberg	tt0079875
1746569699	1957	1957	The Burglar	The Burglar	Wendkos, Paul	Paul Wendkos	tt0049035
1746210668	1973	1973	Shamus	Shamus	Kulik, Buzz	Buzz Kulik	tt0070680
1746394732	1990	1990	Internal Affairs	Internal Affairs	Figgis, Mike	Mike Figgis	tt0099850
1746500675	1924	1924	The Other Kind of Love	The Other Kind of Love	Worne, Duke	Duke Worne	tt0015203
1745293396	2006	2006	Deja Vu	Deja Vu	Scott, Tony	Tony Scott	tt0453467
1746578724	1959	1959	I, Mobster	I Mobster	Corman, Roger	Roger Corman	tt0051760
1746465238	1946	1946	Somewhere in the Night	Somewhere in the Night	Mankiewicz, Joseph L.	Joseph L. Mankiewicz	tt0038965
1746509303	1925	1925	Dick Turpin	Dick Turpin	Blystone, John G.	John G. Blystone	tt0015750
1746435719	1941	1941	Cheers for Miss Bishop	Cheers for Miss Bishop	Garnett, Tay	Tay Garnett	tt0033459
1746501653	1915	1915	Playing Dead	Playing Dead	Drew, Sidney	Sidney Drew	tt0005901
1746183815	1910	1910	Bootles' Baby	Bootles' Baby	Miller, Ashley	Ashley Miller	tt1542608
1746508278	1925	1925	The Prairie Pirate	The Prairie Pirate	Mortimer, Edmund	Edmund Mortimer	tt0016238
1746573837	1957	1957	5 Steps to Danger	5 Steps to Danger	Kesler, Henry S.	Henry S. Kesler	tt0050087
1746532977	1915	1915	The Lone Star Rush	The Lone Star Rush	Mitchell, Edmund	Edmund Mitchell	tt0005644
1746491787	1948	1948	Overland Trails	Overland Trails	Hillyer, Lambert	Lambert Hillyer	tt0040678
1746579002	1962	1962	The Counterfeit Traitor	The Counterfeit Traitor	Seaton, George	George Seaton	tt0055871
1746532821	1923	1923	Rosita	Rosita	Lubitsch, Ernst	Ernst Lubitsch	tt0014416
1746580650	1937	1937	All-American Sweetheart	All American Sweetheart	Hillyer, Lambert	Lambert Hillyer	tt0028570
1746474499	1942	1942	Too Many Women	Too Many Women	Ray, Bernard B.	Bernard B. Ray	tt0035454
1746571366	1933	1933	The Prizefighter and the Lady	The Prizefighter and the Lady	Van Dyke, W. S.	W.S. Van Dyke	tt0024475
1746581746	1950	1950	Train to Tombstone	Train to Tombstone	Berke, William	William Berke	tt0043064
1746503906	1920	1920	Fixed by George	Fixed by George	Moran, Lee; Lyons, Eddie	Eddie Lyons	tt0011188
1746500574	1924	1924	Week End Husbands	Week End Husbands	Griffith, E. H.	Edward H. Griffith	tt0015475
1746394685	1985	1985	Weird Science	Weird Science	Hughes, John	John Hughes	tt0090305
1746475290	1948	1948	Mexican Hayride	Mexican Hayride	Barton, Charles T.	Charles Barton	tt0040582
1746392901	1985	1985	Stick	Stick	Reynolds, Burt	Burt Reynolds	tt0090073
1746421575	1981	1981	Death Hunt	Death Hunt	Hunt, Peter	Peter R. Hunt	tt0082247
1746208912	1966	1966	The Wild Angels	The Wild Angels	Corman, Roger	Roger Corman	tt0061189

1746503034	1916	1916	The Battle of Life	The Battle of Life	Vincent, James	James Vincent	tt0006404
1746211047	1972	1972	The Little Ark	The Little Ark	Clark, James B.	James B. Clark	tt0068864
1746409514	1992	1992	Final Impact	Final Impact	Merhi, Joseph ; Smoke, Steven	Joseph Merhi	tt0104267
1746581121	1959	1959	The Flying Fontaines	The Flying Fontaines	Sherman, George	George Sherman	tt0052814
1746566101	1953	1953	Valley of Head Hunters	Valley of Head Hunters	Berke, William	William Berke	tt0046491
1746569567	1957	1957	Joe Dakota	Joe Dakota	Bartlett, Richard	Richard Bartlett	tt0050565
1746467655	1917	1917	The Scarlet Crystal	The Scarlet Crystal	Swickard, Charles	Charles Swickard	tt0008542
1746554848	1954	1954	Ricochet Romance	Ricochet Romance	Lamont, Charles	Charles Lamont	tt0047410
1746564137	1952	1951	The African Queen	The African Queen	Huston, John	John Huston	tt0043265
1746503379	1919	1919	When a Man Rides Alone	When a Man Rides Alone	King, Henry	Henry King	tt0009791
1746455128	1995	1995	Never Talk to Strangers	Never Talk to Strangers	Hall, Peter	Peter Hall	tt0113965
1746500382	1926	1926	The Greater Glory	The Greater Glory	Rehfeld, Curt	Curt Rehfeld	tt0016940
1746393381	1988	1987	Someone to Love	Someone to Love	Jaglom, Henry	Henry Jaglom	tt0094007
1746566048	1957	1957	The Big Caper	The Big Caper	Stevens, Robert	Robert Stevens	tt0050190
1746439485	1945	1945	Circumstantial Evidence	Circumstantial Evidence	Larkin, John	John Francis Larkin	tt0037598
1746530741	1949	1949	Flaxy Martin	Flaxy Martin	Bare, Richard	Richard L. Bare	tt0041374
1746503634	1919	1919	Wild Oats	Wild Oats	Williams, C. J.	C.J. Williams	tt0138824
1746455414	1997	1996	Schizopolis	Schizopolis	Soderbergh, Steven	Steven Soderbergh	tt0117561
1746424613	1943	1943	The Man from Thunder River	The Man from Thunder River	English, John	John English	tt0036134
1746208453	1966	1966	Troublemakers	Troublemakers	Machover, Robert; Fruchter, Norma	Norman Fruchter	tt2025715
1746573800	1959	1959	The Rookie	The Rookie	O'Hanlon, George	George O'Hanlon	tt0053224
1746570362	1952	1952	Something for the Birds	Something for the Birds	Wise, Robert	Robert Wise	tt0045173
1746516315	1922	1922	One Glorious Day	One Glorious Day	Cruze, James	James Cruze	tt0013459
1746439689	1999	1999	Big Daddy	Big Daddy	Dugan, Dennis	Dennis Dugan	tt0142342
1746565946	1953	1953	Last of the Pony Riders	Last of the Pony Riders	Archainbaud, George	George Archainbaud	tt0045987
1746219014	1980	1980	The Fog	The Fog	Carpenter, John	John Carpenter	tt0080749
1746582973	1938	1937	Hollywood Hotel	Hollywood Hotel	Berkeley, Busby	Busby Berkeley	tt0029010
1746584075	1935	1935	Murder in the Fleet	Murder in the Fleet	Sedgwick, Edward	Edward Sedgwick	tt0026742
1746218890	1976	1976	Bound for Glory	Bound for Glory	Ashby, Hal	Hal Ashby	tt0074235
1745302784	2006	2006	Wrestling with Angels -- Playwright Tony Kushne	Wrestling with Angels: Playwright Tony Kus	Mock, Freida Lee	Freida Lee Mock	tt0493081
1746580681	1938	1938	Little Tough Guy	Little Tough Guy	Young, Harold	Harold Young	tt0030378
1746202414	1970	1970	The Grasshopper	The Grasshopper	Paris, Jerry	Jerry Paris	tt0065795
1769976116	1926	1926	Nobody's Business	Nobody's Business	Taurog, Norman	Norman Taurog	tt0347677
1746567309	1933	1933	The Fighting Parson	The Fighting Parson	Fraser, Harry	Harry L. Fraser	tt0024010
1746574964	1936	1936	Modern Times	Modern Times	Chaplin, Charlie	Charles Chaplin	tt0027977
1746520291	1925	1925	Pals	Pals	McCarthy, John P.	John P. McCarthy	tt0016197
1745342915	2004	2004	Outfoxed: Rupert Murdoch's War on Journalism	Outfoxed: Rupert Murdoch's War on Journa	Greenwald, Robert	Robert Greenwald	tt0418038
1745373551	2008	2008	Never Back Down	Never Back Down	Wadlow, Jeff	Jeff Wadlow	tt1023111
1746534794	1921	1921	The March Hare	The March Hare	Campbell, Maurice	Maurice Campbell	tt0012447
1746186012	1969	1969	The First Time	The First Time	Neilson, James; Grasshoff, Alex	James Neilson	tt0064331
1746453670	1998	1998	Phantoms	Phantoms	Chappelle, Joe	Joe Chappelle	tt0119891
1746248735	1975	1975	The Killer Elite	The Killer Elite	Peckinpah, Sam	Sam Peckinpah	tt0073240
1746514581	1922	1922	A Daughter of Luxury	A Daughter of Luxury	Powell, Paul	Paul Powell	tt0013053
1746434364	1989	1989	Pet Sematary	Pet Sematary	Lambert, Mary	Mary Lambert	tt0098084
1746509523	1926	1926	King of the Saddle	King of the Saddle	Craft, William J.	William James Craft	tt0017030
1745302381	2005	2005	This Divided State	This Divided State	Greenstreet, Steven	Steven Greenstreet	tt0444698
1746577647	1934	1934	Change of Heart	Change of Heart	Blystone, John G.	John G. Blystone	tt0024965
1746583922	1929	1929	Howdy Broadway	Howdy Broadway		Charles J. Hunt	tt0020005
1746503773	1918	1918	The Demon	The Demon	Baker, George D.	George D. Baker	tt0009004
1746495804	1919	1919	You Never Know Your Luck	You Never Know Your Luck	Powell, Frank	Frank Powell	tt0010927
1746503950	1921	1921	The Girl From Porcupine	The Girl from Porcupine	Henderson, Dell	Dell Henderson	tt0133799
1746520185	1924	1924	Curlytop	Curlytop	Elvey, Maurice	Maurice Elvey	tt0014812
1746584214	1938	1938	Danger on the Air	Danger on the Air	Garrett, Otis	Otis Garrett	tt0030037
1746436244	1992	1992	Universal Soldier	Universal Soldier	Emmerich, Roland	Roland Emmerich	tt0105698
1746464836	1946	1946	Claudia and David	Claudia and David	Lang, Walter	Walter Lang	tt0038416
1746232973	1969	1969	Backtrack	Backtrack!	Bellamy, Earl	Earl Bellamy	tt0064059
1745359385	2008	2008	Box Elder	Box Elder	Sklar, Todd	Todd Sklar	tt1215458

1746199222	1969	1968	Operation Cross Eagles	Operation Cross Eagles	Conte, Richard	Richard Conte	tt0064767
1746483159	1917	1916	Intrigue	The Intrigue	Robertson, John	Frank Lloyd	tt0006865
1746423843	1941	1941	The Great Mr. Nobody	The Great Mr. Nobody	Stoloff, Ben	Benjamin Stoloff	tt0033678
1746577925	1936	1936	Mummy's Boys	Mummy's Boys	Guiol, Fred	Fred Guiol	tt0027999
1746550289	1957	1957	Gun Glory	Gun Glory	Rowland, Roy	Roy Rowland	tt0050467
1746423752	1996	1996	Manny & Lo	Manny & Lo	Krueger, Lisa	Lisa Krueger	tt0116985
1746563370	1953	1953	The Big Heat	The Big Heat	Lang, Fritz	Fritz Lang	tt0045555
1746561518	1955	1954	Mambo	Mambo	Rossen, Robert	Robert Rossen	tt0047207
1746509067	1925	1925	Let Women Alone	Let Women Alone	Powell, Paul	Paul Powell	tt0016016
1746561954	1953	1953	Fair Wind to Java	Fair Wind to Java	Kane, Joseph	Joseph Kane	tt0045743
1746208895	1968	1968	The Shoes of the Fisherman	The Shoes of the Fisherman	Anderson, Michael	Michael Anderson	tt0063599
1746500710	1949	1949	The Story of Molly X	The Story of Molly X	Wilbur, Crane	Crane Wilbur	tt0041922
1746488041	1918	1918	Fires of Youth	Fires of Youth	Julian, Rupert	Rupert Julian	tt0009072
1746566194	1953	1953	Return to Paradise	Return to Paradise	Robson, Mark	Mark Robson	tt0046238
1746233132	1969	1969	Sweet Charity	Sweet Charity	Fosse, Bob	Bob Fosse	tt0065054
1746578025	1937	1937	Women of Glamour	Women of Glamour	Wiles, Gordon	Gordon Wiles	tt0029794
1746577651	1933	1933	Night Flight	Night Flight	Brown, Clarence	Clarence Brown	tt0024381
1746549828	1955	1955	Sudden Danger	Sudden Danger	Cornfield, Hubert	Hubert Cornfield	tt0048672
1746565490	1952	1952	Denver & Rio Grande	Denver and Rio Grande	Haskin, Byron	Byron Haskin	tt0044537
1746584193	1935	1935	Crime and Punishment	Crime and Punishment	Sternberg, Josef von	Josef von Sternberg	tt0026246
1746514894	1910	1910	Take Me Out to the Ball Game	Take Me Out to the Ball Game	Anderson, G. M.	Gilbert M. 'Broncho Billy' Anderson	tt0001408
1746474079	1947	1947	Exposed	Exposed	Blair, George	George Blair	tt0039361
1746584987	1940	1940	Misbehaving Husbands	Misbehaving Husbands	Beaudine, William	William Beaudine	tt0032799
1746582230	1930	1930	Burning Up	Burning Up	Sutherland, A. Edward	A. Edward Sutherland	tt0020718
1746566238	1953	1953	The Man Behind the Gun	The Man Behind the Gun	Feist, Felix	Felix E. Feist	tt0044872
1769970067	1912	1912	A Modern Highwayman	A Modern Highwayman	Turner, Otis	Otis Turner	tt0339399
1746465340	1947	1947	The Guilt of Janet Ames	The Guilt of Janet Ames	Levin, Henry; Vidor, Charles	Henry Levin	tt0039438
1746561351	1932	1932	Man from New Mexico	The Man from New Mexico	McGowan, J. P.	J.P. McGowan	tt0023184
1746464819	1945	1945	Follow That Woman	Follow That Woman	Landers, Lew	Lew Landers	tt0037712
1746561192	1965	1965	Bus Riley's Back in Town	Bus Riley's Back in Town	Hart, Harvey	Harvey Hart	tt0058998
1746584666	1930	1930	The Rampant Age	The Rampant Age	Rosen, Phil	Phil Rosen	tt0021284
1746454047	1941	1941	You're in the Army Now	You're in the Army Now	Seiler, Lewis	Lewis Seiler	tt0034411
1746465295	1942	1942	Priorities on Parade	Priorities on Parade	Rogell, Albert S.	Albert S. Rogell	tt0035216
1746439455	1998	1997	Unmade Beds	Unmade Beds	Barker, Nicholas	Nicholas Barker	tt0127952
1746507596	1923	1923	The Heart Raider	The Heart Raider	Ruggles, Wesley	Wesley Ruggles	tt0014120
1746562104	1954	1954	New Faces	New Faces	Horner, Harry	Harry Horner	tt0044963
1746579040	1959	1959	The Five Pennies	The Five Pennies	Shavelson, Melville	Melville Shavelson	tt0052809
1746409943	1988	1988	Salsa	Salsa	Davidson, Boaz	Boaz Davidson	tt0096030
1746571357	1952	1952	Desperadoes' Outpost	Desperadoes' Outpost	Ford, Philip	Philip Ford	tt0044542
1746574592	1933	1933	Private Detective 62	Private Detective 62	Curtiz, Michael	Michael Curtiz	tt0024471
1746584942	1940	1940	The Sea Hawk	The Sea Hawk	Curtiz, Michael	Michael Curtiz	tt0033028
1746437381	1941	1940	The Great Dictator	The Great Dictator	Chaplin, Charles	Charles Chaplin	tt0032553
1746524247	1915	1915	Mortmain	Mortmain	Marston, Theodore	Theodore Marston	tt0005772
1746219653	1979	1979	The China Syndrome	The China Syndrome	Bridges, James	James Bridges	tt0078966
1746520456	1925	1925	Saddle Cyclone	The Saddle Cyclone	Thorpe, Richard	Richard Thorpe	tt0159715
1746423564	1999	1999	The Bachelor	The Bachelor	Sinyor, Gary	Gary Sinyor	tt0120596
1746200000	1966	1966	Chamber of Horrors	Chamber of Horrors	Averback, Hy	Hy Averback	tt0060228
1746416016	1917	1917	The Lady of the Photograph	The Lady of the Photograph	Turbett, Ben	Ben Turbett	tt0176911
1746568137	1952	1952	Models Inc.	Models Inc.	LeBorg, Reginald	Reginald Le Borg	tt0044912
1746584118	1939	1939	The Devil's Daughter	The Devil's Daughter	Leonard, Arthur	Arthur H. Leonard	tt0031229
1746532648	1916	1916	Naked Hearts	Naked Hearts	Julian, Rupert	Rupert Julian	tt0007113
1746523036	1916	1916	Her Great Hour	Her Great Hour	Taylor, S. E. V.	Stanner E.V. Taylor	tt0157746
1746532649	1923	1923	The Daring Years	The Daring Years	Webb, Kenneth	Kenneth S. Webb	tt0013974
1746515809	1919	1919	The Woman Michael Married	The Woman Michael Married	Kolker, Henry	Henry Kolker	tt0010910
1746503062	1915	1915	The Circular Staircase	The Circular Staircase	Le Saint, Edward J.	Edward LeSaint	tt0005095
1746581998	1910	1910	In the Tall Grass Country	In the Tall Grass Country		William F. Haddock	tt0361744
1746581637	1910	1910	Girlies	Girlies		Sam Morris	tt1149225

1746392758	1984	1984	Love Streams	Love Streams	Cassavetes, John	John Cassavetes	tt0087644
1746423549	1940	1940	Opened by Mistake	Opened by Mistake	Archainbaud, George	George Archainbaud	tt0032876
1746504763	1922	1922	A Blind Bargain	A Blind Bargain	Worsley, Wallace	Wallace Worsley	tt0012949
1745282716	2006	2006	Sherrybaby	Sherrybaby	Collyer, Laurie	Laurie Collyer	tt0423169
1746410383	1983	1983	Valley Girl	Valley Girl	Coolidge, Martha	Martha Coolidge	tt0086525
1746270380	1980	1979	Silent Scream	The Silent Scream	Harris, Denny	Denny Harris	tt0081515
1746439435	1997	1997	Excess Baggage	Excess Baggage	Brambilla, Marco	Marco Brambilla	tt0119086
1746573742	1958	1958	Tonka	Tonka	Foster, Lewis R.	Lewis R. Foster	tt0052300
1746524640	1916	1916	The Green Swamp	The Green Swamp	Sidney, Scott	Scott Sidney	tt0006743
1746422135	1999	1999	The 13th Warrior	The 13th Warrior	McTiernan, John	John McTiernan	tt0120657
1746509559	1925	1925	My Pal	My Pal	Hayes, Ward	Ward Hayes	tt0016136
1746581382	1910	1910	The Little Prospector	The Little Prospector		Gilbert M. 'Broncho Billy' Anderson	tt0001288
1746565930	1953	1953	Stolen Identity	Stolen Identity	Fritsch, Gunther	Gunther von Fritsch	tt0046372
1746580449	1938	1938	Annabel Takes a Tour	Annabel Takes a Tour	Landers, Lew	Lew Landers	tt0029872
1746499890	1923	1923	The Fourth Musketeer	The Fourth Musketeer	Howard, William K.	William K. Howard	tt0014058
1746508399	1927	1927	The Rose of Kildare	The Rose of Kildare	Fitzgerald, Dallas M.	Dallas M. Fitzgerald	tt0018342
1745342864	2004	2004	Anchorman: The Legend of Ron Burgundy	Anchorman: The Legend of Ron Burgundy	McKay, Adam	Adam McKay	tt0357413
1746421188	1992	1992	Radio Flyer	Radio Flyer	Donner, Richard	Richard Donner	tt0105211
1746573859	1958	1958	The True Story of Lynn Stuart	The True Story of Lynn Stuart	Seiler, Lewis	Lewis Seiler	tt0052322
1746453545	1944	1944	Weird Woman	Weird Woman	LeBorg, Reginald	Reginald Le Borg	tt0037453
1746219587	1980	1980	Resurrection	Resurrection	Petrie, Daniel	Daniel Petrie	tt0081414
1746553510	1963	1963	Savage Sam	Savage Sam	Tokar, Norman	Norman Tokar	tt0057473
1746211266	1973	1973	Serpico	Serpico	Lumet, Sidney	Sidney Lumet	tt0070666
1746577687	1935	1935	The Daring Young Man	The Daring Young Man	Seiter, William A.	William A. Seiter	tt0026263
1746578382	1962	1962	Hero's Island	Hero's Island	Stevens, Leslie	Leslie Stevens	tt0056065
1746566350	1955	1955	Sincerely Yours	Sincerely Yours	Douglas, Gordon	Gordon Douglas	tt0048623
1746500537	1925	1925	The Danger Zone	The Danger Zone	Bradbury, Robert N.	Robert N. Bradbury	tt0015729
1746435317	1991	1991	Blue Desert	Blue Desert	Battersby, Bradley	Bradley Battersby	tt0101486
1746186042	1969	1968	Buona Sera, Mrs. Campbell	Buona Sera, Mrs. Campbell	Frank, Melvin	Melvin Frank	tt0062767
1746520087	1924	1924	Ridgeway of Montana	Ridgeway of Montana	Smith, Clifford S.	Clifford Smith	tt0015278
1746208723	1969	1969	Linda and Abilene	Linda and Abilene	Hansen, Mark	Herschell Gordon Lewis	tt0064590
1746520651	1949	1949	C-Man	'C' Man	Lerner, Joseph	Joseph Lerner	tt0041218
1746492064	1947	1947	This Time for Keeps	This Time for Keeps	Thorpe, Richard	Richard Thorpe	tt0039898
1746580745	1937	1937	It Happened in Hollywood	It Happened in Hollywood	Lachman, Harry	Harry Lachman	tt0029056
1746504190	1918	1918	Uneasy Money	Uneasy Money	Windom, Lawrence C.	Lawrence C. Windom	tt0008718
1746485538	1918	1918	All the World to Nothing	All the World to Nothing	King, Henry	Henry King	tt0008824
1746577844	1937	1937	Living on Love	Living on Love	Landers, Lew	Lew Landers	tt0029158
1746514964	1916	1916	Let Katie Do It	Let Katie Do It	Franklin, C. M.; Franklin, S. A.	Chester M. Franklin	tt0005613
1746553698	1964	1963	Naked Complex	Naked Complex	Mart, Roy	Ron Mart	tt0057351
1746502671	1915	1915	The Lost House	The Lost House	Cabanne, William Christy	Christy Cabanne	tt0005650
1746492577	1948	1948	I Love Trouble	I Love Trouble	Simon, S. Sylvan	S. Sylvan Simon	tt0040457
1746574580	1933	1933	Lucky Dog	Lucky Dog	Myers, Z.	Zion Myers	tt0024277
1746198512	1966	1966	Flame and the Fire	Flame and the Fire	Gaisseau, Pierre Dominique	Pierre	tt7806900
1746582592	1935	1935	Fighting Coward	The Fighting Coward	Milner, Dan	Dan Milner	tt0027867
1746421262	1994	1994	The Little Rascals	The Little Rascals	Spheeris, Penelope	Penelope Spheeris	tt0110366
1769971985	1969	1969	AC/DC	AC/DC	Winters, Larry	Larry Winters	tt0209872
1746562351	1953	1953	Marshal of Cedar Rock	Marshal of Cedar Rock	Keller, Harry	Harry Keller	tt0046049
1746508700	1926	1926	The Merry Cavalier	The Merry Cavalier	Mason, Noel	Noel M. Smith	tt0137949
1746454500	1946	1946	Stars Over Texas	Stars Over Texas	Tansey, Robert Emmett	Robert Emmett Tansey	tt0038981
1769970754	1912	1912	The Insurgent Senator	The Insurgent Senator		Bannister Merwin	tt0002279
1746524628	1915	1915	The Alien	The Alien	Ince, Thomas H.	Reginald Barker	tt0004874
1746520362	1925	1925	The Limited Mail	The Limited Mail	Hill, George	George W. Hill	tt0016025
1746555481	1955	1955	Seven Angry Men	Seven Angry Men	Warren, Charles Marquis	Charles Marquis Warren	tt0048602
1746577781	1936	1936	The Boss Rider of Gun Creek	The Boss Rider of Gun Creek	Selander, Lesley	Lesley Selander	tt0027389
1746507588	1926	1926	The Fighting Boob	The Fighting Boob	Nelson, Jack	Jack Nelson	tt0016856
1746514222	1923	1923	Mine To Keep	Mine to Keep	Wilson, Ben	Ben F. Wilson	tt0014281
1746514694	1923	1923	Big Brother	Big Brother	Dwan, Allan	Allan Dwan	tt0013870

1746491757	1948	1948	Reaching from Heaven	Reaching from Heaven	Strayer, Frank	Frank R. Strayer	tt0046229
1746434658	1996	1996	The Evening Star	The Evening Star	Harling, Robert	Robert Harling	tt0116240
1746515845	1921	1921	Shadows of Conscience	Shadows of Conscience	McCarthy, John P.	John P. McCarthy	tt0012670
1746562171	1954	1954	Her Twelve Men	Her Twelve Men	Leonard, Robert Z.	Robert Z. Leonard	tt0047076
1746408494	1985	1985	Vision Quest	Vision Quest	Becker, Harold	Harold Becker	tt0090270
1746474838	1947	1946	13 Rue Madeleine	13 Rue Madeleine	Hathaway, Henry	Henry Hathaway	tt0038279
1746435378	1999	1998	Where's Marlowe?	Where's Marlowe?	Pyne, Daniel	Daniel Pyne	tt0118150
1746583649	1931	1931	Bought	Bought!	Mayo, Archie	Archie Mayo	tt0021691
1746579041	1961	1961	Force of Impulse	Force of Impulse	Swimmer, Saul	Saul Swimmer	tt0132974
1746500658	1926	1926	Shadow Ranger	Shadow Ranger			tt0339655
1746475280	1945	1945	Rhythm Round-up	Rhythm Round Up	Keays, Vernon	Vernon Keays	tt0038027
1746394011	1986	1986	Cobra	Cobra	Cosmatos, George P.	George P. Cosmatos	tt0090859
1746563878	1953	1952	Ruby Gentry	Ruby Gentry	Vidor, King	King Vidor	tt0045109
1746583986	1939	1939	The Arizona Wildcat	The Arizona Wildcat	Leeds, Herbert I.	Herbert I. Leeds	tt0029878
[truncated: 985,447 more chars]
