# Supplementary material for: Long-term patterns of gender imbalance in an industry without ability or level of interest differences
Source: PLoS One. 2020 Apr 1;15(4):e0229662. doi: 10.1371/journal.pone.0229662 (PMC7112163; doi:10.1371/journal.pone.0229662)
Supplement: Attachment — Submitted filename: Response_to_reviewers.pdf [file pone.0229662.s012.pdf]

Northwestern | McCORMICK SCHOOL OF ENGINEERING

Luís A. Nunes Amaral
Erastus O. Haven Professor of Chem & Biol Eng.
co-Director, Northwestern Institute on Complex Systems
Chair, Data Science Initiative
Professor, Medicine, Molecular Biosci., Physics & Astronomy

Northwestern University
Dept. Chemical and Biological Engineering
2145 Sheridan Road, Room E-136 Evanston,
IL 60208–3102

Phone 847-491-7850
Fax 847-491-7070
amaral@northwestern.edu
http://amaral.northwestern.edu

February 11, 2020

Bing Xue, Ph.D.
Academic Editor
PLOS One

Dear Dr. Xue:

We have revised the manuscript in order to address the remaining major concern of Reviewer 1. We hope the manuscript is now acceptable for publication.

With warm regards,

Reply to Reviewers

Reviewer #1:

The authors have reworked their MS considerably. However, sadly, they have mostly ignored (or misunderstood) what I was criticising. They have added Miller (1999) and Lange et al. (2013) – two of many works I had recommended. That is basically good. However, althought they cite some of the suggested works now, R1 of the MS leaves many doubts if they understood what those works are asserting (based on empirical data of course). To make it more concrete, they state (p. 3): „Indeed, there is currently a great deal of controversy surrounding the primacy of evolutionary/biological mechanisms versus environmental/cultural mechanisms in explaining observed gender representation imbalances.“ Correct, nothing to criticise here. They go on: „Even though we pursue here an approach informed by cultural primacy approaches, we believe that our study is able to sidestep the controversy….“ Ok too, although this is were an informed biosocial researcher should get nervous. And indeed, the full sentence goes as follows: „Even though we pursue here an approach informed by cultural primacy approaches, we believe that our study is able to sidestep the controversy because the movie industry provides a case study in which evolutionary mechanisms actually would lead to the expectation that women would be over-represented. Specifically, studies informed by evolutionary psychology, suggest that women have a preference for activities where people and inter-personal interactions are prominent.“ First, how can you sidestep a controvery, while in the very same sentence diving fully into the very same controversy? Secondly and more importantly, the statement is utterly wrong. No informed evolutionary psychologist would predict that! The reference they use for this awkward statement is (no wonder) a non-evolutionary one (Lewis P. and Simpson R. Kanter revisited: Gender, power and (in)visibility. International Journal of Management Reviews, 14:141{158, 2012.). They are so many good references for this topic, and I have invested so much time in my first review mentioning many of them to the authors. This is really disappoiting! I am investing so much time to try to help the authors to end up having a sound piece of science. And this is what happened. Λ
As I have already explained in my first review: When it comes to gender differences (on average, of course) in the context of „social“ occupations, you need to be more differentiated. The motivation of an average men in the context of „social“ is to strive for social status, that is to climb the social ladder in order to get an upper spot in the social hierarchy. This pattern is cross-culturally universal and also in terms of time very stable. So the most parsimonious explanation for this would be to assume that this pattern is partially due to evolutionary selection pressures. How exactly? Men can benefit from high social status in terms of so-called fitness benefits over-proportionally compared to women of high social status. On the contrary, „social“ in the context of occupations that women are, on average, more interested in is kind of synonymous with nurturing and caring. The authors themselves state: „women have a preference for activities where people and inter-personal interactions are prominent“.
I won't go into more detail, because (1) I have explained this in my first review already and (2) I have suggested so many works that explain it in detail and support the assertion by strony empirical evidence. I leave it to the authors to change this part of their paper in order to make it correct or not.

We agree that the statement "we believe that our study is able to sidestep the controversy because the movie industry provides a case study in which evolutionary mechanisms actually would lead to the expectation that women would be over-represented" is controversial and un-necessary. In order to address the Reviewer's concerns, we removed it and re-wrote some of the surrounding text.
